# Environmental and Historical Determinants of African Horse Sickness: Insights from Predictive Modeling

**DOI:** 10.1155/2024/5586647

**Published:** 2024-08-13

**Authors:** KwangHyok Kim, TianGang Xu, Arivizhivendhan Kannan Villalan, TianYing Chi, XiaoJing Yu, MyongIl Jin, RenNa Wu, GuanYing Ni, ShiFeng Sui, ZhiLiang Wang, XiaoLong Wang

**Affiliations:** ^1^ College of Wildlife and Protected Area Northeast Forestry University, Harbin, Heilongjiang Province, China; ^2^ Key Laboratory of Wildlife Diseases and Bio-Security Management of Heilongjiang Province, Harbin, Heilongjiang Province, China; ^3^ Institute of Animal Genetic Engineering Branch of Biotechnology State Academy of Sciences, Pyongyang, Democratic People's Republic of Korea; ^4^ China Animal Health and Epidemiology Center, Qingdao, Shandong Province, China; ^5^ Kyeungsang Sariwon University of Agriculture, Sariwon, Democratic People's Republic of Korea; ^6^ HaiXi Animal Disease Control Center, Delingha, Qinghai Province, China; ^7^ Zhaoyuan Forest Resources Monitoring and Protection Service Center, Zhaoyuan, Shandong Province, China

## Abstract

African horse sickness (AHS) is a viral disease transmitted by arthropods that impacts Equidae, specifically horses and related species. Recognized as a notifiable disease by the World Organisation for Animal Health (WOAH), AHS is associated with a high mortality rate of 80%–90% in susceptible hosts and exhibits rapid transmission dynamics. Historical records document numerous instances of mass horse deaths attributed to AHS, with recent occurrences in Thailand and Malaysia in 2020 causing heightened concerns within the local horse industry. The lack of a comprehensive global perspective on the distribution and transmission of AHS poses challenges in comprehending and implementing effective prevention and control strategies. This study marks a pioneering effort in analyzing the global epidemiological patterns of AHS across different regions. By employing predictive modeling with a comprehensive set of environmental variables, we uncovered overarching global patterns in AHS dynamics, a first in this field. Our analysis revealed significant regional differences influenced by specific climatic conditions, highlighting the disease's complexity. The study also identifies new high-risk areas for AHS, underscoring the necessity for regionally tailored disease management strategies. Despite some limitations, such as the exclusion of wild equine data, this research offers critical insights for global AHS intervention and prevention, setting a path for future research incorporating broader datasets and socioeconomic factors.

## 1. Introduction

African horse sickness (AHS), a viral disease affecting equine species, is primarily transmitted by arthropods and significantly influenced by environmental conditions [1, 2, 3] as well as human activities [4]. The complexity of its transmission dynamics, shaped by both natural and anthropogenic factors, sets the stage for a comprehensive exploration of its historical spread and ecological implications. In the context of ongoing global warming and changing land use patterns, it is anticipated that the distribution and prevalence of AHS will undergo notable alterations [1, 2]. The African horse sickness virus (AHSV), classified within the *Orbivirus* genus, encompasses nine distinct serotypes. Among equine species, horses (*Equus ferus*) and donkeys from Europe and Asia (*Equus hemionus*) exhibit the highest susceptibility, with mortality rates reaching up to 90%. In contrast, African donkeys (*Equus africanus*) and various zebra species (*Equus quagga*, *Equus grevyi*, and *Equus zebra*) often remain asymptomatic, thereby serving as natural reservoirs for AHSV [5].

Tracing the historical trajectory of AHS reveals its expanding geographic scope. First documented in 1569 in Central and Eastern Africa [6], the disease has since marked its presence across diverse regions including the Middle East, Western Asia, North Africa, the Mediterranean Basin, and, more recently, Southeast Asia with incidents in Thailand and Malaysia in 2020 [7, 8, 9, 10, 11, 12, 13, 14]. This historical perspective lays a foundation for understanding the disease's current distribution and potential vectors. Contrary to some zoonotic diseases, AHSV transmission does not occur through direct contact between animals. Instead, it primarily depends on hematopoietic arthropod vectors. The *Culicoides* species, including *Culicoides imicola*, *Culicoides bolitinos*, *Culicoides variipennis*, and *Culicoides brevitarsis*, are the principal vectors facilitating regular transmission. Other vectors like mosquitoes (*Aedes aegypti*, *Anopheles stephensi*, and *Culex pipiens*), ticks (*Hyalomma dromedarii* and *Rhipicephalus sanguineus*), and biting flies (*Stomoxys calcitrans* and *Tabanus* spp.) contribute to intermittent transmission modes [15]. The geographical expansion of AHS correlates closely with the widening of the AHSV suitability zone, a phenomenon largely attributable to environmental factors influencing the distribution and behavior of AHSV-associated species.

Building upon the historical context, it is crucial to examine how environmental variables such as temperature and humidity influence the spread of AHS. These factors affect not only the virus's survival but also the behavior and population dynamics of the host and vectors, thereby playing a pivotal role in the epidemiology of the disease. Factors such as temperature, humidity, topography, and land cover have been extensively studied, revealing their significant impact on the virus's viability and the behavioral patterns of both the host species and the vectors. Research indicates that these environmental conditions directly influence the survival duration of the virus and affect the range of activities of the host and vector species [16, 17]. This suggests a complex interplay between the ecological landscape and the epidemiology of AHS, underscoring the need for a comprehensive understanding of environmental influences on the disease's transmission dynamics.

Further delving into specific environmental factors, temperature and relative humidity emerge as key determinants in the transmission of AHSV. Their impact on the life cycle and behavior of vectors such as *Culicoides* and mosquitoes highlights the intricate relationship between climatic conditions and disease dynamics. Specifically, temperature governs the rate of development in immature vectors and markedly affects their behavior and lifespan. Within certain temperature ranges, an increase in temperature results in a higher egg production rate, faster larval development, and an elongated period of biting activity in female *Culicoides* [18]. Conversely, extremely low temperatures inhibit *Culicoides* development and activity, whereas excessively high temperatures lead to increased mortality and reduced lifespans [19]. Similar temperature-dependent behavioral patterns are observed in mosquitoes (*A. aegypti*, *A. stephensi*, and *C. pipiens*) [20, 21, 22, 23], camel ticks (*H. dromedarii*), brown dog ticks (*R. sanguineus*) [24, 25], and biting flies from the *Stomoxys* and *Tabanus* genera [26, 27].

In addition to temperature, precipitation plays a critical role in shaping the transmission patterns of AHS. This aspect of environmental influence provides insights into the breeding habits of vectors and their consequent impact on the spread of the disease, illuminating the multifaceted nature of AHS transmission. Precipitation exerts a substantial influence on the development stages of immature vectors and the activity and dispersal patterns of adult vectors, primarily through its effects on atmospheric and soil humidity levels. In certain regions of South Africa, notably higher populations of *C*. *imicola* were recorded during years with abundant rainfall [28]. Furthermore, significant outbreaks of AHS in South Africa have been closely associated with periods of heavy rainfall followed by drought conditions [29]. These observations suggest a strong correlation between the presence of favorable breeding sites, supporting the immature development of *C*. *imicola*, and the incidence of AHS outbreaks. The breeding environments for mosquito larvae, such as water-filled containers, as well as the flight patterns and egg-to-larva transition process in biting flies, are also directly influenced by specific rainfall patterns [30]. Collectively, these factors may significantly contribute to the spread of AHS through intermittent transmission modes, resulting in a cumulative effect. However, it is important to note that excessive rainfall can negatively impact AHS transmission. For instance, heavy rains can lead to floods that wash away larvae, resulting in a decline in vector populations [31].

Beyond climatic factors, land cover and altitude are also instrumental in understanding the spread of AHS [17, 32, 33]. These elements contribute to the creation of suitable habitats for vectors, thereby influencing the distribution and intensity of AHS outbreaks. Research has pinpointed various land cover types, such as urban areas, croplands, and herbaceous tree shrubs, as conducive environments for AHS vectors, with these habitats being significantly influenced by alterations in altitude and land cover [34, 35, 36]. Additionally, the transportation and trade networks of horses are considered crucial in the dissemination of AHS [37, 38], highlighting the importance of stringent quarantine measures in horse movement. However, monitoring equine populations presents considerable challenges, and obtaining a thorough and accurate assessment of equine movement patterns is a daunting endeavor. Furthermore, given the observed alignment of AHS spread with wind-borne movements of infected midges, researchers have proposed the potential for AHS transmission via wind dispersion. Recent studies have incorporated wind speed as a variable in modeling the spatiotemporal distribution of AHS and *C*. *imicola* [33, 39, 40]. Despite these advancements, the optimization of *Culicoides* spp. dispersal through specific wind characteristics and its potential variations across regions and seasons remains an area of ongoing research and uncertainty.

In light of these diverse environmental influences [17, 33, 41], our study aims to integrate these varied environmental factors into a cohesive model. By employing a global-scale approach and focusing on variable identification, we endeavor to elucidate the general laws governing the spread of AHS and address the geographical variability in prediction accuracy. Our objective is to discern the overarching patterns in AHS distribution by identifying key variables and addressing the challenges posed by geographic scale selection in predictive modeling. To this end, we have utilized the MaxEnt model to define the ecological niches of AHS and its primary and intermittent transmission vectors.

Our research is grounded on the following assumptions, which were necessitated by constraints in data availability: (i) AHSV exclusively circulates within domestic horse populations, (ii) transmission of AHSV occurs solely through bloodsucking vector insects, and (iii) all vector species involved in the circulation of AHSV exhibit equal transmissibility. To the best of our knowledge, this study represents a pioneering effort to model the global dynamic laws governing AHSV distribution, thereby contributing novel insights to the field.

## 2. Materials and Methods

### 2.1. Data Collection

#### 2.1.1. AHS Occurrence Data

Location data pertaining to AHS were obtained from two primary sources: the Global Animal Disease Information System (EMPRES-i) website and reports published by the World Organisation for Animal Health (WOAH). These data form the basis for identifying the geographical distribution of AHS.

#### 2.1.2. AHSV Vector Data

The presence of AHSV vector species was compiled from a variety of sources. This includes a comprehensive review of existing literature accessible through academic databases such as Web of Science, ScienceDirect, and PubMed. Additionally, data were sourced from the Global Biodiversity Information Facility (GBIF) database (available at: https://www.gbif.org/). To ensure the accuracy and relevance of these data, any records that lacked specific geographic details were excluded from our analysis.

#### 2.1.3. Environmental Predictors and Horse Data

Our modeling approach integrated various environmental factors that potentially influence AHS distribution. These factors included climate conditions, terrain features, land cover types, and the distribution of horse populations. The sources for these data were globally recognized databases, ensuring a high standard of data quality and reliability. Prior to integration into our models, all spatial data were preprocessed using ArcGIS software, version 10.6. This preprocessing involved standardizing and resampling the data to achieve a uniform resolution of 30 arc seconds, facilitating more accurate and consistent analyses.

### 2.2. Climate-Based Zonal Segregation

Various climate patterns have a significant impact on the distribution of species and biodiversity gradients within a given area. Climate factors are widely known to be the fundamental drivers of AHS occurrence, and there are noticeable disparities in key climate variables associated with AHS across regions with different climate characteristics [42]. As a solution to address the inconsistencies in the major environmental factors affecting AHS and their level of influence attributable to the chosen geographical scale, using the Köppen climate map, regions were categorized into tropical, arid, temperate, continental, and polar/alpine climates. For modeling efficiency, each climate zone was further segmented by continent.

### 2.3. AHS Ecological Niche Modeling

Spatial autocorrelation was addressed by ensuring a minimum 10-km distance between recorded AHS locations using SDM Toolbox v1.1c [17, 41, 43]. In the identification of crucial variables associated with AHS, careful consideration was given to the process of reducing multicollinearity. This was done to prevent any distortion in the model caused by correlations between variables. Principal component analysis (PCA) and variance inflation factor (VIF) analysis were employed to resolve the problem of multicollinearity among predictors [44]. First, major climate predictors were selected in SPSS 2.2 using eigenvalues larger than 1.0 and the scree plot criterion or “broken stick” stopping rule. Next, the MaxEnt model analysis was performed to remove climate variables with high standard deviation (SD) and low contribution rate based on visual observation of the response curve and percent contribution. Last, VIF analysis was performed on selected climate variables and other variables to assess collinearity between environmental variables. If the VIF value was <10, it was assessed as low multicollinearity, and input was allowed. The regions where AHS occurrence was verified were separated according to their climate zones, modeled individually, and subsequently amalgamated. The modeling process was executed utilizing the MaxEnt program (version 3.4.1), with the random test percentage set at 30 and replicates configured to 10, and employing the random seed method. The result of this process is two AHS suitability maps. One map depicts the AHS suitability across the entirety of the outbreak data, while the other illustrates the AHS suitability based solely on AHS outbreak data predating the 2020 Thailand and Malaysia outbreak. This secondary map serves as a validation tool to assess the accuracy of the modeling outcomes, specifically to ascertain if the regions affected by the 2020 Thailand and Malaysia outbreak align with the identified risk areas. Model reliability was gauged using the area under the curve (AUC), considering AUC > 0.8 as an indicator of a well-fitted model.

### 2.4. AHSV Vectors Niche Modeling

In our research, we extended our focus beyond the commonly studied *Culicoides* spp. to include a broader range of potential carriers. This expansion encompasses mosquitoes, ticks, and biting flies, all recognized by the WOAH as vectors capable of intermittent transmission of AHS [15]. By incorporating vectors linked to both primary and intermittent transmission modes of AHSV, our model achieves a more thorough and nuanced understanding of the potential risk areas for AHS.

The methodology employed to minimize spatial autocorrelation and to execute PCA and VIF analyses is consistent with that used in AHS ecological niche modeling. Our approach involved confirming the presence of vector species based on climate zones, with each zone being modeled individually. We then integrated the modeling outcomes for each climate zone to create suitability maps for individual vector species. These maps were combined to form an overarching vector suitability map that includes all species under consideration. A model was considered well-fitted if its AUC value exceeded 0.8.

### 2.5. Identifying High-Risk Zones for AHS by Integrated Modeling Approach

**Figure 1:**
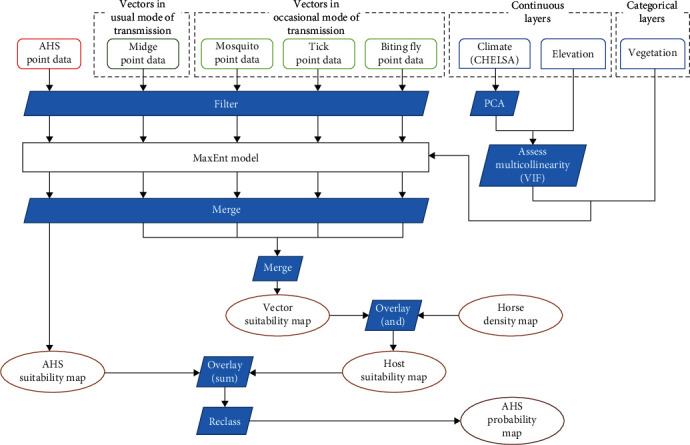
Flow chart of the modeling process for AHS.

To determine the likelihood of AHS occurrence in different regions, we employed an integrated modeling approach. Initially, vector suitability maps were overlaid with horse density data. This was followed by the addition of the AHS suitability map, culminating in the generation of a comprehensive AHS risk map. Two distinct AHS risk maps were generated as a result: one using AHS suitability maps incorporating all outbreak data and the other using AHS suitability maps incorporating only outbreak data from before the 2020 outbreak in Thailand and Malaysia. The risk levels were stratified into four categories: low (<0.25), medium (0.25–0.5), high (0.5–0.75), and very high (>0.75).  Figure 1 in the manuscript illustrates a flow chart detailing our modeling approach.

### 2.6. Assessment of Predictive Accuracy of AHS Risk Map

The accuracy of predicting AHS risk from two maps was assessed by analyzing how AHS cases were distributed across different risk areas. The maps' effectiveness in covering these cases was evaluated using the ratio capability to cover (RCC) values of the models [45]. RCC was calculated as the covered number of AHS cases divided by the area of the high- and very high-risk regions on the risk map. A higher RCC value indicates a greater level of prediction accuracy.

## 3. Results

### 3.1. Results of Data Collection

From our comprehensive data collection efforts spanning 2005–2022, we identified 217 occurrences of AHS. Of these, 199 instances were recorded in Africa and 18 in Asia (details are provided in *Supplementary file 1*). Our data compilation included extensive records of various vector species: 1,307 midges (including *C*. *imicola*, *C*. *bolitinos*, *C*. *variipennis*, and *C*. *brevitarsis*), 21,677 mosquitoes (*A. aegypti*, *A. stephensi*, and *C. pipiens*), 1,005 ticks (*H. dromedarii* and *R. sanguineus*), and 22,821 biting flies (*S. calcitrans* and *Tabanus* spp.), as detailed in *Supplementary file 2*.

### 3.2. Results of Climate-Based Zonal Segregation

**Figure 2:**
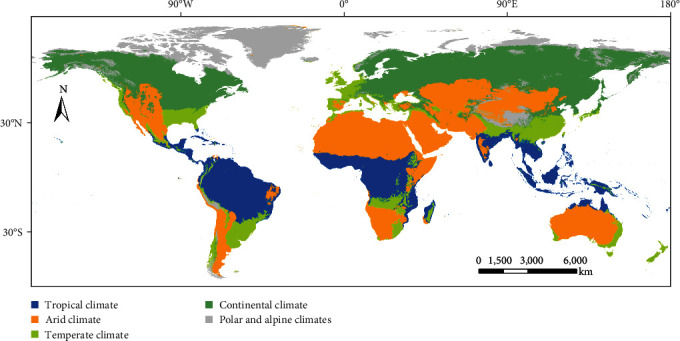
Climate pattern-based classification of the study area.

Our analysis led to the creation of a climate classification map, categorizing regions into five groups: tropical, arid, temperate, continental, and polar/alpine (Figure 2). This classification resulted in a total of 20 distinct prediction units based on geographical regions and climate zones. Specifically, Africa comprises three units (tropical, arid, and temperate), Asia has five units (encompassing all five climate categories), Australasia is represented by three units (tropical, arid, and temperate), Europe by four units (excluding the arid category), and the Americas by five units (including all climate categories).

### 3.3. Modeling Outcomes for AHS Distribution

#### 3.3.1. Data Postfiltering

Post data refinement, we identified 116 presence points of AHS. The modeling was conducted in various African regions (tropical, arid, and temperate) and tropical Asia. This allowed us to analyze AHS distribution in these diverse climatic zones.

#### 3.3.2. Key Predictors in Different Regions

The model identified region-specific key predictors for AHS distribution:

Tropical Africa: Land cover, elevation, and September mean air temperature (temp9) emerged as primary predictors.

Tropical Asia: Key predictors included land cover, February mean air temperature (temp2), February precipitation (prec2), and July precipitation (prec7).

African Arid region: In this region, land cover, elevation, October mean air temperature (temp10), and September maximum air temperature (tmax9) were significant.

African Temperate region: Here, the model highlighted land cover, elevation, precipitation of the driest month (bio14), and February precipitation (prec2) as crucial factors.

#### 3.3.3. Model Reliability and Variable Contribution

**Figure 3:**
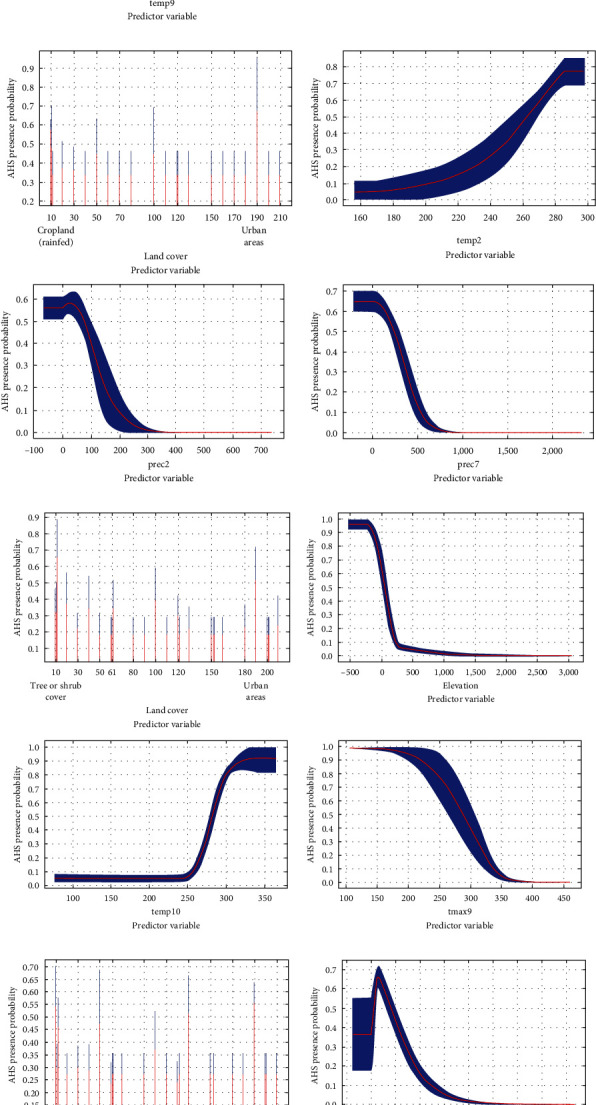
Response curves of the AHS model in various climate regions. (This figure illustrates the response curves for the AHS model across different climate regions. The panels represent: (a) the tropical climate region of Africa, (b) the tropical climate region of Asia, (c) the arid climate region of Africa, and (d) the temperate climate region of Africa. Each curve displays the mean response (in red) and the associated mean standard deviation (in blue), providing insights into the model's sensitivity to environmental variables in each region.).

The VIF values for predictor variables ranged from 1.000 to 4.551, satisfying the low multicollinearity prerequisite (<10). The AUC values varied between 0.941 and 0.986, demonstrating a high degree of model reliability.  Table 1 in the manuscript details the contribution rates, AUC values, and VIF values of the main variables incorporated into the model. Additionally, the response curves for each model are illustrated in Figure 3. For ease of reference, an abbreviation table for the climate variables is provided in *Supplementary file 3*.

### 3.4. Modeling Outcomes for AHSV Vector Distribution

#### 3.4.1. Data Postfiltering

After meticulous filtering, our dataset encompassed presence points for various AHSV vectors: 1,229 midges, 9,751 mosquitoes, 796 ticks, and 10,574 biting flies. This comprehensive dataset facilitated the development of a total of 82 individual models tailored for niche modeling of AHSV vectors. These comprised 16 models for midges, 27 for mosquitoes, 15 for ticks, and 24 for biting flies.

#### 3.4.2. Model Robustness and Key Predictors

The VIF values for the predictors in these models ranged from 1.000 to 6.795, indicating low multicollinearity and, hence, reliability of the models (<10 VIF threshold). The AUC values varied from 0.820 to 0.997, reflecting the robustness of the models. The primary environmental variables influencing vector distribution are detailed in Tables 2, 3, 4, and 5. The response curves for each vector model are available in *Supplementary file 4*.

### 3.5. Identifying High-Risk Zones for AHS by Integrated Modeling Approach

**Figure 4:**
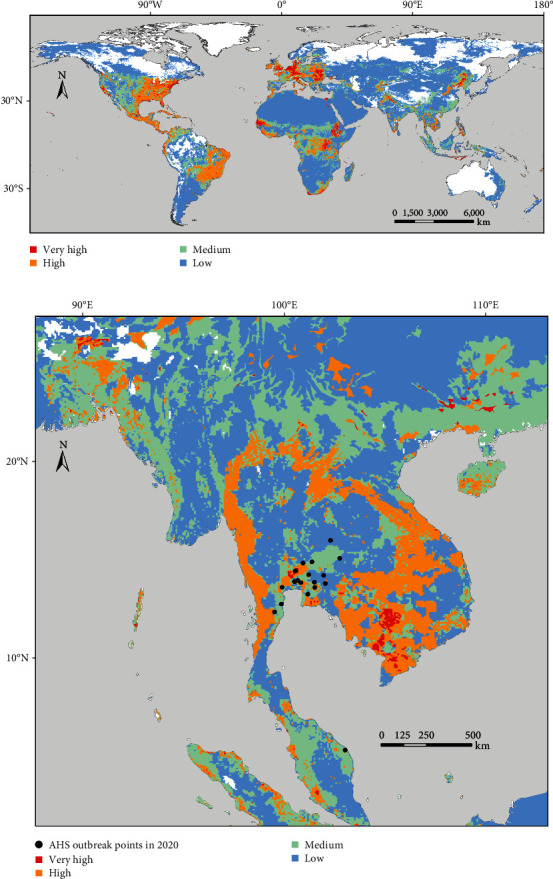
AHS risk map including only AHS outbreak data before 2020: (a) global AHS risk map and (b) enlarged map of Thailand and Malaysia featuring the 2020 outbreak points.

**Figure 5:**
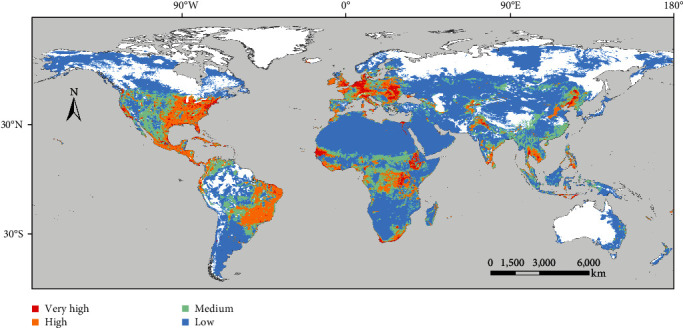
Global distribution of high-risk zones for AHS.

Figures 4 and 5 display the AHS risk maps, one incorporating solely AHS outbreak data predating the Thailand and Malaysia outbreak (Figure 4) and the other encompassing the entire outbreak data (Figure 5). The comprehensive AHS risk map, incorporating global outbreak data, offers an intuitive depiction of AHS risk zones worldwide. Notably, a significant portion of Europe, excluding its northern areas, was identified as high-risk. In Asia, parts of the Indian subcontinent, South Asia, Southeast Asia, and East Asia, along with some regions of the Middle East, showed elevated risk levels. For Africa, the model highlighted the middle and southern regions as particularly vulnerable. In the Americas, extensive areas of the United States, Central America, and specific regions in South America, such as Brazil, were marked as high-risk zones.

### 3.6. Assessment of Predictive Accuracy of AHS Risk Map

The proportion of AHS cases across different risk areas and RCC values in the two AHS risk maps is detailed in Table 6. Risk map1 represents an AHS risk map utilizing the AHS suitability map based on outbreak data predating 2020, while Risk map2 is based on all AHS outbreak data.

The distribution of cases in high- or very high-risk areas was 86.49% and 90.99%, respectively, indicating the high accuracy of the AHS risk maps. In Risk map1, the risk area classification for the 18 outbreak points in Thailand and Malaysia was as follows: 12 case points were situated in medium-, high-, and very high-risk areas (one in a very high-risk area, four in a high-risk area, and seven in a medium-risk area), with three out of the six cases in low-risk areas being in close proximity to medium-risk areas within a 5-km radius (Figure 4(b)). Based on the RCC value, it can be inferred that Risk map2 demonstrates superior prediction accuracy compared to Risk map1.

## 4. Discussion

Our research offers a novel perspective on the environmental factors influencing AHS in diverse climatic regions. We have identified key environmental variables that correlate significantly with AHS occurrences in both Africa's varied climates and Asia's tropical regions. Urban areas, cropland, and mosaic tree and shrub areas, which were predicted to be the main land cover types in our modeling, were suitable habitats for racehorses and backyard horses, which are the main infecting animals, and midges, which are the main arthropod vectors [46, 47, 48]. Temperature- and precipitation-related climate variable layers selected as predictors in different climate zones contributed significantly to the frequency of occurrence of AHS according to the climate characteristics of the region. Considering that AHS occurrence is seasonal (late summer/fall) and easily occurs in areas with a climate characterized by drought followed by heavy rain, it is not surprising that climate factors related to precipitation and temperature were selected as main components of the AHS model [15]. *C*. *imicola* thrives in moist soil, and in years with heavy rain, its population can increase more than 200-fold [49], and the temperature between 12.5 and 29°C is the critical temperature that has the greatest influence on adult activity [50]. The research by Shan Gao et al. and Danica Liebenberg et al., which emphasizes factors like annual temperature ranges and precipitation, resonates with our results, especially regarding precipitation during crucial months and temperature variations [17, 42]. These similarities validate our results and highlight the complex interplay between environmental factors, vertebrate hosts, and vector arthropods of AHSV, echoing previous research [46, 47, 48, 51, 52].

In recognizing the limitations of our study, it's important to note the exclusion of certain equines, including wild species and donkeys, and the reliance on standard climate data. This limitation arises from the challenges in data availability and the difficulty of monitoring wild animal populations [53]. The significant role of donkeys, known to be potential carriers or reservoirs of AHS due to their large population size and wide distribution, cannot be overlooked [54]. Excluding donkey data might lead to an underestimation of risk areas or a failure to fully capture the disease's transmission dynamics. Future predictions, therefore, require reassessment with more comprehensive and reliable data on donkeys and other wild equines when such data become available.

In our study, we relied on standard climate data rather than specific microclimate data, acknowledging the limitations this approach may impose. Microclimate data often provide more accurate predictions for the spread of vector-borne diseases [55], as they account for the specific environmental preferences of vector arthropods like *Culicoides* spp., a known AHSV vector [56, 57, 58, 59]. These vectors select resting areas that offer favorable conditions, significantly influencing disease transmission dynamics [60]. The utilization of microclimate data is essential but also challenging, as it requires access to detailed environmental variables [61, 62]. Our results, therefore, might overlook certain low-risk areas that actually harbor vectors, leading to discrepancies in risk assessment. Future research should aim to include more specific, reliable, and comprehensive data, emphasizing the need for advancements in data collection methodologies.

Our research plays a crucial role in shaping control and management strategies for AHS in high-risk areas. The results underscore the importance of enhancing targeted surveillance efforts, implementing early detection strategies, and adopting proactive control measures in previously unidentified high-risk areas. Furthermore, it emphasizes the necessity of reassessing decision-making processes related to resource allocation, vaccination campaigns, and biosecurity protocols to curb the transmission of AHS in these specific regions. Additionally, we draw attention to the potential impact of climate change in intensifying AHSV vector habitats. Our findings underscore the need for future studies to incorporate more detailed environmental data to better understand and mitigate AHS risks.

The increasing prevalence of AHS is a significant environmental concern, largely attributed to climate change. Rising temperatures are expanding the habitats of AHSV vectors, notably *C*. *imicola*, leading to a northward spread and enhanced AHSV transmission [1, 12, 28, 63, 64]. This change, coupled with the identification of new vector species, suggests an increased risk of AHS in broader areas [65, 66]. Recent outbreaks of bluetongue virus, transmitted by the same vectors, further highlight the growing concern about AHSV expansion [67, 68]. Our findings emphasize the urgent need to consider these environmental changes in AHS management strategies.

In conclusion, our research contributes significantly to understanding AHS and its environmental drivers. Future studies should aim to integrate broader datasets, including microclimatic variables, and consider socioeconomic factors for a more comprehensive approach. Collaboration with various stakeholders is vital for the practical application of our findings in disease management and control.

## 5. Conclusion

AHS presents a significant challenge to the global equine industry, impacting both health and economic aspects. Our study highlights the profound effect of climate change, particularly the frequency of extreme climatic events including temperature and humidity variations, on the transmission dynamics of diseases like AHS. In our research, we focused on overcoming the challenges associated with identifying key environmental factors and determining the appropriate geographic scale for a comprehensive global understanding of AHS. Our aim was to bridge existing knowledge gaps and elucidate the fundamental principles governing AHS transmission. This study establishes a solid foundation for understanding the intricate dynamics of AHS and offers practical pathways for conservation efforts. It emphasizes the urgent need for environmentally conscious strategies to protect horse populations and the industries dependent on them. By highlighting the relationship between environmental factors, vector presence, and AHS transmission, our research underscores the importance of a holistic approach to disease mitigation. In conclusion, the findings of our study not only contribute to the scientific understanding of AHS but also serve as a guide for policymakers and practitioners in developing effective strategies for disease management and prevention, tailored to the specific environmental conditions and challenges faced in different regions around the world.

## Figures and Tables

**Table 1:** Contribution rates of predictors and AUC values in the AHS model.

Region	LC.	Elev.	Climate variables							AUC	VIF
			temp2	temp9	temp10	tmax9	bio14	prec2	prec7		
1_Africa	49.3	23.5	—	27.2	—	—	—	—	—	0.941	1.000–1.000
1_Asia	29.4	—	32.8	—	—	—	—	24.3	13.5	0.951	1.074–1.526
2_Africa	14.0	48.1	—	—	12.3	25.6	—	—	—	0.986	1.011–4.551
3_Africa	11.3	32.2	—	—	—	—	40.1	16.4	—	0.982	1.052–3.509

1, tropical climate region; 2, arid climate region; 3, temperate climate region; LC., land cover; Elev., elevation.

**Table 2:** Contribution rates of predictors and AUC values in the distribution models for *C*. *imicola*, *C*. *bolitinos*, *C*. *variipennis*, and *C*. *brevitarsis*.

Species	Region	LC.	Elev.	bio4	bio8	temp1	temp6	temp7	tmax1	tmin3	tmin9	tmin11	bio12	prec7	AUC	VIF
C.i.	1_Af	46.0	15.8	—	13.1	—	—	—	—	—	25.1	—	—	—	0.906	1.015–2.350
	1_As	42.2	10.4	36.8	—	—	—	10.6	—	—	—	—	—	—	0.966	1.230–1.654
	2_Af	61.9	10.8	—	—	—	27.3	—	—	—	—	—	—	—	0.881	1.000–1.000
	2_As	61.1	—	—	—	28.4	—	—	10.5	—	—	—	—	—	0.974	1.000–1.000
	2_Eu	19.9	—	—	—	33.2	—	—	—	46.9	—	—	—	—	0.968	1.000–1.000
	3_Af	38.4	19.8	—	—	—	—	—	—	—	—	—	—	41.8	0.893	1.000–1.000
	3_As	23.5	20.1	—	—	—	—	—	22.7	—	—	—	33.7	—	0.962	1.077–1.223
	3_Eu	10.8	—	—	—	—	—	—	—	—	—	16.9	—	72.3	0.937	1.000–1.000

	Region	LC.		Elev.	bio5		bio8		temp6	temp7		tmax4	bio14		AUC	VIF

C.bo.	1_Af	30.8		10.3	38.7		20.2		—	—		—	—		0.973	1.101–4.511
	2_Af	19.2		23.3	—		—		47.3	—		—	10.2		0.989	1.098–3.621
	3_Af	59.9		14.7	—		—		—	11.5		13.9	—		0.905	1.005–1.854

	Region	LC.		Elev.	temp10		tmax1		tmin6	bio14		prec7	prec11		AUC	VIF

C.v.	2_nAm	74.5		14.6	—		10.9		—	—		—	—		0.956	1.000–1.000
	3_nAm	24.4		—	—		—		—	14.4		61.2	—		0.939	1.000–1.000
	4_nAm	23.3		—	24.4		—		38.6	—		—	13.7		0.977	1.008–2.020

	Region	LC.			tmin3			tmin11		prec9			prec11		AUC	VIF

C.br.	1_As	28.5			—			23.2		48.3			—		0.950	1.000–1.000
	1_Au	22.6			35.7			—		—			41.7		0.972	1.000–1.000

C.i., *C. imicola*; C.bo., *C. bolitinos*; C.v., *C. variipennis*; C.br., *C. brevitarsis*. 1, tropical climate region; 2, arid climate region; 3, temperate climate region; 4, continental climate region. Af, Africa; As, Asia; Au, Australasia; Eu, Europe; nAm, North America; LC., land cover; Elev., elevation.

**Table 3:** Contribution rates of predictors and AUC values in the distribution models for *A. aegypti*, *A. stephensi*, and *C. pipiens*.

Species	Region	LC.	Elev.	temp5	temp6	temp7	temp8	temp9	temp12	tmax1	tmax2	tmax7	tmax10	tmin3	tmin4	tmin11	prec7	prec8	prec9	prec11	AUC	VIF
A.a.	1_Af	71.6	16.5	—	—	—	—	11.9	—	—	—	—	—	—	—	—	—	—	—	—	0.892	1.000–1.000
	1_Am	72.8	—	—	—	—	—	—	—	—	—	—	—	—	—	—	—	11.5	—	15.7	0.861	1.000–1.000
	1_As	74.7	—	11.9	—	—	—	—	—	—	—	—	—	—	13.4	—	—	—	—	—	0.820	1.000–1.000
	1_Au	68.2	—	—	20.3	—	—	—	—	—	—	—	—	—	—	—	—	—	11.5	—	0.903	1.000–1.000
	2_Af	59.1	28.5	—	—	—	—	—	—	—	—	—	—	—	—	12.4	—	—	—	—	0.962	1.000–1.000
	2_Am	27.3	—	—	28.9	—	—	—	—	—	—	—	—	43.8	—	—	—	—	—	—	0.922	1.000–1.000
	2_As	63.2	—	—	—	—	—	—	—	26.7	—	10.1	—	—	—	—	—	—	—	—	0.974	1.000–1.000
	3_Af	66.3	15.1	—	—	—	18.6	—	—	—	—	—	—	—	—	—	—	—	—	—	0.925	1.000–1.000
	3_Am	33.0	12.7	—	—	—	24.3	—	—	—	30.0	—	—	—	—	—	—	—	—	—	0.883	1.023–1.884
	3_As	40.7	—	—	—	18.9	—	—	40.4	—	—	—	—	—	—	—	—	—	—	—	0.913	1.000–1.000
	3_Au	35.6	—	—	—	—	29.8	—	—	—	13.4	—	—	—	—	—	21.2	—	—	—	0.940	1.004–2.336
	4_nAm	78.1	—	—	—	—	—	—	—	—	—	—	21.9	—	—	—	—	—	—	—	0.983	—

	Region	LC.		Elev.			bio1	temp9		temp11		tmax12		tmin11		bio16		prec11			AUC	VIF

A.s.	1_As	57.5		13.2			—	—		—		—		—		29.3		—			0.951	1.000–1.000
	1_Au	60.4		—			18.4	—		—		—		—		—		21.2			0.943	1.000–1.000
	2_As	72.8		—			—	15.0		—		12.2		—		—		—			0.997	1.000–1.000
	2_Au	58.6		—			—	—		13.3		—		28.1		—		—			0.925	1.000–1.000

	Region	LC.	Elev.	bio6		bio10	temp7		temp9	tmax1	tmax2	tmax9	tmax10	tmax11	tmin2	tmin8	bio18	bio19	prec9	prec12	AUC	VIF

C.p.	1_Af	79.8	—	—		—	—		20.2	—	—	—	—	—	—	—	—	—	—	—	0.961	—
	1_As	85.0	—	—		—	—		—	—	—	—	—	—	—	15.0	—	—	—	—	0.885	—
	2_Af	63.6	14.6	—		—	—		—	—	—	—	—	—	—	—	—	—	21.8	—	0.953	1.000–1.000
	2_Am	68.5	—	—		—	—		—	—	—	—	—	13.9	—	—	—	17.6	—	—	0.941	1.000–1.000
	2_As	40.3	—	—		—	—		—	—	—	—	—	—	22.5	—	—	37.2	—	—	0.962	1.000–1.000
	3_Af	81.6	—	—		—	—		—	—	—	—	—	—	—	—	18.4	—	—	—	0.956	—
	3_Am	45.8	21.9	—		—	—		—	32.3	—	—	—	—	—	—	—	—	—	—	0.962	1.000–1.000
	3_Eu	—	26.6	—		—	18.0		—	—	30.0	—	—	—	—	—	—	—	—	25.4	0.925	1.087–6.795
	4_As	20.1	15.6	—		—	52.3		—	—	—	—	12.0	—	—	—	—	—	—	—	0.927	1.027–1.988
	4_Eu	12.4	—	87.6		—	—		—	—	—	—	—	—	—	—	—	—	—	—	0.942	—
	4_nAm	49.4	—	—		25.3	—		—	—	—	25.3	—	—	—	—	—	—	—	—	0.966	1.000–1.000

A.a., *A. aegypti*; A.s., *A. stephensi*; C.p., *C. pipiens*. 1, tropical climate region; 2, arid climate region; 3, temperate climate region; 4, continental climate region. Af, Africa; Am, America; nAm, North America; As, Asia; Au, Australasia; Eu, Europe; LC., land cover; Elev., elevation.

**Table 4:** Contribution rates of predictors and AUC values in the distribution models for *H. dromedarii* and *R. sanguineus*.

Species	Region	LC.	Elev.	bio9	bio11	temp6	temp7	temp9	temp11	tmax1	tmax6	tmax8	tmin1	tmin2	prec6	prec7	prec8	prec12	AUC	VIF
R.s.	1_Af	87.2	12.8	—	—	—	—	—	—	—	—	—	—	—	—	—	—	—	0.864	—
	1_Am	84.0	—	—	—	—	—	—	16.0	—	—	—	—	—	—	—	—	—	0.934	—
	1_As	83.9	—	16.1	—	—	—	—	—	—	—	—	—	—	—	—	—	—	0.904	—
	2_Af	74.1	—	—	—	15.1	—	—	—	—	10.8	—	—	—	—	—	—	—	0.975	1.000–1.000
	2_Am	65.7	—	—	15.9	—	18.4	—	—	—	—	—	—	—	—	—	—	—	0.949	1.000–1.000
	2_As	49.2	—	—	—	—	—	—	—	39.6	—	—	11.2	—	—	—	—	—	0.916	1.000–1.000
	2_Au	51.4	—	—	—	28.7	—	—	—	—	—	—	—	19.9	—	—	—	—	0.922	1.000–1.000
	3_Af	49.5	28.6	—	—	—	—	—	—	11.4	—	—	—	—	10.5	—	—	—	0.930	1.022–1.963
	3_Am	81.9	—	—	—	—	—	18.1	—	—	—	—	—	—	—	—	—	—	0.922	—
	3_As	55.5	—	—	—	—	—	20.7	—	—	—	—	—	—	—	23.8	—	—	0.933	1.000–1.000
	3_Au	74.3	—	—	—	—	—	—	—	—	—	—	—	—	—	—	25.7	—	0.956	—
	3_Eu	61.1	—	—	—	—	—	—	—	—	—	16.0	—	—	—	—	—	22.9	0.903	1.000–1.000
	4_Eu	83.8	—	—	—	—	—	—	—	—	—	—	—	16.2	—	—	—	—	0.936	—
	4_nAm	83.3	—	—	—	—	—	—	—	—	—	—	—	—	—	—	—	16.7	0.986	—

H.d.	Region	LC.						temp1					prec7						AUC	VIF
	2_As	36.5						38.1					25.4						0.979	1.000–1.000

H.d., *H. dromedarii*; R.s., *R. sanguineus*. 1, tropical climate region; 2, arid climate region; 3, temperate climate region; 4, continental climate region. Af, Africa; Am, America; nAm, North America; As, Asia; Au, Australasia; Eu, Europe; LC., land cover; Elev., elevation.

**Table 5:** Contribution rates of predictors and AUC values in the distribution models for *S. calcitrans* and *Tabanus* spp.

Species/genus	Region	LC.	Elev.	bio8	bio11	temp1	temp6	temp7	tmax4	tmax6	tmax8	tmax9	tmax11	tmin1	tmin2	tmin7	bio14	bio19	prec1	prec2	prec7	AUC	VIF
S.c.	1_Af	53.4	—	16.4	—	—	—	30.2	—	—	—	—	—	—	—	—	—	—	—	—	—	0.977	1.000–1.000
	1_Am	55.3	—	—	—	—	—	—	—	—	—	—	28.3	—	—	—	—	—	16.4	—	—	0.942	1.000–1.000
	1_As	88.9	—	—	—	—	—	—	—	—	—	—	—	—	11.2	—	—	—	—	—	—	0.947	—
	2_Af	23.4	22.1	—	—	—	54.5	—	—	—	—	—	—	—	—	—	—	—	—	—	—	0.989	1.000–1.000
	2_Am	84.8	—	—	—	—	—	—	—	15.2	—	—	—	—	—	—	—	—	—	—	—	0.890	—
	2_As	45.0	—	—	—	—	—	—	—	—	—	—	—	16.3	—	—	—	38.7	—	—	—	0.946	1.000–1.000
	2_Au	43.2	—	—	—	—	—	—	—	—	11.7	—	—	—	45.1	—	—	—	—	—	—	0.955	1.000–1.000
	3_Af	37.7	19.9	—	—	—	—	—	20.5	—	—	—	—	—	—	—	—	—	—	—	21.9	0.910	1.004–1.557
	3_Am	66.6	—	—	—	13.5	—	19.9	—	—	—	—	—	—	—	—	—	—	—	—	—	0.871	1.000–1.000
	3_Eu	22.4	37.3	—	—	—	—	—	—	—	—	—	—	—	—	40.3	—	—	—	—	—	0.914	1.000–1.000
	4_Eu	19.8	—	—	—	—	—	—	—	—	—	—	—	58.4	—	—	—	—	—	21.8	—	0.937	1.000–1.000
	4_nAm	21.0	—	—	31.7	—	—	—	—	—	—	34.6	—	—	—	—	12.7	—	—	—	—	0.937	1.006–2.575

	Region	LC.	Elev.	bio6	bio11	temp1	temp6	temp7	temp10	tmax1	tmax2	tmax6	tmax7	tmax9	tmin1	tmin3	tmin9	prec2	prec3	prec7		AUC	VIF

T.	1_Af	59.5	29.1	—	—	—	—	—	—	—	—	—	—	—	—	11.4	—	—	—	—		0.857	1.000–1.000
	1_Am	29.8	15.5	—	—	—	—	—	16.5	—	—	—	—	—	—	—	13.9	—	24.3	—		0.877	1.031–6.744
	1_As	53.7	—	—	—	—	—	—	—	—	—	—	22.8	—	—	—	—	23.5	—	—		0.860	1.000–1.000
	2_Af	45.1	—	—	—	—	—	—	—	—	—	39.4	—	—	—	—	—	—	—	15.5		0.924	1.000–1.000
	2_Am	44.3	—	—	—	23.5	—	—	—	—	—	—	32.2	—	—	—	—	—	—	—		0.863	1.000–1.000
	2_As	50.9	—	—	—	20.3	—	—	—	—	—	28.8	—	—	—	—	—	—	—	—		0.887	1.000–1.000
	2_Au	63.0	—	—	—	—	—	—	—	—	—	—	—	—	37.0	—	—	—	—	—		0.902	—
	3_Af	43.6	12.9	—	—	—	—	—	—	—	—	—	—	—	—	—	—	—	—	43.5		0.852	1.000–1.000
	3_Am	24.7	—	—	—	—	—	49.8	—	25.5	—	—	—	—	—	—	—	—	—	—		0.889	1.000–1.000
	3_Eu	—	25.9	—	—	—	50.0	—	—	—	24.1	—	—	—	—	—	—	—	—	—		0.874	1.022–3.545
	4_Eu	—	—	56.3	—	—	—	—	—	—	—	—	—	—	43.7	—	—	—	—	—		0.900	1.000–1.000
	4_nAm	21.1	—	—	46.5	—	—	—	—	—	—	—	—	32.4	—	—	—	—	—	—		0.906	1.000–1.000

S.c., *S. calcitrans*; T., *Tabanus* spp. 1, tropical climate region; 2, arid climate region; 3, temperate climate region; 4, continental climate region. Af, Africa; Am, America; nAm, North America; As, Asia; Au, Australasia; Eu, Europe; LC., land cover; Elev., elevation.

**Table 6:** Proportion of AHS cases across different risk areas and RCC values of the two AHS risk maps.

AHS risk map	Proportion of AHS cases distributed among the risk classes (%)				RCC × 10^7^
	Very high	High	Medium	Low	
Risk map1	77.03	9.46	5.40	8.11	8.922
Risk map2	78.38	12.61	3.60	5.41	9.391

## Data Availability

The data that supports the findings of this study are available in the supplementary material of this article.

## Supplementary Materials

Supplementary 1 File 1: African horse sickness country data.

Supplementary 2 File 2: distribution data of African horse sickness virus vector species.

Supplementary 3 File 3: abbreviations for climate variables utilized in the model.

Supplementary 4 File 4: the response curves of AHSV vector distribution model.
