# Supplementary material for: Environmental and Historical Determinants of African Horse Sickness: Insights from Predictive Modeling
Source: Transbound Emerg Dis. 2024 Aug 13;2024:5586647. doi: 10.1155/2024/5586647 (PMC12017013; doi:10.1155/2024/5586647)
Supplement: Supplementary 1 — File 1: African horse sickness country data. [file 5586647.f1.docx]

African Horse Sickness(AHS) country data

| № | Country | Serotype | equidae(domestic) | | plains zebra(wild) | | Latitude | Longitude |
| --- | --- | --- | --- | --- | --- | --- | --- | --- |
|  |  |  | Cases | Deaths | Cases | Deaths |  |  |
| 1 | Nigeria | Untyped or partially typed | 10 | 6 |  |  | 6.5972 | 3.8577 |
| 2 | Nigeria | 2 | 42 | 42 |  |  | 6.4231 | 3.5689 |
| 3 | South Africa | Not typed | 1 | 1 |  |  | -32.1 | 18.9 |
| 4 | South Africa | Not typed | 24 | 8 |  |  | -32 | 19 |
| 5 | South Africa | Not typed | 1 | 1 |  |  | -32.1 | 19.1 |
| 6 | South Africa | Not typed | 2 | 1 |  |  | -31.9 | 19 |
| 7 | South Africa | 1 | 1 | 0 |  |  | -33.6 | 18.9 |
| 8 | South Africa | 1 | 1 | 0 |  |  | -33.6 | 19 |
| 9 | South Africa | 1 | 1 | 0 |  |  | -33.6 | 19 |
| 10 | South Africa | 1 | 2 | 1 |  |  | -33.6 | 18.8 |
| 11 | South Africa | 1 | 1 | 0 |  |  | -33.5 | 18.9 |
| 12 | South Africa | 1 | 10 | 1 |  |  | -33.6 | 18.9 |
| 13 | South Africa | 1 | 1 | 0 |  |  | -33.7 | 19 |
| 14 | South Africa | 1 | 4 | 1 |  |  | -33.7 | 19 |
| 15 | South Africa | 1 | 1 | 0 |  |  | -33.8462 | 19.96263 |
| 16 | South Africa | 1 | 2 | 0 |  |  | -33.83319 | 19.7636 |
| 17 | South Africa | 1 | 6 | 1 |  |  | -33.84211 | 19.83086 |
| 18 | South Africa | 1 | 2 | 0 |  |  | -33.7781 | 19.6791 |
| 19 | South Africa | 1 | 1 | 0 |  |  | -33.8397 | 19.92478 |
| 20 | South Africa | 1 | 1 | 0 |  |  | -33.86184 | 19.70181 |
| 21 | South Africa | 1 | 6 | 0 |  |  | -33.81348 | 19.78239 |
| 22 | South Africa | 1 | 3 | 0 |  |  | -33.83858 | 19.73688 |
| 23 | South Africa | 1 | 1 | 1 |  |  | -33.84211111 | 19.83086111 |
| 24 | South Africa | 1 | 2 | 0 |  |  | -33.2853 | 19.1451 |
| 25 | South Africa | 1 | 3 | 1 |  |  | -33.67511 | 18.94196 |
| 26 | South Africa | 1 | 1 | 0 |  |  | -32.86 | 18.66 |
| 27 | South Africa | 1 | 1 | 0 |  |  | -33.57414 | 19.07234 |
| 28 | South Africa | 1 | 1 | 0 |  |  | -33.015 | 18.997 |
| 29 | South Africa | 1 | 1 | 0 |  |  | -33.18102 | 19.01002 |
| 30 | South Africa | 1 | 1 | 0 |  |  | -33.17845 | 19.01073 |
| 31 | South Africa | 1 | 2 | 0 |  |  | -33.1784 | 19.00394 |
| 32 | South Africa | 1 | 4 | 0 |  |  | -33.16764 | 19.02366 |
| 33 | South Africa | 1 | 2 | 0 |  |  | -33.181 | 19.01 |
| 34 | South Africa | 1 | 6 | 1 |  |  | -33.181 | 19.01 |
| 35 | South Africa | 1 | 1 | 0 |  |  | -33.181 | 19.01 |
| 36 | South Africa | 1 | 1 | 0 |  |  | -33.18101 | 19.01001 |
| 37 | South Africa | 1 | 1 | 0 |  |  | -33.56897 | 19.05458 |
| 38 | South Africa | 1 | 1 | 0 |  |  | -33.00692 | 18.99967 |
| 39 | South Africa | 1 | 5 | 1 |  |  | -33.565 | 18.938 |
| 40 | South Africa | 1 | 5 | 0 |  |  | -33.181 | 19.01 |
| 41 | South Africa | 1 | 5 | 2 |  |  | -33.1854 | 18.9498 |
| 42 | South Africa | 1 | 2 | 1 |  |  | -33.181 | 19.01 |
| 43 | South Africa | 1 | 1 | 1 |  |  | -33.1786 | 18.9952 |
| 44 | South Africa | 1 | 1 | 1 |  |  | -33.0219 | 18.9963 |
| 45 | South Africa | 1 | 1 | 0 |  |  | -33.181 | 19.01 |
| 46 | South Africa | 1 | 2 | 2 |  |  | -33.1775 | 19.0077 |
| 47 | South Africa | 1 | 1 | 1 |  |  | -33.1866 | 18.9933 |
| 48 | South Africa | 1 | 1 | 0 |  |  | -33.048 | 18.9652 |
| 49 | South Africa | 1 | 4 | 0 |  |  | -33.181 | 19.01 |
| 50 | South Africa | 1 | 1 | 0 |  |  | -33.0174 | 19.0084 |
| 51 | South Africa | 1 | 17 | 0 |  |  | -33.111 | 19.02406 |
| 52 | South Africa | 1 | 2 | 0 |  |  | -33.1115 | 19.02406 |
| 53 | South Africa | Not typed | 1 | 0 |  |  | -33.685 | 18.455 |
| 54 | South Africa | Not typed | 3 | 1 |  |  | -33.69071 | 18.46883 |
| 55 | South Africa | 1 | 1 | 1 |  |  | -33.52039 | 18.59587 |
| 56 | South Africa | 1 | 2 | 2 |  |  | 33.50774 | 18.45011 |
| 57 | South Africa | 1 | 1 | 1 |  |  | -33.50596 | 18.44884 |
| 58 | South Africa | 1 | 1 | 1 |  |  | -33.50406 | 18.44343 |
| 59 | South Africa | 1 | 1 | 1 |  |  | -33.53703 | 18.52724 |
| 60 | South Africa | 1 | 1 | 1 |  |  | -33.46481 | 18.47264 |
| 61 | South Africa | 1 | 1 | 1 |  |  | -33.50589 | 18.44868 |
| 62 | South Africa | 1 | 1 | 1 |  |  | -33.61694 | 18.52919 |
| 63 | South Africa | 1 | 1 | 0 |  |  | -33.53989 | 18.52347 |
| 64 | South Africa | 1 | 1 | 1 |  |  | -33.50589 | 18.44868 |
| 65 | South Africa | 1 | 1 | 1 |  |  | -33.4965 | 18.44903 |
| 66 | South Africa | 1 | 1 | 1 |  |  | -33.54067 | 18.52236 |
| 67 | South Africa | 1 | 1 | 1 |  |  | 33.57973 | 18.57947 |
| 68 | South Africa | 1 | 1 | 1 |  |  | -33.50576 | 18.44414 |
| 69 | South Africa | 1 | 1 | 1 |  |  | -33.51062 | 18.47576 |
| 70 | South Africa | 1 | 1 | 1 |  |  | -33.4865 | 18.48886 |
| 71 | South Africa | 1 | 1 | 1 |  |  | -33.54099 | 18.52148 |
| 72 | South Africa | 1 | 1 | 1 |  |  | -33.50539 | 18.44894 |
| 73 | South Africa | 1 | 1 | 1 |  |  | -33.396 | 18.395 |
| 74 | South Africa | 1 | 1 | 1 |  |  | -33.52461 | 18.47303 |
| 75 | South Africa | 1 | 4 | 4 |  |  | -33.5095 | 18.50312 |
| 76 | South Africa | 1 | 5 | 5 |  |  | -33.51114 | 18.469 |
| 77 | South Africa | 1 | 1 | 1 |  |  | -33.47453 | 18.508 |
| 78 | South Africa | 1 | 2 | 1 |  |  | -33.4865 | 18.48886 |
| 79 | South Africa | 1 | 3 | 2 |  |  | -33.51122 | 18.47792 |
| 80 | South Africa | 1 | 1 | 1 |  |  | -33.51195 | 18.47246 |
| 81 | South Africa | 1 | 1 | 0 |  |  | -33.50059 | 18.44875 |
| 82 | South Africa | 1 | 1 | 0 |  |  | -33.4865 | 18.48886 |
| 83 | South Africa | 1 | 1 | 0 |  |  | -33.50619 | 18.44879 |
| 84 | South Africa | 1 | 1 | 1 |  |  | -33.50511 | 18.44814 |
| 85 | South Africa | 1 | 2 | 2 |  |  | -33.50828 | 18.47343 |
| 86 | South Africa | 1 | 1 | 0 |  |  | -33.45893 | 18.48732 |
| 87 | South Africa | 1 | 1 | 1 |  |  | -33.48636 | 18.4784 |
| 88 | South Africa | 1 | 3 | 3 |  |  | -33.50739 | 18.4515 |
| 89 | South Africa | 1 | 2 | 2 |  |  | -33.55119 | 18.49231 |
| 90 | South Africa | 1 | 2 | 2 |  |  | -33.48894 | 18.48699 |
| 91 | South Africa | 1 | 1 | 1 |  |  | -33.60518 | 18.51243 |
| 92 | South Africa | 1 | 1 | 1 |  |  | -33.51978 | 18.47997 |
| 93 | South Africa | 1 | 1 | 1 |  |  | -33.4865 | 18.4888 |
| 94 | South Africa | 1 | 1 | 1 |  |  | -33.51978 | 18.47997 |
| 95 | South Africa | 1 | 2 | 2 |  |  | -33.51386 | 18.48383 |
| 96 | South Africa | 1 | 4 | 3 |  |  | -33.5095 | 18.4683 |
| 97 | South Africa | 1 | 1 | 1 |  |  | -33.50739 | 18.4525 |
| 98 | South Africa | 1 | 1 | 1 |  |  | -33.51353 | 18.48406 |
| 99 | South Africa | 1 | 1 | 1 |  |  | -33.51119 | 18.47558 |
| 100 | South Africa | Other | 1 | 1 |  |  | -33.83 | 19.76 |
| 101 | South Africa | Other | 2 | 0 |  |  | -33.77 | 19.58 |
| 102 | South Africa | Other | 11 | 4 |  |  | -33.77 | 19.68 |
| 103 | Eswatini | 3 | 3 | 0 |  |  | -26.519 | 31.214 |
| 104 | Eswatini | 5 | 2 | 1 |  |  | -26.513987 | 31.269146 |
| 105 | Eswatini | 5 | 1 | 0 |  |  | -26.519201 | 31.214828 |
| 106 | Eswatini | 5 | 1 | 1 |  |  | -26.467 | 31.389 |
| 107 | Eswatini | 5 | 5 | 0 |  |  | -26.485693 | 31.191971 |
| 108 | Eswatini | 5 | 2 | 0 |  |  | -26.3562 | 31.52398 |
| 109 | Eswatini | 7 | 1 | 0 |  |  | -26.485693 | 31.191971 |
| 110 | Chad | Not typed | 159 | 114 |  |  | 13.3907 | 21.1892 |
| 111 | Chad | Not typed | 217 | 206 |  |  | 12.196 | 21.6057 |
| 112 | Cameroon | Pending | 124 | 36 |  |  | 10.339595 | 13.562534 |
| 113 | Cameroon | Pending | 148 | 8 |  |  | 10.339344 | 13.561828 |
| 114 | Swaziland | 7 | 1 | 1 |  |  | -26.485693 | 31.191971 |
| 115 | Swaziland | 2 | 2 | 2 |  |  | -26.499 | 31.344 |
| 116 | Swaziland | 2 | 1 | 0 |  |  | -26.519201 | 31.214828 |
| 117 | Swaziland | 4 | 2 | 1 |  |  | -26.364 | 31.124 |
| 118 | Swaziland | 4 | 1 | 1 |  |  | -26.419 | 31.214 |
| 119 | Swaziland | Not typed | 4 | 0 |  |  | -26.499 | 31.344 |
| 120 | Swaziland | Not typed | 7 | 3 |  |  | -26.499 | 31.344 |
| 121 | Swaziland | Not typed | 1 | 1 |  |  | -27.221 | 31.735 |
| 122 | Mozambique | Pending | 1 | 1 |  |  | -25.87642 | 32.6626 |
| 123 | Mozambique | Not typed | 1 | 1 |  |  | -25.9058 | 32.5812 |
| 124 | Mozambique | Not typed | 1 | 1 |  |  | -25.9058 | 32.5812 |
| 125 | Mozambique | Not typed | 2 | 2 |  |  | -25.89242259 | 32.557787 |
| 126 | Ghana | 2 | 30 | 30 |  |  | 5.61 | -0.18333 |
| 127 | Ethiopia | 2 | 50 | 5 |  |  | 6.916675 | 35.488331 |
| 128 | Ethiopia | 2 | 75 | 5 |  |  | 6.698028 | 35.739725 |
| 129 | Ethiopia | 2 | 50 | 40 |  |  | 6.733333 | 36.239019 |
| 130 | Ethiopia | 2 | 265 | 50 |  |  | 7.608094 | 36.083875 |
| 131 | Ethiopia | 2 | 300 | 200 |  |  | 7.595183 | 35.911614 |
| 132 | Ethiopia | 2 | 250 | 215 |  |  | 7.758644 | 35.488389 |
| 133 | Ethiopia | 2 | 300 | 250 |  |  | 7.104272 | 36.390881 |
| 134 | Ethiopia | 2 | 500 | 250 |  |  | 7.900311 | 35.610056 |
| 135 | Ethiopia | 2 | 200 | 153 |  |  | 6.889778 | 36.331222 |
| 136 | Ethiopia | 2 | 500 | 219 |  |  | 7.674653 | 35.728842 |
| 137 | Ethiopia | 2 | 500 | 222 |  |  | 7.395253 | 35.663433 |
| 138 | Ethiopia | 2 | 500 | 300 |  |  | 8.018767 | 35.829531 |
| 139 | Ethiopia | 2 | 150 | 60 |  |  | 7.441428 | 36.100542 |
| 140 | Ethiopia | 2 | 300 | 210 |  |  | 7.280886 | 35.610056 |
| 141 | Ethiopia | 2 | 60 | 6 |  |  | 7.120747 | 35.663247 |
| 142 | Senegal | Not typed | 1 | 1 |  |  | 16.0487 | -16.4757 |
| 143 | Senegal | Not typed | 1 | 1 |  |  | 15.1271 | -16.5745 |
| 144 | Senegal | Not typed | 2 | 2 |  |  | 15.0173 | -16.8159 |
| 145 | Senegal | Not typed | 2 | 2 |  |  | 15.0064 | -16.6623 |
| 146 | Senegal | Not typed | 1 | 1 |  |  | 14.9734 | -14.4788 |
| 147 | Senegal | Not typed | 1 | 1 |  |  | 15.3465 | -13.3267 |
| 148 | Senegal | Not typed | 1 | 1 |  |  | 15.6428 | -13.5352 |
| 149 | Senegal | Not typed | 1 | 1 |  |  | 15.6757 | -13.2718 |
| 150 | Senegal | Not typed | 5 | 5 |  |  | 14.8637 | -16.7171 |
| 151 | Senegal | Not typed | 5 | 5 |  |  | 14.6552 | -16.761 |
| 152 | Senegal | Not typed | 1 | 1 |  |  | 14.743 | -17.3096 |
| 153 | Senegal | Not typed | 1 | 1 |  |  | 14.7211 | -17.4742 |
| 154 | Senegal | Not typed | 2 | 2 |  |  | 16.4876 | -16.1466 |
| 155 | Senegal | Not typed | 1 | 1 |  |  | 16.4876 | -15.587 |
| 156 | Senegal | Not typed | 2 | 2 |  |  | 15.7634 | -13.4364 |
| 157 | Senegal | Not typed | 1 | 1 |  |  | 14.6991 | -16.75 |
| 158 | Senegal | Not typed | 1 | 1 |  |  | 16.6303 | -14.3471 |
| 159 | Senegal | Not typed | 1 | 1 |  |  | 16.5425 | -15.4992 |
| 160 | Senegal | Not typed | 1 | 1 |  |  | 14.0298 | -14.9286 |
| 161 | Senegal | Not typed | 4 | 4 |  |  | 14.6882 | -16.7391 |
| 162 | Senegal | Not typed | 15 | 15 |  |  | 14.8857 | -16.6952 |
| 163 | Senegal | Not typed | 1 | 1 |  |  | 14.0518 | -14.8409 |
| 164 | Senegal | Not typed | 1 | 1 |  |  | 13.8323 | -15.2359 |
| 165 | Senegal | Not typed | 1 | 1 |  |  | 14.0737 | -14.9835 |
| 166 | Senegal | Not typed | 9 | 9 |  |  | 14.9296 | -16.7061 |
| 167 | Senegal | Not typed | 1 | 1 |  |  | 16.5096 | -15.598 |
| 168 | Senegal | Not typed | 6 | 6 |  |  | 16.4876 | -15.565 |
| 169 | Senegal | Not typed | 28 | 28 |  |  | 15.149 | -12.9097 |
| 170 | Senegal | Not typed | 5 | 5 |  |  | 16.5973 | -14.38 |
| 171 | Senegal | Not typed | 1 | 1 |  |  | 15.4782 | -13.217 |
| 172 | Senegal | Not typed | 1 | 1 |  |  | 14.8198 | -14.1167 |
| 173 | Senegal | Not typed | 1 | 1 |  |  | 15.7086 | -13.3157 |
| 174 | Senegal | Not typed | 1 | 1 |  |  | 14.0847 | -15.4992 |
| 175 | Senegal | Not typed | 1 | 1 |  |  | 14.4468 | -15.2798 |
| 176 | Senegal | Not typed | 1 | 1 |  |  | 14.0737 | -14.8409 |
| 177 | Senegal | Not typed | 14 | 14 |  |  | 14.8857 | -16.7171 |
| 178 | Senegal | Not typed | 1 | 1 |  |  | 14.7211 | -17.2109 |
| 179 | Senegal | Not typed | 1 | 1 |  |  | 15.4233 | -13.7875 |
| 180 | Senegal | Not typed | 17 | 17 |  |  | 14.0847 | -15.4992 |
| 181 | Senegal | Not typed | 5 | 5 |  |  | 15.0832 | -16.5855 |
| 182 | Senegal | Not typed | 23 | 23 |  |  | 14.5016 | -16.6293 |
| 183 | Senegal | Not typed | 5 | 5 |  |  | 16.3779 | -16.2343 |
| 184 | Senegal | Not typed | 82 | 76 |  |  | 14.0518 | -15.5102 |
| 185 | Senegal | Not typed | 5 | 5 |  |  | 13.8433 | -12.5916 |
| 186 | Senegal | Not typed | 5 | 5 |  |  | 16.4437 | -16.2343 |
| 187 | Senegal | Not typed | 3 | 3 |  |  | 14.8527 | -16.7061 |
| 188 | Senegal | Not typed | 4 | 4 |  |  | 13.6458 | -13.7766 |
| 189 | Senegal | Not typed | 3 | 2 |  |  | 13.6897 | -12.6684 |
| 190 | Senegal | Not typed | 100 | 100 |  |  | 14.1066 | -15.5102 |
| 191 | Senegal | Not typed | 33 | 33 |  |  | 14.1066 | -15.5211 |
| 192 | Senegal | Not typed | 15 | 15 |  |  | 14.1286 | -15.0164 |
| 193 | Senegal | Not typed | 20 | 15 |  |  | 14.0957 | -14.7531 |
| 194 | Senegal | Not typed | 10 | 6 |  |  | 13.6677 | -12.7122 |
| 195 | Senegal | Not typed | 15 | 15 |  |  | 14.1725 | -15.1371 |
| 196 | Senegal | Not typed | 3 | 3 |  |  | 13.7116 | -13.5791 |
| 197 | Senegal | Not typed | 31 | 31 |  |  | 15.4343 | -14.8189 |
| 198 | Senegal | Not typed | 1 | 1 |  |  | 14.765 | -17.3974 |
| 199 | Senegal | Not typed | 7 | 7 |  |  | 14.7869 | -17.3096 |
| 200 | Thailand | 1 |  |  | 2 | 2 | 13.867235 | 101.52898 |
| 201 | Thailand | 1 | 1 | 1 |  |  | 15.08204 | 102.7882 |
| 202 | Thailand | 1 | 1 | 1 |  |  | 13.8422 | 100.8555 |
| 203 | Thailand | 1 | 1 | 1 |  |  | 13.9527 | 100.712 |
| 204 | Thailand | 1 | 1 | 1 |  |  | 13.893501 | 100.553017 |
| 205 | Thailand | 1 | 2 | 2 |  |  | 14.212221 | 101.994573 |
| 206 | Thailand | 1 | 8 | 8 |  |  | 13.600276 | 101.550335 |
| 207 | Thailand | 1 | 1 | 1 |  |  | 14.842423 | 100.966163 |
| 208 | Thailand | 1 | 4 | 4 |  |  | 14.4365784 | 100.593129 |
| 209 | Thailand | 1 | 45 | 34 |  |  | 14.903489 | 101.41446 |
| 210 | Thailand | 1 | 4 | 3 |  |  | 13.7998304 | 102.0759295 |
| 211 | Thailand | 1 | 1 | 1 |  |  | 15.998288 | 102.333338 |
| 212 | Thailand | 1 | 46 | 42 |  |  | 12.4759 | 99.581 |
| 213 | Thailand | 1 | 7 | 6 |  |  | 13.601978 | 99.94296 |
| 214 | Thailand | 1 | 6 | 5 |  |  | 13.25 | 101.206 |
| 215 | Thailand | 1 | 20 | 20 |  |  | 12.347 | 99.5728 |
| 216 | Thailand | 1 | 460 | 436 |  |  | 14.2442 | 101.2518 |
| 217 | Malaysia | 9 | 5 | 0 |  |  | 5.319832 | 103.059028 |
