# Supplementary material for: Environmental and Historical Determinants of African Horse Sickness: Insights from Predictive Modeling
Source: Transbound Emerg Dis. 2024 Aug 13;2024:5586647. doi: 10.1155/2024/5586647 (PMC12017013; doi:10.1155/2024/5586647)
Supplement: Supplementary 3 — File 3: abbreviations for climate variables utilized in the model. [file 5586647.f3.docx]

**Abbreviations for climate variables utilized in the model**

| **Short name** | **Long name** | **Unit** | **Explanation** |
| --- | --- | --- | --- |
| bio1 | Mean annual air temperature | ℃*10 | Mean annual daily mean air temperatures averaged over 1 year |
| bio2 | Mean diurnal air temperature range | ℃ | Mean diurnal range of temperatures averaged over 1 year |
| bio3 | Isothermality | - | Ratio of diurnal variation to annual variation in temperatures |
| bio4 | Temperature seasonality | - | Standard deviation of the monthly mean temperatures |
| bio5 | Mean daily maximum air temperature of the warmest month | ℃*10 | The highest temperature of any monthly daily mean maximum temperature |
| bio6 | Mean daily minimum air temperature of the coldest month | ℃*10 | The lowest temperature of any monthly daily mean maximum temperature |
| bio7 | Annual range of air temperature | ℃*10 | The difference between the Maximum Temperature of Warmest month and the Minimum Temperature of Coldest month |
| bio8 | Mean daily mean air temperatures of the wettest quarter | ℃*10 | The wettest quarter of the year is determined (to the nearest month) |
| bio9 | Mean daily mean air temperatures of the driest quarter | ℃*10 | The driest quarter of the year is determined (to the nearest month) |
| bio10 | Mean daily mean air temperatures of the warmest quarter | ℃*10 | The warmest quarter of the year is determined (to the nearest month) |
| bio11 | Mean daily mean air temperatures of the coldest quarter | ℃*10 | The coldest quarter of the year is determined (to the nearest month) |
| bio12 | Annual precipitation amount | mm/year | Accumulated precipitation amount over 1 year |
| bio13 | Precipitation amount of the wettest month | mm/month | The precipitation of the wettest month |
| bio14 | Precipitation amount of the driest month | mm/month | The precipitation of the driest month |
| bio15 | Precipitation seasonality | - | The Coefficient of Variation is the standard deviation of the monthly precipitation estimates expressed as a percentage of the mean of those estimates (i.e. the annual mean) |
| bio16 | Mean monthly precipitation amount of the wettest quarter | mm/quarter | The wettest quarter of the year is determined (to the nearest month) |
| bio17 | Mean monthly precipitation amount of the driest quarter | mm/quarter | The driest quarter of the year is determined (to the nearest month) |
| bio18 | Mean monthly precipitation amount of the warmest quarter | mm/quarter | The warmest quarter of the year is determined (to the nearest month) |
| bio19 | Mean monthly precipitation amount of the warmest quarter | mm/quarter | The coldest quarter of the year is determined (to the nearest month) |
| prec1-prec12 | Monthly precipitation amount | mm/month | “Amount” means mass per unit area. “Precipitation” in the earth's atmosphere means precipitation of water in all phases. |
| temp1-temp12 | Monthly mean air temperature | ℃*10 | Mean monthly temperature at a height of 2 meters |
| tmax1-tmax12 | Monthly maximum air temperature | ℃*10 | Maximum monthly temperature at a height of 2 meters |
| tmin1-tmin12 | Monthly minimum air temperature | ℃*10 | Minimum monthly temperature at a height of 2 meters |
