# Supplementary material for: Environmental and Historical Determinants of African Horse Sickness: Insights from Predictive Modeling
Source: Transbound Emerg Dis. 2024 Aug 13;2024:5586647. doi: 10.1155/2024/5586647 (PMC12017013; doi:10.1155/2024/5586647)
Supplement: Supplementary 4 — File 4: the response curves of AHSV vector distribution model. [file 5586647.f4.pdf]

The response curves of AHSV vector distribution model

C.i._1_Af

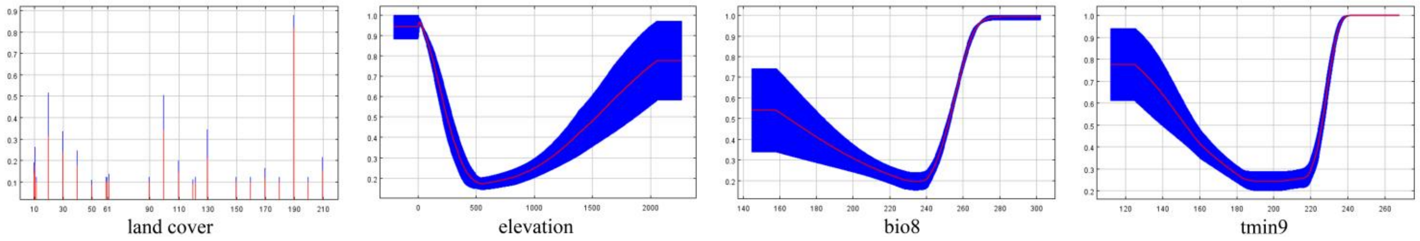

C.i._1_As

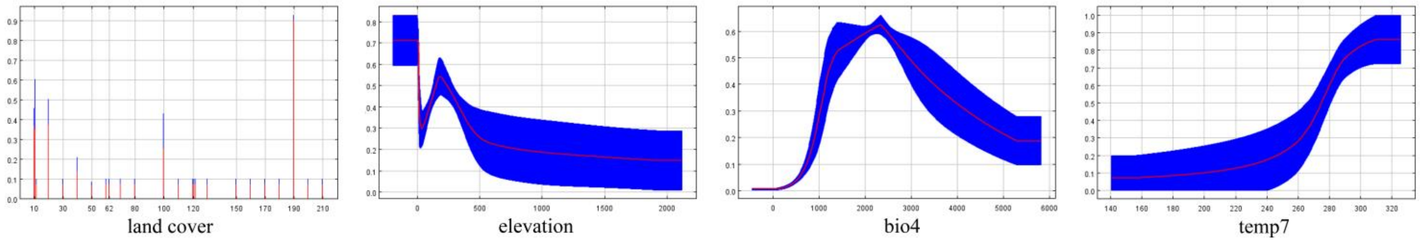

C.i._2_Af

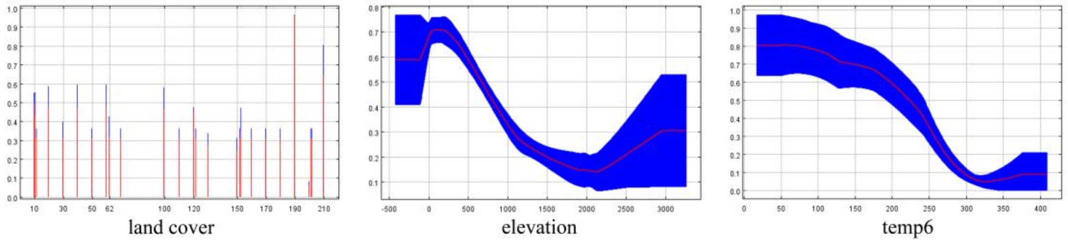

C.i._2_As

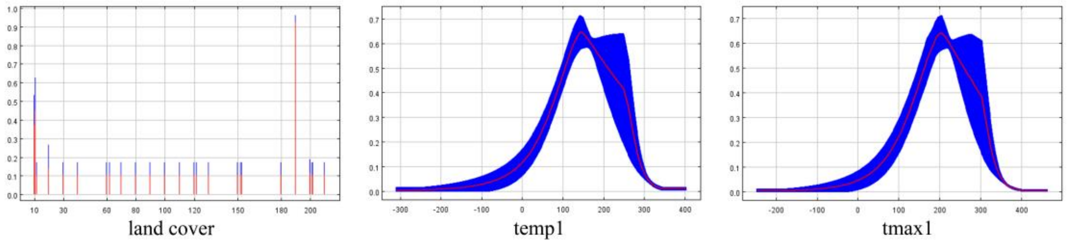

C.i._2_Eu

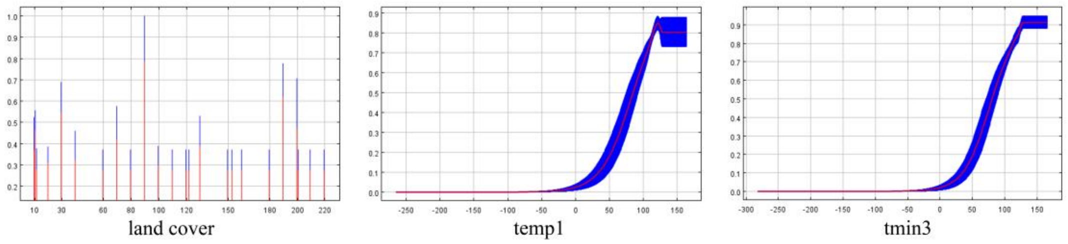

C.i._3_Af

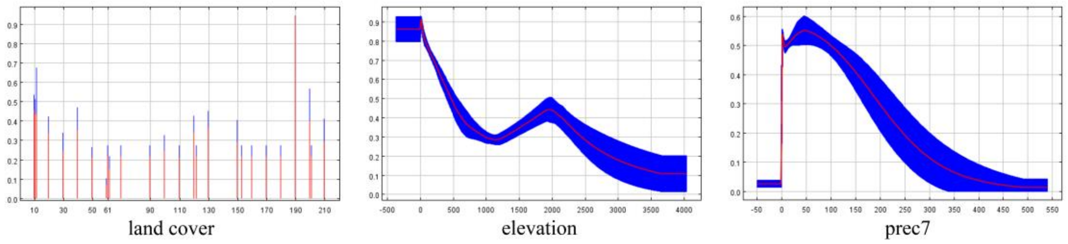

C.i._3_As

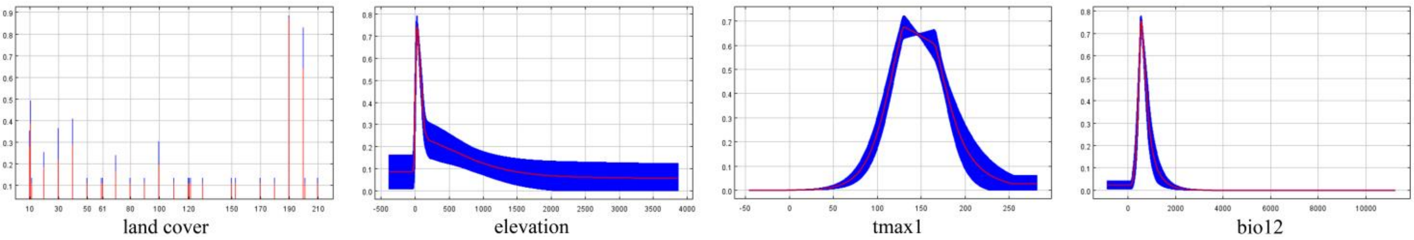

C.i._3_Eu

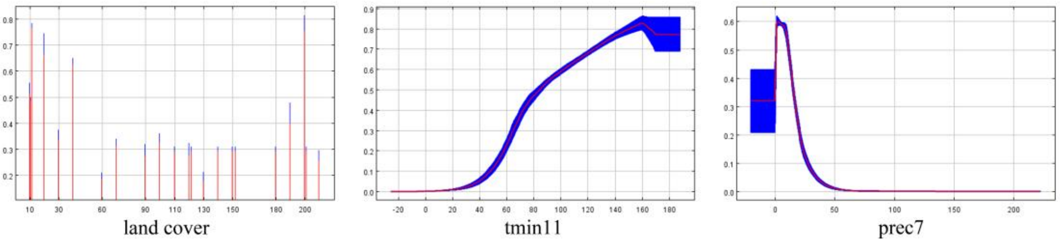

C.bo._1_Af

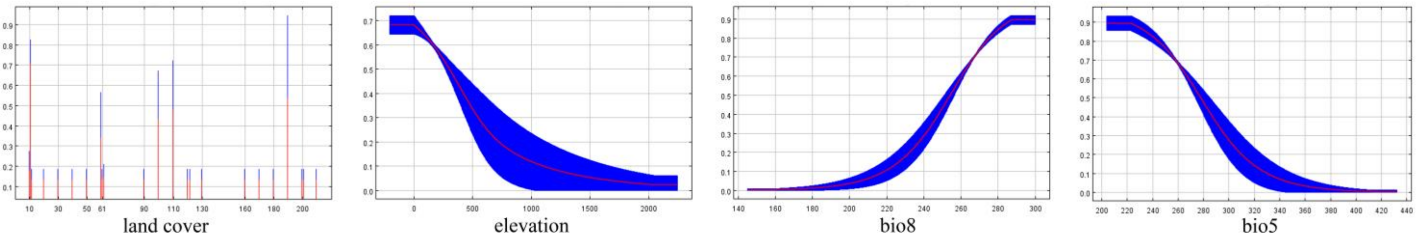

C.bo._2_Af

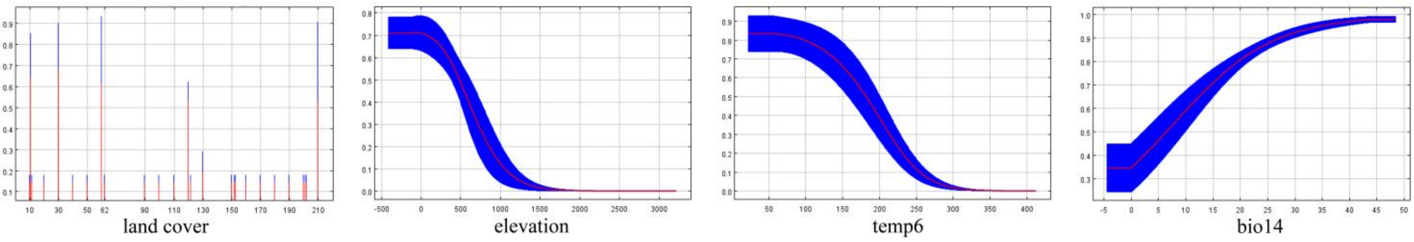

C.bo._3_Af

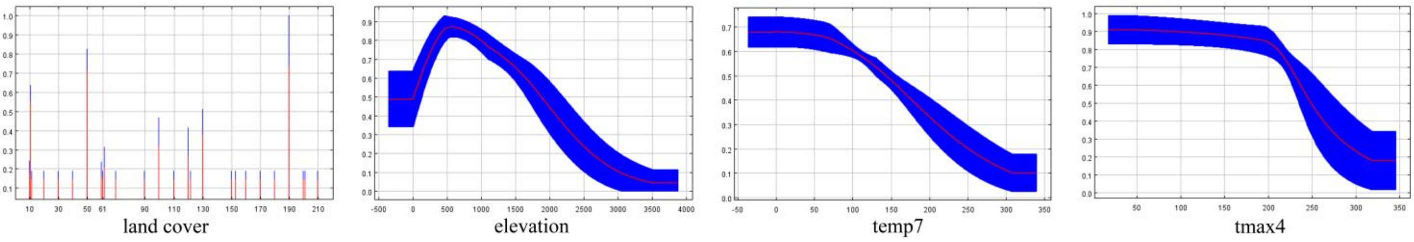

C.v._2_nAm

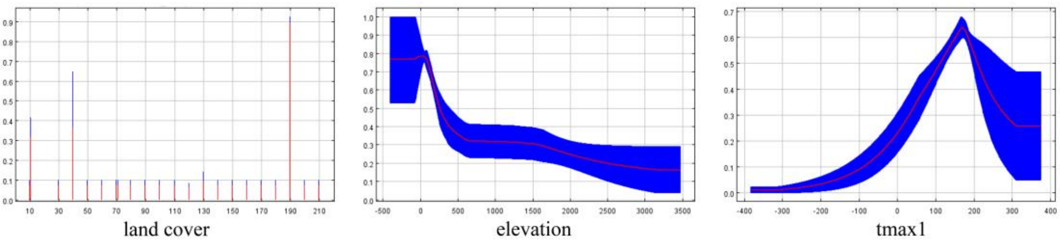

C.v._3_nAm

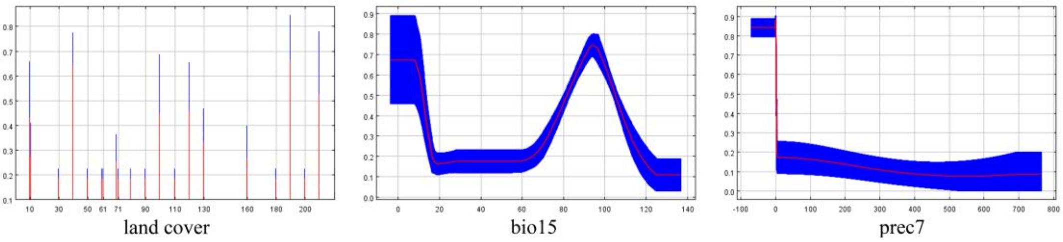

C.v._4_nAm

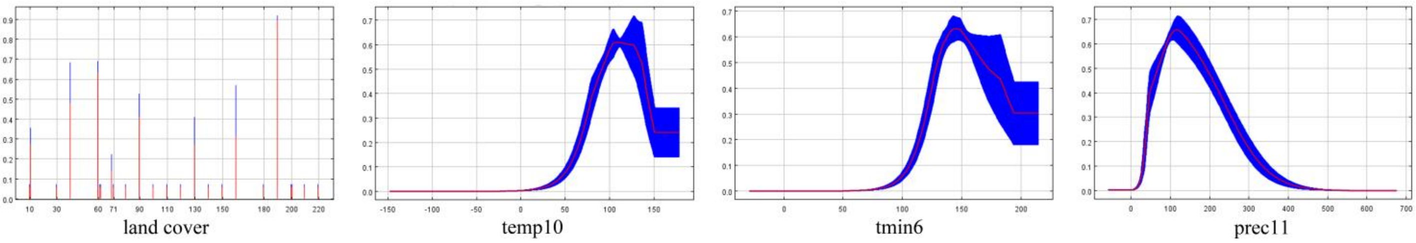

C.br._1_As

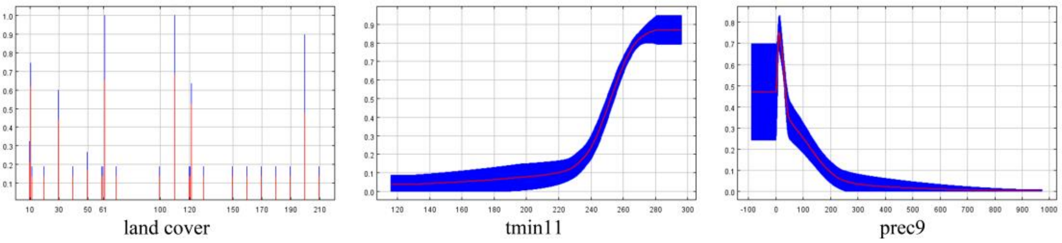

C.br._1_Au

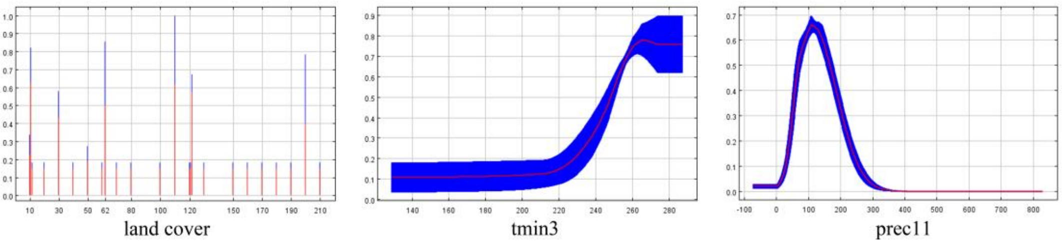

A.a._1_Af

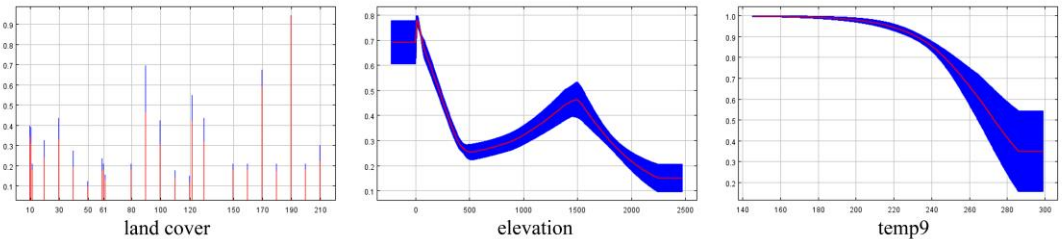

A.a._1_Am

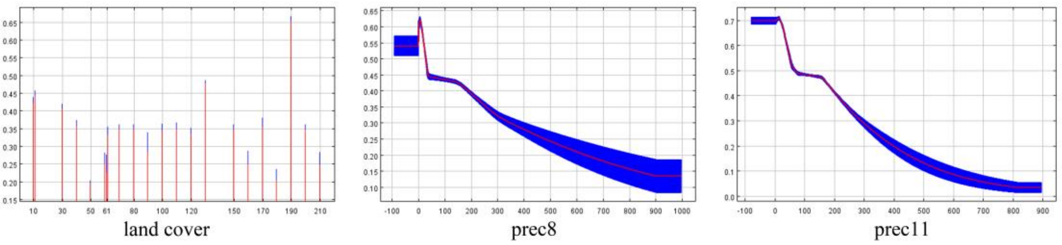

A.a._1_As

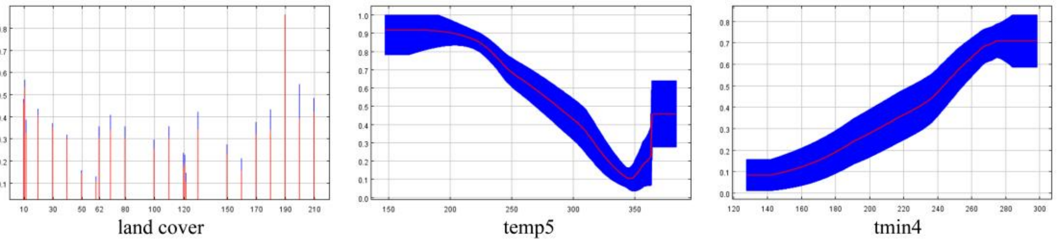

A.a._1_Au

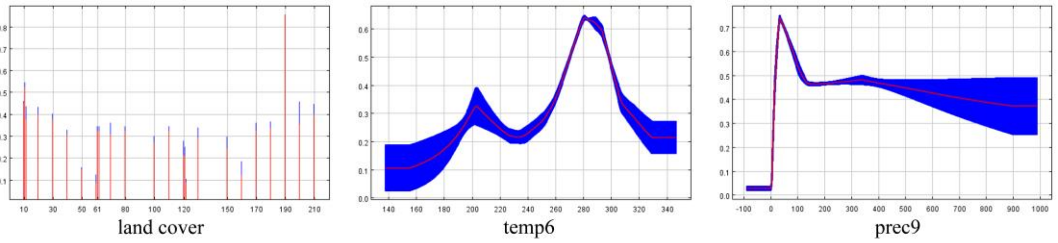

A.a._2_Af

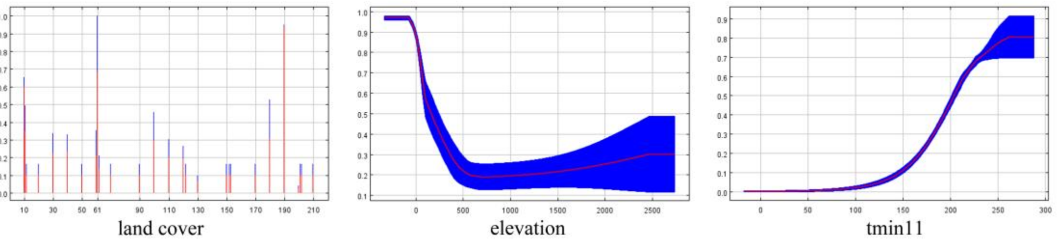

A.a._2_Am

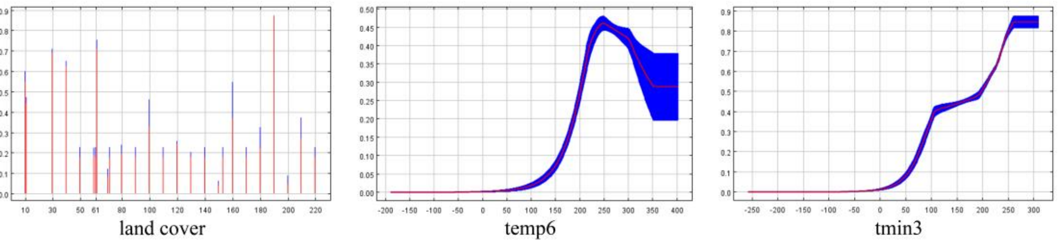

A.a._2_As

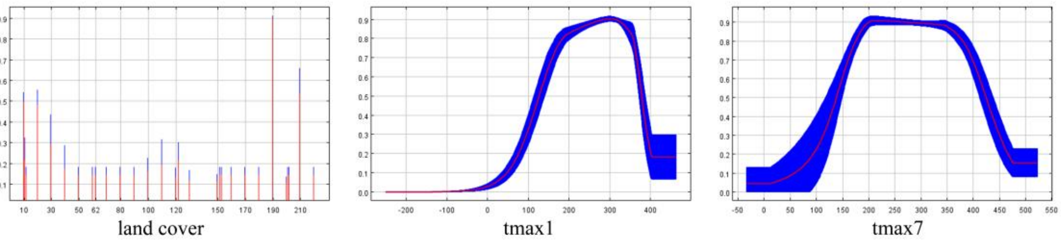

A.a._3_Af

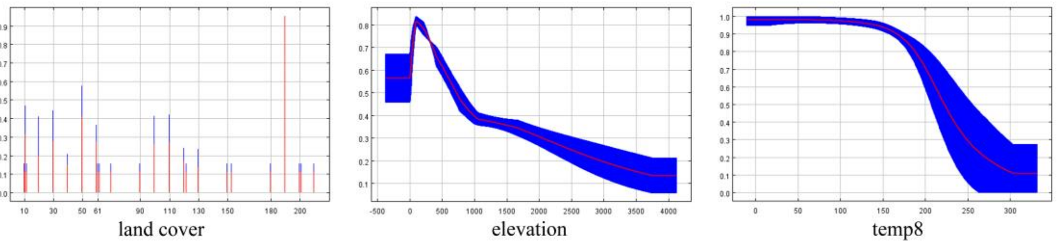

A.a._3_Am

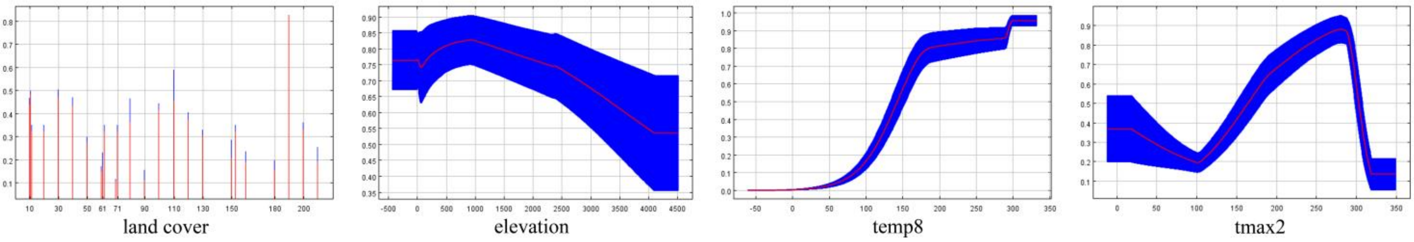

A.a._3_As

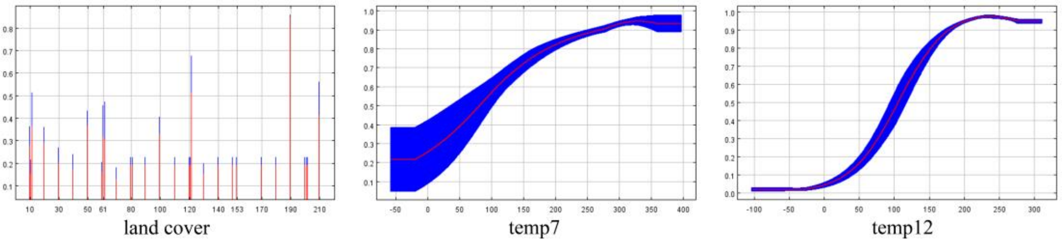

A.a._3_Au

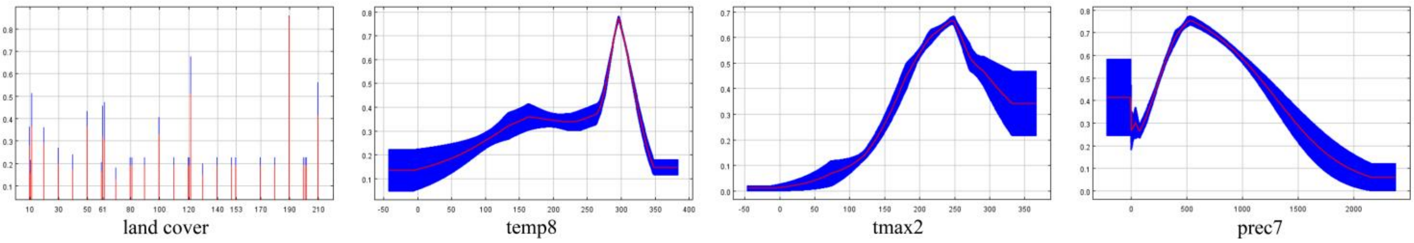

A.a._4_nAm

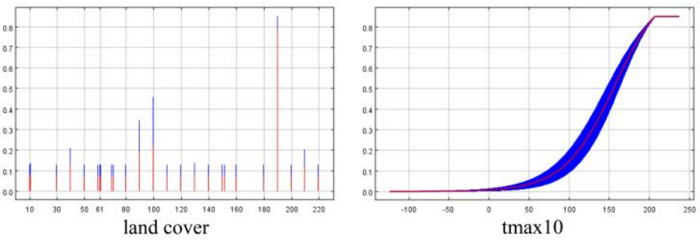

A.s._1_As

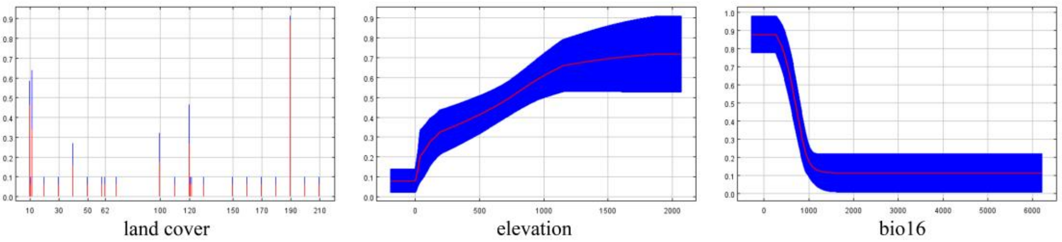

A.s._1_Au

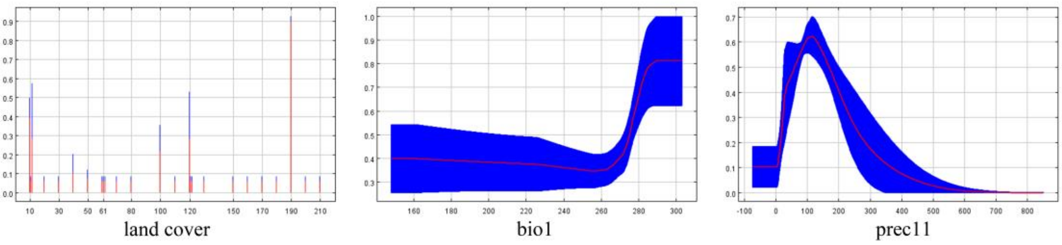

A.s._2_As

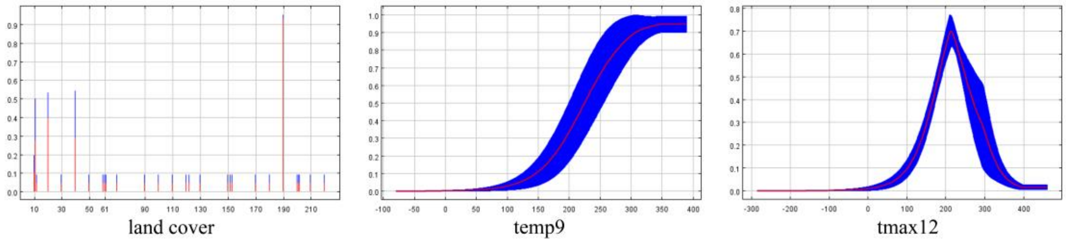

A.s._2_Au

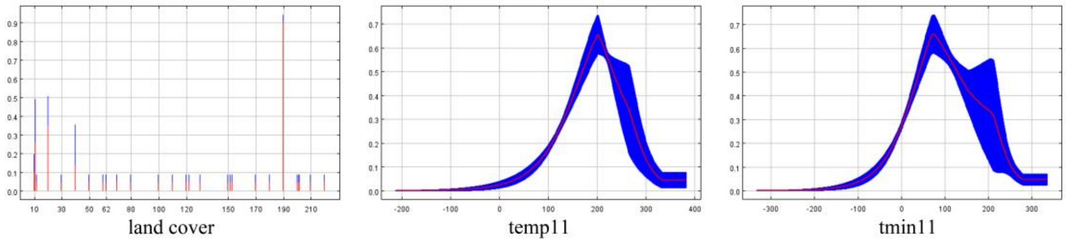

C.p._1_Af

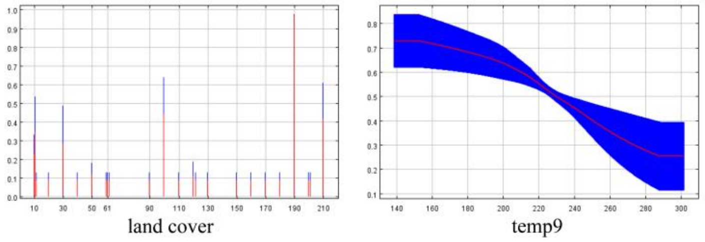

C.p._1_As

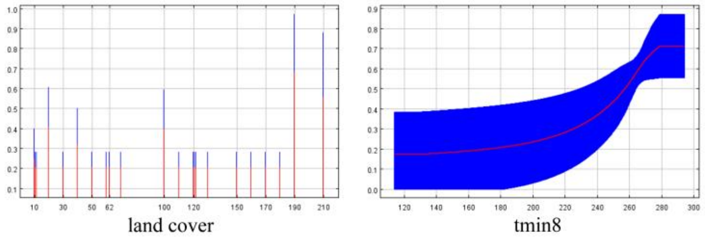

C.p._2_Af

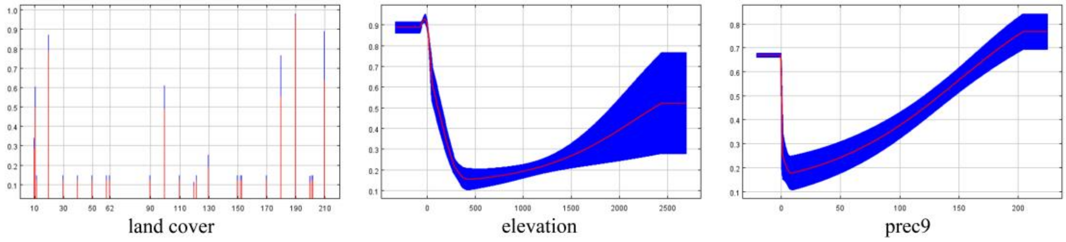

C.p._2_Am

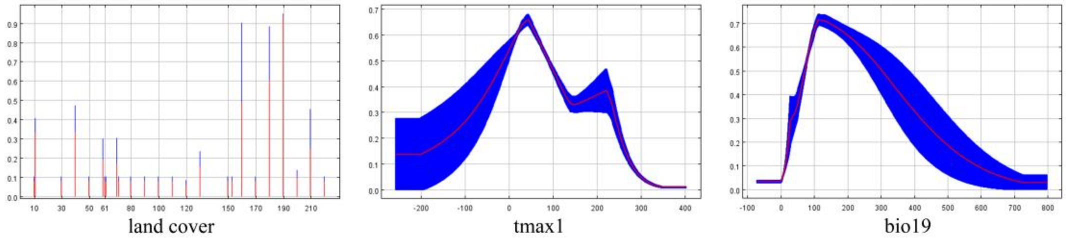

C.p._2_As

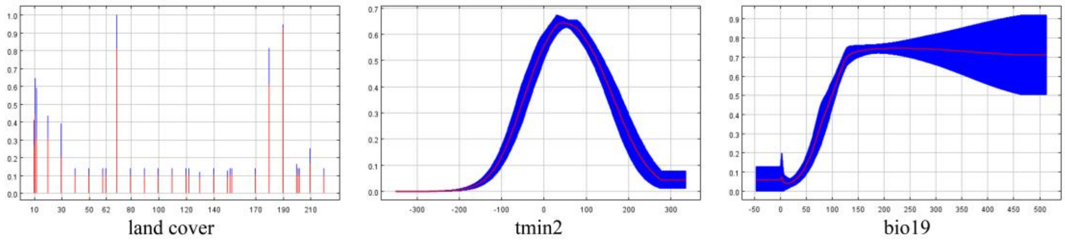

C.p._3_Af

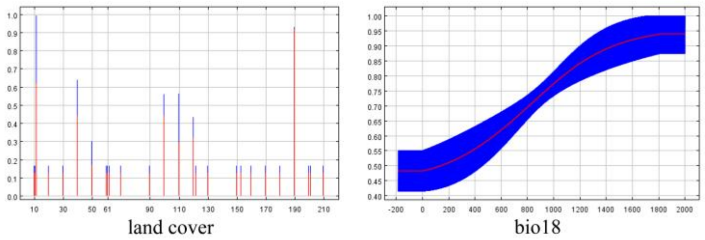

C.p._3_Am

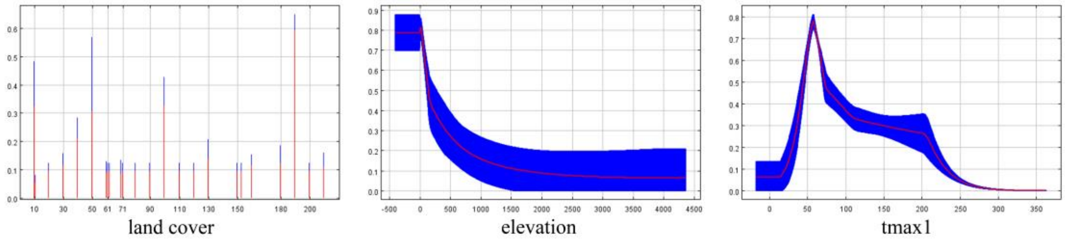

C.p._3_Eu

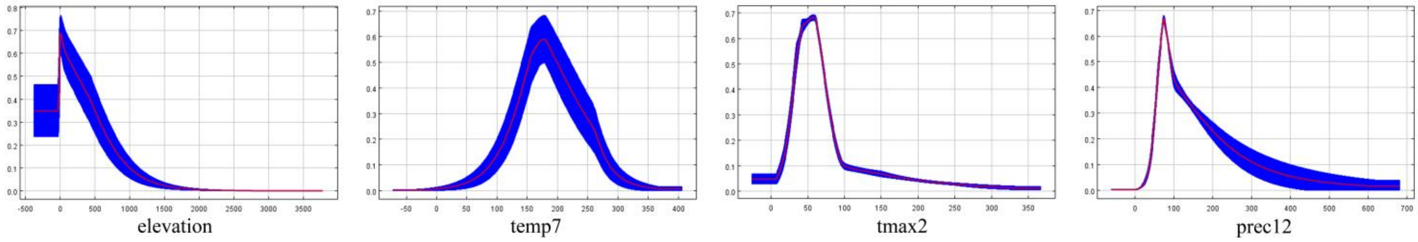

C.p._4_As

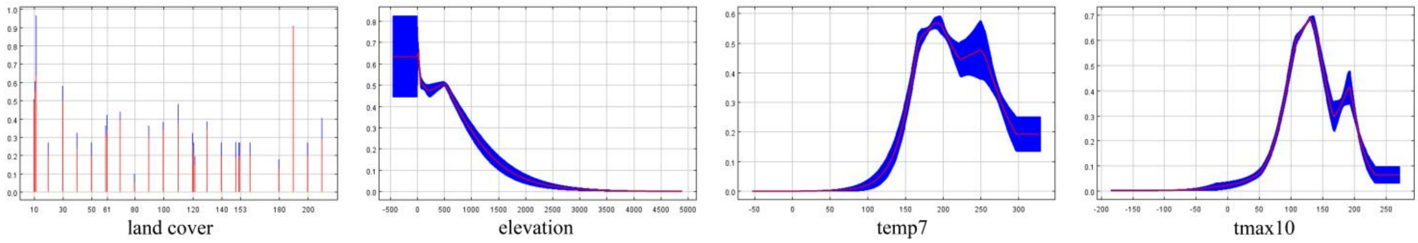

C.p._4_Eu

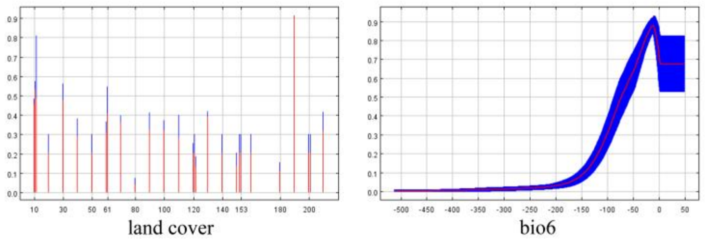

C.p._4_nAm

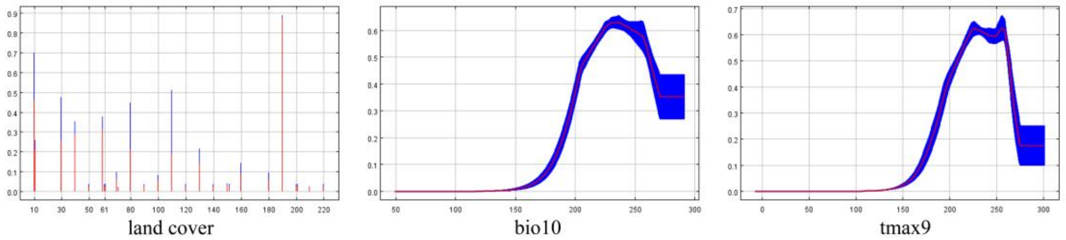

H.d._2_As

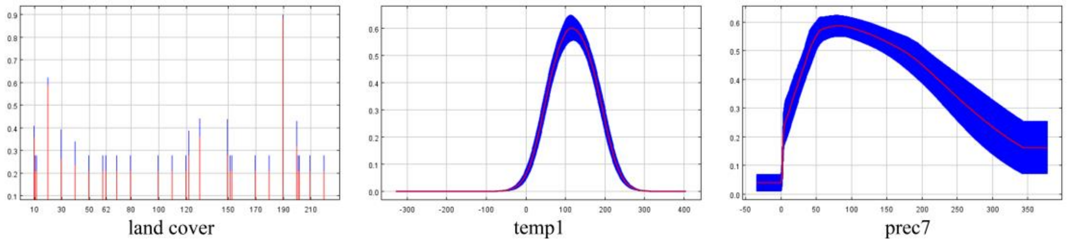

R.s._1_Af

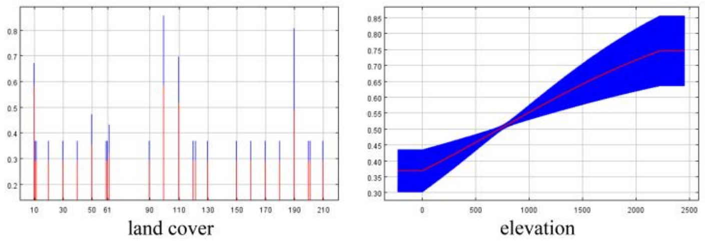

R.s._1_Am

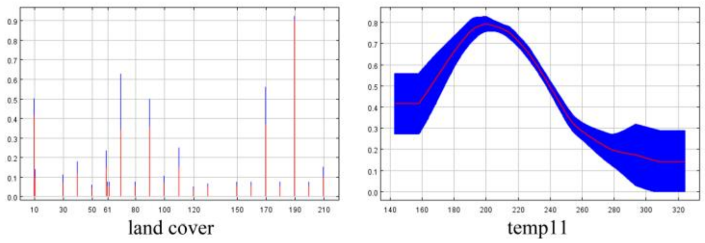

R.s._1_As

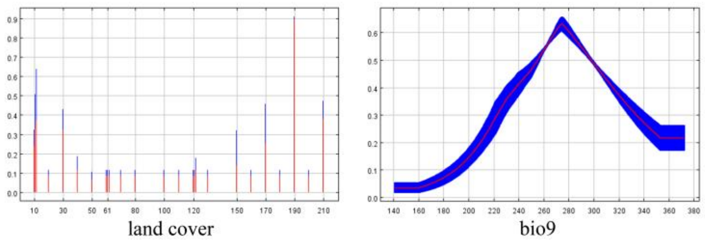

R.s._2_Af

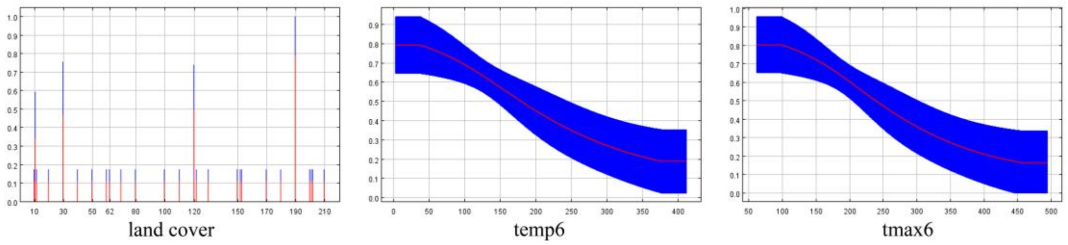

R.s._2_Am

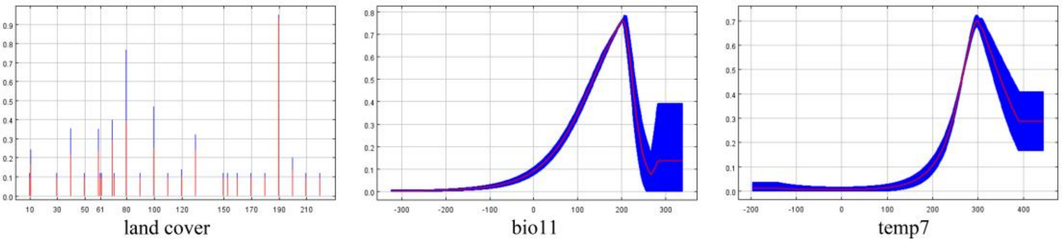

R.s._2_As

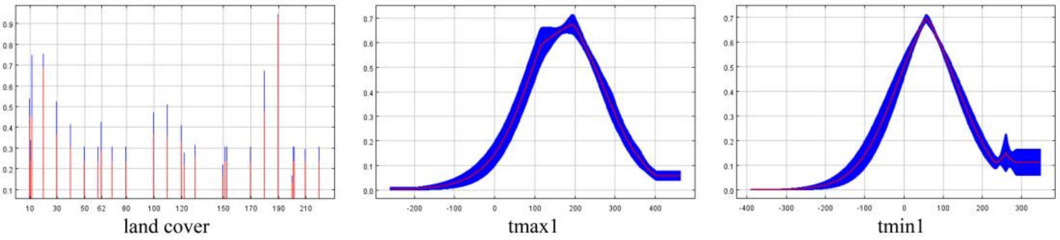

R.s._2_Au

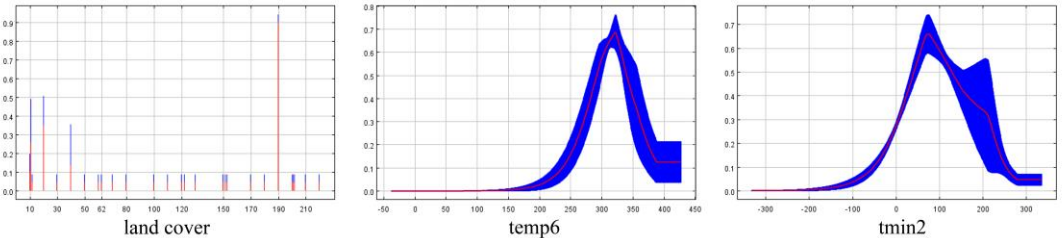

R.s._3_Af

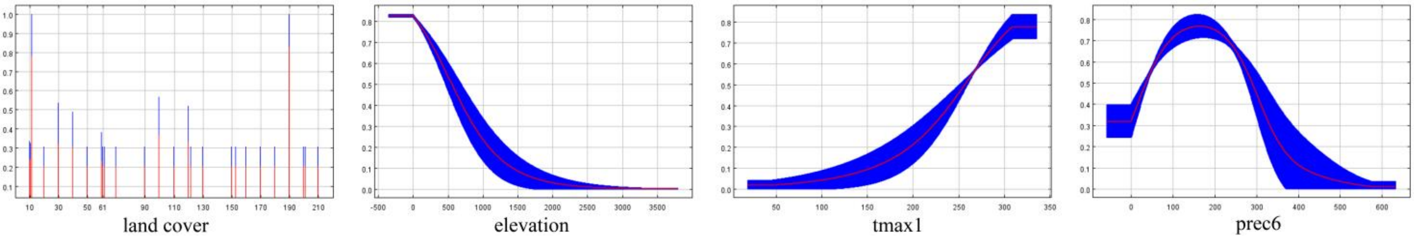

R.s._3_Am

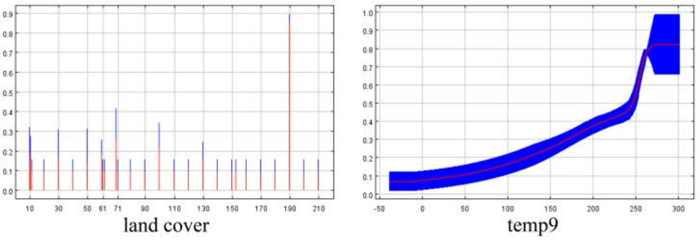

R.s._3_As

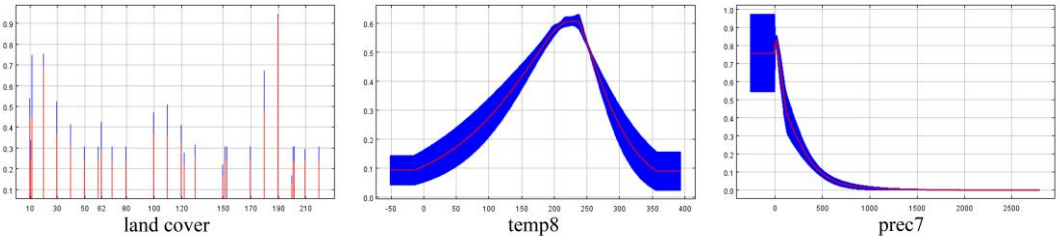

R.s._3_Au

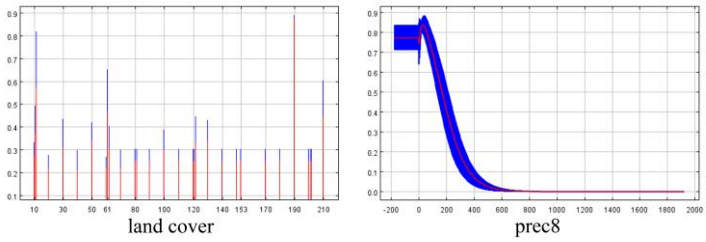

R.s._3_Eu

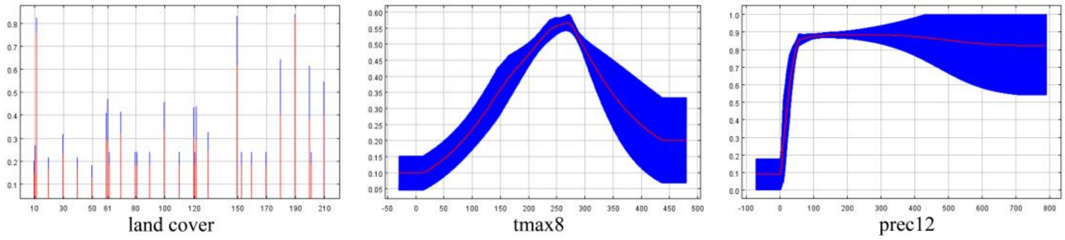

R.s._4_Eu

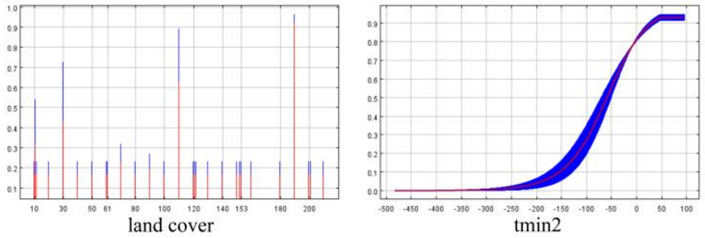

R.s._4_nAm

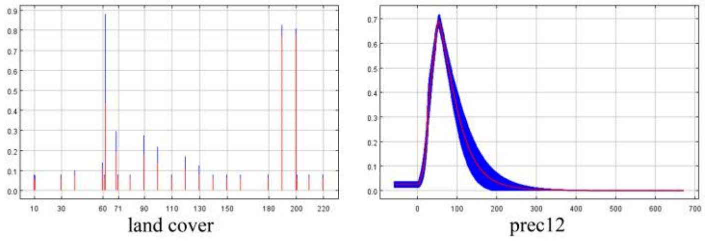

S.c._1_Af

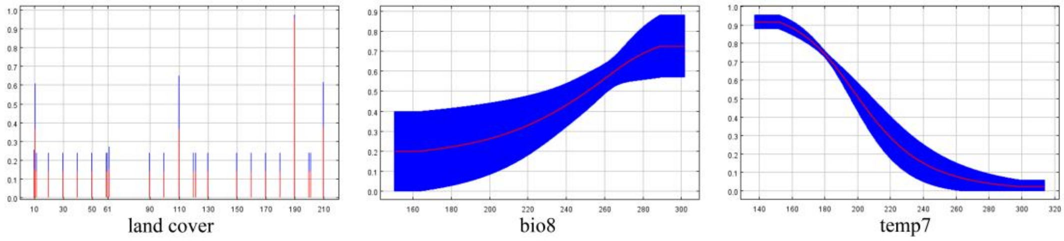

S.c._1_Am

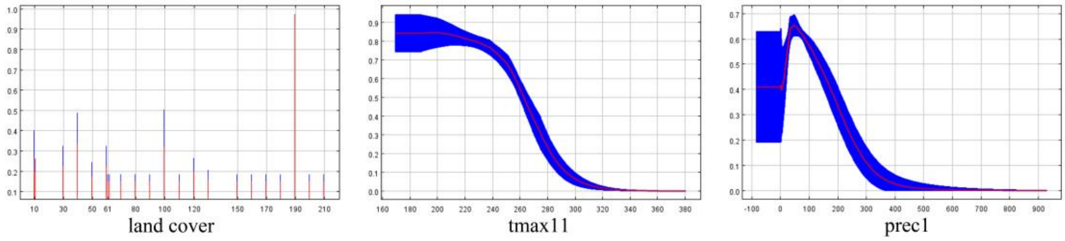

S.c._1_As

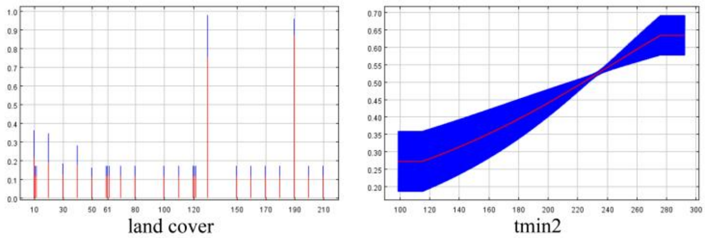

S.c._2_Af

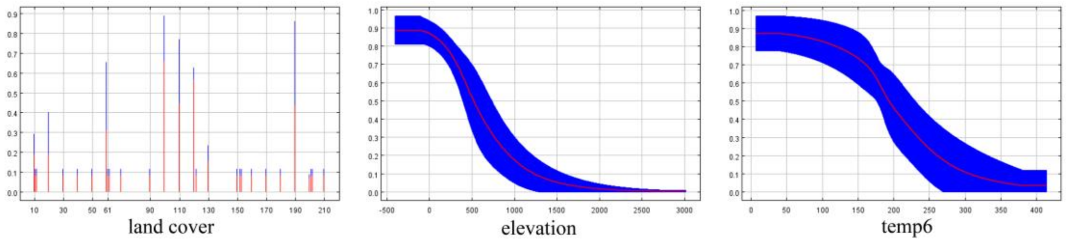

S.c._2_Am

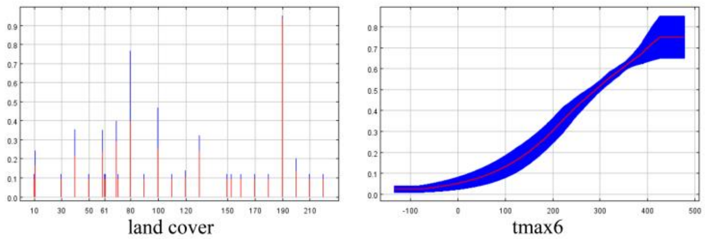

S.c._2_As

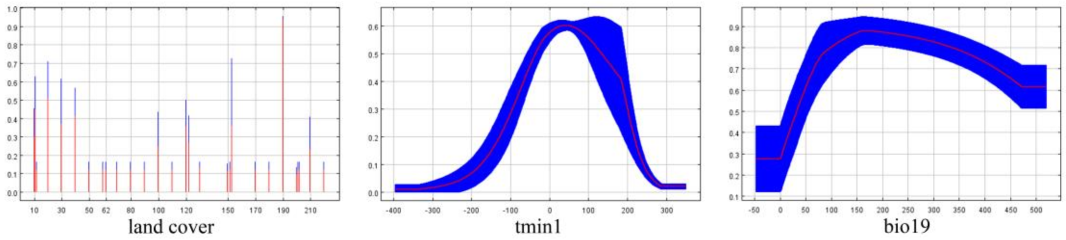

S.c._2_Au

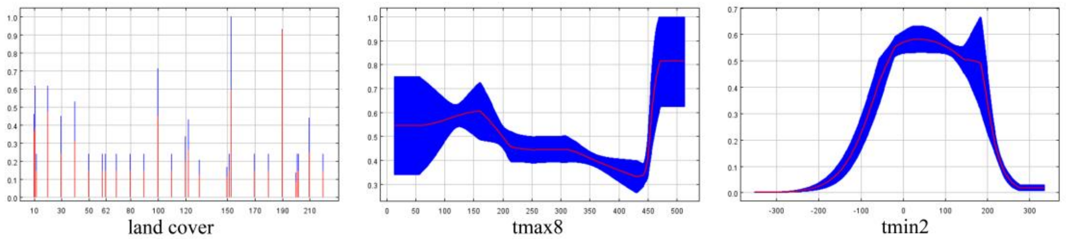

S.c._3_Af

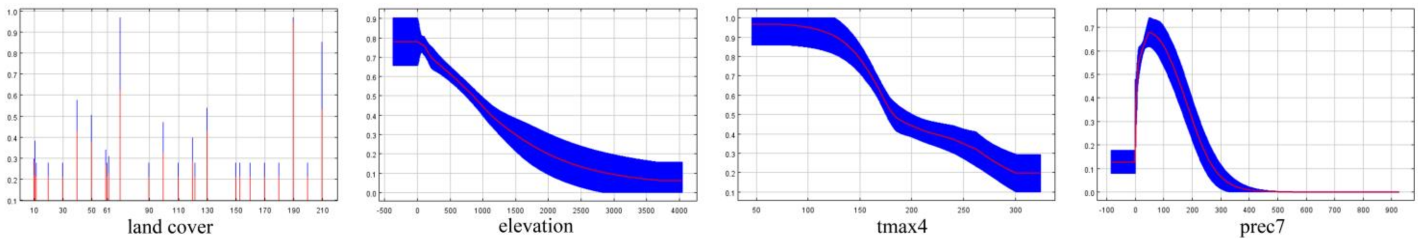

S.c._3_Am

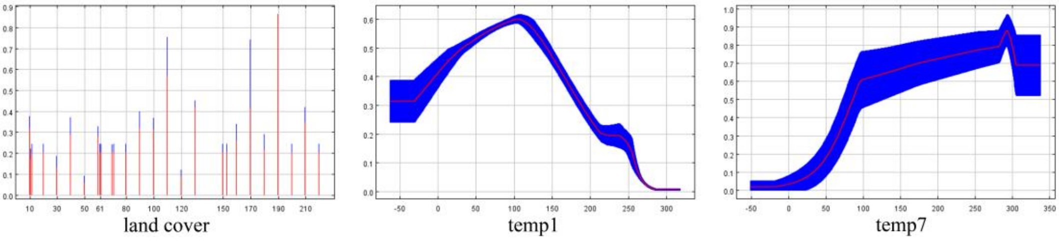

S.c._3_Eu

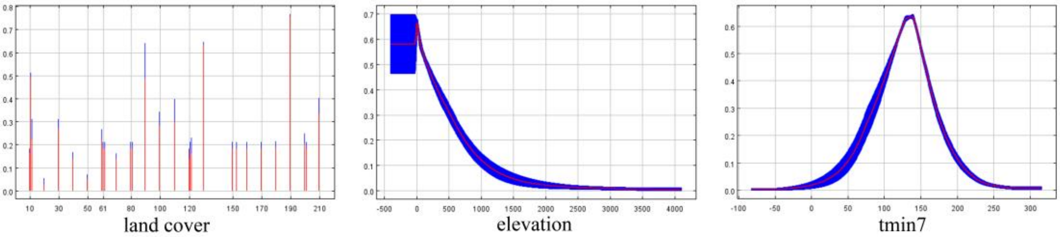

S.c._4_Eu

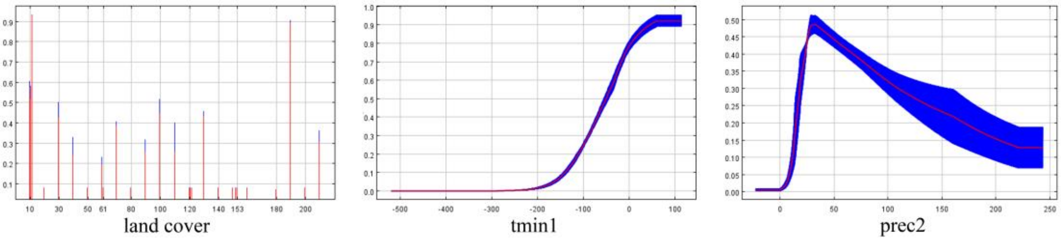

S.c._4_nAm

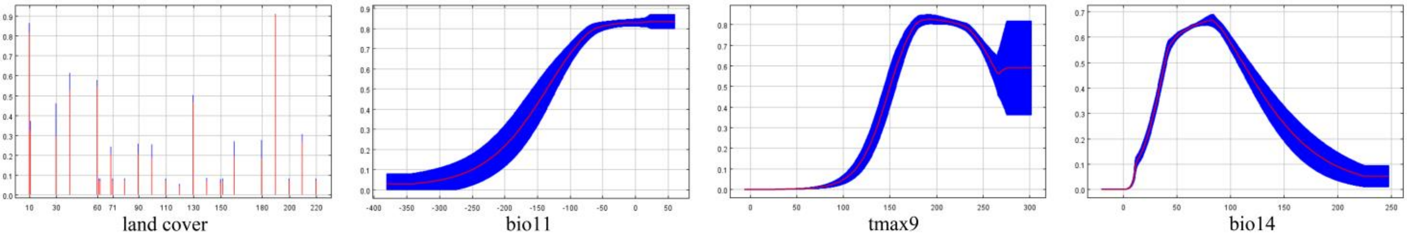

T._1_Af

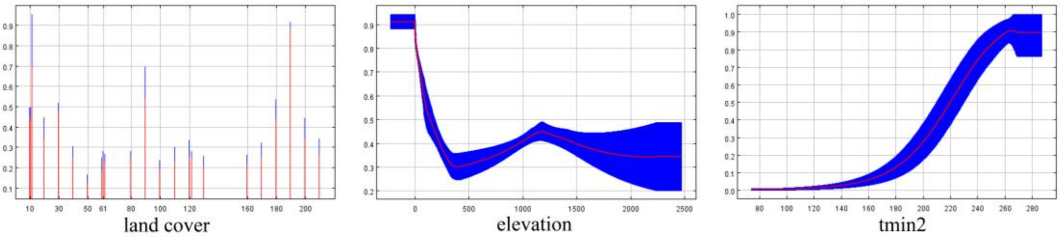

T._1_Am

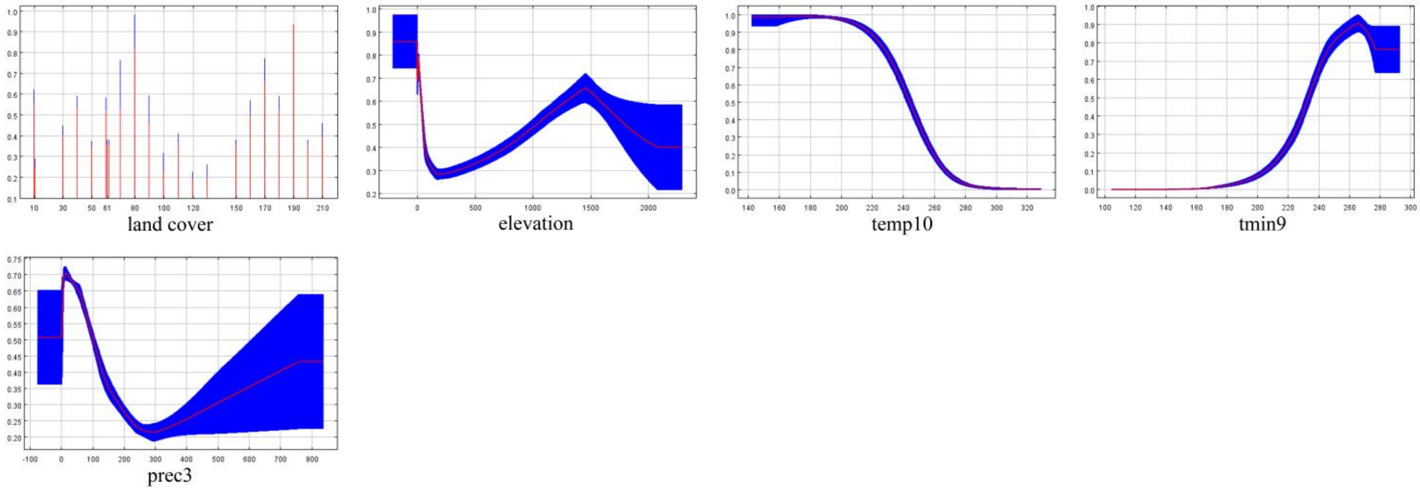

T._1_As

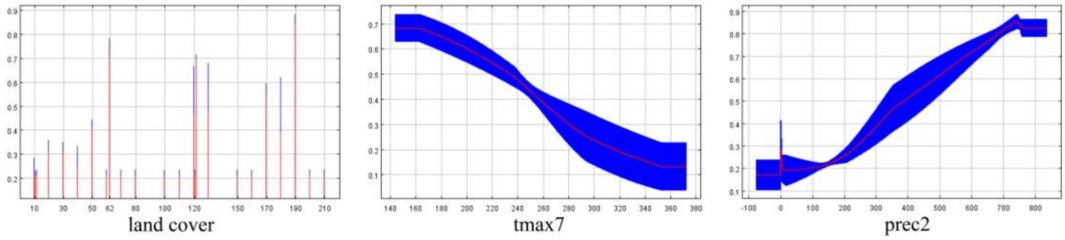

T._2_Af

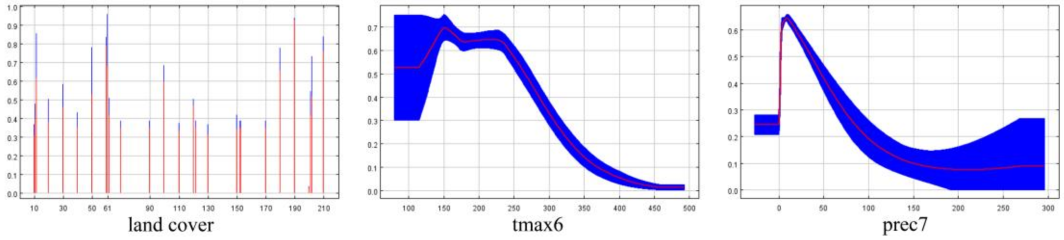

T._2_Am

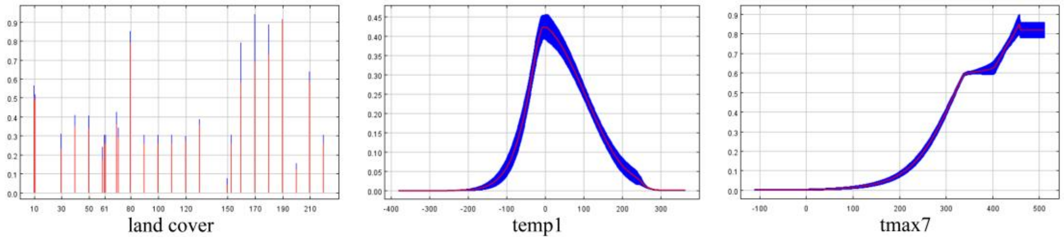

T._2_As

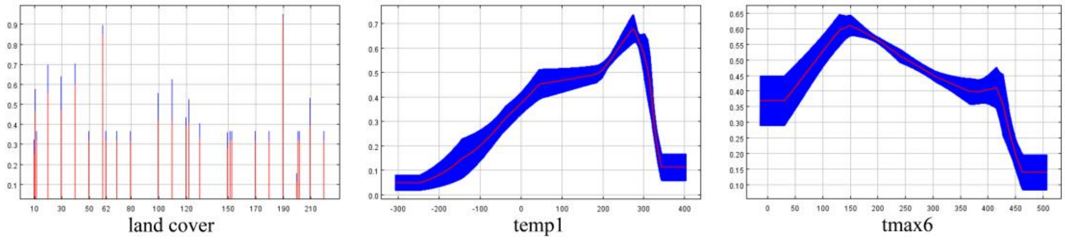

T._2_Au

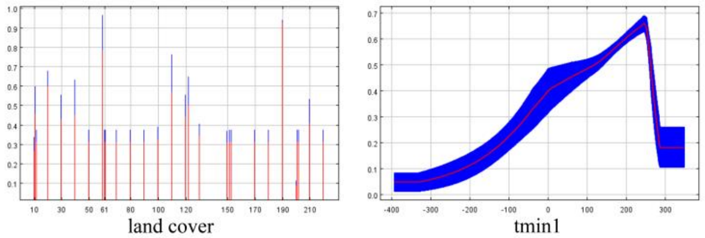

T._3_Af

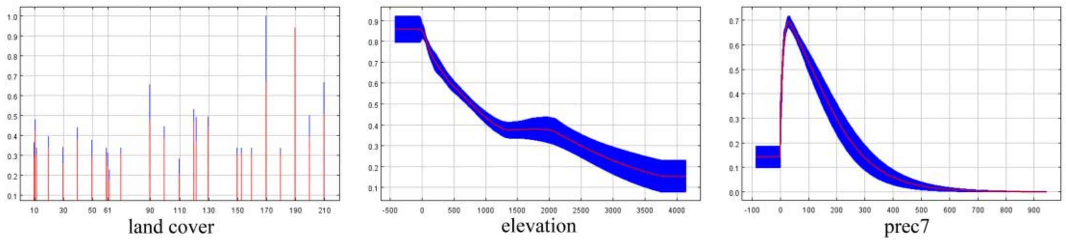

T._3_Am

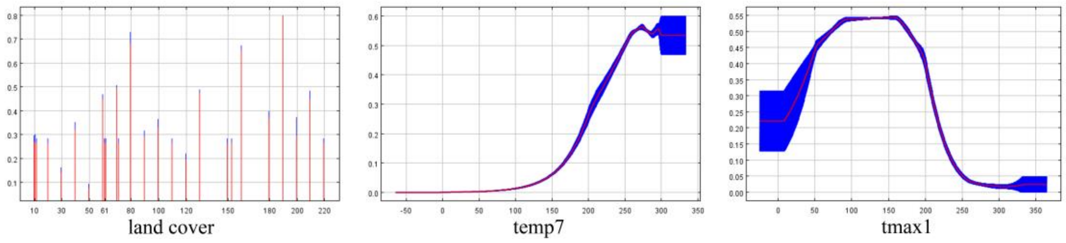

T._3_Eu

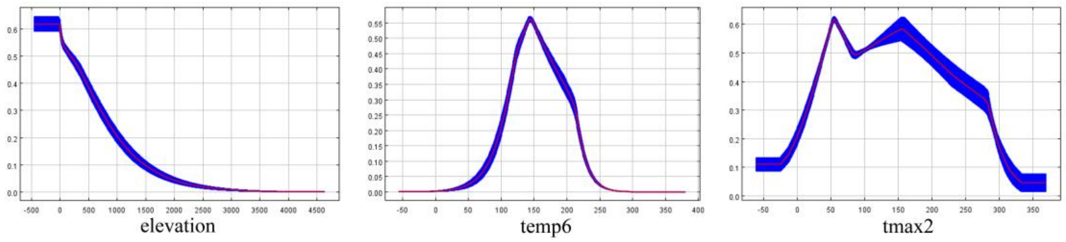

T._4_Eu

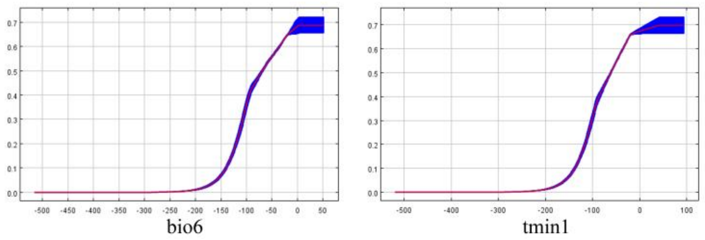

T._4_nAm

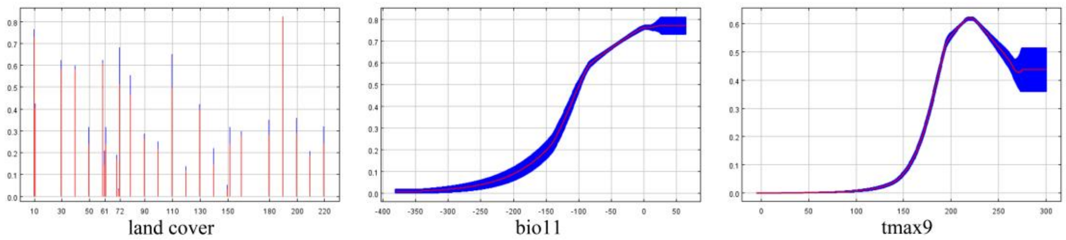

Abbreviation Key

C.i.: *Culicoides imicola*, C.bo.: *Culicoides bolitinos*, C.v.: *Culicoides variipennis*, C.br.: *Culicoides brevitarsis*
A.a.: *Aedes aegypti*, A.s.: *Anopheles stephensi*, C.p.: *Culex pipiens*
H.d.: *Hyalomma dromedarii*, R.s.: *Rhipicephalus sanguineus*
S.c.: *Stomoxys calcitrans*, T.: *Tabanus* spp.
1: Tropical Climate region, 2: Arid Climate region, 3: Temperate Climate region, 4: Continental Climate region
Af: Africa, Am: America, nAm: North America, As: Asia, Au: Australasia, Eu: Europe

Land cover legend

10: Cropland/ rainfed
11: Herbaceous cover
12: Tree or shrub cover
20: Cropland/ irrigated or post-flooding
30: Mosaic cropland (>50%) / natural vegetation (tree, shrub, herbaceous cover) (<50%)
40: Mosaic natural vegetation (tree, shrub, herbaceous cover) (>50%) / cropland (<50%)
50: Tree cover, broadleaved, evergreen, closed to open (>15%)
60: Tree cover, broadleaved, deciduous, closed to open (>15%)
61: Tree cover, broadleaved, deciduous, closed (>40%)
62: Tree cover, broadleaved, deciduous, open (15-40%)
70: Tree cover, needleleaved, evergreen, closed to open (>15%)
71: Tree cover, needleleaved, evergreen, closed (>40%)
72: Tree cover, needleleaved, evergreen, open (15-40%)
80: Tree cover, needleleaved, deciduous, closed to open (>15%)
81: Tree cover, needleleaved, deciduous, closed (>40%)
82: Tree cover, needleleaved, deciduous, open (15-40%)
90: Tree cover, mixed leaf type (broadleaved and needleleaved)
100: Mosaic tree and shrub (>50%) / herbaceous cover (<50%)
110: Mosaic herbaceous cover (>50%) / tree and shrub (<50%)
120: Shrubland
121: Shrubland evergreen
122: Shrubland deciduous
130: Grassland
140: Lichens and mosses
150: Sparse vegetation (tree, shrub, herbaceous cover) (<15%)
151: Sparse tree (<15%)
152: Sparse shrub (<15%)
153: Sparse herbaceous cover (<15%)
160: Tree cover, flooded, fresh or brackish water
170: Tree cover, flooded, saline water
180: Shrub or herbaceous cover, flooded, fresh/saline/brackish water
190: Urban areas
200: Bare areas
201: Consolidated bare areas
202: Unconsolidated bare areas
210: Water bodies
220: Permanent snow and ice
