# Supplementary material for: Environmental and Historical Determinants of African Horse Sickness: Insights from Predictive Modeling
Source: Transbound Emerg Dis. 2024 Aug 13;2024:5586647. doi: 10.1155/2024/5586647 (PMC12017013; doi:10.1155/2024/5586647)
Supplement: Supplementary 2 — File 2: distribution data of African horse sickness virus vector species. [file 5586647.f2.pdf]

Distribution data of AHSV vector species

№	Order	Family	Genus	Species	Taxon Rank	Latitude	Longitude	GBIF_ID
1	Diptera	Ceratopogonidae	Culicoides	Culicoides bolitinos	SPECIES	-29.22000	29.93000	3032874707
2	Diptera	Ceratopogonidae	Culicoides	Culicoides bolitinos	SPECIES	-29.22000	29.93000	3987230200
3	Diptera	Ceratopogonidae	Culicoides	Culicoides bolitinos	SPECIES	-28.92630	28.63860	2248515662
4	Diptera	Ceratopogonidae	Culicoides	Culicoides bolitinos	SPECIES	-26.02600	27.54500	1415483386
5	Diptera	Ceratopogonidae	Culicoides	Culicoides bolitinos	SPECIES	-25.62870	32.36270	2248514822
6	Diptera	Ceratopogonidae	Culicoides	Culicoides bolitinos	SPECIES	-25.47200	31.50800	3442544559
7	Diptera	Ceratopogonidae	Culicoides	Culicoides bolitinos	SPECIES	-25.35400	31.89500	3441581718
8	Diptera	Ceratopogonidae	Culicoides	Culicoides bolitinos	SPECIES	-25.32600	31.39200	3442619181
9	Diptera	Ceratopogonidae	Culicoides	Culicoides bolitinos	SPECIES	-25.12300	31.91600	3443514611
10	Diptera	Ceratopogonidae	Culicoides	Culicoides bolitinos	SPECIES	-24.78300	31.85900	3442551747
11	Diptera	Ceratopogonidae	Culicoides	Culicoides bolitinos	SPECIES	-24.39700	31.77700	3442532001
12	Diptera	Ceratopogonidae	Culicoides	Culicoides bolitinos	SPECIES	-24.10300	31.66900	3442696879
13	Diptera	Ceratopogonidae	Culicoides	Culicoides bolitinos	SPECIES	-23.85200	31.57400	3441488474
14	Diptera	Ceratopogonidae	Culicoides	Culicoides bolitinos	SPECIES	-22.87200	31.23100	3442852255
15	Diptera	Ceratopogonidae	Culicoides	Culicoides bolitinos	SPECIES	-21.25000	55.53000	3032877752
16	Diptera	Ceratopogonidae	Culicoides	Culicoides bolitinos	SPECIES	-21.25000	55.53000	3818398508
17	Diptera	Ceratopogonidae	Culicoides	Culicoides bolitinos	SPECIES	-21.23000	55.54000	2249958274
18	Diptera	Ceratopogonidae	Culicoides	Culicoides bolitinos	SPECIES	-21.23000	55.54000	3346803904
19	Diptera	Ceratopogonidae	Culicoides	Culicoides bolitinos	SPECIES	-21.22520	55.50140	2248515721
20	Diptera	Ceratopogonidae	Culicoides	Culicoides bolitinos	SPECIES	-21.13317	55.53241	4050523198
21	Diptera	Ceratopogonidae	Culicoides	Culicoides bolitinos	SPECIES	-18.13050	44.90970	2248514915
22	Diptera	Ceratopogonidae	Culicoides	Culicoides bolitinos	SPECIES	-18.02000	44.48000	3987251722
23	Diptera	Ceratopogonidae	Culicoides	Culicoides bolitinos	SPECIES	-0.96667	38.14780	2248516385
24	Diptera	Ceratopogonidae	Culicoides	Culicoides bolitinos	SPECIES	0.29300	36.89900	2979614002
25	Diptera	Ceratopogonidae	Culicoides	Culicoides bolitinos	SPECIES	-21.38100	55.60800	Ref.1
26	Diptera	Ceratopogonidae	Culicoides	Culicoides bolitinos	SPECIES	-21.36900	55.63900	Ref.1
27	Diptera	Ceratopogonidae	Culicoides	Culicoides bolitinos	SPECIES	-21.36700	55.73100	Ref.1
28	Diptera	Ceratopogonidae	Culicoides	Culicoides bolitinos	SPECIES	-21.35800	55.61000	Ref.1
29	Diptera	Ceratopogonidae	Culicoides	Culicoides bolitinos	SPECIES	-21.35800	55.53800	Ref.1
30	Diptera	Ceratopogonidae	Culicoides	Culicoides bolitinos	SPECIES	-21.34600	55.66800	Ref.1
31	Diptera	Ceratopogonidae	Culicoides	Culicoides bolitinos	SPECIES	-21.34000	55.59100	Ref.1
32	Diptera	Ceratopogonidae	Culicoides	Culicoides bolitinos	SPECIES	-21.33600	55.61500	Ref.1
33	Diptera	Ceratopogonidae	Culicoides	Culicoides bolitinos	SPECIES	-21.33200	55.65200	Ref.1
34	Diptera	Ceratopogonidae	Culicoides	Culicoides bolitinos	SPECIES	-21.32700	55.61400	Ref.1
35	Diptera	Ceratopogonidae	Culicoides	Culicoides bolitinos	SPECIES	-21.31800	55.56200	Ref.1
36	Diptera	Ceratopogonidae	Culicoides	Culicoides bolitinos	SPECIES	-21.30400	55.55800	Ref.1
37	Diptera	Ceratopogonidae	Culicoides	Culicoides bolitinos	SPECIES	-21.30100	55.53900	Ref.1
38	Diptera	Ceratopogonidae	Culicoides	Culicoides bolitinos	SPECIES	-21.28300	55.38800	Ref.1
39	Diptera	Ceratopogonidae	Culicoides	Culicoides bolitinos	SPECIES	-21.24900	55.51200	Ref.1
40	Diptera	Ceratopogonidae	Culicoides	Culicoides bolitinos	SPECIES	-21.24400	55.53200	Ref.1

№	Order	Family	Genus	Species	Taxon Rank	Latitude	Longitude	GBIF_ID
41	Diptera	Ceratopogonidae	Culicoides	Culicoides bolitinos	SPECIES	-21.24200	55.47600	Ref.1
42	Diptera	Ceratopogonidae	Culicoides	Culicoides bolitinos	SPECIES	-21.23700	55.31800	Ref.1
43	Diptera	Ceratopogonidae	Culicoides	Culicoides bolitinos	SPECIES	-21.23600	55.55900	Ref.1
44	Diptera	Ceratopogonidae	Culicoides	Culicoides bolitinos	SPECIES	-21.23400	55.35000	Ref.1
45	Diptera	Ceratopogonidae	Culicoides	Culicoides bolitinos	SPECIES	-21.21600	55.54500	Ref.1
46	Diptera	Ceratopogonidae	Culicoides	Culicoides bolitinos	SPECIES	-21.21400	55.53000	Ref.1
47	Diptera	Ceratopogonidae	Culicoides	Culicoides bolitinos	SPECIES	-21.20900	55.52900	Ref.1
48	Diptera	Ceratopogonidae	Culicoides	Culicoides bolitinos	SPECIES	-21.15400	55.43700	Ref.1
49	Diptera	Ceratopogonidae	Culicoides	Culicoides bolitinos	SPECIES	-21.14000	55.48700	Ref.1
50	Diptera	Ceratopogonidae	Culicoides	Culicoides bolitinos	SPECIES	-21.11400	55.69100	Ref.1
51	Diptera	Ceratopogonidae	Culicoides	Culicoides bolitinos	SPECIES	-21.11200	55.71600	Ref.1
52	Diptera	Ceratopogonidae	Culicoides	Culicoides bolitinos	SPECIES	-21.10500	55.32500	Ref.1
53	Diptera	Ceratopogonidae	Culicoides	Culicoides bolitinos	SPECIES	-21.09900	55.66100	Ref.1
54	Diptera	Ceratopogonidae	Culicoides	Culicoides bolitinos	SPECIES	-21.09700	55.26200	Ref.1
55	Diptera	Ceratopogonidae	Culicoides	Culicoides bolitinos	SPECIES	-21.09700	55.75500	Ref.1
56	Diptera	Ceratopogonidae	Culicoides	Culicoides bolitinos	SPECIES	-21.09300	55.73600	Ref.1
57	Diptera	Ceratopogonidae	Culicoides	Culicoides bolitinos	SPECIES	-21.07200	55.72000	Ref.1
58	Diptera	Ceratopogonidae	Culicoides	Culicoides bolitinos	SPECIES	-21.06200	55.32800	Ref.1
59	Diptera	Ceratopogonidae	Culicoides	Culicoides bolitinos	SPECIES	-21.05700	55.26700	Ref.1
60	Diptera	Ceratopogonidae	Culicoides	Culicoides bolitinos	SPECIES	-21.04800	55.33400	Ref.1
61	Diptera	Ceratopogonidae	Culicoides	Culicoides bolitinos	SPECIES	-21.04500	55.70000	Ref.1
62	Diptera	Ceratopogonidae	Culicoides	Culicoides bolitinos	SPECIES	-21.02900	55.33100	Ref.1
63	Diptera	Ceratopogonidae	Culicoides	Culicoides bolitinos	SPECIES	-20.98800	55.63600	Ref.1
64	Diptera	Ceratopogonidae	Culicoides	Culicoides bolitinos	SPECIES	-20.90000	55.55800	Ref.1
65	Diptera	Ceratopogonidae	Culicoides	Culicoides bolitinos	SPECIES	-21.36000	55.61000	Ref.2
66	Diptera	Ceratopogonidae	Culicoides	Culicoides bolitinos	SPECIES	-21.31000	55.56000	Ref.2
67	Diptera	Ceratopogonidae	Culicoides	Culicoides bolitinos	SPECIES	-21.28000	55.37000	Ref.2
68	Diptera	Ceratopogonidae	Culicoides	Culicoides bolitinos	SPECIES	-21.27000	55.36000	Ref.2
69	Diptera	Ceratopogonidae	Culicoides	Culicoides bolitinos	SPECIES	-21.26000	55.55000	Ref.2
70	Diptera	Ceratopogonidae	Culicoides	Culicoides bolitinos	SPECIES	-21.23000	55.55000	Ref.2
71	Diptera	Ceratopogonidae	Culicoides	Culicoides bolitinos	SPECIES	-21.23000	55.44000	Ref.2
72	Diptera	Ceratopogonidae	Culicoides	Culicoides bolitinos	SPECIES	-21.18000	55.33000	Ref.2
73	Diptera	Ceratopogonidae	Culicoides	Culicoides bolitinos	SPECIES	-21.16000	55.32000	Ref.2
74	Diptera	Ceratopogonidae	Culicoides	Culicoides bolitinos	SPECIES	-21.15000	55.46000	Ref.2
75	Diptera	Ceratopogonidae	Culicoides	Culicoides bolitinos	SPECIES	-21.14000	55.49000	Ref.2
76	Diptera	Ceratopogonidae	Culicoides	Culicoides bolitinos	SPECIES	-21.10000	55.67000	Ref.2
77	Diptera	Ceratopogonidae	Culicoides	Culicoides bolitinos	SPECIES	-21.07000	55.23000	Ref.2
78	Diptera	Ceratopogonidae	Culicoides	Culicoides bolitinos	SPECIES	-21.06000	55.27000	Ref.2
79	Diptera	Ceratopogonidae	Culicoides	Culicoides bolitinos	SPECIES	-21.03000	55.66000	Ref.2
80	Diptera	Ceratopogonidae	Culicoides	Culicoides bolitinos	SPECIES	-20.99000	55.68000	Ref.2
81	Diptera	Ceratopogonidae	Culicoides	Culicoides bolitinos	SPECIES	-20.95000	55.32000	Ref.2

№	Order	Family	Genus	Species	Taxon Rank	Latitude	Longitude	GBIF_ID
82	Diptera	Ceratopogonidae	Culicoides	Culicoides bolitinos	SPECIES	-20.95000	55.61000	Ref.2
83	Diptera	Ceratopogonidae	Culicoides	Culicoides bolitinos	SPECIES	-20.94000	55.52000	Ref.2
84	Diptera	Ceratopogonidae	Culicoides	Culicoides bolitinos	SPECIES	-20.90000	55.40000	Ref.2
85	Diptera	Ceratopogonidae	Culicoides	Culicoides bolitinos	SPECIES	-20.39099	28.94513	Ref.3
86	Diptera	Ceratopogonidae	Culicoides	Culicoides bolitinos	SPECIES	-20.35120	32.60107	Ref.3
87	Diptera	Ceratopogonidae	Culicoides	Culicoides bolitinos	SPECIES	-19.00189	29.66748	Ref.3
88	Diptera	Ceratopogonidae	Culicoides	Culicoides bolitinos	SPECIES	-18.20398	32.90509	Ref.3
89	Diptera	Ceratopogonidae	Culicoides	Culicoides bolitinos	SPECIES	-17.89948	25.73273	Ref.3
90	Diptera	Ceratopogonidae	Culicoides	Culicoides bolitinos	SPECIES	-17.64771	31.09808	Ref.3
91	Diptera	Ceratopogonidae	Culicoides	Culicoides bolitinos	SPECIES	-17.47241	30.60553	Ref.3
92	Diptera	Ceratopogonidae	Culicoides	Culicoides bolitinos	SPECIES	-15.90521	29.68591	Ref.3
93	Diptera	Ceratopogonidae	Culicoides	Culicoides bolitinos	SPECIES	-34.00350	25.57190	Ref.4
94	Diptera	Ceratopogonidae	Culicoides	Culicoides bolitinos	SPECIES	-33.98360	25.46390	Ref.4
95	Diptera	Ceratopogonidae	Culicoides	Culicoides bolitinos	SPECIES	-33.37460	26.30510	Ref.4
96	Diptera	Ceratopogonidae	Culicoides	Culicoides bolitinos	SPECIES	-33.31360	26.68660	Ref.4
97	Diptera	Ceratopogonidae	Culicoides	Culicoides bolitinos	SPECIES	-28.50000	28.53333	Ref.5
98	Diptera	Ceratopogonidae	Culicoides	Culicoides bolitinos	SPECIES	-28.53333	28.41667	Ref.6
99	Diptera	Ceratopogonidae	Culicoides	Culicoides bolitinos	SPECIES	-28.00000	28.51670	Ref.7
100	Diptera	Ceratopogonidae	Culicoides	Culicoides bolitinos	SPECIES	-25.65000	28.18333	Ref.8
101	Diptera	Ceratopogonidae	Culicoides	Culicoides bolitinos	SPECIES	-25.05450	31.13650	Ref.9
102	Diptera	Ceratopogonidae	Culicoides	Culicoides bolitinos	SPECIES	-21.83000	46.93000	Ref.10

№	Order	Family	Genus	Species	Taxon Rank	Latitude	Longitude	GBIF_ID
1	Diptera	Ceratopogonidae	Culicoides	Culicoides brevitarsis	SPECIES	-29.69000	152.93000	3350730773
2	Diptera	Ceratopogonidae	Culicoides	Culicoides brevitarsis	SPECIES	-29.68300	152.93300	2248812598
3	Diptera	Ceratopogonidae	Culicoides	Culicoides brevitarsis	SPECIES	-29.68000	152.93000	3350284150
4	Diptera	Ceratopogonidae	Culicoides	Culicoides brevitarsis	SPECIES	-27.45000	152.65000	2248810147
5	Diptera	Ceratopogonidae	Culicoides	Culicoides brevitarsis	SPECIES	-27.43300	152.65000	2248811025
6	Diptera	Ceratopogonidae	Culicoides	Culicoides brevitarsis	SPECIES	-17.72000	139.57000	3348011594
7	Diptera	Ceratopogonidae	Culicoides	Culicoides brevitarsis	SPECIES	-16.40000	131.01700	2248811138
8	Diptera	Ceratopogonidae	Culicoides	Culicoides brevitarsis	SPECIES	-16.40000	131.02000	3347912633
9	Diptera	Ceratopogonidae	Culicoides	Culicoides brevitarsis	SPECIES	-15.48000	141.74000	3350707883
10	Diptera	Ceratopogonidae	Culicoides	Culicoides brevitarsis	SPECIES	-14.28700	126.64600	2248811368
11	Diptera	Ceratopogonidae	Culicoides	Culicoides brevitarsis	SPECIES	-14.18000	126.38000	3350746806
12	Diptera	Ceratopogonidae	Culicoides	Culicoides brevitarsis	SPECIES	-13.92000	131.05000	3350837855
13	Diptera	Ceratopogonidae	Culicoides	Culicoides brevitarsis	SPECIES	-13.91700	131.05000	2248812074
14	Diptera	Ceratopogonidae	Culicoides	Culicoides brevitarsis	SPECIES	-12.65000	131.33300	2248811330
15	Diptera	Ceratopogonidae	Culicoides	Culicoides brevitarsis	SPECIES	-12.65000	131.33000	3032882717
16	Diptera	Ceratopogonidae	Culicoides	Culicoides brevitarsis	SPECIES	-12.43000	130.92000	2248812853
17	Diptera	Ceratopogonidae	Culicoides	Culicoides brevitarsis	SPECIES	-12.42400	136.43400	2248811475
18	Diptera	Ceratopogonidae	Culicoides	Culicoides brevitarsis	SPECIES	-12.42000	136.43000	3350789947
19	Diptera	Ceratopogonidae	Culicoides	Culicoides brevitarsis	SPECIES	-11.27000	132.23000	3348039863
20	Diptera	Ceratopogonidae	Culicoides	Culicoides brevitarsis	SPECIES	-10.88300	142.38300	2248812060
21	Diptera	Ceratopogonidae	Culicoides	Culicoides brevitarsis	SPECIES	-10.85000	142.42000	3350808739
22	Diptera	Ceratopogonidae	Culicoides	Culicoides brevitarsis	SPECIES	-10.31000	150.38000	3350859795
23	Diptera	Ceratopogonidae	Culicoides	Culicoides brevitarsis	SPECIES	-9.52000	125.43000	3987351365
24	Diptera	Ceratopogonidae	Culicoides	Culicoides brevitarsis	SPECIES	-9.51700	125.43300	2248810974
25	Diptera	Ceratopogonidae	Culicoides	Culicoides brevitarsis	SPECIES	-9.31000	125.26000	2248811468
26	Diptera	Ceratopogonidae	Culicoides	Culicoides brevitarsis	SPECIES	-9.29000	124.28000	3968895836
27	Diptera	Ceratopogonidae	Culicoides	Culicoides brevitarsis	SPECIES	-9.20000	124.38000	2248812081
28	Diptera	Ceratopogonidae	Culicoides	Culicoides brevitarsis	SPECIES	-9.20000	124.38300	2248812693
29	Diptera	Ceratopogonidae	Culicoides	Culicoides brevitarsis	SPECIES	-9.06000	125.54000	3347883626
30	Diptera	Ceratopogonidae	Culicoides	Culicoides brevitarsis	SPECIES	-8.95000	160.76700	2249073312
31	Diptera	Ceratopogonidae	Culicoides	Culicoides brevitarsis	SPECIES	-8.94000	160.77900	2248812134
32	Diptera	Ceratopogonidae	Culicoides	Culicoides brevitarsis	SPECIES	-8.94000	160.77000	3350372303
33	Diptera	Ceratopogonidae	Culicoides	Culicoides brevitarsis	SPECIES	-8.94000	160.78000	3350845591
34	Diptera	Ceratopogonidae	Culicoides	Culicoides brevitarsis	SPECIES	-8.93700	160.77400	2248811602
35	Diptera	Ceratopogonidae	Culicoides	Culicoides brevitarsis	SPECIES	-8.86000	126.36000	2248812070
36	Diptera	Ceratopogonidae	Culicoides	Culicoides brevitarsis	SPECIES	-8.82000	125.85400	2248810362
37	Diptera	Ceratopogonidae	Culicoides	Culicoides brevitarsis	SPECIES	-8.52000	126.99500	2248810087
38	Diptera	Ceratopogonidae	Culicoides	Culicoides brevitarsis	SPECIES	-8.52000	127.00000	3350868915
39	Diptera	Ceratopogonidae	Culicoides	Culicoides brevitarsis	SPECIES	-7.09500	155.86100	2249073111
40	Diptera	Ceratopogonidae	Culicoides	Culicoides brevitarsis	SPECIES	-7.09200	155.86300	2249073113
41	Diptera	Ceratopogonidae	Culicoides	Culicoides brevitarsis	SPECIES	-7.09000	155.86000	3347883625

№	Order	Family	Genus	Species	Taxon Rank	Latitude	Longitude	GBIF_ID
42	Diptera	Ceratopogonidae	Culicoides	Culicoides brevitarsis	SPECIES	-6.54500	146.64500	2248812282
43	Diptera	Ceratopogonidae	Culicoides	Culicoides brevitarsis	SPECIES	-6.54000	146.65000	3350818835
44	Diptera	Ceratopogonidae	Culicoides	Culicoides brevitarsis	SPECIES	-3.58000	143.65000	3347906894
45	Diptera	Ceratopogonidae	Culicoides	Culicoides brevitarsis	SPECIES	-3.57800	143.64600	2248811987
46	Diptera	Ceratopogonidae	Culicoides	Culicoides brevitarsis	SPECIES	-3.17000	142.35000	3350753877
47	Diptera	Ceratopogonidae	Culicoides	Culicoides brevitarsis	SPECIES	-2.85100	141.29300	2248812942
48	Diptera	Ceratopogonidae	Culicoides	Culicoides brevitarsis	SPECIES	-2.85100	141.28400	2249073389
49	Diptera	Ceratopogonidae	Culicoides	Culicoides brevitarsis	SPECIES	-2.85000	141.28000	3350752004
50	Diptera	Ceratopogonidae	Culicoides	Culicoides brevitarsis	SPECIES	10.43000	78.82000	3348255516
51	Diptera	Ceratopogonidae	Culicoides	Culicoides brevitarsis	SPECIES	10.43300	78.81700	2249073510
52	Diptera	Ceratopogonidae	Culicoides	Culicoides brevitarsis	SPECIES	20.08000	110.37000	2249073323
53	Diptera	Ceratopogonidae	Culicoides	Culicoides brevitarsis	SPECIES	27.02000	74.21000	3348488537
54	Diptera	Ceratopogonidae	Culicoides	Culicoides brevitarsis	SPECIES	30.06000	79.01000	3346579576

№	Order	Family	Genus	Species	Taxon Rank	Latitude	Longitude	GBIF_ID
1	Diptera	Ceratopogonidae	Culicoides	Culicoides imicola	SPECIES	-29.61667	30.38333	1325716921
2	Diptera	Ceratopogonidae	Culicoides	Culicoides imicola	SPECIES	-26.02600	27.54500	1415483447
3	Diptera	Ceratopogonidae	Culicoides	Culicoides imicola	SPECIES	-25.75860	23.71550	2248515048
4	Diptera	Ceratopogonidae	Culicoides	Culicoides imicola	SPECIES	-25.62870	32.36270	2248514518
5	Diptera	Ceratopogonidae	Culicoides	Culicoides imicola	SPECIES	-25.48000	28.22000	3987384082
6	Diptera	Ceratopogonidae	Culicoides	Culicoides imicola	SPECIES	-25.48000	28.22000	3032889788
7	Diptera	Ceratopogonidae	Culicoides	Culicoides imicola	SPECIES	-25.47200	31.50800	3443249372
8	Diptera	Ceratopogonidae	Culicoides	Culicoides imicola	SPECIES	-25.35400	31.89500	3443399997
9	Diptera	Ceratopogonidae	Culicoides	Culicoides imicola	SPECIES	-25.32600	31.39200	3442613189
10	Diptera	Ceratopogonidae	Culicoides	Culicoides imicola	SPECIES	-25.16800	31.27000	3442816335
11	Diptera	Ceratopogonidae	Culicoides	Culicoides imicola	SPECIES	-25.12300	31.91600	3443499049
12	Diptera	Ceratopogonidae	Culicoides	Culicoides imicola	SPECIES	-24.99300	31.58400	3442899743
13	Diptera	Ceratopogonidae	Culicoides	Culicoides imicola	SPECIES	-24.99290	31.58420	2251325686
14	Diptera	Ceratopogonidae	Culicoides	Culicoides imicola	SPECIES	-24.78300	31.85900	3442816756
15	Diptera	Ceratopogonidae	Culicoides	Culicoides imicola	SPECIES	-24.47200	31.97800	3442817683
16	Diptera	Ceratopogonidae	Culicoides	Culicoides imicola	SPECIES	-24.39700	31.77700	3443229549
17	Diptera	Ceratopogonidae	Culicoides	Culicoides imicola	SPECIES	-24.10300	31.66900	3442560898
18	Diptera	Ceratopogonidae	Culicoides	Culicoides imicola	SPECIES	-24.00300	31.74300	3442573310
19	Diptera	Ceratopogonidae	Culicoides	Culicoides imicola	SPECIES	-23.94400	31.16800	3442903425
20	Diptera	Ceratopogonidae	Culicoides	Culicoides imicola	SPECIES	-23.85200	31.57400	3442731602
21	Diptera	Ceratopogonidae	Culicoides	Culicoides imicola	SPECIES	-23.64900	31.14700	3441580452
22	Diptera	Ceratopogonidae	Culicoides	Culicoides imicola	SPECIES	-23.51400	31.40000	3442573802
23	Diptera	Ceratopogonidae	Culicoides	Culicoides imicola	SPECIES	-23.22200	31.21200	3442565006
24	Diptera	Ceratopogonidae	Culicoides	Culicoides imicola	SPECIES	-22.42000	31.22940	3442218381
25	Diptera	Ceratopogonidae	Culicoides	Culicoides imicola	SPECIES	-21.97260	16.92260	2633764963
26	Diptera	Ceratopogonidae	Culicoides	Culicoides imicola	SPECIES	-21.97000	16.92000	3349069424
27	Diptera	Ceratopogonidae	Culicoides	Culicoides imicola	SPECIES	-21.23000	55.54000	3350019807
28	Diptera	Ceratopogonidae	Culicoides	Culicoides imicola	SPECIES	-21.23000	55.54000	2249957990
29	Diptera	Ceratopogonidae	Culicoides	Culicoides imicola	SPECIES	-21.13317	55.53241	4050500318
30	Diptera	Ceratopogonidae	Culicoides	Culicoides imicola	SPECIES	-21.07000	55.22000	3987235265
31	Diptera	Ceratopogonidae	Culicoides	Culicoides imicola	SPECIES	-21.07000	55.22000	3032893774
32	Diptera	Ceratopogonidae	Culicoides	Culicoides imicola	SPECIES	-20.48000	57.37000	3969046767
33	Diptera	Ceratopogonidae	Culicoides	Culicoides imicola	SPECIES	-20.32000	57.42000	3990279409
34	Diptera	Ceratopogonidae	Culicoides	Culicoides imicola	SPECIES	-20.32000	57.42000	3032880640
35	Diptera	Ceratopogonidae	Culicoides	Culicoides imicola	SPECIES	-18.13050	44.90970	2248515562
36	Diptera	Ceratopogonidae	Culicoides	Culicoides imicola	SPECIES	-18.02000	44.48000	3987260910
37	Diptera	Ceratopogonidae	Culicoides	Culicoides imicola	SPECIES	-17.83560	29.94440	2248514590
38	Diptera	Ceratopogonidae	Culicoides	Culicoides imicola	SPECIES	-12.82060	45.14743	4050503272
39	Diptera	Ceratopogonidae	Culicoides	Culicoides imicola	SPECIES	0.29300	36.89900	2979440929
40	Diptera	Ceratopogonidae	Culicoides	Culicoides imicola	SPECIES	6.36890	2.06360	2248515701
41	Diptera	Ceratopogonidae	Culicoides	Culicoides imicola	SPECIES	6.50000	2.13000	3817968610

№	Order	Family	Genus	Species	Taxon Rank	Latitude	Longitude	GBIF_ID
42	Diptera	Ceratopogonidae	Culicoides	Culicoides imicola	SPECIES	7.47000	13.55000	4008311544
43	Diptera	Ceratopogonidae	Culicoides	Culicoides imicola	SPECIES	8.85000	40.72000	4042002153
44	Diptera	Ceratopogonidae	Culicoides	Culicoides imicola	SPECIES	8.85000	40.72000	3032890734
45	Diptera	Ceratopogonidae	Culicoides	Culicoides imicola	SPECIES	9.08333	37.75880	2248514548
46	Diptera	Ceratopogonidae	Culicoides	Culicoides imicola	SPECIES	11.06700	77.05000	2249073495
47	Diptera	Ceratopogonidae	Culicoides	Culicoides imicola	SPECIES	11.07000	77.05000	3350975553
48	Diptera	Ceratopogonidae	Culicoides	Culicoides imicola	SPECIES	11.17000	-4.28000	3351718391
49	Diptera	Ceratopogonidae	Culicoides	Culicoides imicola	SPECIES	11.57000	41.27000	3990315741
50	Diptera	Ceratopogonidae	Culicoides	Culicoides imicola	SPECIES	13.03700	77.58500	2249073504
51	Diptera	Ceratopogonidae	Culicoides	Culicoides imicola	SPECIES	13.04000	77.58000	3350967330
52	Diptera	Ceratopogonidae	Culicoides	Culicoides imicola	SPECIES	13.25140	-15.79550	2248515980
53	Diptera	Ceratopogonidae	Culicoides	Culicoides imicola	SPECIES	14.14000	99.14000	3818494602
54	Diptera	Ceratopogonidae	Culicoides	Culicoides imicola	SPECIES	14.14000	99.14000	3443468585
55	Diptera	Ceratopogonidae	Culicoides	Culicoides imicola	SPECIES	14.72780	-17.43420	2248515018
56	Diptera	Ceratopogonidae	Culicoides	Culicoides imicola	SPECIES	14.72990	-17.32580	2248515137
57	Diptera	Ceratopogonidae	Culicoides	Culicoides imicola	SPECIES	14.76640	-17.03540	2248515637
58	Diptera	Ceratopogonidae	Culicoides	Culicoides imicola	SPECIES	14.77000	-17.06210	2248514960
59	Diptera	Ceratopogonidae	Culicoides	Culicoides imicola	SPECIES	16.26430	-1.13680	2248515005
60	Diptera	Ceratopogonidae	Culicoides	Culicoides imicola	SPECIES	24.45000	118.38000	3032867743
61	Diptera	Ceratopogonidae	Culicoides	Culicoides imicola	SPECIES	27.02000	74.21000	3346593508
62	Diptera	Ceratopogonidae	Culicoides	Culicoides imicola	SPECIES	27.17000	78.00000	3348473601
63	Diptera	Ceratopogonidae	Culicoides	Culicoides imicola	SPECIES	30.06000	79.01000	3351201450
64	Diptera	Ceratopogonidae	Culicoides	Culicoides imicola	SPECIES	30.06000	79.01000	2249578679
65	Diptera	Ceratopogonidae	Culicoides	Culicoides imicola	SPECIES	30.19090	66.96120	2979636051
66	Diptera	Ceratopogonidae	Culicoides	Culicoides imicola	SPECIES	31.14000	75.34000	3351202612
67	Diptera	Ceratopogonidae	Culicoides	Culicoides imicola	SPECIES	31.20400	29.94960	2250441367
68	Diptera	Ceratopogonidae	Culicoides	Culicoides imicola	SPECIES	32.93960	72.68130	2979571906
69	Diptera	Ceratopogonidae	Culicoides	Culicoides imicola	SPECIES	33.89610	-6.83147	2979482531
70	Diptera	Ceratopogonidae	Culicoides	Culicoides imicola	SPECIES	33.90000	-6.83000	3349226038
71	Diptera	Ceratopogonidae	Culicoides	Culicoides imicola	SPECIES	36.85000	7.28000	4017185543
72	Diptera	Ceratopogonidae	Culicoides	Culicoides imicola	SPECIES	36.85000	7.28000	3032880641
73	Diptera	Ceratopogonidae	Culicoides	Culicoides imicola	SPECIES	41.50000	9.08000	3969085369
74	Diptera	Ceratopogonidae	Culicoides	Culicoides imicola	SPECIES	43.40000	6.68000	3032815762
75	Diptera	Ceratopogonidae	Culicoides	Culicoides imicola	SPECIES	43.40000	6.68000	3818389594
76	Diptera	Ceratopogonidae	Culicoides	Culicoides imicola	SPECIES	-33.97500	18.64200	Ref.11
77	Diptera	Ceratopogonidae	Culicoides	Culicoides imicola	SPECIES	-33.97200	23.87800	Ref.11
78	Diptera	Ceratopogonidae	Culicoides	Culicoides imicola	SPECIES	-33.96700	22.46700	Ref.11
79	Diptera	Ceratopogonidae	Culicoides	Culicoides imicola	SPECIES	-33.96500	25.53000	Ref.11
80	Diptera	Ceratopogonidae	Culicoides	Culicoides imicola	SPECIES	-33.93300	18.86700	Ref.11
81	Diptera	Ceratopogonidae	Culicoides	Culicoides imicola	SPECIES	-33.90000	25.50000	Ref.11
82	Diptera	Ceratopogonidae	Culicoides	Culicoides imicola	SPECIES	-33.76300	25.39300	Ref.11

№	Order	Family	Genus	Species	Taxon Rank	Latitude	Longitude	GBIF_ID
83	Diptera	Ceratopogonidae	Culicoides	Culicoides imicola	SPECIES	-33.48300	26.83300	Ref.11
84	Diptera	Ceratopogonidae	Culicoides	Culicoides imicola	SPECIES	-33.36700	24.43300	Ref.11
85	Diptera	Ceratopogonidae	Culicoides	Culicoides imicola	SPECIES	-32.63300	26.33300	Ref.11
86	Diptera	Ceratopogonidae	Culicoides	Culicoides imicola	SPECIES	-32.51700	27.46700	Ref.11
87	Diptera	Ceratopogonidae	Culicoides	Culicoides imicola	SPECIES	-32.25200	24.54000	Ref.11
88	Diptera	Ceratopogonidae	Culicoides	Culicoides imicola	SPECIES	-31.50700	25.01800	Ref.11
89	Diptera	Ceratopogonidae	Culicoides	Culicoides imicola	SPECIES	-30.77800	25.79500	Ref.11
90	Diptera	Ceratopogonidae	Culicoides	Culicoides imicola	SPECIES	-30.71800	25.09700	Ref.11
91	Diptera	Ceratopogonidae	Culicoides	Culicoides imicola	SPECIES	-30.26200	25.70300	Ref.11
92	Diptera	Ceratopogonidae	Culicoides	Culicoides imicola	SPECIES	-29.91300	30.98500	Ref.11
93	Diptera	Ceratopogonidae	Culicoides	Culicoides imicola	SPECIES	-29.85700	30.72000	Ref.11
94	Diptera	Ceratopogonidae	Culicoides	Culicoides imicola	SPECIES	-29.75000	17.15000	Ref.11
95	Diptera	Ceratopogonidae	Culicoides	Culicoides imicola	SPECIES	-29.66700	30.41700	Ref.11
96	Diptera	Ceratopogonidae	Culicoides	Culicoides imicola	SPECIES	-29.61700	30.38300	Ref.11
97	Diptera	Ceratopogonidae	Culicoides	Culicoides imicola	SPECIES	-29.53300	30.28300	Ref.11
98	Diptera	Ceratopogonidae	Culicoides	Culicoides imicola	SPECIES	-29.45000	27.75000	Ref.11
99	Diptera	Ceratopogonidae	Culicoides	Culicoides imicola	SPECIES	-29.17800	31.41300	Ref.11
100	Diptera	Ceratopogonidae	Culicoides	Culicoides imicola	SPECIES	-29.14800	27.74000	Ref.11
101	Diptera	Ceratopogonidae	Culicoides	Culicoides imicola	SPECIES	-28.95000	31.78300	Ref.11
102	Diptera	Ceratopogonidae	Culicoides	Culicoides imicola	SPECIES	-28.95000	26.33300	Ref.11
103	Diptera	Ceratopogonidae	Culicoides	Culicoides imicola	SPECIES	-28.81700	24.75000	Ref.11
104	Diptera	Ceratopogonidae	Culicoides	Culicoides imicola	SPECIES	-28.53300	28.41700	Ref.11
105	Diptera	Ceratopogonidae	Culicoides	Culicoides imicola	SPECIES	-28.47200	20.18800	Ref.11
106	Diptera	Ceratopogonidae	Culicoides	Culicoides imicola	SPECIES	-28.46700	32.16700	Ref.11
107	Diptera	Ceratopogonidae	Culicoides	Culicoides imicola	SPECIES	-28.46700	21.33300	Ref.11
108	Diptera	Ceratopogonidae	Culicoides	Culicoides imicola	SPECIES	-28.45800	21.24300	Ref.11
109	Diptera	Ceratopogonidae	Culicoides	Culicoides imicola	SPECIES	-28.36700	28.08300	Ref.11
110	Diptera	Ceratopogonidae	Culicoides	Culicoides imicola	SPECIES	-28.08300	32.33300	Ref.11
111	Diptera	Ceratopogonidae	Culicoides	Culicoides imicola	SPECIES	-28.02200	32.35800	Ref.11
112	Diptera	Ceratopogonidae	Culicoides	Culicoides imicola	SPECIES	-26.88800	32.27700	Ref.11
113	Diptera	Ceratopogonidae	Culicoides	Culicoides imicola	SPECIES	-26.66000	16.25000	Ref.11
114	Diptera	Ceratopogonidae	Culicoides	Culicoides imicola	SPECIES	-26.64500	31.35300	Ref.11
115	Diptera	Ceratopogonidae	Culicoides	Culicoides imicola	SPECIES	-26.58300	27.23300	Ref.11
116	Diptera	Ceratopogonidae	Culicoides	Culicoides imicola	SPECIES	-26.51700	29.98300	Ref.11
117	Diptera	Ceratopogonidae	Culicoides	Culicoides imicola	SPECIES	-26.20200	28.04500	Ref.11
118	Diptera	Ceratopogonidae	Culicoides	Culicoides imicola	SPECIES	-26.08000	27.92700	Ref.11
119	Diptera	Ceratopogonidae	Culicoides	Culicoides imicola	SPECIES	-25.91700	24.66700	Ref.11
120	Diptera	Ceratopogonidae	Culicoides	Culicoides imicola	SPECIES	-25.90000	32.50000	Ref.11
121	Diptera	Ceratopogonidae	Culicoides	Culicoides imicola	SPECIES	-25.83300	28.20000	Ref.11
122	Diptera	Ceratopogonidae	Culicoides	Culicoides imicola	SPECIES	-25.83300	27.83300	Ref.11
123	Diptera	Ceratopogonidae	Culicoides	Culicoides imicola	SPECIES	-25.80000	29.73300	Ref.11

№	Order	Family	Genus	Species	Taxon Rank	Latitude	Longitude	GBIF_ID
124	Diptera	Ceratopogonidae	Culicoides	Culicoides imicola	SPECIES	-25.76800	29.46000	Ref.11
125	Diptera	Ceratopogonidae	Culicoides	Culicoides imicola	SPECIES	-25.75700	28.25300	Ref.11
126	Diptera	Ceratopogonidae	Culicoides	Culicoides imicola	SPECIES	-25.75000	28.18300	Ref.11
127	Diptera	Ceratopogonidae	Culicoides	Culicoides imicola	SPECIES	-25.68600	28.28800	Ref.11
128	Diptera	Ceratopogonidae	Culicoides	Culicoides imicola	SPECIES	-25.67500	28.52500	Ref.11
129	Diptera	Ceratopogonidae	Culicoides	Culicoides imicola	SPECIES	-25.66500	27.97200	Ref.11
130	Diptera	Ceratopogonidae	Culicoides	Culicoides imicola	SPECIES	-25.66300	28.03600	Ref.11
131	Diptera	Ceratopogonidae	Culicoides	Culicoides imicola	SPECIES	-25.65800	28.20200	Ref.11
132	Diptera	Ceratopogonidae	Culicoides	Culicoides imicola	SPECIES	-25.64400	28.31700	Ref.11
133	Diptera	Ceratopogonidae	Culicoides	Culicoides imicola	SPECIES	-25.63500	28.15900	Ref.11
134	Diptera	Ceratopogonidae	Culicoides	Culicoides imicola	SPECIES	-25.59400	28.21400	Ref.11
135	Diptera	Ceratopogonidae	Culicoides	Culicoides imicola	SPECIES	-25.48300	28.18300	Ref.11
136	Diptera	Ceratopogonidae	Culicoides	Culicoides imicola	SPECIES	-25.46700	29.43300	Ref.11
137	Diptera	Ceratopogonidae	Culicoides	Culicoides imicola	SPECIES	-25.46500	30.98500	Ref.11
138	Diptera	Ceratopogonidae	Culicoides	Culicoides imicola	SPECIES	-25.45800	28.26700	Ref.11
139	Diptera	Ceratopogonidae	Culicoides	Culicoides imicola	SPECIES	-25.32200	31.02200	Ref.11
140	Diptera	Ceratopogonidae	Culicoides	Culicoides imicola	SPECIES	-25.25000	29.43300	Ref.11
141	Diptera	Ceratopogonidae	Culicoides	Culicoides imicola	SPECIES	-25.09500	30.78700	Ref.11
142	Diptera	Ceratopogonidae	Culicoides	Culicoides imicola	SPECIES	-24.98300	31.60000	Ref.11
143	Diptera	Ceratopogonidae	Culicoides	Culicoides imicola	SPECIES	-24.95200	31.37200	Ref.11
144	Diptera	Ceratopogonidae	Culicoides	Culicoides imicola	SPECIES	-24.89200	30.75500	Ref.11
145	Diptera	Ceratopogonidae	Culicoides	Culicoides imicola	SPECIES	-24.71000	31.35000	Ref.11
146	Diptera	Ceratopogonidae	Culicoides	Culicoides imicola	SPECIES	-24.35000	30.31300	Ref.11
147	Diptera	Ceratopogonidae	Culicoides	Culicoides imicola	SPECIES	-24.26000	27.32300	Ref.11
148	Diptera	Ceratopogonidae	Culicoides	Culicoides imicola	SPECIES	-23.93300	31.11700	Ref.11
149	Diptera	Ceratopogonidae	Culicoides	Culicoides imicola	SPECIES	-23.83300	30.16700	Ref.11
150	Diptera	Ceratopogonidae	Culicoides	Culicoides imicola	SPECIES	-23.82200	31.44500	Ref.11
151	Diptera	Ceratopogonidae	Culicoides	Culicoides imicola	SPECIES	-23.69600	30.67200	Ref.11
152	Diptera	Ceratopogonidae	Culicoides	Culicoides imicola	SPECIES	-23.66700	30.75000	Ref.11
153	Diptera	Ceratopogonidae	Culicoides	Culicoides imicola	SPECIES	-23.59200	30.09200	Ref.11
154	Diptera	Ceratopogonidae	Culicoides	Culicoides imicola	SPECIES	-23.10700	31.05200	Ref.11
155	Diptera	Ceratopogonidae	Culicoides	Culicoides imicola	SPECIES	-23.08300	29.08300	Ref.11
156	Diptera	Ceratopogonidae	Culicoides	Culicoides imicola	SPECIES	-23.05000	29.90000	Ref.11
157	Diptera	Ceratopogonidae	Culicoides	Culicoides imicola	SPECIES	-22.98300	30.21700	Ref.11
158	Diptera	Ceratopogonidae	Culicoides	Culicoides imicola	SPECIES	-22.97000	29.33200	Ref.11
159	Diptera	Ceratopogonidae	Culicoides	Culicoides imicola	SPECIES	-22.80700	30.55500	Ref.11
160	Diptera	Ceratopogonidae	Culicoides	Culicoides imicola	SPECIES	-22.69200	31.01000	Ref.11
161	Diptera	Ceratopogonidae	Culicoides	Culicoides imicola	SPECIES	-22.60000	30.14500	Ref.11
162	Diptera	Ceratopogonidae	Culicoides	Culicoides imicola	SPECIES	-22.58000	17.13000	Ref.11
163	Diptera	Ceratopogonidae	Culicoides	Culicoides imicola	SPECIES	-22.55800	17.08200	Ref.11
164	Diptera	Ceratopogonidae	Culicoides	Culicoides imicola	SPECIES	-22.55000	30.25000	Ref.11

№	Order	Family	Genus	Species	Taxon Rank	Latitude	Longitude	GBIF_ID
165	Diptera	Ceratopogonidae	Culicoides	Culicoides imicola	SPECIES	-22.45000	17.59000	Ref.11
166	Diptera	Ceratopogonidae	Culicoides	Culicoides imicola	SPECIES	-22.44800	31.31000	Ref.11
167	Diptera	Ceratopogonidae	Culicoides	Culicoides imicola	SPECIES	-22.33300	29.91700	Ref.11
168	Diptera	Ceratopogonidae	Culicoides	Culicoides imicola	SPECIES	-22.06000	18.19000	Ref.11
169	Diptera	Ceratopogonidae	Culicoides	Culicoides imicola	SPECIES	-21.98000	16.92000	Ref.11
170	Diptera	Ceratopogonidae	Culicoides	Culicoides imicola	SPECIES	-21.94000	15.84000	Ref.11
171	Diptera	Ceratopogonidae	Culicoides	Culicoides imicola	SPECIES	-21.90000	31.60000	Ref.11
172	Diptera	Ceratopogonidae	Culicoides	Culicoides imicola	SPECIES	-21.83000	46.93000	Ref.11
173	Diptera	Ceratopogonidae	Culicoides	Culicoides imicola	SPECIES	-21.72000	19.35000	Ref.11
174	Diptera	Ceratopogonidae	Culicoides	Culicoides imicola	SPECIES	-21.47000	15.92000	Ref.11
175	Diptera	Ceratopogonidae	Culicoides	Culicoides imicola	SPECIES	-20.81700	28.98300	Ref.11
176	Diptera	Ceratopogonidae	Culicoides	Culicoides imicola	SPECIES	-20.28300	57.43300	Ref.11
177	Diptera	Ceratopogonidae	Culicoides	Culicoides imicola	SPECIES	-20.23300	32.66700	Ref.11
178	Diptera	Ceratopogonidae	Culicoides	Culicoides imicola	SPECIES	-20.20000	57.50000	Ref.11
179	Diptera	Ceratopogonidae	Culicoides	Culicoides imicola	SPECIES	-19.13300	29.91700	Ref.11
180	Diptera	Ceratopogonidae	Culicoides	Culicoides imicola	SPECIES	-18.53300	32.01700	Ref.11
181	Diptera	Ceratopogonidae	Culicoides	Culicoides imicola	SPECIES	-18.00000	47.40000	Ref.11
182	Diptera	Ceratopogonidae	Culicoides	Culicoides imicola	SPECIES	-17.96800	49.23100	Ref.11
183	Diptera	Ceratopogonidae	Culicoides	Culicoides imicola	SPECIES	-17.92000	31.04000	Ref.11
184	Diptera	Ceratopogonidae	Culicoides	Culicoides imicola	SPECIES	-17.83000	31.05300	Ref.11
185	Diptera	Ceratopogonidae	Culicoides	Culicoides imicola	SPECIES	-17.78300	31.08300	Ref.11
186	Diptera	Ceratopogonidae	Culicoides	Culicoides imicola	SPECIES	-17.75000	30.91700	Ref.11
187	Diptera	Ceratopogonidae	Culicoides	Culicoides imicola	SPECIES	-17.73300	31.18300	Ref.11
188	Diptera	Ceratopogonidae	Culicoides	Culicoides imicola	SPECIES	-17.66700	30.71700	Ref.11
189	Diptera	Ceratopogonidae	Culicoides	Culicoides imicola	SPECIES	-17.65000	30.78300	Ref.11
190	Diptera	Ceratopogonidae	Culicoides	Culicoides imicola	SPECIES	-17.50000	30.49000	Ref.11
191	Diptera	Ceratopogonidae	Culicoides	Culicoides imicola	SPECIES	-17.48300	30.15000	Ref.11
192	Diptera	Ceratopogonidae	Culicoides	Culicoides imicola	SPECIES	-16.52200	28.85200	Ref.11
193	Diptera	Ceratopogonidae	Culicoides	Culicoides imicola	SPECIES	-16.26700	31.25000	Ref.11
194	Diptera	Ceratopogonidae	Culicoides	Culicoides imicola	SPECIES	-16.13300	29.40000	Ref.11
195	Diptera	Ceratopogonidae	Culicoides	Culicoides imicola	SPECIES	-16.11000	29.58000	Ref.11
196	Diptera	Ceratopogonidae	Culicoides	Culicoides imicola	SPECIES	-13.03800	14.24800	Ref.11
197	Diptera	Ceratopogonidae	Culicoides	Culicoides imicola	SPECIES	-11.46500	34.02300	Ref.11
198	Diptera	Ceratopogonidae	Culicoides	Culicoides imicola	SPECIES	-7.36700	20.81700	Ref.11
199	Diptera	Ceratopogonidae	Culicoides	Culicoides imicola	SPECIES	-5.17700	38.79200	Ref.11
200	Diptera	Ceratopogonidae	Culicoides	Culicoides imicola	SPECIES	-5.11700	12.65000	Ref.11
201	Diptera	Ceratopogonidae	Culicoides	Culicoides imicola	SPECIES	-5.10000	38.63300	Ref.11
202	Diptera	Ceratopogonidae	Culicoides	Culicoides imicola	SPECIES	-4.28300	39.56700	Ref.11
203	Diptera	Ceratopogonidae	Culicoides	Culicoides imicola	SPECIES	-4.23300	12.43300	Ref.11
204	Diptera	Ceratopogonidae	Culicoides	Culicoides imicola	SPECIES	-3.41700	38.58300	Ref.11
205	Diptera	Ceratopogonidae	Culicoides	Culicoides imicola	SPECIES	-3.31700	40.03300	Ref.11

№	Order	Family	Genus	Species	Taxon Rank	Latitude	Longitude	GBIF_ID
206	Diptera	Ceratopogonidae	Culicoides	Culicoides imicola	SPECIES	-3.06700	39.33300	Ref.11
207	Diptera	Ceratopogonidae	Culicoides	Culicoides imicola	SPECIES	-2.63300	37.26700	Ref.11
208	Diptera	Ceratopogonidae	Culicoides	Culicoides imicola	SPECIES	-2.55000	36.78300	Ref.11
209	Diptera	Ceratopogonidae	Culicoides	Culicoides imicola	SPECIES	-2.53300	36.60000	Ref.11
210	Diptera	Ceratopogonidae	Culicoides	Culicoides imicola	SPECIES	-2.51700	37.66700	Ref.11
211	Diptera	Ceratopogonidae	Culicoides	Culicoides imicola	SPECIES	-2.21700	37.73300	Ref.11
212	Diptera	Ceratopogonidae	Culicoides	Culicoides imicola	SPECIES	-2.08300	38.15000	Ref.11
213	Diptera	Ceratopogonidae	Culicoides	Culicoides imicola	SPECIES	-1.95000	36.78300	Ref.11
214	Diptera	Ceratopogonidae	Culicoides	Culicoides imicola	SPECIES	-1.90000	36.11700	Ref.11
215	Diptera	Ceratopogonidae	Culicoides	Culicoides imicola	SPECIES	-1.53300	37.23300	Ref.11
216	Diptera	Ceratopogonidae	Culicoides	Culicoides imicola	SPECIES	-1.46700	37.06700	Ref.11
217	Diptera	Ceratopogonidae	Culicoides	Culicoides imicola	SPECIES	-1.36700	36.96700	Ref.11
218	Diptera	Ceratopogonidae	Culicoides	Culicoides imicola	SPECIES	-1.32200	36.68500	Ref.11
219	Diptera	Ceratopogonidae	Culicoides	Culicoides imicola	SPECIES	-1.28300	36.81700	Ref.11
220	Diptera	Ceratopogonidae	Culicoides	Culicoides imicola	SPECIES	-1.28300	36.95000	Ref.11
221	Diptera	Ceratopogonidae	Culicoides	Culicoides imicola	SPECIES	-1.26700	36.73300	Ref.11
222	Diptera	Ceratopogonidae	Culicoides	Culicoides imicola	SPECIES	-1.21700	37.05000	Ref.11
223	Diptera	Ceratopogonidae	Culicoides	Culicoides imicola	SPECIES	-1.20000	36.96700	Ref.11
224	Diptera	Ceratopogonidae	Culicoides	Culicoides imicola	SPECIES	-1.17500	36.83000	Ref.11
225	Diptera	Ceratopogonidae	Culicoides	Culicoides imicola	SPECIES	-1.13300	36.78300	Ref.11
226	Diptera	Ceratopogonidae	Culicoides	Culicoides imicola	SPECIES	-0.86700	36.41700	Ref.11
227	Diptera	Ceratopogonidae	Culicoides	Culicoides imicola	SPECIES	-0.73300	34.38300	Ref.11
228	Diptera	Ceratopogonidae	Culicoides	Culicoides imicola	SPECIES	-0.71700	37.36700	Ref.11
229	Diptera	Ceratopogonidae	Culicoides	Culicoides imicola	SPECIES	-0.71000	36.43800	Ref.11
230	Diptera	Ceratopogonidae	Culicoides	Culicoides imicola	SPECIES	-0.63300	34.33300	Ref.11
231	Diptera	Ceratopogonidae	Culicoides	Culicoides imicola	SPECIES	-0.56700	36.41700	Ref.11
232	Diptera	Ceratopogonidae	Culicoides	Culicoides imicola	SPECIES	-0.56700	36.36700	Ref.11
233	Diptera	Ceratopogonidae	Culicoides	Culicoides imicola	SPECIES	-0.53300	37.46700	Ref.11
234	Diptera	Ceratopogonidae	Culicoides	Culicoides imicola	SPECIES	-0.47000	35.99000	Ref.11
235	Diptera	Ceratopogonidae	Culicoides	Culicoides imicola	SPECIES	-0.41700	36.11700	Ref.11
236	Diptera	Ceratopogonidae	Culicoides	Culicoides imicola	SPECIES	-0.40000	36.20000	Ref.11
237	Diptera	Ceratopogonidae	Culicoides	Culicoides imicola	SPECIES	-0.28300	35.68300	Ref.11
238	Diptera	Ceratopogonidae	Culicoides	Culicoides imicola	SPECIES	0.03300	37.16700	Ref.11
239	Diptera	Ceratopogonidae	Culicoides	Culicoides imicola	SPECIES	0.05300	32.46500	Ref.11
240	Diptera	Ceratopogonidae	Culicoides	Culicoides imicola	SPECIES	0.10000	37.10000	Ref.11
241	Diptera	Ceratopogonidae	Culicoides	Culicoides imicola	SPECIES	0.10000	36.55000	Ref.11
242	Diptera	Ceratopogonidae	Culicoides	Culicoides imicola	SPECIES	0.12500	34.84500	Ref.11
243	Diptera	Ceratopogonidae	Culicoides	Culicoides imicola	SPECIES	0.25000	32.08300	Ref.11
244	Diptera	Ceratopogonidae	Culicoides	Culicoides imicola	SPECIES	0.28300	36.06700	Ref.11
245	Diptera	Ceratopogonidae	Culicoides	Culicoides imicola	SPECIES	0.43300	37.80000	Ref.11
246	Diptera	Ceratopogonidae	Culicoides	Culicoides imicola	SPECIES	0.50000	35.36700	Ref.11

№	Order	Family	Genus	Species	Taxon Rank	Latitude	Longitude	GBIF_ID
247	Diptera	Ceratopogonidae	Culicoides	Culicoides imicola	SPECIES	0.58300	37.53300	Ref.11
248	Diptera	Ceratopogonidae	Culicoides	Culicoides imicola	SPECIES	0.76700	36.71700	Ref.11
249	Diptera	Ceratopogonidae	Culicoides	Culicoides imicola	SPECIES	0.96700	36.83300	Ref.11
250	Diptera	Ceratopogonidae	Culicoides	Culicoides imicola	SPECIES	1.08300	34.91700	Ref.11
251	Diptera	Ceratopogonidae	Culicoides	Culicoides imicola	SPECIES	1.08300	36.56700	Ref.11
252	Diptera	Ceratopogonidae	Culicoides	Culicoides imicola	SPECIES	1.20000	36.96700	Ref.11
253	Diptera	Ceratopogonidae	Culicoides	Culicoides imicola	SPECIES	3.71000	29.28700	Ref.11
254	Diptera	Ceratopogonidae	Culicoides	Culicoides imicola	SPECIES	4.63300	9.45000	Ref.11
255	Diptera	Ceratopogonidae	Culicoides	Culicoides imicola	SPECIES	5.55500	-0.19700	Ref.11
256	Diptera	Ceratopogonidae	Culicoides	Culicoides imicola	SPECIES	6.97000	-0.97000	Ref.11
257	Diptera	Ceratopogonidae	Culicoides	Culicoides imicola	SPECIES	7.47500	4.55800	Ref.11
258	Diptera	Ceratopogonidae	Culicoides	Culicoides imicola	SPECIES	7.52000	-0.88000	Ref.11
259	Diptera	Ceratopogonidae	Culicoides	Culicoides imicola	SPECIES	7.71000	-0.80000	Ref.11
260	Diptera	Ceratopogonidae	Culicoides	Culicoides imicola	SPECIES	7.75000	-1.05000	Ref.11
261	Diptera	Ceratopogonidae	Culicoides	Culicoides imicola	SPECIES	7.76000	-0.59000	Ref.11
262	Diptera	Ceratopogonidae	Culicoides	Culicoides imicola	SPECIES	7.76000	-0.50000	Ref.11
263	Diptera	Ceratopogonidae	Culicoides	Culicoides imicola	SPECIES	8.24500	34.58800	Ref.11
264	Diptera	Ceratopogonidae	Culicoides	Culicoides imicola	SPECIES	8.70800	40.41300	Ref.11
265	Diptera	Ceratopogonidae	Culicoides	Culicoides imicola	SPECIES	8.80000	40.70000	Ref.11
266	Diptera	Ceratopogonidae	Culicoides	Culicoides imicola	SPECIES	8.85600	40.72100	Ref.11
267	Diptera	Ceratopogonidae	Culicoides	Culicoides imicola	SPECIES	8.91200	39.98300	Ref.11
268	Diptera	Ceratopogonidae	Culicoides	Culicoides imicola	SPECIES	9.06300	18.42800	Ref.11
269	Diptera	Ceratopogonidae	Culicoides	Culicoides imicola	SPECIES	9.30000	13.50000	Ref.11
270	Diptera	Ceratopogonidae	Culicoides	Culicoides imicola	SPECIES	9.66700	8.73300	Ref.11
271	Diptera	Ceratopogonidae	Culicoides	Culicoides imicola	SPECIES	9.96500	40.53700	Ref.11
272	Diptera	Ceratopogonidae	Culicoides	Culicoides imicola	SPECIES	10.51700	7.43300	Ref.11
273	Diptera	Ceratopogonidae	Culicoides	Culicoides imicola	SPECIES	11.00000	-6.60000	Ref.11
274	Diptera	Ceratopogonidae	Culicoides	Culicoides imicola	SPECIES	11.01700	-4.03300	Ref.11
275	Diptera	Ceratopogonidae	Culicoides	Culicoides imicola	SPECIES	11.20000	-4.30000	Ref.11
276	Diptera	Ceratopogonidae	Culicoides	Culicoides imicola	SPECIES	11.56300	108.99500	Ref.11
277	Diptera	Ceratopogonidae	Culicoides	Culicoides imicola	SPECIES	11.56900	41.28300	Ref.11
278	Diptera	Ceratopogonidae	Culicoides	Culicoides imicola	SPECIES	11.90000	3.40000	Ref.11
279	Diptera	Ceratopogonidae	Culicoides	Culicoides imicola	SPECIES	12.01700	24.83300	Ref.11
280	Diptera	Ceratopogonidae	Culicoides	Culicoides imicola	SPECIES	12.50000	-16.65000	Ref.11
281	Diptera	Ceratopogonidae	Culicoides	Culicoides imicola	SPECIES	12.50000	-12.37000	Ref.11
282	Diptera	Ceratopogonidae	Culicoides	Culicoides imicola	SPECIES	12.52000	-12.45000	Ref.11
283	Diptera	Ceratopogonidae	Culicoides	Culicoides imicola	SPECIES	12.53000	-16.57000	Ref.11
284	Diptera	Ceratopogonidae	Culicoides	Culicoides imicola	SPECIES	12.53000	-12.30000	Ref.11
285	Diptera	Ceratopogonidae	Culicoides	Culicoides imicola	SPECIES	12.54300	7.82700	Ref.11
286	Diptera	Ceratopogonidae	Culicoides	Culicoides imicola	SPECIES	12.55000	-13.57000	Ref.11
287	Diptera	Ceratopogonidae	Culicoides	Culicoides imicola	SPECIES	12.60000	-12.20000	Ref.11

№	Order	Family	Genus	Species	Taxon Rank	Latitude	Longitude	GBIF_ID
288	Diptera	Ceratopogonidae	Culicoides	Culicoides imicola	SPECIES	12.63000	-12.45000	Ref.11
289	Diptera	Ceratopogonidae	Culicoides	Culicoides imicola	SPECIES	12.75000	-16.35000	Ref.11
290	Diptera	Ceratopogonidae	Culicoides	Culicoides imicola	SPECIES	12.81700	-14.86700	Ref.11
291	Diptera	Ceratopogonidae	Culicoides	Culicoides imicola	SPECIES	13.06700	-13.30000	Ref.11
292	Diptera	Ceratopogonidae	Culicoides	Culicoides imicola	SPECIES	13.06700	33.95000	Ref.11
293	Diptera	Ceratopogonidae	Culicoides	Culicoides imicola	SPECIES	13.22200	101.00300	Ref.11
294	Diptera	Ceratopogonidae	Culicoides	Culicoides imicola	SPECIES	13.24500	-16.63200	Ref.11
295	Diptera	Ceratopogonidae	Culicoides	Culicoides imicola	SPECIES	13.33200	-16.01000	Ref.11
296	Diptera	Ceratopogonidae	Culicoides	Culicoides imicola	SPECIES	13.40700	-16.70800	Ref.11
297	Diptera	Ceratopogonidae	Culicoides	Culicoides imicola	SPECIES	13.59500	100.72100	Ref.11
298	Diptera	Ceratopogonidae	Culicoides	Culicoides imicola	SPECIES	13.63300	-16.10000	Ref.11
299	Diptera	Ceratopogonidae	Culicoides	Culicoides imicola	SPECIES	13.68000	-16.38000	Ref.11
300	Diptera	Ceratopogonidae	Culicoides	Culicoides imicola	SPECIES	13.73000	-16.47000	Ref.11
301	Diptera	Ceratopogonidae	Culicoides	Culicoides imicola	SPECIES	13.78000	-16.47000	Ref.11
302	Diptera	Ceratopogonidae	Culicoides	Culicoides imicola	SPECIES	13.80600	100.75400	Ref.11
303	Diptera	Ceratopogonidae	Culicoides	Culicoides imicola	SPECIES	13.89500	100.41200	Ref.11
304	Diptera	Ceratopogonidae	Culicoides	Culicoides imicola	SPECIES	13.93400	100.09400	Ref.11
305	Diptera	Ceratopogonidae	Culicoides	Culicoides imicola	SPECIES	14.55000	-17.01700	Ref.11
306	Diptera	Ceratopogonidae	Culicoides	Culicoides imicola	SPECIES	14.56000	80.11000	Ref.11
307	Diptera	Ceratopogonidae	Culicoides	Culicoides imicola	SPECIES	14.60400	100.99300	Ref.11
308	Diptera	Ceratopogonidae	Culicoides	Culicoides imicola	SPECIES	14.71000	17.27000	Ref.11
309	Diptera	Ceratopogonidae	Culicoides	Culicoides imicola	SPECIES	14.73000	-17.33000	Ref.11
310	Diptera	Ceratopogonidae	Culicoides	Culicoides imicola	SPECIES	14.73000	17.33000	Ref.11
311	Diptera	Ceratopogonidae	Culicoides	Culicoides imicola	SPECIES	14.73000	-17.43000	Ref.11
312	Diptera	Ceratopogonidae	Culicoides	Culicoides imicola	SPECIES	14.76700	44.38300	Ref.11
313	Diptera	Ceratopogonidae	Culicoides	Culicoides imicola	SPECIES	14.77000	-17.04000	Ref.11
314	Diptera	Ceratopogonidae	Culicoides	Culicoides imicola	SPECIES	14.78300	-17.23300	Ref.11
315	Diptera	Ceratopogonidae	Culicoides	Culicoides imicola	SPECIES	14.79000	-16.95000	Ref.11
316	Diptera	Ceratopogonidae	Culicoides	Culicoides imicola	SPECIES	14.80000	43.20000	Ref.11
317	Diptera	Ceratopogonidae	Culicoides	Culicoides imicola	SPECIES	14.97500	102.09600	Ref.11
318	Diptera	Ceratopogonidae	Culicoides	Culicoides imicola	SPECIES	15.05500	-23.60500	Ref.11
319	Diptera	Ceratopogonidae	Culicoides	Culicoides imicola	SPECIES	15.38300	44.20000	Ref.11
320	Diptera	Ceratopogonidae	Culicoides	Culicoides imicola	SPECIES	15.65800	32.53000	Ref.11
321	Diptera	Ceratopogonidae	Culicoides	Culicoides imicola	SPECIES	15.83300	43.93300	Ref.11
322	Diptera	Ceratopogonidae	Culicoides	Culicoides imicola	SPECIES	16.45600	102.80400	Ref.11
323	Diptera	Ceratopogonidae	Culicoides	Culicoides imicola	SPECIES	16.88000	42.57000	Ref.11
324	Diptera	Ceratopogonidae	Culicoides	Culicoides imicola	SPECIES	17.02000	54.09000	Ref.11
325	Diptera	Ceratopogonidae	Culicoides	Culicoides imicola	SPECIES	17.16100	104.10000	Ref.11
326	Diptera	Ceratopogonidae	Culicoides	Culicoides imicola	SPECIES	17.32600	103.11900	Ref.11
327	Diptera	Ceratopogonidae	Culicoides	Culicoides imicola	SPECIES	17.40900	102.79400	Ref.11
328	Diptera	Ceratopogonidae	Culicoides	Culicoides imicola	SPECIES	17.49000	44.13000	Ref.11

№	Order	Family	Genus	Species	Taxon Rank	Latitude	Longitude	GBIF_ID
329	Diptera	Ceratopogonidae	Culicoides	Culicoides imicola	SPECIES	17.55300	101.71900	Ref.11
330	Diptera	Ceratopogonidae	Culicoides	Culicoides imicola	SPECIES	17.62300	101.44100	Ref.11
331	Diptera	Ceratopogonidae	Culicoides	Culicoides imicola	SPECIES	17.65400	102.93400	Ref.11
332	Diptera	Ceratopogonidae	Culicoides	Culicoides imicola	SPECIES	17.79400	102.57000	Ref.11
333	Diptera	Ceratopogonidae	Culicoides	Culicoides imicola	SPECIES	17.85000	102.80800	Ref.11
334	Diptera	Ceratopogonidae	Culicoides	Culicoides imicola	SPECIES	18.25000	42.50000	Ref.11
335	Diptera	Ceratopogonidae	Culicoides	Culicoides imicola	SPECIES	18.30000	32.90000	Ref.11
336	Diptera	Ceratopogonidae	Culicoides	Culicoides imicola	SPECIES	18.82400	98.71600	Ref.11
337	Diptera	Ceratopogonidae	Culicoides	Culicoides imicola	SPECIES	19.00000	30.00000	Ref.11
338	Diptera	Ceratopogonidae	Culicoides	Culicoides imicola	SPECIES	19.03300	109.83800	Ref.11
339	Diptera	Ceratopogonidae	Culicoides	Culicoides imicola	SPECIES	19.51000	109.57000	Ref.11
340	Diptera	Ceratopogonidae	Culicoides	Culicoides imicola	SPECIES	19.95000	56.28300	Ref.11
341	Diptera	Ceratopogonidae	Culicoides	Culicoides imicola	SPECIES	19.99000	25.62000	Ref.11
342	Diptera	Ceratopogonidae	Culicoides	Culicoides imicola	SPECIES	20.17000	28.76000	Ref.11
343	Diptera	Ceratopogonidae	Culicoides	Culicoides imicola	SPECIES	20.27000	32.62000	Ref.11
344	Diptera	Ceratopogonidae	Culicoides	Culicoides imicola	SPECIES	21.27000	40.42000	Ref.11
345	Diptera	Ceratopogonidae	Culicoides	Culicoides imicola	SPECIES	21.43000	39.82000	Ref.11
346	Diptera	Ceratopogonidae	Culicoides	Culicoides imicola	SPECIES	21.54000	39.17000	Ref.11
347	Diptera	Ceratopogonidae	Culicoides	Culicoides imicola	SPECIES	22.46000	88.31300	Ref.11
348	Diptera	Ceratopogonidae	Culicoides	Culicoides imicola	SPECIES	22.57300	88.36300	Ref.11
349	Diptera	Ceratopogonidae	Culicoides	Culicoides imicola	SPECIES	22.63000	88.42000	Ref.11
350	Diptera	Ceratopogonidae	Culicoides	Culicoides imicola	SPECIES	23.38000	57.83000	Ref.11
351	Diptera	Ceratopogonidae	Culicoides	Culicoides imicola	SPECIES	23.70000	58.00000	Ref.11
352	Diptera	Ceratopogonidae	Culicoides	Culicoides imicola	SPECIES	24.13300	47.31700	Ref.11
353	Diptera	Ceratopogonidae	Culicoides	Culicoides imicola	SPECIES	24.69000	46.72000	Ref.11
354	Diptera	Ceratopogonidae	Culicoides	Culicoides imicola	SPECIES	25.21700	55.28300	Ref.11
355	Diptera	Ceratopogonidae	Culicoides	Culicoides imicola	SPECIES	25.40000	49.60000	Ref.11
356	Diptera	Ceratopogonidae	Culicoides	Culicoides imicola	SPECIES	25.83000	42.86000	Ref.11
357	Diptera	Ceratopogonidae	Culicoides	Culicoides imicola	SPECIES	26.11700	50.55000	Ref.11
358	Diptera	Ceratopogonidae	Culicoides	Culicoides imicola	SPECIES	26.40000	50.15000	Ref.11
359	Diptera	Ceratopogonidae	Culicoides	Culicoides imicola	SPECIES	27.50000	41.70000	Ref.11
360	Diptera	Ceratopogonidae	Culicoides	Culicoides imicola	SPECIES	28.96700	-10.05000	Ref.11
361	Diptera	Ceratopogonidae	Culicoides	Culicoides imicola	SPECIES	28.99500	-7.14600	Ref.11
362	Diptera	Ceratopogonidae	Culicoides	Culicoides imicola	SPECIES	29.17400	-9.71100	Ref.11
363	Diptera	Ceratopogonidae	Culicoides	Culicoides imicola	SPECIES	29.88300	35.05000	Ref.11
364	Diptera	Ceratopogonidae	Culicoides	Culicoides imicola	SPECIES	29.98300	32.52500	Ref.11
365	Diptera	Ceratopogonidae	Culicoides	Culicoides imicola	SPECIES	30.00000	30.34300	Ref.11
366	Diptera	Ceratopogonidae	Culicoides	Culicoides imicola	SPECIES	30.07700	31.20800	Ref.11
367	Diptera	Ceratopogonidae	Culicoides	Culicoides imicola	SPECIES	30.36600	-9.56700	Ref.11
368	Diptera	Ceratopogonidae	Culicoides	Culicoides imicola	SPECIES	30.49400	-8.85700	Ref.11
369	Diptera	Ceratopogonidae	Culicoides	Culicoides imicola	SPECIES	30.59000	32.26700	Ref.11

№	Order	Family	Genus	Species	Taxon Rank	Latitude	Longitude	GBIF_ID
370	Diptera	Ceratopogonidae	Culicoides	Culicoides imicola	SPECIES	30.96700	-6.78300	Ref.11
371	Diptera	Ceratopogonidae	Culicoides	Culicoides imicola	SPECIES	31.33300	34.63300	Ref.11
372	Diptera	Ceratopogonidae	Culicoides	Culicoides imicola	SPECIES	31.56000	48.18300	Ref.11
373	Diptera	Ceratopogonidae	Culicoides	Culicoides imicola	SPECIES	31.60000	34.55000	Ref.11
374	Diptera	Ceratopogonidae	Culicoides	Culicoides imicola	SPECIES	31.61900	-8.02000	Ref.11
375	Diptera	Ceratopogonidae	Culicoides	Culicoides imicola	SPECIES	31.62700	-8.25700	Ref.11
376	Diptera	Ceratopogonidae	Culicoides	Culicoides imicola	SPECIES	31.76700	35.16700	Ref.11
377	Diptera	Ceratopogonidae	Culicoides	Culicoides imicola	SPECIES	31.80200	34.75200	Ref.11
378	Diptera	Ceratopogonidae	Culicoides	Culicoides imicola	SPECIES	31.81700	34.86700	Ref.11
379	Diptera	Ceratopogonidae	Culicoides	Culicoides imicola	SPECIES	31.81700	35.11700	Ref.11
380	Diptera	Ceratopogonidae	Culicoides	Culicoides imicola	SPECIES	31.93300	34.93300	Ref.11
381	Diptera	Ceratopogonidae	Culicoides	Culicoides imicola	SPECIES	31.97000	-4.47000	Ref.11
382	Diptera	Ceratopogonidae	Culicoides	Culicoides imicola	SPECIES	31.98300	34.81700	Ref.11
383	Diptera	Ceratopogonidae	Culicoides	Culicoides imicola	SPECIES	32.03300	34.83300	Ref.11
384	Diptera	Ceratopogonidae	Culicoides	Culicoides imicola	SPECIES	32.10000	34.93300	Ref.11
385	Diptera	Ceratopogonidae	Culicoides	Culicoides imicola	SPECIES	32.12800	0.56000	Ref.11
386	Diptera	Ceratopogonidae	Culicoides	Culicoides imicola	SPECIES	32.16700	34.90000	Ref.11
387	Diptera	Ceratopogonidae	Culicoides	Culicoides imicola	SPECIES	32.26500	-8.52700	Ref.11
388	Diptera	Ceratopogonidae	Culicoides	Culicoides imicola	SPECIES	32.31100	-6.39300	Ref.11
389	Diptera	Ceratopogonidae	Culicoides	Culicoides imicola	SPECIES	32.35000	34.90000	Ref.11
390	Diptera	Ceratopogonidae	Culicoides	Culicoides imicola	SPECIES	32.40000	34.93300	Ref.11
391	Diptera	Ceratopogonidae	Culicoides	Culicoides imicola	SPECIES	32.51700	35.43300	Ref.11
392	Diptera	Ceratopogonidae	Culicoides	Culicoides imicola	SPECIES	32.53300	35.26700	Ref.11
393	Diptera	Ceratopogonidae	Culicoides	Culicoides imicola	SPECIES	32.56700	-6.26700	Ref.11
394	Diptera	Ceratopogonidae	Culicoides	Culicoides imicola	SPECIES	32.66700	35.21700	Ref.11
395	Diptera	Ceratopogonidae	Culicoides	Culicoides imicola	SPECIES	32.70600	35.17900	Ref.11
396	Diptera	Ceratopogonidae	Culicoides	Culicoides imicola	SPECIES	32.96700	35.36700	Ref.11
397	Diptera	Ceratopogonidae	Culicoides	Culicoides imicola	SPECIES	32.98300	35.10000	Ref.11
398	Diptera	Ceratopogonidae	Culicoides	Culicoides imicola	SPECIES	33.16700	35.70000	Ref.11
399	Diptera	Ceratopogonidae	Culicoides	Culicoides imicola	SPECIES	33.31500	44.39200	Ref.11
400	Diptera	Ceratopogonidae	Culicoides	Culicoides imicola	SPECIES	33.39600	-5.55000	Ref.11
401	Diptera	Ceratopogonidae	Culicoides	Culicoides imicola	SPECIES	33.60000	-7.11700	Ref.11
402	Diptera	Ceratopogonidae	Culicoides	Culicoides imicola	SPECIES	33.73000	10.24000	Ref.11
403	Diptera	Ceratopogonidae	Culicoides	Culicoides imicola	SPECIES	34.03300	-6.74300	Ref.11
404	Diptera	Ceratopogonidae	Culicoides	Culicoides imicola	SPECIES	34.03300	-6.51700	Ref.11
405	Diptera	Ceratopogonidae	Culicoides	Culicoides imicola	SPECIES	34.10700	-5.95400	Ref.11
406	Diptera	Ceratopogonidae	Culicoides	Culicoides imicola	SPECIES	34.25000	-4.77800	Ref.11
407	Diptera	Ceratopogonidae	Culicoides	Culicoides imicola	SPECIES	34.25100	-3.34800	Ref.11
408	Diptera	Ceratopogonidae	Culicoides	Culicoides imicola	SPECIES	34.40000	-6.40000	Ref.11
409	Diptera	Ceratopogonidae	Culicoides	Culicoides imicola	SPECIES	34.41700	8.65000	Ref.11
410	Diptera	Ceratopogonidae	Culicoides	Culicoides imicola	SPECIES	34.55000	9.20000	Ref.11

№	Order	Family	Genus	Species	Taxon Rank	Latitude	Longitude	GBIF_ID
411	Diptera	Ceratopogonidae	Culicoides	Culicoides imicola	SPECIES	34.68000	10.64000	Ref.11
412	Diptera	Ceratopogonidae	Culicoides	Culicoides imicola	SPECIES	34.72400	-1.91400	Ref.11
413	Diptera	Ceratopogonidae	Culicoides	Culicoides imicola	SPECIES	34.79800	-1.43300	Ref.11
414	Diptera	Ceratopogonidae	Culicoides	Culicoides imicola	SPECIES	34.83300	-6.21700	Ref.11
415	Diptera	Ceratopogonidae	Culicoides	Culicoides imicola	SPECIES	34.91700	-5.91900	Ref.11
416	Diptera	Ceratopogonidae	Culicoides	Culicoides imicola	SPECIES	34.92200	-2.34500	Ref.11
417	Diptera	Ceratopogonidae	Culicoides	Culicoides imicola	SPECIES	35.03000	9.45000	Ref.11
418	Diptera	Ceratopogonidae	Culicoides	Culicoides imicola	SPECIES	35.15100	-3.84200	Ref.11
419	Diptera	Ceratopogonidae	Culicoides	Culicoides imicola	SPECIES	35.17600	-5.31100	Ref.11
420	Diptera	Ceratopogonidae	Culicoides	Culicoides imicola	SPECIES	35.18000	8.84000	Ref.11
421	Diptera	Ceratopogonidae	Culicoides	Culicoides imicola	SPECIES	35.21700	-5.93300	Ref.11
422	Diptera	Ceratopogonidae	Culicoides	Culicoides imicola	SPECIES	35.42800	3.57300	Ref.11
423	Diptera	Ceratopogonidae	Culicoides	Culicoides imicola	SPECIES	35.43300	-5.38300	Ref.11
424	Diptera	Ceratopogonidae	Culicoides	Culicoides imicola	SPECIES	35.52000	11.04000	Ref.11
425	Diptera	Ceratopogonidae	Culicoides	Culicoides imicola	SPECIES	35.54200	10.57100	Ref.11
426	Diptera	Ceratopogonidae	Culicoides	Culicoides imicola	SPECIES	35.57000	10.82000	Ref.11
427	Diptera	Ceratopogonidae	Culicoides	Culicoides imicola	SPECIES	35.61700	11.00000	Ref.11
428	Diptera	Ceratopogonidae	Culicoides	Culicoides imicola	SPECIES	35.63000	10.83000	Ref.11
429	Diptera	Ceratopogonidae	Culicoides	Culicoides imicola	SPECIES	35.70200	-5.35500	Ref.11
430	Diptera	Ceratopogonidae	Culicoides	Culicoides imicola	SPECIES	35.71700	10.67700	Ref.11
431	Diptera	Ceratopogonidae	Culicoides	Culicoides imicola	SPECIES	35.73000	10.83000	Ref.11
432	Diptera	Ceratopogonidae	Culicoides	Culicoides imicola	SPECIES	35.77900	-5.84200	Ref.11
433	Diptera	Ceratopogonidae	Culicoides	Culicoides imicola	SPECIES	35.78000	10.83000	Ref.11
434	Diptera	Ceratopogonidae	Culicoides	Culicoides imicola	SPECIES	35.90000	10.05000	Ref.11
435	Diptera	Ceratopogonidae	Culicoides	Culicoides imicola	SPECIES	35.90200	10.35000	Ref.11
436	Diptera	Ceratopogonidae	Culicoides	Culicoides imicola	SPECIES	35.94000	0.09000	Ref.11
437	Diptera	Ceratopogonidae	Culicoides	Culicoides imicola	SPECIES	36.00000	10.00000	Ref.11
438	Diptera	Ceratopogonidae	Culicoides	Culicoides imicola	SPECIES	36.02000	9.40000	Ref.11
439	Diptera	Ceratopogonidae	Culicoides	Culicoides imicola	SPECIES	36.06700	32.81700	Ref.11
440	Diptera	Ceratopogonidae	Culicoides	Culicoides imicola	SPECIES	36.08000	-5.63000	Ref.11
441	Diptera	Ceratopogonidae	Culicoides	Culicoides imicola	SPECIES	36.11000	8.93000	Ref.11
442	Diptera	Ceratopogonidae	Culicoides	Culicoides imicola	SPECIES	36.12000	10.38000	Ref.11
443	Diptera	Ceratopogonidae	Culicoides	Culicoides imicola	SPECIES	36.20000	-5.93300	Ref.11
444	Diptera	Ceratopogonidae	Culicoides	Culicoides imicola	SPECIES	36.21700	-5.38300	Ref.11
445	Diptera	Ceratopogonidae	Culicoides	Culicoides imicola	SPECIES	36.26700	28.03300	Ref.11
446	Diptera	Ceratopogonidae	Culicoides	Culicoides imicola	SPECIES	36.28300	-5.41700	Ref.11
447	Diptera	Ceratopogonidae	Culicoides	Culicoides imicola	SPECIES	36.32000	-5.45000	Ref.11
448	Diptera	Ceratopogonidae	Culicoides	Culicoides imicola	SPECIES	36.45000	-5.91700	Ref.11
449	Diptera	Ceratopogonidae	Culicoides	Culicoides imicola	SPECIES	36.45000	-5.72000	Ref.11
450	Diptera	Ceratopogonidae	Culicoides	Culicoides imicola	SPECIES	36.46300	4.53700	Ref.11
451	Diptera	Ceratopogonidae	Culicoides	Culicoides imicola	SPECIES	36.53000	9.10000	Ref.11

№	Order	Family	Genus	Species	Taxon Rank	Latitude	Longitude	GBIF_ID
452	Diptera	Ceratopogonidae	Culicoides	Culicoides imicola	SPECIES	36.55000	-5.28300	Ref.11
453	Diptera	Ceratopogonidae	Culicoides	Culicoides imicola	SPECIES	36.55000	-4.63300	Ref.11
454	Diptera	Ceratopogonidae	Culicoides	Culicoides imicola	SPECIES	36.56700	36.16700	Ref.11
455	Diptera	Ceratopogonidae	Culicoides	Culicoides imicola	SPECIES	36.57000	-4.89000	Ref.11
456	Diptera	Ceratopogonidae	Culicoides	Culicoides imicola	SPECIES	36.59600	-4.63700	Ref.11
457	Diptera	Ceratopogonidae	Culicoides	Culicoides imicola	SPECIES	36.63300	6.81700	Ref.11
458	Diptera	Ceratopogonidae	Culicoides	Culicoides imicola	SPECIES	36.65000	29.16700	Ref.11
459	Diptera	Ceratopogonidae	Culicoides	Culicoides imicola	SPECIES	36.66000	-4.75000	Ref.11
460	Diptera	Ceratopogonidae	Culicoides	Culicoides imicola	SPECIES	36.66700	-4.56700	Ref.11
461	Diptera	Ceratopogonidae	Culicoides	Culicoides imicola	SPECIES	36.68000	15.08000	Ref.11
462	Diptera	Ceratopogonidae	Culicoides	Culicoides imicola	SPECIES	36.68300	5.78300	Ref.11
463	Diptera	Ceratopogonidae	Culicoides	Culicoides imicola	SPECIES	36.69000	9.88000	Ref.11
464	Diptera	Ceratopogonidae	Culicoides	Culicoides imicola	SPECIES	36.71700	-6.05000	Ref.11
465	Diptera	Ceratopogonidae	Culicoides	Culicoides imicola	SPECIES	36.71700	-4.25000	Ref.11
466	Diptera	Ceratopogonidae	Culicoides	Culicoides imicola	SPECIES	36.72000	14.78000	Ref.11
467	Diptera	Ceratopogonidae	Culicoides	Culicoides imicola	SPECIES	36.73000	-4.11000	Ref.11
468	Diptera	Ceratopogonidae	Culicoides	Culicoides imicola	SPECIES	36.73000	15.11000	Ref.11
469	Diptera	Ceratopogonidae	Culicoides	Culicoides imicola	SPECIES	36.73300	7.10000	Ref.11
470	Diptera	Ceratopogonidae	Culicoides	Culicoides imicola	SPECIES	36.73300	3.95000	Ref.11
471	Diptera	Ceratopogonidae	Culicoides	Culicoides imicola	SPECIES	36.73300	-5.15000	Ref.11
472	Diptera	Ceratopogonidae	Culicoides	Culicoides imicola	SPECIES	36.75000	-3.52000	Ref.11
473	Diptera	Ceratopogonidae	Culicoides	Culicoides imicola	SPECIES	36.75000	14.72000	Ref.11
474	Diptera	Ceratopogonidae	Culicoides	Culicoides imicola	SPECIES	36.75000	14.89000	Ref.11
475	Diptera	Ceratopogonidae	Culicoides	Culicoides imicola	SPECIES	36.75000	14.98000	Ref.11
476	Diptera	Ceratopogonidae	Culicoides	Culicoides imicola	SPECIES	36.75000	4.30000	Ref.11
477	Diptera	Ceratopogonidae	Culicoides	Culicoides imicola	SPECIES	36.75000	28.88300	Ref.11
478	Diptera	Ceratopogonidae	Culicoides	Culicoides imicola	SPECIES	36.75000	-4.66700	Ref.11
479	Diptera	Ceratopogonidae	Culicoides	Culicoides imicola	SPECIES	36.76000	-2.98000	Ref.11
480	Diptera	Ceratopogonidae	Culicoides	Culicoides imicola	SPECIES	36.76700	5.78300	Ref.11
481	Diptera	Ceratopogonidae	Culicoides	Culicoides imicola	SPECIES	36.78000	-5.36000	Ref.11
482	Diptera	Ceratopogonidae	Culicoides	Culicoides imicola	SPECIES	36.78000	14.62000	Ref.11
483	Diptera	Ceratopogonidae	Culicoides	Culicoides imicola	SPECIES	36.78300	-4.10000	Ref.11
484	Diptera	Ceratopogonidae	Culicoides	Culicoides imicola	SPECIES	36.78300	-6.28300	Ref.11
485	Diptera	Ceratopogonidae	Culicoides	Culicoides imicola	SPECIES	36.79000	14.75000	Ref.11
486	Diptera	Ceratopogonidae	Culicoides	Culicoides imicola	SPECIES	36.80000	14.97000	Ref.11
487	Diptera	Ceratopogonidae	Culicoides	Culicoides imicola	SPECIES	36.80000	15.08000	Ref.11
488	Diptera	Ceratopogonidae	Culicoides	Culicoides imicola	SPECIES	36.80000	34.61700	Ref.11
489	Diptera	Ceratopogonidae	Culicoides	Culicoides imicola	SPECIES	36.80000	8.50000	Ref.11
490	Diptera	Ceratopogonidae	Culicoides	Culicoides imicola	SPECIES	36.81000	14.82000	Ref.11
491	Diptera	Ceratopogonidae	Culicoides	Culicoides imicola	SPECIES	36.81700	-4.75000	Ref.11
492	Diptera	Ceratopogonidae	Culicoides	Culicoides imicola	SPECIES	36.82200	10.17200	Ref.11

№	Order	Family	Genus	Species	Taxon Rank	Latitude	Longitude	GBIF_ID
493	Diptera	Ceratopogonidae	Culicoides	Culicoides imicola	SPECIES	36.83000	14.64000	Ref.11
494	Diptera	Ceratopogonidae	Culicoides	Culicoides imicola	SPECIES	36.85000	-5.02000	Ref.11
495	Diptera	Ceratopogonidae	Culicoides	Culicoides imicola	SPECIES	36.85000	14.89000	Ref.11
496	Diptera	Ceratopogonidae	Culicoides	Culicoides imicola	SPECIES	36.85000	14.98000	Ref.11
497	Diptera	Ceratopogonidae	Culicoides	Culicoides imicola	SPECIES	36.85000	27.23300	Ref.11
498	Diptera	Ceratopogonidae	Culicoides	Culicoides imicola	SPECIES	36.86000	-2.14000	Ref.11
499	Diptera	Ceratopogonidae	Culicoides	Culicoides imicola	SPECIES	36.86000	15.04000	Ref.11
500	Diptera	Ceratopogonidae	Culicoides	Culicoides imicola	SPECIES	36.86700	-6.18300	Ref.11
501	Diptera	Ceratopogonidae	Culicoides	Culicoides imicola	SPECIES	36.86700	-3.71700	Ref.11
502	Diptera	Ceratopogonidae	Culicoides	Culicoides imicola	SPECIES	36.87000	14.55000	Ref.11
503	Diptera	Ceratopogonidae	Culicoides	Culicoides imicola	SPECIES	36.87000	10.74000	Ref.11
504	Diptera	Ceratopogonidae	Culicoides	Culicoides imicola	SPECIES	36.88000	14.66000	Ref.11
505	Diptera	Ceratopogonidae	Culicoides	Culicoides imicola	SPECIES	36.88300	8.53300	Ref.11
506	Diptera	Ceratopogonidae	Culicoides	Culicoides imicola	SPECIES	36.88300	-4.45000	Ref.11
507	Diptera	Ceratopogonidae	Culicoides	Culicoides imicola	SPECIES	36.88300	-5.41700	Ref.11
508	Diptera	Ceratopogonidae	Culicoides	Culicoides imicola	SPECIES	36.89000	14.72000	Ref.11
509	Diptera	Ceratopogonidae	Culicoides	Culicoides imicola	SPECIES	36.90000	14.78000	Ref.11
510	Diptera	Ceratopogonidae	Culicoides	Culicoides imicola	SPECIES	36.90000	14.84000	Ref.11
511	Diptera	Ceratopogonidae	Culicoides	Culicoides imicola	SPECIES	36.90000	15.00000	Ref.11
512	Diptera	Ceratopogonidae	Culicoides	Culicoides imicola	SPECIES	36.90000	30.68300	Ref.11
513	Diptera	Ceratopogonidae	Culicoides	Culicoides imicola	SPECIES	36.90000	-3.47000	Ref.11
514	Diptera	Ceratopogonidae	Culicoides	Culicoides imicola	SPECIES	36.91000	14.60000	Ref.11
515	Diptera	Ceratopogonidae	Culicoides	Culicoides imicola	SPECIES	36.91300	10.03700	Ref.11
516	Diptera	Ceratopogonidae	Culicoides	Culicoides imicola	SPECIES	36.91700	34.88300	Ref.11
517	Diptera	Ceratopogonidae	Culicoides	Culicoides imicola	SPECIES	36.91700	-5.25000	Ref.11
518	Diptera	Ceratopogonidae	Culicoides	Culicoides imicola	SPECIES	36.95000	14.75000	Ref.11
519	Diptera	Ceratopogonidae	Culicoides	Culicoides imicola	SPECIES	36.95000	14.81000	Ref.11
520	Diptera	Ceratopogonidae	Culicoides	Culicoides imicola	SPECIES	36.95000	15.04000	Ref.11
521	Diptera	Ceratopogonidae	Culicoides	Culicoides imicola	SPECIES	36.95000	-4.86700	Ref.11
522	Diptera	Ceratopogonidae	Culicoides	Culicoides imicola	SPECIES	36.96700	35.36700	Ref.11
523	Diptera	Ceratopogonidae	Culicoides	Culicoides imicola	SPECIES	36.96700	28.28300	Ref.11
524	Diptera	Ceratopogonidae	Culicoides	Culicoides imicola	SPECIES	36.97000	14.67000	Ref.11
525	Diptera	Ceratopogonidae	Culicoides	Culicoides imicola	SPECIES	36.97000	14.47000	Ref.11
526	Diptera	Ceratopogonidae	Culicoides	Culicoides imicola	SPECIES	36.97000	14.96000	Ref.11
527	Diptera	Ceratopogonidae	Culicoides	Culicoides imicola	SPECIES	36.97000	15.18000	Ref.11
528	Diptera	Ceratopogonidae	Culicoides	Culicoides imicola	SPECIES	36.98000	14.54000	Ref.11
529	Diptera	Ceratopogonidae	Culicoides	Culicoides imicola	SPECIES	36.99000	14.87000	Ref.11
530	Diptera	Ceratopogonidae	Culicoides	Culicoides imicola	SPECIES	37.01700	28.45000	Ref.11
531	Diptera	Ceratopogonidae	Culicoides	Culicoides imicola	SPECIES	37.02000	15.31000	Ref.11
532	Diptera	Ceratopogonidae	Culicoides	Culicoides imicola	SPECIES	37.03000	14.99000	Ref.11
533	Diptera	Ceratopogonidae	Culicoides	Culicoides imicola	SPECIES	37.03000	15.23000	Ref.11

№	Order	Family	Genus	Species	Taxon Rank	Latitude	Longitude	GBIF_ID
534	Diptera	Ceratopogonidae	Culicoides	Culicoides imicola	SPECIES	37.04000	14.54000	Ref.11
535	Diptera	Ceratopogonidae	Culicoides	Culicoides imicola	SPECIES	37.05000	14.78000	Ref.11
536	Diptera	Ceratopogonidae	Culicoides	Culicoides imicola	SPECIES	37.05000	14.88000	Ref.11
537	Diptera	Ceratopogonidae	Culicoides	Culicoides imicola	SPECIES	37.05000	-4.56700	Ref.11
538	Diptera	Ceratopogonidae	Culicoides	Culicoides imicola	SPECIES	37.06000	15.12000	Ref.11
539	Diptera	Ceratopogonidae	Culicoides	Culicoides imicola	SPECIES	37.06000	9.61000	Ref.11
540	Diptera	Ceratopogonidae	Culicoides	Culicoides imicola	SPECIES	37.07000	14.70000	Ref.11
541	Diptera	Ceratopogonidae	Culicoides	Culicoides imicola	SPECIES	37.07000	15.19000	Ref.11
542	Diptera	Ceratopogonidae	Culicoides	Culicoides imicola	SPECIES	37.09000	15.27000	Ref.11
543	Diptera	Ceratopogonidae	Culicoides	Culicoides imicola	SPECIES	37.10000	14.79000	Ref.11
544	Diptera	Ceratopogonidae	Culicoides	Culicoides imicola	SPECIES	37.10000	14.96000	Ref.11
545	Diptera	Ceratopogonidae	Culicoides	Culicoides imicola	SPECIES	37.10000	14.18000	Ref.11
546	Diptera	Ceratopogonidae	Culicoides	Culicoides imicola	SPECIES	37.12000	15.13000	Ref.11
547	Diptera	Ceratopogonidae	Culicoides	Culicoides imicola	SPECIES	37.13000	14.27000	Ref.11
548	Diptera	Ceratopogonidae	Culicoides	Culicoides imicola	SPECIES	37.14000	14.63000	Ref.11
549	Diptera	Ceratopogonidae	Culicoides	Culicoides imicola	SPECIES	37.14000	14.53000	Ref.11
550	Diptera	Ceratopogonidae	Culicoides	Culicoides imicola	SPECIES	37.14000	13.90000	Ref.11
551	Diptera	Ceratopogonidae	Culicoides	Culicoides imicola	SPECIES	37.14000	-6.46000	Ref.11
552	Diptera	Ceratopogonidae	Culicoides	Culicoides imicola	SPECIES	37.15000	14.15000	Ref.11
553	Diptera	Ceratopogonidae	Culicoides	Culicoides imicola	SPECIES	37.15000	14.21000	Ref.11
554	Diptera	Ceratopogonidae	Culicoides	Culicoides imicola	SPECIES	37.15000	14.40000	Ref.11
555	Diptera	Ceratopogonidae	Culicoides	Culicoides imicola	SPECIES	37.16700	-5.73300	Ref.11
556	Diptera	Ceratopogonidae	Culicoides	Culicoides imicola	SPECIES	37.16900	-7.90300	Ref.11
557	Diptera	Ceratopogonidae	Culicoides	Culicoides imicola	SPECIES	37.17000	15.13000	Ref.11
558	Diptera	Ceratopogonidae	Culicoides	Culicoides imicola	SPECIES	37.17000	14.76000	Ref.11
559	Diptera	Ceratopogonidae	Culicoides	Culicoides imicola	SPECIES	37.17000	14.06000	Ref.11
560	Diptera	Ceratopogonidae	Culicoides	Culicoides imicola	SPECIES	37.18000	14.47000	Ref.11
561	Diptera	Ceratopogonidae	Culicoides	Culicoides imicola	SPECIES	37.19000	15.00000	Ref.11
562	Diptera	Ceratopogonidae	Culicoides	Culicoides imicola	SPECIES	37.20000	14.94000	Ref.11
563	Diptera	Ceratopogonidae	Culicoides	Culicoides imicola	SPECIES	37.20000	14.32000	Ref.11
564	Diptera	Ceratopogonidae	Culicoides	Culicoides imicola	SPECIES	37.20600	-8.44200	Ref.11
565	Diptera	Ceratopogonidae	Culicoides	Culicoides imicola	SPECIES	37.21000	14.82000	Ref.11
566	Diptera	Ceratopogonidae	Culicoides	Culicoides imicola	SPECIES	37.21000	13.89000	Ref.11
567	Diptera	Ceratopogonidae	Culicoides	Culicoides imicola	SPECIES	37.22000	14.76000	Ref.11
568	Diptera	Ceratopogonidae	Culicoides	Culicoides imicola	SPECIES	37.23000	-7.48000	Ref.11
569	Diptera	Ceratopogonidae	Culicoides	Culicoides imicola	SPECIES	37.23300	27.71700	Ref.11
570	Diptera	Ceratopogonidae	Culicoides	Culicoides imicola	SPECIES	37.23300	28.28300	Ref.11
571	Diptera	Ceratopogonidae	Culicoides	Culicoides imicola	SPECIES	37.24000	15.13000	Ref.11
572	Diptera	Ceratopogonidae	Culicoides	Culicoides imicola	SPECIES	37.24000	14.17000	Ref.11
573	Diptera	Ceratopogonidae	Culicoides	Culicoides imicola	SPECIES	37.25000	-6.91700	Ref.11
574	Diptera	Ceratopogonidae	Culicoides	Culicoides imicola	SPECIES	37.25000	-4.80000	Ref.11

№	Order	Family	Genus	Species	Taxon Rank	Latitude	Longitude	GBIF_ID
575	Diptera	Ceratopogonidae	Culicoides	Culicoides imicola	SPECIES	37.26000	14.52000	Ref.11
576	Diptera	Ceratopogonidae	Culicoides	Culicoides imicola	SPECIES	37.26000	14.81000	Ref.11
577	Diptera	Ceratopogonidae	Culicoides	Culicoides imicola	SPECIES	37.26000	-8.12000	Ref.11
578	Diptera	Ceratopogonidae	Culicoides	Culicoides imicola	SPECIES	37.27000	15.08000	Ref.11
579	Diptera	Ceratopogonidae	Culicoides	Culicoides imicola	SPECIES	37.27000	14.73000	Ref.11
580	Diptera	Ceratopogonidae	Culicoides	Culicoides imicola	SPECIES	37.27000	13.81000	Ref.11
581	Diptera	Ceratopogonidae	Culicoides	Culicoides imicola	SPECIES	37.28000	14.65000	Ref.11
582	Diptera	Ceratopogonidae	Culicoides	Culicoides imicola	SPECIES	37.28000	13.62000	Ref.11
583	Diptera	Ceratopogonidae	Culicoides	Culicoides imicola	SPECIES	37.28000	14.26000	Ref.11
584	Diptera	Ceratopogonidae	Culicoides	Culicoides imicola	SPECIES	37.29000	14.95000	Ref.11
585	Diptera	Ceratopogonidae	Culicoides	Culicoides imicola	SPECIES	37.30000	14.39000	Ref.11
586	Diptera	Ceratopogonidae	Culicoides	Culicoides imicola	SPECIES	37.30000	-5.16700	Ref.11
587	Diptera	Ceratopogonidae	Culicoides	Culicoides imicola	SPECIES	37.31000	13.53000	Ref.11
588	Diptera	Ceratopogonidae	Culicoides	Culicoides imicola	SPECIES	37.31000	14.19000	Ref.11
589	Diptera	Ceratopogonidae	Culicoides	Culicoides imicola	SPECIES	37.31700	-5.08300	Ref.11
590	Diptera	Ceratopogonidae	Culicoides	Culicoides imicola	SPECIES	37.32000	-4.49000	Ref.11
591	Diptera	Ceratopogonidae	Culicoides	Culicoides imicola	SPECIES	37.32000	14.13000	Ref.11
592	Diptera	Ceratopogonidae	Culicoides	Culicoides imicola	SPECIES	37.33000	14.77000	Ref.11
593	Diptera	Ceratopogonidae	Culicoides	Culicoides imicola	SPECIES	37.33000	13.77000	Ref.11
594	Diptera	Ceratopogonidae	Culicoides	Culicoides imicola	SPECIES	37.33300	-7.46700	Ref.11
595	Diptera	Ceratopogonidae	Culicoides	Culicoides imicola	SPECIES	37.33300	-5.83300	Ref.11
596	Diptera	Ceratopogonidae	Culicoides	Culicoides imicola	SPECIES	37.33300	-6.90000	Ref.11
597	Diptera	Ceratopogonidae	Culicoides	Culicoides imicola	SPECIES	37.34000	14.32000	Ref.11
598	Diptera	Ceratopogonidae	Culicoides	Culicoides imicola	SPECIES	37.34000	15.06000	Ref.11
599	Diptera	Ceratopogonidae	Culicoides	Culicoides imicola	SPECIES	37.34000	13.95000	Ref.11
600	Diptera	Ceratopogonidae	Culicoides	Culicoides imicola	SPECIES	37.35000	13.39000	Ref.11
601	Diptera	Ceratopogonidae	Culicoides	Culicoides imicola	SPECIES	37.35000	13.89000	Ref.11
602	Diptera	Ceratopogonidae	Culicoides	Culicoides imicola	SPECIES	37.36000	-3.88000	Ref.11
603	Diptera	Ceratopogonidae	Culicoides	Culicoides imicola	SPECIES	37.36000	14.93000	Ref.11
604	Diptera	Ceratopogonidae	Culicoides	Culicoides imicola	SPECIES	37.37000	14.18000	Ref.11
605	Diptera	Ceratopogonidae	Culicoides	Culicoides imicola	SPECIES	37.38000	14.35000	Ref.11
606	Diptera	Ceratopogonidae	Culicoides	Culicoides imicola	SPECIES	37.38000	14.28000	Ref.11
607	Diptera	Ceratopogonidae	Culicoides	Culicoides imicola	SPECIES	37.38000	13.64000	Ref.11
608	Diptera	Ceratopogonidae	Culicoides	Culicoides imicola	SPECIES	37.38300	-6.68300	Ref.11
609	Diptera	Ceratopogonidae	Culicoides	Culicoides imicola	SPECIES	37.38300	-5.33300	Ref.11
610	Diptera	Ceratopogonidae	Culicoides	Culicoides imicola	SPECIES	37.39000	13.51000	Ref.11
611	Diptera	Ceratopogonidae	Culicoides	Culicoides imicola	SPECIES	37.40000	-7.11000	Ref.11
612	Diptera	Ceratopogonidae	Culicoides	Culicoides imicola	SPECIES	37.40000	14.89000	Ref.11
613	Diptera	Ceratopogonidae	Culicoides	Culicoides imicola	SPECIES	37.40000	14.80000	Ref.11
614	Diptera	Ceratopogonidae	Culicoides	Culicoides imicola	SPECIES	37.40000	13.30000	Ref.11
615	Diptera	Ceratopogonidae	Culicoides	Culicoides imicola	SPECIES	37.40000	13.97000	Ref.11

№	Order	Family	Genus	Species	Taxon Rank	Latitude	Longitude	GBIF_ID
616	Diptera	Ceratopogonidae	Culicoides	Culicoides imicola	SPECIES	37.40000	-4.38300	Ref.11
617	Diptera	Ceratopogonidae	Culicoides	Culicoides imicola	SPECIES	37.41000	14.48000	Ref.11
618	Diptera	Ceratopogonidae	Culicoides	Culicoides imicola	SPECIES	37.41000	14.13000	Ref.11
619	Diptera	Ceratopogonidae	Culicoides	Culicoides imicola	SPECIES	37.41000	15.05000	Ref.11
620	Diptera	Ceratopogonidae	Culicoides	Culicoides imicola	SPECIES	37.41700	-6.00000	Ref.11
621	Diptera	Ceratopogonidae	Culicoides	Culicoides imicola	SPECIES	37.41700	-6.08300	Ref.11
622	Diptera	Ceratopogonidae	Culicoides	Culicoides imicola	SPECIES	37.42000	13.56000	Ref.11
623	Diptera	Ceratopogonidae	Culicoides	Culicoides imicola	SPECIES	37.43000	13.78000	Ref.11
624	Diptera	Ceratopogonidae	Culicoides	Culicoides imicola	SPECIES	37.44000	14.53000	Ref.11
625	Diptera	Ceratopogonidae	Culicoides	Culicoides imicola	SPECIES	37.44000	14.66000	Ref.11
626	Diptera	Ceratopogonidae	Culicoides	Culicoides imicola	SPECIES	37.44000	14.92000	Ref.11
627	Diptera	Ceratopogonidae	Culicoides	Culicoides imicola	SPECIES	37.44000	13.45000	Ref.11
628	Diptera	Ceratopogonidae	Culicoides	Culicoides imicola	SPECIES	37.45600	-7.50300	Ref.11
629	Diptera	Ceratopogonidae	Culicoides	Culicoides imicola	SPECIES	37.46000	14.43000	Ref.11
630	Diptera	Ceratopogonidae	Culicoides	Culicoides imicola	SPECIES	37.46000	15.05000	Ref.11
631	Diptera	Ceratopogonidae	Culicoides	Culicoides imicola	SPECIES	37.46000	13.61000	Ref.11
632	Diptera	Ceratopogonidae	Culicoides	Culicoides imicola	SPECIES	37.46000	13.89000	Ref.11
633	Diptera	Ceratopogonidae	Culicoides	Culicoides imicola	SPECIES	37.47000	14.35000	Ref.11
634	Diptera	Ceratopogonidae	Culicoides	Culicoides imicola	SPECIES	37.47000	14.27000	Ref.11
635	Diptera	Ceratopogonidae	Culicoides	Culicoides imicola	SPECIES	37.47000	14.12000	Ref.11
636	Diptera	Ceratopogonidae	Culicoides	Culicoides imicola	SPECIES	37.47000	14.98000	Ref.11
637	Diptera	Ceratopogonidae	Culicoides	Culicoides imicola	SPECIES	37.47000	13.72000	Ref.11
638	Diptera	Ceratopogonidae	Culicoides	Culicoides imicola	SPECIES	37.47000	14.03000	Ref.11
639	Diptera	Ceratopogonidae	Culicoides	Culicoides imicola	SPECIES	37.48000	14.63000	Ref.11
640	Diptera	Ceratopogonidae	Culicoides	Culicoides imicola	SPECIES	37.48900	-8.03600	Ref.11
641	Diptera	Ceratopogonidae	Culicoides	Culicoides imicola	SPECIES	37.49000	13.28000	Ref.11
642	Diptera	Ceratopogonidae	Culicoides	Culicoides imicola	SPECIES	37.50000	14.88000	Ref.11
643	Diptera	Ceratopogonidae	Culicoides	Culicoides imicola	SPECIES	37.50000	-5.10000	Ref.11
644	Diptera	Ceratopogonidae	Culicoides	Culicoides imicola	SPECIES	37.50000	-7.52000	Ref.11
645	Diptera	Ceratopogonidae	Culicoides	Culicoides imicola	SPECIES	37.51000	-8.72000	Ref.11
646	Diptera	Ceratopogonidae	Culicoides	Culicoides imicola	SPECIES	37.52000	14.42000	Ref.11
647	Diptera	Ceratopogonidae	Culicoides	Culicoides imicola	SPECIES	37.52000	14.74000	Ref.11
648	Diptera	Ceratopogonidae	Culicoides	Culicoides imicola	SPECIES	37.52000	13.41000	Ref.11
649	Diptera	Ceratopogonidae	Culicoides	Culicoides imicola	SPECIES	37.53000	14.27000	Ref.11
650	Diptera	Ceratopogonidae	Culicoides	Culicoides imicola	SPECIES	37.53000	14.59000	Ref.11
651	Diptera	Ceratopogonidae	Culicoides	Culicoides imicola	SPECIES	37.53000	14.95000	Ref.11
652	Diptera	Ceratopogonidae	Culicoides	Culicoides imicola	SPECIES	37.53000	15.02000	Ref.11
653	Diptera	Ceratopogonidae	Culicoides	Culicoides imicola	SPECIES	37.53300	-5.61700	Ref.11
654	Diptera	Ceratopogonidae	Culicoides	Culicoides imicola	SPECIES	37.54000	-7.35000	Ref.11
655	Diptera	Ceratopogonidae	Culicoides	Culicoides imicola	SPECIES	37.54000	14.18000	Ref.11
656	Diptera	Ceratopogonidae	Culicoides	Culicoides imicola	SPECIES	37.54000	14.35000	Ref.11

№	Order	Family	Genus	Species	Taxon Rank	Latitude	Longitude	GBIF_ID
657	Diptera	Ceratopogonidae	Culicoides	Culicoides imicola	SPECIES	37.54000	14.85000	Ref.11
658	Diptera	Ceratopogonidae	Culicoides	Culicoides imicola	SPECIES	37.54000	14.06000	Ref.11
659	Diptera	Ceratopogonidae	Culicoides	Culicoides imicola	SPECIES	37.55000	13.06000	Ref.11
660	Diptera	Ceratopogonidae	Culicoides	Culicoides imicola	SPECIES	37.55000	13.54000	Ref.11
661	Diptera	Ceratopogonidae	Culicoides	Culicoides imicola	SPECIES	37.55000	13.33000	Ref.11
662	Diptera	Ceratopogonidae	Culicoides	Culicoides imicola	SPECIES	37.55000	13.99000	Ref.11
663	Diptera	Ceratopogonidae	Culicoides	Culicoides imicola	SPECIES	37.56000	13.72000	Ref.11
664	Diptera	Ceratopogonidae	Culicoides	Culicoides imicola	SPECIES	37.56700	-5.40000	Ref.11
665	Diptera	Ceratopogonidae	Culicoides	Culicoides imicola	SPECIES	37.56700	-6.88300	Ref.11
666	Diptera	Ceratopogonidae	Culicoides	Culicoides imicola	SPECIES	37.57000	-5.11000	Ref.11
667	Diptera	Ceratopogonidae	Culicoides	Culicoides imicola	SPECIES	37.57000	14.72000	Ref.11
668	Diptera	Ceratopogonidae	Culicoides	Culicoides imicola	SPECIES	37.58000	-1.99000	Ref.11
669	Diptera	Ceratopogonidae	Culicoides	Culicoides imicola	SPECIES	37.58000	14.55000	Ref.11
670	Diptera	Ceratopogonidae	Culicoides	Culicoides imicola	SPECIES	37.58000	13.41000	Ref.11
671	Diptera	Ceratopogonidae	Culicoides	Culicoides imicola	SPECIES	37.58000	14.92000	Ref.11
672	Diptera	Ceratopogonidae	Culicoides	Culicoides imicola	SPECIES	37.58000	13.16000	Ref.11
673	Diptera	Ceratopogonidae	Culicoides	Culicoides imicola	SPECIES	37.59000	14.40000	Ref.11
674	Diptera	Ceratopogonidae	Culicoides	Culicoides imicola	SPECIES	37.59000	14.33000	Ref.11
675	Diptera	Ceratopogonidae	Culicoides	Culicoides imicola	SPECIES	37.59000	14.49000	Ref.11
676	Diptera	Ceratopogonidae	Culicoides	Culicoides imicola	SPECIES	37.59000	14.61000	Ref.11
677	Diptera	Ceratopogonidae	Culicoides	Culicoides imicola	SPECIES	37.59000	14.98000	Ref.11
678	Diptera	Ceratopogonidae	Culicoides	Culicoides imicola	SPECIES	37.60000	13.92000	Ref.11
679	Diptera	Ceratopogonidae	Culicoides	Culicoides imicola	SPECIES	37.60000	14.26000	Ref.11
680	Diptera	Ceratopogonidae	Culicoides	Culicoides imicola	SPECIES	37.60000	13.28000	Ref.11
681	Diptera	Ceratopogonidae	Culicoides	Culicoides imicola	SPECIES	37.60000	13.06000	Ref.11
682	Diptera	Ceratopogonidae	Culicoides	Culicoides imicola	SPECIES	37.61000	14.67000	Ref.11
683	Diptera	Ceratopogonidae	Culicoides	Culicoides imicola	SPECIES	37.61000	14.84000	Ref.11
684	Diptera	Ceratopogonidae	Culicoides	Culicoides imicola	SPECIES	37.61000	12.92000	Ref.11
685	Diptera	Ceratopogonidae	Culicoides	Culicoides imicola	SPECIES	37.61000	13.79000	Ref.11
686	Diptera	Ceratopogonidae	Culicoides	Culicoides imicola	SPECIES	37.61700	-5.65000	Ref.11
687	Diptera	Ceratopogonidae	Culicoides	Culicoides imicola	SPECIES	37.61700	-4.28300	Ref.11
688	Diptera	Ceratopogonidae	Culicoides	Culicoides imicola	SPECIES	37.62000	13.19000	Ref.11
689	Diptera	Ceratopogonidae	Culicoides	Culicoides imicola	SPECIES	37.62000	12.63000	Ref.11
690	Diptera	Ceratopogonidae	Culicoides	Culicoides imicola	SPECIES	37.62000	12.77000	Ref.11
691	Diptera	Ceratopogonidae	Culicoides	Culicoides imicola	SPECIES	37.62000	-7.53000	Ref.11
692	Diptera	Ceratopogonidae	Culicoides	Culicoides imicola	SPECIES	37.63000	13.42000	Ref.11
693	Diptera	Ceratopogonidae	Culicoides	Culicoides imicola	SPECIES	37.63000	-8.63000	Ref.11
694	Diptera	Ceratopogonidae	Culicoides	Culicoides imicola	SPECIES	37.63300	-5.55000	Ref.11
695	Diptera	Ceratopogonidae	Culicoides	Culicoides imicola	SPECIES	37.64000	13.25000	Ref.11
696	Diptera	Ceratopogonidae	Culicoides	Culicoides imicola	SPECIES	37.64000	13.69000	Ref.11
697	Diptera	Ceratopogonidae	Culicoides	Culicoides imicola	SPECIES	37.64000	12.83000	Ref.11

№	Order	Family	Genus	Species	Taxon Rank	Latitude	Longitude	GBIF_ID
698	Diptera	Ceratopogonidae	Culicoides	Culicoides imicola	SPECIES	37.65000	14.58000	Ref.11
699	Diptera	Ceratopogonidae	Culicoides	Culicoides imicola	SPECIES	37.65000	14.75000	Ref.11
700	Diptera	Ceratopogonidae	Culicoides	Culicoides imicola	SPECIES	37.65000	14.87000	Ref.11
701	Diptera	Ceratopogonidae	Culicoides	Culicoides imicola	SPECIES	37.66000	14.44000	Ref.11
702	Diptera	Ceratopogonidae	Culicoides	Culicoides imicola	SPECIES	37.66000	14.52000	Ref.11
703	Diptera	Ceratopogonidae	Culicoides	Culicoides imicola	SPECIES	37.66000	14.81000	Ref.11
704	Diptera	Ceratopogonidae	Culicoides	Culicoides imicola	SPECIES	37.66000	13.56000	Ref.11
705	Diptera	Ceratopogonidae	Culicoides	Culicoides imicola	SPECIES	37.66700	-5.28300	Ref.11
706	Diptera	Ceratopogonidae	Culicoides	Culicoides imicola	SPECIES	37.67000	12.59000	Ref.11
707	Diptera	Ceratopogonidae	Culicoides	Culicoides imicola	SPECIES	37.67000	-5.51000	Ref.11
708	Diptera	Ceratopogonidae	Culicoides	Culicoides imicola	SPECIES	37.67200	-8.76700	Ref.11
709	Diptera	Ceratopogonidae	Culicoides	Culicoides imicola	SPECIES	37.68000	14.34000	Ref.11
710	Diptera	Ceratopogonidae	Culicoides	Culicoides imicola	SPECIES	37.68000	14.65000	Ref.11
711	Diptera	Ceratopogonidae	Culicoides	Culicoides imicola	SPECIES	37.68000	13.03000	Ref.11
712	Diptera	Ceratopogonidae	Culicoides	Culicoides imicola	SPECIES	37.69000	14.26000	Ref.11
713	Diptera	Ceratopogonidae	Culicoides	Culicoides imicola	SPECIES	37.70000	-7.46700	Ref.11
714	Diptera	Ceratopogonidae	Culicoides	Culicoides imicola	SPECIES	37.70000	-7.16700	Ref.11
715	Diptera	Ceratopogonidae	Culicoides	Culicoides imicola	SPECIES	37.71000	14.91000	Ref.11
716	Diptera	Ceratopogonidae	Culicoides	Culicoides imicola	SPECIES	37.71000	-8.61000	Ref.11
717	Diptera	Ceratopogonidae	Culicoides	Culicoides imicola	SPECIES	37.72000	13.38000	Ref.11
718	Diptera	Ceratopogonidae	Culicoides	Culicoides imicola	SPECIES	37.72000	13.67000	Ref.11
719	Diptera	Ceratopogonidae	Culicoides	Culicoides imicola	SPECIES	37.72000	14.37000	Ref.11
720	Diptera	Ceratopogonidae	Culicoides	Culicoides imicola	SPECIES	37.72000	13.75000	Ref.11
721	Diptera	Ceratopogonidae	Culicoides	Culicoides imicola	SPECIES	37.72000	15.16000	Ref.11
722	Diptera	Ceratopogonidae	Culicoides	Culicoides imicola	SPECIES	37.73000	14.00000	Ref.11
723	Diptera	Ceratopogonidae	Culicoides	Culicoides imicola	SPECIES	37.73000	14.10000	Ref.11
724	Diptera	Ceratopogonidae	Culicoides	Culicoides imicola	SPECIES	37.73000	14.70000	Ref.11
725	Diptera	Ceratopogonidae	Culicoides	Culicoides imicola	SPECIES	37.73000	12.51000	Ref.11
726	Diptera	Ceratopogonidae	Culicoides	Culicoides imicola	SPECIES	37.74000	12.68000	Ref.11
727	Diptera	Ceratopogonidae	Culicoides	Culicoides imicola	SPECIES	37.74000	-6.01000	Ref.11
728	Diptera	Ceratopogonidae	Culicoides	Culicoides imicola	SPECIES	37.75000	13.44000	Ref.11
729	Diptera	Ceratopogonidae	Culicoides	Culicoides imicola	SPECIES	37.75000	14.21000	Ref.11
730	Diptera	Ceratopogonidae	Culicoides	Culicoides imicola	SPECIES	37.75000	14.54000	Ref.11
731	Diptera	Ceratopogonidae	Culicoides	Culicoides imicola	SPECIES	37.75000	12.80000	Ref.11
732	Diptera	Ceratopogonidae	Culicoides	Culicoides imicola	SPECIES	37.75000	-5.15000	Ref.11
733	Diptera	Ceratopogonidae	Culicoides	Culicoides imicola	SPECIES	37.75000	26.83300	Ref.11
734	Diptera	Ceratopogonidae	Culicoides	Culicoides imicola	SPECIES	37.75000	-8.34000	Ref.11
735	Diptera	Ceratopogonidae	Culicoides	Culicoides imicola	SPECIES	37.75000	-7.55000	Ref.11
736	Diptera	Ceratopogonidae	Culicoides	Culicoides imicola	SPECIES	37.76000	13.82000	Ref.11
737	Diptera	Ceratopogonidae	Culicoides	Culicoides imicola	SPECIES	37.76000	13.50000	Ref.11
738	Diptera	Ceratopogonidae	Culicoides	Culicoides imicola	SPECIES	37.76000	13.02000	Ref.11

№	Order	Family	Genus	Species	Taxon Rank	Latitude	Longitude	GBIF_ID
739	Diptera	Ceratopogonidae	Culicoides	Culicoides imicola	SPECIES	37.76000	12.56000	Ref.11
740	Diptera	Ceratopogonidae	Culicoides	Culicoides imicola	SPECIES	37.77000	14.48000	Ref.11
741	Diptera	Ceratopogonidae	Culicoides	Culicoides imicola	SPECIES	37.77000	14.39000	Ref.11
742	Diptera	Ceratopogonidae	Culicoides	Culicoides imicola	SPECIES	37.78000	14.01000	Ref.11
743	Diptera	Ceratopogonidae	Culicoides	Culicoides imicola	SPECIES	37.78000	14.33000	Ref.11
744	Diptera	Ceratopogonidae	Culicoides	Culicoides imicola	SPECIES	37.78000	12.95000	Ref.11
745	Diptera	Ceratopogonidae	Culicoides	Culicoides imicola	SPECIES	37.78000	12.50000	Ref.11
746	Diptera	Ceratopogonidae	Culicoides	Culicoides imicola	SPECIES	37.78300	29.00000	Ref.11
747	Diptera	Ceratopogonidae	Culicoides	Culicoides imicola	SPECIES	37.78300	-7.13300	Ref.11
748	Diptera	Ceratopogonidae	Culicoides	Culicoides imicola	SPECIES	37.79000	13.37000	Ref.11
749	Diptera	Ceratopogonidae	Culicoides	Culicoides imicola	SPECIES	37.79000	13.94000	Ref.11
750	Diptera	Ceratopogonidae	Culicoides	Culicoides imicola	SPECIES	37.79000	13.72000	Ref.11
751	Diptera	Ceratopogonidae	Culicoides	Culicoides imicola	SPECIES	37.79000	13.62000	Ref.11
752	Diptera	Ceratopogonidae	Culicoides	Culicoides imicola	SPECIES	37.80000	14.80000	Ref.11
753	Diptera	Ceratopogonidae	Culicoides	Culicoides imicola	SPECIES	37.80000	12.75000	Ref.11
754	Diptera	Ceratopogonidae	Culicoides	Culicoides imicola	SPECIES	37.81000	13.85000	Ref.11
755	Diptera	Ceratopogonidae	Culicoides	Culicoides imicola	SPECIES	37.81000	14.09000	Ref.11
756	Diptera	Ceratopogonidae	Culicoides	Culicoides imicola	SPECIES	37.81000	14.44000	Ref.11
757	Diptera	Ceratopogonidae	Culicoides	Culicoides imicola	SPECIES	37.81000	14.70000	Ref.11
758	Diptera	Ceratopogonidae	Culicoides	Culicoides imicola	SPECIES	37.81000	15.21000	Ref.11
759	Diptera	Ceratopogonidae	Culicoides	Culicoides imicola	SPECIES	37.81000	14.87000	Ref.11
760	Diptera	Ceratopogonidae	Culicoides	Culicoides imicola	SPECIES	37.81000	12.64000	Ref.11
761	Diptera	Ceratopogonidae	Culicoides	Culicoides imicola	SPECIES	37.81700	27.81700	Ref.11
762	Diptera	Ceratopogonidae	Culicoides	Culicoides imicola	SPECIES	37.81700	-5.13300	Ref.11
763	Diptera	Ceratopogonidae	Culicoides	Culicoides imicola	SPECIES	37.82000	-6.44000	Ref.11
764	Diptera	Ceratopogonidae	Culicoides	Culicoides imicola	SPECIES	37.82000	13.78000	Ref.11
765	Diptera	Ceratopogonidae	Culicoides	Culicoides imicola	SPECIES	37.82000	14.17000	Ref.11
766	Diptera	Ceratopogonidae	Culicoides	Culicoides imicola	SPECIES	37.82000	14.64000	Ref.11
767	Diptera	Ceratopogonidae	Culicoides	Culicoides imicola	SPECIES	37.82000	14.56000	Ref.11
768	Diptera	Ceratopogonidae	Culicoides	Culicoides imicola	SPECIES	37.82000	15.15000	Ref.11
769	Diptera	Ceratopogonidae	Culicoides	Culicoides imicola	SPECIES	37.82000	12.86000	Ref.11
770	Diptera	Ceratopogonidae	Culicoides	Culicoides imicola	SPECIES	37.83000	-6.99000	Ref.11
771	Diptera	Ceratopogonidae	Culicoides	Culicoides imicola	SPECIES	37.83000	13.43000	Ref.11
772	Diptera	Ceratopogonidae	Culicoides	Culicoides imicola	SPECIES	37.83000	13.29000	Ref.11
773	Diptera	Ceratopogonidae	Culicoides	Culicoides imicola	SPECIES	37.83000	12.80000	Ref.11
774	Diptera	Ceratopogonidae	Culicoides	Culicoides imicola	SPECIES	37.83300	-5.58300	Ref.11
775	Diptera	Ceratopogonidae	Culicoides	Culicoides imicola	SPECIES	37.84000	13.56000	Ref.11
776	Diptera	Ceratopogonidae	Culicoides	Culicoides imicola	SPECIES	37.84000	13.15000	Ref.11
777	Diptera	Ceratopogonidae	Culicoides	Culicoides imicola	SPECIES	37.84000	14.25000	Ref.11
778	Diptera	Ceratopogonidae	Culicoides	Culicoides imicola	SPECIES	37.85000	14.47000	Ref.11
779	Diptera	Ceratopogonidae	Culicoides	Culicoides imicola	SPECIES	37.85000	27.95000	Ref.11

№	Order	Family	Genus	Species	Taxon Rank	Latitude	Longitude	GBIF_ID
780	Diptera	Ceratopogonidae	Culicoides	Culicoides imicola	SPECIES	37.85000	-6.28300	Ref.11
781	Diptera	Ceratopogonidae	Culicoides	Culicoides imicola	SPECIES	37.85000	-8.09000	Ref.11
782	Diptera	Ceratopogonidae	Culicoides	Culicoides imicola	SPECIES	37.86000	-2.18000	Ref.11
783	Diptera	Ceratopogonidae	Culicoides	Culicoides imicola	SPECIES	37.86000	14.69000	Ref.11
784	Diptera	Ceratopogonidae	Culicoides	Culicoides imicola	SPECIES	37.86000	14.39000	Ref.11
785	Diptera	Ceratopogonidae	Culicoides	Culicoides imicola	SPECIES	37.86700	-6.55000	Ref.11
786	Diptera	Ceratopogonidae	Culicoides	Culicoides imicola	SPECIES	37.87000	13.85000	Ref.11
787	Diptera	Ceratopogonidae	Culicoides	Culicoides imicola	SPECIES	37.87000	13.67000	Ref.11
788	Diptera	Ceratopogonidae	Culicoides	Culicoides imicola	SPECIES	37.87000	14.82000	Ref.11
789	Diptera	Ceratopogonidae	Culicoides	Culicoides imicola	SPECIES	37.87000	14.91000	Ref.11
790	Diptera	Ceratopogonidae	Culicoides	Culicoides imicola	SPECIES	37.87000	12.55000	Ref.11
791	Diptera	Ceratopogonidae	Culicoides	Culicoides imicola	SPECIES	37.87000	12.64000	Ref.11
792	Diptera	Ceratopogonidae	Culicoides	Culicoides imicola	SPECIES	37.87000	-4.33000	Ref.11
793	Diptera	Ceratopogonidae	Culicoides	Culicoides imicola	SPECIES	37.88000	14.32000	Ref.11
794	Diptera	Ceratopogonidae	Culicoides	Culicoides imicola	SPECIES	37.88000	14.76000	Ref.11
795	Diptera	Ceratopogonidae	Culicoides	Culicoides imicola	SPECIES	37.88000	12.76000	Ref.11
796	Diptera	Ceratopogonidae	Culicoides	Culicoides imicola	SPECIES	37.88300	-5.63300	Ref.11
797	Diptera	Ceratopogonidae	Culicoides	Culicoides imicola	SPECIES	37.89000	13.26000	Ref.11
798	Diptera	Ceratopogonidae	Culicoides	Culicoides imicola	SPECIES	37.89000	15.08000	Ref.11
799	Diptera	Ceratopogonidae	Culicoides	Culicoides imicola	SPECIES	37.90000	13.90000	Ref.11
800	Diptera	Ceratopogonidae	Culicoides	Culicoides imicola	SPECIES	37.90000	13.58000	Ref.11
801	Diptera	Ceratopogonidae	Culicoides	Culicoides imicola	SPECIES	37.90000	13.72000	Ref.11
802	Diptera	Ceratopogonidae	Culicoides	Culicoides imicola	SPECIES	37.90000	13.08000	Ref.11
803	Diptera	Ceratopogonidae	Culicoides	Culicoides imicola	SPECIES	37.90000	15.16000	Ref.11
804	Diptera	Ceratopogonidae	Culicoides	Culicoides imicola	SPECIES	37.90000	12.48000	Ref.11
805	Diptera	Ceratopogonidae	Culicoides	Culicoides imicola	SPECIES	37.90000	12.91000	Ref.11
806	Diptera	Ceratopogonidae	Culicoides	Culicoides imicola	SPECIES	37.90000	12.70000	Ref.11
807	Diptera	Ceratopogonidae	Culicoides	Culicoides imicola	SPECIES	37.90000	28.31700	Ref.11
808	Diptera	Ceratopogonidae	Culicoides	Culicoides imicola	SPECIES	37.90000	29.06700	Ref.11
809	Diptera	Ceratopogonidae	Culicoides	Culicoides imicola	SPECIES	37.91000	13.02000	Ref.11
810	Diptera	Ceratopogonidae	Culicoides	Culicoides imicola	SPECIES	37.91000	14.40000	Ref.11
811	Diptera	Ceratopogonidae	Culicoides	Culicoides imicola	SPECIES	37.91000	14.57000	Ref.11
812	Diptera	Ceratopogonidae	Culicoides	Culicoides imicola	SPECIES	37.91000	14.97000	Ref.11
813	Diptera	Ceratopogonidae	Culicoides	Culicoides imicola	SPECIES	37.91000	12.82000	Ref.11
814	Diptera	Ceratopogonidae	Culicoides	Culicoides imicola	SPECIES	37.91700	13.83300	Ref.11
815	Diptera	Ceratopogonidae	Culicoides	Culicoides imicola	SPECIES	37.92000	12.62000	Ref.11
816	Diptera	Ceratopogonidae	Culicoides	Culicoides imicola	SPECIES	37.92000	12.56000	Ref.11
817	Diptera	Ceratopogonidae	Culicoides	Culicoides imicola	SPECIES	37.93000	14.12000	Ref.11
818	Diptera	Ceratopogonidae	Culicoides	Culicoides imicola	SPECIES	37.93000	13.22000	Ref.11
819	Diptera	Ceratopogonidae	Culicoides	Culicoides imicola	SPECIES	37.93000	14.72000	Ref.11
820	Diptera	Ceratopogonidae	Culicoides	Culicoides imicola	SPECIES	37.93000	15.34000	Ref.11

№	Order	Family	Genus	Species	Taxon Rank	Latitude	Longitude	GBIF_ID
821	Diptera	Ceratopogonidae	Culicoides	Culicoides imicola	SPECIES	37.93000	14.87000	Ref.11
822	Diptera	Ceratopogonidae	Culicoides	Culicoides imicola	SPECIES	37.94000	13.53000	Ref.11
823	Diptera	Ceratopogonidae	Culicoides	Culicoides imicola	SPECIES	37.94000	13.47000	Ref.11
824	Diptera	Ceratopogonidae	Culicoides	Culicoides imicola	SPECIES	37.95000	13.11000	Ref.11
825	Diptera	Ceratopogonidae	Culicoides	Culicoides imicola	SPECIES	37.95000	13.88000	Ref.11
826	Diptera	Ceratopogonidae	Culicoides	Culicoides imicola	SPECIES	37.95000	14.04000	Ref.11
827	Diptera	Ceratopogonidae	Culicoides	Culicoides imicola	SPECIES	37.95000	14.26000	Ref.11
828	Diptera	Ceratopogonidae	Culicoides	Culicoides imicola	SPECIES	37.95000	15.00000	Ref.11
829	Diptera	Ceratopogonidae	Culicoides	Culicoides imicola	SPECIES	37.96000	13.63000	Ref.11
830	Diptera	Ceratopogonidae	Culicoides	Culicoides imicola	SPECIES	37.96000	15.27000	Ref.11
831	Diptera	Ceratopogonidae	Culicoides	Culicoides imicola	SPECIES	37.96000	14.48000	Ref.11
832	Diptera	Ceratopogonidae	Culicoides	Culicoides imicola	SPECIES	37.96000	14.33000	Ref.11
833	Diptera	Ceratopogonidae	Culicoides	Culicoides imicola	SPECIES	37.96000	14.65000	Ref.11
834	Diptera	Ceratopogonidae	Culicoides	Culicoides imicola	SPECIES	37.96000	14.77000	Ref.11
835	Diptera	Ceratopogonidae	Culicoides	Culicoides imicola	SPECIES	37.96000	12.68000	Ref.11
836	Diptera	Ceratopogonidae	Culicoides	Culicoides imicola	SPECIES	37.96700	-7.58300	Ref.11
837	Diptera	Ceratopogonidae	Culicoides	Culicoides imicola	SPECIES	37.96700	-6.96700	Ref.11
838	Diptera	Ceratopogonidae	Culicoides	Culicoides imicola	SPECIES	37.97000	13.31000	Ref.11
839	Diptera	Ceratopogonidae	Culicoides	Culicoides imicola	SPECIES	37.97000	14.83000	Ref.11
840	Diptera	Ceratopogonidae	Culicoides	Culicoides imicola	SPECIES	37.97000	12.53000	Ref.11
841	Diptera	Ceratopogonidae	Culicoides	Culicoides imicola	SPECIES	37.97000	12.98000	Ref.11
842	Diptera	Ceratopogonidae	Culicoides	Culicoides imicola	SPECIES	37.98000	-5.60000	Ref.11
843	Diptera	Ceratopogonidae	Culicoides	Culicoides imicola	SPECIES	37.98000	13.94000	Ref.11
844	Diptera	Ceratopogonidae	Culicoides	Culicoides imicola	SPECIES	37.98000	13.44000	Ref.11
845	Diptera	Ceratopogonidae	Culicoides	Culicoides imicola	SPECIES	37.98000	13.16000	Ref.11
846	Diptera	Ceratopogonidae	Culicoides	Culicoides imicola	SPECIES	37.98000	14.59000	Ref.11
847	Diptera	Ceratopogonidae	Culicoides	Culicoides imicola	SPECIES	37.98000	14.39000	Ref.11
848	Diptera	Ceratopogonidae	Culicoides	Culicoides imicola	SPECIES	37.99000	13.52000	Ref.11
849	Diptera	Ceratopogonidae	Culicoides	Culicoides imicola	SPECIES	37.99000	15.35000	Ref.11
850	Diptera	Ceratopogonidae	Culicoides	Culicoides imicola	SPECIES	37.99000	12.83000	Ref.11
851	Diptera	Ceratopogonidae	Culicoides	Culicoides imicola	SPECIES	37.99000	-7.93000	Ref.11
852	Diptera	Ceratopogonidae	Culicoides	Culicoides imicola	SPECIES	38.00000	14.13000	Ref.11
853	Diptera	Ceratopogonidae	Culicoides	Culicoides imicola	SPECIES	38.00000	15.41000	Ref.11
854	Diptera	Ceratopogonidae	Culicoides	Culicoides imicola	SPECIES	38.00000	12.62000	Ref.11
855	Diptera	Ceratopogonidae	Culicoides	Culicoides imicola	SPECIES	38.00000	12.93000	Ref.11
856	Diptera	Ceratopogonidae	Culicoides	Culicoides imicola	SPECIES	38.01000	15.10000	Ref.11
857	Diptera	Ceratopogonidae	Culicoides	Culicoides imicola	SPECIES	38.01000	12.73000	Ref.11
858	Diptera	Ceratopogonidae	Culicoides	Culicoides imicola	SPECIES	38.01000	12.56000	Ref.11
859	Diptera	Ceratopogonidae	Culicoides	Culicoides imicola	SPECIES	38.01700	-4.35000	Ref.11
860	Diptera	Ceratopogonidae	Culicoides	Culicoides imicola	SPECIES	38.02000	14.69000	Ref.11
861	Diptera	Ceratopogonidae	Culicoides	Culicoides imicola	SPECIES	38.02000	15.02000	Ref.11

№	Order	Family	Genus	Species	Taxon Rank	Latitude	Longitude	GBIF_ID
862	Diptera	Ceratopogonidae	Culicoides	Culicoides imicola	SPECIES	38.02000	14.88000	Ref.11
863	Diptera	Ceratopogonidae	Culicoides	Culicoides imicola	SPECIES	38.02000	14.95000	Ref.11
864	Diptera	Ceratopogonidae	Culicoides	Culicoides imicola	SPECIES	38.02000	14.78000	Ref.11
865	Diptera	Ceratopogonidae	Culicoides	Culicoides imicola	SPECIES	38.02000	-7.81000	Ref.11
866	Diptera	Ceratopogonidae	Culicoides	Culicoides imicola	SPECIES	38.03000	13.42000	Ref.11
867	Diptera	Ceratopogonidae	Culicoides	Culicoides imicola	SPECIES	38.03000	14.48000	Ref.11
868	Diptera	Ceratopogonidae	Culicoides	Culicoides imicola	SPECIES	38.03000	12.67000	Ref.11
869	Diptera	Ceratopogonidae	Culicoides	Culicoides imicola	SPECIES	38.03000	-7.90000	Ref.11
870	Diptera	Ceratopogonidae	Culicoides	Culicoides imicola	SPECIES	38.04000	13.28000	Ref.11
871	Diptera	Ceratopogonidae	Culicoides	Culicoides imicola	SPECIES	38.04000	13.05000	Ref.11
872	Diptera	Ceratopogonidae	Culicoides	Culicoides imicola	SPECIES	38.04000	14.57000	Ref.11
873	Diptera	Ceratopogonidae	Culicoides	Culicoides imicola	SPECIES	38.05000	13.51000	Ref.11
874	Diptera	Ceratopogonidae	Culicoides	Culicoides imicola	SPECIES	38.05000	13.11000	Ref.11
875	Diptera	Ceratopogonidae	Culicoides	Culicoides imicola	SPECIES	38.05000	14.63000	Ref.11
876	Diptera	Ceratopogonidae	Culicoides	Culicoides imicola	SPECIES	38.05000	12.83000	Ref.11
877	Diptera	Ceratopogonidae	Culicoides	Culicoides imicola	SPECIES	38.06000	15.05000	Ref.11
878	Diptera	Ceratopogonidae	Culicoides	Culicoides imicola	SPECIES	38.06000	15.16000	Ref.11
879	Diptera	Ceratopogonidae	Culicoides	Culicoides imicola	SPECIES	38.06000	14.84000	Ref.11
880	Diptera	Ceratopogonidae	Culicoides	Culicoides imicola	SPECIES	38.06000	14.92000	Ref.11
881	Diptera	Ceratopogonidae	Culicoides	Culicoides imicola	SPECIES	38.06000	12.60000	Ref.11
882	Diptera	Ceratopogonidae	Culicoides	Culicoides imicola	SPECIES	38.07000	14.99000	Ref.11
883	Diptera	Ceratopogonidae	Culicoides	Culicoides imicola	SPECIES	38.08000	13.45000	Ref.11
884	Diptera	Ceratopogonidae	Culicoides	Culicoides imicola	SPECIES	38.08000	13.33000	Ref.11
885	Diptera	Ceratopogonidae	Culicoides	Culicoides imicola	SPECIES	38.08000	-7.22000	Ref.11
886	Diptera	Ceratopogonidae	Culicoides	Culicoides imicola	SPECIES	38.08900	-7.48300	Ref.11
887	Diptera	Ceratopogonidae	Culicoides	Culicoides imicola	SPECIES	38.09000	13.18000	Ref.11
888	Diptera	Ceratopogonidae	Culicoides	Culicoides imicola	SPECIES	38.09000	14.79000	Ref.11
889	Diptera	Ceratopogonidae	Culicoides	Culicoides imicola	SPECIES	38.09000	12.71000	Ref.11
890	Diptera	Ceratopogonidae	Culicoides	Culicoides imicola	SPECIES	38.10000	15.08000	Ref.11
891	Diptera	Ceratopogonidae	Culicoides	Culicoides imicola	SPECIES	38.10000	14.71000	Ref.11
892	Diptera	Ceratopogonidae	Culicoides	Culicoides imicola	SPECIES	38.10000	-8.20000	Ref.11
893	Diptera	Ceratopogonidae	Culicoides	Culicoides imicola	SPECIES	38.10000	-3.76700	Ref.11
894	Diptera	Ceratopogonidae	Culicoides	Culicoides imicola	SPECIES	38.10000	-7.41000	Ref.11
895	Diptera	Ceratopogonidae	Culicoides	Culicoides imicola	SPECIES	38.11000	14.89000	Ref.11
896	Diptera	Ceratopogonidae	Culicoides	Culicoides imicola	SPECIES	38.11700	-7.93300	Ref.11
897	Diptera	Ceratopogonidae	Culicoides	Culicoides imicola	SPECIES	38.11700	-7.01700	Ref.11
898	Diptera	Ceratopogonidae	Culicoides	Culicoides imicola	SPECIES	38.12000	13.30000	Ref.11
899	Diptera	Ceratopogonidae	Culicoides	Culicoides imicola	SPECIES	38.12000	14.99000	Ref.11
900	Diptera	Ceratopogonidae	Culicoides	Culicoides imicola	SPECIES	38.12000	15.15000	Ref.11
901	Diptera	Ceratopogonidae	Culicoides	Culicoides imicola	SPECIES	38.12000	15.49000	Ref.11
902	Diptera	Ceratopogonidae	Culicoides	Culicoides imicola	SPECIES	38.12000	15.36000	Ref.11

№	Order	Family	Genus	Species	Taxon Rank	Latitude	Longitude	GBIF_ID
903	Diptera	Ceratopogonidae	Culicoides	Culicoides imicola	SPECIES	38.13000	13.11000	Ref.11
904	Diptera	Ceratopogonidae	Culicoides	Culicoides imicola	SPECIES	38.13000	15.27000	Ref.11
905	Diptera	Ceratopogonidae	Culicoides	Culicoides imicola	SPECIES	38.14000	15.21000	Ref.11
906	Diptera	Ceratopogonidae	Culicoides	Culicoides imicola	SPECIES	38.14000	14.79000	Ref.11
907	Diptera	Ceratopogonidae	Culicoides	Culicoides imicola	SPECIES	38.14000	-8.50000	Ref.11
908	Diptera	Ceratopogonidae	Culicoides	Culicoides imicola	SPECIES	38.15000	27.35000	Ref.11
909	Diptera	Ceratopogonidae	Culicoides	Culicoides imicola	SPECIES	38.15300	-8.71900	Ref.11
910	Diptera	Ceratopogonidae	Culicoides	Culicoides imicola	SPECIES	38.16000	15.53000	Ref.11
911	Diptera	Ceratopogonidae	Culicoides	Culicoides imicola	SPECIES	38.16000	14.90000	Ref.11
912	Diptera	Ceratopogonidae	Culicoides	Culicoides imicola	SPECIES	38.18000	-4.15000	Ref.11
913	Diptera	Ceratopogonidae	Culicoides	Culicoides imicola	SPECIES	38.19000	13.34000	Ref.11
914	Diptera	Ceratopogonidae	Culicoides	Culicoides imicola	SPECIES	38.19000	15.32000	Ref.11
915	Diptera	Ceratopogonidae	Culicoides	Culicoides imicola	SPECIES	38.20000	15.26000	Ref.11
916	Diptera	Ceratopogonidae	Culicoides	Culicoides imicola	SPECIES	38.20000	-4.28300	Ref.11
917	Diptera	Ceratopogonidae	Culicoides	Culicoides imicola	SPECIES	38.21000	15.54000	Ref.11
918	Diptera	Ceratopogonidae	Culicoides	Culicoides imicola	SPECIES	38.21700	-3.61700	Ref.11
919	Diptera	Ceratopogonidae	Culicoides	Culicoides imicola	SPECIES	38.23000	15.43000	Ref.11
920	Diptera	Ceratopogonidae	Culicoides	Culicoides imicola	SPECIES	38.25900	-5.67800	Ref.11
921	Diptera	Ceratopogonidae	Culicoides	Culicoides imicola	SPECIES	38.26000	-3.52000	Ref.11
922	Diptera	Ceratopogonidae	Culicoides	Culicoides imicola	SPECIES	38.27000	15.55000	Ref.11
923	Diptera	Ceratopogonidae	Culicoides	Culicoides imicola	SPECIES	38.30000	-5.21700	Ref.11
924	Diptera	Ceratopogonidae	Culicoides	Culicoides imicola	SPECIES	38.36700	-7.93300	Ref.11
925	Diptera	Ceratopogonidae	Culicoides	Culicoides imicola	SPECIES	38.36700	-4.80000	Ref.11
926	Diptera	Ceratopogonidae	Culicoides	Culicoides imicola	SPECIES	38.37000	-8.51000	Ref.11
927	Diptera	Ceratopogonidae	Culicoides	Culicoides imicola	SPECIES	38.37500	-8.07200	Ref.11
928	Diptera	Ceratopogonidae	Culicoides	Culicoides imicola	SPECIES	38.38100	-7.31100	Ref.11
929	Diptera	Ceratopogonidae	Culicoides	Culicoides imicola	SPECIES	38.40000	-7.55000	Ref.11
930	Diptera	Ceratopogonidae	Culicoides	Culicoides imicola	SPECIES	38.43000	-5.05000	Ref.11
931	Diptera	Ceratopogonidae	Culicoides	Culicoides imicola	SPECIES	38.43300	-5.56700	Ref.11
932	Diptera	Ceratopogonidae	Culicoides	Culicoides imicola	SPECIES	38.45000	27.21700	Ref.11
933	Diptera	Ceratopogonidae	Culicoides	Culicoides imicola	SPECIES	38.45000	24.03300	Ref.11
934	Diptera	Ceratopogonidae	Culicoides	Culicoides imicola	SPECIES	38.50000	14.92000	Ref.11
935	Diptera	Ceratopogonidae	Culicoides	Culicoides imicola	SPECIES	38.50000	-8.03300	Ref.11
936	Diptera	Ceratopogonidae	Culicoides	Culicoides imicola	SPECIES	38.50000	-7.85000	Ref.11
937	Diptera	Ceratopogonidae	Culicoides	Culicoides imicola	SPECIES	38.53300	-7.95000	Ref.11
938	Diptera	Ceratopogonidae	Culicoides	Culicoides imicola	SPECIES	38.54200	-9.05600	Ref.11
939	Diptera	Ceratopogonidae	Culicoides	Culicoides imicola	SPECIES	38.56700	-9.11700	Ref.11
940	Diptera	Ceratopogonidae	Culicoides	Culicoides imicola	SPECIES	38.58000	-8.94000	Ref.11
941	Diptera	Ceratopogonidae	Culicoides	Culicoides imicola	SPECIES	38.60000	-3.88300	Ref.11
942	Diptera	Ceratopogonidae	Culicoides	Culicoides imicola	SPECIES	38.63000	-8.23000	Ref.11
943	Diptera	Ceratopogonidae	Culicoides	Culicoides imicola	SPECIES	38.65000	-7.98000	Ref.11

№	Order	Family	Genus	Species	Taxon Rank	Latitude	Longitude	GBIF_ID
944	Diptera	Ceratopogonidae	Culicoides	Culicoides imicola	SPECIES	38.65000	-8.64000	Ref.11
945	Diptera	Ceratopogonidae	Culicoides	Culicoides imicola	SPECIES	38.70400	-3.77600	Ref.11
946	Diptera	Ceratopogonidae	Culicoides	Culicoides imicola	SPECIES	38.71000	-8.92000	Ref.11
947	Diptera	Ceratopogonidae	Culicoides	Culicoides imicola	SPECIES	38.73000	-8.78000	Ref.11
948	Diptera	Ceratopogonidae	Culicoides	Culicoides imicola	SPECIES	38.74000	-8.08000	Ref.11
949	Diptera	Ceratopogonidae	Culicoides	Culicoides imicola	SPECIES	38.76700	-8.31700	Ref.11
950	Diptera	Ceratopogonidae	Culicoides	Culicoides imicola	SPECIES	38.78000	-7.19000	Ref.11
951	Diptera	Ceratopogonidae	Culicoides	Culicoides imicola	SPECIES	38.79000	-8.05000	Ref.11
952	Diptera	Ceratopogonidae	Culicoides	Culicoides imicola	SPECIES	38.83000	-8.59000	Ref.11
953	Diptera	Ceratopogonidae	Culicoides	Culicoides imicola	SPECIES	38.88300	-6.96700	Ref.11
954	Diptera	Ceratopogonidae	Culicoides	Culicoides imicola	SPECIES	38.91000	-4.27000	Ref.11
955	Diptera	Ceratopogonidae	Culicoides	Culicoides imicola	SPECIES	38.93100	-8.56900	Ref.11
956	Diptera	Ceratopogonidae	Culicoides	Culicoides imicola	SPECIES	38.93300	-6.85000	Ref.11
957	Diptera	Ceratopogonidae	Culicoides	Culicoides imicola	SPECIES	38.96900	-7.29400	Ref.11
958	Diptera	Ceratopogonidae	Culicoides	Culicoides imicola	SPECIES	38.97000	-8.70000	Ref.11
959	Diptera	Ceratopogonidae	Culicoides	Culicoides imicola	SPECIES	38.98000	8.42000	Ref.11
960	Diptera	Ceratopogonidae	Culicoides	Culicoides imicola	SPECIES	38.98000	-8.84000	Ref.11
961	Diptera	Ceratopogonidae	Culicoides	Culicoides imicola	SPECIES	38.99000	-8.46000	Ref.11
962	Diptera	Ceratopogonidae	Culicoides	Culicoides imicola	SPECIES	39.04000	-7.89000	Ref.11
963	Diptera	Ceratopogonidae	Culicoides	Culicoides imicola	SPECIES	39.08000	-9.00000	Ref.11
964	Diptera	Ceratopogonidae	Culicoides	Culicoides imicola	SPECIES	39.08300	-7.46900	Ref.11
965	Diptera	Ceratopogonidae	Culicoides	Culicoides imicola	SPECIES	39.08300	-8.68300	Ref.11
966	Diptera	Ceratopogonidae	Culicoides	Culicoides imicola	SPECIES	39.11000	-8.40000	Ref.11
967	Diptera	Ceratopogonidae	Culicoides	Culicoides imicola	SPECIES	39.13000	8.52000	Ref.11
968	Diptera	Ceratopogonidae	Culicoides	Culicoides imicola	SPECIES	39.15000	26.26700	Ref.11
969	Diptera	Ceratopogonidae	Culicoides	Culicoides imicola	SPECIES	39.16700	26.33300	Ref.11
970	Diptera	Ceratopogonidae	Culicoides	Culicoides imicola	SPECIES	39.22500	-7.69200	Ref.11
971	Diptera	Ceratopogonidae	Culicoides	Culicoides imicola	SPECIES	39.22500	-8.63100	Ref.11
972	Diptera	Ceratopogonidae	Culicoides	Culicoides imicola	SPECIES	39.26000	-8.89000	Ref.11
973	Diptera	Ceratopogonidae	Culicoides	Culicoides imicola	SPECIES	39.26000	-8.32000	Ref.11
974	Diptera	Ceratopogonidae	Culicoides	Culicoides imicola	SPECIES	39.27000	-7.58000	Ref.11
975	Diptera	Ceratopogonidae	Culicoides	Culicoides imicola	SPECIES	39.28000	-7.97000	Ref.11
976	Diptera	Ceratopogonidae	Culicoides	Culicoides imicola	SPECIES	39.29000	-7.49000	Ref.11
977	Diptera	Ceratopogonidae	Culicoides	Culicoides imicola	SPECIES	39.30000	-7.43000	Ref.11
978	Diptera	Ceratopogonidae	Culicoides	Culicoides imicola	SPECIES	39.37500	-8.53300	Ref.11
979	Diptera	Ceratopogonidae	Culicoides	Culicoides imicola	SPECIES	39.39000	-4.12000	Ref.11
980	Diptera	Ceratopogonidae	Culicoides	Culicoides imicola	SPECIES	39.40000	-7.65000	Ref.11
981	Diptera	Ceratopogonidae	Culicoides	Culicoides imicola	SPECIES	39.41000	-8.92000	Ref.11
982	Diptera	Ceratopogonidae	Culicoides	Culicoides imicola	SPECIES	39.43300	-7.46700	Ref.11
983	Diptera	Ceratopogonidae	Culicoides	Culicoides imicola	SPECIES	39.45000	3.26700	Ref.11
984	Diptera	Ceratopogonidae	Culicoides	Culicoides imicola	SPECIES	39.45000	-8.40000	Ref.11

№	Order	Family	Genus	Species	Taxon Rank	Latitude	Longitude	GBIF_ID
985	Diptera	Ceratopogonidae	Culicoides	Culicoides imicola	SPECIES	39.48000	-8.28000	Ref.11
986	Diptera	Ceratopogonidae	Culicoides	Culicoides imicola	SPECIES	39.48300	3.08300	Ref.11
987	Diptera	Ceratopogonidae	Culicoides	Culicoides imicola	SPECIES	39.51000	-8.09000	Ref.11
988	Diptera	Ceratopogonidae	Culicoides	Culicoides imicola	SPECIES	39.53300	-7.63300	Ref.11
989	Diptera	Ceratopogonidae	Culicoides	Culicoides imicola	SPECIES	39.55000	-8.15000	Ref.11
990	Diptera	Ceratopogonidae	Culicoides	Culicoides imicola	SPECIES	39.57000	-8.42000	Ref.11
991	Diptera	Ceratopogonidae	Culicoides	Culicoides imicola	SPECIES	39.58300	2.75000	Ref.11
992	Diptera	Ceratopogonidae	Culicoides	Culicoides imicola	SPECIES	39.58300	-8.08300	Ref.11
993	Diptera	Ceratopogonidae	Culicoides	Culicoides imicola	SPECIES	39.58300	-5.38300	Ref.11
994	Diptera	Ceratopogonidae	Culicoides	Culicoides imicola	SPECIES	39.61000	2.95000	Ref.11
995	Diptera	Ceratopogonidae	Culicoides	Culicoides imicola	SPECIES	39.63300	3.38300	Ref.11
996	Diptera	Ceratopogonidae	Culicoides	Culicoides imicola	SPECIES	39.65000	-7.71000	Ref.11
997	Diptera	Ceratopogonidae	Culicoides	Culicoides imicola	SPECIES	39.66100	-7.63600	Ref.11
998	Diptera	Ceratopogonidae	Culicoides	Culicoides imicola	SPECIES	39.66700	3.43300	Ref.11
999	Diptera	Ceratopogonidae	Culicoides	Culicoides imicola	SPECIES	39.70000	-7.59000	Ref.11
1000	Diptera	Ceratopogonidae	Culicoides	Culicoides imicola	SPECIES	39.72000	-8.06000	Ref.11
1001	Diptera	Ceratopogonidae	Culicoides	Culicoides imicola	SPECIES	39.73000	-8.81000	Ref.11
1002	Diptera	Ceratopogonidae	Culicoides	Culicoides imicola	SPECIES	39.78000	-8.76000	Ref.11
1003	Diptera	Ceratopogonidae	Culicoides	Culicoides imicola	SPECIES	39.78000	-7.26000	Ref.11
1004	Diptera	Ceratopogonidae	Culicoides	Culicoides imicola	SPECIES	39.79400	-3.94300	Ref.11
1005	Diptera	Ceratopogonidae	Culicoides	Culicoides imicola	SPECIES	39.80000	8.78000	Ref.11
1006	Diptera	Ceratopogonidae	Culicoides	Culicoides imicola	SPECIES	39.81700	-6.98300	Ref.11
1007	Diptera	Ceratopogonidae	Culicoides	Culicoides imicola	SPECIES	39.83000	-7.34000	Ref.11
1008	Diptera	Ceratopogonidae	Culicoides	Culicoides imicola	SPECIES	39.83000	-8.13000	Ref.11
1009	Diptera	Ceratopogonidae	Culicoides	Culicoides imicola	SPECIES	39.83000	-8.22000	Ref.11
1010	Diptera	Ceratopogonidae	Culicoides	Culicoides imicola	SPECIES	39.84000	-8.91000	Ref.11
1011	Diptera	Ceratopogonidae	Culicoides	Culicoides imicola	SPECIES	39.84000	-7.50000	Ref.11
1012	Diptera	Ceratopogonidae	Culicoides	Culicoides imicola	SPECIES	39.85000	-6.81700	Ref.11
1013	Diptera	Ceratopogonidae	Culicoides	Culicoides imicola	SPECIES	39.87000	9.55000	Ref.11
1014	Diptera	Ceratopogonidae	Culicoides	Culicoides imicola	SPECIES	39.87000	-7.30000	Ref.11
1015	Diptera	Ceratopogonidae	Culicoides	Culicoides imicola	SPECIES	39.89000	-6.97000	Ref.11
1016	Diptera	Ceratopogonidae	Culicoides	Culicoides imicola	SPECIES	39.89300	-5.54000	Ref.11
1017	Diptera	Ceratopogonidae	Culicoides	Culicoides imicola	SPECIES	39.89400	22.64300	Ref.11
1018	Diptera	Ceratopogonidae	Culicoides	Culicoides imicola	SPECIES	39.91000	-7.21000	Ref.11
1019	Diptera	Ceratopogonidae	Culicoides	Culicoides imicola	SPECIES	39.91700	-7.43300	Ref.11
1020	Diptera	Ceratopogonidae	Culicoides	Culicoides imicola	SPECIES	39.92000	-7.30000	Ref.11
1021	Diptera	Ceratopogonidae	Culicoides	Culicoides imicola	SPECIES	39.93300	4.18300	Ref.11
1022	Diptera	Ceratopogonidae	Culicoides	Culicoides imicola	SPECIES	39.95800	-6.91700	Ref.11
1023	Diptera	Ceratopogonidae	Culicoides	Culicoides imicola	SPECIES	39.96700	-4.85000	Ref.11
1024	Diptera	Ceratopogonidae	Culicoides	Culicoides imicola	SPECIES	39.98300	3.98300	Ref.11
1025	Diptera	Ceratopogonidae	Culicoides	Culicoides imicola	SPECIES	39.98300	-6.53300	Ref.11

№	Order	Family	Genus	Species	Taxon Rank	Latitude	Longitude	GBIF_ID
1026	Diptera	Ceratopogonidae	Culicoides	Culicoides imicola	SPECIES	40.00000	-5.31700	Ref.11
1027	Diptera	Ceratopogonidae	Culicoides	Culicoides imicola	SPECIES	40.01000	-7.46000	Ref.11
1028	Diptera	Ceratopogonidae	Culicoides	Culicoides imicola	SPECIES	40.03300	4.08300	Ref.11
1029	Diptera	Ceratopogonidae	Culicoides	Culicoides imicola	SPECIES	40.05000	8.53000	Ref.11
1030	Diptera	Ceratopogonidae	Culicoides	Culicoides imicola	SPECIES	40.08000	-7.22000	Ref.11
1031	Diptera	Ceratopogonidae	Culicoides	Culicoides imicola	SPECIES	40.10000	-7.42000	Ref.11
1032	Diptera	Ceratopogonidae	Culicoides	Culicoides imicola	SPECIES	40.14400	-7.16900	Ref.11
1033	Diptera	Ceratopogonidae	Culicoides	Culicoides imicola	SPECIES	40.15000	-7.51000	Ref.11
1034	Diptera	Ceratopogonidae	Culicoides	Culicoides imicola	SPECIES	40.17000	-7.26000	Ref.11
1035	Diptera	Ceratopogonidae	Culicoides	Culicoides imicola	SPECIES	40.18300	-7.46700	Ref.11
1036	Diptera	Ceratopogonidae	Culicoides	Culicoides imicola	SPECIES	40.19000	-7.64000	Ref.11
1037	Diptera	Ceratopogonidae	Culicoides	Culicoides imicola	SPECIES	40.21000	-8.43000	Ref.11
1038	Diptera	Ceratopogonidae	Culicoides	Culicoides imicola	SPECIES	40.23000	-7.15000	Ref.11
1039	Diptera	Ceratopogonidae	Culicoides	Culicoides imicola	SPECIES	40.25000	-7.48000	Ref.11
1040	Diptera	Ceratopogonidae	Culicoides	Culicoides imicola	SPECIES	40.33800	23.49900	Ref.11
1041	Diptera	Ceratopogonidae	Culicoides	Culicoides imicola	SPECIES	40.39000	-7.25000	Ref.11
1042	Diptera	Ceratopogonidae	Culicoides	Culicoides imicola	SPECIES	40.46000	-8.44000	Ref.11
1043	Diptera	Ceratopogonidae	Culicoides	Culicoides imicola	SPECIES	40.46000	-8.01000	Ref.11
1044	Diptera	Ceratopogonidae	Culicoides	Culicoides imicola	SPECIES	40.51700	-7.95600	Ref.11
1045	Diptera	Ceratopogonidae	Culicoides	Culicoides imicola	SPECIES	40.52000	-8.02000	Ref.11
1046	Diptera	Ceratopogonidae	Culicoides	Culicoides imicola	SPECIES	40.56700	-8.45000	Ref.11
1047	Diptera	Ceratopogonidae	Culicoides	Culicoides imicola	SPECIES	40.63000	8.37000	Ref.11
1048	Diptera	Ceratopogonidae	Culicoides	Culicoides imicola	SPECIES	40.68000	0.47000	Ref.11
1049	Diptera	Ceratopogonidae	Culicoides	Culicoides imicola	SPECIES	40.73000	8.98000	Ref.11
1050	Diptera	Ceratopogonidae	Culicoides	Culicoides imicola	SPECIES	40.73000	0.56000	Ref.11
1051	Diptera	Ceratopogonidae	Culicoides	Culicoides imicola	SPECIES	40.75000	8.20000	Ref.11
1052	Diptera	Ceratopogonidae	Culicoides	Culicoides imicola	SPECIES	40.95000	25.36700	Ref.11
1053	Diptera	Ceratopogonidae	Culicoides	Culicoides imicola	SPECIES	41.01700	-6.95000	Ref.11
1054	Diptera	Ceratopogonidae	Culicoides	Culicoides imicola	SPECIES	41.02000	-8.33000	Ref.11
1055	Diptera	Ceratopogonidae	Culicoides	Culicoides imicola	SPECIES	41.05600	-7.14200	Ref.11
1056	Diptera	Ceratopogonidae	Culicoides	Culicoides imicola	SPECIES	41.08300	-6.80000	Ref.11
1057	Diptera	Ceratopogonidae	Culicoides	Culicoides imicola	SPECIES	41.08300	-6.90000	Ref.11
1058	Diptera	Ceratopogonidae	Culicoides	Culicoides imicola	SPECIES	41.14000	0.65000	Ref.11
1059	Diptera	Ceratopogonidae	Culicoides	Culicoides imicola	SPECIES	41.15000	9.38000	Ref.11
1060	Diptera	Ceratopogonidae	Culicoides	Culicoides imicola	SPECIES	41.22000	1.42000	Ref.11
1061	Diptera	Ceratopogonidae	Culicoides	Culicoides imicola	SPECIES	41.27000	-8.00000	Ref.11
1062	Diptera	Ceratopogonidae	Culicoides	Culicoides imicola	SPECIES	41.28300	-7.48300	Ref.11
1063	Diptera	Ceratopogonidae	Culicoides	Culicoides imicola	SPECIES	41.32800	-6.72200	Ref.11
1064	Diptera	Ceratopogonidae	Culicoides	Culicoides imicola	SPECIES	41.36700	2.06700	Ref.11
1065	Diptera	Ceratopogonidae	Culicoides	Culicoides imicola	SPECIES	41.38000	-6.43000	Ref.11
1066	Diptera	Ceratopogonidae	Culicoides	Culicoides imicola	SPECIES	41.40000	1.92000	Ref.11

№	Order	Family	Genus	Species	Taxon Rank	Latitude	Longitude	GBIF_ID
1067	Diptera	Ceratopogonidae	Culicoides	Culicoides imicola	SPECIES	41.48300	13.66700	Ref.11
1068	Diptera	Ceratopogonidae	Culicoides	Culicoides imicola	SPECIES	41.48300	9.01700	Ref.11
1069	Diptera	Ceratopogonidae	Culicoides	Culicoides imicola	SPECIES	41.50000	9.09000	Ref.11
1070	Diptera	Ceratopogonidae	Culicoides	Culicoides imicola	SPECIES	41.50000	1.76000	Ref.11
1071	Diptera	Ceratopogonidae	Culicoides	Culicoides imicola	SPECIES	41.51000	-7.17000	Ref.11
1072	Diptera	Ceratopogonidae	Culicoides	Culicoides imicola	SPECIES	41.51700	9.26700	Ref.11
1073	Diptera	Ceratopogonidae	Culicoides	Culicoides imicola	SPECIES	41.51700	2.10000	Ref.11
1074	Diptera	Ceratopogonidae	Culicoides	Culicoides imicola	SPECIES	41.58300	2.41700	Ref.11
1075	Diptera	Ceratopogonidae	Culicoides	Culicoides imicola	SPECIES	41.59000	9.25000	Ref.11
1076	Diptera	Ceratopogonidae	Culicoides	Culicoides imicola	SPECIES	41.64000	2.55000	Ref.11
1077	Diptera	Ceratopogonidae	Culicoides	Culicoides imicola	SPECIES	41.64400	-6.82500	Ref.11
1078	Diptera	Ceratopogonidae	Culicoides	Culicoides imicola	SPECIES	41.77000	2.64000	Ref.11
1079	Diptera	Ceratopogonidae	Culicoides	Culicoides imicola	SPECIES	41.84400	2.85000	Ref.11
1080	Diptera	Ceratopogonidae	Culicoides	Culicoides imicola	SPECIES	41.90000	2.69000	Ref.11
1081	Diptera	Ceratopogonidae	Culicoides	Culicoides imicola	SPECIES	41.92000	-8.57000	Ref.11
1082	Diptera	Ceratopogonidae	Culicoides	Culicoides imicola	SPECIES	41.93300	12.16700	Ref.11
1083	Diptera	Ceratopogonidae	Culicoides	Culicoides imicola	SPECIES	41.94000	8.82000	Ref.11
1084	Diptera	Ceratopogonidae	Culicoides	Culicoides imicola	SPECIES	42.20000	12.60000	Ref.11
1085	Diptera	Ceratopogonidae	Culicoides	Culicoides imicola	SPECIES	42.21000	0.99000	Ref.11
1086	Diptera	Ceratopogonidae	Culicoides	Culicoides imicola	SPECIES	42.26700	3.03300	Ref.11
1087	Diptera	Ceratopogonidae	Culicoides	Culicoides imicola	SPECIES	42.29000	9.54000	Ref.11
1088	Diptera	Ceratopogonidae	Culicoides	Culicoides imicola	SPECIES	42.31000	2.80000	Ref.11
1089	Diptera	Ceratopogonidae	Culicoides	Culicoides imicola	SPECIES	42.44000	2.81000	Ref.11
1090	Diptera	Ceratopogonidae	Culicoides	Culicoides imicola	SPECIES	42.47000	2.75000	Ref.11
1091	Diptera	Ceratopogonidae	Culicoides	Culicoides imicola	SPECIES	42.47000	3.12000	Ref.11
1092	Diptera	Ceratopogonidae	Culicoides	Culicoides imicola	SPECIES	42.47000	9.12000	Ref.11
1093	Diptera	Ceratopogonidae	Culicoides	Culicoides imicola	SPECIES	42.48000	2.87000	Ref.11
1094	Diptera	Ceratopogonidae	Culicoides	Culicoides imicola	SPECIES	42.51000	2.69000	Ref.11
1095	Diptera	Ceratopogonidae	Culicoides	Culicoides imicola	SPECIES	42.51000	3.00000	Ref.11
1096	Diptera	Ceratopogonidae	Culicoides	Culicoides imicola	SPECIES	42.51000	2.93000	Ref.11
1097	Diptera	Ceratopogonidae	Culicoides	Culicoides imicola	SPECIES	42.53000	3.06000	Ref.11
1098	Diptera	Ceratopogonidae	Culicoides	Culicoides imicola	SPECIES	42.53000	2.76000	Ref.11
1099	Diptera	Ceratopogonidae	Culicoides	Culicoides imicola	SPECIES	42.54000	8.76000	Ref.11
1100	Diptera	Ceratopogonidae	Culicoides	Culicoides imicola	SPECIES	42.57000	3.03000	Ref.11
1101	Diptera	Ceratopogonidae	Culicoides	Culicoides imicola	SPECIES	42.61700	11.21700	Ref.11
1102	Diptera	Ceratopogonidae	Culicoides	Culicoides imicola	SPECIES	42.75000	11.13300	Ref.11
1103	Diptera	Ceratopogonidae	Culicoides	Culicoides imicola	SPECIES	42.76000	3.02000	Ref.11
1104	Diptera	Ceratopogonidae	Culicoides	Culicoides imicola	SPECIES	42.78000	1.72000	Ref.11
1105	Diptera	Ceratopogonidae	Culicoides	Culicoides imicola	SPECIES	42.78300	10.35000	Ref.11
1106	Diptera	Ceratopogonidae	Culicoides	Culicoides imicola	SPECIES	42.85000	9.43000	Ref.11
1107	Diptera	Ceratopogonidae	Culicoides	Culicoides imicola	SPECIES	43.03000	1.31000	Ref.11

№	Order	Family	Genus	Species	Taxon Rank	Latitude	Longitude	GBIF_ID
1108	Diptera	Ceratopogonidae	Culicoides	Culicoides imicola	SPECIES	43.20000	6.40000	Ref.11
1109	Diptera	Ceratopogonidae	Culicoides	Culicoides imicola	SPECIES	43.27800	-1.84800	Ref.11
1110	Diptera	Ceratopogonidae	Culicoides	Culicoides imicola	SPECIES	43.49000	6.64000	Ref.11
1111	Diptera	Ceratopogonidae	Culicoides	Culicoides imicola	SPECIES	43.56700	10.88300	Ref.11
1112	Diptera	Ceratopogonidae	Culicoides	Culicoides imicola	SPECIES	44.06000	10.06000	Ref.11
1113	Diptera	Ceratopogonidae	Culicoides	Culicoides imicola	SPECIES	46.00000	8.53300	Ref.11
1114	Diptera	Ceratopogonidae	Culicoides	Culicoides imicola	SPECIES	22.64556	113.36374	Ref.12
1115	Diptera	Ceratopogonidae	Culicoides	Culicoides imicola	SPECIES	23.24000	102.81000	Ref.13

№	Order	Family	Genus	Species	Taxon Rank	Latitude	Longitude	GBIF_ID
1	Diptera	Ceratopogonidae	Culicoides	Culicoides variipennis	SPECIES	18.24000	-99.66111	1894349604
2	Diptera	Ceratopogonidae	Culicoides	Culicoides variipennis	SPECIES	23.23611	-106.41528	1894349601
3	Diptera	Ceratopogonidae	Culicoides	Culicoides variipennis	SPECIES	26.41167	-111.66806	1894349560
4	Diptera	Ceratopogonidae	Culicoides	Culicoides variipennis	SPECIES	26.50278	-100.18417	1894349471
5	Diptera	Ceratopogonidae	Culicoides	Culicoides variipennis	SPECIES	27.02000	74.21000	3348350616
6	Diptera	Ceratopogonidae	Culicoides	Culicoides variipennis	SPECIES	29.09889	-110.95417	1894349541
7	Diptera	Ceratopogonidae	Culicoides	Culicoides variipennis	SPECIES	30.45000	-91.42000	1322540049
8	Diptera	Ceratopogonidae	Culicoides	Culicoides variipennis	SPECIES	30.45056	-91.15444	890659249
9	Diptera	Ceratopogonidae	Culicoides	Culicoides variipennis	SPECIES	30.81950	-90.97250	3443537263
10	Diptera	Ceratopogonidae	Culicoides	Culicoides variipennis	SPECIES	30.82000	-90.97000	3351855845
11	Diptera	Ceratopogonidae	Culicoides	Culicoides variipennis	SPECIES	30.89404	-102.84549	3049097773
12	Diptera	Ceratopogonidae	Culicoides	Culicoides variipennis	SPECIES	31.89810	-110.21800	1322027599
13	Diptera	Ceratopogonidae	Culicoides	Culicoides variipennis	SPECIES	32.00095	-106.59931	1933461705
14	Diptera	Ceratopogonidae	Culicoides	Culicoides variipennis	SPECIES	32.31222	-106.77780	1092820154
15	Diptera	Ceratopogonidae	Culicoides	Culicoides variipennis	SPECIES	32.42556	-106.59610	1092820352
16	Diptera	Ceratopogonidae	Culicoides	Culicoides variipennis	SPECIES	34.16120	-119.20361	890659202
17	Diptera	Ceratopogonidae	Culicoides	Culicoides variipennis	SPECIES	34.75313	-98.66910	890659285
18	Diptera	Ceratopogonidae	Culicoides	Culicoides variipennis	SPECIES	37.55380	-77.46030	1322176713
19	Diptera	Ceratopogonidae	Culicoides	Culicoides variipennis	SPECIES	38.98530	-122.67300	1322916909
20	Diptera	Ceratopogonidae	Culicoides	Culicoides variipennis	SPECIES	38.98833	-122.67667	890659209
21	Diptera	Ceratopogonidae	Culicoides	Culicoides variipennis	SPECIES	39.47360	-118.77640	1092820194
22	Diptera	Ceratopogonidae	Culicoides	Culicoides variipennis	SPECIES	39.80276	-105.08748	2422212194
23	Diptera	Ceratopogonidae	Culicoides	Culicoides variipennis	SPECIES	40.58528	-105.08390	1092820514
24	Diptera	Ceratopogonidae	Culicoides	Culicoides variipennis	SPECIES	41.30969	-105.58424	2422208195
25	Diptera	Ceratopogonidae	Culicoides	Culicoides variipennis	SPECIES	43.05000	-89.47000	3328466446
26	Diptera	Ceratopogonidae	Culicoides	Culicoides variipennis	SPECIES	43.07000	-89.40000	3328466451
27	Diptera	Ceratopogonidae	Culicoides	Culicoides variipennis	SPECIES	43.13000	-90.71000	3328466444
28	Diptera	Ceratopogonidae	Culicoides	Culicoides variipennis	SPECIES	43.20000	-90.60000	3328466462
29	Diptera	Ceratopogonidae	Culicoides	Culicoides variipennis	SPECIES	43.61558	-84.24721	3385745633
30	Diptera	Ceratopogonidae	Culicoides	Culicoides variipennis	SPECIES	45.38000	-91.35000	3328466304
31	Diptera	Ceratopogonidae	Culicoides	Culicoides variipennis	SPECIES	45.40000	-91.20000	3328466296
32	Diptera	Ceratopogonidae	Culicoides	Culicoides variipennis	SPECIES	45.40000	-91.35000	3328466306
33	Diptera	Ceratopogonidae	Culicoides	Culicoides variipennis	SPECIES	45.40000	-91.33000	3328466314
34	Diptera	Ceratopogonidae	Culicoides	Culicoides variipennis	SPECIES	45.49000	-91.29000	3328466313
35	Diptera	Ceratopogonidae	Culicoides	Culicoides variipennis	SPECIES	45.84000	-91.88000	3328466452
36	Diptera	Ceratopogonidae	Culicoides	Culicoides variipennis	SPECIES	45.86000	-91.63000	3328466454

№	Order	Family	Genus	Species	Taxon Rank	Latitude	Longitude	GBIF_ID
1	Diptera	Culicidae	Aedes	Aedes aegypti	SPECIES	-38.95000	-68.05000	1264886861
2	Diptera	Culicidae	Aedes	Aedes aegypti	SPECIES	-38.89195	-62.06920	3044726895
3	Diptera	Culicidae	Aedes	Aedes aegypti	SPECIES	-38.88958	-62.07213	3061796489
4	Diptera	Culicidae	Aedes	Aedes aegypti	SPECIES	-38.88954	-62.07232	3058954510
5	Diptera	Culicidae	Aedes	Aedes aegypti	SPECIES	-38.59268	145.59459	1633782867
6	Diptera	Culicidae	Aedes	Aedes aegypti	SPECIES	-37.67000	144.84000	3350911110
7	Diptera	Culicidae	Aedes	Aedes aegypti	SPECIES	-36.63427	-64.29105	3705586563
8	Diptera	Culicidae	Aedes	Aedes aegypti	SPECIES	-36.32000	-57.68000	1264886606
9	Diptera	Culicidae	Aedes	Aedes aegypti	SPECIES	-36.09000	-57.81000	1264886633
10	Diptera	Culicidae	Aedes	Aedes aegypti	SPECIES	-35.91761	-64.29273	2611135881
11	Diptera	Culicidae	Aedes	Aedes aegypti	SPECIES	-35.88000	-57.90000	1264886649
12	Diptera	Culicidae	Aedes	Aedes aegypti	SPECIES	-35.65196	-63.75768	3398862794
13	Diptera	Culicidae	Aedes	Aedes aegypti	SPECIES	-35.58000	-58.01000	1264886603
14	Diptera	Culicidae	Aedes	Aedes aegypti	SPECIES	-35.48932	-62.97467	4096926470
15	Diptera	Culicidae	Aedes	Aedes aegypti	SPECIES	-35.48930	-62.97462	4028973015
16	Diptera	Culicidae	Aedes	Aedes aegypti	SPECIES	-35.48930	-62.97455	4034618136
17	Diptera	Culicidae	Aedes	Aedes aegypti	SPECIES	-35.48929	-62.97455	4034866140
18	Diptera	Culicidae	Aedes	Aedes aegypti	SPECIES	-35.48928	-62.97453	4028829114
19	Diptera	Culicidae	Aedes	Aedes aegypti	SPECIES	-35.48925	-62.97466	3456210306
20	Diptera	Culicidae	Aedes	Aedes aegypti	SPECIES	-35.26667	149.10000	2446096038
21	Diptera	Culicidae	Aedes	Aedes aegypti	SPECIES	-35.22722	149.03195	2432640374
22	Diptera	Culicidae	Aedes	Aedes aegypti	SPECIES	-34.93657	-57.94215	4015016864
23	Diptera	Culicidae	Aedes	Aedes aegypti	SPECIES	-34.93000	-57.96000	1264889152
24	Diptera	Culicidae	Aedes	Aedes aegypti	SPECIES	-34.92897	-57.99007	3712702788
25	Diptera	Culicidae	Aedes	Aedes aegypti	SPECIES	-34.92000	-57.95000	1264889409
26	Diptera	Culicidae	Aedes	Aedes aegypti	SPECIES	-34.91652	-57.92446	4034824111
27	Diptera	Culicidae	Aedes	Aedes aegypti	SPECIES	-34.90858	-57.94795	2611100554
28	Diptera	Culicidae	Aedes	Aedes aegypti	SPECIES	-34.90840	-57.94785	2596273092
29	Diptera	Culicidae	Aedes	Aedes aegypti	SPECIES	-34.90795	-57.94832	3039229427
30	Diptera	Culicidae	Aedes	Aedes aegypti	SPECIES	-34.90667	-56.14543	3455274313
31	Diptera	Culicidae	Aedes	Aedes aegypti	SPECIES	-34.90000	-56.17000	1264890744
32	Diptera	Culicidae	Aedes	Aedes aegypti	SPECIES	-34.88809	-56.19036	4009920100
33	Diptera	Culicidae	Aedes	Aedes aegypti	SPECIES	-34.88000	-56.15000	1264890703
34	Diptera	Culicidae	Aedes	Aedes aegypti	SPECIES	-34.87000	-58.07000	1264886349
35	Diptera	Culicidae	Aedes	Aedes aegypti	SPECIES	-34.85808	-56.15691	1990583479
36	Diptera	Culicidae	Aedes	Aedes aegypti	SPECIES	-34.84924	-56.19724	3912093396
37	Diptera	Culicidae	Aedes	Aedes aegypti	SPECIES	-34.84919	-56.19712	3912250372
38	Diptera	Culicidae	Aedes	Aedes aegypti	SPECIES	-34.79038	-55.93221	3759835778
39	Diptera	Culicidae	Aedes	Aedes aegypti	SPECIES	-34.78658	-58.37973	2609161735
40	Diptera	Culicidae	Aedes	Aedes aegypti	SPECIES	-34.77457	-58.19535	3079652531
41	Diptera	Culicidae	Aedes	Aedes aegypti	SPECIES	-34.76842	-58.35501	3383886590

№	Order	Family	Genus	Species	Taxon Rank	Latitude	Longitude	GBIF_ID
42	Diptera	Culicidae	Aedes	Aedes aegypti	SPECIES	-34.76000	-58.43000	1264887618
43	Diptera	Culicidae	Aedes	Aedes aegypti	SPECIES	-34.70087	-58.52344	2580250672
44	Diptera	Culicidae	Aedes	Aedes aegypti	SPECIES	-34.70002	-58.25616	3383872573
45	Diptera	Culicidae	Aedes	Aedes aegypti	SPECIES	-34.70000	-58.39000	1264886258
46	Diptera	Culicidae	Aedes	Aedes aegypti	SPECIES	-34.68113	-58.44516	3712546608
47	Diptera	Culicidae	Aedes	Aedes aegypti	SPECIES	-34.67000	-58.50000	1264886596
48	Diptera	Culicidae	Aedes	Aedes aegypti	SPECIES	-34.66000	-58.68000	1264886223
49	Diptera	Culicidae	Aedes	Aedes aegypti	SPECIES	-34.63912	-58.49308	3070504752
50	Diptera	Culicidae	Aedes	Aedes aegypti	SPECIES	-34.63000	-58.46000	1264889562
51	Diptera	Culicidae	Aedes	Aedes aegypti	SPECIES	-34.62543	-58.52347	4126858440
52	Diptera	Culicidae	Aedes	Aedes aegypti	SPECIES	-34.62231	-58.52911	4028959731
53	Diptera	Culicidae	Aedes	Aedes aegypti	SPECIES	-34.61922	-58.41736	4075939491
54	Diptera	Culicidae	Aedes	Aedes aegypti	SPECIES	-34.61823	-58.41928	2596372468
55	Diptera	Culicidae	Aedes	Aedes aegypti	SPECIES	-34.61813	-58.41735	2626370821
56	Diptera	Culicidae	Aedes	Aedes aegypti	SPECIES	-34.61590	-58.41782	2579269424
57	Diptera	Culicidae	Aedes	Aedes aegypti	SPECIES	-34.61000	-58.38000	1264888367
58	Diptera	Culicidae	Aedes	Aedes aegypti	SPECIES	-34.61000	-58.37000	1264890691
59	Diptera	Culicidae	Aedes	Aedes aegypti	SPECIES	-34.60948	-58.56346	4121411526
60	Diptera	Culicidae	Aedes	Aedes aegypti	SPECIES	-34.60142	-58.44336	4111822972
61	Diptera	Culicidae	Aedes	Aedes aegypti	SPECIES	-34.60000	-58.42000	1264886609
62	Diptera	Culicidae	Aedes	Aedes aegypti	SPECIES	-34.60000	-58.38000	1264888402
63	Diptera	Culicidae	Aedes	Aedes aegypti	SPECIES	-34.59972	-58.38194	2237551241
64	Diptera	Culicidae	Aedes	Aedes aegypti	SPECIES	-34.59484	-58.42214	2619942599
65	Diptera	Culicidae	Aedes	Aedes aegypti	SPECIES	-34.59000	-58.47000	1264886281
66	Diptera	Culicidae	Aedes	Aedes aegypti	SPECIES	-34.59000	-58.46000	1264890262
67	Diptera	Culicidae	Aedes	Aedes aegypti	SPECIES	-34.58792	-58.42976	4116647606
68	Diptera	Culicidae	Aedes	Aedes aegypti	SPECIES	-34.58000	-58.50000	1264886422
69	Diptera	Culicidae	Aedes	Aedes aegypti	SPECIES	-34.57302	-58.45964	4091686459
70	Diptera	Culicidae	Aedes	Aedes aegypti	SPECIES	-34.56000	-58.46000	1264886622
71	Diptera	Culicidae	Aedes	Aedes aegypti	SPECIES	-34.55578	-58.43532	4102729862
72	Diptera	Culicidae	Aedes	Aedes aegypti	SPECIES	-34.55463	-58.55268	3466514512
73	Diptera	Culicidae	Aedes	Aedes aegypti	SPECIES	-34.55363	-58.47539	3112717212
74	Diptera	Culicidae	Aedes	Aedes aegypti	SPECIES	-34.54914	-58.55882	3079683323
75	Diptera	Culicidae	Aedes	Aedes aegypti	SPECIES	-34.54000	-58.69000	1264886409
76	Diptera	Culicidae	Aedes	Aedes aegypti	SPECIES	-34.48890	-58.51300	4054916595
77	Diptera	Culicidae	Aedes	Aedes aegypti	SPECIES	-34.37173	-58.68809	3067892612
78	Diptera	Culicidae	Aedes	Aedes aegypti	SPECIES	-34.27000	-60.31000	1264890964
79	Diptera	Culicidae	Aedes	Aedes aegypti	SPECIES	-34.21621	-58.90061	177059929
80	Diptera	Culicidae	Aedes	Aedes aegypti	SPECIES	-34.10757	-59.06164	3301962919
81	Diptera	Culicidae	Aedes	Aedes aegypti	SPECIES	-34.09000	-59.04000	1264888385
82	Diptera	Culicidae	Aedes	Aedes aegypti	SPECIES	-34.06963	23.02396	2609282802

№	Order	Family	Genus	Species	Taxon Rank	Latitude	Longitude	GBIF_ID
83	Diptera	Culicidae	Aedes	Aedes aegypti	SPECIES	-34.06961	23.02385	3391385456
84	Diptera	Culicidae	Aedes	Aedes aegypti	SPECIES	-34.06960	23.02389	2609289572
85	Diptera	Culicidae	Aedes	Aedes aegypti	SPECIES	-34.06959	23.02389	2611128340
86	Diptera	Culicidae	Aedes	Aedes aegypti	SPECIES	-34.03509	23.04647	2611063258
87	Diptera	Culicidae	Aedes	Aedes aegypti	SPECIES	-33.97164	22.61932	3109232387
88	Diptera	Culicidae	Aedes	Aedes aegypti	SPECIES	-33.96639	22.09083	2611394652
89	Diptera	Culicidae	Aedes	Aedes aegypti	SPECIES	-33.81000	151.00000	1264891334
90	Diptera	Culicidae	Aedes	Aedes aegypti	SPECIES	-33.79471	151.26088	1100304672
91	Diptera	Culicidae	Aedes	Aedes aegypti	SPECIES	-33.69460	25.81807	3860302723
92	Diptera	Culicidae	Aedes	Aedes aegypti	SPECIES	-33.44166	-60.79417	3456085526
93	Diptera	Culicidae	Aedes	Aedes aegypti	SPECIES	-33.38644	18.91287	4116338506
94	Diptera	Culicidae	Aedes	Aedes aegypti	SPECIES	-33.31593	26.53391	2597855095
95	Diptera	Culicidae	Aedes	Aedes aegypti	SPECIES	-33.28333	151.41670	1633667655
96	Diptera	Culicidae	Aedes	Aedes aegypti	SPECIES	-33.28333	151.41670	177194651
97	Diptera	Culicidae	Aedes	Aedes aegypti	SPECIES	-33.25000	-58.02000	1264886611
98	Diptera	Culicidae	Aedes	Aedes aegypti	SPECIES	-33.25000	-58.03000	1264887451
99	Diptera	Culicidae	Aedes	Aedes aegypti	SPECIES	-33.23000	-54.38000	1264887471
100	Diptera	Culicidae	Aedes	Aedes aegypti	SPECIES	-33.13000	-58.30000	1264887467
101	Diptera	Culicidae	Aedes	Aedes aegypti	SPECIES	-33.12000	-58.30000	1264886985
102	Diptera	Culicidae	Aedes	Aedes aegypti	SPECIES	-33.09940	-68.97767	2596289949
103	Diptera	Culicidae	Aedes	Aedes aegypti	SPECIES	-33.05682	-68.86699	3712464536
104	Diptera	Culicidae	Aedes	Aedes aegypti	SPECIES	-33.05332	-61.15782	3338109058
105	Diptera	Culicidae	Aedes	Aedes aegypti	SPECIES	-33.01766	-60.74917	3112357794
106	Diptera	Culicidae	Aedes	Aedes aegypti	SPECIES	-33.01000	-58.51000	1264887446
107	Diptera	Culicidae	Aedes	Aedes aegypti	SPECIES	-32.91130	-68.87064	3743118608
108	Diptera	Culicidae	Aedes	Aedes aegypti	SPECIES	-32.90875	-68.84809	4062827947
109	Diptera	Culicidae	Aedes	Aedes aegypti	SPECIES	-32.71667	149.05000	177194644
110	Diptera	Culicidae	Aedes	Aedes aegypti	SPECIES	-32.71667	149.05000	1633667569
111	Diptera	Culicidae	Aedes	Aedes aegypti	SPECIES	-32.60019	27.01630	226099163
112	Diptera	Culicidae	Aedes	Aedes aegypti	SPECIES	-32.43000	-63.25000	1264887916
113	Diptera	Culicidae	Aedes	Aedes aegypti	SPECIES	-32.41000	-63.25000	1264887903
114	Diptera	Culicidae	Aedes	Aedes aegypti	SPECIES	-32.41000	-63.26000	1264888406
115	Diptera	Culicidae	Aedes	Aedes aegypti	SPECIES	-32.40000	-63.23000	1264886636
116	Diptera	Culicidae	Aedes	Aedes aegypti	SPECIES	-32.40000	-63.26000	1264888391
117	Diptera	Culicidae	Aedes	Aedes aegypti	SPECIES	-32.38334	148.50000	177194643
118	Diptera	Culicidae	Aedes	Aedes aegypti	SPECIES	-32.38334	148.50000	1633667634
119	Diptera	Culicidae	Aedes	Aedes aegypti	SPECIES	-32.31129	-58.07577	3892590376
120	Diptera	Culicidae	Aedes	Aedes aegypti	SPECIES	-32.21000	-52.38000	1264871971
121	Diptera	Culicidae	Aedes	Aedes aegypti	SPECIES	-31.86000	115.75000	1264891350
122	Diptera	Culicidae	Aedes	Aedes aegypti	SPECIES	-31.75235	-60.49313	3079487742
123	Diptera	Culicidae	Aedes	Aedes aegypti	SPECIES	-31.75230	-60.49328	3079572702

№	Order	Family	Genus	Species	Taxon Rank	Latitude	Longitude	GBIF_ID
124	Diptera	Culicidae	Aedes	Aedes aegypti	SPECIES	-31.74706	-60.53204	3802365119
125	Diptera	Culicidae	Aedes	Aedes aegypti	SPECIES	-31.74000	-60.51000	1264887456
126	Diptera	Culicidae	Aedes	Aedes aegypti	SPECIES	-31.73106	-60.53801	3466419995
127	Diptera	Culicidae	Aedes	Aedes aegypti	SPECIES	-31.73000	-60.50000	1264886585
128	Diptera	Culicidae	Aedes	Aedes aegypti	SPECIES	-31.66813	-60.76438	2988336442
129	Diptera	Culicidae	Aedes	Aedes aegypti	SPECIES	-31.66166	-60.76081	3456379352
130	Diptera	Culicidae	Aedes	Aedes aegypti	SPECIES	-31.66154	-60.76074	3760058964
131	Diptera	Culicidae	Aedes	Aedes aegypti	SPECIES	-31.63217	-60.70694	4111683842
132	Diptera	Culicidae	Aedes	Aedes aegypti	SPECIES	-31.63216	-60.70690	4062866149
133	Diptera	Culicidae	Aedes	Aedes aegypti	SPECIES	-31.58242	-60.70670	4075958495
134	Diptera	Culicidae	Aedes	Aedes aegypti	SPECIES	-31.56000	-52.31000	1264871964
135	Diptera	Culicidae	Aedes	Aedes aegypti	SPECIES	-31.53265	-68.56681	2236203167
136	Diptera	Culicidae	Aedes	Aedes aegypti	SPECIES	-31.51424	-68.56966	2576444211
137	Diptera	Culicidae	Aedes	Aedes aegypti	SPECIES	-31.47000	-64.13000	1264887910
138	Diptera	Culicidae	Aedes	Aedes aegypti	SPECIES	-31.46000	-64.21000	1264886888
139	Diptera	Culicidae	Aedes	Aedes aegypti	SPECIES	-31.45000	-64.11000	1264886870
140	Diptera	Culicidae	Aedes	Aedes aegypti	SPECIES	-31.43464	-64.19305	4022175063
141	Diptera	Culicidae	Aedes	Aedes aegypti	SPECIES	-31.42000	-64.20000	1264887921
142	Diptera	Culicidae	Aedes	Aedes aegypti	SPECIES	-31.42000	-64.18000	1264888352
143	Diptera	Culicidae	Aedes	Aedes aegypti	SPECIES	-31.41000	-64.19000	1264887945
144	Diptera	Culicidae	Aedes	Aedes aegypti	SPECIES	-31.40000	-64.27000	1264886857
145	Diptera	Culicidae	Aedes	Aedes aegypti	SPECIES	-31.40000	-64.18000	1264886977
146	Diptera	Culicidae	Aedes	Aedes aegypti	SPECIES	-31.40000	-64.17000	1264887050
147	Diptera	Culicidae	Aedes	Aedes aegypti	SPECIES	-31.40000	-64.19000	1264890632
148	Diptera	Culicidae	Aedes	Aedes aegypti	SPECIES	-31.39815	-64.18877	3499518935
149	Diptera	Culicidae	Aedes	Aedes aegypti	SPECIES	-31.39811	-64.18897	2005306926
150	Diptera	Culicidae	Aedes	Aedes aegypti	SPECIES	-31.39000	-64.13000	1264886886
151	Diptera	Culicidae	Aedes	Aedes aegypti	SPECIES	-31.39000	-57.96000	1264887449
152	Diptera	Culicidae	Aedes	Aedes aegypti	SPECIES	-31.38000	-64.17000	1264886981
153	Diptera	Culicidae	Aedes	Aedes aegypti	SPECIES	-31.38000	-64.21000	1264887883
154	Diptera	Culicidae	Aedes	Aedes aegypti	SPECIES	-31.38000	-64.18000	1264887941
155	Diptera	Culicidae	Aedes	Aedes aegypti	SPECIES	-31.37000	-64.20000	1264886840
156	Diptera	Culicidae	Aedes	Aedes aegypti	SPECIES	-31.36791	-64.28606	4075621152
157	Diptera	Culicidae	Aedes	Aedes aegypti	SPECIES	-31.36771	-64.28588	4075732188
158	Diptera	Culicidae	Aedes	Aedes aegypti	SPECIES	-31.36000	-64.24000	1264886869
159	Diptera	Culicidae	Aedes	Aedes aegypti	SPECIES	-31.36000	-64.16000	1264886880
160	Diptera	Culicidae	Aedes	Aedes aegypti	SPECIES	-31.35724	-64.22281	2557731470
161	Diptera	Culicidae	Aedes	Aedes aegypti	SPECIES	-31.33000	-66.58000	1264886996
162	Diptera	Culicidae	Aedes	Aedes aegypti	SPECIES	-31.32000	-64.17000	1264887860
163	Diptera	Culicidae	Aedes	Aedes aegypti	SPECIES	-31.27000	-64.17000	1264887897
164	Diptera	Culicidae	Aedes	Aedes aegypti	SPECIES	-31.25000	-61.48000	1264887007

№	Order	Family	Genus	Species	Taxon Rank	Latitude	Longitude	GBIF_ID
165	Diptera	Culicidae	Aedes	Aedes aegypti	SPECIES	-31.25000	-61.49000	1264887026
166	Diptera	Culicidae	Aedes	Aedes aegypti	SPECIES	-31.02000	30.25000	1264889069
167	Diptera	Culicidae	Aedes	Aedes aegypti	SPECIES	-31.00000	30.27000	1264889063
168	Diptera	Culicidae	Aedes	Aedes aegypti	SPECIES	-30.98000	-64.09000	1264887922
169	Diptera	Culicidae	Aedes	Aedes aegypti	SPECIES	-30.80000	-55.63000	1264871977
170	Diptera	Culicidae	Aedes	Aedes aegypti	SPECIES	-30.77000	30.43000	1264889096
171	Diptera	Culicidae	Aedes	Aedes aegypti	SPECIES	-30.37000	-51.32000	1264871999
172	Diptera	Culicidae	Aedes	Aedes aegypti	SPECIES	-30.34000	-54.37000	1264871991
173	Diptera	Culicidae	Aedes	Aedes aegypti	SPECIES	-30.29000	-56.16000	1264871967
174	Diptera	Culicidae	Aedes	Aedes aegypti	SPECIES	-30.21000	-50.87000	1264871983
175	Diptera	Culicidae	Aedes	Aedes aegypti	SPECIES	-30.17000	-51.44000	1264871957
176	Diptera	Culicidae	Aedes	Aedes aegypti	SPECIES	-30.10000	-51.16000	1264872010
177	Diptera	Culicidae	Aedes	Aedes aegypti	SPECIES	-30.08333	145.95000	1633667623
178	Diptera	Culicidae	Aedes	Aedes aegypti	SPECIES	-30.08333	145.95000	177194601
179	Diptera	Culicidae	Aedes	Aedes aegypti	SPECIES	-30.04000	-51.22000	1264888333
180	Diptera	Culicidae	Aedes	Aedes aegypti	SPECIES	-30.03000	-50.23000	1264871990
181	Diptera	Culicidae	Aedes	Aedes aegypti	SPECIES	-30.03000	-51.23000	1264887954
182	Diptera	Culicidae	Aedes	Aedes aegypti	SPECIES	-30.03000	-51.20000	1264891322
183	Diptera	Culicidae	Aedes	Aedes aegypti	SPECIES	-30.01939	-51.17948	3090648870
184	Diptera	Culicidae	Aedes	Aedes aegypti	SPECIES	-30.00000	-51.06000	1264871955
185	Diptera	Culicidae	Aedes	Aedes aegypti	SPECIES	-29.98333	30.96667	1224613107
186	Diptera	Culicidae	Aedes	Aedes aegypti	SPECIES	-29.92000	-51.10000	1264871969
187	Diptera	Culicidae	Aedes	Aedes aegypti	SPECIES	-29.92000	-51.18000	1264871979
188	Diptera	Culicidae	Aedes	Aedes aegypti	SPECIES	-29.90000	30.98333	1224613076
189	Diptera	Culicidae	Aedes	Aedes aegypti	SPECIES	-29.89223	-50.26203	2626606314
190	Diptera	Culicidae	Aedes	Aedes aegypti	SPECIES	-29.89056	30.97778	1275275683
191	Diptera	Culicidae	Aedes	Aedes aegypti	SPECIES	-29.89000	-50.96000	1264871976
192	Diptera	Culicidae	Aedes	Aedes aegypti	SPECIES	-29.85417	31.05000	1275275821
193	Diptera	Culicidae	Aedes	Aedes aegypti	SPECIES	-29.84000	30.94000	1264889075
194	Diptera	Culicidae	Aedes	Aedes aegypti	SPECIES	-29.80000	-56.74000	1264871958
195	Diptera	Culicidae	Aedes	Aedes aegypti	SPECIES	-29.79552	31.03940	3773715720
196	Diptera	Culicidae	Aedes	Aedes aegypti	SPECIES	-29.76667	30.85000	1224613081
197	Diptera	Culicidae	Aedes	Aedes aegypti	SPECIES	-29.74000	-51.14000	1264871961
198	Diptera	Culicidae	Aedes	Aedes aegypti	SPECIES	-29.73000	-51.06000	1264871975
199	Diptera	Culicidae	Aedes	Aedes aegypti	SPECIES	-29.71000	-51.51000	1264871970
200	Diptera	Culicidae	Aedes	Aedes aegypti	SPECIES	-29.62000	-51.00000	1264871959
201	Diptera	Culicidae	Aedes	Aedes aegypti	SPECIES	-29.55710	30.36219	2456496122
202	Diptera	Culicidae	Aedes	Aedes aegypti	SPECIES	-29.55000	-52.20000	1264871950
203	Diptera	Culicidae	Aedes	Aedes aegypti	SPECIES	-29.53333	150.58333	1633663751
204	Diptera	Culicidae	Aedes	Aedes aegypti	SPECIES	-29.53333	150.58333	177194649
205	Diptera	Culicidae	Aedes	Aedes aegypti	SPECIES	-29.46000	-50.16000	1264871989

№	Order	Family	Genus	Species	Taxon Rank	Latitude	Longitude	GBIF_ID
206	Diptera	Culicidae	Aedes	Aedes aegypti	SPECIES	-29.45000	-55.13000	1264871946
207	Diptera	Culicidae	Aedes	Aedes aegypti	SPECIES	-29.42000	-66.85000	1264886618
208	Diptera	Culicidae	Aedes	Aedes aegypti	SPECIES	-29.41000	-66.86000	1264886995
209	Diptera	Culicidae	Aedes	Aedes aegypti	SPECIES	-29.19000	-51.38000	1264871962
210	Diptera	Culicidae	Aedes	Aedes aegypti	SPECIES	-29.12000	-59.66000	1264887013
211	Diptera	Culicidae	Aedes	Aedes aegypti	SPECIES	-29.11000	-51.54000	1264871968
212	Diptera	Culicidae	Aedes	Aedes aegypti	SPECIES	-29.10000	-54.75000	1264871978
213	Diptera	Culicidae	Aedes	Aedes aegypti	SPECIES	-29.10000	-59.65000	1264886988
214	Diptera	Culicidae	Aedes	Aedes aegypti	SPECIES	-29.09830	26.14358	3335097318
215	Diptera	Culicidae	Aedes	Aedes aegypti	SPECIES	-29.09000	26.15600	3441571434
216	Diptera	Culicidae	Aedes	Aedes aegypti	SPECIES	-29.08972	26.15639	3398822086
217	Diptera	Culicidae	Aedes	Aedes aegypti	SPECIES	-29.08000	-51.04000	1264871996
218	Diptera	Culicidae	Aedes	Aedes aegypti	SPECIES	-29.05000	-55.76000	1264871988
219	Diptera	Culicidae	Aedes	Aedes aegypti	SPECIES	-28.89000	-52.53000	1264872002
220	Diptera	Culicidae	Aedes	Aedes aegypti	SPECIES	-28.75000	31.89000	1264888980
221	Diptera	Culicidae	Aedes	Aedes aegypti	SPECIES	-28.72000	-53.52000	1264872024
222	Diptera	Culicidae	Aedes	Aedes aegypti	SPECIES	-28.71000	-55.81000	1264872016
223	Diptera	Culicidae	Aedes	Aedes aegypti	SPECIES	-28.65054	-53.59370	2641579808
224	Diptera	Culicidae	Aedes	Aedes aegypti	SPECIES	-28.65000	-54.99000	1264871992
225	Diptera	Culicidae	Aedes	Aedes aegypti	SPECIES	-28.58333	148.23334	1633667826
226	Diptera	Culicidae	Aedes	Aedes aegypti	SPECIES	-28.58333	148.23334	177194641
227	Diptera	Culicidae	Aedes	Aedes aegypti	SPECIES	-28.58000	-54.25000	1264872009
228	Diptera	Culicidae	Aedes	Aedes aegypti	SPECIES	-28.53000	-54.01000	1264871982
229	Diptera	Culicidae	Aedes	Aedes aegypti	SPECIES	-28.50000	153.50000	177194660
230	Diptera	Culicidae	Aedes	Aedes aegypti	SPECIES	-28.49000	-52.29000	1264872015
231	Diptera	Culicidae	Aedes	Aedes aegypti	SPECIES	-28.49000	-54.28000	1264872033
232	Diptera	Culicidae	Aedes	Aedes aegypti	SPECIES	-28.47000	-65.78000	1264888044
233	Diptera	Culicidae	Aedes	Aedes aegypti	SPECIES	-28.46000	-52.81000	1264871980
234	Diptera	Culicidae	Aedes	Aedes aegypti	SPECIES	-28.46000	-55.46000	1264871993
235	Diptera	Culicidae	Aedes	Aedes aegypti	SPECIES	-28.45000	-65.78000	1264886643
236	Diptera	Culicidae	Aedes	Aedes aegypti	SPECIES	-28.43016	26.22208	3335118343
237	Diptera	Culicidae	Aedes	Aedes aegypti	SPECIES	-28.41000	-54.07000	1264872004
238	Diptera	Culicidae	Aedes	Aedes aegypti	SPECIES	-28.39000	-54.87000	1264872000
239	Diptera	Culicidae	Aedes	Aedes aegypti	SPECIES	-28.39000	-53.91000	1264889468
240	Diptera	Culicidae	Aedes	Aedes aegypti	SPECIES	-28.38000	32.42000	1264889056
241	Diptera	Culicidae	Aedes	Aedes aegypti	SPECIES	-28.37620	32.41810	2813932445
242	Diptera	Culicidae	Aedes	Aedes aegypti	SPECIES	-28.37000	-51.92000	1264871981
243	Diptera	Culicidae	Aedes	Aedes aegypti	SPECIES	-28.37000	-50.93000	1264871987
244	Diptera	Culicidae	Aedes	Aedes aegypti	SPECIES	-28.30000	-53.92000	1264872001
245	Diptera	Culicidae	Aedes	Aedes aegypti	SPECIES	-28.28000	-52.87000	1264871994
246	Diptera	Culicidae	Aedes	Aedes aegypti	SPECIES	-28.27000	-52.45000	1264871984

№	Order	Family	Genus	Species	Taxon Rank	Latitude	Longitude	GBIF_ID
247	Diptera	Culicidae	Aedes	Aedes aegypti	SPECIES	-28.25000	-55.54000	1264871972
248	Diptera	Culicidae	Aedes	Aedes aegypti	SPECIES	-28.25000	-54.32000	1264871973
249	Diptera	Culicidae	Aedes	Aedes aegypti	SPECIES	-28.25000	-54.66000	1264872014
250	Diptera	Culicidae	Aedes	Aedes aegypti	SPECIES	-28.21000	-52.06000	1264871986
251	Diptera	Culicidae	Aedes	Aedes aegypti	SPECIES	-28.21000	-53.73000	1264871995
252	Diptera	Culicidae	Aedes	Aedes aegypti	SPECIES	-28.20000	-51.51000	1264872003
253	Diptera	Culicidae	Aedes	Aedes aegypti	SPECIES	-28.19000	-55.25000	1264872021
254	Diptera	Culicidae	Aedes	Aedes aegypti	SPECIES	-28.18000	-54.59000	1264872013
255	Diptera	Culicidae	Aedes	Aedes aegypti	SPECIES	-28.18000	-54.04000	1264872027
256	Diptera	Culicidae	Aedes	Aedes aegypti	SPECIES	-28.16000	-54.47000	1264872050
257	Diptera	Culicidae	Aedes	Aedes aegypti	SPECIES	-28.14000	-54.72000	1264872041
258	Diptera	Culicidae	Aedes	Aedes aegypti	SPECIES	-28.10000	-54.83000	1264872018
259	Diptera	Culicidae	Aedes	Aedes aegypti	SPECIES	-28.09000	-53.13000	1264872036
260	Diptera	Culicidae	Aedes	Aedes aegypti	SPECIES	-28.08000	-55.24000	1264872008
261	Diptera	Culicidae	Aedes	Aedes aegypti	SPECIES	-28.06000	-52.02000	1264872042
262	Diptera	Culicidae	Aedes	Aedes aegypti	SPECIES	-28.06000	-55.13000	1264872059
263	Diptera	Culicidae	Aedes	Aedes aegypti	SPECIES	-28.05971	-51.18665	3456525079
264	Diptera	Culicidae	Aedes	Aedes aegypti	SPECIES	-28.05966	-51.18669	3416105109
265	Diptera	Culicidae	Aedes	Aedes aegypti	SPECIES	-28.03000	-54.53000	1264872005
266	Diptera	Culicidae	Aedes	Aedes aegypti	SPECIES	-28.02000	-54.66000	1264872017
267	Diptera	Culicidae	Aedes	Aedes aegypti	SPECIES	-28.01000	-52.33000	1264872029
268	Diptera	Culicidae	Aedes	Aedes aegypti	SPECIES	-28.00000	32.10000	1264889064
269	Diptera	Culicidae	Aedes	Aedes aegypti	SPECIES	-27.99000	-54.29000	1264872025
270	Diptera	Culicidae	Aedes	Aedes aegypti	SPECIES	-27.98000	-53.89000	1264872006
271	Diptera	Culicidae	Aedes	Aedes aegypti	SPECIES	-27.98000	-54.97000	1264872012
272	Diptera	Culicidae	Aedes	Aedes aegypti	SPECIES	-27.96000	-54.83000	1264872026
273	Diptera	Culicidae	Aedes	Aedes aegypti	SPECIES	-27.94000	-55.16000	1264872007
274	Diptera	Culicidae	Aedes	Aedes aegypti	SPECIES	-27.94000	-53.36000	1264872019
275	Diptera	Culicidae	Aedes	Aedes aegypti	SPECIES	-27.93000	-54.72000	1264871998
276	Diptera	Culicidae	Aedes	Aedes aegypti	SPECIES	-27.92000	-54.03000	1264872039
277	Diptera	Culicidae	Aedes	Aedes aegypti	SPECIES	-27.91000	-54.19000	1264871997
278	Diptera	Culicidae	Aedes	Aedes aegypti	SPECIES	-27.90000	-53.73000	1264872011
279	Diptera	Culicidae	Aedes	Aedes aegypti	SPECIES	-27.89920	-48.47197	2882995365
280	Diptera	Culicidae	Aedes	Aedes aegypti	SPECIES	-27.88000	-55.13000	1264890387
281	Diptera	Culicidae	Aedes	Aedes aegypti	SPECIES	-27.87000	-55.13000	1264886864
282	Diptera	Culicidae	Aedes	Aedes aegypti	SPECIES	-27.87000	-54.45000	1264889436
283	Diptera	Culicidae	Aedes	Aedes aegypti	SPECIES	-27.85000	-52.17000	1264872020
284	Diptera	Culicidae	Aedes	Aedes aegypti	SPECIES	-27.85000	-54.95000	1264872028
285	Diptera	Culicidae	Aedes	Aedes aegypti	SPECIES	-27.85000	-52.47000	1264872048
286	Diptera	Culicidae	Aedes	Aedes aegypti	SPECIES	-27.84000	-54.52000	1264872038
287	Diptera	Culicidae	Aedes	Aedes aegypti	SPECIES	-27.81000	-53.65000	1264872051

№	Order	Family	Genus	Species	Taxon Rank	Latitude	Longitude	GBIF_ID
288	Diptera	Culicidae	Aedes	Aedes aegypti	SPECIES	-27.81000	-54.06000	1264872074
289	Diptera	Culicidae	Aedes	Aedes aegypti	SPECIES	-27.79000	-54.71000	1264872065
290	Diptera	Culicidae	Aedes	Aedes aegypti	SPECIES	-27.78000	-52.55000	1264872043
291	Diptera	Culicidae	Aedes	Aedes aegypti	SPECIES	-27.78000	-64.27000	1264887014
292	Diptera	Culicidae	Aedes	Aedes aegypti	SPECIES	-27.76000	-54.91000	1264872061
293	Diptera	Culicidae	Aedes	Aedes aegypti	SPECIES	-27.74000	-52.66000	1264872037
294	Diptera	Culicidae	Aedes	Aedes aegypti	SPECIES	-27.74000	-54.13000	1264872083
295	Diptera	Culicidae	Aedes	Aedes aegypti	SPECIES	-27.72000	-54.27000	1264872030
296	Diptera	Culicidae	Aedes	Aedes aegypti	SPECIES	-27.72000	-53.95000	1264872067
297	Diptera	Culicidae	Aedes	Aedes aegypti	SPECIES	-27.71250	32.25139	1275275832
298	Diptera	Culicidae	Aedes	Aedes aegypti	SPECIES	-27.69000	-54.56000	1264872040
299	Diptera	Culicidae	Aedes	Aedes aegypti	SPECIES	-27.69000	-53.01000	1264872052
300	Diptera	Culicidae	Aedes	Aedes aegypti	SPECIES	-27.68000	-54.10000	1264872049
301	Diptera	Culicidae	Aedes	Aedes aegypti	SPECIES	-27.65000	-54.78000	1264872032
302	Diptera	Culicidae	Aedes	Aedes aegypti	SPECIES	-27.64000	-53.96000	1264872034
303	Diptera	Culicidae	Aedes	Aedes aegypti	SPECIES	-27.63000	-54.43000	1264872044
304	Diptera	Culicidae	Aedes	Aedes aegypti	SPECIES	-27.63000	-52.25000	1264872054
305	Diptera	Culicidae	Aedes	Aedes aegypti	SPECIES	-27.59000	-53.99000	1264872023
306	Diptera	Culicidae	Aedes	Aedes aegypti	SPECIES	-27.59000	-54.30000	1264872031
307	Diptera	Culicidae	Aedes	Aedes aegypti	SPECIES	-27.58000	-54.66000	1264872064
308	Diptera	Culicidae	Aedes	Aedes aegypti	SPECIES	-27.54000	-54.53000	1264872022
309	Diptera	Culicidae	Aedes	Aedes aegypti	SPECIES	-27.53000	-52.90000	1264872035
310	Diptera	Culicidae	Aedes	Aedes aegypti	SPECIES	-27.50000	-53.38000	1264872045
311	Diptera	Culicidae	Aedes	Aedes aegypti	SPECIES	-27.50000	-59.00000	1264887468
312	Diptera	Culicidae	Aedes	Aedes aegypti	SPECIES	-27.49000	-54.35000	1264872053
313	Diptera	Culicidae	Aedes	Aedes aegypti	SPECIES	-27.48000	-54.13000	1264872072
314	Diptera	Culicidae	Aedes	Aedes aegypti	SPECIES	-27.47099	153.02427	1100304601
315	Diptera	Culicidae	Aedes	Aedes aegypti	SPECIES	-27.47000	-58.83000	1264886648
316	Diptera	Culicidae	Aedes	Aedes aegypti	SPECIES	-27.47000	-58.84000	1264887009
317	Diptera	Culicidae	Aedes	Aedes aegypti	SPECIES	-27.46667	153.01666	177194656
318	Diptera	Culicidae	Aedes	Aedes aegypti	SPECIES	-27.46667	153.01666	1633667696
319	Diptera	Culicidae	Aedes	Aedes aegypti	SPECIES	-27.46499	-58.82914	4067446351
320	Diptera	Culicidae	Aedes	Aedes aegypti	SPECIES	-27.46245	-58.82559	3759156093
321	Diptera	Culicidae	Aedes	Aedes aegypti	SPECIES	-27.46000	-51.91000	1264872062
322	Diptera	Culicidae	Aedes	Aedes aegypti	SPECIES	-27.46000	-58.99000	1264889996
323	Diptera	Culicidae	Aedes	Aedes aegypti	SPECIES	-27.45000	-58.99000	1264887000
324	Diptera	Culicidae	Aedes	Aedes aegypti	SPECIES	-27.43000	-53.93000	1264872079
325	Diptera	Culicidae	Aedes	Aedes aegypti	SPECIES	-27.41667	153.05000	177194659
326	Diptera	Culicidae	Aedes	Aedes aegypti	SPECIES	-27.41667	153.05000	1633663706
327	Diptera	Culicidae	Aedes	Aedes aegypti	SPECIES	-27.40000	-55.91000	1264890384
328	Diptera	Culicidae	Aedes	Aedes aegypti	SPECIES	-27.38000	-65.74000	1264887477

№	Order	Family	Genus	Species	Taxon Rank	Latitude	Longitude	GBIF_ID
329	Diptera	Culicidae	Aedes	Aedes aegypti	SPECIES	-27.37000	-55.88000	1264888373
330	Diptera	Culicidae	Aedes	Aedes aegypti	SPECIES	-27.36000	-53.77000	1264872089
331	Diptera	Culicidae	Aedes	Aedes aegypti	SPECIES	-27.36000	-52.59000	1264872099
332	Diptera	Culicidae	Aedes	Aedes aegypti	SPECIES	-27.36000	-55.90000	1264887432
333	Diptera	Culicidae	Aedes	Aedes aegypti	SPECIES	-27.35000	-55.85000	1264886607
334	Diptera	Culicidae	Aedes	Aedes aegypti	SPECIES	-27.34000	-65.66000	1264887514
335	Diptera	Culicidae	Aedes	Aedes aegypti	SPECIES	-27.32000	-53.37000	1264872069
336	Diptera	Culicidae	Aedes	Aedes aegypti	SPECIES	-27.26000	-53.24000	1264872085
337	Diptera	Culicidae	Aedes	Aedes aegypti	SPECIES	-27.25000	-53.47000	1264872063
338	Diptera	Culicidae	Aedes	Aedes aegypti	SPECIES	-27.20000	-53.06000	1264872104
339	Diptera	Culicidae	Aedes	Aedes aegypti	SPECIES	-27.17000	-53.40000	1264872092
340	Diptera	Culicidae	Aedes	Aedes aegypti	SPECIES	-27.11000	-52.66000	1264872057
341	Diptera	Culicidae	Aedes	Aedes aegypti	SPECIES	-27.08399	-48.91362	3008505711
342	Diptera	Culicidae	Aedes	Aedes aegypti	SPECIES	-27.04000	32.42000	3987361680
343	Diptera	Culicidae	Aedes	Aedes aegypti	SPECIES	-27.00000	-109.00000	3350763474
344	Diptera	Culicidae	Aedes	Aedes aegypti	SPECIES	-26.93000	-65.27000	1264886991
345	Diptera	Culicidae	Aedes	Aedes aegypti	SPECIES	-26.93000	-60.05000	1264889991
346	Diptera	Culicidae	Aedes	Aedes aegypti	SPECIES	-26.92000	-60.05000	1264886615
347	Diptera	Culicidae	Aedes	Aedes aegypti	SPECIES	-26.92000	32.25000	1264889092
348	Diptera	Culicidae	Aedes	Aedes aegypti	SPECIES	-26.85000	-65.17000	1264886586
349	Diptera	Culicidae	Aedes	Aedes aegypti	SPECIES	-26.83682	-65.23828	3496613045
350	Diptera	Culicidae	Aedes	Aedes aegypti	SPECIES	-26.81772	-65.21311	2611233755
351	Diptera	Culicidae	Aedes	Aedes aegypti	SPECIES	-26.81198	-65.30135	4075955554
352	Diptera	Culicidae	Aedes	Aedes aegypti	SPECIES	-26.81000	-65.22000	1264887006
353	Diptera	Culicidae	Aedes	Aedes aegypti	SPECIES	-26.81000	-55.03000	1264887051
354	Diptera	Culicidae	Aedes	Aedes aegypti	SPECIES	-26.80000	-65.22000	1264886632
355	Diptera	Culicidae	Aedes	Aedes aegypti	SPECIES	-26.79000	-60.44000	1264890037
356	Diptera	Culicidae	Aedes	Aedes aegypti	SPECIES	-26.73000	150.62000	1264886345
357	Diptera	Culicidae	Aedes	Aedes aegypti	SPECIES	-26.72000	-53.48000	1264872066
358	Diptera	Culicidae	Aedes	Aedes aegypti	SPECIES	-26.68333	153.06667	1633663708
359	Diptera	Culicidae	Aedes	Aedes aegypti	SPECIES	-26.68333	153.06667	177194540
360	Diptera	Culicidae	Aedes	Aedes aegypti	SPECIES	-26.58333	149.18333	1633663704
361	Diptera	Culicidae	Aedes	Aedes aegypti	SPECIES	-26.58333	149.18333	177194648
362	Diptera	Culicidae	Aedes	Aedes aegypti	SPECIES	-26.57000	148.78000	1264886370
363	Diptera	Culicidae	Aedes	Aedes aegypti	SPECIES	-26.57000	148.79000	1264887763
364	Diptera	Culicidae	Aedes	Aedes aegypti	SPECIES	-26.41000	-54.58000	1264890353
365	Diptera	Culicidae	Aedes	Aedes aegypti	SPECIES	-26.40000	146.23000	1264886368
366	Diptera	Culicidae	Aedes	Aedes aegypti	SPECIES	-26.25000	-53.09000	1264872075
367	Diptera	Culicidae	Aedes	Aedes aegypti	SPECIES	-26.25000	-53.53000	1264872076
368	Diptera	Culicidae	Aedes	Aedes aegypti	SPECIES	-26.25000	-53.65000	1264890378
369	Diptera	Culicidae	Aedes	Aedes aegypti	SPECIES	-26.19337	-58.18197	2634377919

№	Order	Family	Genus	Species	Taxon Rank	Latitude	Longitude	GBIF_ID
370	Diptera	Culicidae	Aedes	Aedes aegypti	SPECIES	-26.19000	152.07000	1264886359
371	Diptera	Culicidae	Aedes	Aedes aegypti	SPECIES	-26.15000	-53.57000	1264872056
372	Diptera	Culicidae	Aedes	Aedes aegypti	SPECIES	-26.15000	-52.67000	1264872058
373	Diptera	Culicidae	Aedes	Aedes aegypti	SPECIES	-26.10752	28.05670	3988679161
374	Diptera	Culicidae	Aedes	Aedes aegypti	SPECIES	-26.07000	-53.63000	1264872068
375	Diptera	Culicidae	Aedes	Aedes aegypti	SPECIES	-26.07000	-53.13000	1264872078
376	Diptera	Culicidae	Aedes	Aedes aegypti	SPECIES	-26.05858	27.97539	3465924773
377	Diptera	Culicidae	Aedes	Aedes aegypti	SPECIES	-26.04520	27.96007	3456060347
378	Diptera	Culicidae	Aedes	Aedes aegypti	SPECIES	-25.98000	27.55000	1264889112
379	Diptera	Culicidae	Aedes	Aedes aegypti	SPECIES	-25.97000	-53.69000	1264872055
380	Diptera	Culicidae	Aedes	Aedes aegypti	SPECIES	-25.97000	-54.58000	1264890489
381	Diptera	Culicidae	Aedes	Aedes aegypti	SPECIES	-25.97000	-54.59000	1264890520
382	Diptera	Culicidae	Aedes	Aedes aegypti	SPECIES	-25.96000	-54.59000	1264887075
383	Diptera	Culicidae	Aedes	Aedes aegypti	SPECIES	-25.92000	-54.58000	1264886827
384	Diptera	Culicidae	Aedes	Aedes aegypti	SPECIES	-25.92000	-54.59000	1264887015
385	Diptera	Culicidae	Aedes	Aedes aegypti	SPECIES	-25.92000	-54.60000	1264890377
386	Diptera	Culicidae	Aedes	Aedes aegypti	SPECIES	-25.91000	-53.48000	1264872046
387	Diptera	Culicidae	Aedes	Aedes aegypti	SPECIES	-25.88000	-53.64000	1264872088
388	Diptera	Culicidae	Aedes	Aedes aegypti	SPECIES	-25.85000	-57.90000	1264889299
389	Diptera	Culicidae	Aedes	Aedes aegypti	SPECIES	-25.82343	28.27225	3079713588
390	Diptera	Culicidae	Aedes	Aedes aegypti	SPECIES	-25.78000	-53.42000	1264872047
391	Diptera	Culicidae	Aedes	Aedes aegypti	SPECIES	-25.77000	-53.27000	1264872060
392	Diptera	Culicidae	Aedes	Aedes aegypti	SPECIES	-25.75584	28.31846	1802767245
393	Diptera	Culicidae	Aedes	Aedes aegypti	SPECIES	-25.74213	28.28555	4015192965
394	Diptera	Culicidae	Aedes	Aedes aegypti	SPECIES	-25.74000	-53.11000	1264872070
395	Diptera	Culicidae	Aedes	Aedes aegypti	SPECIES	-25.73000	-53.72000	1264872080
396	Diptera	Culicidae	Aedes	Aedes aegypti	SPECIES	-25.71355	28.24433	3759863788
397	Diptera	Culicidae	Aedes	Aedes aegypti	SPECIES	-25.67000	-53.55000	1264872097
398	Diptera	Culicidae	Aedes	Aedes aegypti	SPECIES	-25.64000	149.79000	1264886326
399	Diptera	Culicidae	Aedes	Aedes aegypti	SPECIES	-25.62000	151.60000	1264886364
400	Diptera	Culicidae	Aedes	Aedes aegypti	SPECIES	-25.62000	-54.57000	1264886564
401	Diptera	Culicidae	Aedes	Aedes aegypti	SPECIES	-25.61000	-53.79000	1264872087
402	Diptera	Culicidae	Aedes	Aedes aegypti	SPECIES	-25.61000	-54.57000	1264886549
403	Diptera	Culicidae	Aedes	Aedes aegypti	SPECIES	-25.61000	-54.58000	1264890721
404	Diptera	Culicidae	Aedes	Aedes aegypti	SPECIES	-25.60000	-54.59000	1264886527
405	Diptera	Culicidae	Aedes	Aedes aegypti	SPECIES	-25.60000	-54.58000	1264887008
406	Diptera	Culicidae	Aedes	Aedes aegypti	SPECIES	-25.60000	-54.57000	1264887033
407	Diptera	Culicidae	Aedes	Aedes aegypti	SPECIES	-25.59000	-54.57000	1264886540
408	Diptera	Culicidae	Aedes	Aedes aegypti	SPECIES	-25.58333	151.30000	177194549
409	Diptera	Culicidae	Aedes	Aedes aegypti	SPECIES	-25.58333	151.30000	1633667697
410	Diptera	Culicidae	Aedes	Aedes aegypti	SPECIES	-25.58000	-54.57000	1264887010

№	Order	Family	Genus	Species	Taxon Rank	Latitude	Longitude	GBIF_ID
411	Diptera	Culicidae	Aedes	Aedes aegypti	SPECIES	-25.55154	-54.58128	4062942152
412	Diptera	Culicidae	Aedes	Aedes aegypti	SPECIES	-25.55000	-52.60000	1264872105
413	Diptera	Culicidae	Aedes	Aedes aegypti	SPECIES	-25.55000	-48.49000	1264872123
414	Diptera	Culicidae	Aedes	Aedes aegypti	SPECIES	-25.55000	-54.57000	1264886659
415	Diptera	Culicidae	Aedes	Aedes aegypti	SPECIES	-25.55000	-54.58000	1264889160
416	Diptera	Culicidae	Aedes	Aedes aegypti	SPECIES	-25.55000	-54.59000	1264889497
417	Diptera	Culicidae	Aedes	Aedes aegypti	SPECIES	-25.55000	-54.58330	3499791626
418	Diptera	Culicidae	Aedes	Aedes aegypti	SPECIES	-25.54000	-54.58000	1264888420
419	Diptera	Culicidae	Aedes	Aedes aegypti	SPECIES	-25.51667	152.05000	177194653
420	Diptera	Culicidae	Aedes	Aedes aegypti	SPECIES	-25.51667	152.05000	1633663682
421	Diptera	Culicidae	Aedes	Aedes aegypti	SPECIES	-25.50000	-49.29000	1264872113
422	Diptera	Culicidae	Aedes	Aedes aegypti	SPECIES	-25.49000	-50.86000	1264872091
423	Diptera	Culicidae	Aedes	Aedes aegypti	SPECIES	-25.48000	-54.02000	1264872107
424	Diptera	Culicidae	Aedes	Aedes aegypti	SPECIES	-25.48000	-54.48000	1264890787
425	Diptera	Culicidae	Aedes	Aedes aegypti	SPECIES	-25.46000	-53.59000	1264872093
426	Diptera	Culicidae	Aedes	Aedes aegypti	SPECIES	-25.45000	-52.96000	1264872082
427	Diptera	Culicidae	Aedes	Aedes aegypti	SPECIES	-25.45000	-53.23000	1264872114
428	Diptera	Culicidae	Aedes	Aedes aegypti	SPECIES	-25.45000	-54.55000	1264872129
429	Diptera	Culicidae	Aedes	Aedes aegypti	SPECIES	-25.44000	-53.43000	1264872090
430	Diptera	Culicidae	Aedes	Aedes aegypti	SPECIES	-25.44000	-54.41000	1264872102
431	Diptera	Culicidae	Aedes	Aedes aegypti	SPECIES	-25.43681	-49.26997	3759214127
432	Diptera	Culicidae	Aedes	Aedes aegypti	SPECIES	-25.40000	-54.25000	1264872100
433	Diptera	Culicidae	Aedes	Aedes aegypti	SPECIES	-25.39000	-53.55000	1264872077
434	Diptera	Culicidae	Aedes	Aedes aegypti	SPECIES	-25.38000	-57.61000	1264890694
435	Diptera	Culicidae	Aedes	Aedes aegypti	SPECIES	-25.38000	-57.33000	1264891294
436	Diptera	Culicidae	Aedes	Aedes aegypti	SPECIES	-25.37060	151.12257	1100304641
437	Diptera	Culicidae	Aedes	Aedes aegypti	SPECIES	-25.37000	-57.60000	1264890701
438	Diptera	Culicidae	Aedes	Aedes aegypti	SPECIES	-25.37000	-57.62000	1264890723
439	Diptera	Culicidae	Aedes	Aedes aegypti	SPECIES	-25.37000	-57.45000	1264890999
440	Diptera	Culicidae	Aedes	Aedes aegypti	SPECIES	-25.33000	-53.90000	1264872084
441	Diptera	Culicidae	Aedes	Aedes aegypti	SPECIES	-25.33000	-57.58000	1264890706
442	Diptera	Culicidae	Aedes	Aedes aegypti	SPECIES	-25.32000	-57.58000	1264890655
443	Diptera	Culicidae	Aedes	Aedes aegypti	SPECIES	-25.32000	-54.35000	3350011904
444	Diptera	Culicidae	Aedes	Aedes aegypti	SPECIES	-25.31000	-52.39000	1264872103
445	Diptera	Culicidae	Aedes	Aedes aegypti	SPECIES	-25.31000	-57.58000	1264890684
446	Diptera	Culicidae	Aedes	Aedes aegypti	SPECIES	-25.30468	-57.57319	2988687557
447	Diptera	Culicidae	Aedes	Aedes aegypti	SPECIES	-25.30000	-49.34000	1264872094
448	Diptera	Culicidae	Aedes	Aedes aegypti	SPECIES	-25.30000	-57.63000	1264890692
449	Diptera	Culicidae	Aedes	Aedes aegypti	SPECIES	-25.29000	-53.79000	1264872081
450	Diptera	Culicidae	Aedes	Aedes aegypti	SPECIES	-25.29000	-57.72000	1264887027
451	Diptera	Culicidae	Aedes	Aedes aegypti	SPECIES	-25.28000	-57.72000	1264886853

№	Order	Family	Genus	Species	Taxon Rank	Latitude	Longitude	GBIF_ID
452	Diptera	Culicidae	Aedes	Aedes aegypti	SPECIES	-25.28000	-57.64000	1264887030
453	Diptera	Culicidae	Aedes	Aedes aegypti	SPECIES	-25.28000	-57.71000	1264890856
454	Diptera	Culicidae	Aedes	Aedes aegypti	SPECIES	-25.27000	-54.10000	1264872071
455	Diptera	Culicidae	Aedes	Aedes aegypti	SPECIES	-25.26729	-57.66657	177060576
456	Diptera	Culicidae	Aedes	Aedes aegypti	SPECIES	-25.26000	-57.61000	1264891258
457	Diptera	Culicidae	Aedes	Aedes aegypti	SPECIES	-25.25000	44.46667	732501234
458	Diptera	Culicidae	Aedes	Aedes aegypti	SPECIES	-25.25000	-53.54000	1264872112
459	Diptera	Culicidae	Aedes	Aedes aegypti	SPECIES	-25.23000	-53.15000	1264872073
460	Diptera	Culicidae	Aedes	Aedes aegypti	SPECIES	-25.20000	-52.62000	1264872086
461	Diptera	Culicidae	Aedes	Aedes aegypti	SPECIES	-25.19000	-55.88000	1264890960
462	Diptera	Culicidae	Aedes	Aedes aegypti	SPECIES	-25.15000	-50.07000	1264872096
463	Diptera	Culicidae	Aedes	Aedes aegypti	SPECIES	-25.14000	-54.33000	1264872109
464	Diptera	Culicidae	Aedes	Aedes aegypti	SPECIES	-25.11000	-54.26000	1264872121
465	Diptera	Culicidae	Aedes	Aedes aegypti	SPECIES	-25.10000	-54.09000	1264872115
466	Diptera	Culicidae	Aedes	Aedes aegypti	SPECIES	-25.06000	-53.40000	1264872131
467	Diptera	Culicidae	Aedes	Aedes aegypti	SPECIES	-25.05000	-52.86000	1264872148
468	Diptera	Culicidae	Aedes	Aedes aegypti	SPECIES	-25.02000	-53.62000	1264872137
469	Diptera	Culicidae	Aedes	Aedes aegypti	SPECIES	-25.02000	46.98000	1264888609
470	Diptera	Culicidae	Aedes	Aedes aegypti	SPECIES	-25.00000	151.95000	177194550
471	Diptera	Culicidae	Aedes	Aedes aegypti	SPECIES	-25.00000	-52.69000	1264872116
472	Diptera	Culicidae	Aedes	Aedes aegypti	SPECIES	-25.00000	-53.93000	1264872134
473	Diptera	Culicidae	Aedes	Aedes aegypti	SPECIES	-25.00000	151.95000	1633667802
474	Diptera	Culicidae	Aedes	Aedes aegypti	SPECIES	-24.98000	31.60000	1264889060
475	Diptera	Culicidae	Aedes	Aedes aegypti	SPECIES	-24.97000	-54.09000	1264872119
476	Diptera	Culicidae	Aedes	Aedes aegypti	SPECIES	-24.95000	-53.45000	1264886631
477	Diptera	Culicidae	Aedes	Aedes aegypti	SPECIES	-24.91000	-51.90000	1264872152
478	Diptera	Culicidae	Aedes	Aedes aegypti	SPECIES	-24.90000	-53.01000	1264872139
479	Diptera	Culicidae	Aedes	Aedes aegypti	SPECIES	-24.89000	-53.87000	1264872106
480	Diptera	Culicidae	Aedes	Aedes aegypti	SPECIES	-24.88000	152.35000	1264888874
481	Diptera	Culicidae	Aedes	Aedes aegypti	SPECIES	-24.87000	-54.30000	1264872117
482	Diptera	Culicidae	Aedes	Aedes aegypti	SPECIES	-24.86000	-47.74000	1264872126
483	Diptera	Culicidae	Aedes	Aedes aegypti	SPECIES	-24.86000	151.12000	1264886407
484	Diptera	Culicidae	Aedes	Aedes aegypti	SPECIES	-24.82000	-54.12000	1264872101
485	Diptera	Culicidae	Aedes	Aedes aegypti	SPECIES	-24.82000	-52.71000	1264872125
486	Diptera	Culicidae	Aedes	Aedes aegypti	SPECIES	-24.80000	-53.92000	1264872111
487	Diptera	Culicidae	Aedes	Aedes aegypti	SPECIES	-24.79000	-53.07000	1264872128
488	Diptera	Culicidae	Aedes	Aedes aegypti	SPECIES	-24.79000	-65.41000	1264888425
489	Diptera	Culicidae	Aedes	Aedes aegypti	SPECIES	-24.78000	-48.18000	1264872108
490	Diptera	Culicidae	Aedes	Aedes aegypti	SPECIES	-24.78000	-48.07000	1264872118
491	Diptera	Culicidae	Aedes	Aedes aegypti	SPECIES	-24.78000	-65.42000	1264888394
492	Diptera	Culicidae	Aedes	Aedes aegypti	SPECIES	-24.77000	-65.40000	1264886677

№	Order	Family	Genus	Species	Taxon Rank	Latitude	Longitude	GBIF_ID
493	Diptera	Culicidae	Aedes	Aedes aegypti	SPECIES	-24.74000	-53.26000	1264872095
494	Diptera	Culicidae	Aedes	Aedes aegypti	SPECIES	-24.71773	-65.40195	4075921947
495	Diptera	Culicidae	Aedes	Aedes aegypti	SPECIES	-24.71000	-54.22000	1264872136
496	Diptera	Culicidae	Aedes	Aedes aegypti	SPECIES	-24.70000	-53.79000	1264872098
497	Diptera	Culicidae	Aedes	Aedes aegypti	SPECIES	-24.70000	-52.24000	1264872110
498	Diptera	Culicidae	Aedes	Aedes aegypti	SPECIES	-24.69000	-53.35000	1264872122
499	Diptera	Culicidae	Aedes	Aedes aegypti	SPECIES	-24.68000	-50.48000	1264872135
500	Diptera	Culicidae	Aedes	Aedes aegypti	SPECIES	-24.68000	-51.23000	1264872141
501	Diptera	Culicidae	Aedes	Aedes aegypti	SPECIES	-24.68000	-47.85000	1264872145
502	Diptera	Culicidae	Aedes	Aedes aegypti	SPECIES	-24.68000	-53.09000	1264872155
503	Diptera	Culicidae	Aedes	Aedes aegypti	SPECIES	-24.67873	25.88126	2826277412
504	Diptera	Culicidae	Aedes	Aedes aegypti	SPECIES	-24.67684	25.87779	2814156482
505	Diptera	Culicidae	Aedes	Aedes aegypti	SPECIES	-24.67591	25.88003	2963818311
506	Diptera	Culicidae	Aedes	Aedes aegypti	SPECIES	-24.64000	-54.25000	1264872162
507	Diptera	Culicidae	Aedes	Aedes aegypti	SPECIES	-24.64000	-53.15000	1264872176
508	Diptera	Culicidae	Aedes	Aedes aegypti	SPECIES	-24.63000	-53.51000	1264872140
509	Diptera	Culicidae	Aedes	Aedes aegypti	SPECIES	-24.63000	-52.56000	1264872160
510	Diptera	Culicidae	Aedes	Aedes aegypti	SPECIES	-24.63000	-51.63000	1264891022
511	Diptera	Culicidae	Aedes	Aedes aegypti	SPECIES	-24.62000	-52.79000	1264872147
512	Diptera	Culicidae	Aedes	Aedes aegypti	SPECIES	-24.59000	-54.16000	1264872164
513	Diptera	Culicidae	Aedes	Aedes aegypti	SPECIES	-24.59000	-47.45000	1264872175
514	Diptera	Culicidae	Aedes	Aedes aegypti	SPECIES	-24.57000	-53.97000	1264872130
515	Diptera	Culicidae	Aedes	Aedes aegypti	SPECIES	-24.57000	-52.27000	1264872142
516	Diptera	Culicidae	Aedes	Aedes aegypti	SPECIES	-24.56000	-49.22000	1264872151
517	Diptera	Culicidae	Aedes	Aedes aegypti	SPECIES	-24.51000	-53.03000	1264872150
518	Diptera	Culicidae	Aedes	Aedes aegypti	SPECIES	-24.50000	-48.23000	1264872127
519	Diptera	Culicidae	Aedes	Aedes aegypti	SPECIES	-24.50000	-51.65000	1264872138
520	Diptera	Culicidae	Aedes	Aedes aegypti	SPECIES	-24.50000	-47.86000	1264872154
521	Diptera	Culicidae	Aedes	Aedes aegypti	SPECIES	-24.49000	-53.30000	1264872146
522	Diptera	Culicidae	Aedes	Aedes aegypti	SPECIES	-24.47000	-53.81000	1264872133
523	Diptera	Culicidae	Aedes	Aedes aegypti	SPECIES	-24.45000	-53.99000	1264872120
524	Diptera	Culicidae	Aedes	Aedes aegypti	SPECIES	-24.45000	-53.57000	1264872161
525	Diptera	Culicidae	Aedes	Aedes aegypti	SPECIES	-24.44000	-54.19000	1264872124
526	Diptera	Culicidae	Aedes	Aedes aegypti	SPECIES	-24.43000	-53.35000	1264872132
527	Diptera	Culicidae	Aedes	Aedes aegypti	SPECIES	-24.43000	-51.94000	1264872144
528	Diptera	Culicidae	Aedes	Aedes aegypti	SPECIES	-24.42000	145.47000	1264886347
529	Diptera	Culicidae	Aedes	Aedes aegypti	SPECIES	-24.41000	-53.41000	1264872158
530	Diptera	Culicidae	Aedes	Aedes aegypti	SPECIES	-24.41000	-52.84000	1264872167
531	Diptera	Culicidae	Aedes	Aedes aegypti	SPECIES	-24.40000	150.50000	1264886385
532	Diptera	Culicidae	Aedes	Aedes aegypti	SPECIES	-24.38000	-51.55000	1264872166
533	Diptera	Culicidae	Aedes	Aedes aegypti	SPECIES	-24.36000	-52.11000	1264872179

№	Order	Family	Genus	Species	Taxon Rank	Latitude	Longitude	GBIF_ID
534	Diptera	Culicidae	Aedes	Aedes aegypti	SPECIES	-24.35000	-52.30000	1264872185
535	Diptera	Culicidae	Aedes	Aedes aegypti	SPECIES	-24.35000	-52.60000	1264872204
536	Diptera	Culicidae	Aedes	Aedes aegypti	SPECIES	-24.34000	-51.33000	1264872165
537	Diptera	Culicidae	Aedes	Aedes aegypti	SPECIES	-24.33000	-51.83000	1264872182
538	Diptera	Culicidae	Aedes	Aedes aegypti	SPECIES	-24.32000	-53.15000	1264872171
539	Diptera	Culicidae	Aedes	Aedes aegypti	SPECIES	-24.31000	-51.21000	1264872199
540	Diptera	Culicidae	Aedes	Aedes aegypti	SPECIES	-24.30000	-47.95000	1264872156
541	Diptera	Culicidae	Aedes	Aedes aegypti	SPECIES	-24.30000	-53.35000	1264872189
542	Diptera	Culicidae	Aedes	Aedes aegypti	SPECIES	-24.29000	-53.81000	1264872168
543	Diptera	Culicidae	Aedes	Aedes aegypti	SPECIES	-24.29000	-47.14000	1264872177
544	Diptera	Culicidae	Aedes	Aedes aegypti	SPECIES	-24.28000	-51.62000	1264872153
545	Diptera	Culicidae	Aedes	Aedes aegypti	SPECIES	-24.28000	-47.01000	1264872172
546	Diptera	Culicidae	Aedes	Aedes aegypti	SPECIES	-24.27000	-52.97000	1264872163
547	Diptera	Culicidae	Aedes	Aedes aegypti	SPECIES	-24.26000	-52.75000	1264872186
548	Diptera	Culicidae	Aedes	Aedes aegypti	SPECIES	-24.22000	-47.64000	1264872173
549	Diptera	Culicidae	Aedes	Aedes aegypti	SPECIES	-24.21000	-51.75000	1264872159
550	Diptera	Culicidae	Aedes	Aedes aegypti	SPECIES	-24.19000	-47.36000	1264872143
551	Diptera	Culicidae	Aedes	Aedes aegypti	SPECIES	-24.19000	-51.46000	1264872149
552	Diptera	Culicidae	Aedes	Aedes aegypti	SPECIES	-24.19000	-54.07000	1264872157
553	Diptera	Culicidae	Aedes	Aedes aegypti	SPECIES	-24.19000	-54.22000	1264872183
554	Diptera	Culicidae	Aedes	Aedes aegypti	SPECIES	-24.18000	-47.16000	1264872169
555	Diptera	Culicidae	Aedes	Aedes aegypti	SPECIES	-24.17000	-53.06000	1264872188
556	Diptera	Culicidae	Aedes	Aedes aegypti	SPECIES	-24.16000	-53.55000	1264872192
557	Diptera	Culicidae	Aedes	Aedes aegypti	SPECIES	-24.15000	-51.91000	1264872191
558	Diptera	Culicidae	Aedes	Aedes aegypti	SPECIES	-24.15000	-52.79000	1264872202
559	Diptera	Culicidae	Aedes	Aedes aegypti	SPECIES	-24.15000	30.83000	1264889090
560	Diptera	Culicidae	Aedes	Aedes aegypti	SPECIES	-24.13000	-52.37000	1264872207
561	Diptera	Culicidae	Aedes	Aedes aegypti	SPECIES	-24.13000	-52.14000	1264872226
562	Diptera	Culicidae	Aedes	Aedes aegypti	SPECIES	-24.12000	-52.64000	1264872190
563	Diptera	Culicidae	Aedes	Aedes aegypti	SPECIES	-24.10000	-53.88000	1264872193
564	Diptera	Culicidae	Aedes	Aedes aegypti	SPECIES	-24.10000	-49.32000	1264872211
565	Diptera	Culicidae	Aedes	Aedes aegypti	SPECIES	-24.10000	-46.81000	1264872220
566	Diptera	Culicidae	Aedes	Aedes aegypti	SPECIES	-24.09000	-53.42000	1264872214
567	Diptera	Culicidae	Aedes	Aedes aegypti	SPECIES	-24.08000	-51.78000	1264872181
568	Diptera	Culicidae	Aedes	Aedes aegypti	SPECIES	-24.07000	-46.67000	1264872178
569	Diptera	Culicidae	Aedes	Aedes aegypti	SPECIES	-24.07000	-51.63000	1264872197
570	Diptera	Culicidae	Aedes	Aedes aegypti	SPECIES	-24.07000	-49.98000	1264872200
571	Diptera	Culicidae	Aedes	Aedes aegypti	SPECIES	-24.07000	-52.06000	1264872203
572	Diptera	Culicidae	Aedes	Aedes aegypti	SPECIES	-24.06670	-54.25000	3499791912
573	Diptera	Culicidae	Aedes	Aedes aegypti	SPECIES	-24.06000	-53.76000	1264872187
574	Diptera	Culicidae	Aedes	Aedes aegypti	SPECIES	-24.06000	-53.22000	1264872208

№	Order	Family	Genus	Species	Taxon Rank	Latitude	Longitude	GBIF_ID
575	Diptera	Culicidae	Aedes	Aedes aegypti	SPECIES	-24.05000	-48.27000	1264872195
576	Diptera	Culicidae	Aedes	Aedes aegypti	SPECIES	-24.02000	-46.50000	1264872170
577	Diptera	Culicidae	Aedes	Aedes aegypti	SPECIES	-24.02000	-52.99000	1264872180
578	Diptera	Culicidae	Aedes	Aedes aegypti	SPECIES	-24.00000	-51.82000	1264872174
579	Diptera	Culicidae	Aedes	Aedes aegypti	SPECIES	-24.00000	-51.50000	1264872184
580	Diptera	Culicidae	Aedes	Aedes aegypti	SPECIES	-24.00000	-51.27000	1264872206
581	Diptera	Culicidae	Aedes	Aedes aegypti	SPECIES	-23.96000	-53.31000	1264872194
582	Diptera	Culicidae	Aedes	Aedes aegypti	SPECIES	-23.96000	-53.57000	1264872212
583	Diptera	Culicidae	Aedes	Aedes aegypti	SPECIES	-23.95000	-51.60000	1264872216
584	Diptera	Culicidae	Aedes	Aedes aegypti	SPECIES	-23.95000	-46.50000	1264872217
585	Diptera	Culicidae	Aedes	Aedes aegypti	SPECIES	-23.95000	-46.22000	1264872227
586	Diptera	Culicidae	Aedes	Aedes aegypti	SPECIES	-23.95000	-46.32000	1264890481
587	Diptera	Culicidae	Aedes	Aedes aegypti	SPECIES	-23.94000	-48.67000	1264872233
588	Diptera	Culicidae	Aedes	Aedes aegypti	SPECIES	-23.94000	-54.25000	1264872251
589	Diptera	Culicidae	Aedes	Aedes aegypti	SPECIES	-23.93000	-48.01000	1264872215
590	Diptera	Culicidae	Aedes	Aedes aegypti	SPECIES	-23.93000	-52.59000	1264872218
591	Diptera	Culicidae	Aedes	Aedes aegypti	SPECIES	-23.93000	-52.31000	1264872235
592	Diptera	Culicidae	Aedes	Aedes aegypti	SPECIES	-23.92000	-51.18000	1264872244
593	Diptera	Culicidae	Aedes	Aedes aegypti	SPECIES	-23.91000	-49.15000	1264872209
594	Diptera	Culicidae	Aedes	Aedes aegypti	SPECIES	-23.91000	-53.95000	1264872238
595	Diptera	Culicidae	Aedes	Aedes aegypti	SPECIES	-23.90000	-48.87000	1264872219
596	Diptera	Culicidae	Aedes	Aedes aegypti	SPECIES	-23.89000	-52.04000	1264872229
597	Diptera	Culicidae	Aedes	Aedes aegypti	SPECIES	-23.88000	-50.61000	1264872223
598	Diptera	Culicidae	Aedes	Aedes aegypti	SPECIES	-23.88000	-61.85000	1264886647
599	Diptera	Culicidae	Aedes	Aedes aegypti	SPECIES	-23.87000	-54.89000	1264872201
600	Diptera	Culicidae	Aedes	Aedes aegypti	SPECIES	-23.87000	-49.80000	1264872213
601	Diptera	Culicidae	Aedes	Aedes aegypti	SPECIES	-23.87000	-46.29000	1264890797
602	Diptera	Culicidae	Aedes	Aedes aegypti	SPECIES	-23.86000	-52.86000	1264872198
603	Diptera	Culicidae	Aedes	Aedes aegypti	SPECIES	-23.86000	-46.41000	1264872205
604	Diptera	Culicidae	Aedes	Aedes aegypti	SPECIES	-23.86000	-51.69000	1264872224
605	Diptera	Culicidae	Aedes	Aedes aegypti	SPECIES	-23.86000	-47.72000	1264872231
606	Diptera	Culicidae	Aedes	Aedes aegypti	SPECIES	-23.86000	-46.29000	1264872239
607	Diptera	Culicidae	Aedes	Aedes aegypti	SPECIES	-23.85000	151.26000	1264887734
608	Diptera	Culicidae	Aedes	Aedes aegypti	SPECIES	-23.84000	-45.34000	1264872196
609	Diptera	Culicidae	Aedes	Aedes aegypti	SPECIES	-23.83000	-51.87000	1264872210
610	Diptera	Culicidae	Aedes	Aedes aegypti	SPECIES	-23.83000	-52.15000	1264872225
611	Diptera	Culicidae	Aedes	Aedes aegypti	SPECIES	-23.83000	-64.79000	1264887003
612	Diptera	Culicidae	Aedes	Aedes aegypti	SPECIES	-23.83000	30.16000	1264889054
613	Diptera	Culicidae	Aedes	Aedes aegypti	SPECIES	-23.82000	-53.70000	1264872236
614	Diptera	Culicidae	Aedes	Aedes aegypti	SPECIES	-23.82000	-54.54000	1264872241
615	Diptera	Culicidae	Aedes	Aedes aegypti	SPECIES	-23.82000	-51.03000	1264872243

№	Order	Family	Genus	Species	Taxon Rank	Latitude	Longitude	GBIF_ID
616	Diptera	Culicidae	Aedes	Aedes aegypti	SPECIES	-23.82000	-64.78000	1264887017
617	Diptera	Culicidae	Aedes	Aedes aegypti	SPECIES	-23.81000	-47.19000	1264872252
618	Diptera	Culicidae	Aedes	Aedes aegypti	SPECIES	-23.80000	-51.43000	1264872240
619	Diptera	Culicidae	Aedes	Aedes aegypti	SPECIES	-23.80000	-47.43000	1264872257
620	Diptera	Culicidae	Aedes	Aedes aegypti	SPECIES	-23.80000	-46.53000	1264872275
621	Diptera	Culicidae	Aedes	Aedes aegypti	SPECIES	-23.79000	-50.30000	1264872259
622	Diptera	Culicidae	Aedes	Aedes aegypti	SPECIES	-23.79000	-45.41000	1264888081
623	Diptera	Culicidae	Aedes	Aedes aegypti	SPECIES	-23.78000	-51.53000	1264872242
624	Diptera	Culicidae	Aedes	Aedes aegypti	SPECIES	-23.77000	-46.02000	1264872269
625	Diptera	Culicidae	Aedes	Aedes aegypti	SPECIES	-23.76000	-52.28000	1264872264
626	Diptera	Culicidae	Aedes	Aedes aegypti	SPECIES	-23.75000	-51.67000	1264872232
627	Diptera	Culicidae	Aedes	Aedes aegypti	SPECIES	-23.75000	-53.60000	1264872247
628	Diptera	Culicidae	Aedes	Aedes aegypti	SPECIES	-23.75000	-45.62000	1264872254
629	Diptera	Culicidae	Aedes	Aedes aegypti	SPECIES	-23.74000	-54.23000	1264872228
630	Diptera	Culicidae	Aedes	Aedes aegypti	SPECIES	-23.74000	-52.60000	1264872237
631	Diptera	Culicidae	Aedes	Aedes aegypti	SPECIES	-23.74000	-51.29000	1264872249
632	Diptera	Culicidae	Aedes	Aedes aegypti	SPECIES	-23.73000	-55.34000	1264872248
633	Diptera	Culicidae	Aedes	Aedes aegypti	SPECIES	-23.73000	-53.09000	1264872262
634	Diptera	Culicidae	Aedes	Aedes aegypti	SPECIES	-23.72849	-46.56677	3391643407
635	Diptera	Culicidae	Aedes	Aedes aegypti	SPECIES	-23.72000	-51.83000	1264872221
636	Diptera	Culicidae	Aedes	Aedes aegypti	SPECIES	-23.72000	-53.81000	1264872222
637	Diptera	Culicidae	Aedes	Aedes aegypti	SPECIES	-23.72000	-53.92000	1264872230
638	Diptera	Culicidae	Aedes	Aedes aegypti	SPECIES	-23.72000	-52.02000	1264872234
639	Diptera	Culicidae	Aedes	Aedes aegypti	SPECIES	-23.72000	-49.96000	1264872258
640	Diptera	Culicidae	Aedes	Aedes aegypti	SPECIES	-23.72000	149.67000	1264887698
641	Diptera	Culicidae	Aedes	Aedes aegypti	SPECIES	-23.71000	-46.50000	1264872250
642	Diptera	Culicidae	Aedes	Aedes aegypti	SPECIES	-23.71000	-50.75000	1264872263
643	Diptera	Culicidae	Aedes	Aedes aegypti	SPECIES	-23.71000	-53.42000	1264872268
644	Diptera	Culicidae	Aedes	Aedes aegypti	SPECIES	-23.70589	-46.54063	4046747855
645	Diptera	Culicidae	Aedes	Aedes aegypti	SPECIES	-23.70082	-64.90832	2597753780
646	Diptera	Culicidae	Aedes	Aedes aegypti	SPECIES	-23.70000	-46.61000	1264872266
647	Diptera	Culicidae	Aedes	Aedes aegypti	SPECIES	-23.70000	-52.39000	1264872276
648	Diptera	Culicidae	Aedes	Aedes aegypti	SPECIES	-23.68000	-46.72000	1264872284
649	Diptera	Culicidae	Aedes	Aedes aegypti	SPECIES	-23.68000	-46.95000	1264872299
650	Diptera	Culicidae	Aedes	Aedes aegypti	SPECIES	-23.67000	-51.34000	1264872265
651	Diptera	Culicidae	Aedes	Aedes aegypti	SPECIES	-23.67000	-46.44000	1264872281
652	Diptera	Culicidae	Aedes	Aedes aegypti	SPECIES	-23.66455	-46.55067	3872872007
653	Diptera	Culicidae	Aedes	Aedes aegypti	SPECIES	-23.66455	-46.55067	3902224392
654	Diptera	Culicidae	Aedes	Aedes aegypti	SPECIES	-23.66000	-47.59000	1264872267
655	Diptera	Culicidae	Aedes	Aedes aegypti	SPECIES	-23.66000	-52.44000	1264872291
656	Diptera	Culicidae	Aedes	Aedes aegypti	SPECIES	-23.66000	-49.78000	1264872294

№	Order	Family	Genus	Species	Taxon Rank	Latitude	Longitude	GBIF_ID
657	Diptera	Culicidae	Aedes	Aedes aegypti	SPECIES	-23.65000	-49.44000	1264872256
658	Diptera	Culicidae	Aedes	Aedes aegypti	SPECIES	-23.65000	-46.86000	1264872272
659	Diptera	Culicidae	Aedes	Aedes aegypti	SPECIES	-23.65000	-47.78000	1264872278
660	Diptera	Culicidae	Aedes	Aedes aegypti	SPECIES	-23.65000	146.63000	1264886333
661	Diptera	Culicidae	Aedes	Aedes aegypti	SPECIES	-23.65000	-46.65000	1264890816
662	Diptera	Culicidae	Aedes	Aedes aegypti	SPECIES	-23.65000	-46.45000	1264890945
663	Diptera	Culicidae	Aedes	Aedes aegypti	SPECIES	-23.64000	-52.87000	1264872274
664	Diptera	Culicidae	Aedes	Aedes aegypti	SPECIES	-23.64000	150.39000	1264887710
665	Diptera	Culicidae	Aedes	Aedes aegypti	SPECIES	-23.64000	150.40000	3818475992
666	Diptera	Culicidae	Aedes	Aedes aegypti	SPECIES	-23.63000	-48.11000	1264872253
667	Diptera	Culicidae	Aedes	Aedes aegypti	SPECIES	-23.63000	-54.96000	1264872261
668	Diptera	Culicidae	Aedes	Aedes aegypti	SPECIES	-23.63000	-46.56000	1264872273
669	Diptera	Culicidae	Aedes	Aedes aegypti	SPECIES	-23.63000	-51.70000	1264872288
670	Diptera	Culicidae	Aedes	Aedes aegypti	SPECIES	-23.62000	-45.49000	1264872246
671	Diptera	Culicidae	Aedes	Aedes aegypti	SPECIES	-23.62000	-50.50000	1264872255
672	Diptera	Culicidae	Aedes	Aedes aegypti	SPECIES	-23.62000	-46.79000	1264872283
673	Diptera	Culicidae	Aedes	Aedes aegypti	SPECIES	-23.62000	-45.42000	1264890719
674	Diptera	Culicidae	Aedes	Aedes aegypti	SPECIES	-23.61667	144.25000	177194587
675	Diptera	Culicidae	Aedes	Aedes aegypti	SPECIES	-23.61667	144.25000	1633663779
676	Diptera	Culicidae	Aedes	Aedes aegypti	SPECIES	-23.61110	-46.69051	2814003278
677	Diptera	Culicidae	Aedes	Aedes aegypti	SPECIES	-23.61091	-46.69043	3759482311
678	Diptera	Culicidae	Aedes	Aedes aegypti	SPECIES	-23.61000	-48.45000	1264872245
679	Diptera	Culicidae	Aedes	Aedes aegypti	SPECIES	-23.61000	-51.55000	1264872260
680	Diptera	Culicidae	Aedes	Aedes aegypti	SPECIES	-23.61000	-52.10000	1264872279
681	Diptera	Culicidae	Aedes	Aedes aegypti	SPECIES	-23.59000	-50.75000	1264872287
682	Diptera	Culicidae	Aedes	Aedes aegypti	SPECIES	-23.59000	-52.18000	1264872290
683	Diptera	Culicidae	Aedes	Aedes aegypti	SPECIES	-23.59000	-53.23000	1264872293
684	Diptera	Culicidae	Aedes	Aedes aegypti	SPECIES	-23.58000	-47.41000	1264872301
685	Diptera	Culicidae	Aedes	Aedes aegypti	SPECIES	-23.58000	-51.42000	1264872307
686	Diptera	Culicidae	Aedes	Aedes aegypti	SPECIES	-23.58000	-50.63000	1264872325
687	Diptera	Culicidae	Aedes	Aedes aegypti	SPECIES	-23.57057	-46.74018	2860094245
688	Diptera	Culicidae	Aedes	Aedes aegypti	SPECIES	-23.57000	-52.20000	1264872289
689	Diptera	Culicidae	Aedes	Aedes aegypti	SPECIES	-23.57000	-49.56000	1264872303
690	Diptera	Culicidae	Aedes	Aedes aegypti	SPECIES	-23.57000	149.07000	1264886369
691	Diptera	Culicidae	Aedes	Aedes aegypti	SPECIES	-23.56000	-46.37000	1264872292
692	Diptera	Culicidae	Aedes	Aedes aegypti	SPECIES	-23.55731	-46.66738	3859676172
693	Diptera	Culicidae	Aedes	Aedes aegypti	SPECIES	-23.55577	-46.63956	3759679001
694	Diptera	Culicidae	Aedes	Aedes aegypti	SPECIES	-23.55000	-46.85000	1264872280
695	Diptera	Culicidae	Aedes	Aedes aegypti	SPECIES	-23.55000	-47.87000	1264872317
696	Diptera	Culicidae	Aedes	Aedes aegypti	SPECIES	-23.55000	-46.97000	1264872320
697	Diptera	Culicidae	Aedes	Aedes aegypti	SPECIES	-23.55000	145.28000	1264886387

№	Order	Family	Genus	Species	Taxon Rank	Latitude	Longitude	GBIF_ID
698	Diptera	Culicidae	Aedes	Aedes aegypti	SPECIES	-23.54000	-47.66000	1264872296
699	Diptera	Culicidae	Aedes	Aedes aegypti	SPECIES	-23.54000	-50.79000	1264872300
700	Diptera	Culicidae	Aedes	Aedes aegypti	SPECIES	-23.54000	-46.90000	1264872305
701	Diptera	Culicidae	Aedes	Aedes aegypti	SPECIES	-23.54000	-46.63000	1264890702
702	Diptera	Culicidae	Aedes	Aedes aegypti	SPECIES	-23.53884	-46.54662	4039449707
703	Diptera	Culicidae	Aedes	Aedes aegypti	SPECIES	-23.53384	-46.53916	2574161904
704	Diptera	Culicidae	Aedes	Aedes aegypti	SPECIES	-23.53265	-46.70731	3802583419
705	Diptera	Culicidae	Aedes	Aedes aegypti	SPECIES	-23.53000	-49.24000	1264872277
706	Diptera	Culicidae	Aedes	Aedes aegypti	SPECIES	-23.53000	-47.28000	1264872286
707	Diptera	Culicidae	Aedes	Aedes aegypti	SPECIES	-23.53000	-47.10000	1264872298
708	Diptera	Culicidae	Aedes	Aedes aegypti	SPECIES	-23.53000	-46.78000	1264872314
709	Diptera	Culicidae	Aedes	Aedes aegypti	SPECIES	-23.53000	148.16000	1264887700
710	Diptera	Culicidae	Aedes	Aedes aegypti	SPECIES	-23.53000	-46.79000	1264890780
711	Diptera	Culicidae	Aedes	Aedes aegypti	SPECIES	-23.52000	-47.20000	1264872271
712	Diptera	Culicidae	Aedes	Aedes aegypti	SPECIES	-23.52000	-52.53000	1264872282
713	Diptera	Culicidae	Aedes	Aedes aegypti	SPECIES	-23.52000	-53.84000	1264872306
714	Diptera	Culicidae	Aedes	Aedes aegypti	SPECIES	-23.52000	-51.11000	1264890775
715	Diptera	Culicidae	Aedes	Aedes aegypti	SPECIES	-23.51000	-46.88000	1264872270
716	Diptera	Culicidae	Aedes	Aedes aegypti	SPECIES	-23.50000	-51.78000	1264872285
717	Diptera	Culicidae	Aedes	Aedes aegypti	SPECIES	-23.50000	-46.72000	1264889214
718	Diptera	Culicidae	Aedes	Aedes aegypti	SPECIES	-23.49000	-51.72000	1264872302
719	Diptera	Culicidae	Aedes	Aedes aegypti	SPECIES	-23.48000	-52.67000	1264872311
720	Diptera	Culicidae	Aedes	Aedes aegypti	SPECIES	-23.48000	-51.10000	1264872313
721	Diptera	Culicidae	Aedes	Aedes aegypti	SPECIES	-23.48000	-51.86000	1264872318
722	Diptera	Culicidae	Aedes	Aedes aegypti	SPECIES	-23.47000	-49.40000	1264872315
723	Diptera	Culicidae	Aedes	Aedes aegypti	SPECIES	-23.47000	-52.81000	1264872326
724	Diptera	Culicidae	Aedes	Aedes aegypti	SPECIES	-23.47000	-49.02000	1264872330
725	Diptera	Culicidae	Aedes	Aedes aegypti	SPECIES	-23.47000	-47.44000	1264872331
726	Diptera	Culicidae	Aedes	Aedes aegypti	SPECIES	-23.47000	-47.73000	1264872348
727	Diptera	Culicidae	Aedes	Aedes aegypti	SPECIES	-23.46000	-50.72000	1264872309
728	Diptera	Culicidae	Aedes	Aedes aegypti	SPECIES	-23.46000	-52.12000	1264872316
729	Diptera	Culicidae	Aedes	Aedes aegypti	SPECIES	-23.46000	-48.80000	1264872323
730	Diptera	Culicidae	Aedes	Aedes aegypti	SPECIES	-23.46000	-49.72000	1264872342
731	Diptera	Culicidae	Aedes	Aedes aegypti	SPECIES	-23.46000	-52.23000	1264872345
732	Diptera	Culicidae	Aedes	Aedes aegypti	SPECIES	-23.45000	-51.45000	1264872304
733	Diptera	Culicidae	Aedes	Aedes aegypti	SPECIES	-23.45000	-48.42000	1264872324
734	Diptera	Culicidae	Aedes	Aedes aegypti	SPECIES	-23.45000	-46.92000	1264872327
735	Diptera	Culicidae	Aedes	Aedes aegypti	SPECIES	-23.44000	-49.92000	1264872312
736	Diptera	Culicidae	Aedes	Aedes aegypti	SPECIES	-23.44000	-52.30000	1264872321
737	Diptera	Culicidae	Aedes	Aedes aegypti	SPECIES	-23.44000	-52.54000	1264872338
738	Diptera	Culicidae	Aedes	Aedes aegypti	SPECIES	-23.44000	144.24000	1264886350

№	Order	Family	Genus	Species	Taxon Rank	Latitude	Longitude	GBIF_ID
739	Diptera	Culicidae	Aedes	Aedes aegypti	SPECIES	-23.44000	144.26000	3818482876
740	Diptera	Culicidae	Aedes	Aedes aegypti	SPECIES	-23.43000	-47.07000	1264872308
741	Diptera	Culicidae	Aedes	Aedes aegypti	SPECIES	-23.42000	-50.88000	1264872295
742	Diptera	Culicidae	Aedes	Aedes aegypti	SPECIES	-23.42000	-50.40000	1264872297
743	Diptera	Culicidae	Aedes	Aedes aegypti	SPECIES	-23.42000	-54.56000	1264872310
744	Diptera	Culicidae	Aedes	Aedes aegypti	SPECIES	-23.42000	-53.07000	1264872329
745	Diptera	Culicidae	Aedes	Aedes aegypti	SPECIES	-23.42000	-51.92000	1264889193
746	Diptera	Culicidae	Aedes	Aedes aegypti	SPECIES	-23.42000	-51.94000	1264890572
747	Diptera	Culicidae	Aedes	Aedes aegypti	SPECIES	-23.41000	-50.54000	1264872332
748	Diptera	Culicidae	Aedes	Aedes aegypti	SPECIES	-23.41000	-51.97000	1264872336
749	Diptera	Culicidae	Aedes	Aedes aegypti	SPECIES	-23.41000	-47.64000	1264872343
750	Diptera	Culicidae	Aedes	Aedes aegypti	SPECIES	-23.41000	-51.93000	1264888043
751	Diptera	Culicidae	Aedes	Aedes aegypti	SPECIES	-23.41000	-51.92000	1264890466
752	Diptera	Culicidae	Aedes	Aedes aegypti	SPECIES	-23.40618	-51.93146	3881898315
753	Diptera	Culicidae	Aedes	Aedes aegypti	SPECIES	-23.40614	-51.93145	2005326921
754	Diptera	Culicidae	Aedes	Aedes aegypti	SPECIES	-23.40611	-51.93143	2529253445
755	Diptera	Culicidae	Aedes	Aedes aegypti	SPECIES	-23.40000	-45.00000	1264872337
756	Diptera	Culicidae	Aedes	Aedes aegypti	SPECIES	-23.40000	-46.45000	1264890756
757	Diptera	Culicidae	Aedes	Aedes aegypti	SPECIES	-23.40000	-51.97000	1264890820
758	Diptera	Culicidae	Aedes	Aedes aegypti	SPECIES	-23.39000	-46.45000	1264872360
759	Diptera	Culicidae	Aedes	Aedes aegypti	SPECIES	-23.39000	-54.11000	1264872377
760	Diptera	Culicidae	Aedes	Aedes aegypti	SPECIES	-23.38050	150.50100	3442455431
761	Diptera	Culicidae	Aedes	Aedes aegypti	SPECIES	-23.38000	-46.74000	1264872340
762	Diptera	Culicidae	Aedes	Aedes aegypti	SPECIES	-23.38000	-53.44000	1264872341
763	Diptera	Culicidae	Aedes	Aedes aegypti	SPECIES	-23.38000	-48.22000	1264872353
764	Diptera	Culicidae	Aedes	Aedes aegypti	SPECIES	-23.38000	-52.60000	1264872354
765	Diptera	Culicidae	Aedes	Aedes aegypti	SPECIES	-23.38000	150.51000	1264887669
766	Diptera	Culicidae	Aedes	Aedes aegypti	SPECIES	-23.38000	150.50000	3818512023
767	Diptera	Culicidae	Aedes	Aedes aegypti	SPECIES	-23.37000	-52.50000	1264872328
768	Diptera	Culicidae	Aedes	Aedes aegypti	SPECIES	-23.37000	-52.94000	1264872362
769	Diptera	Culicidae	Aedes	Aedes aegypti	SPECIES	-23.37000	-53.60000	1264872366
770	Diptera	Culicidae	Aedes	Aedes aegypti	SPECIES	-23.36667	150.53334	177194557
771	Diptera	Culicidae	Aedes	Aedes aegypti	SPECIES	-23.36667	150.53334	1633663687
772	Diptera	Culicidae	Aedes	Aedes aegypti	SPECIES	-23.36000	-47.90000	1264872351
773	Diptera	Culicidae	Aedes	Aedes aegypti	SPECIES	-23.36000	43.66000	1264890612
774	Diptera	Culicidae	Aedes	Aedes aegypti	SPECIES	-23.35000	-51.60000	1264872333
775	Diptera	Culicidae	Aedes	Aedes aegypti	SPECIES	-23.35000	-46.88000	1264872349
776	Diptera	Culicidae	Aedes	Aedes aegypti	SPECIES	-23.35000	-53.29000	1264872350
777	Diptera	Culicidae	Aedes	Aedes aegypti	SPECIES	-23.34000	-53.12000	1264872339
778	Diptera	Culicidae	Aedes	Aedes aegypti	SPECIES	-23.34000	-50.70000	1264872357
779	Diptera	Culicidae	Aedes	Aedes aegypti	SPECIES	-23.34000	-57.05000	1264891029

№	Order	Family	Genus	Species	Taxon Rank	Latitude	Longitude	GBIF_ID
780	Diptera	Culicidae	Aedes	Aedes aegypti	SPECIES	-23.33000	-55.36000	1264872322
781	Diptera	Culicidae	Aedes	Aedes aegypti	SPECIES	-23.33000	-52.32000	1264872334
782	Diptera	Culicidae	Aedes	Aedes aegypti	SPECIES	-23.33000	-52.83000	1264872346
783	Diptera	Culicidae	Aedes	Aedes aegypti	SPECIES	-23.32000	-51.21000	1264890327
784	Diptera	Culicidae	Aedes	Aedes aegypti	SPECIES	-23.31000	-46.74000	1264872319
785	Diptera	Culicidae	Aedes	Aedes aegypti	SPECIES	-23.31000	-47.08000	1264872356
786	Diptera	Culicidae	Aedes	Aedes aegypti	SPECIES	-23.31000	-51.16000	1264889578
787	Diptera	Culicidae	Aedes	Aedes aegypti	SPECIES	-23.30000	-50.34000	1264872335
788	Diptera	Culicidae	Aedes	Aedes aegypti	SPECIES	-23.30000	-47.28000	1264872355
789	Diptera	Culicidae	Aedes	Aedes aegypti	SPECIES	-23.30000	-46.23000	1264872363
790	Diptera	Culicidae	Aedes	Aedes aegypti	SPECIES	-23.30000	-45.97000	1264872364
791	Diptera	Culicidae	Aedes	Aedes aegypti	SPECIES	-23.30000	-51.17000	1264890459
792	Diptera	Culicidae	Aedes	Aedes aegypti	SPECIES	-23.29000	-50.08000	1264872372
793	Diptera	Culicidae	Aedes	Aedes aegypti	SPECIES	-23.29000	-52.07000	1264872385
794	Diptera	Culicidae	Aedes	Aedes aegypti	SPECIES	-23.28000	-46.71000	1264872368
795	Diptera	Culicidae	Aedes	Aedes aegypti	SPECIES	-23.28000	-51.42000	1264872384
796	Diptera	Culicidae	Aedes	Aedes aegypti	SPECIES	-23.28000	-47.68000	1264872403
797	Diptera	Culicidae	Aedes	Aedes aegypti	SPECIES	-23.28000	-51.27000	1264890580
798	Diptera	Culicidae	Aedes	Aedes aegypti	SPECIES	-23.27000	-49.76000	1264872382
799	Diptera	Culicidae	Aedes	Aedes aegypti	SPECIES	-23.27000	-51.05000	1264890604
800	Diptera	Culicidae	Aedes	Aedes aegypti	SPECIES	-23.26000	-52.15000	1264872358
801	Diptera	Culicidae	Aedes	Aedes aegypti	SPECIES	-23.26000	-49.48000	1264872386
802	Diptera	Culicidae	Aedes	Aedes aegypti	SPECIES	-23.26000	-52.66000	1264872395
803	Diptera	Culicidae	Aedes	Aedes aegypti	SPECIES	-23.26000	-55.54000	1264891040
804	Diptera	Culicidae	Aedes	Aedes aegypti	SPECIES	-23.25000	-50.39000	1264872352
805	Diptera	Culicidae	Aedes	Aedes aegypti	SPECIES	-23.25000	-50.92000	1264872375
806	Diptera	Culicidae	Aedes	Aedes aegypti	SPECIES	-23.25000	-51.56000	3350115964
807	Diptera	Culicidae	Aedes	Aedes aegypti	SPECIES	-23.24000	-51.73000	1264872374
808	Diptera	Culicidae	Aedes	Aedes aegypti	SPECIES	-23.24000	-47.52000	1264872376
809	Diptera	Culicidae	Aedes	Aedes aegypti	SPECIES	-23.23000	-51.06000	1264872369
810	Diptera	Culicidae	Aedes	Aedes aegypti	SPECIES	-23.22000	-47.91000	1264872359
811	Diptera	Culicidae	Aedes	Aedes aegypti	SPECIES	-23.22000	-46.83000	1264872361
812	Diptera	Culicidae	Aedes	Aedes aegypti	SPECIES	-23.22000	-51.84000	1264872370
813	Diptera	Culicidae	Aedes	Aedes aegypti	SPECIES	-23.22000	-54.95000	1264872378
814	Diptera	Culicidae	Aedes	Aedes aegypti	SPECIES	-23.21000	-50.82000	1264872344
815	Diptera	Culicidae	Aedes	Aedes aegypti	SPECIES	-23.21000	-46.76000	1264872347
816	Diptera	Culicidae	Aedes	Aedes aegypti	SPECIES	-23.21000	-51.29000	1264872367
817	Diptera	Culicidae	Aedes	Aedes aegypti	SPECIES	-23.21000	-51.57000	1264872379
818	Diptera	Culicidae	Aedes	Aedes aegypti	SPECIES	-23.21000	-64.10000	1264891208
819	Diptera	Culicidae	Aedes	Aedes aegypti	SPECIES	-23.20000	-52.76000	1264872381
820	Diptera	Culicidae	Aedes	Aedes aegypti	SPECIES	-23.20000	-46.91000	1264872388

№	Order	Family	Genus	Species	Taxon Rank	Latitude	Longitude	GBIF_ID
821	Diptera	Culicidae	Aedes	Aedes aegypti	SPECIES	-23.20000	-52.48000	1264872390
822	Diptera	Culicidae	Aedes	Aedes aegypti	SPECIES	-23.20000	-49.62000	1264872394
823	Diptera	Culicidae	Aedes	Aedes aegypti	SPECIES	-23.20000	-51.92000	1264872410
824	Diptera	Culicidae	Aedes	Aedes aegypti	SPECIES	-23.19000	-47.76000	1264872391
825	Diptera	Culicidae	Aedes	Aedes aegypti	SPECIES	-23.19000	-49.39000	1264872409
826	Diptera	Culicidae	Aedes	Aedes aegypti	SPECIES	-23.19000	-50.64000	1264872426
827	Diptera	Culicidae	Aedes	Aedes aegypti	SPECIES	-23.18000	-49.06000	1264872380
828	Diptera	Culicidae	Aedes	Aedes aegypti	SPECIES	-23.18000	-48.12000	1264872408
829	Diptera	Culicidae	Aedes	Aedes aegypti	SPECIES	-23.18000	-50.65000	1264888324
830	Diptera	Culicidae	Aedes	Aedes aegypti	SPECIES	-23.18000	-51.28000	1264890807
831	Diptera	Culicidae	Aedes	Aedes aegypti	SPECIES	-23.17000	-47.29000	1264872415
832	Diptera	Culicidae	Aedes	Aedes aegypti	SPECIES	-23.17000	-52.62000	1264872418
833	Diptera	Culicidae	Aedes	Aedes aegypti	SPECIES	-23.17000	-52.18000	3350040763
834	Diptera	Culicidae	Aedes	Aedes aegypti	SPECIES	-23.16000	-52.18000	1264872373
835	Diptera	Culicidae	Aedes	Aedes aegypti	SPECIES	-23.16000	-49.98000	1264890597
836	Diptera	Culicidae	Aedes	Aedes aegypti	SPECIES	-23.15000	-49.97000	1264872398
837	Diptera	Culicidae	Aedes	Aedes aegypti	SPECIES	-23.15000	-47.07000	1264872400
838	Diptera	Culicidae	Aedes	Aedes aegypti	SPECIES	-23.15000	-50.34000	1264872406
839	Diptera	Culicidae	Aedes	Aedes aegypti	SPECIES	-23.15000	-49.94000	1264888351
840	Diptera	Culicidae	Aedes	Aedes aegypti	SPECIES	-23.14000	-48.60000	1264872387
841	Diptera	Culicidae	Aedes	Aedes aegypti	SPECIES	-23.14000	-53.12000	1264872396
842	Diptera	Culicidae	Aedes	Aedes aegypti	SPECIES	-23.14000	-52.83000	1264872399
843	Diptera	Culicidae	Aedes	Aedes aegypti	SPECIES	-23.14000	-44.76000	1264872402
844	Diptera	Culicidae	Aedes	Aedes aegypti	SPECIES	-23.14000	-64.32000	1264888422
845	Diptera	Culicidae	Aedes	Aedes aegypti	SPECIES	-23.13000	-52.06000	1264872365
846	Diptera	Culicidae	Aedes	Aedes aegypti	SPECIES	-23.13000	-48.31000	1264872371
847	Diptera	Culicidae	Aedes	Aedes aegypti	SPECIES	-23.13000	-51.74000	1264872383
848	Diptera	Culicidae	Aedes	Aedes aegypti	SPECIES	-23.13000	-46.57000	1264872404
849	Diptera	Culicidae	Aedes	Aedes aegypti	SPECIES	-23.13000	-64.32000	1264887023
850	Diptera	Culicidae	Aedes	Aedes aegypti	SPECIES	-23.13000	-64.33000	1264891163
851	Diptera	Culicidae	Aedes	Aedes aegypti	SPECIES	-23.12000	-47.98000	1264872393
852	Diptera	Culicidae	Aedes	Aedes aegypti	SPECIES	-23.12000	150.74000	1264886380
853	Diptera	Culicidae	Aedes	Aedes aegypti	SPECIES	-23.11810	-46.55681	3859822151
854	Diptera	Culicidae	Aedes	Aedes aegypti	SPECIES	-23.11000	-47.19000	1264872405
855	Diptera	Culicidae	Aedes	Aedes aegypti	SPECIES	-23.11000	-50.16000	1264872411
856	Diptera	Culicidae	Aedes	Aedes aegypti	SPECIES	-23.11000	-53.24000	1264872413
857	Diptera	Culicidae	Aedes	Aedes aegypti	SPECIES	-23.11000	-51.97000	1264872419
858	Diptera	Culicidae	Aedes	Aedes aegypti	SPECIES	-23.10000	-48.39000	1264872416
859	Diptera	Culicidae	Aedes	Aedes aegypti	SPECIES	-23.10000	-47.79000	1264872429
860	Diptera	Culicidae	Aedes	Aedes aegypti	SPECIES	-23.10000	-45.72000	1264872435
861	Diptera	Culicidae	Aedes	Aedes aegypti	SPECIES	-23.10000	-54.05000	1264872452

№	Order	Family	Genus	Species	Taxon Rank	Latitude	Longitude	GBIF_ID
862	Diptera	Culicidae	Aedes	Aedes aegypti	SPECIES	-23.10000	-45.18000	1264890711
863	Diptera	Culicidae	Aedes	Aedes aegypti	SPECIES	-23.09000	-45.51000	1264872401
864	Diptera	Culicidae	Aedes	Aedes aegypti	SPECIES	-23.09000	-52.97000	1264872433
865	Diptera	Culicidae	Aedes	Aedes aegypti	SPECIES	-23.09000	148.02000	1264887706
866	Diptera	Culicidae	Aedes	Aedes aegypti	SPECIES	-23.08000	-47.37000	1264872397
867	Diptera	Culicidae	Aedes	Aedes aegypti	SPECIES	-23.08000	-46.93000	1264872440
868	Diptera	Culicidae	Aedes	Aedes aegypti	SPECIES	-23.08000	-53.37000	1264872442
869	Diptera	Culicidae	Aedes	Aedes aegypti	SPECIES	-23.08000	148.02000	1264886382
870	Diptera	Culicidae	Aedes	Aedes aegypti	SPECIES	-23.08000	-52.46000	1264890578
871	Diptera	Culicidae	Aedes	Aedes aegypti	SPECIES	-23.07000	-52.10000	1264872421
872	Diptera	Culicidae	Aedes	Aedes aegypti	SPECIES	-23.07000	-51.06000	1264872424
873	Diptera	Culicidae	Aedes	Aedes aegypti	SPECIES	-23.07000	-52.33000	1264872425
874	Diptera	Culicidae	Aedes	Aedes aegypti	SPECIES	-23.07000	-49.12000	1264872434
875	Diptera	Culicidae	Aedes	Aedes aegypti	SPECIES	-23.07000	44.20000	1264886041
876	Diptera	Culicidae	Aedes	Aedes aegypti	SPECIES	-23.06000	-51.83000	1264872389
877	Diptera	Culicidae	Aedes	Aedes aegypti	SPECIES	-23.06000	-46.97000	1264872392
878	Diptera	Culicidae	Aedes	Aedes aegypti	SPECIES	-23.06000	-45.91000	1264872407
879	Diptera	Culicidae	Aedes	Aedes aegypti	SPECIES	-23.06000	-50.92000	1264872414
880	Diptera	Culicidae	Aedes	Aedes aegypti	SPECIES	-23.06000	-50.54000	1264872417
881	Diptera	Culicidae	Aedes	Aedes aegypti	SPECIES	-23.06000	-49.61000	1264872420
882	Diptera	Culicidae	Aedes	Aedes aegypti	SPECIES	-23.06000	-53.52000	1264872427
883	Diptera	Culicidae	Aedes	Aedes aegypti	SPECIES	-23.06000	-48.91000	1264872431
884	Diptera	Culicidae	Aedes	Aedes aegypti	SPECIES	-23.06000	-50.21000	3350129913
885	Diptera	Culicidae	Aedes	Aedes aegypti	SPECIES	-23.05000	-47.71000	1264872432
886	Diptera	Culicidae	Aedes	Aedes aegypti	SPECIES	-23.05000	-49.30000	1264872436
887	Diptera	Culicidae	Aedes	Aedes aegypti	SPECIES	-23.05000	-51.57000	1264872438
888	Diptera	Culicidae	Aedes	Aedes aegypti	SPECIES	-23.04000	-47.59000	1264872445
889	Diptera	Culicidae	Aedes	Aedes aegypti	SPECIES	-23.04000	-46.29000	1264872457
890	Diptera	Culicidae	Aedes	Aedes aegypti	SPECIES	-23.04000	-49.74000	1264872478
891	Diptera	Culicidae	Aedes	Aedes aegypti	SPECIES	-23.04000	-45.56000	1264890401
892	Diptera	Culicidae	Aedes	Aedes aegypti	SPECIES	-23.03000	-50.27000	1264872441
893	Diptera	Culicidae	Aedes	Aedes aegypti	SPECIES	-23.03000	-51.40000	1264872454
894	Diptera	Culicidae	Aedes	Aedes aegypti	SPECIES	-23.03000	-49.49000	1264872455
895	Diptera	Culicidae	Aedes	Aedes aegypti	SPECIES	-23.02000	-49.79000	1264872428
896	Diptera	Culicidae	Aedes	Aedes aegypti	SPECIES	-23.02000	-47.85000	1264872464
897	Diptera	Culicidae	Aedes	Aedes aegypti	SPECIES	-23.02000	-50.73000	1264872469
898	Diptera	Culicidae	Aedes	Aedes aegypti	SPECIES	-23.01000	-46.80000	1264872422
899	Diptera	Culicidae	Aedes	Aedes aegypti	SPECIES	-23.01000	-51.26000	1264872458
900	Diptera	Culicidae	Aedes	Aedes aegypti	SPECIES	-23.01000	-53.50000	3350107801
901	Diptera	Culicidae	Aedes	Aedes aegypti	SPECIES	-23.00000	-50.09000	1264872450
902	Diptera	Culicidae	Aedes	Aedes aegypti	SPECIES	-22.99000	-47.50000	1264872439

№	Order	Family	Genus	Species	Taxon Rank	Latitude	Longitude	GBIF_ID
903	Diptera	Culicidae	Aedes	Aedes aegypti	SPECIES	-22.99000	-51.48000	1264872446
904	Diptera	Culicidae	Aedes	Aedes aegypti	SPECIES	-22.99000	-50.44000	1264872449
905	Diptera	Culicidae	Aedes	Aedes aegypti	SPECIES	-22.99000	-43.25000	1264889170
906	Diptera	Culicidae	Aedes	Aedes aegypti	SPECIES	-22.99000	-43.36000	1264889447
907	Diptera	Culicidae	Aedes	Aedes aegypti	SPECIES	-22.99000	-44.34000	1264891068
908	Diptera	Culicidae	Aedes	Aedes aegypti	SPECIES	-22.98000	-52.00000	1264872430
909	Diptera	Culicidae	Aedes	Aedes aegypti	SPECIES	-22.98000	-46.98000	1264872444
910	Diptera	Culicidae	Aedes	Aedes aegypti	SPECIES	-22.98000	-52.15000	1264872460
911	Diptera	Culicidae	Aedes	Aedes aegypti	SPECIES	-22.98000	-43.36000	1264888306
912	Diptera	Culicidae	Aedes	Aedes aegypti	SPECIES	-22.98000	-43.49000	1264888339
913	Diptera	Culicidae	Aedes	Aedes aegypti	SPECIES	-22.97000	-49.37000	1264872412
914	Diptera	Culicidae	Aedes	Aedes aegypti	SPECIES	-22.97000	-55.37000	1264872423
915	Diptera	Culicidae	Aedes	Aedes aegypti	SPECIES	-22.97000	-53.08000	1264872453
916	Diptera	Culicidae	Aedes	Aedes aegypti	SPECIES	-22.97000	-56.54000	1264891019
917	Diptera	Culicidae	Aedes	Aedes aegypti	SPECIES	-22.96000	-48.05000	1264872443
918	Diptera	Culicidae	Aedes	Aedes aegypti	SPECIES	-22.96000	-51.69000	1264872459
919	Diptera	Culicidae	Aedes	Aedes aegypti	SPECIES	-22.96000	-49.87000	1264872465
920	Diptera	Culicidae	Aedes	Aedes aegypti	SPECIES	-22.96000	-56.54000	1264890700
921	Diptera	Culicidae	Aedes	Aedes aegypti	SPECIES	-22.96000	-43.18000	1264891343
922	Diptera	Culicidae	Aedes	Aedes aegypti	SPECIES	-22.95000	-47.30000	1264872461
923	Diptera	Culicidae	Aedes	Aedes aegypti	SPECIES	-22.95000	-50.89000	1264872474
924	Diptera	Culicidae	Aedes	Aedes aegypti	SPECIES	-22.95000	-44.40000	1264872489
925	Diptera	Culicidae	Aedes	Aedes aegypti	SPECIES	-22.95000	-43.19000	1264889419
926	Diptera	Culicidae	Aedes	Aedes aegypti	SPECIES	-22.94578	-46.99475	3017963780
927	Diptera	Culicidae	Aedes	Aedes aegypti	SPECIES	-22.94291	-43.19950	2563537150
928	Diptera	Culicidae	Aedes	Aedes aegypti	SPECIES	-22.94000	-46.56000	1264872451
929	Diptera	Culicidae	Aedes	Aedes aegypti	SPECIES	-22.94000	-43.47000	1264872466
930	Diptera	Culicidae	Aedes	Aedes aegypti	SPECIES	-22.94000	-47.60000	1264872479
931	Diptera	Culicidae	Aedes	Aedes aegypti	SPECIES	-22.94000	-45.60000	1264872482
932	Diptera	Culicidae	Aedes	Aedes aegypti	SPECIES	-22.94000	-44.04000	1264872505
933	Diptera	Culicidae	Aedes	Aedes aegypti	SPECIES	-22.94000	-43.24000	1264889483
934	Diptera	Culicidae	Aedes	Aedes aegypti	SPECIES	-22.93507	-43.20550	3398698590
935	Diptera	Culicidae	Aedes	Aedes aegypti	SPECIES	-22.93000	-42.14000	1264872448
936	Diptera	Culicidae	Aedes	Aedes aegypti	SPECIES	-22.93000	-46.22000	1264872487
937	Diptera	Culicidae	Aedes	Aedes aegypti	SPECIES	-22.93000	-45.30000	1264872495
938	Diptera	Culicidae	Aedes	Aedes aegypti	SPECIES	-22.93000	-43.25000	1264888327
939	Diptera	Culicidae	Aedes	Aedes aegypti	SPECIES	-22.93000	-43.38000	1264888338
940	Diptera	Culicidae	Aedes	Aedes aegypti	SPECIES	-22.93000	-43.45000	1264890907
941	Diptera	Culicidae	Aedes	Aedes aegypti	SPECIES	-22.92948	-43.39166	3005145756
942	Diptera	Culicidae	Aedes	Aedes aegypti	SPECIES	-22.92000	-43.05000	1264872471
943	Diptera	Culicidae	Aedes	Aedes aegypti	SPECIES	-22.92000	-51.79000	1264872472

№	Order	Family	Genus	Species	Taxon Rank	Latitude	Longitude	GBIF_ID
944	Diptera	Culicidae	Aedes	Aedes aegypti	SPECIES	-22.92000	-52.74000	1264872480
945	Diptera	Culicidae	Aedes	Aedes aegypti	SPECIES	-22.92000	-43.30000	1264889136
946	Diptera	Culicidae	Aedes	Aedes aegypti	SPECIES	-22.92000	-43.38000	1264889467
947	Diptera	Culicidae	Aedes	Aedes aegypti	SPECIES	-22.92000	-47.08000	1264890442
948	Diptera	Culicidae	Aedes	Aedes aegypti	SPECIES	-22.92000	-52.51000	1264890784
949	Diptera	Culicidae	Aedes	Aedes aegypti	SPECIES	-22.92000	-43.06000	1264890844
950	Diptera	Culicidae	Aedes	Aedes aegypti	SPECIES	-22.92000	-43.40000	1264891354
951	Diptera	Culicidae	Aedes	Aedes aegypti	SPECIES	-22.91000	-45.24000	1264872463
952	Diptera	Culicidae	Aedes	Aedes aegypti	SPECIES	-22.91000	-42.83000	1264872468
953	Diptera	Culicidae	Aedes	Aedes aegypti	SPECIES	-22.91000	139.91000	1264887726
954	Diptera	Culicidae	Aedes	Aedes aegypti	SPECIES	-22.91000	-47.11000	1264888346
955	Diptera	Culicidae	Aedes	Aedes aegypti	SPECIES	-22.91000	-43.20000	1264888360
956	Diptera	Culicidae	Aedes	Aedes aegypti	SPECIES	-22.91000	-43.28000	1264891312
957	Diptera	Culicidae	Aedes	Aedes aegypti	SPECIES	-22.90280	-43.20750	1703045416
958	Diptera	Culicidae	Aedes	Aedes aegypti	SPECIES	-22.90000	-47.03000	1264872481
959	Diptera	Culicidae	Aedes	Aedes aegypti	SPECIES	-22.90000	-43.52000	1264888102
960	Diptera	Culicidae	Aedes	Aedes aegypti	SPECIES	-22.90000	-43.20000	1264888132
961	Diptera	Culicidae	Aedes	Aedes aegypti	SPECIES	-22.90000	-43.53000	1264888331
962	Diptera	Culicidae	Aedes	Aedes aegypti	SPECIES	-22.90000	-43.57000	1264888336
963	Diptera	Culicidae	Aedes	Aedes aegypti	SPECIES	-22.90000	-43.13000	1264889407
964	Diptera	Culicidae	Aedes	Aedes aegypti	SPECIES	-22.90000	-43.25000	1264890509
965	Diptera	Culicidae	Aedes	Aedes aegypti	SPECIES	-22.90000	-43.23000	1264890722
966	Diptera	Culicidae	Aedes	Aedes aegypti	SPECIES	-22.89000	-45.49000	1264872447
967	Diptera	Culicidae	Aedes	Aedes aegypti	SPECIES	-22.89000	-46.79000	1264872456
968	Diptera	Culicidae	Aedes	Aedes aegypti	SPECIES	-22.89000	-51.40000	1264872470
969	Diptera	Culicidae	Aedes	Aedes aegypti	SPECIES	-22.89000	-43.22000	1264888113
970	Diptera	Culicidae	Aedes	Aedes aegypti	SPECIES	-22.89000	-43.23000	1264888362
971	Diptera	Culicidae	Aedes	Aedes aegypti	SPECIES	-22.89000	-42.04000	1264888368
972	Diptera	Culicidae	Aedes	Aedes aegypti	SPECIES	-22.89000	-42.03000	1264889431
973	Diptera	Culicidae	Aedes	Aedes aegypti	SPECIES	-22.89000	-48.45000	1264889558
974	Diptera	Culicidae	Aedes	Aedes aegypti	SPECIES	-22.89000	-43.39000	1264890516
975	Diptera	Culicidae	Aedes	Aedes aegypti	SPECIES	-22.88577	-42.02665	3044580894
976	Diptera	Culicidae	Aedes	Aedes aegypti	SPECIES	-22.88000	-49.27000	1264872477
977	Diptera	Culicidae	Aedes	Aedes aegypti	SPECIES	-22.88000	-43.46000	1264888100
978	Diptera	Culicidae	Aedes	Aedes aegypti	SPECIES	-22.88000	-43.43000	1264888363
979	Diptera	Culicidae	Aedes	Aedes aegypti	SPECIES	-22.88000	-43.30000	1264888377
980	Diptera	Culicidae	Aedes	Aedes aegypti	SPECIES	-22.88000	-43.29000	1264889452
981	Diptera	Culicidae	Aedes	Aedes aegypti	SPECIES	-22.88000	-43.47000	1264889453
982	Diptera	Culicidae	Aedes	Aedes aegypti	SPECIES	-22.88000	-48.45000	1264890456
983	Diptera	Culicidae	Aedes	Aedes aegypti	SPECIES	-22.88000	-43.09000	1264890750
984	Diptera	Culicidae	Aedes	Aedes aegypti	SPECIES	-22.88000	-43.28000	1264890754

№	Order	Family	Genus	Species	Taxon Rank	Latitude	Longitude	GBIF_ID
985	Diptera	Culicidae	Aedes	Aedes aegypti	SPECIES	-22.88000	-43.25000	1264890870
986	Diptera	Culicidae	Aedes	Aedes aegypti	SPECIES	-22.87034	-46.53066	3705682799
987	Diptera	Culicidae	Aedes	Aedes aegypti	SPECIES	-22.87000	-49.97000	1264872437
988	Diptera	Culicidae	Aedes	Aedes aegypti	SPECIES	-22.87000	-52.55000	1264872467
989	Diptera	Culicidae	Aedes	Aedes aegypti	SPECIES	-22.87000	-50.69000	1264872484
990	Diptera	Culicidae	Aedes	Aedes aegypti	SPECIES	-22.87000	-42.52000	1264872486
991	Diptera	Culicidae	Aedes	Aedes aegypti	SPECIES	-22.87000	-47.21000	1264872490
992	Diptera	Culicidae	Aedes	Aedes aegypti	SPECIES	-22.87000	-47.74000	1264872494
993	Diptera	Culicidae	Aedes	Aedes aegypti	SPECIES	-22.87000	-43.42000	1264888139
994	Diptera	Culicidae	Aedes	Aedes aegypti	SPECIES	-22.87000	-43.28000	1264888350
995	Diptera	Culicidae	Aedes	Aedes aegypti	SPECIES	-22.87000	-43.25000	1264888750
996	Diptera	Culicidae	Aedes	Aedes aegypti	SPECIES	-22.87000	-43.26000	1264889168
997	Diptera	Culicidae	Aedes	Aedes aegypti	SPECIES	-22.86000	-51.06000	1264872509
998	Diptera	Culicidae	Aedes	Aedes aegypti	SPECIES	-22.86000	-43.35000	1264888364
999	Diptera	Culicidae	Aedes	Aedes aegypti	SPECIES	-22.85000	-47.60000	1264872500
1000	Diptera	Culicidae	Aedes	Aedes aegypti	SPECIES	-22.85000	-47.26000	1264872529
1001	Diptera	Culicidae	Aedes	Aedes aegypti	SPECIES	-22.85000	-43.35000	1264889487
1002	Diptera	Culicidae	Aedes	Aedes aegypti	SPECIES	-22.85000	-43.27000	1264890512
1003	Diptera	Culicidae	Aedes	Aedes aegypti	SPECIES	-22.85000	-43.28000	1264891331
1004	Diptera	Culicidae	Aedes	Aedes aegypti	SPECIES	-22.84000	-48.40000	1264872491
1005	Diptera	Culicidae	Aedes	Aedes aegypti	SPECIES	-22.84000	-43.07000	1264888330
1006	Diptera	Culicidae	Aedes	Aedes aegypti	SPECIES	-22.84000	-45.25000	1264889931
1007	Diptera	Culicidae	Aedes	Aedes aegypti	SPECIES	-22.84000	-43.27000	1264890533
1008	Diptera	Culicidae	Aedes	Aedes aegypti	SPECIES	-22.83000	-52.15000	1264872476
1009	Diptera	Culicidae	Aedes	Aedes aegypti	SPECIES	-22.83000	-53.32000	1264872513
1010	Diptera	Culicidae	Aedes	Aedes aegypti	SPECIES	-22.83000	-52.33000	1264872515
1011	Diptera	Culicidae	Aedes	Aedes aegypti	SPECIES	-22.83000	-42.22000	1264872520
1012	Diptera	Culicidae	Aedes	Aedes aegypti	SPECIES	-22.83000	-43.07000	1264888140
1013	Diptera	Culicidae	Aedes	Aedes aegypti	SPECIES	-22.83000	-43.27000	1264888143
1014	Diptera	Culicidae	Aedes	Aedes aegypti	SPECIES	-22.83000	-43.29000	1264888316
1015	Diptera	Culicidae	Aedes	Aedes aegypti	SPECIES	-22.83000	-43.28000	1264889437
1016	Diptera	Culicidae	Aedes	Aedes aegypti	SPECIES	-22.83000	-43.00000	1264890813
1017	Diptera	Culicidae	Aedes	Aedes aegypti	SPECIES	-22.82000	-45.25000	1264872473
1018	Diptera	Culicidae	Aedes	Aedes aegypti	SPECIES	-22.82000	-51.96000	1264872485
1019	Diptera	Culicidae	Aedes	Aedes aegypti	SPECIES	-22.82000	-43.43000	1264872492
1020	Diptera	Culicidae	Aedes	Aedes aegypti	SPECIES	-22.82000	-48.68000	1264872493
1021	Diptera	Culicidae	Aedes	Aedes aegypti	SPECIES	-22.82000	-54.42000	1264872496
1022	Diptera	Culicidae	Aedes	Aedes aegypti	SPECIES	-22.82000	-50.23000	1264872498
1023	Diptera	Culicidae	Aedes	Aedes aegypti	SPECIES	-22.82000	-43.00000	1264872502
1024	Diptera	Culicidae	Aedes	Aedes aegypti	SPECIES	-22.82000	-51.26000	1264872503
1025	Diptera	Culicidae	Aedes	Aedes aegypti	SPECIES	-22.82000	147.63000	1264886381

№	Order	Family	Genus	Species	Taxon Rank	Latitude	Longitude	GBIF_ID
1026	Diptera	Culicidae	Aedes	Aedes aegypti	SPECIES	-22.82000	147.64000	1264887741
1027	Diptera	Culicidae	Aedes	Aedes aegypti	SPECIES	-22.82000	-43.27000	1264888337
1028	Diptera	Culicidae	Aedes	Aedes aegypti	SPECIES	-22.81000	-43.81000	1264872462
1029	Diptera	Culicidae	Aedes	Aedes aegypti	SPECIES	-22.81000	-45.23000	1264872475
1030	Diptera	Culicidae	Aedes	Aedes aegypti	SPECIES	-22.81000	-50.82000	1264872483
1031	Diptera	Culicidae	Aedes	Aedes aegypti	SPECIES	-22.81000	-50.07000	1264872497
1032	Diptera	Culicidae	Aedes	Aedes aegypti	SPECIES	-22.81000	-49.10000	1264872504
1033	Diptera	Culicidae	Aedes	Aedes aegypti	SPECIES	-22.81000	-50.42000	1264872512
1034	Diptera	Culicidae	Aedes	Aedes aegypti	SPECIES	-22.81000	149.89000	1264887731
1035	Diptera	Culicidae	Aedes	Aedes aegypti	SPECIES	-22.81000	-43.33000	1264888344
1036	Diptera	Culicidae	Aedes	Aedes aegypti	SPECIES	-22.81000	-43.32000	1264889477
1037	Diptera	Culicidae	Aedes	Aedes aegypti	SPECIES	-22.81000	-43.19000	1264889945
1038	Diptera	Culicidae	Aedes	Aedes aegypti	SPECIES	-22.81000	-45.31000	1264890394
1039	Diptera	Culicidae	Aedes	Aedes aegypti	SPECIES	-22.80390	-43.37220	1703045332
1040	Diptera	Culicidae	Aedes	Aedes aegypti	SPECIES	-22.80000	-43.45000	1264872507
1041	Diptera	Culicidae	Aedes	Aedes aegypti	SPECIES	-22.80000	-47.44000	1264872514
1042	Diptera	Culicidae	Aedes	Aedes aegypti	SPECIES	-22.80000	-48.16000	1264872518
1043	Diptera	Culicidae	Aedes	Aedes aegypti	SPECIES	-22.80000	-51.72000	1264872536
1044	Diptera	Culicidae	Aedes	Aedes aegypti	SPECIES	-22.80000	-43.29000	1264888332
1045	Diptera	Culicidae	Aedes	Aedes aegypti	SPECIES	-22.80000	-43.18000	1264888353
1046	Diptera	Culicidae	Aedes	Aedes aegypti	SPECIES	-22.80000	-43.35000	1264888359
1047	Diptera	Culicidae	Aedes	Aedes aegypti	SPECIES	-22.80000	-43.42000	1264889658
1048	Diptera	Culicidae	Aedes	Aedes aegypti	SPECIES	-22.80000	-47.28000	1264890929
1049	Diptera	Culicidae	Aedes	Aedes aegypti	SPECIES	-22.80000	-43.41000	1264891367
1050	Diptera	Culicidae	Aedes	Aedes aegypti	SPECIES	-22.80000	-43.36000	1264891377
1051	Diptera	Culicidae	Aedes	Aedes aegypti	SPECIES	-22.79000	-44.10000	1264872524
1052	Diptera	Culicidae	Aedes	Aedes aegypti	SPECIES	-22.79000	-51.56000	1264872552
1053	Diptera	Culicidae	Aedes	Aedes aegypti	SPECIES	-22.79000	-43.23000	1264889227
1054	Diptera	Culicidae	Aedes	Aedes aegypti	SPECIES	-22.79000	-43.17000	1264889441
1055	Diptera	Culicidae	Aedes	Aedes aegypti	SPECIES	-22.79000	-43.37000	1264891095
1056	Diptera	Culicidae	Aedes	Aedes aegypti	SPECIES	-22.78000	-53.19000	1264872499
1057	Diptera	Culicidae	Aedes	Aedes aegypti	SPECIES	-22.78000	-42.14000	1264872506
1058	Diptera	Culicidae	Aedes	Aedes aegypti	SPECIES	-22.78000	-47.28000	1264872534
1059	Diptera	Culicidae	Aedes	Aedes aegypti	SPECIES	-22.78000	-45.05000	1264872540
1060	Diptera	Culicidae	Aedes	Aedes aegypti	SPECIES	-22.78000	-41.94000	1264872545
1061	Diptera	Culicidae	Aedes	Aedes aegypti	SPECIES	-22.78000	-43.17000	1264888317
1062	Diptera	Culicidae	Aedes	Aedes aegypti	SPECIES	-22.78000	-43.30000	1264891368
1063	Diptera	Culicidae	Aedes	Aedes aegypti	SPECIES	-22.77000	-42.72000	1264872521
1064	Diptera	Culicidae	Aedes	Aedes aegypti	SPECIES	-22.77000	-50.61000	1264872531
1065	Diptera	Culicidae	Aedes	Aedes aegypti	SPECIES	-22.77000	-43.31000	1264888358
1066	Diptera	Culicidae	Aedes	Aedes aegypti	SPECIES	-22.77000	-43.44000	1264890539

№	Order	Family	Genus	Species	Taxon Rank	Latitude	Longitude	GBIF_ID
1067	Diptera	Culicidae	Aedes	Aedes aegypti	SPECIES	-22.76000	-46.89000	1264872517
1068	Diptera	Culicidae	Aedes	Aedes aegypti	SPECIES	-22.76000	-49.57000	1264872523
1069	Diptera	Culicidae	Aedes	Aedes aegypti	SPECIES	-22.76000	-43.45000	1264890504
1070	Diptera	Culicidae	Aedes	Aedes aegypti	SPECIES	-22.76000	-43.42000	1264890510
1071	Diptera	Culicidae	Aedes	Aedes aegypti	SPECIES	-22.76000	-43.44000	1264890514
1072	Diptera	Culicidae	Aedes	Aedes aegypti	SPECIES	-22.76000	-43.47000	1264890522
1073	Diptera	Culicidae	Aedes	Aedes aegypti	SPECIES	-22.75000	-49.92000	1264872501
1074	Diptera	Culicidae	Aedes	Aedes aegypti	SPECIES	-22.75000	-42.88000	1264872510
1075	Diptera	Culicidae	Aedes	Aedes aegypti	SPECIES	-22.75000	-47.14000	1264872511
1076	Diptera	Culicidae	Aedes	Aedes aegypti	SPECIES	-22.75000	-52.98000	1264872522
1077	Diptera	Culicidae	Aedes	Aedes aegypti	SPECIES	-22.75000	-43.71000	1264872532
1078	Diptera	Culicidae	Aedes	Aedes aegypti	SPECIES	-22.75000	-43.40000	1264889642
1079	Diptera	Culicidae	Aedes	Aedes aegypti	SPECIES	-22.75000	-43.49000	1264890479
1080	Diptera	Culicidae	Aedes	Aedes aegypti	SPECIES	-22.75000	-43.46000	1264890495
1081	Diptera	Culicidae	Aedes	Aedes aegypti	SPECIES	-22.75000	-43.43000	1264890501
1082	Diptera	Culicidae	Aedes	Aedes aegypti	SPECIES	-22.75000	-43.45000	1264890525
1083	Diptera	Culicidae	Aedes	Aedes aegypti	SPECIES	-22.75000	-43.44000	1264890731
1084	Diptera	Culicidae	Aedes	Aedes aegypti	SPECIES	-22.75000	-42.85000	1264890799
1085	Diptera	Culicidae	Aedes	Aedes aegypti	SPECIES	-22.74000	-51.41000	1264872488
1086	Diptera	Culicidae	Aedes	Aedes aegypti	SPECIES	-22.74000	-51.68000	1264872519
1087	Diptera	Culicidae	Aedes	Aedes aegypti	SPECIES	-22.74000	-42.29000	1264872527
1088	Diptera	Culicidae	Aedes	Aedes aegypti	SPECIES	-22.74000	-53.06000	1264872530
1089	Diptera	Culicidae	Aedes	Aedes aegypti	SPECIES	-22.74000	-42.02000	1264872537
1090	Diptera	Culicidae	Aedes	Aedes aegypti	SPECIES	-22.74000	-45.02000	1264872539
1091	Diptera	Culicidae	Aedes	Aedes aegypti	SPECIES	-22.74000	-44.33000	1264872544
1092	Diptera	Culicidae	Aedes	Aedes aegypti	SPECIES	-22.74000	-50.75000	1264872562
1093	Diptera	Culicidae	Aedes	Aedes aegypti	SPECIES	-22.74000	-42.59000	1264872577
1094	Diptera	Culicidae	Aedes	Aedes aegypti	SPECIES	-22.74000	-64.35000	1264887011
1095	Diptera	Culicidae	Aedes	Aedes aegypti	SPECIES	-22.74000	-43.44000	1264890543
1096	Diptera	Culicidae	Aedes	Aedes aegypti	SPECIES	-22.74000	-43.47000	1264890555
1097	Diptera	Culicidae	Aedes	Aedes aegypti	SPECIES	-22.73000	-51.81000	1264872526
1098	Diptera	Culicidae	Aedes	Aedes aegypti	SPECIES	-22.73000	-43.58000	1264872541
1099	Diptera	Culicidae	Aedes	Aedes aegypti	SPECIES	-22.73000	-52.89000	1264872558
1100	Diptera	Culicidae	Aedes	Aedes aegypti	SPECIES	-22.73000	-43.48000	1264890546
1101	Diptera	Culicidae	Aedes	Aedes aegypti	SPECIES	-22.73000	-43.45000	1264890558
1102	Diptera	Culicidae	Aedes	Aedes aegypti	SPECIES	-22.73000	-47.78000	1264890804
1103	Diptera	Culicidae	Aedes	Aedes aegypti	SPECIES	-22.73000	-43.38000	1264890837
1104	Diptera	Culicidae	Aedes	Aedes aegypti	SPECIES	-22.72000	-51.91000	1264872525
1105	Diptera	Culicidae	Aedes	Aedes aegypti	SPECIES	-22.72000	-52.23000	1264872555
1106	Diptera	Culicidae	Aedes	Aedes aegypti	SPECIES	-22.72000	-47.29000	1264872567
1107	Diptera	Culicidae	Aedes	Aedes aegypti	SPECIES	-22.72000	-52.69000	1264872570

№	Order	Family	Genus	Species	Taxon Rank	Latitude	Longitude	GBIF_ID
1108	Diptera	Culicidae	Aedes	Aedes aegypti	SPECIES	-22.72000	-43.47000	1264890511
1109	Diptera	Culicidae	Aedes	Aedes aegypti	SPECIES	-22.71000	-46.67000	1264872535
1110	Diptera	Culicidae	Aedes	Aedes aegypti	SPECIES	-22.71000	-47.79000	1264872542
1111	Diptera	Culicidae	Aedes	Aedes aegypti	SPECIES	-22.71000	-53.96000	1264872547
1112	Diptera	Culicidae	Aedes	Aedes aegypti	SPECIES	-22.71000	-45.01000	1264872548
1113	Diptera	Culicidae	Aedes	Aedes aegypti	SPECIES	-22.71000	-43.45000	1264888872
1114	Diptera	Culicidae	Aedes	Aedes aegypti	SPECIES	-22.71000	-42.06000	1264890798
1115	Diptera	Culicidae	Aedes	Aedes aegypti	SPECIES	-22.70000	-43.52000	1264872538
1116	Diptera	Culicidae	Aedes	Aedes aegypti	SPECIES	-22.70000	-46.81000	1264872546
1117	Diptera	Culicidae	Aedes	Aedes aegypti	SPECIES	-22.70000	-48.55000	1264872559
1118	Diptera	Culicidae	Aedes	Aedes aegypti	SPECIES	-22.70000	-43.50000	1264890808
1119	Diptera	Culicidae	Aedes	Aedes aegypti	SPECIES	-22.69000	-48.99000	1264872508
1120	Diptera	Culicidae	Aedes	Aedes aegypti	SPECIES	-22.69000	-52.35000	1264872528
1121	Diptera	Culicidae	Aedes	Aedes aegypti	SPECIES	-22.69000	-49.75000	1264872551
1122	Diptera	Culicidae	Aedes	Aedes aegypti	SPECIES	-22.68000	-47.02000	1264872543
1123	Diptera	Culicidae	Aedes	Aedes aegypti	SPECIES	-22.67000	-51.97000	1264872556
1124	Diptera	Culicidae	Aedes	Aedes aegypti	SPECIES	-22.67000	-53.47000	1264872563
1125	Diptera	Culicidae	Aedes	Aedes aegypti	SPECIES	-22.67000	-48.81000	1264872564
1126	Diptera	Culicidae	Aedes	Aedes aegypti	SPECIES	-22.67000	-43.60000	1264872569
1127	Diptera	Culicidae	Aedes	Aedes aegypti	SPECIES	-22.66000	-50.75000	1264872572
1128	Diptera	Culicidae	Aedes	Aedes aegypti	SPECIES	-22.66000	-49.42000	1264872587
1129	Diptera	Culicidae	Aedes	Aedes aegypti	SPECIES	-22.66000	-43.93000	1264872609
1130	Diptera	Culicidae	Aedes	Aedes aegypti	SPECIES	-22.65000	-52.09000	1264872550
1131	Diptera	Culicidae	Aedes	Aedes aegypti	SPECIES	-22.65000	-47.19000	1264872565
1132	Diptera	Culicidae	Aedes	Aedes aegypti	SPECIES	-22.65000	-55.24000	1264872591
1133	Diptera	Culicidae	Aedes	Aedes aegypti	SPECIES	-22.64000	-50.01000	1264872553
1134	Diptera	Culicidae	Aedes	Aedes aegypti	SPECIES	-22.64000	-43.31000	1264872594
1135	Diptera	Culicidae	Aedes	Aedes aegypti	SPECIES	-22.64000	-52.90000	1264872595
1136	Diptera	Culicidae	Aedes	Aedes aegypti	SPECIES	-22.63000	-54.80000	1264872568
1137	Diptera	Culicidae	Aedes	Aedes aegypti	SPECIES	-22.63000	-47.07000	1264872573
1138	Diptera	Culicidae	Aedes	Aedes aegypti	SPECIES	-22.63000	-51.07000	1264872582
1139	Diptera	Culicidae	Aedes	Aedes aegypti	SPECIES	-22.63000	-47.20000	1264890320
1140	Diptera	Culicidae	Aedes	Aedes aegypti	SPECIES	-22.63000	-43.30000	1264891082
1141	Diptera	Culicidae	Aedes	Aedes aegypti	SPECIES	-22.62000	-47.53000	1264872561
1142	Diptera	Culicidae	Aedes	Aedes aegypti	SPECIES	-22.62000	-50.22000	1264872571
1143	Diptera	Culicidae	Aedes	Aedes aegypti	SPECIES	-22.62000	-48.65000	1264872583
1144	Diptera	Culicidae	Aedes	Aedes aegypti	SPECIES	-22.62000	-43.71000	1264888326
1145	Diptera	Culicidae	Aedes	Aedes aegypti	SPECIES	-22.61000	-43.12000	1264872554
1146	Diptera	Culicidae	Aedes	Aedes aegypti	SPECIES	-22.61000	-46.96000	1264872560
1147	Diptera	Culicidae	Aedes	Aedes aegypti	SPECIES	-22.61000	-43.72000	1264872575
1148	Diptera	Culicidae	Aedes	Aedes aegypti	SPECIES	-22.61000	-43.11000	1264890822

№	Order	Family	Genus	Species	Taxon Rank	Latitude	Longitude	GBIF_ID
1149	Diptera	Culicidae	Aedes	Aedes aegypti	SPECIES	-22.60000	-51.20000	1264872533
1150	Diptera	Culicidae	Aedes	Aedes aegypti	SPECIES	-22.60000	-47.88000	1264872576
1151	Diptera	Culicidae	Aedes	Aedes aegypti	SPECIES	-22.60000	-43.43000	1264889145
1152	Diptera	Culicidae	Aedes	Aedes aegypti	SPECIES	-22.60000	-43.71000	1264891297
1153	Diptera	Culicidae	Aedes	Aedes aegypti	SPECIES	-22.59000	-47.36000	1264872566
1154	Diptera	Culicidae	Aedes	Aedes aegypti	SPECIES	-22.59000	-42.96000	1264872589
1155	Diptera	Culicidae	Aedes	Aedes aegypti	SPECIES	-22.58000	-50.44000	1264872579
1156	Diptera	Culicidae	Aedes	Aedes aegypti	SPECIES	-22.58000	-52.08000	1264872580
1157	Diptera	Culicidae	Aedes	Aedes aegypti	SPECIES	-22.58000	-53.76000	1264872585
1158	Diptera	Culicidae	Aedes	Aedes aegypti	SPECIES	-22.58000	-45.18000	1264872611
1159	Diptera	Culicidae	Aedes	Aedes aegypti	SPECIES	-22.57000	-49.30000	1264872597
1160	Diptera	Culicidae	Aedes	Aedes aegypti	SPECIES	-22.57000	-42.39000	1264872630
1161	Diptera	Culicidae	Aedes	Aedes aegypti	SPECIES	-22.57000	144.54000	1264891091
1162	Diptera	Culicidae	Aedes	Aedes aegypti	SPECIES	-22.56000	-49.12000	1264872574
1163	Diptera	Culicidae	Aedes	Aedes aegypti	SPECIES	-22.56000	-48.16000	1264872586
1164	Diptera	Culicidae	Aedes	Aedes aegypti	SPECIES	-22.56000	-45.01000	1264872617
1165	Diptera	Culicidae	Aedes	Aedes aegypti	SPECIES	-22.56000	-47.93000	1264872619
1166	Diptera	Culicidae	Aedes	Aedes aegypti	SPECIES	-22.56000	-47.14000	1264872620
1167	Diptera	Culicidae	Aedes	Aedes aegypti	SPECIES	-22.56000	-44.17000	1264888342
1168	Diptera	Culicidae	Aedes	Aedes aegypti	SPECIES	-22.55000	-49.67000	1264872581
1169	Diptera	Culicidae	Aedes	Aedes aegypti	SPECIES	-22.55000	-63.81000	1264890484
1170	Diptera	Culicidae	Aedes	Aedes aegypti	SPECIES	-22.55000	-48.64000	1264891277
1171	Diptera	Culicidae	Aedes	Aedes aegypti	SPECIES	-22.54000	-43.99000	1264872601
1172	Diptera	Culicidae	Aedes	Aedes aegypti	SPECIES	-22.54000	-48.57000	1264872606
1173	Diptera	Culicidae	Aedes	Aedes aegypti	SPECIES	-22.53000	-47.75000	1264872590
1174	Diptera	Culicidae	Aedes	Aedes aegypti	SPECIES	-22.53000	-43.74000	1264872593
1175	Diptera	Culicidae	Aedes	Aedes aegypti	SPECIES	-22.53000	-43.47000	1264872596
1176	Diptera	Culicidae	Aedes	Aedes aegypti	SPECIES	-22.53000	-63.82000	1264886651
1177	Diptera	Culicidae	Aedes	Aedes aegypti	SPECIES	-22.53000	-55.75000	1264891032
1178	Diptera	Culicidae	Aedes	Aedes aegypti	SPECIES	-22.53000	-63.81000	1264891150
1179	Diptera	Culicidae	Aedes	Aedes aegypti	SPECIES	-22.52000	-42.74000	1264872578
1180	Diptera	Culicidae	Aedes	Aedes aegypti	SPECIES	-22.52000	-43.64000	1264872588
1181	Diptera	Culicidae	Aedes	Aedes aegypti	SPECIES	-22.52000	-44.08000	1264872600
1182	Diptera	Culicidae	Aedes	Aedes aegypti	SPECIES	-22.52000	-51.54000	1264872610
1183	Diptera	Culicidae	Aedes	Aedes aegypti	SPECIES	-22.52000	-63.78000	1264886986
1184	Diptera	Culicidae	Aedes	Aedes aegypti	SPECIES	-22.52000	-63.81000	1264887005
1185	Diptera	Culicidae	Aedes	Aedes aegypti	SPECIES	-22.52000	-44.12000	1264889418
1186	Diptera	Culicidae	Aedes	Aedes aegypti	SPECIES	-22.51000	-52.61000	1264872557
1187	Diptera	Culicidae	Aedes	Aedes aegypti	SPECIES	-22.51000	-44.77000	1264872592
1188	Diptera	Culicidae	Aedes	Aedes aegypti	SPECIES	-22.51000	-46.66000	1264872599
1189	Diptera	Culicidae	Aedes	Aedes aegypti	SPECIES	-22.50000	-51.34000	1264872615

№	Order	Family	Genus	Species	Taxon Rank	Latitude	Longitude	GBIF_ID
1190	Diptera	Culicidae	Aedes	Aedes aegypti	SPECIES	-22.49000	-44.18000	1264872603
1191	Diptera	Culicidae	Aedes	Aedes aegypti	SPECIES	-22.49000	-54.42000	1264872605
1192	Diptera	Culicidae	Aedes	Aedes aegypti	SPECIES	-22.48000	-47.40000	1264872598
1193	Diptera	Culicidae	Aedes	Aedes aegypti	SPECIES	-22.48000	-51.68000	1264872607
1194	Diptera	Culicidae	Aedes	Aedes aegypti	SPECIES	-22.48000	-48.54000	1264872613
1195	Diptera	Culicidae	Aedes	Aedes aegypti	SPECIES	-22.48000	-49.55000	1264872623
1196	Diptera	Culicidae	Aedes	Aedes aegypti	SPECIES	-22.48000	-49.38000	1264872636
1197	Diptera	Culicidae	Aedes	Aedes aegypti	SPECIES	-22.48000	-47.17000	1264872642
1198	Diptera	Culicidae	Aedes	Aedes aegypti	SPECIES	-22.48000	-42.14000	1264872643
1199	Diptera	Culicidae	Aedes	Aedes aegypti	SPECIES	-22.48000	-46.60000	1264872645
1200	Diptera	Culicidae	Aedes	Aedes aegypti	SPECIES	-22.48000	-52.86000	1264872655
1201	Diptera	Culicidae	Aedes	Aedes aegypti	SPECIES	-22.47000	-47.52000	1264872602
1202	Diptera	Culicidae	Aedes	Aedes aegypti	SPECIES	-22.47000	-48.44000	1264872618
1203	Diptera	Culicidae	Aedes	Aedes aegypti	SPECIES	-22.47000	-50.64000	1264872621
1204	Diptera	Culicidae	Aedes	Aedes aegypti	SPECIES	-22.47000	-51.87000	1264872628
1205	Diptera	Culicidae	Aedes	Aedes aegypti	SPECIES	-22.47000	-41.94000	1264872632
1206	Diptera	Culicidae	Aedes	Aedes aegypti	SPECIES	-22.46000	-48.72000	1264872614
1207	Diptera	Culicidae	Aedes	Aedes aegypti	SPECIES	-22.46000	-46.02000	1264872635
1208	Diptera	Culicidae	Aedes	Aedes aegypti	SPECIES	-22.45000	-48.17000	1264872612
1209	Diptera	Culicidae	Aedes	Aedes aegypti	SPECIES	-22.45000	-49.76000	1264872625
1210	Diptera	Culicidae	Aedes	Aedes aegypti	SPECIES	-22.45000	31.33000	1264889062
1211	Diptera	Culicidae	Aedes	Aedes aegypti	SPECIES	-22.44000	-44.42000	1264872584
1212	Diptera	Culicidae	Aedes	Aedes aegypti	SPECIES	-22.44000	-44.32000	1264872608
1213	Diptera	Culicidae	Aedes	Aedes aegypti	SPECIES	-22.44000	-49.81000	1264872616
1214	Diptera	Culicidae	Aedes	Aedes aegypti	SPECIES	-22.44000	-44.57000	1264872624
1215	Diptera	Culicidae	Aedes	Aedes aegypti	SPECIES	-22.44000	-46.75000	1264872631
1216	Diptera	Culicidae	Aedes	Aedes aegypti	SPECIES	-22.44000	-47.00000	1264872640
1217	Diptera	Culicidae	Aedes	Aedes aegypti	SPECIES	-22.44000	-49.92000	1264890628
1218	Diptera	Culicidae	Aedes	Aedes aegypti	SPECIES	-22.44000	-44.49000	1264890790
1219	Diptera	Culicidae	Aedes	Aedes aegypti	SPECIES	-22.43000	-47.72000	1264872627
1220	Diptera	Culicidae	Aedes	Aedes aegypti	SPECIES	-22.43000	-49.96000	1264872634
1221	Diptera	Culicidae	Aedes	Aedes aegypti	SPECIES	-22.43000	-52.40000	1264872648
1222	Diptera	Culicidae	Aedes	Aedes aegypti	SPECIES	-22.43000	-51.57000	1264872660
1223	Diptera	Culicidae	Aedes	Aedes aegypti	SPECIES	-22.43000	-54.19000	1264872680
1224	Diptera	Culicidae	Aedes	Aedes aegypti	SPECIES	-22.42000	-49.19000	1264872622
1225	Diptera	Culicidae	Aedes	Aedes aegypti	SPECIES	-22.42000	-43.92000	1264872638
1226	Diptera	Culicidae	Aedes	Aedes aegypti	SPECIES	-22.42000	-50.21000	1264872667
1227	Diptera	Culicidae	Aedes	Aedes aegypti	SPECIES	-22.40538	-47.54769	3923380371
1228	Diptera	Culicidae	Aedes	Aedes aegypti	SPECIES	-22.40533	-47.54776	3923308452
1229	Diptera	Culicidae	Aedes	Aedes aegypti	SPECIES	-22.40532	-47.54760	3923546338
1230	Diptera	Culicidae	Aedes	Aedes aegypti	SPECIES	-22.40524	-47.54764	3994138847

№	Order	Family	Genus	Species	Taxon Rank	Latitude	Longitude	GBIF_ID
1231	Diptera	Culicidae	Aedes	Aedes aegypti	SPECIES	-22.40000	-47.61000	1264872626
1232	Diptera	Culicidae	Aedes	Aedes aegypti	SPECIES	-22.40000	-48.36000	1264872668
1233	Diptera	Culicidae	Aedes	Aedes aegypti	SPECIES	-22.40000	-49.40000	1264872670
1234	Diptera	Culicidae	Aedes	Aedes aegypti	SPECIES	-22.39000	-43.18000	1264872657
1235	Diptera	Culicidae	Aedes	Aedes aegypti	SPECIES	-22.39000	143.04000	1264887716
1236	Diptera	Culicidae	Aedes	Aedes aegypti	SPECIES	-22.38000	-49.54000	1264872644
1237	Diptera	Culicidae	Aedes	Aedes aegypti	SPECIES	-22.38000	-43.40000	1264872646
1238	Diptera	Culicidae	Aedes	Aedes aegypti	SPECIES	-22.38000	-47.14000	1264872653
1239	Diptera	Culicidae	Aedes	Aedes aegypti	SPECIES	-22.37000	-43.56000	1264872639
1240	Diptera	Culicidae	Aedes	Aedes aegypti	SPECIES	-22.37000	-53.15000	1264872661
1241	Diptera	Culicidae	Aedes	Aedes aegypti	SPECIES	-22.36000	-53.80000	1264872633
1242	Diptera	Culicidae	Aedes	Aedes aegypti	SPECIES	-22.36000	-44.24000	1264872637
1243	Diptera	Culicidae	Aedes	Aedes aegypti	SPECIES	-22.36000	-51.43000	1264872649
1244	Diptera	Culicidae	Aedes	Aedes aegypti	SPECIES	-22.36000	-51.62000	1264872650
1245	Diptera	Culicidae	Aedes	Aedes aegypti	SPECIES	-22.35000	-46.29000	1264872604
1246	Diptera	Culicidae	Aedes	Aedes aegypti	SPECIES	-22.35000	-51.99000	1264872641
1247	Diptera	Culicidae	Aedes	Aedes aegypti	SPECIES	-22.34000	-47.33000	1264872656
1248	Diptera	Culicidae	Aedes	Aedes aegypti	SPECIES	-22.34000	-54.45000	1264872665
1249	Diptera	Culicidae	Aedes	Aedes aegypti	SPECIES	-22.33000	-49.58000	1264872651
1250	Diptera	Culicidae	Aedes	Aedes aegypti	SPECIES	-22.33000	-49.08000	1264890482
1251	Diptera	Culicidae	Aedes	Aedes aegypti	SPECIES	-22.32000	-50.36000	1264872659
1252	Diptera	Culicidae	Aedes	Aedes aegypti	SPECIES	-22.31000	-50.91000	1264872684
1253	Diptera	Culicidae	Aedes	Aedes aegypti	SPECIES	-22.31000	-42.47000	1264872705
1254	Diptera	Culicidae	Aedes	Aedes aegypti	SPECIES	-22.31000	-49.06000	1264888476
1255	Diptera	Culicidae	Aedes	Aedes aegypti	SPECIES	-22.31000	-49.66000	1264889563
1256	Diptera	Culicidae	Aedes	Aedes aegypti	SPECIES	-22.30000	-42.88000	1264872663
1257	Diptera	Culicidae	Aedes	Aedes aegypti	SPECIES	-22.30000	-48.54000	1264872673
1258	Diptera	Culicidae	Aedes	Aedes aegypti	SPECIES	-22.30000	166.45000	1264888535
1259	Diptera	Culicidae	Aedes	Aedes aegypti	SPECIES	-22.30000	166.44000	1264890116
1260	Diptera	Culicidae	Aedes	Aedes aegypti	SPECIES	-22.30000	-41.98000	1264890789
1261	Diptera	Culicidae	Aedes	Aedes aegypti	SPECIES	-22.29000	-47.84000	1264872647
1262	Diptera	Culicidae	Aedes	Aedes aegypti	SPECIES	-22.29000	-50.25000	1264872692
1263	Diptera	Culicidae	Aedes	Aedes aegypti	SPECIES	-22.29000	-48.10000	1264872693
1264	Diptera	Culicidae	Aedes	Aedes aegypti	SPECIES	-22.29000	-48.88000	1264872694
1265	Diptera	Culicidae	Aedes	Aedes aegypti	SPECIES	-22.28000	-46.60000	1264872652
1266	Diptera	Culicidae	Aedes	Aedes aegypti	SPECIES	-22.28000	-41.98000	1264872682
1267	Diptera	Culicidae	Aedes	Aedes aegypti	SPECIES	-22.28000	166.46000	1264886616
1268	Diptera	Culicidae	Aedes	Aedes aegypti	SPECIES	-22.28000	-63.76000	1264889394
1269	Diptera	Culicidae	Aedes	Aedes aegypti	SPECIES	-22.27000	-46.38000	1264872678
1270	Diptera	Culicidae	Aedes	Aedes aegypti	SPECIES	-22.27000	-48.73000	1264890927
1271	Diptera	Culicidae	Aedes	Aedes aegypti	SPECIES	-22.27000	166.45000	1264891107

№	Order	Family	Genus	Species	Taxon Rank	Latitude	Longitude	GBIF_ID
1272	Diptera	Culicidae	Aedes	Aedes aegypti	SPECIES	-22.26667	166.45000	177195199
1273	Diptera	Culicidae	Aedes	Aedes aegypti	SPECIES	-22.26667	166.45000	1633663730
1274	Diptera	Culicidae	Aedes	Aedes aegypti	SPECIES	-22.26000	166.45000	1264891170
1275	Diptera	Culicidae	Aedes	Aedes aegypti	SPECIES	-22.25000	-46.17000	1264872669
1276	Diptera	Culicidae	Aedes	Aedes aegypti	SPECIES	-22.25000	-48.70000	1264872671
1277	Diptera	Culicidae	Aedes	Aedes aegypti	SPECIES	-22.24887	-43.03457	2898482272
1278	Diptera	Culicidae	Aedes	Aedes aegypti	SPECIES	-22.24000	-50.51000	1264872658
1279	Diptera	Culicidae	Aedes	Aedes aegypti	SPECIES	-22.24000	-43.11000	1264872662
1280	Diptera	Culicidae	Aedes	Aedes aegypti	SPECIES	-22.24000	-47.60000	1264872664
1281	Diptera	Culicidae	Aedes	Aedes aegypti	SPECIES	-22.24000	-49.11000	1264872675
1282	Diptera	Culicidae	Aedes	Aedes aegypti	SPECIES	-22.24000	-46.94000	1264872686
1283	Diptera	Culicidae	Aedes	Aedes aegypti	SPECIES	-22.24000	-63.73000	1264887110
1284	Diptera	Culicidae	Aedes	Aedes aegypti	SPECIES	-22.23000	-50.82000	1264872629
1285	Diptera	Culicidae	Aedes	Aedes aegypti	SPECIES	-22.23000	-43.89000	1264872666
1286	Diptera	Culicidae	Aedes	Aedes aegypti	SPECIES	-22.23000	-47.02000	1264872674
1287	Diptera	Culicidae	Aedes	Aedes aegypti	SPECIES	-22.23000	-49.83000	1264872676
1288	Diptera	Culicidae	Aedes	Aedes aegypti	SPECIES	-22.23000	-50.64000	1264872681
1289	Diptera	Culicidae	Aedes	Aedes aegypti	SPECIES	-22.23000	-49.69000	1264872690
1290	Diptera	Culicidae	Aedes	Aedes aegypti	SPECIES	-22.23000	166.44000	1264888531
1291	Diptera	Culicidae	Aedes	Aedes aegypti	SPECIES	-22.22000	-55.99000	1264872685
1292	Diptera	Culicidae	Aedes	Aedes aegypti	SPECIES	-22.22000	-49.95000	1264890451
1293	Diptera	Culicidae	Aedes	Aedes aegypti	SPECIES	-22.21000	-42.38000	1264872709
1294	Diptera	Culicidae	Aedes	Aedes aegypti	SPECIES	-22.21000	-49.93000	1264890729
1295	Diptera	Culicidae	Aedes	Aedes aegypti	SPECIES	-22.20000	-46.61000	1264872672
1296	Diptera	Culicidae	Aedes	Aedes aegypti	SPECIES	-22.20000	-51.30000	1264872688
1297	Diptera	Culicidae	Aedes	Aedes aegypti	SPECIES	-22.20000	-51.11000	1264872698
1298	Diptera	Culicidae	Aedes	Aedes aegypti	SPECIES	-22.20000	-55.54000	1264872716
1299	Diptera	Culicidae	Aedes	Aedes aegypti	SPECIES	-22.20000	-41.66000	1264872730
1300	Diptera	Culicidae	Aedes	Aedes aegypti	SPECIES	-22.20000	-42.66000	1264891042
1301	Diptera	Culicidae	Aedes	Aedes aegypti	SPECIES	-22.19000	-46.79000	1264872677
1302	Diptera	Culicidae	Aedes	Aedes aegypti	SPECIES	-22.19000	-49.31000	1264872717
1303	Diptera	Culicidae	Aedes	Aedes aegypti	SPECIES	-22.19000	-49.96000	1264872719
1304	Diptera	Culicidae	Aedes	Aedes aegypti	SPECIES	-22.18000	-47.34000	1264872703
1305	Diptera	Culicidae	Aedes	Aedes aegypti	SPECIES	-22.18000	-43.30000	1264872708
1306	Diptera	Culicidae	Aedes	Aedes aegypti	SPECIES	-22.18000	-42.94000	1264890793
1307	Diptera	Culicidae	Aedes	Aedes aegypti	SPECIES	-22.18000	-49.98000	1264890803
1308	Diptera	Culicidae	Aedes	Aedes aegypti	SPECIES	-22.17000	-48.79000	1264872689
1309	Diptera	Culicidae	Aedes	Aedes aegypti	SPECIES	-22.17000	-42.90000	1264872695
1310	Diptera	Culicidae	Aedes	Aedes aegypti	SPECIES	-22.17000	-43.54000	1264872696
1311	Diptera	Culicidae	Aedes	Aedes aegypti	SPECIES	-22.17000	-42.95000	1264889424
1312	Diptera	Culicidae	Aedes	Aedes aegypti	SPECIES	-22.16000	-42.13000	1264872687

№	Order	Family	Genus	Species	Taxon Rank	Latitude	Longitude	GBIF_ID
1313	Diptera	Culicidae	Aedes	Aedes aegypti	SPECIES	-22.16000	-54.54000	1264872700
1314	Diptera	Culicidae	Aedes	Aedes aegypti	SPECIES	-22.16000	-41.78000	1264872710
1315	Diptera	Culicidae	Aedes	Aedes aegypti	SPECIES	-22.16000	-43.29000	1264888354
1316	Diptera	Culicidae	Aedes	Aedes aegypti	SPECIES	-22.15000	-52.77000	1264872683
1317	Diptera	Culicidae	Aedes	Aedes aegypti	SPECIES	-22.14000	-54.15000	1264872654
1318	Diptera	Culicidae	Aedes	Aedes aegypti	SPECIES	-22.14000	-50.09000	1264872699
1319	Diptera	Culicidae	Aedes	Aedes aegypti	SPECIES	-22.13000	-51.26000	1264872691
1320	Diptera	Culicidae	Aedes	Aedes aegypti	SPECIES	-22.13000	-47.49000	1264872701
1321	Diptera	Culicidae	Aedes	Aedes aegypti	SPECIES	-22.13000	-47.67000	1264872706
1322	Diptera	Culicidae	Aedes	Aedes aegypti	SPECIES	-22.13000	-46.68000	1264872715
1323	Diptera	Culicidae	Aedes	Aedes aegypti	SPECIES	-22.12457	-51.41132	4034918455
1324	Diptera	Culicidae	Aedes	Aedes aegypti	SPECIES	-22.12000	-49.41000	1264872711
1325	Diptera	Culicidae	Aedes	Aedes aegypti	SPECIES	-22.12000	-51.51000	1264872713
1326	Diptera	Culicidae	Aedes	Aedes aegypti	SPECIES	-22.12000	-43.11000	1264872723
1327	Diptera	Culicidae	Aedes	Aedes aegypti	SPECIES	-22.12000	-48.35000	1264872737
1328	Diptera	Culicidae	Aedes	Aedes aegypti	SPECIES	-22.12000	-42.66000	1264872741
1329	Diptera	Culicidae	Aedes	Aedes aegypti	SPECIES	-22.12000	-52.07000	1264872755
1330	Diptera	Culicidae	Aedes	Aedes aegypti	SPECIES	-22.12000	-43.21000	1264888356
1331	Diptera	Culicidae	Aedes	Aedes aegypti	SPECIES	-22.12000	-51.39000	1264890695
1332	Diptera	Culicidae	Aedes	Aedes aegypti	SPECIES	-22.11000	-51.63000	1264872697
1333	Diptera	Culicidae	Aedes	Aedes aegypti	SPECIES	-22.11000	-43.21000	1264889435
1334	Diptera	Culicidae	Aedes	Aedes aegypti	SPECIES	-22.10000	-41.41000	1264872746
1335	Diptera	Culicidae	Aedes	Aedes aegypti	SPECIES	-22.09000	-45.29000	1264872702
1336	Diptera	Culicidae	Aedes	Aedes aegypti	SPECIES	-22.09000	-48.52000	1264872742
1337	Diptera	Culicidae	Aedes	Aedes aegypti	SPECIES	-22.08000	-48.99000	1264872720
1338	Diptera	Culicidae	Aedes	Aedes aegypti	SPECIES	-22.08000	-50.38000	1264872728
1339	Diptera	Culicidae	Aedes	Aedes aegypti	SPECIES	-22.08000	-49.74000	1264872732
1340	Diptera	Culicidae	Aedes	Aedes aegypti	SPECIES	-22.08000	-43.03000	1264888126
1341	Diptera	Culicidae	Aedes	Aedes aegypti	SPECIES	-22.07000	-46.40000	1264872714
1342	Diptera	Culicidae	Aedes	Aedes aegypti	SPECIES	-22.07000	-53.41000	1264872721
1343	Diptera	Culicidae	Aedes	Aedes aegypti	SPECIES	-22.07000	-63.72000	1264889376
1344	Diptera	Culicidae	Aedes	Aedes aegypti	SPECIES	-22.07000	-42.92000	1264891337
1345	Diptera	Culicidae	Aedes	Aedes aegypti	SPECIES	-22.06000	-47.05000	1264872707
1346	Diptera	Culicidae	Aedes	Aedes aegypti	SPECIES	-22.06000	-53.86000	1264872712
1347	Diptera	Culicidae	Aedes	Aedes aegypti	SPECIES	-22.06000	-48.73000	1264872724
1348	Diptera	Culicidae	Aedes	Aedes aegypti	SPECIES	-22.06000	-46.58000	1264872725
1349	Diptera	Culicidae	Aedes	Aedes aegypti	SPECIES	-22.06000	-42.34000	1264872734
1350	Diptera	Culicidae	Aedes	Aedes aegypti	SPECIES	-22.06000	-63.71000	1264891176
1351	Diptera	Culicidae	Aedes	Aedes aegypti	SPECIES	-22.05000	-43.24000	1264872679
1352	Diptera	Culicidae	Aedes	Aedes aegypti	SPECIES	-22.05000	-63.69000	1264886612
1353	Diptera	Culicidae	Aedes	Aedes aegypti	SPECIES	-22.04000	-48.17000	1264872718

№	Order	Family	Genus	Species	Taxon Rank	Latitude	Longitude	GBIF_ID
1354	Diptera	Culicidae	Aedes	Aedes aegypti	SPECIES	-22.04000	-42.48000	1264872740
1355	Diptera	Culicidae	Aedes	Aedes aegypti	SPECIES	-22.03000	-42.27000	1264872727
1356	Diptera	Culicidae	Aedes	Aedes aegypti	SPECIES	-22.03000	-50.19000	1264872731
1357	Diptera	Culicidae	Aedes	Aedes aegypti	SPECIES	-22.03000	-48.37000	1264872735
1358	Diptera	Culicidae	Aedes	Aedes aegypti	SPECIES	-22.03000	-63.67000	1264886978
1359	Diptera	Culicidae	Aedes	Aedes aegypti	SPECIES	-22.03000	-63.68000	1264888052
1360	Diptera	Culicidae	Aedes	Aedes aegypti	SPECIES	-22.02000	-42.84000	1264872764
1361	Diptera	Culicidae	Aedes	Aedes aegypti	SPECIES	-22.02000	-44.83000	1264872780
1362	Diptera	Culicidae	Aedes	Aedes aegypti	SPECIES	-22.02000	-63.68000	1264887235
1363	Diptera	Culicidae	Aedes	Aedes aegypti	SPECIES	-22.01293	166.22231	3859669680
1364	Diptera	Culicidae	Aedes	Aedes aegypti	SPECIES	-22.01000	-54.59000	1264872738
1365	Diptera	Culicidae	Aedes	Aedes aegypti	SPECIES	-22.01000	-51.72000	1264872747
1366	Diptera	Culicidae	Aedes	Aedes aegypti	SPECIES	-22.01000	166.21000	1264889141
1367	Diptera	Culicidae	Aedes	Aedes aegypti	SPECIES	-22.01000	-50.36000	1264890714
1368	Diptera	Culicidae	Aedes	Aedes aegypti	SPECIES	-22.00000	-54.87000	1264872769
1369	Diptera	Culicidae	Aedes	Aedes aegypti	SPECIES	-21.98000	-43.01000	1264872722
1370	Diptera	Culicidae	Aedes	Aedes aegypti	SPECIES	-21.98000	-49.81000	1264872771
1371	Diptera	Culicidae	Aedes	Aedes aegypti	SPECIES	-21.98000	-63.67000	1264886653
1372	Diptera	Culicidae	Aedes	Aedes aegypti	SPECIES	-21.97000	-46.80000	1264872726
1373	Diptera	Culicidae	Aedes	Aedes aegypti	SPECIES	-21.97000	-47.41000	1264872767
1374	Diptera	Culicidae	Aedes	Aedes aegypti	SPECIES	-21.96000	-50.53000	1264872753
1375	Diptera	Culicidae	Aedes	Aedes aegypti	SPECIES	-21.96000	-51.35000	1264872757
1376	Diptera	Culicidae	Aedes	Aedes aegypti	SPECIES	-21.95000	-48.47000	1264872739
1377	Diptera	Culicidae	Aedes	Aedes aegypti	SPECIES	-21.95000	-48.79000	1264872748
1378	Diptera	Culicidae	Aedes	Aedes aegypti	SPECIES	-21.95000	-50.76000	1264872749
1379	Diptera	Culicidae	Aedes	Aedes aegypti	SPECIES	-21.95000	-41.92000	1264872750
1380	Diptera	Culicidae	Aedes	Aedes aegypti	SPECIES	-21.95000	-51.22000	1264872759
1381	Diptera	Culicidae	Aedes	Aedes aegypti	SPECIES	-21.95000	-51.42000	1264887871
1382	Diptera	Culicidae	Aedes	Aedes aegypti	SPECIES	-21.94000	-48.04000	1264872733
1383	Diptera	Culicidae	Aedes	Aedes aegypti	SPECIES	-21.94000	-50.39000	1264872736
1384	Diptera	Culicidae	Aedes	Aedes aegypti	SPECIES	-21.94000	-51.40000	1264872745
1385	Diptera	Culicidae	Aedes	Aedes aegypti	SPECIES	-21.92000	-56.51000	1264872704
1386	Diptera	Culicidae	Aedes	Aedes aegypti	SPECIES	-21.92000	-46.69000	1264872743
1387	Diptera	Culicidae	Aedes	Aedes aegypti	SPECIES	-21.92000	-50.74000	1264890593
1388	Diptera	Culicidae	Aedes	Aedes aegypti	SPECIES	-21.91000	-49.44000	1264872756
1389	Diptera	Culicidae	Aedes	Aedes aegypti	SPECIES	-21.91000	-49.58000	1264872763
1390	Diptera	Culicidae	Aedes	Aedes aegypti	SPECIES	-21.90000	-57.14000	1264872752
1391	Diptera	Culicidae	Aedes	Aedes aegypti	SPECIES	-21.90000	-45.59000	1264872761
1392	Diptera	Culicidae	Aedes	Aedes aegypti	SPECIES	-21.90000	-49.33000	1264872770
1393	Diptera	Culicidae	Aedes	Aedes aegypti	SPECIES	-21.90000	-42.56000	1264872787
1394	Diptera	Culicidae	Aedes	Aedes aegypti	SPECIES	-21.90000	-49.02000	1264872805

№	Order	Family	Genus	Species	Taxon Rank	Latitude	Longitude	GBIF_ID
1395	Diptera	Culicidae	Aedes	Aedes aegypti	SPECIES	-21.90000	-157.96000	1264890341
1396	Diptera	Culicidae	Aedes	Aedes aegypti	SPECIES	-21.89000	-45.10000	1264872765
1397	Diptera	Culicidae	Aedes	Aedes aegypti	SPECIES	-21.88000	-47.65000	1264872744
1398	Diptera	Culicidae	Aedes	Aedes aegypti	SPECIES	-21.88000	-42.09000	1264872792
1399	Diptera	Culicidae	Aedes	Aedes aegypti	SPECIES	-21.88000	-52.20000	1264872794
1400	Diptera	Culicidae	Aedes	Aedes aegypti	SPECIES	-21.88000	-47.91000	1264872795
1401	Diptera	Culicidae	Aedes	Aedes aegypti	SPECIES	-21.87000	-43.30000	1264872751
1402	Diptera	Culicidae	Aedes	Aedes aegypti	SPECIES	-21.87000	-52.62000	1264872774
1403	Diptera	Culicidae	Aedes	Aedes aegypti	SPECIES	-21.87000	-49.87000	1264872775
1404	Diptera	Culicidae	Aedes	Aedes aegypti	SPECIES	-21.87000	-46.88000	1264872777
1405	Diptera	Culicidae	Aedes	Aedes aegypti	SPECIES	-21.87000	-42.34000	1264872784
1406	Diptera	Culicidae	Aedes	Aedes aegypti	SPECIES	-21.87000	-63.61000	1264887289
1407	Diptera	Culicidae	Aedes	Aedes aegypti	SPECIES	-21.86000	-49.18000	1264872758
1408	Diptera	Culicidae	Aedes	Aedes aegypti	SPECIES	-21.86000	-45.25000	1264872760
1409	Diptera	Culicidae	Aedes	Aedes aegypti	SPECIES	-21.86000	-43.02000	1264872762
1410	Diptera	Culicidae	Aedes	Aedes aegypti	SPECIES	-21.86000	-47.25000	1264872773
1411	Diptera	Culicidae	Aedes	Aedes aegypti	SPECIES	-21.86000	-50.99000	1264872783
1412	Diptera	Culicidae	Aedes	Aedes aegypti	SPECIES	-21.85000	-47.44000	1264872768
1413	Diptera	Culicidae	Aedes	Aedes aegypti	SPECIES	-21.84000	-51.75000	1264872729
1414	Diptera	Culicidae	Aedes	Aedes aegypti	SPECIES	-21.84000	-51.07000	1264872766
1415	Diptera	Culicidae	Aedes	Aedes aegypti	SPECIES	-21.83000	-42.76000	1264872781
1416	Diptera	Culicidae	Aedes	Aedes aegypti	SPECIES	-21.83000	-50.84000	1264872789
1417	Diptera	Culicidae	Aedes	Aedes aegypti	SPECIES	-21.82000	-45.42000	1264872778
1418	Diptera	Culicidae	Aedes	Aedes aegypti	SPECIES	-21.82000	-46.54000	1264872786
1419	Diptera	Culicidae	Aedes	Aedes aegypti	SPECIES	-21.81000	-51.59000	1264872790
1420	Diptera	Culicidae	Aedes	Aedes aegypti	SPECIES	-21.81000	-46.00000	1264872796
1421	Diptera	Culicidae	Aedes	Aedes aegypti	SPECIES	-21.81000	-51.37000	1264872810
1422	Diptera	Culicidae	Aedes	Aedes aegypti	SPECIES	-21.81000	-50.06000	1264872830
1423	Diptera	Culicidae	Aedes	Aedes aegypti	SPECIES	-21.80000	-50.23000	1264872772
1424	Diptera	Culicidae	Aedes	Aedes aegypti	SPECIES	-21.80000	-51.99000	1264872817
1425	Diptera	Culicidae	Aedes	Aedes aegypti	SPECIES	-21.80000	-43.87000	1264872819
1426	Diptera	Culicidae	Aedes	Aedes aegypti	SPECIES	-21.80000	-50.62000	1264872822
1427	Diptera	Culicidae	Aedes	Aedes aegypti	SPECIES	-21.80000	-63.56000	1264887278
1428	Diptera	Culicidae	Aedes	Aedes aegypti	SPECIES	-21.79000	-51.86000	1264872776
1429	Diptera	Culicidae	Aedes	Aedes aegypti	SPECIES	-21.79000	-51.48000	1264872799
1430	Diptera	Culicidae	Aedes	Aedes aegypti	SPECIES	-21.79000	-47.10000	1264872803
1431	Diptera	Culicidae	Aedes	Aedes aegypti	SPECIES	-21.79000	-48.86000	1264872812
1432	Diptera	Culicidae	Aedes	Aedes aegypti	SPECIES	-21.79000	-48.17000	1264888092
1433	Diptera	Culicidae	Aedes	Aedes aegypti	SPECIES	-21.79000	-48.18000	1264891096
1434	Diptera	Culicidae	Aedes	Aedes aegypti	SPECIES	-21.78000	-51.17000	1264872788
1435	Diptera	Culicidae	Aedes	Aedes aegypti	SPECIES	-21.78000	-48.46000	1264872798

№	Order	Family	Genus	Species	Taxon Rank	Latitude	Longitude	GBIF_ID
1436	Diptera	Culicidae	Aedes	Aedes aegypti	SPECIES	-21.78000	-48.56000	1264872807
1437	Diptera	Culicidae	Aedes	Aedes aegypti	SPECIES	-21.78000	-52.15000	1264887867
1438	Diptera	Culicidae	Aedes	Aedes aegypti	SPECIES	-21.77907	-48.17926	3468916613
1439	Diptera	Culicidae	Aedes	Aedes aegypti	SPECIES	-21.77195	-45.96645	3466499967
1440	Diptera	Culicidae	Aedes	Aedes aegypti	SPECIES	-21.77000	-42.56000	1264872782
1441	Diptera	Culicidae	Aedes	Aedes aegypti	SPECIES	-21.77000	-50.42000	1264872785
1442	Diptera	Culicidae	Aedes	Aedes aegypti	SPECIES	-21.77000	-45.65000	1264872800
1443	Diptera	Culicidae	Aedes	Aedes aegypti	SPECIES	-21.77000	-63.57000	1264887267
1444	Diptera	Culicidae	Aedes	Aedes aegypti	SPECIES	-21.77000	-52.11000	1264890584
1445	Diptera	Culicidae	Aedes	Aedes aegypti	SPECIES	-21.76000	-41.09000	1264872754
1446	Diptera	Culicidae	Aedes	Aedes aegypti	SPECIES	-21.76000	-43.42000	1264872791
1447	Diptera	Culicidae	Aedes	Aedes aegypti	SPECIES	-21.76000	-43.02000	1264872793
1448	Diptera	Culicidae	Aedes	Aedes aegypti	SPECIES	-21.76000	-48.17000	1264872804
1449	Diptera	Culicidae	Aedes	Aedes aegypti	SPECIES	-21.76000	-46.93000	1264872806
1450	Diptera	Culicidae	Aedes	Aedes aegypti	SPECIES	-21.76000	-49.31000	1264872815
1451	Diptera	Culicidae	Aedes	Aedes aegypti	SPECIES	-21.76000	-41.33000	1264889449
1452	Diptera	Culicidae	Aedes	Aedes aegypti	SPECIES	-21.75000	-46.75000	1264872813
1453	Diptera	Culicidae	Aedes	Aedes aegypti	SPECIES	-21.75000	-41.30000	1264888167
1454	Diptera	Culicidae	Aedes	Aedes aegypti	SPECIES	-21.75000	-43.36000	1264890712
1455	Diptera	Culicidae	Aedes	Aedes aegypti	SPECIES	-21.75000	-41.40000	1264890791
1456	Diptera	Culicidae	Aedes	Aedes aegypti	SPECIES	-21.75000	-43.46000	1264891016
1457	Diptera	Culicidae	Aedes	Aedes aegypti	SPECIES	-21.74000	-44.96000	1264872835
1458	Diptera	Culicidae	Aedes	Aedes aegypti	SPECIES	-21.73000	-50.96000	1264872801
1459	Diptera	Culicidae	Aedes	Aedes aegypti	SPECIES	-21.73000	-43.10000	1264872814
1460	Diptera	Culicidae	Aedes	Aedes aegypti	SPECIES	-21.73000	-49.56000	1264872823
1461	Diptera	Culicidae	Aedes	Aedes aegypti	SPECIES	-21.73000	-42.09000	1264872844
1462	Diptera	Culicidae	Aedes	Aedes aegypti	SPECIES	-21.73000	-48.62000	1264872848
1463	Diptera	Culicidae	Aedes	Aedes aegypti	SPECIES	-21.73000	-44.28000	1264872853
1464	Diptera	Culicidae	Aedes	Aedes aegypti	SPECIES	-21.72000	-49.35000	1264872797
1465	Diptera	Culicidae	Aedes	Aedes aegypti	SPECIES	-21.72000	-48.03000	1264872842
1466	Diptera	Culicidae	Aedes	Aedes aegypti	SPECIES	-21.71000	-50.86000	1264872824
1467	Diptera	Culicidae	Aedes	Aedes aegypti	SPECIES	-21.71000	-42.47000	1264872837
1468	Diptera	Culicidae	Aedes	Aedes aegypti	SPECIES	-21.70000	-42.96000	1264872809
1469	Diptera	Culicidae	Aedes	Aedes aegypti	SPECIES	-21.70000	-45.22000	1264872820
1470	Diptera	Culicidae	Aedes	Aedes aegypti	SPECIES	-21.70000	-51.38000	1264872828
1471	Diptera	Culicidae	Aedes	Aedes aegypti	SPECIES	-21.69000	-43.21000	1264872832
1472	Diptera	Culicidae	Aedes	Aedes aegypti	SPECIES	-21.68000	-45.90000	1264872808
1473	Diptera	Culicidae	Aedes	Aedes aegypti	SPECIES	-21.68000	-47.51000	1264872811
1474	Diptera	Culicidae	Aedes	Aedes aegypti	SPECIES	-21.68000	-50.72000	1264872825
1475	Diptera	Culicidae	Aedes	Aedes aegypti	SPECIES	-21.67000	-50.35000	1264872779
1476	Diptera	Culicidae	Aedes	Aedes aegypti	SPECIES	-21.67000	-41.75000	1264872816

№	Order	Family	Genus	Species	Taxon Rank	Latitude	Longitude	GBIF_ID
1477	Diptera	Culicidae	Aedes	Aedes aegypti	SPECIES	-21.67000	-44.60000	1264872818
1478	Diptera	Culicidae	Aedes	Aedes aegypti	SPECIES	-21.67000	-48.05000	1264872833
1479	Diptera	Culicidae	Aedes	Aedes aegypti	SPECIES	-21.67000	-46.71000	1264872841
1480	Diptera	Culicidae	Aedes	Aedes aegypti	SPECIES	-21.67000	-49.76000	1264889212
1481	Diptera	Culicidae	Aedes	Aedes aegypti	SPECIES	-21.66000	-42.35000	1264872827
1482	Diptera	Culicidae	Aedes	Aedes aegypti	SPECIES	-21.66000	-50.53000	1264872839
1483	Diptera	Culicidae	Aedes	Aedes aegypti	SPECIES	-21.65000	-49.69000	1264872838
1484	Diptera	Culicidae	Aedes	Aedes aegypti	SPECIES	-21.65000	-41.43000	1264872847
1485	Diptera	Culicidae	Aedes	Aedes aegypti	SPECIES	-21.65000	-42.13000	1264872860
1486	Diptera	Culicidae	Aedes	Aedes aegypti	SPECIES	-21.65000	-54.46000	1264872879
1487	Diptera	Culicidae	Aedes	Aedes aegypti	SPECIES	-21.64000	166.20000	1264889098
1488	Diptera	Culicidae	Aedes	Aedes aegypti	SPECIES	-21.63000	-51.01000	1264872870
1489	Diptera	Culicidae	Aedes	Aedes aegypti	SPECIES	-21.62000	-48.44000	1264872829
1490	Diptera	Culicidae	Aedes	Aedes aegypti	SPECIES	-21.61000	-49.08000	1264872821
1491	Diptera	Culicidae	Aedes	Aedes aegypti	SPECIES	-21.61000	-56.42000	1264872867
1492	Diptera	Culicidae	Aedes	Aedes aegypti	SPECIES	-21.61000	-50.18000	1264872872
1493	Diptera	Culicidae	Aedes	Aedes aegypti	SPECIES	-21.61000	166.20000	1264888533
1494	Diptera	Culicidae	Aedes	Aedes aegypti	SPECIES	-21.61000	166.22000	1264889117
1495	Diptera	Culicidae	Aedes	Aedes aegypti	SPECIES	-21.60000	-47.24000	1264872850
1496	Diptera	Culicidae	Aedes	Aedes aegypti	SPECIES	-21.60000	-46.90000	1264872856
1497	Diptera	Culicidae	Aedes	Aedes aegypti	SPECIES	-21.60000	-45.59000	1264872864
1498	Diptera	Culicidae	Aedes	Aedes aegypti	SPECIES	-21.59000	-43.01000	1264872834
1499	Diptera	Culicidae	Aedes	Aedes aegypti	SPECIES	-21.59000	-43.95000	1264872845
1500	Diptera	Culicidae	Aedes	Aedes aegypti	SPECIES	-21.59000	-50.87000	1264872858
1501	Diptera	Culicidae	Aedes	Aedes aegypti	SPECIES	-21.58000	-48.01000	1264872831
1502	Diptera	Culicidae	Aedes	Aedes aegypti	SPECIES	-21.58000	-50.66000	1264872836
1503	Diptera	Culicidae	Aedes	Aedes aegypti	SPECIES	-21.58000	-50.47000	1264872843
1504	Diptera	Culicidae	Aedes	Aedes aegypti	SPECIES	-21.58000	-51.07000	1264872851
1505	Diptera	Culicidae	Aedes	Aedes aegypti	SPECIES	-21.57000	-42.18000	1264872802
1506	Diptera	Culicidae	Aedes	Aedes aegypti	SPECIES	-21.57000	-45.40000	1264872840
1507	Diptera	Culicidae	Aedes	Aedes aegypti	SPECIES	-21.57000	-45.07000	1264872857
1508	Diptera	Culicidae	Aedes	Aedes aegypti	SPECIES	-21.57000	-45.76000	1264872865
1509	Diptera	Culicidae	Aedes	Aedes aegypti	SPECIES	-21.57000	165.48000	1264889821
1510	Diptera	Culicidae	Aedes	Aedes aegypti	SPECIES	-21.56000	-43.18000	1264872852
1511	Diptera	Culicidae	Aedes	Aedes aegypti	SPECIES	-21.56000	-51.60000	1264872862
1512	Diptera	Culicidae	Aedes	Aedes aegypti	SPECIES	-21.56000	-46.63000	1264872890
1513	Diptera	Culicidae	Aedes	Aedes aegypti	SPECIES	-21.55000	-47.78000	1264872863
1514	Diptera	Culicidae	Aedes	Aedes aegypti	SPECIES	-21.55000	-51.17000	1264872875
1515	Diptera	Culicidae	Aedes	Aedes aegypti	SPECIES	-21.55000	-50.34000	1264872895
1516	Diptera	Culicidae	Aedes	Aedes aegypti	SPECIES	-21.55000	-49.74000	1264872906
1517	Diptera	Culicidae	Aedes	Aedes aegypti	SPECIES	-21.54000	-41.51000	1264872846

№	Order	Family	Genus	Species	Taxon Rank	Latitude	Longitude	GBIF_ID
1518	Diptera	Culicidae	Aedes	Aedes aegypti	SPECIES	-21.54000	-42.66000	1264872854
1519	Diptera	Culicidae	Aedes	Aedes aegypti	SPECIES	-21.54000	-55.98000	1264872889
1520	Diptera	Culicidae	Aedes	Aedes aegypti	SPECIES	-21.54000	-48.79000	1264872897
1521	Diptera	Culicidae	Aedes	Aedes aegypti	SPECIES	-21.52000	-46.25000	1264872869
1522	Diptera	Culicidae	Aedes	Aedes aegypti	SPECIES	-21.52000	-48.17000	1264872877
1523	Diptera	Culicidae	Aedes	Aedes aegypti	SPECIES	-21.52000	-42.45000	1264872883
1524	Diptera	Culicidae	Aedes	Aedes aegypti	SPECIES	-21.52000	-44.90000	1264872887
1525	Diptera	Culicidae	Aedes	Aedes aegypti	SPECIES	-21.51000	-48.35000	1264872859
1526	Diptera	Culicidae	Aedes	Aedes aegypti	SPECIES	-21.51000	-50.57000	1264872882
1527	Diptera	Culicidae	Aedes	Aedes aegypti	SPECIES	-21.50000	-51.74000	1264872855
1528	Diptera	Culicidae	Aedes	Aedes aegypti	SPECIES	-21.50000	-51.28000	1264872861
1529	Diptera	Culicidae	Aedes	Aedes aegypti	SPECIES	-21.50000	-47.36000	1264872874
1530	Diptera	Culicidae	Aedes	Aedes aegypti	SPECIES	-21.49000	-49.81000	1264872868
1531	Diptera	Culicidae	Aedes	Aedes aegypti	SPECIES	-21.48000	-41.91000	1264872826
1532	Diptera	Culicidae	Aedes	Aedes aegypti	SPECIES	-21.48000	-49.57000	1264872866
1533	Diptera	Culicidae	Aedes	Aedes aegypti	SPECIES	-21.48000	-49.31000	1264872876
1534	Diptera	Culicidae	Aedes	Aedes aegypti	SPECIES	-21.48000	-43.15000	1264872880
1535	Diptera	Culicidae	Aedes	Aedes aegypti	SPECIES	-21.48000	-43.50000	1264872893
1536	Diptera	Culicidae	Aedes	Aedes aegypti	SPECIES	-21.47000	-51.35000	1264872885
1537	Diptera	Culicidae	Aedes	Aedes aegypti	SPECIES	-21.47000	-55.33000	1264872899
1538	Diptera	Culicidae	Aedes	Aedes aegypti	SPECIES	-21.47000	-49.95000	1264872914
1539	Diptera	Culicidae	Aedes	Aedes aegypti	SPECIES	-21.47000	-47.60000	1264872933
1540	Diptera	Culicidae	Aedes	Aedes aegypti	SPECIES	-21.47000	-49.22000	1264887857
1541	Diptera	Culicidae	Aedes	Aedes aegypti	SPECIES	-21.46000	-41.67000	1264872871
1542	Diptera	Culicidae	Aedes	Aedes aegypti	SPECIES	-21.46000	-51.86000	1264872878
1543	Diptera	Culicidae	Aedes	Aedes aegypti	SPECIES	-21.46000	-51.43000	1264872888
1544	Diptera	Culicidae	Aedes	Aedes aegypti	SPECIES	-21.46000	-46.73000	1264872915
1545	Diptera	Culicidae	Aedes	Aedes aegypti	SPECIES	-21.46000	-45.82000	1264872921
1546	Diptera	Culicidae	Aedes	Aedes aegypti	SPECIES	-21.46000	-54.16000	1264872922
1547	Diptera	Culicidae	Aedes	Aedes aegypti	SPECIES	-21.45000	-42.97000	1264872884
1548	Diptera	Culicidae	Aedes	Aedes aegypti	SPECIES	-21.45000	-47.01000	1264872894
1549	Diptera	Culicidae	Aedes	Aedes aegypti	SPECIES	-21.45000	-41.14000	1264872898
1550	Diptera	Culicidae	Aedes	Aedes aegypti	SPECIES	-21.45000	-48.00000	1264872907
1551	Diptera	Culicidae	Aedes	Aedes aegypti	SPECIES	-21.45000	-48.37000	1264872910
1552	Diptera	Culicidae	Aedes	Aedes aegypti	SPECIES	-21.45000	-45.20000	1264872913
1553	Diptera	Culicidae	Aedes	Aedes aegypti	SPECIES	-21.44000	-42.33000	1264872886
1554	Diptera	Culicidae	Aedes	Aedes aegypti	SPECIES	-21.44000	-48.52000	1264872904
1555	Diptera	Culicidae	Aedes	Aedes aegypti	SPECIES	-21.44000	47.07000	1264888625
1556	Diptera	Culicidae	Aedes	Aedes aegypti	SPECIES	-21.43000	-42.85000	1264872881
1557	Diptera	Culicidae	Aedes	Aedes aegypti	SPECIES	-21.42000	-50.49000	1264872849
1558	Diptera	Culicidae	Aedes	Aedes aegypti	SPECIES	-21.42000	-44.95000	1264872891

№	Order	Family	Genus	Species	Taxon Rank	Latitude	Longitude	GBIF_ID
1559	Diptera	Culicidae	Aedes	Aedes aegypti	SPECIES	-21.41000	-45.49000	1264872892
1560	Diptera	Culicidae	Aedes	Aedes aegypti	SPECIES	-21.40000	-48.22000	1264872918
1561	Diptera	Culicidae	Aedes	Aedes aegypti	SPECIES	-21.40000	165.82000	1264889785
1562	Diptera	Culicidae	Aedes	Aedes aegypti	SPECIES	-21.40000	-50.06000	1264890747
1563	Diptera	Culicidae	Aedes	Aedes aegypti	SPECIES	-21.39000	-42.15000	1264872902
1564	Diptera	Culicidae	Aedes	Aedes aegypti	SPECIES	-21.39000	-50.13000	1264872903
1565	Diptera	Culicidae	Aedes	Aedes aegypti	SPECIES	-21.39000	-41.94000	1264872909
1566	Diptera	Culicidae	Aedes	Aedes aegypti	SPECIES	-21.38000	-51.60000	1264872938
1567	Diptera	Culicidae	Aedes	Aedes aegypti	SPECIES	-21.37000	-43.05000	1264872912
1568	Diptera	Culicidae	Aedes	Aedes aegypti	SPECIES	-21.37000	-49.07000	1264872925
1569	Diptera	Culicidae	Aedes	Aedes aegypti	SPECIES	-21.37000	-46.52000	1264872954
1570	Diptera	Culicidae	Aedes	Aedes aegypti	SPECIES	-21.36000	-50.28000	1264872905
1571	Diptera	Culicidae	Aedes	Aedes aegypti	SPECIES	-21.36000	-43.26000	1264872946
1572	Diptera	Culicidae	Aedes	Aedes aegypti	SPECIES	-21.36000	-46.00000	1264872951
1573	Diptera	Culicidae	Aedes	Aedes aegypti	SPECIES	-21.35000	-42.46000	1264872896
1574	Diptera	Culicidae	Aedes	Aedes aegypti	SPECIES	-21.35000	-48.07000	1264872924
1575	Diptera	Culicidae	Aedes	Aedes aegypti	SPECIES	-21.35000	-50.75000	1264872929
1576	Diptera	Culicidae	Aedes	Aedes aegypti	SPECIES	-21.35000	-46.94000	1264872940
1577	Diptera	Culicidae	Aedes	Aedes aegypti	SPECIES	-21.35000	-46.13000	1264872941
1578	Diptera	Culicidae	Aedes	Aedes aegypti	SPECIES	-21.35000	-48.23000	1264888418
1579	Diptera	Culicidae	Aedes	Aedes aegypti	SPECIES	-21.34000	-48.63000	1264872908
1580	Diptera	Culicidae	Aedes	Aedes aegypti	SPECIES	-21.34000	-44.71000	1264872919
1581	Diptera	Culicidae	Aedes	Aedes aegypti	SPECIES	-21.34000	-49.51000	1264872936
1582	Diptera	Culicidae	Aedes	Aedes aegypti	SPECIES	-21.33000	-42.80000	1264872911
1583	Diptera	Culicidae	Aedes	Aedes aegypti	SPECIES	-21.33000	-42.65000	1264872927
1584	Diptera	Culicidae	Aedes	Aedes aegypti	SPECIES	-21.32000	-48.83000	1264872873
1585	Diptera	Culicidae	Aedes	Aedes aegypti	SPECIES	-21.32000	-42.88000	1264872901
1586	Diptera	Culicidae	Aedes	Aedes aegypti	SPECIES	-21.32000	-51.65000	1264872916
1587	Diptera	Culicidae	Aedes	Aedes aegypti	SPECIES	-21.32000	-48.69000	1264872917
1588	Diptera	Culicidae	Aedes	Aedes aegypti	SPECIES	-21.32000	-50.86000	1264872928
1589	Diptera	Culicidae	Aedes	Aedes aegypti	SPECIES	-21.32000	-50.21000	1264872943
1590	Diptera	Culicidae	Aedes	Aedes aegypti	SPECIES	-21.31000	-51.74000	1264872931
1591	Diptera	Culicidae	Aedes	Aedes aegypti	SPECIES	-21.31000	-47.77000	1264872934
1592	Diptera	Culicidae	Aedes	Aedes aegypti	SPECIES	-21.31000	-47.56000	1264872963
1593	Diptera	Culicidae	Aedes	Aedes aegypti	SPECIES	-21.31000	-46.32000	1264872981
1594	Diptera	Culicidae	Aedes	Aedes aegypti	SPECIES	-21.30000	-50.31000	1264890767
1595	Diptera	Culicidae	Aedes	Aedes aegypti	SPECIES	-21.30000	165.66000	1264891065
1596	Diptera	Culicidae	Aedes	Aedes aegypti	SPECIES	-21.29000	-47.44000	1264872930
1597	Diptera	Culicidae	Aedes	Aedes aegypti	SPECIES	-21.29000	-49.92000	1264872937
1598	Diptera	Culicidae	Aedes	Aedes aegypti	SPECIES	-21.29000	-46.68000	1264872947
1599	Diptera	Culicidae	Aedes	Aedes aegypti	SPECIES	-21.29000	-45.77000	1264872976

№	Order	Family	Genus	Species	Taxon Rank	Latitude	Longitude	GBIF_ID
1600	Diptera	Culicidae	Aedes	Aedes aegypti	SPECIES	-21.28000	-44.83000	1264872920
1601	Diptera	Culicidae	Aedes	Aedes aegypti	SPECIES	-21.28000	-50.62000	1264872948
1602	Diptera	Culicidae	Aedes	Aedes aegypti	SPECIES	-21.28000	-46.81000	1264872965
1603	Diptera	Culicidae	Aedes	Aedes aegypti	SPECIES	-21.28000	-49.66000	1264872967
1604	Diptera	Culicidae	Aedes	Aedes aegypti	SPECIES	-21.28000	-47.33000	1264872973
1605	Diptera	Culicidae	Aedes	Aedes aegypti	SPECIES	-21.27000	-47.14000	1264872944
1606	Diptera	Culicidae	Aedes	Aedes aegypti	SPECIES	-21.27000	-49.40000	1264872958
1607	Diptera	Culicidae	Aedes	Aedes aegypti	SPECIES	-21.26000	-48.57000	1264872926
1608	Diptera	Culicidae	Aedes	Aedes aegypti	SPECIES	-21.26000	-50.35000	1264872932
1609	Diptera	Culicidae	Aedes	Aedes aegypti	SPECIES	-21.26000	-45.49000	1264872935
1610	Diptera	Culicidae	Aedes	Aedes aegypti	SPECIES	-21.26000	-49.15000	1264872942
1611	Diptera	Culicidae	Aedes	Aedes aegypti	SPECIES	-21.26000	-43.02000	1264872949
1612	Diptera	Culicidae	Aedes	Aedes aegypti	SPECIES	-21.26000	-45.02000	1264872962
1613	Diptera	Culicidae	Aedes	Aedes aegypti	SPECIES	-21.25000	-43.80000	1264872900
1614	Diptera	Culicidae	Aedes	Aedes aegypti	SPECIES	-21.25000	-42.13000	1264872939
1615	Diptera	Culicidae	Aedes	Aedes aegypti	SPECIES	-21.25000	-47.98000	1264872970
1616	Diptera	Culicidae	Aedes	Aedes aegypti	SPECIES	-21.25000	-50.63000	1264890771
1617	Diptera	Culicidae	Aedes	Aedes aegypti	SPECIES	-21.24000	-51.57000	1264872953
1618	Diptera	Culicidae	Aedes	Aedes aegypti	SPECIES	-21.24000	-44.28000	1264872960
1619	Diptera	Culicidae	Aedes	Aedes aegypti	SPECIES	-21.23982	-48.34105	2814184723
1620	Diptera	Culicidae	Aedes	Aedes aegypti	SPECIES	-21.23000	-51.68000	1264872945
1621	Diptera	Culicidae	Aedes	Aedes aegypti	SPECIES	-21.23000	-43.17000	1264872955
1622	Diptera	Culicidae	Aedes	Aedes aegypti	SPECIES	-21.23000	-52.67000	1264872956
1623	Diptera	Culicidae	Aedes	Aedes aegypti	SPECIES	-21.23000	-46.18000	1264872961
1624	Diptera	Culicidae	Aedes	Aedes aegypti	SPECIES	-21.23000	-48.08000	1264872988
1625	Diptera	Culicidae	Aedes	Aedes aegypti	SPECIES	-21.23000	-46.51000	1264873001
1626	Diptera	Culicidae	Aedes	Aedes aegypti	SPECIES	-21.23000	-159.77000	1264890117
1627	Diptera	Culicidae	Aedes	Aedes aegypti	SPECIES	-21.22000	-49.76000	1264872969
1628	Diptera	Culicidae	Aedes	Aedes aegypti	SPECIES	-21.22000	-41.89000	1264872985
1629	Diptera	Culicidae	Aedes	Aedes aegypti	SPECIES	-21.22000	-49.27000	1264872990
1630	Diptera	Culicidae	Aedes	Aedes aegypti	SPECIES	-21.21000	-48.29000	1264872923
1631	Diptera	Culicidae	Aedes	Aedes aegypti	SPECIES	-21.21000	-57.32000	1264872952
1632	Diptera	Culicidae	Aedes	Aedes aegypti	SPECIES	-21.21000	-51.78000	1264872957
1633	Diptera	Culicidae	Aedes	Aedes aegypti	SPECIES	-21.21000	-47.60000	1264872959
1634	Diptera	Culicidae	Aedes	Aedes aegypti	SPECIES	-21.21000	-45.27000	1264872966
1635	Diptera	Culicidae	Aedes	Aedes aegypti	SPECIES	-21.21000	-44.61000	1264872974
1636	Diptera	Culicidae	Aedes	Aedes aegypti	SPECIES	-21.21000	-42.85000	1264872987
1637	Diptera	Culicidae	Aedes	Aedes aegypti	SPECIES	-21.21000	-50.43000	1264888481
1638	Diptera	Culicidae	Aedes	Aedes aegypti	SPECIES	-21.21000	-41.89000	1264889429
1639	Diptera	Culicidae	Aedes	Aedes aegypti	SPECIES	-21.21000	-50.45000	1264890513
1640	Diptera	Culicidae	Aedes	Aedes aegypti	SPECIES	-21.21000	-44.82000	1264890925

№	Order	Family	Genus	Species	Taxon Rank	Latitude	Longitude	GBIF_ID
1641	Diptera	Culicidae	Aedes	Aedes aegypti	SPECIES	-21.21000	-47.82000	1264890977
1642	Diptera	Culicidae	Aedes	Aedes aegypti	SPECIES	-21.20000	-46.95000	1264872964
1643	Diptera	Culicidae	Aedes	Aedes aegypti	SPECIES	-21.20000	-48.93000	1264872979
1644	Diptera	Culicidae	Aedes	Aedes aegypti	SPECIES	-21.20000	-49.57000	1264872995
1645	Diptera	Culicidae	Aedes	Aedes aegypti	SPECIES	-21.20000	-50.41000	1264890718
1646	Diptera	Culicidae	Aedes	Aedes aegypti	SPECIES	-21.20000	-47.81000	1264890930
1647	Diptera	Culicidae	Aedes	Aedes aegypti	SPECIES	-21.19000	-50.94000	1264872983
1648	Diptera	Culicidae	Aedes	Aedes aegypti	SPECIES	-21.19000	-47.80000	1264890746
1649	Diptera	Culicidae	Aedes	Aedes aegypti	SPECIES	-21.18000	-43.02000	1264872968
1650	Diptera	Culicidae	Aedes	Aedes aegypti	SPECIES	-21.18000	-48.65000	1264872972
1651	Diptera	Culicidae	Aedes	Aedes aegypti	SPECIES	-21.18000	-42.78000	1264872982
1652	Diptera	Culicidae	Aedes	Aedes aegypti	SPECIES	-21.18000	-50.21000	1264872986
1653	Diptera	Culicidae	Aedes	Aedes aegypti	SPECIES	-21.18000	-48.78000	1264872994
1654	Diptera	Culicidae	Aedes	Aedes aegypti	SPECIES	-21.18000	-43.35000	1264872996
1655	Diptera	Culicidae	Aedes	Aedes aegypti	SPECIES	-21.18000	-45.42000	1264873016
1656	Diptera	Culicidae	Aedes	Aedes aegypti	SPECIES	-21.18000	55.31000	1264886083
1657	Diptera	Culicidae	Aedes	Aedes aegypti	SPECIES	-21.17000	-44.93000	1264872980
1658	Diptera	Culicidae	Aedes	Aedes aegypti	SPECIES	-21.17000	-42.26000	1264872989
1659	Diptera	Culicidae	Aedes	Aedes aegypti	SPECIES	-21.17000	-47.81000	1264888449
1660	Diptera	Culicidae	Aedes	Aedes aegypti	SPECIES	-21.16000	-41.08000	1264873013
1661	Diptera	Culicidae	Aedes	Aedes aegypti	SPECIES	-21.16000	149.17000	1264887743
1662	Diptera	Culicidae	Aedes	Aedes aegypti	SPECIES	-21.16000	-47.80000	1264891230
1663	Diptera	Culicidae	Aedes	Aedes aegypti	SPECIES	-21.15000	-51.04000	1264872978
1664	Diptera	Culicidae	Aedes	Aedes aegypti	SPECIES	-21.15000	-45.08000	1264872999
1665	Diptera	Culicidae	Aedes	Aedes aegypti	SPECIES	-21.15000	-49.09000	1264873002
1666	Diptera	Culicidae	Aedes	Aedes aegypti	SPECIES	-21.15000	-175.22000	1264886025
1667	Diptera	Culicidae	Aedes	Aedes aegypti	SPECIES	-21.15000	149.17000	1264887757
1668	Diptera	Culicidae	Aedes	Aedes aegypti	SPECIES	-21.15000	-44.49000	1264891038
1669	Diptera	Culicidae	Aedes	Aedes aegypti	SPECIES	-21.14000	-50.49000	1264872950
1670	Diptera	Culicidae	Aedes	Aedes aegypti	SPECIES	-21.14000	-42.63000	1264872977
1671	Diptera	Culicidae	Aedes	Aedes aegypti	SPECIES	-21.14000	-48.42000	1264872991
1672	Diptera	Culicidae	Aedes	Aedes aegypti	SPECIES	-21.14000	-51.52000	1264873020
1673	Diptera	Culicidae	Aedes	Aedes aegypti	SPECIES	-21.14000	-48.97000	1264888163
1674	Diptera	Culicidae	Aedes	Aedes aegypti	SPECIES	-21.14000	-175.22000	1264888764
1675	Diptera	Culicidae	Aedes	Aedes aegypti	SPECIES	-21.13960	-175.20100	1843579034
1676	Diptera	Culicidae	Aedes	Aedes aegypti	SPECIES	-21.13449	164.91295	3031948398
1677	Diptera	Culicidae	Aedes	Aedes aegypti	SPECIES	-21.13317	55.53241	4050529993
1678	Diptera	Culicidae	Aedes	Aedes aegypti	SPECIES	-21.13000	-48.54000	1264873005
1679	Diptera	Culicidae	Aedes	Aedes aegypti	SPECIES	-21.13000	-48.95000	1264873006
1680	Diptera	Culicidae	Aedes	Aedes aegypti	SPECIES	-21.13000	164.89000	1264889825
1681	Diptera	Culicidae	Aedes	Aedes aegypti	SPECIES	-21.13000	-48.96000	1264891197

№	Order	Family	Genus	Species	Taxon Rank	Latitude	Longitude	GBIF_ID
1682	Diptera	Culicidae	Aedes	Aedes aegypti	SPECIES	-21.13000	-175.20000	1264891265
1683	Diptera	Culicidae	Aedes	Aedes aegypti	SPECIES	-21.12000	-55.83000	1264872984
1684	Diptera	Culicidae	Aedes	Aedes aegypti	SPECIES	-21.12000	-44.22000	1264872997
1685	Diptera	Culicidae	Aedes	Aedes aegypti	SPECIES	-21.12000	-44.16000	1264873037
1686	Diptera	Culicidae	Aedes	Aedes aegypti	SPECIES	-21.12000	55.54000	1264886346
1687	Diptera	Culicidae	Aedes	Aedes aegypti	SPECIES	-21.11000	-50.05000	1264872993
1688	Diptera	Culicidae	Aedes	Aedes aegypti	SPECIES	-21.11000	-45.83000	1264873003
1689	Diptera	Culicidae	Aedes	Aedes aegypti	SPECIES	-21.11000	-48.03000	1264873010
1690	Diptera	Culicidae	Aedes	Aedes aegypti	SPECIES	-21.11000	-42.98000	1264873022
1691	Diptera	Culicidae	Aedes	Aedes aegypti	SPECIES	-21.11000	-50.57000	1264890782
1692	Diptera	Culicidae	Aedes	Aedes aegypti	SPECIES	-21.10694	55.25912	3986363438
1693	Diptera	Culicidae	Aedes	Aedes aegypti	SPECIES	-21.10000	-49.77000	1264872975
1694	Diptera	Culicidae	Aedes	Aedes aegypti	SPECIES	-21.10000	-46.21000	1264873004
1695	Diptera	Culicidae	Aedes	Aedes aegypti	SPECIES	-21.10000	-48.83000	1264873009
1696	Diptera	Culicidae	Aedes	Aedes aegypti	SPECIES	-21.09000	-42.41000	1264872992
1697	Diptera	Culicidae	Aedes	Aedes aegypti	SPECIES	-21.09000	-54.91000	1264873007
1698	Diptera	Culicidae	Aedes	Aedes aegypti	SPECIES	-21.09000	-51.11000	1264873012
1699	Diptera	Culicidae	Aedes	Aedes aegypti	SPECIES	-21.09000	-40.88000	1264873015
1700	Diptera	Culicidae	Aedes	Aedes aegypti	SPECIES	-21.09000	-41.78000	1264873045
1701	Diptera	Culicidae	Aedes	Aedes aegypti	SPECIES	-21.08000	-49.39000	1264872998
1702	Diptera	Culicidae	Aedes	Aedes aegypti	SPECIES	-21.08000	-47.21000	1264873011
1703	Diptera	Culicidae	Aedes	Aedes aegypti	SPECIES	-21.08000	-48.67000	1264873014
1704	Diptera	Culicidae	Aedes	Aedes aegypti	SPECIES	-21.08000	-47.04000	1264873027
1705	Diptera	Culicidae	Aedes	Aedes aegypti	SPECIES	-21.07000	-41.36000	1264873000
1706	Diptera	Culicidae	Aedes	Aedes aegypti	SPECIES	-21.07000	-49.05000	1264873008
1707	Diptera	Culicidae	Aedes	Aedes aegypti	SPECIES	-21.07000	-48.39000	1264873018
1708	Diptera	Culicidae	Aedes	Aedes aegypti	SPECIES	-21.07000	-51.30000	1264873023
1709	Diptera	Culicidae	Aedes	Aedes aegypti	SPECIES	-21.07000	-41.60000	1264873025
1710	Diptera	Culicidae	Aedes	Aedes aegypti	SPECIES	-21.07000	-41.57000	1264873039
1711	Diptera	Culicidae	Aedes	Aedes aegypti	SPECIES	-21.07000	-49.22000	1264873040
1712	Diptera	Culicidae	Aedes	Aedes aegypti	SPECIES	-21.07000	31.67000	1264889845
1713	Diptera	Culicidae	Aedes	Aedes aegypti	SPECIES	-21.06000	-45.06000	1264873026
1714	Diptera	Culicidae	Aedes	Aedes aegypti	SPECIES	-21.06000	-50.21000	1264873031
1715	Diptera	Culicidae	Aedes	Aedes aegypti	SPECIES	-21.06000	-45.66000	1264873036
1716	Diptera	Culicidae	Aedes	Aedes aegypti	SPECIES	-21.06000	-52.49000	1264873060
1717	Diptera	Culicidae	Aedes	Aedes aegypti	SPECIES	-21.06000	164.85000	1264889836
1718	Diptera	Culicidae	Aedes	Aedes aegypti	SPECIES	-21.05000	-49.53000	1264873030
1719	Diptera	Culicidae	Aedes	Aedes aegypti	SPECIES	-21.05000	31.67000	1264889587
1720	Diptera	Culicidae	Aedes	Aedes aegypti	SPECIES	-21.05000	-49.68000	1264890730
1721	Diptera	Culicidae	Aedes	Aedes aegypti	SPECIES	-21.04000	-47.62000	1264873029
1722	Diptera	Culicidae	Aedes	Aedes aegypti	SPECIES	-21.04000	-41.94000	1264873047

№	Order	Family	Genus	Species	Taxon Rank	Latitude	Longitude	GBIF_ID
1723	Diptera	Culicidae	Aedes	Aedes aegypti	SPECIES	-21.04000	-49.38000	1264887854
1724	Diptera	Culicidae	Aedes	Aedes aegypti	SPECIES	-21.03000	-45.19000	1264873033
1725	Diptera	Culicidae	Aedes	Aedes aegypti	SPECIES	-21.02000	-49.94000	1264873017
1726	Diptera	Culicidae	Aedes	Aedes aegypti	SPECIES	-21.02000	-46.75000	1264873019
1727	Diptera	Culicidae	Aedes	Aedes aegypti	SPECIES	-21.02000	-44.80000	1264873021
1728	Diptera	Culicidae	Aedes	Aedes aegypti	SPECIES	-21.02000	-42.85000	1264873028
1729	Diptera	Culicidae	Aedes	Aedes aegypti	SPECIES	-21.02000	-42.70000	1264873032
1730	Diptera	Culicidae	Aedes	Aedes aegypti	SPECIES	-21.02000	-48.77000	1264873070
1731	Diptera	Culicidae	Aedes	Aedes aegypti	SPECIES	-21.01000	-47.41000	1264873034
1732	Diptera	Culicidae	Aedes	Aedes aegypti	SPECIES	-21.01000	-46.53000	1264873041
1733	Diptera	Culicidae	Aedes	Aedes aegypti	SPECIES	-21.00000	-47.84000	1264873038
1734	Diptera	Culicidae	Aedes	Aedes aegypti	SPECIES	-21.00000	-42.21000	1264873054
1735	Diptera	Culicidae	Aedes	Aedes aegypti	SPECIES	-21.00000	-51.30000	1264873063
1736	Diptera	Culicidae	Aedes	Aedes aegypti	SPECIES	-21.00000	-48.28000	1264873064
1737	Diptera	Culicidae	Aedes	Aedes aegypti	SPECIES	-20.99000	-48.06000	1264873024
1738	Diptera	Culicidae	Aedes	Aedes aegypti	SPECIES	-20.99000	-40.94000	1264873035
1739	Diptera	Culicidae	Aedes	Aedes aegypti	SPECIES	-20.99000	-48.91000	1264873042
1740	Diptera	Culicidae	Aedes	Aedes aegypti	SPECIES	-20.99000	-43.00000	1264873049
1741	Diptera	Culicidae	Aedes	Aedes aegypti	SPECIES	-20.99000	-46.12000	1264873050
1742	Diptera	Culicidae	Aedes	Aedes aegypti	SPECIES	-20.98000	-45.44000	1264873053
1743	Diptera	Culicidae	Aedes	Aedes aegypti	SPECIES	-20.98000	-41.65000	1264873061
1744	Diptera	Culicidae	Aedes	Aedes aegypti	SPECIES	-20.97000	-41.19000	1264873085
1745	Diptera	Culicidae	Aedes	Aedes aegypti	SPECIES	-20.96000	-56.48000	1264873051
1746	Diptera	Culicidae	Aedes	Aedes aegypti	SPECIES	-20.95000	-48.85000	1264873055
1747	Diptera	Culicidae	Aedes	Aedes aegypti	SPECIES	-20.95000	-50.24000	1264873057
1748	Diptera	Culicidae	Aedes	Aedes aegypti	SPECIES	-20.95000	-49.17000	1264890616
1749	Diptera	Culicidae	Aedes	Aedes aegypti	SPECIES	-20.94000	-45.25000	1264873044
1750	Diptera	Culicidae	Aedes	Aedes aegypti	SPECIES	-20.94000	-45.81000	1264873046
1751	Diptera	Culicidae	Aedes	Aedes aegypti	SPECIES	-20.94000	-48.53000	1264873052
1752	Diptera	Culicidae	Aedes	Aedes aegypti	SPECIES	-20.94000	-49.02000	1264873058
1753	Diptera	Culicidae	Aedes	Aedes aegypti	SPECIES	-20.94000	-49.58000	1264873059
1754	Diptera	Culicidae	Aedes	Aedes aegypti	SPECIES	-20.94000	-50.10000	1264873065
1755	Diptera	Culicidae	Aedes	Aedes aegypti	SPECIES	-20.94000	-44.58000	1264873072
1756	Diptera	Culicidae	Aedes	Aedes aegypti	SPECIES	-20.93000	-49.17000	1264873043
1757	Diptera	Culicidae	Aedes	Aedes aegypti	SPECIES	-20.93000	-41.35000	1264873067
1758	Diptera	Culicidae	Aedes	Aedes aegypti	SPECIES	-20.93000	-49.43000	1264873098
1759	Diptera	Culicidae	Aedes	Aedes aegypti	SPECIES	-20.92000	-44.94000	1264873062
1760	Diptera	Culicidae	Aedes	Aedes aegypti	SPECIES	-20.92000	-49.26000	1264873089
1761	Diptera	Culicidae	Aedes	Aedes aegypti	SPECIES	-20.91000	-42.83000	1264873048
1762	Diptera	Culicidae	Aedes	Aedes aegypti	SPECIES	-20.91000	-48.69000	1264873076
1763	Diptera	Culicidae	Aedes	Aedes aegypti	SPECIES	-20.91000	-54.25000	1264890989

№	Order	Family	Genus	Species	Taxon Rank	Latitude	Longitude	GBIF_ID
1764	Diptera	Culicidae	Aedes	Aedes aegypti	SPECIES	-20.90000	-43.10000	1264873074
1765	Diptera	Culicidae	Aedes	Aedes aegypti	SPECIES	-20.90000	-51.60000	1264873075
1766	Diptera	Culicidae	Aedes	Aedes aegypti	SPECIES	-20.90000	-47.02000	1264873088
1767	Diptera	Culicidae	Aedes	Aedes aegypti	SPECIES	-20.89000	-41.96000	1264873056
1768	Diptera	Culicidae	Aedes	Aedes aegypti	SPECIES	-20.89000	-44.06000	1264873069
1769	Diptera	Culicidae	Aedes	Aedes aegypti	SPECIES	-20.89000	-41.83000	1264873080
1770	Diptera	Culicidae	Aedes	Aedes aegypti	SPECIES	-20.89000	-49.90000	1264873084
1771	Diptera	Culicidae	Aedes	Aedes aegypti	SPECIES	-20.89000	-42.06000	1264873086
1772	Diptera	Culicidae	Aedes	Aedes aegypti	SPECIES	-20.89000	-49.78000	1264873110
1773	Diptera	Culicidae	Aedes	Aedes aegypti	SPECIES	-20.89000	-46.77000	1264873122
1774	Diptera	Culicidae	Aedes	Aedes aegypti	SPECIES	-20.88000	-45.07000	1264873066
1775	Diptera	Culicidae	Aedes	Aedes aegypti	SPECIES	-20.88000	-54.26000	1264873078
1776	Diptera	Culicidae	Aedes	Aedes aegypti	SPECIES	-20.88000	-50.09000	1264873082
1777	Diptera	Culicidae	Aedes	Aedes aegypti	SPECIES	-20.88000	-48.85000	1264873083
1778	Diptera	Culicidae	Aedes	Aedes aegypti	SPECIES	-20.88000	-49.64000	1264873087
1779	Diptera	Culicidae	Aedes	Aedes aegypti	SPECIES	-20.88000	-48.31000	1264873095
1780	Diptera	Culicidae	Aedes	Aedes aegypti	SPECIES	-20.87000	-42.98000	1264873068
1781	Diptera	Culicidae	Aedes	Aedes aegypti	SPECIES	-20.87000	-50.57000	1264873071
1782	Diptera	Culicidae	Aedes	Aedes aegypti	SPECIES	-20.87000	-47.60000	1264873077
1783	Diptera	Culicidae	Aedes	Aedes aegypti	SPECIES	-20.87000	-51.31000	1264873079
1784	Diptera	Culicidae	Aedes	Aedes aegypti	SPECIES	-20.87000	-50.25000	1264873090
1785	Diptera	Culicidae	Aedes	Aedes aegypti	SPECIES	-20.87000	-42.60000	1264873091
1786	Diptera	Culicidae	Aedes	Aedes aegypti	SPECIES	-20.86000	-44.30000	1264873101
1787	Diptera	Culicidae	Aedes	Aedes aegypti	SPECIES	-20.85000	144.20000	177194553
1788	Diptera	Culicidae	Aedes	Aedes aegypti	SPECIES	-20.85000	-49.98000	1264873073
1789	Diptera	Culicidae	Aedes	Aedes aegypti	SPECIES	-20.85000	-42.39000	1264873111
1790	Diptera	Culicidae	Aedes	Aedes aegypti	SPECIES	-20.85000	144.20000	1633663658
1791	Diptera	Culicidae	Aedes	Aedes aegypti	SPECIES	-20.84000	-40.76000	1264873119
1792	Diptera	Culicidae	Aedes	Aedes aegypti	SPECIES	-20.84000	144.20000	1264887738
1793	Diptera	Culicidae	Aedes	Aedes aegypti	SPECIES	-20.83000	-49.51000	1264873108
1794	Diptera	Culicidae	Aedes	Aedes aegypti	SPECIES	-20.83000	-46.39000	1264873113
1795	Diptera	Culicidae	Aedes	Aedes aegypti	SPECIES	-20.83000	-47.85000	1264873120
1796	Diptera	Culicidae	Aedes	Aedes aegypti	SPECIES	-20.82000	-49.52000	1264890615
1797	Diptera	Culicidae	Aedes	Aedes aegypti	SPECIES	-20.81000	-45.56000	1264873092
1798	Diptera	Culicidae	Aedes	Aedes aegypti	SPECIES	-20.81000	-50.20000	1264873105
1799	Diptera	Culicidae	Aedes	Aedes aegypti	SPECIES	-20.81000	-41.39000	1264873112
1800	Diptera	Culicidae	Aedes	Aedes aegypti	SPECIES	-20.81000	-49.38000	1264890570
1801	Diptera	Culicidae	Aedes	Aedes aegypti	SPECIES	-20.80000	-49.39000	1264887946
1802	Diptera	Culicidae	Aedes	Aedes aegypti	SPECIES	-20.80000	-49.38000	1264890726
1803	Diptera	Culicidae	Aedes	Aedes aegypti	SPECIES	-20.79000	-48.80000	1264873099
1804	Diptera	Culicidae	Aedes	Aedes aegypti	SPECIES	-20.79000	-42.05000	1264873102

№	Order	Family	Genus	Species	Taxon Rank	Latitude	Longitude	GBIF_ID
1805	Diptera	Culicidae	Aedes	Aedes aegypti	SPECIES	-20.79000	-41.72000	1264873106
1806	Diptera	Culicidae	Aedes	Aedes aegypti	SPECIES	-20.79000	-51.71000	1264890575
1807	Diptera	Culicidae	Aedes	Aedes aegypti	SPECIES	-20.78000	-40.96000	1264873081
1808	Diptera	Culicidae	Aedes	Aedes aegypti	SPECIES	-20.78000	-41.20000	1264873100
1809	Diptera	Culicidae	Aedes	Aedes aegypti	SPECIES	-20.78000	-49.36000	1264873103
1810	Diptera	Culicidae	Aedes	Aedes aegypti	SPECIES	-20.78000	-46.85000	1264873135
1811	Diptera	Culicidae	Aedes	Aedes aegypti	SPECIES	-20.78000	-50.35000	1264873140
1812	Diptera	Culicidae	Aedes	Aedes aegypti	SPECIES	-20.78000	-47.13000	1264873146
1813	Diptera	Culicidae	Aedes	Aedes aegypti	SPECIES	-20.77000	-48.35000	1264873094
1814	Diptera	Culicidae	Aedes	Aedes aegypti	SPECIES	-20.77000	-55.61000	1264873118
1815	Diptera	Culicidae	Aedes	Aedes aegypti	SPECIES	-20.77000	-49.71000	1264887865
1816	Diptera	Culicidae	Aedes	Aedes aegypti	SPECIES	-20.76000	-40.86000	1264873096
1817	Diptera	Culicidae	Aedes	Aedes aegypti	SPECIES	-20.76000	-46.24000	1264873104
1818	Diptera	Culicidae	Aedes	Aedes aegypti	SPECIES	-20.75000	-45.28000	1264873093
1819	Diptera	Culicidae	Aedes	Aedes aegypti	SPECIES	-20.75000	-42.88000	1264873114
1820	Diptera	Culicidae	Aedes	Aedes aegypti	SPECIES	-20.75000	-44.69000	1264873126
1821	Diptera	Culicidae	Aedes	Aedes aegypti	SPECIES	-20.74312	40.83955	4050507305
1822	Diptera	Culicidae	Aedes	Aedes aegypti	SPECIES	-20.74000	-48.59000	1264873097
1823	Diptera	Culicidae	Aedes	Aedes aegypti	SPECIES	-20.74000	-49.83000	1264873117
1824	Diptera	Culicidae	Aedes	Aedes aegypti	SPECIES	-20.74000	-45.92000	1264873137
1825	Diptera	Culicidae	Aedes	Aedes aegypti	SPECIES	-20.73000	-49.69000	1264873115
1826	Diptera	Culicidae	Aedes	Aedes aegypti	SPECIES	-20.73000	-41.50000	1264873124
1827	Diptera	Culicidae	Aedes	Aedes aegypti	SPECIES	-20.73000	-46.62000	1264873136
1828	Diptera	Culicidae	Aedes	Aedes aegypti	SPECIES	-20.73000	-46.77000	1264873143
1829	Diptera	Culicidae	Aedes	Aedes aegypti	SPECIES	-20.73000	143.14000	1264889093
1830	Diptera	Culicidae	Aedes	Aedes aegypti	SPECIES	-20.72000	-49.19000	1264873132
1831	Diptera	Culicidae	Aedes	Aedes aegypti	SPECIES	-20.72000	139.49000	1264887739
1832	Diptera	Culicidae	Aedes	Aedes aegypti	SPECIES	-20.71000	-42.11000	1264873127
1833	Diptera	Culicidae	Aedes	Aedes aegypti	SPECIES	-20.71000	-48.96000	1264873164
1834	Diptera	Culicidae	Aedes	Aedes aegypti	SPECIES	-20.70000	-49.56000	1264873109
1835	Diptera	Culicidae	Aedes	Aedes aegypti	SPECIES	-20.70000	-47.90000	1264873134
1836	Diptera	Culicidae	Aedes	Aedes aegypti	SPECIES	-20.70000	-47.72000	1264873160
1837	Diptera	Culicidae	Aedes	Aedes aegypti	SPECIES	-20.70000	-44.11000	1264873169
1838	Diptera	Culicidae	Aedes	Aedes aegypti	SPECIES	-20.70000	140.51000	1264887717
1839	Diptera	Culicidae	Aedes	Aedes aegypti	SPECIES	-20.69000	-48.17000	1264873107
1840	Diptera	Culicidae	Aedes	Aedes aegypti	SPECIES	-20.69000	-44.99000	1264873125
1841	Diptera	Culicidae	Aedes	Aedes aegypti	SPECIES	-20.69000	-40.71000	1264873149
1842	Diptera	Culicidae	Aedes	Aedes aegypti	SPECIES	-20.69000	-47.30000	1264873175
1843	Diptera	Culicidae	Aedes	Aedes aegypti	SPECIES	-20.68430	-43.29743	3456943321
1844	Diptera	Culicidae	Aedes	Aedes aegypti	SPECIES	-20.68000	-51.09000	1264873133
1845	Diptera	Culicidae	Aedes	Aedes aegypti	SPECIES	-20.68000	-50.91000	1264873158

№	Order	Family	Genus	Species	Taxon Rank	Latitude	Longitude	GBIF_ID
1846	Diptera	Culicidae	Aedes	Aedes aegypti	SPECIES	-20.67000	-42.35000	1264873129
1847	Diptera	Culicidae	Aedes	Aedes aegypti	SPECIES	-20.67000	-50.03000	1264873153
1848	Diptera	Culicidae	Aedes	Aedes aegypti	SPECIES	-20.67000	-43.08000	1264873159
1849	Diptera	Culicidae	Aedes	Aedes aegypti	SPECIES	-20.67000	-43.80000	1264873178
1850	Diptera	Culicidae	Aedes	Aedes aegypti	SPECIES	-20.66000	-48.42000	1264873128
1851	Diptera	Culicidae	Aedes	Aedes aegypti	SPECIES	-20.66000	-47.02000	1264873184
1852	Diptera	Culicidae	Aedes	Aedes aegypti	SPECIES	-20.66000	141.75000	1264887732
1853	Diptera	Culicidae	Aedes	Aedes aegypti	SPECIES	-20.66000	166.54000	1264889820
1854	Diptera	Culicidae	Aedes	Aedes aegypti	SPECIES	-20.65000	-44.48000	1264873130
1855	Diptera	Culicidae	Aedes	Aedes aegypti	SPECIES	-20.65000	-47.50000	1264873131
1856	Diptera	Culicidae	Aedes	Aedes aegypti	SPECIES	-20.65000	-45.15000	1264873154
1857	Diptera	Culicidae	Aedes	Aedes aegypti	SPECIES	-20.65000	-50.17000	1264873155
1858	Diptera	Culicidae	Aedes	Aedes aegypti	SPECIES	-20.65000	-47.17000	1264873157
1859	Diptera	Culicidae	Aedes	Aedes aegypti	SPECIES	-20.64000	-50.42000	1264873121
1860	Diptera	Culicidae	Aedes	Aedes aegypti	SPECIES	-20.64000	-50.58000	1264873145
1861	Diptera	Culicidae	Aedes	Aedes aegypti	SPECIES	-20.64000	-49.41000	1264873151
1862	Diptera	Culicidae	Aedes	Aedes aegypti	SPECIES	-20.64000	-44.27000	1264873180
1863	Diptera	Culicidae	Aedes	Aedes aegypti	SPECIES	-20.63000	-49.91000	1264873188
1864	Diptera	Culicidae	Aedes	Aedes aegypti	SPECIES	-20.63000	-42.86000	1264873194
1865	Diptera	Culicidae	Aedes	Aedes aegypti	SPECIES	-20.62000	-50.71000	1264873147
1866	Diptera	Culicidae	Aedes	Aedes aegypti	SPECIES	-20.61000	-49.25000	1264873162
1867	Diptera	Culicidae	Aedes	Aedes aegypti	SPECIES	-20.61000	-40.54000	1264873173
1868	Diptera	Culicidae	Aedes	Aedes aegypti	SPECIES	-20.61000	-50.22000	1264873187
1869	Diptera	Culicidae	Aedes	Aedes aegypti	SPECIES	-20.61000	-46.15000	1264873198
1870	Diptera	Culicidae	Aedes	Aedes aegypti	SPECIES	-20.60000	-49.48000	1264873123
1871	Diptera	Culicidae	Aedes	Aedes aegypti	SPECIES	-20.60000	-47.35000	1264873148
1872	Diptera	Culicidae	Aedes	Aedes aegypti	SPECIES	-20.60000	166.57000	1264889814
1873	Diptera	Culicidae	Aedes	Aedes aegypti	SPECIES	-20.59000	-47.64000	1264873161
1874	Diptera	Culicidae	Aedes	Aedes aegypti	SPECIES	-20.59000	-42.72000	1264873166
1875	Diptera	Culicidae	Aedes	Aedes aegypti	SPECIES	-20.59000	-41.95000	1264873186
1876	Diptera	Culicidae	Aedes	Aedes aegypti	SPECIES	-20.59000	-55.37000	1264873191
1877	Diptera	Culicidae	Aedes	Aedes aegypti	SPECIES	-20.58000	-53.51000	1264873138
1878	Diptera	Culicidae	Aedes	Aedes aegypti	SPECIES	-20.58000	-51.46000	1264873150
1879	Diptera	Culicidae	Aedes	Aedes aegypti	SPECIES	-20.58000	-42.13000	1264873163
1880	Diptera	Culicidae	Aedes	Aedes aegypti	SPECIES	-20.57000	-40.83000	1264873156
1881	Diptera	Culicidae	Aedes	Aedes aegypti	SPECIES	-20.56000	-46.93000	1264873116
1882	Diptera	Culicidae	Aedes	Aedes aegypti	SPECIES	-20.56000	-44.66000	1264873139
1883	Diptera	Culicidae	Aedes	Aedes aegypti	SPECIES	-20.56000	-43.02000	1264873144
1884	Diptera	Culicidae	Aedes	Aedes aegypti	SPECIES	-20.56000	-44.89000	1264873172
1885	Diptera	Culicidae	Aedes	Aedes aegypti	SPECIES	-20.56000	-48.57000	1264889565
1886	Diptera	Culicidae	Aedes	Aedes aegypti	SPECIES	-20.55000	-44.04000	1264873141

№	Order	Family	Genus	Species	Taxon Rank	Latitude	Longitude	GBIF_ID
1887	Diptera	Culicidae	Aedes	Aedes aegypti	SPECIES	-20.55000	147.84000	1264887766
1888	Diptera	Culicidae	Aedes	Aedes aegypti	SPECIES	-20.55000	-48.58000	1264890490
1889	Diptera	Culicidae	Aedes	Aedes aegypti	SPECIES	-20.54000	-41.22000	1264873152
1890	Diptera	Culicidae	Aedes	Aedes aegypti	SPECIES	-20.54000	-47.96000	1264873165
1891	Diptera	Culicidae	Aedes	Aedes aegypti	SPECIES	-20.54000	-47.39000	1264888369
1892	Diptera	Culicidae	Aedes	Aedes aegypti	SPECIES	-20.53000	-42.80000	1264873142
1893	Diptera	Culicidae	Aedes	Aedes aegypti	SPECIES	-20.53000	-46.44000	1264873177
1894	Diptera	Culicidae	Aedes	Aedes aegypti	SPECIES	-20.53000	-43.69000	1264873179
1895	Diptera	Culicidae	Aedes	Aedes aegypti	SPECIES	-20.53000	-49.10000	1264873183
1896	Diptera	Culicidae	Aedes	Aedes aegypti	SPECIES	-20.53000	-56.69000	1264873209
1897	Diptera	Culicidae	Aedes	Aedes aegypti	SPECIES	-20.53000	47.24000	1264888584
1898	Diptera	Culicidae	Aedes	Aedes aegypti	SPECIES	-20.52000	-45.54000	1264873167
1899	Diptera	Culicidae	Aedes	Aedes aegypti	SPECIES	-20.52000	-43.84000	1264873168
1900	Diptera	Culicidae	Aedes	Aedes aegypti	SPECIES	-20.52000	-50.37000	1264873171
1901	Diptera	Culicidae	Aedes	Aedes aegypti	SPECIES	-20.52000	-49.68000	1264873176
1902	Diptera	Culicidae	Aedes	Aedes aegypti	SPECIES	-20.52000	-48.62000	1264873197
1903	Diptera	Culicidae	Aedes	Aedes aegypti	SPECIES	-20.52000	-44.42000	1264873205
1904	Diptera	Culicidae	Aedes	Aedes aegypti	SPECIES	-20.52000	145.40000	1264887758
1905	Diptera	Culicidae	Aedes	Aedes aegypti	SPECIES	-20.51000	-45.84000	1264873190
1906	Diptera	Culicidae	Aedes	Aedes aegypti	SPECIES	-20.51000	-48.65000	1264890801
1907	Diptera	Culicidae	Aedes	Aedes aegypti	SPECIES	-20.50000	-54.61167	3008444049
1908	Diptera	Culicidae	Aedes	Aedes aegypti	SPECIES	-20.49000	-47.78000	1264873223
1909	Diptera	Culicidae	Aedes	Aedes aegypti	SPECIES	-20.48000	-50.84000	1264873174
1910	Diptera	Culicidae	Aedes	Aedes aegypti	SPECIES	-20.48000	-50.91000	1264873182
1911	Diptera	Culicidae	Aedes	Aedes aegypti	SPECIES	-20.48000	-42.63000	1264873212
1912	Diptera	Culicidae	Aedes	Aedes aegypti	SPECIES	-20.48000	-44.25000	1264873214
1913	Diptera	Culicidae	Aedes	Aedes aegypti	SPECIES	-20.48000	-51.33000	1264873219
1914	Diptera	Culicidae	Aedes	Aedes aegypti	SPECIES	-20.47000	-51.10000	1264873170
1915	Diptera	Culicidae	Aedes	Aedes aegypti	SPECIES	-20.46000	-46.06000	1264873189
1916	Diptera	Culicidae	Aedes	Aedes aegypti	SPECIES	-20.46000	-45.10000	1264873192
1917	Diptera	Culicidae	Aedes	Aedes aegypti	SPECIES	-20.46000	-47.58000	1264873211
1918	Diptera	Culicidae	Aedes	Aedes aegypti	SPECIES	-20.46000	-55.08000	1264873216
1919	Diptera	Culicidae	Aedes	Aedes aegypti	SPECIES	-20.45000	-49.33000	1264873196
1920	Diptera	Culicidae	Aedes	Aedes aegypti	SPECIES	-20.45000	-50.62000	1264873200
1921	Diptera	Culicidae	Aedes	Aedes aegypti	SPECIES	-20.45000	-45.54000	1264873217
1922	Diptera	Culicidae	Aedes	Aedes aegypti	SPECIES	-20.45000	-50.00000	1264873225
1923	Diptera	Culicidae	Aedes	Aedes aegypti	SPECIES	-20.45000	-50.53000	1264873230
1924	Diptera	Culicidae	Aedes	Aedes aegypti	SPECIES	-20.45000	166.60000	1264889834
1925	Diptera	Culicidae	Aedes	Aedes aegypti	SPECIES	-20.45000	-54.62000	1264890436
1926	Diptera	Culicidae	Aedes	Aedes aegypti	SPECIES	-20.44000	-46.34000	1264873193
1927	Diptera	Culicidae	Aedes	Aedes aegypti	SPECIES	-20.44000	-42.79000	1264873215

№	Order	Family	Genus	Species	Taxon Rank	Latitude	Longitude	GBIF_ID
1928	Diptera	Culicidae	Aedes	Aedes aegypti	SPECIES	-20.44000	-50.75000	1264873218
1929	Diptera	Culicidae	Aedes	Aedes aegypti	SPECIES	-20.44000	-42.26000	1264873221
1930	Diptera	Culicidae	Aedes	Aedes aegypti	SPECIES	-20.44000	-41.95000	1264873245
1931	Diptera	Culicidae	Aedes	Aedes aegypti	SPECIES	-20.44000	-54.62000	1264889199
1932	Diptera	Culicidae	Aedes	Aedes aegypti	SPECIES	-20.44000	-40.38000	1264890835
1933	Diptera	Culicidae	Aedes	Aedes aegypti	SPECIES	-20.43000	-40.77000	1264873199
1934	Diptera	Culicidae	Aedes	Aedes aegypti	SPECIES	-20.43000	-49.77000	1264873208
1935	Diptera	Culicidae	Aedes	Aedes aegypti	SPECIES	-20.43000	-40.38000	1264873224
1936	Diptera	Culicidae	Aedes	Aedes aegypti	SPECIES	-20.42000	-42.91000	1264873232
1937	Diptera	Culicidae	Aedes	Aedes aegypti	SPECIES	-20.41000	-44.36000	1264873195
1938	Diptera	Culicidae	Aedes	Aedes aegypti	SPECIES	-20.41000	-50.38000	1264873213
1939	Diptera	Culicidae	Aedes	Aedes aegypti	SPECIES	-20.41000	-44.04000	1264873236
1940	Diptera	Culicidae	Aedes	Aedes aegypti	SPECIES	-20.41000	-48.06000	1264873239
1941	Diptera	Culicidae	Aedes	Aedes aegypti	SPECIES	-20.41000	-50.12000	1264873241
1942	Diptera	Culicidae	Aedes	Aedes aegypti	SPECIES	-20.41000	166.61000	1264889828
1943	Diptera	Culicidae	Aedes	Aedes aegypti	SPECIES	-20.40000	-45.70000	1264873181
1944	Diptera	Culicidae	Aedes	Aedes aegypti	SPECIES	-20.40000	-52.18000	1264873185
1945	Diptera	Culicidae	Aedes	Aedes aegypti	SPECIES	-20.40000	-43.10000	1264873201
1946	Diptera	Culicidae	Aedes	Aedes aegypti	SPECIES	-20.40000	-50.20000	1264873210
1947	Diptera	Culicidae	Aedes	Aedes aegypti	SPECIES	-20.40000	-43.67000	1264873228
1948	Diptera	Culicidae	Aedes	Aedes aegypti	SPECIES	-20.40000	-44.77000	1264873235
1949	Diptera	Culicidae	Aedes	Aedes aegypti	SPECIES	-20.40000	148.58000	1264887771
1950	Diptera	Culicidae	Aedes	Aedes aegypti	SPECIES	-20.40000	-49.98000	1264890724
1951	Diptera	Culicidae	Aedes	Aedes aegypti	SPECIES	-20.39000	-47.11000	1264873202
1952	Diptera	Culicidae	Aedes	Aedes aegypti	SPECIES	-20.39000	-40.50000	1264873206
1953	Diptera	Culicidae	Aedes	Aedes aegypti	SPECIES	-20.38044	-43.51240	4153920303
1954	Diptera	Culicidae	Aedes	Aedes aegypti	SPECIES	-20.38000	-41.26000	1264873207
1955	Diptera	Culicidae	Aedes	Aedes aegypti	SPECIES	-20.38000	-42.15000	1264873226
1956	Diptera	Culicidae	Aedes	Aedes aegypti	SPECIES	-20.38000	-40.50000	1264888381
1957	Diptera	Culicidae	Aedes	Aedes aegypti	SPECIES	-20.38000	-50.78000	1264890732
1958	Diptera	Culicidae	Aedes	Aedes aegypti	SPECIES	-20.37000	-50.68000	1264873203
1959	Diptera	Culicidae	Aedes	Aedes aegypti	SPECIES	-20.37000	-46.79000	1264873204
1960	Diptera	Culicidae	Aedes	Aedes aegypti	SPECIES	-20.37000	-48.99000	1264873227
1961	Diptera	Culicidae	Aedes	Aedes aegypti	SPECIES	-20.37000	-47.40000	1264873229
1962	Diptera	Culicidae	Aedes	Aedes aegypti	SPECIES	-20.37000	-41.13000	1264873233
1963	Diptera	Culicidae	Aedes	Aedes aegypti	SPECIES	-20.37000	-44.53000	1264873234
1964	Diptera	Culicidae	Aedes	Aedes aegypti	SPECIES	-20.37000	-49.19000	1264873249
1965	Diptera	Culicidae	Aedes	Aedes aegypti	SPECIES	-20.37000	-50.91000	1264873259
1966	Diptera	Culicidae	Aedes	Aedes aegypti	SPECIES	-20.36000	-47.24000	1264873238
1967	Diptera	Culicidae	Aedes	Aedes aegypti	SPECIES	-20.36000	-41.53000	1264873244
1968	Diptera	Culicidae	Aedes	Aedes aegypti	SPECIES	-20.35000	-41.93000	1264873220

№	Order	Family	Genus	Species	Taxon Rank	Latitude	Longitude	GBIF_ID
1969	Diptera	Culicidae	Aedes	Aedes aegypti	SPECIES	-20.34000	-48.40000	1264873240
1970	Diptera	Culicidae	Aedes	Aedes aegypti	SPECIES	-20.34000	-47.57000	1264873247
1971	Diptera	Culicidae	Aedes	Aedes aegypti	SPECIES	-20.34000	-41.67000	1264873248
1972	Diptera	Culicidae	Aedes	Aedes aegypti	SPECIES	-20.34000	-43.33000	1264873265
1973	Diptera	Culicidae	Aedes	Aedes aegypti	SPECIES	-20.34000	-49.50000	1264873266
1974	Diptera	Culicidae	Aedes	Aedes aegypti	SPECIES	-20.33000	-42.72000	1264873274
1975	Diptera	Culicidae	Aedes	Aedes aegypti	SPECIES	-20.33000	-44.01000	1264873297
1976	Diptera	Culicidae	Aedes	Aedes aegypti	SPECIES	-20.33000	-54.84000	1264890919
1977	Diptera	Culicidae	Aedes	Aedes aegypti	SPECIES	-20.32000	-40.84000	1264873250
1978	Diptera	Culicidae	Aedes	Aedes aegypti	SPECIES	-20.32000	-44.22000	1264873263
1979	Diptera	Culicidae	Aedes	Aedes aegypti	SPECIES	-20.32000	-47.83000	1264873269
1980	Diptera	Culicidae	Aedes	Aedes aegypti	SPECIES	-20.32000	-40.35000	1264890865
1981	Diptera	Culicidae	Aedes	Aedes aegypti	SPECIES	-20.31000	-40.47000	1264873243
1982	Diptera	Culicidae	Aedes	Aedes aegypti	SPECIES	-20.31000	-40.35000	1264890239
1983	Diptera	Culicidae	Aedes	Aedes aegypti	SPECIES	-20.30000	-42.31000	1264873270
1984	Diptera	Culicidae	Aedes	Aedes aegypti	SPECIES	-20.30000	-40.29000	1264888347
1985	Diptera	Culicidae	Aedes	Aedes aegypti	SPECIES	-20.30000	44.27000	1264890128
1986	Diptera	Culicidae	Aedes	Aedes aegypti	SPECIES	-20.30000	-40.30000	1264890192
1987	Diptera	Culicidae	Aedes	Aedes aegypti	SPECIES	-20.29047	-40.29128	3456699426
1988	Diptera	Culicidae	Aedes	Aedes aegypti	SPECIES	-20.29000	-49.91000	1264873222
1989	Diptera	Culicidae	Aedes	Aedes aegypti	SPECIES	-20.29000	-43.07000	1264873231
1990	Diptera	Culicidae	Aedes	Aedes aegypti	SPECIES	-20.29000	-49.76000	1264873246
1991	Diptera	Culicidae	Aedes	Aedes aegypti	SPECIES	-20.29000	-45.23000	1264873258
1992	Diptera	Culicidae	Aedes	Aedes aegypti	SPECIES	-20.28000	-40.24000	1264873257
1993	Diptera	Culicidae	Aedes	Aedes aegypti	SPECIES	-20.28000	-50.27000	1264873262
1994	Diptera	Culicidae	Aedes	Aedes aegypti	SPECIES	-20.28000	-42.44000	1264873264
1995	Diptera	Culicidae	Aedes	Aedes aegypti	SPECIES	-20.28000	-45.89000	1264873284
1996	Diptera	Culicidae	Aedes	Aedes aegypti	SPECIES	-20.28000	-50.88000	1264873291
1997	Diptera	Culicidae	Aedes	Aedes aegypti	SPECIES	-20.28000	-40.29000	1264890802
1998	Diptera	Culicidae	Aedes	Aedes aegypti	SPECIES	-20.27000	-48.70000	1264873237
1999	Diptera	Culicidae	Aedes	Aedes aegypti	SPECIES	-20.27000	-50.94000	1264873255
2000	Diptera	Culicidae	Aedes	Aedes aegypti	SPECIES	-20.27000	-51.73000	1264873256
2001	Diptera	Culicidae	Aedes	Aedes aegypti	SPECIES	-20.27000	-41.55000	1264873260
2002	Diptera	Culicidae	Aedes	Aedes aegypti	SPECIES	-20.27000	-50.73000	1264873280
2003	Diptera	Culicidae	Aedes	Aedes aegypti	SPECIES	-20.26670	148.81670	177194571
2004	Diptera	Culicidae	Aedes	Aedes aegypti	SPECIES	-20.26667	148.86670	1633667642
2005	Diptera	Culicidae	Aedes	Aedes aegypti	SPECIES	-20.26000	-50.56000	1264873242
2006	Diptera	Culicidae	Aedes	Aedes aegypti	SPECIES	-20.26000	-51.05000	1264873253
2007	Diptera	Culicidae	Aedes	Aedes aegypti	SPECIES	-20.26000	-50.41000	1264873254
2008	Diptera	Culicidae	Aedes	Aedes aegypti	SPECIES	-20.26000	-44.35000	1264873277
2009	Diptera	Culicidae	Aedes	Aedes aegypti	SPECIES	-20.26000	-43.80000	1264873278

№	Order	Family	Genus	Species	Taxon Rank	Latitude	Longitude	GBIF_ID
2010	Diptera	Culicidae	Aedes	Aedes aegypti	SPECIES	-20.26000	-50.04000	1264873305
2011	Diptera	Culicidae	Aedes	Aedes aegypti	SPECIES	-20.26000	-50.23000	1264890741
2012	Diptera	Culicidae	Aedes	Aedes aegypti	SPECIES	-20.25792	-40.25985	4028776027
2013	Diptera	Culicidae	Aedes	Aedes aegypti	SPECIES	-20.25000	-41.84000	1264873251
2014	Diptera	Culicidae	Aedes	Aedes aegypti	SPECIES	-20.25000	-50.80000	1264873252
2015	Diptera	Culicidae	Aedes	Aedes aegypti	SPECIES	-20.25000	-54.33000	1264873275
2016	Diptera	Culicidae	Aedes	Aedes aegypti	SPECIES	-20.25000	-45.03000	1264873279
2017	Diptera	Culicidae	Aedes	Aedes aegypti	SPECIES	-20.25000	-40.38000	1264889164
2018	Diptera	Culicidae	Aedes	Aedes aegypti	SPECIES	-20.24000	-45.53000	1264873281
2019	Diptera	Culicidae	Aedes	Aedes aegypti	SPECIES	-20.24000	-40.37000	1264888334
2020	Diptera	Culicidae	Aedes	Aedes aegypti	SPECIES	-20.23000	-41.94000	1264873286
2021	Diptera	Culicidae	Aedes	Aedes aegypti	SPECIES	-20.22000	-49.17000	1264873307
2022	Diptera	Culicidae	Aedes	Aedes aegypti	SPECIES	-20.21000	-44.46000	1264873272
2023	Diptera	Culicidae	Aedes	Aedes aegypti	SPECIES	-20.21000	-49.36000	1264873276
2024	Diptera	Culicidae	Aedes	Aedes aegypti	SPECIES	-20.21000	-42.89000	1264873289
2025	Diptera	Culicidae	Aedes	Aedes aegypti	SPECIES	-20.20827	28.57796	3384611596
2026	Diptera	Culicidae	Aedes	Aedes aegypti	SPECIES	-20.20000	-50.13000	1264873261
2027	Diptera	Culicidae	Aedes	Aedes aegypti	SPECIES	-20.20000	-47.71000	1264873282
2028	Diptera	Culicidae	Aedes	Aedes aegypti	SPECIES	-20.20000	-50.49000	1264873285
2029	Diptera	Culicidae	Aedes	Aedes aegypti	SPECIES	-20.20000	-44.71000	1264873288
2030	Diptera	Culicidae	Aedes	Aedes aegypti	SPECIES	-20.20000	-50.66000	1264873315
2031	Diptera	Culicidae	Aedes	Aedes aegypti	SPECIES	-20.19000	-44.16000	1264873273
2032	Diptera	Culicidae	Aedes	Aedes aegypti	SPECIES	-20.19000	-48.17000	1264873293
2033	Diptera	Culicidae	Aedes	Aedes aegypti	SPECIES	-20.18000	-46.50000	1264873267
2034	Diptera	Culicidae	Aedes	Aedes aegypti	SPECIES	-20.18000	-50.73000	1264873268
2035	Diptera	Culicidae	Aedes	Aedes aegypti	SPECIES	-20.18000	-42.09000	1264873290
2036	Diptera	Culicidae	Aedes	Aedes aegypti	SPECIES	-20.18000	-42.26000	1264873295
2037	Diptera	Culicidae	Aedes	Aedes aegypti	SPECIES	-20.18000	-49.74000	1264873308
2038	Diptera	Culicidae	Aedes	Aedes aegypti	SPECIES	-20.17027	-44.91094	3499734404
2039	Diptera	Culicidae	Aedes	Aedes aegypti	SPECIES	-20.16000	-47.82000	1264873283
2040	Diptera	Culicidae	Aedes	Aedes aegypti	SPECIES	-20.16000	-47.43000	1264873322
2041	Diptera	Culicidae	Aedes	Aedes aegypti	SPECIES	-20.15000	-41.81000	1264873271
2042	Diptera	Culicidae	Aedes	Aedes aegypti	SPECIES	-20.14000	-40.30000	1264873304
2043	Diptera	Culicidae	Aedes	Aedes aegypti	SPECIES	-20.14000	-56.60000	1264873309
2044	Diptera	Culicidae	Aedes	Aedes aegypti	SPECIES	-20.13000	-42.66000	1264873298
2045	Diptera	Culicidae	Aedes	Aedes aegypti	SPECIES	-20.13000	-45.76000	1264873314
2046	Diptera	Culicidae	Aedes	Aedes aegypti	SPECIES	-20.13000	-45.53000	1264873320
2047	Diptera	Culicidae	Aedes	Aedes aegypti	SPECIES	-20.13000	-40.30000	1264890840
2048	Diptera	Culicidae	Aedes	Aedes aegypti	SPECIES	-20.12000	-41.58000	1264873299
2049	Diptera	Culicidae	Aedes	Aedes aegypti	SPECIES	-20.12000	-42.96000	1264873301
2050	Diptera	Culicidae	Aedes	Aedes aegypti	SPECIES	-20.12000	-45.99000	1264873312

№	Order	Family	Genus	Species	Taxon Rank	Latitude	Longitude	GBIF_ID
2051	Diptera	Culicidae	Aedes	Aedes aegypti	SPECIES	-20.11000	-43.14000	1264873287
2052	Diptera	Culicidae	Aedes	Aedes aegypti	SPECIES	-20.11000	-49.02000	1264873303
2053	Diptera	Culicidae	Aedes	Aedes aegypti	SPECIES	-20.11000	-40.56000	1264873310
2054	Diptera	Culicidae	Aedes	Aedes aegypti	SPECIES	-20.11000	-50.53000	1264873313
2055	Diptera	Culicidae	Aedes	Aedes aegypti	SPECIES	-20.11000	-44.94000	1264873330
2056	Diptera	Culicidae	Aedes	Aedes aegypti	SPECIES	-20.11000	-41.32000	1264873341
2057	Diptera	Culicidae	Aedes	Aedes aegypti	SPECIES	-20.10000	-50.17000	1264873292
2058	Diptera	Culicidae	Aedes	Aedes aegypti	SPECIES	-20.10000	-43.77000	1264873300
2059	Diptera	Culicidae	Aedes	Aedes aegypti	SPECIES	-20.10000	146.89000	1264887709
2060	Diptera	Culicidae	Aedes	Aedes aegypti	SPECIES	-20.10000	-50.59000	1264887842
2061	Diptera	Culicidae	Aedes	Aedes aegypti	SPECIES	-20.09000	-40.82000	1264873294
2062	Diptera	Culicidae	Aedes	Aedes aegypti	SPECIES	-20.09000	-41.11000	1264873316
2063	Diptera	Culicidae	Aedes	Aedes aegypti	SPECIES	-20.09000	-50.81000	1264873317
2064	Diptera	Culicidae	Aedes	Aedes aegypti	SPECIES	-20.09000	-45.28000	1264873326
2065	Diptera	Culicidae	Aedes	Aedes aegypti	SPECIES	-20.09000	146.27000	3818497062
2066	Diptera	Culicidae	Aedes	Aedes aegypti	SPECIES	-20.08000	-44.58000	1264873306
2067	Diptera	Culicidae	Aedes	Aedes aegypti	SPECIES	-20.08000	-43.89000	1264873331
2068	Diptera	Culicidae	Aedes	Aedes aegypti	SPECIES	-20.08000	146.26000	1264889851
2069	Diptera	Culicidae	Aedes	Aedes aegypti	SPECIES	-20.08000	146.28000	3818067897
2070	Diptera	Culicidae	Aedes	Aedes aegypti	SPECIES	-20.07000	-51.34000	1264873296
2071	Diptera	Culicidae	Aedes	Aedes aegypti	SPECIES	-20.07000	-44.17000	1264873302
2072	Diptera	Culicidae	Aedes	Aedes aegypti	SPECIES	-20.07000	-49.96000	1264873311
2073	Diptera	Culicidae	Aedes	Aedes aegypti	SPECIES	-20.07000	-50.46000	1264873319
2074	Diptera	Culicidae	Aedes	Aedes aegypti	SPECIES	-20.07000	-50.91000	1264873327
2075	Diptera	Culicidae	Aedes	Aedes aegypti	SPECIES	-20.07000	-43.42000	1264873333
2076	Diptera	Culicidae	Aedes	Aedes aegypti	SPECIES	-20.07000	-47.69000	1264873335
2077	Diptera	Culicidae	Aedes	Aedes aegypti	SPECIES	-20.07000	-49.43000	1264873336
2078	Diptera	Culicidae	Aedes	Aedes aegypti	SPECIES	-20.07000	-47.44000	1264873338
2079	Diptera	Culicidae	Aedes	Aedes aegypti	SPECIES	-20.07000	-42.57000	1264873343
2080	Diptera	Culicidae	Aedes	Aedes aegypti	SPECIES	-20.07000	-48.64000	1264873352
2081	Diptera	Culicidae	Aedes	Aedes aegypti	SPECIES	-20.07000	44.66000	1264886092
2082	Diptera	Culicidae	Aedes	Aedes aegypti	SPECIES	-20.07000	146.25000	3818474986
2083	Diptera	Culicidae	Aedes	Aedes aegypti	SPECIES	-20.06000	-50.59000	1264873318
2084	Diptera	Culicidae	Aedes	Aedes aegypti	SPECIES	-20.06000	-44.24000	1264873325
2085	Diptera	Culicidae	Aedes	Aedes aegypti	SPECIES	-20.06000	-44.12000	1264873337
2086	Diptera	Culicidae	Aedes	Aedes aegypti	SPECIES	-20.06000	-44.32000	1264873348
2087	Diptera	Culicidae	Aedes	Aedes aegypti	SPECIES	-20.06000	-50.35000	1264873369
2088	Diptera	Culicidae	Aedes	Aedes aegypti	SPECIES	-20.05000	-49.73000	1264873324
2089	Diptera	Culicidae	Aedes	Aedes aegypti	SPECIES	-20.05000	-43.30000	1264873328
2090	Diptera	Culicidae	Aedes	Aedes aegypti	SPECIES	-20.05000	-41.89000	1264873355
2091	Diptera	Culicidae	Aedes	Aedes aegypti	SPECIES	-20.05000	146.25000	1264887727

№	Order	Family	Genus	Species	Taxon Rank	Latitude	Longitude	GBIF_ID
2092	Diptera	Culicidae	Aedes	Aedes aegypti	SPECIES	-20.04000	-63.52000	1264887261
2093	Diptera	Culicidae	Aedes	Aedes aegypti	SPECIES	-20.03000	-50.75000	1264873329
2094	Diptera	Culicidae	Aedes	Aedes aegypti	SPECIES	-20.03000	-42.26000	1264873339
2095	Diptera	Culicidae	Aedes	Aedes aegypti	SPECIES	-20.03000	-44.44000	1264873346
2096	Diptera	Culicidae	Aedes	Aedes aegypti	SPECIES	-20.02000	-42.36000	1264873323
2097	Diptera	Culicidae	Aedes	Aedes aegypti	SPECIES	-20.02000	-41.65000	1264873332
2098	Diptera	Culicidae	Aedes	Aedes aegypti	SPECIES	-20.02000	-44.07000	1264873340
2099	Diptera	Culicidae	Aedes	Aedes aegypti	SPECIES	-20.02000	-46.37000	1264873357
2100	Diptera	Culicidae	Aedes	Aedes aegypti	SPECIES	-20.01000	-53.00000	1264873367
2101	Diptera	Culicidae	Aedes	Aedes aegypti	SPECIES	-20.01000	148.25000	1264887755
2102	Diptera	Culicidae	Aedes	Aedes aegypti	SPECIES	-20.00000	-45.49000	1264873321
2103	Diptera	Culicidae	Aedes	Aedes aegypti	SPECIES	-20.00000	-48.09000	1264873351
2104	Diptera	Culicidae	Aedes	Aedes aegypti	SPECIES	-20.00000	-42.05000	1264873362
2105	Diptera	Culicidae	Aedes	Aedes aegypti	SPECIES	-19.98000	-43.78000	1264873347
2106	Diptera	Culicidae	Aedes	Aedes aegypti	SPECIES	-19.98000	-44.71000	1264873363
2107	Diptera	Culicidae	Aedes	Aedes aegypti	SPECIES	-19.98000	-54.73000	1264873366
2108	Diptera	Culicidae	Aedes	Aedes aegypti	SPECIES	-19.98000	148.23000	1264887795
2109	Diptera	Culicidae	Aedes	Aedes aegypti	SPECIES	-19.97000	-44.83000	1264873334
2110	Diptera	Culicidae	Aedes	Aedes aegypti	SPECIES	-19.96000	-44.35000	1264873349
2111	Diptera	Culicidae	Aedes	Aedes aegypti	SPECIES	-19.96000	-50.12000	1264873356
2112	Diptera	Culicidae	Aedes	Aedes aegypti	SPECIES	-19.96000	-43.16000	1264873358
2113	Diptera	Culicidae	Aedes	Aedes aegypti	SPECIES	-19.96000	-50.26000	1264873365
2114	Diptera	Culicidae	Aedes	Aedes aegypti	SPECIES	-19.96000	-50.62000	1264873379
2115	Diptera	Culicidae	Aedes	Aedes aegypti	SPECIES	-19.95033	-43.92897	3337382957
2116	Diptera	Culicidae	Aedes	Aedes aegypti	SPECIES	-19.95000	-42.09000	1264873344
2117	Diptera	Culicidae	Aedes	Aedes aegypti	SPECIES	-19.95000	-40.33000	1264873350
2118	Diptera	Culicidae	Aedes	Aedes aegypti	SPECIES	-19.95000	-40.87000	1264873360
2119	Diptera	Culicidae	Aedes	Aedes aegypti	SPECIES	-19.95000	-44.20000	1264890838
2120	Diptera	Culicidae	Aedes	Aedes aegypti	SPECIES	-19.94972	-43.86539	3090635852
2121	Diptera	Culicidae	Aedes	Aedes aegypti	SPECIES	-19.94783	-43.92847	3858774423
2122	Diptera	Culicidae	Aedes	Aedes aegypti	SPECIES	-19.94000	-48.66000	1264873342
2123	Diptera	Culicidae	Aedes	Aedes aegypti	SPECIES	-19.94000	-42.67000	1264873354
2124	Diptera	Culicidae	Aedes	Aedes aegypti	SPECIES	-19.94000	-44.19000	1264873359
2125	Diptera	Culicidae	Aedes	Aedes aegypti	SPECIES	-19.94000	-48.35000	1264873370
2126	Diptera	Culicidae	Aedes	Aedes aegypti	SPECIES	-19.93000	-47.81000	1264873376
2127	Diptera	Culicidae	Aedes	Aedes aegypti	SPECIES	-19.93000	-41.46000	1264873377
2128	Diptera	Culicidae	Aedes	Aedes aegypti	SPECIES	-19.92000	-45.05000	1264873364
2129	Diptera	Culicidae	Aedes	Aedes aegypti	SPECIES	-19.92000	-50.41000	1264873371
2130	Diptera	Culicidae	Aedes	Aedes aegypti	SPECIES	-19.92000	-43.96000	1264873391
2131	Diptera	Culicidae	Aedes	Aedes aegypti	SPECIES	-19.92000	138.12000	1264886406
2132	Diptera	Culicidae	Aedes	Aedes aegypti	SPECIES	-19.92000	-43.94000	1264888340

№	Order	Family	Genus	Species	Taxon Rank	Latitude	Longitude	GBIF_ID
2133	Diptera	Culicidae	Aedes	Aedes aegypti	SPECIES	-19.91000	-50.52000	1264873384
2134	Diptera	Culicidae	Aedes	Aedes aegypti	SPECIES	-19.91000	-46.89000	1264873392
2135	Diptera	Culicidae	Aedes	Aedes aegypti	SPECIES	-19.91000	138.12000	1264888031
2136	Diptera	Culicidae	Aedes	Aedes aegypti	SPECIES	-19.91000	-43.93000	1264891340
2137	Diptera	Culicidae	Aedes	Aedes aegypti	SPECIES	-19.90000	-41.71000	1264873353
2138	Diptera	Culicidae	Aedes	Aedes aegypti	SPECIES	-19.90000	-43.51000	1264873388
2139	Diptera	Culicidae	Aedes	Aedes aegypti	SPECIES	-19.90000	-43.93000	1264888637
2140	Diptera	Culicidae	Aedes	Aedes aegypti	SPECIES	-19.90000	-43.96000	1264891081
2141	Diptera	Culicidae	Aedes	Aedes aegypti	SPECIES	-19.89000	-43.63000	1264873345
2142	Diptera	Culicidae	Aedes	Aedes aegypti	SPECIES	-19.89000	-45.18000	1264873372
2143	Diptera	Culicidae	Aedes	Aedes aegypti	SPECIES	-19.89000	-44.08000	1264890809
2144	Diptera	Culicidae	Aedes	Aedes aegypti	SPECIES	-19.88000	-42.90000	1264873389
2145	Diptera	Culicidae	Aedes	Aedes aegypti	SPECIES	-19.88000	146.63000	1264890205
2146	Diptera	Culicidae	Aedes	Aedes aegypti	SPECIES	-19.87266	-44.00801	3408226698
2147	Diptera	Culicidae	Aedes	Aedes aegypti	SPECIES	-19.87000	-46.16000	1264873361
2148	Diptera	Culicidae	Aedes	Aedes aegypti	SPECIES	-19.87000	-41.06000	1264873381
2149	Diptera	Culicidae	Aedes	Aedes aegypti	SPECIES	-19.87000	-40.64000	1264873390
2150	Diptera	Culicidae	Aedes	Aedes aegypti	SPECIES	-19.86000	-45.70000	1264873374
2151	Diptera	Culicidae	Aedes	Aedes aegypti	SPECIES	-19.86000	-47.22000	1264873375
2152	Diptera	Culicidae	Aedes	Aedes aegypti	SPECIES	-19.86000	-47.63000	1264873380
2153	Diptera	Culicidae	Aedes	Aedes aegypti	SPECIES	-19.86000	-44.44000	1264873386
2154	Diptera	Culicidae	Aedes	Aedes aegypti	SPECIES	-19.86000	-45.00000	1264873404
2155	Diptera	Culicidae	Aedes	Aedes aegypti	SPECIES	-19.85000	-44.61000	1264873368
2156	Diptera	Culicidae	Aedes	Aedes aegypti	SPECIES	-19.85000	-43.79000	1264873400
2157	Diptera	Culicidae	Aedes	Aedes aegypti	SPECIES	-19.85000	-43.98000	1264890772
2158	Diptera	Culicidae	Aedes	Aedes aegypti	SPECIES	-19.85000	-43.78000	1264890834
2159	Diptera	Culicidae	Aedes	Aedes aegypti	SPECIES	-19.84000	-45.40000	1264873382
2160	Diptera	Culicidae	Aedes	Aedes aegypti	SPECIES	-19.84000	-43.16000	1264873413
2161	Diptera	Culicidae	Aedes	Aedes aegypti	SPECIES	-19.83000	-42.67000	1264873378
2162	Diptera	Culicidae	Aedes	Aedes aegypti	SPECIES	-19.83000	-40.43000	1264873385
2163	Diptera	Culicidae	Aedes	Aedes aegypti	SPECIES	-19.82000	-54.33000	1264873419
2164	Diptera	Culicidae	Aedes	Aedes aegypti	SPECIES	-19.82000	-43.95000	1264890872
2165	Diptera	Culicidae	Aedes	Aedes aegypti	SPECIES	-19.81000	-43.10000	1264873387
2166	Diptera	Culicidae	Aedes	Aedes aegypti	SPECIES	-19.81000	-55.06000	1264873410
2167	Diptera	Culicidae	Aedes	Aedes aegypti	SPECIES	-19.80244	-43.91524	3090714896
2168	Diptera	Culicidae	Aedes	Aedes aegypti	SPECIES	-19.80000	-43.31000	1264873403
2169	Diptera	Culicidae	Aedes	Aedes aegypti	SPECIES	-19.79000	-44.88000	1264873401
2170	Diptera	Culicidae	Aedes	Aedes aegypti	SPECIES	-19.78000	-50.80000	1264873417
2171	Diptera	Culicidae	Aedes	Aedes aegypti	SPECIES	-19.78000	-40.13000	1264873420
2172	Diptera	Culicidae	Aedes	Aedes aegypti	SPECIES	-19.78000	-44.07000	1264890845
2173	Diptera	Culicidae	Aedes	Aedes aegypti	SPECIES	-19.77000	-49.87000	1264873397

№	Order	Family	Genus	Species	Taxon Rank	Latitude	Longitude	GBIF_ID
2174	Diptera	Culicidae	Aedes	Aedes aegypti	SPECIES	-19.76000	-42.05000	1264873383
2175	Diptera	Culicidae	Aedes	Aedes aegypti	SPECIES	-19.76000	-46.40000	1264873393
2176	Diptera	Culicidae	Aedes	Aedes aegypti	SPECIES	-19.76000	-45.97000	1264873409
2177	Diptera	Culicidae	Aedes	Aedes aegypti	SPECIES	-19.76000	-44.32000	1264873415
2178	Diptera	Culicidae	Aedes	Aedes aegypti	SPECIES	-19.76000	-49.45000	1264873429
2179	Diptera	Culicidae	Aedes	Aedes aegypti	SPECIES	-19.75113	-47.93856	2963882460
2180	Diptera	Culicidae	Aedes	Aedes aegypti	SPECIES	-19.75000	-41.75000	1264873408
2181	Diptera	Culicidae	Aedes	Aedes aegypti	SPECIES	-19.74000	-42.42000	1264873394
2182	Diptera	Culicidae	Aedes	Aedes aegypti	SPECIES	-19.73000	-40.65000	1264873395
2183	Diptera	Culicidae	Aedes	Aedes aegypti	SPECIES	-19.73000	-43.85000	1264873398
2184	Diptera	Culicidae	Aedes	Aedes aegypti	SPECIES	-19.73000	-43.95000	1264873406
2185	Diptera	Culicidae	Aedes	Aedes aegypti	SPECIES	-19.73000	-40.44000	1264873414
2186	Diptera	Culicidae	Aedes	Aedes aegypti	SPECIES	-19.73000	-42.36000	1264873426
2187	Diptera	Culicidae	Aedes	Aedes aegypti	SPECIES	-19.72000	-40.88000	1264873405
2188	Diptera	Culicidae	Aedes	Aedes aegypti	SPECIES	-19.71000	-48.65000	1264873402
2189	Diptera	Culicidae	Aedes	Aedes aegypti	SPECIES	-19.71000	-50.39000	1264873411
2190	Diptera	Culicidae	Aedes	Aedes aegypti	SPECIES	-19.71000	-45.30000	1264873441
2191	Diptera	Culicidae	Aedes	Aedes aegypti	SPECIES	-19.70000	-44.74000	1264873399
2192	Diptera	Culicidae	Aedes	Aedes aegypti	SPECIES	-19.70000	-44.56000	1264873412
2193	Diptera	Culicidae	Aedes	Aedes aegypti	SPECIES	-19.70000	-43.01000	1264873427
2194	Diptera	Culicidae	Aedes	Aedes aegypti	SPECIES	-19.70000	-43.99000	1264873434
2195	Diptera	Culicidae	Aedes	Aedes aegypti	SPECIES	-19.70000	-45.03000	1264889206
2196	Diptera	Culicidae	Aedes	Aedes aegypti	SPECIES	-19.69000	-42.15000	1264873448
2197	Diptera	Culicidae	Aedes	Aedes aegypti	SPECIES	-19.68000	-42.64000	1264873425
2198	Diptera	Culicidae	Aedes	Aedes aegypti	SPECIES	-19.67000	-49.10000	1264873423
2199	Diptera	Culicidae	Aedes	Aedes aegypti	SPECIES	-19.67000	-42.06000	1264873436
2200	Diptera	Culicidae	Aedes	Aedes aegypti	SPECIES	-19.67000	-45.04000	1264873447
2201	Diptera	Culicidae	Aedes	Aedes aegypti	SPECIES	-19.67000	147.41000	1264887722
2202	Diptera	Culicidae	Aedes	Aedes aegypti	SPECIES	-19.66000	-41.19000	1264873418
2203	Diptera	Culicidae	Aedes	Aedes aegypti	SPECIES	-19.66000	-52.00000	1264873437
2204	Diptera	Culicidae	Aedes	Aedes aegypti	SPECIES	-19.65000	-42.24000	1264873446
2205	Diptera	Culicidae	Aedes	Aedes aegypti	SPECIES	-19.65000	134.19000	1264890689
2206	Diptera	Culicidae	Aedes	Aedes aegypti	SPECIES	-19.64322	-44.04370	3320850909
2207	Diptera	Culicidae	Aedes	Aedes aegypti	SPECIES	-19.64000	-43.97000	1264873454
2208	Diptera	Culicidae	Aedes	Aedes aegypti	SPECIES	-19.63000	-43.88000	1264873407
2209	Diptera	Culicidae	Aedes	Aedes aegypti	SPECIES	-19.63000	-42.72000	1264873416
2210	Diptera	Culicidae	Aedes	Aedes aegypti	SPECIES	-19.63000	-43.68000	1264873421
2211	Diptera	Culicidae	Aedes	Aedes aegypti	SPECIES	-19.63000	-46.99000	1264873432
2212	Diptera	Culicidae	Aedes	Aedes aegypti	SPECIES	-19.63000	-44.06000	1264873438
2213	Diptera	Culicidae	Aedes	Aedes aegypti	SPECIES	-19.62000	-44.64000	1264873424
2214	Diptera	Culicidae	Aedes	Aedes aegypti	SPECIES	-19.62000	-43.58000	1264873433

№	Order	Family	Genus	Species	Taxon Rank	Latitude	Longitude	GBIF_ID
2215	Diptera	Culicidae	Aedes	Aedes aegypti	SPECIES	-19.62000	-48.35000	1264873452
2216	Diptera	Culicidae	Aedes	Aedes aegypti	SPECIES	-19.61000	-46.59000	1264873428
2217	Diptera	Culicidae	Aedes	Aedes aegypti	SPECIES	-19.61000	-46.20000	1264873430
2218	Diptera	Culicidae	Aedes	Aedes aegypti	SPECIES	-19.60000	-43.30000	1264873422
2219	Diptera	Culicidae	Aedes	Aedes aegypti	SPECIES	-19.60000	-47.97000	1264873435
2220	Diptera	Culicidae	Aedes	Aedes aegypti	SPECIES	-19.60000	-42.33000	1264873442
2221	Diptera	Culicidae	Aedes	Aedes aegypti	SPECIES	-19.59000	-44.88000	1264873468
2222	Diptera	Culicidae	Aedes	Aedes aegypti	SPECIES	-19.58000	-42.87000	1264873451
2223	Diptera	Culicidae	Aedes	Aedes aegypti	SPECIES	-19.58000	-44.16000	1264873460
2224	Diptera	Culicidae	Aedes	Aedes aegypti	SPECIES	-19.58000	147.41000	1264887752
2225	Diptera	Culicidae	Aedes	Aedes aegypti	SPECIES	-19.57000	-45.79000	1264873439
2226	Diptera	Culicidae	Aedes	Aedes aegypti	SPECIES	-19.56000	-46.01000	1264873450
2227	Diptera	Culicidae	Aedes	Aedes aegypti	SPECIES	-19.56000	-41.56000	1264873461
2228	Diptera	Culicidae	Aedes	Aedes aegypti	SPECIES	-19.55000	-51.40000	1264873462
2229	Diptera	Culicidae	Aedes	Aedes aegypti	SPECIES	-19.55000	-56.03000	1264873471
2230	Diptera	Culicidae	Aedes	Aedes aegypti	SPECIES	-19.55000	-42.61000	1264873473
2231	Diptera	Culicidae	Aedes	Aedes aegypti	SPECIES	-19.55000	134.23000	1264886398
2232	Diptera	Culicidae	Aedes	Aedes aegypti	SPECIES	-19.53000	-44.53000	1264873431
2233	Diptera	Culicidae	Aedes	Aedes aegypti	SPECIES	-19.53000	-44.06000	1264873444
2234	Diptera	Culicidae	Aedes	Aedes aegypti	SPECIES	-19.51000	-44.47000	1264873445
2235	Diptera	Culicidae	Aedes	Aedes aegypti	SPECIES	-19.51000	-42.03000	1264873449
2236	Diptera	Culicidae	Aedes	Aedes aegypti	SPECIES	-19.51000	-41.00000	1264873459
2237	Diptera	Culicidae	Aedes	Aedes aegypti	SPECIES	-19.51000	-44.67000	1264873480
2238	Diptera	Culicidae	Aedes	Aedes aegypti	SPECIES	-19.50000	-45.54000	1264873440
2239	Diptera	Culicidae	Aedes	Aedes aegypti	SPECIES	-19.50000	-44.42000	1264873457
2240	Diptera	Culicidae	Aedes	Aedes aegypti	SPECIES	-19.48270	47.25057	732501103
2241	Diptera	Culicidae	Aedes	Aedes aegypti	SPECIES	-19.48000	-49.77000	1264873463
2242	Diptera	Culicidae	Aedes	Aedes aegypti	SPECIES	-19.48000	-41.68000	1264873469
2243	Diptera	Culicidae	Aedes	Aedes aegypti	SPECIES	-19.47000	-44.11000	1264873464
2244	Diptera	Culicidae	Aedes	Aedes aegypti	SPECIES	-19.47000	-40.63000	1264873487
2245	Diptera	Culicidae	Aedes	Aedes aegypti	SPECIES	-19.46000	-42.69000	1264891018
2246	Diptera	Culicidae	Aedes	Aedes aegypti	SPECIES	-19.45000	-44.29000	1264873474
2247	Diptera	Culicidae	Aedes	Aedes aegypti	SPECIES	-19.44000	-42.68000	1264873465
2248	Diptera	Culicidae	Aedes	Aedes aegypti	SPECIES	-19.44000	-42.60000	1264873475
2249	Diptera	Culicidae	Aedes	Aedes aegypti	SPECIES	-19.44000	-54.98000	1264873499
2250	Diptera	Culicidae	Aedes	Aedes aegypti	SPECIES	-19.43000	-40.51000	1264873453
2251	Diptera	Culicidae	Aedes	Aedes aegypti	SPECIES	-19.43000	-47.15000	1264873455
2252	Diptera	Culicidae	Aedes	Aedes aegypti	SPECIES	-19.43000	-42.96000	1264873466
2253	Diptera	Culicidae	Aedes	Aedes aegypti	SPECIES	-19.42000	-50.67000	1264873483
2254	Diptera	Culicidae	Aedes	Aedes aegypti	SPECIES	-19.42000	-41.70000	1264873486
2255	Diptera	Culicidae	Aedes	Aedes aegypti	SPECIES	-19.42000	-43.72000	1264873500

№	Order	Family	Genus	Species	Taxon Rank	Latitude	Longitude	GBIF_ID
2256	Diptera	Culicidae	Aedes	Aedes aegypti	SPECIES	-19.41000	-50.34000	1264873476
2257	Diptera	Culicidae	Aedes	Aedes aegypti	SPECIES	-19.40000	-42.36000	1264873458
2258	Diptera	Culicidae	Aedes	Aedes aegypti	SPECIES	-19.39000	-42.54000	1264873443
2259	Diptera	Culicidae	Aedes	Aedes aegypti	SPECIES	-19.39000	-45.20000	1264873477
2260	Diptera	Culicidae	Aedes	Aedes aegypti	SPECIES	-19.39000	-42.08000	1264873492
2261	Diptera	Culicidae	Aedes	Aedes aegypti	SPECIES	-19.38000	-47.50000	1264873478
2262	Diptera	Culicidae	Aedes	Aedes aegypti	SPECIES	-19.38000	-45.78000	1264873494
2263	Diptera	Culicidae	Aedes	Aedes aegypti	SPECIES	-19.37000	-45.98000	1264873481
2264	Diptera	Culicidae	Aedes	Aedes aegypti	SPECIES	-19.37000	-41.10000	1264873490
2265	Diptera	Culicidae	Aedes	Aedes aegypti	SPECIES	-19.37000	-44.70000	1264873493
2266	Diptera	Culicidae	Aedes	Aedes aegypti	SPECIES	-19.36000	-42.31000	1264873507
2267	Diptera	Culicidae	Aedes	Aedes aegypti	SPECIES	-19.36000	-44.07000	1264873525
2268	Diptera	Culicidae	Aedes	Aedes aegypti	SPECIES	-19.35000	-42.17000	1264873489
2269	Diptera	Culicidae	Aedes	Aedes aegypti	SPECIES	-19.35000	-42.25000	1264873512
2270	Diptera	Culicidae	Aedes	Aedes aegypti	SPECIES	-19.35000	146.73000	1264887749
2271	Diptera	Culicidae	Aedes	Aedes aegypti	SPECIES	-19.34000	-40.11000	1264873472
2272	Diptera	Culicidae	Aedes	Aedes aegypti	SPECIES	-19.34000	-44.39000	1264873517
2273	Diptera	Culicidae	Aedes	Aedes aegypti	SPECIES	-19.33000	146.82000	1264887740
2274	Diptera	Culicidae	Aedes	Aedes aegypti	SPECIES	-19.33000	146.72000	1264887767
2275	Diptera	Culicidae	Aedes	Aedes aegypti	SPECIES	-19.32000	-53.88000	1264873484
2276	Diptera	Culicidae	Aedes	Aedes aegypti	SPECIES	-19.32000	146.76000	1264887769
2277	Diptera	Culicidae	Aedes	Aedes aegypti	SPECIES	-19.31000	146.73000	1264887781
2278	Diptera	Culicidae	Aedes	Aedes aegypti	SPECIES	-19.31000	146.75000	1264890676
2279	Diptera	Culicidae	Aedes	Aedes aegypti	SPECIES	-19.30000	146.74000	1264887750
2280	Diptera	Culicidae	Aedes	Aedes aegypti	SPECIES	-19.30000	146.81000	1264887754
2281	Diptera	Culicidae	Aedes	Aedes aegypti	SPECIES	-19.30000	146.76000	1264887761
2282	Diptera	Culicidae	Aedes	Aedes aegypti	SPECIES	-19.30000	146.80000	1264887772
2283	Diptera	Culicidae	Aedes	Aedes aegypti	SPECIES	-19.30000	146.82000	1264887783
2284	Diptera	Culicidae	Aedes	Aedes aegypti	SPECIES	-19.30000	146.79000	1264890174
2285	Diptera	Culicidae	Aedes	Aedes aegypti	SPECIES	-19.29000	-41.90000	1264873488
2286	Diptera	Culicidae	Aedes	Aedes aegypti	SPECIES	-19.29000	146.78000	1264887779
2287	Diptera	Culicidae	Aedes	Aedes aegypti	SPECIES	-19.29000	146.76000	1264887786
2288	Diptera	Culicidae	Aedes	Aedes aegypti	SPECIES	-19.29000	146.77000	1264890207
2289	Diptera	Culicidae	Aedes	Aedes aegypti	SPECIES	-19.29000	146.80000	1264890245
2290	Diptera	Culicidae	Aedes	Aedes aegypti	SPECIES	-19.29000	146.75000	1264890687
2291	Diptera	Culicidae	Aedes	Aedes aegypti	SPECIES	-19.28290	146.80100	2633412528
2292	Diptera	Culicidae	Aedes	Aedes aegypti	SPECIES	-19.28280	146.80100	2633025301
2293	Diptera	Culicidae	Aedes	Aedes aegypti	SPECIES	-19.28278	146.80083	2294528197
2294	Diptera	Culicidae	Aedes	Aedes aegypti	SPECIES	-19.28278	146.80083	2451585773
2295	Diptera	Culicidae	Aedes	Aedes aegypti	SPECIES	-19.28000	-48.91000	1264873467
2296	Diptera	Culicidae	Aedes	Aedes aegypti	SPECIES	-19.28000	-44.34000	1264873491

№	Order	Family	Genus	Species	Taxon Rank	Latitude	Longitude	GBIF_ID
2297	Diptera	Culicidae	Aedes	Aedes aegypti	SPECIES	-19.28000	146.78000	1264887790
2298	Diptera	Culicidae	Aedes	Aedes aegypti	SPECIES	-19.28000	146.79000	1264890518
2299	Diptera	Culicidae	Aedes	Aedes aegypti	SPECIES	-19.28000	146.77000	1264890669
2300	Diptera	Culicidae	Aedes	Aedes aegypti	SPECIES	-19.28000	146.80000	1264890679
2301	Diptera	Culicidae	Aedes	Aedes aegypti	SPECIES	-19.28000	146.82000	1264890682
2302	Diptera	Culicidae	Aedes	Aedes aegypti	SPECIES	-19.27000	146.78000	1264887780
2303	Diptera	Culicidae	Aedes	Aedes aegypti	SPECIES	-19.27000	146.79000	1264887794
2304	Diptera	Culicidae	Aedes	Aedes aegypti	SPECIES	-19.27000	146.82000	1264889059
2305	Diptera	Culicidae	Aedes	Aedes aegypti	SPECIES	-19.27000	146.76000	1264889122
2306	Diptera	Culicidae	Aedes	Aedes aegypti	SPECIES	-19.26667	146.81667	177194636
2307	Diptera	Culicidae	Aedes	Aedes aegypti	SPECIES	-19.26667	146.81670	177194638
2308	Diptera	Culicidae	Aedes	Aedes aegypti	SPECIES	-19.26667	146.81670	1633667769
2309	Diptera	Culicidae	Aedes	Aedes aegypti	SPECIES	-19.26667	146.81667	1633667754
2310	Diptera	Culicidae	Aedes	Aedes aegypti	SPECIES	-19.26000	-45.59000	1264873479
2311	Diptera	Culicidae	Aedes	Aedes aegypti	SPECIES	-19.26000	-42.43000	1264873505
2312	Diptera	Culicidae	Aedes	Aedes aegypti	SPECIES	-19.26000	146.82000	1264886671
2313	Diptera	Culicidae	Aedes	Aedes aegypti	SPECIES	-19.25983	146.81693	1100304607
2314	Diptera	Culicidae	Aedes	Aedes aegypti	SPECIES	-19.25000	-47.71000	1264873456
2315	Diptera	Culicidae	Aedes	Aedes aegypti	SPECIES	-19.25000	-42.61000	1264873470
2316	Diptera	Culicidae	Aedes	Aedes aegypti	SPECIES	-19.25000	-44.22000	1264873482
2317	Diptera	Culicidae	Aedes	Aedes aegypti	SPECIES	-19.25000	-43.88000	1264873498
2318	Diptera	Culicidae	Aedes	Aedes aegypti	SPECIES	-19.25000	-46.31000	1264873510
2319	Diptera	Culicidae	Aedes	Aedes aegypti	SPECIES	-19.25000	146.81000	1264887774
2320	Diptera	Culicidae	Aedes	Aedes aegypti	SPECIES	-19.25000	146.80000	1264891388
2321	Diptera	Culicidae	Aedes	Aedes aegypti	SPECIES	-19.24000	146.80000	1264890173
2322	Diptera	Culicidae	Aedes	Aedes aegypti	SPECIES	-19.23000	-42.71000	1264873501
2323	Diptera	Culicidae	Aedes	Aedes aegypti	SPECIES	-19.22000	-41.15000	1264873496
2324	Diptera	Culicidae	Aedes	Aedes aegypti	SPECIES	-19.22000	-42.15000	1264873524
2325	Diptera	Culicidae	Aedes	Aedes aegypti	SPECIES	-19.22000	-40.31000	1264873530
2326	Diptera	Culicidae	Aedes	Aedes aegypti	SPECIES	-19.21000	-44.00000	1264873503
2327	Diptera	Culicidae	Aedes	Aedes aegypti	SPECIES	-19.20000	-40.49000	1264873508
2328	Diptera	Culicidae	Aedes	Aedes aegypti	SPECIES	-19.19000	-51.28000	1264873495
2329	Diptera	Culicidae	Aedes	Aedes aegypti	SPECIES	-19.19000	-47.52000	1264873518
2330	Diptera	Culicidae	Aedes	Aedes aegypti	SPECIES	-19.18000	-43.64000	1264873502
2331	Diptera	Culicidae	Aedes	Aedes aegypti	SPECIES	-19.18000	-45.91000	1264873506
2332	Diptera	Culicidae	Aedes	Aedes aegypti	SPECIES	-19.18000	-42.32000	1264873509
2333	Diptera	Culicidae	Aedes	Aedes aegypti	SPECIES	-19.17000	-41.42000	1264873513
2334	Diptera	Culicidae	Aedes	Aedes aegypti	SPECIES	-19.16000	-40.62000	1264873516
2335	Diptera	Culicidae	Aedes	Aedes aegypti	SPECIES	-19.16000	146.61000	1264889082
2336	Diptera	Culicidae	Aedes	Aedes aegypti	SPECIES	-19.15000	-54.50000	1264873497
2337	Diptera	Culicidae	Aedes	Aedes aegypti	SPECIES	-19.15000	-40.81000	1264873515

№	Order	Family	Genus	Species	Taxon Rank	Latitude	Longitude	GBIF_ID
2338	Diptera	Culicidae	Aedes	Aedes aegypti	SPECIES	-19.15000	-41.82000	1264873535
2339	Diptera	Culicidae	Aedes	Aedes aegypti	SPECIES	-19.15000	-44.93000	1264873537
2340	Diptera	Culicidae	Aedes	Aedes aegypti	SPECIES	-19.14000	-51.16000	1264873485
2341	Diptera	Culicidae	Aedes	Aedes aegypti	SPECIES	-19.14000	146.84000	1264887765
2342	Diptera	Culicidae	Aedes	Aedes aegypti	SPECIES	-19.13333	146.83333	1633663744
2343	Diptera	Culicidae	Aedes	Aedes aegypti	SPECIES	-19.13333	146.83333	177194639
2344	Diptera	Culicidae	Aedes	Aedes aegypti	SPECIES	-19.13000	-46.68000	1264873520
2345	Diptera	Culicidae	Aedes	Aedes aegypti	SPECIES	-19.12000	-42.03000	1264873504
2346	Diptera	Culicidae	Aedes	Aedes aegypti	SPECIES	-19.11000	-42.10000	1264873542
2347	Diptera	Culicidae	Aedes	Aedes aegypti	SPECIES	-19.10000	-57.57000	1264873523
2348	Diptera	Culicidae	Aedes	Aedes aegypti	SPECIES	-19.10000	-45.37000	1264873550
2349	Diptera	Culicidae	Aedes	Aedes aegypti	SPECIES	-19.09000	-44.21000	1264873532
2350	Diptera	Culicidae	Aedes	Aedes aegypti	SPECIES	-19.08000	-42.24000	1264873526
2351	Diptera	Culicidae	Aedes	Aedes aegypti	SPECIES	-19.08000	-42.43000	1264873534
2352	Diptera	Culicidae	Aedes	Aedes aegypti	SPECIES	-19.08000	-52.75000	1264873545
2353	Diptera	Culicidae	Aedes	Aedes aegypti	SPECIES	-19.07000	-40.14000	1264873538
2354	Diptera	Culicidae	Aedes	Aedes aegypti	SPECIES	-19.06000	-49.86000	1264873521
2355	Diptera	Culicidae	Aedes	Aedes aegypti	SPECIES	-19.06000	-52.07000	1264873541
2356	Diptera	Culicidae	Aedes	Aedes aegypti	SPECIES	-19.06000	-56.65000	1264873555
2357	Diptera	Culicidae	Aedes	Aedes aegypti	SPECIES	-19.06000	-169.87000	1264890057
2358	Diptera	Culicidae	Aedes	Aedes aegypti	SPECIES	-19.05000	-47.46000	1264873519
2359	Diptera	Culicidae	Aedes	Aedes aegypti	SPECIES	-19.05000	-41.82000	1264873527
2360	Diptera	Culicidae	Aedes	Aedes aegypti	SPECIES	-19.04000	-41.73000	1264873531
2361	Diptera	Culicidae	Aedes	Aedes aegypti	SPECIES	-19.04000	-41.22000	1264873533
2362	Diptera	Culicidae	Aedes	Aedes aegypti	SPECIES	-19.03000	-40.98000	1264873549
2363	Diptera	Culicidae	Aedes	Aedes aegypti	SPECIES	-19.03000	-42.70000	1264873557
2364	Diptera	Culicidae	Aedes	Aedes aegypti	SPECIES	-19.03000	-46.13000	1264873560
2365	Diptera	Culicidae	Aedes	Aedes aegypti	SPECIES	-19.03000	-48.33000	1264890982
2366	Diptera	Culicidae	Aedes	Aedes aegypti	SPECIES	-19.02000	-50.59000	1264873564
2367	Diptera	Culicidae	Aedes	Aedes aegypti	SPECIES	-19.01000	-53.00000	1264873558
2368	Diptera	Culicidae	Aedes	Aedes aegypti	SPECIES	-19.00000	-48.36000	1264873574
2369	Diptera	Culicidae	Aedes	Aedes aegypti	SPECIES	-19.00000	-42.01000	1264873580
2370	Diptera	Culicidae	Aedes	Aedes aegypti	SPECIES	-18.99000	-49.53000	1264873514
2371	Diptera	Culicidae	Aedes	Aedes aegypti	SPECIES	-18.99000	-47.07000	1264873539
2372	Diptera	Culicidae	Aedes	Aedes aegypti	SPECIES	-18.99000	-41.13000	1264873561
2373	Diptera	Culicidae	Aedes	Aedes aegypti	SPECIES	-18.98000	-50.35000	1264873511
2374	Diptera	Culicidae	Aedes	Aedes aegypti	SPECIES	-18.97000	-47.86000	1264873522
2375	Diptera	Culicidae	Aedes	Aedes aegypti	SPECIES	-18.97000	-39.97000	1264873536
2376	Diptera	Culicidae	Aedes	Aedes aegypti	SPECIES	-18.97000	-57.80000	1264887262
2377	Diptera	Culicidae	Aedes	Aedes aegypti	SPECIES	-18.96000	-46.67000	1264873546
2378	Diptera	Culicidae	Aedes	Aedes aegypti	SPECIES	-18.96000	-40.33000	1264873567

№	Order	Family	Genus	Species	Taxon Rank	Latitude	Longitude	GBIF_ID
2379	Diptera	Culicidae	Aedes	Aedes aegypti	SPECIES	-18.95000	-40.51000	1264873552
2380	Diptera	Culicidae	Aedes	Aedes aegypti	SPECIES	-18.94000	-48.30000	1264890602
2381	Diptera	Culicidae	Aedes	Aedes aegypti	SPECIES	-18.93000	-41.36000	1264873544
2382	Diptera	Culicidae	Aedes	Aedes aegypti	SPECIES	-18.93000	-43.48000	1264873559
2383	Diptera	Culicidae	Aedes	Aedes aegypti	SPECIES	-18.93000	48.41000	1264888607
2384	Diptera	Culicidae	Aedes	Aedes aegypti	SPECIES	-18.91000	-47.55000	1264873551
2385	Diptera	Culicidae	Aedes	Aedes aegypti	SPECIES	-18.91000	47.53000	1264888621
2386	Diptera	Culicidae	Aedes	Aedes aegypti	SPECIES	-18.91000	-48.28000	1264890396
2387	Diptera	Culicidae	Aedes	Aedes aegypti	SPECIES	-18.91000	-48.27000	1264890595
2388	Diptera	Culicidae	Aedes	Aedes aegypti	SPECIES	-18.90000	-45.26000	1264873528
2389	Diptera	Culicidae	Aedes	Aedes aegypti	SPECIES	-18.90000	-42.31000	1264873553
2390	Diptera	Culicidae	Aedes	Aedes aegypti	SPECIES	-18.90000	47.97000	1264888564
2391	Diptera	Culicidae	Aedes	Aedes aegypti	SPECIES	-18.89000	-43.92000	1264873529
2392	Diptera	Culicidae	Aedes	Aedes aegypti	SPECIES	-18.89000	-51.33000	1264873543
2393	Diptera	Culicidae	Aedes	Aedes aegypti	SPECIES	-18.89000	-41.53000	1264873568
2394	Diptera	Culicidae	Aedes	Aedes aegypti	SPECIES	-18.88000	-46.19000	1264873554
2395	Diptera	Culicidae	Aedes	Aedes aegypti	SPECIES	-18.88000	-48.26000	1264890596
2396	Diptera	Culicidae	Aedes	Aedes aegypti	SPECIES	-18.87000	-41.07000	1264873540
2397	Diptera	Culicidae	Aedes	Aedes aegypti	SPECIES	-18.87000	-42.48000	1264873565
2398	Diptera	Culicidae	Aedes	Aedes aegypti	SPECIES	-18.86000	-42.81000	1264873566
2399	Diptera	Culicidae	Aedes	Aedes aegypti	SPECIES	-18.86000	-42.40000	1264873569
2400	Diptera	Culicidae	Aedes	Aedes aegypti	SPECIES	-18.85710	-41.94430	4067339658
2401	Diptera	Culicidae	Aedes	Aedes aegypti	SPECIES	-18.84000	-45.84000	1264873547
2402	Diptera	Culicidae	Aedes	Aedes aegypti	SPECIES	-18.81000	-50.60000	1264873548
2403	Diptera	Culicidae	Aedes	Aedes aegypti	SPECIES	-18.81000	-48.89000	1264873556
2404	Diptera	Culicidae	Aedes	Aedes aegypti	SPECIES	-18.81000	-52.08000	1264873589
2405	Diptera	Culicidae	Aedes	Aedes aegypti	SPECIES	-18.80000	-46.74000	1264873572
2406	Diptera	Culicidae	Aedes	Aedes aegypti	SPECIES	-18.80000	-42.66000	1264873575
2407	Diptera	Culicidae	Aedes	Aedes aegypti	SPECIES	-18.80000	-40.06000	1264873581
2408	Diptera	Culicidae	Aedes	Aedes aegypti	SPECIES	-18.80000	-42.61000	1264873591
2409	Diptera	Culicidae	Aedes	Aedes aegypti	SPECIES	-18.80000	47.48000	1264888589
2410	Diptera	Culicidae	Aedes	Aedes aegypti	SPECIES	-18.79000	-54.93000	1264873578
2411	Diptera	Culicidae	Aedes	Aedes aegypti	SPECIES	-18.79000	-41.94000	1264873602
2412	Diptera	Culicidae	Aedes	Aedes aegypti	SPECIES	-18.78000	-42.40000	1264873570
2413	Diptera	Culicidae	Aedes	Aedes aegypti	SPECIES	-18.78000	-41.30000	1264873577
2414	Diptera	Culicidae	Aedes	Aedes aegypti	SPECIES	-18.78000	48.60000	1264888591
2415	Diptera	Culicidae	Aedes	Aedes aegypti	SPECIES	-18.78000	-41.96000	1264890825
2416	Diptera	Culicidae	Aedes	Aedes aegypti	SPECIES	-18.77000	-45.53000	1264873563
2417	Diptera	Culicidae	Aedes	Aedes aegypti	SPECIES	-18.77000	-53.87000	1264873584
2418	Diptera	Culicidae	Aedes	Aedes aegypti	SPECIES	-18.77000	-44.27000	1264873598
2419	Diptera	Culicidae	Aedes	Aedes aegypti	SPECIES	-18.76000	-46.30000	1264873562

№	Order	Family	Genus	Species	Taxon Rank	Latitude	Longitude	GBIF_ID
2420	Diptera	Culicidae	Aedes	Aedes aegypti	SPECIES	-18.76000	-49.24000	1264873590
2421	Diptera	Culicidae	Aedes	Aedes aegypti	SPECIES	-18.75000	-44.44000	1264873573
2422	Diptera	Culicidae	Aedes	Aedes aegypti	SPECIES	-18.74000	-47.73000	1264873587
2423	Diptera	Culicidae	Aedes	Aedes aegypti	SPECIES	-18.74000	-41.15000	1264873597
2424	Diptera	Culicidae	Aedes	Aedes aegypti	SPECIES	-18.73829	-39.75098	4137603929
2425	Diptera	Culicidae	Aedes	Aedes aegypti	SPECIES	-18.73000	-51.08000	1264873588
2426	Diptera	Culicidae	Aedes	Aedes aegypti	SPECIES	-18.73000	48.95000	1264888606
2427	Diptera	Culicidae	Aedes	Aedes aegypti	SPECIES	-18.72000	-47.50000	1264873615
2428	Diptera	Culicidae	Aedes	Aedes aegypti	SPECIES	-18.71000	-49.94000	1264873593
2429	Diptera	Culicidae	Aedes	Aedes aegypti	SPECIES	-18.71000	-44.08000	1264873600
2430	Diptera	Culicidae	Aedes	Aedes aegypti	SPECIES	-18.70000	-41.49000	1264873592
2431	Diptera	Culicidae	Aedes	Aedes aegypti	SPECIES	-18.69000	-44.95000	1264873605
2432	Diptera	Culicidae	Aedes	Aedes aegypti	SPECIES	-18.68000	-49.57000	1264873579
2433	Diptera	Culicidae	Aedes	Aedes aegypti	SPECIES	-18.68000	-41.09000	1264873599
2434	Diptera	Culicidae	Aedes	Aedes aegypti	SPECIES	-18.67000	35.53000	3987197860
2435	Diptera	Culicidae	Aedes	Aedes aegypti	SPECIES	-18.66667	146.55000	177194624
2436	Diptera	Culicidae	Aedes	Aedes aegypti	SPECIES	-18.66667	146.55000	1633667800
2437	Diptera	Culicidae	Aedes	Aedes aegypti	SPECIES	-18.66000	-40.84000	1264873576
2438	Diptera	Culicidae	Aedes	Aedes aegypti	SPECIES	-18.66000	-40.40000	1264873582
2439	Diptera	Culicidae	Aedes	Aedes aegypti	SPECIES	-18.65000	-49.17000	1264873583
2440	Diptera	Culicidae	Aedes	Aedes aegypti	SPECIES	-18.65000	-43.05000	1264873585
2441	Diptera	Culicidae	Aedes	Aedes aegypti	SPECIES	-18.65000	146.15000	1264887775
2442	Diptera	Culicidae	Aedes	Aedes aegypti	SPECIES	-18.64000	-46.11000	1264873586
2443	Diptera	Culicidae	Aedes	Aedes aegypti	SPECIES	-18.63000	-42.25000	1264873616
2444	Diptera	Culicidae	Aedes	Aedes aegypti	SPECIES	-18.61000	-53.17000	1264873571
2445	Diptera	Culicidae	Aedes	Aedes aegypti	SPECIES	-18.61000	-48.27000	1264873595
2446	Diptera	Culicidae	Aedes	Aedes aegypti	SPECIES	-18.61000	-41.37000	1264873606
2447	Diptera	Culicidae	Aedes	Aedes aegypti	SPECIES	-18.61000	-40.64000	1264873624
2448	Diptera	Culicidae	Aedes	Aedes aegypti	SPECIES	-18.60000	-51.00000	1264873604
2449	Diptera	Culicidae	Aedes	Aedes aegypti	SPECIES	-18.60000	-41.92000	1264873618
2450	Diptera	Culicidae	Aedes	Aedes aegypti	SPECIES	-18.60000	-49.46000	1264873623
2451	Diptera	Culicidae	Aedes	Aedes aegypti	SPECIES	-18.60000	-44.63000	1264873626
2452	Diptera	Culicidae	Aedes	Aedes aegypti	SPECIES	-18.58333	146.28334	1633663696
2453	Diptera	Culicidae	Aedes	Aedes aegypti	SPECIES	-18.58333	146.28334	177194492
2454	Diptera	Culicidae	Aedes	Aedes aegypti	SPECIES	-18.58000	143.61000	1264891356
2455	Diptera	Culicidae	Aedes	Aedes aegypti	SPECIES	-18.57000	-47.87000	1264873609
2456	Diptera	Culicidae	Aedes	Aedes aegypti	SPECIES	-18.57000	-48.78000	1264873617
2457	Diptera	Culicidae	Aedes	Aedes aegypti	SPECIES	-18.57000	-41.45000	1264873622
2458	Diptera	Culicidae	Aedes	Aedes aegypti	SPECIES	-18.56000	-45.41000	1264873610
2459	Diptera	Culicidae	Aedes	Aedes aegypti	SPECIES	-18.55000	-41.24000	1264873621
2460	Diptera	Culicidae	Aedes	Aedes aegypti	SPECIES	-18.54000	-42.54000	1264873639

№	Order	Family	Genus	Species	Taxon Rank	Latitude	Longitude	GBIF_ID
2461	Diptera	Culicidae	Aedes	Aedes aegypti	SPECIES	-18.53333	146.33333	1633663711
2462	Diptera	Culicidae	Aedes	Aedes aegypti	SPECIES	-18.53333	146.33333	177194622
2463	Diptera	Culicidae	Aedes	Aedes aegypti	SPECIES	-18.52000	-40.97000	1264873596
2464	Diptera	Culicidae	Aedes	Aedes aegypti	SPECIES	-18.52000	-41.87000	1264873612
2465	Diptera	Culicidae	Aedes	Aedes aegypti	SPECIES	-18.52000	-43.40000	1264873627
2466	Diptera	Culicidae	Aedes	Aedes aegypti	SPECIES	-18.51000	-42.65000	1264873603
2467	Diptera	Culicidae	Aedes	Aedes aegypti	SPECIES	-18.51000	-43.87000	1264873607
2468	Diptera	Culicidae	Aedes	Aedes aegypti	SPECIES	-18.50000	-42.80000	1264873594
2469	Diptera	Culicidae	Aedes	Aedes aegypti	SPECIES	-18.50000	-42.06000	1264873601
2470	Diptera	Culicidae	Aedes	Aedes aegypti	SPECIES	-18.50000	-50.17000	1264873608
2471	Diptera	Culicidae	Aedes	Aedes aegypti	SPECIES	-18.50000	-49.90000	1264873614
2472	Diptera	Culicidae	Aedes	Aedes aegypti	SPECIES	-18.49000	-42.18000	1264873620
2473	Diptera	Culicidae	Aedes	Aedes aegypti	SPECIES	-18.49000	-49.60000	1264873631
2474	Diptera	Culicidae	Aedes	Aedes aegypti	SPECIES	-18.49000	-47.76000	1264873640
2475	Diptera	Culicidae	Aedes	Aedes aegypti	SPECIES	-18.49000	-41.10000	1264873647
2476	Diptera	Culicidae	Aedes	Aedes aegypti	SPECIES	-18.48000	-41.76000	1264873611
2477	Diptera	Culicidae	Aedes	Aedes aegypti	SPECIES	-18.48000	-49.13000	1264873613
2478	Diptera	Culicidae	Aedes	Aedes aegypti	SPECIES	-18.47000	-43.02000	1264873635
2479	Diptera	Culicidae	Aedes	Aedes aegypti	SPECIES	-18.47000	-40.31000	1264873641
2480	Diptera	Culicidae	Aedes	Aedes aegypti	SPECIES	-18.46000	-45.93000	1264873634
2481	Diptera	Culicidae	Aedes	Aedes aegypti	SPECIES	-18.46000	-42.31000	1264873646
2482	Diptera	Culicidae	Aedes	Aedes aegypti	SPECIES	-18.46000	-44.67000	1264891058
2483	Diptera	Culicidae	Aedes	Aedes aegypti	SPECIES	-18.45000	-50.53000	1264873628
2484	Diptera	Culicidae	Aedes	Aedes aegypti	SPECIES	-18.45000	-39.85000	1264873654
2485	Diptera	Culicidae	Aedes	Aedes aegypti	SPECIES	-18.44000	-47.61000	1264873632
2486	Diptera	Culicidae	Aedes	Aedes aegypti	SPECIES	-18.44000	-41.52000	1264873643
2487	Diptera	Culicidae	Aedes	Aedes aegypti	SPECIES	-18.40000	-41.37000	1264873619
2488	Diptera	Culicidae	Aedes	Aedes aegypti	SPECIES	-18.39000	-44.01000	1264873637
2489	Diptera	Culicidae	Aedes	Aedes aegypti	SPECIES	-18.39000	-44.16000	1264873665
2490	Diptera	Culicidae	Aedes	Aedes aegypti	SPECIES	-18.38000	-49.47000	1264873625
2491	Diptera	Culicidae	Aedes	Aedes aegypti	SPECIES	-18.38000	-52.58000	1264873636
2492	Diptera	Culicidae	Aedes	Aedes aegypti	SPECIES	-18.37000	-47.49000	1264873629
2493	Diptera	Culicidae	Aedes	Aedes aegypti	SPECIES	-18.37000	-47.76000	1264873630
2494	Diptera	Culicidae	Aedes	Aedes aegypti	SPECIES	-18.37000	-40.21000	1264873633
2495	Diptera	Culicidae	Aedes	Aedes aegypti	SPECIES	-18.37000	-42.58000	1264873650
2496	Diptera	Culicidae	Aedes	Aedes aegypti	SPECIES	-18.34000	-47.12000	1264873642
2497	Diptera	Culicidae	Aedes	Aedes aegypti	SPECIES	-18.34000	-41.55000	1264873648
2498	Diptera	Culicidae	Aedes	Aedes aegypti	SPECIES	-18.34000	-59.60000	1264887271
2499	Diptera	Culicidae	Aedes	Aedes aegypti	SPECIES	-18.33000	-42.13000	1264873653
2500	Diptera	Culicidae	Aedes	Aedes aegypti	SPECIES	-18.33000	-59.76000	1264887257
2501	Diptera	Culicidae	Aedes	Aedes aegypti	SPECIES	-18.32000	-48.15000	1264873638

№	Order	Family	Genus	Species	Taxon Rank	Latitude	Longitude	GBIF_ID
2502	Diptera	Culicidae	Aedes	Aedes aegypti	SPECIES	-18.31000	-44.59000	1264873655
2503	Diptera	Culicidae	Aedes	Aedes aegypti	SPECIES	-18.31000	-48.22000	1264873670
2504	Diptera	Culicidae	Aedes	Aedes aegypti	SPECIES	-18.30000	143.55000	177194578
2505	Diptera	Culicidae	Aedes	Aedes aegypti	SPECIES	-18.30000	-45.05000	1264873645
2506	Diptera	Culicidae	Aedes	Aedes aegypti	SPECIES	-18.30000	143.55000	1633663802
2507	Diptera	Culicidae	Aedes	Aedes aegypti	SPECIES	-18.29000	143.55000	1264887759
2508	Diptera	Culicidae	Aedes	Aedes aegypti	SPECIES	-18.28000	-41.73000	1264873664
2509	Diptera	Culicidae	Aedes	Aedes aegypti	SPECIES	-18.27000	146.03000	1264887792
2510	Diptera	Culicidae	Aedes	Aedes aegypti	SPECIES	-18.27000	146.02000	1264889050
2511	Diptera	Culicidae	Aedes	Aedes aegypti	SPECIES	-18.26000	-40.52000	1264873651
2512	Diptera	Culicidae	Aedes	Aedes aegypti	SPECIES	-18.26000	-42.49000	1264873680
2513	Diptera	Culicidae	Aedes	Aedes aegypti	SPECIES	-18.25255	32.01092	3032169931
2514	Diptera	Culicidae	Aedes	Aedes aegypti	SPECIES	-18.25000	-43.07000	1264873652
2515	Diptera	Culicidae	Aedes	Aedes aegypti	SPECIES	-18.24000	-42.83000	1264873672
2516	Diptera	Culicidae	Aedes	Aedes aegypti	SPECIES	-18.24000	-178.81000	1264890085
2517	Diptera	Culicidae	Aedes	Aedes aegypti	SPECIES	-18.23000	-40.84000	1264873659
2518	Diptera	Culicidae	Aedes	Aedes aegypti	SPECIES	-18.23000	-53.71000	1264873666
2519	Diptera	Culicidae	Aedes	Aedes aegypti	SPECIES	-18.22000	-41.11000	1264873644
2520	Diptera	Culicidae	Aedes	Aedes aegypti	SPECIES	-18.22000	-52.21000	1264873649
2521	Diptera	Culicidae	Aedes	Aedes aegypti	SPECIES	-18.22000	-47.74000	1264873656
2522	Diptera	Culicidae	Aedes	Aedes aegypti	SPECIES	-18.22000	-51.28000	1264873658
2523	Diptera	Culicidae	Aedes	Aedes aegypti	SPECIES	-18.22000	-178.80000	1264890052
2524	Diptera	Culicidae	Aedes	Aedes aegypti	SPECIES	-18.21000	-49.39000	1264873668
2525	Diptera	Culicidae	Aedes	Aedes aegypti	SPECIES	-18.21000	-42.30000	1264873676
2526	Diptera	Culicidae	Aedes	Aedes aegypti	SPECIES	-18.20000	-49.87000	1264873674
2527	Diptera	Culicidae	Aedes	Aedes aegypti	SPECIES	-18.19000	-54.69000	1264873679
2528	Diptera	Culicidae	Aedes	Aedes aegypti	SPECIES	-18.18000	-41.83000	1264873657
2529	Diptera	Culicidae	Aedes	Aedes aegypti	SPECIES	-18.18000	-45.59000	1264873660
2530	Diptera	Culicidae	Aedes	Aedes aegypti	SPECIES	-18.16000	-46.38000	1264873661
2531	Diptera	Culicidae	Aedes	Aedes aegypti	SPECIES	-18.15000	-50.30000	1264873663
2532	Diptera	Culicidae	Aedes	Aedes aegypti	SPECIES	-18.15000	-40.06000	1264873690
2533	Diptera	Culicidae	Aedes	Aedes aegypti	SPECIES	-18.15000	178.43000	1264889079
2534	Diptera	Culicidae	Aedes	Aedes aegypti	SPECIES	-18.14000	-49.02000	1264873662
2535	Diptera	Culicidae	Aedes	Aedes aegypti	SPECIES	-18.14000	-41.51000	1264873667
2536	Diptera	Culicidae	Aedes	Aedes aegypti	SPECIES	-18.14000	178.44000	1264889783
2537	Diptera	Culicidae	Aedes	Aedes aegypti	SPECIES	-18.14000	178.43000	1264890114
2538	Diptera	Culicidae	Aedes	Aedes aegypti	SPECIES	-18.13333	178.41670	177195174
2539	Diptera	Culicidae	Aedes	Aedes aegypti	SPECIES	-18.13333	178.41670	1633667680
2540	Diptera	Culicidae	Aedes	Aedes aegypti	SPECIES	-18.13000	49.39000	1264888615
2541	Diptera	Culicidae	Aedes	Aedes aegypti	SPECIES	-18.13000	178.43000	1264890253
2542	Diptera	Culicidae	Aedes	Aedes aegypti	SPECIES	-18.12000	-48.14000	1264873677

№	Order	Family	Genus	Species	Taxon Rank	Latitude	Longitude	GBIF_ID
2543	Diptera	Culicidae	Aedes	Aedes aegypti	SPECIES	-18.12000	-40.27000	1264873691
2544	Diptera	Culicidae	Aedes	Aedes aegypti	SPECIES	-18.11000	-47.56000	1264873671
2545	Diptera	Culicidae	Aedes	Aedes aegypti	SPECIES	-18.11000	-48.61000	1264873678
2546	Diptera	Culicidae	Aedes	Aedes aegypti	SPECIES	-18.11000	178.41000	1264891133
2547	Diptera	Culicidae	Aedes	Aedes aegypti	SPECIES	-18.10000	-44.22000	1264873708
2548	Diptera	Culicidae	Aedes	Aedes aegypti	SPECIES	-18.08000	-43.41000	1264873695
2549	Diptera	Culicidae	Aedes	Aedes aegypti	SPECIES	-18.08000	-48.30000	1264873696
2550	Diptera	Culicidae	Aedes	Aedes aegypti	SPECIES	-18.07000	-48.79000	1264873689
2551	Diptera	Culicidae	Aedes	Aedes aegypti	SPECIES	-18.04000	-39.88000	1264873675
2552	Diptera	Culicidae	Aedes	Aedes aegypti	SPECIES	-18.04000	-50.35000	1264873682
2553	Diptera	Culicidae	Aedes	Aedes aegypti	SPECIES	-18.03000	-41.30000	1264873669
2554	Diptera	Culicidae	Aedes	Aedes aegypti	SPECIES	-18.03000	-46.75000	1264873681
2555	Diptera	Culicidae	Aedes	Aedes aegypti	SPECIES	-18.02000	-40.52000	1264873673
2556	Diptera	Culicidae	Aedes	Aedes aegypti	SPECIES	-18.01000	-42.27000	1264873685
2557	Diptera	Culicidae	Aedes	Aedes aegypti	SPECIES	-18.00000	178.00000	177195167
2558	Diptera	Culicidae	Aedes	Aedes aegypti	SPECIES	-18.00000	-41.97000	1264873693
2559	Diptera	Culicidae	Aedes	Aedes aegypti	SPECIES	-17.99000	-49.67000	1264873687
2560	Diptera	Culicidae	Aedes	Aedes aegypti	SPECIES	-17.98000	-47.72000	1264873692
2561	Diptera	Culicidae	Aedes	Aedes aegypti	SPECIES	-17.97000	-48.69000	1264873714
2562	Diptera	Culicidae	Aedes	Aedes aegypti	SPECIES	-17.95000	-54.26000	1264873705
2563	Diptera	Culicidae	Aedes	Aedes aegypti	SPECIES	-17.93000	145.92000	1264887803
2564	Diptera	Culicidae	Aedes	Aedes aegypti	SPECIES	-17.92000	-50.13000	1264873688
2565	Diptera	Culicidae	Aedes	Aedes aegypti	SPECIES	-17.91000	-43.65000	1264873700
2566	Diptera	Culicidae	Aedes	Aedes aegypti	SPECIES	-17.90000	-51.73000	1264873704
2567	Diptera	Culicidae	Aedes	Aedes aegypti	SPECIES	-17.89000	-39.67000	1264873684
2568	Diptera	Culicidae	Aedes	Aedes aegypti	SPECIES	-17.89000	-63.33000	1264887274
2569	Diptera	Culicidae	Aedes	Aedes aegypti	SPECIES	-17.88000	-44.70000	1264873683
2570	Diptera	Culicidae	Aedes	Aedes aegypti	SPECIES	-17.87000	146.11000	1264887796
2571	Diptera	Culicidae	Aedes	Aedes aegypti	SPECIES	-17.86000	-46.88000	1264873686
2572	Diptera	Culicidae	Aedes	Aedes aegypti	SPECIES	-17.86000	-42.10000	1264873716
2573	Diptera	Culicidae	Aedes	Aedes aegypti	SPECIES	-17.85000	-42.87000	1264873697
2574	Diptera	Culicidae	Aedes	Aedes aegypti	SPECIES	-17.85000	-43.95000	1264873701
2575	Diptera	Culicidae	Aedes	Aedes aegypti	SPECIES	-17.85000	-149.14000	1264890212
2576	Diptera	Culicidae	Aedes	Aedes aegypti	SPECIES	-17.82000	-48.78000	1264873702
2577	Diptera	Culicidae	Aedes	Aedes aegypti	SPECIES	-17.81101	31.10351	3032035480
2578	Diptera	Culicidae	Aedes	Aedes aegypti	SPECIES	-17.81000	-41.80000	1264873713
2579	Diptera	Culicidae	Aedes	Aedes aegypti	SPECIES	-17.80000	-50.31000	1264873721
2580	Diptera	Culicidae	Aedes	Aedes aegypti	SPECIES	-17.80000	-49.14000	1264873733
2581	Diptera	Culicidae	Aedes	Aedes aegypti	SPECIES	-17.80000	-149.30000	1264888516
2582	Diptera	Culicidae	Aedes	Aedes aegypti	SPECIES	-17.80000	-149.29000	1264889635
2583	Diptera	Culicidae	Aedes	Aedes aegypti	SPECIES	-17.80000	177.42000	1264889769

№	Order	Family	Genus	Species	Taxon Rank	Latitude	Longitude	GBIF_ID
2584	Diptera	Culicidae	Aedes	Aedes aegypti	SPECIES	-17.80000	-63.16000	1264891402
2585	Diptera	Culicidae	Aedes	Aedes aegypti	SPECIES	-17.80000	35.40000	3987193860
2586	Diptera	Culicidae	Aedes	Aedes aegypti	SPECIES	-17.79208	-63.14096	4021964344
2587	Diptera	Culicidae	Aedes	Aedes aegypti	SPECIES	-17.79019	31.12133	3031777985
2588	Diptera	Culicidae	Aedes	Aedes aegypti	SPECIES	-17.79000	-49.60000	1264873703
2589	Diptera	Culicidae	Aedes	Aedes aegypti	SPECIES	-17.79000	-50.57000	1264873706
2590	Diptera	Culicidae	Aedes	Aedes aegypti	SPECIES	-17.79000	-63.20000	1264890619
2591	Diptera	Culicidae	Aedes	Aedes aegypti	SPECIES	-17.78000	-63.17000	1264886676
2592	Diptera	Culicidae	Aedes	Aedes aegypti	SPECIES	-17.78000	-63.18000	1264887265
2593	Diptera	Culicidae	Aedes	Aedes aegypti	SPECIES	-17.77000	-48.69000	1264873694
2594	Diptera	Culicidae	Aedes	Aedes aegypti	SPECIES	-17.77000	-53.31000	1264873720
2595	Diptera	Culicidae	Aedes	Aedes aegypti	SPECIES	-17.76000	-149.51000	1264888500
2596	Diptera	Culicidae	Aedes	Aedes aegypti	SPECIES	-17.75000	-47.12000	1264873698
2597	Diptera	Culicidae	Aedes	Aedes aegypti	SPECIES	-17.75000	-40.26000	1264873699
2598	Diptera	Culicidae	Aedes	Aedes aegypti	SPECIES	-17.75000	-46.50000	1264873711
2599	Diptera	Culicidae	Aedes	Aedes aegypti	SPECIES	-17.75000	-149.28000	1264889614
2600	Diptera	Culicidae	Aedes	Aedes aegypti	SPECIES	-17.75000	-149.35000	1264889648
2601	Diptera	Culicidae	Aedes	Aedes aegypti	SPECIES	-17.75000	-149.16000	1264890228
2602	Diptera	Culicidae	Aedes	Aedes aegypti	SPECIES	-17.75000	-39.25000	1264890769
2603	Diptera	Culicidae	Aedes	Aedes aegypti	SPECIES	-17.74510	-46.17655	1802768875
2604	Diptera	Culicidae	Aedes	Aedes aegypti	SPECIES	-17.74000	-49.89000	1264873712
2605	Diptera	Culicidae	Aedes	Aedes aegypti	SPECIES	-17.74000	-39.98000	1264873717
2606	Diptera	Culicidae	Aedes	Aedes aegypti	SPECIES	-17.74000	-40.51000	1264873718
2607	Diptera	Culicidae	Aedes	Aedes aegypti	SPECIES	-17.74000	168.32000	1264888567
2608	Diptera	Culicidae	Aedes	Aedes aegypti	SPECIES	-17.74000	-51.04000	1264890847
2609	Diptera	Culicidae	Aedes	Aedes aegypti	SPECIES	-17.72000	-51.04000	1264873715
2610	Diptera	Culicidae	Aedes	Aedes aegypti	SPECIES	-17.72000	-149.32000	1264890238
2611	Diptera	Culicidae	Aedes	Aedes aegypti	SPECIES	-17.70000	-42.49000	1264873724
2612	Diptera	Culicidae	Aedes	Aedes aegypti	SPECIES	-17.70000	-149.57000	1264889604
2613	Diptera	Culicidae	Aedes	Aedes aegypti	SPECIES	-17.69000	-41.36000	1264873709
2614	Diptera	Culicidae	Aedes	Aedes aegypti	SPECIES	-17.69000	-49.46000	1264873710
2615	Diptera	Culicidae	Aedes	Aedes aegypti	SPECIES	-17.69000	178.81000	1264890046
2616	Diptera	Culicidae	Aedes	Aedes aegypti	SPECIES	-17.68000	-149.31000	1264890234
2617	Diptera	Culicidae	Aedes	Aedes aegypti	SPECIES	-17.68000	-149.58000	1264891110
2618	Diptera	Culicidae	Aedes	Aedes aegypti	SPECIES	-17.67000	-40.85000	1264873707
2619	Diptera	Culicidae	Aedes	Aedes aegypti	SPECIES	-17.67000	-149.59000	1264888512
2620	Diptera	Culicidae	Aedes	Aedes aegypti	SPECIES	-17.66667	141.06667	177194551
2621	Diptera	Culicidae	Aedes	Aedes aegypti	SPECIES	-17.66667	141.06667	1633663738
2622	Diptera	Culicidae	Aedes	Aedes aegypti	SPECIES	-17.65000	-45.97000	1264873728
2623	Diptera	Culicidae	Aedes	Aedes aegypti	SPECIES	-17.65000	-44.13000	1264873729
2624	Diptera	Culicidae	Aedes	Aedes aegypti	SPECIES	-17.65000	-149.32000	1264888497

№	Order	Family	Genus	Species	Taxon Rank	Latitude	Longitude	GBIF_ID
2625	Diptera	Culicidae	Aedes	Aedes aegypti	SPECIES	-17.64000	-149.60000	1264890170
2626	Diptera	Culicidae	Aedes	Aedes aegypti	SPECIES	-17.63000	-39.70000	1264873726
2627	Diptera	Culicidae	Aedes	Aedes aegypti	SPECIES	-17.63000	145.48000	1264891336
2628	Diptera	Culicidae	Aedes	Aedes aegypti	SPECIES	-17.62000	-54.42000	1264873741
2629	Diptera	Culicidae	Aedes	Aedes aegypti	SPECIES	-17.61000	-41.84000	1264873732
2630	Diptera	Culicidae	Aedes	Aedes aegypti	SPECIES	-17.61000	-42.13000	1264873739
2631	Diptera	Culicidae	Aedes	Aedes aegypti	SPECIES	-17.61000	177.46000	1264889087
2632	Diptera	Culicidae	Aedes	Aedes aegypti	SPECIES	-17.61000	177.45000	1264889749
2633	Diptera	Culicidae	Aedes	Aedes aegypti	SPECIES	-17.61000	-149.31000	1264890196
2634	Diptera	Culicidae	Aedes	Aedes aegypti	SPECIES	-17.58000	-149.82000	1264890229
2635	Diptera	Culicidae	Aedes	Aedes aegypti	SPECIES	-17.57000	-149.61000	1264888499
2636	Diptera	Culicidae	Aedes	Aedes aegypti	SPECIES	-17.57000	-149.85000	1264889653
2637	Diptera	Culicidae	Aedes	Aedes aegypti	SPECIES	-17.56000	-52.67000	1264873722
2638	Diptera	Culicidae	Aedes	Aedes aegypti	SPECIES	-17.56000	-40.30000	1264873738
2639	Diptera	Culicidae	Aedes	Aedes aegypti	SPECIES	-17.55000	-43.38000	1264873746
2640	Diptera	Culicidae	Aedes	Aedes aegypti	SPECIES	-17.55000	-149.34000	1264889619
2641	Diptera	Culicidae	Aedes	Aedes aegypti	SPECIES	-17.55000	-149.60000	1264890227
2642	Diptera	Culicidae	Aedes	Aedes aegypti	SPECIES	-17.54000	-49.56000	1264873730
2643	Diptera	Culicidae	Aedes	Aedes aegypti	SPECIES	-17.54000	-149.55000	1264889829
2644	Diptera	Culicidae	Aedes	Aedes aegypti	SPECIES	-17.54000	-149.57000	1264890213
2645	Diptera	Culicidae	Aedes	Aedes aegypti	SPECIES	-17.53500	-149.52500	177195176
2646	Diptera	Culicidae	Aedes	Aedes aegypti	SPECIES	-17.53333	146.01666	1633667497
2647	Diptera	Culicidae	Aedes	Aedes aegypti	SPECIES	-17.53333	-149.56670	1633663746
2648	Diptera	Culicidae	Aedes	Aedes aegypti	SPECIES	-17.53333	146.01666	177194620
2649	Diptera	Culicidae	Aedes	Aedes aegypti	SPECIES	-17.53333	-149.56670	177195175
2650	Diptera	Culicidae	Aedes	Aedes aegypti	SPECIES	-17.53000	-47.75000	1264873719
2651	Diptera	Culicidae	Aedes	Aedes aegypti	SPECIES	-17.53000	-149.57000	1264888098
2652	Diptera	Culicidae	Aedes	Aedes aegypti	SPECIES	-17.53000	146.03000	1264888831
2653	Diptera	Culicidae	Aedes	Aedes aegypti	SPECIES	-17.53000	-149.53000	1264890210
2654	Diptera	Culicidae	Aedes	Aedes aegypti	SPECIES	-17.53000	-149.54000	1264890241
2655	Diptera	Culicidae	Aedes	Aedes aegypti	SPECIES	-17.53000	-149.56000	1264891211
2656	Diptera	Culicidae	Aedes	Aedes aegypti	SPECIES	-17.52000	-50.63000	1264873758
2657	Diptera	Culicidae	Aedes	Aedes aegypti	SPECIES	-17.52000	146.03000	1264887715
2658	Diptera	Culicidae	Aedes	Aedes aegypti	SPECIES	-17.52000	-149.78000	1264889883
2659	Diptera	Culicidae	Aedes	Aedes aegypti	SPECIES	-17.52000	-149.85000	1264890224
2660	Diptera	Culicidae	Aedes	Aedes aegypti	SPECIES	-17.52000	-39.71000	1264890753
2661	Diptera	Culicidae	Aedes	Aedes aegypti	SPECIES	-17.51000	-49.96000	1264873725
2662	Diptera	Culicidae	Aedes	Aedes aegypti	SPECIES	-17.51000	-43.04000	1264873727
2663	Diptera	Culicidae	Aedes	Aedes aegypti	SPECIES	-17.51000	-42.71000	1264873745
2664	Diptera	Culicidae	Aedes	Aedes aegypti	SPECIES	-17.51000	-149.44000	1264888510
2665	Diptera	Culicidae	Aedes	Aedes aegypti	SPECIES	-17.51000	-149.83000	1264889835

№	Order	Family	Genus	Species	Taxon Rank	Latitude	Longitude	GBIF_ID
2666	Diptera	Culicidae	Aedes	Aedes aegypti	SPECIES	-17.51000	-149.43000	1264890194
2667	Diptera	Culicidae	Aedes	Aedes aegypti	SPECIES	-17.51000	-149.49000	1264890242
2668	Diptera	Culicidae	Aedes	Aedes aegypti	SPECIES	-17.50000	-149.84000	1264889863
2669	Diptera	Culicidae	Aedes	Aedes aegypti	SPECIES	-17.50000	-39.21000	1264890742
2670	Diptera	Culicidae	Aedes	Aedes aegypti	SPECIES	-17.50000	-149.83000	1264891134
2671	Diptera	Culicidae	Aedes	Aedes aegypti	SPECIES	-17.49062	-149.82699	3947092580
2672	Diptera	Culicidae	Aedes	Aedes aegypti	SPECIES	-17.49020	-149.82645	3912017638
2673	Diptera	Culicidae	Aedes	Aedes aegypti	SPECIES	-17.49000	-52.26000	1264873743
2674	Diptera	Culicidae	Aedes	Aedes aegypti	SPECIES	-17.49000	140.84000	1264889066
2675	Diptera	Culicidae	Aedes	Aedes aegypti	SPECIES	-17.49000	-149.76000	3348220868
2676	Diptera	Culicidae	Aedes	Aedes aegypti	SPECIES	-17.49000	-149.83000	3348377137
2677	Diptera	Culicidae	Aedes	Aedes aegypti	SPECIES	-17.48000	-149.78000	1264889650
2678	Diptera	Culicidae	Aedes	Aedes aegypti	SPECIES	-17.47000	-39.38000	1264873723
2679	Diptera	Culicidae	Aedes	Aedes aegypti	SPECIES	-17.46000	-41.09000	1264873736
2680	Diptera	Culicidae	Aedes	Aedes aegypti	SPECIES	-17.45190	-66.12838	2620065329
2681	Diptera	Culicidae	Aedes	Aedes aegypti	SPECIES	-17.45000	-48.03000	1264873753
2682	Diptera	Culicidae	Aedes	Aedes aegypti	SPECIES	-17.43000	-48.21000	1264873731
2683	Diptera	Culicidae	Aedes	Aedes aegypti	SPECIES	-17.43000	-44.27000	1264873737
2684	Diptera	Culicidae	Aedes	Aedes aegypti	SPECIES	-17.43000	-39.83000	1264873740
2685	Diptera	Culicidae	Aedes	Aedes aegypti	SPECIES	-17.42000	-53.48000	1264873734
2686	Diptera	Culicidae	Aedes	Aedes aegypti	SPECIES	-17.42000	-44.74000	1264873742
2687	Diptera	Culicidae	Aedes	Aedes aegypti	SPECIES	-17.42000	-41.67000	1264873749
2688	Diptera	Culicidae	Aedes	Aedes aegypti	SPECIES	-17.42000	-50.31000	1264873754
2689	Diptera	Culicidae	Aedes	Aedes aegypti	SPECIES	-17.41000	-49.70000	1264873751
2690	Diptera	Culicidae	Aedes	Aedes aegypti	SPECIES	-17.39042	-66.32572	2610977786
2691	Diptera	Culicidae	Aedes	Aedes aegypti	SPECIES	-17.39000	-43.24000	1264873735
2692	Diptera	Culicidae	Aedes	Aedes aegypti	SPECIES	-17.39000	-48.61000	1264873755
2693	Diptera	Culicidae	Aedes	Aedes aegypti	SPECIES	-17.38333	145.10001	1633667622
2694	Diptera	Culicidae	Aedes	Aedes aegypti	SPECIES	-17.38333	145.10001	177194588
2695	Diptera	Culicidae	Aedes	Aedes aegypti	SPECIES	-17.38000	-44.87000	1264873763
2696	Diptera	Culicidae	Aedes	Aedes aegypti	SPECIES	-17.37000	-41.50000	1264873747
2697	Diptera	Culicidae	Aedes	Aedes aegypti	SPECIES	-17.36000	-41.97000	1264873744
2698	Diptera	Culicidae	Aedes	Aedes aegypti	SPECIES	-17.36000	-40.28000	1264873750
2699	Diptera	Culicidae	Aedes	Aedes aegypti	SPECIES	-17.36000	-42.39000	1264873761
2700	Diptera	Culicidae	Aedes	Aedes aegypti	SPECIES	-17.35000	144.68333	177194489
2701	Diptera	Culicidae	Aedes	Aedes aegypti	SPECIES	-17.35000	-39.21000	1264891149
2702	Diptera	Culicidae	Aedes	Aedes aegypti	SPECIES	-17.35000	144.68333	1633663753
2703	Diptera	Culicidae	Aedes	Aedes aegypti	SPECIES	-17.34000	-52.71000	1264873778
2704	Diptera	Culicidae	Aedes	Aedes aegypti	SPECIES	-17.34000	-63.26000	1264887268
2705	Diptera	Culicidae	Aedes	Aedes aegypti	SPECIES	-17.33333	145.21666	1633663732
2706	Diptera	Culicidae	Aedes	Aedes aegypti	SPECIES	-17.33333	145.21666	177194490

№	Order	Family	Genus	Species	Taxon Rank	Latitude	Longitude	GBIF_ID
2707	Diptera	Culicidae	Aedes	Aedes aegypti	SPECIES	-17.33000	-49.40000	1264873748
2708	Diptera	Culicidae	Aedes	Aedes aegypti	SPECIES	-17.32000	-54.64000	1264873762
2709	Diptera	Culicidae	Aedes	Aedes aegypti	SPECIES	-17.31000	-48.29000	1264873765
2710	Diptera	Culicidae	Aedes	Aedes aegypti	SPECIES	-17.31000	-49.00000	1264873767
2711	Diptera	Culicidae	Aedes	Aedes aegypti	SPECIES	-17.31000	-48.40000	1264873771
2712	Diptera	Culicidae	Aedes	Aedes aegypti	SPECIES	-17.30000	-44.00000	1264873783
2713	Diptera	Culicidae	Aedes	Aedes aegypti	SPECIES	-17.28000	-45.18000	1264873752
2714	Diptera	Culicidae	Aedes	Aedes aegypti	SPECIES	-17.28000	-49.25000	1264873770
2715	Diptera	Culicidae	Aedes	Aedes aegypti	SPECIES	-17.27000	-40.67000	1264873768
2716	Diptera	Culicidae	Aedes	Aedes aegypti	SPECIES	-17.26000	-42.82000	1264873774
2717	Diptera	Culicidae	Aedes	Aedes aegypti	SPECIES	-17.26000	145.48000	1264891358
2718	Diptera	Culicidae	Aedes	Aedes aegypti	SPECIES	-17.25000	-53.07000	1264873760
2719	Diptera	Culicidae	Aedes	Aedes aegypti	SPECIES	-17.25000	-49.37000	1264873772
2720	Diptera	Culicidae	Aedes	Aedes aegypti	SPECIES	-17.25000	-51.24000	1264873780
2721	Diptera	Culicidae	Aedes	Aedes aegypti	SPECIES	-17.24000	-48.75000	1264873757
2722	Diptera	Culicidae	Aedes	Aedes aegypti	SPECIES	-17.24000	-41.21000	1264873769
2723	Diptera	Culicidae	Aedes	Aedes aegypti	SPECIES	-17.24000	-40.98000	1264873788
2724	Diptera	Culicidae	Aedes	Aedes aegypti	SPECIES	-17.24000	-61.51000	1264891003
2725	Diptera	Culicidae	Aedes	Aedes aegypti	SPECIES	-17.23000	145.48000	1264891357
2726	Diptera	Culicidae	Aedes	Aedes aegypti	SPECIES	-17.21000	-49.93000	1264873779
2727	Diptera	Culicidae	Aedes	Aedes aegypti	SPECIES	-17.20000	-44.48000	1264873764
2728	Diptera	Culicidae	Aedes	Aedes aegypti	SPECIES	-17.19000	-42.21000	1264873790
2729	Diptera	Culicidae	Aedes	Aedes aegypti	SPECIES	-17.17000	-39.37000	1264873756
2730	Diptera	Culicidae	Aedes	Aedes aegypti	SPECIES	-17.16000	-41.59000	1264873781
2731	Diptera	Culicidae	Aedes	Aedes aegypti	SPECIES	-17.16000	-42.39000	1264873792
2732	Diptera	Culicidae	Aedes	Aedes aegypti	SPECIES	-17.16000	144.52000	1264886680
2733	Diptera	Culicidae	Aedes	Aedes aegypti	SPECIES	-17.15000	144.51666	177194487
2734	Diptera	Culicidae	Aedes	Aedes aegypti	SPECIES	-17.15000	145.82000	1264891048
2735	Diptera	Culicidae	Aedes	Aedes aegypti	SPECIES	-17.15000	144.53000	1264891373
2736	Diptera	Culicidae	Aedes	Aedes aegypti	SPECIES	-17.15000	144.51666	1633667375
2737	Diptera	Culicidae	Aedes	Aedes aegypti	SPECIES	-17.13000	-39.60000	1264873759
2738	Diptera	Culicidae	Aedes	Aedes aegypti	SPECIES	-17.13000	-50.17000	1264873797
2739	Diptera	Culicidae	Aedes	Aedes aegypti	SPECIES	-17.11000	-46.87000	1264873766
2740	Diptera	Culicidae	Aedes	Aedes aegypti	SPECIES	-17.11000	-40.35000	1264873776
2741	Diptera	Culicidae	Aedes	Aedes aegypti	SPECIES	-17.11000	-43.81000	1264891449
2742	Diptera	Culicidae	Aedes	Aedes aegypti	SPECIES	-17.10000	-49.75000	1264873808
2743	Diptera	Culicidae	Aedes	Aedes aegypti	SPECIES	-17.09000	-49.61000	1264873777
2744	Diptera	Culicidae	Aedes	Aedes aegypti	SPECIES	-17.09000	-40.71000	1264873787
2745	Diptera	Culicidae	Aedes	Aedes aegypti	SPECIES	-17.09000	145.79000	1264887712
2746	Diptera	Culicidae	Aedes	Aedes aegypti	SPECIES	-17.08000	145.78000	1264891360
2747	Diptera	Culicidae	Aedes	Aedes aegypti	SPECIES	-17.07000	-43.60000	1264873789

№	Order	Family	Genus	Species	Taxon Rank	Latitude	Longitude	GBIF_ID
2748	Diptera	Culicidae	Aedes	Aedes aegypti	SPECIES	-17.06000	-44.22000	1264873782
2749	Diptera	Culicidae	Aedes	Aedes aegypti	SPECIES	-17.06000	-41.54000	1264873793
2750	Diptera	Culicidae	Aedes	Aedes aegypti	SPECIES	-17.06000	-42.73000	1264873795
2751	Diptera	Culicidae	Aedes	Aedes aegypti	SPECIES	-17.06000	-39.53000	1264890773
2752	Diptera	Culicidae	Aedes	Aedes aegypti	SPECIES	-17.05000	-50.64000	1264873804
2753	Diptera	Culicidae	Aedes	Aedes aegypti	SPECIES	-17.04000	-40.98000	1264873775
2754	Diptera	Culicidae	Aedes	Aedes aegypti	SPECIES	-17.02000	-48.20000	1264873786
2755	Diptera	Culicidae	Aedes	Aedes aegypti	SPECIES	-17.01000	-44.66000	1264873773
2756	Diptera	Culicidae	Aedes	Aedes aegypti	SPECIES	-17.01000	-49.28000	1264873803
2757	Diptera	Culicidae	Aedes	Aedes aegypti	SPECIES	-17.01000	-65.46000	1264887249
2758	Diptera	Culicidae	Aedes	Aedes aegypti	SPECIES	-17.00000	145.43333	177194593
2759	Diptera	Culicidae	Aedes	Aedes aegypti	SPECIES	-17.00000	-48.67000	1264873801
2760	Diptera	Culicidae	Aedes	Aedes aegypti	SPECIES	-17.00000	145.43000	1264891387
2761	Diptera	Culicidae	Aedes	Aedes aegypti	SPECIES	-17.00000	145.43333	1633663681
2762	Diptera	Culicidae	Aedes	Aedes aegypti	SPECIES	-16.99000	-39.76000	1264873784
2763	Diptera	Culicidae	Aedes	Aedes aegypti	SPECIES	-16.99000	-42.29000	1264873791
2764	Diptera	Culicidae	Aedes	Aedes aegypti	SPECIES	-16.99000	145.42000	1264887729
2765	Diptera	Culicidae	Aedes	Aedes aegypti	SPECIES	-16.98000	-51.74000	1264873799
2766	Diptera	Culicidae	Aedes	Aedes aegypti	SPECIES	-16.98000	-61.15000	1264891193
2767	Diptera	Culicidae	Aedes	Aedes aegypti	SPECIES	-16.97000	-48.90000	1264873818
2768	Diptera	Culicidae	Aedes	Aedes aegypti	SPECIES	-16.97000	-65.42000	1264887285
2769	Diptera	Culicidae	Aedes	Aedes aegypti	SPECIES	-16.96000	145.73000	1264887773
2770	Diptera	Culicidae	Aedes	Aedes aegypti	SPECIES	-16.95532	-49.22063	4022278372
2771	Diptera	Culicidae	Aedes	Aedes aegypti	SPECIES	-16.95000	-42.97000	1264873796
2772	Diptera	Culicidae	Aedes	Aedes aegypti	SPECIES	-16.94000	-45.86000	1264873805
2773	Diptera	Culicidae	Aedes	Aedes aegypti	SPECIES	-16.94000	-49.42000	1264873815
2774	Diptera	Culicidae	Aedes	Aedes aegypti	SPECIES	-16.94000	145.75000	1264887787
2775	Diptera	Culicidae	Aedes	Aedes aegypti	SPECIES	-16.93000	145.77000	1264887330
2776	Diptera	Culicidae	Aedes	Aedes aegypti	SPECIES	-16.93000	145.76000	1264887810
2777	Diptera	Culicidae	Aedes	Aedes aegypti	SPECIES	-16.93000	145.74000	1264889097
2778	Diptera	Culicidae	Aedes	Aedes aegypti	SPECIES	-16.92000	-49.59000	1264873822
2779	Diptera	Culicidae	Aedes	Aedes aegypti	SPECIES	-16.92000	145.77000	1264887615
2780	Diptera	Culicidae	Aedes	Aedes aegypti	SPECIES	-16.92000	145.76000	1264890949
2781	Diptera	Culicidae	Aedes	Aedes aegypti	SPECIES	-16.91667	145.76666	177194599
2782	Diptera	Culicidae	Aedes	Aedes aegypti	SPECIES	-16.91667	145.76666	1633667692
2783	Diptera	Culicidae	Aedes	Aedes aegypti	SPECIES	-16.91000	-40.66000	1264873807
2784	Diptera	Culicidae	Aedes	Aedes aegypti	SPECIES	-16.91000	145.74000	1264887799
2785	Diptera	Culicidae	Aedes	Aedes aegypti	SPECIES	-16.91000	-61.58000	1264891136
2786	Diptera	Culicidae	Aedes	Aedes aegypti	SPECIES	-16.91000	145.76000	1264891370
2787	Diptera	Culicidae	Aedes	Aedes aegypti	SPECIES	-16.90000	-42.65000	1264873785
2788	Diptera	Culicidae	Aedes	Aedes aegypti	SPECIES	-16.90000	-52.48000	1264873794

№	Order	Family	Genus	Species	Taxon Rank	Latitude	Longitude	GBIF_ID
2789	Diptera	Culicidae	Aedes	Aedes aegypti	SPECIES	-16.90000	-43.63000	1264873798
2790	Diptera	Culicidae	Aedes	Aedes aegypti	SPECIES	-16.90000	-41.94000	1264873813
2791	Diptera	Culicidae	Aedes	Aedes aegypti	SPECIES	-16.90000	145.72000	1264887575
2792	Diptera	Culicidae	Aedes	Aedes aegypti	SPECIES	-16.90000	145.74000	1264887785
2793	Diptera	Culicidae	Aedes	Aedes aegypti	SPECIES	-16.90000	145.73000	1264887801
2794	Diptera	Culicidae	Aedes	Aedes aegypti	SPECIES	-16.89000	145.70000	1264887814
2795	Diptera	Culicidae	Aedes	Aedes aegypti	SPECIES	-16.88000	-41.25000	1264873802
2796	Diptera	Culicidae	Aedes	Aedes aegypti	SPECIES	-16.88000	-48.43000	1264873806
2797	Diptera	Culicidae	Aedes	Aedes aegypti	SPECIES	-16.88000	-40.08000	1264873811
2798	Diptera	Culicidae	Aedes	Aedes aegypti	SPECIES	-16.88000	-42.50000	1264873812
2799	Diptera	Culicidae	Aedes	Aedes aegypti	SPECIES	-16.88000	145.71000	1264887788
2800	Diptera	Culicidae	Aedes	Aedes aegypti	SPECIES	-16.88000	145.73000	1264887809
2801	Diptera	Culicidae	Aedes	Aedes aegypti	SPECIES	-16.87000	145.76000	1264888783
2802	Diptera	Culicidae	Aedes	Aedes aegypti	SPECIES	-16.85000	-44.32000	1264873800
2803	Diptera	Culicidae	Aedes	Aedes aegypti	SPECIES	-16.85000	-43.57000	1264873820
2804	Diptera	Culicidae	Aedes	Aedes aegypti	SPECIES	-16.85000	145.75000	1264887316
2805	Diptera	Culicidae	Aedes	Aedes aegypti	SPECIES	-16.85000	145.74000	1264888790
2806	Diptera	Culicidae	Aedes	Aedes aegypti	SPECIES	-16.84000	-49.94000	1264873814
2807	Diptera	Culicidae	Aedes	Aedes aegypti	SPECIES	-16.84000	-41.52000	1264873833
2808	Diptera	Culicidae	Aedes	Aedes aegypti	SPECIES	-16.84000	-151.48000	1264887161
2809	Diptera	Culicidae	Aedes	Aedes aegypti	SPECIES	-16.84000	-179.98000	1264890070
2810	Diptera	Culicidae	Aedes	Aedes aegypti	SPECIES	-16.84000	145.74000	3818482877
2811	Diptera	Culicidae	Aedes	Aedes aegypti	SPECIES	-16.83000	-53.58000	1264873819
2812	Diptera	Culicidae	Aedes	Aedes aegypti	SPECIES	-16.83000	-44.81000	1264873828
2813	Diptera	Culicidae	Aedes	Aedes aegypti	SPECIES	-16.82000	-151.42000	3348302302
2814	Diptera	Culicidae	Aedes	Aedes aegypti	SPECIES	-16.81000	-49.26000	1264873809
2815	Diptera	Culicidae	Aedes	Aedes aegypti	SPECIES	-16.81000	-50.20000	1264873829
2816	Diptera	Culicidae	Aedes	Aedes aegypti	SPECIES	-16.81000	-50.37000	1264873840
2817	Diptera	Culicidae	Aedes	Aedes aegypti	SPECIES	-16.81000	145.72000	1264890564
2818	Diptera	Culicidae	Aedes	Aedes aegypti	SPECIES	-16.81000	-49.27000	1264890826
2819	Diptera	Culicidae	Aedes	Aedes aegypti	SPECIES	-16.81000	145.65000	1264891391
2820	Diptera	Culicidae	Aedes	Aedes aegypti	SPECIES	-16.80000	-40.99000	1264873816
2821	Diptera	Culicidae	Aedes	Aedes aegypti	SPECIES	-16.80000	-56.95000	1264873843
2822	Diptera	Culicidae	Aedes	Aedes aegypti	SPECIES	-16.80000	-151.44000	1264890137
2823	Diptera	Culicidae	Aedes	Aedes aegypti	SPECIES	-16.79000	-49.47000	1264873824
2824	Diptera	Culicidae	Aedes	Aedes aegypti	SPECIES	-16.79000	-54.23000	1264873832
2825	Diptera	Culicidae	Aedes	Aedes aegypti	SPECIES	-16.79000	145.69000	1264887703
2826	Diptera	Culicidae	Aedes	Aedes aegypti	SPECIES	-16.78000	-49.69000	1264873826
2827	Diptera	Culicidae	Aedes	Aedes aegypti	SPECIES	-16.78000	-40.37000	1264873830
2828	Diptera	Culicidae	Aedes	Aedes aegypti	SPECIES	-16.76000	-56.11000	1264873821
2829	Diptera	Culicidae	Aedes	Aedes aegypti	SPECIES	-16.74000	-46.31000	1264873838

№	Order	Family	Genus	Species	Taxon Rank	Latitude	Longitude	GBIF_ID
2830	Diptera	Culicidae	Aedes	Aedes aegypti	SPECIES	-16.73000	-45.56000	1264873817
2831	Diptera	Culicidae	Aedes	Aedes aegypti	SPECIES	-16.73000	-48.96000	1264873823
2832	Diptera	Culicidae	Aedes	Aedes aegypti	SPECIES	-16.72914	-43.86707	3925497649
2833	Diptera	Culicidae	Aedes	Aedes aegypti	SPECIES	-16.71000	-49.10000	1264873810
2834	Diptera	Culicidae	Aedes	Aedes aegypti	SPECIES	-16.71000	-42.80000	1264873836
2835	Diptera	Culicidae	Aedes	Aedes aegypti	SPECIES	-16.71000	-50.70000	1264873837
2836	Diptera	Culicidae	Aedes	Aedes aegypti	SPECIES	-16.71000	-151.03000	1264890132
2837	Diptera	Culicidae	Aedes	Aedes aegypti	SPECIES	-16.70878	-49.09185	3067778488
2838	Diptera	Culicidae	Aedes	Aedes aegypti	SPECIES	-16.70000	-53.14000	1264873827
2839	Diptera	Culicidae	Aedes	Aedes aegypti	SPECIES	-16.70000	-51.44000	1264873831
2840	Diptera	Culicidae	Aedes	Aedes aegypti	SPECIES	-16.70000	-47.52000	1264873847
2841	Diptera	Culicidae	Aedes	Aedes aegypti	SPECIES	-16.69000	-50.79000	1264873845
2842	Diptera	Culicidae	Aedes	Aedes aegypti	SPECIES	-16.69000	-42.32000	1264873859
2843	Diptera	Culicidae	Aedes	Aedes aegypti	SPECIES	-16.69000	-49.26000	1264890674
2844	Diptera	Culicidae	Aedes	Aedes aegypti	SPECIES	-16.68333	145.33333	1633663757
2845	Diptera	Culicidae	Aedes	Aedes aegypti	SPECIES	-16.68333	145.33333	177194592
2846	Diptera	Culicidae	Aedes	Aedes aegypti	SPECIES	-16.68000	-40.56000	1264873825
2847	Diptera	Culicidae	Aedes	Aedes aegypti	SPECIES	-16.68000	-50.51000	1264873839
2848	Diptera	Culicidae	Aedes	Aedes aegypti	SPECIES	-16.67000	-52.94000	1264873844
2849	Diptera	Culicidae	Aedes	Aedes aegypti	SPECIES	-16.67000	-39.52000	1264873854
2850	Diptera	Culicidae	Aedes	Aedes aegypti	SPECIES	-16.66000	-51.12000	1264873853
2851	Diptera	Culicidae	Aedes	Aedes aegypti	SPECIES	-16.66000	-40.74000	1264873868
2852	Diptera	Culicidae	Aedes	Aedes aegypti	SPECIES	-16.65000	-49.54000	1264873841
2853	Diptera	Culicidae	Aedes	Aedes aegypti	SPECIES	-16.64000	-49.26000	1264873849
2854	Diptera	Culicidae	Aedes	Aedes aegypti	SPECIES	-16.64000	-49.28000	1264890823
2855	Diptera	Culicidae	Aedes	Aedes aegypti	SPECIES	-16.63000	-39.29000	1264873846
2856	Diptera	Culicidae	Aedes	Aedes aegypti	SPECIES	-16.63000	-50.35000	1264873851
2857	Diptera	Culicidae	Aedes	Aedes aegypti	SPECIES	-16.62000	-44.41000	1264873834
2858	Diptera	Culicidae	Aedes	Aedes aegypti	SPECIES	-16.62000	-43.93000	1264890832
2859	Diptera	Culicidae	Aedes	Aedes aegypti	SPECIES	-16.62000	-39.29000	1264891014
2860	Diptera	Culicidae	Aedes	Aedes aegypti	SPECIES	-16.61000	-44.96000	1264873835
2861	Diptera	Culicidae	Aedes	Aedes aegypti	SPECIES	-16.61000	-43.94000	1264873852
2862	Diptera	Culicidae	Aedes	Aedes aegypti	SPECIES	-16.60525	-49.34527	3408231167
2863	Diptera	Culicidae	Aedes	Aedes aegypti	SPECIES	-16.60000	-48.55000	1264873842
2864	Diptera	Culicidae	Aedes	Aedes aegypti	SPECIES	-16.60000	-41.85000	1264873848
2865	Diptera	Culicidae	Aedes	Aedes aegypti	SPECIES	-16.60000	-42.20000	1264873855
2866	Diptera	Culicidae	Aedes	Aedes aegypti	SPECIES	-16.60000	-49.67000	1264873864
2867	Diptera	Culicidae	Aedes	Aedes aegypti	SPECIES	-16.59000	-49.00000	1264873865
2868	Diptera	Culicidae	Aedes	Aedes aegypti	SPECIES	-16.58000	-49.87000	1264873857
2869	Diptera	Culicidae	Aedes	Aedes aegypti	SPECIES	-16.58000	-50.12000	1264873862
2870	Diptera	Culicidae	Aedes	Aedes aegypti	SPECIES	-16.58000	-41.52000	1264873872

№	Order	Family	Genus	Species	Taxon Rank	Latitude	Longitude	GBIF_ID
2871	Diptera	Culicidae	Aedes	Aedes aegypti	SPECIES	-16.57000	-54.61000	1264873856
2872	Diptera	Culicidae	Aedes	Aedes aegypti	SPECIES	-16.56000	-48.87000	1264873861
2873	Diptera	Culicidae	Aedes	Aedes aegypti	SPECIES	-16.56000	-39.56000	1264890751
2874	Diptera	Culicidae	Aedes	Aedes aegypti	SPECIES	-16.55000	-48.02000	1264873850
2875	Diptera	Culicidae	Aedes	Aedes aegypti	SPECIES	-16.55000	-44.54000	1264873870
2876	Diptera	Culicidae	Aedes	Aedes aegypti	SPECIES	-16.54000	-57.92000	1264873863
2877	Diptera	Culicidae	Aedes	Aedes aegypti	SPECIES	-16.54000	-46.48000	1264873884
2878	Diptera	Culicidae	Aedes	Aedes aegypti	SPECIES	-16.53000	-40.26000	1264873869
2879	Diptera	Culicidae	Aedes	Aedes aegypti	SPECIES	-16.52000	-44.78000	1264873866
2880	Diptera	Culicidae	Aedes	Aedes aegypti	SPECIES	-16.52000	-46.20000	1264873878
2881	Diptera	Culicidae	Aedes	Aedes aegypti	SPECIES	-16.52000	-42.54000	1264873879
2882	Diptera	Culicidae	Aedes	Aedes aegypti	SPECIES	-16.52000	-57.84000	1264890831
2883	Diptera	Culicidae	Aedes	Aedes aegypti	SPECIES	-16.51000	-50.79000	1264873874
2884	Diptera	Culicidae	Aedes	Aedes aegypti	SPECIES	-16.51000	-39.98000	1264873892
2885	Diptera	Culicidae	Aedes	Aedes aegypti	SPECIES	-16.50000	145.48330	177194569
2886	Diptera	Culicidae	Aedes	Aedes aegypti	SPECIES	-16.50000	-49.41000	1264873871
2887	Diptera	Culicidae	Aedes	Aedes aegypti	SPECIES	-16.50000	-49.04000	1264873876
2888	Diptera	Culicidae	Aedes	Aedes aegypti	SPECIES	-16.50000	-151.74000	1264887113
2889	Diptera	Culicidae	Aedes	Aedes aegypti	SPECIES	-16.49000	-55.49000	1264873858
2890	Diptera	Culicidae	Aedes	Aedes aegypti	SPECIES	-16.49000	-49.77000	1264873880
2891	Diptera	Culicidae	Aedes	Aedes aegypti	SPECIES	-16.49000	145.46000	1264890551
2892	Diptera	Culicidae	Aedes	Aedes aegypti	SPECIES	-16.48333	145.46666	1633663719
2893	Diptera	Culicidae	Aedes	Aedes aegypti	SPECIES	-16.48333	145.46666	177194595
2894	Diptera	Culicidae	Aedes	Aedes aegypti	SPECIES	-16.48000	-49.29000	1264873860
2895	Diptera	Culicidae	Aedes	Aedes aegypti	SPECIES	-16.48000	145.47000	1264887824
2896	Diptera	Culicidae	Aedes	Aedes aegypti	SPECIES	-16.48000	145.46000	1264891349
2897	Diptera	Culicidae	Aedes	Aedes aegypti	SPECIES	-16.47000	-42.97000	1264873890
2898	Diptera	Culicidae	Aedes	Aedes aegypti	SPECIES	-16.46000	-54.28000	1264873867
2899	Diptera	Culicidae	Aedes	Aedes aegypti	SPECIES	-16.46000	-49.09000	1264873873
2900	Diptera	Culicidae	Aedes	Aedes aegypti	SPECIES	-16.46000	-52.78000	1264873877
2901	Diptera	Culicidae	Aedes	Aedes aegypti	SPECIES	-16.46000	-50.36000	1264873889
2902	Diptera	Culicidae	Aedes	Aedes aegypti	SPECIES	-16.46000	145.37000	1264889074
2903	Diptera	Culicidae	Aedes	Aedes aegypti	SPECIES	-16.46000	145.36000	1264891404
2904	Diptera	Culicidae	Aedes	Aedes aegypti	SPECIES	-16.45000	145.36667	1633663816
2905	Diptera	Culicidae	Aedes	Aedes aegypti	SPECIES	-16.45000	145.36667	177194554
2906	Diptera	Culicidae	Aedes	Aedes aegypti	SPECIES	-16.45000	-51.19000	1264873886
2907	Diptera	Culicidae	Aedes	Aedes aegypti	SPECIES	-16.45000	-49.57000	1264873898
2908	Diptera	Culicidae	Aedes	Aedes aegypti	SPECIES	-16.44000	-43.45000	1264873882
2909	Diptera	Culicidae	Aedes	Aedes aegypti	SPECIES	-16.44000	-40.51000	1264873887
2910	Diptera	Culicidae	Aedes	Aedes aegypti	SPECIES	-16.43000	-49.19000	1264873875
2911	Diptera	Culicidae	Aedes	Aedes aegypti	SPECIES	-16.43000	145.41000	1264887815

№	Order	Family	Genus	Species	Taxon Rank	Latitude	Longitude	GBIF_ID
2912	Diptera	Culicidae	Aedes	Aedes aegypti	SPECIES	-16.43000	-39.08000	1264890752
2913	Diptera	Culicidae	Aedes	Aedes aegypti	SPECIES	-16.41667	145.45000	1633663717
2914	Diptera	Culicidae	Aedes	Aedes aegypti	SPECIES	-16.41000	-49.96000	1264873881
2915	Diptera	Culicidae	Aedes	Aedes aegypti	SPECIES	-16.41000	-48.71000	1264873895
2916	Diptera	Culicidae	Aedes	Aedes aegypti	SPECIES	-16.40000	-41.06000	1264873888
2917	Diptera	Culicidae	Aedes	Aedes aegypti	SPECIES	-16.39000	-50.19000	1264873893
2918	Diptera	Culicidae	Aedes	Aedes aegypti	SPECIES	-16.39000	-46.83000	1264873909
2919	Diptera	Culicidae	Aedes	Aedes aegypti	SPECIES	-16.38000	-53.61000	1264873903
2920	Diptera	Culicidae	Aedes	Aedes aegypti	SPECIES	-16.37000	-51.86000	1264873894
2921	Diptera	Culicidae	Aedes	Aedes aegypti	SPECIES	-16.37000	-44.87000	1264873900
2922	Diptera	Culicidae	Aedes	Aedes aegypti	SPECIES	-16.37000	-49.38000	1264873904
2923	Diptera	Culicidae	Aedes	Aedes aegypti	SPECIES	-16.37000	-49.71000	1264873918
2924	Diptera	Culicidae	Aedes	Aedes aegypti	SPECIES	-16.36000	-50.88000	1264873883
2925	Diptera	Culicidae	Aedes	Aedes aegypti	SPECIES	-16.36000	-42.19000	1264873896
2926	Diptera	Culicidae	Aedes	Aedes aegypti	SPECIES	-16.36000	-49.30000	1264873901
2927	Diptera	Culicidae	Aedes	Aedes aegypti	SPECIES	-16.36000	-50.57000	1264873902
2928	Diptera	Culicidae	Aedes	Aedes aegypti	SPECIES	-16.36000	-39.58000	1264890766
2929	Diptera	Culicidae	Aedes	Aedes aegypti	SPECIES	-16.35000	-45.43000	1264873885
2930	Diptera	Culicidae	Aedes	Aedes aegypti	SPECIES	-16.35000	145.41000	1264887806
2931	Diptera	Culicidae	Aedes	Aedes aegypti	SPECIES	-16.34000	-51.61000	1264873891
2932	Diptera	Culicidae	Aedes	Aedes aegypti	SPECIES	-16.34000	-52.44000	1264873905
2933	Diptera	Culicidae	Aedes	Aedes aegypti	SPECIES	-16.34000	46.85000	1264886071
2934	Diptera	Culicidae	Aedes	Aedes aegypti	SPECIES	-16.33000	-50.23000	1264873897
2935	Diptera	Culicidae	Aedes	Aedes aegypti	SPECIES	-16.31000	-42.60000	1264873907
2936	Diptera	Culicidae	Aedes	Aedes aegypti	SPECIES	-16.31000	-48.97000	1264873911
2937	Diptera	Culicidae	Aedes	Aedes aegypti	SPECIES	-16.31000	-49.52000	1264873914
2938	Diptera	Culicidae	Aedes	Aedes aegypti	SPECIES	-16.31000	-41.50000	1264873916
2939	Diptera	Culicidae	Aedes	Aedes aegypti	SPECIES	-16.30000	-52.84000	1264873906
2940	Diptera	Culicidae	Aedes	Aedes aegypti	SPECIES	-16.30000	-40.12000	1264873912
2941	Diptera	Culicidae	Aedes	Aedes aegypti	SPECIES	-16.30000	-39.63000	1264873924
2942	Diptera	Culicidae	Aedes	Aedes aegypti	SPECIES	-16.29000	-41.77000	1264873899
2943	Diptera	Culicidae	Aedes	Aedes aegypti	SPECIES	-16.28000	-49.99000	1264873913
2944	Diptera	Culicidae	Aedes	Aedes aegypti	SPECIES	-16.28000	-49.10000	1264873920
2945	Diptera	Culicidae	Aedes	Aedes aegypti	SPECIES	-16.28000	-39.03000	1264890740
2946	Diptera	Culicidae	Aedes	Aedes aegypti	SPECIES	-16.27000	-45.96000	1264873934
2947	Diptera	Culicidae	Aedes	Aedes aegypti	SPECIES	-16.26000	-44.14000	1264873917
2948	Diptera	Culicidae	Aedes	Aedes aegypti	SPECIES	-16.25000	-49.34000	1264873928
2949	Diptera	Culicidae	Aedes	Aedes aegypti	SPECIES	-16.25000	-49.23000	1264873929
2950	Diptera	Culicidae	Aedes	Aedes aegypti	SPECIES	-16.25000	168.16000	1264890048
2951	Diptera	Culicidae	Aedes	Aedes aegypti	SPECIES	-16.25000	145.32000	1264890540
2952	Diptera	Culicidae	Aedes	Aedes aegypti	SPECIES	-16.24000	-44.47000	1264873919

№	Order	Family	Genus	Species	Taxon Rank	Latitude	Longitude	GBIF_ID
2953	Diptera	Culicidae	Aedes	Aedes aegypti	SPECIES	-16.23000	-44.87000	1264873944
2954	Diptera	Culicidae	Aedes	Aedes aegypti	SPECIES	-16.22000	-49.45000	1264873921
2955	Diptera	Culicidae	Aedes	Aedes aegypti	SPECIES	-16.22000	-39.22000	1264873925
2956	Diptera	Culicidae	Aedes	Aedes aegypti	SPECIES	-16.20000	-44.60000	1264873908
2957	Diptera	Culicidae	Aedes	Aedes aegypti	SPECIES	-16.20000	-51.23000	1264873926
2958	Diptera	Culicidae	Aedes	Aedes aegypti	SPECIES	-16.20000	-54.85000	1264873927
2959	Diptera	Culicidae	Aedes	Aedes aegypti	SPECIES	-16.19000	-52.07000	1264873910
2960	Diptera	Culicidae	Aedes	Aedes aegypti	SPECIES	-16.19000	-40.32000	1264873915
2961	Diptera	Culicidae	Aedes	Aedes aegypti	SPECIES	-16.18000	-50.77000	1264873922
2962	Diptera	Culicidae	Aedes	Aedes aegypti	SPECIES	-16.18000	-50.16000	1264873930
2963	Diptera	Culicidae	Aedes	Aedes aegypti	SPECIES	-16.17000	-48.69000	1264873932
2964	Diptera	Culicidae	Aedes	Aedes aegypti	SPECIES	-16.17000	-51.05000	1264873935
2965	Diptera	Culicidae	Aedes	Aedes aegypti	SPECIES	-16.17000	-50.46000	1264873941
2966	Diptera	Culicidae	Aedes	Aedes aegypti	SPECIES	-16.15000	-47.82000	1264873938
2967	Diptera	Culicidae	Aedes	Aedes aegypti	SPECIES	-16.13000	-42.52000	1264873923
2968	Diptera	Culicidae	Aedes	Aedes aegypti	SPECIES	-16.13000	-40.71000	1264873931
2969	Diptera	Culicidae	Aedes	Aedes aegypti	SPECIES	-16.13000	-39.70000	1264873950
2970	Diptera	Culicidae	Aedes	Aedes aegypti	SPECIES	-16.12000	-48.49000	1264873942
2971	Diptera	Culicidae	Aedes	Aedes aegypti	SPECIES	-16.12000	-40.04000	1264873945
2972	Diptera	Culicidae	Aedes	Aedes aegypti	SPECIES	-16.12000	-48.08000	1264873946
2973	Diptera	Culicidae	Aedes	Aedes aegypti	SPECIES	-16.12000	-49.84000	1264873955
2974	Diptera	Culicidae	Aedes	Aedes aegypti	SPECIES	-16.12000	47.10000	1264886077
2975	Diptera	Culicidae	Aedes	Aedes aegypti	SPECIES	-16.11000	-49.29000	1264873969
2976	Diptera	Culicidae	Aedes	Aedes aegypti	SPECIES	-16.10000	-46.32000	1264873937
2977	Diptera	Culicidae	Aedes	Aedes aegypti	SPECIES	-16.10000	-43.66000	1264873949
2978	Diptera	Culicidae	Aedes	Aedes aegypti	SPECIES	-16.09000	-44.10000	1264873939
2979	Diptera	Culicidae	Aedes	Aedes aegypti	SPECIES	-16.09000	-42.13000	1264873943
2980	Diptera	Culicidae	Aedes	Aedes aegypti	SPECIES	-16.09000	-47.98000	1264873959
2981	Diptera	Culicidae	Aedes	Aedes aegypti	SPECIES	-16.08000	-39.59000	1264890757
2982	Diptera	Culicidae	Aedes	Aedes aegypti	SPECIES	-16.07000	-48.25000	1264873952
2983	Diptera	Culicidae	Aedes	Aedes aegypti	SPECIES	-16.07000	-49.47000	1264873953
2984	Diptera	Culicidae	Aedes	Aedes aegypti	SPECIES	-16.07000	-47.12000	1264873960
2985	Diptera	Culicidae	Aedes	Aedes aegypti	SPECIES	-16.06000	-45.24000	1264873957
2986	Diptera	Culicidae	Aedes	Aedes aegypti	SPECIES	-16.06000	-50.65000	1264873975
2987	Diptera	Culicidae	Aedes	Aedes aegypti	SPECIES	-16.05000	-41.78000	1264873947
2988	Diptera	Culicidae	Aedes	Aedes aegypti	SPECIES	-16.05000	-49.60000	1264873961
2989	Diptera	Culicidae	Aedes	Aedes aegypti	SPECIES	-16.05000	-47.98000	1264888343
2990	Diptera	Culicidae	Aedes	Aedes aegypti	SPECIES	-16.04000	-42.99000	1264873966
2991	Diptera	Culicidae	Aedes	Aedes aegypti	SPECIES	-16.03000	-48.06000	1264888382
2992	Diptera	Culicidae	Aedes	Aedes aegypti	SPECIES	-16.02000	-45.61000	1264873951
2993	Diptera	Culicidae	Aedes	Aedes aegypti	SPECIES	-16.00242	-48.05505	3124592545

№	Order	Family	Genus	Species	Taxon Rank	Latitude	Longitude	GBIF_ID
2994	Diptera	Culicidae	Aedes	Aedes aegypti	SPECIES	-16.00000	-42.41000	1264873964
2995	Diptera	Culicidae	Aedes	Aedes aegypti	SPECIES	-15.99000	-52.20000	1264873956
2996	Diptera	Culicidae	Aedes	Aedes aegypti	SPECIES	-15.97000	-54.75000	1264873948
2997	Diptera	Culicidae	Aedes	Aedes aegypti	SPECIES	-15.96000	-41.50000	1264873933
2998	Diptera	Culicidae	Aedes	Aedes aegypti	SPECIES	-15.96000	-49.41000	1264873936
2999	Diptera	Culicidae	Aedes	Aedes aegypti	SPECIES	-15.95000	-41.18000	1264873940
3000	Diptera	Culicidae	Aedes	Aedes aegypti	SPECIES	-15.95000	-48.66000	1264873954
3001	Diptera	Culicidae	Aedes	Aedes aegypti	SPECIES	-15.95000	-39.21000	1264873963
3002	Diptera	Culicidae	Aedes	Aedes aegypti	SPECIES	-15.95000	-49.25000	1264873970
3003	Diptera	Culicidae	Aedes	Aedes aegypti	SPECIES	-15.95000	-49.58000	1264891223
3004	Diptera	Culicidae	Aedes	Aedes aegypti	SPECIES	-15.94000	-49.62000	1264873968
3005	Diptera	Culicidae	Aedes	Aedes aegypti	SPECIES	-15.93000	-44.32000	1264873977
3006	Diptera	Culicidae	Aedes	Aedes aegypti	SPECIES	-15.93000	-56.35000	1264873978
3007	Diptera	Culicidae	Aedes	Aedes aegypti	SPECIES	-15.92000	-52.66000	1264873967
3008	Diptera	Culicidae	Aedes	Aedes aegypti	SPECIES	-15.92000	-53.45000	1264873980
3009	Diptera	Culicidae	Aedes	Aedes aegypti	SPECIES	-15.91000	-43.91000	1264873984
3010	Diptera	Culicidae	Aedes	Aedes aegypti	SPECIES	-15.90000	-39.68000	1264873994
3011	Diptera	Culicidae	Aedes	Aedes aegypti	SPECIES	-15.89000	-58.95000	1264873974
3012	Diptera	Culicidae	Aedes	Aedes aegypti	SPECIES	-15.88000	-55.11000	1264873971
3013	Diptera	Culicidae	Aedes	Aedes aegypti	SPECIES	-15.87000	-40.32000	1264873958
3014	Diptera	Culicidae	Aedes	Aedes aegypti	SPECIES	-15.87000	-42.87000	1264873962
3015	Diptera	Culicidae	Aedes	Aedes aegypti	SPECIES	-15.87000	-58.33000	1264873976
3016	Diptera	Culicidae	Aedes	Aedes aegypti	SPECIES	-15.87000	-47.97000	1264888181
3017	Diptera	Culicidae	Aedes	Aedes aegypti	SPECIES	-15.86000	-40.58000	1264873965
3018	Diptera	Culicidae	Aedes	Aedes aegypti	SPECIES	-15.86000	-41.75000	1264873972
3019	Diptera	Culicidae	Aedes	Aedes aegypti	SPECIES	-15.86000	-44.83000	1264873979
3020	Diptera	Culicidae	Aedes	Aedes aegypti	SPECIES	-15.85000	-51.54000	1264873991
3021	Diptera	Culicidae	Aedes	Aedes aegypti	SPECIES	-15.85000	-38.90000	1264890748
3022	Diptera	Culicidae	Aedes	Aedes aegypti	SPECIES	-15.84000	-50.22000	1264873981
3023	Diptera	Culicidae	Aedes	Aedes aegypti	SPECIES	-15.84000	-44.27000	1264873990
3024	Diptera	Culicidae	Aedes	Aedes aegypti	SPECIES	-15.84000	-67.57000	1264887303
3025	Diptera	Culicidae	Aedes	Aedes aegypti	SPECIES	-15.83426	-47.87858	3008431893
3026	Diptera	Culicidae	Aedes	Aedes aegypti	SPECIES	-15.83000	-49.00000	1264874000
3027	Diptera	Culicidae	Aedes	Aedes aegypti	SPECIES	-15.83000	-47.99000	1264888197
3028	Diptera	Culicidae	Aedes	Aedes aegypti	SPECIES	-15.82000	-42.09000	1264873982
3029	Diptera	Culicidae	Aedes	Aedes aegypti	SPECIES	-15.82000	-48.12000	1264888161
3030	Diptera	Culicidae	Aedes	Aedes aegypti	SPECIES	-15.81728	-47.95208	3903661653
3031	Diptera	Culicidae	Aedes	Aedes aegypti	SPECIES	-15.81510	-48.05911	2803140771
3032	Diptera	Culicidae	Aedes	Aedes aegypti	SPECIES	-15.81049	-48.06575	3764231469
3033	Diptera	Culicidae	Aedes	Aedes aegypti	SPECIES	-15.81000	-54.20000	1264873986
3034	Diptera	Culicidae	Aedes	Aedes aegypti	SPECIES	-15.81000	-67.62000	1264887295

№	Order	Family	Genus	Species	Taxon Rank	Latitude	Longitude	GBIF_ID
3035	Diptera	Culicidae	Aedes	Aedes aegypti	SPECIES	-15.80000	-46.01000	1264873985
3036	Diptera	Culicidae	Aedes	Aedes aegypti	SPECIES	-15.79944	-47.80950	3398889468
3037	Diptera	Culicidae	Aedes	Aedes aegypti	SPECIES	-15.79936	-47.80947	3005190989
3038	Diptera	Culicidae	Aedes	Aedes aegypti	SPECIES	-15.79931	-47.80936	3988439212
3039	Diptera	Culicidae	Aedes	Aedes aegypti	SPECIES	-15.78390	-48.13912	4153420530
3040	Diptera	Culicidae	Aedes	Aedes aegypti	SPECIES	-15.78387	-48.13909	3399236728
3041	Diptera	Culicidae	Aedes	Aedes aegypti	SPECIES	-15.78386	-48.13911	3963356691
3042	Diptera	Culicidae	Aedes	Aedes aegypti	SPECIES	-15.78000	-47.80000	1264890818
3043	Diptera	Culicidae	Aedes	Aedes aegypti	SPECIES	-15.77958	-47.87857	2603463549
3044	Diptera	Culicidae	Aedes	Aedes aegypti	SPECIES	-15.77000	-42.50000	1264873973
3045	Diptera	Culicidae	Aedes	Aedes aegypti	SPECIES	-15.77000	-40.93000	1264873983
3046	Diptera	Culicidae	Aedes	Aedes aegypti	SPECIES	-15.77000	-40.71000	1264874019
3047	Diptera	Culicidae	Aedes	Aedes aegypti	SPECIES	-15.77000	-47.93000	1264888201
3048	Diptera	Culicidae	Aedes	Aedes aegypti	SPECIES	-15.76010	-47.88311	2237526138
3049	Diptera	Culicidae	Aedes	Aedes aegypti	SPECIES	-15.76000	-49.61000	1264873996
3050	Diptera	Culicidae	Aedes	Aedes aegypti	SPECIES	-15.75000	-43.04000	1264873999
3051	Diptera	Culicidae	Aedes	Aedes aegypti	SPECIES	-15.75000	-48.30000	1264874005
3052	Diptera	Culicidae	Aedes	Aedes aegypti	SPECIES	-15.75000	-46.99000	1264874009
3053	Diptera	Culicidae	Aedes	Aedes aegypti	SPECIES	-15.75000	-67.67000	1264887307
3054	Diptera	Culicidae	Aedes	Aedes aegypti	SPECIES	-15.74000	-50.79000	1264873992
3055	Diptera	Culicidae	Aedes	Aedes aegypti	SPECIES	-15.73760	-47.86226	3112523254
3056	Diptera	Culicidae	Aedes	Aedes aegypti	SPECIES	-15.73582	-47.83688	2573980539
3057	Diptera	Culicidae	Aedes	Aedes aegypti	SPECIES	-15.73364	-47.90190	3079885176
3058	Diptera	Culicidae	Aedes	Aedes aegypti	SPECIES	-15.73307	-47.87627	3333248342
3059	Diptera	Culicidae	Aedes	Aedes aegypti	SPECIES	-15.73291	-47.87611	2576332013
3060	Diptera	Culicidae	Aedes	Aedes aegypti	SPECIES	-15.73000	-49.81000	1264873988
3061	Diptera	Culicidae	Aedes	Aedes aegypti	SPECIES	-15.73000	-43.42000	1264873993
3062	Diptera	Culicidae	Aedes	Aedes aegypti	SPECIES	-15.72944	-47.85861	3455980917
3063	Diptera	Culicidae	Aedes	Aedes aegypti	SPECIES	-15.72944	-47.85889	3465992904
3064	Diptera	Culicidae	Aedes	Aedes aegypti	SPECIES	-15.72000	-41.55000	1264873987
3065	Diptera	Culicidae	Aedes	Aedes aegypti	SPECIES	-15.72000	-43.27000	1264873997
3066	Diptera	Culicidae	Aedes	Aedes aegypti	SPECIES	-15.72000	-39.78000	1264874003
3067	Diptera	Culicidae	Aedes	Aedes aegypti	SPECIES	-15.72000	-49.37000	1264874004
3068	Diptera	Culicidae	Aedes	Aedes aegypti	SPECIES	-15.72000	46.30000	1264890620
3069	Diptera	Culicidae	Aedes	Aedes aegypti	SPECIES	-15.71000	-41.38000	1264874001
3070	Diptera	Culicidae	Aedes	Aedes aegypti	SPECIES	-15.70000	-41.75000	1264874002
3071	Diptera	Culicidae	Aedes	Aedes aegypti	SPECIES	-15.70000	46.33000	1264886119
3072	Diptera	Culicidae	Aedes	Aedes aegypti	SPECIES	-15.68000	-40.01000	1264874015
3073	Diptera	Culicidae	Aedes	Aedes aegypti	SPECIES	-15.68000	-50.06000	1264874025
3074	Diptera	Culicidae	Aedes	Aedes aegypti	SPECIES	-15.67000	-54.77000	1264874006
3075	Diptera	Culicidae	Aedes	Aedes aegypti	SPECIES	-15.67000	-40.29000	1264874010

№	Order	Family	Genus	Species	Taxon Rank	Latitude	Longitude	GBIF_ID
3076	Diptera	Culicidae	Aedes	Aedes aegypti	SPECIES	-15.66865	-48.20153	3097092752
3077	Diptera	Culicidae	Aedes	Aedes aegypti	SPECIES	-15.66000	-39.41000	1264873989
3078	Diptera	Culicidae	Aedes	Aedes aegypti	SPECIES	-15.66000	-51.11000	1264874011
3079	Diptera	Culicidae	Aedes	Aedes aegypti	SPECIES	-15.65000	-44.10000	1264874013
3080	Diptera	Culicidae	Aedes	Aedes aegypti	SPECIES	-15.65000	-56.14000	1264888083
3081	Diptera	Culicidae	Aedes	Aedes aegypti	SPECIES	-15.64000	-48.55000	1264874016
3082	Diptera	Culicidae	Aedes	Aedes aegypti	SPECIES	-15.64000	-43.93000	1264874030
3083	Diptera	Culicidae	Aedes	Aedes aegypti	SPECIES	-15.63000	-47.82000	1264888173
3084	Diptera	Culicidae	Aedes	Aedes aegypti	SPECIES	-15.62000	-57.89000	1264874007
3085	Diptera	Culicidae	Aedes	Aedes aegypti	SPECIES	-15.62000	-39.12000	1264874020
3086	Diptera	Culicidae	Aedes	Aedes aegypti	SPECIES	-15.62000	-47.67000	1264888215
3087	Diptera	Culicidae	Aedes	Aedes aegypti	SPECIES	-15.62000	-56.09000	1264890412
3088	Diptera	Culicidae	Aedes	Aedes aegypti	SPECIES	-15.61000	-49.65000	1264873998
3089	Diptera	Culicidae	Aedes	Aedes aegypti	SPECIES	-15.61000	-53.02000	1264874017
3090	Diptera	Culicidae	Aedes	Aedes aegypti	SPECIES	-15.61000	-56.09000	1264888186
3091	Diptera	Culicidae	Aedes	Aedes aegypti	SPECIES	-15.60000	-58.08000	1264874018
3092	Diptera	Culicidae	Aedes	Aedes aegypti	SPECIES	-15.58000	-44.36000	1264874012
3093	Diptera	Culicidae	Aedes	Aedes aegypti	SPECIES	-15.58000	-56.08000	1264886069
3094	Diptera	Culicidae	Aedes	Aedes aegypti	SPECIES	-15.57000	-49.93000	1264874027
3095	Diptera	Culicidae	Aedes	Aedes aegypti	SPECIES	-15.56000	-56.30000	1264874021
3096	Diptera	Culicidae	Aedes	Aedes aegypti	SPECIES	-15.56000	-43.64000	1264874029
3097	Diptera	Culicidae	Aedes	Aedes aegypti	SPECIES	-15.55886	-56.06006	3925038783
3098	Diptera	Culicidae	Aedes	Aedes aegypti	SPECIES	-15.55886	-56.06006	3925041891
3099	Diptera	Culicidae	Aedes	Aedes aegypti	SPECIES	-15.54000	-40.42000	1264874034
3100	Diptera	Culicidae	Aedes	Aedes aegypti	SPECIES	-15.53000	-57.24000	1264874014
3101	Diptera	Culicidae	Aedes	Aedes aegypti	SPECIES	-15.53000	-51.44000	1264874022
3102	Diptera	Culicidae	Aedes	Aedes aegypti	SPECIES	-15.53000	-40.94000	1264874024
3103	Diptera	Culicidae	Aedes	Aedes aegypti	SPECIES	-15.53000	-58.30000	1264874045
3104	Diptera	Culicidae	Aedes	Aedes aegypti	SPECIES	-15.51000	167.21000	1264888547
3105	Diptera	Culicidae	Aedes	Aedes aegypti	SPECIES	-15.49000	-58.71000	1264874008
3106	Diptera	Culicidae	Aedes	Aedes aegypti	SPECIES	-15.48000	-49.44000	1264874038
3107	Diptera	Culicidae	Aedes	Aedes aegypti	SPECIES	-15.48000	-145.43000	1264890889
3108	Diptera	Culicidae	Aedes	Aedes aegypti	SPECIES	-15.46667	145.25000	177194590
3109	Diptera	Culicidae	Aedes	Aedes aegypti	SPECIES	-15.46667	145.25000	1633663655
3110	Diptera	Culicidae	Aedes	Aedes aegypti	SPECIES	-15.46000	-39.72000	1264874026
3111	Diptera	Culicidae	Aedes	Aedes aegypti	SPECIES	-15.46000	-40.68000	1264874028
3112	Diptera	Culicidae	Aedes	Aedes aegypti	SPECIES	-15.46000	-42.89000	1264874041
3113	Diptera	Culicidae	Aedes	Aedes aegypti	SPECIES	-15.46000	-45.46000	1264874046
3114	Diptera	Culicidae	Aedes	Aedes aegypti	SPECIES	-15.46000	-39.25000	1264874050
3115	Diptera	Culicidae	Aedes	Aedes aegypti	SPECIES	-15.45000	-50.41000	1264874036
3116	Diptera	Culicidae	Aedes	Aedes aegypti	SPECIES	-15.45000	-57.81000	1264874040

№	Order	Family	Genus	Species	Taxon Rank	Latitude	Longitude	GBIF_ID
3117	Diptera	Culicidae	Aedes	Aedes aegypti	SPECIES	-15.44000	-39.50000	1264874032
3118	Diptera	Culicidae	Aedes	Aedes aegypti	SPECIES	-15.44000	-46.60000	1264874033
3119	Diptera	Culicidae	Aedes	Aedes aegypti	SPECIES	-15.43000	-54.94000	1264874023
3120	Diptera	Culicidae	Aedes	Aedes aegypti	SPECIES	-15.43000	-59.30000	1264874035
3121	Diptera	Culicidae	Aedes	Aedes aegypti	SPECIES	-15.42000	-49.74000	1264874031
3122	Diptera	Culicidae	Aedes	Aedes aegypti	SPECIES	-15.42000	-55.88000	1264874044
3123	Diptera	Culicidae	Aedes	Aedes aegypti	SPECIES	-15.37000	-49.83000	1264874051
3124	Diptera	Culicidae	Aedes	Aedes aegypti	SPECIES	-15.37000	-48.27000	1264874055
3125	Diptera	Culicidae	Aedes	Aedes aegypti	SPECIES	-15.36986	28.31576	4080499398
3126	Diptera	Culicidae	Aedes	Aedes aegypti	SPECIES	-15.36000	-52.64000	1264874043
3127	Diptera	Culicidae	Aedes	Aedes aegypti	SPECIES	-15.36000	-41.98000	1264874054
3128	Diptera	Culicidae	Aedes	Aedes aegypti	SPECIES	-15.35973	28.44569	3058993104
3129	Diptera	Culicidae	Aedes	Aedes aegypti	SPECIES	-15.35972	28.44572	3058903642
3130	Diptera	Culicidae	Aedes	Aedes aegypti	SPECIES	-15.35000	-43.22000	1264874047
3131	Diptera	Culicidae	Aedes	Aedes aegypti	SPECIES	-15.34000	-43.09000	1264874042
3132	Diptera	Culicidae	Aedes	Aedes aegypti	SPECIES	-15.33000	-42.31000	1264874052
3133	Diptera	Culicidae	Aedes	Aedes aegypti	SPECIES	-15.33000	-58.60000	1264874053
3134	Diptera	Culicidae	Aedes	Aedes aegypti	SPECIES	-15.33000	-40.08000	1264874075
3135	Diptera	Culicidae	Aedes	Aedes aegypti	SPECIES	-15.32000	-49.53000	1264874057
3136	Diptera	Culicidae	Aedes	Aedes aegypti	SPECIES	-15.32000	-44.26000	1264874058
3137	Diptera	Culicidae	Aedes	Aedes aegypti	SPECIES	-15.32000	-56.56000	1264874061
3138	Diptera	Culicidae	Aedes	Aedes aegypti	SPECIES	-15.32000	-41.40000	1264874066
3139	Diptera	Culicidae	Aedes	Aedes aegypti	SPECIES	-15.31000	-41.62000	1264874039
3140	Diptera	Culicidae	Aedes	Aedes aegypti	SPECIES	-15.31000	-50.02000	1264874049
3141	Diptera	Culicidae	Aedes	Aedes aegypti	SPECIES	-15.31000	-50.79000	1264874069
3142	Diptera	Culicidae	Aedes	Aedes aegypti	SPECIES	-15.30000	-49.13000	1264874037
3143	Diptera	Culicidae	Aedes	Aedes aegypti	SPECIES	-15.30000	-58.90000	1264874048
3144	Diptera	Culicidae	Aedes	Aedes aegypti	SPECIES	-15.29000	-47.21000	1264874056
3145	Diptera	Culicidae	Aedes	Aedes aegypti	SPECIES	-15.28000	-58.13000	1264874063
3146	Diptera	Culicidae	Aedes	Aedes aegypti	SPECIES	-15.27997	166.91830	3356900315
3147	Diptera	Culicidae	Aedes	Aedes aegypti	SPECIES	-15.27000	-47.79000	1264874060
3148	Diptera	Culicidae	Aedes	Aedes aegypti	SPECIES	-15.27000	-58.49000	1264874064
3149	Diptera	Culicidae	Aedes	Aedes aegypti	SPECIES	-15.27000	-49.66000	1264874076
3150	Diptera	Culicidae	Aedes	Aedes aegypti	SPECIES	-15.25000	-43.69000	1264874067
3151	Diptera	Culicidae	Aedes	Aedes aegypti	SPECIES	-15.24000	-39.44000	1264874071
3152	Diptera	Culicidae	Aedes	Aedes aegypti	SPECIES	-15.24000	-49.39000	1264874079
3153	Diptera	Culicidae	Aedes	Aedes aegypti	SPECIES	-15.22000	-43.01000	1264874077
3154	Diptera	Culicidae	Aedes	Aedes aegypti	SPECIES	-15.22000	-48.83000	1264874078
3155	Diptera	Culicidae	Aedes	Aedes aegypti	SPECIES	-15.22000	-39.18000	1264874100
3156	Diptera	Culicidae	Aedes	Aedes aegypti	SPECIES	-15.21000	-44.06000	1264874082
3157	Diptera	Culicidae	Aedes	Aedes aegypti	SPECIES	-15.21000	-42.46000	1264874091

№	Order	Family	Genus	Species	Taxon Rank	Latitude	Longitude	GBIF_ID
3158	Diptera	Culicidae	Aedes	Aedes aegypti	SPECIES	-15.20000	-51.28000	1264874068
3159	Diptera	Culicidae	Aedes	Aedes aegypti	SPECIES	-15.20000	-56.30000	1264874074
3160	Diptera	Culicidae	Aedes	Aedes aegypti	SPECIES	-15.20000	-49.87000	1264874080
3161	Diptera	Culicidae	Aedes	Aedes aegypti	SPECIES	-15.18000	-40.47000	1264874062
3162	Diptera	Culicidae	Aedes	Aedes aegypti	SPECIES	-15.18000	-57.53000	1264874070
3163	Diptera	Culicidae	Aedes	Aedes aegypti	SPECIES	-15.16000	-39.74000	1264874065
3164	Diptera	Culicidae	Aedes	Aedes aegypti	SPECIES	-15.15000	-49.68000	1264874059
3165	Diptera	Culicidae	Aedes	Aedes aegypti	SPECIES	-15.15000	-39.49000	1264874073
3166	Diptera	Culicidae	Aedes	Aedes aegypti	SPECIES	-15.15000	-56.36000	1264891298
3167	Diptera	Culicidae	Aedes	Aedes aegypti	SPECIES	-15.14000	-60.00000	1264874072
3168	Diptera	Culicidae	Aedes	Aedes aegypti	SPECIES	-15.14000	-50.43000	1264874094
3169	Diptera	Culicidae	Aedes	Aedes aegypti	SPECIES	-15.13000	-54.25000	1264874086
3170	Diptera	Culicidae	Aedes	Aedes aegypti	SPECIES	-15.12000	-148.23000	1264889102
3171	Diptera	Culicidae	Aedes	Aedes aegypti	SPECIES	-15.10000	-55.54000	1264874084
3172	Diptera	Culicidae	Aedes	Aedes aegypti	SPECIES	-15.10000	-39.37000	1264874099
3173	Diptera	Culicidae	Aedes	Aedes aegypti	SPECIES	-15.08000	-51.76000	1264874083
3174	Diptera	Culicidae	Aedes	Aedes aegypti	SPECIES	-15.08000	-49.48000	1264874107
3175	Diptera	Culicidae	Aedes	Aedes aegypti	SPECIES	-15.07000	-40.87000	1264874090
3176	Diptera	Culicidae	Aedes	Aedes aegypti	SPECIES	-15.07000	-46.08000	1264874093
3177	Diptera	Culicidae	Aedes	Aedes aegypti	SPECIES	-15.06000	-53.29000	1264874088
3178	Diptera	Culicidae	Aedes	Aedes aegypti	SPECIES	-15.04000	-44.59000	1264874089
3179	Diptera	Culicidae	Aedes	Aedes aegypti	SPECIES	-15.04000	-49.94000	1264874092
3180	Diptera	Culicidae	Aedes	Aedes aegypti	SPECIES	-15.04000	-40.02000	1264874101
3181	Diptera	Culicidae	Aedes	Aedes aegypti	SPECIES	-15.03000	-41.90000	1264874102
3182	Diptera	Culicidae	Aedes	Aedes aegypti	SPECIES	-15.02000	-42.94000	1264874097
3183	Diptera	Culicidae	Aedes	Aedes aegypti	SPECIES	-15.02000	-58.99000	1264874103
3184	Diptera	Culicidae	Aedes	Aedes aegypti	SPECIES	-15.01000	-41.49000	1264874104
3185	Diptera	Culicidae	Aedes	Aedes aegypti	SPECIES	-15.01000	-48.35000	1264874117
3186	Diptera	Culicidae	Aedes	Aedes aegypti	SPECIES	-15.01000	-40.33000	1264874121
3187	Diptera	Culicidae	Aedes	Aedes aegypti	SPECIES	-15.00000	-41.71000	1264874085
3188	Diptera	Culicidae	Aedes	Aedes aegypti	SPECIES	-15.00000	-39.30000	1264874095
3189	Diptera	Culicidae	Aedes	Aedes aegypti	SPECIES	-15.00000	-58.01000	1264874124
3190	Diptera	Culicidae	Aedes	Aedes aegypti	SPECIES	-15.00000	-140.00000	1633663748
3191	Diptera	Culicidae	Aedes	Aedes aegypti	SPECIES	-14.99000	-47.05000	1264874081
3192	Diptera	Culicidae	Aedes	Aedes aegypti	SPECIES	-14.98000	-43.26000	1264874096
3193	Diptera	Culicidae	Aedes	Aedes aegypti	SPECIES	-14.96000	-42.37000	1264874106
3194	Diptera	Culicidae	Aedes	Aedes aegypti	SPECIES	-14.95000	-41.23000	1264874087
3195	Diptera	Culicidae	Aedes	Aedes aegypti	SPECIES	-14.95000	-39.53000	1264874108
3196	Diptera	Culicidae	Aedes	Aedes aegypti	SPECIES	-14.93000	-42.06000	1264874109
3197	Diptera	Culicidae	Aedes	Aedes aegypti	SPECIES	-14.93000	-58.49000	1264874111
3198	Diptera	Culicidae	Aedes	Aedes aegypti	SPECIES	-14.93000	-147.71000	1264890140

№	Order	Family	Genus	Species	Taxon Rank	Latitude	Longitude	GBIF_ID
3199	Diptera	Culicidae	Aedes	Aedes aegypti	SPECIES	-14.92000	-49.63000	1264874119
3200	Diptera	Culicidae	Aedes	Aedes aegypti	SPECIES	-14.92000	-39.92000	1264874125
3201	Diptera	Culicidae	Aedes	Aedes aegypti	SPECIES	-14.91000	-49.26000	1264874098
3202	Diptera	Culicidae	Aedes	Aedes aegypti	SPECIES	-14.90000	-48.90000	1264874105
3203	Diptera	Culicidae	Aedes	Aedes aegypti	SPECIES	-14.90000	-43.00000	1264874123
3204	Diptera	Culicidae	Aedes	Aedes aegypti	SPECIES	-14.89000	-47.79000	1264874133
3205	Diptera	Culicidae	Aedes	Aedes aegypti	SPECIES	-14.88000	-40.57000	1264874130
3206	Diptera	Culicidae	Aedes	Aedes aegypti	SPECIES	-14.88000	-44.24000	1264874132
3207	Diptera	Culicidae	Aedes	Aedes aegypti	SPECIES	-14.88000	-39.84000	1264874142
3208	Diptera	Culicidae	Aedes	Aedes aegypti	SPECIES	-14.87000	-49.05000	1264874150
3209	Diptera	Culicidae	Aedes	Aedes aegypti	SPECIES	-14.87000	-64.97000	1264887254
3210	Diptera	Culicidae	Aedes	Aedes aegypti	SPECIES	-14.86000	-39.36000	1264874131
3211	Diptera	Culicidae	Aedes	Aedes aegypti	SPECIES	-14.86000	-56.25000	1264874146
3212	Diptera	Culicidae	Aedes	Aedes aegypti	SPECIES	-14.85000	-40.19000	1264874122
3213	Diptera	Culicidae	Aedes	Aedes aegypti	SPECIES	-14.85000	-39.57000	1264874127
3214	Diptera	Culicidae	Aedes	Aedes aegypti	SPECIES	-14.85000	-39.33000	1264890992
3215	Diptera	Culicidae	Aedes	Aedes aegypti	SPECIES	-14.84000	-43.76000	1264874129
3216	Diptera	Culicidae	Aedes	Aedes aegypti	SPECIES	-14.83000	-50.59000	1264874110
3217	Diptera	Culicidae	Aedes	Aedes aegypti	SPECIES	-14.83000	-53.75000	1264874118
3218	Diptera	Culicidae	Aedes	Aedes aegypti	SPECIES	-14.83000	-39.72000	1264874128
3219	Diptera	Culicidae	Aedes	Aedes aegypti	SPECIES	-14.83000	-64.90000	1264887263
3220	Diptera	Culicidae	Aedes	Aedes aegypti	SPECIES	-14.80000	-39.26000	1264890762
3221	Diptera	Culicidae	Aedes	Aedes aegypti	SPECIES	-14.79000	-64.97000	1264887259
3222	Diptera	Culicidae	Aedes	Aedes aegypti	SPECIES	-14.79000	-39.28000	1264890708
3223	Diptera	Culicidae	Aedes	Aedes aegypti	SPECIES	-14.78000	-42.34000	1264874126
3224	Diptera	Culicidae	Aedes	Aedes aegypti	SPECIES	-14.77000	-56.69000	1264874112
3225	Diptera	Culicidae	Aedes	Aedes aegypti	SPECIES	-14.77000	-39.15000	1264874120
3226	Diptera	Culicidae	Aedes	Aedes aegypti	SPECIES	-14.76000	-57.41000	1264874114
3227	Diptera	Culicidae	Aedes	Aedes aegypti	SPECIES	-14.75000	-39.43000	1264874115
3228	Diptera	Culicidae	Aedes	Aedes aegypti	SPECIES	-14.75000	-55.06000	1264874140
3229	Diptera	Culicidae	Aedes	Aedes aegypti	SPECIES	-14.74000	-49.35000	1264874116
3230	Diptera	Culicidae	Aedes	Aedes aegypti	SPECIES	-14.74000	-39.20000	1264891023
3231	Diptera	Culicidae	Aedes	Aedes aegypti	SPECIES	-14.73333	134.73334	1633667687
3232	Diptera	Culicidae	Aedes	Aedes aegypti	SPECIES	-14.73333	134.73334	177194575
3233	Diptera	Culicidae	Aedes	Aedes aegypti	SPECIES	-14.73000	-42.71000	1264874143
3234	Diptera	Culicidae	Aedes	Aedes aegypti	SPECIES	-14.72000	-56.94000	1264874113
3235	Diptera	Culicidae	Aedes	Aedes aegypti	SPECIES	-14.72000	-50.96000	1264874141
3236	Diptera	Culicidae	Aedes	Aedes aegypti	SPECIES	-14.71000	-40.48000	1264874148
3237	Diptera	Culicidae	Aedes	Aedes aegypti	SPECIES	-14.71000	-39.66000	1264874156
3238	Diptera	Culicidae	Aedes	Aedes aegypti	SPECIES	-14.70000	-46.25000	1264874137
3239	Diptera	Culicidae	Aedes	Aedes aegypti	SPECIES	-14.70000	-49.73000	1264874144

№	Order	Family	Genus	Species	Taxon Rank	Latitude	Longitude	GBIF_ID
3240	Diptera	Culicidae	Aedes	Aedes aegypti	SPECIES	-14.69000	-41.73000	1264874134
3241	Diptera	Culicidae	Aedes	Aedes aegypti	SPECIES	-14.68000	-52.33000	1264874135
3242	Diptera	Culicidae	Aedes	Aedes aegypti	SPECIES	-14.68000	-41.52000	1264874166
3243	Diptera	Culicidae	Aedes	Aedes aegypti	SPECIES	-14.68000	-39.41000	1264874170
3244	Diptera	Culicidae	Aedes	Aedes aegypti	SPECIES	-14.67000	-39.57000	1264874147
3245	Diptera	Culicidae	Aedes	Aedes aegypti	SPECIES	-14.67000	-44.11000	1264874153
3246	Diptera	Culicidae	Aedes	Aedes aegypti	SPECIES	-14.66000	-39.87000	1264874149
3247	Diptera	Culicidae	Aedes	Aedes aegypti	SPECIES	-14.65000	-40.05000	1264874157
3248	Diptera	Culicidae	Aedes	Aedes aegypti	SPECIES	-14.63000	-41.27000	1264874138
3249	Diptera	Culicidae	Aedes	Aedes aegypti	SPECIES	-14.63000	-50.07000	1264874155
3250	Diptera	Culicidae	Aedes	Aedes aegypti	SPECIES	-14.62000	-59.34000	1264874145
3251	Diptera	Culicidae	Aedes	Aedes aegypti	SPECIES	-14.62000	-42.46000	1264874159
3252	Diptera	Culicidae	Aedes	Aedes aegypti	SPECIES	-14.61000	-40.92000	1264874175
3253	Diptera	Culicidae	Aedes	Aedes aegypti	SPECIES	-14.59000	-46.86000	1264874151
3254	Diptera	Culicidae	Aedes	Aedes aegypti	SPECIES	-14.58000	-41.98000	1264874160
3255	Diptera	Culicidae	Aedes	Aedes aegypti	SPECIES	-14.58000	-40.41000	1264874172
3256	Diptera	Culicidae	Aedes	Aedes aegypti	SPECIES	-14.57000	-46.63000	1264874158
3257	Diptera	Culicidae	Aedes	Aedes aegypti	SPECIES	-14.57000	-43.18000	1264874163
3258	Diptera	Culicidae	Aedes	Aedes aegypti	SPECIES	-14.56000	-46.18000	1264874179
3259	Diptera	Culicidae	Aedes	Aedes aegypti	SPECIES	-14.54000	-45.23000	1264874136
3260	Diptera	Culicidae	Aedes	Aedes aegypti	SPECIES	-14.54000	-43.55000	1264874139
3261	Diptera	Culicidae	Aedes	Aedes aegypti	SPECIES	-14.54000	-54.64000	1264874154
3262	Diptera	Culicidae	Aedes	Aedes aegypti	SPECIES	-14.54000	-49.48000	1264874173
3263	Diptera	Culicidae	Aedes	Aedes aegypti	SPECIES	-14.52000	-39.19000	1264874169
3264	Diptera	Culicidae	Aedes	Aedes aegypti	SPECIES	-14.50000	-56.85000	1264874165
3265	Diptera	Culicidae	Aedes	Aedes aegypti	SPECIES	-14.49000	-42.34000	1264874152
3266	Diptera	Culicidae	Aedes	Aedes aegypti	SPECIES	-14.48000	-48.41000	1264874161
3267	Diptera	Culicidae	Aedes	Aedes aegypti	SPECIES	-14.47000	-39.61000	1264874162
3268	Diptera	Culicidae	Aedes	Aedes aegypti	SPECIES	-14.47000	-44.49000	1264874167
3269	Diptera	Culicidae	Aedes	Aedes aegypti	SPECIES	-14.47000	-42.68000	1264874174
3270	Diptera	Culicidae	Aedes	Aedes aegypti	SPECIES	-14.44000	-57.32000	1264874171
3271	Diptera	Culicidae	Aedes	Aedes aegypti	SPECIES	-14.44000	-46.58000	1264874180
3272	Diptera	Culicidae	Aedes	Aedes aegypti	SPECIES	-14.43000	-58.13000	1264874164
3273	Diptera	Culicidae	Aedes	Aedes aegypti	SPECIES	-14.43000	-47.42000	1264874177
3274	Diptera	Culicidae	Aedes	Aedes aegypti	SPECIES	-14.43000	-42.84000	1264874184
3275	Diptera	Culicidae	Aedes	Aedes aegypti	SPECIES	-14.43000	-46.05000	1264874192
3276	Diptera	Culicidae	Aedes	Aedes aegypti	SPECIES	-14.40000	-43.95000	1264874178
3277	Diptera	Culicidae	Aedes	Aedes aegypti	SPECIES	-14.40000	-59.81000	1264874196
3278	Diptera	Culicidae	Aedes	Aedes aegypti	SPECIES	-14.39000	-46.31000	1264874168
3279	Diptera	Culicidae	Aedes	Aedes aegypti	SPECIES	-14.39000	-55.84000	1264874181
3280	Diptera	Culicidae	Aedes	Aedes aegypti	SPECIES	-14.39000	-56.73000	1264874183

№	Order	Family	Genus	Species	Taxon Rank	Latitude	Longitude	GBIF_ID
3281	Diptera	Culicidae	Aedes	Aedes aegypti	SPECIES	-14.38000	-40.54000	1264874176
3282	Diptera	Culicidae	Aedes	Aedes aegypti	SPECIES	-14.38000	-39.96000	1264874193
3283	Diptera	Culicidae	Aedes	Aedes aegypti	SPECIES	-14.37000	-40.27000	1264874188
3284	Diptera	Culicidae	Aedes	Aedes aegypti	SPECIES	-14.36000	-39.49000	1264874186
3285	Diptera	Culicidae	Aedes	Aedes aegypti	SPECIES	-14.35000	-39.16000	1264874182
3286	Diptera	Culicidae	Aedes	Aedes aegypti	SPECIES	-14.35000	-41.85000	1264874213
3287	Diptera	Culicidae	Aedes	Aedes aegypti	SPECIES	-14.33000	-49.08000	1264874197
3288	Diptera	Culicidae	Aedes	Aedes aegypti	SPECIES	-14.33000	-41.35000	1264874202
3289	Diptera	Culicidae	Aedes	Aedes aegypti	SPECIES	-14.33000	-170.73000	1264890068
3290	Diptera	Culicidae	Aedes	Aedes aegypti	SPECIES	-14.33000	-170.84000	1264890079
3291	Diptera	Culicidae	Aedes	Aedes aegypti	SPECIES	-14.32000	-42.08000	1264874199
3292	Diptera	Culicidae	Aedes	Aedes aegypti	SPECIES	-14.32000	-170.70000	1264890071
3293	Diptera	Culicidae	Aedes	Aedes aegypti	SPECIES	-14.31000	-40.90000	1264874185
3294	Diptera	Culicidae	Aedes	Aedes aegypti	SPECIES	-14.31000	-49.58000	1264874189
3295	Diptera	Culicidae	Aedes	Aedes aegypti	SPECIES	-14.31000	-49.29000	1264874194
3296	Diptera	Culicidae	Aedes	Aedes aegypti	SPECIES	-14.30000	-57.30000	1264874195
3297	Diptera	Culicidae	Aedes	Aedes aegypti	SPECIES	-14.29000	-39.57000	1264874204
3298	Diptera	Culicidae	Aedes	Aedes aegypti	SPECIES	-14.29000	-170.66000	1264890080
3299	Diptera	Culicidae	Aedes	Aedes aegypti	SPECIES	-14.28000	-170.72000	1264888162
3300	Diptera	Culicidae	Aedes	Aedes aegypti	SPECIES	-14.28000	-170.69000	1264890091
3301	Diptera	Culicidae	Aedes	Aedes aegypti	SPECIES	-14.28000	-170.70000	1264890107
3302	Diptera	Culicidae	Aedes	Aedes aegypti	SPECIES	-14.27000	-42.31000	1264874187
3303	Diptera	Culicidae	Aedes	Aedes aegypti	SPECIES	-14.27000	-170.58000	1264888175
3304	Diptera	Culicidae	Aedes	Aedes aegypti	SPECIES	-14.27000	-170.62000	1264890118
3305	Diptera	Culicidae	Aedes	Aedes aegypti	SPECIES	-14.26000	-39.42000	1264874201
3306	Diptera	Culicidae	Aedes	Aedes aegypti	SPECIES	-14.26000	-53.08000	1264874216
3307	Diptera	Culicidae	Aedes	Aedes aegypti	SPECIES	-14.26000	-170.59000	1264890074
3308	Diptera	Culicidae	Aedes	Aedes aegypti	SPECIES	-14.24000	-39.82000	1264874206
3309	Diptera	Culicidae	Aedes	Aedes aegypti	SPECIES	-14.22000	-46.43000	1264874200
3310	Diptera	Culicidae	Aedes	Aedes aegypti	SPECIES	-14.21000	-41.70000	1264874191
3311	Diptera	Culicidae	Aedes	Aedes aegypti	SPECIES	-14.20000	-43.21000	1264874190
3312	Diptera	Culicidae	Aedes	Aedes aegypti	SPECIES	-14.20000	-49.66000	1264874198
3313	Diptera	Culicidae	Aedes	Aedes aegypti	SPECIES	-14.20000	49.92000	1264888575
3314	Diptera	Culicidae	Aedes	Aedes aegypti	SPECIES	-14.18000	-42.83000	1264874208
3315	Diptera	Culicidae	Aedes	Aedes aegypti	SPECIES	-14.18000	-49.43000	1264874221
3316	Diptera	Culicidae	Aedes	Aedes aegypti	SPECIES	-14.17000	-40.02000	1264874210
3317	Diptera	Culicidae	Aedes	Aedes aegypti	SPECIES	-14.16000	-47.52000	1264874211
3318	Diptera	Culicidae	Aedes	Aedes aegypti	SPECIES	-14.16000	-40.71000	1264874217
3319	Diptera	Culicidae	Aedes	Aedes aegypti	SPECIES	-14.15000	-51.80000	1264874212
3320	Diptera	Culicidae	Aedes	Aedes aegypti	SPECIES	-14.14000	-56.85000	1264874203
3321	Diptera	Culicidae	Aedes	Aedes aegypti	SPECIES	-14.14000	-49.97000	1264874235

№	Order	Family	Genus	Species	Taxon Rank	Latitude	Longitude	GBIF_ID
3322	Diptera	Culicidae	Aedes	Aedes aegypti	SPECIES	-14.13000	-41.20000	1264874228
3323	Diptera	Culicidae	Aedes	Aedes aegypti	SPECIES	-14.12000	-43.65000	1264874214
3324	Diptera	Culicidae	Aedes	Aedes aegypti	SPECIES	-14.11000	-45.25000	1264874222
3325	Diptera	Culicidae	Aedes	Aedes aegypti	SPECIES	-14.10000	-39.87000	1264874205
3326	Diptera	Culicidae	Aedes	Aedes aegypti	SPECIES	-14.09000	-46.71000	1264874207
3327	Diptera	Culicidae	Aedes	Aedes aegypti	SPECIES	-14.09000	-52.47000	1264874225
3328	Diptera	Culicidae	Aedes	Aedes aegypti	SPECIES	-14.08000	-38.97000	1264874218
3329	Diptera	Culicidae	Aedes	Aedes aegypti	SPECIES	-14.08000	-40.58000	1264874231
3330	Diptera	Culicidae	Aedes	Aedes aegypti	SPECIES	-14.06000	-39.52000	1264874209
3331	Diptera	Culicidae	Aedes	Aedes aegypti	SPECIES	-14.06000	-39.42000	1264874224
3332	Diptera	Culicidae	Aedes	Aedes aegypti	SPECIES	-14.05000	-39.60000	1264874227
3333	Diptera	Culicidae	Aedes	Aedes aegypti	SPECIES	-14.04000	-39.74000	1264874246
3334	Diptera	Culicidae	Aedes	Aedes aegypti	SPECIES	-14.03000	-49.36000	1264874219
3335	Diptera	Culicidae	Aedes	Aedes aegypti	SPECIES	-14.03000	-42.18000	1264874220
3336	Diptera	Culicidae	Aedes	Aedes aegypti	SPECIES	-14.03000	-50.51000	1264874223
3337	Diptera	Culicidae	Aedes	Aedes aegypti	SPECIES	-14.01000	-48.93000	1264874215
3338	Diptera	Culicidae	Aedes	Aedes aegypti	SPECIES	-14.00000	-43.84000	1264874232
3339	Diptera	Culicidae	Aedes	Aedes aegypti	SPECIES	-14.00000	-42.48000	1264874236
3340	Diptera	Culicidae	Aedes	Aedes aegypti	SPECIES	-14.00000	-39.64000	1264874238
3341	Diptera	Culicidae	Aedes	Aedes aegypti	SPECIES	-14.00000	-39.18000	1264874243
3342	Diptera	Culicidae	Aedes	Aedes aegypti	SPECIES	-14.00000	-171.82000	1264890076
3343	Diptera	Culicidae	Aedes	Aedes aegypti	SPECIES	-13.99000	-48.09000	1264874237
3344	Diptera	Culicidae	Aedes	Aedes aegypti	SPECIES	-13.98000	-44.23000	1264874226
3345	Diptera	Culicidae	Aedes	Aedes aegypti	SPECIES	-13.98000	-39.86000	1264874234
3346	Diptera	Culicidae	Aedes	Aedes aegypti	SPECIES	-13.97000	136.45000	1264886383
3347	Diptera	Culicidae	Aedes	Aedes aegypti	SPECIES	-13.95000	-40.13000	1264874239
3348	Diptera	Culicidae	Aedes	Aedes aegypti	SPECIES	-13.91000	-171.73000	1264890043
3349	Diptera	Culicidae	Aedes	Aedes aegypti	SPECIES	-13.90000	-171.57000	1264889868
3350	Diptera	Culicidae	Aedes	Aedes aegypti	SPECIES	-13.89000	-48.55000	1264874247
3351	Diptera	Culicidae	Aedes	Aedes aegypti	SPECIES	-13.88000	-46.48000	1264874252
3352	Diptera	Culicidae	Aedes	Aedes aegypti	SPECIES	-13.88000	-171.63000	1264889888
3353	Diptera	Culicidae	Aedes	Aedes aegypti	SPECIES	-13.87000	-40.16000	1264890978
3354	Diptera	Culicidae	Aedes	Aedes aegypti	SPECIES	-13.86000	-41.10000	1264874229
3355	Diptera	Culicidae	Aedes	Aedes aegypti	SPECIES	-13.86000	-41.41000	1264874241
3356	Diptera	Culicidae	Aedes	Aedes aegypti	SPECIES	-13.86000	-42.96000	1264874261
3357	Diptera	Culicidae	Aedes	Aedes aegypti	SPECIES	-13.84000	-49.60000	1264874233
3358	Diptera	Culicidae	Aedes	Aedes aegypti	SPECIES	-13.84000	-51.12000	1264874242
3359	Diptera	Culicidae	Aedes	Aedes aegypti	SPECIES	-13.84000	-39.18000	1264874248
3360	Diptera	Culicidae	Aedes	Aedes aegypti	SPECIES	-13.84000	-172.10000	1264890027
3361	Diptera	Culicidae	Aedes	Aedes aegypti	SPECIES	-13.84000	-172.08000	1264890047
3362	Diptera	Culicidae	Aedes	Aedes aegypti	SPECIES	-13.83333	-171.73331	177195349

№	Order	Family	Genus	Species	Taxon Rank	Latitude	Longitude	GBIF_ID
3363	Diptera	Culicidae	Aedes	Aedes aegypti	SPECIES	-13.83333	-171.73331	1633667720
3364	Diptera	Culicidae	Aedes	Aedes aegypti	SPECIES	-13.83000	-42.73000	1264874245
3365	Diptera	Culicidae	Aedes	Aedes aegypti	SPECIES	-13.83000	-41.72000	1264874255
3366	Diptera	Culicidae	Aedes	Aedes aegypti	SPECIES	-13.83000	-171.98000	1264889897
3367	Diptera	Culicidae	Aedes	Aedes aegypti	SPECIES	-13.83000	-171.75000	1264890090
3368	Diptera	Culicidae	Aedes	Aedes aegypti	SPECIES	-13.83000	-65.25000	1264890950
3369	Diptera	Culicidae	Aedes	Aedes aegypti	SPECIES	-13.81000	-49.08000	1264874230
3370	Diptera	Culicidae	Aedes	Aedes aegypti	SPECIES	-13.81000	-39.58000	1264874244
3371	Diptera	Culicidae	Aedes	Aedes aegypti	SPECIES	-13.81000	-39.76000	1264874249
3372	Diptera	Culicidae	Aedes	Aedes aegypti	SPECIES	-13.80000	-39.36000	1264874250
3373	Diptera	Culicidae	Aedes	Aedes aegypti	SPECIES	-13.80000	-39.47000	1264874263
3374	Diptera	Culicidae	Aedes	Aedes aegypti	SPECIES	-13.80000	-42.05000	1264874267
3375	Diptera	Culicidae	Aedes	Aedes aegypti	SPECIES	-13.78000	-50.20000	1264874251
3376	Diptera	Culicidae	Aedes	Aedes aegypti	SPECIES	-13.78000	-55.32000	1264874260
3377	Diptera	Culicidae	Aedes	Aedes aegypti	SPECIES	-13.78000	-39.65000	1264874269
3378	Diptera	Culicidae	Aedes	Aedes aegypti	SPECIES	-13.77000	-46.99000	1264874259
3379	Diptera	Culicidae	Aedes	Aedes aegypti	SPECIES	-13.75548	-177.08021	4050498043
3380	Diptera	Culicidae	Aedes	Aedes aegypti	SPECIES	-13.75000	-39.05000	1264874253
3381	Diptera	Culicidae	Aedes	Aedes aegypti	SPECIES	-13.74000	-44.44000	1264874264
3382	Diptera	Culicidae	Aedes	Aedes aegypti	SPECIES	-13.72000	-172.21000	1264890063
3383	Diptera	Culicidae	Aedes	Aedes aegypti	SPECIES	-13.71000	-48.86000	1264874240
3384	Diptera	Culicidae	Aedes	Aedes aegypti	SPECIES	-13.71000	-49.33000	1264874258
3385	Diptera	Culicidae	Aedes	Aedes aegypti	SPECIES	-13.69000	-47.24000	1264874262
3386	Diptera	Culicidae	Aedes	Aedes aegypti	SPECIES	-13.68000	-40.96000	1264874278
3387	Diptera	Culicidae	Aedes	Aedes aegypti	SPECIES	-13.68000	-172.19000	1264890092
3388	Diptera	Culicidae	Aedes	Aedes aegypti	SPECIES	-13.66000	-57.88000	1264874271
3389	Diptera	Culicidae	Aedes	Aedes aegypti	SPECIES	-13.66000	-43.11000	1264874272
3390	Diptera	Culicidae	Aedes	Aedes aegypti	SPECIES	-13.66000	-172.43000	1264890104
3391	Diptera	Culicidae	Aedes	Aedes aegypti	SPECIES	-13.65000	-39.60000	1264874270
3392	Diptera	Culicidae	Aedes	Aedes aegypti	SPECIES	-13.65000	-39.09000	1264874287
3393	Diptera	Culicidae	Aedes	Aedes aegypti	SPECIES	-13.64000	-47.72000	1264874254
3394	Diptera	Culicidae	Aedes	Aedes aegypti	SPECIES	-13.63000	-41.73000	1264874257
3395	Diptera	Culicidae	Aedes	Aedes aegypti	SPECIES	-13.63000	-172.62000	1264889889
3396	Diptera	Culicidae	Aedes	Aedes aegypti	SPECIES	-13.58000	-59.18000	1264874256
3397	Diptera	Culicidae	Aedes	Aedes aegypti	SPECIES	-13.58000	-39.22000	1264874265
3398	Diptera	Culicidae	Aedes	Aedes aegypti	SPECIES	-13.58000	-40.33000	1264874277
3399	Diptera	Culicidae	Aedes	Aedes aegypti	SPECIES	-13.57000	-39.52000	1264874275
3400	Diptera	Culicidae	Aedes	Aedes aegypti	SPECIES	-13.56000	-48.98000	1264874273
3401	Diptera	Culicidae	Aedes	Aedes aegypti	SPECIES	-13.55000	-42.54000	1264874274
3402	Diptera	Culicidae	Aedes	Aedes aegypti	SPECIES	-13.54000	-49.91000	1264874268
3403	Diptera	Culicidae	Aedes	Aedes aegypti	SPECIES	-13.54000	-40.53000	1264874288

№	Order	Family	Genus	Species	Taxon Rank	Latitude	Longitude	GBIF_ID
3404	Diptera	Culicidae	Aedes	Aedes aegypti	SPECIES	-13.54000	-40.95000	1264874292
3405	Diptera	Culicidae	Aedes	Aedes aegypti	SPECIES	-13.53000	-39.93000	1264874284
3406	Diptera	Culicidae	Aedes	Aedes aegypti	SPECIES	-13.52000	-56.08000	1264874276
3407	Diptera	Culicidae	Aedes	Aedes aegypti	SPECIES	-13.52000	-38.99000	1264874289
3408	Diptera	Culicidae	Aedes	Aedes aegypti	SPECIES	-13.51000	-60.65000	1264874293
3409	Diptera	Culicidae	Aedes	Aedes aegypti	SPECIES	-13.50000	-41.59000	1264874266
3410	Diptera	Culicidae	Aedes	Aedes aegypti	SPECIES	-13.50000	-40.15000	1264874279
3411	Diptera	Culicidae	Aedes	Aedes aegypti	SPECIES	-13.50000	-44.04000	1264874283
3412	Diptera	Culicidae	Aedes	Aedes aegypti	SPECIES	-13.50000	-48.40000	1264874301
3413	Diptera	Culicidae	Aedes	Aedes aegypti	SPECIES	-13.49000	-42.24000	1264874285
3414	Diptera	Culicidae	Aedes	Aedes aegypti	SPECIES	-13.49000	-45.31000	1264874286
3415	Diptera	Culicidae	Aedes	Aedes aegypti	SPECIES	-13.49000	-40.34000	1264874296
3416	Diptera	Culicidae	Aedes	Aedes aegypti	SPECIES	-13.48000	-43.71000	1264874312
3417	Diptera	Culicidae	Aedes	Aedes aegypti	SPECIES	-13.46667	131.39999	177194572
3418	Diptera	Culicidae	Aedes	Aedes aegypti	SPECIES	-13.46667	131.39999	1633667682
3419	Diptera	Culicidae	Aedes	Aedes aegypti	SPECIES	-13.46000	-39.44000	1264874280
3420	Diptera	Culicidae	Aedes	Aedes aegypti	SPECIES	-13.45000	-46.45000	1264874313
3421	Diptera	Culicidae	Aedes	Aedes aegypti	SPECIES	-13.44000	-39.89000	1264874281
3422	Diptera	Culicidae	Aedes	Aedes aegypti	SPECIES	-13.44000	-56.86000	1264874295
3423	Diptera	Culicidae	Aedes	Aedes aegypti	SPECIES	-13.44000	-42.00000	1264890795
3424	Diptera	Culicidae	Aedes	Aedes aegypti	SPECIES	-13.44000	-64.51000	1264891287
3425	Diptera	Culicidae	Aedes	Aedes aegypti	SPECIES	-13.43000	-42.06000	1264874282
3426	Diptera	Culicidae	Aedes	Aedes aegypti	SPECIES	-13.43000	-48.75000	1264874290
3427	Diptera	Culicidae	Aedes	Aedes aegypti	SPECIES	-13.43000	-39.79000	1264874314
3428	Diptera	Culicidae	Aedes	Aedes aegypti	SPECIES	-13.40000	36.13000	3987357025
3429	Diptera	Culicidae	Aedes	Aedes aegypti	SPECIES	-13.38000	-41.36000	1264874298
3430	Diptera	Culicidae	Aedes	Aedes aegypti	SPECIES	-13.38000	-39.20000	1264874302
3431	Diptera	Culicidae	Aedes	Aedes aegypti	SPECIES	-13.34000	-54.16000	1264874297
3432	Diptera	Culicidae	Aedes	Aedes aegypti	SPECIES	-13.34000	-70.75000	1264887649
3433	Diptera	Culicidae	Aedes	Aedes aegypti	SPECIES	-13.33000	-39.51000	1264874307
3434	Diptera	Culicidae	Aedes	Aedes aegypti	SPECIES	-13.33000	-39.59000	1264874311
3435	Diptera	Culicidae	Aedes	Aedes aegypti	SPECIES	-13.32000	-42.54000	1264874321
3436	Diptera	Culicidae	Aedes	Aedes aegypti	SPECIES	-13.31000	-46.92000	1264874294
3437	Diptera	Culicidae	Aedes	Aedes aegypti	SPECIES	-13.30000	-39.87000	1264874300
3438	Diptera	Culicidae	Aedes	Aedes aegypti	SPECIES	-13.30000	-39.69000	1264874303
3439	Diptera	Culicidae	Aedes	Aedes aegypti	SPECIES	-13.30000	-41.75000	1264874310
3440	Diptera	Culicidae	Aedes	Aedes aegypti	SPECIES	-13.29000	-176.22000	1264891177
3441	Diptera	Culicidae	Aedes	Aedes aegypti	SPECIES	-13.28000	-49.27000	1264874322
3442	Diptera	Culicidae	Aedes	Aedes aegypti	SPECIES	-13.28000	-43.31000	1264874336
3443	Diptera	Culicidae	Aedes	Aedes aegypti	SPECIES	-13.26000	-42.68000	1264874317
3444	Diptera	Culicidae	Aedes	Aedes aegypti	SPECIES	-13.24000	-49.68000	1264874308

№	Order	Family	Genus	Species	Taxon Rank	Latitude	Longitude	GBIF_ID
3445	Diptera	Culicidae	Aedes	Aedes aegypti	SPECIES	-13.23000	-42.30000	1264874291
3446	Diptera	Culicidae	Aedes	Aedes aegypti	SPECIES	-13.23000	-40.03000	1264874309
3447	Diptera	Culicidae	Aedes	Aedes aegypti	SPECIES	-13.22000	-46.55000	1264874306
3448	Diptera	Culicidae	Aedes	Aedes aegypti	SPECIES	-13.20000	-59.78000	1264874328
3449	Diptera	Culicidae	Aedes	Aedes aegypti	SPECIES	-13.19000	-52.08000	1264874305
3450	Diptera	Culicidae	Aedes	Aedes aegypti	SPECIES	-13.19000	-44.36000	1264874315
3451	Diptera	Culicidae	Aedes	Aedes aegypti	SPECIES	-13.19000	-39.34000	1264874338
3452	Diptera	Culicidae	Aedes	Aedes aegypti	SPECIES	-13.18000	-61.31000	1264874319
3453	Diptera	Culicidae	Aedes	Aedes aegypti	SPECIES	-13.18000	-40.31000	1264874340
3454	Diptera	Culicidae	Aedes	Aedes aegypti	SPECIES	-13.16000	-40.65000	1264874304
3455	Diptera	Culicidae	Aedes	Aedes aegypti	SPECIES	-13.14000	-42.14000	1264874299
3456	Diptera	Culicidae	Aedes	Aedes aegypti	SPECIES	-13.14000	-60.54000	1264874327
3457	Diptera	Culicidae	Aedes	Aedes aegypti	SPECIES	-13.11000	-48.74000	1264874323
3458	Diptera	Culicidae	Aedes	Aedes aegypti	SPECIES	-13.10000	-44.22000	1264874318
3459	Diptera	Culicidae	Aedes	Aedes aegypti	SPECIES	-13.10000	-40.99000	1264874326
3460	Diptera	Culicidae	Aedes	Aedes aegypti	SPECIES	-13.09000	-38.88000	1264874320
3461	Diptera	Culicidae	Aedes	Aedes aegypti	SPECIES	-13.09000	-43.95000	1264874332
3462	Diptera	Culicidae	Aedes	Aedes aegypti	SPECIES	-13.09000	-39.08000	1264874345
3463	Diptera	Culicidae	Aedes	Aedes aegypti	SPECIES	-13.08000	-39.84000	1264874339
3464	Diptera	Culicidae	Aedes	Aedes aegypti	SPECIES	-13.05000	-56.27000	1264874333
3465	Diptera	Culicidae	Aedes	Aedes aegypti	SPECIES	-13.05000	-41.87000	1264874344
3466	Diptera	Culicidae	Aedes	Aedes aegypti	SPECIES	-13.05000	-41.42000	1264874353
3467	Diptera	Culicidae	Aedes	Aedes aegypti	SPECIES	-13.05000	-40.03000	1264874360
3468	Diptera	Culicidae	Aedes	Aedes aegypti	SPECIES	-13.04000	-39.39000	1264874334
3469	Diptera	Culicidae	Aedes	Aedes aegypti	SPECIES	-13.04000	-48.35000	1264874342
3470	Diptera	Culicidae	Aedes	Aedes aegypti	SPECIES	-13.03000	-38.68000	1264874316
3471	Diptera	Culicidae	Aedes	Aedes aegypti	SPECIES	-13.02000	-39.65000	1264874329
3472	Diptera	Culicidae	Aedes	Aedes aegypti	SPECIES	-13.01000	-61.74000	1264874330
3473	Diptera	Culicidae	Aedes	Aedes aegypti	SPECIES	-13.01000	-39.22000	1264874343
3474	Diptera	Culicidae	Aedes	Aedes aegypti	SPECIES	-13.01000	-38.53000	1264889478
3475	Diptera	Culicidae	Aedes	Aedes aegypti	SPECIES	-13.01000	-40.01000	1264890792
3476	Diptera	Culicidae	Aedes	Aedes aegypti	SPECIES	-13.00000	-39.09000	1264874337
3477	Diptera	Culicidae	Aedes	Aedes aegypti	SPECIES	-12.99000	-50.21000	1264874365
3478	Diptera	Culicidae	Aedes	Aedes aegypti	SPECIES	-12.99000	-38.44000	1264889512
3479	Diptera	Culicidae	Aedes	Aedes aegypti	SPECIES	-12.98000	-38.43000	1264888109
3480	Diptera	Culicidae	Aedes	Aedes aegypti	SPECIES	-12.98000	-38.51000	1264891189
3481	Diptera	Culicidae	Aedes	Aedes aegypti	SPECIES	-12.97000	-46.20000	1264874324
3482	Diptera	Culicidae	Aedes	Aedes aegypti	SPECIES	-12.97000	-54.62000	1264874325
3483	Diptera	Culicidae	Aedes	Aedes aegypti	SPECIES	-12.97000	-38.50000	1264888097
3484	Diptera	Culicidae	Aedes	Aedes aegypti	SPECIES	-12.97000	-38.51000	1264889175
3485	Diptera	Culicidae	Aedes	Aedes aegypti	SPECIES	-12.97000	45.17000	1264890631

№	Order	Family	Genus	Species	Taxon Rank	Latitude	Longitude	GBIF_ID
3486	Diptera	Culicidae	Aedes	Aedes aegypti	SPECIES	-12.96000	-58.56000	1264874335
3487	Diptera	Culicidae	Aedes	Aedes aegypti	SPECIES	-12.96000	45.17000	1264886277
3488	Diptera	Culicidae	Aedes	Aedes aegypti	SPECIES	-12.96000	-38.50000	1264889493
3489	Diptera	Culicidae	Aedes	Aedes aegypti	SPECIES	-12.96000	-38.48000	1264889509
3490	Diptera	Culicidae	Aedes	Aedes aegypti	SPECIES	-12.95097	-38.38765	1831158750
3491	Diptera	Culicidae	Aedes	Aedes aegypti	SPECIES	-12.95095	-38.38769	1831158789
3492	Diptera	Culicidae	Aedes	Aedes aegypti	SPECIES	-12.95085	-38.38769	1913721970
3493	Diptera	Culicidae	Aedes	Aedes aegypti	SPECIES	-12.95000	-38.99000	1264874346
3494	Diptera	Culicidae	Aedes	Aedes aegypti	SPECIES	-12.95000	-39.38000	1264874368
3495	Diptera	Culicidae	Aedes	Aedes aegypti	SPECIES	-12.95000	-38.50000	1264889500
3496	Diptera	Culicidae	Aedes	Aedes aegypti	SPECIES	-12.94000	-39.79000	1264874366
3497	Diptera	Culicidae	Aedes	Aedes aegypti	SPECIES	-12.93000	-39.57000	1264874348
3498	Diptera	Culicidae	Aedes	Aedes aegypti	SPECIES	-12.93000	-38.50000	1264888099
3499	Diptera	Culicidae	Aedes	Aedes aegypti	SPECIES	-12.92000	-38.49000	1264889471
3500	Diptera	Culicidae	Aedes	Aedes aegypti	SPECIES	-12.91000	-39.17000	1264874351
3501	Diptera	Culicidae	Aedes	Aedes aegypti	SPECIES	-12.91000	-43.49000	1264874356
3502	Diptera	Culicidae	Aedes	Aedes aegypti	SPECIES	-12.91000	49.13000	1264886087
3503	Diptera	Culicidae	Aedes	Aedes aegypti	SPECIES	-12.91000	45.18000	1264890642
3504	Diptera	Culicidae	Aedes	Aedes aegypti	SPECIES	-12.90000	-38.44000	1264891053
3505	Diptera	Culicidae	Aedes	Aedes aegypti	SPECIES	-12.89000	-46.58000	1264874361
3506	Diptera	Culicidae	Aedes	Aedes aegypti	SPECIES	-12.88000	-51.53000	1264874331
3507	Diptera	Culicidae	Aedes	Aedes aegypti	SPECIES	-12.88000	-41.30000	1264874347
3508	Diptera	Culicidae	Aedes	Aedes aegypti	SPECIES	-12.88000	-45.44000	1264874350
3509	Diptera	Culicidae	Aedes	Aedes aegypti	SPECIES	-12.88000	-41.70000	1264874359
3510	Diptera	Culicidae	Aedes	Aedes aegypti	SPECIES	-12.88000	-38.76000	1264874362
3511	Diptera	Culicidae	Aedes	Aedes aegypti	SPECIES	-12.88000	-61.08000	1264874384
3512	Diptera	Culicidae	Aedes	Aedes aegypti	SPECIES	-12.87000	-53.45000	1264874352
3513	Diptera	Culicidae	Aedes	Aedes aegypti	SPECIES	-12.87000	-42.35000	1264874370
3514	Diptera	Culicidae	Aedes	Aedes aegypti	SPECIES	-12.87000	-42.14000	1264874374
3515	Diptera	Culicidae	Aedes	Aedes aegypti	SPECIES	-12.87000	-38.47000	1264889445
3516	Diptera	Culicidae	Aedes	Aedes aegypti	SPECIES	-12.86000	-39.23000	1264874341
3517	Diptera	Culicidae	Aedes	Aedes aegypti	SPECIES	-12.86000	-38.33000	1264874357
3518	Diptera	Culicidae	Aedes	Aedes aegypti	SPECIES	-12.86000	-48.66000	1264874358
3519	Diptera	Culicidae	Aedes	Aedes aegypti	SPECIES	-12.86000	-72.69000	1264887616
3520	Diptera	Culicidae	Aedes	Aedes aegypti	SPECIES	-12.85000	-41.14000	1264874369
3521	Diptera	Culicidae	Aedes	Aedes aegypti	SPECIES	-12.85000	-39.09000	1264874385
3522	Diptera	Culicidae	Aedes	Aedes aegypti	SPECIES	-12.85000	45.11000	1264886078
3523	Diptera	Culicidae	Aedes	Aedes aegypti	SPECIES	-12.84000	-49.77000	1264874355
3524	Diptera	Culicidae	Aedes	Aedes aegypti	SPECIES	-12.84000	45.12000	1264886065
3525	Diptera	Culicidae	Aedes	Aedes aegypti	SPECIES	-12.84000	-38.46000	1264889466
3526	Diptera	Culicidae	Aedes	Aedes aegypti	SPECIES	-12.83000	-38.94000	1264874349

№	Order	Family	Genus	Species	Taxon Rank	Latitude	Longitude	GBIF_ID
3527	Diptera	Culicidae	Aedes	Aedes aegypti	SPECIES	-12.83000	-46.51000	1264874378
3528	Diptera	Culicidae	Aedes	Aedes aegypti	SPECIES	-12.83000	45.17000	1264886959
3529	Diptera	Culicidae	Aedes	Aedes aegypti	SPECIES	-12.83000	45.11000	1264890622
3530	Diptera	Culicidae	Aedes	Aedes aegypti	SPECIES	-12.82060	45.14743	4050442541
3531	Diptera	Culicidae	Aedes	Aedes aegypti	SPECIES	-12.82000	-46.37000	1264874364
3532	Diptera	Culicidae	Aedes	Aedes aegypti	SPECIES	-12.82000	-47.08000	1264874382
3533	Diptera	Culicidae	Aedes	Aedes aegypti	SPECIES	-12.81000	-43.86000	1264874354
3534	Diptera	Culicidae	Aedes	Aedes aegypti	SPECIES	-12.80000	-40.14000	1264874367
3535	Diptera	Culicidae	Aedes	Aedes aegypti	SPECIES	-12.79000	45.29000	1264886085
3536	Diptera	Culicidae	Aedes	Aedes aegypti	SPECIES	-12.79000	45.27000	1264890648
3537	Diptera	Culicidae	Aedes	Aedes aegypti	SPECIES	-12.78000	45.21000	1264886046
3538	Diptera	Culicidae	Aedes	Aedes aegypti	SPECIES	-12.78000	45.22000	1264890626
3539	Diptera	Culicidae	Aedes	Aedes aegypti	SPECIES	-12.77000	-38.40000	1264874377
3540	Diptera	Culicidae	Aedes	Aedes aegypti	SPECIES	-12.76000	-57.24000	1264874392
3541	Diptera	Culicidae	Aedes	Aedes aegypti	SPECIES	-12.75000	-43.09000	1264874375
3542	Diptera	Culicidae	Aedes	Aedes aegypti	SPECIES	-12.75000	-47.76000	1264874391
3543	Diptera	Culicidae	Aedes	Aedes aegypti	SPECIES	-12.74000	-40.69000	1264874371
3544	Diptera	Culicidae	Aedes	Aedes aegypti	SPECIES	-12.74000	-60.15000	1264889549
3545	Diptera	Culicidae	Aedes	Aedes aegypti	SPECIES	-12.73000	-39.21000	1264874372
3546	Diptera	Culicidae	Aedes	Aedes aegypti	SPECIES	-12.73000	-42.72000	1264874376
3547	Diptera	Culicidae	Aedes	Aedes aegypti	SPECIES	-12.72000	-39.56000	1264874383
3548	Diptera	Culicidae	Aedes	Aedes aegypti	SPECIES	-12.72000	-39.75000	1264874390
3549	Diptera	Culicidae	Aedes	Aedes aegypti	SPECIES	-12.72000	-39.37000	1264874393
3550	Diptera	Culicidae	Aedes	Aedes aegypti	SPECIES	-12.72000	45.11000	1264886308
3551	Diptera	Culicidae	Aedes	Aedes aegypti	SPECIES	-12.72000	45.12000	1264890634
3552	Diptera	Culicidae	Aedes	Aedes aegypti	SPECIES	-12.71000	-60.11000	1264890452
3553	Diptera	Culicidae	Aedes	Aedes aegypti	SPECIES	-12.69000	-39.13000	1264874399
3554	Diptera	Culicidae	Aedes	Aedes aegypti	SPECIES	-12.69000	-38.49000	1264874401
3555	Diptera	Culicidae	Aedes	Aedes aegypti	SPECIES	-12.69000	-55.60000	1264874408
3556	Diptera	Culicidae	Aedes	Aedes aegypti	SPECIES	-12.67000	-38.20000	1264874363
3557	Diptera	Culicidae	Aedes	Aedes aegypti	SPECIES	-12.67000	-49.04000	1264874380
3558	Diptera	Culicidae	Aedes	Aedes aegypti	SPECIES	-12.67000	-44.47000	1264874381
3559	Diptera	Culicidae	Aedes	Aedes aegypti	SPECIES	-12.67000	-38.90000	1264874395
3560	Diptera	Culicidae	Aedes	Aedes aegypti	SPECIES	-12.66000	-39.00000	1264874379
3561	Diptera	Culicidae	Aedes	Aedes aegypti	SPECIES	-12.64000	-38.63000	1264874411
3562	Diptera	Culicidae	Aedes	Aedes aegypti	SPECIES	-12.63000	-46.43000	1264874388
3563	Diptera	Culicidae	Aedes	Aedes aegypti	SPECIES	-12.62000	-39.00000	1264874412
3564	Diptera	Culicidae	Aedes	Aedes aegypti	SPECIES	-12.62000	49.15000	1264886051
3565	Diptera	Culicidae	Aedes	Aedes aegypti	SPECIES	-12.61000	-38.14000	1264874373
3566	Diptera	Culicidae	Aedes	Aedes aegypti	SPECIES	-12.61000	-40.86000	1264874387
3567	Diptera	Culicidae	Aedes	Aedes aegypti	SPECIES	-12.59000	-39.08000	1264874402

№	Order	Family	Genus	Species	Taxon Rank	Latitude	Longitude	GBIF_ID
3568	Diptera	Culicidae	Aedes	Aedes aegypti	SPECIES	-12.59000	-69.20000	1264887622
3569	Diptera	Culicidae	Aedes	Aedes aegypti	SPECIES	-12.58000	-39.19000	1264874389
3570	Diptera	Culicidae	Aedes	Aedes aegypti	SPECIES	-12.58000	-60.90000	1264874403
3571	Diptera	Culicidae	Aedes	Aedes aegypti	SPECIES	-12.57000	-61.97000	1264874398
3572	Diptera	Culicidae	Aedes	Aedes aegypti	SPECIES	-12.57000	-56.52000	1264874417
3573	Diptera	Culicidae	Aedes	Aedes aegypti	SPECIES	-12.55000	-38.76000	1264874394
3574	Diptera	Culicidae	Aedes	Aedes aegypti	SPECIES	-12.55000	-42.33000	1264874418
3575	Diptera	Culicidae	Aedes	Aedes aegypti	SPECIES	-12.54000	-48.38000	1264874400
3576	Diptera	Culicidae	Aedes	Aedes aegypti	SPECIES	-12.53000	-41.57000	1264874397
3577	Diptera	Culicidae	Aedes	Aedes aegypti	SPECIES	-12.52000	-38.49000	1264874406
3578	Diptera	Culicidae	Aedes	Aedes aegypti	SPECIES	-12.52000	-40.25000	1264874416
3579	Diptera	Culicidae	Aedes	Aedes aegypti	SPECIES	-12.51000	-38.98000	1264874415
3580	Diptera	Culicidae	Aedes	Aedes aegypti	SPECIES	-12.50000	-44.13000	1264874434
3581	Diptera	Culicidae	Aedes	Aedes aegypti	SPECIES	-12.49000	-55.33000	1264874407
3582	Diptera	Culicidae	Aedes	Aedes aegypti	SPECIES	-12.49000	-43.71000	1264874421
3583	Diptera	Culicidae	Aedes	Aedes aegypti	SPECIES	-12.49000	-38.14000	1264874428
3584	Diptera	Culicidae	Aedes	Aedes aegypti	SPECIES	-12.48221	130.82860	1100304599
3585	Diptera	Culicidae	Aedes	Aedes aegypti	SPECIES	-12.47000	-62.14000	1264874386
3586	Diptera	Culicidae	Aedes	Aedes aegypti	SPECIES	-12.47000	-41.73000	1264891074
3587	Diptera	Culicidae	Aedes	Aedes aegypti	SPECIES	-12.46000	-39.26000	1264874404
3588	Diptera	Culicidae	Aedes	Aedes aegypti	SPECIES	-12.46000	-39.62000	1264874405
3589	Diptera	Culicidae	Aedes	Aedes aegypti	SPECIES	-12.45200	130.82800	177194672
3590	Diptera	Culicidae	Aedes	Aedes aegypti	SPECIES	-12.45000	-41.91000	1264874419
3591	Diptera	Culicidae	Aedes	Aedes aegypti	SPECIES	-12.45000	130.86667	1633667574
3592	Diptera	Culicidae	Aedes	Aedes aegypti	SPECIES	-12.45000	130.86670	1633667727
3593	Diptera	Culicidae	Aedes	Aedes aegypti	SPECIES	-12.44000	-38.73000	1264874436
3594	Diptera	Culicidae	Aedes	Aedes aegypti	SPECIES	-12.42000	-41.32000	1264874413
3595	Diptera	Culicidae	Aedes	Aedes aegypti	SPECIES	-12.41000	-49.12000	1264874409
3596	Diptera	Culicidae	Aedes	Aedes aegypti	SPECIES	-12.41000	-38.95000	1264874424
3597	Diptera	Culicidae	Aedes	Aedes aegypti	SPECIES	-12.39000	-49.87000	1264874396
3598	Diptera	Culicidae	Aedes	Aedes aegypti	SPECIES	-12.39000	-38.60000	1264874435
3599	Diptera	Culicidae	Aedes	Aedes aegypti	SPECIES	-12.38000	-63.17000	1264874414
3600	Diptera	Culicidae	Aedes	Aedes aegypti	SPECIES	-12.38000	-40.95000	1264874420
3601	Diptera	Culicidae	Aedes	Aedes aegypti	SPECIES	-12.38000	-39.15000	1264874426
3602	Diptera	Culicidae	Aedes	Aedes aegypti	SPECIES	-12.35000	-38.26000	1264874422
3603	Diptera	Culicidae	Aedes	Aedes aegypti	SPECIES	-12.35000	-46.55000	1264874442
3604	Diptera	Culicidae	Aedes	Aedes aegypti	SPECIES	-12.35000	49.20000	1264889533
3605	Diptera	Culicidae	Aedes	Aedes aegypti	SPECIES	-12.33000	-38.74000	1264874427
3606	Diptera	Culicidae	Aedes	Aedes aegypti	SPECIES	-12.33000	-38.41000	1264874443
3607	Diptera	Culicidae	Aedes	Aedes aegypti	SPECIES	-12.31000	-50.96000	1264874423
3608	Diptera	Culicidae	Aedes	Aedes aegypti	SPECIES	-12.31000	-61.41000	1264874431

№	Order	Family	Genus	Species	Taxon Rank	Latitude	Longitude	GBIF_ID
3609	Diptera	Culicidae	Aedes	Aedes aegypti	SPECIES	-12.31000	-38.03000	1264874441
3610	Diptera	Culicidae	Aedes	Aedes aegypti	SPECIES	-12.30000	-39.33000	1264874463
3611	Diptera	Culicidae	Aedes	Aedes aegypti	SPECIES	-12.28000	-44.70000	1264874439
3612	Diptera	Culicidae	Aedes	Aedes aegypti	SPECIES	-12.28000	49.28000	1264888583
3613	Diptera	Culicidae	Aedes	Aedes aegypti	SPECIES	-12.27000	-40.61000	1264874432
3614	Diptera	Culicidae	Aedes	Aedes aegypti	SPECIES	-12.26000	-41.60000	1264874448
3615	Diptera	Culicidae	Aedes	Aedes aegypti	SPECIES	-12.26000	-38.61000	1264874451
3616	Diptera	Culicidae	Aedes	Aedes aegypti	SPECIES	-12.25000	-38.97000	1264889169
3617	Diptera	Culicidae	Aedes	Aedes aegypti	SPECIES	-12.25000	-38.96000	1264889201
3618	Diptera	Culicidae	Aedes	Aedes aegypti	SPECIES	-12.25000	44.41000	1264891182
3619	Diptera	Culicidae	Aedes	Aedes aegypti	SPECIES	-12.24000	-49.28000	1264874410
3620	Diptera	Culicidae	Aedes	Aedes aegypti	SPECIES	-12.24000	-41.17000	1264874430
3621	Diptera	Culicidae	Aedes	Aedes aegypti	SPECIES	-12.23000	-52.82000	1264874429
3622	Diptera	Culicidae	Aedes	Aedes aegypti	SPECIES	-12.22000	-38.78000	1264874458
3623	Diptera	Culicidae	Aedes	Aedes aegypti	SPECIES	-12.21691	-76.99743	2883086582
3624	Diptera	Culicidae	Aedes	Aedes aegypti	SPECIES	-12.21000	-39.78000	1264874446
3625	Diptera	Culicidae	Aedes	Aedes aegypti	SPECIES	-12.20000	-46.03000	1264874425
3626	Diptera	Culicidae	Aedes	Aedes aegypti	SPECIES	-12.20000	-51.65000	1264874433
3627	Diptera	Culicidae	Aedes	Aedes aegypti	SPECIES	-12.20000	-39.05000	1264874438
3628	Diptera	Culicidae	Aedes	Aedes aegypti	SPECIES	-12.20000	-43.56000	1264874452
3629	Diptera	Culicidae	Aedes	Aedes aegypti	SPECIES	-12.20000	-42.75000	1264874454
3630	Diptera	Culicidae	Aedes	Aedes aegypti	SPECIES	-12.20000	136.77000	1264888034
3631	Diptera	Culicidae	Aedes	Aedes aegypti	SPECIES	-12.19000	-39.04000	1264890993
3632	Diptera	Culicidae	Aedes	Aedes aegypti	SPECIES	-12.18000	-39.24000	1264874462
3633	Diptera	Culicidae	Aedes	Aedes aegypti	SPECIES	-12.18000	-44.25000	1264874467
3634	Diptera	Culicidae	Aedes	Aedes aegypti	SPECIES	-12.17306	-76.93281	3760320960
3635	Diptera	Culicidae	Aedes	Aedes aegypti	SPECIES	-12.17000	26.38000	1264887797
3636	Diptera	Culicidae	Aedes	Aedes aegypti	SPECIES	-12.15000	-46.99000	1264874444
3637	Diptera	Culicidae	Aedes	Aedes aegypti	SPECIES	-12.15000	-47.31000	1264874445
3638	Diptera	Culicidae	Aedes	Aedes aegypti	SPECIES	-12.14000	-56.77000	1264874440
3639	Diptera	Culicidae	Aedes	Aedes aegypti	SPECIES	-12.14000	-61.71000	1264874453
3640	Diptera	Culicidae	Aedes	Aedes aegypti	SPECIES	-12.14000	-38.21000	1264874468
3641	Diptera	Culicidae	Aedes	Aedes aegypti	SPECIES	-12.13000	-38.65000	1264874449
3642	Diptera	Culicidae	Aedes	Aedes aegypti	SPECIES	-12.13000	-39.37000	1264874455
3643	Diptera	Culicidae	Aedes	Aedes aegypti	SPECIES	-12.12000	-60.29000	1264874466
3644	Diptera	Culicidae	Aedes	Aedes aegypti	SPECIES	-12.12000	-49.55000	1264874489
3645	Diptera	Culicidae	Aedes	Aedes aegypti	SPECIES	-12.10000	-38.02000	1264874457
3646	Diptera	Culicidae	Aedes	Aedes aegypti	SPECIES	-12.10000	-38.44000	1264874464
3647	Diptera	Culicidae	Aedes	Aedes aegypti	SPECIES	-12.10000	-40.25000	1264874475
3648	Diptera	Culicidae	Aedes	Aedes aegypti	SPECIES	-12.10000	-57.91000	1264874476
3649	Diptera	Culicidae	Aedes	Aedes aegypti	SPECIES	-12.09000	-64.07000	1264874456

№	Order	Family	Genus	Species	Taxon Rank	Latitude	Longitude	GBIF_ID
3650	Diptera	Culicidae	Aedes	Aedes aegypti	SPECIES	-12.09000	-48.81000	1264874471
3651	Diptera	Culicidae	Aedes	Aedes aegypti	SPECIES	-12.09000	-77.04000	1264887868
3652	Diptera	Culicidae	Aedes	Aedes aegypti	SPECIES	-12.08565	-76.99370	4162568984
3653	Diptera	Culicidae	Aedes	Aedes aegypti	SPECIES	-12.08000	-77.10000	1264887872
3654	Diptera	Culicidae	Aedes	Aedes aegypti	SPECIES	-12.07000	-51.37000	1264874437
3655	Diptera	Culicidae	Aedes	Aedes aegypti	SPECIES	-12.07000	-42.47000	1264874450
3656	Diptera	Culicidae	Aedes	Aedes aegypti	SPECIES	-12.07000	-46.58000	1264874483
3657	Diptera	Culicidae	Aedes	Aedes aegypti	SPECIES	-12.06995	-76.95059	2814154279
3658	Diptera	Culicidae	Aedes	Aedes aegypti	SPECIES	-12.06000	-38.52000	1264874460
3659	Diptera	Culicidae	Aedes	Aedes aegypti	SPECIES	-12.06000	-38.75000	1264874481
3660	Diptera	Culicidae	Aedes	Aedes aegypti	SPECIES	-12.05553	-76.94468	2603383404
3661	Diptera	Culicidae	Aedes	Aedes aegypti	SPECIES	-12.05310	-76.94885	2456492287
3662	Diptera	Culicidae	Aedes	Aedes aegypti	SPECIES	-12.04000	-37.93000	1264874461
3663	Diptera	Culicidae	Aedes	Aedes aegypti	SPECIES	-12.04000	-77.03000	1264887859
3664	Diptera	Culicidae	Aedes	Aedes aegypti	SPECIES	-12.04000	-77.02000	1264887885
3665	Diptera	Culicidae	Aedes	Aedes aegypti	SPECIES	-12.04000	-77.04000	1264890649
3666	Diptera	Culicidae	Aedes	Aedes aegypti	SPECIES	-12.03816	-77.19091	3764328721
3667	Diptera	Culicidae	Aedes	Aedes aegypti	SPECIES	-12.03000	-77.08000	1264888122
3668	Diptera	Culicidae	Aedes	Aedes aegypti	SPECIES	-12.03000	-77.03000	1264888569
3669	Diptera	Culicidae	Aedes	Aedes aegypti	SPECIES	-12.02000	-38.65000	1264874447
3670	Diptera	Culicidae	Aedes	Aedes aegypti	SPECIES	-12.02000	-45.49000	1264874469
3671	Diptera	Culicidae	Aedes	Aedes aegypti	SPECIES	-12.02000	-41.52000	1264874470
3672	Diptera	Culicidae	Aedes	Aedes aegypti	SPECIES	-12.02000	-42.15000	1264874477
3673	Diptera	Culicidae	Aedes	Aedes aegypti	SPECIES	-12.02000	-41.06000	1264874487
3674	Diptera	Culicidae	Aedes	Aedes aegypti	SPECIES	-12.02000	-77.03000	1264887852
3675	Diptera	Culicidae	Aedes	Aedes aegypti	SPECIES	-12.01000	-77.03000	1264887880
3676	Diptera	Culicidae	Aedes	Aedes aegypti	SPECIES	-12.00000	-76.88000	1264887850
3677	Diptera	Culicidae	Aedes	Aedes aegypti	SPECIES	-12.00000	-76.86000	1264887864
3678	Diptera	Culicidae	Aedes	Aedes aegypti	SPECIES	-11.98000	-41.74000	1264874494
3679	Diptera	Culicidae	Aedes	Aedes aegypti	SPECIES	-11.97000	-43.16000	1264874479
3680	Diptera	Culicidae	Aedes	Aedes aegypti	SPECIES	-11.97000	-41.33000	1264874490
3681	Diptera	Culicidae	Aedes	Aedes aegypti	SPECIES	-11.96889	-76.78378	3031809121
3682	Diptera	Culicidae	Aedes	Aedes aegypti	SPECIES	-11.96000	-38.86000	1264874472
3683	Diptera	Culicidae	Aedes	Aedes aegypti	SPECIES	-11.96000	-40.15000	1264874501
3684	Diptera	Culicidae	Aedes	Aedes aegypti	SPECIES	-11.96000	-44.64000	1264874514
3685	Diptera	Culicidae	Aedes	Aedes aegypti	SPECIES	-11.96000	-77.06000	1264888592
3686	Diptera	Culicidae	Aedes	Aedes aegypti	SPECIES	-11.95000	-54.85000	1264874474
3687	Diptera	Culicidae	Aedes	Aedes aegypti	SPECIES	-11.95000	-49.20000	1264874482
3688	Diptera	Culicidae	Aedes	Aedes aegypti	SPECIES	-11.95000	-37.92000	1264874488
3689	Diptera	Culicidae	Aedes	Aedes aegypti	SPECIES	-11.94000	-39.09000	1264874480
3690	Diptera	Culicidae	Aedes	Aedes aegypti	SPECIES	-11.94000	-56.05000	1264874502

№	Order	Family	Genus	Species	Taxon Rank	Latitude	Longitude	GBIF_ID
3691	Diptera	Culicidae	Aedes	Aedes aegypti	SPECIES	-11.93000	-48.56000	1264874465
3692	Diptera	Culicidae	Aedes	Aedes aegypti	SPECIES	-11.93000	-38.99000	1264874493
3693	Diptera	Culicidae	Aedes	Aedes aegypti	SPECIES	-11.93000	-77.05000	1264887648
3694	Diptera	Culicidae	Aedes	Aedes aegypti	SPECIES	-11.92000	-61.46000	1264874498
3695	Diptera	Culicidae	Aedes	Aedes aegypti	SPECIES	-11.91000	-61.31000	1264874478
3696	Diptera	Culicidae	Aedes	Aedes aegypti	SPECIES	-11.89000	-39.60000	1264874459
3697	Diptera	Culicidae	Aedes	Aedes aegypti	SPECIES	-11.89000	-40.78000	1264874486
3698	Diptera	Culicidae	Aedes	Aedes aegypti	SPECIES	-11.89000	-63.26000	1264874512
3699	Diptera	Culicidae	Aedes	Aedes aegypti	SPECIES	-11.88000	-39.18000	1264874506
3700	Diptera	Culicidae	Aedes	Aedes aegypti	SPECIES	-11.88000	-39.89000	1264874519
3701	Diptera	Culicidae	Aedes	Aedes aegypti	SPECIES	-11.87000	-40.59000	1264874511
3702	Diptera	Culicidae	Aedes	Aedes aegypti	SPECIES	-11.86000	-60.94000	1264874485
3703	Diptera	Culicidae	Aedes	Aedes aegypti	SPECIES	-11.86000	-37.69000	1264874492
3704	Diptera	Culicidae	Aedes	Aedes aegypti	SPECIES	-11.85000	-41.85000	1264874500
3705	Diptera	Culicidae	Aedes	Aedes aegypti	SPECIES	-11.84000	-42.51000	1264874473
3706	Diptera	Culicidae	Aedes	Aedes aegypti	SPECIES	-11.83000	-38.73000	1264874518
3707	Diptera	Culicidae	Aedes	Aedes aegypti	SPECIES	-11.83000	43.45000	1264890446
3708	Diptera	Culicidae	Aedes	Aedes aegypti	SPECIES	-11.82000	-39.44000	1264874491
3709	Diptera	Culicidae	Aedes	Aedes aegypti	SPECIES	-11.82000	-54.24000	1264874505
3710	Diptera	Culicidae	Aedes	Aedes aegypti	SPECIES	-11.81000	-46.34000	1264874495
3711	Diptera	Culicidae	Aedes	Aedes aegypti	SPECIES	-11.81000	-42.61000	1264891126
3712	Diptera	Culicidae	Aedes	Aedes aegypti	SPECIES	-11.80000	-46.77000	1264874503
3713	Diptera	Culicidae	Aedes	Aedes aegypti	SPECIES	-11.80000	-43.90000	1264874526
3714	Diptera	Culicidae	Aedes	Aedes aegypti	SPECIES	-11.79000	-43.02000	1264874510
3715	Diptera	Culicidae	Aedes	Aedes aegypti	SPECIES	-11.79000	-51.76000	1264874516
3716	Diptera	Culicidae	Aedes	Aedes aegypti	SPECIES	-11.78000	-38.91000	1264874496
3717	Diptera	Culicidae	Aedes	Aedes aegypti	SPECIES	-11.78000	-41.72000	1264874508
3718	Diptera	Culicidae	Aedes	Aedes aegypti	SPECIES	-11.78000	-76.64000	1264891098
3719	Diptera	Culicidae	Aedes	Aedes aegypti	SPECIES	-11.77000	-56.71000	1264874499
3720	Diptera	Culicidae	Aedes	Aedes aegypti	SPECIES	-11.77000	-48.12000	1264874504
3721	Diptera	Culicidae	Aedes	Aedes aegypti	SPECIES	-11.75000	-38.41000	1264874484
3722	Diptera	Culicidae	Aedes	Aedes aegypti	SPECIES	-11.74000	-61.79000	1264874527
3723	Diptera	Culicidae	Aedes	Aedes aegypti	SPECIES	-11.74000	-44.25000	1264874532
3724	Diptera	Culicidae	Aedes	Aedes aegypti	SPECIES	-11.74000	-55.45000	1264874537
3725	Diptera	Culicidae	Aedes	Aedes aegypti	SPECIES	-11.74000	-76.70000	1264887650
3726	Diptera	Culicidae	Aedes	Aedes aegypti	SPECIES	-11.74000	43.23000	1264890415
3727	Diptera	Culicidae	Aedes	Aedes aegypti	SPECIES	-11.73000	-40.14000	1264874513
3728	Diptera	Culicidae	Aedes	Aedes aegypti	SPECIES	-11.73000	-38.18000	1264874525
3729	Diptera	Culicidae	Aedes	Aedes aegypti	SPECIES	-11.73000	-47.75000	1264874531
3730	Diptera	Culicidae	Aedes	Aedes aegypti	SPECIES	-11.72000	-41.51000	1264874517
3731	Diptera	Culicidae	Aedes	Aedes aegypti	SPECIES	-11.72000	43.25000	1264887374

№	Order	Family	Genus	Species	Taxon Rank	Latitude	Longitude	GBIF_ID
3732	Diptera	Culicidae	Aedes	Aedes aegypti	SPECIES	-11.71000	-39.15000	1264874507
3733	Diptera	Culicidae	Aedes	Aedes aegypti	SPECIES	-11.70000	43.25000	1264886100
3734	Diptera	Culicidae	Aedes	Aedes aegypti	SPECIES	-11.70000	43.24000	1264891141
3735	Diptera	Culicidae	Aedes	Aedes aegypti	SPECIES	-11.69000	-62.06000	1264874497
3736	Diptera	Culicidae	Aedes	Aedes aegypti	SPECIES	-11.69000	-40.59000	1264874533
3737	Diptera	Culicidae	Aedes	Aedes aegypti	SPECIES	-11.68000	-45.37000	1264874515
3738	Diptera	Culicidae	Aedes	Aedes aegypti	SPECIES	-11.68000	-39.82000	1264874521
3739	Diptera	Culicidae	Aedes	Aedes aegypti	SPECIES	-11.68000	-39.00000	1264874530
3740	Diptera	Culicidae	Aedes	Aedes aegypti	SPECIES	-11.68000	-37.99000	1264874543
3741	Diptera	Culicidae	Aedes	Aedes aegypti	SPECIES	-11.66000	-48.87000	1264874541
3742	Diptera	Culicidae	Aedes	Aedes aegypti	SPECIES	-11.63000	-62.22000	1264874528
3743	Diptera	Culicidae	Aedes	Aedes aegypti	SPECIES	-11.62000	-62.72000	1264874520
3744	Diptera	Culicidae	Aedes	Aedes aegypti	SPECIES	-11.62000	-39.65000	1264874523
3745	Diptera	Culicidae	Aedes	Aedes aegypti	SPECIES	-11.61000	-37.59000	1264874539
3746	Diptera	Culicidae	Aedes	Aedes aegypti	SPECIES	-11.60000	-40.08000	1264874538
3747	Diptera	Culicidae	Aedes	Aedes aegypti	SPECIES	-11.57000	-38.77000	1264874549
3748	Diptera	Culicidae	Aedes	Aedes aegypti	SPECIES	-11.57000	47.29000	1264886034
3749	Diptera	Culicidae	Aedes	Aedes aegypti	SPECIES	-11.56000	-41.90000	1264874548
3750	Diptera	Culicidae	Aedes	Aedes aegypti	SPECIES	-11.56000	160.07000	1264890083
3751	Diptera	Culicidae	Aedes	Aedes aegypti	SPECIES	-11.55000	-38.60000	1264874536
3752	Diptera	Culicidae	Aedes	Aedes aegypti	SPECIES	-11.55000	-47.07000	1264874557
3753	Diptera	Culicidae	Aedes	Aedes aegypti	SPECIES	-11.55000	43.39000	1264886058
3754	Diptera	Culicidae	Aedes	Aedes aegypti	SPECIES	-11.54000	-37.92000	1264874529
3755	Diptera	Culicidae	Aedes	Aedes aegypti	SPECIES	-11.53000	-54.26000	1264874509
3756	Diptera	Culicidae	Aedes	Aedes aegypti	SPECIES	-11.53000	-42.13000	1264874524
3757	Diptera	Culicidae	Aedes	Aedes aegypti	SPECIES	-11.52000	-40.31000	1264874522
3758	Diptera	Culicidae	Aedes	Aedes aegypti	SPECIES	-11.52000	-39.09000	1264874535
3759	Diptera	Culicidae	Aedes	Aedes aegypti	SPECIES	-11.52000	-41.80000	1264874552
3760	Diptera	Culicidae	Aedes	Aedes aegypti	SPECIES	-11.51000	-47.89000	1264874558
3761	Diptera	Culicidae	Aedes	Aedes aegypti	SPECIES	-11.49000	-38.96000	1264874534
3762	Diptera	Culicidae	Aedes	Aedes aegypti	SPECIES	-11.49000	-39.58000	1264874542
3763	Diptera	Culicidae	Aedes	Aedes aegypti	SPECIES	-11.49000	-38.14000	1264874544
3764	Diptera	Culicidae	Aedes	Aedes aegypti	SPECIES	-11.49000	-39.39000	1264874551
3765	Diptera	Culicidae	Aedes	Aedes aegypti	SPECIES	-11.49000	-39.31000	1264874554
3766	Diptera	Culicidae	Aedes	Aedes aegypti	SPECIES	-11.49000	-39.76000	1264874556
3767	Diptera	Culicidae	Aedes	Aedes aegypti	SPECIES	-11.49000	-59.39000	1264874562
3768	Diptera	Culicidae	Aedes	Aedes aegypti	SPECIES	-11.48000	-51.99000	1264874561
3769	Diptera	Culicidae	Aedes	Aedes aegypti	SPECIES	-11.48000	-55.07000	1264874565
3770	Diptera	Culicidae	Aedes	Aedes aegypti	SPECIES	-11.48000	-37.73000	1264874568
3771	Diptera	Culicidae	Aedes	Aedes aegypti	SPECIES	-11.47000	-37.51000	1264874545
3772	Diptera	Culicidae	Aedes	Aedes aegypti	SPECIES	-11.47000	-47.13000	1264874555

№	Order	Family	Genus	Species	Taxon Rank	Latitude	Longitude	GBIF_ID
3773	Diptera	Culicidae	Aedes	Aedes aegypti	SPECIES	-11.45000	-40.28000	1264874567
3774	Diptera	Culicidae	Aedes	Aedes aegypti	SPECIES	-11.44000	-61.85000	1264874564
3775	Diptera	Culicidae	Aedes	Aedes aegypti	SPECIES	-11.43000	-39.94000	1264874546
3776	Diptera	Culicidae	Aedes	Aedes aegypti	SPECIES	-11.42000	-40.63000	1264874553
3777	Diptera	Culicidae	Aedes	Aedes aegypti	SPECIES	-11.42000	-38.36000	1264874571
3778	Diptera	Culicidae	Aedes	Aedes aegypti	SPECIES	-11.41000	-42.12000	1264874540
3779	Diptera	Culicidae	Aedes	Aedes aegypti	SPECIES	-11.41000	-48.11000	1264874547
3780	Diptera	Culicidae	Aedes	Aedes aegypti	SPECIES	-11.40000	-41.54000	1264874550
3781	Diptera	Culicidae	Aedes	Aedes aegypti	SPECIES	-11.40000	-37.67000	1264874569
3782	Diptera	Culicidae	Aedes	Aedes aegypti	SPECIES	-11.40000	-39.45000	1264874577
3783	Diptera	Culicidae	Aedes	Aedes aegypti	SPECIES	-11.40000	-57.30000	1264874580
3784	Diptera	Culicidae	Aedes	Aedes aegypti	SPECIES	-11.40000	-41.18000	1264874582
3785	Diptera	Culicidae	Aedes	Aedes aegypti	SPECIES	-11.40000	43.28000	1264890430
3786	Diptera	Culicidae	Aedes	Aedes aegypti	SPECIES	-11.39000	-42.60000	1264874578
3787	Diptera	Culicidae	Aedes	Aedes aegypti	SPECIES	-11.38000	-49.39000	1264874589
3788	Diptera	Culicidae	Aedes	Aedes aegypti	SPECIES	-11.37000	-60.82000	1264874559
3789	Diptera	Culicidae	Aedes	Aedes aegypti	SPECIES	-11.37000	-40.10000	1264874560
3790	Diptera	Culicidae	Aedes	Aedes aegypti	SPECIES	-11.37000	-37.53000	1264874575
3791	Diptera	Culicidae	Aedes	Aedes aegypti	SPECIES	-11.37000	-37.87000	1264874583
3792	Diptera	Culicidae	Aedes	Aedes aegypti	SPECIES	-11.36000	-48.94000	1264874573
3793	Diptera	Culicidae	Aedes	Aedes aegypti	SPECIES	-11.33000	-62.64000	1264874566
3794	Diptera	Culicidae	Aedes	Aedes aegypti	SPECIES	-11.32000	-64.47000	1264874570
3795	Diptera	Culicidae	Aedes	Aedes aegypti	SPECIES	-11.32000	-38.58000	1264874587
3796	Diptera	Culicidae	Aedes	Aedes aegypti	SPECIES	-11.31000	-41.86000	1264874588
3797	Diptera	Culicidae	Aedes	Aedes aegypti	SPECIES	-11.31000	-46.74000	1264874594
3798	Diptera	Culicidae	Aedes	Aedes aegypti	SPECIES	-11.30000	-61.40000	1264874572
3799	Diptera	Culicidae	Aedes	Aedes aegypti	SPECIES	-11.30000	-39.97000	1264874599
3800	Diptera	Culicidae	Aedes	Aedes aegypti	SPECIES	-11.30000	-61.32000	1264890828
3801	Diptera	Culicidae	Aedes	Aedes aegypti	SPECIES	-11.29000	-41.98000	1264874563
3802	Diptera	Culicidae	Aedes	Aedes aegypti	SPECIES	-11.27000	-37.80000	1264874593
3803	Diptera	Culicidae	Aedes	Aedes aegypti	SPECIES	-11.26000	-37.59000	1264874576
3804	Diptera	Culicidae	Aedes	Aedes aegypti	SPECIES	-11.25000	-37.41000	1264874602
3805	Diptera	Culicidae	Aedes	Aedes aegypti	SPECIES	-11.25000	-42.02000	1264891070
3806	Diptera	Culicidae	Aedes	Aedes aegypti	SPECIES	-11.24000	-39.05000	1264874586
3807	Diptera	Culicidae	Aedes	Aedes aegypti	SPECIES	-11.24000	-68.69000	1264887301
3808	Diptera	Culicidae	Aedes	Aedes aegypti	SPECIES	-11.23000	-51.81000	1264874579
3809	Diptera	Culicidae	Aedes	Aedes aegypti	SPECIES	-11.23000	-57.57000	1264874581
3810	Diptera	Culicidae	Aedes	Aedes aegypti	SPECIES	-11.22000	-41.56000	1264874584
3811	Diptera	Culicidae	Aedes	Aedes aegypti	SPECIES	-11.21000	-37.65000	1264874606
3812	Diptera	Culicidae	Aedes	Aedes aegypti	SPECIES	-11.20000	-39.55000	1264874574
3813	Diptera	Culicidae	Aedes	Aedes aegypti	SPECIES	-11.20000	-47.56000	1264874592

№	Order	Family	Genus	Species	Taxon Rank	Latitude	Longitude	GBIF_ID
3814	Diptera	Culicidae	Aedes	Aedes aegypti	SPECIES	-11.19000	-61.91000	1264874607
3815	Diptera	Culicidae	Aedes	Aedes aegypti	SPECIES	-11.16000	-49.08000	1264874585
3816	Diptera	Culicidae	Aedes	Aedes aegypti	SPECIES	-11.16000	-61.59000	1264874608
3817	Diptera	Culicidae	Aedes	Aedes aegypti	SPECIES	-11.16000	-38.21000	1264874616
3818	Diptera	Culicidae	Aedes	Aedes aegypti	SPECIES	-11.14000	-40.23000	1264874605
3819	Diptera	Culicidae	Aedes	Aedes aegypti	SPECIES	-11.14000	-42.08000	1264874610
3820	Diptera	Culicidae	Aedes	Aedes aegypti	SPECIES	-11.13000	-55.62000	1264874612
3821	Diptera	Culicidae	Aedes	Aedes aegypti	SPECIES	-11.12000	-48.39000	1264874590
3822	Diptera	Culicidae	Aedes	Aedes aegypti	SPECIES	-11.12000	-41.02000	1264874595
3823	Diptera	Culicidae	Aedes	Aedes aegypti	SPECIES	-11.11000	-62.42000	1264874596
3824	Diptera	Culicidae	Aedes	Aedes aegypti	SPECIES	-11.11000	-38.51000	1264874614
3825	Diptera	Culicidae	Aedes	Aedes aegypti	SPECIES	-11.11000	-47.79000	1264874619
3826	Diptera	Culicidae	Aedes	Aedes aegypti	SPECIES	-11.10000	-37.62000	1264874591
3827	Diptera	Culicidae	Aedes	Aedes aegypti	SPECIES	-11.09000	-40.67000	1264874609
3828	Diptera	Culicidae	Aedes	Aedes aegypti	SPECIES	-11.08000	-43.32000	1264874600
3829	Diptera	Culicidae	Aedes	Aedes aegypti	SPECIES	-11.08000	-37.99000	1264874618
3830	Diptera	Culicidae	Aedes	Aedes aegypti	SPECIES	-11.07000	-48.63000	1264874598
3831	Diptera	Culicidae	Aedes	Aedes aegypti	SPECIES	-11.07000	-56.78000	1264874601
3832	Diptera	Culicidae	Aedes	Aedes aegypti	SPECIES	-11.06000	-37.47000	1264874604
3833	Diptera	Culicidae	Aedes	Aedes aegypti	SPECIES	-11.06000	-37.33000	1264874624
3834	Diptera	Culicidae	Aedes	Aedes aegypti	SPECIES	-11.05000	-50.09000	1264874627
3835	Diptera	Culicidae	Aedes	Aedes aegypti	SPECIES	-11.05000	-62.87000	1264874630
3836	Diptera	Culicidae	Aedes	Aedes aegypti	SPECIES	-11.05000	-39.73000	1264874631
3837	Diptera	Culicidae	Aedes	Aedes aegypti	SPECIES	-11.04000	-40.18000	1264874597
3838	Diptera	Culicidae	Aedes	Aedes aegypti	SPECIES	-11.04000	-44.16000	1264874615
3839	Diptera	Culicidae	Aedes	Aedes aegypti	SPECIES	-11.04000	-41.68000	1264874638
3840	Diptera	Culicidae	Aedes	Aedes aegypti	SPECIES	-11.02000	-37.79000	1264874633
3841	Diptera	Culicidae	Aedes	Aedes aegypti	SPECIES	-11.02000	-68.76000	1264887284
3842	Diptera	Culicidae	Aedes	Aedes aegypti	SPECIES	-11.01000	-37.10000	1264874632
3843	Diptera	Culicidae	Aedes	Aedes aegypti	SPECIES	-11.00000	-38.82000	1264874611
3844	Diptera	Culicidae	Aedes	Aedes aegypti	SPECIES	-10.99000	-40.13000	1264874603
3845	Diptera	Culicidae	Aedes	Aedes aegypti	SPECIES	-10.99000	-44.56000	1264874636
3846	Diptera	Culicidae	Aedes	Aedes aegypti	SPECIES	-10.99000	-37.09000	1264890987
3847	Diptera	Culicidae	Aedes	Aedes aegypti	SPECIES	-10.98000	-51.04000	1264874620
3848	Diptera	Culicidae	Aedes	Aedes aegypti	SPECIES	-10.97000	-37.21000	1264874617
3849	Diptera	Culicidae	Aedes	Aedes aegypti	SPECIES	-10.96000	-42.80000	1264874623
3850	Diptera	Culicidae	Aedes	Aedes aegypti	SPECIES	-10.96000	-38.40000	1264874634
3851	Diptera	Culicidae	Aedes	Aedes aegypti	SPECIES	-10.96000	-49.31000	1264874639
3852	Diptera	Culicidae	Aedes	Aedes aegypti	SPECIES	-10.95000	-58.66000	1264874628
3853	Diptera	Culicidae	Aedes	Aedes aegypti	SPECIES	-10.95000	-41.87000	1264874641
3854	Diptera	Culicidae	Aedes	Aedes aegypti	SPECIES	-10.95000	39.27000	1264890145

№	Order	Family	Genus	Species	Taxon Rank	Latitude	Longitude	GBIF_ID
3855	Diptera	Culicidae	Aedes	Aedes aegypti	SPECIES	-10.93000	-62.31000	1264874644
3856	Diptera	Culicidae	Aedes	Aedes aegypti	SPECIES	-10.92000	-54.00000	1264874613
3857	Diptera	Culicidae	Aedes	Aedes aegypti	SPECIES	-10.91000	-54.95000	1264874621
3858	Diptera	Culicidae	Aedes	Aedes aegypti	SPECIES	-10.91000	-40.38000	1264874625
3859	Diptera	Culicidae	Aedes	Aedes aegypti	SPECIES	-10.91000	-37.06000	1264890462
3860	Diptera	Culicidae	Aedes	Aedes aegypti	SPECIES	-10.90000	-37.05000	1264890494
3861	Diptera	Culicidae	Aedes	Aedes aegypti	SPECIES	-10.89000	-39.46000	1264874643
3862	Diptera	Culicidae	Aedes	Aedes aegypti	SPECIES	-10.89000	-62.53000	1264874651
3863	Diptera	Culicidae	Aedes	Aedes aegypti	SPECIES	-10.88000	-37.67000	1264874656
3864	Diptera	Culicidae	Aedes	Aedes aegypti	SPECIES	-10.88000	-68.61000	1264874662
3865	Diptera	Culicidae	Aedes	Aedes aegypti	SPECIES	-10.87000	-37.17000	1264874657
3866	Diptera	Culicidae	Aedes	Aedes aegypti	SPECIES	-10.86000	-45.78000	1264874622
3867	Diptera	Culicidae	Aedes	Aedes aegypti	SPECIES	-10.85000	-36.97000	1264874659
3868	Diptera	Culicidae	Aedes	Aedes aegypti	SPECIES	-10.83000	-41.16000	1264874640
3869	Diptera	Culicidae	Aedes	Aedes aegypti	SPECIES	-10.83000	-40.63000	1264874652
3870	Diptera	Culicidae	Aedes	Aedes aegypti	SPECIES	-10.83000	-48.90000	1264874658
3871	Diptera	Culicidae	Aedes	Aedes aegypti	SPECIES	-10.82000	-63.10000	1264874626
3872	Diptera	Culicidae	Aedes	Aedes aegypti	SPECIES	-10.82000	-38.11000	1264874635
3873	Diptera	Culicidae	Aedes	Aedes aegypti	SPECIES	-10.81000	-39.12000	1264874629
3874	Diptera	Culicidae	Aedes	Aedes aegypti	SPECIES	-10.80000	-37.19000	1264874660
3875	Diptera	Culicidae	Aedes	Aedes aegypti	SPECIES	-10.79000	-37.33000	1264874645
3876	Diptera	Culicidae	Aedes	Aedes aegypti	SPECIES	-10.79000	-36.98000	1264874663
3877	Diptera	Culicidae	Aedes	Aedes aegypti	SPECIES	-10.78000	-37.50000	1264874642
3878	Diptera	Culicidae	Aedes	Aedes aegypti	SPECIES	-10.77000	-37.57000	1264874665
3879	Diptera	Culicidae	Aedes	Aedes aegypti	SPECIES	-10.76000	-39.49000	1264874637
3880	Diptera	Culicidae	Aedes	Aedes aegypti	SPECIES	-10.76000	-51.61000	1264874647
3881	Diptera	Culicidae	Aedes	Aedes aegypti	SPECIES	-10.76000	-38.51000	1264874661
3882	Diptera	Culicidae	Aedes	Aedes aegypti	SPECIES	-10.76000	-45.14000	1264874667
3883	Diptera	Culicidae	Aedes	Aedes aegypti	SPECIES	-10.75000	-47.28000	1264874646
3884	Diptera	Culicidae	Aedes	Aedes aegypti	SPECIES	-10.75000	-38.29000	1264874669
3885	Diptera	Culicidae	Aedes	Aedes aegypti	SPECIES	-10.74000	-40.15000	1264874648
3886	Diptera	Culicidae	Aedes	Aedes aegypti	SPECIES	-10.74000	-42.22000	1264874650
3887	Diptera	Culicidae	Aedes	Aedes aegypti	SPECIES	-10.74000	-37.82000	1264874653
3888	Diptera	Culicidae	Aedes	Aedes aegypti	SPECIES	-10.74000	-48.02000	1264874666
3889	Diptera	Culicidae	Aedes	Aedes aegypti	SPECIES	-10.73000	-37.22000	1264874686
3890	Diptera	Culicidae	Aedes	Aedes aegypti	SPECIES	-10.72000	-70.01000	1264874668
3891	Diptera	Culicidae	Aedes	Aedes aegypti	SPECIES	-10.72000	-40.34000	1264874680
3892	Diptera	Culicidae	Aedes	Aedes aegypti	SPECIES	-10.72000	-37.11000	1264874683
3893	Diptera	Culicidae	Aedes	Aedes aegypti	SPECIES	-10.70000	-37.43000	1264874677
3894	Diptera	Culicidae	Aedes	Aedes aegypti	SPECIES	-10.70000	-69.23000	1264874682
3895	Diptera	Culicidae	Aedes	Aedes aegypti	SPECIES	-10.70000	-46.41000	1264874688

№	Order	Family	Genus	Species	Taxon Rank	Latitude	Longitude	GBIF_ID
3896	Diptera	Culicidae	Aedes	Aedes aegypti	SPECIES	-10.69000	-36.97000	1264874670
3897	Diptera	Culicidae	Aedes	Aedes aegypti	SPECIES	-10.69000	-39.84000	1264874689
3898	Diptera	Culicidae	Aedes	Aedes aegypti	SPECIES	-10.69000	-37.42000	1264887970
3899	Diptera	Culicidae	Aedes	Aedes aegypti	SPECIES	-10.69000	-37.43000	1264890474
3900	Diptera	Culicidae	Aedes	Aedes aegypti	SPECIES	-10.68000	-52.64000	1264874655
3901	Diptera	Culicidae	Aedes	Aedes aegypti	SPECIES	-10.68000	-37.02000	1264874675
3902	Diptera	Culicidae	Aedes	Aedes aegypti	SPECIES	-10.68000	-37.15000	1264874685
3903	Diptera	Culicidae	Aedes	Aedes aegypti	SPECIES	-10.68000	142.17000	1264887697
3904	Diptera	Culicidae	Aedes	Aedes aegypti	SPECIES	-10.68000	-37.42000	1264888483
3905	Diptera	Culicidae	Aedes	Aedes aegypti	SPECIES	-10.67000	-37.28000	1264874649
3906	Diptera	Culicidae	Aedes	Aedes aegypti	SPECIES	-10.67000	-56.09000	1264874654
3907	Diptera	Culicidae	Aedes	Aedes aegypti	SPECIES	-10.67000	-36.96000	1264874687
3908	Diptera	Culicidae	Aedes	Aedes aegypti	SPECIES	-10.66667	142.53330	1633667603
3909	Diptera	Culicidae	Aedes	Aedes aegypti	SPECIES	-10.66000	-44.85000	1264874664
3910	Diptera	Culicidae	Aedes	Aedes aegypti	SPECIES	-10.66000	-36.80000	1264874684
3911	Diptera	Culicidae	Aedes	Aedes aegypti	SPECIES	-10.66000	-48.88000	1264874691
3912	Diptera	Culicidae	Aedes	Aedes aegypti	SPECIES	-10.66000	-37.58000	1264874693
3913	Diptera	Culicidae	Aedes	Aedes aegypti	SPECIES	-10.65000	-62.63000	1264874671
3914	Diptera	Culicidae	Aedes	Aedes aegypti	SPECIES	-10.64000	-49.36000	1264874672
3915	Diptera	Culicidae	Aedes	Aedes aegypti	SPECIES	-10.63000	-37.68000	1264874676
3916	Diptera	Culicidae	Aedes	Aedes aegypti	SPECIES	-10.63000	-37.24000	1264874678
3917	Diptera	Culicidae	Aedes	Aedes aegypti	SPECIES	-10.62000	-38.64000	1264874696
3918	Diptera	Culicidae	Aedes	Aedes aegypti	SPECIES	-10.62000	142.17000	1264891324
3919	Diptera	Culicidae	Aedes	Aedes aegypti	SPECIES	-10.61667	150.66667	177195216
3920	Diptera	Culicidae	Aedes	Aedes aegypti	SPECIES	-10.61667	150.66667	1633667790
3921	Diptera	Culicidae	Aedes	Aedes aegypti	SPECIES	-10.61000	-68.58000	1264874704
3922	Diptera	Culicidae	Aedes	Aedes aegypti	SPECIES	-10.61000	142.29000	1264887696
3923	Diptera	Culicidae	Aedes	Aedes aegypti	SPECIES	-10.60000	150.64999	177195213
3924	Diptera	Culicidae	Aedes	Aedes aegypti	SPECIES	-10.60000	-37.32000	1264874690
3925	Diptera	Culicidae	Aedes	Aedes aegypti	SPECIES	-10.60000	-37.93000	1264874697
3926	Diptera	Culicidae	Aedes	Aedes aegypti	SPECIES	-10.60000	-40.25000	1264874710
3927	Diptera	Culicidae	Aedes	Aedes aegypti	SPECIES	-10.60000	150.64999	1633667593
3928	Diptera	Culicidae	Aedes	Aedes aegypti	SPECIES	-10.59000	-38.21000	1264874715
3929	Diptera	Culicidae	Aedes	Aedes aegypti	SPECIES	-10.59000	-37.45000	1264891080
3930	Diptera	Culicidae	Aedes	Aedes aegypti	SPECIES	-10.58110	142.22300	3443537530
3931	Diptera	Culicidae	Aedes	Aedes aegypti	SPECIES	-10.58000	-38.03000	1264874694
3932	Diptera	Culicidae	Aedes	Aedes aegypti	SPECIES	-10.58000	-37.76000	1264874703
3933	Diptera	Culicidae	Aedes	Aedes aegypti	SPECIES	-10.58000	142.21000	1264888798
3934	Diptera	Culicidae	Aedes	Aedes aegypti	SPECIES	-10.58000	142.22000	3818059817
3935	Diptera	Culicidae	Aedes	Aedes aegypti	SPECIES	-10.57000	-43.73000	1264874695
3936	Diptera	Culicidae	Aedes	Aedes aegypti	SPECIES	-10.57000	-41.19000	1264874705

№	Order	Family	Genus	Species	Taxon Rank	Latitude	Longitude	GBIF_ID
3937	Diptera	Culicidae	Aedes	Aedes aegypti	SPECIES	-10.56000	-37.14000	1264874674
3938	Diptera	Culicidae	Aedes	Aedes aegypti	SPECIES	-10.56000	-55.43000	1264874679
3939	Diptera	Culicidae	Aedes	Aedes aegypti	SPECIES	-10.56000	142.21000	1264887724
3940	Diptera	Culicidae	Aedes	Aedes aegypti	SPECIES	-10.55000	-48.48000	1264874681
3941	Diptera	Culicidae	Aedes	Aedes aegypti	SPECIES	-10.54000	-36.90000	1264874699
3942	Diptera	Culicidae	Aedes	Aedes aegypti	SPECIES	-10.53000	-48.94000	1264874709
3943	Diptera	Culicidae	Aedes	Aedes aegypti	SPECIES	-10.52000	-38.48000	1264874713
3944	Diptera	Culicidae	Aedes	Aedes aegypti	SPECIES	-10.51000	-36.62000	1264874698
3945	Diptera	Culicidae	Aedes	Aedes aegypti	SPECIES	-10.51000	-37.60000	1264874707
3946	Diptera	Culicidae	Aedes	Aedes aegypti	SPECIES	-10.51000	-62.01000	1264874712
3947	Diptera	Culicidae	Aedes	Aedes aegypti	SPECIES	-10.50000	-63.83000	1264874708
3948	Diptera	Culicidae	Aedes	Aedes aegypti	SPECIES	-10.50000	-37.43000	1264874720
3949	Diptera	Culicidae	Aedes	Aedes aegypti	SPECIES	-10.49000	-54.99000	1264874692
3950	Diptera	Culicidae	Aedes	Aedes aegypti	SPECIES	-10.48000	-37.04000	1264874673
3951	Diptera	Culicidae	Aedes	Aedes aegypti	SPECIES	-10.47000	-38.92000	1264874716
3952	Diptera	Culicidae	Aedes	Aedes aegypti	SPECIES	-10.47000	-36.49000	1264874717
3953	Diptera	Culicidae	Aedes	Aedes aegypti	SPECIES	-10.46000	-36.54000	1264874725
3954	Diptera	Culicidae	Aedes	Aedes aegypti	SPECIES	-10.46000	-37.24000	1264874735
3955	Diptera	Culicidae	Aedes	Aedes aegypti	SPECIES	-10.45000	-61.81000	1264874726
3956	Diptera	Culicidae	Aedes	Aedes aegypti	SPECIES	-10.45000	-40.10000	1264874731
3957	Diptera	Culicidae	Aedes	Aedes aegypti	SPECIES	-10.45000	-67.83000	1264874736
3958	Diptera	Culicidae	Aedes	Aedes aegypti	SPECIES	-10.44000	-36.80000	1264874714
3959	Diptera	Culicidae	Aedes	Aedes aegypti	SPECIES	-10.42000	-48.85000	1264874748
3960	Diptera	Culicidae	Aedes	Aedes aegypti	SPECIES	-10.41000	-44.19000	1264874701
3961	Diptera	Culicidae	Aedes	Aedes aegypti	SPECIES	-10.41000	-51.69000	1264874718
3962	Diptera	Culicidae	Aedes	Aedes aegypti	SPECIES	-10.40000	-64.55000	1264874702
3963	Diptera	Culicidae	Aedes	Aedes aegypti	SPECIES	-10.40000	-36.40000	1264874706
3964	Diptera	Culicidae	Aedes	Aedes aegypti	SPECIES	-10.40000	-58.59000	1264874739
3965	Diptera	Culicidae	Aedes	Aedes aegypti	SPECIES	-10.39000	-45.14000	1264874700
3966	Diptera	Culicidae	Aedes	Aedes aegypti	SPECIES	-10.39000	-38.29000	1264874722
3967	Diptera	Culicidae	Aedes	Aedes aegypti	SPECIES	-10.39000	142.14000	1264891212
3968	Diptera	Culicidae	Aedes	Aedes aegypti	SPECIES	-10.38000	-36.96000	1264874737
3969	Diptera	Culicidae	Aedes	Aedes aegypti	SPECIES	-10.38000	-36.68000	1264874740
3970	Diptera	Culicidae	Aedes	Aedes aegypti	SPECIES	-10.38000	-38.46000	1264874741
3971	Diptera	Culicidae	Aedes	Aedes aegypti	SPECIES	-10.37000	-37.38000	1264874719
3972	Diptera	Culicidae	Aedes	Aedes aegypti	SPECIES	-10.36000	-62.09000	1264874721
3973	Diptera	Culicidae	Aedes	Aedes aegypti	SPECIES	-10.35000	-37.72000	1264874711
3974	Diptera	Culicidae	Aedes	Aedes aegypti	SPECIES	-10.35000	-37.51000	1264874728
3975	Diptera	Culicidae	Aedes	Aedes aegypti	SPECIES	-10.35000	-47.49000	1264874742
3976	Diptera	Culicidae	Aedes	Aedes aegypti	SPECIES	-10.33000	-36.96000	1264874723
3977	Diptera	Culicidae	Aedes	Aedes aegypti	SPECIES	-10.33000	-62.97000	1264874733

№	Order	Family	Genus	Species	Taxon Rank	Latitude	Longitude	GBIF_ID
3978	Diptera	Culicidae	Aedes	Aedes aegypti	SPECIES	-10.33000	-36.87000	1264874738
3979	Diptera	Culicidae	Aedes	Aedes aegypti	SPECIES	-10.33000	-37.20000	1264874756
3980	Diptera	Culicidae	Aedes	Aedes aegypti	SPECIES	-10.32000	-39.46000	1264874759
3981	Diptera	Culicidae	Aedes	Aedes aegypti	SPECIES	-10.32000	-38.17000	1264874763
3982	Diptera	Culicidae	Aedes	Aedes aegypti	SPECIES	-10.31000	-61.00000	1264874752
3983	Diptera	Culicidae	Aedes	Aedes aegypti	SPECIES	-10.31000	-44.56000	1264874771
3984	Diptera	Culicidae	Aedes	Aedes aegypti	SPECIES	-10.30000	-37.32000	1264874727
3985	Diptera	Culicidae	Aedes	Aedes aegypti	SPECIES	-10.29000	-55.36000	1264874730
3986	Diptera	Culicidae	Aedes	Aedes aegypti	SPECIES	-10.29000	-47.72000	1264874734
3987	Diptera	Culicidae	Aedes	Aedes aegypti	SPECIES	-10.29000	-36.64000	1264874747
3988	Diptera	Culicidae	Aedes	Aedes aegypti	SPECIES	-10.29000	-36.34000	1264874754
3989	Diptera	Culicidae	Aedes	Aedes aegypti	SPECIES	-10.29000	-50.80000	1264874762
3990	Diptera	Culicidae	Aedes	Aedes aegypti	SPECIES	-10.28000	-37.97000	1264874757
3991	Diptera	Culicidae	Aedes	Aedes aegypti	SPECIES	-10.28000	-40.75000	1264874769
3992	Diptera	Culicidae	Aedes	Aedes aegypti	SPECIES	-10.27000	-37.05000	1264874724
3993	Diptera	Culicidae	Aedes	Aedes aegypti	SPECIES	-10.27000	-36.90000	1264874729
3994	Diptera	Culicidae	Aedes	Aedes aegypti	SPECIES	-10.25000	-39.91000	1264874745
3995	Diptera	Culicidae	Aedes	Aedes aegypti	SPECIES	-10.25000	-63.36000	1264874767
3996	Diptera	Culicidae	Aedes	Aedes aegypti	SPECIES	-10.24000	-67.36000	1264874743
3997	Diptera	Culicidae	Aedes	Aedes aegypti	SPECIES	-10.24000	-37.23000	1264874751
3998	Diptera	Culicidae	Aedes	Aedes aegypti	SPECIES	-10.24000	-36.78000	1264874764
3999	Diptera	Culicidae	Aedes	Aedes aegypti	SPECIES	-10.23000	-36.50000	1264874765
4000	Diptera	Culicidae	Aedes	Aedes aegypti	SPECIES	-10.22000	-48.85000	1264874744
4001	Diptera	Culicidae	Aedes	Aedes aegypti	SPECIES	-10.19000	-37.55000	1264874732
4002	Diptera	Culicidae	Aedes	Aedes aegypti	SPECIES	-10.19000	-36.89000	1264874750
4003	Diptera	Culicidae	Aedes	Aedes aegypti	SPECIES	-10.19000	-53.89000	1264874760
4004	Diptera	Culicidae	Aedes	Aedes aegypti	SPECIES	-10.19000	-48.11000	1264874777
4005	Diptera	Culicidae	Aedes	Aedes aegypti	SPECIES	-10.19000	123.60000	1264876389
4006	Diptera	Culicidae	Aedes	Aedes aegypti	SPECIES	-10.18000	-43.95000	1264874775
4007	Diptera	Culicidae	Aedes	Aedes aegypti	SPECIES	-10.17000	-49.21000	1264874772
4008	Diptera	Culicidae	Aedes	Aedes aegypti	SPECIES	-10.17000	-36.91000	1264874778
4009	Diptera	Culicidae	Aedes	Aedes aegypti	SPECIES	-10.17000	142.25000	1264887721
4010	Diptera	Culicidae	Aedes	Aedes aegypti	SPECIES	-10.16000	-47.40000	1264874811
4011	Diptera	Culicidae	Aedes	Aedes aegypti	SPECIES	-10.15000	-36.62000	1264874749
4012	Diptera	Culicidae	Aedes	Aedes aegypti	SPECIES	-10.15000	-37.04000	1264874779
4013	Diptera	Culicidae	Aedes	Aedes aegypti	SPECIES	-10.14000	-41.68000	1264874758
4014	Diptera	Culicidae	Aedes	Aedes aegypti	SPECIES	-10.12000	-45.29000	1264874785
4015	Diptera	Culicidae	Aedes	Aedes aegypti	SPECIES	-10.12000	-37.96000	1264874788
4016	Diptera	Culicidae	Aedes	Aedes aegypti	SPECIES	-10.12000	142.13000	1264887718
4017	Diptera	Culicidae	Aedes	Aedes aegypti	SPECIES	-10.11000	-36.75000	1264874753
4018	Diptera	Culicidae	Aedes	Aedes aegypti	SPECIES	-10.11000	-36.86000	1264874755

№	Order	Family	Genus	Species	Taxon Rank	Latitude	Longitude	GBIF_ID
4019	Diptera	Culicidae	Aedes	Aedes aegypti	SPECIES	-10.10000	-36.24000	1264874746
4020	Diptera	Culicidae	Aedes	Aedes aegypti	SPECIES	-10.10000	-37.15000	1264874773
4021	Diptera	Culicidae	Aedes	Aedes aegypti	SPECIES	-10.10000	-59.69000	1264874812
4022	Diptera	Culicidae	Aedes	Aedes aegypti	SPECIES	-10.09000	-63.94000	1264874766
4023	Diptera	Culicidae	Aedes	Aedes aegypti	SPECIES	-10.09000	-62.31000	1264874774
4024	Diptera	Culicidae	Aedes	Aedes aegypti	SPECIES	-10.08000	-37.01000	1264874768
4025	Diptera	Culicidae	Aedes	Aedes aegypti	SPECIES	-10.08000	-40.08000	1264874793
4026	Diptera	Culicidae	Aedes	Aedes aegypti	SPECIES	-10.07000	-37.62000	1264874805
4027	Diptera	Culicidae	Aedes	Aedes aegypti	SPECIES	-10.07000	-68.32000	1264890881
4028	Diptera	Culicidae	Aedes	Aedes aegypti	SPECIES	-10.06000	-36.79000	1264874780
4029	Diptera	Culicidae	Aedes	Aedes aegypti	SPECIES	-10.06000	-55.89000	1264874794
4030	Diptera	Culicidae	Aedes	Aedes aegypti	SPECIES	-10.04000	-46.65000	1264874761
4031	Diptera	Culicidae	Aedes	Aedes aegypti	SPECIES	-10.04000	-56.40000	1264874784
4032	Diptera	Culicidae	Aedes	Aedes aegypti	SPECIES	-10.04000	-52.53000	1264874814
4033	Diptera	Culicidae	Aedes	Aedes aegypti	SPECIES	-10.03000	-37.09000	1264874781
4034	Diptera	Culicidae	Aedes	Aedes aegypti	SPECIES	-10.02000	-51.42000	1264874790
4035	Diptera	Culicidae	Aedes	Aedes aegypti	SPECIES	-10.01000	-38.86000	1264874786
4036	Diptera	Culicidae	Aedes	Aedes aegypti	SPECIES	-10.00000	-68.42000	1264874783
4037	Diptera	Culicidae	Aedes	Aedes aegypti	SPECIES	-9.99000	-49.12000	1264874810
4038	Diptera	Culicidae	Aedes	Aedes aegypti	SPECIES	-9.98000	-63.02000	1264874770
4039	Diptera	Culicidae	Aedes	Aedes aegypti	SPECIES	-9.98000	-48.02000	1264874787
4040	Diptera	Culicidae	Aedes	Aedes aegypti	SPECIES	-9.98000	-67.46000	1264874789
4041	Diptera	Culicidae	Aedes	Aedes aegypti	SPECIES	-9.98000	-54.16000	1264874795
4042	Diptera	Culicidae	Aedes	Aedes aegypti	SPECIES	-9.98000	-67.82000	1264889472
4043	Diptera	Culicidae	Aedes	Aedes aegypti	SPECIES	-9.98000	-67.81000	1264890422
4044	Diptera	Culicidae	Aedes	Aedes aegypti	SPECIES	-9.97000	-45.67000	1264874801
4045	Diptera	Culicidae	Aedes	Aedes aegypti	SPECIES	-9.97000	-44.34000	1264874813
4046	Diptera	Culicidae	Aedes	Aedes aegypti	SPECIES	-9.97000	-38.60000	1264874831
4047	Diptera	Culicidae	Aedes	Aedes aegypti	SPECIES	-9.96000	-36.76000	1264874776
4048	Diptera	Culicidae	Aedes	Aedes aegypti	SPECIES	-9.96000	-36.38000	1264874791
4049	Diptera	Culicidae	Aedes	Aedes aegypti	SPECIES	-9.96000	-36.56000	1264874806
4050	Diptera	Culicidae	Aedes	Aedes aegypti	SPECIES	-9.96000	142.18000	1264887753
4051	Diptera	Culicidae	Aedes	Aedes aegypti	SPECIES	-9.96000	-67.80000	1264891481
4052	Diptera	Culicidae	Aedes	Aedes aegypti	SPECIES	-9.95000	-66.91000	1264874819
4053	Diptera	Culicidae	Aedes	Aedes aegypti	SPECIES	-9.95000	-49.79000	1264874829
4054	Diptera	Culicidae	Aedes	Aedes aegypti	SPECIES	-9.94000	-43.07000	1264874817
4055	Diptera	Culicidae	Aedes	Aedes aegypti	SPECIES	-9.93000	-57.27000	1264874782
4056	Diptera	Culicidae	Aedes	Aedes aegypti	SPECIES	-9.93000	-44.84000	1264874808
4057	Diptera	Culicidae	Aedes	Aedes aegypti	SPECIES	-9.92000	-36.12000	1264874797
4058	Diptera	Culicidae	Aedes	Aedes aegypti	SPECIES	-9.92000	-39.50000	1264874828
4059	Diptera	Culicidae	Aedes	Aedes aegypti	SPECIES	-9.92000	144.05000	1264889100

№	Order	Family	Genus	Species	Taxon Rank	Latitude	Longitude	GBIF_ID
4060	Diptera	Culicidae	Aedes	Aedes aegypti	SPECIES	-9.91000	-37.45000	1264874800
4061	Diptera	Culicidae	Aedes	Aedes aegypti	SPECIES	-9.90000	-48.80000	1264874804
4062	Diptera	Culicidae	Aedes	Aedes aegypti	SPECIES	-9.90000	-36.67000	1264874837
4063	Diptera	Culicidae	Aedes	Aedes aegypti	SPECIES	-9.90000	142.78000	1264888776
4064	Diptera	Culicidae	Aedes	Aedes aegypti	SPECIES	-9.89000	-36.97000	1264874816
4065	Diptera	Culicidae	Aedes	Aedes aegypti	SPECIES	-9.87000	-35.97000	1264874792
4066	Diptera	Culicidae	Aedes	Aedes aegypti	SPECIES	-9.87000	-36.43000	1264874815
4067	Diptera	Culicidae	Aedes	Aedes aegypti	SPECIES	-9.85000	-37.70000	1264874826
4068	Diptera	Culicidae	Aedes	Aedes aegypti	SPECIES	-9.84000	-48.28000	1264874834
4069	Diptera	Culicidae	Aedes	Aedes aegypti	SPECIES	-9.82000	-35.94000	1264874844
4070	Diptera	Culicidae	Aedes	Aedes aegypti	SPECIES	-9.81000	-36.29000	1264874807
4071	Diptera	Culicidae	Aedes	Aedes aegypti	SPECIES	-9.81000	-37.17000	1264874842
4072	Diptera	Culicidae	Aedes	Aedes aegypti	SPECIES	-9.81000	-55.44000	1264874854
4073	Diptera	Culicidae	Aedes	Aedes aegypti	SPECIES	-9.79000	-36.86000	1264874798
4074	Diptera	Culicidae	Aedes	Aedes aegypti	SPECIES	-9.79000	-36.73000	1264874799
4075	Diptera	Culicidae	Aedes	Aedes aegypti	SPECIES	-9.78000	-54.62000	1264874802
4076	Diptera	Culicidae	Aedes	Aedes aegypti	SPECIES	-9.78000	-36.12000	1264874833
4077	Diptera	Culicidae	Aedes	Aedes aegypti	SPECIES	-9.77000	-49.71000	1264874796
4078	Diptera	Culicidae	Aedes	Aedes aegypti	SPECIES	-9.77000	-43.92000	1264874830
4079	Diptera	Culicidae	Aedes	Aedes aegypti	SPECIES	-9.77000	-69.42000	1264874857
4080	Diptera	Culicidae	Aedes	Aedes aegypti	SPECIES	-9.76000	-36.65000	1264874835
4081	Diptera	Culicidae	Aedes	Aedes aegypti	SPECIES	-9.75420	143.41130	177194674
4082	Diptera	Culicidae	Aedes	Aedes aegypti	SPECIES	-9.75000	-36.46000	1264874803
4083	Diptera	Culicidae	Aedes	Aedes aegypti	SPECIES	-9.75000	-36.66000	1264890455
4084	Diptera	Culicidae	Aedes	Aedes aegypti	SPECIES	-9.74000	-37.86000	1264874818
4085	Diptera	Culicidae	Aedes	Aedes aegypti	SPECIES	-9.74000	-38.17000	1264874824
4086	Diptera	Culicidae	Aedes	Aedes aegypti	SPECIES	-9.73874	120.18161	3993826384
4087	Diptera	Culicidae	Aedes	Aedes aegypti	SPECIES	-9.73333	143.41667	1633667717
4088	Diptera	Culicidae	Aedes	Aedes aegypti	SPECIES	-9.72000	-35.91000	1264874809
4089	Diptera	Culicidae	Aedes	Aedes aegypti	SPECIES	-9.72000	-48.60000	1264874853
4090	Diptera	Culicidae	Aedes	Aedes aegypti	SPECIES	-9.70000	-37.11000	1264874820
4091	Diptera	Culicidae	Aedes	Aedes aegypti	SPECIES	-9.70000	-63.42000	1264874823
4092	Diptera	Culicidae	Aedes	Aedes aegypti	SPECIES	-9.70000	-58.12000	1264874840
4093	Diptera	Culicidae	Aedes	Aedes aegypti	SPECIES	-9.69000	-62.77000	1264874836
4094	Diptera	Culicidae	Aedes	Aedes aegypti	SPECIES	-9.69000	-61.55000	1264874865
4095	Diptera	Culicidae	Aedes	Aedes aegypti	SPECIES	-9.69000	-37.31000	1264891084
4096	Diptera	Culicidae	Aedes	Aedes aegypti	SPECIES	-9.67000	-36.32000	1264874843
4097	Diptera	Culicidae	Aedes	Aedes aegypti	SPECIES	-9.67000	-40.84000	1264874863
4098	Diptera	Culicidae	Aedes	Aedes aegypti	SPECIES	-9.67000	-35.74000	1264889543
4099	Diptera	Culicidae	Aedes	Aedes aegypti	SPECIES	-9.67000	-73.43000	1264891123
4100	Diptera	Culicidae	Aedes	Aedes aegypti	SPECIES	-9.66000	-37.23000	1264874822

№	Order	Family	Genus	Species	Taxon Rank	Latitude	Longitude	GBIF_ID
4101	Diptera	Culicidae	Aedes	Aedes aegypti	SPECIES	-9.66000	-37.46000	1264874832
4102	Diptera	Culicidae	Aedes	Aedes aegypti	SPECIES	-9.66000	-36.98000	1264874856
4103	Diptera	Culicidae	Aedes	Aedes aegypti	SPECIES	-9.66000	-36.17000	1264874867
4104	Diptera	Culicidae	Aedes	Aedes aegypti	SPECIES	-9.66000	-35.77000	1264887929
4105	Diptera	Culicidae	Aedes	Aedes aegypti	SPECIES	-9.66000	-35.73000	1264888503
4106	Diptera	Culicidae	Aedes	Aedes aegypti	SPECIES	-9.66000	-35.74000	1264890502
4107	Diptera	Culicidae	Aedes	Aedes aegypti	SPECIES	-9.66000	-36.69000	1264890758
4108	Diptera	Culicidae	Aedes	Aedes aegypti	SPECIES	-9.64000	-36.79000	1264874821
4109	Diptera	Culicidae	Aedes	Aedes aegypti	SPECIES	-9.64000	-67.71000	1264874847
4110	Diptera	Culicidae	Aedes	Aedes aegypti	SPECIES	-9.64000	-47.50000	1264874848
4111	Diptera	Culicidae	Aedes	Aedes aegypti	SPECIES	-9.64000	-35.82000	1264874849
4112	Diptera	Culicidae	Aedes	Aedes aegypti	SPECIES	-9.64000	-49.41000	1264874872
4113	Diptera	Culicidae	Aedes	Aedes aegypti	SPECIES	-9.63000	-42.31000	1264874827
4114	Diptera	Culicidae	Aedes	Aedes aegypti	SPECIES	-9.63000	-36.58000	1264874852
4115	Diptera	Culicidae	Aedes	Aedes aegypti	SPECIES	-9.63000	-36.79000	1264891109
4116	Diptera	Culicidae	Aedes	Aedes aegypti	SPECIES	-9.62000	-48.15000	1264874860
4117	Diptera	Culicidae	Aedes	Aedes aegypti	SPECIES	-9.61000	-35.83000	1264874825
4118	Diptera	Culicidae	Aedes	Aedes aegypti	SPECIES	-9.61000	-36.48000	1264874896
4119	Diptera	Culicidae	Aedes	Aedes aegypti	SPECIES	-9.60000	-68.13000	1264874851
4120	Diptera	Culicidae	Aedes	Aedes aegypti	SPECIES	-9.60000	-36.02000	1264874879
4121	Diptera	Culicidae	Aedes	Aedes aegypti	SPECIES	-9.59000	-37.30000	1264874855
4122	Diptera	Culicidae	Aedes	Aedes aegypti	SPECIES	-9.59000	118.98000	1264874905
4123	Diptera	Culicidae	Aedes	Aedes aegypti	SPECIES	-9.59000	143.76000	1264887782
4124	Diptera	Culicidae	Aedes	Aedes aegypti	SPECIES	-9.58333	143.76666	177194581
4125	Diptera	Culicidae	Aedes	Aedes aegypti	SPECIES	-9.58000	-45.40000	1264874858
4126	Diptera	Culicidae	Aedes	Aedes aegypti	SPECIES	-9.58000	-35.84000	1264874876
4127	Diptera	Culicidae	Aedes	Aedes aegypti	SPECIES	-9.58000	-45.00000	1264874880
4128	Diptera	Culicidae	Aedes	Aedes aegypti	SPECIES	-9.58000	160.15000	1264890020
4129	Diptera	Culicidae	Aedes	Aedes aegypti	SPECIES	-9.57000	-36.69000	1264874838
4130	Diptera	Culicidae	Aedes	Aedes aegypti	SPECIES	-9.57000	-56.69000	1264874859
4131	Diptera	Culicidae	Aedes	Aedes aegypti	SPECIES	-9.57000	-36.42000	1264874862
4132	Diptera	Culicidae	Aedes	Aedes aegypti	SPECIES	-9.55000	-36.29000	1264874850
4133	Diptera	Culicidae	Aedes	Aedes aegypti	SPECIES	-9.55000	-36.49000	1264874885
4134	Diptera	Culicidae	Aedes	Aedes aegypti	SPECIES	-9.54000	-38.26000	1264874866
4135	Diptera	Culicidae	Aedes	Aedes aegypti	SPECIES	-9.54000	-35.69000	1264874878
4136	Diptera	Culicidae	Aedes	Aedes aegypti	SPECIES	-9.53000	-37.49000	1264874875
4137	Diptera	Culicidae	Aedes	Aedes aegypti	SPECIES	-9.53000	-37.25000	1264874891
4138	Diptera	Culicidae	Aedes	Aedes aegypti	SPECIES	-9.52780	-37.29683	3760366555
4139	Diptera	Culicidae	Aedes	Aedes aegypti	SPECIES	-9.52000	-44.43000	1264874899
4140	Diptera	Culicidae	Aedes	Aedes aegypti	SPECIES	-9.52000	-35.71000	1264891090
4141	Diptera	Culicidae	Aedes	Aedes aegypti	SPECIES	-9.51000	-37.00000	1264874841

№	Order	Family	Genus	Species	Taxon Rank	Latitude	Longitude	GBIF_ID
4142	Diptera	Culicidae	Aedes	Aedes aegypti	SPECIES	-9.51000	-37.19000	1264874864
4143	Diptera	Culicidae	Aedes	Aedes aegypti	SPECIES	-9.51000	-46.23000	1264874868
4144	Diptera	Culicidae	Aedes	Aedes aegypti	SPECIES	-9.51000	-37.74000	1264874887
4145	Diptera	Culicidae	Aedes	Aedes aegypti	SPECIES	-9.50000	119.36000	1264874839
4146	Diptera	Culicidae	Aedes	Aedes aegypti	SPECIES	-9.50000	-43.11000	1264874861
4147	Diptera	Culicidae	Aedes	Aedes aegypti	SPECIES	-9.50000	-36.08000	1264874874
4148	Diptera	Culicidae	Aedes	Aedes aegypti	SPECIES	-9.50000	-46.91000	1264874898
4149	Diptera	Culicidae	Aedes	Aedes aegypti	SPECIES	-9.49000	-40.34000	1264874845
4150	Diptera	Culicidae	Aedes	Aedes aegypti	SPECIES	-9.48000	-42.78000	1264874846
4151	Diptera	Culicidae	Aedes	Aedes aegypti	SPECIES	-9.48000	-35.86000	1264874882
4152	Diptera	Culicidae	Aedes	Aedes aegypti	SPECIES	-9.48000	147.19000	1264888036
4153	Diptera	Culicidae	Aedes	Aedes aegypti	SPECIES	-9.47000	-36.28000	1264874869
4154	Diptera	Culicidae	Aedes	Aedes aegypti	SPECIES	-9.47000	-49.30000	1264874870
4155	Diptera	Culicidae	Aedes	Aedes aegypti	SPECIES	-9.47000	-37.36000	1264874881
4156	Diptera	Culicidae	Aedes	Aedes aegypti	SPECIES	-9.47000	-58.67000	1264874884
4157	Diptera	Culicidae	Aedes	Aedes aegypti	SPECIES	-9.47000	-36.40000	1264874903
4158	Diptera	Culicidae	Aedes	Aedes aegypti	SPECIES	-9.46667	147.13330	177195210
4159	Diptera	Culicidae	Aedes	Aedes aegypti	SPECIES	-9.46667	147.14999	177195212
4160	Diptera	Culicidae	Aedes	Aedes aegypti	SPECIES	-9.46667	147.13330	1633667787
4161	Diptera	Culicidae	Aedes	Aedes aegypti	SPECIES	-9.46667	147.14999	1633667794
4162	Diptera	Culicidae	Aedes	Aedes aegypti	SPECIES	-9.45000	147.13330	177195208
4163	Diptera	Culicidae	Aedes	Aedes aegypti	SPECIES	-9.45000	-36.93000	1264874889
4164	Diptera	Culicidae	Aedes	Aedes aegypti	SPECIES	-9.45000	159.97000	3818475993
4165	Diptera	Culicidae	Aedes	Aedes aegypti	SPECIES	-9.44612	159.97300	3442450659
4166	Diptera	Culicidae	Aedes	Aedes aegypti	SPECIES	-9.44342	119.23400	3442480701
4167	Diptera	Culicidae	Aedes	Aedes aegypti	SPECIES	-9.44000	-35.60000	1264874918
4168	Diptera	Culicidae	Aedes	Aedes aegypti	SPECIES	-9.44000	-37.81000	1264874920
4169	Diptera	Culicidae	Aedes	Aedes aegypti	SPECIES	-9.44000	119.23000	3818475995
4170	Diptera	Culicidae	Aedes	Aedes aegypti	SPECIES	-9.44000	147.15000	3818510006
4171	Diptera	Culicidae	Aedes	Aedes aegypti	SPECIES	-9.43000	160.05000	1264890110
4172	Diptera	Culicidae	Aedes	Aedes aegypti	SPECIES	-9.42000	-59.43000	1264874947
4173	Diptera	Culicidae	Aedes	Aedes aegypti	SPECIES	-9.42000	142.53000	1264887704
4174	Diptera	Culicidae	Aedes	Aedes aegypti	SPECIES	-9.42000	179.86000	1264890049
4175	Diptera	Culicidae	Aedes	Aedes aegypti	SPECIES	-9.41000	-36.59000	1264874873
4176	Diptera	Culicidae	Aedes	Aedes aegypti	SPECIES	-9.40000	-48.69000	1264874892
4177	Diptera	Culicidae	Aedes	Aedes aegypti	SPECIES	-9.40000	-38.06000	1264874900
4178	Diptera	Culicidae	Aedes	Aedes aegypti	SPECIES	-9.40000	142.70000	1264887733
4179	Diptera	Culicidae	Aedes	Aedes aegypti	SPECIES	-9.39000	-36.77000	1264874886
4180	Diptera	Culicidae	Aedes	Aedes aegypti	SPECIES	-9.39000	-37.49000	1264874901
4181	Diptera	Culicidae	Aedes	Aedes aegypti	SPECIES	-9.39000	-36.16000	1264874908
4182	Diptera	Culicidae	Aedes	Aedes aegypti	SPECIES	-9.38333	142.66667	1633663703

№	Order	Family	Genus	Species	Taxon Rank	Latitude	Longitude	GBIF_ID
4183	Diptera	Culicidae	Aedes	Aedes aegypti	SPECIES	-9.38333	142.66667	177194577
4184	Diptera	Culicidae	Aedes	Aedes aegypti	SPECIES	-9.38000	-35.58000	1264874871
4185	Diptera	Culicidae	Aedes	Aedes aegypti	SPECIES	-9.38000	-37.06000	1264874931
4186	Diptera	Culicidae	Aedes	Aedes aegypti	SPECIES	-9.37000	-36.41000	1264874883
4187	Diptera	Culicidae	Aedes	Aedes aegypti	SPECIES	-9.37000	-49.84000	1264874912
4188	Diptera	Culicidae	Aedes	Aedes aegypti	SPECIES	-9.37000	-171.22000	1264889669
4189	Diptera	Culicidae	Aedes	Aedes aegypti	SPECIES	-9.35000	-37.25000	1264874888
4190	Diptera	Culicidae	Aedes	Aedes aegypti	SPECIES	-9.34000	-35.82000	1264874877
4191	Diptera	Culicidae	Aedes	Aedes aegypti	SPECIES	-9.34000	-36.09000	1264874907
4192	Diptera	Culicidae	Aedes	Aedes aegypti	SPECIES	-9.34000	-42.47000	1264874911
4193	Diptera	Culicidae	Aedes	Aedes aegypti	SPECIES	-9.34000	-36.28000	1264874916
4194	Diptera	Culicidae	Aedes	Aedes aegypti	SPECIES	-9.33000	-49.08000	1264874921
4195	Diptera	Culicidae	Aedes	Aedes aegypti	SPECIES	-9.33000	-36.87000	1264874923
4196	Diptera	Culicidae	Aedes	Aedes aegypti	SPECIES	-9.32000	142.60000	1264890347
4197	Diptera	Culicidae	Aedes	Aedes aegypti	SPECIES	-9.31000	-36.47000	1264874927
4198	Diptera	Culicidae	Aedes	Aedes aegypti	SPECIES	-9.31000	-70.45000	1264891034
4199	Diptera	Culicidae	Aedes	Aedes aegypti	SPECIES	-9.29000	-37.71000	1264874890
4200	Diptera	Culicidae	Aedes	Aedes aegypti	SPECIES	-9.29000	-42.91000	1264874895
4201	Diptera	Culicidae	Aedes	Aedes aegypti	SPECIES	-9.29000	-37.37000	1264874897
4202	Diptera	Culicidae	Aedes	Aedes aegypti	SPECIES	-9.28333	142.21666	1633663774
4203	Diptera	Culicidae	Aedes	Aedes aegypti	SPECIES	-9.28333	142.21666	177194576
4204	Diptera	Culicidae	Aedes	Aedes aegypti	SPECIES	-9.28000	-35.95000	1264874894
4205	Diptera	Culicidae	Aedes	Aedes aegypti	SPECIES	-9.28000	-50.59000	1264874902
4206	Diptera	Culicidae	Aedes	Aedes aegypti	SPECIES	-9.26000	-35.79000	1264874906
4207	Diptera	Culicidae	Aedes	Aedes aegypti	SPECIES	-9.26000	-44.55000	1264874913
4208	Diptera	Culicidae	Aedes	Aedes aegypti	SPECIES	-9.26000	-37.89000	1264874925
4209	Diptera	Culicidae	Aedes	Aedes aegypti	SPECIES	-9.26000	-38.93000	1264874937
4210	Diptera	Culicidae	Aedes	Aedes aegypti	SPECIES	-9.25000	-38.06000	1264874919
4211	Diptera	Culicidae	Aedes	Aedes aegypti	SPECIES	-9.24000	-43.04000	1264874904
4212	Diptera	Culicidae	Aedes	Aedes aegypti	SPECIES	-9.24000	-35.51000	1264874910
4213	Diptera	Culicidae	Aedes	Aedes aegypti	SPECIES	-9.24000	-35.41000	1264874915
4214	Diptera	Culicidae	Aedes	Aedes aegypti	SPECIES	-9.24000	-37.41000	1264874928
4215	Diptera	Culicidae	Aedes	Aedes aegypti	SPECIES	-9.24000	-41.29000	1264874929
4216	Diptera	Culicidae	Aedes	Aedes aegypti	SPECIES	-9.24000	-48.44000	1264874930
4217	Diptera	Culicidae	Aedes	Aedes aegypti	SPECIES	-9.24000	-36.27000	1264874936
4218	Diptera	Culicidae	Aedes	Aedes aegypti	SPECIES	-9.24000	-38.63000	1264874943
4219	Diptera	Culicidae	Aedes	Aedes aegypti	SPECIES	-9.24000	-35.62000	1264874957
4220	Diptera	Culicidae	Aedes	Aedes aegypti	SPECIES	-9.23000	-62.17000	1264889147
4221	Diptera	Culicidae	Aedes	Aedes aegypti	SPECIES	-9.22000	-38.20000	1264874944
4222	Diptera	Culicidae	Aedes	Aedes aegypti	SPECIES	-9.21000	-36.03000	1264874946
4223	Diptera	Culicidae	Aedes	Aedes aegypti	SPECIES	-9.20000	-48.02000	1264874974

№	Order	Family	Genus	Species	Taxon Rank	Latitude	Longitude	GBIF_ID
4224	Diptera	Culicidae	Aedes	Aedes aegypti	SPECIES	-9.20000	-171.85000	1264889697
4225	Diptera	Culicidae	Aedes	Aedes aegypti	SPECIES	-9.19000	-39.67000	1264874909
4226	Diptera	Culicidae	Aedes	Aedes aegypti	SPECIES	-9.19000	-39.20000	1264874914
4227	Diptera	Culicidae	Aedes	Aedes aegypti	SPECIES	-9.19000	-42.90000	1264874922
4228	Diptera	Culicidae	Aedes	Aedes aegypti	SPECIES	-9.19000	-38.40000	1264874948
4229	Diptera	Culicidae	Aedes	Aedes aegypti	SPECIES	-9.19000	-62.01000	1264874951
4230	Diptera	Culicidae	Aedes	Aedes aegypti	SPECIES	-9.18000	-42.51000	1264874933
4231	Diptera	Culicidae	Aedes	Aedes aegypti	SPECIES	-9.18000	-36.69000	1264874950
4232	Diptera	Culicidae	Aedes	Aedes aegypti	SPECIES	-9.17000	-36.47000	1264874940
4233	Diptera	Culicidae	Aedes	Aedes aegypti	SPECIES	-9.15000	-35.41000	1264874939
4234	Diptera	Culicidae	Aedes	Aedes aegypti	SPECIES	-9.14000	-36.91000	1264874959
4235	Diptera	Culicidae	Aedes	Aedes aegypti	SPECIES	-9.13000	-37.36000	1264874917
4236	Diptera	Culicidae	Aedes	Aedes aegypti	SPECIES	-9.13000	-62.69000	1264874926
4237	Diptera	Culicidae	Aedes	Aedes aegypti	SPECIES	-9.13000	-37.55000	1264874935
4238	Diptera	Culicidae	Aedes	Aedes aegypti	SPECIES	-9.13000	-63.08000	1264874962
4239	Diptera	Culicidae	Aedes	Aedes aegypti	SPECIES	-9.12000	-36.33000	1264874934
4240	Diptera	Culicidae	Aedes	Aedes aegypti	SPECIES	-9.12000	-36.02000	1264874953
4241	Diptera	Culicidae	Aedes	Aedes aegypti	SPECIES	-9.12000	-36.20000	1264874972
4242	Diptera	Culicidae	Aedes	Aedes aegypti	SPECIES	-9.11000	-35.57000	1264874924
4243	Diptera	Culicidae	Aedes	Aedes aegypti	SPECIES	-9.10000	-37.03000	1264874932
4244	Diptera	Culicidae	Aedes	Aedes aegypti	SPECIES	-9.09000	-35.29000	1264874941
4245	Diptera	Culicidae	Aedes	Aedes aegypti	SPECIES	-9.09000	-48.55000	1264874965
4246	Diptera	Culicidae	Aedes	Aedes aegypti	SPECIES	-9.08000	117.01000	1264871678
4247	Diptera	Culicidae	Aedes	Aedes aegypti	SPECIES	-9.08000	-43.69000	1264874954
4248	Diptera	Culicidae	Aedes	Aedes aegypti	SPECIES	-9.08000	-43.36000	1264874960
4249	Diptera	Culicidae	Aedes	Aedes aegypti	SPECIES	-9.08000	-47.47000	1264874981
4250	Diptera	Culicidae	Aedes	Aedes aegypti	SPECIES	-9.07000	-35.73000	1264874967
4251	Diptera	Culicidae	Aedes	Aedes aegypti	SPECIES	-9.07000	-36.62000	1264874999
4252	Diptera	Culicidae	Aedes	Aedes aegypti	SPECIES	-9.06000	-44.11000	1264874938
4253	Diptera	Culicidae	Aedes	Aedes aegypti	SPECIES	-9.06000	-43.15000	1264874976
4254	Diptera	Culicidae	Aedes	Aedes aegypti	SPECIES	-9.05000	-40.58000	1264874945
4255	Diptera	Culicidae	Aedes	Aedes aegypti	SPECIES	-9.04000	-37.74000	1264874971
4256	Diptera	Culicidae	Aedes	Aedes aegypti	SPECIES	-9.03000	116.83000	1264871660
4257	Diptera	Culicidae	Aedes	Aedes aegypti	SPECIES	-9.03000	-42.65000	1264874958
4258	Diptera	Culicidae	Aedes	Aedes aegypti	SPECIES	-9.03000	-35.43000	1264874964
4259	Diptera	Culicidae	Aedes	Aedes aegypti	SPECIES	-9.03000	-37.31000	1264874975
4260	Diptera	Culicidae	Aedes	Aedes aegypti	SPECIES	-9.03000	-36.54000	1264874985
4261	Diptera	Culicidae	Aedes	Aedes aegypti	SPECIES	-9.00000	-36.75000	1264874949
4262	Diptera	Culicidae	Aedes	Aedes aegypti	SPECIES	-9.00000	-47.86000	1264874966
4263	Diptera	Culicidae	Aedes	Aedes aegypti	SPECIES	-8.99000	-36.31000	1264874961
4264	Diptera	Culicidae	Aedes	Aedes aegypti	SPECIES	-8.99000	-36.05000	1264874979

№	Order	Family	Genus	Species	Taxon Rank	Latitude	Longitude	GBIF_ID
4265	Diptera	Culicidae	Aedes	Aedes aegypti	SPECIES	-8.98000	-64.52000	1264874942
4266	Diptera	Culicidae	Aedes	Aedes aegypti	SPECIES	-8.97000	-35.26000	1264874955
4267	Diptera	Culicidae	Aedes	Aedes aegypti	SPECIES	-8.97000	-45.69000	1264874988
4268	Diptera	Culicidae	Aedes	Aedes aegypti	SPECIES	-8.97000	36.30000	1264890209
4269	Diptera	Culicidae	Aedes	Aedes aegypti	SPECIES	-8.96000	116.81000	1264871696
4270	Diptera	Culicidae	Aedes	Aedes aegypti	SPECIES	-8.96000	-42.28000	1264874952
4271	Diptera	Culicidae	Aedes	Aedes aegypti	SPECIES	-8.96000	-35.52000	1264874956
4272	Diptera	Culicidae	Aedes	Aedes aegypti	SPECIES	-8.96000	-63.08000	1264874982
4273	Diptera	Culicidae	Aedes	Aedes aegypti	SPECIES	-8.96000	-35.91000	1264874983
4274	Diptera	Culicidae	Aedes	Aedes aegypti	SPECIES	-8.95000	-35.64000	1264874998
4275	Diptera	Culicidae	Aedes	Aedes aegypti	SPECIES	-8.95000	-139.50000	1264891295
4276	Diptera	Culicidae	Aedes	Aedes aegypti	SPECIES	-8.94000	-36.50000	1264874969
4277	Diptera	Culicidae	Aedes	Aedes aegypti	SPECIES	-8.94000	-48.25000	1264874989
4278	Diptera	Culicidae	Aedes	Aedes aegypti	SPECIES	-8.94000	-35.74000	1264875005
4279	Diptera	Culicidae	Aedes	Aedes aegypti	SPECIES	-8.92000	116.75000	1264871687
4280	Diptera	Culicidae	Aedes	Aedes aegypti	SPECIES	-8.92000	-41.82000	1264874990
4281	Diptera	Culicidae	Aedes	Aedes aegypti	SPECIES	-8.92000	-42.98000	1264875002
4282	Diptera	Culicidae	Aedes	Aedes aegypti	SPECIES	-8.92000	-49.01000	1264875024
4283	Diptera	Culicidae	Aedes	Aedes aegypti	SPECIES	-8.91000	-38.03000	1264874963
4284	Diptera	Culicidae	Aedes	Aedes aegypti	SPECIES	-8.91000	-38.29000	1264874970
4285	Diptera	Culicidae	Aedes	Aedes aegypti	SPECIES	-8.91000	-49.54000	1264875000
4286	Diptera	Culicidae	Aedes	Aedes aegypti	SPECIES	-8.90000	-37.56000	1264874986
4287	Diptera	Culicidae	Aedes	Aedes aegypti	SPECIES	-8.90000	-70.81000	1264874996
4288	Diptera	Culicidae	Aedes	Aedes aegypti	SPECIES	-8.89000	-48.79000	1264874973
4289	Diptera	Culicidae	Aedes	Aedes aegypti	SPECIES	-8.89000	-36.73000	1264874991
4290	Diptera	Culicidae	Aedes	Aedes aegypti	SPECIES	-8.89000	-35.54000	1264874992
4291	Diptera	Culicidae	Aedes	Aedes aegypti	SPECIES	-8.89000	-35.45000	1264875009
4292	Diptera	Culicidae	Aedes	Aedes aegypti	SPECIES	-8.88000	-36.38000	1264874968
4293	Diptera	Culicidae	Aedes	Aedes aegypti	SPECIES	-8.88000	-36.28000	1264874978
4294	Diptera	Culicidae	Aedes	Aedes aegypti	SPECIES	-8.87000	-35.20000	1264874984
4295	Diptera	Culicidae	Aedes	Aedes aegypti	SPECIES	-8.87000	-36.17000	1264875013
4296	Diptera	Culicidae	Aedes	Aedes aegypti	SPECIES	-8.85000	-39.28000	1264875007
4297	Diptera	Culicidae	Aedes	Aedes aegypti	SPECIES	-8.84000	-35.65000	1264874977
4298	Diptera	Culicidae	Aedes	Aedes aegypti	SPECIES	-8.83000	-35.76000	1264875004
4299	Diptera	Culicidae	Aedes	Aedes aegypti	SPECIES	-8.83000	-36.04000	1264875011
4300	Diptera	Culicidae	Aedes	Aedes aegypti	SPECIES	-8.82000	-68.14000	1264874980
4301	Diptera	Culicidae	Aedes	Aedes aegypti	SPECIES	-8.82000	-36.68000	1264874995
4302	Diptera	Culicidae	Aedes	Aedes aegypti	SPECIES	-8.82000	-35.26000	1264875010
4303	Diptera	Culicidae	Aedes	Aedes aegypti	SPECIES	-8.82000	-35.91000	1264875031
4304	Diptera	Culicidae	Aedes	Aedes aegypti	SPECIES	-8.81000	-37.79000	1264875023
4305	Diptera	Culicidae	Aedes	Aedes aegypti	SPECIES	-8.81000	13.23000	3987197821

№	Order	Family	Genus	Species	Taxon Rank	Latitude	Longitude	GBIF_ID
4306	Diptera	Culicidae	Aedes	Aedes aegypti	SPECIES	-8.80000	-47.80000	1264874994
4307	Diptera	Culicidae	Aedes	Aedes aegypti	SPECIES	-8.80000	-43.99000	1264875017
4308	Diptera	Culicidae	Aedes	Aedes aegypti	SPECIES	-8.80000	-40.23000	1264875044
4309	Diptera	Culicidae	Aedes	Aedes aegypti	SPECIES	-8.77000	-48.42000	1264874987
4310	Diptera	Culicidae	Aedes	Aedes aegypti	SPECIES	-8.77000	-38.74000	1264875028
4311	Diptera	Culicidae	Aedes	Aedes aegypti	SPECIES	-8.76000	-63.91000	1264890472
4312	Diptera	Culicidae	Aedes	Aedes aegypti	SPECIES	-8.75000	-36.15000	1264874997
4313	Diptera	Culicidae	Aedes	Aedes aegypti	SPECIES	-8.75000	-35.22000	1264875025
4314	Diptera	Culicidae	Aedes	Aedes aegypti	SPECIES	-8.75000	-63.91000	1264888116
4315	Diptera	Culicidae	Aedes	Aedes aegypti	SPECIES	-8.74000	-35.80000	1264875008
4316	Diptera	Culicidae	Aedes	Aedes aegypti	SPECIES	-8.74000	115.17000	3818510855
4317	Diptera	Culicidae	Aedes	Aedes aegypti	SPECIES	-8.73000	-44.69000	1264875015
4318	Diptera	Culicidae	Aedes	Aedes aegypti	SPECIES	-8.73000	-36.32000	1264875020
4319	Diptera	Culicidae	Aedes	Aedes aegypti	SPECIES	-8.73000	-36.46000	1264875036
4320	Diptera	Culicidae	Aedes	Aedes aegypti	SPECIES	-8.72000	-36.39000	1264875016
4321	Diptera	Culicidae	Aedes	Aedes aegypti	SPECIES	-8.72000	115.19000	1264876414
4322	Diptera	Culicidae	Aedes	Aedes aegypti	SPECIES	-8.70000	115.22000	1264871727
4323	Diptera	Culicidae	Aedes	Aedes aegypti	SPECIES	-8.70000	-47.13000	1264874993
4324	Diptera	Culicidae	Aedes	Aedes aegypti	SPECIES	-8.70000	-39.91000	1264875039
4325	Diptera	Culicidae	Aedes	Aedes aegypti	SPECIES	-8.69000	-35.73000	1264875003
4326	Diptera	Culicidae	Aedes	Aedes aegypti	SPECIES	-8.69000	-37.14000	1264875012
4327	Diptera	Culicidae	Aedes	Aedes aegypti	SPECIES	-8.69000	115.22000	1264876438
4328	Diptera	Culicidae	Aedes	Aedes aegypti	SPECIES	-8.68000	-35.89000	1264875001
4329	Diptera	Culicidae	Aedes	Aedes aegypti	SPECIES	-8.68000	-35.48000	1264875029
4330	Diptera	Culicidae	Aedes	Aedes aegypti	SPECIES	-8.68000	-36.59000	1264875033
4331	Diptera	Culicidae	Aedes	Aedes aegypti	SPECIES	-8.68000	115.21000	1264876410
4332	Diptera	Culicidae	Aedes	Aedes aegypti	SPECIES	-8.67000	116.11000	1264871621
4333	Diptera	Culicidae	Aedes	Aedes aegypti	SPECIES	-8.67000	-36.29000	1264875006
4334	Diptera	Culicidae	Aedes	Aedes aegypti	SPECIES	-8.67000	-37.35000	1264875038
4335	Diptera	Culicidae	Aedes	Aedes aegypti	SPECIES	-8.66000	-36.92000	1264875021
4336	Diptera	Culicidae	Aedes	Aedes aegypti	SPECIES	-8.66000	-35.23000	1264875047
4337	Diptera	Culicidae	Aedes	Aedes aegypti	SPECIES	-8.66000	-36.06000	1264875056
4338	Diptera	Culicidae	Aedes	Aedes aegypti	SPECIES	-8.66000	-35.63000	1264875070
4339	Diptera	Culicidae	Aedes	Aedes aegypti	SPECIES	-8.66000	115.19000	1264876381
4340	Diptera	Culicidae	Aedes	Aedes aegypti	SPECIES	-8.66000	115.20000	1264876440
4341	Diptera	Culicidae	Aedes	Aedes aegypti	SPECIES	-8.65000	115.22000	1264876456
4342	Diptera	Culicidae	Aedes	Aedes aegypti	SPECIES	-8.64000	-50.26000	1264875034
4343	Diptera	Culicidae	Aedes	Aedes aegypti	SPECIES	-8.64000	115.19000	1264876390
4344	Diptera	Culicidae	Aedes	Aedes aegypti	SPECIES	-8.63000	-36.19000	1264875046
4345	Diptera	Culicidae	Aedes	Aedes aegypti	SPECIES	-8.62000	-35.38000	1264875019
4346	Diptera	Culicidae	Aedes	Aedes aegypti	SPECIES	-8.62000	-38.24000	1264875051

№	Order	Family	Genus	Species	Taxon Rank	Latitude	Longitude	GBIF_ID
4347	Diptera	Culicidae	Aedes	Aedes aegypti	SPECIES	-8.62000	116.11000	1264876363
4348	Diptera	Culicidae	Aedes	Aedes aegypti	SPECIES	-8.61000	116.12000	1264876388
4349	Diptera	Culicidae	Aedes	Aedes aegypti	SPECIES	-8.60000	-35.81000	1264875014
4350	Diptera	Culicidae	Aedes	Aedes aegypti	SPECIES	-8.60000	-41.06000	1264875050
4351	Diptera	Culicidae	Aedes	Aedes aegypti	SPECIES	-8.60000	117.27000	1264891261
4352	Diptera	Culicidae	Aedes	Aedes aegypti	SPECIES	-8.59000	116.11000	1264876411
4353	Diptera	Culicidae	Aedes	Aedes aegypti	SPECIES	-8.58000	-35.92000	1264875022
4354	Diptera	Culicidae	Aedes	Aedes aegypti	SPECIES	-8.58000	-57.80000	1264875032
4355	Diptera	Culicidae	Aedes	Aedes aegypti	SPECIES	-8.58000	-36.80000	1264875042
4356	Diptera	Culicidae	Aedes	Aedes aegypti	SPECIES	-8.58000	-35.16000	1264875043
4357	Diptera	Culicidae	Aedes	Aedes aegypti	SPECIES	-8.58000	-37.63000	1264875060
4358	Diptera	Culicidae	Aedes	Aedes aegypti	SPECIES	-8.57000	115.34000	1264871759
4359	Diptera	Culicidae	Aedes	Aedes aegypti	SPECIES	-8.57000	-38.95000	1264875018
4360	Diptera	Culicidae	Aedes	Aedes aegypti	SPECIES	-8.57000	-35.54000	1264875037
4361	Diptera	Culicidae	Aedes	Aedes aegypti	SPECIES	-8.56000	-172.50000	1264889681
4362	Diptera	Culicidae	Aedes	Aedes aegypti	SPECIES	-8.55000	-42.45000	1264875035
4363	Diptera	Culicidae	Aedes	Aedes aegypti	SPECIES	-8.55000	-41.57000	1264875062
4364	Diptera	Culicidae	Aedes	Aedes aegypti	SPECIES	-8.54000	115.32000	1264871761
4365	Diptera	Culicidae	Aedes	Aedes aegypti	SPECIES	-8.54000	-36.46000	1264875059
4366	Diptera	Culicidae	Aedes	Aedes aegypti	SPECIES	-8.53000	-44.43000	1264875026
4367	Diptera	Culicidae	Aedes	Aedes aegypti	SPECIES	-8.53000	-41.93000	1264875027
4368	Diptera	Culicidae	Aedes	Aedes aegypti	SPECIES	-8.52000	140.42000	1264871587
4369	Diptera	Culicidae	Aedes	Aedes aegypti	SPECIES	-8.52000	-35.39000	1264875054
4370	Diptera	Culicidae	Aedes	Aedes aegypti	SPECIES	-8.52000	-45.19000	1264875065
4371	Diptera	Culicidae	Aedes	Aedes aegypti	SPECIES	-8.51000	-36.76000	1264875030
4372	Diptera	Culicidae	Aedes	Aedes aegypti	SPECIES	-8.51000	-51.21000	1264875048
4373	Diptera	Culicidae	Aedes	Aedes aegypti	SPECIES	-8.51000	-41.35000	1264875073
4374	Diptera	Culicidae	Aedes	Aedes aegypti	SPECIES	-8.51000	179.19000	1264890050
4375	Diptera	Culicidae	Aedes	Aedes aegypti	SPECIES	-8.51000	120.59000	1264891249
4376	Diptera	Culicidae	Aedes	Aedes aegypti	SPECIES	-8.50000	-47.64000	1264875057
4377	Diptera	Culicidae	Aedes	Aedes aegypti	SPECIES	-8.50000	-36.29000	1264875080
4378	Diptera	Culicidae	Aedes	Aedes aegypti	SPECIES	-8.49000	-35.69000	1264875069
4379	Diptera	Culicidae	Aedes	Aedes aegypti	SPECIES	-8.49000	-43.06000	1264875076
4380	Diptera	Culicidae	Aedes	Aedes aegypti	SPECIES	-8.48000	140.39000	1264871596
4381	Diptera	Culicidae	Aedes	Aedes aegypti	SPECIES	-8.48000	-36.12000	1264875040
4382	Diptera	Culicidae	Aedes	Aedes aegypti	SPECIES	-8.48000	-35.86000	1264875045
4383	Diptera	Culicidae	Aedes	Aedes aegypti	SPECIES	-8.48000	-40.61000	1264875093
4384	Diptera	Culicidae	Aedes	Aedes aegypti	SPECIES	-8.47000	-48.47000	1264875074
4385	Diptera	Culicidae	Aedes	Aedes aegypti	SPECIES	-8.46000	-35.10000	1264875058
4386	Diptera	Culicidae	Aedes	Aedes aegypti	SPECIES	-8.46000	-35.95000	1264875068
4387	Diptera	Culicidae	Aedes	Aedes aegypti	SPECIES	-8.45000	-39.58000	1264875049

№	Order	Family	Genus	Species	Taxon Rank	Latitude	Longitude	GBIF_ID
4388	Diptera	Culicidae	Aedes	Aedes aegypti	SPECIES	-8.45000	115.08000	1264891256
4389	Diptera	Culicidae	Aedes	Aedes aegypti	SPECIES	-8.44000	-44.02000	1264875066
4390	Diptera	Culicidae	Aedes	Aedes aegypti	SPECIES	-8.44000	-48.77000	1264875067
4391	Diptera	Culicidae	Aedes	Aedes aegypti	SPECIES	-8.44000	-49.18000	1264875084
4392	Diptera	Culicidae	Aedes	Aedes aegypti	SPECIES	-8.43000	-36.65000	1264875041
4393	Diptera	Culicidae	Aedes	Aedes aegypti	SPECIES	-8.43000	-38.73000	1264875087
4394	Diptera	Culicidae	Aedes	Aedes aegypti	SPECIES	-8.42000	-35.52000	1264875053
4395	Diptera	Culicidae	Aedes	Aedes aegypti	SPECIES	-8.42000	-35.62000	1264875061
4396	Diptera	Culicidae	Aedes	Aedes aegypti	SPECIES	-8.40000	-48.91000	1264875085
4397	Diptera	Culicidae	Aedes	Aedes aegypti	SPECIES	-8.39000	-39.31000	1264875052
4398	Diptera	Culicidae	Aedes	Aedes aegypti	SPECIES	-8.39000	-74.58000	1264887633
4399	Diptera	Culicidae	Aedes	Aedes aegypti	SPECIES	-8.38000	-37.02000	1264875079
4400	Diptera	Culicidae	Aedes	Aedes aegypti	SPECIES	-8.38000	-65.92000	1264875091
4401	Diptera	Culicidae	Aedes	Aedes aegypti	SPECIES	-8.37000	-42.82000	1264875055
4402	Diptera	Culicidae	Aedes	Aedes aegypti	SPECIES	-8.37000	-35.25000	1264875075
4403	Diptera	Culicidae	Aedes	Aedes aegypti	SPECIES	-8.37000	-36.56000	1264875082
4404	Diptera	Culicidae	Aedes	Aedes aegypti	SPECIES	-8.37000	115.13000	1264891116
4405	Diptera	Culicidae	Aedes	Aedes aegypti	SPECIES	-8.36000	-48.22000	1264875096
4406	Diptera	Culicidae	Aedes	Aedes aegypti	SPECIES	-8.36000	-35.47000	1264875097
4407	Diptera	Culicidae	Aedes	Aedes aegypti	SPECIES	-8.34000	-36.15000	1264875071
4408	Diptera	Culicidae	Aedes	Aedes aegypti	SPECIES	-8.34000	-35.38000	1264875099
4409	Diptera	Culicidae	Aedes	Aedes aegypti	SPECIES	-8.34000	-35.78000	1264875107
4410	Diptera	Culicidae	Aedes	Aedes aegypti	SPECIES	-8.34000	-36.27000	1264875117
4411	Diptera	Culicidae	Aedes	Aedes aegypti	SPECIES	-8.34000	122.97000	1264888176
4412	Diptera	Culicidae	Aedes	Aedes aegypti	SPECIES	-8.33000	-42.27000	1264875063
4413	Diptera	Culicidae	Aedes	Aedes aegypti	SPECIES	-8.33000	-40.99000	1264875083
4414	Diptera	Culicidae	Aedes	Aedes aegypti	SPECIES	-8.33000	-43.28000	1264875094
4415	Diptera	Culicidae	Aedes	Aedes aegypti	SPECIES	-8.33000	123.25000	1264888157
4416	Diptera	Culicidae	Aedes	Aedes aegypti	SPECIES	-8.31000	-45.90000	1264875100
4417	Diptera	Culicidae	Aedes	Aedes aegypti	SPECIES	-8.31000	114.61000	1264876436
4418	Diptera	Culicidae	Aedes	Aedes aegypti	SPECIES	-8.30000	-48.02000	1264875072
4419	Diptera	Culicidae	Aedes	Aedes aegypti	SPECIES	-8.30000	-35.69000	1264875090
4420	Diptera	Culicidae	Aedes	Aedes aegypti	SPECIES	-8.29732	158.49259	1838182782
4421	Diptera	Culicidae	Aedes	Aedes aegypti	SPECIES	-8.29000	-46.37000	1264875092
4422	Diptera	Culicidae	Aedes	Aedes aegypti	SPECIES	-8.29000	-40.27000	1264875109
4423	Diptera	Culicidae	Aedes	Aedes aegypti	SPECIES	-8.28000	-35.09000	1264875064
4424	Diptera	Culicidae	Aedes	Aedes aegypti	SPECIES	-8.27000	-36.41000	1264875078
4425	Diptera	Culicidae	Aedes	Aedes aegypti	SPECIES	-8.27000	-48.46000	1264875115
4426	Diptera	Culicidae	Aedes	Aedes aegypti	SPECIES	-8.27000	113.54000	1264876383
4427	Diptera	Culicidae	Aedes	Aedes aegypti	SPECIES	-8.26000	-38.00000	1264875086
4428	Diptera	Culicidae	Aedes	Aedes aegypti	SPECIES	-8.24000	-35.78000	1264875077

№	Order	Family	Genus	Species	Taxon Rank	Latitude	Longitude	GBIF_ID
4429	Diptera	Culicidae	Aedes	Aedes aegypti	SPECIES	-8.24000	-35.47000	1264875108
4430	Diptera	Culicidae	Aedes	Aedes aegypti	SPECIES	-8.24000	-41.81000	1264875110
4431	Diptera	Culicidae	Aedes	Aedes aegypti	SPECIES	-8.22000	-35.55000	1264875116
4432	Diptera	Culicidae	Aedes	Aedes aegypti	SPECIES	-8.21000	-46.82000	1264875081
4433	Diptera	Culicidae	Aedes	Aedes aegypti	SPECIES	-8.21000	-40.60000	1264875101
4434	Diptera	Culicidae	Aedes	Aedes aegypti	SPECIES	-8.21000	-49.53000	1264875106
4435	Diptera	Culicidae	Aedes	Aedes aegypti	SPECIES	-8.21000	-36.71000	1264875122
4436	Diptera	Culicidae	Aedes	Aedes aegypti	SPECIES	-8.21000	113.67000	1264876408
4437	Diptera	Culicidae	Aedes	Aedes aegypti	SPECIES	-8.21000	111.10000	1264876433
4438	Diptera	Culicidae	Aedes	Aedes aegypti	SPECIES	-8.20000	-37.32000	1264875132
4439	Diptera	Culicidae	Aedes	Aedes aegypti	SPECIES	-8.19789	-35.55644	4129714961
4440	Diptera	Culicidae	Aedes	Aedes aegypti	SPECIES	-8.19000	-41.58000	1264875095
4441	Diptera	Culicidae	Aedes	Aedes aegypti	SPECIES	-8.19000	-36.02000	1264875121
4442	Diptera	Culicidae	Aedes	Aedes aegypti	SPECIES	-8.19000	-44.09000	1264875124
4443	Diptera	Culicidae	Aedes	Aedes aegypti	SPECIES	-8.19000	-35.41000	1264875142
4444	Diptera	Culicidae	Aedes	Aedes aegypti	SPECIES	-8.16000	-35.31000	1264875088
4445	Diptera	Culicidae	Aedes	Aedes aegypti	SPECIES	-8.16000	-38.73000	1264875105
4446	Diptera	Culicidae	Aedes	Aedes aegypti	SPECIES	-8.16000	-39.41000	1264875112
4447	Diptera	Culicidae	Aedes	Aedes aegypti	SPECIES	-8.16000	-39.81000	1264875119
4448	Diptera	Culicidae	Aedes	Aedes aegypti	SPECIES	-8.16000	-37.02000	1264875125
4449	Diptera	Culicidae	Aedes	Aedes aegypti	SPECIES	-8.16000	-39.77000	1264890829
4450	Diptera	Culicidae	Aedes	Aedes aegypti	SPECIES	-8.15000	-37.70000	1264875089
4451	Diptera	Culicidae	Aedes	Aedes aegypti	SPECIES	-8.15000	-35.00000	1264875098
4452	Diptera	Culicidae	Aedes	Aedes aegypti	SPECIES	-8.15000	-35.14000	1264875118
4453	Diptera	Culicidae	Aedes	Aedes aegypti	SPECIES	-8.15000	-43.67000	1264875134
4454	Diptera	Culicidae	Aedes	Aedes aegypti	SPECIES	-8.13000	-49.10000	1264875113
4455	Diptera	Culicidae	Aedes	Aedes aegypti	SPECIES	-8.13000	-40.77000	1264875137
4456	Diptera	Culicidae	Aedes	Aedes aegypti	SPECIES	-8.12000	-36.77000	1264875102
4457	Diptera	Culicidae	Aedes	Aedes aegypti	SPECIES	-8.12000	-41.09000	1264875104
4458	Diptera	Culicidae	Aedes	Aedes aegypti	SPECIES	-8.12000	-42.01000	1264875141
4459	Diptera	Culicidae	Aedes	Aedes aegypti	SPECIES	-8.12000	-34.90000	1264887900
4460	Diptera	Culicidae	Aedes	Aedes aegypti	SPECIES	-8.12000	-35.01000	1264887918
4461	Diptera	Culicidae	Aedes	Aedes aegypti	SPECIES	-8.11000	-79.02000	1264887636
4462	Diptera	Culicidae	Aedes	Aedes aegypti	SPECIES	-8.11000	-35.01000	1264890486
4463	Diptera	Culicidae	Aedes	Aedes aegypti	SPECIES	-8.10966	-79.03580	3496615736
4464	Diptera	Culicidae	Aedes	Aedes aegypti	SPECIES	-8.10958	-79.03581	3496613630
4465	Diptera	Culicidae	Aedes	Aedes aegypti	SPECIES	-8.10664	-79.03434	3496608539
4466	Diptera	Culicidae	Aedes	Aedes aegypti	SPECIES	-8.10655	-79.03483	4052769967
4467	Diptera	Culicidae	Aedes	Aedes aegypti	SPECIES	-8.10653	-79.03473	3496606378
4468	Diptera	Culicidae	Aedes	Aedes aegypti	SPECIES	-8.10653	-79.03471	3496607566
4469	Diptera	Culicidae	Aedes	Aedes aegypti	SPECIES	-8.10651	-79.03471	3496615362

№	Order	Family	Genus	Species	Taxon Rank	Latitude	Longitude	GBIF_ID
4470	Diptera	Culicidae	Aedes	Aedes aegypti	SPECIES	-8.10650	-79.03459	3496613026
4471	Diptera	Culicidae	Aedes	Aedes aegypti	SPECIES	-8.10647	-79.03456	3496611831
4472	Diptera	Culicidae	Aedes	Aedes aegypti	SPECIES	-8.10646	-79.03467	3496609813
4473	Diptera	Culicidae	Aedes	Aedes aegypti	SPECIES	-8.10646	-79.03464	3496612410
4474	Diptera	Culicidae	Aedes	Aedes aegypti	SPECIES	-8.10642	-79.03461	3496612205
4475	Diptera	Culicidae	Aedes	Aedes aegypti	SPECIES	-8.09434	-34.93841	4022153750
4476	Diptera	Culicidae	Aedes	Aedes aegypti	SPECIES	-8.09000	-35.87000	1264875133
4477	Diptera	Culicidae	Aedes	Aedes aegypti	SPECIES	-8.09000	-42.28000	1264875135
4478	Diptera	Culicidae	Aedes	Aedes aegypti	SPECIES	-8.09000	-34.92000	1264890527
4479	Diptera	Culicidae	Aedes	Aedes aegypti	SPECIES	-8.08000	-36.28000	1264875103
4480	Diptera	Culicidae	Aedes	Aedes aegypti	SPECIES	-8.08000	-38.99000	1264875126
4481	Diptera	Culicidae	Aedes	Aedes aegypti	SPECIES	-8.08000	-37.09000	1264875146
4482	Diptera	Culicidae	Aedes	Aedes aegypti	SPECIES	-8.08000	-34.88000	1264890424
4483	Diptera	Culicidae	Aedes	Aedes aegypti	SPECIES	-8.07000	-48.52000	1264875131
4484	Diptera	Culicidae	Aedes	Aedes aegypti	SPECIES	-8.07000	-34.95000	1264887951
4485	Diptera	Culicidae	Aedes	Aedes aegypti	SPECIES	-8.06180	-34.87125	4063057570
4486	Diptera	Culicidae	Aedes	Aedes aegypti	SPECIES	-8.06000	-50.22000	1264875147
4487	Diptera	Culicidae	Aedes	Aedes aegypti	SPECIES	-8.06000	-45.44000	1264875150
4488	Diptera	Culicidae	Aedes	Aedes aegypti	SPECIES	-8.06000	-38.33000	1264875159
4489	Diptera	Culicidae	Aedes	Aedes aegypti	SPECIES	-8.06000	-36.53000	1264875166
4490	Diptera	Culicidae	Aedes	Aedes aegypti	SPECIES	-8.06000	-34.90000	1264887933
4491	Diptera	Culicidae	Aedes	Aedes aegypti	SPECIES	-8.06000	-34.94000	1264890419
4492	Diptera	Culicidae	Aedes	Aedes aegypti	SPECIES	-8.05000	112.82000	1264871881
4493	Diptera	Culicidae	Aedes	Aedes aegypti	SPECIES	-8.05000	-34.88000	1264888517
4494	Diptera	Culicidae	Aedes	Aedes aegypti	SPECIES	-8.04000	-34.94000	1264875111
4495	Diptera	Culicidae	Aedes	Aedes aegypti	SPECIES	-8.04000	111.92000	1264876336
4496	Diptera	Culicidae	Aedes	Aedes aegypti	SPECIES	-8.03000	-35.72000	1264875120
4497	Diptera	Culicidae	Aedes	Aedes aegypti	SPECIES	-8.03000	-34.92000	1264890421
4498	Diptera	Culicidae	Aedes	Aedes aegypti	SPECIES	-8.02000	-38.10000	1264875129
4499	Diptera	Culicidae	Aedes	Aedes aegypti	SPECIES	-8.02000	-35.11000	1264875139
4500	Diptera	Culicidae	Aedes	Aedes aegypti	SPECIES	-8.02000	-47.48000	1264875143
4501	Diptera	Culicidae	Aedes	Aedes aegypti	SPECIES	-8.02000	-34.96000	1264890427
4502	Diptera	Culicidae	Aedes	Aedes aegypti	SPECIES	-8.02000	-34.91000	1264890447
4503	Diptera	Culicidae	Aedes	Aedes aegypti	SPECIES	-8.01829	139.56873	3993824513
4504	Diptera	Culicidae	Aedes	Aedes aegypti	SPECIES	-8.01629	-34.95091	4022197642
4505	Diptera	Culicidae	Aedes	Aedes aegypti	SPECIES	-8.01000	112.66000	1264871877
4506	Diptera	Culicidae	Aedes	Aedes aegypti	SPECIES	-8.01000	-35.21000	1264875145
4507	Diptera	Culicidae	Aedes	Aedes aegypti	SPECIES	-8.01000	-35.55000	1264875149
4508	Diptera	Culicidae	Aedes	Aedes aegypti	SPECIES	-8.01000	-35.35000	1264875158
4509	Diptera	Culicidae	Aedes	Aedes aegypti	SPECIES	-8.01000	-34.85000	1264888492
4510	Diptera	Culicidae	Aedes	Aedes aegypti	SPECIES	-8.01000	-34.95000	1264890439

№	Order	Family	Genus	Species	Taxon Rank	Latitude	Longitude	GBIF_ID
4511	Diptera	Culicidae	Aedes	Aedes aegypti	SPECIES	-8.00000	-34.87000	1264875114
4512	Diptera	Culicidae	Aedes	Aedes aegypti	SPECIES	-8.00000	-36.05000	1264875123
4513	Diptera	Culicidae	Aedes	Aedes aegypti	SPECIES	-8.00000	-41.86000	1264875162
4514	Diptera	Culicidae	Aedes	Aedes aegypti	SPECIES	-8.00000	158.00000	1633667658
4515	Diptera	Culicidae	Aedes	Aedes aegypti	SPECIES	-7.99000	-48.98000	1264875130
4516	Diptera	Culicidae	Aedes	Aedes aegypti	SPECIES	-7.99000	-48.67000	1264875136
4517	Diptera	Culicidae	Aedes	Aedes aegypti	SPECIES	-7.98000	112.63000	1264871917
4518	Diptera	Culicidae	Aedes	Aedes aegypti	SPECIES	-7.98000	-35.00000	1264875127
4519	Diptera	Culicidae	Aedes	Aedes aegypti	SPECIES	-7.98000	113.82000	1264891037
4520	Diptera	Culicidae	Aedes	Aedes aegypti	SPECIES	-7.97483	-34.99197	3892391329
4521	Diptera	Culicidae	Aedes	Aedes aegypti	SPECIES	-7.97482	-34.99196	3090761913
4522	Diptera	Culicidae	Aedes	Aedes aegypti	SPECIES	-7.97000	112.66000	1264871786
4523	Diptera	Culicidae	Aedes	Aedes aegypti	SPECIES	-7.96000	-72.79000	1264875160
4524	Diptera	Culicidae	Aedes	Aedes aegypti	SPECIES	-7.96000	110.63000	1264876397
4525	Diptera	Culicidae	Aedes	Aedes aegypti	SPECIES	-7.95000	-35.38000	1264875128
4526	Diptera	Culicidae	Aedes	Aedes aegypti	SPECIES	-7.95000	-40.21000	1264875157
4527	Diptera	Culicidae	Aedes	Aedes aegypti	SPECIES	-7.95000	-38.99000	1264875167
4528	Diptera	Culicidae	Aedes	Aedes aegypti	SPECIES	-7.95000	-78.34000	1264891089
4529	Diptera	Culicidae	Aedes	Aedes aegypti	SPECIES	-7.94000	-43.65000	1264875151
4530	Diptera	Culicidae	Aedes	Aedes aegypti	SPECIES	-7.94000	-42.61000	1264875171
4531	Diptera	Culicidae	Aedes	Aedes aegypti	SPECIES	-7.93710	-34.83242	3457038694
4532	Diptera	Culicidae	Aedes	Aedes aegypti	SPECIES	-7.93000	-35.89000	1264875156
4533	Diptera	Culicidae	Aedes	Aedes aegypti	SPECIES	-7.93000	-37.90000	1264875183
4534	Diptera	Culicidae	Aedes	Aedes aegypti	SPECIES	-7.93000	110.35000	1264891392
4535	Diptera	Culicidae	Aedes	Aedes aegypti	SPECIES	-7.92000	110.32000	1264871638
4536	Diptera	Culicidae	Aedes	Aedes aegypti	SPECIES	-7.92000	110.31000	1264871648
4537	Diptera	Culicidae	Aedes	Aedes aegypti	SPECIES	-7.92000	110.34000	1264871870
4538	Diptera	Culicidae	Aedes	Aedes aegypti	SPECIES	-7.91000	110.30000	1264871605
4539	Diptera	Culicidae	Aedes	Aedes aegypti	SPECIES	-7.91000	110.31000	1264871606
4540	Diptera	Culicidae	Aedes	Aedes aegypti	SPECIES	-7.91000	-41.25000	1264875138
4541	Diptera	Culicidae	Aedes	Aedes aegypti	SPECIES	-7.91000	-41.56000	1264875144
4542	Diptera	Culicidae	Aedes	Aedes aegypti	SPECIES	-7.91000	-43.32000	1264875154
4543	Diptera	Culicidae	Aedes	Aedes aegypti	SPECIES	-7.91000	-36.77000	1264875163
4544	Diptera	Culicidae	Aedes	Aedes aegypti	SPECIES	-7.91000	-55.43000	1264875168
4545	Diptera	Culicidae	Aedes	Aedes aegypti	SPECIES	-7.91000	-34.85000	1264875170
4546	Diptera	Culicidae	Aedes	Aedes aegypti	SPECIES	-7.91000	-35.60000	1264875175
4547	Diptera	Culicidae	Aedes	Aedes aegypti	SPECIES	-7.91000	-35.30000	1264875189
4548	Diptera	Culicidae	Aedes	Aedes aegypti	SPECIES	-7.90000	110.32000	1264871603
4549	Diptera	Culicidae	Aedes	Aedes aegypti	SPECIES	-7.90000	110.34000	1264871609
4550	Diptera	Culicidae	Aedes	Aedes aegypti	SPECIES	-7.90000	110.33000	1264871630
4551	Diptera	Culicidae	Aedes	Aedes aegypti	SPECIES	-7.90000	110.30000	1264888982

№	Order	Family	Genus	Species	Taxon Rank	Latitude	Longitude	GBIF_ID
4552	Diptera	Culicidae	Aedes	Aedes aegypti	SPECIES	-7.89000	-35.14000	1264875140
4553	Diptera	Culicidae	Aedes	Aedes aegypti	SPECIES	-7.89000	-36.31000	1264875148
4554	Diptera	Culicidae	Aedes	Aedes aegypti	SPECIES	-7.89000	-35.99000	1264875172
4555	Diptera	Culicidae	Aedes	Aedes aegypti	SPECIES	-7.89000	-35.75000	1264875174
4556	Diptera	Culicidae	Aedes	Aedes aegypti	SPECIES	-7.89000	-37.14000	1264875181
4557	Diptera	Culicidae	Aedes	Aedes aegypti	SPECIES	-7.88000	-42.47000	1264875152
4558	Diptera	Culicidae	Aedes	Aedes aegypti	SPECIES	-7.88000	-42.86000	1264875155
4559	Diptera	Culicidae	Aedes	Aedes aegypti	SPECIES	-7.88000	-38.76000	1264875161
4560	Diptera	Culicidae	Aedes	Aedes aegypti	SPECIES	-7.88000	-36.13000	1264875185
4561	Diptera	Culicidae	Aedes	Aedes aegypti	SPECIES	-7.88000	110.33000	1264890670
4562	Diptera	Culicidae	Aedes	Aedes aegypti	SPECIES	-7.88000	110.42000	1264891270
4563	Diptera	Culicidae	Aedes	Aedes aegypti	SPECIES	-7.87000	-35.08000	1264875184
4564	Diptera	Culicidae	Aedes	Aedes aegypti	SPECIES	-7.87000	111.47000	1264876361
4565	Diptera	Culicidae	Aedes	Aedes aegypti	SPECIES	-7.86000	110.36000	1264871864
4566	Diptera	Culicidae	Aedes	Aedes aegypti	SPECIES	-7.86000	-35.60000	1264875186
4567	Diptera	Culicidae	Aedes	Aedes aegypti	SPECIES	-7.86000	-35.34000	1264875194
4568	Diptera	Culicidae	Aedes	Aedes aegypti	SPECIES	-7.85000	-37.40000	1264875153
4569	Diptera	Culicidae	Aedes	Aedes aegypti	SPECIES	-7.85000	-48.15000	1264875192
4570	Diptera	Culicidae	Aedes	Aedes aegypti	SPECIES	-7.84000	112.45000	1264871871
4571	Diptera	Culicidae	Aedes	Aedes aegypti	SPECIES	-7.84000	-38.18000	1264875176
4572	Diptera	Culicidae	Aedes	Aedes aegypti	SPECIES	-7.84000	-38.07000	1264875180
4573	Diptera	Culicidae	Aedes	Aedes aegypti	SPECIES	-7.84000	-42.29000	1264875200
4574	Diptera	Culicidae	Aedes	Aedes aegypti	SPECIES	-7.84000	-35.44000	1264875209
4575	Diptera	Culicidae	Aedes	Aedes aegypti	SPECIES	-7.83000	-35.31000	1264875165
4576	Diptera	Culicidae	Aedes	Aedes aegypti	SPECIES	-7.83000	-39.35000	1264875195
4577	Diptera	Culicidae	Aedes	Aedes aegypti	SPECIES	-7.83000	110.42000	1264876386
4578	Diptera	Culicidae	Aedes	Aedes aegypti	SPECIES	-7.83000	110.39000	1264887540
4579	Diptera	Culicidae	Aedes	Aedes aegypti	SPECIES	-7.82000	-35.88000	1264875169
4580	Diptera	Culicidae	Aedes	Aedes aegypti	SPECIES	-7.82000	110.16000	1264876394
4581	Diptera	Culicidae	Aedes	Aedes aegypti	SPECIES	-7.81000	110.40000	1264871718
4582	Diptera	Culicidae	Aedes	Aedes aegypti	SPECIES	-7.81000	110.39000	1264871842
4583	Diptera	Culicidae	Aedes	Aedes aegypti	SPECIES	-7.81000	-36.65000	1264875219
4584	Diptera	Culicidae	Aedes	Aedes aegypti	SPECIES	-7.81000	110.38000	1264887586
4585	Diptera	Culicidae	Aedes	Aedes aegypti	SPECIES	-7.80000	110.36000	1264871832
4586	Diptera	Culicidae	Aedes	Aedes aegypti	SPECIES	-7.80000	110.41000	1264871852
4587	Diptera	Culicidae	Aedes	Aedes aegypti	SPECIES	-7.80000	110.34000	1264871875
4588	Diptera	Culicidae	Aedes	Aedes aegypti	SPECIES	-7.80000	-37.73000	1264875182
4589	Diptera	Culicidae	Aedes	Aedes aegypti	SPECIES	-7.80000	-34.97000	1264875188
4590	Diptera	Culicidae	Aedes	Aedes aegypti	SPECIES	-7.80000	-35.08000	1264875190
4591	Diptera	Culicidae	Aedes	Aedes aegypti	SPECIES	-7.80000	110.94000	1264876424
4592	Diptera	Culicidae	Aedes	Aedes aegypti	SPECIES	-7.80000	110.37000	1264887638

№	Order	Family	Genus	Species	Taxon Rank	Latitude	Longitude	GBIF_ID
4593	Diptera	Culicidae	Aedes	Aedes aegypti	SPECIES	-7.79946	110.38028	2517945440
4594	Diptera	Culicidae	Aedes	Aedes aegypti	SPECIES	-7.79000	-43.16000	1264875173
4595	Diptera	Culicidae	Aedes	Aedes aegypti	SPECIES	-7.79000	-44.56000	1264875197
4596	Diptera	Culicidae	Aedes	Aedes aegypti	SPECIES	-7.79000	-39.69000	1264875198
4597	Diptera	Culicidae	Aedes	Aedes aegypti	SPECIES	-7.79000	-41.98000	1264875203
4598	Diptera	Culicidae	Aedes	Aedes aegypti	SPECIES	-7.79000	110.38000	1264876373
4599	Diptera	Culicidae	Aedes	Aedes aegypti	SPECIES	-7.79000	110.34000	1264876377
4600	Diptera	Culicidae	Aedes	Aedes aegypti	SPECIES	-7.79000	110.35000	1264876385
4601	Diptera	Culicidae	Aedes	Aedes aegypti	SPECIES	-7.79000	110.36000	1264876412
4602	Diptera	Culicidae	Aedes	Aedes aegypti	SPECIES	-7.79000	110.15000	1264876448
4603	Diptera	Culicidae	Aedes	Aedes aegypti	SPECIES	-7.79000	110.37000	1264887541
4604	Diptera	Culicidae	Aedes	Aedes aegypti	SPECIES	-7.79000	110.39000	1264887578
4605	Diptera	Culicidae	Aedes	Aedes aegypti	SPECIES	-7.78000	-35.82000	1264875164
4606	Diptera	Culicidae	Aedes	Aedes aegypti	SPECIES	-7.78000	-36.51000	1264875212
4607	Diptera	Culicidae	Aedes	Aedes aegypti	SPECIES	-7.78000	110.35000	1264887572
4608	Diptera	Culicidae	Aedes	Aedes aegypti	SPECIES	-7.78000	110.38000	1264888744
4609	Diptera	Culicidae	Aedes	Aedes aegypti	SPECIES	-7.78000	110.75000	1264891254
4610	Diptera	Culicidae	Aedes	Aedes aegypti	SPECIES	-7.77000	110.41000	1264871844
4611	Diptera	Culicidae	Aedes	Aedes aegypti	SPECIES	-7.77000	-35.57000	1264875177
4612	Diptera	Culicidae	Aedes	Aedes aegypti	SPECIES	-7.77000	-44.01000	1264875179
4613	Diptera	Culicidae	Aedes	Aedes aegypti	SPECIES	-7.77000	-39.05000	1264875187
4614	Diptera	Culicidae	Aedes	Aedes aegypti	SPECIES	-7.76000	110.45000	1264871853
4615	Diptera	Culicidae	Aedes	Aedes aegypti	SPECIES	-7.76000	-35.71000	1264875207
4616	Diptera	Culicidae	Aedes	Aedes aegypti	SPECIES	-7.75000	-35.17000	1264875178
4617	Diptera	Culicidae	Aedes	Aedes aegypti	SPECIES	-7.75000	-34.90000	1264875204
4618	Diptera	Culicidae	Aedes	Aedes aegypti	SPECIES	-7.75000	-46.69000	1264875205
4619	Diptera	Culicidae	Aedes	Aedes aegypti	SPECIES	-7.75000	-37.62000	1264875211
4620	Diptera	Culicidae	Aedes	Aedes aegypti	SPECIES	-7.75000	-43.85000	1264875218
4621	Diptera	Culicidae	Aedes	Aedes aegypti	SPECIES	-7.75000	-34.86000	1264875220
4622	Diptera	Culicidae	Aedes	Aedes aegypti	SPECIES	-7.74000	108.94000	1264871733
4623	Diptera	Culicidae	Aedes	Aedes aegypti	SPECIES	-7.74000	110.48000	1264871856
4624	Diptera	Culicidae	Aedes	Aedes aegypti	SPECIES	-7.74000	-40.30000	1264875234
4625	Diptera	Culicidae	Aedes	Aedes aegypti	SPECIES	-7.74000	110.41000	1264876409
4626	Diptera	Culicidae	Aedes	Aedes aegypti	SPECIES	-7.73000	-49.04000	1264875191
4627	Diptera	Culicidae	Aedes	Aedes aegypti	SPECIES	-7.73000	-35.35000	1264875196
4628	Diptera	Culicidae	Aedes	Aedes aegypti	SPECIES	-7.73000	-39.00000	1264875208
4629	Diptera	Culicidae	Aedes	Aedes aegypti	SPECIES	-7.73000	-50.14000	1264875213
4630	Diptera	Culicidae	Aedes	Aedes aegypti	SPECIES	-7.73000	-35.93000	1264875215
4631	Diptera	Culicidae	Aedes	Aedes aegypti	SPECIES	-7.73000	-35.20000	1264875225
4632	Diptera	Culicidae	Aedes	Aedes aegypti	SPECIES	-7.73000	-39.19000	1264875246
4633	Diptera	Culicidae	Aedes	Aedes aegypti	SPECIES	-7.72000	109.74000	1264871736

№	Order	Family	Genus	Species	Taxon Rank	Latitude	Longitude	GBIF_ID
4634	Diptera	Culicidae	Aedes	Aedes aegypti	SPECIES	-7.72000	110.34000	1264871833
4635	Diptera	Culicidae	Aedes	Aedes aegypti	SPECIES	-7.71000	-37.42000	1264875199
4636	Diptera	Culicidae	Aedes	Aedes aegypti	SPECIES	-7.71000	-37.86000	1264875214
4637	Diptera	Culicidae	Aedes	Aedes aegypti	SPECIES	-7.71000	-40.95000	1264875222
4638	Diptera	Culicidae	Aedes	Aedes aegypti	SPECIES	-7.71000	-35.60000	1264875224
4639	Diptera	Culicidae	Aedes	Aedes aegypti	SPECIES	-7.71000	-47.58000	1264875227
4640	Diptera	Culicidae	Aedes	Aedes aegypti	SPECIES	-7.70000	-40.77000	1264875193
4641	Diptera	Culicidae	Aedes	Aedes aegypti	SPECIES	-7.70000	-38.09000	1264875201
4642	Diptera	Culicidae	Aedes	Aedes aegypti	SPECIES	-7.70000	-38.21000	1264875210
4643	Diptera	Culicidae	Aedes	Aedes aegypti	SPECIES	-7.70000	-37.26000	1264875230
4644	Diptera	Culicidae	Aedes	Aedes aegypti	SPECIES	-7.70000	-35.50000	1264875237
4645	Diptera	Culicidae	Aedes	Aedes aegypti	SPECIES	-7.69000	-36.05000	1264875202
4646	Diptera	Culicidae	Aedes	Aedes aegypti	SPECIES	-7.69000	-37.09000	1264875240
4647	Diptera	Culicidae	Aedes	Aedes aegypti	SPECIES	-7.69000	108.65000	1264876434
4648	Diptera	Culicidae	Aedes	Aedes aegypti	SPECIES	-7.68000	109.67000	1264871642
4649	Diptera	Culicidae	Aedes	Aedes aegypti	SPECIES	-7.68000	-39.96000	1264875206
4650	Diptera	Culicidae	Aedes	Aedes aegypti	SPECIES	-7.68000	-35.04000	1264875229
4651	Diptera	Culicidae	Aedes	Aedes aegypti	SPECIES	-7.68000	-41.88000	1264875231
4652	Diptera	Culicidae	Aedes	Aedes aegypti	SPECIES	-7.68000	-36.13000	1264875235
4653	Diptera	Culicidae	Aedes	Aedes aegypti	SPECIES	-7.68000	-36.29000	1264875243
4654	Diptera	Culicidae	Aedes	Aedes aegypti	SPECIES	-7.68000	-35.73000	1264875258
4655	Diptera	Culicidae	Aedes	Aedes aegypti	SPECIES	-7.68000	110.53000	1264888998
4656	Diptera	Culicidae	Aedes	Aedes aegypti	SPECIES	-7.68000	110.35000	1264891372
4657	Diptera	Culicidae	Aedes	Aedes aegypti	SPECIES	-7.67000	109.66000	1264871652
4658	Diptera	Culicidae	Aedes	Aedes aegypti	SPECIES	-7.67000	-35.36000	1264875247
4659	Diptera	Culicidae	Aedes	Aedes aegypti	SPECIES	-7.67000	-38.58000	1264875250
4660	Diptera	Culicidae	Aedes	Aedes aegypti	SPECIES	-7.66000	110.52000	1264871659
4661	Diptera	Culicidae	Aedes	Aedes aegypti	SPECIES	-7.66000	109.67000	1264871667
4662	Diptera	Culicidae	Aedes	Aedes aegypti	SPECIES	-7.66000	109.66000	1264871671
4663	Diptera	Culicidae	Aedes	Aedes aegypti	SPECIES	-7.66000	-36.91000	1264875216
4664	Diptera	Culicidae	Aedes	Aedes aegypti	SPECIES	-7.66000	-41.27000	1264875217
4665	Diptera	Culicidae	Aedes	Aedes aegypti	SPECIES	-7.66000	111.53000	1264876367
4666	Diptera	Culicidae	Aedes	Aedes aegypti	SPECIES	-7.65000	-38.00000	1264875233
4667	Diptera	Culicidae	Aedes	Aedes aegypti	SPECIES	-7.65000	-42.87000	1264875238
4668	Diptera	Culicidae	Aedes	Aedes aegypti	SPECIES	-7.65000	-36.63000	1264875239
4669	Diptera	Culicidae	Aedes	Aedes aegypti	SPECIES	-7.65000	-48.37000	1264875269
4670	Diptera	Culicidae	Aedes	Aedes aegypti	SPECIES	-7.65000	111.33000	1264876395
4671	Diptera	Culicidae	Aedes	Aedes aegypti	SPECIES	-7.64000	109.63000	1264871960
4672	Diptera	Culicidae	Aedes	Aedes aegypti	SPECIES	-7.63000	-38.31000	1264875248
4673	Diptera	Culicidae	Aedes	Aedes aegypti	SPECIES	-7.63000	-40.50000	1264875252
4674	Diptera	Culicidae	Aedes	Aedes aegypti	SPECIES	-7.62000	112.38000	1264871891

№	Order	Family	Genus	Species	Taxon Rank	Latitude	Longitude	GBIF_ID
4675	Diptera	Culicidae	Aedes	Aedes aegypti	SPECIES	-7.62000	-41.68000	1264875223
4676	Diptera	Culicidae	Aedes	Aedes aegypti	SPECIES	-7.61000	-39.50000	1264875221
4677	Diptera	Culicidae	Aedes	Aedes aegypti	SPECIES	-7.61000	-35.19000	1264875232
4678	Diptera	Culicidae	Aedes	Aedes aegypti	SPECIES	-7.61000	-41.52000	1264875245
4679	Diptera	Culicidae	Aedes	Aedes aegypti	SPECIES	-7.61000	-37.14000	1264875263
4680	Diptera	Culicidae	Aedes	Aedes aegypti	SPECIES	-7.60000	108.94000	1264871747
4681	Diptera	Culicidae	Aedes	Aedes aegypti	SPECIES	-7.60000	-35.09000	1264875226
4682	Diptera	Culicidae	Aedes	Aedes aegypti	SPECIES	-7.60000	-35.49000	1264875254
4683	Diptera	Culicidae	Aedes	Aedes aegypti	SPECIES	-7.60000	111.45000	1264876329
4684	Diptera	Culicidae	Aedes	Aedes aegypti	SPECIES	-7.60000	110.95000	1264876418
4685	Diptera	Culicidae	Aedes	Aedes aegypti	SPECIES	-7.59000	-42.33000	1264875228
4686	Diptera	Culicidae	Aedes	Aedes aegypti	SPECIES	-7.59000	-37.86000	1264875236
4687	Diptera	Culicidae	Aedes	Aedes aegypti	SPECIES	-7.59000	-34.94000	1264875251
4688	Diptera	Culicidae	Aedes	Aedes aegypti	SPECIES	-7.59000	-39.22000	1264875259
4689	Diptera	Culicidae	Aedes	Aedes aegypti	SPECIES	-7.59000	-59.42000	1264875265
4690	Diptera	Culicidae	Aedes	Aedes aegypti	SPECIES	-7.59000	-38.93000	1264875268
4691	Diptera	Culicidae	Aedes	Aedes aegypti	SPECIES	-7.58000	-36.39000	1264875262
4692	Diptera	Culicidae	Aedes	Aedes aegypti	SPECIES	-7.58000	-37.52000	1264875271
4693	Diptera	Culicidae	Aedes	Aedes aegypti	SPECIES	-7.58000	-37.65000	1264875278
4694	Diptera	Culicidae	Aedes	Aedes aegypti	SPECIES	-7.58000	-43.54000	1264875282
4695	Diptera	Culicidae	Aedes	Aedes aegypti	SPECIES	-7.58000	112.16000	1264876354
4696	Diptera	Culicidae	Aedes	Aedes aegypti	SPECIES	-7.57000	110.79000	1264871837
4697	Diptera	Culicidae	Aedes	Aedes aegypti	SPECIES	-7.57000	-35.97000	1264875241
4698	Diptera	Culicidae	Aedes	Aedes aegypti	SPECIES	-7.56000	110.83000	1264871827
4699	Diptera	Culicidae	Aedes	Aedes aegypti	SPECIES	-7.56000	110.82000	1264871847
4700	Diptera	Culicidae	Aedes	Aedes aegypti	SPECIES	-7.56000	110.86000	1264871848
4701	Diptera	Culicidae	Aedes	Aedes aegypti	SPECIES	-7.56000	-37.04000	1264875242
4702	Diptera	Culicidae	Aedes	Aedes aegypti	SPECIES	-7.56000	-36.72000	1264875264
4703	Diptera	Culicidae	Aedes	Aedes aegypti	SPECIES	-7.56000	-35.80000	1264875296
4704	Diptera	Culicidae	Aedes	Aedes aegypti	SPECIES	-7.56000	110.88000	1264876378
4705	Diptera	Culicidae	Aedes	Aedes aegypti	SPECIES	-7.55000	-39.11000	1264875249
4706	Diptera	Culicidae	Aedes	Aedes aegypti	SPECIES	-7.55000	-49.52000	1264875257
4707	Diptera	Culicidae	Aedes	Aedes aegypti	SPECIES	-7.55000	-48.95000	1264875260
4708	Diptera	Culicidae	Aedes	Aedes aegypti	SPECIES	-7.55000	-35.71000	1264875272
4709	Diptera	Culicidae	Aedes	Aedes aegypti	SPECIES	-7.55000	-35.57000	1264875279
4710	Diptera	Culicidae	Aedes	Aedes aegypti	SPECIES	-7.54000	-37.24000	1264875244
4711	Diptera	Culicidae	Aedes	Aedes aegypti	SPECIES	-7.54000	-38.17000	1264875273
4712	Diptera	Culicidae	Aedes	Aedes aegypti	SPECIES	-7.54000	110.21000	1264876351
4713	Diptera	Culicidae	Aedes	Aedes aegypti	SPECIES	-7.53000	-41.09000	1264875253
4714	Diptera	Culicidae	Aedes	Aedes aegypti	SPECIES	-7.53000	-38.54000	1264875255
4715	Diptera	Culicidae	Aedes	Aedes aegypti	SPECIES	-7.53000	-35.45000	1264875287

№	Order	Family	Genus	Species	Taxon Rank	Latitude	Longitude	GBIF_ID
4716	Diptera	Culicidae	Aedes	Aedes aegypti	SPECIES	-7.52000	-42.58000	1264875256
4717	Diptera	Culicidae	Aedes	Aedes aegypti	SPECIES	-7.52000	-35.32000	1264875276
4718	Diptera	Culicidae	Aedes	Aedes aegypti	SPECIES	-7.52000	-39.73000	1264875284
4719	Diptera	Culicidae	Aedes	Aedes aegypti	SPECIES	-7.51000	-37.81000	1264875261
4720	Diptera	Culicidae	Aedes	Aedes aegypti	SPECIES	-7.51000	-62.64000	1264875274
4721	Diptera	Culicidae	Aedes	Aedes aegypti	SPECIES	-7.51000	-47.79000	1264875290
4722	Diptera	Culicidae	Aedes	Aedes aegypti	SPECIES	-7.51000	-38.40000	1264875291
4723	Diptera	Culicidae	Aedes	Aedes aegypti	SPECIES	-7.51000	-63.03000	1264890406
4724	Diptera	Culicidae	Aedes	Aedes aegypti	SPECIES	-7.50000	-36.14000	1264875298
4725	Diptera	Culicidae	Aedes	Aedes aegypti	SPECIES	-7.50000	-40.24000	1264875304
4726	Diptera	Culicidae	Aedes	Aedes aegypti	SPECIES	-7.49000	-37.66000	1264875281
4727	Diptera	Culicidae	Aedes	Aedes aegypti	SPECIES	-7.49000	-36.14000	1264888056
4728	Diptera	Culicidae	Aedes	Aedes aegypti	SPECIES	-7.49000	178.68000	1264890062
4729	Diptera	Culicidae	Aedes	Aedes aegypti	SPECIES	-7.48000	110.23000	1264871830
4730	Diptera	Culicidae	Aedes	Aedes aegypti	SPECIES	-7.48000	-50.67000	1264875266
4731	Diptera	Culicidae	Aedes	Aedes aegypti	SPECIES	-7.48000	-34.92000	1264875267
4732	Diptera	Culicidae	Aedes	Aedes aegypti	SPECIES	-7.48000	-43.15000	1264875303
4733	Diptera	Culicidae	Aedes	Aedes aegypti	SPECIES	-7.48000	-36.48000	1264875313
4734	Diptera	Culicidae	Aedes	Aedes aegypti	SPECIES	-7.48000	112.41000	1264876376
4735	Diptera	Culicidae	Aedes	Aedes aegypti	SPECIES	-7.48000	-36.13000	1264888051
4736	Diptera	Culicidae	Aedes	Aedes aegypti	SPECIES	-7.47000	110.23000	1264871824
4737	Diptera	Culicidae	Aedes	Aedes aegypti	SPECIES	-7.47000	110.22000	1264871865
4738	Diptera	Culicidae	Aedes	Aedes aegypti	SPECIES	-7.47000	-35.24000	1264875289
4739	Diptera	Culicidae	Aedes	Aedes aegypti	SPECIES	-7.46000	109.44000	1264871939
4740	Diptera	Culicidae	Aedes	Aedes aegypti	SPECIES	-7.46000	-52.09000	1264875277
4741	Diptera	Culicidae	Aedes	Aedes aegypti	SPECIES	-7.46000	-38.02000	1264875286
4742	Diptera	Culicidae	Aedes	Aedes aegypti	SPECIES	-7.46000	-38.09000	1264875292
4743	Diptera	Culicidae	Aedes	Aedes aegypti	SPECIES	-7.46000	-42.02000	1264875299
4744	Diptera	Culicidae	Aedes	Aedes aegypti	SPECIES	-7.46000	-35.16000	1264875305
4745	Diptera	Culicidae	Aedes	Aedes aegypti	SPECIES	-7.46000	112.75000	1264876401
4746	Diptera	Culicidae	Aedes	Aedes aegypti	SPECIES	-7.45000	109.23000	1264871841
4747	Diptera	Culicidae	Aedes	Aedes aegypti	SPECIES	-7.45000	-51.34000	1264875270
4748	Diptera	Culicidae	Aedes	Aedes aegypti	SPECIES	-7.45000	-37.44000	1264875275
4749	Diptera	Culicidae	Aedes	Aedes aegypti	SPECIES	-7.45000	-34.83000	1264875283
4750	Diptera	Culicidae	Aedes	Aedes aegypti	SPECIES	-7.45000	-47.24000	1264875295
4751	Diptera	Culicidae	Aedes	Aedes aegypti	SPECIES	-7.45000	-38.32000	1264875302
4752	Diptera	Culicidae	Aedes	Aedes aegypti	SPECIES	-7.45000	-36.28000	1264875317
4753	Diptera	Culicidae	Aedes	Aedes aegypti	SPECIES	-7.45000	112.74000	1264876427
4754	Diptera	Culicidae	Aedes	Aedes aegypti	SPECIES	-7.45000	110.75000	1264889023
4755	Diptera	Culicidae	Aedes	Aedes aegypti	SPECIES	-7.44000	-35.29000	1264875280
4756	Diptera	Culicidae	Aedes	Aedes aegypti	SPECIES	-7.44000	-41.80000	1264875307

№	Order	Family	Genus	Species	Taxon Rank	Latitude	Longitude	GBIF_ID
4757	Diptera	Culicidae	Aedes	Aedes aegypti	SPECIES	-7.44000	109.19000	1264876420
4758	Diptera	Culicidae	Aedes	Aedes aegypti	SPECIES	-7.43000	109.24000	1264871874
4759	Diptera	Culicidae	Aedes	Aedes aegypti	SPECIES	-7.43000	-38.18000	1264875312
4760	Diptera	Culicidae	Aedes	Aedes aegypti	SPECIES	-7.42000	109.23000	1264871860
4761	Diptera	Culicidae	Aedes	Aedes aegypti	SPECIES	-7.42000	-39.33000	1264875285
4762	Diptera	Culicidae	Aedes	Aedes aegypti	SPECIES	-7.42000	-38.71000	1264875297
4763	Diptera	Culicidae	Aedes	Aedes aegypti	SPECIES	-7.42000	-36.06000	1264875311
4764	Diptera	Culicidae	Aedes	Aedes aegypti	SPECIES	-7.42000	-40.74000	1264875326
4765	Diptera	Culicidae	Aedes	Aedes aegypti	SPECIES	-7.41000	-41.48000	1264875293
4766	Diptera	Culicidae	Aedes	Aedes aegypti	SPECIES	-7.41000	-41.19000	1264875306
4767	Diptera	Culicidae	Aedes	Aedes aegypti	SPECIES	-7.41000	-35.64000	1264875325
4768	Diptera	Culicidae	Aedes	Aedes aegypti	SPECIES	-7.40000	110.49000	1264871771
4769	Diptera	Culicidae	Aedes	Aedes aegypti	SPECIES	-7.40000	109.74000	1264871902
4770	Diptera	Culicidae	Aedes	Aedes aegypti	SPECIES	-7.40000	109.69000	1264871919
4771	Diptera	Culicidae	Aedes	Aedes aegypti	SPECIES	-7.40000	-36.85000	1264875288
4772	Diptera	Culicidae	Aedes	Aedes aegypti	SPECIES	-7.40000	-35.92000	1264875301
4773	Diptera	Culicidae	Aedes	Aedes aegypti	SPECIES	-7.40000	-45.55000	1264875323
4774	Diptera	Culicidae	Aedes	Aedes aegypti	SPECIES	-7.40000	-37.56000	1264875324
4775	Diptera	Culicidae	Aedes	Aedes aegypti	SPECIES	-7.40000	-35.47000	1264875334
4776	Diptera	Culicidae	Aedes	Aedes aegypti	SPECIES	-7.40000	-35.77000	1264875335
4777	Diptera	Culicidae	Aedes	Aedes aegypti	SPECIES	-7.39000	109.71000	1264871928
4778	Diptera	Culicidae	Aedes	Aedes aegypti	SPECIES	-7.39000	109.60000	1264871929
4779	Diptera	Culicidae	Aedes	Aedes aegypti	SPECIES	-7.39000	-40.98000	1264875294
4780	Diptera	Culicidae	Aedes	Aedes aegypti	SPECIES	-7.39000	-37.97000	1264875315
4781	Diptera	Culicidae	Aedes	Aedes aegypti	SPECIES	-7.39000	-37.13000	1264875330
4782	Diptera	Culicidae	Aedes	Aedes aegypti	SPECIES	-7.38000	-40.88000	1264875329
4783	Diptera	Culicidae	Aedes	Aedes aegypti	SPECIES	-7.37000	109.63000	1264871779
4784	Diptera	Culicidae	Aedes	Aedes aegypti	SPECIES	-7.37000	-50.10000	1264875320
4785	Diptera	Culicidae	Aedes	Aedes aegypti	SPECIES	-7.36000	-35.07000	1264875336
4786	Diptera	Culicidae	Aedes	Aedes aegypti	SPECIES	-7.36000	109.92000	1264876435
4787	Diptera	Culicidae	Aedes	Aedes aegypti	SPECIES	-7.35057	108.21716	3903145059
4788	Diptera	Culicidae	Aedes	Aedes aegypti	SPECIES	-7.35000	110.50000	1264871713
4789	Diptera	Culicidae	Aedes	Aedes aegypti	SPECIES	-7.35000	-35.35000	1264875300
4790	Diptera	Culicidae	Aedes	Aedes aegypti	SPECIES	-7.35000	-35.22000	1264875310
4791	Diptera	Culicidae	Aedes	Aedes aegypti	SPECIES	-7.35000	-34.95000	1264875319
4792	Diptera	Culicidae	Aedes	Aedes aegypti	SPECIES	-7.35000	112.75000	1264876415
4793	Diptera	Culicidae	Aedes	Aedes aegypti	SPECIES	-7.35000	109.98000	1264876425
4794	Diptera	Culicidae	Aedes	Aedes aegypti	SPECIES	-7.34000	110.50000	1264871711
4795	Diptera	Culicidae	Aedes	Aedes aegypti	SPECIES	-7.34000	-40.80000	1264875309
4796	Diptera	Culicidae	Aedes	Aedes aegypti	SPECIES	-7.34000	-39.03000	1264875331
4797	Diptera	Culicidae	Aedes	Aedes aegypti	SPECIES	-7.34000	110.51000	1264876480

№	Order	Family	Genus	Species	Taxon Rank	Latitude	Longitude	GBIF_ID
4798	Diptera	Culicidae	Aedes	Aedes aegypti	SPECIES	-7.34000	-48.62000	1264890861
4799	Diptera	Culicidae	Aedes	Aedes aegypti	SPECIES	-7.33000	110.49000	1264871722
4800	Diptera	Culicidae	Aedes	Aedes aegypti	SPECIES	-7.33000	-36.91000	1264875308
4801	Diptera	Culicidae	Aedes	Aedes aegypti	SPECIES	-7.33000	-37.35000	1264875349
4802	Diptera	Culicidae	Aedes	Aedes aegypti	SPECIES	-7.33000	110.50000	1264876404
4803	Diptera	Culicidae	Aedes	Aedes aegypti	SPECIES	-7.33000	110.51000	1264876405
4804	Diptera	Culicidae	Aedes	Aedes aegypti	SPECIES	-7.32000	110.51000	1264871746
4805	Diptera	Culicidae	Aedes	Aedes aegypti	SPECIES	-7.32000	110.50000	1264871906
4806	Diptera	Culicidae	Aedes	Aedes aegypti	SPECIES	-7.32000	-40.63000	1264875316
4807	Diptera	Culicidae	Aedes	Aedes aegypti	SPECIES	-7.32000	-36.68000	1264875346
4808	Diptera	Culicidae	Aedes	Aedes aegypti	SPECIES	-7.32000	-39.14000	1264875350
4809	Diptera	Culicidae	Aedes	Aedes aegypti	SPECIES	-7.32000	-39.30000	1264889158
4810	Diptera	Culicidae	Aedes	Aedes aegypti	SPECIES	-7.31000	110.49000	1264871910
4811	Diptera	Culicidae	Aedes	Aedes aegypti	SPECIES	-7.31000	-37.09000	1264875332
4812	Diptera	Culicidae	Aedes	Aedes aegypti	SPECIES	-7.31000	-43.93000	1264875348
4813	Diptera	Culicidae	Aedes	Aedes aegypti	SPECIES	-7.31000	-38.15000	1264888066
4814	Diptera	Culicidae	Aedes	Aedes aegypti	SPECIES	-7.30000	110.52000	1264871911
4815	Diptera	Culicidae	Aedes	Aedes aegypti	SPECIES	-7.30000	110.48000	1264871913
4816	Diptera	Culicidae	Aedes	Aedes aegypti	SPECIES	-7.30000	110.50000	1264871924
4817	Diptera	Culicidae	Aedes	Aedes aegypti	SPECIES	-7.30000	-35.48000	1264875314
4818	Diptera	Culicidae	Aedes	Aedes aegypti	SPECIES	-7.30000	-34.87000	1264875359
4819	Diptera	Culicidae	Aedes	Aedes aegypti	SPECIES	-7.30000	110.47000	1264888128
4820	Diptera	Culicidae	Aedes	Aedes aegypti	SPECIES	-7.29000	112.80000	1264871808
4821	Diptera	Culicidae	Aedes	Aedes aegypti	SPECIES	-7.29000	-37.36000	1264875318
4822	Diptera	Culicidae	Aedes	Aedes aegypti	SPECIES	-7.29000	-37.74000	1264875339
4823	Diptera	Culicidae	Aedes	Aedes aegypti	SPECIES	-7.29000	-38.48000	1264875345
4824	Diptera	Culicidae	Aedes	Aedes aegypti	SPECIES	-7.29000	-39.47000	1264875360
4825	Diptera	Culicidae	Aedes	Aedes aegypti	SPECIES	-7.29000	112.82000	1264887175
4826	Diptera	Culicidae	Aedes	Aedes aegypti	SPECIES	-7.29000	112.72000	1264887185
4827	Diptera	Culicidae	Aedes	Aedes aegypti	SPECIES	-7.28000	112.72000	1264871797
4828	Diptera	Culicidae	Aedes	Aedes aegypti	SPECIES	-7.28000	112.71000	1264871811
4829	Diptera	Culicidae	Aedes	Aedes aegypti	SPECIES	-7.28000	-35.63000	1264875321
4830	Diptera	Culicidae	Aedes	Aedes aegypti	SPECIES	-7.28000	-35.27000	1264875333
4831	Diptera	Culicidae	Aedes	Aedes aegypti	SPECIES	-7.28000	-38.19000	1264875344
4832	Diptera	Culicidae	Aedes	Aedes aegypti	SPECIES	-7.28000	112.74000	1264887197
4833	Diptera	Culicidae	Aedes	Aedes aegypti	SPECIES	-7.28000	112.75000	1264887213
4834	Diptera	Culicidae	Aedes	Aedes aegypti	SPECIES	-7.27000	112.75000	1264871803
4835	Diptera	Culicidae	Aedes	Aedes aegypti	SPECIES	-7.27000	-42.16000	1264875322
4836	Diptera	Culicidae	Aedes	Aedes aegypti	SPECIES	-7.27000	-35.92000	1264875337
4837	Diptera	Culicidae	Aedes	Aedes aegypti	SPECIES	-7.27000	-36.47000	1264875342
4838	Diptera	Culicidae	Aedes	Aedes aegypti	SPECIES	-7.27000	-41.89000	1264875353

№	Order	Family	Genus	Species	Taxon Rank	Latitude	Longitude	GBIF_ID
4839	Diptera	Culicidae	Aedes	Aedes aegypti	SPECIES	-7.27000	-38.94000	1264875354
4840	Diptera	Culicidae	Aedes	Aedes aegypti	SPECIES	-7.27000	112.72000	1264876449
4841	Diptera	Culicidae	Aedes	Aedes aegypti	SPECIES	-7.27000	112.79000	1264887226
4842	Diptera	Culicidae	Aedes	Aedes aegypti	SPECIES	-7.27000	112.70000	1264887231
4843	Diptera	Culicidae	Aedes	Aedes aegypti	SPECIES	-7.27000	110.47000	1264890995
4844	Diptera	Culicidae	Aedes	Aedes aegypti	SPECIES	-7.26000	110.41000	1264871634
4845	Diptera	Culicidae	Aedes	Aedes aegypti	SPECIES	-7.26000	110.40000	1264871668
4846	Diptera	Culicidae	Aedes	Aedes aegypti	SPECIES	-7.26000	-36.22000	1264875347
4847	Diptera	Culicidae	Aedes	Aedes aegypti	SPECIES	-7.26000	112.76000	1264876398
4848	Diptera	Culicidae	Aedes	Aedes aegypti	SPECIES	-7.26000	112.68000	1264876431
4849	Diptera	Culicidae	Aedes	Aedes aegypti	SPECIES	-7.26000	112.77000	1264876445
4850	Diptera	Culicidae	Aedes	Aedes aegypti	SPECIES	-7.26000	112.67000	1264887180
4851	Diptera	Culicidae	Aedes	Aedes aegypti	SPECIES	-7.26000	112.72000	1264887208
4852	Diptera	Culicidae	Aedes	Aedes aegypti	SPECIES	-7.26000	112.75000	1264887218
4853	Diptera	Culicidae	Aedes	Aedes aegypti	SPECIES	-7.25000	110.41000	1264871657
4854	Diptera	Culicidae	Aedes	Aedes aegypti	SPECIES	-7.25000	-37.24000	1264875340
4855	Diptera	Culicidae	Aedes	Aedes aegypti	SPECIES	-7.25000	-38.33000	1264875355
4856	Diptera	Culicidae	Aedes	Aedes aegypti	SPECIES	-7.25000	-48.28000	1264875365
4857	Diptera	Culicidae	Aedes	Aedes aegypti	SPECIES	-7.25000	112.76000	1264876471
4858	Diptera	Culicidae	Aedes	Aedes aegypti	SPECIES	-7.25000	112.77000	1264887187
4859	Diptera	Culicidae	Aedes	Aedes aegypti	SPECIES	-7.24000	-37.44000	1264875341
4860	Diptera	Culicidae	Aedes	Aedes aegypti	SPECIES	-7.24000	-35.37000	1264875343
4861	Diptera	Culicidae	Aedes	Aedes aegypti	SPECIES	-7.24000	-36.60000	1264875357
4862	Diptera	Culicidae	Aedes	Aedes aegypti	SPECIES	-7.24000	112.77000	1264875371
4863	Diptera	Culicidae	Aedes	Aedes aegypti	SPECIES	-7.24000	-35.20000	1264875373
4864	Diptera	Culicidae	Aedes	Aedes aegypti	SPECIES	-7.24000	-35.66000	1264875374
4865	Diptera	Culicidae	Aedes	Aedes aegypti	SPECIES	-7.24000	-41.54000	1264875375
4866	Diptera	Culicidae	Aedes	Aedes aegypti	SPECIES	-7.24000	112.75000	1264887186
4867	Diptera	Culicidae	Aedes	Aedes aegypti	SPECIES	-7.24000	112.79000	1264887195
4868	Diptera	Culicidae	Aedes	Aedes aegypti	SPECIES	-7.24000	-35.90000	1264888048
4869	Diptera	Culicidae	Aedes	Aedes aegypti	SPECIES	-7.24000	112.74000	1264890635
4870	Diptera	Culicidae	Aedes	Aedes aegypti	SPECIES	-7.23000	-44.21000	1264875338
4871	Diptera	Culicidae	Aedes	Aedes aegypti	SPECIES	-7.23000	-38.37000	1264875368
4872	Diptera	Culicidae	Aedes	Aedes aegypti	SPECIES	-7.23000	-39.75000	1264875372
4873	Diptera	Culicidae	Aedes	Aedes aegypti	SPECIES	-7.23000	-41.76000	1264875382
4874	Diptera	Culicidae	Aedes	Aedes aegypti	SPECIES	-7.23000	112.76000	1264876444
4875	Diptera	Culicidae	Aedes	Aedes aegypti	SPECIES	-7.23000	112.73000	1264876465
4876	Diptera	Culicidae	Aedes	Aedes aegypti	SPECIES	-7.23000	-35.90000	1264888059
4877	Diptera	Culicidae	Aedes	Aedes aegypti	SPECIES	-7.23000	-39.41000	1264889138
4878	Diptera	Culicidae	Aedes	Aedes aegypti	SPECIES	-7.23000	-39.32000	1264889161
4879	Diptera	Culicidae	Aedes	Aedes aegypti	SPECIES	-7.23000	-35.87000	1264890381

№	Order	Family	Genus	Species	Taxon Rank	Latitude	Longitude	GBIF_ID
4880	Diptera	Culicidae	Aedes	Aedes aegypti	SPECIES	-7.22000	-36.85000	1264875351
4881	Diptera	Culicidae	Aedes	Aedes aegypti	SPECIES	-7.22000	112.76000	1264876455
4882	Diptera	Culicidae	Aedes	Aedes aegypti	SPECIES	-7.22000	112.77000	1264887182
4883	Diptera	Culicidae	Aedes	Aedes aegypti	SPECIES	-7.21000	-40.08000	1264875327
4884	Diptera	Culicidae	Aedes	Aedes aegypti	SPECIES	-7.21000	-40.93000	1264875356
4885	Diptera	Culicidae	Aedes	Aedes aegypti	SPECIES	-7.21000	-38.52000	1264875366
4886	Diptera	Culicidae	Aedes	Aedes aegypti	SPECIES	-7.21000	-37.09000	1264875393
4887	Diptera	Culicidae	Aedes	Aedes aegypti	SPECIES	-7.21000	112.73000	1264876450
4888	Diptera	Culicidae	Aedes	Aedes aegypti	SPECIES	-7.21000	-39.31000	1264889167
4889	Diptera	Culicidae	Aedes	Aedes aegypti	SPECIES	-7.21000	-39.32000	1264889569
4890	Diptera	Culicidae	Aedes	Aedes aegypti	SPECIES	-7.20000	-45.29000	1264875362
4891	Diptera	Culicidae	Aedes	Aedes aegypti	SPECIES	-7.20000	-41.33000	1264875370
4892	Diptera	Culicidae	Aedes	Aedes aegypti	SPECIES	-7.20000	-37.93000	1264875377
4893	Diptera	Culicidae	Aedes	Aedes aegypti	SPECIES	-7.20000	-40.78000	1264875390
4894	Diptera	Culicidae	Aedes	Aedes aegypti	SPECIES	-7.20000	-48.20000	1264890232
4895	Diptera	Culicidae	Aedes	Aedes aegypti	SPECIES	-7.19000	-35.76000	1264875358
4896	Diptera	Culicidae	Aedes	Aedes aegypti	SPECIES	-7.19000	-42.49000	1264875363
4897	Diptera	Culicidae	Aedes	Aedes aegypti	SPECIES	-7.19000	-40.32000	1264875367
4898	Diptera	Culicidae	Aedes	Aedes aegypti	SPECIES	-7.19000	-39.28000	1264875380
4899	Diptera	Culicidae	Aedes	Aedes aegypti	SPECIES	-7.18000	-41.06000	1264875364
4900	Diptera	Culicidae	Aedes	Aedes aegypti	SPECIES	-7.18000	-35.66000	1264875395
4901	Diptera	Culicidae	Aedes	Aedes aegypti	SPECIES	-7.18000	-39.28000	1264890815
4902	Diptera	Culicidae	Aedes	Aedes aegypti	SPECIES	-7.17000	-35.26000	1264875398
4903	Diptera	Culicidae	Aedes	Aedes aegypti	SPECIES	-7.17000	-46.26000	1264875400
4904	Diptera	Culicidae	Aedes	Aedes aegypti	SPECIES	-7.17000	-39.33000	1264890458
4905	Diptera	Culicidae	Aedes	Aedes aegypti	SPECIES	-7.16000	-35.57000	1264875361
4906	Diptera	Culicidae	Aedes	Aedes aegypti	SPECIES	-7.16000	-44.62000	1264875369
4907	Diptera	Culicidae	Aedes	Aedes aegypti	SPECIES	-7.16000	-41.70000	1264875379
4908	Diptera	Culicidae	Aedes	Aedes aegypti	SPECIES	-7.16000	-38.14000	1264875383
4909	Diptera	Culicidae	Aedes	Aedes aegypti	SPECIES	-7.16000	-47.84000	1264875399
4910	Diptera	Culicidae	Aedes	Aedes aegypti	SPECIES	-7.16000	-37.60000	1264875407
4911	Diptera	Culicidae	Aedes	Aedes aegypti	SPECIES	-7.16000	-48.05000	1264891166
4912	Diptera	Culicidae	Aedes	Aedes aegypti	SPECIES	-7.15822	-34.79661	3018063222
4913	Diptera	Culicidae	Aedes	Aedes aegypti	SPECIES	-7.15000	-35.29000	1264875352
4914	Diptera	Culicidae	Aedes	Aedes aegypti	SPECIES	-7.15000	-37.32000	1264875376
4915	Diptera	Culicidae	Aedes	Aedes aegypti	SPECIES	-7.15000	-35.86000	1264875381
4916	Diptera	Culicidae	Aedes	Aedes aegypti	SPECIES	-7.15000	-34.88000	1264875385
4917	Diptera	Culicidae	Aedes	Aedes aegypti	SPECIES	-7.15000	-35.97000	1264875397
4918	Diptera	Culicidae	Aedes	Aedes aegypti	SPECIES	-7.14000	110.41000	1264871616
4919	Diptera	Culicidae	Aedes	Aedes aegypti	SPECIES	-7.14000	-35.45000	1264875384
4920	Diptera	Culicidae	Aedes	Aedes aegypti	SPECIES	-7.14000	-35.11000	1264875396

№	Order	Family	Genus	Species	Taxon Rank	Latitude	Longitude	GBIF_ID
4921	Diptera	Culicidae	Aedes	Aedes aegypti	SPECIES	-7.14000	-37.17000	1264875419
4922	Diptera	Culicidae	Aedes	Aedes aegypti	SPECIES	-7.13000	110.41000	1264871608
4923	Diptera	Culicidae	Aedes	Aedes aegypti	SPECIES	-7.13000	-34.93000	1264875392
4924	Diptera	Culicidae	Aedes	Aedes aegypti	SPECIES	-7.13000	-34.87000	1264888050
4925	Diptera	Culicidae	Aedes	Aedes aegypti	SPECIES	-7.12000	110.42000	1264871631
4926	Diptera	Culicidae	Aedes	Aedes aegypti	SPECIES	-7.12000	110.40000	1264871636
4927	Diptera	Culicidae	Aedes	Aedes aegypti	SPECIES	-7.12000	-35.77000	1264875386
4928	Diptera	Culicidae	Aedes	Aedes aegypti	SPECIES	-7.11460	-34.83845	4054887028
4929	Diptera	Culicidae	Aedes	Aedes aegypti	SPECIES	-7.11000	110.43000	1264871610
4930	Diptera	Culicidae	Aedes	Aedes aegypti	SPECIES	-7.11000	110.40000	1264871613
4931	Diptera	Culicidae	Aedes	Aedes aegypti	SPECIES	-7.11000	110.42000	1264871650
4932	Diptera	Culicidae	Aedes	Aedes aegypti	SPECIES	-7.11000	110.89000	1264871731
4933	Diptera	Culicidae	Aedes	Aedes aegypti	SPECIES	-7.11000	-44.89000	1264875388
4934	Diptera	Culicidae	Aedes	Aedes aegypti	SPECIES	-7.11000	-38.79000	1264875391
4935	Diptera	Culicidae	Aedes	Aedes aegypti	SPECIES	-7.11000	-36.96000	1264875394
4936	Diptera	Culicidae	Aedes	Aedes aegypti	SPECIES	-7.11000	-43.51000	1264875405
4937	Diptera	Culicidae	Aedes	Aedes aegypti	SPECIES	-7.11000	-37.05000	1264875406
4938	Diptera	Culicidae	Aedes	Aedes aegypti	SPECIES	-7.11000	-35.33000	1264875417
4939	Diptera	Culicidae	Aedes	Aedes aegypti	SPECIES	-7.11000	-37.45000	1264875423
4940	Diptera	Culicidae	Aedes	Aedes aegypti	SPECIES	-7.11000	-39.67000	1264875426
4941	Diptera	Culicidae	Aedes	Aedes aegypti	SPECIES	-7.10000	-35.01000	1264875389
4942	Diptera	Culicidae	Aedes	Aedes aegypti	SPECIES	-7.10000	-37.75000	1264875424
4943	Diptera	Culicidae	Aedes	Aedes aegypti	SPECIES	-7.10000	113.61000	1264891289
4944	Diptera	Culicidae	Aedes	Aedes aegypti	SPECIES	-7.09000	-35.85000	1264875387
4945	Diptera	Culicidae	Aedes	Aedes aegypti	SPECIES	-7.09000	-36.84000	1264875402
4946	Diptera	Culicidae	Aedes	Aedes aegypti	SPECIES	-7.09000	-36.35000	1264875409
4947	Diptera	Culicidae	Aedes	Aedes aegypti	SPECIES	-7.09000	-38.20000	1264875411
4948	Diptera	Culicidae	Aedes	Aedes aegypti	SPECIES	-7.09000	-38.49000	1264875420
4949	Diptera	Culicidae	Aedes	Aedes aegypti	SPECIES	-7.09000	-35.94000	1264875432
4950	Diptera	Culicidae	Aedes	Aedes aegypti	SPECIES	-7.08000	-42.76000	1264875378
4951	Diptera	Culicidae	Aedes	Aedes aegypti	SPECIES	-7.08000	-40.60000	1264875403
4952	Diptera	Culicidae	Aedes	Aedes aegypti	SPECIES	-7.08000	-43.89000	1264875410
4953	Diptera	Culicidae	Aedes	Aedes aegypti	SPECIES	-7.08000	111.39000	1264890948
4954	Diptera	Culicidae	Aedes	Aedes aegypti	SPECIES	-7.07000	110.56000	1264871923
4955	Diptera	Culicidae	Aedes	Aedes aegypti	SPECIES	-7.07000	-36.72000	1264875401
4956	Diptera	Culicidae	Aedes	Aedes aegypti	SPECIES	-7.07000	-40.84000	1264875408
4957	Diptera	Culicidae	Aedes	Aedes aegypti	SPECIES	-7.07000	-35.76000	1264875413
4958	Diptera	Culicidae	Aedes	Aedes aegypti	SPECIES	-7.07000	-40.03000	1264875422
4959	Diptera	Culicidae	Aedes	Aedes aegypti	SPECIES	-7.07000	110.42000	1264876429
4960	Diptera	Culicidae	Aedes	Aedes aegypti	SPECIES	-7.06000	110.43000	1264871704
4961	Diptera	Culicidae	Aedes	Aedes aegypti	SPECIES	-7.06000	110.88000	1264871744

№	Order	Family	Genus	Species	Taxon Rank	Latitude	Longitude	GBIF_ID
4962	Diptera	Culicidae	Aedes	Aedes aegypti	SPECIES	-7.06000	110.46000	1264871879
4963	Diptera	Culicidae	Aedes	Aedes aegypti	SPECIES	-7.06000	-35.20000	1264875414
4964	Diptera	Culicidae	Aedes	Aedes aegypti	SPECIES	-7.06000	-37.96000	1264875416
4965	Diptera	Culicidae	Aedes	Aedes aegypti	SPECIES	-7.06000	-36.09000	1264875429
4966	Diptera	Culicidae	Aedes	Aedes aegypti	SPECIES	-7.06000	110.41000	1264876514
4967	Diptera	Culicidae	Aedes	Aedes aegypti	SPECIES	-7.06000	109.62000	1264890997
4968	Diptera	Culicidae	Aedes	Aedes aegypti	SPECIES	-7.05000	-35.94000	1264875415
4969	Diptera	Culicidae	Aedes	Aedes aegypti	SPECIES	-7.05000	-41.49000	1264875418
4970	Diptera	Culicidae	Aedes	Aedes aegypti	SPECIES	-7.05000	-37.12000	1264875443
4971	Diptera	Culicidae	Aedes	Aedes aegypti	SPECIES	-7.05000	-45.69000	1264875445
4972	Diptera	Culicidae	Aedes	Aedes aegypti	SPECIES	-7.05000	-38.33000	1264875448
4973	Diptera	Culicidae	Aedes	Aedes aegypti	SPECIES	-7.05000	-35.60000	1264875451
4974	Diptera	Culicidae	Aedes	Aedes aegypti	SPECIES	-7.05000	110.46000	1264876426
4975	Diptera	Culicidae	Aedes	Aedes aegypti	SPECIES	-7.04000	110.47000	1264871857
4976	Diptera	Culicidae	Aedes	Aedes aegypti	SPECIES	-7.04000	110.52000	1264871921
4977	Diptera	Culicidae	Aedes	Aedes aegypti	SPECIES	-7.04000	-36.57000	1264875431
4978	Diptera	Culicidae	Aedes	Aedes aegypti	SPECIES	-7.04000	-48.94000	1264875434
4979	Diptera	Culicidae	Aedes	Aedes aegypti	SPECIES	-7.04000	-48.38000	1264875435
4980	Diptera	Culicidae	Aedes	Aedes aegypti	SPECIES	-7.04000	-35.31000	1264875457
4981	Diptera	Culicidae	Aedes	Aedes aegypti	SPECIES	-7.04000	110.48000	1264876477
4982	Diptera	Culicidae	Aedes	Aedes aegypti	SPECIES	-7.04000	109.39000	1264890981
4983	Diptera	Culicidae	Aedes	Aedes aegypti	SPECIES	-7.03000	-34.85000	1264875404
4984	Diptera	Culicidae	Aedes	Aedes aegypti	SPECIES	-7.03000	-37.35000	1264875427
4985	Diptera	Culicidae	Aedes	Aedes aegypti	SPECIES	-7.03000	-39.28000	1264875450
4986	Diptera	Culicidae	Aedes	Aedes aegypti	SPECIES	-7.03000	110.32000	1264876483
4987	Diptera	Culicidae	Aedes	Aedes aegypti	SPECIES	-7.02000	111.42000	1264871730
4988	Diptera	Culicidae	Aedes	Aedes aegypti	SPECIES	-7.02000	-48.79000	1264875412
4989	Diptera	Culicidae	Aedes	Aedes aegypti	SPECIES	-7.02000	110.47000	1264876428
4990	Diptera	Culicidae	Aedes	Aedes aegypti	SPECIES	-7.02000	109.86000	1264890954
4991	Diptera	Culicidae	Aedes	Aedes aegypti	SPECIES	-7.01000	110.39000	1264871714
4992	Diptera	Culicidae	Aedes	Aedes aegypti	SPECIES	-7.01000	-46.61000	1264875421
4993	Diptera	Culicidae	Aedes	Aedes aegypti	SPECIES	-7.01000	-38.95000	1264875436
4994	Diptera	Culicidae	Aedes	Aedes aegypti	SPECIES	-7.01000	-35.88000	1264875442
4995	Diptera	Culicidae	Aedes	Aedes aegypti	SPECIES	-7.01000	110.41000	1264876452
4996	Diptera	Culicidae	Aedes	Aedes aegypti	SPECIES	-7.01000	110.46000	1264876476
4997	Diptera	Culicidae	Aedes	Aedes aegypti	SPECIES	-7.00000	110.39000	1264871787
4998	Diptera	Culicidae	Aedes	Aedes aegypti	SPECIES	-7.00000	106.72000	1264871802
4999	Diptera	Culicidae	Aedes	Aedes aegypti	SPECIES	-7.00000	110.71000	1264871905
5000	Diptera	Culicidae	Aedes	Aedes aegypti	SPECIES	-7.00000	-41.34000	1264875425
5001	Diptera	Culicidae	Aedes	Aedes aegypti	SPECIES	-7.00000	-43.12000	1264875428
5002	Diptera	Culicidae	Aedes	Aedes aegypti	SPECIES	-7.00000	-35.42000	1264875438

№	Order	Family	Genus	Species	Taxon Rank	Latitude	Longitude	GBIF_ID
5003	Diptera	Culicidae	Aedes	Aedes aegypti	SPECIES	-7.00000	-41.17000	1264875447
5004	Diptera	Culicidae	Aedes	Aedes aegypti	SPECIES	-7.00000	110.40000	1264876458
5005	Diptera	Culicidae	Aedes	Aedes aegypti	SPECIES	-7.00000	110.47000	1264876463
5006	Diptera	Culicidae	Aedes	Aedes aegypti	SPECIES	-6.99000	-36.76000	1264875433
5007	Diptera	Culicidae	Aedes	Aedes aegypti	SPECIES	-6.99000	-48.53000	1264875439
5008	Diptera	Culicidae	Aedes	Aedes aegypti	SPECIES	-6.99000	110.49000	1264875440
5009	Diptera	Culicidae	Aedes	Aedes aegypti	SPECIES	-6.99000	-46.11000	1264875454
5010	Diptera	Culicidae	Aedes	Aedes aegypti	SPECIES	-6.99000	109.85000	1264876437
5011	Diptera	Culicidae	Aedes	Aedes aegypti	SPECIES	-6.99000	109.86000	1264876486
5012	Diptera	Culicidae	Aedes	Aedes aegypti	SPECIES	-6.98000	110.61000	1264871900
5013	Diptera	Culicidae	Aedes	Aedes aegypti	SPECIES	-6.98000	-36.25000	1264875444
5014	Diptera	Culicidae	Aedes	Aedes aegypti	SPECIES	-6.98000	-44.06000	1264875469
5015	Diptera	Culicidae	Aedes	Aedes aegypti	SPECIES	-6.98000	-39.71000	1264875473
5016	Diptera	Culicidae	Aedes	Aedes aegypti	SPECIES	-6.98000	110.32000	1264876461
5017	Diptera	Culicidae	Aedes	Aedes aegypti	SPECIES	-6.98000	110.20000	1264891276
5018	Diptera	Culicidae	Aedes	Aedes aegypti	SPECIES	-6.97000	110.36000	1264871715
5019	Diptera	Culicidae	Aedes	Aedes aegypti	SPECIES	-6.97000	111.44000	1264871859
5020	Diptera	Culicidae	Aedes	Aedes aegypti	SPECIES	-6.97000	111.42000	1264871892
5021	Diptera	Culicidae	Aedes	Aedes aegypti	SPECIES	-6.97000	-35.52000	1264875441
5022	Diptera	Culicidae	Aedes	Aedes aegypti	SPECIES	-6.97000	-41.71000	1264875459
5023	Diptera	Culicidae	Aedes	Aedes aegypti	SPECIES	-6.97000	-42.25000	1264875476
5024	Diptera	Culicidae	Aedes	Aedes aegypti	SPECIES	-6.97000	110.43000	1264889674
5025	Diptera	Culicidae	Aedes	Aedes aegypti	SPECIES	-6.97000	110.42000	1264890267
5026	Diptera	Culicidae	Aedes	Aedes aegypti	SPECIES	-6.97000	-35.79000	1264890594
5027	Diptera	Culicidae	Aedes	Aedes aegypti	SPECIES	-6.96000	111.43000	1264871882
5028	Diptera	Culicidae	Aedes	Aedes aegypti	SPECIES	-6.96000	110.74000	1264871942
5029	Diptera	Culicidae	Aedes	Aedes aegypti	SPECIES	-6.96000	106.95000	1264876468
5030	Diptera	Culicidae	Aedes	Aedes aegypti	SPECIES	-6.95000	-41.87000	1264875430
5031	Diptera	Culicidae	Aedes	Aedes aegypti	SPECIES	-6.95000	-36.63000	1264875453
5032	Diptera	Culicidae	Aedes	Aedes aegypti	SPECIES	-6.95000	-38.70000	1264875455
5033	Diptera	Culicidae	Aedes	Aedes aegypti	SPECIES	-6.95000	-38.34000	1264875461
5034	Diptera	Culicidae	Aedes	Aedes aegypti	SPECIES	-6.95000	-37.79000	1264875467
5035	Diptera	Culicidae	Aedes	Aedes aegypti	SPECIES	-6.95000	-40.91000	1264875468
5036	Diptera	Culicidae	Aedes	Aedes aegypti	SPECIES	-6.95000	-35.69000	1264875482
5037	Diptera	Culicidae	Aedes	Aedes aegypti	SPECIES	-6.95000	110.47000	1264876454
5038	Diptera	Culicidae	Aedes	Aedes aegypti	SPECIES	-6.94000	-41.04000	1264875437
5039	Diptera	Culicidae	Aedes	Aedes aegypti	SPECIES	-6.94000	-40.26000	1264875446
5040	Diptera	Culicidae	Aedes	Aedes aegypti	SPECIES	-6.94000	-35.86000	1264875472
5041	Diptera	Culicidae	Aedes	Aedes aegypti	SPECIES	-6.94000	-38.14000	1264875475
5042	Diptera	Culicidae	Aedes	Aedes aegypti	SPECIES	-6.93000	107.64000	1264876441
5043	Diptera	Culicidae	Aedes	Aedes aegypti	SPECIES	-6.92000	107.56000	1264871858

№	Order	Family	Genus	Species	Taxon Rank	Latitude	Longitude	GBIF_ID
5044	Diptera	Culicidae	Aedes	Aedes aegypti	SPECIES	-6.92000	110.58000	1264871894
5045	Diptera	Culicidae	Aedes	Aedes aegypti	SPECIES	-6.92000	110.52000	1264871912
5046	Diptera	Culicidae	Aedes	Aedes aegypti	SPECIES	-6.92000	-38.53000	1264875449
5047	Diptera	Culicidae	Aedes	Aedes aegypti	SPECIES	-6.92000	-39.26000	1264875452
5048	Diptera	Culicidae	Aedes	Aedes aegypti	SPECIES	-6.92000	-39.86000	1264875458
5049	Diptera	Culicidae	Aedes	Aedes aegypti	SPECIES	-6.92000	-37.08000	1264875460
5050	Diptera	Culicidae	Aedes	Aedes aegypti	SPECIES	-6.92000	-36.90000	1264875463
5051	Diptera	Culicidae	Aedes	Aedes aegypti	SPECIES	-6.92000	-41.52000	1264875464
5052	Diptera	Culicidae	Aedes	Aedes aegypti	SPECIES	-6.92000	-34.92000	1264875465
5053	Diptera	Culicidae	Aedes	Aedes aegypti	SPECIES	-6.92000	-36.45000	1264875494
5054	Diptera	Culicidae	Aedes	Aedes aegypti	SPECIES	-6.92000	-41.31000	1264875499
5055	Diptera	Culicidae	Aedes	Aedes aegypti	SPECIES	-6.91000	106.30000	1264871825
5056	Diptera	Culicidae	Aedes	Aedes aegypti	SPECIES	-6.91000	-39.54000	1264875470
5057	Diptera	Culicidae	Aedes	Aedes aegypti	SPECIES	-6.91000	107.56000	1264876422
5058	Diptera	Culicidae	Aedes	Aedes aegypti	SPECIES	-6.91000	107.61000	1264890621
5059	Diptera	Culicidae	Aedes	Aedes aegypti	SPECIES	-6.90000	110.75000	1264871915
5060	Diptera	Culicidae	Aedes	Aedes aegypti	SPECIES	-6.90000	-35.25000	1264875466
5061	Diptera	Culicidae	Aedes	Aedes aegypti	SPECIES	-6.90000	-35.52000	1264875479
5062	Diptera	Culicidae	Aedes	Aedes aegypti	SPECIES	-6.90000	-37.71000	1264875493
5063	Diptera	Culicidae	Aedes	Aedes aegypti	SPECIES	-6.90000	-35.16000	1264875501
5064	Diptera	Culicidae	Aedes	Aedes aegypti	SPECIES	-6.90000	39.27000	1264890189
5065	Diptera	Culicidae	Aedes	Aedes aegypti	SPECIES	-6.89000	107.55000	1264871872
5066	Diptera	Culicidae	Aedes	Aedes aegypti	SPECIES	-6.89000	-37.51000	1264875456
5067	Diptera	Culicidae	Aedes	Aedes aegypti	SPECIES	-6.89000	-36.66000	1264875480
5068	Diptera	Culicidae	Aedes	Aedes aegypti	SPECIES	-6.89000	-35.96000	1264875486
5069	Diptera	Culicidae	Aedes	Aedes aegypti	SPECIES	-6.89000	-35.46000	1264875507
5070	Diptera	Culicidae	Aedes	Aedes aegypti	SPECIES	-6.89000	107.54000	1264876497
5071	Diptera	Culicidae	Aedes	Aedes aegypti	SPECIES	-6.88626	107.54112	4017659871
5072	Diptera	Culicidae	Aedes	Aedes aegypti	SPECIES	-6.88000	109.15000	1264871855
5073	Diptera	Culicidae	Aedes	Aedes aegypti	SPECIES	-6.88000	110.65000	1264871927
5074	Diptera	Culicidae	Aedes	Aedes aegypti	SPECIES	-6.88000	-41.18000	1264875477
5075	Diptera	Culicidae	Aedes	Aedes aegypti	SPECIES	-6.88000	-47.97000	1264875484
5076	Diptera	Culicidae	Aedes	Aedes aegypti	SPECIES	-6.88000	-35.61000	1264875492
5077	Diptera	Culicidae	Aedes	Aedes aegypti	SPECIES	-6.88000	107.57000	1264876460
5078	Diptera	Culicidae	Aedes	Aedes aegypti	SPECIES	-6.88000	109.62000	1264876474
5079	Diptera	Culicidae	Aedes	Aedes aegypti	SPECIES	-6.87000	109.12000	1264871862
5080	Diptera	Culicidae	Aedes	Aedes aegypti	SPECIES	-6.87000	-37.61000	1264875462
5081	Diptera	Culicidae	Aedes	Aedes aegypti	SPECIES	-6.87000	-36.32000	1264875471
5082	Diptera	Culicidae	Aedes	Aedes aegypti	SPECIES	-6.87000	-35.36000	1264875497
5083	Diptera	Culicidae	Aedes	Aedes aegypti	SPECIES	-6.87000	107.56000	1264876470
5084	Diptera	Culicidae	Aedes	Aedes aegypti	SPECIES	-6.87000	107.55000	1264876490

№	Order	Family	Genus	Species	Taxon Rank	Latitude	Longitude	GBIF_ID
5085	Diptera	Culicidae	Aedes	Aedes aegypti	SPECIES	-6.86000	109.15000	1264871866
5086	Diptera	Culicidae	Aedes	Aedes aegypti	SPECIES	-6.86000	-35.73000	1264875500
5087	Diptera	Culicidae	Aedes	Aedes aegypti	SPECIES	-6.85000	107.54000	1264871878
5088	Diptera	Culicidae	Aedes	Aedes aegypti	SPECIES	-6.85000	110.59000	1264871937
5089	Diptera	Culicidae	Aedes	Aedes aegypti	SPECIES	-6.85000	110.78000	1264871948
5090	Diptera	Culicidae	Aedes	Aedes aegypti	SPECIES	-6.85000	-35.55000	1264875474
5091	Diptera	Culicidae	Aedes	Aedes aegypti	SPECIES	-6.85000	-46.44000	1264875478
5092	Diptera	Culicidae	Aedes	Aedes aegypti	SPECIES	-6.85000	-44.60000	1264875483
5093	Diptera	Culicidae	Aedes	Aedes aegypti	SPECIES	-6.85000	-49.26000	1264875488
5094	Diptera	Culicidae	Aedes	Aedes aegypti	SPECIES	-6.85000	39.28000	1264889734
5095	Diptera	Culicidae	Aedes	Aedes aegypti	SPECIES	-6.84000	-43.71000	1264875519
5096	Diptera	Culicidae	Aedes	Aedes aegypti	SPECIES	-6.84000	39.29000	1264889730
5097	Diptera	Culicidae	Aedes	Aedes aegypti	SPECIES	-6.83000	-60.51000	1264875485
5098	Diptera	Culicidae	Aedes	Aedes aegypti	SPECIES	-6.83000	-38.62000	1264875489
5099	Diptera	Culicidae	Aedes	Aedes aegypti	SPECIES	-6.83000	-35.64000	1264875490
5100	Diptera	Culicidae	Aedes	Aedes aegypti	SPECIES	-6.83000	-49.54000	1264875524
5101	Diptera	Culicidae	Aedes	Aedes aegypti	SPECIES	-6.83000	39.25000	1264889702
5102	Diptera	Culicidae	Aedes	Aedes aegypti	SPECIES	-6.83000	39.27000	1264889767
5103	Diptera	Culicidae	Aedes	Aedes aegypti	SPECIES	-6.82000	-37.83000	1264875491
5104	Diptera	Culicidae	Aedes	Aedes aegypti	SPECIES	-6.82000	-38.33000	1264875495
5105	Diptera	Culicidae	Aedes	Aedes aegypti	SPECIES	-6.82000	-36.82000	1264875504
5106	Diptera	Culicidae	Aedes	Aedes aegypti	SPECIES	-6.82000	39.25000	1264888295
5107	Diptera	Culicidae	Aedes	Aedes aegypti	SPECIES	-6.82000	39.29000	1264889743
5108	Diptera	Culicidae	Aedes	Aedes aegypti	SPECIES	-6.82000	39.32000	1264889747
5109	Diptera	Culicidae	Aedes	Aedes aegypti	SPECIES	-6.82000	39.28000	1264890218
5110	Diptera	Culicidae	Aedes	Aedes aegypti	SPECIES	-6.81000	110.70000	1264871952
5111	Diptera	Culicidae	Aedes	Aedes aegypti	SPECIES	-6.81000	-38.73000	1264875481
5112	Diptera	Culicidae	Aedes	Aedes aegypti	SPECIES	-6.81000	-42.24000	1264875505
5113	Diptera	Culicidae	Aedes	Aedes aegypti	SPECIES	-6.81000	-51.41000	1264875518
5114	Diptera	Culicidae	Aedes	Aedes aegypti	SPECIES	-6.81000	-37.38000	1264875528
5115	Diptera	Culicidae	Aedes	Aedes aegypti	SPECIES	-6.81000	-35.26000	1264875532
5116	Diptera	Culicidae	Aedes	Aedes aegypti	SPECIES	-6.81000	39.29000	1264889698
5117	Diptera	Culicidae	Aedes	Aedes aegypti	SPECIES	-6.81000	107.00000	1264891271
5118	Diptera	Culicidae	Aedes	Aedes aegypti	SPECIES	-6.80000	-37.95000	1264875511
5119	Diptera	Culicidae	Aedes	Aedes aegypti	SPECIES	-6.80000	39.25000	1264889728
5120	Diptera	Culicidae	Aedes	Aedes aegypti	SPECIES	-6.79000	-37.05000	1264875487
5121	Diptera	Culicidae	Aedes	Aedes aegypti	SPECIES	-6.79000	-61.11000	1264875496
5122	Diptera	Culicidae	Aedes	Aedes aegypti	SPECIES	-6.79000	-42.46000	1264875498
5123	Diptera	Culicidae	Aedes	Aedes aegypti	SPECIES	-6.79000	-41.77000	1264875502
5124	Diptera	Culicidae	Aedes	Aedes aegypti	SPECIES	-6.79000	-35.59000	1264875509
5125	Diptera	Culicidae	Aedes	Aedes aegypti	SPECIES	-6.79000	-41.50000	1264875517

№	Order	Family	Genus	Species	Taxon Rank	Latitude	Longitude	GBIF_ID
5126	Diptera	Culicidae	Aedes	Aedes aegypti	SPECIES	-6.79000	-35.49000	1264875522
5127	Diptera	Culicidae	Aedes	Aedes aegypti	SPECIES	-6.79000	39.23000	1264889755
5128	Diptera	Culicidae	Aedes	Aedes aegypti	SPECIES	-6.79000	108.62000	1264891222
5129	Diptera	Culicidae	Aedes	Aedes aegypti	SPECIES	-6.78000	-38.07000	1264875525
5130	Diptera	Culicidae	Aedes	Aedes aegypti	SPECIES	-6.78000	39.28000	1264888857
5131	Diptera	Culicidae	Aedes	Aedes aegypti	SPECIES	-6.78000	39.27000	1264889723
5132	Diptera	Culicidae	Aedes	Aedes aegypti	SPECIES	-6.77000	110.63000	1264871974
5133	Diptera	Culicidae	Aedes	Aedes aegypti	SPECIES	-6.77000	-47.81000	1264875503
5134	Diptera	Culicidae	Aedes	Aedes aegypti	SPECIES	-6.77000	-41.27000	1264875508
5135	Diptera	Culicidae	Aedes	Aedes aegypti	SPECIES	-6.77000	-40.73000	1264875510
5136	Diptera	Culicidae	Aedes	Aedes aegypti	SPECIES	-6.77000	-37.17000	1264875513
5137	Diptera	Culicidae	Aedes	Aedes aegypti	SPECIES	-6.77000	-44.14000	1264875514
5138	Diptera	Culicidae	Aedes	Aedes aegypti	SPECIES	-6.77000	-35.85000	1264875520
5139	Diptera	Culicidae	Aedes	Aedes aegypti	SPECIES	-6.77000	-36.40000	1264875531
5140	Diptera	Culicidae	Aedes	Aedes aegypti	SPECIES	-6.77000	-36.08000	1264875544
5141	Diptera	Culicidae	Aedes	Aedes aegypti	SPECIES	-6.76000	-39.99000	1264875506
5142	Diptera	Culicidae	Aedes	Aedes aegypti	SPECIES	-6.76000	-50.40000	1264875515
5143	Diptera	Culicidae	Aedes	Aedes aegypti	SPECIES	-6.76000	-47.17000	1264875516
5144	Diptera	Culicidae	Aedes	Aedes aegypti	SPECIES	-6.76000	-37.58000	1264875543
5145	Diptera	Culicidae	Aedes	Aedes aegypti	SPECIES	-6.76000	-38.99000	1264875549
5146	Diptera	Culicidae	Aedes	Aedes aegypti	SPECIES	-6.76000	108.55000	1264891263
5147	Diptera	Culicidae	Aedes	Aedes aegypti	SPECIES	-6.75000	111.05000	1264871755
5148	Diptera	Culicidae	Aedes	Aedes aegypti	SPECIES	-6.75000	-38.45000	1264875526
5149	Diptera	Culicidae	Aedes	Aedes aegypti	SPECIES	-6.75000	-38.23000	1264875536
5150	Diptera	Culicidae	Aedes	Aedes aegypti	SPECIES	-6.75000	-35.01000	1264875553
5151	Diptera	Culicidae	Aedes	Aedes aegypti	SPECIES	-6.75000	39.28000	1264890185
5152	Diptera	Culicidae	Aedes	Aedes aegypti	SPECIES	-6.75000	111.04000	1264890971
5153	Diptera	Culicidae	Aedes	Aedes aegypti	SPECIES	-6.74000	-36.18000	1264875512
5154	Diptera	Culicidae	Aedes	Aedes aegypti	SPECIES	-6.74000	-48.49000	1264875521
5155	Diptera	Culicidae	Aedes	Aedes aegypti	SPECIES	-6.74000	-36.78000	1264875534
5156	Diptera	Culicidae	Aedes	Aedes aegypti	SPECIES	-6.74000	-48.25000	1264875542
5157	Diptera	Culicidae	Aedes	Aedes aegypti	SPECIES	-6.74000	-35.42000	1264875547
5158	Diptera	Culicidae	Aedes	Aedes aegypti	SPECIES	-6.74000	-35.72000	1264875550
5159	Diptera	Culicidae	Aedes	Aedes aegypti	SPECIES	-6.74000	-39.22000	1264875557
5160	Diptera	Culicidae	Aedes	Aedes aegypti	SPECIES	-6.73000	-35.29000	1264875529
5161	Diptera	Culicidae	Aedes	Aedes aegypti	SPECIES	-6.73000	-67.95000	1264875538
5162	Diptera	Culicidae	Aedes	Aedes aegypti	SPECIES	-6.72000	-39.75000	1264875523
5163	Diptera	Culicidae	Aedes	Aedes aegypti	SPECIES	-6.72000	-35.39000	1264875539
5164	Diptera	Culicidae	Aedes	Aedes aegypti	SPECIES	-6.71000	-41.39000	1264875527
5165	Diptera	Culicidae	Aedes	Aedes aegypti	SPECIES	-6.71000	-35.48000	1264875533
5166	Diptera	Culicidae	Aedes	Aedes aegypti	SPECIES	-6.71000	-36.62000	1264875556

№	Order	Family	Genus	Species	Taxon Rank	Latitude	Longitude	GBIF_ID
5167	Diptera	Culicidae	Aedes	Aedes aegypti	SPECIES	-6.71000	-38.75000	1264875570
5168	Diptera	Culicidae	Aedes	Aedes aegypti	SPECIES	-6.71000	-38.58000	1264875574
5169	Diptera	Culicidae	Aedes	Aedes aegypti	SPECIES	-6.70000	-35.16000	1264875530
5170	Diptera	Culicidae	Aedes	Aedes aegypti	SPECIES	-6.70000	-35.61000	1264875541
5171	Diptera	Culicidae	Aedes	Aedes aegypti	SPECIES	-6.70000	-35.46000	1264875546
5172	Diptera	Culicidae	Aedes	Aedes aegypti	SPECIES	-6.69000	-37.17000	1264875552
5173	Diptera	Culicidae	Aedes	Aedes aegypti	SPECIES	-6.69000	39.27000	1264889617
5174	Diptera	Culicidae	Aedes	Aedes aegypti	SPECIES	-6.68014	-36.41909	3302332639
5175	Diptera	Culicidae	Aedes	Aedes aegypti	SPECIES	-6.68008	106.93233	3993822303
5176	Diptera	Culicidae	Aedes	Aedes aegypti	SPECIES	-6.68000	-35.37000	1264875540
5177	Diptera	Culicidae	Aedes	Aedes aegypti	SPECIES	-6.68000	-41.70000	1264875562
5178	Diptera	Culicidae	Aedes	Aedes aegypti	SPECIES	-6.68000	-36.91000	1264875568
5179	Diptera	Culicidae	Aedes	Aedes aegypti	SPECIES	-6.68000	-42.73000	1264875572
5180	Diptera	Culicidae	Aedes	Aedes aegypti	SPECIES	-6.68000	108.55000	1264876469
5181	Diptera	Culicidae	Aedes	Aedes aegypti	SPECIES	-6.67000	-36.42000	1264875559
5182	Diptera	Culicidae	Aedes	Aedes aegypti	SPECIES	-6.67000	-35.29000	1264875560
5183	Diptera	Culicidae	Aedes	Aedes aegypti	SPECIES	-6.66000	-34.98000	1264875535
5184	Diptera	Culicidae	Aedes	Aedes aegypti	SPECIES	-6.66000	-39.51000	1264875580
5185	Diptera	Culicidae	Aedes	Aedes aegypti	SPECIES	-6.65000	-42.48000	1264875537
5186	Diptera	Culicidae	Aedes	Aedes aegypti	SPECIES	-6.65000	-44.15000	1264875545
5187	Diptera	Culicidae	Aedes	Aedes aegypti	SPECIES	-6.65000	39.25000	1264890171
5188	Diptera	Culicidae	Aedes	Aedes aegypti	SPECIES	-6.64000	-42.26000	1264875567
5189	Diptera	Culicidae	Aedes	Aedes aegypti	SPECIES	-6.64000	-35.94000	1264875575
5190	Diptera	Culicidae	Aedes	Aedes aegypti	SPECIES	-6.64000	-38.04000	1264875578
5191	Diptera	Culicidae	Aedes	Aedes aegypti	SPECIES	-6.64000	106.24000	1264891255
5192	Diptera	Culicidae	Aedes	Aedes aegypti	SPECIES	-6.62000	110.69000	1264871788
5193	Diptera	Culicidae	Aedes	Aedes aegypti	SPECIES	-6.62000	-35.81000	1264875548
5194	Diptera	Culicidae	Aedes	Aedes aegypti	SPECIES	-6.62000	-36.27000	1264875554
5195	Diptera	Culicidae	Aedes	Aedes aegypti	SPECIES	-6.62000	-38.74000	1264875565
5196	Diptera	Culicidae	Aedes	Aedes aegypti	SPECIES	-6.62000	-37.63000	1264875598
5197	Diptera	Culicidae	Aedes	Aedes aegypti	SPECIES	-6.61000	106.78000	1264871887
5198	Diptera	Culicidae	Aedes	Aedes aegypti	SPECIES	-6.61000	-35.65000	1264875558
5199	Diptera	Culicidae	Aedes	Aedes aegypti	SPECIES	-6.60000	-48.46000	1264875551
5200	Diptera	Culicidae	Aedes	Aedes aegypti	SPECIES	-6.60000	-38.57000	1264875555
5201	Diptera	Culicidae	Aedes	Aedes aegypti	SPECIES	-6.60000	-36.81000	1264875571
5202	Diptera	Culicidae	Aedes	Aedes aegypti	SPECIES	-6.60000	-35.27000	1264875595
5203	Diptera	Culicidae	Aedes	Aedes aegypti	SPECIES	-6.59000	110.67000	1264871899
5204	Diptera	Culicidae	Aedes	Aedes aegypti	SPECIES	-6.59000	-37.88000	1264875564
5205	Diptera	Culicidae	Aedes	Aedes aegypti	SPECIES	-6.59000	-38.49000	1264875566
5206	Diptera	Culicidae	Aedes	Aedes aegypti	SPECIES	-6.59000	-47.67000	1264875577
5207	Diptera	Culicidae	Aedes	Aedes aegypti	SPECIES	-6.59000	-43.16000	1264875582

№	Order	Family	Genus	Species	Taxon Rank	Latitude	Longitude	GBIF_ID
5208	Diptera	Culicidae	Aedes	Aedes aegypti	SPECIES	-6.59000	-40.37000	1264875593
5209	Diptera	Culicidae	Aedes	Aedes aegypti	SPECIES	-6.59000	106.79000	1264890697
5210	Diptera	Culicidae	Aedes	Aedes aegypti	SPECIES	-6.58000	110.68000	1264871903
5211	Diptera	Culicidae	Aedes	Aedes aegypti	SPECIES	-6.58000	-46.14000	1264875573
5212	Diptera	Culicidae	Aedes	Aedes aegypti	SPECIES	-6.58000	-39.11000	1264875581
5213	Diptera	Culicidae	Aedes	Aedes aegypti	SPECIES	-6.58000	-35.42000	1264875585
5214	Diptera	Culicidae	Aedes	Aedes aegypti	SPECIES	-6.58000	106.74000	1264876467
5215	Diptera	Culicidae	Aedes	Aedes aegypti	SPECIES	-6.58000	105.68000	1264891268
5216	Diptera	Culicidae	Aedes	Aedes aegypti	SPECIES	-6.57000	-37.35000	1264875563
5217	Diptera	Culicidae	Aedes	Aedes aegypti	SPECIES	-6.57000	-43.63000	1264875600
5218	Diptera	Culicidae	Aedes	Aedes aegypti	SPECIES	-6.56000	110.67000	1264871922
5219	Diptera	Culicidae	Aedes	Aedes aegypti	SPECIES	-6.56000	106.73000	1264871926
5220	Diptera	Culicidae	Aedes	Aedes aegypti	SPECIES	-6.56000	-35.05000	1264875587
5221	Diptera	Culicidae	Aedes	Aedes aegypti	SPECIES	-6.56000	-38.27000	1264875597
5222	Diptera	Culicidae	Aedes	Aedes aegypti	SPECIES	-6.56000	-35.43000	1264875603
5223	Diptera	Culicidae	Aedes	Aedes aegypti	SPECIES	-6.55000	106.73000	1264871749
5224	Diptera	Culicidae	Aedes	Aedes aegypti	SPECIES	-6.55000	106.79000	1264871750
5225	Diptera	Culicidae	Aedes	Aedes aegypti	SPECIES	-6.55000	-36.56000	1264875569
5226	Diptera	Culicidae	Aedes	Aedes aegypti	SPECIES	-6.55000	-35.54000	1264875583
5227	Diptera	Culicidae	Aedes	Aedes aegypti	SPECIES	-6.55000	-42.23000	1264875591
5228	Diptera	Culicidae	Aedes	Aedes aegypti	SPECIES	-6.55000	-38.18000	1264875605
5229	Diptera	Culicidae	Aedes	Aedes aegypti	SPECIES	-6.55000	-49.02000	1264875606
5230	Diptera	Culicidae	Aedes	Aedes aegypti	SPECIES	-6.55000	110.77000	1264890968
5231	Diptera	Culicidae	Aedes	Aedes aegypti	SPECIES	-6.54000	-42.10000	1264875561
5232	Diptera	Culicidae	Aedes	Aedes aegypti	SPECIES	-6.54000	-36.05000	1264875576
5233	Diptera	Culicidae	Aedes	Aedes aegypti	SPECIES	-6.54000	-37.73000	1264875590
5234	Diptera	Culicidae	Aedes	Aedes aegypti	SPECIES	-6.54000	-35.64000	1264875596
5235	Diptera	Culicidae	Aedes	Aedes aegypti	SPECIES	-6.53000	-35.75000	1264875584
5236	Diptera	Culicidae	Aedes	Aedes aegypti	SPECIES	-6.53000	-38.06000	1264875623
5237	Diptera	Culicidae	Aedes	Aedes aegypti	SPECIES	-6.52000	110.72000	1264871762
5238	Diptera	Culicidae	Aedes	Aedes aegypti	SPECIES	-6.52000	-37.82000	1264875579
5239	Diptera	Culicidae	Aedes	Aedes aegypti	SPECIES	-6.51000	-76.38000	1264890962
5240	Diptera	Culicidae	Aedes	Aedes aegypti	SPECIES	-6.50000	-35.28000	1264875589
5241	Diptera	Culicidae	Aedes	Aedes aegypti	SPECIES	-6.50000	-38.39000	1264875592
5242	Diptera	Culicidae	Aedes	Aedes aegypti	SPECIES	-6.50000	-44.34000	1264875602
5243	Diptera	Culicidae	Aedes	Aedes aegypti	SPECIES	-6.50000	-41.48000	1264875619
5244	Diptera	Culicidae	Aedes	Aedes aegypti	SPECIES	-6.50000	-47.53000	1264875620
5245	Diptera	Culicidae	Aedes	Aedes aegypti	SPECIES	-6.49000	-36.20000	1264875588
5246	Diptera	Culicidae	Aedes	Aedes aegypti	SPECIES	-6.49000	-47.72000	1264875594
5247	Diptera	Culicidae	Aedes	Aedes aegypti	SPECIES	-6.49000	-36.35000	1264875622
5248	Diptera	Culicidae	Aedes	Aedes aegypti	SPECIES	-6.49000	-37.25000	1264875629

№	Order	Family	Genus	Species	Taxon Rank	Latitude	Longitude	GBIF_ID
5249	Diptera	Culicidae	Aedes	Aedes aegypti	SPECIES	-6.48000	-38.56000	1264875586
5250	Diptera	Culicidae	Aedes	Aedes aegypti	SPECIES	-6.48000	-48.15000	1264875599
5251	Diptera	Culicidae	Aedes	Aedes aegypti	SPECIES	-6.48000	-36.86000	1264875607
5252	Diptera	Culicidae	Aedes	Aedes aegypti	SPECIES	-6.48000	-41.99000	1264875610
5253	Diptera	Culicidae	Aedes	Aedes aegypti	SPECIES	-6.48000	-50.11000	1264875615
5254	Diptera	Culicidae	Aedes	Aedes aegypti	SPECIES	-6.48000	-76.37000	1264887656
5255	Diptera	Culicidae	Aedes	Aedes aegypti	SPECIES	-6.47000	110.72000	1264871765
5256	Diptera	Culicidae	Aedes	Aedes aegypti	SPECIES	-6.47000	-43.50000	1264875609
5257	Diptera	Culicidae	Aedes	Aedes aegypti	SPECIES	-6.47000	-37.62000	1264875612
5258	Diptera	Culicidae	Aedes	Aedes aegypti	SPECIES	-6.47000	-37.95000	1264875624
5259	Diptera	Culicidae	Aedes	Aedes aegypti	SPECIES	-6.47000	-39.59000	1264875627
5260	Diptera	Culicidae	Aedes	Aedes aegypti	SPECIES	-6.47000	-38.49000	1264875649
5261	Diptera	Culicidae	Aedes	Aedes aegypti	SPECIES	-6.47000	-37.10000	1264889460
5262	Diptera	Culicidae	Aedes	Aedes aegypti	SPECIES	-6.46000	-35.23000	1264875601
5263	Diptera	Culicidae	Aedes	Aedes aegypti	SPECIES	-6.46000	-37.48000	1264875608
5264	Diptera	Culicidae	Aedes	Aedes aegypti	SPECIES	-6.46000	-35.44000	1264875616
5265	Diptera	Culicidae	Aedes	Aedes aegypti	SPECIES	-6.46000	-39.90000	1264875621
5266	Diptera	Culicidae	Aedes	Aedes aegypti	SPECIES	-6.46000	-37.05000	1264875642
5267	Diptera	Culicidae	Aedes	Aedes aegypti	SPECIES	-6.45000	-38.29000	1264875617
5268	Diptera	Culicidae	Aedes	Aedes aegypti	SPECIES	-6.45000	-35.62000	1264875626
5269	Diptera	Culicidae	Aedes	Aedes aegypti	SPECIES	-6.44000	-38.16000	1264875604
5270	Diptera	Culicidae	Aedes	Aedes aegypti	SPECIES	-6.44000	-46.91000	1264875631
5271	Diptera	Culicidae	Aedes	Aedes aegypti	SPECIES	-6.43000	-35.81000	1264875614
5272	Diptera	Culicidae	Aedes	Aedes aegypti	SPECIES	-6.42000	-42.24000	1264875611
5273	Diptera	Culicidae	Aedes	Aedes aegypti	SPECIES	-6.42000	-35.87000	1264875613
5274	Diptera	Culicidae	Aedes	Aedes aegypti	SPECIES	-6.42000	-35.05000	1264875618
5275	Diptera	Culicidae	Aedes	Aedes aegypti	SPECIES	-6.42000	-35.72000	1264875625
5276	Diptera	Culicidae	Aedes	Aedes aegypti	SPECIES	-6.42000	-35.15000	1264875636
5277	Diptera	Culicidae	Aedes	Aedes aegypti	SPECIES	-6.42000	-47.91000	1264875645
5278	Diptera	Culicidae	Aedes	Aedes aegypti	SPECIES	-6.42000	-36.64000	1264875647
5279	Diptera	Culicidae	Aedes	Aedes aegypti	SPECIES	-6.42000	-36.43000	1264875654
5280	Diptera	Culicidae	Aedes	Aedes aegypti	SPECIES	-6.42000	-79.83000	1264891041
5281	Diptera	Culicidae	Aedes	Aedes aegypti	SPECIES	-6.41000	-43.94000	1264875646
5282	Diptera	Culicidae	Aedes	Aedes aegypti	SPECIES	-6.41000	-38.31000	1264875674
5283	Diptera	Culicidae	Aedes	Aedes aegypti	SPECIES	-6.40000	-42.79000	1264875633
5284	Diptera	Culicidae	Aedes	Aedes aegypti	SPECIES	-6.40000	-45.01000	1264875634
5285	Diptera	Culicidae	Aedes	Aedes aegypti	SPECIES	-6.40000	-41.26000	1264875648
5286	Diptera	Culicidae	Aedes	Aedes aegypti	SPECIES	-6.40000	-36.24000	1264875652
5287	Diptera	Culicidae	Aedes	Aedes aegypti	SPECIES	-6.39000	-37.86000	1264875628
5288	Diptera	Culicidae	Aedes	Aedes aegypti	SPECIES	-6.39000	-36.05000	1264875632
5289	Diptera	Culicidae	Aedes	Aedes aegypti	SPECIES	-6.39000	-41.78000	1264875635

№	Order	Family	Genus	Species	Taxon Rank	Latitude	Longitude	GBIF_ID
5290	Diptera	Culicidae	Aedes	Aedes aegypti	SPECIES	-6.39000	-38.53000	1264875667
5291	Diptera	Culicidae	Aedes	Aedes aegypti	SPECIES	-6.39000	106.82000	1264876472
5292	Diptera	Culicidae	Aedes	Aedes aegypti	SPECIES	-6.38785	106.81780	3901425513
5293	Diptera	Culicidae	Aedes	Aedes aegypti	SPECIES	-6.38000	-38.41000	1264875637
5294	Diptera	Culicidae	Aedes	Aedes aegypti	SPECIES	-6.38000	-47.11000	1264875641
5295	Diptera	Culicidae	Aedes	Aedes aegypti	SPECIES	-6.38000	-37.83000	1264888088
5296	Diptera	Culicidae	Aedes	Aedes aegypti	SPECIES	-6.37000	-35.61000	1264875640
5297	Diptera	Culicidae	Aedes	Aedes aegypti	SPECIES	-6.37000	-37.98000	1264875651
5298	Diptera	Culicidae	Aedes	Aedes aegypti	SPECIES	-6.36000	107.18000	1264871763
5299	Diptera	Culicidae	Aedes	Aedes aegypti	SPECIES	-6.36000	-36.83000	1264875644
5300	Diptera	Culicidae	Aedes	Aedes aegypti	SPECIES	-6.36000	-38.83000	1264875657
5301	Diptera	Culicidae	Aedes	Aedes aegypti	SPECIES	-6.36000	-36.20000	1264875672
5302	Diptera	Culicidae	Aedes	Aedes aegypti	SPECIES	-6.36000	-79.82000	1264891085
5303	Diptera	Culicidae	Aedes	Aedes aegypti	SPECIES	-6.35000	-39.26000	1264875630
5304	Diptera	Culicidae	Aedes	Aedes aegypti	SPECIES	-6.35000	-38.28000	1264875639
5305	Diptera	Culicidae	Aedes	Aedes aegypti	SPECIES	-6.35000	-42.50000	1264875671
5306	Diptera	Culicidae	Aedes	Aedes aegypti	SPECIES	-6.33000	-37.71000	1264875643
5307	Diptera	Culicidae	Aedes	Aedes aegypti	SPECIES	-6.33000	-37.47000	1264875680
5308	Diptera	Culicidae	Aedes	Aedes aegypti	SPECIES	-6.32000	-35.52000	1264875638
5309	Diptera	Culicidae	Aedes	Aedes aegypti	SPECIES	-6.32000	-53.64000	1264875650
5310	Diptera	Culicidae	Aedes	Aedes aegypti	SPECIES	-6.32000	-47.76000	1264875660
5311	Diptera	Culicidae	Aedes	Aedes aegypti	SPECIES	-6.32000	-38.52000	1264875662
5312	Diptera	Culicidae	Aedes	Aedes aegypti	SPECIES	-6.32000	-35.36000	1264875669
5313	Diptera	Culicidae	Aedes	Aedes aegypti	SPECIES	-6.31000	-37.15000	1264875677
5314	Diptera	Culicidae	Aedes	Aedes aegypti	SPECIES	-6.30000	-35.31000	1264875653
5315	Diptera	Culicidae	Aedes	Aedes aegypti	SPECIES	-6.30000	-37.26000	1264875658
5316	Diptera	Culicidae	Aedes	Aedes aegypti	SPECIES	-6.30000	-40.59000	1264875659
5317	Diptera	Culicidae	Aedes	Aedes aegypti	SPECIES	-6.30000	-35.76000	1264875673
5318	Diptera	Culicidae	Aedes	Aedes aegypti	SPECIES	-6.30000	-35.08000	1264875702
5319	Diptera	Culicidae	Aedes	Aedes aegypti	SPECIES	-6.30000	106.78000	1264889516
5320	Diptera	Culicidae	Aedes	Aedes aegypti	SPECIES	-6.29000	106.79000	1264871757
5321	Diptera	Culicidae	Aedes	Aedes aegypti	SPECIES	-6.29000	-38.03000	1264875656
5322	Diptera	Culicidae	Aedes	Aedes aegypti	SPECIES	-6.29000	-37.83000	1264875664
5323	Diptera	Culicidae	Aedes	Aedes aegypti	SPECIES	-6.29000	-35.22000	1264875676
5324	Diptera	Culicidae	Aedes	Aedes aegypti	SPECIES	-6.28000	-35.39000	1264875655
5325	Diptera	Culicidae	Aedes	Aedes aegypti	SPECIES	-6.28000	-38.33000	1264875663
5326	Diptera	Culicidae	Aedes	Aedes aegypti	SPECIES	-6.28000	-38.16000	1264875682
5327	Diptera	Culicidae	Aedes	Aedes aegypti	SPECIES	-6.27000	106.80000	1264871604
5328	Diptera	Culicidae	Aedes	Aedes aegypti	SPECIES	-6.27000	106.71000	1264871726
5329	Diptera	Culicidae	Aedes	Aedes aegypti	SPECIES	-6.27000	-36.01000	1264875665
5330	Diptera	Culicidae	Aedes	Aedes aegypti	SPECIES	-6.27000	-46.63000	1264875666

№	Order	Family	Genus	Species	Taxon Rank	Latitude	Longitude	GBIF_ID
5331	Diptera	Culicidae	Aedes	Aedes aegypti	SPECIES	-6.27000	106.87000	1264876451
5332	Diptera	Culicidae	Aedes	Aedes aegypti	SPECIES	-6.26905	106.82322	3335120634
5333	Diptera	Culicidae	Aedes	Aedes aegypti	SPECIES	-6.26000	106.86000	1264871626
5334	Diptera	Culicidae	Aedes	Aedes aegypti	SPECIES	-6.26000	-35.57000	1264875693
5335	Diptera	Culicidae	Aedes	Aedes aegypti	SPECIES	-6.25000	107.09000	1264871739
5336	Diptera	Culicidae	Aedes	Aedes aegypti	SPECIES	-6.25000	-49.62000	1264875668
5337	Diptera	Culicidae	Aedes	Aedes aegypti	SPECIES	-6.25000	-38.42000	1264875670
5338	Diptera	Culicidae	Aedes	Aedes aegypti	SPECIES	-6.25000	-35.34000	1264875698
5339	Diptera	Culicidae	Aedes	Aedes aegypti	SPECIES	-6.25000	107.14000	1264889106
5340	Diptera	Culicidae	Aedes	Aedes aegypti	SPECIES	-6.24000	106.78000	1264871740
5341	Diptera	Culicidae	Aedes	Aedes aegypti	SPECIES	-6.24000	106.80000	1264871754
5342	Diptera	Culicidae	Aedes	Aedes aegypti	SPECIES	-6.24000	107.05000	1264871764
5343	Diptera	Culicidae	Aedes	Aedes aegypti	SPECIES	-6.24000	107.02000	1264871774
5344	Diptera	Culicidae	Aedes	Aedes aegypti	SPECIES	-6.24000	-38.97000	1264875661
5345	Diptera	Culicidae	Aedes	Aedes aegypti	SPECIES	-6.24000	-39.94000	1264875684
5346	Diptera	Culicidae	Aedes	Aedes aegypti	SPECIES	-6.24000	-37.36000	1264875685
5347	Diptera	Culicidae	Aedes	Aedes aegypti	SPECIES	-6.24000	-36.21000	1264875687
5348	Diptera	Culicidae	Aedes	Aedes aegypti	SPECIES	-6.24000	-36.44000	1264875696
5349	Diptera	Culicidae	Aedes	Aedes aegypti	SPECIES	-6.24000	-40.15000	1264875701
5350	Diptera	Culicidae	Aedes	Aedes aegypti	SPECIES	-6.24000	-35.10000	1264875728
5351	Diptera	Culicidae	Aedes	Aedes aegypti	SPECIES	-6.24000	106.83000	1264876485
5352	Diptera	Culicidae	Aedes	Aedes aegypti	SPECIES	-6.24000	-38.94000	1264891120
5353	Diptera	Culicidae	Aedes	Aedes aegypti	SPECIES	-6.23000	106.57000	1264871785
5354	Diptera	Culicidae	Aedes	Aedes aegypti	SPECIES	-6.23000	-47.55000	1264875694
5355	Diptera	Culicidae	Aedes	Aedes aegypti	SPECIES	-6.22975	106.82952	4017654670
5356	Diptera	Culicidae	Aedes	Aedes aegypti	SPECIES	-6.22000	-35.82000	1264875675
5357	Diptera	Culicidae	Aedes	Aedes aegypti	SPECIES	-6.22000	-35.39000	1264875708
5358	Diptera	Culicidae	Aedes	Aedes aegypti	SPECIES	-6.22000	106.87000	1264887912
5359	Diptera	Culicidae	Aedes	Aedes aegypti	SPECIES	-6.22000	106.93000	1264891093
5360	Diptera	Culicidae	Aedes	Aedes aegypti	SPECIES	-6.21413	106.84273	3993826063
5361	Diptera	Culicidae	Aedes	Aedes aegypti	SPECIES	-6.21000	106.84000	1264871627
5362	Diptera	Culicidae	Aedes	Aedes aegypti	SPECIES	-6.21000	-47.85000	1264875679
5363	Diptera	Culicidae	Aedes	Aedes aegypti	SPECIES	-6.21000	-37.92000	1264875681
5364	Diptera	Culicidae	Aedes	Aedes aegypti	SPECIES	-6.21000	-38.46000	1264875697
5365	Diptera	Culicidae	Aedes	Aedes aegypti	SPECIES	-6.21000	-43.12000	1264875699
5366	Diptera	Culicidae	Aedes	Aedes aegypti	SPECIES	-6.21000	-38.31000	1264875716
5367	Diptera	Culicidae	Aedes	Aedes aegypti	SPECIES	-6.21000	106.66000	1264889491
5368	Diptera	Culicidae	Aedes	Aedes aegypti	SPECIES	-6.21000	106.85000	1264889564
5369	Diptera	Culicidae	Aedes	Aedes aegypti	SPECIES	-6.21000	106.87000	1264891306
5370	Diptera	Culicidae	Aedes	Aedes aegypti	SPECIES	-6.20417	106.84528	4017666198
5371	Diptera	Culicidae	Aedes	Aedes aegypti	SPECIES	-6.20000	39.20000	1100304655

№	Order	Family	Genus	Species	Taxon Rank	Latitude	Longitude	GBIF_ID
5372	Diptera	Culicidae	Aedes	Aedes aegypti	SPECIES	-6.20000	106.86000	1264871645
5373	Diptera	Culicidae	Aedes	Aedes aegypti	SPECIES	-6.20000	-38.22000	1264875678
5374	Diptera	Culicidae	Aedes	Aedes aegypti	SPECIES	-6.20000	-42.20000	1264875689
5375	Diptera	Culicidae	Aedes	Aedes aegypti	SPECIES	-6.20000	-35.20000	1264875691
5376	Diptera	Culicidae	Aedes	Aedes aegypti	SPECIES	-6.20000	-36.67000	1264875692
5377	Diptera	Culicidae	Aedes	Aedes aegypti	SPECIES	-6.20000	-50.47000	1264875709
5378	Diptera	Culicidae	Aedes	Aedes aegypti	SPECIES	-6.20000	106.87000	1264889519
5379	Diptera	Culicidae	Aedes	Aedes aegypti	SPECIES	-6.20000	106.83000	1264891013
5380	Diptera	Culicidae	Aedes	Aedes aegypti	SPECIES	-6.20000	106.63000	1264891301
5381	Diptera	Culicidae	Aedes	Aedes aegypti	SPECIES	-6.19000	106.81000	1264871589
5382	Diptera	Culicidae	Aedes	Aedes aegypti	SPECIES	-6.19000	106.88000	1264871625
5383	Diptera	Culicidae	Aedes	Aedes aegypti	SPECIES	-6.19000	106.86000	1264871644
5384	Diptera	Culicidae	Aedes	Aedes aegypti	SPECIES	-6.19000	106.85000	1264871813
5385	Diptera	Culicidae	Aedes	Aedes aegypti	SPECIES	-6.19000	-48.19000	1264875695
5386	Diptera	Culicidae	Aedes	Aedes aegypti	SPECIES	-6.19000	-35.47000	1264875733
5387	Diptera	Culicidae	Aedes	Aedes aegypti	SPECIES	-6.19000	106.83000	1264876533
5388	Diptera	Culicidae	Aedes	Aedes aegypti	SPECIES	-6.18173	39.20319	2563432579
5389	Diptera	Culicidae	Aedes	Aedes aegypti	SPECIES	-6.18000	106.85000	1264871585
5390	Diptera	Culicidae	Aedes	Aedes aegypti	SPECIES	-6.18000	106.87000	1264871893
5391	Diptera	Culicidae	Aedes	Aedes aegypti	SPECIES	-6.18000	-47.24000	1264875683
5392	Diptera	Culicidae	Aedes	Aedes aegypti	SPECIES	-6.18000	-36.83000	1264875722
5393	Diptera	Culicidae	Aedes	Aedes aegypti	SPECIES	-6.18000	106.86000	1264889552
5394	Diptera	Culicidae	Aedes	Aedes aegypti	SPECIES	-6.18000	35.74000	1264889866
5395	Diptera	Culicidae	Aedes	Aedes aegypti	SPECIES	-6.18000	106.83000	1264890668
5396	Diptera	Culicidae	Aedes	Aedes aegypti	SPECIES	-6.17593	106.82703	3993821302
5397	Diptera	Culicidae	Aedes	Aedes aegypti	SPECIES	-6.17000	-48.79000	1264875724
5398	Diptera	Culicidae	Aedes	Aedes aegypti	SPECIES	-6.17000	35.74000	1264888795
5399	Diptera	Culicidae	Aedes	Aedes aegypti	SPECIES	-6.17000	106.83000	1264890639
5400	Diptera	Culicidae	Aedes	Aedes aegypti	SPECIES	-6.16667	106.81667	177195178
5401	Diptera	Culicidae	Aedes	Aedes aegypti	SPECIES	-6.16667	106.81667	1633663662
5402	Diptera	Culicidae	Aedes	Aedes aegypti	SPECIES	-6.16000	106.79000	1264871622
5403	Diptera	Culicidae	Aedes	Aedes aegypti	SPECIES	-6.16000	-36.72000	1264875686
5404	Diptera	Culicidae	Aedes	Aedes aegypti	SPECIES	-6.16000	-37.75000	1264875688
5405	Diptera	Culicidae	Aedes	Aedes aegypti	SPECIES	-6.16000	-35.13000	1264875700
5406	Diptera	Culicidae	Aedes	Aedes aegypti	SPECIES	-6.16000	-37.84000	1264875712
5407	Diptera	Culicidae	Aedes	Aedes aegypti	SPECIES	-6.16000	106.81000	1264888702
5408	Diptera	Culicidae	Aedes	Aedes aegypti	SPECIES	-6.16000	106.80000	1264889539
5409	Diptera	Culicidae	Aedes	Aedes aegypti	SPECIES	-6.15278	106.83250	4017665829
5410	Diptera	Culicidae	Aedes	Aedes aegypti	SPECIES	-6.15000	106.83000	1264871628
5411	Diptera	Culicidae	Aedes	Aedes aegypti	SPECIES	-6.15000	-37.39000	1264875690
5412	Diptera	Culicidae	Aedes	Aedes aegypti	SPECIES	-6.15000	-35.50000	1264875706

№	Order	Family	Genus	Species	Taxon Rank	Latitude	Longitude	GBIF_ID
5413	Diptera	Culicidae	Aedes	Aedes aegypti	SPECIES	-6.15000	-36.15000	1264875717
5414	Diptera	Culicidae	Aedes	Aedes aegypti	SPECIES	-6.15000	-39.47000	1264875754
5415	Diptera	Culicidae	Aedes	Aedes aegypti	SPECIES	-6.15000	106.84000	1264889518
5416	Diptera	Culicidae	Aedes	Aedes aegypti	SPECIES	-6.14573	106.79502	3993826487
5417	Diptera	Culicidae	Aedes	Aedes aegypti	SPECIES	-6.14000	106.80000	1264871617
5418	Diptera	Culicidae	Aedes	Aedes aegypti	SPECIES	-6.14000	-42.51000	1264875707
5419	Diptera	Culicidae	Aedes	Aedes aegypti	SPECIES	-6.14000	-37.99000	1264875710
5420	Diptera	Culicidae	Aedes	Aedes aegypti	SPECIES	-6.14000	-39.05000	1264875726
5421	Diptera	Culicidae	Aedes	Aedes aegypti	SPECIES	-6.13243	106.87133	3993820001
5422	Diptera	Culicidae	Aedes	Aedes aegypti	SPECIES	-6.13228	106.87239	3993817849
5423	Diptera	Culicidae	Aedes	Aedes aegypti	SPECIES	-6.13000	106.87000	1264871666
5424	Diptera	Culicidae	Aedes	Aedes aegypti	SPECIES	-6.13000	-38.31000	1264875720
5425	Diptera	Culicidae	Aedes	Aedes aegypti	SPECIES	-6.13000	-35.64000	1264875742
5426	Diptera	Culicidae	Aedes	Aedes aegypti	SPECIES	-6.13000	106.88000	1264876500
5427	Diptera	Culicidae	Aedes	Aedes aegypti	SPECIES	-6.13000	106.82000	1264890786
5428	Diptera	Culicidae	Aedes	Aedes aegypti	SPECIES	-6.13000	106.75000	1264891214
5429	Diptera	Culicidae	Aedes	Aedes aegypti	SPECIES	-6.12718	106.79865	3993825172
5430	Diptera	Culicidae	Aedes	Aedes aegypti	SPECIES	-6.12677	106.88631	3993825099
5431	Diptera	Culicidae	Aedes	Aedes aegypti	SPECIES	-6.12000	-49.21000	1264875704
5432	Diptera	Culicidae	Aedes	Aedes aegypti	SPECIES	-6.12000	-43.76000	1264875715
5433	Diptera	Culicidae	Aedes	Aedes aegypti	SPECIES	-6.12000	-38.40000	1264875735
5434	Diptera	Culicidae	Aedes	Aedes aegypti	SPECIES	-6.12000	106.87000	1264889496
5435	Diptera	Culicidae	Aedes	Aedes aegypti	SPECIES	-6.11000	-37.83000	1264875713
5436	Diptera	Culicidae	Aedes	Aedes aegypti	SPECIES	-6.11000	-35.42000	1264875719
5437	Diptera	Culicidae	Aedes	Aedes aegypti	SPECIES	-6.11000	-35.71000	1264875721
5438	Diptera	Culicidae	Aedes	Aedes aegypti	SPECIES	-6.11000	106.90000	1264876457
5439	Diptera	Culicidae	Aedes	Aedes aegypti	SPECIES	-6.11000	106.88000	1264876478
5440	Diptera	Culicidae	Aedes	Aedes aegypti	SPECIES	-6.11000	35.89000	1264890215
5441	Diptera	Culicidae	Aedes	Aedes aegypti	SPECIES	-6.10000	-47.84000	1264875703
5442	Diptera	Culicidae	Aedes	Aedes aegypti	SPECIES	-6.10000	-46.95000	1264875705
5443	Diptera	Culicidae	Aedes	Aedes aegypti	SPECIES	-6.10000	-38.16000	1264875714
5444	Diptera	Culicidae	Aedes	Aedes aegypti	SPECIES	-6.09000	-36.52000	1264875723
5445	Diptera	Culicidae	Aedes	Aedes aegypti	SPECIES	-6.09000	-37.91000	1264875730
5446	Diptera	Culicidae	Aedes	Aedes aegypti	SPECIES	-6.09000	-42.75000	1264875734
5447	Diptera	Culicidae	Aedes	Aedes aegypti	SPECIES	-6.09000	-35.93000	1264875748
5448	Diptera	Culicidae	Aedes	Aedes aegypti	SPECIES	-6.08000	-35.30000	1264875737
5449	Diptera	Culicidae	Aedes	Aedes aegypti	SPECIES	-6.08000	-44.25000	1264875741
5450	Diptera	Culicidae	Aedes	Aedes aegypti	SPECIES	-6.08000	-37.41000	1264875753
5451	Diptera	Culicidae	Aedes	Aedes aegypti	SPECIES	-6.07000	-37.62000	1264875711
5452	Diptera	Culicidae	Aedes	Aedes aegypti	SPECIES	-6.07000	-37.73000	1264875725
5453	Diptera	Culicidae	Aedes	Aedes aegypti	SPECIES	-6.07000	106.15000	1264891292

№	Order	Family	Genus	Species	Taxon Rank	Latitude	Longitude	GBIF_ID
5454	Diptera	Culicidae	Aedes	Aedes aegypti	SPECIES	-6.05000	-35.17000	1264875744
5455	Diptera	Culicidae	Aedes	Aedes aegypti	SPECIES	-6.05000	-43.52000	1264875779
5456	Diptera	Culicidae	Aedes	Aedes aegypti	SPECIES	-6.04000	106.53000	1264871662
5457	Diptera	Culicidae	Aedes	Aedes aegypti	SPECIES	-6.04000	-35.68000	1264875718
5458	Diptera	Culicidae	Aedes	Aedes aegypti	SPECIES	-6.04000	-38.10000	1264875729
5459	Diptera	Culicidae	Aedes	Aedes aegypti	SPECIES	-6.04000	-42.49000	1264875750
5460	Diptera	Culicidae	Aedes	Aedes aegypti	SPECIES	-6.03345	106.73498	3993826500
5461	Diptera	Culicidae	Aedes	Aedes aegypti	SPECIES	-6.03000	-35.46000	1264875731
5462	Diptera	Culicidae	Aedes	Aedes aegypti	SPECIES	-6.03000	-56.44000	1264875732
5463	Diptera	Culicidae	Aedes	Aedes aegypti	SPECIES	-6.03000	-37.01000	1264875746
5464	Diptera	Culicidae	Aedes	Aedes aegypti	SPECIES	-6.03000	-38.02000	1264875758
5465	Diptera	Culicidae	Aedes	Aedes aegypti	SPECIES	-6.02000	-42.66000	1264875727
5466	Diptera	Culicidae	Aedes	Aedes aegypti	SPECIES	-6.02000	-47.60000	1264875739
5467	Diptera	Culicidae	Aedes	Aedes aegypti	SPECIES	-6.01000	-38.50000	1264875740
5468	Diptera	Culicidae	Aedes	Aedes aegypti	SPECIES	-6.01000	-35.85000	1264875747
5469	Diptera	Culicidae	Aedes	Aedes aegypti	SPECIES	-6.01000	-35.59000	1264875760
5470	Diptera	Culicidae	Aedes	Aedes aegypti	SPECIES	-6.00000	-37.48000	1264875766
5471	Diptera	Culicidae	Aedes	Aedes aegypti	SPECIES	-6.00000	-37.83000	1264875775
5472	Diptera	Culicidae	Aedes	Aedes aegypti	SPECIES	-6.00000	-36.13000	1264875787
5473	Diptera	Culicidae	Aedes	Aedes aegypti	SPECIES	-5.99000	-38.18000	1264875743
5474	Diptera	Culicidae	Aedes	Aedes aegypti	SPECIES	-5.99000	-37.96000	1264875745
5475	Diptera	Culicidae	Aedes	Aedes aegypti	SPECIES	-5.98000	-35.93000	1264875738
5476	Diptera	Culicidae	Aedes	Aedes aegypti	SPECIES	-5.98000	-36.34000	1264875756
5477	Diptera	Culicidae	Aedes	Aedes aegypti	SPECIES	-5.98000	-38.29000	1264875757
5478	Diptera	Culicidae	Aedes	Aedes aegypti	SPECIES	-5.98000	-37.71000	1264875778
5479	Diptera	Culicidae	Aedes	Aedes aegypti	SPECIES	-5.97762	106.70683	3993825934
5480	Diptera	Culicidae	Aedes	Aedes aegypti	SPECIES	-5.96000	-47.30000	1264875762
5481	Diptera	Culicidae	Aedes	Aedes aegypti	SPECIES	-5.95000	-36.44000	1264875736
5482	Diptera	Culicidae	Aedes	Aedes aegypti	SPECIES	-5.95000	-47.96000	1264875768
5483	Diptera	Culicidae	Aedes	Aedes aegypti	SPECIES	-5.94000	-38.04000	1264875749
5484	Diptera	Culicidae	Aedes	Aedes aegypti	SPECIES	-5.94000	-35.43000	1264875781
5485	Diptera	Culicidae	Aedes	Aedes aegypti	SPECIES	-5.93000	-64.95000	1264875776
5486	Diptera	Culicidae	Aedes	Aedes aegypti	SPECIES	-5.93000	-35.76000	1264875799
5487	Diptera	Culicidae	Aedes	Aedes aegypti	SPECIES	-5.92608	-35.26073	3712721123
5488	Diptera	Culicidae	Aedes	Aedes aegypti	SPECIES	-5.92000	-37.15000	1264875751
5489	Diptera	Culicidae	Aedes	Aedes aegypti	SPECIES	-5.92000	-42.12000	1264875755
5490	Diptera	Culicidae	Aedes	Aedes aegypti	SPECIES	-5.92000	-37.97000	1264875759
5491	Diptera	Culicidae	Aedes	Aedes aegypti	SPECIES	-5.92000	-35.21000	1264875767
5492	Diptera	Culicidae	Aedes	Aedes aegypti	SPECIES	-5.92000	-35.25000	1264889432
5493	Diptera	Culicidae	Aedes	Aedes aegypti	SPECIES	-5.91000	-48.35000	1264875752
5494	Diptera	Culicidae	Aedes	Aedes aegypti	SPECIES	-5.91000	-42.62000	1264875763

№	Order	Family	Genus	Species	Taxon Rank	Latitude	Longitude	GBIF_ID
5495	Diptera	Culicidae	Aedes	Aedes aegypti	SPECIES	-5.91000	-41.88000	1264875765
5496	Diptera	Culicidae	Aedes	Aedes aegypti	SPECIES	-5.91000	-36.78000	1264891241
5497	Diptera	Culicidae	Aedes	Aedes aegypti	SPECIES	-5.90000	-37.31000	1264875770
5498	Diptera	Culicidae	Aedes	Aedes aegypti	SPECIES	-5.90000	-36.59000	1264875786
5499	Diptera	Culicidae	Aedes	Aedes aegypti	SPECIES	-5.89000	-35.61000	1264875773
5500	Diptera	Culicidae	Aedes	Aedes aegypti	SPECIES	-5.88000	-41.07000	1264875774
5501	Diptera	Culicidae	Aedes	Aedes aegypti	SPECIES	-5.88000	-40.29000	1264875802
5502	Diptera	Culicidae	Aedes	Aedes aegypti	SPECIES	-5.88000	-42.40000	1264875805
5503	Diptera	Culicidae	Aedes	Aedes aegypti	SPECIES	-5.87000	-40.72000	1264875764
5504	Diptera	Culicidae	Aedes	Aedes aegypti	SPECIES	-5.86000	-39.45000	1264875761
5505	Diptera	Culicidae	Aedes	Aedes aegypti	SPECIES	-5.86000	-44.29000	1264875792
5506	Diptera	Culicidae	Aedes	Aedes aegypti	SPECIES	-5.85000	-35.93000	1264875771
5507	Diptera	Culicidae	Aedes	Aedes aegypti	SPECIES	-5.85000	-38.08000	1264875777
5508	Diptera	Culicidae	Aedes	Aedes aegypti	SPECIES	-5.85000	-42.80000	1264875785
5509	Diptera	Culicidae	Aedes	Aedes aegypti	SPECIES	-5.84000	-42.92000	1264875769
5510	Diptera	Culicidae	Aedes	Aedes aegypti	SPECIES	-5.84000	-36.87000	1264875794
5511	Diptera	Culicidae	Aedes	Aedes aegypti	SPECIES	-5.84000	-39.74000	1264875798
5512	Diptera	Culicidae	Aedes	Aedes aegypti	SPECIES	-5.84000	-47.06000	1264875803
5513	Diptera	Culicidae	Aedes	Aedes aegypti	SPECIES	-5.84000	-36.68000	1264891117
5514	Diptera	Culicidae	Aedes	Aedes aegypti	SPECIES	-5.82167	106.55417	3993826376
5515	Diptera	Culicidae	Aedes	Aedes aegypti	SPECIES	-5.82000	-42.49000	1264875782
5516	Diptera	Culicidae	Aedes	Aedes aegypti	SPECIES	-5.82000	-42.57000	1264875816
5517	Diptera	Culicidae	Aedes	Aedes aegypti	SPECIES	-5.82000	-35.86000	1264875823
5518	Diptera	Culicidae	Aedes	Aedes aegypti	SPECIES	-5.81000	-45.92000	1264875772
5519	Diptera	Culicidae	Aedes	Aedes aegypti	SPECIES	-5.81000	-42.63000	1264875790
5520	Diptera	Culicidae	Aedes	Aedes aegypti	SPECIES	-5.81000	-35.21000	1264889463
5521	Diptera	Culicidae	Aedes	Aedes aegypti	SPECIES	-5.80000	-37.94000	1264875783
5522	Diptera	Culicidae	Aedes	Aedes aegypti	SPECIES	-5.80000	-35.22000	1264875789
5523	Diptera	Culicidae	Aedes	Aedes aegypti	SPECIES	-5.80000	-42.26000	1264875796
5524	Diptera	Culicidae	Aedes	Aedes aegypti	SPECIES	-5.79000	-35.72000	1264875791
5525	Diptera	Culicidae	Aedes	Aedes aegypti	SPECIES	-5.79000	-35.55000	1264875795
5526	Diptera	Culicidae	Aedes	Aedes aegypti	SPECIES	-5.79000	-47.60000	1264875809
5527	Diptera	Culicidae	Aedes	Aedes aegypti	SPECIES	-5.78000	-37.97000	1264875784
5528	Diptera	Culicidae	Aedes	Aedes aegypti	SPECIES	-5.78000	-38.16000	1264875788
5529	Diptera	Culicidae	Aedes	Aedes aegypti	SPECIES	-5.78000	-38.99000	1264875793
5530	Diptera	Culicidae	Aedes	Aedes aegypti	SPECIES	-5.78000	-36.42000	1264875817
5531	Diptera	Culicidae	Aedes	Aedes aegypti	SPECIES	-5.78000	-41.53000	1264875826
5532	Diptera	Culicidae	Aedes	Aedes aegypti	SPECIES	-5.78000	-43.83000	1264875828
5533	Diptera	Culicidae	Aedes	Aedes aegypti	SPECIES	-5.77000	-35.36000	1264875797
5534	Diptera	Culicidae	Aedes	Aedes aegypti	SPECIES	-5.77000	-37.59000	1264875807
5535	Diptera	Culicidae	Aedes	Aedes aegypti	SPECIES	-5.77000	-42.68000	1264875808

№	Order	Family	Genus	Species	Taxon Rank	Latitude	Longitude	GBIF_ID
5536	Diptera	Culicidae	Aedes	Aedes aegypti	SPECIES	-5.77000	-37.11000	1264875824
5537	Diptera	Culicidae	Aedes	Aedes aegypti	SPECIES	-5.77000	38.85000	1264889861
5538	Diptera	Culicidae	Aedes	Aedes aegypti	SPECIES	-5.76000	-48.40000	1264875780
5539	Diptera	Culicidae	Aedes	Aedes aegypti	SPECIES	-5.76000	-38.32000	1264875818
5540	Diptera	Culicidae	Aedes	Aedes aegypti	SPECIES	-5.76000	-36.02000	1264875839
5541	Diptera	Culicidae	Aedes	Aedes aegypti	SPECIES	-5.73000	-47.29000	1264875800
5542	Diptera	Culicidae	Aedes	Aedes aegypti	SPECIES	-5.72000	-42.16000	1264875830
5543	Diptera	Culicidae	Aedes	Aedes aegypti	SPECIES	-5.71000	-42.71000	1264875834
5544	Diptera	Culicidae	Aedes	Aedes aegypti	SPECIES	-5.71000	-78.81000	1264887639
5545	Diptera	Culicidae	Aedes	Aedes aegypti	SPECIES	-5.68000	-36.80000	1264875819
5546	Diptera	Culicidae	Aedes	Aedes aegypti	SPECIES	-5.68000	-35.28000	1264875844
5547	Diptera	Culicidae	Aedes	Aedes aegypti	SPECIES	-5.68000	-48.70000	1264875850
5548	Diptera	Culicidae	Aedes	Aedes aegypti	SPECIES	-5.68000	-47.76000	1264875872
5549	Diptera	Culicidae	Aedes	Aedes aegypti	SPECIES	-5.68000	-39.20000	1264889190
5550	Diptera	Culicidae	Aedes	Aedes aegypti	SPECIES	-5.68000	-79.03000	1264891083
5551	Diptera	Culicidae	Aedes	Aedes aegypti	SPECIES	-5.67000	-35.79000	1264875815
5552	Diptera	Culicidae	Aedes	Aedes aegypti	SPECIES	-5.67000	-44.33000	1264875843
5553	Diptera	Culicidae	Aedes	Aedes aegypti	SPECIES	-5.67000	-36.17000	1264875855
5554	Diptera	Culicidae	Aedes	Aedes aegypti	SPECIES	-5.66000	-44.03000	1264875862
5555	Diptera	Culicidae	Aedes	Aedes aegypti	SPECIES	-5.65000	-47.71000	1264875811
5556	Diptera	Culicidae	Aedes	Aedes aegypti	SPECIES	-5.64000	-48.11000	1264875825
5557	Diptera	Culicidae	Aedes	Aedes aegypti	SPECIES	-5.64000	-36.52000	1264875831
5558	Diptera	Culicidae	Aedes	Aedes aegypti	SPECIES	-5.64000	-39.15000	1264875838
5559	Diptera	Culicidae	Aedes	Aedes aegypti	SPECIES	-5.63000	-37.87000	1264875813
5560	Diptera	Culicidae	Aedes	Aedes aegypti	SPECIES	-5.62000	-37.29000	1264875833
5561	Diptera	Culicidae	Aedes	Aedes aegypti	SPECIES	-5.61000	-42.59000	1264875812
5562	Diptera	Culicidae	Aedes	Aedes aegypti	SPECIES	-5.60000	-43.48000	1264875804
5563	Diptera	Culicidae	Aedes	Aedes aegypti	SPECIES	-5.60000	-40.75000	1264875814
5564	Diptera	Culicidae	Aedes	Aedes aegypti	SPECIES	-5.60000	-42.85000	1264875820
5565	Diptera	Culicidae	Aedes	Aedes aegypti	SPECIES	-5.60000	-44.53000	1264875849
5566	Diptera	Culicidae	Aedes	Aedes aegypti	SPECIES	-5.60000	-35.39000	1264875851
5567	Diptera	Culicidae	Aedes	Aedes aegypti	SPECIES	-5.60000	-35.58000	1264875854
5568	Diptera	Culicidae	Aedes	Aedes aegypti	SPECIES	-5.59000	-35.69000	1264875801
5569	Diptera	Culicidae	Aedes	Aedes aegypti	SPECIES	-5.59000	-35.99000	1264875810
5570	Diptera	Culicidae	Aedes	Aedes aegypti	SPECIES	-5.59000	-37.01000	1264875821
5571	Diptera	Culicidae	Aedes	Aedes aegypti	SPECIES	-5.58000	-47.28000	1264875806
5572	Diptera	Culicidae	Aedes	Aedes aegypti	SPECIES	-5.58000	-39.46000	1264875822
5573	Diptera	Culicidae	Aedes	Aedes aegypti	SPECIES	-5.57000	-44.08000	1264875832
5574	Diptera	Culicidae	Aedes	Aedes aegypti	SPECIES	-5.56000	120.18000	1264871835
5575	Diptera	Culicidae	Aedes	Aedes aegypti	SPECIES	-5.55000	120.19000	1264871845
5576	Diptera	Culicidae	Aedes	Aedes aegypti	SPECIES	-5.55000	-41.90000	1264875848

№	Order	Family	Genus	Species	Taxon Rank	Latitude	Longitude	GBIF_ID
5577	Diptera	Culicidae	Aedes	Aedes aegypti	SPECIES	-5.55000	-47.61000	1264875860
5578	Diptera	Culicidae	Aedes	Aedes aegypti	SPECIES	-5.54000	120.22000	1264871817
5579	Diptera	Culicidae	Aedes	Aedes aegypti	SPECIES	-5.54000	120.20000	1264871839
5580	Diptera	Culicidae	Aedes	Aedes aegypti	SPECIES	-5.54000	-36.80000	1264875859
5581	Diptera	Culicidae	Aedes	Aedes aegypti	SPECIES	-5.53000	-36.08000	1264875835
5582	Diptera	Culicidae	Aedes	Aedes aegypti	SPECIES	-5.53000	-37.66000	1264875842
5583	Diptera	Culicidae	Aedes	Aedes aegypti	SPECIES	-5.53000	-50.07000	1264875876
5584	Diptera	Culicidae	Aedes	Aedes aegypti	SPECIES	-5.52000	-39.82000	1264875845
5585	Diptera	Culicidae	Aedes	Aedes aegypti	SPECIES	-5.52000	-38.17000	1264875847
5586	Diptera	Culicidae	Aedes	Aedes aegypti	SPECIES	-5.52000	-47.95000	1264875856
5587	Diptera	Culicidae	Aedes	Aedes aegypti	SPECIES	-5.51000	-44.61000	1264875827
5588	Diptera	Culicidae	Aedes	Aedes aegypti	SPECIES	-5.51000	-47.09000	1264875829
5589	Diptera	Culicidae	Aedes	Aedes aegypti	SPECIES	-5.51000	-45.17000	1264875837
5590	Diptera	Culicidae	Aedes	Aedes aegypti	SPECIES	-5.51000	119.94000	1264889880
5591	Diptera	Culicidae	Aedes	Aedes aegypti	SPECIES	-5.50000	-40.34000	1264875846
5592	Diptera	Culicidae	Aedes	Aedes aegypti	SPECIES	-5.49000	119.79000	1264871908
5593	Diptera	Culicidae	Aedes	Aedes aegypti	SPECIES	-5.49000	-47.78000	1264875836
5594	Diptera	Culicidae	Aedes	Aedes aegypti	SPECIES	-5.49000	-38.72000	1264875858
5595	Diptera	Culicidae	Aedes	Aedes aegypti	SPECIES	-5.48000	119.76000	1264871880
5596	Diptera	Culicidae	Aedes	Aedes aegypti	SPECIES	-5.48000	-36.65000	1264875841
5597	Diptera	Culicidae	Aedes	Aedes aegypti	SPECIES	-5.48000	-42.41000	1264875878
5598	Diptera	Culicidae	Aedes	Aedes aegypti	SPECIES	-5.47000	-36.32000	1264875869
5599	Diptera	Culicidae	Aedes	Aedes aegypti	SPECIES	-5.47000	-35.88000	1264875875
5600	Diptera	Culicidae	Aedes	Aedes aegypti	SPECIES	-5.45000	-35.38000	1264875840
5601	Diptera	Culicidae	Aedes	Aedes aegypti	SPECIES	-5.45000	-42.60000	1264875870
5602	Diptera	Culicidae	Aedes	Aedes aegypti	SPECIES	-5.44000	119.77000	1264871836
5603	Diptera	Culicidae	Aedes	Aedes aegypti	SPECIES	-5.44000	119.74000	1264871936
5604	Diptera	Culicidae	Aedes	Aedes aegypti	SPECIES	-5.44000	-48.68000	1264875881
5605	Diptera	Culicidae	Aedes	Aedes aegypti	SPECIES	-5.44000	-42.88000	1264875885
5606	Diptera	Culicidae	Aedes	Aedes aegypti	SPECIES	-5.42000	-45.57000	1264875903
5607	Diptera	Culicidae	Aedes	Aedes aegypti	SPECIES	-5.41016	106.49350	3901423256
5608	Diptera	Culicidae	Aedes	Aedes aegypti	SPECIES	-5.41000	-35.59000	1264875865
5609	Diptera	Culicidae	Aedes	Aedes aegypti	SPECIES	-5.40000	-37.55000	1264875864
5610	Diptera	Culicidae	Aedes	Aedes aegypti	SPECIES	-5.40000	-42.16000	1264875871
5611	Diptera	Culicidae	Aedes	Aedes aegypti	SPECIES	-5.39000	119.82000	1264871861
5612	Diptera	Culicidae	Aedes	Aedes aegypti	SPECIES	-5.39000	-44.32000	1264875852
5613	Diptera	Culicidae	Aedes	Aedes aegypti	SPECIES	-5.39000	-43.39000	1264875853
5614	Diptera	Culicidae	Aedes	Aedes aegypti	SPECIES	-5.39000	-44.06000	1264875861
5615	Diptera	Culicidae	Aedes	Aedes aegypti	SPECIES	-5.39000	-47.62000	1264875880
5616	Diptera	Culicidae	Aedes	Aedes aegypti	SPECIES	-5.38000	105.21000	1264871963
5617	Diptera	Culicidae	Aedes	Aedes aegypti	SPECIES	-5.38000	-46.48000	1264875857

№	Order	Family	Genus	Species	Taxon Rank	Latitude	Longitude	GBIF_ID
5618	Diptera	Culicidae	Aedes	Aedes aegypti	SPECIES	-5.36000	105.24000	1264871741
5619	Diptera	Culicidae	Aedes	Aedes aegypti	SPECIES	-5.36000	-35.40000	1264875904
5620	Diptera	Culicidae	Aedes	Aedes aegypti	SPECIES	-5.35000	-48.15000	1264875883
5621	Diptera	Culicidae	Aedes	Aedes aegypti	SPECIES	-5.35000	-36.13000	1264875886
5622	Diptera	Culicidae	Aedes	Aedes aegypti	SPECIES	-5.34000	-47.92000	1264875867
5623	Diptera	Culicidae	Aedes	Aedes aegypti	SPECIES	-5.34000	-36.75000	1264875873
5624	Diptera	Culicidae	Aedes	Aedes aegypti	SPECIES	-5.34000	-42.67000	1264875891
5625	Diptera	Culicidae	Aedes	Aedes aegypti	SPECIES	-5.33000	-48.01000	1264875874
5626	Diptera	Culicidae	Aedes	Aedes aegypti	SPECIES	-5.33000	-59.24000	1264875902
5627	Diptera	Culicidae	Aedes	Aedes aegypti	SPECIES	-5.32000	-39.34000	1264891274
5628	Diptera	Culicidae	Aedes	Aedes aegypti	SPECIES	-5.31000	-48.57000	1264875868
5629	Diptera	Culicidae	Aedes	Aedes aegypti	SPECIES	-5.31000	-38.28000	1264875889
5630	Diptera	Culicidae	Aedes	Aedes aegypti	SPECIES	-5.31000	-47.14000	1264875897
5631	Diptera	Culicidae	Aedes	Aedes aegypti	SPECIES	-5.30906	122.03857	4017640965
5632	Diptera	Culicidae	Aedes	Aedes aegypti	SPECIES	-5.30000	-38.01000	1264875884
5633	Diptera	Culicidae	Aedes	Aedes aegypti	SPECIES	-5.30000	-35.95000	1264875900
5634	Diptera	Culicidae	Aedes	Aedes aegypti	SPECIES	-5.29000	-36.63000	1264875866
5635	Diptera	Culicidae	Aedes	Aedes aegypti	SPECIES	-5.29000	-44.46000	1264875877
5636	Diptera	Culicidae	Aedes	Aedes aegypti	SPECIES	-5.29000	-41.54000	1264875879
5637	Diptera	Culicidae	Aedes	Aedes aegypti	SPECIES	-5.29000	-35.61000	1264875887
5638	Diptera	Culicidae	Aedes	Aedes aegypti	SPECIES	-5.29000	-38.96000	1264875888
5639	Diptera	Culicidae	Aedes	Aedes aegypti	SPECIES	-5.29000	-41.92000	1264875895
5640	Diptera	Culicidae	Aedes	Aedes aegypti	SPECIES	-5.27000	-36.88000	1264875910
5641	Diptera	Culicidae	Aedes	Aedes aegypti	SPECIES	-5.26000	-43.96000	1264875882
5642	Diptera	Culicidae	Aedes	Aedes aegypti	SPECIES	-5.24000	-48.33000	1264875863
5643	Diptera	Culicidae	Aedes	Aedes aegypti	SPECIES	-5.24000	-57.92000	1264875896
5644	Diptera	Culicidae	Aedes	Aedes aegypti	SPECIES	-5.24000	-47.16000	1264875911
5645	Diptera	Culicidae	Aedes	Aedes aegypti	SPECIES	-5.23000	-36.47000	1264875926
5646	Diptera	Culicidae	Aedes	Aedes aegypti	SPECIES	-5.22000	-45.78000	1264875906
5647	Diptera	Culicidae	Aedes	Aedes aegypti	SPECIES	-5.21000	119.46000	1264871914
5648	Diptera	Culicidae	Aedes	Aedes aegypti	SPECIES	-5.21000	119.40000	1264871943
5649	Diptera	Culicidae	Aedes	Aedes aegypti	SPECIES	-5.21000	-40.75000	1264875894
5650	Diptera	Culicidae	Aedes	Aedes aegypti	SPECIES	-5.20583	-37.32361	4096863787
5651	Diptera	Culicidae	Aedes	Aedes aegypti	SPECIES	-5.20250	-37.32833	4046474285
5652	Diptera	Culicidae	Aedes	Aedes aegypti	SPECIES	-5.20000	119.46000	1264871884
5653	Diptera	Culicidae	Aedes	Aedes aegypti	SPECIES	-5.20000	119.45000	1264871895
5654	Diptera	Culicidae	Aedes	Aedes aegypti	SPECIES	-5.20000	-39.29000	1264889494
5655	Diptera	Culicidae	Aedes	Aedes aegypti	SPECIES	-5.19000	-42.78000	1264875892
5656	Diptera	Culicidae	Aedes	Aedes aegypti	SPECIES	-5.19000	-39.35000	1264875918
5657	Diptera	Culicidae	Aedes	Aedes aegypti	SPECIES	-5.19000	119.45000	1264876406
5658	Diptera	Culicidae	Aedes	Aedes aegypti	SPECIES	-5.19000	-39.30000	1264888080

№	Order	Family	Genus	Species	Taxon Rank	Latitude	Longitude	GBIF_ID
5659	Diptera	Culicidae	Aedes	Aedes aegypti	SPECIES	-5.19000	-80.63000	1264888341
5660	Diptera	Culicidae	Aedes	Aedes aegypti	SPECIES	-5.19000	-37.34000	1264889473
5661	Diptera	Culicidae	Aedes	Aedes aegypti	SPECIES	-5.18000	-36.35000	1264875890
5662	Diptera	Culicidae	Aedes	Aedes aegypti	SPECIES	-5.18000	-35.73000	1264875905
5663	Diptera	Culicidae	Aedes	Aedes aegypti	SPECIES	-5.18000	-36.20000	1264875929
5664	Diptera	Culicidae	Aedes	Aedes aegypti	SPECIES	-5.17000	-47.35000	1264875922
5665	Diptera	Culicidae	Aedes	Aedes aegypti	SPECIES	-5.17000	119.45000	1264875925
5666	Diptera	Culicidae	Aedes	Aedes aegypti	SPECIES	-5.17000	-43.01000	1264875932
5667	Diptera	Culicidae	Aedes	Aedes aegypti	SPECIES	-5.17000	119.46000	1264875936
5668	Diptera	Culicidae	Aedes	Aedes aegypti	SPECIES	-5.17000	38.78000	1264890169
5669	Diptera	Culicidae	Aedes	Aedes aegypti	SPECIES	-5.17000	-42.77000	1264890805
5670	Diptera	Culicidae	Aedes	Aedes aegypti	SPECIES	-5.16000	-36.08000	1264875901
5671	Diptera	Culicidae	Aedes	Aedes aegypti	SPECIES	-5.15000	119.43000	1264871956
5672	Diptera	Culicidae	Aedes	Aedes aegypti	SPECIES	-5.15000	-35.87000	1264875909
5673	Diptera	Culicidae	Aedes	Aedes aegypti	SPECIES	-5.15000	-35.99000	1264875914
5674	Diptera	Culicidae	Aedes	Aedes aegypti	SPECIES	-5.15000	-44.16000	1264875916
5675	Diptera	Culicidae	Aedes	Aedes aegypti	SPECIES	-5.15000	119.41000	1264889895
5676	Diptera	Culicidae	Aedes	Aedes aegypti	SPECIES	-5.15000	119.46000	1264890725
5677	Diptera	Culicidae	Aedes	Aedes aegypti	SPECIES	-5.14000	-48.47000	1264875920
5678	Diptera	Culicidae	Aedes	Aedes aegypti	SPECIES	-5.14000	-37.03000	1264875952
5679	Diptera	Culicidae	Aedes	Aedes aegypti	SPECIES	-5.14000	119.44000	1264876407
5680	Diptera	Culicidae	Aedes	Aedes aegypti	SPECIES	-5.14000	119.51000	1264876419
5681	Diptera	Culicidae	Aedes	Aedes aegypti	SPECIES	-5.14000	119.43000	1264891278
5682	Diptera	Culicidae	Aedes	Aedes aegypti	SPECIES	-5.13000	119.43000	1264871932
5683	Diptera	Culicidae	Aedes	Aedes aegypti	SPECIES	-5.13000	-38.02000	1264875893
5684	Diptera	Culicidae	Aedes	Aedes aegypti	SPECIES	-5.13000	119.52000	1264876421
5685	Diptera	Culicidae	Aedes	Aedes aegypti	SPECIES	-5.13000	-80.34000	1264891106
5686	Diptera	Culicidae	Aedes	Aedes aegypti	SPECIES	-5.12000	-42.27000	1264875899
5687	Diptera	Culicidae	Aedes	Aedes aegypti	SPECIES	-5.12000	119.44000	1264876365
5688	Diptera	Culicidae	Aedes	Aedes aegypti	SPECIES	-5.12000	105.31000	1264876462
5689	Diptera	Culicidae	Aedes	Aedes aegypti	SPECIES	-5.12000	119.41000	1264876484
5690	Diptera	Culicidae	Aedes	Aedes aegypti	SPECIES	-5.11670	119.40000	177195179
5691	Diptera	Culicidae	Aedes	Aedes aegypti	SPECIES	-5.11670	119.40000	1633663702
5692	Diptera	Culicidae	Aedes	Aedes aegypti	SPECIES	-5.11000	-55.97000	1264875898
5693	Diptera	Culicidae	Aedes	Aedes aegypti	SPECIES	-5.11000	-80.50000	1264887660
5694	Diptera	Culicidae	Aedes	Aedes aegypti	SPECIES	-5.10000	-45.16000	1264875912
5695	Diptera	Culicidae	Aedes	Aedes aegypti	SPECIES	-5.10000	-37.87000	1264875913
5696	Diptera	Culicidae	Aedes	Aedes aegypti	SPECIES	-5.10000	38.63000	1264889844
5697	Diptera	Culicidae	Aedes	Aedes aegypti	SPECIES	-5.10000	-80.16000	1264890940
5698	Diptera	Culicidae	Aedes	Aedes aegypti	SPECIES	-5.09000	-41.20000	1264875954
5699	Diptera	Culicidae	Aedes	Aedes aegypti	SPECIES	-5.09000	-45.29000	1264891097

№	Order	Family	Genus	Species	Taxon Rank	Latitude	Longitude	GBIF_ID
5700	Diptera	Culicidae	Aedes	Aedes aegypti	SPECIES	-5.08000	-49.91000	1264875917
5701	Diptera	Culicidae	Aedes	Aedes aegypti	SPECIES	-5.08000	-37.63000	1264875930
5702	Diptera	Culicidae	Aedes	Aedes aegypti	SPECIES	-5.07000	-36.84000	1264875944
5703	Diptera	Culicidae	Aedes	Aedes aegypti	SPECIES	-5.07000	39.12000	1264888775
5704	Diptera	Culicidae	Aedes	Aedes aegypti	SPECIES	-5.07000	39.10000	1264889570
5705	Diptera	Culicidae	Aedes	Aedes aegypti	SPECIES	-5.06000	-47.80000	1264875923
5706	Diptera	Culicidae	Aedes	Aedes aegypti	SPECIES	-5.05000	-39.85000	1264875908
5707	Diptera	Culicidae	Aedes	Aedes aegypti	SPECIES	-5.04000	-48.06000	1264875933
5708	Diptera	Culicidae	Aedes	Aedes aegypti	SPECIES	-5.03000	-48.74000	1264875907
5709	Diptera	Culicidae	Aedes	Aedes aegypti	SPECIES	-5.03000	-43.78000	1264875915
5710	Diptera	Culicidae	Aedes	Aedes aegypti	SPECIES	-5.03000	119.57000	1264876488
5711	Diptera	Culicidae	Aedes	Aedes aegypti	SPECIES	-5.02000	-42.46000	1264875919
5712	Diptera	Culicidae	Aedes	Aedes aegypti	SPECIES	-5.02000	-44.58000	1264875921
5713	Diptera	Culicidae	Aedes	Aedes aegypti	SPECIES	-5.02000	-37.07000	1264875948
5714	Diptera	Culicidae	Aedes	Aedes aegypti	SPECIES	-5.02000	-44.40000	1264875949
5715	Diptera	Culicidae	Aedes	Aedes aegypti	SPECIES	-5.01000	-49.21000	1264875934
5716	Diptera	Culicidae	Aedes	Aedes aegypti	SPECIES	-5.01000	-44.77000	1264875979
5717	Diptera	Culicidae	Aedes	Aedes aegypti	SPECIES	-5.01000	119.63000	1264876442
5718	Diptera	Culicidae	Aedes	Aedes aegypti	SPECIES	-5.00000	-45.96000	1264875927
5719	Diptera	Culicidae	Aedes	Aedes aegypti	SPECIES	-5.00000	-44.21000	1264875931
5720	Diptera	Culicidae	Aedes	Aedes aegypti	SPECIES	-5.00000	-41.57000	1264875945
5721	Diptera	Culicidae	Aedes	Aedes aegypti	SPECIES	-5.00000	119.61000	1264876459
5722	Diptera	Culicidae	Aedes	Aedes aegypti	SPECIES	-5.00000	119.70000	1264876515
5723	Diptera	Culicidae	Aedes	Aedes aegypti	SPECIES	-4.98000	-38.55000	1264875957
5724	Diptera	Culicidae	Aedes	Aedes aegypti	SPECIES	-4.98000	119.65000	1264876491
5725	Diptera	Culicidae	Aedes	Aedes aegypti	SPECIES	-4.98000	119.63000	1264876505
5726	Diptera	Culicidae	Aedes	Aedes aegypti	SPECIES	-4.96000	-48.40000	1264875962
5727	Diptera	Culicidae	Aedes	Aedes aegypti	SPECIES	-4.96000	119.70000	1264876501
5728	Diptera	Culicidae	Aedes	Aedes aegypti	SPECIES	-4.95000	-41.81000	1264875940
5729	Diptera	Culicidae	Aedes	Aedes aegypti	SPECIES	-4.95000	-37.22000	1264875953
5730	Diptera	Culicidae	Aedes	Aedes aegypti	SPECIES	-4.95000	119.65000	1264876489
5731	Diptera	Culicidae	Aedes	Aedes aegypti	SPECIES	-4.95000	-38.95000	1264891094
5732	Diptera	Culicidae	Aedes	Aedes aegypti	SPECIES	-4.94000	-40.04000	1264875939
5733	Diptera	Culicidae	Aedes	Aedes aegypti	SPECIES	-4.94000	119.62000	1264876446
5734	Diptera	Culicidae	Aedes	Aedes aegypti	SPECIES	-4.92000	-44.84000	1264875924
5735	Diptera	Culicidae	Aedes	Aedes aegypti	SPECIES	-4.92000	-38.93000	1264875942
5736	Diptera	Culicidae	Aedes	Aedes aegypti	SPECIES	-4.91000	-45.42000	1264875937
5737	Diptera	Culicidae	Aedes	Aedes aegypti	SPECIES	-4.90000	-40.80000	1264875969
5738	Diptera	Culicidae	Aedes	Aedes aegypti	SPECIES	-4.89000	-37.76000	1264875947
5739	Diptera	Culicidae	Aedes	Aedes aegypti	SPECIES	-4.89000	-40.37000	1264875960
5740	Diptera	Culicidae	Aedes	Aedes aegypti	SPECIES	-4.89000	-37.33000	1264875980

№	Order	Family	Genus	Species	Taxon Rank	Latitude	Longitude	GBIF_ID
5741	Diptera	Culicidae	Aedes	Aedes aegypti	SPECIES	-4.87000	-39.51000	1264875938
5742	Diptera	Culicidae	Aedes	Aedes aegypti	SPECIES	-4.86000	-42.13000	1264875941
5743	Diptera	Culicidae	Aedes	Aedes aegypti	SPECIES	-4.85000	-41.92000	1264875956
5744	Diptera	Culicidae	Aedes	Aedes aegypti	SPECIES	-4.84000	-38.14000	1264875943
5745	Diptera	Culicidae	Aedes	Aedes aegypti	SPECIES	-4.83000	-43.35000	1264875972
5746	Diptera	Culicidae	Aedes	Aedes aegypti	SPECIES	-4.81000	-44.48000	1264875959
5747	Diptera	Culicidae	Aedes	Aedes aegypti	SPECIES	-4.80000	-41.05000	1264875951
5748	Diptera	Culicidae	Aedes	Aedes aegypti	SPECIES	-4.80000	-39.17000	1264875975
5749	Diptera	Culicidae	Aedes	Aedes aegypti	SPECIES	-4.79000	-44.87000	1264875958
5750	Diptera	Culicidae	Aedes	Aedes aegypti	SPECIES	-4.79000	11.84000	1264891411
5751	Diptera	Culicidae	Aedes	Aedes aegypti	SPECIES	-4.78000	-40.76000	1264875964
5752	Diptera	Culicidae	Aedes	Aedes aegypti	SPECIES	-4.77000	-38.72000	1264875987
5753	Diptera	Culicidae	Aedes	Aedes aegypti	SPECIES	-4.75000	-45.63000	1264875968
5754	Diptera	Culicidae	Aedes	Aedes aegypti	SPECIES	-4.74000	-37.41000	1264875981
5755	Diptera	Culicidae	Aedes	Aedes aegypti	SPECIES	-4.72000	-41.54000	1264875983
5756	Diptera	Culicidae	Aedes	Aedes aegypti	SPECIES	-4.72000	-45.23000	1264876006
5757	Diptera	Culicidae	Aedes	Aedes aegypti	SPECIES	-4.71000	-38.04000	1264875946
5758	Diptera	Culicidae	Aedes	Aedes aegypti	SPECIES	-4.71000	-40.52000	1264875955
5759	Diptera	Culicidae	Aedes	Aedes aegypti	SPECIES	-4.71000	-37.79000	1264875971
5760	Diptera	Culicidae	Aedes	Aedes aegypti	SPECIES	-4.70000	-42.54000	1264875961
5761	Diptera	Culicidae	Aedes	Aedes aegypti	SPECIES	-4.70000	-47.43000	1264875967
5762	Diptera	Culicidae	Aedes	Aedes aegypti	SPECIES	-4.69000	-44.32000	1264875963
5763	Diptera	Culicidae	Aedes	Aedes aegypti	SPECIES	-4.68000	-37.68000	1264875950
5764	Diptera	Culicidae	Aedes	Aedes aegypti	SPECIES	-4.67000	-44.69000	1264875973
5765	Diptera	Culicidae	Aedes	Aedes aegypti	SPECIES	-4.66000	-44.55000	1264875978
5766	Diptera	Culicidae	Aedes	Aedes aegypti	SPECIES	-4.66000	105.71000	1264890705
5767	Diptera	Culicidae	Aedes	Aedes aegypti	SPECIES	-4.65000	-80.57000	1264891245
5768	Diptera	Culicidae	Aedes	Aedes aegypti	SPECIES	-4.64000	-42.18000	1264876008
5769	Diptera	Culicidae	Aedes	Aedes aegypti	SPECIES	-4.63000	-40.17000	1264875985
5770	Diptera	Culicidae	Aedes	Aedes aegypti	SPECIES	-4.62000	-41.95000	1264875982
5771	Diptera	Culicidae	Aedes	Aedes aegypti	SPECIES	-4.62000	-43.86000	1264875984
5772	Diptera	Culicidae	Aedes	Aedes aegypti	SPECIES	-4.62000	-42.77000	1264875996
5773	Diptera	Culicidae	Aedes	Aedes aegypti	SPECIES	-4.61000	-44.94000	1264875966
5774	Diptera	Culicidae	Aedes	Aedes aegypti	SPECIES	-4.61000	-39.57000	1264875989
5775	Diptera	Culicidae	Aedes	Aedes aegypti	SPECIES	-4.60000	119.69000	1264871635
5776	Diptera	Culicidae	Aedes	Aedes aegypti	SPECIES	-4.60000	-38.95000	1264875977
5777	Diptera	Culicidae	Aedes	Aedes aegypti	SPECIES	-4.59000	-49.25000	1264875997
5778	Diptera	Culicidae	Aedes	Aedes aegypti	SPECIES	-4.58000	119.70000	1264871673
5779	Diptera	Culicidae	Aedes	Aedes aegypti	SPECIES	-4.58000	-44.84000	1264875965
5780	Diptera	Culicidae	Aedes	Aedes aegypti	SPECIES	-4.57000	-40.91000	1264875991
5781	Diptera	Culicidae	Aedes	Aedes aegypti	SPECIES	-4.57000	-45.02000	1264876000

№	Order	Family	Genus	Species	Taxon Rank	Latitude	Longitude	GBIF_ID
5782	Diptera	Culicidae	Aedes	Aedes aegypti	SPECIES	-4.57000	-44.47000	1264876014
5783	Diptera	Culicidae	Aedes	Aedes aegypti	SPECIES	-4.55000	119.72000	1264871663
5784	Diptera	Culicidae	Aedes	Aedes aegypti	SPECIES	-4.54951	129.85717	3993818100
5785	Diptera	Culicidae	Aedes	Aedes aegypti	SPECIES	-4.54000	119.78000	1264871633
5786	Diptera	Culicidae	Aedes	Aedes aegypti	SPECIES	-4.54000	119.60000	1264871643
5787	Diptera	Culicidae	Aedes	Aedes aegypti	SPECIES	-4.54000	-42.13000	1264875992
5788	Diptera	Culicidae	Aedes	Aedes aegypti	SPECIES	-4.54000	-44.66000	1264875995
5789	Diptera	Culicidae	Aedes	Aedes aegypti	SPECIES	-4.54000	-41.43000	1264876009
5790	Diptera	Culicidae	Aedes	Aedes aegypti	SPECIES	-4.53000	-45.37000	1264875970
5791	Diptera	Culicidae	Aedes	Aedes aegypti	SPECIES	-4.53000	-50.20000	1264875974
5792	Diptera	Culicidae	Aedes	Aedes aegypti	SPECIES	-4.53000	-41.91000	1264875986
5793	Diptera	Culicidae	Aedes	Aedes aegypti	SPECIES	-4.52042	129.87988	3993822835
5794	Diptera	Culicidae	Aedes	Aedes aegypti	SPECIES	-4.52000	-38.50000	1264875999
5795	Diptera	Culicidae	Aedes	Aedes aegypti	SPECIES	-4.52000	-46.70000	1264876004
5796	Diptera	Culicidae	Aedes	Aedes aegypti	SPECIES	-4.50000	154.25000	177195225
5797	Diptera	Culicidae	Aedes	Aedes aegypti	SPECIES	-4.50000	-38.69000	1264875976
5798	Diptera	Culicidae	Aedes	Aedes aegypti	SPECIES	-4.50000	-73.58000	1264888715
5799	Diptera	Culicidae	Aedes	Aedes aegypti	SPECIES	-4.48000	119.61000	1264871646
5800	Diptera	Culicidae	Aedes	Aedes aegypti	SPECIES	-4.48000	-48.42000	1264876030
5801	Diptera	Culicidae	Aedes	Aedes aegypti	SPECIES	-4.48000	-42.55000	1264876031
5802	Diptera	Culicidae	Aedes	Aedes aegypti	SPECIES	-4.48000	39.46000	3349201239
5803	Diptera	Culicidae	Aedes	Aedes aegypti	SPECIES	-4.47000	-43.42000	1264876007
5804	Diptera	Culicidae	Aedes	Aedes aegypti	SPECIES	-4.47000	-38.90000	1264876022
5805	Diptera	Culicidae	Aedes	Aedes aegypti	SPECIES	-4.46000	-37.87000	1264876002
5806	Diptera	Culicidae	Aedes	Aedes aegypti	SPECIES	-4.46000	-40.42000	1264876011
5807	Diptera	Culicidae	Aedes	Aedes aegypti	SPECIES	-4.44000	-42.35000	1264876005
5808	Diptera	Culicidae	Aedes	Aedes aegypti	SPECIES	-4.44000	-39.04000	1264876021
5809	Diptera	Culicidae	Aedes	Aedes aegypti	SPECIES	-4.44000	-64.14000	1264890896
5810	Diptera	Culicidae	Aedes	Aedes aegypti	SPECIES	-4.42486	15.30150	4141722161
5811	Diptera	Culicidae	Aedes	Aedes aegypti	SPECIES	-4.42486	15.30388	4141722218
5812	Diptera	Culicidae	Aedes	Aedes aegypti	SPECIES	-4.42000	-46.30000	1264875993
5813	Diptera	Culicidae	Aedes	Aedes aegypti	SPECIES	-4.42000	119.62000	1264888939
5814	Diptera	Culicidae	Aedes	Aedes aegypti	SPECIES	-4.41995	15.30388	4141722210
5815	Diptera	Culicidae	Aedes	Aedes aegypti	SPECIES	-4.41667	15.29937	4141722173
5816	Diptera	Culicidae	Aedes	Aedes aegypti	SPECIES	-4.41000	-65.52000	1264875988
5817	Diptera	Culicidae	Aedes	Aedes aegypti	SPECIES	-4.41000	-44.26000	1264876013
5818	Diptera	Culicidae	Aedes	Aedes aegypti	SPECIES	-4.40000	39.54667	226099166
5819	Diptera	Culicidae	Aedes	Aedes aegypti	SPECIES	-4.40000	-39.41000	1264875990
5820	Diptera	Culicidae	Aedes	Aedes aegypti	SPECIES	-4.39430	136.93200	3443487589
5821	Diptera	Culicidae	Aedes	Aedes aegypti	SPECIES	-4.39000	-45.53000	1264876003
5822	Diptera	Culicidae	Aedes	Aedes aegypti	SPECIES	-4.39000	136.93000	3818512024

№	Order	Family	Genus	Species	Taxon Rank	Latitude	Longitude	GBIF_ID
5823	Diptera	Culicidae	Aedes	Aedes aegypti	SPECIES	-4.38000	-44.69000	1264876020
5824	Diptera	Culicidae	Aedes	Aedes aegypti	SPECIES	-4.38000	-40.64000	1264876025
5825	Diptera	Culicidae	Aedes	Aedes aegypti	SPECIES	-4.38000	-40.97000	1264876038
5826	Diptera	Culicidae	Aedes	Aedes aegypti	SPECIES	-4.36000	-38.09000	1264875998
5827	Diptera	Culicidae	Aedes	Aedes aegypti	SPECIES	-4.36000	-40.07000	1264876017
5828	Diptera	Culicidae	Aedes	Aedes aegypti	SPECIES	-4.36000	-38.86000	1264876019
5829	Diptera	Culicidae	Aedes	Aedes aegypti	SPECIES	-4.36000	-41.76000	1264876035
5830	Diptera	Culicidae	Aedes	Aedes aegypti	SPECIES	-4.36000	-41.58000	1264876036
5831	Diptera	Culicidae	Aedes	Aedes aegypti	SPECIES	-4.36000	-73.92000	1264890946
5832	Diptera	Culicidae	Aedes	Aedes aegypti	SPECIES	-4.33750	39.56667	226099170
5833	Diptera	Culicidae	Aedes	Aedes aegypti	SPECIES	-4.33000	-38.62000	1264875994
5834	Diptera	Culicidae	Aedes	Aedes aegypti	SPECIES	-4.32000	-38.48000	1264876049
5835	Diptera	Culicidae	Aedes	Aedes aegypti	SPECIES	-4.31667	39.56667	226099167
5836	Diptera	Culicidae	Aedes	Aedes aegypti	SPECIES	-4.31000	15.19000	1264886052
5837	Diptera	Culicidae	Aedes	Aedes aegypti	SPECIES	-4.31000	15.31000	1264888838
5838	Diptera	Culicidae	Aedes	Aedes aegypti	SPECIES	-4.31000	15.32000	1264889021
5839	Diptera	Culicidae	Aedes	Aedes aegypti	SPECIES	-4.30000	39.58330	226099165
5840	Diptera	Culicidae	Aedes	Aedes aegypti	SPECIES	-4.30000	-45.09000	1264876018
5841	Diptera	Culicidae	Aedes	Aedes aegypti	SPECIES	-4.30000	-47.56000	1264889446
5842	Diptera	Culicidae	Aedes	Aedes aegypti	SPECIES	-4.29000	-41.31000	1264876010
5843	Diptera	Culicidae	Aedes	Aedes aegypti	SPECIES	-4.29000	-45.91000	1264876015
5844	Diptera	Culicidae	Aedes	Aedes aegypti	SPECIES	-4.29000	-38.99000	1264876032
5845	Diptera	Culicidae	Aedes	Aedes aegypti	SPECIES	-4.27000	15.30000	1264889796
5846	Diptera	Culicidae	Aedes	Aedes aegypti	SPECIES	-4.26000	-40.57000	1264876001
5847	Diptera	Culicidae	Aedes	Aedes aegypti	SPECIES	-4.26000	-38.77000	1264876054
5848	Diptera	Culicidae	Aedes	Aedes aegypti	SPECIES	-4.26000	15.20000	1264886029
5849	Diptera	Culicidae	Aedes	Aedes aegypti	SPECIES	-4.25000	-38.96000	1264876028
5850	Diptera	Culicidae	Aedes	Aedes aegypti	SPECIES	-4.25000	-38.30000	1264876033
5851	Diptera	Culicidae	Aedes	Aedes aegypti	SPECIES	-4.25000	-40.93000	1264876039
5852	Diptera	Culicidae	Aedes	Aedes aegypti	SPECIES	-4.25000	119.62000	1264889416
5853	Diptera	Culicidae	Aedes	Aedes aegypti	SPECIES	-4.24000	-38.30000	1264890846
5854	Diptera	Culicidae	Aedes	Aedes aegypti	SPECIES	-4.23000	-38.65000	1264876024
5855	Diptera	Culicidae	Aedes	Aedes aegypti	SPECIES	-4.23000	-43.10000	1264876046
5856	Diptera	Culicidae	Aedes	Aedes aegypti	SPECIES	-4.22179	35.74815	4065056009
5857	Diptera	Culicidae	Aedes	Aedes aegypti	SPECIES	-4.22000	-43.32000	1264876060
5858	Diptera	Culicidae	Aedes	Aedes aegypti	SPECIES	-4.21000	-44.38000	1264876027
5859	Diptera	Culicidae	Aedes	Aedes aegypti	SPECIES	-4.20000	-38.90000	1264876012
5860	Diptera	Culicidae	Aedes	Aedes aegypti	SPECIES	-4.20000	-47.87000	1264876029
5861	Diptera	Culicidae	Aedes	Aedes aegypti	SPECIES	-4.20000	-55.30000	1264876045
5862	Diptera	Culicidae	Aedes	Aedes aegypti	SPECIES	-4.20000	-42.77000	1264876050
5863	Diptera	Culicidae	Aedes	Aedes aegypti	SPECIES	-4.19000	-38.50000	1264876044

№	Order	Family	Genus	Species	Taxon Rank	Latitude	Longitude	GBIF_ID
5864	Diptera	Culicidae	Aedes	Aedes aegypti	SPECIES	-4.18000	39.45000	1264889111
5865	Diptera	Culicidae	Aedes	Aedes aegypti	SPECIES	-4.17000	104.22000	1264871780
5866	Diptera	Culicidae	Aedes	Aedes aegypti	SPECIES	-4.17000	-47.31000	1264876023
5867	Diptera	Culicidae	Aedes	Aedes aegypti	SPECIES	-4.17000	-44.18000	1264876034
5868	Diptera	Culicidae	Aedes	Aedes aegypti	SPECIES	-4.17000	-43.83000	1264876042
5869	Diptera	Culicidae	Aedes	Aedes aegypti	SPECIES	-4.17000	-39.37000	1264876052
5870	Diptera	Culicidae	Aedes	Aedes aegypti	SPECIES	-4.17000	-40.52000	1264876065
5871	Diptera	Culicidae	Aedes	Aedes aegypti	SPECIES	-4.17000	39.45000	3351677096
5872	Diptera	Culicidae	Aedes	Aedes aegypti	SPECIES	-4.16667	152.16667	177195224
5873	Diptera	Culicidae	Aedes	Aedes aegypti	SPECIES	-4.16667	152.16667	1633667801
5874	Diptera	Culicidae	Aedes	Aedes aegypti	SPECIES	-4.16000	-42.32000	1264876016
5875	Diptera	Culicidae	Aedes	Aedes aegypti	SPECIES	-4.16000	-45.31000	1264876037
5876	Diptera	Culicidae	Aedes	Aedes aegypti	SPECIES	-4.15000	-39.14000	1264876053
5877	Diptera	Culicidae	Aedes	Aedes aegypti	SPECIES	-4.15000	-74.44000	1264891115
5878	Diptera	Culicidae	Aedes	Aedes aegypti	SPECIES	-4.14000	104.15000	1264871719
5879	Diptera	Culicidae	Aedes	Aedes aegypti	SPECIES	-4.14000	-41.01000	1264876048
5880	Diptera	Culicidae	Aedes	Aedes aegypti	SPECIES	-4.14000	39.32000	3349239149
5881	Diptera	Culicidae	Aedes	Aedes aegypti	SPECIES	-4.13000	-41.62000	1264876059
5882	Diptera	Culicidae	Aedes	Aedes aegypti	SPECIES	-4.12000	-44.78000	1264876041
5883	Diptera	Culicidae	Aedes	Aedes aegypti	SPECIES	-4.12000	-45.13000	1264876056
5884	Diptera	Culicidae	Aedes	Aedes aegypti	SPECIES	-4.12000	-38.85000	1264876062
5885	Diptera	Culicidae	Aedes	Aedes aegypti	SPECIES	-4.11000	-40.60000	1264876026
5886	Diptera	Culicidae	Aedes	Aedes aegypti	SPECIES	-4.10000	-38.68000	1264876057
5887	Diptera	Culicidae	Aedes	Aedes aegypti	SPECIES	-4.10000	-43.02000	1264876064
5888	Diptera	Culicidae	Aedes	Aedes aegypti	SPECIES	-4.10000	-63.12000	1264890403
5889	Diptera	Culicidae	Aedes	Aedes aegypti	SPECIES	-4.09000	-45.49000	1264876070
5890	Diptera	Culicidae	Aedes	Aedes aegypti	SPECIES	-4.09000	-38.47000	1264876078
5891	Diptera	Culicidae	Aedes	Aedes aegypti	SPECIES	-4.07000	-40.89000	1264876077
5892	Diptera	Culicidae	Aedes	Aedes aegypti	SPECIES	-4.06000	-38.31000	1264876043
5893	Diptera	Culicidae	Aedes	Aedes aegypti	SPECIES	-4.05000	-40.77000	1264876089
5894	Diptera	Culicidae	Aedes	Aedes aegypti	SPECIES	-4.05000	39.67000	1264889662
5895	Diptera	Culicidae	Aedes	Aedes aegypti	SPECIES	-4.05000	39.70000	1264890256
5896	Diptera	Culicidae	Aedes	Aedes aegypti	SPECIES	-4.04000	-39.43000	1264876055
5897	Diptera	Culicidae	Aedes	Aedes aegypti	SPECIES	-4.04000	-64.08000	1264876071
5898	Diptera	Culicidae	Aedes	Aedes aegypti	SPECIES	-4.03222	35.77491	4065055787
5899	Diptera	Culicidae	Aedes	Aedes aegypti	SPECIES	-4.03000	-58.67000	1264876072
5900	Diptera	Culicidae	Aedes	Aedes aegypti	SPECIES	-4.03000	-69.68000	1264876087
5901	Diptera	Culicidae	Aedes	Aedes aegypti	SPECIES	-4.03000	39.69000	1264890088
5902	Diptera	Culicidae	Aedes	Aedes aegypti	SPECIES	-4.02000	-42.60000	1264876047
5903	Diptera	Culicidae	Aedes	Aedes aegypti	SPECIES	-4.02000	-48.99000	1264876069
5904	Diptera	Culicidae	Aedes	Aedes aegypti	SPECIES	-4.02000	122.55000	1264876506

№	Order	Family	Genus	Species	Taxon Rank	Latitude	Longitude	GBIF_ID
5905	Diptera	Culicidae	Aedes	Aedes aegypti	SPECIES	-4.00000	-38.82000	1264876061
5906	Diptera	Culicidae	Aedes	Aedes aegypti	SPECIES	-4.00000	-40.69000	1264876067
5907	Diptera	Culicidae	Aedes	Aedes aegypti	SPECIES	-4.00000	122.52000	1264876466
5908	Diptera	Culicidae	Aedes	Aedes aegypti	SPECIES	-4.00000	39.59000	1264890225
5909	Diptera	Culicidae	Aedes	Aedes aegypti	SPECIES	-3.99000	-38.56000	1264876040
5910	Diptera	Culicidae	Aedes	Aedes aegypti	SPECIES	-3.99000	-44.54000	1264876058
5911	Diptera	Culicidae	Aedes	Aedes aegypti	SPECIES	-3.98000	-44.82000	1264876079
5912	Diptera	Culicidae	Aedes	Aedes aegypti	SPECIES	-3.98000	122.51000	1264876498
5913	Diptera	Culicidae	Aedes	Aedes aegypti	SPECIES	-3.98000	-40.69000	1264889186
5914	Diptera	Culicidae	Aedes	Aedes aegypti	SPECIES	-3.97000	-45.97000	1264876066
5915	Diptera	Culicidae	Aedes	Aedes aegypti	SPECIES	-3.97000	-41.29000	1264876073
5916	Diptera	Culicidae	Aedes	Aedes aegypti	SPECIES	-3.97000	122.60000	1264876495
5917	Diptera	Culicidae	Aedes	Aedes aegypti	SPECIES	-3.97000	39.50000	1264890134
5918	Diptera	Culicidae	Aedes	Aedes aegypti	SPECIES	-3.96000	-38.61000	1264876074
5919	Diptera	Culicidae	Aedes	Aedes aegypti	SPECIES	-3.96000	-39.33000	1264876081
5920	Diptera	Culicidae	Aedes	Aedes aegypti	SPECIES	-3.96000	-42.06000	1264876083
5921	Diptera	Culicidae	Aedes	Aedes aegypti	SPECIES	-3.96000	-46.51000	1264876085
5922	Diptera	Culicidae	Aedes	Aedes aegypti	SPECIES	-3.95000	-38.38000	1264876051
5923	Diptera	Culicidae	Aedes	Aedes aegypti	SPECIES	-3.95000	-40.54000	1264876080
5924	Diptera	Culicidae	Aedes	Aedes aegypti	SPECIES	-3.95000	-41.02000	1264876084
5925	Diptera	Culicidae	Aedes	Aedes aegypti	SPECIES	-3.95000	-51.33000	1264876103
5926	Diptera	Culicidae	Aedes	Aedes aegypti	SPECIES	-3.95000	-42.71000	1264876107
5927	Diptera	Culicidae	Aedes	Aedes aegypti	SPECIES	-3.95000	39.53000	1264889667
5928	Diptera	Culicidae	Aedes	Aedes aegypti	SPECIES	-3.94000	-45.24000	1264876097
5929	Diptera	Culicidae	Aedes	Aedes aegypti	SPECIES	-3.94000	39.57000	1264887202
5930	Diptera	Culicidae	Aedes	Aedes aegypti	SPECIES	-3.93383	39.55860	1843533119
5931	Diptera	Culicidae	Aedes	Aedes aegypti	SPECIES	-3.93370	39.56920	2633845706
5932	Diptera	Culicidae	Aedes	Aedes aegypti	SPECIES	-3.93000	39.55000	1264887073
5933	Diptera	Culicidae	Aedes	Aedes aegypti	SPECIES	-3.93000	39.57000	4008349026
5934	Diptera	Culicidae	Aedes	Aedes aegypti	SPECIES	-3.92000	-40.36000	1264876082
5935	Diptera	Culicidae	Aedes	Aedes aegypti	SPECIES	-3.92000	-39.58000	1264876114
5936	Diptera	Culicidae	Aedes	Aedes aegypti	SPECIES	-3.92000	122.50000	1264876464
5937	Diptera	Culicidae	Aedes	Aedes aegypti	SPECIES	-3.92000	39.60000	1264887178
5938	Diptera	Culicidae	Aedes	Aedes aegypti	SPECIES	-3.91426	39.58660	1843573665
5939	Diptera	Culicidae	Aedes	Aedes aegypti	SPECIES	-3.91000	-54.50000	1264876095
5940	Diptera	Culicidae	Aedes	Aedes aegypti	SPECIES	-3.91000	39.60000	1264887166
5941	Diptera	Culicidae	Aedes	Aedes aegypti	SPECIES	-3.91000	-70.93000	1264889480
5942	Diptera	Culicidae	Aedes	Aedes aegypti	SPECIES	-3.89000	-40.77000	1264876063
5943	Diptera	Culicidae	Aedes	Aedes aegypti	SPECIES	-3.89000	-38.63000	1264876068
5944	Diptera	Culicidae	Aedes	Aedes aegypti	SPECIES	-3.89000	-39.83000	1264876091
5945	Diptera	Culicidae	Aedes	Aedes aegypti	SPECIES	-3.88000	-39.19000	1264876076

№	Order	Family	Genus	Species	Taxon Rank	Latitude	Longitude	GBIF_ID
5946	Diptera	Culicidae	Aedes	Aedes aegypti	SPECIES	-3.88000	-42.97000	1264876111
5947	Diptera	Culicidae	Aedes	Aedes aegypti	SPECIES	-3.88000	-38.63000	1264890836
5948	Diptera	Culicidae	Aedes	Aedes aegypti	SPECIES	-3.87000	-41.01000	1264876094
5949	Diptera	Culicidae	Aedes	Aedes aegypti	SPECIES	-3.87000	-45.09000	1264876098
5950	Diptera	Culicidae	Aedes	Aedes aegypti	SPECIES	-3.87000	-38.46000	1264876099
5951	Diptera	Culicidae	Aedes	Aedes aegypti	SPECIES	-3.86000	-40.04000	1264876086
5952	Diptera	Culicidae	Aedes	Aedes aegypti	SPECIES	-3.86000	-32.43000	1264876090
5953	Diptera	Culicidae	Aedes	Aedes aegypti	SPECIES	-3.86000	39.47000	1264886630
5954	Diptera	Culicidae	Aedes	Aedes aegypti	SPECIES	-3.85000	-41.59000	1264876108
5955	Diptera	Culicidae	Aedes	Aedes aegypti	SPECIES	-3.83000	-40.25000	1264876106
5956	Diptera	Culicidae	Aedes	Aedes aegypti	SPECIES	-3.83000	102.32000	1264876493
5957	Diptera	Culicidae	Aedes	Aedes aegypti	SPECIES	-3.82000	-42.46000	1264876109
5958	Diptera	Culicidae	Aedes	Aedes aegypti	SPECIES	-3.82000	-40.21000	1264890830
5959	Diptera	Culicidae	Aedes	Aedes aegypti	SPECIES	-3.82000	-80.59000	1264891257
5960	Diptera	Culicidae	Aedes	Aedes aegypti	SPECIES	-3.81000	-45.46000	1264876092
5961	Diptera	Culicidae	Aedes	Aedes aegypti	SPECIES	-3.80000	-47.53000	1264876075
5962	Diptera	Culicidae	Aedes	Aedes aegypti	SPECIES	-3.80000	-49.86000	1264876112
5963	Diptera	Culicidae	Aedes	Aedes aegypti	SPECIES	-3.80000	-38.59000	1264889144
5964	Diptera	Culicidae	Aedes	Aedes aegypti	SPECIES	-3.80000	-38.58000	1264889148
5965	Diptera	Culicidae	Aedes	Aedes aegypti	SPECIES	-3.79000	-44.83000	1264876102
5966	Diptera	Culicidae	Aedes	Aedes aegypti	SPECIES	-3.79000	-42.19000	1264876104
5967	Diptera	Culicidae	Aedes	Aedes aegypti	SPECIES	-3.79000	-38.52000	1264876134
5968	Diptera	Culicidae	Aedes	Aedes aegypti	SPECIES	-3.79000	-64.95000	1264891237
5969	Diptera	Culicidae	Aedes	Aedes aegypti	SPECIES	-3.79000	39.26000	3349297196
5970	Diptera	Culicidae	Aedes	Aedes aegypti	SPECIES	-3.78000	-45.28000	1264876122
5971	Diptera	Culicidae	Aedes	Aedes aegypti	SPECIES	-3.78000	-44.17000	1264876124
5972	Diptera	Culicidae	Aedes	Aedes aegypti	SPECIES	-3.78000	-41.83000	1264876138
5973	Diptera	Culicidae	Aedes	Aedes aegypti	SPECIES	-3.78000	-38.60000	1264889162
5974	Diptera	Culicidae	Aedes	Aedes aegypti	SPECIES	-3.78000	-38.59000	1264890657
5975	Diptera	Culicidae	Aedes	Aedes aegypti	SPECIES	-3.78000	-38.81000	1264891101
5976	Diptera	Culicidae	Aedes	Aedes aegypti	SPECIES	-3.78000	-38.53000	1264891121
5977	Diptera	Culicidae	Aedes	Aedes aegypti	SPECIES	-3.77000	-45.58000	1264876093
5978	Diptera	Culicidae	Aedes	Aedes aegypti	SPECIES	-3.77000	-38.79000	1264876100
5979	Diptera	Culicidae	Aedes	Aedes aegypti	SPECIES	-3.77000	-43.46000	1264876105
5980	Diptera	Culicidae	Aedes	Aedes aegypti	SPECIES	-3.76000	-49.29000	1264876119
5981	Diptera	Culicidae	Aedes	Aedes aegypti	SPECIES	-3.76000	-73.26000	1264887612
5982	Diptera	Culicidae	Aedes	Aedes aegypti	SPECIES	-3.76000	-38.56000	1264889135
5983	Diptera	Culicidae	Aedes	Aedes aegypti	SPECIES	-3.76000	-38.60000	1264890349
5984	Diptera	Culicidae	Aedes	Aedes aegypti	SPECIES	-3.76000	-38.55000	1264890372
5985	Diptera	Culicidae	Aedes	Aedes aegypti	SPECIES	-3.76000	-73.25000	1264890974
5986	Diptera	Culicidae	Aedes	Aedes aegypti	SPECIES	-3.75000	-44.45000	1264876116

№	Order	Family	Genus	Species	Taxon Rank	Latitude	Longitude	GBIF_ID
5987	Diptera	Culicidae	Aedes	Aedes aegypti	SPECIES	-3.75000	-60.23000	1264876133
5988	Diptera	Culicidae	Aedes	Aedes aegypti	SPECIES	-3.75000	-73.26000	1264887720
5989	Diptera	Culicidae	Aedes	Aedes aegypti	SPECIES	-3.75000	-38.57000	1264889131
5990	Diptera	Culicidae	Aedes	Aedes aegypti	SPECIES	-3.75000	-38.55000	1264889149
5991	Diptera	Culicidae	Aedes	Aedes aegypti	SPECIES	-3.75000	-73.27000	1264889490
5992	Diptera	Culicidae	Aedes	Aedes aegypti	SPECIES	-3.75000	39.82000	1264890328
5993	Diptera	Culicidae	Aedes	Aedes aegypti	SPECIES	-3.75000	-73.19000	1264890796
5994	Diptera	Culicidae	Aedes	Aedes aegypti	SPECIES	-3.75000	-38.58000	1264891395
5995	Diptera	Culicidae	Aedes	Aedes aegypti	SPECIES	-3.74000	-39.56000	1264876096
5996	Diptera	Culicidae	Aedes	Aedes aegypti	SPECIES	-3.74000	-43.85000	1264876115
5997	Diptera	Culicidae	Aedes	Aedes aegypti	SPECIES	-3.74000	-60.67000	1264876135
5998	Diptera	Culicidae	Aedes	Aedes aegypti	SPECIES	-3.74000	-73.25000	1264887662
5999	Diptera	Culicidae	Aedes	Aedes aegypti	SPECIES	-3.74000	-73.26000	1264887674
6000	Diptera	Culicidae	Aedes	Aedes aegypti	SPECIES	-3.74000	-38.52000	1264889142
6001	Diptera	Culicidae	Aedes	Aedes aegypti	SPECIES	-3.74000	-38.50000	1264890389
6002	Diptera	Culicidae	Aedes	Aedes aegypti	SPECIES	-3.74000	-73.24000	1264891167
6003	Diptera	Culicidae	Aedes	Aedes aegypti	SPECIES	-3.73000	-73.25000	1264887693
6004	Diptera	Culicidae	Aedes	Aedes aegypti	SPECIES	-3.73000	-73.26000	1264889479
6005	Diptera	Culicidae	Aedes	Aedes aegypti	SPECIES	-3.73000	-38.58000	1264890361
6006	Diptera	Culicidae	Aedes	Aedes aegypti	SPECIES	-3.72000	-73.24000	1264889488
6007	Diptera	Culicidae	Aedes	Aedes aegypti	SPECIES	-3.72000	-38.54000	1264889683
6008	Diptera	Culicidae	Aedes	Aedes aegypti	SPECIES	-3.72000	-38.52000	1264890354
6009	Diptera	Culicidae	Aedes	Aedes aegypti	SPECIES	-3.72000	-38.51000	1264891188
6010	Diptera	Culicidae	Aedes	Aedes aegypti	SPECIES	-3.71000	-40.83000	1264876110
6011	Diptera	Culicidae	Aedes	Aedes aegypti	SPECIES	-3.71000	-42.59000	1264876117
6012	Diptera	Culicidae	Aedes	Aedes aegypti	SPECIES	-3.71000	-42.83000	1264876127
6013	Diptera	Culicidae	Aedes	Aedes aegypti	SPECIES	-3.71000	-45.48000	1264876169
6014	Diptera	Culicidae	Aedes	Aedes aegypti	SPECIES	-3.71000	-38.61000	1264890352
6015	Diptera	Culicidae	Aedes	Aedes aegypti	SPECIES	-3.70000	-42.27000	1264876131
6016	Diptera	Culicidae	Aedes	Aedes aegypti	SPECIES	-3.70000	-50.55000	1264876150
6017	Diptera	Culicidae	Aedes	Aedes aegypti	SPECIES	-3.69000	-40.38000	1264890710
6018	Diptera	Culicidae	Aedes	Aedes aegypti	SPECIES	-3.68000	-40.73000	1264876128
6019	Diptera	Culicidae	Aedes	Aedes aegypti	SPECIES	-3.68000	-39.40000	1264876132
6020	Diptera	Culicidae	Aedes	Aedes aegypti	SPECIES	-3.67000	-80.57000	1264887680
6021	Diptera	Culicidae	Aedes	Aedes aegypti	SPECIES	-3.66083	133.77444	3901436383
6022	Diptera	Culicidae	Aedes	Aedes aegypti	SPECIES	-3.66000	-39.26000	1264876130
6023	Diptera	Culicidae	Aedes	Aedes aegypti	SPECIES	-3.66000	-40.96000	1264876137
6024	Diptera	Culicidae	Aedes	Aedes aegypti	SPECIES	-3.65000	-41.38000	1264876121
6025	Diptera	Culicidae	Aedes	Aedes aegypti	SPECIES	-3.65000	-45.14000	1264876123
6026	Diptera	Culicidae	Aedes	Aedes aegypti	SPECIES	-3.65000	-44.24000	1264876140
6027	Diptera	Culicidae	Aedes	Aedes aegypti	SPECIES	-3.65000	-43.24000	1264876156

№	Order	Family	Genus	Species	Taxon Rank	Latitude	Longitude	GBIF_ID
6028	Diptera	Culicidae	Aedes	Aedes aegypti	SPECIES	-3.64229	128.13730	3993826449
6029	Diptera	Culicidae	Aedes	Aedes aegypti	SPECIES	-3.64000	39.85000	1264887242
6030	Diptera	Culicidae	Aedes	Aedes aegypti	SPECIES	-3.63000	39.86000	1264887264
6031	Diptera	Culicidae	Aedes	Aedes aegypti	SPECIES	-3.63000	143.05000	1264891339
6032	Diptera	Culicidae	Aedes	Aedes aegypti	SPECIES	-3.62000	-39.51000	1264876136
6033	Diptera	Culicidae	Aedes	Aedes aegypti	SPECIES	-3.62000	30.58000	1264889740
6034	Diptera	Culicidae	Aedes	Aedes aegypti	SPECIES	-3.60000	-46.12000	1264876088
6035	Diptera	Culicidae	Aedes	Aedes aegypti	SPECIES	-3.60000	-42.32000	1264876101
6036	Diptera	Culicidae	Aedes	Aedes aegypti	SPECIES	-3.59000	-39.06000	1264876120
6037	Diptera	Culicidae	Aedes	Aedes aegypti	SPECIES	-3.59000	-41.81000	1264876157
6038	Diptera	Culicidae	Aedes	Aedes aegypti	SPECIES	-3.58000	-53.73000	1264876118
6039	Diptera	Culicidae	Aedes	Aedes aegypti	SPECIES	-3.58000	-39.89000	1264876125
6040	Diptera	Culicidae	Aedes	Aedes aegypti	SPECIES	-3.58000	-40.55000	1264876146
6041	Diptera	Culicidae	Aedes	Aedes aegypti	SPECIES	-3.57000	-40.44000	1264876144
6042	Diptera	Culicidae	Aedes	Aedes aegypti	SPECIES	-3.56000	-43.04000	1264876126
6043	Diptera	Culicidae	Aedes	Aedes aegypti	SPECIES	-3.56000	-42.54000	1264876141
6044	Diptera	Culicidae	Aedes	Aedes aegypti	SPECIES	-3.55000	-44.92000	1264876142
6045	Diptera	Culicidae	Aedes	Aedes aegypti	SPECIES	-3.55000	-44.74000	1264876148
6046	Diptera	Culicidae	Aedes	Aedes aegypti	SPECIES	-3.55000	-44.51000	1264876153
6047	Diptera	Culicidae	Aedes	Aedes aegypti	SPECIES	-3.55000	143.63000	1264891345
6048	Diptera	Culicidae	Aedes	Aedes aegypti	SPECIES	-3.54000	-42.43000	1264876159
6049	Diptera	Culicidae	Aedes	Aedes aegypti	SPECIES	-3.54000	-73.45000	1264887004
6050	Diptera	Culicidae	Aedes	Aedes aegypti	SPECIES	-3.53000	-39.38000	1264876113
6051	Diptera	Culicidae	Aedes	Aedes aegypti	SPECIES	-3.51000	-42.78000	1264876152
6052	Diptera	Culicidae	Aedes	Aedes aegypti	SPECIES	-3.51000	-42.08000	1264876168
6053	Diptera	Culicidae	Aedes	Aedes aegypti	SPECIES	-3.50000	-41.12000	1264876147
6054	Diptera	Culicidae	Aedes	Aedes aegypti	SPECIES	-3.50000	-40.16000	1264876155
6055	Diptera	Culicidae	Aedes	Aedes aegypti	SPECIES	-3.49000	-40.40000	1264876170
6056	Diptera	Culicidae	Aedes	Aedes aegypti	SPECIES	-3.49000	119.12000	1264889903
6057	Diptera	Culicidae	Aedes	Aedes aegypti	SPECIES	-3.49000	38.44000	1264890200
6058	Diptera	Culicidae	Aedes	Aedes aegypti	SPECIES	-3.48000	-45.33000	1264876129
6059	Diptera	Culicidae	Aedes	Aedes aegypti	SPECIES	-3.48000	-39.05000	1264876192
6060	Diptera	Culicidae	Aedes	Aedes aegypti	SPECIES	-3.48000	-80.26000	1264887667
6061	Diptera	Culicidae	Aedes	Aedes aegypti	SPECIES	-3.46000	114.82000	1264871949
6062	Diptera	Culicidae	Aedes	Aedes aegypti	SPECIES	-3.45000	115.99000	1264871828
6063	Diptera	Culicidae	Aedes	Aedes aegypti	SPECIES	-3.45000	-40.70000	1264876158
6064	Diptera	Culicidae	Aedes	Aedes aegypti	SPECIES	-3.43333	114.51667	3993821007
6065	Diptera	Culicidae	Aedes	Aedes aegypti	SPECIES	-3.43000	114.82000	1264871965
6066	Diptera	Culicidae	Aedes	Aedes aegypti	SPECIES	-3.43000	-41.52000	1264876174
6067	Diptera	Culicidae	Aedes	Aedes aegypti	SPECIES	-3.42000	114.87000	1264871934
6068	Diptera	Culicidae	Aedes	Aedes aegypti	SPECIES	-3.42000	-43.80000	1264876143

№	Order	Family	Genus	Species	Taxon Rank	Latitude	Longitude	GBIF_ID
6069	Diptera	Culicidae	Aedes	Aedes aegypti	SPECIES	-3.42000	-39.17000	1264876187
6070	Diptera	Culicidae	Aedes	Aedes aegypti	SPECIES	-3.42000	119.34000	1264889886
6071	Diptera	Culicidae	Aedes	Aedes aegypti	SPECIES	-3.41000	114.85000	1264871930
6072	Diptera	Culicidae	Aedes	Aedes aegypti	SPECIES	-3.41000	114.90000	1264871951
6073	Diptera	Culicidae	Aedes	Aedes aegypti	SPECIES	-3.41000	114.83000	1264871954
6074	Diptera	Culicidae	Aedes	Aedes aegypti	SPECIES	-3.41000	-45.07000	1264876149
6075	Diptera	Culicidae	Aedes	Aedes aegypti	SPECIES	-3.41000	114.88000	1264876479
6076	Diptera	Culicidae	Aedes	Aedes aegypti	SPECIES	-3.40000	114.88000	1264871935
6077	Diptera	Culicidae	Aedes	Aedes aegypti	SPECIES	-3.40000	114.87000	1264871947
6078	Diptera	Culicidae	Aedes	Aedes aegypti	SPECIES	-3.40000	-43.63000	1264876163
6079	Diptera	Culicidae	Aedes	Aedes aegypti	SPECIES	-3.40000	-46.00000	1264876182
6080	Diptera	Culicidae	Aedes	Aedes aegypti	SPECIES	-3.39000	114.90000	1264871931
6081	Diptera	Culicidae	Aedes	Aedes aegypti	SPECIES	-3.39000	114.88000	1264871945
6082	Diptera	Culicidae	Aedes	Aedes aegypti	SPECIES	-3.39000	114.87000	1264871966
6083	Diptera	Culicidae	Aedes	Aedes aegypti	SPECIES	-3.39000	-41.87000	1264876164
6084	Diptera	Culicidae	Aedes	Aedes aegypti	SPECIES	-3.39000	39.93000	1264887230
6085	Diptera	Culicidae	Aedes	Aedes aegypti	SPECIES	-3.38000	-39.34000	1264876180
6086	Diptera	Culicidae	Aedes	Aedes aegypti	SPECIES	-3.38000	-43.11000	1264891316
6087	Diptera	Culicidae	Aedes	Aedes aegypti	SPECIES	-3.37167	35.85500	4065056061
6088	Diptera	Culicidae	Aedes	Aedes aegypti	SPECIES	-3.37000	-39.59000	1264876166
6089	Diptera	Culicidae	Aedes	Aedes aegypti	SPECIES	-3.36000	-44.31000	1264876175
6090	Diptera	Culicidae	Aedes	Aedes aegypti	SPECIES	-3.36000	-42.02000	1264876206
6091	Diptera	Culicidae	Aedes	Aedes aegypti	SPECIES	-3.35000	-43.92000	1264876178
6092	Diptera	Culicidae	Aedes	Aedes aegypti	SPECIES	-3.35000	114.60000	1264876496
6093	Diptera	Culicidae	Aedes	Aedes aegypti	SPECIES	-3.33076	35.70220	4065055455
6094	Diptera	Culicidae	Aedes	Aedes aegypti	SPECIES	-3.33000	-43.37000	1264876186
6095	Diptera	Culicidae	Aedes	Aedes aegypti	SPECIES	-3.32000	-42.13000	1264876162
6096	Diptera	Culicidae	Aedes	Aedes aegypti	SPECIES	-3.32000	-40.68000	1264876176
6097	Diptera	Culicidae	Aedes	Aedes aegypti	SPECIES	-3.32000	114.62000	1264876473
6098	Diptera	Culicidae	Aedes	Aedes aegypti	SPECIES	-3.32000	114.57000	1264876482
6099	Diptera	Culicidae	Aedes	Aedes aegypti	SPECIES	-3.32000	114.59000	1264876518
6100	Diptera	Culicidae	Aedes	Aedes aegypti	SPECIES	-3.31000	115.98000	1264871849
6101	Diptera	Culicidae	Aedes	Aedes aegypti	SPECIES	-3.31000	-41.41000	1264891275
6102	Diptera	Culicidae	Aedes	Aedes aegypti	SPECIES	-3.30000	-42.89000	1264876196
6103	Diptera	Culicidae	Aedes	Aedes aegypti	SPECIES	-3.30000	-52.61000	1264876218
6104	Diptera	Culicidae	Aedes	Aedes aegypti	SPECIES	-3.29709	-79.88732	4054820582
6105	Diptera	Culicidae	Aedes	Aedes aegypti	SPECIES	-3.29000	-42.44000	1264876145
6106	Diptera	Culicidae	Aedes	Aedes aegypti	SPECIES	-3.29000	114.59000	1264876517
6107	Diptera	Culicidae	Aedes	Aedes aegypti	SPECIES	-3.29000	-60.62000	1264890410
6108	Diptera	Culicidae	Aedes	Aedes aegypti	SPECIES	-3.28000	-40.14000	1264876160
6109	Diptera	Culicidae	Aedes	Aedes aegypti	SPECIES	-3.28000	-60.90000	1264876167

№	Order	Family	Genus	Species	Taxon Rank	Latitude	Longitude	GBIF_ID
6110	Diptera	Culicidae	Aedes	Aedes aegypti	SPECIES	-3.28000	-40.46000	1264876181
6111	Diptera	Culicidae	Aedes	Aedes aegypti	SPECIES	-3.27000	-41.73000	1264876173
6112	Diptera	Culicidae	Aedes	Aedes aegypti	SPECIES	-3.27000	-40.96000	1264876193
6113	Diptera	Culicidae	Aedes	Aedes aegypti	SPECIES	-3.26000	102.87000	1264876528
6114	Diptera	Culicidae	Aedes	Aedes aegypti	SPECIES	-3.25000	-55.02000	1264876139
6115	Diptera	Culicidae	Aedes	Aedes aegypti	SPECIES	-3.25000	-44.59000	1264876154
6116	Diptera	Culicidae	Aedes	Aedes aegypti	SPECIES	-3.25000	-45.95000	1264876213
6117	Diptera	Culicidae	Aedes	Aedes aegypti	SPECIES	-3.24000	-45.33000	1264876171
6118	Diptera	Culicidae	Aedes	Aedes aegypti	SPECIES	-3.24000	-39.78000	1264891015
6119	Diptera	Culicidae	Aedes	Aedes aegypti	SPECIES	-3.23000	-39.79000	1264876214
6120	Diptera	Culicidae	Aedes	Aedes aegypti	SPECIES	-3.23000	40.13000	1264887215
6121	Diptera	Culicidae	Aedes	Aedes aegypti	SPECIES	-3.23000	-60.14000	1264890569
6122	Diptera	Culicidae	Aedes	Aedes aegypti	SPECIES	-3.22000	40.12000	1264887596
6123	Diptera	Culicidae	Aedes	Aedes aegypti	SPECIES	-3.22000	40.07000	1264889592
6124	Diptera	Culicidae	Aedes	Aedes aegypti	SPECIES	-3.22000	40.11000	1264890464
6125	Diptera	Culicidae	Aedes	Aedes aegypti	SPECIES	-3.21000	-42.58000	1264876151
6126	Diptera	Culicidae	Aedes	Aedes aegypti	SPECIES	-3.19000	-40.26000	1264876183
6127	Diptera	Culicidae	Aedes	Aedes aegypti	SPECIES	-3.17000	-51.99000	1264876165
6128	Diptera	Culicidae	Aedes	Aedes aegypti	SPECIES	-3.17000	-49.68000	1264876197
6129	Diptera	Culicidae	Aedes	Aedes aegypti	SPECIES	-3.17000	-53.26000	1264876208
6130	Diptera	Culicidae	Aedes	Aedes aegypti	SPECIES	-3.17000	115.43000	1264891281
6131	Diptera	Culicidae	Aedes	Aedes aegypti	SPECIES	-3.16000	-41.62000	1264876219
6132	Diptera	Culicidae	Aedes	Aedes aegypti	SPECIES	-3.15000	-40.64000	1264876177
6133	Diptera	Culicidae	Aedes	Aedes aegypti	SPECIES	-3.15000	-44.32000	1264876237
6134	Diptera	Culicidae	Aedes	Aedes aegypti	SPECIES	-3.14000	-58.78000	1264876185
6135	Diptera	Culicidae	Aedes	Aedes aegypti	SPECIES	-3.13925	-60.00159	3109138342
6136	Diptera	Culicidae	Aedes	Aedes aegypti	SPECIES	-3.13654	-60.00138	3499649065
6137	Diptera	Culicidae	Aedes	Aedes aegypti	SPECIES	-3.13000	-44.71000	1264876199
6138	Diptera	Culicidae	Aedes	Aedes aegypti	SPECIES	-3.13000	-63.09000	1264876232
6139	Diptera	Culicidae	Aedes	Aedes aegypti	SPECIES	-3.12000	-47.65000	1264876241
6140	Diptera	Culicidae	Aedes	Aedes aegypti	SPECIES	-3.12000	40.00000	1264887234
6141	Diptera	Culicidae	Aedes	Aedes aegypti	SPECIES	-3.12000	-60.02000	1264890440
6142	Diptera	Culicidae	Aedes	Aedes aegypti	SPECIES	-3.12000	-60.01000	1264890457
6143	Diptera	Culicidae	Aedes	Aedes aegypti	SPECIES	-3.12000	-59.93000	1264890585
6144	Diptera	Culicidae	Aedes	Aedes aegypti	SPECIES	-3.11333	-60.02528	1933070211
6145	Diptera	Culicidae	Aedes	Aedes aegypti	SPECIES	-3.11000	-41.48000	1264876252
6146	Diptera	Culicidae	Aedes	Aedes aegypti	SPECIES	-3.11000	-60.06000	1264890444
6147	Diptera	Culicidae	Aedes	Aedes aegypti	SPECIES	-3.11000	-59.98000	1264890573
6148	Diptera	Culicidae	Aedes	Aedes aegypti	SPECIES	-3.11000	-59.99000	1264890577
6149	Diptera	Culicidae	Aedes	Aedes aegypti	SPECIES	-3.11000	-60.02000	1264891344
6150	Diptera	Culicidae	Aedes	Aedes aegypti	SPECIES	-3.10000	-44.07000	1264876212

№	Order	Family	Genus	Species	Taxon Rank	Latitude	Longitude	GBIF_ID
6151	Diptera	Culicidae	Aedes	Aedes aegypti	SPECIES	-3.10000	-60.02000	1264888496
6152	Diptera	Culicidae	Aedes	Aedes aegypti	SPECIES	-3.10000	-60.05000	1264891335
6153	Diptera	Culicidae	Aedes	Aedes aegypti	SPECIES	-3.09000	-43.92000	1264876204
6154	Diptera	Culicidae	Aedes	Aedes aegypti	SPECIES	-3.09000	-60.25000	1264876224
6155	Diptera	Culicidae	Aedes	Aedes aegypti	SPECIES	-3.09000	-59.96000	1264890416
6156	Diptera	Culicidae	Aedes	Aedes aegypti	SPECIES	-3.08000	-41.24000	1264876210
6157	Diptera	Culicidae	Aedes	Aedes aegypti	SPECIES	-3.08000	-60.03000	1264891329
6158	Diptera	Culicidae	Aedes	Aedes aegypti	SPECIES	-3.07000	-39.85000	1264876172
6159	Diptera	Culicidae	Aedes	Aedes aegypti	SPECIES	-3.07000	-46.55000	1264876189
6160	Diptera	Culicidae	Aedes	Aedes aegypti	SPECIES	-3.07000	-40.30000	1264876233
6161	Diptera	Culicidae	Aedes	Aedes aegypti	SPECIES	-3.07000	-59.96000	1264890557
6162	Diptera	Culicidae	Aedes	Aedes aegypti	SPECIES	-3.06000	-45.79000	1264876191
6163	Diptera	Culicidae	Aedes	Aedes aegypti	SPECIES	-3.06000	-40.04000	1264876200
6164	Diptera	Culicidae	Aedes	Aedes aegypti	SPECIES	-3.06000	-44.98000	1264876220
6165	Diptera	Culicidae	Aedes	Aedes aegypti	SPECIES	-3.06000	-60.01000	1264887934
6166	Diptera	Culicidae	Aedes	Aedes aegypti	SPECIES	-3.06000	-59.94000	1264890423
6167	Diptera	Culicidae	Aedes	Aedes aegypti	SPECIES	-3.05000	-59.94000	1264886021
6168	Diptera	Culicidae	Aedes	Aedes aegypti	SPECIES	-3.04000	40.14000	1264890206
6169	Diptera	Culicidae	Aedes	Aedes aegypti	SPECIES	-3.02000	-59.97000	1264890574
6170	Diptera	Culicidae	Aedes	Aedes aegypti	SPECIES	-3.01000	-44.78000	1264876203
6171	Diptera	Culicidae	Aedes	Aedes aegypti	SPECIES	-3.00000	-41.14000	1264876223
6172	Diptera	Culicidae	Aedes	Aedes aegypti	SPECIES	-3.00000	104.75000	1264876542
6173	Diptera	Culicidae	Aedes	Aedes aegypti	SPECIES	-2.99000	-41.35000	1264876188
6174	Diptera	Culicidae	Aedes	Aedes aegypti	SPECIES	-2.99000	104.75000	1264876494
6175	Diptera	Culicidae	Aedes	Aedes aegypti	SPECIES	-2.99000	104.76000	1264890666
6176	Diptera	Culicidae	Aedes	Aedes aegypti	SPECIES	-2.98000	104.73000	1264871843
6177	Diptera	Culicidae	Aedes	Aedes aegypti	SPECIES	-2.98000	-45.01000	1264876209
6178	Diptera	Culicidae	Aedes	Aedes aegypti	SPECIES	-2.98000	104.75000	1264890761
6179	Diptera	Culicidae	Aedes	Aedes aegypti	SPECIES	-2.97000	104.67000	1264871809
6180	Diptera	Culicidae	Aedes	Aedes aegypti	SPECIES	-2.97000	-40.81000	1264876205
6181	Diptera	Culicidae	Aedes	Aedes aegypti	SPECIES	-2.97000	-43.81000	1264876221
6182	Diptera	Culicidae	Aedes	Aedes aegypti	SPECIES	-2.97000	104.75000	1264876513
6183	Diptera	Culicidae	Aedes	Aedes aegypti	SPECIES	-2.96000	104.73000	1264871738
6184	Diptera	Culicidae	Aedes	Aedes aegypti	SPECIES	-2.96000	-44.21000	1264876207
6185	Diptera	Culicidae	Aedes	Aedes aegypti	SPECIES	-2.96000	104.75000	1264876524
6186	Diptera	Culicidae	Aedes	Aedes aegypti	SPECIES	-2.96000	-41.76000	1264890827
6187	Diptera	Culicidae	Aedes	Aedes aegypti	SPECIES	-2.95000	-46.02000	1264876194
6188	Diptera	Culicidae	Aedes	Aedes aegypti	SPECIES	-2.95000	-41.76000	1264876222
6189	Diptera	Culicidae	Aedes	Aedes aegypti	SPECIES	-2.95000	-42.05000	1264876234
6190	Diptera	Culicidae	Aedes	Aedes aegypti	SPECIES	-2.95000	104.81000	1264876481
6191	Diptera	Culicidae	Aedes	Aedes aegypti	SPECIES	-2.95000	-60.03000	1264890662

№	Order	Family	Genus	Species	Taxon Rank	Latitude	Longitude	GBIF_ID
6192	Diptera	Culicidae	Aedes	Aedes aegypti	SPECIES	-2.94000	-47.97000	1264876198
6193	Diptera	Culicidae	Aedes	Aedes aegypti	SPECIES	-2.94000	104.80000	1264876531
6194	Diptera	Culicidae	Aedes	Aedes aegypti	SPECIES	-2.93000	-44.38000	1264876228
6195	Diptera	Culicidae	Aedes	Aedes aegypti	SPECIES	-2.93000	104.74000	1264876475
6196	Diptera	Culicidae	Aedes	Aedes aegypti	SPECIES	-2.92000	-42.18000	1264876240
6197	Diptera	Culicidae	Aedes	Aedes aegypti	SPECIES	-2.92000	-42.41000	1264876262
6198	Diptera	Culicidae	Aedes	Aedes aegypti	SPECIES	-2.90000	-48.75000	1264876226
6199	Diptera	Culicidae	Aedes	Aedes aegypti	SPECIES	-2.90000	104.72000	1264876503
6200	Diptera	Culicidae	Aedes	Aedes aegypti	SPECIES	-2.90000	104.73000	1264876558
6201	Diptera	Culicidae	Aedes	Aedes aegypti	SPECIES	-2.89000	-40.30000	1264876247
6202	Diptera	Culicidae	Aedes	Aedes aegypti	SPECIES	-2.89000	104.70000	1264876529
6203	Diptera	Culicidae	Aedes	Aedes aegypti	SPECIES	-2.88000	-40.50000	1264876184
6204	Diptera	Culicidae	Aedes	Aedes aegypti	SPECIES	-2.84000	-42.98000	1264876201
6205	Diptera	Culicidae	Aedes	Aedes aegypti	SPECIES	-2.84000	-42.61000	1264876231
6206	Diptera	Culicidae	Aedes	Aedes aegypti	SPECIES	-2.83000	-41.81000	1264876161
6207	Diptera	Culicidae	Aedes	Aedes aegypti	SPECIES	-2.83000	-55.10000	1264890895
6208	Diptera	Culicidae	Aedes	Aedes aegypti	SPECIES	-2.82000	-44.10000	1264876179
6209	Diptera	Culicidae	Aedes	Aedes aegypti	SPECIES	-2.81000	-55.19000	1264876202
6210	Diptera	Culicidae	Aedes	Aedes aegypti	SPECIES	-2.79000	-56.69000	1264876190
6211	Diptera	Culicidae	Aedes	Aedes aegypti	SPECIES	-2.77000	104.73000	1264871672
6212	Diptera	Culicidae	Aedes	Aedes aegypti	SPECIES	-2.76000	104.96000	1264876509
6213	Diptera	Culicidae	Aedes	Aedes aegypti	SPECIES	-2.67000	-43.34000	1264876215
6214	Diptera	Culicidae	Aedes	Aedes aegypti	SPECIES	-2.66000	-48.24000	1264876238
6215	Diptera	Culicidae	Aedes	Aedes aegypti	SPECIES	-2.65000	-56.19000	1264876243
6216	Diptera	Culicidae	Aedes	Aedes aegypti	SPECIES	-2.65000	-43.19000	1264876290
6217	Diptera	Culicidae	Aedes	Aedes aegypti	SPECIES	-2.63000	-43.59000	1264876195
6218	Diptera	Culicidae	Aedes	Aedes aegypti	SPECIES	-2.63000	-44.30000	1264876225
6219	Diptera	Culicidae	Aedes	Aedes aegypti	SPECIES	-2.63000	-44.28000	1264890866
6220	Diptera	Culicidae	Aedes	Aedes aegypti	SPECIES	-2.63000	-60.26000	1264890892
6221	Diptera	Culicidae	Aedes	Aedes aegypti	SPECIES	-2.62000	-50.92000	1264876254
6222	Diptera	Culicidae	Aedes	Aedes aegypti	SPECIES	-2.60000	-44.35000	1264891347
6223	Diptera	Culicidae	Aedes	Aedes aegypti	SPECIES	-2.59000	-45.37000	1264876271
6224	Diptera	Culicidae	Aedes	Aedes aegypti	SPECIES	-2.58333	150.75000	1633667685
6225	Diptera	Culicidae	Aedes	Aedes aegypti	SPECIES	-2.58000	-51.91000	1264891467
6226	Diptera	Culicidae	Aedes	Aedes aegypti	SPECIES	-2.57000	140.51000	1264871601
6227	Diptera	Culicidae	Aedes	Aedes aegypti	SPECIES	-2.57000	-59.98000	1264876313
6228	Diptera	Culicidae	Aedes	Aedes aegypti	SPECIES	-2.56700	150.80000	177195209
6229	Diptera	Culicidae	Aedes	Aedes aegypti	SPECIES	-2.56000	-44.88000	1264876227
6230	Diptera	Culicidae	Aedes	Aedes aegypti	SPECIES	-2.56000	-44.23000	1264891328
6231	Diptera	Culicidae	Aedes	Aedes aegypti	SPECIES	-2.55000	-49.48000	1264876211
6232	Diptera	Culicidae	Aedes	Aedes aegypti	SPECIES	-2.54000	-49.03000	1264876235

№	Order	Family	Genus	Species	Taxon Rank	Latitude	Longitude	GBIF_ID
6233	Diptera	Culicidae	Aedes	Aedes aegypti	SPECIES	-2.54000	37.53000	1264890072
6234	Diptera	Culicidae	Aedes	Aedes aegypti	SPECIES	-2.53000	-45.82000	1264876264
6235	Diptera	Culicidae	Aedes	Aedes aegypti	SPECIES	-2.53000	112.95000	1264876487
6236	Diptera	Culicidae	Aedes	Aedes aegypti	SPECIES	-2.52213	133.43552	3993826517
6237	Diptera	Culicidae	Aedes	Aedes aegypti	SPECIES	-2.51000	-44.12000	1264876257
6238	Diptera	Culicidae	Aedes	Aedes aegypti	SPECIES	-2.51000	-59.60000	1264876294
6239	Diptera	Culicidae	Aedes	Aedes aegypti	SPECIES	-2.51000	-44.31000	1264888123
6240	Diptera	Culicidae	Aedes	Aedes aegypti	SPECIES	-2.51000	-44.30000	1264889157
6241	Diptera	Culicidae	Aedes	Aedes aegypti	SPECIES	-2.50827	-44.26851	2550067381
6242	Diptera	Culicidae	Aedes	Aedes aegypti	SPECIES	-2.50000	-45.13000	1264876279
6243	Diptera	Culicidae	Aedes	Aedes aegypti	SPECIES	-2.48000	-50.21000	1264876297
6244	Diptera	Culicidae	Aedes	Aedes aegypti	SPECIES	-2.48000	-44.23000	1264890356
6245	Diptera	Culicidae	Aedes	Aedes aegypti	SPECIES	-2.47000	-45.54000	1264876239
6246	Diptera	Culicidae	Aedes	Aedes aegypti	SPECIES	-2.44306	-54.70830	1456937823
6247	Diptera	Culicidae	Aedes	Aedes aegypti	SPECIES	-2.44000	-44.10000	1264876216
6248	Diptera	Culicidae	Aedes	Aedes aegypti	SPECIES	-2.44000	-44.80000	1264876246
6249	Diptera	Culicidae	Aedes	Aedes aegypti	SPECIES	-2.43000	-44.78000	1264891342
6250	Diptera	Culicidae	Aedes	Aedes aegypti	SPECIES	-2.42000	-45.96000	1264876275
6251	Diptera	Culicidae	Aedes	Aedes aegypti	SPECIES	-2.41000	-48.15000	1264891438
6252	Diptera	Culicidae	Aedes	Aedes aegypti	SPECIES	-2.39000	-49.92000	1264876263
6253	Diptera	Culicidae	Aedes	Aedes aegypti	SPECIES	-2.39000	-46.92000	1264876298
6254	Diptera	Culicidae	Aedes	Aedes aegypti	SPECIES	-2.37000	-44.54000	1264876333
6255	Diptera	Culicidae	Aedes	Aedes aegypti	SPECIES	-2.34000	-46.15000	1264876217
6256	Diptera	Culicidae	Aedes	Aedes aegypti	SPECIES	-2.34000	119.42000	1264891071
6257	Diptera	Culicidae	Aedes	Aedes aegypti	SPECIES	-2.33000	106.22000	1264889291
6258	Diptera	Culicidae	Aedes	Aedes aegypti	SPECIES	-2.31000	-45.77000	1264876258
6259	Diptera	Culicidae	Aedes	Aedes aegypti	SPECIES	-2.28000	-49.51000	1264876245
6260	Diptera	Culicidae	Aedes	Aedes aegypti	SPECIES	-2.26000	-47.74000	1264876266
6261	Diptera	Culicidae	Aedes	Aedes aegypti	SPECIES	-2.25000	113.95000	1264871920
6262	Diptera	Culicidae	Aedes	Aedes aegypti	SPECIES	-2.25000	-44.83000	1264876250
6263	Diptera	Culicidae	Aedes	Aedes aegypti	SPECIES	-2.24000	113.90000	1264871938
6264	Diptera	Culicidae	Aedes	Aedes aegypti	SPECIES	-2.22000	-47.07000	1264876274
6265	Diptera	Culicidae	Aedes	Aedes aegypti	SPECIES	-2.20000	113.79000	1264871940
6266	Diptera	Culicidae	Aedes	Aedes aegypti	SPECIES	-2.17000	-52.62000	1264876236
6267	Diptera	Culicidae	Aedes	Aedes aegypti	SPECIES	-2.16330	-79.96494	1933070209
6268	Diptera	Culicidae	Aedes	Aedes aegypti	SPECIES	-2.15000	-44.63000	1264876255
6269	Diptera	Culicidae	Aedes	Aedes aegypti	SPECIES	-2.13000	105.97000	1264871796
6270	Diptera	Culicidae	Aedes	Aedes aegypti	SPECIES	-2.12000	-45.41000	1264876276
6271	Diptera	Culicidae	Aedes	Aedes aegypti	SPECIES	-2.08840	132.19667	3901436442
6272	Diptera	Culicidae	Aedes	Aedes aegypti	SPECIES	-2.08000	-49.12000	1264876251
6273	Diptera	Culicidae	Aedes	Aedes aegypti	SPECIES	-2.07000	-47.22000	1264876260

№	Order	Family	Genus	Species	Taxon Rank	Latitude	Longitude	GBIF_ID
6274	Diptera	Culicidae	Aedes	Aedes aegypti	SPECIES	-2.07000	-44.83000	1264876282
6275	Diptera	Culicidae	Aedes	Aedes aegypti	SPECIES	-2.04000	-45.82000	1264876284
6276	Diptera	Culicidae	Aedes	Aedes aegypti	SPECIES	-2.03000	106.10000	1264871767
6277	Diptera	Culicidae	Aedes	Aedes aegypti	SPECIES	-2.03000	105.86000	1264871791
6278	Diptera	Culicidae	Aedes	Aedes aegypti	SPECIES	-2.03000	-48.46000	1264876229
6279	Diptera	Culicidae	Aedes	Aedes aegypti	SPECIES	-2.01000	-46.42000	1264876249
6280	Diptera	Culicidae	Aedes	Aedes aegypti	SPECIES	-2.00000	-46.01000	1264876253
6281	Diptera	Culicidae	Aedes	Aedes aegypti	SPECIES	-1.99000	-47.51000	1264876277
6282	Diptera	Culicidae	Aedes	Aedes aegypti	SPECIES	-1.98000	-44.60000	1264876265
6283	Diptera	Culicidae	Aedes	Aedes aegypti	SPECIES	-1.93000	-47.78000	1264876288
6284	Diptera	Culicidae	Aedes	Aedes aegypti	SPECIES	-1.92000	105.89000	1264871756
6285	Diptera	Culicidae	Aedes	Aedes aegypti	SPECIES	-1.92000	106.12000	1264871777
6286	Diptera	Culicidae	Aedes	Aedes aegypti	SPECIES	-1.92000	-53.62000	1264876242
6287	Diptera	Culicidae	Aedes	Aedes aegypti	SPECIES	-1.91804	30.06401	2529407529
6288	Diptera	Culicidae	Aedes	Aedes aegypti	SPECIES	-1.91000	-44.63000	1264876268
6289	Diptera	Culicidae	Aedes	Aedes aegypti	SPECIES	-1.91000	-56.42000	1264876283
6290	Diptera	Culicidae	Aedes	Aedes aegypti	SPECIES	-1.89000	-45.00000	1264876272
6291	Diptera	Culicidae	Aedes	Aedes aegypti	SPECIES	-1.89000	-49.46000	1264876295
6292	Diptera	Culicidae	Aedes	Aedes aegypti	SPECIES	-1.89000	-46.10000	1264876306
6293	Diptera	Culicidae	Aedes	Aedes aegypti	SPECIES	-1.88000	-47.98000	1264876230
6294	Diptera	Culicidae	Aedes	Aedes aegypti	SPECIES	-1.87000	106.04000	1264871798
6295	Diptera	Culicidae	Aedes	Aedes aegypti	SPECIES	-1.86000	106.12000	1264871793
6296	Diptera	Culicidae	Aedes	Aedes aegypti	SPECIES	-1.84000	-47.41000	1264876308
6297	Diptera	Culicidae	Aedes	Aedes aegypti	SPECIES	-1.81000	106.10000	1264871760
6298	Diptera	Culicidae	Aedes	Aedes aegypti	SPECIES	-1.80000	-61.02000	1264876248
6299	Diptera	Culicidae	Aedes	Aedes aegypti	SPECIES	-1.77000	-46.19000	1264876307
6300	Diptera	Culicidae	Aedes	Aedes aegypti	SPECIES	-1.76000	105.92000	1264871770
6301	Diptera	Culicidae	Aedes	Aedes aegypti	SPECIES	-1.74000	-48.84000	1264876312
6302	Diptera	Culicidae	Aedes	Aedes aegypti	SPECIES	-1.73000	-46.90000	1264876273
6303	Diptera	Culicidae	Aedes	Aedes aegypti	SPECIES	-1.73000	-45.78000	1264876286
6304	Diptera	Culicidae	Aedes	Aedes aegypti	SPECIES	-1.71000	-45.46000	1264876304
6305	Diptera	Culicidae	Aedes	Aedes aegypti	SPECIES	-1.69000	-45.12000	1264876335
6306	Diptera	Culicidae	Aedes	Aedes aegypti	SPECIES	-1.68000	120.50000	1264891073
6307	Diptera	Culicidae	Aedes	Aedes aegypti	SPECIES	-1.66000	-45.94000	1264876319
6308	Diptera	Culicidae	Aedes	Aedes aegypti	SPECIES	-1.65000	-48.08000	1264876332
6309	Diptera	Culicidae	Aedes	Aedes aegypti	SPECIES	-1.65000	11.98000	1264890606
6310	Diptera	Culicidae	Aedes	Aedes aegypti	SPECIES	-1.64000	105.89000	1264871775
6311	Diptera	Culicidae	Aedes	Aedes aegypti	SPECIES	-1.63000	13.60000	1264890204
6312	Diptera	Culicidae	Aedes	Aedes aegypti	SPECIES	-1.62000	103.61000	1264871933
6313	Diptera	Culicidae	Aedes	Aedes aegypti	SPECIES	-1.61000	105.84000	1264871782
6314	Diptera	Culicidae	Aedes	Aedes aegypti	SPECIES	-1.57000	-50.01000	1264876357

№	Order	Family	Genus	Species	Taxon Rank	Latitude	Longitude	GBIF_ID
6315	Diptera	Culicidae	Aedes	Aedes aegypti	SPECIES	-1.56000	-46.46000	1264876269
6316	Diptera	Culicidae	Aedes	Aedes aegypti	SPECIES	-1.55000	-47.62000	1264876300
6317	Diptera	Culicidae	Aedes	Aedes aegypti	SPECIES	-1.55000	35.02000	3351615048
6318	Diptera	Culicidae	Aedes	Aedes aegypti	SPECIES	-1.53000	-45.06000	1264876327
6319	Diptera	Culicidae	Aedes	Aedes aegypti	SPECIES	-1.49000	-47.15000	1264876340
6320	Diptera	Culicidae	Aedes	Aedes aegypti	SPECIES	-1.46432	-48.50213	177060144
6321	Diptera	Culicidae	Aedes	Aedes aegypti	SPECIES	-1.46000	-47.95000	1264876291
6322	Diptera	Culicidae	Aedes	Aedes aegypti	SPECIES	-1.45000	-48.64000	1264876310
6323	Diptera	Culicidae	Aedes	Aedes aegypti	SPECIES	-1.44000	-48.50000	1264890871
6324	Diptera	Culicidae	Aedes	Aedes aegypti	SPECIES	-1.43000	-49.71000	1264876334
6325	Diptera	Culicidae	Aedes	Aedes aegypti	SPECIES	-1.43000	-48.50000	1264889155
6326	Diptera	Culicidae	Aedes	Aedes aegypti	SPECIES	-1.40000	-48.32000	1264876359
6327	Diptera	Culicidae	Aedes	Aedes aegypti	SPECIES	-1.40000	-48.45000	1264889461
6328	Diptera	Culicidae	Aedes	Aedes aegypti	SPECIES	-1.39000	-46.00000	1264876324
6329	Diptera	Culicidae	Aedes	Aedes aegypti	SPECIES	-1.39000	-47.31000	1264876343
6330	Diptera	Culicidae	Aedes	Aedes aegypti	SPECIES	-1.39000	-68.79000	1264876350
6331	Diptera	Culicidae	Aedes	Aedes aegypti	SPECIES	-1.38000	-48.38000	1264890842
6332	Diptera	Culicidae	Aedes	Aedes aegypti	SPECIES	-1.37000	-47.54000	1264876259
6333	Diptera	Culicidae	Aedes	Aedes aegypti	SPECIES	-1.37000	-48.12000	1264876270
6334	Diptera	Culicidae	Aedes	Aedes aegypti	SPECIES	-1.37000	-45.71000	1264876287
6335	Diptera	Culicidae	Aedes	Aedes aegypti	SPECIES	-1.37000	-45.90000	1264876314
6336	Diptera	Culicidae	Aedes	Aedes aegypti	SPECIES	-1.37000	-48.38000	1264888114
6337	Diptera	Culicidae	Aedes	Aedes aegypti	SPECIES	-1.36057	36.72159	1950037052
6338	Diptera	Culicidae	Aedes	Aedes aegypti	SPECIES	-1.36000	-48.28000	1264876285
6339	Diptera	Culicidae	Aedes	Aedes aegypti	SPECIES	-1.34000	-48.39000	1264876305
6340	Diptera	Culicidae	Aedes	Aedes aegypti	SPECIES	-1.31000	-49.29000	1264876244
6341	Diptera	Culicidae	Aedes	Aedes aegypti	SPECIES	-1.29000	36.82000	1264886638
6342	Diptera	Culicidae	Aedes	Aedes aegypti	SPECIES	-1.29000	36.85000	1264889890
6343	Diptera	Culicidae	Aedes	Aedes aegypti	SPECIES	-1.27000	-48.46000	1264876267
6344	Diptera	Culicidae	Aedes	Aedes aegypti	SPECIES	-1.27000	-47.94000	1264876301
6345	Diptera	Culicidae	Aedes	Aedes aegypti	SPECIES	-1.26000	-69.73000	1264890434
6346	Diptera	Culicidae	Aedes	Aedes aegypti	SPECIES	-1.26000	-48.42000	1264890893
6347	Diptera	Culicidae	Aedes	Aedes aegypti	SPECIES	-1.22139	36.89632	3858505176
6348	Diptera	Culicidae	Aedes	Aedes aegypti	SPECIES	-1.22000	-60.02000	1264876323
6349	Diptera	Culicidae	Aedes	Aedes aegypti	SPECIES	-1.22000	36.83000	3968999538
6350	Diptera	Culicidae	Aedes	Aedes aegypti	SPECIES	-1.21000	-47.74000	1264876325
6351	Diptera	Culicidae	Aedes	Aedes aegypti	SPECIES	-1.19000	136.11000	1264871592
6352	Diptera	Culicidae	Aedes	Aedes aegypti	SPECIES	-1.19000	-47.10000	1264876256
6353	Diptera	Culicidae	Aedes	Aedes aegypti	SPECIES	-1.19000	-48.25000	1264876349
6354	Diptera	Culicidae	Aedes	Aedes aegypti	SPECIES	-1.18000	136.08000	1264871590
6355	Diptera	Culicidae	Aedes	Aedes aegypti	SPECIES	-1.18000	-46.79000	1264876280

№	Order	Family	Genus	Species	Taxon Rank	Latitude	Longitude	GBIF_ID
6356	Diptera	Culicidae	Aedes	Aedes aegypti	SPECIES	-1.17000	-47.57000	1264876311
6357	Diptera	Culicidae	Aedes	Aedes aegypti	SPECIES	-1.17000	-47.39000	1264876317
6358	Diptera	Culicidae	Aedes	Aedes aegypti	SPECIES	-1.16000	-50.57000	1264876338
6359	Diptera	Culicidae	Aedes	Aedes aegypti	SPECIES	-1.13000	-47.27000	1264876303
6360	Diptera	Culicidae	Aedes	Aedes aegypti	SPECIES	-1.12000	-51.67000	1264876337
6361	Diptera	Culicidae	Aedes	Aedes aegypti	SPECIES	-1.11000	-49.08000	1264876296
6362	Diptera	Culicidae	Aedes	Aedes aegypti	SPECIES	-1.11000	-57.74000	1264876322
6363	Diptera	Culicidae	Aedes	Aedes aegypti	SPECIES	-1.10000	-46.50000	1264876375
6364	Diptera	Culicidae	Aedes	Aedes aegypti	SPECIES	-1.10000	-48.22000	1264876400
6365	Diptera	Culicidae	Aedes	Aedes aegypti	SPECIES	-1.08000	-58.44000	1264876348
6366	Diptera	Culicidae	Aedes	Aedes aegypti	SPECIES	-1.06000	-46.92000	1264876374
6367	Diptera	Culicidae	Aedes	Aedes aegypti	SPECIES	-1.02000	-54.36000	1264876342
6368	Diptera	Culicidae	Aedes	Aedes aegypti	SPECIES	-1.02000	-80.39000	4077340592
6369	Diptera	Culicidae	Aedes	Aedes aegypti	SPECIES	-1.00008	-80.68788	2963933329
6370	Diptera	Culicidae	Aedes	Aedes aegypti	SPECIES	-0.99000	-47.86000	1264876326
6371	Diptera	Culicidae	Aedes	Aedes aegypti	SPECIES	-0.95410	-80.70711	4120926547
6372	Diptera	Culicidae	Aedes	Aedes aegypti	SPECIES	-0.95410	-80.70706	3330697010
6373	Diptera	Culicidae	Aedes	Aedes aegypti	SPECIES	-0.95160	122.78700	3443558460
6374	Diptera	Culicidae	Aedes	Aedes aegypti	SPECIES	-0.95000	122.79000	3818537210
6375	Diptera	Culicidae	Aedes	Aedes aegypti	SPECIES	-0.94000	100.35000	1264871675
6376	Diptera	Culicidae	Aedes	Aedes aegypti	SPECIES	-0.94000	-47.13000	1264876261
6377	Diptera	Culicidae	Aedes	Aedes aegypti	SPECIES	-0.93000	119.89000	1264876289
6378	Diptera	Culicidae	Aedes	Aedes aegypti	SPECIES	-0.92000	119.90000	1264871766
6379	Diptera	Culicidae	Aedes	Aedes aegypti	SPECIES	-0.92000	119.88000	1264871769
6380	Diptera	Culicidae	Aedes	Aedes aegypti	SPECIES	-0.92000	119.91000	1264871953
6381	Diptera	Culicidae	Aedes	Aedes aegypti	SPECIES	-0.92000	-48.14000	1264876316
6382	Diptera	Culicidae	Aedes	Aedes aegypti	SPECIES	-0.90472	-89.60373	2850632527
6383	Diptera	Culicidae	Aedes	Aedes aegypti	SPECIES	-0.90228	-89.60962	3330728534
6384	Diptera	Culicidae	Aedes	Aedes aegypti	SPECIES	-0.90000	100.37000	1264871647
6385	Diptera	Culicidae	Aedes	Aedes aegypti	SPECIES	-0.90000	119.88000	1264871743
6386	Diptera	Culicidae	Aedes	Aedes aegypti	SPECIES	-0.90000	119.85000	1264871753
6387	Diptera	Culicidae	Aedes	Aedes aegypti	SPECIES	-0.90000	-47.32000	1264876339
6388	Diptera	Culicidae	Aedes	Aedes aegypti	SPECIES	-0.89000	131.29000	1264871598
6389	Diptera	Culicidae	Aedes	Aedes aegypti	SPECIES	-0.89000	134.05000	1264871600
6390	Diptera	Culicidae	Aedes	Aedes aegypti	SPECIES	-0.89000	119.86000	1264871773
6391	Diptera	Culicidae	Aedes	Aedes aegypti	SPECIES	-0.87000	-47.96000	1264876293
6392	Diptera	Culicidae	Aedes	Aedes aegypti	SPECIES	-0.86000	-47.01000	1264876320
6393	Diptera	Culicidae	Aedes	Aedes aegypti	SPECIES	-0.85000	119.91000	1264871768
6394	Diptera	Culicidae	Aedes	Aedes aegypti	SPECIES	-0.84000	119.93000	1264871707
6395	Diptera	Culicidae	Aedes	Aedes aegypti	SPECIES	-0.84000	119.91000	1264871800
6396	Diptera	Culicidae	Aedes	Aedes aegypti	SPECIES	-0.83000	12.72000	1264886122

№	Order	Family	Genus	Species	Taxon Rank	Latitude	Longitude	GBIF_ID
6397	Diptera	Culicidae	Aedes	Aedes aegypti	SPECIES	-0.82000	119.79000	1264871789
6398	Diptera	Culicidae	Aedes	Aedes aegypti	SPECIES	-0.82000	-48.03000	1264876341
6399	Diptera	Culicidae	Aedes	Aedes aegypti	SPECIES	-0.82000	-47.64000	1264876370
6400	Diptera	Culicidae	Aedes	Aedes aegypti	SPECIES	-0.81000	-48.67000	1264876356
6401	Diptera	Culicidae	Aedes	Aedes aegypti	SPECIES	-0.81000	-47.49000	1264876379
6402	Diptera	Culicidae	Aedes	Aedes aegypti	SPECIES	-0.81000	-80.26000	4077340612
6403	Diptera	Culicidae	Aedes	Aedes aegypti	SPECIES	-0.80000	-47.76000	1264876403
6404	Diptera	Culicidae	Aedes	Aedes aegypti	SPECIES	-0.79000	119.90000	1264871810
6405	Diptera	Culicidae	Aedes	Aedes aegypti	SPECIES	-0.79000	-50.00000	1264876362
6406	Diptera	Culicidae	Aedes	Aedes aegypti	SPECIES	-0.77000	-47.22000	1264876387
6407	Diptera	Culicidae	Aedes	Aedes aegypti	SPECIES	-0.75000	-47.87000	1264876360
6408	Diptera	Culicidae	Aedes	Aedes aegypti	SPECIES	-0.73916	-90.32294	2963912339
6409	Diptera	Culicidae	Aedes	Aedes aegypti	SPECIES	-0.71000	119.91000	1264871716
6410	Diptera	Culicidae	Aedes	Aedes aegypti	SPECIES	-0.71000	-47.34000	1264876331
6411	Diptera	Culicidae	Aedes	Aedes aegypti	SPECIES	-0.67000	37.35000	1264889505
6412	Diptera	Culicidae	Aedes	Aedes aegypti	SPECIES	-0.63936	-90.33719	3330460533
6413	Diptera	Culicidae	Aedes	Aedes aegypti	SPECIES	-0.61000	30.65000	1264890214
6414	Diptera	Culicidae	Aedes	Aedes aegypti	SPECIES	-0.55000	166.91670	177195198
6415	Diptera	Culicidae	Aedes	Aedes aegypti	SPECIES	-0.55000	166.91670	1633667451
6416	Diptera	Culicidae	Aedes	Aedes aegypti	SPECIES	-0.54000	-48.62000	1264876278
6417	Diptera	Culicidae	Aedes	Aedes aegypti	SPECIES	-0.53330	166.91670	177195197
6418	Diptera	Culicidae	Aedes	Aedes aegypti	SPECIES	-0.53330	166.91670	1633663715
6419	Diptera	Culicidae	Aedes	Aedes aegypti	SPECIES	-0.53000	166.91000	3818467959
6420	Diptera	Culicidae	Aedes	Aedes aegypti	SPECIES	-0.52000	166.93000	1264890066
6421	Diptera	Culicidae	Aedes	Aedes aegypti	SPECIES	-0.52000	166.92000	1264890084
6422	Diptera	Culicidae	Aedes	Aedes aegypti	SPECIES	-0.50000	13.10000	1264886040
6423	Diptera	Culicidae	Aedes	Aedes aegypti	SPECIES	-0.50000	30.50000	1264889596
6424	Diptera	Culicidae	Aedes	Aedes aegypti	SPECIES	-0.47294	34.06900	2249985062
6425	Diptera	Culicidae	Aedes	Aedes aegypti	SPECIES	-0.47000	34.07000	3348124300
6426	Diptera	Culicidae	Aedes	Aedes aegypti	SPECIES	-0.47000	34.09000	3350889563
6427	Diptera	Culicidae	Aedes	Aedes aegypti	SPECIES	-0.46000	33.96000	3351021141
6428	Diptera	Culicidae	Aedes	Aedes aegypti	SPECIES	-0.45000	34.06000	3351031082
6429	Diptera	Culicidae	Aedes	Aedes aegypti	SPECIES	-0.43000	34.20000	3350878426
6430	Diptera	Culicidae	Aedes	Aedes aegypti	SPECIES	-0.40000	31.15000	1264889566
6431	Diptera	Culicidae	Aedes	Aedes aegypti	SPECIES	-0.34000	31.73000	1264889545
6432	Diptera	Culicidae	Aedes	Aedes aegypti	SPECIES	-0.33000	31.73000	1264890188
6433	Diptera	Culicidae	Aedes	Aedes aegypti	SPECIES	-0.25000	-65.81000	1264876302
6434	Diptera	Culicidae	Aedes	Aedes aegypti	SPECIES	-0.22000	-60.95000	1264876328
6435	Diptera	Culicidae	Aedes	Aedes aegypti	SPECIES	-0.18000	29.90000	1264887227
6436	Diptera	Culicidae	Aedes	Aedes aegypti	SPECIES	-0.16000	-74.78000	1264888198
6437	Diptera	Culicidae	Aedes	Aedes aegypti	SPECIES	-0.16000	-50.38000	1264891432

№	Order	Family	Genus	Species	Taxon Rank	Latitude	Longitude	GBIF_ID
6438	Diptera	Culicidae	Aedes	Aedes aegypti	SPECIES	-0.15667	102.51606	3993825877
6439	Diptera	Culicidae	Aedes	Aedes aegypti	SPECIES	-0.14000	-63.38000	1264876353
6440	Diptera	Culicidae	Aedes	Aedes aegypti	SPECIES	-0.10000	34.75000	1264890465
6441	Diptera	Culicidae	Aedes	Aedes aegypti	SPECIES	-0.09000	34.73000	1264887092
6442	Diptera	Culicidae	Aedes	Aedes aegypti	SPECIES	-0.05390	34.77180	2633766736
6443	Diptera	Culicidae	Aedes	Aedes aegypti	SPECIES	-0.05000	34.77000	3969038782
6444	Diptera	Culicidae	Aedes	Aedes aegypti	SPECIES	-0.04000	-51.98000	1264876292
6445	Diptera	Culicidae	Aedes	Aedes aegypti	SPECIES	0.00000	0.00000	1434744700
6446	Diptera	Culicidae	Aedes	Aedes aegypti	SPECIES	0.07000	-55.67000	1264876318
6447	Diptera	Culicidae	Aedes	Aedes aegypti	SPECIES	0.07000	32.47000	1264888111
6448	Diptera	Culicidae	Aedes	Aedes aegypti	SPECIES	0.14000	117.48000	1264871820
6449	Diptera	Culicidae	Aedes	Aedes aegypti	SPECIES	0.15000	32.58000	1264890221
6450	Diptera	Culicidae	Aedes	Aedes aegypti	SPECIES	0.16000	117.46000	1264871816
6451	Diptera	Culicidae	Aedes	Aedes aegypti	SPECIES	0.17000	32.48000	1264889853
6452	Diptera	Culicidae	Aedes	Aedes aegypti	SPECIES	0.18000	-51.34000	1264876344
6453	Diptera	Culicidae	Aedes	Aedes aegypti	SPECIES	0.18000	32.50000	1264888813
6454	Diptera	Culicidae	Aedes	Aedes aegypti	SPECIES	0.27000	-57.24000	1264876369
6455	Diptera	Culicidae	Aedes	Aedes aegypti	SPECIES	0.27000	-76.85000	1264886691
6456	Diptera	Culicidae	Aedes	Aedes aegypti	SPECIES	0.28000	34.75000	1264886664
6457	Diptera	Culicidae	Aedes	Aedes aegypti	SPECIES	0.29000	9.51000	1264889101
6458	Diptera	Culicidae	Aedes	Aedes aegypti	SPECIES	0.30000	34.20000	1264887094
6459	Diptera	Culicidae	Aedes	Aedes aegypti	SPECIES	0.31000	-62.11000	1264876392
6460	Diptera	Culicidae	Aedes	Aedes aegypti	SPECIES	0.31000	9.51000	1264886147
6461	Diptera	Culicidae	Aedes	Aedes aegypti	SPECIES	0.31632	32.58373	1453279546
6462	Diptera	Culicidae	Aedes	Aedes aegypti	SPECIES	0.32000	32.58000	1264889588
6463	Diptera	Culicidae	Aedes	Aedes aegypti	SPECIES	0.32522	127.39277	3901425903
6464	Diptera	Culicidae	Aedes	Aedes aegypti	SPECIES	0.33452	6.73005	4138466924
6465	Diptera	Culicidae	Aedes	Aedes aegypti	SPECIES	0.36000	-67.97000	1264876281
6466	Diptera	Culicidae	Aedes	Aedes aegypti	SPECIES	0.37000	9.46000	1264886101
6467	Diptera	Culicidae	Aedes	Aedes aegypti	SPECIES	0.37000	34.78000	1264887063
6468	Diptera	Culicidae	Aedes	Aedes aegypti	SPECIES	0.38000	9.46000	1264886126
6469	Diptera	Culicidae	Aedes	Aedes aegypti	SPECIES	0.39000	9.48000	1264886151
6470	Diptera	Culicidae	Aedes	Aedes aegypti	SPECIES	0.39000	9.45000	1264890617
6471	Diptera	Culicidae	Aedes	Aedes aegypti	SPECIES	0.40000	9.48000	1264886089
6472	Diptera	Culicidae	Aedes	Aedes aegypti	SPECIES	0.40000	-76.78000	1264886706
6473	Diptera	Culicidae	Aedes	Aedes aegypti	SPECIES	0.41000	9.44000	1264886099
6474	Diptera	Culicidae	Aedes	Aedes aegypti	SPECIES	0.41000	9.52000	1264886115
6475	Diptera	Culicidae	Aedes	Aedes aegypti	SPECIES	0.41000	9.46000	1264886138
6476	Diptera	Culicidae	Aedes	Aedes aegypti	SPECIES	0.43000	9.47000	1264886124
6477	Diptera	Culicidae	Aedes	Aedes aegypti	SPECIES	0.43000	9.49000	1264886146
6478	Diptera	Culicidae	Aedes	Aedes aegypti	SPECIES	0.44000	9.44000	1264886166

№	Order	Family	Genus	Species	Taxon Rank	Latitude	Longitude	GBIF_ID
6479	Diptera	Culicidae	Aedes	Aedes aegypti	SPECIES	0.44000	9.43000	1264886176
6480	Diptera	Culicidae	Aedes	Aedes aegypti	SPECIES	0.44000	9.50000	1264886193
6481	Diptera	Culicidae	Aedes	Aedes aegypti	SPECIES	0.47000	9.41000	1264886113
6482	Diptera	Culicidae	Aedes	Aedes aegypti	SPECIES	0.49000	-50.69000	1264876309
6483	Diptera	Culicidae	Aedes	Aedes aegypti	SPECIES	0.49000	9.40000	1264886140
6484	Diptera	Culicidae	Aedes	Aedes aegypti	SPECIES	0.56000	-50.74000	1264890841
6485	Diptera	Culicidae	Aedes	Aedes aegypti	SPECIES	0.57000	30.37000	1264887216
6486	Diptera	Culicidae	Aedes	Aedes aegypti	SPECIES	0.59000	-50.66000	1264876330
6487	Diptera	Culicidae	Aedes	Aedes aegypti	SPECIES	0.61000	-51.70000	1264876358
6488	Diptera	Culicidae	Aedes	Aedes aegypti	SPECIES	0.64000	35.61000	1264886669
6489	Diptera	Culicidae	Aedes	Aedes aegypti	SPECIES	0.66000	123.95000	1264891063
6490	Diptera	Culicidae	Aedes	Aedes aegypti	SPECIES	0.67000	-76.87000	1264886697
6491	Diptera	Culicidae	Aedes	Aedes aegypti	SPECIES	0.68000	-76.58000	1264886715
6492	Diptera	Culicidae	Aedes	Aedes aegypti	SPECIES	0.69000	123.07000	1264876382
6493	Diptera	Culicidae	Aedes	Aedes aegypti	SPECIES	0.71000	30.05000	1264889586
6494	Diptera	Culicidae	Aedes	Aedes aegypti	SPECIES	0.77000	-59.79000	1264876299
6495	Diptera	Culicidae	Aedes	Aedes aegypti	SPECIES	0.78000	-52.94000	1264876321
6496	Diptera	Culicidae	Aedes	Aedes aegypti	SPECIES	0.80000	-79.07000	4077340796
6497	Diptera	Culicidae	Aedes	Aedes aegypti	SPECIES	0.82000	30.17000	1264887238
6498	Diptera	Culicidae	Aedes	Aedes aegypti	SPECIES	0.85000	-60.19000	1264876347
6499	Diptera	Culicidae	Aedes	Aedes aegypti	SPECIES	0.87683	120.75769	3993826504
6500	Diptera	Culicidae	Aedes	Aedes aegypti	SPECIES	0.88000	-51.45000	1264876371
6501	Diptera	Culicidae	Aedes	Aedes aegypti	SPECIES	0.95000	-59.35000	1264876372
6502	Diptera	Culicidae	Aedes	Aedes aegypti	SPECIES	0.99000	9.59000	1264886116
6503	Diptera	Culicidae	Aedes	Aedes aegypti	SPECIES	1.07000	-50.33000	1264876399
6504	Diptera	Culicidae	Aedes	Aedes aegypti	SPECIES	1.09000	34.89000	1264887068
6505	Diptera	Culicidae	Aedes	Aedes aegypti	SPECIES	1.09000	-78.99000	4077340798
6506	Diptera	Culicidae	Aedes	Aedes aegypti	SPECIES	1.11233	-76.32485	3886057845
6507	Diptera	Culicidae	Aedes	Aedes aegypti	SPECIES	1.11234	-76.32487	4075356356
6508	Diptera	Culicidae	Aedes	Aedes aegypti	SPECIES	1.11248	-76.32377	3886057657
6509	Diptera	Culicidae	Aedes	Aedes aegypti	SPECIES	1.11256	-76.32380	4075356408
6510	Diptera	Culicidae	Aedes	Aedes aegypti	SPECIES	1.11272	-76.32377	3459452331
6511	Diptera	Culicidae	Aedes	Aedes aegypti	SPECIES	1.11300	-76.32386	4122376448
6512	Diptera	Culicidae	Aedes	Aedes aegypti	SPECIES	1.11343	-76.32398	3459452333
6513	Diptera	Culicidae	Aedes	Aedes aegypti	SPECIES	1.11356	-76.32456	4122376307
6514	Diptera	Culicidae	Aedes	Aedes aegypti	SPECIES	1.11361	-76.32411	3886057654
6515	Diptera	Culicidae	Aedes	Aedes aegypti	SPECIES	1.11378	-76.32402	4122376303
6516	Diptera	Culicidae	Aedes	Aedes aegypti	SPECIES	1.11393	-76.32406	3459452313
6517	Diptera	Culicidae	Aedes	Aedes aegypti	SPECIES	1.11397	-76.32406	3886057839
6518	Diptera	Culicidae	Aedes	Aedes aegypti	SPECIES	1.11445	-76.32569	4122376449
6519	Diptera	Culicidae	Aedes	Aedes aegypti	SPECIES	1.11456	-76.32556	3459452309

№	Order	Family	Genus	Species	Taxon Rank	Latitude	Longitude	GBIF_ID
6520	Diptera	Culicidae	Aedes	Aedes aegypti	SPECIES	1.11461	-76.32553	4075356428
6521	Diptera	Culicidae	Aedes	Aedes aegypti	SPECIES	1.11508	-76.32574	4075356336
6522	Diptera	Culicidae	Aedes	Aedes aegypti	SPECIES	1.11512	-76.32446	4075356403
6523	Diptera	Culicidae	Aedes	Aedes aegypti	SPECIES	1.11521	-76.32420	3459452317
6524	Diptera	Culicidae	Aedes	Aedes aegypti	SPECIES	1.11532	-76.32564	3886057326
6525	Diptera	Culicidae	Aedes	Aedes aegypti	SPECIES	1.11534	-76.32574	4122376306
6526	Diptera	Culicidae	Aedes	Aedes aegypti	SPECIES	1.11535	-76.32565	4122376378
6527	Diptera	Culicidae	Aedes	Aedes aegypti	SPECIES	1.11541	-76.32457	3459452303
6528	Diptera	Culicidae	Aedes	Aedes aegypti	SPECIES	1.11553	-76.32417	3886057844
6529	Diptera	Culicidae	Aedes	Aedes aegypti	SPECIES	1.11561	-76.32407	4122376305
6530	Diptera	Culicidae	Aedes	Aedes aegypti	SPECIES	1.11663	-76.32512	3459452301
6531	Diptera	Culicidae	Aedes	Aedes aegypti	SPECIES	1.11664	-76.32451	4122376304
6532	Diptera	Culicidae	Aedes	Aedes aegypti	SPECIES	1.11676	-76.32446	3886057846
6533	Diptera	Culicidae	Aedes	Aedes aegypti	SPECIES	1.11710	-76.32544	3459452319
6534	Diptera	Culicidae	Aedes	Aedes aegypti	SPECIES	1.11794	-76.32915	4122376353
6535	Diptera	Culicidae	Aedes	Aedes aegypti	SPECIES	1.11813	-76.32866	3886057362
6536	Diptera	Culicidae	Aedes	Aedes aegypti	SPECIES	1.11815	-76.32889	4122376372
6537	Diptera	Culicidae	Aedes	Aedes aegypti	SPECIES	1.11825	-76.32964	3886057392
6538	Diptera	Culicidae	Aedes	Aedes aegypti	SPECIES	1.11826	-76.32891	3886057376
6539	Diptera	Culicidae	Aedes	Aedes aegypti	SPECIES	1.11835	-76.32996	4075356429
6540	Diptera	Culicidae	Aedes	Aedes aegypti	SPECIES	1.11840	-76.32811	4122376380
6541	Diptera	Culicidae	Aedes	Aedes aegypti	SPECIES	1.11842	-76.33242	4122376453
6542	Diptera	Culicidae	Aedes	Aedes aegypti	SPECIES	1.11844	-76.32980	4075356380
6543	Diptera	Culicidae	Aedes	Aedes aegypti	SPECIES	1.11845	-76.32993	4122376349
6544	Diptera	Culicidae	Aedes	Aedes aegypti	SPECIES	1.11846	-76.32931	4122376351
6545	Diptera	Culicidae	Aedes	Aedes aegypti	SPECIES	1.11853	-76.32926	3886057377
6546	Diptera	Culicidae	Aedes	Aedes aegypti	SPECIES	1.11857	-76.32970	3459452345
6547	Diptera	Culicidae	Aedes	Aedes aegypti	SPECIES	1.11871	-76.32920	3459452347
6548	Diptera	Culicidae	Aedes	Aedes aegypti	SPECIES	1.11871	-76.32957	3886057398
6549	Diptera	Culicidae	Aedes	Aedes aegypti	SPECIES	1.11874	-76.32919	4075356405
6550	Diptera	Culicidae	Aedes	Aedes aegypti	SPECIES	1.11876	-76.32786	4075356317
6551	Diptera	Culicidae	Aedes	Aedes aegypti	SPECIES	1.11892	-76.32830	3459452682
6552	Diptera	Culicidae	Aedes	Aedes aegypti	SPECIES	1.11905	-76.32975	3459452346
6553	Diptera	Culicidae	Aedes	Aedes aegypti	SPECIES	1.11910	-76.32839	3886057361
6554	Diptera	Culicidae	Aedes	Aedes aegypti	SPECIES	1.11910	-76.32922	3886057364
6555	Diptera	Culicidae	Aedes	Aedes aegypti	SPECIES	1.11911	-76.32975	4122376382
6556	Diptera	Culicidae	Aedes	Aedes aegypti	SPECIES	1.11913	-76.32907	3886057408
6557	Diptera	Culicidae	Aedes	Aedes aegypti	SPECIES	1.11913	-76.32898	4075356407
6558	Diptera	Culicidae	Aedes	Aedes aegypti	SPECIES	1.11916	-76.33139	3459452353
6559	Diptera	Culicidae	Aedes	Aedes aegypti	SPECIES	1.11916	-76.32895	4122376355
6560	Diptera	Culicidae	Aedes	Aedes aegypti	SPECIES	1.11916	-76.32836	4122376368

№	Order	Family	Genus	Species	Taxon Rank	Latitude	Longitude	GBIF_ID
6561	Diptera	Culicidae	Aedes	Aedes aegypti	SPECIES	1.11917	-76.32826	4075356310
6562	Diptera	Culicidae	Aedes	Aedes aegypti	SPECIES	1.11918	-76.33126	3886057369
6563	Diptera	Culicidae	Aedes	Aedes aegypti	SPECIES	1.11920	-76.32892	3886057406
6564	Diptera	Culicidae	Aedes	Aedes aegypti	SPECIES	1.11921	-76.32804	3886057837
6565	Diptera	Culicidae	Aedes	Aedes aegypti	SPECIES	1.11925	-76.32808	4075356412
6566	Diptera	Culicidae	Aedes	Aedes aegypti	SPECIES	1.11927	-76.32792	4075356386
6567	Diptera	Culicidae	Aedes	Aedes aegypti	SPECIES	1.11931	-76.32809	4075356377
6568	Diptera	Culicidae	Aedes	Aedes aegypti	SPECIES	1.11931	-76.32833	3886057840
6569	Diptera	Culicidae	Aedes	Aedes aegypti	SPECIES	1.11940	-76.32807	3886057360
6570	Diptera	Culicidae	Aedes	Aedes aegypti	SPECIES	1.11940	-76.32865	3886057790
6571	Diptera	Culicidae	Aedes	Aedes aegypti	SPECIES	1.11943	-76.32851	4075356381
6572	Diptera	Culicidae	Aedes	Aedes aegypti	SPECIES	1.11951	-76.32604	4122376394
6573	Diptera	Culicidae	Aedes	Aedes aegypti	SPECIES	1.11952	-76.32801	3886057452
6574	Diptera	Culicidae	Aedes	Aedes aegypti	SPECIES	1.11954	-76.33224	4122376363
6575	Diptera	Culicidae	Aedes	Aedes aegypti	SPECIES	1.11955	-76.33222	4122376364
6576	Diptera	Culicidae	Aedes	Aedes aegypti	SPECIES	1.11956	-76.33152	3459452358
6577	Diptera	Culicidae	Aedes	Aedes aegypti	SPECIES	1.11958	-76.33301	4122376451
6578	Diptera	Culicidae	Aedes	Aedes aegypti	SPECIES	1.11959	-76.33232	3459452354
6579	Diptera	Culicidae	Aedes	Aedes aegypti	SPECIES	1.11960	-76.33160	3459452359
6580	Diptera	Culicidae	Aedes	Aedes aegypti	SPECIES	1.11961	-76.33089	3459452311
6581	Diptera	Culicidae	Aedes	Aedes aegypti	SPECIES	1.11961	-76.32638	4075356404
6582	Diptera	Culicidae	Aedes	Aedes aegypti	SPECIES	1.11962	-76.33240	3459452355
6583	Diptera	Culicidae	Aedes	Aedes aegypti	SPECIES	1.11964	-76.33111	3459452357
6584	Diptera	Culicidae	Aedes	Aedes aegypti	SPECIES	1.11967	-76.32784	4122376369
6585	Diptera	Culicidae	Aedes	Aedes aegypti	SPECIES	1.11968	-76.32849	4122376370
6586	Diptera	Culicidae	Aedes	Aedes aegypti	SPECIES	1.11969	-76.32646	4122376393
6587	Diptera	Culicidae	Aedes	Aedes aegypti	SPECIES	1.11969	-76.32686	3886057381
6588	Diptera	Culicidae	Aedes	Aedes aegypti	SPECIES	1.11972	-76.32634	4122376376
6589	Diptera	Culicidae	Aedes	Aedes aegypti	SPECIES	1.11983	-76.32840	4075356384
6590	Diptera	Culicidae	Aedes	Aedes aegypti	SPECIES	1.11984	-76.32592	4075356375
6591	Diptera	Culicidae	Aedes	Aedes aegypti	SPECIES	1.11984	-76.32766	4122376367
6592	Diptera	Culicidae	Aedes	Aedes aegypti	SPECIES	1.11985	-76.32830	4075356411
6593	Diptera	Culicidae	Aedes	Aedes aegypti	SPECIES	1.11985	-76.32791	3886057357
6594	Diptera	Culicidae	Aedes	Aedes aegypti	SPECIES	1.11989	-76.32653	4075356409
6595	Diptera	Culicidae	Aedes	Aedes aegypti	SPECIES	1.11991	-76.33142	3459452360
6596	Diptera	Culicidae	Aedes	Aedes aegypti	SPECIES	1.11992	-76.32705	3459452341
6597	Diptera	Culicidae	Aedes	Aedes aegypti	SPECIES	1.11993	-76.33120	4122376357
6598	Diptera	Culicidae	Aedes	Aedes aegypti	SPECIES	1.11994	-76.32826	3886057402
6599	Diptera	Culicidae	Aedes	Aedes aegypti	SPECIES	1.11998	-76.32665	3459452342
6600	Diptera	Culicidae	Aedes	Aedes aegypti	SPECIES	1.12012	-76.32789	3886057356
6601	Diptera	Culicidae	Aedes	Aedes aegypti	SPECIES	1.12013	-76.32784	4122376366

№	Order	Family	Genus	Species	Taxon Rank	Latitude	Longitude	GBIF_ID
6602	Diptera	Culicidae	Aedes	Aedes aegypti	SPECIES	1.12020	-76.32767	4122376365
6603	Diptera	Culicidae	Aedes	Aedes aegypti	SPECIES	1.12027	-76.32845	4122376302
6604	Diptera	Culicidae	Aedes	Aedes aegypti	SPECIES	1.12039	-76.32743	3886057365
6605	Diptera	Culicidae	Aedes	Aedes aegypti	SPECIES	1.12054	-76.32679	4122376392
6606	Diptera	Culicidae	Aedes	Aedes aegypti	SPECIES	1.12062	-76.32716	4122376361
6607	Diptera	Culicidae	Aedes	Aedes aegypti	SPECIES	1.12062	-76.32629	3886057383
6608	Diptera	Culicidae	Aedes	Aedes aegypti	SPECIES	1.12067	-76.32629	4122376375
6609	Diptera	Culicidae	Aedes	Aedes aegypti	SPECIES	1.12070	-76.32858	4075356316
6610	Diptera	Culicidae	Aedes	Aedes aegypti	SPECIES	1.12071	-76.32651	4075356319
6611	Diptera	Culicidae	Aedes	Aedes aegypti	SPECIES	1.12081	-76.32665	4122376347
6612	Diptera	Culicidae	Aedes	Aedes aegypti	SPECIES	1.12093	-76.32848	4075356431
6613	Diptera	Culicidae	Aedes	Aedes aegypti	SPECIES	1.12113	-76.32802	4075356325
6614	Diptera	Culicidae	Aedes	Aedes aegypti	SPECIES	1.12118	-76.32703	4122376359
6615	Diptera	Culicidae	Aedes	Aedes aegypti	SPECIES	1.12127	-76.32611	3459452343
6616	Diptera	Culicidae	Aedes	Aedes aegypti	SPECIES	1.15974	103.89949	3901430915
6617	Diptera	Culicidae	Aedes	Aedes aegypti	SPECIES	1.16000	127.84000	1264889404
6618	Diptera	Culicidae	Aedes	Aedes aegypti	SPECIES	1.18000	124.51000	1264871907
6619	Diptera	Culicidae	Aedes	Aedes aegypti	SPECIES	1.18000	127.90000	1264889430
6620	Diptera	Culicidae	Aedes	Aedes aegypti	SPECIES	1.23000	-52.41000	1264876366
6621	Diptera	Culicidae	Aedes	Aedes aegypti	SPECIES	1.25000	124.83000	1264891087
6622	Diptera	Culicidae	Aedes	Aedes aegypti	SPECIES	1.27000	103.81000	1264886990
6623	Diptera	Culicidae	Aedes	Aedes aegypti	SPECIES	1.28000	103.84000	1264888250
6624	Diptera	Culicidae	Aedes	Aedes aegypti	SPECIES	1.28000	103.83000	1264888284
6625	Diptera	Culicidae	Aedes	Aedes aegypti	SPECIES	1.28000	103.85000	1264888293
6626	Diptera	Culicidae	Aedes	Aedes aegypti	SPECIES	1.28000	103.82000	1264888868
6627	Diptera	Culicidae	Aedes	Aedes aegypti	SPECIES	1.29000	103.82000	1264888282
6628	Diptera	Culicidae	Aedes	Aedes aegypti	SPECIES	1.29000	103.85000	1264891272
6629	Diptera	Culicidae	Aedes	Aedes aegypti	SPECIES	1.29480	103.88067	4054807405
6630	Diptera	Culicidae	Aedes	Aedes aegypti	SPECIES	1.30000	103.76000	1264886966
6631	Diptera	Culicidae	Aedes	Aedes aegypti	SPECIES	1.30000	103.86000	1264888271
6632	Diptera	Culicidae	Aedes	Aedes aegypti	SPECIES	1.30000	103.85000	1264889151
6633	Diptera	Culicidae	Aedes	Aedes aegypti	SPECIES	1.31000	103.84000	1264886963
6634	Diptera	Culicidae	Aedes	Aedes aegypti	SPECIES	1.31000	103.80000	1264886983
6635	Diptera	Culicidae	Aedes	Aedes aegypti	SPECIES	1.31000	103.85000	1264888856
6636	Diptera	Culicidae	Aedes	Aedes aegypti	SPECIES	1.31000	103.89000	1264888862
6637	Diptera	Culicidae	Aedes	Aedes aegypti	SPECIES	1.32000	103.86000	1264888249
6638	Diptera	Culicidae	Aedes	Aedes aegypti	SPECIES	1.32000	103.88000	1264888288
6639	Diptera	Culicidae	Aedes	Aedes aegypti	SPECIES	1.32000	103.85000	1264888845
6640	Diptera	Culicidae	Aedes	Aedes aegypti	SPECIES	1.32000	103.83000	1264890921
6641	Diptera	Culicidae	Aedes	Aedes aegypti	SPECIES	1.33000	103.93000	1264888251
6642	Diptera	Culicidae	Aedes	Aedes aegypti	SPECIES	1.33000	103.90000	1264888309

№	Order	Family	Genus	Species	Taxon Rank	Latitude	Longitude	GBIF_ID
6643	Diptera	Culicidae	Aedes	Aedes aegypti	SPECIES	1.35000	103.87000	1264886953
6644	Diptera	Culicidae	Aedes	Aedes aegypti	SPECIES	1.35000	103.82000	1264886987
6645	Diptera	Culicidae	Aedes	Aedes aegypti	SPECIES	1.35000	172.93000	1264890105
6646	Diptera	Culicidae	Aedes	Aedes aegypti	SPECIES	1.36000	103.84000	1264886999
6647	Diptera	Culicidae	Aedes	Aedes aegypti	SPECIES	1.36000	173.15000	1264890101
6648	Diptera	Culicidae	Aedes	Aedes aegypti	SPECIES	1.36000	173.13000	1264890106
6649	Diptera	Culicidae	Aedes	Aedes aegypti	SPECIES	1.37000	103.88000	1264886952
6650	Diptera	Culicidae	Aedes	Aedes aegypti	SPECIES	1.37000	103.95000	1264886969
6651	Diptera	Culicidae	Aedes	Aedes aegypti	SPECIES	1.37000	103.94000	1264886976
6652	Diptera	Culicidae	Aedes	Aedes aegypti	SPECIES	1.37000	103.85000	1264887417
6653	Diptera	Culicidae	Aedes	Aedes aegypti	SPECIES	1.38000	103.83000	1264886162
6654	Diptera	Culicidae	Aedes	Aedes aegypti	SPECIES	1.41000	103.76000	1264886136
6655	Diptera	Culicidae	Aedes	Aedes aegypti	SPECIES	1.42000	103.75000	1264886165
6656	Diptera	Culicidae	Aedes	Aedes aegypti	SPECIES	1.43000	103.76000	1264886095
6657	Diptera	Culicidae	Aedes	Aedes aegypti	SPECIES	1.45000	124.82000	1264876535
6658	Diptera	Culicidae	Aedes	Aedes aegypti	SPECIES	1.45000	125.20000	1264889916
6659	Diptera	Culicidae	Aedes	Aedes aegypti	SPECIES	1.46000	103.75000	1264891147
6660	Diptera	Culicidae	Aedes	Aedes aegypti	SPECIES	1.47777	98.95026	2850661925
6661	Diptera	Culicidae	Aedes	Aedes aegypti	SPECIES	1.51000	103.51000	1264891238
6662	Diptera	Culicidae	Aedes	Aedes aegypti	SPECIES	1.53000	124.91000	1264890736
6663	Diptera	Culicidae	Aedes	Aedes aegypti	SPECIES	1.53000	124.86000	1264891114
6664	Diptera	Culicidae	Aedes	Aedes aegypti	SPECIES	1.55000	125.00000	1264876492
6665	Diptera	Culicidae	Aedes	Aedes aegypti	SPECIES	1.55000	124.93000	1264876522
6666	Diptera	Culicidae	Aedes	Aedes aegypti	SPECIES	1.55000	110.34000	1264889191
6667	Diptera	Culicidae	Aedes	Aedes aegypti	SPECIES	1.56000	124.92000	1264876521
6668	Diptera	Culicidae	Aedes	Aedes aegypti	SPECIES	1.56000	124.93000	1264876532
6669	Diptera	Culicidae	Aedes	Aedes aegypti	SPECIES	1.58000	124.91000	1264876502
6670	Diptera	Culicidae	Aedes	Aedes aegypti	SPECIES	1.60000	11.58000	1264886144
6671	Diptera	Culicidae	Aedes	Aedes aegypti	SPECIES	1.61000	-75.61000	1264887407
6672	Diptera	Culicidae	Aedes	Aedes aegypti	SPECIES	1.62000	-75.60000	1264887349
6673	Diptera	Culicidae	Aedes	Aedes aegypti	SPECIES	1.64000	-52.34000	1264876352
6674	Diptera	Culicidae	Aedes	Aedes aegypti	SPECIES	1.65000	44.67000	1264889492
6675	Diptera	Culicidae	Aedes	Aedes aegypti	SPECIES	1.68000	-50.25000	1264876346
6676	Diptera	Culicidae	Aedes	Aedes aegypti	SPECIES	1.75000	104.07000	1264886128
6677	Diptera	Culicidae	Aedes	Aedes aegypti	SPECIES	1.84000	-61.85000	1264891361
6678	Diptera	Culicidae	Aedes	Aedes aegypti	SPECIES	1.87991	-76.27021	3112306447
6679	Diptera	Culicidae	Aedes	Aedes aegypti	SPECIES	2.02000	-75.76000	1264887221
6680	Diptera	Culicidae	Aedes	Aedes aegypti	SPECIES	2.05000	103.39000	1264891020
6681	Diptera	Culicidae	Aedes	Aedes aegypti	SPECIES	2.05000	103.51000	1264891366
6682	Diptera	Culicidae	Aedes	Aedes aegypti	SPECIES	2.07181	99.82960	3067906011
6683	Diptera	Culicidae	Aedes	Aedes aegypti	SPECIES	2.09021	96.64980	3993825661

№	Order	Family	Genus	Species	Taxon Rank	Latitude	Longitude	GBIF_ID
6684	Diptera	Culicidae	Aedes	Aedes aegypti	SPECIES	2.10796	-76.98844	3886057334
6685	Diptera	Culicidae	Aedes	Aedes aegypti	SPECIES	2.10819	-76.98359	3886057315
6686	Diptera	Culicidae	Aedes	Aedes aegypti	SPECIES	2.10830	-76.98369	4122376440
6687	Diptera	Culicidae	Aedes	Aedes aegypti	SPECIES	2.10848	-76.98383	4122376422
6688	Diptera	Culicidae	Aedes	Aedes aegypti	SPECIES	2.10850	-76.98254	3886057422
6689	Diptera	Culicidae	Aedes	Aedes aegypti	SPECIES	2.10854	-76.98237	4122376411
6690	Diptera	Culicidae	Aedes	Aedes aegypti	SPECIES	2.10855	-76.98229	4122376441
6691	Diptera	Culicidae	Aedes	Aedes aegypti	SPECIES	2.10864	-76.98260	4122376413
6692	Diptera	Culicidae	Aedes	Aedes aegypti	SPECIES	2.10865	-76.98307	3886057596
6693	Diptera	Culicidae	Aedes	Aedes aegypti	SPECIES	2.10871	-76.98353	3302601340
6694	Diptera	Culicidae	Aedes	Aedes aegypti	SPECIES	2.10876	-76.98261	3302601375
6695	Diptera	Culicidae	Aedes	Aedes aegypti	SPECIES	2.10879	-76.98347	3302601338
6696	Diptera	Culicidae	Aedes	Aedes aegypti	SPECIES	2.10880	-76.98521	4122376405
6697	Diptera	Culicidae	Aedes	Aedes aegypti	SPECIES	2.10880	-76.98586	3886057591
6698	Diptera	Culicidae	Aedes	Aedes aegypti	SPECIES	2.10883	-76.98533	4122376430
6699	Diptera	Culicidae	Aedes	Aedes aegypti	SPECIES	2.10890	-76.98346	3302601341
6700	Diptera	Culicidae	Aedes	Aedes aegypti	SPECIES	2.10893	-76.98175	3886057598
6701	Diptera	Culicidae	Aedes	Aedes aegypti	SPECIES	2.10894	-76.98160	4122376409
6702	Diptera	Culicidae	Aedes	Aedes aegypti	SPECIES	2.10896	-76.98055	3459452678
6703	Diptera	Culicidae	Aedes	Aedes aegypti	SPECIES	2.10896	-76.98340	3302601339
6704	Diptera	Culicidae	Aedes	Aedes aegypti	SPECIES	2.10896	-76.98067	3886057473
6705	Diptera	Culicidae	Aedes	Aedes aegypti	SPECIES	2.10897	-76.98193	3302601381
6706	Diptera	Culicidae	Aedes	Aedes aegypti	SPECIES	2.10897	-76.98098	4122376415
6707	Diptera	Culicidae	Aedes	Aedes aegypti	SPECIES	2.10897	-76.98220	3886057584
6708	Diptera	Culicidae	Aedes	Aedes aegypti	SPECIES	2.10898	-76.98096	4122376321
6709	Diptera	Culicidae	Aedes	Aedes aegypti	SPECIES	2.10901	-76.98554	4122376432
6710	Diptera	Culicidae	Aedes	Aedes aegypti	SPECIES	2.10901	-76.98489	3886057324
6711	Diptera	Culicidae	Aedes	Aedes aegypti	SPECIES	2.10902	-76.98085	3886057526
6712	Diptera	Culicidae	Aedes	Aedes aegypti	SPECIES	2.10902	-76.98077	3886057569
6713	Diptera	Culicidae	Aedes	Aedes aegypti	SPECIES	2.10905	-76.98155	3302601380
6714	Diptera	Culicidae	Aedes	Aedes aegypti	SPECIES	2.10906	-76.98507	3886057597
6715	Diptera	Culicidae	Aedes	Aedes aegypti	SPECIES	2.10919	-76.98333	3886057313
6716	Diptera	Culicidae	Aedes	Aedes aegypti	SPECIES	2.10921	-76.98523	4122376434
6717	Diptera	Culicidae	Aedes	Aedes aegypti	SPECIES	2.10926	-76.98313	3886057620
6718	Diptera	Culicidae	Aedes	Aedes aegypti	SPECIES	2.10928	-76.98509	3886057599
6719	Diptera	Culicidae	Aedes	Aedes aegypti	SPECIES	2.10933	-76.98554	3886057582
6720	Diptera	Culicidae	Aedes	Aedes aegypti	SPECIES	2.10936	-76.98307	4122376439
6721	Diptera	Culicidae	Aedes	Aedes aegypti	SPECIES	2.10938	-76.98301	3302601337
6722	Diptera	Culicidae	Aedes	Aedes aegypti	SPECIES	2.10942	-76.98178	4122376320
6723	Diptera	Culicidae	Aedes	Aedes aegypti	SPECIES	2.10948	-76.98074	3459452675
6724	Diptera	Culicidae	Aedes	Aedes aegypti	SPECIES	2.10948	-76.98581	3886057568

№	Order	Family	Genus	Species	Taxon Rank	Latitude	Longitude	GBIF_ID
6725	Diptera	Culicidae	Aedes	Aedes aegypti	SPECIES	2.10951	-76.98850	3886057328
6726	Diptera	Culicidae	Aedes	Aedes aegypti	SPECIES	2.10951	-76.98140	3886057607
6727	Diptera	Culicidae	Aedes	Aedes aegypti	SPECIES	2.10960	-76.98290	3302601344
6728	Diptera	Culicidae	Aedes	Aedes aegypti	SPECIES	2.10961	-76.98051	4122376435
6729	Diptera	Culicidae	Aedes	Aedes aegypti	SPECIES	2.10965	-76.98111	4122376318
6730	Diptera	Culicidae	Aedes	Aedes aegypti	SPECIES	2.10966	-76.98095	3886057742
6731	Diptera	Culicidae	Aedes	Aedes aegypti	SPECIES	2.10967	-76.98244	3302601327
6732	Diptera	Culicidae	Aedes	Aedes aegypti	SPECIES	2.10967	-76.98293	3886057800
6733	Diptera	Culicidae	Aedes	Aedes aegypti	SPECIES	2.10973	-76.98285	3302601307
6734	Diptera	Culicidae	Aedes	Aedes aegypti	SPECIES	2.10976	-76.98002	3459452676
6735	Diptera	Culicidae	Aedes	Aedes aegypti	SPECIES	2.10987	-76.98281	3886057749
6736	Diptera	Culicidae	Aedes	Aedes aegypti	SPECIES	2.10991	-76.98250	4122376319
6737	Diptera	Culicidae	Aedes	Aedes aegypti	SPECIES	2.10998	-76.98832	3886057333
6738	Diptera	Culicidae	Aedes	Aedes aegypti	SPECIES	2.11005	-76.98066	3886057700
6739	Diptera	Culicidae	Aedes	Aedes aegypti	SPECIES	2.11020	-76.98020	3886057574
6740	Diptera	Culicidae	Aedes	Aedes aegypti	SPECIES	2.11021	-76.98263	3302601377
6741	Diptera	Culicidae	Aedes	Aedes aegypti	SPECIES	2.11027	-76.98825	3886057575
6742	Diptera	Culicidae	Aedes	Aedes aegypti	SPECIES	2.11027	-76.98280	3302601325
6743	Diptera	Culicidae	Aedes	Aedes aegypti	SPECIES	2.11029	-76.98814	3886057573
6744	Diptera	Culicidae	Aedes	Aedes aegypti	SPECIES	2.11041	-76.98599	3886057572
6745	Diptera	Culicidae	Aedes	Aedes aegypti	SPECIES	2.11043	-76.98273	3886057687
6746	Diptera	Culicidae	Aedes	Aedes aegypti	SPECIES	2.11046	-76.98269	3302601323
6747	Diptera	Culicidae	Aedes	Aedes aegypti	SPECIES	2.11061	-76.98030	4122376436
6748	Diptera	Culicidae	Aedes	Aedes aegypti	SPECIES	2.11078	-76.98258	3302601335
6749	Diptera	Culicidae	Aedes	Aedes aegypti	SPECIES	2.11079	-76.98303	3459452670
6750	Diptera	Culicidae	Aedes	Aedes aegypti	SPECIES	2.11081	-76.97995	3886057566
6751	Diptera	Culicidae	Aedes	Aedes aegypti	SPECIES	2.11089	-76.98305	4122376438
6752	Diptera	Culicidae	Aedes	Aedes aegypti	SPECIES	2.11094	-76.98279	3459452703
6753	Diptera	Culicidae	Aedes	Aedes aegypti	SPECIES	2.11095	-76.98305	3459452669
6754	Diptera	Culicidae	Aedes	Aedes aegypti	SPECIES	2.11103	-76.98274	3886057613
6755	Diptera	Culicidae	Aedes	Aedes aegypti	SPECIES	2.11103	-76.98134	3886057610
6756	Diptera	Culicidae	Aedes	Aedes aegypti	SPECIES	2.11118	-76.98348	4122376437
6757	Diptera	Culicidae	Aedes	Aedes aegypti	SPECIES	2.11119	-76.98111	3886057609
6758	Diptera	Culicidae	Aedes	Aedes aegypti	SPECIES	2.11121	-76.98310	3459452668
6759	Diptera	Culicidae	Aedes	Aedes aegypti	SPECIES	2.11124	-76.98068	3886057570
6760	Diptera	Culicidae	Aedes	Aedes aegypti	SPECIES	2.11131	-76.98111	3302601376
6761	Diptera	Culicidae	Aedes	Aedes aegypti	SPECIES	2.11132	-76.98667	3886057336
6762	Diptera	Culicidae	Aedes	Aedes aegypti	SPECIES	2.11143	-76.98264	3886057668
6763	Diptera	Culicidae	Aedes	Aedes aegypti	SPECIES	2.11144	-76.98245	3886057678
6764	Diptera	Culicidae	Aedes	Aedes aegypti	SPECIES	2.11151	-76.98270	3302601329
6765	Diptera	Culicidae	Aedes	Aedes aegypti	SPECIES	2.11153	-76.98271	3302601342

№	Order	Family	Genus	Species	Taxon Rank	Latitude	Longitude	GBIF_ID
6766	Diptera	Culicidae	Aedes	Aedes aegypti	SPECIES	2.11164	-76.98352	4122376314
6767	Diptera	Culicidae	Aedes	Aedes aegypti	SPECIES	2.11164	-76.98112	3459452660
6768	Diptera	Culicidae	Aedes	Aedes aegypti	SPECIES	2.11171	-76.98359	4122376315
6769	Diptera	Culicidae	Aedes	Aedes aegypti	SPECIES	2.11190	-76.98618	3886057335
6770	Diptera	Culicidae	Aedes	Aedes aegypti	SPECIES	2.11197	-76.98214	3886057772
6771	Diptera	Culicidae	Aedes	Aedes aegypti	SPECIES	2.11198	-76.98259	4122376401
6772	Diptera	Culicidae	Aedes	Aedes aegypti	SPECIES	2.11199	-76.98224	4122376316
6773	Diptera	Culicidae	Aedes	Aedes aegypti	SPECIES	2.11200	-76.98060	3886057503
6774	Diptera	Culicidae	Aedes	Aedes aegypti	SPECIES	2.11203	-76.98378	3886057770
6775	Diptera	Culicidae	Aedes	Aedes aegypti	SPECIES	2.11217	-76.98025	3459452701
6776	Diptera	Culicidae	Aedes	Aedes aegypti	SPECIES	2.11217	-76.98374	3886057330
6777	Diptera	Culicidae	Aedes	Aedes aegypti	SPECIES	2.11221	-76.98363	3886057744
6778	Diptera	Culicidae	Aedes	Aedes aegypti	SPECIES	2.11225	-76.98356	4075356390
6779	Diptera	Culicidae	Aedes	Aedes aegypti	SPECIES	2.11225	-76.98047	4122376419
6780	Diptera	Culicidae	Aedes	Aedes aegypti	SPECIES	2.11229	-76.98072	3886057707
6781	Diptera	Culicidae	Aedes	Aedes aegypti	SPECIES	2.11232	-76.98334	3302601392
6782	Diptera	Culicidae	Aedes	Aedes aegypti	SPECIES	2.11233	-76.98412	3886057559
6783	Diptera	Culicidae	Aedes	Aedes aegypti	SPECIES	2.11236	-76.98224	3886057759
6784	Diptera	Culicidae	Aedes	Aedes aegypti	SPECIES	2.11238	-76.98111	3886057819
6785	Diptera	Culicidae	Aedes	Aedes aegypti	SPECIES	2.11240	-76.98110	3302601398
6786	Diptera	Culicidae	Aedes	Aedes aegypti	SPECIES	2.11243	-76.98343	3302601408
6787	Diptera	Culicidae	Aedes	Aedes aegypti	SPECIES	2.11246	-76.98293	3886057545
6788	Diptera	Culicidae	Aedes	Aedes aegypti	SPECIES	2.11246	-76.98348	3886057726
6789	Diptera	Culicidae	Aedes	Aedes aegypti	SPECIES	2.11251	-76.98278	3886057750
6790	Diptera	Culicidae	Aedes	Aedes aegypti	SPECIES	2.11252	-76.98358	3302601396
6791	Diptera	Culicidae	Aedes	Aedes aegypti	SPECIES	2.11255	-76.98422	3302601420
6792	Diptera	Culicidae	Aedes	Aedes aegypti	SPECIES	2.11255	-76.98081	3302601399
6793	Diptera	Culicidae	Aedes	Aedes aegypti	SPECIES	2.11257	-76.98083	3886057833
6794	Diptera	Culicidae	Aedes	Aedes aegypti	SPECIES	2.11260	-76.98300	3302601312
6795	Diptera	Culicidae	Aedes	Aedes aegypti	SPECIES	2.11264	-76.98096	4122376417
6796	Diptera	Culicidae	Aedes	Aedes aegypti	SPECIES	2.11265	-76.98367	3302601395
6797	Diptera	Culicidae	Aedes	Aedes aegypti	SPECIES	2.11273	-76.98323	4122376402
6798	Diptera	Culicidae	Aedes	Aedes aegypti	SPECIES	2.11274	-76.98205	4122376404
6799	Diptera	Culicidae	Aedes	Aedes aegypti	SPECIES	2.11279	-76.98392	3886057316
6800	Diptera	Culicidae	Aedes	Aedes aegypti	SPECIES	2.11284	-76.98413	3886057391
6801	Diptera	Culicidae	Aedes	Aedes aegypti	SPECIES	2.11285	-76.98394	3886057426
6802	Diptera	Culicidae	Aedes	Aedes aegypti	SPECIES	2.11287	-76.98240	4122376398
6803	Diptera	Culicidae	Aedes	Aedes aegypti	SPECIES	2.11287	-76.98456	3302601419
6804	Diptera	Culicidae	Aedes	Aedes aegypti	SPECIES	2.11290	-76.98342	4122376391
6805	Diptera	Culicidae	Aedes	Aedes aegypti	SPECIES	2.11295	-76.98129	4122376421
6806	Diptera	Culicidae	Aedes	Aedes aegypti	SPECIES	2.11298	-76.98460	3459452679

№	Order	Family	Genus	Species	Taxon Rank	Latitude	Longitude	GBIF_ID
6807	Diptera	Culicidae	Aedes	Aedes aegypti	SPECIES	2.11301	-76.98405	3459452680
6808	Diptera	Culicidae	Aedes	Aedes aegypti	SPECIES	2.11308	-76.98071	3302601310
6809	Diptera	Culicidae	Aedes	Aedes aegypti	SPECIES	2.11308	-76.98512	3886057331
6810	Diptera	Culicidae	Aedes	Aedes aegypti	SPECIES	2.11314	-76.98483	4122376385
6811	Diptera	Culicidae	Aedes	Aedes aegypti	SPECIES	2.11315	-76.98105	3302601311
6812	Diptera	Culicidae	Aedes	Aedes aegypti	SPECIES	2.11324	-76.98501	4122376395
6813	Diptera	Culicidae	Aedes	Aedes aegypti	SPECIES	2.11333	-76.98218	3302601314
6814	Diptera	Culicidae	Aedes	Aedes aegypti	SPECIES	2.11346	-76.98543	3459452683
6815	Diptera	Culicidae	Aedes	Aedes aegypti	SPECIES	2.11349	-76.98541	3886057736
6816	Diptera	Culicidae	Aedes	Aedes aegypti	SPECIES	2.11349	-76.98361	4122376399
6817	Diptera	Culicidae	Aedes	Aedes aegypti	SPECIES	2.11357	-76.98583	3302601345
6818	Diptera	Culicidae	Aedes	Aedes aegypti	SPECIES	2.11368	-76.98174	3459452661
6819	Diptera	Culicidae	Aedes	Aedes aegypti	SPECIES	2.11369	-76.98207	4122376407
6820	Diptera	Culicidae	Aedes	Aedes aegypti	SPECIES	2.11372	-76.98438	4122376396
6821	Diptera	Culicidae	Aedes	Aedes aegypti	SPECIES	2.11375	-76.98233	4122376397
6822	Diptera	Culicidae	Aedes	Aedes aegypti	SPECIES	2.11386	-76.98423	3886057321
6823	Diptera	Culicidae	Aedes	Aedes aegypti	SPECIES	2.11386	-76.98244	3302601315
6824	Diptera	Culicidae	Aedes	Aedes aegypti	SPECIES	2.11409	-76.98561	3459452684
6825	Diptera	Culicidae	Aedes	Aedes aegypti	SPECIES	2.11410	-76.98466	3886057785
6826	Diptera	Culicidae	Aedes	Aedes aegypti	SPECIES	2.11431	-76.98270	3886057576
6827	Diptera	Culicidae	Aedes	Aedes aegypti	SPECIES	2.11441	-76.98519	4122376308
6828	Diptera	Culicidae	Aedes	Aedes aegypti	SPECIES	2.11453	-76.98524	3302601378
6829	Diptera	Culicidae	Aedes	Aedes aegypti	SPECIES	2.11454	-76.98515	3459452686
6830	Diptera	Culicidae	Aedes	Aedes aegypti	SPECIES	2.11461	-76.98507	3302601358
6831	Diptera	Culicidae	Aedes	Aedes aegypti	SPECIES	2.11466	-76.98419	4075356432
6832	Diptera	Culicidae	Aedes	Aedes aegypti	SPECIES	2.11471	-76.98425	3886057323
6833	Diptera	Culicidae	Aedes	Aedes aegypti	SPECIES	2.11477	-76.98426	3302601359
6834	Diptera	Culicidae	Aedes	Aedes aegypti	SPECIES	2.11492	-76.98467	4122376374
6835	Diptera	Culicidae	Aedes	Aedes aegypti	SPECIES	2.11531	-76.98418	4122376383
6836	Diptera	Culicidae	Aedes	Aedes aegypti	SPECIES	2.11532	-76.98476	4122376381
6837	Diptera	Culicidae	Aedes	Aedes aegypti	SPECIES	2.11533	-76.98429	3886057371
6838	Diptera	Culicidae	Aedes	Aedes aegypti	SPECIES	2.11535	-76.98471	4122376377
6839	Diptera	Culicidae	Aedes	Aedes aegypti	SPECIES	2.11536	-76.98430	3302601363
6840	Diptera	Culicidae	Aedes	Aedes aegypti	SPECIES	2.11536	-76.98471	4122376379
6841	Diptera	Culicidae	Aedes	Aedes aegypti	SPECIES	2.11544	-76.98403	3302601367
6842	Diptera	Culicidae	Aedes	Aedes aegypti	SPECIES	2.11553	-76.98030	3886057828
6843	Diptera	Culicidae	Aedes	Aedes aegypti	SPECIES	2.11553	-76.98487	4122376309
6844	Diptera	Culicidae	Aedes	Aedes aegypti	SPECIES	2.11558	-76.98032	3302601415
6845	Diptera	Culicidae	Aedes	Aedes aegypti	SPECIES	2.11567	-76.98262	3886057701
6846	Diptera	Culicidae	Aedes	Aedes aegypti	SPECIES	2.11597	-76.98122	4122376387
6847	Diptera	Culicidae	Aedes	Aedes aegypti	SPECIES	2.11601	-76.98090	4122376317

№	Order	Family	Genus	Species	Taxon Rank	Latitude	Longitude	GBIF_ID
6848	Diptera	Culicidae	Aedes	Aedes aegypti	SPECIES	2.11612	-76.98114	3459452693
6849	Diptera	Culicidae	Aedes	Aedes aegypti	SPECIES	2.11744	-76.98265	4122376389
6850	Diptera	Culicidae	Aedes	Aedes aegypti	SPECIES	2.11755	-76.98149	3302601357
6851	Diptera	Culicidae	Aedes	Aedes aegypti	SPECIES	2.11762	-76.98072	4122376312
6852	Diptera	Culicidae	Aedes	Aedes aegypti	SPECIES	2.11768	-76.98133	3886057439
6853	Diptera	Culicidae	Aedes	Aedes aegypti	SPECIES	2.11783	-76.98095	3886057816
6854	Diptera	Culicidae	Aedes	Aedes aegypti	SPECIES	2.11803	-76.98139	3302601355
6855	Diptera	Culicidae	Aedes	Aedes aegypti	SPECIES	2.11804	-76.98145	4122376310
6856	Diptera	Culicidae	Aedes	Aedes aegypti	SPECIES	2.11831	-76.98121	4122376311
6857	Diptera	Culicidae	Aedes	Aedes aegypti	SPECIES	2.11918	-76.98055	4122376313
6858	Diptera	Culicidae	Aedes	Aedes aegypti	SPECIES	2.11931	-76.97887	4122376455
6859	Diptera	Culicidae	Aedes	Aedes aegypti	SPECIES	2.11933	-76.97789	4122376454
6860	Diptera	Culicidae	Aedes	Aedes aegypti	SPECIES	2.11951	-76.97811	4122376452
6861	Diptera	Culicidae	Aedes	Aedes aegypti	SPECIES	2.12033	-76.97726	4122376450
6862	Diptera	Culicidae	Aedes	Aedes aegypti	SPECIES	2.12621	-76.97493	3886057757
6863	Diptera	Culicidae	Aedes	Aedes aegypti	SPECIES	2.12890	-76.97304	3886057521
6864	Diptera	Culicidae	Aedes	Aedes aegypti	SPECIES	2.12939	-76.97272	3302601309
6865	Diptera	Culicidae	Aedes	Aedes aegypti	SPECIES	2.12966	-76.97329	3886057605
6866	Diptera	Culicidae	Aedes	Aedes aegypti	SPECIES	2.13014	-76.97292	3886057618
6867	Diptera	Culicidae	Aedes	Aedes aegypti	SPECIES	2.15000	45.18000	1264889508
6868	Diptera	Culicidae	Aedes	Aedes aegypti	SPECIES	2.18000	-61.67000	1264876355
6869	Diptera	Culicidae	Aedes	Aedes aegypti	SPECIES	2.20000	102.25000	1264891130
6870	Diptera	Culicidae	Aedes	Aedes aegypti	SPECIES	2.29000	111.83000	1264889928
6871	Diptera	Culicidae	Aedes	Aedes aegypti	SPECIES	2.30000	-60.60000	1264876384
6872	Diptera	Culicidae	Aedes	Aedes aegypti	SPECIES	2.30000	45.80000	1264889485
6873	Diptera	Culicidae	Aedes	Aedes aegypti	SPECIES	2.33000	102.31000	1264891036
6874	Diptera	Culicidae	Aedes	Aedes aegypti	SPECIES	2.35000	-78.33000	1264887243
6875	Diptera	Culicidae	Aedes	Aedes aegypti	SPECIES	2.39000	-75.89000	1264887219
6876	Diptera	Culicidae	Aedes	Aedes aegypti	SPECIES	2.41000	-60.86000	1264891470
6877	Diptera	Culicidae	Aedes	Aedes aegypti	SPECIES	2.53000	-61.97000	1264876430
6878	Diptera	Culicidae	Aedes	Aedes aegypti	SPECIES	2.57000	-72.63000	1264887368
6879	Diptera	Culicidae	Aedes	Aedes aegypti	SPECIES	2.58000	-72.65000	1264887363
6880	Diptera	Culicidae	Aedes	Aedes aegypti	SPECIES	2.59000	113.30000	1264891050
6881	Diptera	Culicidae	Aedes	Aedes aegypti	SPECIES	2.63000	-51.37000	1264876380
6882	Diptera	Culicidae	Aedes	Aedes aegypti	SPECIES	2.69000	101.98000	1264886311
6883	Diptera	Culicidae	Aedes	Aedes aegypti	SPECIES	2.69000	-75.33000	1264887245
6884	Diptera	Culicidae	Aedes	Aedes aegypti	SPECIES	2.75000	102.16000	1264891158
6885	Diptera	Culicidae	Aedes	Aedes aegypti	SPECIES	2.79000	-60.14000	1264876393
6886	Diptera	Culicidae	Aedes	Aedes aegypti	SPECIES	2.79593	101.72000	3443529781
6887	Diptera	Culicidae	Aedes	Aedes aegypti	SPECIES	2.80000	101.72000	3818512025
6888	Diptera	Culicidae	Aedes	Aedes aegypti	SPECIES	2.81000	-60.72000	1264890528

№	Order	Family	Genus	Species	Taxon Rank	Latitude	Longitude	GBIF_ID
6889	Diptera	Culicidae	Aedes	Aedes aegypti	SPECIES	2.82000	-60.69000	1264890340
6890	Diptera	Culicidae	Aedes	Aedes aegypti	SPECIES	2.83000	-60.66000	1264890625
6891	Diptera	Culicidae	Aedes	Aedes aegypti	SPECIES	2.92000	11.15000	1269973368
6892	Diptera	Culicidae	Aedes	Aedes aegypti	SPECIES	2.93000	-75.29000	1264887241
6893	Diptera	Culicidae	Aedes	Aedes aegypti	SPECIES	2.93000	11.98000	1264889909
6894	Diptera	Culicidae	Aedes	Aedes aegypti	SPECIES	3.02000	-60.64000	1264876364
6895	Diptera	Culicidae	Aedes	Aedes aegypti	SPECIES	3.02000	101.25000	1264886097
6896	Diptera	Culicidae	Aedes	Aedes aegypti	SPECIES	3.02000	-76.48000	1264887211
6897	Diptera	Culicidae	Aedes	Aedes aegypti	SPECIES	3.03000	101.25000	1264886033
6898	Diptera	Culicidae	Aedes	Aedes aegypti	SPECIES	3.04000	101.51000	1264886008
6899	Diptera	Culicidae	Aedes	Aedes aegypti	SPECIES	3.04000	101.50000	1264886020
6900	Diptera	Culicidae	Aedes	Aedes aegypti	SPECIES	3.04000	101.28000	1264886075
6901	Diptera	Culicidae	Aedes	Aedes aegypti	SPECIES	3.04000	101.45000	1264889900
6902	Diptera	Culicidae	Aedes	Aedes aegypti	SPECIES	3.04000	101.44000	1264890650
6903	Diptera	Culicidae	Aedes	Aedes aegypti	SPECIES	3.05000	101.76000	1264891171
6904	Diptera	Culicidae	Aedes	Aedes aegypti	SPECIES	3.06000	101.55000	1264891008
6905	Diptera	Culicidae	Aedes	Aedes aegypti	SPECIES	3.07000	101.52000	1264887396
6906	Diptera	Culicidae	Aedes	Aedes aegypti	SPECIES	3.07000	101.72000	1264887630
6907	Diptera	Culicidae	Aedes	Aedes aegypti	SPECIES	3.08000	101.62000	1264890044
6908	Diptera	Culicidae	Aedes	Aedes aegypti	SPECIES	3.08000	101.53000	1264890988
6909	Diptera	Culicidae	Aedes	Aedes aegypti	SPECIES	3.09000	101.62000	1264886965
6910	Diptera	Culicidae	Aedes	Aedes aegypti	SPECIES	3.09000	101.72000	1264890078
6911	Diptera	Culicidae	Aedes	Aedes aegypti	SPECIES	3.10000	98.51000	1264871735
6912	Diptera	Culicidae	Aedes	Aedes aegypti	SPECIES	3.10000	-62.63000	1264876423
6913	Diptera	Culicidae	Aedes	Aedes aegypti	SPECIES	3.11000	101.66000	1264887598
6914	Diptera	Culicidae	Aedes	Aedes aegypti	SPECIES	3.11000	101.73000	1264890051
6915	Diptera	Culicidae	Aedes	Aedes aegypti	SPECIES	3.12000	101.68000	1264889156
6916	Diptera	Culicidae	Aedes	Aedes aegypti	SPECIES	3.12000	101.72000	1264890082
6917	Diptera	Culicidae	Aedes	Aedes aegypti	SPECIES	3.12000	-60.72000	1264890878
6918	Diptera	Culicidae	Aedes	Aedes aegypti	SPECIES	3.13000	101.70000	1264886980
6919	Diptera	Culicidae	Aedes	Aedes aegypti	SPECIES	3.13000	101.63000	1264891113
6920	Diptera	Culicidae	Aedes	Aedes aegypti	SPECIES	3.14000	101.73000	1264888314
6921	Diptera	Culicidae	Aedes	Aedes aegypti	SPECIES	3.14000	101.69000	1264891378
6922	Diptera	Culicidae	Aedes	Aedes aegypti	SPECIES	3.15000	101.72000	1264890030
6923	Diptera	Culicidae	Aedes	Aedes aegypti	SPECIES	3.16000	101.71000	1264888280
6924	Diptera	Culicidae	Aedes	Aedes aegypti	SPECIES	3.16000	101.66000	1264890002
6925	Diptera	Culicidae	Aedes	Aedes aegypti	SPECIES	3.16000	101.76000	1264890086
6926	Diptera	Culicidae	Aedes	Aedes aegypti	SPECIES	3.16000	101.70000	1264891355
6927	Diptera	Culicidae	Aedes	Aedes aegypti	SPECIES	3.17000	101.71000	1264889869
6928	Diptera	Culicidae	Aedes	Aedes aegypti	SPECIES	3.18000	101.70000	1264891062
6929	Diptera	Culicidae	Aedes	Aedes aegypti	SPECIES	3.19000	-52.22000	1264876413

№	Order	Family	Genus	Species	Taxon Rank	Latitude	Longitude	GBIF_ID
6930	Diptera	Culicidae	Aedes	Aedes aegypti	SPECIES	3.21000	101.64000	1264890237
6931	Diptera	Culicidae	Aedes	Aedes aegypti	SPECIES	3.21000	101.63000	1264891086
6932	Diptera	Culicidae	Aedes	Aedes aegypti	SPECIES	3.22000	101.73000	1264887626
6933	Diptera	Culicidae	Aedes	Aedes aegypti	SPECIES	3.23000	-76.42000	1264887222
6934	Diptera	Culicidae	Aedes	Aedes aegypti	SPECIES	3.23000	101.70000	1264890503
6935	Diptera	Culicidae	Aedes	Aedes aegypti	SPECIES	3.23878	-76.23216	4122376350
6936	Diptera	Culicidae	Aedes	Aedes aegypti	SPECIES	3.24173	-76.23313	4122376346
6937	Diptera	Culicidae	Aedes	Aedes aegypti	SPECIES	3.24175	-76.23223	4122376348
6938	Diptera	Culicidae	Aedes	Aedes aegypti	SPECIES	3.24599	-76.23035	3459452339
6939	Diptera	Culicidae	Aedes	Aedes aegypti	SPECIES	3.24599	-76.23183	4122376343
6940	Diptera	Culicidae	Aedes	Aedes aegypti	SPECIES	3.24603	-76.23072	3886057679
6941	Diptera	Culicidae	Aedes	Aedes aegypti	SPECIES	3.24603	-76.23172	4122376341
6942	Diptera	Culicidae	Aedes	Aedes aegypti	SPECIES	3.24605	-76.22609	4122376352
6943	Diptera	Culicidae	Aedes	Aedes aegypti	SPECIES	3.24613	-76.22611	3886057662
6944	Diptera	Culicidae	Aedes	Aedes aegypti	SPECIES	3.24614	-76.23037	3459452321
6945	Diptera	Culicidae	Aedes	Aedes aegypti	SPECIES	3.24627	-76.22743	3886057698
6946	Diptera	Culicidae	Aedes	Aedes aegypti	SPECIES	3.24631	-76.22771	4122376356
6947	Diptera	Culicidae	Aedes	Aedes aegypti	SPECIES	3.24634	-76.22959	4122376344
6948	Diptera	Culicidae	Aedes	Aedes aegypti	SPECIES	3.24636	-76.22829	3886057684
6949	Diptera	Culicidae	Aedes	Aedes aegypti	SPECIES	3.24639	-76.22762	3886057695
6950	Diptera	Culicidae	Aedes	Aedes aegypti	SPECIES	3.24642	-76.22670	4122376456
6951	Diptera	Culicidae	Aedes	Aedes aegypti	SPECIES	3.24648	-76.22758	4122376354
6952	Diptera	Culicidae	Aedes	Aedes aegypti	SPECIES	3.24659	-76.23035	4122376339
6953	Diptera	Culicidae	Aedes	Aedes aegypti	SPECIES	3.24661	-76.22781	4122376457
6954	Diptera	Culicidae	Aedes	Aedes aegypti	SPECIES	3.24677	-76.22760	3886057651
6955	Diptera	Culicidae	Aedes	Aedes aegypti	SPECIES	3.24679	-76.22776	3886057696
6956	Diptera	Culicidae	Aedes	Aedes aegypti	SPECIES	3.24680	-76.22721	3886057685
6957	Diptera	Culicidae	Aedes	Aedes aegypti	SPECIES	3.24692	-76.23017	3459452327
6958	Diptera	Culicidae	Aedes	Aedes aegypti	SPECIES	3.24698	-76.22702	3886057714
6959	Diptera	Culicidae	Aedes	Aedes aegypti	SPECIES	3.24709	-76.23023	3886057311
6960	Diptera	Culicidae	Aedes	Aedes aegypti	SPECIES	3.24726	-76.22651	4122376328
6961	Diptera	Culicidae	Aedes	Aedes aegypti	SPECIES	3.24727	-76.22780	4122376420
6962	Diptera	Culicidae	Aedes	Aedes aegypti	SPECIES	3.24743	-76.22764	4122376418
6963	Diptera	Culicidae	Aedes	Aedes aegypti	SPECIES	3.24746	-76.22966	3886057429
6964	Diptera	Culicidae	Aedes	Aedes aegypti	SPECIES	3.24762	-76.22952	4075356357
6965	Diptera	Culicidae	Aedes	Aedes aegypti	SPECIES	3.24763	-76.22910	3459452325
6966	Diptera	Culicidae	Aedes	Aedes aegypti	SPECIES	3.24765	-76.23087	4122376332
6967	Diptera	Culicidae	Aedes	Aedes aegypti	SPECIES	3.24772	-76.22656	4122376326
6968	Diptera	Culicidae	Aedes	Aedes aegypti	SPECIES	3.24773	-76.22564	3886057693
6969	Diptera	Culicidae	Aedes	Aedes aegypti	SPECIES	3.24775	-76.23053	4122376334
6970	Diptera	Culicidae	Aedes	Aedes aegypti	SPECIES	3.24775	-76.22950	4122376336

№	Order	Family	Genus	Species	Taxon Rank	Latitude	Longitude	GBIF_ID
6971	Diptera	Culicidae	Aedes	Aedes aegypti	SPECIES	3.24780	-76.22626	4122376324
6972	Diptera	Culicidae	Aedes	Aedes aegypti	SPECIES	3.24786	-76.22603	3886057674
6973	Diptera	Culicidae	Aedes	Aedes aegypti	SPECIES	3.24796	-76.22677	3886057692
6974	Diptera	Culicidae	Aedes	Aedes aegypti	SPECIES	3.24798	-76.22893	3459452320
6975	Diptera	Culicidae	Aedes	Aedes aegypti	SPECIES	3.24802	-76.22564	3886057665
6976	Diptera	Culicidae	Aedes	Aedes aegypti	SPECIES	3.24821	-76.23097	3459452356
6977	Diptera	Culicidae	Aedes	Aedes aegypti	SPECIES	3.24822	-76.22930	3886057407
6978	Diptera	Culicidae	Aedes	Aedes aegypti	SPECIES	3.24829	-76.23094	3886057699
6979	Diptera	Culicidae	Aedes	Aedes aegypti	SPECIES	3.24831	-76.22931	4075356414
6980	Diptera	Culicidae	Aedes	Aedes aegypti	SPECIES	3.24833	-76.23136	3459452384
6981	Diptera	Culicidae	Aedes	Aedes aegypti	SPECIES	3.24836	-76.23061	4122376338
6982	Diptera	Culicidae	Aedes	Aedes aegypti	SPECIES	3.24838	-76.23045	3459452386
6983	Diptera	Culicidae	Aedes	Aedes aegypti	SPECIES	3.24840	-76.22813	3886057694
6984	Diptera	Culicidae	Aedes	Aedes aegypti	SPECIES	3.24846	-76.22897	3886057832
6985	Diptera	Culicidae	Aedes	Aedes aegypti	SPECIES	3.24855	-76.22957	3459452323
6986	Diptera	Culicidae	Aedes	Aedes aegypti	SPECIES	3.24855	-76.22664	4122376322
6987	Diptera	Culicidae	Aedes	Aedes aegypti	SPECIES	3.24858	-76.23072	4122376337
6988	Diptera	Culicidae	Aedes	Aedes aegypti	SPECIES	3.24858	-76.23169	3886057646
6989	Diptera	Culicidae	Aedes	Aedes aegypti	SPECIES	3.24864	-76.22891	3886057730
6990	Diptera	Culicidae	Aedes	Aedes aegypti	SPECIES	3.24870	-76.22892	4122376442
6991	Diptera	Culicidae	Aedes	Aedes aegypti	SPECIES	3.24872	-76.23084	4122376301
6992	Diptera	Culicidae	Aedes	Aedes aegypti	SPECIES	3.24877	-76.23066	4122376330
6993	Diptera	Culicidae	Aedes	Aedes aegypti	SPECIES	3.24881	-76.22901	3886057347
6994	Diptera	Culicidae	Aedes	Aedes aegypti	SPECIES	3.24881	-76.23167	3886057544
6995	Diptera	Culicidae	Aedes	Aedes aegypti	SPECIES	3.24886	-76.23172	4075356383
6996	Diptera	Culicidae	Aedes	Aedes aegypti	SPECIES	3.24886	-76.23116	4122376335
6997	Diptera	Culicidae	Aedes	Aedes aegypti	SPECIES	3.24887	-76.23081	3886057644
6998	Diptera	Culicidae	Aedes	Aedes aegypti	SPECIES	3.24887	-76.23180	4122376443
6999	Diptera	Culicidae	Aedes	Aedes aegypti	SPECIES	3.24893	-76.23179	3886057602
7000	Diptera	Culicidae	Aedes	Aedes aegypti	SPECIES	3.24894	-76.22839	3886057738
7001	Diptera	Culicidae	Aedes	Aedes aegypti	SPECIES	3.24895	-76.23177	3459452308
7002	Diptera	Culicidae	Aedes	Aedes aegypti	SPECIES	3.24944	-76.23510	3459452395
7003	Diptera	Culicidae	Aedes	Aedes aegypti	SPECIES	3.24946	-76.23122	3886057677
7004	Diptera	Culicidae	Aedes	Aedes aegypti	SPECIES	3.24950	-76.23164	3886057629
7005	Diptera	Culicidae	Aedes	Aedes aegypti	SPECIES	3.24952	-76.23119	3886057645
7006	Diptera	Culicidae	Aedes	Aedes aegypti	SPECIES	3.24954	-76.23360	3459452396
7007	Diptera	Culicidae	Aedes	Aedes aegypti	SPECIES	3.24959	-76.23358	3459452397
7008	Diptera	Culicidae	Aedes	Aedes aegypti	SPECIES	3.24966	-76.23080	4122376445
7009	Diptera	Culicidae	Aedes	Aedes aegypti	SPECIES	3.24966	-76.23080	3459452393
7010	Diptera	Culicidae	Aedes	Aedes aegypti	SPECIES	3.24969	-76.23364	3459452372
7011	Diptera	Culicidae	Aedes	Aedes aegypti	SPECIES	3.24970	-76.23163	4122376444

№	Order	Family	Genus	Species	Taxon Rank	Latitude	Longitude	GBIF_ID
7012	Diptera	Culicidae	Aedes	Aedes aegypti	SPECIES	3.24972	-76.23292	3459452398
7013	Diptera	Culicidae	Aedes	Aedes aegypti	SPECIES	3.24983	-76.22812	4075356416
7014	Diptera	Culicidae	Aedes	Aedes aegypti	SPECIES	3.24992	-76.23116	3886057642
7015	Diptera	Culicidae	Aedes	Aedes aegypti	SPECIES	3.25000	125.47000	1264891027
7016	Diptera	Culicidae	Aedes	Aedes aegypti	SPECIES	3.25005	-76.23157	3459452394
7017	Diptera	Culicidae	Aedes	Aedes aegypti	SPECIES	3.25033	-76.23285	3459452399
7018	Diptera	Culicidae	Aedes	Aedes aegypti	SPECIES	3.25034	-76.23337	3886057420
7019	Diptera	Culicidae	Aedes	Aedes aegypti	SPECIES	3.25034	-76.23337	4122376431
7020	Diptera	Culicidae	Aedes	Aedes aegypti	SPECIES	3.25047	-76.23400	3459452374
7021	Diptera	Culicidae	Aedes	Aedes aegypti	SPECIES	3.25070	-76.23308	3459452389
7022	Diptera	Culicidae	Aedes	Aedes aegypti	SPECIES	3.25071	-76.22652	3459452318
7023	Diptera	Culicidae	Aedes	Aedes aegypti	SPECIES	3.25073	-76.23112	4122376460
7024	Diptera	Culicidae	Aedes	Aedes aegypti	SPECIES	3.25079	-76.23386	3886057351
7025	Diptera	Culicidae	Aedes	Aedes aegypti	SPECIES	3.25079	-76.23073	4122376345
7026	Diptera	Culicidae	Aedes	Aedes aegypti	SPECIES	3.25082	-76.23359	3459452391
7027	Diptera	Culicidae	Aedes	Aedes aegypti	SPECIES	3.25101	-76.23319	3459452375
7028	Diptera	Culicidae	Aedes	Aedes aegypti	SPECIES	3.25118	-76.23272	3459452377
7029	Diptera	Culicidae	Aedes	Aedes aegypti	SPECIES	3.25146	-76.23358	4122376447
7030	Diptera	Culicidae	Aedes	Aedes aegypti	SPECIES	3.25170	-76.23287	3459452379
7031	Diptera	Culicidae	Aedes	Aedes aegypti	SPECIES	3.25176	-76.23278	3459452383
7032	Diptera	Culicidae	Aedes	Aedes aegypti	SPECIES	3.25179	-76.23359	3459452322
7033	Diptera	Culicidae	Aedes	Aedes aegypti	SPECIES	3.25179	-76.23367	3886057786
7034	Diptera	Culicidae	Aedes	Aedes aegypti	SPECIES	3.25199	-76.23292	3886057778
7035	Diptera	Culicidae	Aedes	Aedes aegypti	SPECIES	3.25203	-76.23337	3459452376
7036	Diptera	Culicidae	Aedes	Aedes aegypti	SPECIES	3.25204	-76.23347	3459452336
7037	Diptera	Culicidae	Aedes	Aedes aegypti	SPECIES	3.25206	-76.23345	3459452338
7038	Diptera	Culicidae	Aedes	Aedes aegypti	SPECIES	3.25207	-76.23341	3886057512
7039	Diptera	Culicidae	Aedes	Aedes aegypti	SPECIES	3.25221	-76.23277	3886057505
7040	Diptera	Culicidae	Aedes	Aedes aegypti	SPECIES	3.25240	-76.23312	3886057312
7041	Diptera	Culicidae	Aedes	Aedes aegypti	SPECIES	3.25251	-76.23332	4075356341
7042	Diptera	Culicidae	Aedes	Aedes aegypti	SPECIES	3.25306	-76.23179	3886057656
7043	Diptera	Culicidae	Aedes	Aedes aegypti	SPECIES	3.25307	-76.23188	3886057782
7044	Diptera	Culicidae	Aedes	Aedes aegypti	SPECIES	3.25330	-76.23099	4122376358
7045	Diptera	Culicidae	Aedes	Aedes aegypti	SPECIES	3.25335	-76.23153	3886057721
7046	Diptera	Culicidae	Aedes	Aedes aegypti	SPECIES	3.25336	-76.23052	3886057722
7047	Diptera	Culicidae	Aedes	Aedes aegypti	SPECIES	3.25344	-76.22995	4122376373
7048	Diptera	Culicidae	Aedes	Aedes aegypti	SPECIES	3.25344	-76.23083	3886057624
7049	Diptera	Culicidae	Aedes	Aedes aegypti	SPECIES	3.25346	-76.23075	3886057718
7050	Diptera	Culicidae	Aedes	Aedes aegypti	SPECIES	3.25348	-76.22689	3459452392
7051	Diptera	Culicidae	Aedes	Aedes aegypti	SPECIES	3.25351	-76.22676	3886057803
7052	Diptera	Culicidae	Aedes	Aedes aegypti	SPECIES	3.25352	-76.23185	3886057626

№	Order	Family	Genus	Species	Taxon Rank	Latitude	Longitude	GBIF_ID
7053	Diptera	Culicidae	Aedes	Aedes aegypti	SPECIES	3.25352	-76.23190	3886057636
7054	Diptera	Culicidae	Aedes	Aedes aegypti	SPECIES	3.25371	-76.22847	3886057737
7055	Diptera	Culicidae	Aedes	Aedes aegypti	SPECIES	3.25374	-76.22850	3459452370
7056	Diptera	Culicidae	Aedes	Aedes aegypti	SPECIES	3.25378	-76.23038	3886057716
7057	Diptera	Culicidae	Aedes	Aedes aegypti	SPECIES	3.25388	-76.23109	3886057661
7058	Diptera	Culicidae	Aedes	Aedes aegypti	SPECIES	3.25393	-76.22719	3459452400
7059	Diptera	Culicidae	Aedes	Aedes aegypti	SPECIES	3.25394	-76.23093	4122376360
7060	Diptera	Culicidae	Aedes	Aedes aegypti	SPECIES	3.25396	-76.22858	4122376429
7061	Diptera	Culicidae	Aedes	Aedes aegypti	SPECIES	3.25398	-76.22774	3459452667
7062	Diptera	Culicidae	Aedes	Aedes aegypti	SPECIES	3.25401	-76.22673	3459452367
7063	Diptera	Culicidae	Aedes	Aedes aegypti	SPECIES	3.25406	-76.22811	3459452362
7064	Diptera	Culicidae	Aedes	Aedes aegypti	SPECIES	3.25407	-76.23048	3886057717
7065	Diptera	Culicidae	Aedes	Aedes aegypti	SPECIES	3.25412	-76.22815	4122376329
7066	Diptera	Culicidae	Aedes	Aedes aegypti	SPECIES	3.25412	-76.22815	3459452388
7067	Diptera	Culicidae	Aedes	Aedes aegypti	SPECIES	3.25416	-76.22976	3886057715
7068	Diptera	Culicidae	Aedes	Aedes aegypti	SPECIES	3.25430	-76.23188	3886057735
7069	Diptera	Culicidae	Aedes	Aedes aegypti	SPECIES	3.25455	-76.22743	3459452666
7070	Diptera	Culicidae	Aedes	Aedes aegypti	SPECIES	3.25476	-76.22882	4122376428
7071	Diptera	Culicidae	Aedes	Aedes aegypti	SPECIES	3.25504	-76.22888	3459452369
7072	Diptera	Culicidae	Aedes	Aedes aegypti	SPECIES	3.25510	-76.22774	4122376331
7073	Diptera	Culicidae	Aedes	Aedes aegypti	SPECIES	3.25511	-76.22805	3886057352
7074	Diptera	Culicidae	Aedes	Aedes aegypti	SPECIES	3.25529	-76.22931	3886057732
7075	Diptera	Culicidae	Aedes	Aedes aegypti	SPECIES	3.25542	-76.22738	4122376427
7076	Diptera	Culicidae	Aedes	Aedes aegypti	SPECIES	3.25543	-76.22764	3459452366
7077	Diptera	Culicidae	Aedes	Aedes aegypti	SPECIES	3.25605	-76.22899	3886057627
7078	Diptera	Culicidae	Aedes	Aedes aegypti	SPECIES	3.25616	-76.22742	3459452364
7079	Diptera	Culicidae	Aedes	Aedes aegypti	SPECIES	3.25623	-76.23212	3886057606
7080	Diptera	Culicidae	Aedes	Aedes aegypti	SPECIES	3.25623	-76.23159	4122376323
7081	Diptera	Culicidae	Aedes	Aedes aegypti	SPECIES	3.25624	-76.23239	4122376425
7082	Diptera	Culicidae	Aedes	Aedes aegypti	SPECIES	3.25655	-76.23210	3459452689
7083	Diptera	Culicidae	Aedes	Aedes aegypti	SPECIES	3.25688	-76.23287	3459452639
7084	Diptera	Culicidae	Aedes	Aedes aegypti	SPECIES	3.25700	-76.23271	3886057797
7085	Diptera	Culicidae	Aedes	Aedes aegypti	SPECIES	3.25703	-76.23272	3459452690
7086	Diptera	Culicidae	Aedes	Aedes aegypti	SPECIES	3.25704	-76.23244	3886057756
7087	Diptera	Culicidae	Aedes	Aedes aegypti	SPECIES	3.25704	-76.23268	3459452654
7088	Diptera	Culicidae	Aedes	Aedes aegypti	SPECIES	3.25705	-76.23254	3459452638
7089	Diptera	Culicidae	Aedes	Aedes aegypti	SPECIES	3.25711	-76.23266	4122376426
7090	Diptera	Culicidae	Aedes	Aedes aegypti	SPECIES	3.25722	-76.23304	3886057734
7091	Diptera	Culicidae	Aedes	Aedes aegypti	SPECIES	3.25735	-76.23196	3886057728
7092	Diptera	Culicidae	Aedes	Aedes aegypti	SPECIES	3.25738	-76.23253	3459452655
7093	Diptera	Culicidae	Aedes	Aedes aegypti	SPECIES	3.25740	-76.23277	3886057739

№	Order	Family	Genus	Species	Taxon Rank	Latitude	Longitude	GBIF_ID
7094	Diptera	Culicidae	Aedes	Aedes aegypti	SPECIES	3.25746	-76.23188	3886057633
7095	Diptera	Culicidae	Aedes	Aedes aegypti	SPECIES	3.25749	-76.23248	3886057761
7096	Diptera	Culicidae	Aedes	Aedes aegypti	SPECIES	3.25751	-76.23212	3886057758
7097	Diptera	Culicidae	Aedes	Aedes aegypti	SPECIES	3.25753	-76.23279	3459452653
7098	Diptera	Culicidae	Aedes	Aedes aegypti	SPECIES	3.25758	-76.23183	3886057731
7099	Diptera	Culicidae	Aedes	Aedes aegypti	SPECIES	3.25760	-76.23218	3459452640
7100	Diptera	Culicidae	Aedes	Aedes aegypti	SPECIES	3.25760	-76.23251	3886057775
7101	Diptera	Culicidae	Aedes	Aedes aegypti	SPECIES	3.25761	-76.23190	3886057310
7102	Diptera	Culicidae	Aedes	Aedes aegypti	SPECIES	3.25761	-76.23291	3886057784
7103	Diptera	Culicidae	Aedes	Aedes aegypti	SPECIES	3.25767	-76.23291	4122376371
7104	Diptera	Culicidae	Aedes	Aedes aegypti	SPECIES	3.25771	-76.23210	3886057340
7105	Diptera	Culicidae	Aedes	Aedes aegypti	SPECIES	3.25776	-76.23240	3886057760
7106	Diptera	Culicidae	Aedes	Aedes aegypti	SPECIES	3.25783	-76.23262	3886057733
7107	Diptera	Culicidae	Aedes	Aedes aegypti	SPECIES	3.25783	-76.23179	3459452658
7108	Diptera	Culicidae	Aedes	Aedes aegypti	SPECIES	3.25785	-76.23265	3886057660
7109	Diptera	Culicidae	Aedes	Aedes aegypti	SPECIES	3.25786	-76.23284	3886057635
7110	Diptera	Culicidae	Aedes	Aedes aegypti	SPECIES	3.25787	-76.23269	4122376362
7111	Diptera	Culicidae	Aedes	Aedes aegypti	SPECIES	3.25792	-76.23176	3886057729
7112	Diptera	Culicidae	Aedes	Aedes aegypti	SPECIES	3.25799	-76.23208	3886057753
7113	Diptera	Culicidae	Aedes	Aedes aegypti	SPECIES	3.25817	-76.23207	3886057755
7114	Diptera	Culicidae	Aedes	Aedes aegypti	SPECIES	3.25827	-76.23203	3886057710
7115	Diptera	Culicidae	Aedes	Aedes aegypti	SPECIES	3.25835	-76.23171	3886057632
7116	Diptera	Culicidae	Aedes	Aedes aegypti	SPECIES	3.25839	-76.23161	3459452641
7117	Diptera	Culicidae	Aedes	Aedes aegypti	SPECIES	3.25842	-76.23212	4122376327
7118	Diptera	Culicidae	Aedes	Aedes aegypti	SPECIES	3.25849	-76.23177	3459452662
7119	Diptera	Culicidae	Aedes	Aedes aegypti	SPECIES	3.25852	-76.23182	3886057745
7120	Diptera	Culicidae	Aedes	Aedes aegypti	SPECIES	3.25854	-76.23176	3886057740
7121	Diptera	Culicidae	Aedes	Aedes aegypti	SPECIES	3.25870	-76.23228	4122376433
7122	Diptera	Culicidae	Aedes	Aedes aegypti	SPECIES	3.25876	-76.22817	4075356353
7123	Diptera	Culicidae	Aedes	Aedes aegypti	SPECIES	3.25882	-76.22878	3459452647
7124	Diptera	Culicidae	Aedes	Aedes aegypti	SPECIES	3.25890	-76.23125	3886057720
7125	Diptera	Culicidae	Aedes	Aedes aegypti	SPECIES	3.25903	-76.23148	3886057630
7126	Diptera	Culicidae	Aedes	Aedes aegypti	SPECIES	3.25906	-76.23126	3459452664
7127	Diptera	Culicidae	Aedes	Aedes aegypti	SPECIES	3.25906	-76.23122	3886057703
7128	Diptera	Culicidae	Aedes	Aedes aegypti	SPECIES	3.25908	-76.22994	4122376342
7129	Diptera	Culicidae	Aedes	Aedes aegypti	SPECIES	3.25919	-76.23042	4122376340
7130	Diptera	Culicidae	Aedes	Aedes aegypti	SPECIES	3.25921	-76.23043	3886057523
7131	Diptera	Culicidae	Aedes	Aedes aegypti	SPECIES	3.25930	-76.22949	3459452699
7132	Diptera	Culicidae	Aedes	Aedes aegypti	SPECIES	3.25930	-76.22921	3886057712
7133	Diptera	Culicidae	Aedes	Aedes aegypti	SPECIES	3.25931	-76.23204	3459452371
7134	Diptera	Culicidae	Aedes	Aedes aegypti	SPECIES	3.25935	-76.22967	3886057724

№	Order	Family	Genus	Species	Taxon Rank	Latitude	Longitude	GBIF_ID
7135	Diptera	Culicidae	Aedes	Aedes aegypti	SPECIES	3.25936	-76.22839	3459452650
7136	Diptera	Culicidae	Aedes	Aedes aegypti	SPECIES	3.25939	-76.22857	3886057673
7137	Diptera	Culicidae	Aedes	Aedes aegypti	SPECIES	3.25940	-76.22980	3886057649
7138	Diptera	Culicidae	Aedes	Aedes aegypti	SPECIES	3.25944	-76.22844	3459452651
7139	Diptera	Culicidae	Aedes	Aedes aegypti	SPECIES	3.25945	-76.22917	3886057709
7140	Diptera	Culicidae	Aedes	Aedes aegypti	SPECIES	3.25949	-76.23098	3459452663
7141	Diptera	Culicidae	Aedes	Aedes aegypti	SPECIES	3.25954	-76.23003	3886057706
7142	Diptera	Culicidae	Aedes	Aedes aegypti	SPECIES	3.25959	-76.23026	4122376459
7143	Diptera	Culicidae	Aedes	Aedes aegypti	SPECIES	3.25962	-76.23083	3886057676
7144	Diptera	Culicidae	Aedes	Aedes aegypti	SPECIES	3.25969	-76.23084	3459452696
7145	Diptera	Culicidae	Aedes	Aedes aegypti	SPECIES	3.25971	-76.22955	3886057708
7146	Diptera	Culicidae	Aedes	Aedes aegypti	SPECIES	3.25972	-76.23168	3886057702
7147	Diptera	Culicidae	Aedes	Aedes aegypti	SPECIES	3.25974	-76.23081	4122376423
7148	Diptera	Culicidae	Aedes	Aedes aegypti	SPECIES	3.25975	-76.23079	3459452697
7149	Diptera	Culicidae	Aedes	Aedes aegypti	SPECIES	3.25981	-76.22841	3886057640
7150	Diptera	Culicidae	Aedes	Aedes aegypti	SPECIES	3.25981	-76.23145	4122376325
7151	Diptera	Culicidae	Aedes	Aedes aegypti	SPECIES	3.25983	-76.22812	3886057689
7152	Diptera	Culicidae	Aedes	Aedes aegypti	SPECIES	3.25986	-76.23036	4075356323
7153	Diptera	Culicidae	Aedes	Aedes aegypti	SPECIES	3.25987	-76.22909	3886057653
7154	Diptera	Culicidae	Aedes	Aedes aegypti	SPECIES	3.25989	-76.23056	3459452645
7155	Diptera	Culicidae	Aedes	Aedes aegypti	SPECIES	3.25993	-76.23057	4122376458
7156	Diptera	Culicidae	Aedes	Aedes aegypti	SPECIES	3.25995	-76.22989	3886057671
7157	Diptera	Culicidae	Aedes	Aedes aegypti	SPECIES	3.26000	117.62000	1264876512
7158	Diptera	Culicidae	Aedes	Aedes aegypti	SPECIES	3.26004	-76.23016	3886057719
7159	Diptera	Culicidae	Aedes	Aedes aegypti	SPECIES	3.26004	-76.22868	3886057688
7160	Diptera	Culicidae	Aedes	Aedes aegypti	SPECIES	3.26009	-76.23016	3886057776
7161	Diptera	Culicidae	Aedes	Aedes aegypti	SPECIES	3.26009	-76.23016	4122376424
7162	Diptera	Culicidae	Aedes	Aedes aegypti	SPECIES	3.26009	-76.22892	3459452694
7163	Diptera	Culicidae	Aedes	Aedes aegypti	SPECIES	3.26010	-76.22982	3459452643
7164	Diptera	Culicidae	Aedes	Aedes aegypti	SPECIES	3.26014	-76.22923	3459452695
7165	Diptera	Culicidae	Aedes	Aedes aegypti	SPECIES	3.26018	-76.22868	3886057638
7166	Diptera	Culicidae	Aedes	Aedes aegypti	SPECIES	3.26020	-76.22892	3886057809
7167	Diptera	Culicidae	Aedes	Aedes aegypti	SPECIES	3.26023	-76.22834	3886057690
7168	Diptera	Culicidae	Aedes	Aedes aegypti	SPECIES	3.26024	-76.22863	3459452642
7169	Diptera	Culicidae	Aedes	Aedes aegypti	SPECIES	3.27000	117.64000	1264876511
7170	Diptera	Culicidae	Aedes	Aedes aegypti	SPECIES	3.27000	101.50000	1264891046
7171	Diptera	Culicidae	Aedes	Aedes aegypti	SPECIES	3.31000	117.60000	1264876508
7172	Diptera	Culicidae	Aedes	Aedes aegypti	SPECIES	3.35000	101.25000	177195193
7173	Diptera	Culicidae	Aedes	Aedes aegypti	SPECIES	3.37000	117.60000	1264876536
7174	Diptera	Culicidae	Aedes	Aedes aegypti	SPECIES	3.41000	-76.51000	1264887258
7175	Diptera	Culicidae	Aedes	Aedes aegypti	SPECIES	3.41778	-76.48805	2550023081

№	Order	Family	Genus	Species	Taxon Rank	Latitude	Longitude	GBIF_ID
7176	Diptera	Culicidae	Aedes	Aedes aegypti	SPECIES	3.42000	-76.52000	1264886354
7177	Diptera	Culicidae	Aedes	Aedes aegypti	SPECIES	3.43000	-76.53000	1264889511
7178	Diptera	Culicidae	Aedes	Aedes aegypti	SPECIES	3.43000	-76.52000	1264891323
7179	Diptera	Culicidae	Aedes	Aedes aegypti	SPECIES	3.45000	-76.52000	1264890562
7180	Diptera	Culicidae	Aedes	Aedes aegypti	SPECIES	3.46000	-76.47000	1264889531
7181	Diptera	Culicidae	Aedes	Aedes aegypti	SPECIES	3.47000	-76.52000	1264889530
7182	Diptera	Culicidae	Aedes	Aedes aegypti	SPECIES	3.48000	-76.47000	1264889526
7183	Diptera	Culicidae	Aedes	Aedes aegypti	SPECIES	3.48000	-76.48000	1264889550
7184	Diptera	Culicidae	Aedes	Aedes aegypti	SPECIES	3.52000	11.50000	1264889949
7185	Diptera	Culicidae	Aedes	Aedes aegypti	SPECIES	3.53000	-76.30000	1264891305
7186	Diptera	Culicidae	Aedes	Aedes aegypti	SPECIES	3.54000	98.71000	1264871792
7187	Diptera	Culicidae	Aedes	Aedes aegypti	SPECIES	3.55000	98.63000	1264871804
7188	Diptera	Culicidae	Aedes	Aedes aegypti	SPECIES	3.55000	-76.31000	1264889345
7189	Diptera	Culicidae	Aedes	Aedes aegypti	SPECIES	3.56000	98.71000	1264876527
7190	Diptera	Culicidae	Aedes	Aedes aegypti	SPECIES	3.56109	-76.71198	4137636307
7191	Diptera	Culicidae	Aedes	Aedes aegypti	SPECIES	3.57000	98.66000	1264871781
7192	Diptera	Culicidae	Aedes	Aedes aegypti	SPECIES	3.57000	101.89000	1264886948
7193	Diptera	Culicidae	Aedes	Aedes aegypti	SPECIES	3.57000	101.88000	1264886961
7194	Diptera	Culicidae	Aedes	Aedes aegypti	SPECIES	3.58000	98.63000	1264871822
7195	Diptera	Culicidae	Aedes	Aedes aegypti	SPECIES	3.58000	98.66000	1264891251
7196	Diptera	Culicidae	Aedes	Aedes aegypti	SPECIES	3.59546	98.67263	3901436234
7197	Diptera	Culicidae	Aedes	Aedes aegypti	SPECIES	3.60000	98.63000	1264871818
7198	Diptera	Culicidae	Aedes	Aedes aegypti	SPECIES	3.61667	125.48330	177195181
7199	Diptera	Culicidae	Aedes	Aedes aegypti	SPECIES	3.61667	125.48330	1633663747
7200	Diptera	Culicidae	Aedes	Aedes aegypti	SPECIES	3.64000	-54.03000	1264890908
7201	Diptera	Culicidae	Aedes	Aedes aegypti	SPECIES	3.72000	-62.71000	1264876396
7202	Diptera	Culicidae	Aedes	Aedes aegypti	SPECIES	3.73000	102.55000	1264890963
7203	Diptera	Culicidae	Aedes	Aedes aegypti	SPECIES	3.80000	10.13000	1264886102
7204	Diptera	Culicidae	Aedes	Aedes aegypti	SPECIES	3.82056	-159.33841	1633667586
7205	Diptera	Culicidae	Aedes	Aedes aegypti	SPECIES	3.84000	-60.03000	1264876315
7206	Diptera	Culicidae	Aedes	Aedes aegypti	SPECIES	3.84000	11.50000	1264886011
7207	Diptera	Culicidae	Aedes	Aedes aegypti	SPECIES	3.85000	10.52000	1264886127
7208	Diptera	Culicidae	Aedes	Aedes aegypti	SPECIES	3.85000	11.50000	1264886159
7209	Diptera	Culicidae	Aedes	Aedes aegypti	SPECIES	3.86700	-159.33300	177195192
7210	Diptera	Culicidae	Aedes	Aedes aegypti	SPECIES	3.87000	100.82000	1264889103
7211	Diptera	Culicidae	Aedes	Aedes aegypti	SPECIES	3.87000	11.52000	1264889926
7212	Diptera	Culicidae	Aedes	Aedes aegypti	SPECIES	3.88000	-77.04000	1264889384
7213	Diptera	Culicidae	Aedes	Aedes aegypti	SPECIES	3.89000	-51.81000	1264886646
7214	Diptera	Culicidae	Aedes	Aedes aegypti	SPECIES	3.89847	11.51985	732501180
7215	Diptera	Culicidae	Aedes	Aedes aegypti	SPECIES	3.90000	-51.81000	1264888192
7216	Diptera	Culicidae	Aedes	Aedes aegypti	SPECIES	3.90000	12.53000	1264889985

№	Order	Family	Genus	Species	Taxon Rank	Latitude	Longitude	GBIF_ID
7217	Diptera	Culicidae	Aedes	Aedes aegypti	SPECIES	3.91000	12.53000	1264886137
7218	Diptera	Culicidae	Aedes	Aedes aegypti	SPECIES	3.92404	-53.23477	4050447583
7219	Diptera	Culicidae	Aedes	Aedes aegypti	SPECIES	3.98000	13.17000	1264886107
7220	Diptera	Culicidae	Aedes	Aedes aegypti	SPECIES	3.98000	-73.76000	1264890488
7221	Diptera	Culicidae	Aedes	Aedes aegypti	SPECIES	3.98000	101.03000	1264890507
7222	Diptera	Culicidae	Aedes	Aedes aegypti	SPECIES	4.02000	9.19000	1264889992
7223	Diptera	Culicidae	Aedes	Aedes aegypti	SPECIES	4.05000	9.70000	1264886154
7224	Diptera	Culicidae	Aedes	Aedes aegypti	SPECIES	4.05000	9.71000	1264889952
7225	Diptera	Culicidae	Aedes	Aedes aegypti	SPECIES	4.07000	9.37000	1264889975
7226	Diptera	Culicidae	Aedes	Aedes aegypti	SPECIES	4.08000	-76.20000	1264891321
7227	Diptera	Culicidae	Aedes	Aedes aegypti	SPECIES	4.09000	-72.95000	1264887379
7228	Diptera	Culicidae	Aedes	Aedes aegypti	SPECIES	4.09000	-72.96000	1264887412
7229	Diptera	Culicidae	Aedes	Aedes aegypti	SPECIES	4.09000	-76.21000	1264889348
7230	Diptera	Culicidae	Aedes	Aedes aegypti	SPECIES	4.15000	-73.64000	1264887370
7231	Diptera	Culicidae	Aedes	Aedes aegypti	SPECIES	4.15000	-73.63000	1264891199
7232	Diptera	Culicidae	Aedes	Aedes aegypti	SPECIES	4.16000	9.23000	1264889920
7233	Diptera	Culicidae	Aedes	Aedes aegypti	SPECIES	4.17000	-60.97000	1264876345
7234	Diptera	Culicidae	Aedes	Aedes aegypti	SPECIES	4.17000	9.23000	1264886131
7235	Diptera	Culicidae	Aedes	Aedes aegypti	SPECIES	4.17000	-60.84000	1264890935
7236	Diptera	Culicidae	Aedes	Aedes aegypti	SPECIES	4.17000	73.50000	1264890969
7237	Diptera	Culicidae	Aedes	Aedes aegypti	SPECIES	4.21000	-74.65000	1264890417
7238	Diptera	Culicidae	Aedes	Aedes aegypti	SPECIES	4.23000	96.91000	1264891044
7239	Diptera	Culicidae	Aedes	Aedes aegypti	SPECIES	4.25000	117.88000	1264889184
7240	Diptera	Culicidae	Aedes	Aedes aegypti	SPECIES	4.26000	-54.37000	1264888218
7241	Diptera	Culicidae	Aedes	Aedes aegypti	SPECIES	4.30000	-74.80000	1264887377
7242	Diptera	Culicidae	Aedes	Aedes aegypti	SPECIES	4.30000	-74.81000	1264890483
7243	Diptera	Culicidae	Aedes	Aedes aegypti	SPECIES	4.31000	-52.14000	1264888195
7244	Diptera	Culicidae	Aedes	Aedes aegypti	SPECIES	4.37000	18.55000	1264886076
7245	Diptera	Culicidae	Aedes	Aedes aegypti	SPECIES	4.38000	18.55000	1264889269
7246	Diptera	Culicidae	Aedes	Aedes aegypti	SPECIES	4.40000	113.98000	1264889182
7247	Diptera	Culicidae	Aedes	Aedes aegypti	SPECIES	4.41000	-73.95000	1264891291
7248	Diptera	Culicidae	Aedes	Aedes aegypti	SPECIES	4.43000	14.36000	1264889973
7249	Diptera	Culicidae	Aedes	Aedes aegypti	SPECIES	4.47000	118.60000	1264889172
7250	Diptera	Culicidae	Aedes	Aedes aegypti	SPECIES	4.50000	9.57000	1264889964
7251	Diptera	Culicidae	Aedes	Aedes aegypti	SPECIES	4.53000	-75.71000	1264887240
7252	Diptera	Culicidae	Aedes	Aedes aegypti	SPECIES	4.53000	-75.70000	1264887280
7253	Diptera	Culicidae	Aedes	Aedes aegypti	SPECIES	4.57000	-52.47000	1264888617
7254	Diptera	Culicidae	Aedes	Aedes aegypti	SPECIES	4.58000	13.68000	1264889943
7255	Diptera	Culicidae	Aedes	Aedes aegypti	SPECIES	4.63000	-74.47000	1264887384
7256	Diptera	Culicidae	Aedes	Aedes aegypti	SPECIES	4.63000	-74.09000	1264888871
7257	Diptera	Culicidae	Aedes	Aedes aegypti	SPECIES	4.64000	-74.47000	1264887353

№	Order	Family	Genus	Species	Taxon Rank	Latitude	Longitude	GBIF_ID
7258	Diptera	Culicidae	Aedes	Aedes aegypti	SPECIES	4.64000	9.44000	1264889839
7259	Diptera	Culicidae	Aedes	Aedes aegypti	SPECIES	4.72000	-60.20000	1264876368
7260	Diptera	Culicidae	Aedes	Aedes aegypti	SPECIES	4.75000	11.22000	1264886167
7261	Diptera	Culicidae	Aedes	Aedes aegypti	SPECIES	4.75000	-75.91000	1264887223
7262	Diptera	Culicidae	Aedes	Aedes aegypti	SPECIES	4.78000	101.11000	1264891047
7263	Diptera	Culicidae	Aedes	Aedes aegypti	SPECIES	4.78010	-52.43459	177059755
7264	Diptera	Culicidae	Aedes	Aedes aegypti	SPECIES	4.80000	101.06000	1264891369
7265	Diptera	Culicidae	Aedes	Aedes aegypti	SPECIES	4.81145	-75.70383	3112811028
7266	Diptera	Culicidae	Aedes	Aedes aegypti	SPECIES	4.83000	-52.45000	1264888532
7267	Diptera	Culicidae	Aedes	Aedes aegypti	SPECIES	4.83000	-52.33000	3817924310
7268	Diptera	Culicidae	Aedes	Aedes aegypti	SPECIES	4.83010	-52.30125	177060075
7269	Diptera	Culicidae	Aedes	Aedes aegypti	SPECIES	4.83010	-52.33458	177060565
7270	Diptera	Culicidae	Aedes	Aedes aegypti	SPECIES	4.83400	-52.32500	2249160105
7271	Diptera	Culicidae	Aedes	Aedes aegypti	SPECIES	4.83409	-52.32488	1095884752
7272	Diptera	Culicidae	Aedes	Aedes aegypti	SPECIES	4.85000	-52.33000	1264888600
7273	Diptera	Culicidae	Aedes	Aedes aegypti	SPECIES	4.86000	-52.27000	1264888610
7274	Diptera	Culicidae	Aedes	Aedes aegypti	SPECIES	4.88000	-52.28000	1264888633
7275	Diptera	Culicidae	Aedes	Aedes aegypti	SPECIES	4.89677	-52.33458	177060566
7276	Diptera	Culicidae	Aedes	Aedes aegypti	SPECIES	4.90000	-52.50000	1264888506
7277	Diptera	Culicidae	Aedes	Aedes aegypti	SPECIES	4.91000	-52.28000	1264888623
7278	Diptera	Culicidae	Aedes	Aedes aegypti	SPECIES	4.91000	103.04000	1264891351
7279	Diptera	Culicidae	Aedes	Aedes aegypti	SPECIES	4.92000	-52.33000	1264886815
7280	Diptera	Culicidae	Aedes	Aedes aegypti	SPECIES	4.92000	-52.37000	1264888178
7281	Diptera	Culicidae	Aedes	Aedes aegypti	SPECIES	4.93000	-52.33000	1264891175
7282	Diptera	Culicidae	Aedes	Aedes aegypti	SPECIES	4.93011	-52.33458	177060041
7283	Diptera	Culicidae	Aedes	Aedes aegypti	SPECIES	4.94000	-52.33000	1264888603
7284	Diptera	Culicidae	Aedes	Aedes aegypti	SPECIES	4.94677	-52.31792	177059757
7285	Diptera	Culicidae	Aedes	Aedes aegypti	SPECIES	5.00000	118.00000	1264889233
7286	Diptera	Culicidae	Aedes	Aedes aegypti	SPECIES	5.00000	101.00000	1264891146
7287	Diptera	Culicidae	Aedes	Aedes aegypti	SPECIES	5.02000	-52.48000	1264888504
7288	Diptera	Culicidae	Aedes	Aedes aegypti	SPECIES	5.02000	97.17000	1264891012
7289	Diptera	Culicidae	Aedes	Aedes aegypti	SPECIES	5.05000	21.20000	1264885975
7290	Diptera	Culicidae	Aedes	Aedes aegypti	SPECIES	5.07000	7.80000	1264889709
7291	Diptera	Culicidae	Aedes	Aedes aegypti	SPECIES	5.13000	18.48000	1264889659
7292	Diptera	Culicidae	Aedes	Aedes aegypti	SPECIES	5.14000	10.52000	1264886189
7293	Diptera	Culicidae	Aedes	Aedes aegypti	SPECIES	5.14000	97.06000	1264891002
7294	Diptera	Culicidae	Aedes	Aedes aegypti	SPECIES	5.15000	-64.83000	1264887331
7295	Diptera	Culicidae	Aedes	Aedes aegypti	SPECIES	5.15000	-54.34000	1264888207
7296	Diptera	Culicidae	Aedes	Aedes aegypti	SPECIES	5.15000	-52.65000	1264888209
7297	Diptera	Culicidae	Aedes	Aedes aegypti	SPECIES	5.15900	-52.63700	2249159789
7298	Diptera	Culicidae	Aedes	Aedes aegypti	SPECIES	5.15907	-52.63712	1095883873

№	Order	Family	Genus	Species	Taxon Rank	Latitude	Longitude	GBIF_ID
7299	Diptera	Culicidae	Aedes	Aedes aegypti	SPECIES	5.16000	-54.34000	1264888596
7300	Diptera	Culicidae	Aedes	Aedes aegypti	SPECIES	5.16000	-52.65000	1264888646
7301	Diptera	Culicidae	Aedes	Aedes aegypti	SPECIES	5.16183	-52.64225	1095884656
7302	Diptera	Culicidae	Aedes	Aedes aegypti	SPECIES	5.16200	-52.64200	2249159082
7303	Diptera	Culicidae	Aedes	Aedes aegypti	SPECIES	5.16900	-52.65300	2249160361
7304	Diptera	Culicidae	Aedes	Aedes aegypti	SPECIES	5.16934	-52.65344	1095883951
7305	Diptera	Culicidae	Aedes	Aedes aegypti	SPECIES	5.17000	-52.65000	3818339246
7306	Diptera	Culicidae	Aedes	Aedes aegypti	SPECIES	5.19000	97.14000	1264876548
7307	Diptera	Culicidae	Aedes	Aedes aegypti	SPECIES	5.22000	115.60000	1264889189
7308	Diptera	Culicidae	Aedes	Aedes aegypti	SPECIES	5.25000	-4.00000	1264886949
7309	Diptera	Culicidae	Aedes	Aedes aegypti	SPECIES	5.25000	-3.97000	1264889735
7310	Diptera	Culicidae	Aedes	Aedes aegypti	SPECIES	5.27000	17.65000	1264885998
7311	Diptera	Culicidae	Aedes	Aedes aegypti	SPECIES	5.27000	-4.01000	1264886063
7312	Diptera	Culicidae	Aedes	Aedes aegypti	SPECIES	5.28000	115.25000	1264889192
7313	Diptera	Culicidae	Aedes	Aedes aegypti	SPECIES	5.28000	102.03000	1264891039
7314	Diptera	Culicidae	Aedes	Aedes aegypti	SPECIES	5.29000	-4.01000	1264886950
7315	Diptera	Culicidae	Aedes	Aedes aegypti	SPECIES	5.29000	100.29000	1264887346
7316	Diptera	Culicidae	Aedes	Aedes aegypti	SPECIES	5.29000	100.26000	1264887350
7317	Diptera	Culicidae	Aedes	Aedes aegypti	SPECIES	5.30000	103.14000	1264887635
7318	Diptera	Culicidae	Aedes	Aedes aegypti	SPECIES	5.30000	9.90000	1264889953
7319	Diptera	Culicidae	Aedes	Aedes aegypti	SPECIES	5.32000	-4.02000	1264886928
7320	Diptera	Culicidae	Aedes	Aedes aegypti	SPECIES	5.32000	100.29000	1264889123
7321	Diptera	Culicidae	Aedes	Aedes aegypti	SPECIES	5.32000	-4.04000	1264889748
7322	Diptera	Culicidae	Aedes	Aedes aegypti	SPECIES	5.33000	103.12000	1264891137
7323	Diptera	Culicidae	Aedes	Aedes aegypti	SPECIES	5.34000	95.96000	1264871783
7324	Diptera	Culicidae	Aedes	Aedes aegypti	SPECIES	5.34000	100.30000	1264889116
7325	Diptera	Culicidae	Aedes	Aedes aegypti	SPECIES	5.34000	-4.02000	1264891430
7326	Diptera	Culicidae	Aedes	Aedes aegypti	SPECIES	5.35000	96.01000	1264871794
7327	Diptera	Culicidae	Aedes	Aedes aegypti	SPECIES	5.35000	95.96000	1264871801
7328	Diptera	Culicidae	Aedes	Aedes aegypti	SPECIES	5.35000	100.30000	1264886067
7329	Diptera	Culicidae	Aedes	Aedes aegypti	SPECIES	5.35000	100.31000	1264887324
7330	Diptera	Culicidae	Aedes	Aedes aegypti	SPECIES	5.35000	117.13000	1264891056
7331	Diptera	Culicidae	Aedes	Aedes aegypti	SPECIES	5.35000	100.45000	1264891363
7332	Diptera	Culicidae	Aedes	Aedes aegypti	SPECIES	5.37000	100.31000	1264887344
7333	Diptera	Culicidae	Aedes	Aedes aegypti	SPECIES	5.37000	-52.95000	1264888635
7334	Diptera	Culicidae	Aedes	Aedes aegypti	SPECIES	5.37000	115.23000	1264889171
7335	Diptera	Culicidae	Aedes	Aedes aegypti	SPECIES	5.40000	-54.08000	1264888594
7336	Diptera	Culicidae	Aedes	Aedes aegypti	SPECIES	5.40000	100.28000	1264889110
7337	Diptera	Culicidae	Aedes	Aedes aegypti	SPECIES	5.40000	100.38000	1264889137
7338	Diptera	Culicidae	Aedes	Aedes aegypti	SPECIES	5.41000	100.31000	1264886123
7339	Diptera	Culicidae	Aedes	Aedes aegypti	SPECIES	5.42000	100.34000	1264889879

№	Order	Family	Genus	Species	Taxon Rank	Latitude	Longitude	GBIF_ID
7340	Diptera	Culicidae	Aedes	Aedes aegypti	SPECIES	5.44684	-55.20135	177060039
7341	Diptera	Culicidae	Aedes	Aedes aegypti	SPECIES	5.45000	-74.67000	1264887247
7342	Diptera	Culicidae	Aedes	Aedes aegypti	SPECIES	5.45000	119.13000	1264889187
7343	Diptera	Culicidae	Aedes	Aedes aegypti	SPECIES	5.45000	10.05000	1264889922
7344	Diptera	Culicidae	Aedes	Aedes aegypti	SPECIES	5.46000	100.31000	1264890332
7345	Diptera	Culicidae	Aedes	Aedes aegypti	SPECIES	5.47000	10.42000	1264886179
7346	Diptera	Culicidae	Aedes	Aedes aegypti	SPECIES	5.48000	-53.21000	1264888611
7347	Diptera	Culicidae	Aedes	Aedes aegypti	SPECIES	5.49000	10.41000	1264889962
7348	Diptera	Culicidae	Aedes	Aedes aegypti	SPECIES	5.50000	-54.08000	1264890179
7349	Diptera	Culicidae	Aedes	Aedes aegypti	SPECIES	5.50000	-54.03000	1264891269
7350	Diptera	Culicidae	Aedes	Aedes aegypti	SPECIES	5.52000	95.33000	1264876541
7351	Diptera	Culicidae	Aedes	Aedes aegypti	SPECIES	5.52231	102.94123	3993826212
7352	Diptera	Culicidae	Aedes	Aedes aegypti	SPECIES	5.53000	7.48000	1264889535
7353	Diptera	Culicidae	Aedes	Aedes aegypti	SPECIES	5.54000	95.34000	1264891017
7354	Diptera	Culicidae	Aedes	Aedes aegypti	SPECIES	5.55000	95.32000	1264890696
7355	Diptera	Culicidae	Aedes	Aedes aegypti	SPECIES	5.56000	-0.20000	1264889557
7356	Diptera	Culicidae	Aedes	Aedes aegypti	SPECIES	5.56000	100.37000	1264889892
7357	Diptera	Culicidae	Aedes	Aedes aegypti	SPECIES	5.57000	95.32000	1264876583
7358	Diptera	Culicidae	Aedes	Aedes aegypti	SPECIES	5.57000	115.60000	1264889208
7359	Diptera	Culicidae	Aedes	Aedes aegypti	SPECIES	5.60000	-53.82000	1264888524
7360	Diptera	Culicidae	Aedes	Aedes aegypti	SPECIES	5.64000	18.91000	1264886024
7361	Diptera	Culicidae	Aedes	Aedes aegypti	SPECIES	5.67000	-53.78000	1264888642
7362	Diptera	Culicidae	Aedes	Aedes aegypti	SPECIES	5.69000	-76.65000	1264886723
7363	Diptera	Culicidae	Aedes	Aedes aegypti	SPECIES	5.78000	7.03000	1264887886
7364	Diptera	Culicidae	Aedes	Aedes aegypti	SPECIES	5.78000	118.15000	1264889222
7365	Diptera	Culicidae	Aedes	Aedes aegypti	SPECIES	5.79423	-55.47799	3901425084
7366	Diptera	Culicidae	Aedes	Aedes aegypti	SPECIES	5.82000	6.93000	1264887878
7367	Diptera	Culicidae	Aedes	Aedes aegypti	SPECIES	5.82729	-55.14533	1946350178
7368	Diptera	Culicidae	Aedes	Aedes aegypti	SPECIES	5.82762	-55.14645	1948746965
7369	Diptera	Culicidae	Aedes	Aedes aegypti	SPECIES	5.83000	-55.17000	1264890291
7370	Diptera	Culicidae	Aedes	Aedes aegypti	SPECIES	5.83000	-5.66000	1264891429
7371	Diptera	Culicidae	Aedes	Aedes aegypti	SPECIES	5.83000	-5.36000	1264891431
7372	Diptera	Culicidae	Aedes	Aedes aegypti	SPECIES	5.84000	118.11000	1264889202
7373	Diptera	Culicidae	Aedes	Aedes aegypti	SPECIES	5.86000	-7.45000	1264891471
7374	Diptera	Culicidae	Aedes	Aedes aegypti	SPECIES	5.87000	-74.65000	1264887406
7375	Diptera	Culicidae	Aedes	Aedes aegypti	SPECIES	5.87000	-74.64000	1264891143
7376	Diptera	Culicidae	Aedes	Aedes aegypti	SPECIES	5.88000	-4.82000	1264889867
7377	Diptera	Culicidae	Aedes	Aedes aegypti	SPECIES	5.88000	-10.04000	1264890654
7378	Diptera	Culicidae	Aedes	Aedes aegypti	SPECIES	5.89000	-74.64000	1264889882
7379	Diptera	Culicidae	Aedes	Aedes aegypti	SPECIES	5.89000	14.55000	1264889982
7380	Diptera	Culicidae	Aedes	Aedes aegypti	SPECIES	5.96000	10.16000	1264886192

№	Order	Family	Genus	Species	Taxon Rank	Latitude	Longitude	GBIF_ID
7381	Diptera	Culicidae	Aedes	Aedes aegypti	SPECIES	5.96000	10.15000	1264889983
7382	Diptera	Culicidae	Aedes	Aedes aegypti	SPECIES	5.97000	116.07000	1264887608
7383	Diptera	Culicidae	Aedes	Aedes aegypti	SPECIES	6.01000	1.05000	1264888323
7384	Diptera	Culicidae	Aedes	Aedes aegypti	SPECIES	6.02000	80.21000	3348917207
7385	Diptera	Culicidae	Aedes	Aedes aegypti	SPECIES	6.03000	80.21000	3969132375
7386	Diptera	Culicidae	Aedes	Aedes aegypti	SPECIES	6.03082	101.63772	4145854377
7387	Diptera	Culicidae	Aedes	Aedes aegypti	SPECIES	6.05000	80.21000	1264891215
7388	Diptera	Culicidae	Aedes	Aedes aegypti	SPECIES	6.07610	101.66531	4145857182
7389	Diptera	Culicidae	Aedes	Aedes aegypti	SPECIES	6.09000	11.49000	1264889961
7390	Diptera	Culicidae	Aedes	Aedes aegypti	SPECIES	6.12000	102.26000	1264887632
7391	Diptera	Culicidae	Aedes	Aedes aegypti	SPECIES	6.14000	81.12000	3348854419
7392	Diptera	Culicidae	Aedes	Aedes aegypti	SPECIES	6.15689	-75.62450	2992713800
7393	Diptera	Culicidae	Aedes	Aedes aegypti	SPECIES	6.15689	-75.62436	3760345705
7394	Diptera	Culicidae	Aedes	Aedes aegypti	SPECIES	6.22000	-75.57000	1264887306
7395	Diptera	Culicidae	Aedes	Aedes aegypti	SPECIES	6.22000	6.22000	1264890362
7396	Diptera	Culicidae	Aedes	Aedes aegypti	SPECIES	6.24000	-57.52000	1264888191
7397	Diptera	Culicidae	Aedes	Aedes aegypti	SPECIES	6.24199	-10.35318	3356884313
7398	Diptera	Culicidae	Aedes	Aedes aegypti	SPECIES	6.24279	-75.59602	2826360460
7399	Diptera	Culicidae	Aedes	Aedes aegypti	SPECIES	6.25000	-75.59000	1264887311
7400	Diptera	Culicidae	Aedes	Aedes aegypti	SPECIES	6.26000	-75.62000	1264887309
7401	Diptera	Culicidae	Aedes	Aedes aegypti	SPECIES	6.26070	-75.56741	2802538551
7402	Diptera	Culicidae	Aedes	Aedes aegypti	SPECIES	6.27000	-75.61000	1264886703
7403	Diptera	Culicidae	Aedes	Aedes aegypti	SPECIES	6.27000	81.28000	3348836469
7404	Diptera	Culicidae	Aedes	Aedes aegypti	SPECIES	6.30000	0.05000	1264890247
7405	Diptera	Culicidae	Aedes	Aedes aegypti	SPECIES	6.30000	6.38000	1264890360
7406	Diptera	Culicidae	Aedes	Aedes aegypti	SPECIES	6.31000	-5.93000	1264891450
7407	Diptera	Culicidae	Aedes	Aedes aegypti	SPECIES	6.31097	101.90482	4145857535
7408	Diptera	Culicidae	Aedes	Aedes aegypti	SPECIES	6.32000	0.09000	1264889838
7409	Diptera	Culicidae	Aedes	Aedes aegypti	SPECIES	6.34000	-75.56000	1264886342
7410	Diptera	Culicidae	Aedes	Aedes aegypti	SPECIES	6.34000	5.63000	1264890198
7411	Diptera	Culicidae	Aedes	Aedes aegypti	SPECIES	6.36000	2.44000	1264889830
7412	Diptera	Culicidae	Aedes	Aedes aegypti	SPECIES	6.41329	2.29259	2556241698
7413	Diptera	Culicidae	Aedes	Aedes aegypti	SPECIES	6.45000	7.49000	1264890255
7414	Diptera	Culicidae	Aedes	Aedes aegypti	SPECIES	6.47000	12.62000	1264889966
7415	Diptera	Culicidae	Aedes	Aedes aegypti	SPECIES	6.47000	7.42000	1264890251
7416	Diptera	Culicidae	Aedes	Aedes aegypti	SPECIES	6.48000	2.63000	1264890284
7417	Diptera	Culicidae	Aedes	Aedes aegypti	SPECIES	6.48000	-6.28000	1264891439
7418	Diptera	Culicidae	Aedes	Aedes aegypti	SPECIES	6.49000	-58.27000	1264888172
7419	Diptera	Culicidae	Aedes	Aedes aegypti	SPECIES	6.49000	3.37000	1264888285
7420	Diptera	Culicidae	Aedes	Aedes aegypti	SPECIES	6.50000	7.42000	1264890240
7421	Diptera	Culicidae	Aedes	Aedes aegypti	SPECIES	6.50335	2.13731	2556241752

№	Order	Family	Genus	Species	Taxon Rank	Latitude	Longitude	GBIF_ID
7422	Diptera	Culicidae	Aedes	Aedes aegypti	SPECIES	6.51000	3.36000	1264888426
7423	Diptera	Culicidae	Aedes	Aedes aegypti	SPECIES	6.52000	14.29000	1264886133
7424	Diptera	Culicidae	Aedes	Aedes aegypti	SPECIES	6.52000	100.42000	1264888057
7425	Diptera	Culicidae	Aedes	Aedes aegypti	SPECIES	6.52000	3.40000	1264888408
7426	Diptera	Culicidae	Aedes	Aedes aegypti	SPECIES	6.53000	-58.25000	1264888136
7427	Diptera	Culicidae	Aedes	Aedes aegypti	SPECIES	6.53000	3.39000	1264888365
7428	Diptera	Culicidae	Aedes	Aedes aegypti	SPECIES	6.54000	-7.48000	1264891433
7429	Diptera	Culicidae	Aedes	Aedes aegypti	SPECIES	6.55000	-74.33000	1264886661
7430	Diptera	Culicidae	Aedes	Aedes aegypti	SPECIES	6.55000	-57.75000	1264888156
7431	Diptera	Culicidae	Aedes	Aedes aegypti	SPECIES	6.55000	7.37000	1264890254
7432	Diptera	Culicidae	Aedes	Aedes aegypti	SPECIES	6.55000	-5.25000	1264891464
7433	Diptera	Culicidae	Aedes	Aedes aegypti	SPECIES	6.59000	3.35000	1264888971
7434	Diptera	Culicidae	Aedes	Aedes aegypti	SPECIES	6.61172	100.06294	4145855512
7435	Diptera	Culicidae	Aedes	Aedes aegypti	SPECIES	6.61172	100.06293	4145850819
7436	Diptera	Culicidae	Aedes	Aedes aegypti	SPECIES	6.62000	100.05000	1264887560
7437	Diptera	Culicidae	Aedes	Aedes aegypti	SPECIES	6.65000	117.48000	1264889178
7438	Diptera	Culicidae	Aedes	Aedes aegypti	SPECIES	6.66000	-7.25000	1264891443
7439	Diptera	Culicidae	Aedes	Aedes aegypti	SPECIES	6.69000	-1.62000	1264887870
7440	Diptera	Culicidae	Aedes	Aedes aegypti	SPECIES	6.70503	-1.68885	2579330179
7441	Diptera	Culicidae	Aedes	Aedes aegypti	SPECIES	6.72000	-1.58000	1264887834
7442	Diptera	Culicidae	Aedes	Aedes aegypti	SPECIES	6.72000	-1.47000	1264887866
7443	Diptera	Culicidae	Aedes	Aedes aegypti	SPECIES	6.72000	-61.64000	1264888834
7444	Diptera	Culicidae	Aedes	Aedes aegypti	SPECIES	6.73000	-1.73000	1264887836
7445	Diptera	Culicidae	Aedes	Aedes aegypti	SPECIES	6.73380	3.03254	226099056
7446	Diptera	Culicidae	Aedes	Aedes aegypti	SPECIES	6.75000	100.40000	1264886500
7447	Diptera	Culicidae	Aedes	Aedes aegypti	SPECIES	6.77000	-75.08000	1264887690
7448	Diptera	Culicidae	Aedes	Aedes aegypti	SPECIES	6.77742	100.07219	4145853703
7449	Diptera	Culicidae	Aedes	Aedes aegypti	SPECIES	6.78801	100.07882	4145858404
7450	Diptera	Culicidae	Aedes	Aedes aegypti	SPECIES	6.79000	-58.15000	1264888151
7451	Diptera	Culicidae	Aedes	Aedes aegypti	SPECIES	6.79961	-58.16706	177060559
7452	Diptera	Culicidae	Aedes	Aedes aegypti	SPECIES	6.80000	-58.18000	1264888125
7453	Diptera	Culicidae	Aedes	Aedes aegypti	SPECIES	6.80000	-58.16000	1264889746
7454	Diptera	Culicidae	Aedes	Aedes aegypti	SPECIES	6.81000	-58.16000	1264890226
7455	Diptera	Culicidae	Aedes	Aedes aegypti	SPECIES	6.82000	-58.13000	1264888166
7456	Diptera	Culicidae	Aedes	Aedes aegypti	SPECIES	6.82000	-58.23000	1264888169
7457	Diptera	Culicidae	Aedes	Aedes aegypti	SPECIES	6.84000	100.66000	1264887487
7458	Diptera	Culicidae	Aedes	Aedes aegypti	SPECIES	6.87000	-75.18000	1264887678
7459	Diptera	Culicidae	Aedes	Aedes aegypti	SPECIES	6.87000	3.72000	1264887858
7460	Diptera	Culicidae	Aedes	Aedes aegypti	SPECIES	6.87000	-6.45000	1264891463
7461	Diptera	Culicidae	Aedes	Aedes aegypti	SPECIES	6.89000	81.34000	3348738275
7462	Diptera	Culicidae	Aedes	Aedes aegypti	SPECIES	6.90000	100.53000	1264886501

№	Order	Family	Genus	Species	Taxon Rank	Latitude	Longitude	GBIF_ID
7463	Diptera	Culicidae	Aedes	Aedes aegypti	SPECIES	6.90000	116.84000	1264889179
7464	Diptera	Culicidae	Aedes	Aedes aegypti	SPECIES	6.90000	79.87000	3348238115
7465	Diptera	Culicidae	Aedes	Aedes aegypti	SPECIES	6.90000	79.86000	3968873821
7466	Diptera	Culicidae	Aedes	Aedes aegypti	SPECIES	6.91000	122.07000	1264891286
7467	Diptera	Culicidae	Aedes	Aedes aegypti	SPECIES	6.91000	-8.20000	1264891447
7468	Diptera	Culicidae	Aedes	Aedes aegypti	SPECIES	6.92000	-58.41000	1264888159
7469	Diptera	Culicidae	Aedes	Aedes aegypti	SPECIES	6.92000	79.86000	1264889850
7470	Diptera	Culicidae	Aedes	Aedes aegypti	SPECIES	6.92000	79.87000	3969101991
7471	Diptera	Culicidae	Aedes	Aedes aegypti	SPECIES	6.93000	79.84000	1264891229
7472	Diptera	Culicidae	Aedes	Aedes aegypti	SPECIES	6.94000	79.87000	3969100811
7473	Diptera	Culicidae	Aedes	Aedes aegypti	SPECIES	6.99000	-58.43000	1264888164
7474	Diptera	Culicidae	Aedes	Aedes aegypti	SPECIES	7.00000	100.45000	1264886474
7475	Diptera	Culicidae	Aedes	Aedes aegypti	SPECIES	7.00000	-58.50000	1264888160
7476	Diptera	Culicidae	Aedes	Aedes aegypti	SPECIES	7.01000	100.47000	1264887482
7477	Diptera	Culicidae	Aedes	Aedes aegypti	SPECIES	7.01000	100.04000	1264888933
7478	Diptera	Culicidae	Aedes	Aedes aegypti	SPECIES	7.01000	100.08000	1264889677
7479	Diptera	Culicidae	Aedes	Aedes aegypti	SPECIES	7.02000	100.06000	1264888961
7480	Diptera	Culicidae	Aedes	Aedes aegypti	SPECIES	7.02000	125.09000	1264889422
7481	Diptera	Culicidae	Aedes	Aedes aegypti	SPECIES	7.03000	100.05000	1264888956
7482	Diptera	Culicidae	Aedes	Aedes aegypti	SPECIES	7.03000	100.07000	1264888963
7483	Diptera	Culicidae	Aedes	Aedes aegypti	SPECIES	7.07000	100.41000	1264887478
7484	Diptera	Culicidae	Aedes	Aedes aegypti	SPECIES	7.07000	80.01000	1264887604
7485	Diptera	Culicidae	Aedes	Aedes aegypti	SPECIES	7.07000	3.75000	1264887887
7486	Diptera	Culicidae	Aedes	Aedes aegypti	SPECIES	7.07000	3.91000	1264890244
7487	Diptera	Culicidae	Aedes	Aedes aegypti	SPECIES	7.08856	125.58044	2963844191
7488	Diptera	Culicidae	Aedes	Aedes aegypti	SPECIES	7.09000	-4.00000	1264891466
7489	Diptera	Culicidae	Aedes	Aedes aegypti	SPECIES	7.10000	-58.49000	1264888154
7490	Diptera	Culicidae	Aedes	Aedes aegypti	SPECIES	7.11000	-73.12000	1264887366
7491	Diptera	Culicidae	Aedes	Aedes aegypti	SPECIES	7.12000	171.37000	1264890075
7492	Diptera	Culicidae	Aedes	Aedes aegypti	SPECIES	7.12000	171.06000	1264890077
7493	Diptera	Culicidae	Aedes	Aedes aegypti	SPECIES	7.12000	-68.78000	1264890966
7494	Diptera	Culicidae	Aedes	Aedes aegypti	SPECIES	7.13000	-73.13000	1264887398
7495	Diptera	Culicidae	Aedes	Aedes aegypti	SPECIES	7.14000	122.14000	1264891067
7496	Diptera	Culicidae	Aedes	Aedes aegypti	SPECIES	7.14050	-73.12165	3399112394
7497	Diptera	Culicidae	Aedes	Aedes aegypti	SPECIES	7.15000	3.36000	1264886169
7498	Diptera	Culicidae	Aedes	Aedes aegypti	SPECIES	7.16000	3.35000	1264890624
7499	Diptera	Culicidae	Aedes	Aedes aegypti	SPECIES	7.17000	80.12000	1264886249
7500	Diptera	Culicidae	Aedes	Aedes aegypti	SPECIES	7.18000	100.61000	1264887489
7501	Diptera	Culicidae	Aedes	Aedes aegypti	SPECIES	7.19000	-3.62000	1264891426
7502	Diptera	Culicidae	Aedes	Aedes aegypti	SPECIES	7.20000	100.60000	1264887577
7503	Diptera	Culicidae	Aedes	Aedes aegypti	SPECIES	7.21500	2.31400	3416659717

№	Order	Family	Genus	Species	Taxon Rank	Latitude	Longitude	GBIF_ID
7504	Diptera	Culicidae	Aedes	Aedes aegypti	SPECIES	7.25000	80.60000	1264886339
7505	Diptera	Culicidae	Aedes	Aedes aegypti	SPECIES	7.25000	100.51000	1264887492
7506	Diptera	Culicidae	Aedes	Aedes aegypti	SPECIES	7.27000	100.15000	1264886488
7507	Diptera	Culicidae	Aedes	Aedes aegypti	SPECIES	7.27000	-61.50000	1264888836
7508	Diptera	Culicidae	Aedes	Aedes aegypti	SPECIES	7.29000	80.63000	1264891207
7509	Diptera	Culicidae	Aedes	Aedes aegypti	SPECIES	7.30245	125.66853	2981047153
7510	Diptera	Culicidae	Aedes	Aedes aegypti	SPECIES	7.32000	13.58000	1264886194
7511	Diptera	Culicidae	Aedes	Aedes aegypti	SPECIES	7.33000	13.58000	1264889980
7512	Diptera	Culicidae	Aedes	Aedes aegypti	SPECIES	7.35000	-11.70000	1264889842
7513	Diptera	Culicidae	Aedes	Aedes aegypti	SPECIES	7.36000	-61.81000	1264888839
7514	Diptera	Culicidae	Aedes	Aedes aegypti	SPECIES	7.40000	-73.50000	1264887399
7515	Diptera	Culicidae	Aedes	Aedes aegypti	SPECIES	7.41000	3.90000	1264890932
7516	Diptera	Culicidae	Aedes	Aedes aegypti	SPECIES	7.43000	-11.83000	1264889803
7517	Diptera	Culicidae	Aedes	Aedes aegypti	SPECIES	7.43000	-80.33000	3351070631
7518	Diptera	Culicidae	Aedes	Aedes aegypti	SUBSPECIES	7.43373	3.89922	226099060
7519	Diptera	Culicidae	Aedes	Aedes aegypti	SPECIES	7.45000	-58.60000	1264888152
7520	Diptera	Culicidae	Aedes	Aedes aegypti	SPECIES	7.45000	-4.34000	1264891474
7521	Diptera	Culicidae	Aedes	Aedes aegypti	SPECIES	7.47000	80.38000	1264886284
7522	Diptera	Culicidae	Aedes	Aedes aegypti	SPECIES	7.47000	-73.21000	1264886421
7523	Diptera	Culicidae	Aedes	Aedes aegypti	SPECIES	7.47000	-73.20000	1264886436
7524	Diptera	Culicidae	Aedes	Aedes aegypti	SPECIES	7.48000	-73.21000	1264886416
7525	Diptera	Culicidae	Aedes	Aedes aegypti	SPECIES	7.48000	80.36000	1264891205
7526	Diptera	Culicidae	Aedes	Aedes aegypti	SPECIES	7.50000	100.05000	1264889694
7527	Diptera	Culicidae	Aedes	Aedes aegypti	SPECIES	7.54181	99.62228	4145857303
7528	Diptera	Culicidae	Aedes	Aedes aegypti	SPECIES	7.54967	99.49425	4145849763
7529	Diptera	Culicidae	Aedes	Aedes aegypti	SPECIES	7.57000	99.35000	1264886493
7530	Diptera	Culicidae	Aedes	Aedes aegypti	SPECIES	7.58000	79.80000	1264886293
7531	Diptera	Culicidae	Aedes	Aedes aegypti	SPECIES	7.59724	100.11642	4145852642
7532	Diptera	Culicidae	Aedes	Aedes aegypti	SPECIES	7.60004	99.30886	4145860209
7533	Diptera	Culicidae	Aedes	Aedes aegypti	SPECIES	7.63000	100.39000	1264887668
7534	Diptera	Culicidae	Aedes	Aedes aegypti	SPECIES	7.66000	-4.20000	1264891453
7535	Diptera	Culicidae	Aedes	Aedes aegypti	SPECIES	7.66000	-4.23000	1264891482
7536	Diptera	Culicidae	Aedes	Aedes aegypti	SPECIES	7.67000	-10.97000	1264891476
7537	Diptera	Culicidae	Aedes	Aedes aegypti	SPECIES	7.67483	99.46921	4145854675
7538	Diptera	Culicidae	Aedes	Aedes aegypti	SPECIES	7.68000	81.73000	1264887515
7539	Diptera	Culicidae	Aedes	Aedes aegypti	SPECIES	7.68000	-4.20000	1264891469
7540	Diptera	Culicidae	Aedes	Aedes aegypti	SPECIES	7.68000	-4.21000	1264891483
7541	Diptera	Culicidae	Aedes	Aedes aegypti	SPECIES	7.69000	-5.03000	1264889527
7542	Diptera	Culicidae	Aedes	Aedes aegypti	SPECIES	7.70000	-4.22000	1264891472
7543	Diptera	Culicidae	Aedes	Aedes aegypti	SPECIES	7.71000	81.70000	1264891221
7544	Diptera	Culicidae	Aedes	Aedes aegypti	SPECIES	7.71000	-11.93000	1264891454

№	Order	Family	Genus	Species	Taxon Rank	Latitude	Longitude	GBIF_ID
7545	Diptera	Culicidae	Aedes	Aedes aegypti	SPECIES	7.73000	81.70000	1264886079
7546	Diptera	Culicidae	Aedes	Aedes aegypti	SPECIES	7.73000	-4.26000	1264891455
7547	Diptera	Culicidae	Aedes	Aedes aegypti	SPECIES	7.73539	100.04875	4145851951
7548	Diptera	Culicidae	Aedes	Aedes aegypti	SPECIES	7.74000	-4.25000	1264891434
7549	Diptera	Culicidae	Aedes	Aedes aegypti	SPECIES	7.76000	-4.25000	1264891460
7550	Diptera	Culicidae	Aedes	Aedes aegypti	SPECIES	7.76000	-80.27000	3348346778
7551	Diptera	Culicidae	Aedes	Aedes aegypti	SPECIES	7.77000	4.57000	1264886042
7552	Diptera	Culicidae	Aedes	Aedes aegypti	SPECIES	7.77000	81.61000	1264886093
7553	Diptera	Culicidae	Aedes	Aedes aegypti	SPECIES	7.77000	-12.08000	1264889776
7554	Diptera	Culicidae	Aedes	Aedes aegypti	SPECIES	7.77000	-4.24000	1264891485
7555	Diptera	Culicidae	Aedes	Aedes aegypti	SPECIES	7.80037	6.73259	226099058
7556	Diptera	Culicidae	Aedes	Aedes aegypti	SPECIES	7.83000	81.56000	1264887542
7557	Diptera	Culicidae	Aedes	Aedes aegypti	SPECIES	7.83000	-80.75000	1264891138
7558	Diptera	Culicidae	Aedes	Aedes aegypti	SPECIES	7.85000	-4.44000	1264891428
7559	Diptera	Culicidae	Aedes	Aedes aegypti	SPECIES	7.86000	13.60000	1264886190
7560	Diptera	Culicidae	Aedes	Aedes aegypti	SPECIES	7.87000	100.30000	1264886483
7561	Diptera	Culicidae	Aedes	Aedes aegypti	SPECIES	7.87994	-72.49520	2963863452
7562	Diptera	Culicidae	Aedes	Aedes aegypti	SPECIES	7.88000	98.38000	1264890460
7563	Diptera	Culicidae	Aedes	Aedes aegypti	SPECIES	7.89000	-67.47000	1264887594
7564	Diptera	Culicidae	Aedes	Aedes aegypti	SPECIES	7.89000	-4.52000	1264891427
7565	Diptera	Culicidae	Aedes	Aedes aegypti	SPECIES	7.90000	-4.49000	1264891442
7566	Diptera	Culicidae	Aedes	Aedes aegypti	SPECIES	7.92000	-72.44000	1264886365
7567	Diptera	Culicidae	Aedes	Aedes aegypti	SPECIES	7.92085	99.85594	4145853571
7568	Diptera	Culicidae	Aedes	Aedes aegypti	SPECIES	7.93000	-80.41000	1264891161
7569	Diptera	Culicidae	Aedes	Aedes aegypti	SPECIES	7.98000	-10.68000	1264891462
7570	Diptera	Culicidae	Aedes	Aedes aegypti	SPECIES	8.01000	-4.58000	1264891445
7571	Diptera	Culicidae	Aedes	Aedes aegypti	SPECIES	8.04000	80.94000	3348932378
7572	Diptera	Culicidae	Aedes	Aedes aegypti	SPECIES	8.07000	77.54000	3348175526
7573	Diptera	Culicidae	Aedes	Aedes aegypti	SPECIES	8.08000	4.18000	1264891389
7574	Diptera	Culicidae	Aedes	Aedes aegypti	SPECIES	8.10000	-76.73000	1264886709
7575	Diptera	Culicidae	Aedes	Aedes aegypti	SPECIES	8.10000	-72.25000	1264887565
7576	Diptera	Culicidae	Aedes	Aedes aegypti	SPECIES	8.12000	-63.55000	1264887570
7577	Diptera	Culicidae	Aedes	Aedes aegypti	SPECIES	8.12000	4.25000	1264888387
7578	Diptera	Culicidae	Aedes	Aedes aegypti	SPECIES	8.13000	3.63000	1264891408
7579	Diptera	Culicidae	Aedes	Aedes aegypti	SPECIES	8.14000	-69.86000	1264890967
7580	Diptera	Culicidae	Aedes	Aedes aegypti	SPECIES	8.15572	99.83236	4145852728
7581	Diptera	Culicidae	Aedes	Aedes aegypti	SPECIES	8.16058	99.68039	4145860468
7582	Diptera	Culicidae	Aedes	Aedes aegypti	SPECIES	8.17306	99.69222	4145853942
7583	Diptera	Culicidae	Aedes	Aedes aegypti	SPECIES	8.18000	-4.98000	1264889722
7584	Diptera	Culicidae	Aedes	Aedes aegypti	SPECIES	8.20000	-59.78000	1264889727
7585	Diptera	Culicidae	Aedes	Aedes aegypti	SPECIES	8.21000	-80.95000	3351187731

№	Order	Family	Genus	Species	Taxon Rank	Latitude	Longitude	GBIF_ID
7586	Diptera	Culicidae	Aedes	Aedes aegypti	SPECIES	8.24831	98.39315	4145852951
7587	Diptera	Culicidae	Aedes	Aedes aegypti	SPECIES	8.25000	13.82000	1264886117
7588	Diptera	Culicidae	Aedes	Aedes aegypti	SPECIES	8.26000	-4.58000	1264891459
7589	Diptera	Culicidae	Aedes	Aedes aegypti	SPECIES	8.29000	76.56000	3349930333
7590	Diptera	Culicidae	Aedes	Aedes aegypti	SPECIES	8.30081	98.42031	4145853849
7591	Diptera	Culicidae	Aedes	Aedes aegypti	SPECIES	8.33000	-13.07000	1264889815
7592	Diptera	Culicidae	Aedes	Aedes aegypti	SPECIES	8.34000	-10.38000	1264889813
7593	Diptera	Culicidae	Aedes	Aedes aegypti	SPECIES	8.37000	98.43000	1264887002
7594	Diptera	Culicidae	Aedes	Aedes aegypti	SPECIES	8.38030	-82.41817	3415951911
7595	Diptera	Culicidae	Aedes	Aedes aegypti	SPECIES	8.40000	-79.70000	3350924909
7596	Diptera	Culicidae	Aedes	Aedes aegypti	SPECIES	8.41647	99.94808	4145852895
7597	Diptera	Culicidae	Aedes	Aedes aegypti	SPECIES	8.42000	-13.27000	1264889792
7598	Diptera	Culicidae	Aedes	Aedes aegypti	SPECIES	8.43000	99.96000	1264887508
7599	Diptera	Culicidae	Aedes	Aedes aegypti	SPECIES	8.43000	-82.43000	1264891192
7600	Diptera	Culicidae	Aedes	Aedes aegypti	SPECIES	8.44000	98.52000	1264886867
7601	Diptera	Culicidae	Aedes	Aedes aegypti	SPECIES	8.44000	77.73000	3348214668
7602	Diptera	Culicidae	Aedes	Aedes aegypti	SPECIES	8.44111	99.95833	4145850374
7603	Diptera	Culicidae	Aedes	Aedes aegypti	SPECIES	8.44278	99.95972	4145858435
7604	Diptera	Culicidae	Aedes	Aedes aegypti	SPECIES	8.45000	98.54000	1264890431
7605	Diptera	Culicidae	Aedes	Aedes aegypti	SPECIES	8.45000	-4.51000	1264891436
7606	Diptera	Culicidae	Aedes	Aedes aegypti	SPECIES	8.45000	-11.70000	1264891468
7607	Diptera	Culicidae	Aedes	Aedes aegypti	SPECIES	8.47000	-13.26000	1264889826
7608	Diptera	Culicidae	Aedes	Aedes aegypti	SPECIES	8.48000	-13.24000	1264889812
7609	Diptera	Culicidae	Aedes	Aedes aegypti	SPECIES	8.48000	76.92000	1264889957
7610	Diptera	Culicidae	Aedes	Aedes aegypti	SPECIES	8.48000	78.18000	3350923599
7611	Diptera	Culicidae	Aedes	Aedes aegypti	SPECIES	8.49000	-13.23000	1264891461
7612	Diptera	Culicidae	Aedes	Aedes aegypti	SPECIES	8.49000	-77.97000	3348230022
7613	Diptera	Culicidae	Aedes	Aedes aegypti	SPECIES	8.49680	-82.84323	226099203
7614	Diptera	Culicidae	Aedes	Aedes aegypti	SPECIES	8.52000	76.92000	1264890737
7615	Diptera	Culicidae	Aedes	Aedes aegypti	SPECIES	8.52000	-82.79000	3351058877
7616	Diptera	Culicidae	Aedes	Aedes aegypti	SPECIES	8.53794	-82.84360	226099202
7617	Diptera	Culicidae	Aedes	Aedes aegypti	SPECIES	8.54000	-70.99000	1264888843
7618	Diptera	Culicidae	Aedes	Aedes aegypti	SPECIES	8.55000	-71.24000	1264886850
7619	Diptera	Culicidae	Aedes	Aedes aegypti	SPECIES	8.55000	99.28000	1264891079
7620	Diptera	Culicidae	Aedes	Aedes aegypti	SPECIES	8.56690	98.54532	4145851944
7621	Diptera	Culicidae	Aedes	Aedes aegypti	SPECIES	8.56889	98.22750	4145851938
7622	Diptera	Culicidae	Aedes	Aedes aegypti	SPECIES	8.58000	98.31000	1264888082
7623	Diptera	Culicidae	Aedes	Aedes aegypti	SPECIES	8.58330	98.66616	4145855372
7624	Diptera	Culicidae	Aedes	Aedes aegypti	SPECIES	8.60000	-71.15000	1264886837
7625	Diptera	Culicidae	Aedes	Aedes aegypti	SPECIES	8.61000	-71.66000	1264886832
7626	Diptera	Culicidae	Aedes	Aedes aegypti	SPECIES	8.64398	-82.95738	226099201

№	Order	Family	Genus	Species	Taxon Rank	Latitude	Longitude	GBIF_ID
7627	Diptera	Culicidae	Aedes	Aedes aegypti	SPECIES	8.64931	-83.17862	226099204
7628	Diptera	Culicidae	Aedes	Aedes aegypti	SPECIES	8.67988	-83.06817	226099205
7629	Diptera	Culicidae	Aedes	Aedes aegypti	SPECIES	8.70000	-13.07000	1264889809
7630	Diptera	Culicidae	Aedes	Aedes aegypti	SPECIES	8.70440	-83.13356	226099183
7631	Diptera	Culicidae	Aedes	Aedes aegypti	SPECIES	8.72000	-11.95000	1264889831
7632	Diptera	Culicidae	Aedes	Aedes aegypti	SPECIES	8.76000	-75.88000	1264889462
7633	Diptera	Culicidae	Aedes	Aedes aegypti	SPECIES	8.76000	-11.83000	1264891456
7634	Diptera	Culicidae	Aedes	Aedes aegypti	SPECIES	8.77000	99.15000	1264887497
7635	Diptera	Culicidae	Aedes	Aedes aegypti	SPECIES	8.78000	105.00000	1264886247
7636	Diptera	Culicidae	Aedes	Aedes aegypti	SPECIES	8.86000	98.34000	1264890433
7637	Diptera	Culicidae	Aedes	Aedes aegypti	SPECIES	8.88000	-79.63000	1264889613
7638	Diptera	Culicidae	Aedes	Aedes aegypti	SPECIES	8.88028	-79.78333	3356891315
7639	Diptera	Culicidae	Aedes	Aedes aegypti	SPECIES	8.88052	-83.36242	226099206
7640	Diptera	Culicidae	Aedes	Aedes aegypti	SPECIES	8.90000	-79.85000	1264891235
7641	Diptera	Culicidae	Aedes	Aedes aegypti	SPECIES	8.90000	-79.70000	3351011900
7642	Diptera	Culicidae	Aedes	Aedes aegypti	SPECIES	8.92000	99.30000	1264887536
7643	Diptera	Culicidae	Aedes	Aedes aegypti	SPECIES	8.93000	-67.44000	1264888818
7644	Diptera	Culicidae	Aedes	Aedes aegypti	SPECIES	8.93439	-79.70817	3330416506
7645	Diptera	Culicidae	Aedes	Aedes aegypti	SPECIES	8.94000	105.01000	1264886155
7646	Diptera	Culicidae	Aedes	Aedes aegypti	SPECIES	8.95000	-79.53000	1264888423
7647	Diptera	Culicidae	Aedes	Aedes aegypti	SPECIES	8.95000	-79.55000	1264888436
7648	Diptera	Culicidae	Aedes	Aedes aegypti	SPECIES	8.95000	-79.54000	1264889638
7649	Diptera	Culicidae	Aedes	Aedes aegypti	SPECIES	8.96000	-79.54000	1264888421
7650	Diptera	Culicidae	Aedes	Aedes aegypti	SPECIES	8.96000	-79.53000	1264891264
7651	Diptera	Culicidae	Aedes	Aedes aegypti	SPECIES	8.96000	-79.67000	3348211105
7652	Diptera	Culicidae	Aedes	Aedes aegypti	SPECIES	8.96797	-79.53313	225864841
7653	Diptera	Culicidae	Aedes	Aedes aegypti	SPECIES	8.97000	-79.54000	1264888439
7654	Diptera	Culicidae	Aedes	Aedes aegypti	SPECIES	8.97000	-79.55000	1264888457
7655	Diptera	Culicidae	Aedes	Aedes aegypti	SPECIES	8.97802	-79.51947	2814145470
7656	Diptera	Culicidae	Aedes	Aedes aegypti	SPECIES	8.98000	-79.53000	1264888445
7657	Diptera	Culicidae	Aedes	Aedes aegypti	SPECIES	8.98000	-79.50000	1264889655
7658	Diptera	Culicidae	Aedes	Aedes aegypti	SPECIES	8.98000	-79.55000	1264889660
7659	Diptera	Culicidae	Aedes	Aedes aegypti	SPECIES	8.99000	-79.51000	1264888437
7660	Diptera	Culicidae	Aedes	Aedes aegypti	SPECIES	8.99000	104.98000	1264891224
7661	Diptera	Culicidae	Aedes	Aedes aegypti	SPECIES	8.99436	-79.52522	4046391601
7662	Diptera	Culicidae	Aedes	Aedes aegypti	SPECIES	8.99575	-79.53949	2236841332
7663	Diptera	Culicidae	Aedes	Aedes aegypti	SPECIES	9.00000	-79.52000	1264888417
7664	Diptera	Culicidae	Aedes	Aedes aegypti	SPECIES	9.00381	-79.57526	3356902311
7665	Diptera	Culicidae	Aedes	Aedes aegypti	SPECIES	9.01296	-79.53014	3772668601
7666	Diptera	Culicidae	Aedes	Aedes aegypti	SPECIES	9.02000	-79.51000	1264888389
7667	Diptera	Culicidae	Aedes	Aedes aegypti	SPECIES	9.02000	-79.49000	1264888447

№	Order	Family	Genus	Species	Taxon Rank	Latitude	Longitude	GBIF_ID
7668	Diptera	Culicidae	Aedes	Aedes aegypti	SPECIES	9.02000	-79.50000	1264889630
7669	Diptera	Culicidae	Aedes	Aedes aegypti	SPECIES	9.02000	-79.48000	1264889657
7670	Diptera	Culicidae	Aedes	Aedes aegypti	SPECIES	9.02000	-79.52000	1264889673
7671	Diptera	Culicidae	Aedes	Aedes aegypti	SPECIES	9.03000	99.37000	1264886498
7672	Diptera	Culicidae	Aedes	Aedes aegypti	SPECIES	9.03000	-79.49000	1264890690
7673	Diptera	Culicidae	Aedes	Aedes aegypti	SPECIES	9.03000	-79.50000	1264891152
7674	Diptera	Culicidae	Aedes	Aedes aegypti	SPECIES	9.03535	-79.52781	3385102003
7675	Diptera	Culicidae	Aedes	Aedes aegypti	SPECIES	9.04000	-69.75000	1264887564
7676	Diptera	Culicidae	Aedes	Aedes aegypti	SPECIES	9.04000	-79.44000	1264888444
7677	Diptera	Culicidae	Aedes	Aedes aegypti	SPECIES	9.05000	-76.00000	1264889481
7678	Diptera	Culicidae	Aedes	Aedes aegypti	SPECIES	9.05000	-79.45000	1264889678
7679	Diptera	Culicidae	Aedes	Aedes aegypti	SPECIES	9.05000	104.83000	1264891043
7680	Diptera	Culicidae	Aedes	Aedes aegypti	SPECIES	9.07000	-79.43000	1264889665
7681	Diptera	Culicidae	Aedes	Aedes aegypti	SPECIES	9.07000	-1.81000	1264890788
7682	Diptera	Culicidae	Aedes	Aedes aegypti	SPECIES	9.07000	-79.50000	3351031975
7683	Diptera	Culicidae	Aedes	Aedes aegypti	SPECIES	9.08000	-79.38000	1264891209
7684	Diptera	Culicidae	Aedes	Aedes aegypti	SPECIES	9.08000	-79.29000	3348211742
7685	Diptera	Culicidae	Aedes	Aedes aegypti	SPECIES	9.08208	-79.29063	3416015882
7686	Diptera	Culicidae	Aedes	Aedes aegypti	SPECIES	9.09906	99.23500	4145857203
7687	Diptera	Culicidae	Aedes	Aedes aegypti	SPECIES	9.10000	-79.36000	3348163861
7688	Diptera	Culicidae	Aedes	Aedes aegypti	SPECIES	9.11328	99.23194	4145849455
7689	Diptera	Culicidae	Aedes	Aedes aegypti	SPECIES	9.12000	-79.55000	1264888434
7690	Diptera	Culicidae	Aedes	Aedes aegypti	SPECIES	9.13000	104.92000	1264891059
7691	Diptera	Culicidae	Aedes	Aedes aegypti	SPECIES	9.14000	99.32000	1264887518
7692	Diptera	Culicidae	Aedes	Aedes aegypti	SPECIES	9.14000	-71.09000	1264888880
7693	Diptera	Culicidae	Aedes	Aedes aegypti	SPECIES	9.15000	-83.73000	1264891049
7694	Diptera	Culicidae	Aedes	Aedes aegypti	SPECIES	9.15167	99.38861	4145856454
7695	Diptera	Culicidae	Aedes	Aedes aegypti	SPECIES	9.16000	105.22000	1264886204
7696	Diptera	Culicidae	Aedes	Aedes aegypti	SPECIES	9.16378	98.26826	4145849705
7697	Diptera	Culicidae	Aedes	Aedes aegypti	SPECIES	9.16600	-1.16600	1100304666
7698	Diptera	Culicidae	Aedes	Aedes aegypti	SPECIES	9.17000	-79.56000	1264888448
7699	Diptera	Culicidae	Aedes	Aedes aegypti	SPECIES	9.17000	105.15000	1264888891
7700	Diptera	Culicidae	Aedes	Aedes aegypti	SPECIES	9.17000	105.19000	1264891075
7701	Diptera	Culicidae	Aedes	Aedes aegypti	SPECIES	9.17203	99.37803	4145853945
7702	Diptera	Culicidae	Aedes	Aedes aegypti	SPECIES	9.18000	-71.22000	1264888827
7703	Diptera	Culicidae	Aedes	Aedes aegypti	SPECIES	9.21000	99.70000	1264887526
7704	Diptera	Culicidae	Aedes	Aedes aegypti	SPECIES	9.22000	78.52000	3348074644
7705	Diptera	Culicidae	Aedes	Aedes aegypti	SPECIES	9.23000	105.45000	1264888926
7706	Diptera	Culicidae	Aedes	Aedes aegypti	SPECIES	9.24000	105.48000	1264886149
7707	Diptera	Culicidae	Aedes	Aedes aegypti	SPECIES	9.24000	-71.04000	1264887587
7708	Diptera	Culicidae	Aedes	Aedes aegypti	SPECIES	9.25583	-83.86813	226099200

№	Order	Family	Genus	Species	Taxon Rank	Latitude	Longitude	GBIF_ID
7709	Diptera	Culicidae	Aedes	Aedes aegypti	SPECIES	9.26000	-9.01000	1264891420
7710	Diptera	Culicidae	Aedes	Aedes aegypti	SPECIES	9.28000	-70.62000	1264888842
7711	Diptera	Culicidae	Aedes	Aedes aegypti	SPECIES	9.28000	105.73000	1264888898
7712	Diptera	Culicidae	Aedes	Aedes aegypti	SPECIES	9.28522	-83.85495	226099199
7713	Diptera	Culicidae	Aedes	Aedes aegypti	SPECIES	9.30000	99.68000	1264888296
7714	Diptera	Culicidae	Aedes	Aedes aegypti	SPECIES	9.30000	13.40000	1264889954
7715	Diptera	Culicidae	Aedes	Aedes aegypti	SPECIES	9.31000	123.31000	1264886358
7716	Diptera	Culicidae	Aedes	Aedes aegypti	SPECIES	9.32000	-70.59000	1264886295
7717	Diptera	Culicidae	Aedes	Aedes aegypti	SPECIES	9.32000	-70.61000	1264887625
7718	Diptera	Culicidae	Aedes	Aedes aegypti	SPECIES	9.33000	-12.30000	1264891457
7719	Diptera	Culicidae	Aedes	Aedes aegypti	SPECIES	9.33099	98.40778	4145849149
7720	Diptera	Culicidae	Aedes	Aedes aegypti	SPECIES	9.34000	-79.86000	3348321035
7721	Diptera	Culicidae	Aedes	Aedes aegypti	SPECIES	9.35000	-70.57000	1264886302
7722	Diptera	Culicidae	Aedes	Aedes aegypti	SPECIES	9.35000	77.57000	3348206588
7723	Diptera	Culicidae	Aedes	Aedes aegypti	SPECIES	9.36000	-70.44000	1264890566
7724	Diptera	Culicidae	Aedes	Aedes aegypti	SPECIES	9.36000	-79.79000	3350943802
7725	Diptera	Culicidae	Aedes	Aedes aegypti	SPECIES	9.37000	-70.43000	1264890545
7726	Diptera	Culicidae	Aedes	Aedes aegypti	SPECIES	9.37000	105.14000	1264891045
7727	Diptera	Culicidae	Aedes	Aedes aegypti	SPECIES	9.37000	104.95000	1264891069
7728	Diptera	Culicidae	Aedes	Aedes aegypti	SPECIES	9.38000	-70.43000	1264890554
7729	Diptera	Culicidae	Aedes	Aedes aegypti	SPECIES	9.39000	-70.59000	1264886318
7730	Diptera	Culicidae	Aedes	Aedes aegypti	SPECIES	9.39000	-70.43000	1264887484
7731	Diptera	Culicidae	Aedes	Aedes aegypti	SPECIES	9.39000	-63.02000	1264890985
7732	Diptera	Culicidae	Aedes	Aedes aegypti	SPECIES	9.40000	-70.56000	1264887619
7733	Diptera	Culicidae	Aedes	Aedes aegypti	SPECIES	9.40000	13.51000	1264890473
7734	Diptera	Culicidae	Aedes	Aedes aegypti	SPECIES	9.41000	-70.49000	1264886309
7735	Diptera	Culicidae	Aedes	Aedes aegypti	SPECIES	9.42000	-70.42000	1264886315
7736	Diptera	Culicidae	Aedes	Aedes aegypti	SPECIES	9.42000	100.00000	1264891104
7737	Diptera	Culicidae	Aedes	Aedes aegypti	SPECIES	9.42257	99.93687	226099238
7738	Diptera	Culicidae	Aedes	Aedes aegypti	SPECIES	9.43000	100.01000	1264888799
7739	Diptera	Culicidae	Aedes	Aedes aegypti	SPECIES	9.43000	-70.45000	1264890550
7740	Diptera	Culicidae	Aedes	Aedes aegypti	SPECIES	9.45000	-70.47000	1264886310
7741	Diptera	Culicidae	Aedes	Aedes aegypti	SPECIES	9.45000	-70.46000	1264886323
7742	Diptera	Culicidae	Aedes	Aedes aegypti	SPECIES	9.45000	99.03000	1264887533
7743	Diptera	Culicidae	Aedes	Aedes aegypti	SPECIES	9.45000	99.94000	1264888771
7744	Diptera	Culicidae	Aedes	Aedes aegypti	SPECIES	9.45000	100.03000	1264891127
7745	Diptera	Culicidae	Aedes	Aedes aegypti	SPECIES	9.45178	98.49281	4145858066
7746	Diptera	Culicidae	Aedes	Aedes aegypti	SPECIES	9.46000	99.99000	1264890139
7747	Diptera	Culicidae	Aedes	Aedes aegypti	SPECIES	9.47000	99.95000	1264888298
7748	Diptera	Culicidae	Aedes	Aedes aegypti	SPECIES	9.47000	100.06000	1264888315
7749	Diptera	Culicidae	Aedes	Aedes aegypti	SPECIES	9.48000	99.97000	1264888753

№	Order	Family	Genus	Species	Taxon Rank	Latitude	Longitude	GBIF_ID
7750	Diptera	Culicidae	Aedes	Aedes aegypti	SPECIES	9.49000	76.33000	1264890521
7751	Diptera	Culicidae	Aedes	Aedes aegypti	SPECIES	9.49478	99.98626	226099236
7752	Diptera	Culicidae	Aedes	Aedes aegypti	SPECIES	9.50000	-13.72000	1264888142
7753	Diptera	Culicidae	Aedes	Aedes aegypti	SPECIES	9.50000	99.93000	1264888756
7754	Diptera	Culicidae	Aedes	Aedes aegypti	SPECIES	9.50000	100.01000	1264891111
7755	Diptera	Culicidae	Aedes	Aedes aegypti	SPECIES	9.51000	-13.72000	1264889482
7756	Diptera	Culicidae	Aedes	Aedes aegypti	SPECIES	9.52000	-11.37000	1264889801
7757	Diptera	Culicidae	Aedes	Aedes aegypti	SPECIES	9.52109	99.95809	226099239
7758	Diptera	Culicidae	Aedes	Aedes aegypti	SPECIES	9.53000	104.85000	1264888855
7759	Diptera	Culicidae	Aedes	Aedes aegypti	SPECIES	9.53000	-11.36000	1264891465
7760	Diptera	Culicidae	Aedes	Aedes aegypti	SPECIES	9.54000	-70.46000	1264886319
7761	Diptera	Culicidae	Aedes	Aedes aegypti	SPECIES	9.54000	99.95000	1264890172
7762	Diptera	Culicidae	Aedes	Aedes aegypti	SPECIES	9.54719	-13.65297	3873206878
7763	Diptera	Culicidae	Aedes	Aedes aegypti	SPECIES	9.56000	99.94000	1264888300
7764	Diptera	Culicidae	Aedes	Aedes aegypti	SPECIES	9.57000	100.00000	1264888758
7765	Diptera	Culicidae	Aedes	Aedes aegypti	SPECIES	9.58000	99.98000	1264888297
7766	Diptera	Culicidae	Aedes	Aedes aegypti	SPECIES	9.58000	77.96000	1264890918
7767	Diptera	Culicidae	Aedes	Aedes aegypti	SPECIES	9.58000	105.92000	1264891266
7768	Diptera	Culicidae	Aedes	Aedes aegypti	SPECIES	9.58000	78.10000	3348094340
7769	Diptera	Culicidae	Aedes	Aedes aegypti	SPECIES	9.59000	76.52000	1264890535
7770	Diptera	Culicidae	Aedes	Aedes aegypti	SPECIES	9.60000	105.97000	1264888885
7771	Diptera	Culicidae	Aedes	Aedes aegypti	SPECIES	9.61000	-83.99000	1264891078
7772	Diptera	Culicidae	Aedes	Aedes aegypti	SPECIES	9.62519	-82.83294	226099177
7773	Diptera	Culicidae	Aedes	Aedes aegypti	SPECIES	9.62980	-82.87210	226099176
7774	Diptera	Culicidae	Aedes	Aedes aegypti	SPECIES	9.65000	80.03000	1264889731
7775	Diptera	Culicidae	Aedes	Aedes aegypti	SPECIES	9.65471	99.67137	226099224
7776	Diptera	Culicidae	Aedes	Aedes aegypti	SPECIES	9.66000	105.93000	1264886243
7777	Diptera	Culicidae	Aedes	Aedes aegypti	SPECIES	9.66000	-66.83000	1264887291
7778	Diptera	Culicidae	Aedes	Aedes aegypti	SPECIES	9.67000	80.03000	1264886049
7779	Diptera	Culicidae	Aedes	Aedes aegypti	SPECIES	9.67000	80.02000	1264890087
7780	Diptera	Culicidae	Aedes	Aedes aegypti	SPECIES	9.68000	80.03000	1264886286
7781	Diptera	Culicidae	Aedes	Aedes aegypti	SPECIES	9.68000	-68.57000	1264888829
7782	Diptera	Culicidae	Aedes	Aedes aegypti	SPECIES	9.68000	80.02000	1264889717
7783	Diptera	Culicidae	Aedes	Aedes aegypti	SPECIES	9.68000	-11.73000	1264891473
7784	Diptera	Culicidae	Aedes	Aedes aegypti	SPECIES	9.69000	80.01000	1264889686
7785	Diptera	Culicidae	Aedes	Aedes aegypti	SPECIES	9.69000	-11.72000	1264889819
7786	Diptera	Culicidae	Aedes	Aedes aegypti	SPECIES	9.71000	78.81000	3348043913
7787	Diptera	Culicidae	Aedes	Aedes aegypti	SPECIES	9.73000	118.73000	1264889451
7788	Diptera	Culicidae	Aedes	Aedes aegypti	SPECIES	9.74177	-82.84317	226099208
7789	Diptera	Culicidae	Aedes	Aedes aegypti	SPECIES	9.75000	105.00000	1264888875
7790	Diptera	Culicidae	Aedes	Aedes aegypti	SPECIES	9.75000	80.02000	1264889739

№	Order	Family	Genus	Species	Taxon Rank	Latitude	Longitude	GBIF_ID
7791	Diptera	Culicidae	Aedes	Aedes aegypti	SPECIES	9.80000	106.45000	1264888909
7792	Diptera	Culicidae	Aedes	Aedes aegypti	SPECIES	9.81000	105.82000	1264886199
7793	Diptera	Culicidae	Aedes	Aedes aegypti	SPECIES	9.82000	-7.38000	1264886975
7794	Diptera	Culicidae	Aedes	Aedes aegypti	SPECIES	9.82425	-84.93441	226099188
7795	Diptera	Culicidae	Aedes	Aedes aegypti	SPECIES	9.86000	105.67000	1264887445
7796	Diptera	Culicidae	Aedes	Aedes aegypti	SPECIES	9.86000	-12.63000	1264891413
7797	Diptera	Culicidae	Aedes	Aedes aegypti	SPECIES	9.88000	78.05000	1264889589
7798	Diptera	Culicidae	Aedes	Aedes aegypti	SPECIES	9.89000	-66.88000	1264891204
7799	Diptera	Culicidae	Aedes	Aedes aegypti	SPECIES	9.91000	78.14000	1264889568
7800	Diptera	Culicidae	Aedes	Aedes aegypti	SPECIES	9.91000	78.12000	1264889605
7801	Diptera	Culicidae	Aedes	Aedes aegypti	SPECIES	9.91224	98.62705	4145858441
7802	Diptera	Culicidae	Aedes	Aedes aegypti	SPECIES	9.92000	105.32000	1264888887
7803	Diptera	Culicidae	Aedes	Aedes aegypti	SPECIES	9.93000	-67.34000	1264888833
7804	Diptera	Culicidae	Aedes	Aedes aegypti	SPECIES	9.93000	106.34000	1264888896
7805	Diptera	Culicidae	Aedes	Aedes aegypti	SPECIES	9.93000	8.89000	1264888972
7806	Diptera	Culicidae	Aedes	Aedes aegypti	SPECIES	9.93000	78.13000	1264889611
7807	Diptera	Culicidae	Aedes	Aedes aegypti	SPECIES	9.94000	78.13000	1264889577
7808	Diptera	Culicidae	Aedes	Aedes aegypti	SPECIES	9.94801	98.59031	4145849869
7809	Diptera	Culicidae	Aedes	Aedes aegypti	SPECIES	9.95000	99.08000	1264886451
7810	Diptera	Culicidae	Aedes	Aedes aegypti	SPECIES	9.95000	-83.35000	1264891099
7811	Diptera	Culicidae	Aedes	Aedes aegypti	SPECIES	9.95439	98.63455	3455912400
7812	Diptera	Culicidae	Aedes	Aedes aegypti	SPECIES	9.96000	76.24000	1264891203
7813	Diptera	Culicidae	Aedes	Aedes aegypti	SPECIES	9.96394	-84.07239	226099181
7814	Diptera	Culicidae	Aedes	Aedes aegypti	SPECIES	9.96536	-84.15264	226099182
7815	Diptera	Culicidae	Aedes	Aedes aegypti	SPECIES	9.96581	-85.11343	226099189
7816	Diptera	Culicidae	Aedes	Aedes aegypti	SPECIES	9.97000	98.64000	1264887503
7817	Diptera	Culicidae	Aedes	Aedes aegypti	SPECIES	9.97000	76.28000	1264889967
7818	Diptera	Culicidae	Aedes	Aedes aegypti	SPECIES	9.97000	-84.84000	1264890369
7819	Diptera	Culicidae	Aedes	Aedes aegypti	SPECIES	9.97000	-67.10000	1264891000
7820	Diptera	Culicidae	Aedes	Aedes aegypti	SPECIES	9.97000	-84.83000	1264891232
7821	Diptera	Culicidae	Aedes	Aedes aegypti	SPECIES	9.97000	78.06000	3351574084
7822	Diptera	Culicidae	Aedes	Aedes aegypti	SPECIES	9.98000	-84.76000	1264886399
7823	Diptera	Culicidae	Aedes	Aedes aegypti	SPECIES	9.98000	-84.77000	1264886419
7824	Diptera	Culicidae	Aedes	Aedes aegypti	SPECIES	9.98000	-84.78000	1264886428
7825	Diptera	Culicidae	Aedes	Aedes aegypti	SPECIES	9.98000	-84.81000	1264886456
7826	Diptera	Culicidae	Aedes	Aedes aegypti	SPECIES	9.98175	-84.82481	226099187
7827	Diptera	Culicidae	Aedes	Aedes aegypti	SPECIES	9.98452	-84.66656	177060567
7828	Diptera	Culicidae	Aedes	Aedes aegypti	SPECIES	9.98942	76.57898	2814465410
7829	Diptera	Culicidae	Aedes	Aedes aegypti	SPECIES	9.99000	-84.77000	1264886376
7830	Diptera	Culicidae	Aedes	Aedes aegypti	SPECIES	9.99187	-83.03413	177060564
7831	Diptera	Culicidae	Aedes	Aedes aegypti	SPECIES	9.99608	-84.11933	226099207

№	Order	Family	Genus	Species	Taxon Rank	Latitude	Longitude	GBIF_ID
7832	Diptera	Culicidae	Aedes	Aedes aegypti	SPECIES	9.99666	-83.03441	226099178
7833	Diptera	Culicidae	Aedes	Aedes aegypti	SPECIES	9.99684	-85.25352	226099190
7834	Diptera	Culicidae	Aedes	Aedes aegypti	SPECIES	10.00000	105.09000	1264888903
7835	Diptera	Culicidae	Aedes	Aedes aegypti	SPECIES	10.00000	-83.25000	1264891313
7836	Diptera	Culicidae	Aedes	Aedes aegypti	SPECIES	10.00000	-61.00000	3328467140
7837	Diptera	Culicidae	Aedes	Aedes aegypti	SPECIES	10.00000	77.47000	3348154862
7838	Diptera	Culicidae	Aedes	Aedes aegypti	SPECIES	10.00187	-84.65017	226099186
7839	Diptera	Culicidae	Aedes	Aedes aegypti	SPECIES	10.00925	-84.26846	226099180
7840	Diptera	Culicidae	Aedes	Aedes aegypti	SPECIES	10.01000	77.50000	1264890910
7841	Diptera	Culicidae	Aedes	Aedes aegypti	SPECIES	10.01000	77.46000	3351562254
7842	Diptera	Culicidae	Aedes	Aedes aegypti	SPECIES	10.03000	105.79000	1264888892
7843	Diptera	Culicidae	Aedes	Aedes aegypti	SPECIES	10.03000	-10.73000	1264891424
7844	Diptera	Culicidae	Aedes	Aedes aegypti	SPECIES	10.03452	-83.26653	177060572
7845	Diptera	Culicidae	Aedes	Aedes aegypti	SPECIES	10.04000	-65.03000	1264887899
7846	Diptera	Culicidae	Aedes	Aedes aegypti	SPECIES	10.04000	-67.48000	1264888837
7847	Diptera	Culicidae	Aedes	Aedes aegypti	SPECIES	10.05000	-65.04000	1264887979
7848	Diptera	Culicidae	Aedes	Aedes aegypti	SPECIES	10.05646	-69.28896	3496608601
7849	Diptera	Culicidae	Aedes	Aedes aegypti	SPECIES	10.06000	-69.49000	1264886328
7850	Diptera	Culicidae	Aedes	Aedes aegypti	SPECIES	10.06000	-65.04000	1264888825
7851	Diptera	Culicidae	Aedes	Aedes aegypti	SPECIES	10.06000	-12.85000	1264891382
7852	Diptera	Culicidae	Aedes	Aedes aegypti	SPECIES	10.07000	73.64000	1264886211
7853	Diptera	Culicidae	Aedes	Aedes aegypti	SPECIES	10.07000	105.56000	1264887559
7854	Diptera	Culicidae	Aedes	Aedes aegypti	SPECIES	10.08000	-67.99000	1264891092
7855	Diptera	Culicidae	Aedes	Aedes aegypti	SPECIES	10.08452	-83.28320	177060574
7856	Diptera	Culicidae	Aedes	Aedes aegypti	SPECIES	10.08742	-84.47239	3456817455
7857	Diptera	Culicidae	Aedes	Aedes aegypti	SPECIES	10.09679	-83.51026	226099179
7858	Diptera	Culicidae	Aedes	Aedes aegypti	SPECIES	10.10000	106.43000	1264887545
7859	Diptera	Culicidae	Aedes	Aedes aegypti	SPECIES	10.10000	99.83000	1264888276
7860	Diptera	Culicidae	Aedes	Aedes aegypti	SPECIES	10.10000	-83.51000	1264890663
7861	Diptera	Culicidae	Aedes	Aedes aegypti	SPECIES	10.10181	-85.37205	226099191
7862	Diptera	Culicidae	Aedes	Aedes aegypti	SPECIES	10.11000	105.92000	1264887419
7863	Diptera	Culicidae	Aedes	Aedes aegypti	SPECIES	10.12000	106.45000	1264887439
7864	Diptera	Culicidae	Aedes	Aedes aegypti	SPECIES	10.13000	105.88000	1264887443
7865	Diptera	Culicidae	Aedes	Aedes aegypti	SPECIES	10.13000	-69.86000	1264891183
7866	Diptera	Culicidae	Aedes	Aedes aegypti	SPECIES	10.13000	-3.45000	1264891421
7867	Diptera	Culicidae	Aedes	Aedes aegypti	SPECIES	10.13700	-85.44812	226099192
7868	Diptera	Culicidae	Aedes	Aedes aegypti	SPECIES	10.14000	-65.44000	1264887957
7869	Diptera	Culicidae	Aedes	Aedes aegypti	SPECIES	10.14000	-64.68000	1264887994
7870	Diptera	Culicidae	Aedes	Aedes aegypti	SPECIES	10.14000	-61.51000	1264890598
7871	Diptera	Culicidae	Aedes	Aedes aegypti	SPECIES	10.14000	-61.58000	1264891317
7872	Diptera	Culicidae	Aedes	Aedes aegypti	SPECIES	10.15000	105.97000	1264887579

№	Order	Family	Genus	Species	Taxon Rank	Latitude	Longitude	GBIF_ID
7873	Diptera	Culicidae	Aedes	Aedes aegypti	SPECIES	10.15000	76.40000	1264889925
7874	Diptera	Culicidae	Aedes	Aedes aegypti	SPECIES	10.15000	-61.51000	1264890610
7875	Diptera	Culicidae	Aedes	Aedes aegypti	SPECIES	10.16000	105.91000	1264886684
7876	Diptera	Culicidae	Aedes	Aedes aegypti	SPECIES	10.16000	106.67000	1264887488
7877	Diptera	Culicidae	Aedes	Aedes aegypti	SPECIES	10.16000	-64.66000	1264888835
7878	Diptera	Culicidae	Aedes	Aedes aegypti	SPECIES	10.16000	-61.46000	1264891394
7879	Diptera	Culicidae	Aedes	Aedes aegypti	SPECIES	10.17000	106.65000	1264887480
7880	Diptera	Culicidae	Aedes	Aedes aegypti	SPECIES	10.17000	106.15000	1264887481
7881	Diptera	Culicidae	Aedes	Aedes aegypti	SPECIES	10.17000	-61.08000	1264888561
7882	Diptera	Culicidae	Aedes	Aedes aegypti	SPECIES	10.17000	-61.67000	1264888576
7883	Diptera	Culicidae	Aedes	Aedes aegypti	SPECIES	10.17000	-61.47000	1264888945
7884	Diptera	Culicidae	Aedes	Aedes aegypti	SPECIES	10.18000	-61.55000	1264888187
7885	Diptera	Culicidae	Aedes	Aedes aegypti	SPECIES	10.18000	-61.68000	1264889791
7886	Diptera	Culicidae	Aedes	Aedes aegypti	SPECIES	10.18000	-69.77000	1264890983
7887	Diptera	Culicidae	Aedes	Aedes aegypti	SPECIES	10.18018	-67.45188	177060126
7888	Diptera	Culicidae	Aedes	Aedes aegypti	SPECIES	10.19000	106.09000	1264887232
7889	Diptera	Culicidae	Aedes	Aedes aegypti	SPECIES	10.19000	-69.31000	1264887581
7890	Diptera	Culicidae	Aedes	Aedes aegypti	SPECIES	10.19000	-67.46000	1264888848
7891	Diptera	Culicidae	Aedes	Aedes aegypti	SPECIES	10.19000	-67.96000	1264890955
7892	Diptera	Culicidae	Aedes	Aedes aegypti	SPECIES	10.20000	-61.30000	1264891308
7893	Diptera	Culicidae	Aedes	Aedes aegypti	SPECIES	10.21510	-83.89603	226099185
7894	Diptera	Culicidae	Aedes	Aedes aegypti	SPECIES	10.21855	-83.79152	226099184
7895	Diptera	Culicidae	Aedes	Aedes aegypti	SPECIES	10.22000	-61.52000	1264886839
7896	Diptera	Culicidae	Aedes	Aedes aegypti	SPECIES	10.22000	106.58000	1264887210
7897	Diptera	Culicidae	Aedes	Aedes aegypti	SPECIES	10.22000	106.54000	1264887453
7898	Diptera	Culicidae	Aedes	Aedes aegypti	SPECIES	10.22000	78.00000	3350975627
7899	Diptera	Culicidae	Aedes	Aedes aegypti	SPECIES	10.22463	-67.48638	177060119
7900	Diptera	Culicidae	Aedes	Aedes aegypti	SPECIES	10.23000	-67.33000	1264888853
7901	Diptera	Culicidae	Aedes	Aedes aegypti	SPECIES	10.23000	78.52000	3350875858
7902	Diptera	Culicidae	Aedes	Aedes aegypti	SPECIES	10.24000	106.38000	1264888899
7903	Diptera	Culicidae	Aedes	Aedes aegypti	SPECIES	10.24000	-67.60000	1264889708
7904	Diptera	Culicidae	Aedes	Aedes aegypti	SPECIES	10.24000	-67.59000	1264891284
7905	Diptera	Culicidae	Aedes	Aedes aegypti	SPECIES	10.24685	-67.60189	225864845
7906	Diptera	Culicidae	Aedes	Aedes aegypti	SPECIES	10.25000	106.55000	1264887474
7907	Diptera	Culicidae	Aedes	Aedes aegypti	SPECIES	10.25000	-61.00000	1264888224
7908	Diptera	Culicidae	Aedes	Aedes aegypti	SPECIES	10.25000	105.47000	1264888881
7909	Diptera	Culicidae	Aedes	Aedes aegypti	SPECIES	10.25000	-67.60000	1264890259
7910	Diptera	Culicidae	Aedes	Aedes aegypti	SPECIES	10.25000	14.23000	1264890468
7911	Diptera	Culicidae	Aedes	Aedes aegypti	SPECIES	10.25176	-67.48625	177059964
7912	Diptera	Culicidae	Aedes	Aedes aegypti	SPECIES	10.26000	-67.59000	1264888832
7913	Diptera	Culicidae	Aedes	Aedes aegypti	SPECIES	10.26294	-85.58235	226099193

№	Order	Family	Genus	Species	Taxon Rank	Latitude	Longitude	GBIF_ID
7914	Diptera	Culicidae	Aedes	Aedes aegypti	SPECIES	10.27000	105.92000	1264886177
7915	Diptera	Culicidae	Aedes	Aedes aegypti	SPECIES	10.27000	-61.43000	1264890699
7916	Diptera	Culicidae	Aedes	Aedes aegypti	SPECIES	10.27000	-61.10000	1264891311
7917	Diptera	Culicidae	Aedes	Aedes aegypti	SPECIES	10.28000	-61.28000	1264888258
7918	Diptera	Culicidae	Aedes	Aedes aegypti	SPECIES	10.28000	-61.42000	1264888573
7919	Diptera	Culicidae	Aedes	Aedes aegypti	SPECIES	10.28000	-61.46000	1264890325
7920	Diptera	Culicidae	Aedes	Aedes aegypti	SPECIES	10.29000	-75.52000	1264886414
7921	Diptera	Culicidae	Aedes	Aedes aegypti	SPECIES	10.30000	-67.63000	1264887281
7922	Diptera	Culicidae	Aedes	Aedes aegypti	SPECIES	10.30000	-61.45000	1264888580
7923	Diptera	Culicidae	Aedes	Aedes aegypti	SPECIES	10.30594	-61.17556	4073374455
7924	Diptera	Culicidae	Aedes	Aedes aegypti	SPECIES	10.31000	-67.63000	1264888849
7925	Diptera	Culicidae	Aedes	Aedes aegypti	SPECIES	10.31000	123.90000	1264889818
7926	Diptera	Culicidae	Aedes	Aedes aegypti	SPECIES	10.31000	-66.28000	1264891250
7927	Diptera	Culicidae	Aedes	Aedes aegypti	SPECIES	10.31000	123.89000	1264891318
7928	Diptera	Culicidae	Aedes	Aedes aegypti	SPECIES	10.31352	-67.55189	177060136
7929	Diptera	Culicidae	Aedes	Aedes aegypti	SPECIES	10.33000	-67.03000	1264887580
7930	Diptera	Culicidae	Aedes	Aedes aegypti	SPECIES	10.33024	98.77457	4145850929
7931	Diptera	Culicidae	Aedes	Aedes aegypti	SPECIES	10.34000	107.08000	1264888884
7932	Diptera	Culicidae	Aedes	Aedes aegypti	SPECIES	10.34000	-67.04000	1264890045
7933	Diptera	Culicidae	Aedes	Aedes aegypti	SPECIES	10.35000	106.36000	1264889034
7934	Diptera	Culicidae	Aedes	Aedes aegypti	SPECIES	10.35000	-66.99000	1264889855
7935	Diptera	Culicidae	Aedes	Aedes aegypti	SPECIES	10.36000	77.98000	1264889015
7936	Diptera	Culicidae	Aedes	Aedes aegypti	SPECIES	10.36407	4.83729	3068151345
7937	Diptera	Culicidae	Aedes	Aedes aegypti	SPECIES	10.37000	-61.43000	1264887294
7938	Diptera	Culicidae	Aedes	Aedes aegypti	SPECIES	10.38000	105.44000	1264888890
7939	Diptera	Culicidae	Aedes	Aedes aegypti	SPECIES	10.38000	104.49000	1264888922
7940	Diptera	Culicidae	Aedes	Aedes aegypti	SPECIES	10.38000	-9.30000	1264891419
7941	Diptera	Culicidae	Aedes	Aedes aegypti	SPECIES	10.38000	77.83000	3351508278
7942	Diptera	Culicidae	Aedes	Aedes aegypti	SPECIES	10.38792	-67.60433	177060101
7943	Diptera	Culicidae	Aedes	Aedes aegypti	SPECIES	10.39000	106.90000	1264888165
7944	Diptera	Culicidae	Aedes	Aedes aegypti	SPECIES	10.40000	-75.50000	1264886413
7945	Diptera	Culicidae	Aedes	Aedes aegypti	SPECIES	10.40000	-75.48000	1264886439
7946	Diptera	Culicidae	Aedes	Aedes aegypti	SPECIES	10.40000	-67.59000	1264887286
7947	Diptera	Culicidae	Aedes	Aedes aegypti	SPECIES	10.40000	106.12000	1264888918
7948	Diptera	Culicidae	Aedes	Aedes aegypti	SPECIES	10.40472	-85.58773	226099194
7949	Diptera	Culicidae	Aedes	Aedes aegypti	SPECIES	10.41000	106.07000	1264886195
7950	Diptera	Culicidae	Aedes	Aedes aegypti	SPECIES	10.41217	-61.32093	4073374449
7951	Diptera	Culicidae	Aedes	Aedes aegypti	SPECIES	10.41477	-85.08670	226099198
7952	Diptera	Culicidae	Aedes	Aedes aegypti	SPECIES	10.42000	123.89000	1264885999
7953	Diptera	Culicidae	Aedes	Aedes aegypti	SPECIES	10.42000	123.90000	1264886016
7954	Diptera	Culicidae	Aedes	Aedes aegypti	SPECIES	10.42000	-61.32000	1264888307

№	Order	Family	Genus	Species	Taxon Rank	Latitude	Longitude	GBIF_ID
7955	Diptera	Culicidae	Aedes	Aedes aegypti	SPECIES	10.42000	-61.45000	1264890366
7956	Diptera	Culicidae	Aedes	Aedes aegypti	SPECIES	10.43000	123.88000	1264885987
7957	Diptera	Culicidae	Aedes	Aedes aegypti	SPECIES	10.43000	123.89000	1264886031
7958	Diptera	Culicidae	Aedes	Aedes aegypti	SPECIES	10.43000	123.87000	1264886066
7959	Diptera	Culicidae	Aedes	Aedes aegypti	SPECIES	10.43000	-66.82000	1264888153
7960	Diptera	Culicidae	Aedes	Aedes aegypti	SPECIES	10.43000	-61.13000	1264888539
7961	Diptera	Culicidae	Aedes	Aedes aegypti	SPECIES	10.43000	45.01000	1264889470
7962	Diptera	Culicidae	Aedes	Aedes aegypti	SPECIES	10.45000	-64.18000	1264887936
7963	Diptera	Culicidae	Aedes	Aedes aegypti	SPECIES	10.45000	-61.47000	1264888286
7964	Diptera	Culicidae	Aedes	Aedes aegypti	SPECIES	10.45000	105.15000	1264888893
7965	Diptera	Culicidae	Aedes	Aedes aegypti	SPECIES	10.45000	-85.40000	1264891051
7966	Diptera	Culicidae	Aedes	Aedes aegypti	SPECIES	10.45000	-61.35000	1264891285
7967	Diptera	Culicidae	Aedes	Aedes aegypti	SPECIES	10.46000	106.60000	1264887582
7968	Diptera	Culicidae	Aedes	Aedes aegypti	SPECIES	10.46000	-64.17000	1264887590
7969	Diptera	Culicidae	Aedes	Aedes aegypti	SPECIES	10.46000	105.64000	1264888902
7970	Diptera	Culicidae	Aedes	Aedes aegypti	SPECIES	10.46140	-64.17506	3963644664
7971	Diptera	Culicidae	Aedes	Aedes aegypti	SPECIES	10.47000	-67.77000	1264887972
7972	Diptera	Culicidae	Aedes	Aedes aegypti	SPECIES	10.47000	-66.11000	1264887984
7973	Diptera	Culicidae	Aedes	Aedes aegypti	SPECIES	10.47000	-68.01000	1264887991
7974	Diptera	Culicidae	Aedes	Aedes aegypti	SPECIES	10.47000	-68.02000	1264888851
7975	Diptera	Culicidae	Aedes	Aedes aegypti	SPECIES	10.47000	79.10000	3348195809
7976	Diptera	Culicidae	Aedes	Aedes aegypti	SPECIES	10.47386	-66.80930	177059928
7977	Diptera	Culicidae	Aedes	Aedes aegypti	SPECIES	10.48000	-63.55000	1264887975
7978	Diptera	Culicidae	Aedes	Aedes aegypti	SPECIES	10.48000	-68.20000	1264887992
7979	Diptera	Culicidae	Aedes	Aedes aegypti	SPECIES	10.48000	-67.62000	1264888000
7980	Diptera	Culicidae	Aedes	Aedes aegypti	SPECIES	10.48000	-63.29000	1264888846
7981	Diptera	Culicidae	Aedes	Aedes aegypti	SPECIES	10.48823	-67.81402	177060137
7982	Diptera	Culicidae	Aedes	Aedes aegypti	SPECIES	10.49000	-61.22000	1264886875
7983	Diptera	Culicidae	Aedes	Aedes aegypti	SPECIES	10.49000	106.64000	1264887440
7984	Diptera	Culicidae	Aedes	Aedes aegypti	SPECIES	10.49000	-68.12000	1264887950
7985	Diptera	Culicidae	Aedes	Aedes aegypti	SPECIES	10.49000	-66.87000	1264889180
7986	Diptera	Culicidae	Aedes	Aedes aegypti	SPECIES	10.49161	99.17656	4145858454
7987	Diptera	Culicidae	Aedes	Aedes aegypti	SPECIES	10.49686	-66.91853	177060560
7988	Diptera	Culicidae	Aedes	Aedes aegypti	SPECIES	10.50000	99.12000	1264886525
7989	Diptera	Culicidae	Aedes	Aedes aegypti	SPECIES	10.50000	-61.35000	1264886843
7990	Diptera	Culicidae	Aedes	Aedes aegypti	SPECIES	10.50000	-61.42000	1264888303
7991	Diptera	Culicidae	Aedes	Aedes aegypti	SPECIES	10.50000	-66.92000	1264890653
7992	Diptera	Culicidae	Aedes	Aedes aegypti	SPECIES	10.50000	78.46000	3348099473
7993	Diptera	Culicidae	Aedes	Aedes aegypti	SPECIES	10.50013	7.44927	226099057
7994	Diptera	Culicidae	Aedes	Aedes aegypti	SPECIES	10.50650	99.13122	4145850730
7995	Diptera	Culicidae	Aedes	Aedes aegypti	SPECIES	10.50944	-61.40265	3988450056

№	Order	Family	Genus	Species	Taxon Rank	Latitude	Longitude	GBIF_ID
7996	Diptera	Culicidae	Aedes	Aedes aegypti	SPECIES	10.51000	-69.49000	1264886316
7997	Diptera	Culicidae	Aedes	Aedes aegypti	SPECIES	10.51074	-66.91864	177060112
7998	Diptera	Culicidae	Aedes	Aedes aegypti	SPECIES	10.51879	-85.24181	226099197
7999	Diptera	Culicidae	Aedes	Aedes aegypti	SPECIES	10.52000	-61.40000	1264888585
8000	Diptera	Culicidae	Aedes	Aedes aegypti	SPECIES	10.53000	106.63000	1264886927
8001	Diptera	Culicidae	Aedes	Aedes aegypti	SPECIES	10.53000	106.41000	1264887447
8002	Diptera	Culicidae	Aedes	Aedes aegypti	SPECIES	10.53000	-61.45000	1264888237
8003	Diptera	Culicidae	Aedes	Aedes aegypti	SPECIES	10.53000	-61.43000	1264890280
8004	Diptera	Culicidae	Aedes	Aedes aegypti	SPECIES	10.53000	-2.70000	1264890811
8005	Diptera	Culicidae	Aedes	Aedes aegypti	SPECIES	10.54000	106.39000	1264886206
8006	Diptera	Culicidae	Aedes	Aedes aegypti	SPECIES	10.54613	-85.59046	226099195
8007	Diptera	Culicidae	Aedes	Aedes aegypti	SPECIES	10.55000	-61.37000	1264891076
8008	Diptera	Culicidae	Aedes	Aedes aegypti	SPECIES	10.56000	-61.42000	1264886878
8009	Diptera	Culicidae	Aedes	Aedes aegypti	SPECIES	10.58000	14.33000	1264886174
8010	Diptera	Culicidae	Aedes	Aedes aegypti	SPECIES	10.58000	106.40000	1264887430
8011	Diptera	Culicidae	Aedes	Aedes aegypti	SPECIES	10.58000	78.07000	3348156342
8012	Diptera	Culicidae	Aedes	Aedes aegypti	SPECIES	10.59000	-74.78000	1264887088
8013	Diptera	Culicidae	Aedes	Aedes aegypti	SPECIES	10.59000	-61.13000	1264888608
8014	Diptera	Culicidae	Aedes	Aedes aegypti	SPECIES	10.59000	-69.77000	1264888809
8015	Diptera	Culicidae	Aedes	Aedes aegypti	SPECIES	10.59406	99.14083	4145856061
8016	Diptera	Culicidae	Aedes	Aedes aegypti	SPECIES	10.59658	99.27986	4145855234
8017	Diptera	Culicidae	Aedes	Aedes aegypti	SPECIES	10.60000	-61.28000	1264886849
8018	Diptera	Culicidae	Aedes	Aedes aegypti	SPECIES	10.60000	106.05000	1264887459
8019	Diptera	Culicidae	Aedes	Aedes aegypti	SPECIES	10.60000	-66.96000	1264887985
8020	Diptera	Culicidae	Aedes	Aedes aegypti	SPECIES	10.60000	-61.45000	1264888558
8021	Diptera	Culicidae	Aedes	Aedes aegypti	SPECIES	10.60000	-61.40000	1264888565
8022	Diptera	Culicidae	Aedes	Aedes aegypti	SPECIES	10.60000	106.41000	1264888914
8023	Diptera	Culicidae	Aedes	Aedes aegypti	SPECIES	10.60000	-61.38000	1264889756
8024	Diptera	Culicidae	Aedes	Aedes aegypti	SPECIES	10.61000	106.45000	1264886172
8025	Diptera	Culicidae	Aedes	Aedes aegypti	SPECIES	10.61000	-66.87000	1264888018
8026	Diptera	Culicidae	Aedes	Aedes aegypti	SPECIES	10.61000	-67.03000	1264888867
8027	Diptera	Culicidae	Aedes	Aedes aegypti	SPECIES	10.61000	103.53000	1264889629
8028	Diptera	Culicidae	Aedes	Aedes aegypti	SPECIES	10.62000	-66.73000	1264887980
8029	Diptera	Culicidae	Aedes	Aedes aegypti	SPECIES	10.62000	-66.74000	1264888858
8030	Diptera	Culicidae	Aedes	Aedes aegypti	SPECIES	10.62000	-61.38000	1264890250
8031	Diptera	Culicidae	Aedes	Aedes aegypti	SPECIES	10.62889	-85.44639	226099196
8032	Diptera	Culicidae	Aedes	Aedes aegypti	SPECIES	10.63000	-61.43000	1264888267
8033	Diptera	Culicidae	Aedes	Aedes aegypti	SPECIES	10.63000	-61.40000	1264891103
8034	Diptera	Culicidae	Aedes	Aedes aegypti	SPECIES	10.63000	-85.43000	1264891217
8035	Diptera	Culicidae	Aedes	Aedes aegypti	SPECIES	10.63000	-61.38000	1264891359
8036	Diptera	Culicidae	Aedes	Aedes aegypti	SPECIES	10.63345	7.48260	226099059

№	Order	Family	Genus	Species	Taxon Rank	Latitude	Longitude	GBIF_ID
8037	Diptera	Culicidae	Aedes	Aedes aegypti	SPECIES	10.63737	-61.28228	4073374462
8038	Diptera	Culicidae	Aedes	Aedes aegypti	SPECIES	10.64000	-61.41000	1264887293
8039	Diptera	Culicidae	Aedes	Aedes aegypti	SPECIES	10.64000	-61.50000	1264887298
8040	Diptera	Culicidae	Aedes	Aedes aegypti	SPECIES	10.65000	-61.20000	1264888225
8041	Diptera	Culicidae	Aedes	Aedes aegypti	SPECIES	10.65000	-61.40000	1264888226
8042	Diptera	Culicidae	Aedes	Aedes aegypti	SPECIES	10.65000	-61.50000	1264888236
8043	Diptera	Culicidae	Aedes	Aedes aegypti	SPECIES	10.65000	-71.41000	1264888841
8044	Diptera	Culicidae	Aedes	Aedes aegypti	SPECIES	10.65000	-61.38000	1264888900
8045	Diptera	Culicidae	Aedes	Aedes aegypti	SPECIES	10.65000	-61.47000	1264888938
8046	Diptera	Culicidae	Aedes	Aedes aegypti	SPECIES	10.65000	-61.08000	1264891338
8047	Diptera	Culicidae	Aedes	Aedes aegypti	SPECIES	10.65000	-61.46000	1264891379
8048	Diptera	Culicidae	Aedes	Aedes aegypti	SPECIES	10.66000	106.56000	1264886212
8049	Diptera	Culicidae	Aedes	Aedes aegypti	SPECIES	10.66000	-61.52000	1264888952
8050	Diptera	Culicidae	Aedes	Aedes aegypti	SPECIES	10.66000	-61.42000	1264890600
8051	Diptera	Culicidae	Aedes	Aedes aegypti	SPECIES	10.66000	-61.50000	1264891390
8052	Diptera	Culicidae	Aedes	Aedes aegypti	SPECIES	10.66000	-61.51000	1264891405
8053	Diptera	Culicidae	Aedes	Aedes aegypti	SPECIES	10.66389	-61.51667	4073374470
8054	Diptera	Culicidae	Aedes	Aedes aegypti	SPECIES	10.66464	-61.65679	4073374465
8055	Diptera	Culicidae	Aedes	Aedes aegypti	SPECIES	10.67000	-61.53000	1264887292
8056	Diptera	Culicidae	Aedes	Aedes aegypti	SPECIES	10.67000	-63.25000	1264887986
8057	Diptera	Culicidae	Aedes	Aedes aegypti	SPECIES	10.67000	-61.50000	1264888174
8058	Diptera	Culicidae	Aedes	Aedes aegypti	SPECIES	10.67000	-61.07000	1264888223
8059	Diptera	Culicidae	Aedes	Aedes aegypti	SPECIES	10.67000	106.57000	1264888924
8060	Diptera	Culicidae	Aedes	Aedes aegypti	SPECIES	10.67000	-61.52000	1264889712
8061	Diptera	Culicidae	Aedes	Aedes aegypti	SPECIES	10.67000	106.62000	1264890119
8062	Diptera	Culicidae	Aedes	Aedes aegypti	SPECIES	10.67000	106.71000	1264890135
8063	Diptera	Culicidae	Aedes	Aedes aegypti	SPECIES	10.67000	106.60000	1264890157
8064	Diptera	Culicidae	Aedes	Aedes aegypti	SPECIES	10.67000	-61.51000	1264890222
8065	Diptera	Culicidae	Aedes	Aedes aegypti	SPECIES	10.68000	-61.55000	1264888273
8066	Diptera	Culicidae	Aedes	Aedes aegypti	SPECIES	10.68000	-61.30000	1264891300
8067	Diptera	Culicidae	Aedes	Aedes aegypti	SPECIES	10.68251	-61.64468	4073374464
8068	Diptera	Culicidae	Aedes	Aedes aegypti	SPECIES	10.69000	-68.30000	1264887988
8069	Diptera	Culicidae	Aedes	Aedes aegypti	SPECIES	10.69000	-61.60000	1264891393
8070	Diptera	Culicidae	Aedes	Aedes aegypti	SPECIES	10.70000	-61.47000	1264888572
8071	Diptera	Culicidae	Aedes	Aedes aegypti	SPECIES	10.70000	106.74000	1264888950
8072	Diptera	Culicidae	Aedes	Aedes aegypti	SPECIES	10.70000	-12.25000	1264891409
8073	Diptera	Culicidae	Aedes	Aedes aegypti	SPECIES	10.70741	-61.50959	4073374468
8074	Diptera	Culicidae	Aedes	Aedes aegypti	SPECIES	10.71000	105.12000	1264888910
8075	Diptera	Culicidae	Aedes	Aedes aegypti	SPECIES	10.71000	106.71000	1264890183
8076	Diptera	Culicidae	Aedes	Aedes aegypti	SPECIES	10.71000	-61.63000	1264891319
8077	Diptera	Culicidae	Aedes	Aedes aegypti	SPECIES	10.71000	-61.56000	1264891383

№	Order	Family	Genus	Species	Taxon Rank	Latitude	Longitude	GBIF_ID
8078	Diptera	Culicidae	Aedes	Aedes aegypti	SPECIES	10.71372	-61.58369	3337711921
8079	Diptera	Culicidae	Aedes	Aedes aegypti	SUBSPECIES	10.71677	7.53260	226099062
8080	Diptera	Culicidae	Aedes	Aedes aegypti	SPECIES	10.72000	-61.51000	1264887308
8081	Diptera	Culicidae	Aedes	Aedes aegypti	SPECIES	10.72000	-61.57000	1264888973
8082	Diptera	Culicidae	Aedes	Aedes aegypti	SPECIES	10.73000	106.64000	1264887583
8083	Diptera	Culicidae	Aedes	Aedes aegypti	SPECIES	10.73000	106.74000	1264890158
8084	Diptera	Culicidae	Aedes	Aedes aegypti	SPECIES	10.73623	-61.55448	3337903516
8085	Diptera	Culicidae	Aedes	Aedes aegypti	SPECIES	10.74000	106.55000	1264890120
8086	Diptera	Culicidae	Aedes	Aedes aegypti	SPECIES	10.74000	106.50000	1264890186
8087	Diptera	Culicidae	Aedes	Aedes aegypti	SPECIES	10.74000	13.81000	1264890443
8088	Diptera	Culicidae	Aedes	Aedes aegypti	SPECIES	10.74000	13.80000	1264891452
8089	Diptera	Culicidae	Aedes	Aedes aegypti	SPECIES	10.75000	106.38000	1264887568
8090	Diptera	Culicidae	Aedes	Aedes aegypti	SPECIES	10.75000	106.75000	1264888919
8091	Diptera	Culicidae	Aedes	Aedes aegypti	SPECIES	10.75000	106.71000	1264890146
8092	Diptera	Culicidae	Aedes	Aedes aegypti	SPECIES	10.75000	106.66000	1264891194
8093	Diptera	Culicidae	Aedes	Aedes aegypti	SPECIES	10.75000	-11.11000	1264891418
8094	Diptera	Culicidae	Aedes	Aedes aegypti	SPECIES	10.75000	78.69000	3351571054
8095	Diptera	Culicidae	Aedes	Aedes aegypti	SPECIES	10.76000	106.70000	1264890163
8096	Diptera	Culicidae	Aedes	Aedes aegypti	SPECIES	10.76000	106.66000	1264890164
8097	Diptera	Culicidae	Aedes	Aedes aegypti	SPECIES	10.76000	106.76000	1264890182
8098	Diptera	Culicidae	Aedes	Aedes aegypti	SPECIES	10.77000	79.63000	3348087456
8099	Diptera	Culicidae	Aedes	Aedes aegypti	SPECIES	10.77000	79.83000	3350850609
8100	Diptera	Culicidae	Aedes	Aedes aegypti	SPECIES	10.78000	-68.33000	1264887969
8101	Diptera	Culicidae	Aedes	Aedes aegypti	SPECIES	10.78000	106.76000	1264888906
8102	Diptera	Culicidae	Aedes	Aedes aegypti	SPECIES	10.78000	106.68000	1264888913
8103	Diptera	Culicidae	Aedes	Aedes aegypti	SPECIES	10.78000	106.69000	1264889800
8104	Diptera	Culicidae	Aedes	Aedes aegypti	SPECIES	10.78000	106.72000	1264890121
8105	Diptera	Culicidae	Aedes	Aedes aegypti	SPECIES	10.79000	-61.32000	1264890386
8106	Diptera	Culicidae	Aedes	Aedes aegypti	SPECIES	10.79000	-0.85000	1264890781
8107	Diptera	Culicidae	Aedes	Aedes aegypti	SPECIES	10.79686	-68.31859	177059592
8108	Diptera	Culicidae	Aedes	Aedes aegypti	SPECIES	10.79919	106.68026	3915336548
8109	Diptera	Culicidae	Aedes	Aedes aegypti	SPECIES	10.80000	-74.76000	1264887099
8110	Diptera	Culicidae	Aedes	Aedes aegypti	SPECIES	10.80000	-74.92000	1264887100
8111	Diptera	Culicidae	Aedes	Aedes aegypti	SPECIES	10.80000	-60.93000	1264888581
8112	Diptera	Culicidae	Aedes	Aedes aegypti	SPECIES	10.80000	-69.05000	1264888840
8113	Diptera	Culicidae	Aedes	Aedes aegypti	SPECIES	10.80000	106.69000	1264888949
8114	Diptera	Culicidae	Aedes	Aedes aegypti	SPECIES	10.81000	106.53000	1264890177
8115	Diptera	Culicidae	Aedes	Aedes aegypti	SPECIES	10.82000	-74.83000	1264887127
8116	Diptera	Culicidae	Aedes	Aedes aegypti	SPECIES	10.83000	-75.03000	1264887090
8117	Diptera	Culicidae	Aedes	Aedes aegypti	SPECIES	10.83000	-71.69000	1264887974
8118	Diptera	Culicidae	Aedes	Aedes aegypti	SPECIES	10.83000	106.69000	1264890151

№	Order	Family	Genus	Species	Taxon Rank	Latitude	Longitude	GBIF_ID
8119	Diptera	Culicidae	Aedes	Aedes aegypti	SPECIES	10.84000	106.31000	1264887205
8120	Diptera	Culicidae	Aedes	Aedes aegypti	SPECIES	10.85000	-74.79000	1264887106
8121	Diptera	Culicidae	Aedes	Aedes aegypti	SPECIES	10.85000	-74.78000	1264887130
8122	Diptera	Culicidae	Aedes	Aedes aegypti	SPECIES	10.85000	106.63000	1264888936
8123	Diptera	Culicidae	Aedes	Aedes aegypti	SPECIES	10.85000	106.76000	1264888946
8124	Diptera	Culicidae	Aedes	Aedes aegypti	SPECIES	10.85000	106.71000	1264890193
8125	Diptera	Culicidae	Aedes	Aedes aegypti	SPECIES	10.86000	106.80000	1264890162
8126	Diptera	Culicidae	Aedes	Aedes aegypti	SPECIES	10.87000	-74.83000	1264887101
8127	Diptera	Culicidae	Aedes	Aedes aegypti	SPECIES	10.87000	106.29000	1264887431
8128	Diptera	Culicidae	Aedes	Aedes aegypti	SPECIES	10.87000	-74.77000	1264890476
8129	Diptera	Culicidae	Aedes	Aedes aegypti	SPECIES	10.87000	-1.98000	1264890765
8130	Diptera	Culicidae	Aedes	Aedes aegypti	SPECIES	10.88000	-74.77000	1264887125
8131	Diptera	Culicidae	Aedes	Aedes aegypti	SPECIES	10.89000	-74.88000	1264887079
8132	Diptera	Culicidae	Aedes	Aedes aegypti	SPECIES	10.89000	-72.85000	1264887097
8133	Diptera	Culicidae	Aedes	Aedes aegypti	SPECIES	10.89000	-74.78000	1264887121
8134	Diptera	Culicidae	Aedes	Aedes aegypti	SPECIES	10.89000	106.59000	1264890148
8135	Diptera	Culicidae	Aedes	Aedes aegypti	SPECIES	10.90000	106.70000	1264888889
8136	Diptera	Culicidae	Aedes	Aedes aegypti	SPECIES	10.90000	106.54000	1264890191
8137	Diptera	Culicidae	Aedes	Aedes aegypti	SPECIES	10.90000	-13.03000	1264891415
8138	Diptera	Culicidae	Aedes	Aedes aegypti	SPECIES	10.91000	-74.79000	1264887116
8139	Diptera	Culicidae	Aedes	Aedes aegypti	SPECIES	10.92000	-74.79000	1264887083
8140	Diptera	Culicidae	Aedes	Aedes aegypti	SPECIES	10.92000	-74.75000	1264890485
8141	Diptera	Culicidae	Aedes	Aedes aegypti	SPECIES	10.93000	-68.29000	1264887958
8142	Diptera	Culicidae	Aedes	Aedes aegypti	SPECIES	10.94000	107.12000	1264886217
8143	Diptera	Culicidae	Aedes	Aedes aegypti	SPECIES	10.95000	106.99000	1264886181
8144	Diptera	Culicidae	Aedes	Aedes aegypti	SPECIES	10.96000	106.96000	1264886168
8145	Diptera	Culicidae	Aedes	Aedes aegypti	SPECIES	10.96000	-74.80000	1264887093
8146	Diptera	Culicidae	Aedes	Aedes aegypti	SPECIES	10.96000	-71.72000	1264888009
8147	Diptera	Culicidae	Aedes	Aedes aegypti	SPECIES	10.97000	-74.83000	1264887458
8148	Diptera	Culicidae	Aedes	Aedes aegypti	SPECIES	10.97000	106.47000	1264888927
8149	Diptera	Culicidae	Aedes	Aedes aegypti	SPECIES	10.97000	13.92000	1264890461
8150	Diptera	Culicidae	Aedes	Aedes aegypti	SPECIES	10.99000	106.65000	1264886219
8151	Diptera	Culicidae	Aedes	Aedes aegypti	SPECIES	11.00000	-63.80000	1264887989
8152	Diptera	Culicidae	Aedes	Aedes aegypti	SPECIES	11.00000	76.96000	1264891165
8153	Diptera	Culicidae	Aedes	Aedes aegypti	SPECIES	11.00000	77.00000	3350848623
8154	Diptera	Culicidae	Aedes	Aedes aegypti	SPECIES	11.01000	76.97000	1264890371
8155	Diptera	Culicidae	Aedes	Aedes aegypti	SPECIES	11.01000	78.40000	1264890941
8156	Diptera	Culicidae	Aedes	Aedes aegypti	SPECIES	11.01056	99.30790	4145857496
8157	Diptera	Culicidae	Aedes	Aedes aegypti	SPECIES	11.01348	-74.25206	225864834
8158	Diptera	Culicidae	Aedes	Aedes aegypti	SPECIES	11.02000	76.96000	1264886856
8159	Diptera	Culicidae	Aedes	Aedes aegypti	SPECIES	11.02000	-74.88000	1264887119

№	Order	Family	Genus	Species	Taxon Rank	Latitude	Longitude	GBIF_ID
8160	Diptera	Culicidae	Aedes	Aedes aegypti	SPECIES	11.02000	-63.87000	1264888020
8161	Diptera	Culicidae	Aedes	Aedes aegypti	SPECIES	11.02000	76.95000	3351537131
8162	Diptera	Culicidae	Aedes	Aedes aegypti	SPECIES	11.03000	76.89000	1264886852
8163	Diptera	Culicidae	Aedes	Aedes aegypti	SPECIES	11.03000	76.88000	1264886866
8164	Diptera	Culicidae	Aedes	Aedes aegypti	SPECIES	11.03000	76.98000	1264889988
8165	Diptera	Culicidae	Aedes	Aedes aegypti	SPECIES	11.04000	76.88000	1264886903
8166	Diptera	Culicidae	Aedes	Aedes aegypti	SPECIES	11.04000	14.14000	1264891446
8167	Diptera	Culicidae	Aedes	Aedes aegypti	SPECIES	11.05000	-68.35000	1264887998
8168	Diptera	Culicidae	Aedes	Aedes aegypti	SPECIES	11.05000	76.99000	1264889972
8169	Diptera	Culicidae	Aedes	Aedes aegypti	SPECIES	11.05000	77.20000	3348191333
8170	Diptera	Culicidae	Aedes	Aedes aegypti	SPECIES	11.08000	7.72000	1264887923
8171	Diptera	Culicidae	Aedes	Aedes aegypti	SPECIES	11.08000	-63.95000	1264888021
8172	Diptera	Culicidae	Aedes	Aedes aegypti	SPECIES	11.08000	-12.40000	1264891425
8173	Diptera	Culicidae	Aedes	Aedes aegypti	SPECIES	11.09000	77.36000	1264887967
8174	Diptera	Culicidae	Aedes	Aedes aegypti	SPECIES	11.09000	-71.85000	1264888007
8175	Diptera	Culicidae	Aedes	Aedes aegypti	SPECIES	11.10000	76.95000	1264889946
8176	Diptera	Culicidae	Aedes	Aedes aegypti	SPECIES	11.10000	77.34000	3351580292
8177	Diptera	Culicidae	Aedes	Aedes aegypti	SPECIES	11.13000	79.07000	3350821708
8178	Diptera	Culicidae	Aedes	Aedes aegypti	SPECIES	11.13000	78.13000	3350841700
8179	Diptera	Culicidae	Aedes	Aedes aegypti	SPECIES	11.14000	75.96000	1264889898
8180	Diptera	Culicidae	Aedes	Aedes aegypti	SPECIES	11.14000	78.56000	3350824746
8181	Diptera	Culicidae	Aedes	Aedes aegypti	SPECIES	11.15000	-60.82000	1264888205
8182	Diptera	Culicidae	Aedes	Aedes aegypti	SPECIES	11.15000	-60.83000	1264888208
8183	Diptera	Culicidae	Aedes	Aedes aegypti	SPECIES	11.15000	-60.78000	1264888214
8184	Diptera	Culicidae	Aedes	Aedes aegypti	SPECIES	11.15000	-60.80000	1264888217
8185	Diptera	Culicidae	Aedes	Aedes aegypti	SPECIES	11.17000	-60.80000	1264888220
8186	Diptera	Culicidae	Aedes	Aedes aegypti	SPECIES	11.17000	-60.75000	1264888232
8187	Diptera	Culicidae	Aedes	Aedes aegypti	SPECIES	11.17000	78.45000	1264889256
8188	Diptera	Culicidae	Aedes	Aedes aegypti	SPECIES	11.18000	-60.78000	1264888202
8189	Diptera	Culicidae	Aedes	Aedes aegypti	SPECIES	11.18000	-60.77000	1264888219
8190	Diptera	Culicidae	Aedes	Aedes aegypti	SPECIES	11.18000	-4.29000	1264890129
8191	Diptera	Culicidae	Aedes	Aedes aegypti	SPECIES	11.19000	-60.74000	1264887313
8192	Diptera	Culicidae	Aedes	Aedes aegypti	SPECIES	11.19000	-60.67000	1264888233
8193	Diptera	Culicidae	Aedes	Aedes aegypti	SPECIES	11.20000	-60.63000	1264888204
8194	Diptera	Culicidae	Aedes	Aedes aegypti	SPECIES	11.20000	-60.75000	1264888211
8195	Diptera	Culicidae	Aedes	Aedes aegypti	SPECIES	11.20000	-60.78000	1264888242
8196	Diptera	Culicidae	Aedes	Aedes aegypti	SPECIES	11.20000	-3.90000	1264889805
8197	Diptera	Culicidae	Aedes	Aedes aegypti	SPECIES	11.20000	-4.30000	1264891451
8198	Diptera	Culicidae	Aedes	Aedes aegypti	SPECIES	11.20000	77.46000	3350977739
8199	Diptera	Culicidae	Aedes	Aedes aegypti	SPECIES	11.21000	-60.78000	1264888243
8200	Diptera	Culicidae	Aedes	Aedes aegypti	SPECIES	11.22000	108.43000	1264886094

№	Order	Family	Genus	Species	Taxon Rank	Latitude	Longitude	GBIF_ID
8201	Diptera	Culicidae	Aedes	Aedes aegypti	SPECIES	11.22000	108.50000	1264886178
8202	Diptera	Culicidae	Aedes	Aedes aegypti	SPECIES	11.22000	-60.60000	1264888227
8203	Diptera	Culicidae	Aedes	Aedes aegypti	SPECIES	11.22000	-60.75000	1264888246
8204	Diptera	Culicidae	Aedes	Aedes aegypti	SPECIES	11.22000	-60.62000	1264888247
8205	Diptera	Culicidae	Aedes	Aedes aegypti	SPECIES	11.22000	78.17000	3351506285
8206	Diptera	Culicidae	Aedes	Aedes aegypti	SPECIES	11.22333	99.49626	4145853730
8207	Diptera	Culicidae	Aedes	Aedes aegypti	SPECIES	11.23000	-60.73000	1264888230
8208	Diptera	Culicidae	Aedes	Aedes aegypti	SPECIES	11.23000	-60.75000	1264888263
8209	Diptera	Culicidae	Aedes	Aedes aegypti	SPECIES	11.24000	-60.59000	1264888239
8210	Diptera	Culicidae	Aedes	Aedes aegypti	SPECIES	11.24000	-60.60000	1264888248
8211	Diptera	Culicidae	Aedes	Aedes aegypti	SPECIES	11.25000	-60.72000	1264888199
8212	Diptera	Culicidae	Aedes	Aedes aegypti	SPECIES	11.25000	-60.58000	1264888221
8213	Diptera	Culicidae	Aedes	Aedes aegypti	SPECIES	11.25000	-69.74000	1264891216
8214	Diptera	Culicidae	Aedes	Aedes aegypti	SPECIES	11.25042	-73.83122	2985565621
8215	Diptera	Culicidae	Aedes	Aedes aegypti	SPECIES	11.25042	-73.83125	2985565693
8216	Diptera	Culicidae	Aedes	Aedes aegypti	SPECIES	11.25089	-73.82911	2985565568
8217	Diptera	Culicidae	Aedes	Aedes aegypti	SPECIES	11.26000	-60.69000	1264888231
8218	Diptera	Culicidae	Aedes	Aedes aegypti	SPECIES	11.26000	-60.60000	1264888241
8219	Diptera	Culicidae	Aedes	Aedes aegypti	SPECIES	11.26000	106.36000	1264888901
8220	Diptera	Culicidae	Aedes	Aedes aegypti	SPECIES	11.27000	-60.57000	1264888210
8221	Diptera	Culicidae	Aedes	Aedes aegypti	SPECIES	11.28000	-60.68000	1264888234
8222	Diptera	Culicidae	Aedes	Aedes aegypti	SPECIES	11.29000	-60.53000	1264888257
8223	Diptera	Culicidae	Aedes	Aedes aegypti	SPECIES	11.29343	99.37558	4145853165
8224	Diptera	Culicidae	Aedes	Aedes aegypti	SPECIES	11.30000	-60.63000	1264888238
8225	Diptera	Culicidae	Aedes	Aedes aegypti	SPECIES	11.30000	-60.62000	1264888240
8226	Diptera	Culicidae	Aedes	Aedes aegypti	SPECIES	11.30000	-60.64000	1264888244
8227	Diptera	Culicidae	Aedes	Aedes aegypti	SPECIES	11.30000	104.80000	3818458192
8228	Diptera	Culicidae	Aedes	Aedes aegypti	SPECIES	11.30020	104.80100	3442515534
8229	Diptera	Culicidae	Aedes	Aedes aegypti	SPECIES	11.31000	-12.28000	1264891403
8230	Diptera	Culicidae	Aedes	Aedes aegypti	SPECIES	11.32000	-60.55000	1264888254
8231	Diptera	Culicidae	Aedes	Aedes aegypti	SPECIES	11.32000	-60.58000	1264888266
8232	Diptera	Culicidae	Aedes	Aedes aegypti	SPECIES	11.34000	77.71000	3351527394
8233	Diptera	Culicidae	Aedes	Aedes aegypti	SPECIES	11.35000	-71.97000	1264888028
8234	Diptera	Culicidae	Aedes	Aedes aegypti	SPECIES	11.35000	77.73000	1264891144
8235	Diptera	Culicidae	Aedes	Aedes aegypti	SPECIES	11.38000	-72.24000	1264887098
8236	Diptera	Culicidae	Aedes	Aedes aegypti	SPECIES	11.39000	78.12000	3350906727
8237	Diptera	Culicidae	Aedes	Aedes aegypti	SPECIES	11.40000	-69.68000	1264889326
8238	Diptera	Culicidae	Aedes	Aedes aegypti	SPECIES	11.40000	104.87000	1264889609
8239	Diptera	Culicidae	Aedes	Aedes aegypti	SPECIES	11.40000	104.98000	1264891310
8240	Diptera	Culicidae	Aedes	Aedes aegypti	SPECIES	11.40000	76.69000	3350768400
8241	Diptera	Culicidae	Aedes	Aedes aegypti	SPECIES	11.40000	76.70000	3350854518

№	Order	Family	Genus	Species	Taxon Rank	Latitude	Longitude	GBIF_ID
8242	Diptera	Culicidae	Aedes	Aedes aegypti	SPECIES	11.40000	76.87000	3351527023
8243	Diptera	Culicidae	Aedes	Aedes aegypti	SPECIES	11.43000	106.86000	1264886216
8244	Diptera	Culicidae	Aedes	Aedes aegypti	SPECIES	11.43000	79.49000	3350967398
8245	Diptera	Culicidae	Aedes	Aedes aegypti	SPECIES	11.45000	-11.68000	1264891401
8246	Diptera	Culicidae	Aedes	Aedes aegypti	SPECIES	11.46000	106.88000	1264886182
8247	Diptera	Culicidae	Aedes	Aedes aegypti	SPECIES	11.46000	-69.57000	1264888001
8248	Diptera	Culicidae	Aedes	Aedes aegypti	SPECIES	11.47000	105.00000	1264889618
8249	Diptera	Culicidae	Aedes	Aedes aegypti	SPECIES	11.47000	104.95000	1264889624
8250	Diptera	Culicidae	Aedes	Aedes aegypti	SPECIES	11.48000	104.82000	1264889606
8251	Diptera	Culicidae	Aedes	Aedes aegypti	SPECIES	11.49000	-69.36000	1264888026
8252	Diptera	Culicidae	Aedes	Aedes aegypti	SPECIES	11.50000	104.93000	1264889597
8253	Diptera	Culicidae	Aedes	Aedes aegypti	SPECIES	11.53000	107.79000	1264888916
8254	Diptera	Culicidae	Aedes	Aedes aegypti	SPECIES	11.53000	104.95000	1264889602
8255	Diptera	Culicidae	Aedes	Aedes aegypti	SPECIES	11.53989	99.57860	4145856232
8256	Diptera	Culicidae	Aedes	Aedes aegypti	SPECIES	11.54000	106.91000	1264886242
8257	Diptera	Culicidae	Aedes	Aedes aegypti	SPECIES	11.54000	-72.91000	1264887114
8258	Diptera	Culicidae	Aedes	Aedes aegypti	SPECIES	11.55000	106.90000	1264886233
8259	Diptera	Culicidae	Aedes	Aedes aegypti	SPECIES	11.55000	104.90000	1264889571
8260	Diptera	Culicidae	Aedes	Aedes aegypti	SPECIES	11.55000	104.92000	1264889634
8261	Diptera	Culicidae	Aedes	Aedes aegypti	SPECIES	11.55000	-72.90000	1264890671
8262	Diptera	Culicidae	Aedes	Aedes aegypti	SPECIES	11.55000	104.91000	1264890885
8263	Diptera	Culicidae	Aedes	Aedes aegypti	SPECIES	11.55000	43.16000	1264891118
8264	Diptera	Culicidae	Aedes	Aedes aegypti	SPECIES	11.55000	104.88000	1264891299
8265	Diptera	Culicidae	Aedes	Aedes aegypti	SPECIES	11.56000	104.92000	1264889612
8266	Diptera	Culicidae	Aedes	Aedes aegypti	SPECIES	11.56000	104.95000	1264890875
8267	Diptera	Culicidae	Aedes	Aedes aegypti	SPECIES	11.57000	104.92000	1264889646
8268	Diptera	Culicidae	Aedes	Aedes aegypti	SPECIES	11.57000	79.32000	3351085486
8269	Diptera	Culicidae	Aedes	Aedes aegypti	SPECIES	11.58000	108.08000	1264888895
8270	Diptera	Culicidae	Aedes	Aedes aegypti	SPECIES	11.58000	104.90000	1264889639
8271	Diptera	Culicidae	Aedes	Aedes aegypti	SPECIES	11.58000	92.67000	1264890399
8272	Diptera	Culicidae	Aedes	Aedes aegypti	SPECIES	11.58000	-11.90000	1264891422
8273	Diptera	Culicidae	Aedes	Aedes aegypti	SPECIES	11.59000	108.95000	1264886186
8274	Diptera	Culicidae	Aedes	Aedes aegypti	SPECIES	11.59000	43.15000	1264889847
8275	Diptera	Culicidae	Aedes	Aedes aegypti	SPECIES	11.59000	75.59000	1264890683
8276	Diptera	Culicidae	Aedes	Aedes aegypti	SPECIES	11.60000	104.93000	1264889625
8277	Diptera	Culicidae	Aedes	Aedes aegypti	SPECIES	11.60000	92.70000	1264890407
8278	Diptera	Culicidae	Aedes	Aedes aegypti	SPECIES	11.61000	108.99000	1264886231
8279	Diptera	Culicidae	Aedes	Aedes aegypti	SPECIES	11.61000	92.73000	1264889896
8280	Diptera	Culicidae	Aedes	Aedes aegypti	SPECIES	11.61000	92.70000	1264890437
8281	Diptera	Culicidae	Aedes	Aedes aegypti	SPECIES	11.62000	92.75000	1264886104
8282	Diptera	Culicidae	Aedes	Aedes aegypti	SPECIES	11.62000	-2.75000	1264889837

№	Order	Family	Genus	Species	Taxon Rank	Latitude	Longitude	GBIF_ID
8283	Diptera	Culicidae	Aedes	Aedes aegypti	SPECIES	11.63000	92.75000	1264889955
8284	Diptera	Culicidae	Aedes	Aedes aegypti	SPECIES	11.64000	92.75000	1264889944
8285	Diptera	Culicidae	Aedes	Aedes aegypti	SPECIES	11.65000	92.75000	1264889929
8286	Diptera	Culicidae	Aedes	Aedes aegypti	SPECIES	11.65000	92.73000	1264889940
8287	Diptera	Culicidae	Aedes	Aedes aegypti	SPECIES	11.66000	106.90000	1264886263
8288	Diptera	Culicidae	Aedes	Aedes aegypti	SPECIES	11.66000	78.14000	3351610159
8289	Diptera	Culicidae	Aedes	Aedes aegypti	SPECIES	11.67000	105.68000	1264889628
8290	Diptera	Culicidae	Aedes	Aedes aegypti	SPECIES	11.67000	92.76000	1264889947
8291	Diptera	Culicidae	Aedes	Aedes aegypti	SPECIES	11.67000	92.73000	1264889963
8292	Diptera	Culicidae	Aedes	Aedes aegypti	SPECIES	11.71000	107.10000	1264886201
8293	Diptera	Culicidae	Aedes	Aedes aegypti	SPECIES	11.72000	-86.27000	1264890957
8294	Diptera	Culicidae	Aedes	Aedes aegypti	SPECIES	11.72000	-12.02000	1264891407
8295	Diptera	Culicidae	Aedes	Aedes aegypti	SPECIES	11.73000	79.78000	1264889603
8296	Diptera	Culicidae	Aedes	Aedes aegypti	SPECIES	11.73000	0.37000	1264889775
8297	Diptera	Culicidae	Aedes	Aedes aegypti	SPECIES	11.74356	99.78556	4145850453
8298	Diptera	Culicidae	Aedes	Aedes aegypti	SPECIES	11.75000	108.39000	1264886203
8299	Diptera	Culicidae	Aedes	Aedes aegypti	SPECIES	11.75000	79.77000	1264890470
8300	Diptera	Culicidae	Aedes	Aedes aegypti	SPECIES	11.75000	-13.20000	1264891437
8301	Diptera	Culicidae	Aedes	Aedes aegypti	SPECIES	11.76000	107.01000	1264886207
8302	Diptera	Culicidae	Aedes	Aedes aegypti	SPECIES	11.78000	-72.45000	1264890665
8303	Diptera	Culicidae	Aedes	Aedes aegypti	SPECIES	11.78000	-86.51000	1264890970
8304	Diptera	Culicidae	Aedes	Aedes aegypti	SPECIES	11.79000	107.18000	1264886238
8305	Diptera	Culicidae	Aedes	Aedes aegypti	SPECIES	11.79366	99.79467	4145848529
8306	Diptera	Culicidae	Aedes	Aedes aegypti	SPECIES	11.80000	107.19000	1264886239
8307	Diptera	Culicidae	Aedes	Aedes aegypti	SPECIES	11.80000	76.00000	4152810243
8308	Diptera	Culicidae	Aedes	Aedes aegypti	SPECIES	11.81000	106.95000	1264886224
8309	Diptera	Culicidae	Aedes	Aedes aegypti	SPECIES	11.85000	-86.46000	1264890986
8310	Diptera	Culicidae	Aedes	Aedes aegypti	SPECIES	11.87000	104.75000	1264889633
8311	Diptera	Culicidae	Aedes	Aedes aegypti	SPECIES	11.91564	99.75014	4145852136
8312	Diptera	Culicidae	Aedes	Aedes aegypti	SPECIES	11.92000	109.15000	1264886141
8313	Diptera	Culicidae	Aedes	Aedes aegypti	SPECIES	11.92000	79.83000	1264889969
8314	Diptera	Culicidae	Aedes	Aedes aegypti	SPECIES	11.93000	79.84000	1264887949
8315	Diptera	Culicidae	Aedes	Aedes aegypti	SPECIES	11.93000	79.83000	1264888991
8316	Diptera	Culicidae	Aedes	Aedes aegypti	SPECIES	11.95000	108.45000	1264888921
8317	Diptera	Culicidae	Aedes	Aedes aegypti	SPECIES	11.95000	79.83000	1264890015
8318	Diptera	Culicidae	Aedes	Aedes aegypti	SPECIES	11.98000	107.59000	1264886262
8319	Diptera	Culicidae	Aedes	Aedes aegypti	SPECIES	11.99000	107.67000	1264886191
8320	Diptera	Culicidae	Aedes	Aedes aegypti	SPECIES	12.00000	105.45000	1264889590
8321	Diptera	Culicidae	Aedes	Aedes aegypti	SPECIES	12.00000	-83.75000	1264890779
8322	Diptera	Culicidae	Aedes	Aedes aegypti	SPECIES	12.00000	-61.77000	1264890914
8323	Diptera	Culicidae	Aedes	Aedes aegypti	SPECIES	12.01000	-61.75000	1264891326

№	Order	Family	Genus	Species	Taxon Rank	Latitude	Longitude	GBIF_ID
8324	Diptera	Culicidae	Aedes	Aedes aegypti	SPECIES	12.02000	-61.75000	1264889759
8325	Diptera	Culicidae	Aedes	Aedes aegypti	SPECIES	12.05000	102.32000	1264887671
8326	Diptera	Culicidae	Aedes	Aedes aegypti	SPECIES	12.05000	-61.74000	1264890917
8327	Diptera	Culicidae	Aedes	Aedes aegypti	SPECIES	12.05000	105.64000	1264891288
8328	Diptera	Culicidae	Aedes	Aedes aegypti	SPECIES	12.06000	-61.74000	1264890933
8329	Diptera	Culicidae	Aedes	Aedes aegypti	SPECIES	12.06613	-68.85168	3496611628
8330	Diptera	Culicidae	Aedes	Aedes aegypti	SPECIES	12.07000	-61.67000	1264890900
8331	Diptera	Culicidae	Aedes	Aedes aegypti	SPECIES	12.08000	15.03000	1264886170
8332	Diptera	Culicidae	Aedes	Aedes aegypti	SPECIES	12.08000	-12.30000	1264891441
8333	Diptera	Culicidae	Aedes	Aedes aegypti	SPECIES	12.08000	78.13000	3350960667
8334	Diptera	Culicidae	Aedes	Aedes aegypti	SPECIES	12.08000	78.12000	3351475201
8335	Diptera	Culicidae	Aedes	Aedes aegypti	SPECIES	12.12000	-61.63000	1264890903
8336	Diptera	Culicidae	Aedes	Aedes aegypti	SPECIES	12.12000	-61.65000	1264890958
8337	Diptera	Culicidae	Aedes	Aedes aegypti	SPECIES	12.12778	-86.14677	2456530656
8338	Diptera	Culicidae	Aedes	Aedes aegypti	SPECIES	12.13000	78.16000	1264891131
8339	Diptera	Culicidae	Aedes	Aedes aegypti	SPECIES	12.13432	-68.90980	3496606627
8340	Diptera	Culicidae	Aedes	Aedes aegypti	SPECIES	12.13433	-68.90984	3496613830
8341	Diptera	Culicidae	Aedes	Aedes aegypti	SPECIES	12.14000	-61.71000	1264890934
8342	Diptera	Culicidae	Aedes	Aedes aegypti	SPECIES	12.15000	-86.27000	1264890777
8343	Diptera	Culicidae	Aedes	Aedes aegypti	SPECIES	12.15000	79.07000	3348255594
8344	Diptera	Culicidae	Aedes	Aedes aegypti	SPECIES	12.15052	-68.97137	3496615472
8345	Diptera	Culicidae	Aedes	Aedes aegypti	SPECIES	12.15139	-68.97049	3496615129
8346	Diptera	Culicidae	Aedes	Aedes aegypti	SPECIES	12.15141	-68.97041	4052770312
8347	Diptera	Culicidae	Aedes	Aedes aegypti	SPECIES	12.17000	-61.73000	1264890931
8348	Diptera	Culicidae	Aedes	Aedes aegypti	SPECIES	12.17041	-68.23540	4158755048
8349	Diptera	Culicidae	Aedes	Aedes aegypti	SPECIES	12.18000	108.14000	1264886209
8350	Diptera	Culicidae	Aedes	Aedes aegypti	SPECIES	12.18987	102.45847	4145849131
8351	Diptera	Culicidae	Aedes	Aedes aegypti	SPECIES	12.19000	109.17000	1264886251
8352	Diptera	Culicidae	Aedes	Aedes aegypti	SPECIES	12.19000	109.22000	1264886871
8353	Diptera	Culicidae	Aedes	Aedes aegypti	SPECIES	12.19000	-61.70000	1264890938
8354	Diptera	Culicidae	Aedes	Aedes aegypti	SPECIES	12.19000	-61.69000	1264890947
8355	Diptera	Culicidae	Aedes	Aedes aegypti	SPECIES	12.20000	-61.64000	1264890920
8356	Diptera	Culicidae	Aedes	Aedes aegypti	SPECIES	12.21000	-86.28000	1264890953
8357	Diptera	Culicidae	Aedes	Aedes aegypti	SPECIES	12.22000	-4.16000	1264890296
8358	Diptera	Culicidae	Aedes	Aedes aegypti	SPECIES	12.22000	-61.64000	1264890937
8359	Diptera	Culicidae	Aedes	Aedes aegypti	SPECIES	12.23000	102.51000	1264886476
8360	Diptera	Culicidae	Aedes	Aedes aegypti	SPECIES	12.23447	99.85534	4145856426
8361	Diptera	Culicidae	Aedes	Aedes aegypti	SPECIES	12.24000	109.19000	1264886218
8362	Diptera	Culicidae	Aedes	Aedes aegypti	SPECIES	12.24000	102.52000	1264887475
8363	Diptera	Culicidae	Aedes	Aedes aegypti	SPECIES	12.24000	79.23000	1264889547
8364	Diptera	Culicidae	Aedes	Aedes aegypti	SPECIES	12.24511	99.95768	4145854145

№	Order	Family	Genus	Species	Taxon Rank	Latitude	Longitude	GBIF_ID
8365	Diptera	Culicidae	Aedes	Aedes aegypti	SPECIES	12.25000	79.33000	1264890138
8366	Diptera	Culicidae	Aedes	Aedes aegypti	SPECIES	12.25000	79.23000	1264891180
8367	Diptera	Culicidae	Aedes	Aedes aegypti	SPECIES	12.27000	109.19000	1264886180
8368	Diptera	Culicidae	Aedes	Aedes aegypti	SPECIES	12.28000	109.19000	1264886164
8369	Diptera	Culicidae	Aedes	Aedes aegypti	SPECIES	12.29000	109.19000	1264886226
8370	Diptera	Culicidae	Aedes	Aedes aegypti	SPECIES	12.30000	76.64000	1264887072
8371	Diptera	Culicidae	Aedes	Aedes aegypti	SPECIES	12.30000	-3.55000	1264889705
8372	Diptera	Culicidae	Aedes	Aedes aegypti	SPECIES	12.31000	107.48000	1264886252
8373	Diptera	Culicidae	Aedes	Aedes aegypti	SPECIES	12.31000	-86.02000	1264890998
8374	Diptera	Culicidae	Aedes	Aedes aegypti	SPECIES	12.32000	78.16000	3348113506
8375	Diptera	Culicidae	Aedes	Aedes aegypti	SPECIES	12.33000	109.03000	1264891028
8376	Diptera	Culicidae	Aedes	Aedes aegypti	SPECIES	12.35645	102.43059	4145859400
8377	Diptera	Culicidae	Aedes	Aedes aegypti	SPECIES	12.36000	-1.53000	1264890261
8378	Diptera	Culicidae	Aedes	Aedes aegypti	SPECIES	12.37000	109.08000	1264891262
8379	Diptera	Culicidae	Aedes	Aedes aegypti	SPECIES	12.38000	76.60000	1264888723
8380	Diptera	Culicidae	Aedes	Aedes aegypti	SPECIES	12.40000	109.18000	1264886205
8381	Diptera	Culicidae	Aedes	Aedes aegypti	SPECIES	12.40086	99.99143	4145849963
8382	Diptera	Culicidae	Aedes	Aedes aegypti	SPECIES	12.41000	-12.01000	1264890337
8383	Diptera	Culicidae	Aedes	Aedes aegypti	SPECIES	12.42000	107.58000	1264886225
8384	Diptera	Culicidae	Aedes	Aedes aegypti	SPECIES	12.42000	-3.17000	1264890257
8385	Diptera	Culicidae	Aedes	Aedes aegypti	SPECIES	12.43000	9.20000	1264888289
8386	Diptera	Culicidae	Aedes	Aedes aegypti	SPECIES	12.43000	-86.89000	1264888787
8387	Diptera	Culicidae	Aedes	Aedes aegypti	SPECIES	12.43488	-86.87132	4021956610
8388	Diptera	Culicidae	Aedes	Aedes aegypti	SPECIES	12.47154	-61.44438	177059737
8389	Diptera	Culicidae	Aedes	Aedes aegypti	SPECIES	12.48068	-61.46272	177059738
8390	Diptera	Culicidae	Aedes	Aedes aegypti	SPECIES	12.49000	109.09000	1264886889
8391	Diptera	Culicidae	Aedes	Aedes aegypti	SPECIES	12.50000	109.00000	1264890231
8392	Diptera	Culicidae	Aedes	Aedes aegypti	SPECIES	12.50000	79.45000	3348087455
8393	Diptera	Culicidae	Aedes	Aedes aegypti	SPECIES	12.51000	109.04000	1264886912
8394	Diptera	Culicidae	Aedes	Aedes aegypti	SPECIES	12.52000	-70.03000	1264890246
8395	Diptera	Culicidae	Aedes	Aedes aegypti	SPECIES	12.53000	-12.20000	1264890534
8396	Diptera	Culicidae	Aedes	Aedes aegypti	SPECIES	12.53000	-12.31000	1264891330
8397	Diptera	Culicidae	Aedes	Aedes aegypti	SPECIES	12.55000	109.17000	1264886227
8398	Diptera	Culicidae	Aedes	Aedes aegypti	SPECIES	12.55000	99.88000	1264886510
8399	Diptera	Culicidae	Aedes	Aedes aegypti	SPECIES	12.55000	-12.18000	1264888380
8400	Diptera	Culicidae	Aedes	Aedes aegypti	SPECIES	12.55000	79.11000	3350939720
8401	Diptera	Culicidae	Aedes	Aedes aegypti	SPECIES	12.56000	-12.19000	1264887827
8402	Diptera	Culicidae	Aedes	Aedes aegypti	SPECIES	12.56000	-12.17000	1264890614
8403	Diptera	Culicidae	Aedes	Aedes aegypti	SPECIES	12.57010	99.95239	4145853350
8404	Diptera	Culicidae	Aedes	Aedes aegypti	SPECIES	12.58000	-16.28000	1264887194
8405	Diptera	Culicidae	Aedes	Aedes aegypti	SPECIES	12.58000	101.45000	1264890199

№	Order	Family	Genus	Species	Taxon Rank	Latitude	Longitude	GBIF_ID
8406	Diptera	Culicidae	Aedes	Aedes aegypti	SPECIES	12.59000	-61.43000	1264890143
8407	Diptera	Culicidae	Aedes	Aedes aegypti	SPECIES	12.59000	-61.42000	1264890180
8408	Diptera	Culicidae	Aedes	Aedes aegypti	SPECIES	12.59206	99.93489	4145851271
8409	Diptera	Culicidae	Aedes	Aedes aegypti	SPECIES	12.59527	102.46227	4145853708
8410	Diptera	Culicidae	Aedes	Aedes aegypti	SPECIES	12.59867	102.10254	4145856910
8411	Diptera	Culicidae	Aedes	Aedes aegypti	SPECIES	12.60000	-12.24000	1264886605
8412	Diptera	Culicidae	Aedes	Aedes aegypti	SPECIES	12.60000	102.14000	1264889684
8413	Diptera	Culicidae	Aedes	Aedes aegypti	SPECIES	12.60000	-12.23000	1264890538
8414	Diptera	Culicidae	Aedes	Aedes aegypti	SPECIES	12.61000	107.92000	1264886198
8415	Diptera	Culicidae	Aedes	Aedes aegypti	SPECIES	12.61000	102.10000	1264887491
8416	Diptera	Culicidae	Aedes	Aedes aegypti	SPECIES	12.61000	-12.25000	1264887831
8417	Diptera	Culicidae	Aedes	Aedes aegypti	SPECIES	12.61000	-86.59000	1264890776
8418	Diptera	Culicidae	Aedes	Aedes aegypti	SPECIES	12.62000	-12.24000	1264886614
8419	Diptera	Culicidae	Aedes	Aedes aegypti	SPECIES	12.62000	101.38000	1264887297
8420	Diptera	Culicidae	Aedes	Aedes aegypti	SPECIES	12.63000	101.38000	1264887338
8421	Diptera	Culicidae	Aedes	Aedes aegypti	SPECIES	12.63000	-12.23000	1264887519
8422	Diptera	Culicidae	Aedes	Aedes aegypti	SPECIES	12.63000	-12.25000	1264890376
8423	Diptera	Culicidae	Aedes	Aedes aegypti	SPECIES	12.66000	-12.26000	1264886641
8424	Diptera	Culicidae	Aedes	Aedes aegypti	SPECIES	12.66000	77.99000	3349067854
8425	Diptera	Culicidae	Aedes	Aedes aegypti	SPECIES	12.66484	99.68780	4145852325
8426	Diptera	Culicidae	Aedes	Aedes aegypti	SPECIES	12.67000	101.28000	1264886441
8427	Diptera	Culicidae	Aedes	Aedes aegypti	SPECIES	12.67000	102.20000	1264886503
8428	Diptera	Culicidae	Aedes	Aedes aegypti	SPECIES	12.67000	-12.28000	1264886628
8429	Diptera	Culicidae	Aedes	Aedes aegypti	SPECIES	12.67000	108.04000	1264886692
8430	Diptera	Culicidae	Aedes	Aedes aegypti	SPECIES	12.67000	101.26000	1264887552
8431	Diptera	Culicidae	Aedes	Aedes aegypti	SPECIES	12.68344	102.19362	4145859706
8432	Diptera	Culicidae	Aedes	Aedes aegypti	SPECIES	12.68881	101.21344	4145858703
8433	Diptera	Culicidae	Aedes	Aedes aegypti	SPECIES	12.69000	108.11000	1264886196
8434	Diptera	Culicidae	Aedes	Aedes aegypti	SPECIES	12.70000	-15.56000	1264887188
8435	Diptera	Culicidae	Aedes	Aedes aegypti	SPECIES	12.72000	101.39000	1264886470
8436	Diptera	Culicidae	Aedes	Aedes aegypti	SPECIES	12.76000	108.73000	1264886264
8437	Diptera	Culicidae	Aedes	Aedes aegypti	SPECIES	12.77000	79.78000	1264889724
8438	Diptera	Culicidae	Aedes	Aedes aegypti	SPECIES	12.78525	102.05849	4145854246
8439	Diptera	Culicidae	Aedes	Aedes aegypti	SPECIES	12.78550	99.64556	4145860166
8440	Diptera	Culicidae	Aedes	Aedes aegypti	SPECIES	12.80000	-16.23000	1264887841
8441	Diptera	Culicidae	Aedes	Aedes aegypti	SPECIES	12.81000	77.58000	1264887437
8442	Diptera	Culicidae	Aedes	Aedes aegypti	SPECIES	12.81000	-3.61000	1264888777
8443	Diptera	Culicidae	Aedes	Aedes aegypti	SPECIES	12.81000	79.15000	1264891172
8444	Diptera	Culicidae	Aedes	Aedes aegypti	SPECIES	12.82000	79.15000	1264890073
8445	Diptera	Culicidae	Aedes	Aedes aegypti	SPECIES	12.83000	-11.75000	1264887535
8446	Diptera	Culicidae	Aedes	Aedes aegypti	SPECIES	12.83000	-11.76000	1264890357

№	Order	Family	Genus	Species	Taxon Rank	Latitude	Longitude	GBIF_ID
8447	Diptera	Culicidae	Aedes	Aedes aegypti	SPECIES	12.85000	101.43000	1264891031
8448	Diptera	Culicidae	Aedes	Aedes aegypti	SPECIES	12.87000	74.85000	1264889574
8449	Diptera	Culicidae	Aedes	Aedes aegypti	SPECIES	12.87749	99.92554	4145850709
8450	Diptera	Culicidae	Aedes	Aedes aegypti	SPECIES	12.90000	79.33000	1264889707
8451	Diptera	Culicidae	Aedes	Aedes aegypti	SPECIES	12.90326	101.71993	4145858740
8452	Diptera	Culicidae	Aedes	Aedes aegypti	SPECIES	12.91000	77.59000	1264887401
8453	Diptera	Culicidae	Aedes	Aedes aegypti	SPECIES	12.92000	79.14000	1264889771
8454	Diptera	Culicidae	Aedes	Aedes aegypti	SPECIES	12.92009	99.29646	4145858706
8455	Diptera	Culicidae	Aedes	Aedes aegypti	SPECIES	12.92557	77.61382	4011776492
8456	Diptera	Culicidae	Aedes	Aedes aegypti	SPECIES	12.93000	77.63000	1264887380
8457	Diptera	Culicidae	Aedes	Aedes aegypti	SPECIES	12.93000	77.60000	1264887386
8458	Diptera	Culicidae	Aedes	Aedes aegypti	SPECIES	12.93000	79.33000	1264889713
8459	Diptera	Culicidae	Aedes	Aedes aegypti	SPECIES	12.94000	77.75000	1264887427
8460	Diptera	Culicidae	Aedes	Aedes aegypti	SPECIES	12.94000	77.51000	1264887433
8461	Diptera	Culicidae	Aedes	Aedes aegypti	SPECIES	12.94000	100.91000	1264887505
8462	Diptera	Culicidae	Aedes	Aedes aegypti	SPECIES	12.94631	99.87525	4145855208
8463	Diptera	Culicidae	Aedes	Aedes aegypti	SPECIES	12.95000	77.67000	1264889917
8464	Diptera	Culicidae	Aedes	Aedes aegypti	SPECIES	12.96000	77.51000	1264887400
8465	Diptera	Culicidae	Aedes	Aedes aegypti	SPECIES	12.96000	100.90000	1264889012
8466	Diptera	Culicidae	Aedes	Aedes aegypti	SPECIES	12.96000	77.58000	1264889989
8467	Diptera	Culicidae	Aedes	Aedes aegypti	SPECIES	12.96055	101.73927	4145860153
8468	Diptera	Culicidae	Aedes	Aedes aegypti	SPECIES	12.97000	99.89000	1264886471
8469	Diptera	Culicidae	Aedes	Aedes aegypti	SPECIES	12.97000	77.58000	1264889695
8470	Diptera	Culicidae	Aedes	Aedes aegypti	SPECIES	12.98000	-3.15000	1264889833
8471	Diptera	Culicidae	Aedes	Aedes aegypti	SPECIES	12.99000	77.54000	1264887413
8472	Diptera	Culicidae	Aedes	Aedes aegypti	SPECIES	12.99000	77.70000	1264887423
8473	Diptera	Culicidae	Aedes	Aedes aegypti	SPECIES	13.00538	80.25210	2350355763
8474	Diptera	Culicidae	Aedes	Aedes aegypti	SPECIES	13.01000	-12.55000	1264887525
8475	Diptera	Culicidae	Aedes	Aedes aegypti	SPECIES	13.01000	101.00000	1264888967
8476	Diptera	Culicidae	Aedes	Aedes aegypti	SPECIES	13.02000	-12.55000	1264890404
8477	Diptera	Culicidae	Aedes	Aedes aegypti	SPECIES	13.03000	-13.30000	1264887538
8478	Diptera	Culicidae	Aedes	Aedes aegypti	SPECIES	13.03000	101.02000	1264888996
8479	Diptera	Culicidae	Aedes	Aedes aegypti	SPECIES	13.04000	77.60000	1264887409
8480	Diptera	Culicidae	Aedes	Aedes aegypti	SPECIES	13.04000	101.01000	1264888957
8481	Diptera	Culicidae	Aedes	Aedes aegypti	SPECIES	13.04000	80.19000	1264889902
8482	Diptera	Culicidae	Aedes	Aedes aegypti	SPECIES	13.04000	-13.30000	1264890367
8483	Diptera	Culicidae	Aedes	Aedes aegypti	SPECIES	13.04000	121.48000	1264890661
8484	Diptera	Culicidae	Aedes	Aedes aegypti	SPECIES	13.05000	80.25000	1264887393
8485	Diptera	Culicidae	Aedes	Aedes aegypti	SPECIES	13.05000	-86.58000	1264890637
8486	Diptera	Culicidae	Aedes	Aedes aegypti	SPECIES	13.06000	80.23000	1264887600
8487	Diptera	Culicidae	Aedes	Aedes aegypti	SPECIES	13.06000	123.71000	1264888193

№	Order	Family	Genus	Species	Taxon Rank	Latitude	Longitude	GBIF_ID
8488	Diptera	Culicidae	Aedes	Aedes aegypti	SPECIES	13.07000	100.07000	1264889637
8489	Diptera	Culicidae	Aedes	Aedes aegypti	SPECIES	13.07774	-59.60902	177059935
8490	Diptera	Culicidae	Aedes	Aedes aegypti	SPECIES	13.07811	-59.57216	177059939
8491	Diptera	Culicidae	Aedes	Aedes aegypti	SPECIES	13.08000	100.07000	1264889652
8492	Diptera	Culicidae	Aedes	Aedes aegypti	SPECIES	13.08000	100.06000	1264889854
8493	Diptera	Culicidae	Aedes	Aedes aegypti	SPECIES	13.08000	80.27000	1264890652
8494	Diptera	Culicidae	Aedes	Aedes aegypti	SPECIES	13.08000	80.28000	1264891381
8495	Diptera	Culicidae	Aedes	Aedes aegypti	SPECIES	13.08977	80.18088	3415742029
8496	Diptera	Culicidae	Aedes	Aedes aegypti	SPECIES	13.09000	100.06000	1264888981
8497	Diptera	Culicidae	Aedes	Aedes aegypti	SPECIES	13.09000	79.57000	3348052422
8498	Diptera	Culicidae	Aedes	Aedes aegypti	SPECIES	13.09475	100.06169	4145855738
8499	Diptera	Culicidae	Aedes	Aedes aegypti	SPECIES	13.10000	100.92000	1264886296
8500	Diptera	Culicidae	Aedes	Aedes aegypti	SPECIES	13.10000	-59.62000	1264889753
8501	Diptera	Culicidae	Aedes	Aedes aegypti	SPECIES	13.10000	80.29000	1264889941
8502	Diptera	Culicidae	Aedes	Aedes aegypti	SPECIES	13.11000	99.95000	1264887653
8503	Diptera	Culicidae	Aedes	Aedes aegypti	SPECIES	13.13114	99.93273	4145854088
8504	Diptera	Culicidae	Aedes	Aedes aegypti	SPECIES	13.14000	101.05000	1264887523
8505	Diptera	Culicidae	Aedes	Aedes aegypti	SPECIES	13.14000	99.61000	1264887661
8506	Diptera	Culicidae	Aedes	Aedes aegypti	SPECIES	13.14367	99.60589	4145855718
8507	Diptera	Culicidae	Aedes	Aedes aegypti	SPECIES	13.15813	100.97378	2611141927
8508	Diptera	Culicidae	Aedes	Aedes aegypti	SPECIES	13.16000	-61.23000	1264890312
8509	Diptera	Culicidae	Aedes	Aedes aegypti	SPECIES	13.16667	-59.55000	1933070521
8510	Diptera	Culicidae	Aedes	Aedes aegypti	SPECIES	13.18886	-59.56821	1933070157
8511	Diptera	Culicidae	Aedes	Aedes aegypti	SPECIES	13.19565	-59.63586	4029192803
8512	Diptera	Culicidae	Aedes	Aedes aegypti	SPECIES	13.21000	-13.11000	1264887532
8513	Diptera	Culicidae	Aedes	Aedes aegypti	SPECIES	13.21000	79.11000	1264889733
8514	Diptera	Culicidae	Aedes	Aedes aegypti	SPECIES	13.21810	99.67008	4145859452
8515	Diptera	Culicidae	Aedes	Aedes aegypti	SPECIES	13.22000	-13.12000	1264890425
8516	Diptera	Culicidae	Aedes	Aedes aegypti	SPECIES	13.22937	99.68732	4145855338
8517	Diptera	Culicidae	Aedes	Aedes aegypti	SPECIES	13.23973	101.50137	4145852162
8518	Diptera	Culicidae	Aedes	Aedes aegypti	SPECIES	13.27000	79.33000	3346676141
8519	Diptera	Culicidae	Aedes	Aedes aegypti	SPECIES	13.31654	99.65233	4145856145
8520	Diptera	Culicidae	Aedes	Aedes aegypti	SPECIES	13.32000	-14.53000	1264888721
8521	Diptera	Culicidae	Aedes	Aedes aegypti	SPECIES	13.34900	102.18258	4145851131
8522	Diptera	Culicidae	Aedes	Aedes aegypti	SPECIES	13.35531	99.58240	4145850180
8523	Diptera	Culicidae	Aedes	Aedes aegypti	SPECIES	13.36000	100.98000	1264887506
8524	Diptera	Culicidae	Aedes	Aedes aegypti	SPECIES	13.36000	99.91000	1264887647
8525	Diptera	Culicidae	Aedes	Aedes aegypti	SPECIES	13.37000	-14.65000	1264888707
8526	Diptera	Culicidae	Aedes	Aedes aegypti	SPECIES	13.37000	101.00000	1264890426
8527	Diptera	Culicidae	Aedes	Aedes aegypti	SPECIES	13.37981	99.27432	4145859544
8528	Diptera	Culicidae	Aedes	Aedes aegypti	SPECIES	13.38000	99.99000	1264890149

№	Order	Family	Genus	Species	Taxon Rank	Latitude	Longitude	GBIF_ID
8529	Diptera	Culicidae	Aedes	Aedes aegypti	SPECIES	13.38000	-14.41000	1264890289
8530	Diptera	Culicidae	Aedes	Aedes aegypti	SPECIES	13.38189	102.29806	4145854640
8531	Diptera	Culicidae	Aedes	Aedes aegypti	SPECIES	13.38639	99.92364	4145855819
8532	Diptera	Culicidae	Aedes	Aedes aegypti	SPECIES	13.39000	-14.18000	1264891487
8533	Diptera	Culicidae	Aedes	Aedes aegypti	SPECIES	13.39658	99.41395	4145860026
8534	Diptera	Culicidae	Aedes	Aedes aegypti	SPECIES	13.40000	-16.47000	1264888692
8535	Diptera	Culicidae	Aedes	Aedes aegypti	SPECIES	13.40000	-14.27000	1264888695
8536	Diptera	Culicidae	Aedes	Aedes aegypti	SPECIES	13.40000	-16.45000	1264891480
8537	Diptera	Culicidae	Aedes	Aedes aegypti	SPECIES	13.40000	79.20000	3349914397
8538	Diptera	Culicidae	Aedes	Aedes aegypti	SPECIES	13.40398	99.64625	4145857545
8539	Diptera	Culicidae	Aedes	Aedes aegypti	SPECIES	13.42199	99.37042	4145853587
8540	Diptera	Culicidae	Aedes	Aedes aegypti	SPECIES	13.42745	99.95983	4145859899
8541	Diptera	Culicidae	Aedes	Aedes aegypti	SPECIES	13.42768	99.38324	4145860350
8542	Diptera	Culicidae	Aedes	Aedes aegypti	SPECIES	13.42943	99.51186	4145858020
8543	Diptera	Culicidae	Aedes	Aedes aegypti	SPECIES	13.42995	99.24471	4145848745
8544	Diptera	Culicidae	Aedes	Aedes aegypti	SPECIES	13.43000	101.21000	1264887494
8545	Diptera	Culicidae	Aedes	Aedes aegypti	SPECIES	13.43000	-14.66000	1264888701
8546	Diptera	Culicidae	Aedes	Aedes aegypti	SPECIES	13.44100	100.03508	4145851399
8547	Diptera	Culicidae	Aedes	Aedes aegypti	SPECIES	13.45658	99.94702	4145852203
8548	Diptera	Culicidae	Aedes	Aedes aegypti	SPECIES	13.46000	-16.59000	1264888712
8549	Diptera	Culicidae	Aedes	Aedes aegypti	SPECIES	13.47000	-14.22000	1264888691
8550	Diptera	Culicidae	Aedes	Aedes aegypti	SPECIES	13.48000	-16.09000	1264891475
8551	Diptera	Culicidae	Aedes	Aedes aegypti	SPECIES	13.49000	-14.22000	1264888703
8552	Diptera	Culicidae	Aedes	Aedes aegypti	SPECIES	13.49000	-16.10000	1264888717
8553	Diptera	Culicidae	Aedes	Aedes aegypti	SPECIES	13.49000	-15.93000	1264891478
8554	Diptera	Culicidae	Aedes	Aedes aegypti	SPECIES	13.49568	101.79434	4145855895
8555	Diptera	Culicidae	Aedes	Aedes aegypti	SPECIES	13.50000	101.35000	1264887250
8556	Diptera	Culicidae	Aedes	Aedes aegypti	SPECIES	13.50000	99.85000	1264889575
8557	Diptera	Culicidae	Aedes	Aedes aegypti	SPECIES	13.50760	-87.63046	2963790047
8558	Diptera	Culicidae	Aedes	Aedes aegypti	SPECIES	13.51000	99.80000	1264886960
8559	Diptera	Culicidae	Aedes	Aedes aegypti	SPECIES	13.52000	99.96000	1264886485
8560	Diptera	Culicidae	Aedes	Aedes aegypti	SPECIES	13.52000	99.93000	1264889561
8561	Diptera	Culicidae	Aedes	Aedes aegypti	SPECIES	13.53000	99.81000	1264886513
8562	Diptera	Culicidae	Aedes	Aedes aegypti	SPECIES	13.53000	99.58000	1264890990
8563	Diptera	Culicidae	Aedes	Aedes aegypti	SPECIES	13.54000	101.37000	1264886675
8564	Diptera	Culicidae	Aedes	Aedes aegypti	SPECIES	13.54000	99.85000	1264887461
8565	Diptera	Culicidae	Aedes	Aedes aegypti	SPECIES	13.54000	99.81000	1264889555
8566	Diptera	Culicidae	Aedes	Aedes aegypti	SPECIES	13.55000	100.27000	1264886524
8567	Diptera	Culicidae	Aedes	Aedes aegypti	SPECIES	13.55838	100.21741	4145856945
8568	Diptera	Culicidae	Aedes	Aedes aegypti	SPECIES	13.56000	109.25000	1264886114
8569	Diptera	Culicidae	Aedes	Aedes aegypti	SPECIES	13.57000	-15.87000	1264888704

№	Order	Family	Genus	Species	Taxon Rank	Latitude	Longitude	GBIF_ID
8570	Diptera	Culicidae	Aedes	Aedes aegypti	SPECIES	13.57000	101.30000	1264890884
8571	Diptera	Culicidae	Aedes	Aedes aegypti	SPECIES	13.58000	101.52000	1264887248
8572	Diptera	Culicidae	Aedes	Aedes aegypti	SPECIES	13.58000	101.29000	1264890529
8573	Diptera	Culicidae	Aedes	Aedes aegypti	SPECIES	13.59000	99.52000	1264891303
8574	Diptera	Culicidae	Aedes	Aedes aegypti	SPECIES	13.60000	101.39000	1264887251
8575	Diptera	Culicidae	Aedes	Aedes aegypti	SPECIES	13.60000	101.30000	1264890526
8576	Diptera	Culicidae	Aedes	Aedes aegypti	SPECIES	13.62000	101.26000	1264887255
8577	Diptera	Culicidae	Aedes	Aedes aegypti	SPECIES	13.62000	99.59000	1264889951
8578	Diptera	Culicidae	Aedes	Aedes aegypti	SPECIES	13.62000	101.30000	1264890019
8579	Diptera	Culicidae	Aedes	Aedes aegypti	SPECIES	13.62000	99.52000	1264890928
8580	Diptera	Culicidae	Aedes	Aedes aegypti	SPECIES	13.62222	100.16500	4145849336
8581	Diptera	Culicidae	Aedes	Aedes aegypti	SPECIES	13.62625	101.01533	4145852335
8582	Diptera	Culicidae	Aedes	Aedes aegypti	SPECIES	13.63000	100.43000	1264887571
8583	Diptera	Culicidae	Aedes	Aedes aegypti	SPECIES	13.63000	101.30000	1264889107
8584	Diptera	Culicidae	Aedes	Aedes aegypti	SPECIES	13.63000	101.29000	1264889974
8585	Diptera	Culicidae	Aedes	Aedes aegypti	SPECIES	13.63000	100.50000	1264890565
8586	Diptera	Culicidae	Aedes	Aedes aegypti	SPECIES	13.63000	101.28000	1264890909
8587	Diptera	Culicidae	Aedes	Aedes aegypti	SPECIES	13.63296	-89.19067	177060504
8588	Diptera	Culicidae	Aedes	Aedes aegypti	SPECIES	13.64000	100.38000	1264890400
8589	Diptera	Culicidae	Aedes	Aedes aegypti	SPECIES	13.64000	101.29000	1264890609
8590	Diptera	Culicidae	Aedes	Aedes aegypti	SPECIES	13.65000	100.41000	1264887448
8591	Diptera	Culicidae	Aedes	Aedes aegypti	SPECIES	13.65000	101.29000	1264888810
8592	Diptera	Culicidae	Aedes	Aedes aegypti	SPECIES	13.65000	101.28000	1264889930
8593	Diptera	Culicidae	Aedes	Aedes aegypti	SPECIES	13.65000	101.30000	1264890012
8594	Diptera	Culicidae	Aedes	Aedes aegypti	SPECIES	13.65056	102.08278	4145860341
8595	Diptera	Culicidae	Aedes	Aedes aegypti	SPECIES	13.65427	100.46964	2251870098
8596	Diptera	Culicidae	Aedes	Aedes aegypti	SPECIES	13.66000	100.49000	1264886895
8597	Diptera	Culicidae	Aedes	Aedes aegypti	SPECIES	13.67000	100.61000	1264886920
8598	Diptera	Culicidae	Aedes	Aedes aegypti	SPECIES	13.67000	100.50000	1264890054
8599	Diptera	Culicidae	Aedes	Aedes aegypti	SPECIES	13.67136	99.37936	4145853393
8600	Diptera	Culicidae	Aedes	Aedes aegypti	SPECIES	13.68000	100.36000	1264886885
8601	Diptera	Culicidae	Aedes	Aedes aegypti	SPECIES	13.68000	100.54000	1264886898
8602	Diptera	Culicidae	Aedes	Aedes aegypti	SPECIES	13.68000	100.51000	1264886908
8603	Diptera	Culicidae	Aedes	Aedes aegypti	SPECIES	13.69000	100.61000	1264886946
8604	Diptera	Culicidae	Aedes	Aedes aegypti	SPECIES	13.69000	101.42000	1264887275
8605	Diptera	Culicidae	Aedes	Aedes aegypti	SPECIES	13.69000	101.08000	1264888322
8606	Diptera	Culicidae	Aedes	Aedes aegypti	SPECIES	13.69000	100.50000	1264890310
8607	Diptera	Culicidae	Aedes	Aedes aegypti	SPECIES	13.69000	100.41000	1264890358
8608	Diptera	Culicidae	Aedes	Aedes aegypti	SPECIES	13.69000	100.47000	1264890390
8609	Diptera	Culicidae	Aedes	Aedes aegypti	SPECIES	13.69044	102.49917	4145859406
8610	Diptera	Culicidae	Aedes	Aedes aegypti	SPECIES	13.69242	102.49767	4145851351

№	Order	Family	Genus	Species	Taxon Rank	Latitude	Longitude	GBIF_ID
8611	Diptera	Culicidae	Aedes	Aedes aegypti	SPECIES	13.69341	100.93137	4145854182
8612	Diptera	Culicidae	Aedes	Aedes aegypti	SPECIES	13.69378	100.59435	4145852143
8613	Diptera	Culicidae	Aedes	Aedes aegypti	SPECIES	13.69598	99.38649	4145856030
8614	Diptera	Culicidae	Aedes	Aedes aegypti	SPECIES	13.69828	100.49502	4145848678
8615	Diptera	Culicidae	Aedes	Aedes aegypti	SPECIES	13.70000	100.50000	1264886915
8616	Diptera	Culicidae	Aedes	Aedes aegypti	SPECIES	13.70000	100.49000	1264886918
8617	Diptera	Culicidae	Aedes	Aedes aegypti	SPECIES	13.70000	100.59000	1264886926
8618	Diptera	Culicidae	Aedes	Aedes aegypti	SPECIES	13.70000	100.41000	1264887455
8619	Diptera	Culicidae	Aedes	Aedes aegypti	SPECIES	13.70000	100.43000	1264887485
8620	Diptera	Culicidae	Aedes	Aedes aegypti	SPECIES	13.70000	100.46000	1264890414
8621	Diptera	Culicidae	Aedes	Aedes aegypti	SPECIES	13.70025	100.64466	4145850779
8622	Diptera	Culicidae	Aedes	Aedes aegypti	SPECIES	13.70109	99.35792	4145853803
8623	Diptera	Culicidae	Aedes	Aedes aegypti	SPECIES	13.71000	100.48000	1264886913
8624	Diptera	Culicidae	Aedes	Aedes aegypti	SPECIES	13.71000	100.52000	1264886916
8625	Diptera	Culicidae	Aedes	Aedes aegypti	SPECIES	13.71000	100.60000	1264886917
8626	Diptera	Culicidae	Aedes	Aedes aegypti	SPECIES	13.71000	100.41000	1264887462
8627	Diptera	Culicidae	Aedes	Aedes aegypti	SPECIES	13.71000	74.66000	1264889245
8628	Diptera	Culicidae	Aedes	Aedes aegypti	SPECIES	13.71000	100.58000	1264890141
8629	Diptera	Culicidae	Aedes	Aedes aegypti	SPECIES	13.71000	100.44000	1264890395
8630	Diptera	Culicidae	Aedes	Aedes aegypti	SPECIES	13.71108	100.57655	4145858690
8631	Diptera	Culicidae	Aedes	Aedes aegypti	SPECIES	13.71189	100.55210	4145849636
8632	Diptera	Culicidae	Aedes	Aedes aegypti	SPECIES	13.71423	100.42519	4145859610
8633	Diptera	Culicidae	Aedes	Aedes aegypti	SPECIES	13.71524	100.43333	4145854847
8634	Diptera	Culicidae	Aedes	Aedes aegypti	SPECIES	13.72000	100.45000	1264886901
8635	Diptera	Culicidae	Aedes	Aedes aegypti	SPECIES	13.72000	100.48000	1264886933
8636	Diptera	Culicidae	Aedes	Aedes aegypti	SPECIES	13.72000	100.78000	1264887496
8637	Diptera	Culicidae	Aedes	Aedes aegypti	SPECIES	13.72000	100.46000	1264890176
8638	Diptera	Culicidae	Aedes	Aedes aegypti	SPECIES	13.72000	100.70000	1264890287
8639	Diptera	Culicidae	Aedes	Aedes aegypti	SPECIES	13.72541	100.53353	4145859919
8640	Diptera	Culicidae	Aedes	Aedes aegypti	SPECIES	13.72562	100.75112	4145859815
8641	Diptera	Culicidae	Aedes	Aedes aegypti	SPECIES	13.73000	100.59000	1264886883
8642	Diptera	Culicidae	Aedes	Aedes aegypti	SPECIES	13.73000	100.49000	1264886921
8643	Diptera	Culicidae	Aedes	Aedes aegypti	SPECIES	13.73000	100.51000	1264886922
8644	Diptera	Culicidae	Aedes	Aedes aegypti	SPECIES	13.73000	100.58000	1264886931
8645	Diptera	Culicidae	Aedes	Aedes aegypti	SPECIES	13.73000	100.48000	1264886935
8646	Diptera	Culicidae	Aedes	Aedes aegypti	SPECIES	13.73000	100.52000	1264890175
8647	Diptera	Culicidae	Aedes	Aedes aegypti	SPECIES	13.73000	-14.81000	1264891486
8648	Diptera	Culicidae	Aedes	Aedes aegypti	SPECIES	13.73745	100.52987	4006594687
8649	Diptera	Culicidae	Aedes	Aedes aegypti	SPECIES	13.73768	100.52982	3743053166
8650	Diptera	Culicidae	Aedes	Aedes aegypti	SPECIES	13.74000	100.52000	1264886941
8651	Diptera	Culicidae	Aedes	Aedes aegypti	SPECIES	13.74000	100.79000	1264890023

№	Order	Family	Genus	Species	Taxon Rank	Latitude	Longitude	GBIF_ID
8652	Diptera	Culicidae	Aedes	Aedes aegypti	SPECIES	13.74000	-89.18000	1264890693
8653	Diptera	Culicidae	Aedes	Aedes aegypti	SPECIES	13.74000	-89.39000	1264891242
8654	Diptera	Culicidae	Aedes	Aedes aegypti	SPECIES	13.74392	102.32925	4145858664
8655	Diptera	Culicidae	Aedes	Aedes aegypti	SPECIES	13.74619	100.63066	4145855446
8656	Diptera	Culicidae	Aedes	Aedes aegypti	SPECIES	13.74795	100.59084	3117759429
8657	Diptera	Culicidae	Aedes	Aedes aegypti	SPECIES	13.74833	101.34583	4145850132
8658	Diptera	Culicidae	Aedes	Aedes aegypti	SPECIES	13.75000	100.59000	1264886938
8659	Diptera	Culicidae	Aedes	Aedes aegypti	SPECIES	13.75000	100.50000	1264887993
8660	Diptera	Culicidae	Aedes	Aedes aegypti	SPECIES	13.75000	100.52000	1264889607
8661	Diptera	Culicidae	Aedes	Aedes aegypti	SPECIES	13.75000	100.53000	1264889948
8662	Diptera	Culicidae	Aedes	Aedes aegypti	SPECIES	13.75000	100.55000	1264890152
8663	Diptera	Culicidae	Aedes	Aedes aegypti	SPECIES	13.75000	100.49000	1264890190
8664	Diptera	Culicidae	Aedes	Aedes aegypti	SPECIES	13.75000	100.51000	1264890524
8665	Diptera	Culicidae	Aedes	Aedes aegypti	SPECIES	13.75007	100.65089	4145854713
8666	Diptera	Culicidae	Aedes	Aedes aegypti	SPECIES	13.75924	100.65893	3985874602
8667	Diptera	Culicidae	Aedes	Aedes aegypti	SPECIES	13.75997	100.10164	4145848333
8668	Diptera	Culicidae	Aedes	Aedes aegypti	SPECIES	13.76000	100.53000	1264886939
8669	Diptera	Culicidae	Aedes	Aedes aegypti	SPECIES	13.76000	100.50000	1264886955
8670	Diptera	Culicidae	Aedes	Aedes aegypti	SPECIES	13.76000	100.58000	1264890055
8671	Diptera	Culicidae	Aedes	Aedes aegypti	SPECIES	13.76000	100.47000	1264890058
8672	Diptera	Culicidae	Aedes	Aedes aegypti	SPECIES	13.76000	100.48000	1264890181
8673	Diptera	Culicidae	Aedes	Aedes aegypti	SPECIES	13.76622	100.69225	4145853815
8674	Diptera	Culicidae	Aedes	Aedes aegypti	SPECIES	13.77000	100.55000	1264886937
8675	Diptera	Culicidae	Aedes	Aedes aegypti	SPECIES	13.77000	100.43000	1264886944
8676	Diptera	Culicidae	Aedes	Aedes aegypti	SPECIES	13.77000	100.68000	1264886964
8677	Diptera	Culicidae	Aedes	Aedes aegypti	SPECIES	13.77000	100.58000	1264887501
8678	Diptera	Culicidae	Aedes	Aedes aegypti	SPECIES	13.77000	100.47000	1264887511
8679	Diptera	Culicidae	Aedes	Aedes aegypti	SPECIES	13.77000	100.53000	1264890216
8680	Diptera	Culicidae	Aedes	Aedes aegypti	SPECIES	13.77000	100.50000	1264890295
8681	Diptera	Culicidae	Aedes	Aedes aegypti	SPECIES	13.77000	-13.68000	1264890438
8682	Diptera	Culicidae	Aedes	Aedes aegypti	SPECIES	13.77000	100.63000	1264891030
8683	Diptera	Culicidae	Aedes	Aedes aegypti	SPECIES	13.77000	-89.16000	1264891304
8684	Diptera	Culicidae	Aedes	Aedes aegypti	SPECIES	13.78000	100.52000	1264886930
8685	Diptera	Culicidae	Aedes	Aedes aegypti	SPECIES	13.78000	100.36000	1264886957
8686	Diptera	Culicidae	Aedes	Aedes aegypti	SPECIES	13.78000	100.62000	1264887925
8687	Diptera	Culicidae	Aedes	Aedes aegypti	SPECIES	13.78000	100.57000	1264888806
8688	Diptera	Culicidae	Aedes	Aedes aegypti	SPECIES	13.78000	100.56000	1264889198
8689	Diptera	Culicidae	Aedes	Aedes aegypti	SPECIES	13.78000	100.55000	1264889823
8690	Diptera	Culicidae	Aedes	Aedes aegypti	SPECIES	13.78000	100.58000	1264890178
8691	Diptera	Culicidae	Aedes	Aedes aegypti	SPECIES	13.78094	100.67382	4145859046
8692	Diptera	Culicidae	Aedes	Aedes aegypti	SPECIES	13.78353	99.28000	4145860564

№	Order	Family	Genus	Species	Taxon Rank	Latitude	Longitude	GBIF_ID
8693	Diptera	Culicidae	Aedes	Aedes aegypti	SPECIES	13.78809	100.48931	3455886816
8694	Diptera	Culicidae	Aedes	Aedes aegypti	SPECIES	13.79000	100.57000	1264889593
8695	Diptera	Culicidae	Aedes	Aedes aegypti	SPECIES	13.79042	100.55053	3858748958
8696	Diptera	Culicidae	Aedes	Aedes aegypti	SPECIES	13.79071	99.31702	4145859121
8697	Diptera	Culicidae	Aedes	Aedes aegypti	SPECIES	13.79076	100.55039	3903474002
8698	Diptera	Culicidae	Aedes	Aedes aegypti	SPECIES	13.79670	100.08192	4145859793
8699	Diptera	Culicidae	Aedes	Aedes aegypti	SPECIES	13.80000	109.15000	1264886139
8700	Diptera	Culicidae	Aedes	Aedes aegypti	SPECIES	13.80000	100.53000	1264887953
8701	Diptera	Culicidae	Aedes	Aedes aegypti	SPECIES	13.80000	100.32000	1264890286
8702	Diptera	Culicidae	Aedes	Aedes aegypti	SPECIES	13.80000	-15.92000	1264890618
8703	Diptera	Culicidae	Aedes	Aedes aegypti	SPECIES	13.80000	102.89000	1264891259
8704	Diptera	Culicidae	Aedes	Aedes aegypti	SPECIES	13.80203	100.53877	3456549902
8705	Diptera	Culicidae	Aedes	Aedes aegypti	SPECIES	13.80753	100.69242	4145848461
8706	Diptera	Culicidae	Aedes	Aedes aegypti	SPECIES	13.81000	102.07000	1264886458
8707	Diptera	Culicidae	Aedes	Aedes aegypti	SPECIES	13.81000	100.52000	1264890187
8708	Diptera	Culicidae	Aedes	Aedes aegypti	SPECIES	13.81038	100.54110	4145857944
8709	Diptera	Culicidae	Aedes	Aedes aegypti	SPECIES	13.81936	102.08029	4145854351
8710	Diptera	Culicidae	Aedes	Aedes aegypti	SPECIES	13.82000	100.07000	1264886438
8711	Diptera	Culicidae	Aedes	Aedes aegypti	SPECIES	13.82000	100.06000	1264886490
8712	Diptera	Culicidae	Aedes	Aedes aegypti	SPECIES	13.82000	102.06000	1264887499
8713	Diptera	Culicidae	Aedes	Aedes aegypti	SPECIES	13.82631	100.81649	4145854161
8714	Diptera	Culicidae	Aedes	Aedes aegypti	SPECIES	13.82711	100.79469	4145857372
8715	Diptera	Culicidae	Aedes	Aedes aegypti	SPECIES	13.82784	99.26803	4145855665
8716	Diptera	Culicidae	Aedes	Aedes aegypti	SPECIES	13.82966	99.26340	4145857826
8717	Diptera	Culicidae	Aedes	Aedes aegypti	SPECIES	13.83000	100.67000	1264886511
8718	Diptera	Culicidae	Aedes	Aedes aegypti	SPECIES	13.83000	100.56000	1264886943
8719	Diptera	Culicidae	Aedes	Aedes aegypti	SPECIES	13.83000	100.68000	1264886979
8720	Diptera	Culicidae	Aedes	Aedes aegypti	SPECIES	13.83293	100.58008	3416152831
8721	Diptera	Culicidae	Aedes	Aedes aegypti	SPECIES	13.83305	100.58021	3772447732
8722	Diptera	Culicidae	Aedes	Aedes aegypti	SPECIES	13.83990	100.49200	3443512421
8723	Diptera	Culicidae	Aedes	Aedes aegypti	SPECIES	13.84000	100.49000	3818067134
8724	Diptera	Culicidae	Aedes	Aedes aegypti	SPECIES	13.84315	100.48296	4145859915
8725	Diptera	Culicidae	Aedes	Aedes aegypti	SPECIES	13.84526	100.44845	4145848401
8726	Diptera	Culicidae	Aedes	Aedes aegypti	SPECIES	13.84586	100.60025	4145855997
8727	Diptera	Culicidae	Aedes	Aedes aegypti	SPECIES	13.84845	99.40871	4145854478
8728	Diptera	Culicidae	Aedes	Aedes aegypti	SPECIES	13.84886	100.56792	4145851805
8729	Diptera	Culicidae	Aedes	Aedes aegypti	SPECIES	13.84981	100.57142	4145851197
8730	Diptera	Culicidae	Aedes	Aedes aegypti	SPECIES	13.85000	100.50000	1264889576
8731	Diptera	Culicidae	Aedes	Aedes aegypti	SPECIES	13.85000	106.16000	1264891283
8732	Diptera	Culicidae	Aedes	Aedes aegypti	SPECIES	13.85175	100.56425	4145854765
8733	Diptera	Culicidae	Aedes	Aedes aegypti	SPECIES	13.85210	100.57937	4145856756

№	Order	Family	Genus	Species	Taxon Rank	Latitude	Longitude	GBIF_ID
8734	Diptera	Culicidae	Aedes	Aedes aegypti	SPECIES	13.86000	100.51000	1264886434
8735	Diptera	Culicidae	Aedes	Aedes aegypti	SPECIES	13.86000	100.70000	1264886940
8736	Diptera	Culicidae	Aedes	Aedes aegypti	SPECIES	13.86000	100.86000	1264886947
8737	Diptera	Culicidae	Aedes	Aedes aegypti	SPECIES	13.86000	100.49000	1264889937
8738	Diptera	Culicidae	Aedes	Aedes aegypti	SPECIES	13.86197	100.58686	4145858550
8739	Diptera	Culicidae	Aedes	Aedes aegypti	SPECIES	13.86944	99.26345	4145859740
8740	Diptera	Culicidae	Aedes	Aedes aegypti	SPECIES	13.87000	100.56000	1264890040
8741	Diptera	Culicidae	Aedes	Aedes aegypti	SPECIES	13.87000	100.60000	1264890326
8742	Diptera	Culicidae	Aedes	Aedes aegypti	SPECIES	13.88000	100.58000	1264887504
8743	Diptera	Culicidae	Aedes	Aedes aegypti	SPECIES	13.88000	100.52000	1264887567
8744	Diptera	Culicidae	Aedes	Aedes aegypti	SPECIES	13.88000	100.59000	1264887938
8745	Diptera	Culicidae	Aedes	Aedes aegypti	SPECIES	13.89000	109.12000	1264886163
8746	Diptera	Culicidae	Aedes	Aedes aegypti	SPECIES	13.89000	100.49000	1264889874
8747	Diptera	Culicidae	Aedes	Aedes aegypti	SPECIES	13.89061	99.43300	4145859460
8748	Diptera	Culicidae	Aedes	Aedes aegypti	SPECIES	13.89287	100.56095	4145851483
8749	Diptera	Culicidae	Aedes	Aedes aegypti	SPECIES	13.89305	99.14963	4145850243
8750	Diptera	Culicidae	Aedes	Aedes aegypti	SPECIES	13.89761	99.15193	4145859143
8751	Diptera	Culicidae	Aedes	Aedes aegypti	SPECIES	13.89865	100.62607	4145854895
8752	Diptera	Culicidae	Aedes	Aedes aegypti	SPECIES	13.91000	100.40000	1264886528
8753	Diptera	Culicidae	Aedes	Aedes aegypti	SPECIES	13.91000	100.60000	1264889130
8754	Diptera	Culicidae	Aedes	Aedes aegypti	SPECIES	13.91197	100.57163	4145850050
8755	Diptera	Culicidae	Aedes	Aedes aegypti	SPECIES	13.91456	99.18060	4145850523
8756	Diptera	Culicidae	Aedes	Aedes aegypti	SPECIES	13.92590	100.41124	4145853073
8757	Diptera	Culicidae	Aedes	Aedes aegypti	SPECIES	13.93000	100.59000	1264889086
8758	Diptera	Culicidae	Aedes	Aedes aegypti	SPECIES	13.93000	-15.55000	1264891406
8759	Diptera	Culicidae	Aedes	Aedes aegypti	SPECIES	13.94252	100.43838	3892643530
8760	Diptera	Culicidae	Aedes	Aedes aegypti	SPECIES	13.95000	-89.18000	1264891385
8761	Diptera	Culicidae	Aedes	Aedes aegypti	SPECIES	13.95000	-15.51000	1264891458
8762	Diptera	Culicidae	Aedes	Aedes aegypti	SPECIES	13.96000	109.07000	1264886220
8763	Diptera	Culicidae	Aedes	Aedes aegypti	SPECIES	13.96097	102.03368	4145855953
8764	Diptera	Culicidae	Aedes	Aedes aegypti	SPECIES	13.97538	99.63528	4145856860
8765	Diptera	Culicidae	Aedes	Aedes aegypti	SPECIES	13.98000	-14.80000	1264890136
8766	Diptera	Culicidae	Aedes	Aedes aegypti	SPECIES	13.98000	-14.82000	1264890383
8767	Diptera	Culicidae	Aedes	Aedes aegypti	SPECIES	13.98707	99.17376	4145856346
8768	Diptera	Culicidae	Aedes	Aedes aegypti	SPECIES	13.98879	99.26821	4145858513
8769	Diptera	Culicidae	Aedes	Aedes aegypti	SPECIES	13.99842	100.56992	4145850535
8770	Diptera	Culicidae	Aedes	Aedes aegypti	SPECIES	14.00000	99.55000	1264886464
8771	Diptera	Culicidae	Aedes	Aedes aegypti	SPECIES	14.00167	101.70700	4145856035
8772	Diptera	Culicidae	Aedes	Aedes aegypti	SPECIES	14.01643	99.33071	4145850074
8773	Diptera	Culicidae	Aedes	Aedes aegypti	SPECIES	14.01708	100.53066	4145858093
8774	Diptera	Culicidae	Aedes	Aedes aegypti	SPECIES	14.01764	99.53347	4145851869

№	Order	Family	Genus	Species	Taxon Rank	Latitude	Longitude	GBIF_ID
8775	Diptera	Culicidae	Aedes	Aedes aegypti	SPECIES	14.02159	99.33337	4145850605
8776	Diptera	Culicidae	Aedes	Aedes aegypti	SPECIES	14.02318	99.24047	4145851548
8777	Diptera	Culicidae	Aedes	Aedes aegypti	SPECIES	14.02541	99.53256	4145858428
8778	Diptera	Culicidae	Aedes	Aedes aegypti	SPECIES	14.02609	99.18713	4145860224
8779	Diptera	Culicidae	Aedes	Aedes aegypti	SPECIES	14.02688	99.12841	4145860183
8780	Diptera	Culicidae	Aedes	Aedes aegypti	SPECIES	14.02826	-87.22404	3496613636
8781	Diptera	Culicidae	Aedes	Aedes aegypti	SPECIES	14.02838	99.37854	4145854437
8782	Diptera	Culicidae	Aedes	Aedes aegypti	SPECIES	14.03000	-60.98000	1264889721
8783	Diptera	Culicidae	Aedes	Aedes aegypti	SPECIES	14.03723	-86.55371	3747094759
8784	Diptera	Culicidae	Aedes	Aedes aegypti	SPECIES	14.04000	102.05000	1264889026
8785	Diptera	Culicidae	Aedes	Aedes aegypti	SPECIES	14.04672	99.21178	4145859028
8786	Diptera	Culicidae	Aedes	Aedes aegypti	SPECIES	14.05000	101.37000	1264886480
8787	Diptera	Culicidae	Aedes	Aedes aegypti	SPECIES	14.05000	100.40000	1264890317
8788	Diptera	Culicidae	Aedes	Aedes aegypti	SPECIES	14.05057	-87.15474	3090528559
8789	Diptera	Culicidae	Aedes	Aedes aegypti	SPECIES	14.05254	99.12327	4145859566
8790	Diptera	Culicidae	Aedes	Aedes aegypti	SPECIES	14.05742	101.38200	4145853557
8791	Diptera	Culicidae	Aedes	Aedes aegypti	SPECIES	14.06000	102.03000	1264889664
8792	Diptera	Culicidae	Aedes	Aedes aegypti	SPECIES	14.06000	-15.58000	1264891444
8793	Diptera	Culicidae	Aedes	Aedes aegypti	SPECIES	14.06550	101.51442	4145848897
8794	Diptera	Culicidae	Aedes	Aedes aegypti	SPECIES	14.07000	-15.59000	1264890153
8795	Diptera	Culicidae	Aedes	Aedes aegypti	SPECIES	14.07000	-15.30000	1264890418
8796	Diptera	Culicidae	Aedes	Aedes aegypti	SPECIES	14.07711	99.01348	4145857644
8797	Diptera	Culicidae	Aedes	Aedes aegypti	SPECIES	14.08000	-87.22000	1264887913
8798	Diptera	Culicidae	Aedes	Aedes aegypti	SPECIES	14.08000	-15.55000	1264888412
8799	Diptera	Culicidae	Aedes	Aedes aegypti	SPECIES	14.08000	-87.21000	1264891139
8800	Diptera	Culicidae	Aedes	Aedes aegypti	SPECIES	14.08833	100.64194	4145848750
8801	Diptera	Culicidae	Aedes	Aedes aegypti	SPECIES	14.08833	100.64167	4145853345
8802	Diptera	Culicidae	Aedes	Aedes aegypti	SPECIES	14.09000	101.07000	1264889696
8803	Diptera	Culicidae	Aedes	Aedes aegypti	SPECIES	14.09258	99.13502	4145849249
8804	Diptera	Culicidae	Aedes	Aedes aegypti	SPECIES	14.10000	-87.21000	1264891198
8805	Diptera	Culicidae	Aedes	Aedes aegypti	SPECIES	14.10014	99.08121	4145859928
8806	Diptera	Culicidae	Aedes	Aedes aegypti	SPECIES	14.11000	101.33000	1264887457
8807	Diptera	Culicidae	Aedes	Aedes aegypti	SPECIES	14.11000	-15.55000	1264890392
8808	Diptera	Culicidae	Aedes	Aedes aegypti	SPECIES	14.11000	-89.61000	1264891280
8809	Diptera	Culicidae	Aedes	Aedes aegypti	SPECIES	14.11000	-15.43000	1264891448
8810	Diptera	Culicidae	Aedes	Aedes aegypti	SPECIES	14.11817	99.18498	4145856331
8811	Diptera	Culicidae	Aedes	Aedes aegypti	SPECIES	14.11889	101.78722	4145852568
8812	Diptera	Culicidae	Aedes	Aedes aegypti	SPECIES	14.12000	-15.43000	1264890156
8813	Diptera	Culicidae	Aedes	Aedes aegypti	SPECIES	14.13000	-15.58000	1264891440
8814	Diptera	Culicidae	Aedes	Aedes aegypti	SPECIES	14.13537	99.15720	4145848676
8815	Diptera	Culicidae	Aedes	Aedes aegypti	SPECIES	14.14972	102.91115	4145859080

№	Order	Family	Genus	Species	Taxon Rank	Latitude	Longitude	GBIF_ID
8816	Diptera	Culicidae	Aedes	Aedes aegypti	SPECIES	14.15000	100.64028	4145850864
8817	Diptera	Culicidae	Aedes	Aedes aegypti	SPECIES	14.15000	100.64000	4145858429
8818	Diptera	Culicidae	Aedes	Aedes aegypti	SPECIES	14.16976	99.09884	4145859343
8819	Diptera	Culicidae	Aedes	Aedes aegypti	SPECIES	14.17000	-16.82000	1264887193
8820	Diptera	Culicidae	Aedes	Aedes aegypti	SPECIES	14.18000	-12.72000	1264887838
8821	Diptera	Culicidae	Aedes	Aedes aegypti	SPECIES	14.19000	-12.71000	1264890397
8822	Diptera	Culicidae	Aedes	Aedes aegypti	SPECIES	14.19418	99.08866	4145858814
8823	Diptera	Culicidae	Aedes	Aedes aegypti	SPECIES	14.19522	101.21850	4145853583
8824	Diptera	Culicidae	Aedes	Aedes aegypti	SPECIES	14.20000	101.22000	1264886452
8825	Diptera	Culicidae	Aedes	Aedes aegypti	SPECIES	14.20000	101.12000	1264887546
8826	Diptera	Culicidae	Aedes	Aedes aegypti	SPECIES	14.20000	101.21000	1264887550
8827	Diptera	Culicidae	Aedes	Aedes aegypti	SPECIES	14.20000	101.23000	1264887553
8828	Diptera	Culicidae	Aedes	Aedes aegypti	SPECIES	14.20221	99.23788	4145856735
8829	Diptera	Culicidae	Aedes	Aedes aegypti	SPECIES	14.21554	98.93817	4145857446
8830	Diptera	Culicidae	Aedes	Aedes aegypti	SPECIES	14.21763	99.18506	4145856529
8831	Diptera	Culicidae	Aedes	Aedes aegypti	SPECIES	14.21891	99.33755	4145856951
8832	Diptera	Culicidae	Aedes	Aedes aegypti	SPECIES	14.22591	99.31910	4145857888
8833	Diptera	Culicidae	Aedes	Aedes aegypti	SPECIES	14.23759	99.03120	4145852867
8834	Diptera	Culicidae	Aedes	Aedes aegypti	SPECIES	14.26000	101.13000	1264887563
8835	Diptera	Culicidae	Aedes	Aedes aegypti	SPECIES	14.26000	101.21000	1264887566
8836	Diptera	Culicidae	Aedes	Aedes aegypti	SPECIES	14.27022	99.36118	4145859986
8837	Diptera	Culicidae	Aedes	Aedes aegypti	SPECIES	14.28000	99.28000	1264890450
8838	Diptera	Culicidae	Aedes	Aedes aegypti	SPECIES	14.28000	-14.93000	1264891417
8839	Diptera	Culicidae	Aedes	Aedes aegypti	SPECIES	14.29694	101.02361	4145856565
8840	Diptera	Culicidae	Aedes	Aedes aegypti	SPECIES	14.30000	-90.79000	1264890293
8841	Diptera	Culicidae	Aedes	Aedes aegypti	SPECIES	14.30170	99.20831	4145858503
8842	Diptera	Culicidae	Aedes	Aedes aegypti	SPECIES	14.30514	99.03717	4145851381
8843	Diptera	Culicidae	Aedes	Aedes aegypti	SPECIES	14.31583	101.03139	4145852100
8844	Diptera	Culicidae	Aedes	Aedes aegypti	SPECIES	14.31879	98.94095	4145860429
8845	Diptera	Culicidae	Aedes	Aedes aegypti	SPECIES	14.32000	98.98000	1264887191
8846	Diptera	Culicidae	Aedes	Aedes aegypti	SPECIES	14.32901	99.01841	4145858015
8847	Diptera	Culicidae	Aedes	Aedes aegypti	SPECIES	14.33000	98.98000	1264887818
8848	Diptera	Culicidae	Aedes	Aedes aegypti	SPECIES	14.33525	98.98839	4145852916
8849	Diptera	Culicidae	Aedes	Aedes aegypti	SPECIES	14.33578	98.98814	4145860083
8850	Diptera	Culicidae	Aedes	Aedes aegypti	SPECIES	14.33679	98.98867	4145857767
8851	Diptera	Culicidae	Aedes	Aedes aegypti	SPECIES	14.34000	98.99000	1264886039
8852	Diptera	Culicidae	Aedes	Aedes aegypti	SPECIES	14.34000	99.00000	1264886256
8853	Diptera	Culicidae	Aedes	Aedes aegypti	SPECIES	14.34000	-16.41000	1264887813
8854	Diptera	Culicidae	Aedes	Aedes aegypti	SPECIES	14.34511	100.56246	4145852388
8855	Diptera	Culicidae	Aedes	Aedes aegypti	SPECIES	14.35000	-14.70000	1264891400
8856	Diptera	Culicidae	Aedes	Aedes aegypti	SPECIES	14.36000	-14.68000	1264891399

№	Order	Family	Genus	Species	Taxon Rank	Latitude	Longitude	GBIF_ID
8857	Diptera	Culicidae	Aedes	Aedes aegypti	SPECIES	14.36417	100.66694	4145852447
8858	Diptera	Culicidae	Aedes	Aedes aegypti	SPECIES	14.37000	102.48000	1264887486
8859	Diptera	Culicidae	Aedes	Aedes aegypti	SPECIES	14.38000	-14.69000	1264890098
8860	Diptera	Culicidae	Aedes	Aedes aegypti	SPECIES	14.40000	100.15000	1264886487
8861	Diptera	Culicidae	Aedes	Aedes aegypti	SPECIES	14.41000	121.04000	1264887589
8862	Diptera	Culicidae	Aedes	Aedes aegypti	SPECIES	14.41000	-12.90000	1264891332
8863	Diptera	Culicidae	Aedes	Aedes aegypti	SPECIES	14.41000	-14.80000	1264891386
8864	Diptera	Culicidae	Aedes	Aedes aegypti	SPECIES	14.41000	-14.88000	1264891414
8865	Diptera	Culicidae	Aedes	Aedes aegypti	SPECIES	14.41000	-14.75000	1264891423
8866	Diptera	Culicidae	Aedes	Aedes aegypti	SPECIES	14.42000	101.86000	1264886472
8867	Diptera	Culicidae	Aedes	Aedes aegypti	SPECIES	14.42000	-16.97000	1264890301
8868	Diptera	Culicidae	Aedes	Aedes aegypti	SPECIES	14.42000	-14.75000	1264890531
8869	Diptera	Culicidae	Aedes	Aedes aegypti	SPECIES	14.42000	-14.80000	1264890605
8870	Diptera	Culicidae	Aedes	Aedes aegypti	SPECIES	14.42076	100.90483	226099248
8871	Diptera	Culicidae	Aedes	Aedes aegypti	SPECIES	14.42163	100.90855	226099251
8872	Diptera	Culicidae	Aedes	Aedes aegypti	SPECIES	14.43000	121.03000	1264886623
8873	Diptera	Culicidae	Aedes	Aedes aegypti	SPECIES	14.43000	-60.83000	1264887145
8874	Diptera	Culicidae	Aedes	Aedes aegypti	SPECIES	14.43386	104.06301	4145852880
8875	Diptera	Culicidae	Aedes	Aedes aegypti	SPECIES	14.44000	-60.84000	1264887266
8876	Diptera	Culicidae	Aedes	Aedes aegypti	SPECIES	14.45000	-16.28000	1264888413
8877	Diptera	Culicidae	Aedes	Aedes aegypti	SPECIES	14.45820	99.08319	4145859340
8878	Diptera	Culicidae	Aedes	Aedes aegypti	SPECIES	14.46000	100.37000	1264886463
8879	Diptera	Culicidae	Aedes	Aedes aegypti	SPECIES	14.46844	102.88610	4145850838
8880	Diptera	Culicidae	Aedes	Aedes aegypti	SPECIES	14.47553	98.75291	4145853681
8881	Diptera	Culicidae	Aedes	Aedes aegypti	SPECIES	14.47969	100.11547	4145852056
8882	Diptera	Culicidae	Aedes	Aedes aegypti	SPECIES	14.48000	100.12000	1264886467
8883	Diptera	Culicidae	Aedes	Aedes aegypti	SPECIES	14.48000	-60.98000	1264887135
8884	Diptera	Culicidae	Aedes	Aedes aegypti	SPECIES	14.48000	-16.54000	1264887269
8885	Diptera	Culicidae	Aedes	Aedes aegypti	SPECIES	14.48000	-16.40000	1264889787
8886	Diptera	Culicidae	Aedes	Aedes aegypti	SPECIES	14.48000	-14.98000	1264891477
8887	Diptera	Culicidae	Aedes	Aedes aegypti	SPECIES	14.48070	104.69179	4145859568
8888	Diptera	Culicidae	Aedes	Aedes aegypti	SPECIES	14.49000	120.99000	1264889914
8889	Diptera	Culicidae	Aedes	Aedes aegypti	SPECIES	14.49160	101.54103	4145849646
8890	Diptera	Culicidae	Aedes	Aedes aegypti	SPECIES	14.49769	99.37925	4145849180
8891	Diptera	Culicidae	Aedes	Aedes aegypti	SPECIES	14.50000	-60.98000	1264887137
8892	Diptera	Culicidae	Aedes	Aedes aegypti	SPECIES	14.50290	105.24231	4145857059
8893	Diptera	Culicidae	Aedes	Aedes aegypti	SPECIES	14.50305	104.20874	4145855254
8894	Diptera	Culicidae	Aedes	Aedes aegypti	SPECIES	14.50694	104.21142	4145858502
8895	Diptera	Culicidae	Aedes	Aedes aegypti	SPECIES	14.50726	99.89275	4145850385
8896	Diptera	Culicidae	Aedes	Aedes aegypti	SPECIES	14.51000	-60.98000	1264887282
8897	Diptera	Culicidae	Aedes	Aedes aegypti	SPECIES	14.51000	102.25000	1264891327

№	Order	Family	Genus	Species	Taxon Rank	Latitude	Longitude	GBIF_ID
8898	Diptera	Culicidae	Aedes	Aedes aegypti	SPECIES	14.51766	98.98060	4145855044
8899	Diptera	Culicidae	Aedes	Aedes aegypti	SPECIES	14.52215	-90.55454	3496615680
8900	Diptera	Culicidae	Aedes	Aedes aegypti	SPECIES	14.53000	100.91000	1264886475
8901	Diptera	Culicidae	Aedes	Aedes aegypti	SPECIES	14.53000	-60.98000	1264887141
8902	Diptera	Culicidae	Aedes	Aedes aegypti	SPECIES	14.54000	-60.84000	1264887152
8903	Diptera	Culicidae	Aedes	Aedes aegypti	SPECIES	14.54290	99.37747	4145859419
8904	Diptera	Culicidae	Aedes	Aedes aegypti	SPECIES	14.55000	-60.93000	1264887147
8905	Diptera	Culicidae	Aedes	Aedes aegypti	SPECIES	14.55000	-60.84000	1264887157
8906	Diptera	Culicidae	Aedes	Aedes aegypti	SPECIES	14.55000	121.00000	1264889859
8907	Diptera	Culicidae	Aedes	Aedes aegypti	SPECIES	14.55084	121.06643	4153540565
8908	Diptera	Culicidae	Aedes	Aedes aegypti	SPECIES	14.55121	98.75184	4145859746
8909	Diptera	Culicidae	Aedes	Aedes aegypti	SPECIES	14.55404	104.31203	4145856281
8910	Diptera	Culicidae	Aedes	Aedes aegypti	SPECIES	14.56000	-60.88000	1264887107
8911	Diptera	Culicidae	Aedes	Aedes aegypti	SPECIES	14.56204	98.70789	4145857313
8912	Diptera	Culicidae	Aedes	Aedes aegypti	SPECIES	14.56598	101.23459	4145854318
8913	Diptera	Culicidae	Aedes	Aedes aegypti	SPECIES	14.57000	121.02000	1264889910
8914	Diptera	Culicidae	Aedes	Aedes aegypti	SPECIES	14.57019	98.74532	4145848993
8915	Diptera	Culicidae	Aedes	Aedes aegypti	SPECIES	14.57019	98.74625	4145851989
8916	Diptera	Culicidae	Aedes	Aedes aegypti	SPECIES	14.57569	98.83258	4145850683
8917	Diptera	Culicidae	Aedes	Aedes aegypti	SPECIES	14.58000	-60.98000	1264887117
8918	Diptera	Culicidae	Aedes	Aedes aegypti	SPECIES	14.58000	-60.97000	1264887143
8919	Diptera	Culicidae	Aedes	Aedes aegypti	SPECIES	14.58000	120.99000	1264889456
8920	Diptera	Culicidae	Aedes	Aedes aegypti	SPECIES	14.59000	100.45000	1264886462
8921	Diptera	Culicidae	Aedes	Aedes aegypti	SPECIES	14.59182	105.37403	4145860000
8922	Diptera	Culicidae	Aedes	Aedes aegypti	SPECIES	14.59267	98.97528	4145854354
8923	Diptera	Culicidae	Aedes	Aedes aegypti	SPECIES	14.59825	120.99897	3059278385
8924	Diptera	Culicidae	Aedes	Aedes aegypti	SPECIES	14.60000	-61.05000	1264887128
8925	Diptera	Culicidae	Aedes	Aedes aegypti	SPECIES	14.60000	-61.07000	1264890269
8926	Diptera	Culicidae	Aedes	Aedes aegypti	SPECIES	14.60000	-61.08000	1264891142
8927	Diptera	Culicidae	Aedes	Aedes aegypti	SPECIES	14.60000	-61.00000	1264891195
8928	Diptera	Culicidae	Aedes	Aedes aegypti	SPECIES	14.60917	99.12967	4145851764
8929	Diptera	Culicidae	Aedes	Aedes aegypti	SPECIES	14.61000	121.02000	1264887102
8930	Diptera	Culicidae	Aedes	Aedes aegypti	SPECIES	14.61000	-60.99000	1264887124
8931	Diptera	Culicidae	Aedes	Aedes aegypti	SPECIES	14.61000	-16.41000	1264887528
8932	Diptera	Culicidae	Aedes	Aedes aegypti	SPECIES	14.61176	98.72299	4145859713
8933	Diptera	Culicidae	Aedes	Aedes aegypti	SPECIES	14.61524	104.22271	4145850273
8934	Diptera	Culicidae	Aedes	Aedes aegypti	SPECIES	14.62000	-61.06000	1264887131
8935	Diptera	Culicidae	Aedes	Aedes aegypti	SPECIES	14.62000	-60.98000	1264887153
8936	Diptera	Culicidae	Aedes	Aedes aegypti	SPECIES	14.62000	-60.99000	1264887253
8937	Diptera	Culicidae	Aedes	Aedes aegypti	SPECIES	14.62000	-92.19000	1264887911
8938	Diptera	Culicidae	Aedes	Aedes aegypti	SPECIES	14.62000	-16.31000	1264887961

№	Order	Family	Genus	Species	Taxon Rank	Latitude	Longitude	GBIF_ID
8939	Diptera	Culicidae	Aedes	Aedes aegypti	SPECIES	14.62000	-92.18000	1264888655
8940	Diptera	Culicidae	Aedes	Aedes aegypti	SPECIES	14.62000	-16.30000	1264888999
8941	Diptera	Culicidae	Aedes	Aedes aegypti	SPECIES	14.62589	-90.47176	4046483472
8942	Diptera	Culicidae	Aedes	Aedes aegypti	SPECIES	14.63000	-61.01000	1264887111
8943	Diptera	Culicidae	Aedes	Aedes aegypti	SPECIES	14.63000	-61.03000	1264887123
8944	Diptera	Culicidae	Aedes	Aedes aegypti	SPECIES	14.63000	-90.53000	1264888091
8945	Diptera	Culicidae	Aedes	Aedes aegypti	SPECIES	14.63000	121.03000	1264889010
8946	Diptera	Culicidae	Aedes	Aedes aegypti	SPECIES	14.63000	120.99000	1264889881
8947	Diptera	Culicidae	Aedes	Aedes aegypti	SPECIES	14.63000	-16.44000	1264890282
8948	Diptera	Culicidae	Aedes	Aedes aegypti	SPECIES	14.63000	-16.45000	1264890420
8949	Diptera	Culicidae	Aedes	Aedes aegypti	SPECIES	14.63255	99.36375	4145848865
8950	Diptera	Culicidae	Aedes	Aedes aegypti	SPECIES	14.63444	99.40971	4145858887
8951	Diptera	Culicidae	Aedes	Aedes aegypti	SPECIES	14.63980	98.73967	4145850262
8952	Diptera	Culicidae	Aedes	Aedes aegypti	SPECIES	14.64000	-60.99000	1264887118
8953	Diptera	Culicidae	Aedes	Aedes aegypti	SPECIES	14.64000	120.99000	1264889915
8954	Diptera	Culicidae	Aedes	Aedes aegypti	SPECIES	14.64000	-16.44000	1264890587
8955	Diptera	Culicidae	Aedes	Aedes aegypti	SPECIES	14.64750	100.57478	4145856740
8956	Diptera	Culicidae	Aedes	Aedes aegypti	SPECIES	14.65000	109.06000	1264886183
8957	Diptera	Culicidae	Aedes	Aedes aegypti	SPECIES	14.65485	-61.01929	4050478616
8958	Diptera	Culicidae	Aedes	Aedes aegypti	SPECIES	14.65599	98.66814	4145855841
8959	Diptera	Culicidae	Aedes	Aedes aegypti	SPECIES	14.65691	104.58582	4145856419
8960	Diptera	Culicidae	Aedes	Aedes aegypti	SPECIES	14.66305	100.89298	226099244
8961	Diptera	Culicidae	Aedes	Aedes aegypti	SPECIES	14.66396	100.89299	226099245
8962	Diptera	Culicidae	Aedes	Aedes aegypti	SPECIES	14.66424	100.85772	226099255
8963	Diptera	Culicidae	Aedes	Aedes aegypti	SPECIES	14.66485	100.89393	226099242
8964	Diptera	Culicidae	Aedes	Aedes aegypti	SPECIES	14.66511	100.86144	226099254
8965	Diptera	Culicidae	Aedes	Aedes aegypti	SPECIES	14.67000	-61.04000	1264887108
8966	Diptera	Culicidae	Aedes	Aedes aegypti	SPECIES	14.67000	-61.00000	1264888155
8967	Diptera	Culicidae	Aedes	Aedes aegypti	SPECIES	14.67000	-17.44000	1264890627
8968	Diptera	Culicidae	Aedes	Aedes aegypti	SPECIES	14.67057	98.55782	4145857726
8969	Diptera	Culicidae	Aedes	Aedes aegypti	SPECIES	14.67066	98.72093	4145860228
8970	Diptera	Culicidae	Aedes	Aedes aegypti	SPECIES	14.67863	98.59528	4145852294
8971	Diptera	Culicidae	Aedes	Aedes aegypti	SPECIES	14.67917	-92.15139	4168708755
8972	Diptera	Culicidae	Aedes	Aedes aegypti	SPECIES	14.68000	-92.15000	1264886811
8973	Diptera	Culicidae	Aedes	Aedes aegypti	SPECIES	14.68000	-60.94000	1264886846
8974	Diptera	Culicidae	Aedes	Aedes aegypti	SPECIES	14.68000	-60.98000	1264887120
8975	Diptera	Culicidae	Aedes	Aedes aegypti	SPECIES	14.68000	-61.02000	1264887151
8976	Diptera	Culicidae	Aedes	Aedes aegypti	SPECIES	14.68000	121.04000	1264887605
8977	Diptera	Culicidae	Aedes	Aedes aegypti	SPECIES	14.68000	-17.44000	1264890413
8978	Diptera	Culicidae	Aedes	Aedes aegypti	SPECIES	14.70000	-61.00000	1264887139
8979	Diptera	Culicidae	Aedes	Aedes aegypti	SPECIES	14.70000	-60.99000	1264887246

№	Order	Family	Genus	Species	Taxon Rank	Latitude	Longitude	GBIF_ID
8980	Diptera	Culicidae	Aedes	Aedes aegypti	SPECIES	14.70000	-91.86000	1264888636
8981	Diptera	Culicidae	Aedes	Aedes aegypti	SPECIES	14.70000	-92.25000	1264888709
8982	Diptera	Culicidae	Aedes	Aedes aegypti	SPECIES	14.70139	101.41611	4145852994
8983	Diptera	Culicidae	Aedes	Aedes aegypti	SPECIES	14.70333	101.41611	4145857966
8984	Diptera	Culicidae	Aedes	Aedes aegypti	SPECIES	14.71000	-86.00000	1264891213
8985	Diptera	Culicidae	Aedes	Aedes aegypti	SPECIES	14.71917	-92.42556	4168708863
8986	Diptera	Culicidae	Aedes	Aedes aegypti	SPECIES	14.72000	-92.43000	1264886806
8987	Diptera	Culicidae	Aedes	Aedes aegypti	SPECIES	14.72000	-17.49000	1264887179
8988	Diptera	Culicidae	Aedes	Aedes aegypti	SPECIES	14.72000	-17.27000	1264887800
8989	Diptera	Culicidae	Aedes	Aedes aegypti	SPECIES	14.72500	-92.22500	2629264049
8990	Diptera	Culicidae	Aedes	Aedes aegypti	SPECIES	14.73000	-61.13000	1264887184
8991	Diptera	Culicidae	Aedes	Aedes aegypti	SPECIES	14.73000	-92.42000	1264888663
8992	Diptera	Culicidae	Aedes	Aedes aegypti	SPECIES	14.73000	-60.96000	1264891164
8993	Diptera	Culicidae	Aedes	Aedes aegypti	SPECIES	14.74000	-61.18000	1264887133
8994	Diptera	Culicidae	Aedes	Aedes aegypti	SPECIES	14.74000	46.94000	1264891072
8995	Diptera	Culicidae	Aedes	Aedes aegypti	SPECIES	14.74364	98.63270	4145853213
8996	Diptera	Culicidae	Aedes	Aedes aegypti	SPECIES	14.75000	-17.45000	1264886582
8997	Diptera	Culicidae	Aedes	Aedes aegypti	SPECIES	14.75000	-60.90000	1264887136
8998	Diptera	Culicidae	Aedes	Aedes aegypti	SPECIES	14.75000	-61.18000	1264887150
8999	Diptera	Culicidae	Aedes	Aedes aegypti	SPECIES	14.75000	-17.47000	1264887531
9000	Diptera	Culicidae	Aedes	Aedes aegypti	SPECIES	14.75000	-60.99000	1264889523
9001	Diptera	Culicidae	Aedes	Aedes aegypti	SPECIES	14.75000	-17.42000	1264890298
9002	Diptera	Culicidae	Aedes	Aedes aegypti	SPECIES	14.75851	99.32280	4145854646
9003	Diptera	Culicidae	Aedes	Aedes aegypti	SPECIES	14.75967	99.38252	4145858244
9004	Diptera	Culicidae	Aedes	Aedes aegypti	SPECIES	14.76324	99.32122	4145860512
9005	Diptera	Culicidae	Aedes	Aedes aegypti	SPECIES	14.77000	105.34000	1264887620
9006	Diptera	Culicidae	Aedes	Aedes aegypti	SPECIES	14.77259	99.35892	4145859255
9007	Diptera	Culicidae	Aedes	Aedes aegypti	SPECIES	14.78000	-60.99000	1264887177
9008	Diptera	Culicidae	Aedes	Aedes aegypti	SPECIES	14.79000	102.84000	1264886491
9009	Diptera	Culicidae	Aedes	Aedes aegypti	SPECIES	14.79252	98.67351	4145855127
9010	Diptera	Culicidae	Aedes	Aedes aegypti	SPECIES	14.79311	99.33170	4145851107
9011	Diptera	Culicidae	Aedes	Aedes aegypti	SPECIES	14.80000	100.65000	1264886444
9012	Diptera	Culicidae	Aedes	Aedes aegypti	SPECIES	14.80000	-61.10000	1264887144
9013	Diptera	Culicidae	Aedes	Aedes aegypti	SPECIES	14.81000	-16.83000	1264890323
9014	Diptera	Culicidae	Aedes	Aedes aegypti	SPECIES	14.82000	-92.20000	1264886657
9015	Diptera	Culicidae	Aedes	Aedes aegypti	SPECIES	14.83000	-61.11000	1264887138
9016	Diptera	Culicidae	Aedes	Aedes aegypti	SPECIES	14.83000	43.18000	1264891064
9017	Diptera	Culicidae	Aedes	Aedes aegypti	SPECIES	14.83611	-92.19111	4168708636
9018	Diptera	Culicidae	Aedes	Aedes aegypti	SPECIES	14.84000	-92.19000	1264886012
9019	Diptera	Culicidae	Aedes	Aedes aegypti	SPECIES	14.84000	105.11000	1264887631
9020	Diptera	Culicidae	Aedes	Aedes aegypti	SPECIES	14.85000	-92.32000	1264886803

№	Order	Family	Genus	Species	Taxon Rank	Latitude	Longitude	GBIF_ID
9021	Diptera	Culicidae	Aedes	Aedes aegypti	SPECIES	14.85500	-92.34083	2629263823
9022	Diptera	Culicidae	Aedes	Aedes aegypti	SPECIES	14.86000	-92.45000	1264886826
9023	Diptera	Culicidae	Aedes	Aedes aegypti	SPECIES	14.86000	-15.88000	1264887549
9024	Diptera	Culicidae	Aedes	Aedes aegypti	SPECIES	14.86250	-92.45000	4168708513
9025	Diptera	Culicidae	Aedes	Aedes aegypti	SPECIES	14.87000	-92.43000	1264886064
9026	Diptera	Culicidae	Aedes	Aedes aegypti	SPECIES	14.87000	-15.90000	1264890408
9027	Diptera	Culicidae	Aedes	Aedes aegypti	SPECIES	14.87000	-15.89000	1264890664
9028	Diptera	Culicidae	Aedes	Aedes aegypti	SPECIES	14.88000	-92.23000	1264886073
9029	Diptera	Culicidae	Aedes	Aedes aegypti	SPECIES	14.88889	-92.48111	2629264254
9030	Diptera	Culicidae	Aedes	Aedes aegypti	SPECIES	14.89000	100.40000	1264886445
9031	Diptera	Culicidae	Aedes	Aedes aegypti	SPECIES	14.89000	-92.48000	1264887603
9032	Diptera	Culicidae	Aedes	Aedes aegypti	SPECIES	14.89053	103.48440	4145859585
9033	Diptera	Culicidae	Aedes	Aedes aegypti	SPECIES	14.89167	-92.24167	2629265163
9034	Diptera	Culicidae	Aedes	Aedes aegypti	SPECIES	14.90000	-92.25000	1264886842
9035	Diptera	Culicidae	Aedes	Aedes aegypti	SPECIES	14.90000	104.92000	1264887654
9036	Diptera	Culicidae	Aedes	Aedes aegypti	SPECIES	14.90000	-92.27000	1264889668
9037	Diptera	Culicidae	Aedes	Aedes aegypti	SPECIES	14.90806	-92.26056	1421475774
9038	Diptera	Culicidae	Aedes	Aedes aegypti	SPECIES	14.90806	-92.26056	4168708918
9039	Diptera	Culicidae	Aedes	Aedes aegypti	SPECIES	14.90806	-92.26057	1421476012
9040	Diptera	Culicidae	Aedes	Aedes aegypti	SPECIES	14.91000	-92.26000	1264887614
9041	Diptera	Culicidae	Aedes	Aedes aegypti	SPECIES	14.91000	-92.24000	1264888733
9042	Diptera	Culicidae	Aedes	Aedes aegypti	SPECIES	14.92000	-16.99000	1264887805
9043	Diptera	Culicidae	Aedes	Aedes aegypti	SPECIES	14.92050	13.43410	3030818397
9044	Diptera	Culicidae	Aedes	Aedes aegypti	SPECIES	14.92080	13.43480	3030357879
9045	Diptera	Culicidae	Aedes	Aedes aegypti	SPECIES	14.92090	13.43470	3030808579
9046	Diptera	Culicidae	Aedes	Aedes aegypti	SPECIES	14.92110	13.43490	3030818519
9047	Diptera	Culicidae	Aedes	Aedes aegypti	SPECIES	14.92292	-92.25158	4168708947
9048	Diptera	Culicidae	Aedes	Aedes aegypti	SPECIES	14.92500	-92.27500	2629264188
9049	Diptera	Culicidae	Aedes	Aedes aegypti	SPECIES	14.93889	-92.16806	2629265559
9050	Diptera	Culicidae	Aedes	Aedes aegypti	SPECIES	14.94000	-92.17000	1264886321
9051	Diptera	Culicidae	Aedes	Aedes aegypti	SPECIES	14.94071	98.61563	4145860354
9052	Diptera	Culicidae	Aedes	Aedes aegypti	SPECIES	14.95000	102.03000	1264887190
9053	Diptera	Culicidae	Aedes	Aedes aegypti	SPECIES	14.95909	98.91040	4145849047
9054	Diptera	Culicidae	Aedes	Aedes aegypti	SPECIES	14.96333	-92.14722	4168708834
9055	Diptera	Culicidae	Aedes	Aedes aegypti	SPECIES	14.97000	101.59000	1264887500
9056	Diptera	Culicidae	Aedes	Aedes aegypti	SPECIES	14.97000	-92.15000	1264888666
9057	Diptera	Culicidae	Aedes	Aedes aegypti	SPECIES	14.97115	102.08397	4145850506
9058	Diptera	Culicidae	Aedes	Aedes aegypti	SPECIES	14.97417	98.62022	4145854000
9059	Diptera	Culicidae	Aedes	Aedes aegypti	SPECIES	14.98000	-92.17000	1264887915
9060	Diptera	Culicidae	Aedes	Aedes aegypti	SPECIES	14.98917	-92.16528	4168709006
9061	Diptera	Culicidae	Aedes	Aedes aegypti	SPECIES	14.99002	-92.16520	1894399442

№	Order	Family	Genus	Species	Taxon Rank	Latitude	Longitude	GBIF_ID
9062	Diptera	Culicidae	Aedes	Aedes aegypti	SPECIES	15.00000	102.83000	1264886494
9063	Diptera	Culicidae	Aedes	Aedes aegypti	SPECIES	15.01917	-92.38083	4168708406
9064	Diptera	Culicidae	Aedes	Aedes aegypti	SPECIES	15.02000	-92.38000	1264886793
9065	Diptera	Culicidae	Aedes	Aedes aegypti	SPECIES	15.02080	103.56707	4145849599
9066	Diptera	Culicidae	Aedes	Aedes aegypti	SPECIES	15.02500	-92.10833	2629264500
9067	Diptera	Culicidae	Aedes	Aedes aegypti	SPECIES	15.03000	100.03000	1264889009
9068	Diptera	Culicidae	Aedes	Aedes aegypti	SPECIES	15.04000	105.23000	1264887628
9069	Diptera	Culicidae	Aedes	Aedes aegypti	SPECIES	15.05000	100.01000	1264889623
9070	Diptera	Culicidae	Aedes	Aedes aegypti	SPECIES	15.06000	99.05000	1264889649
9071	Diptera	Culicidae	Aedes	Aedes aegypti	SPECIES	15.07000	102.06000	1264888964
9072	Diptera	Culicidae	Aedes	Aedes aegypti	SPECIES	15.07000	102.03000	1264888987
9073	Diptera	Culicidae	Aedes	Aedes aegypti	SPECIES	15.07783	104.29922	4145849035
9074	Diptera	Culicidae	Aedes	Aedes aegypti	SPECIES	15.08000	104.84000	1264887634
9075	Diptera	Culicidae	Aedes	Aedes aegypti	SPECIES	15.08000	99.05000	1264889690
9076	Diptera	Culicidae	Aedes	Aedes aegypti	SPECIES	15.08000	100.01000	1264889714
9077	Diptera	Culicidae	Aedes	Aedes aegypti	SPECIES	15.08000	101.90000	1264890467
9078	Diptera	Culicidae	Aedes	Aedes aegypti	SPECIES	15.10000	104.58000	1264885988
9079	Diptera	Culicidae	Aedes	Aedes aegypti	SPECIES	15.12000	105.32000	1264887623
9080	Diptera	Culicidae	Aedes	Aedes aegypti	SPECIES	15.13000	104.58000	1264886003
9081	Diptera	Culicidae	Aedes	Aedes aegypti	SPECIES	15.13000	104.20000	1264886482
9082	Diptera	Culicidae	Aedes	Aedes aegypti	SPECIES	15.13000	-92.47000	1264888388
9083	Diptera	Culicidae	Aedes	Aedes aegypti	SPECIES	15.13889	-92.46583	4168708955
9084	Diptera	Culicidae	Aedes	Aedes aegypti	SPECIES	15.14000	76.92000	1264889204
9085	Diptera	Culicidae	Aedes	Aedes aegypti	SPECIES	15.14000	-92.47000	1264889293
9086	Diptera	Culicidae	Aedes	Aedes aegypti	SPECIES	15.14000	145.72000	1264890089
9087	Diptera	Culicidae	Aedes	Aedes aegypti	SPECIES	15.14499	120.58870	1933070522
9088	Diptera	Culicidae	Aedes	Aedes aegypti	SPECIES	15.14583	-92.42222	4168708383
9089	Diptera	Culicidae	Aedes	Aedes aegypti	SPECIES	15.17000	99.32000	1264886505
9090	Diptera	Culicidae	Aedes	Aedes aegypti	SPECIES	15.18000	104.86000	1264887645
9091	Diptera	Culicidae	Aedes	Aedes aegypti	SPECIES	15.18000	-92.88000	1264888660
9092	Diptera	Culicidae	Aedes	Aedes aegypti	SPECIES	15.18139	100.12833	4145855764
9093	Diptera	Culicidae	Aedes	Aedes aegypti	SPECIES	15.18167	100.12806	4145851048
9094	Diptera	Culicidae	Aedes	Aedes aegypti	SPECIES	15.18414	98.32660	4145855170
9095	Diptera	Culicidae	Aedes	Aedes aegypti	SPECIES	15.20222	100.09833	4145858620
9096	Diptera	Culicidae	Aedes	Aedes aegypti	SPECIES	15.21981	102.49224	4145854781
9097	Diptera	Culicidae	Aedes	Aedes aegypti	SPECIES	15.22041	99.56608	4145857325
9098	Diptera	Culicidae	Aedes	Aedes aegypti	SPECIES	15.23000	103.50000	1264886514
9099	Diptera	Culicidae	Aedes	Aedes aegypti	SPECIES	15.23000	-61.37000	1264890283
9100	Diptera	Culicidae	Aedes	Aedes aegypti	SPECIES	15.24000	101.75000	1264887490
9101	Diptera	Culicidae	Aedes	Aedes aegypti	SPECIES	15.24000	100.37000	1264887520
9102	Diptera	Culicidae	Aedes	Aedes aegypti	SPECIES	15.24000	104.85000	1264887652

№	Order	Family	Genus	Species	Taxon Rank	Latitude	Longitude	GBIF_ID
9103	Diptera	Culicidae	Aedes	Aedes aegypti	SPECIES	15.26000	104.89000	1264887627
9104	Diptera	Culicidae	Aedes	Aedes aegypti	SPECIES	15.26000	-61.37000	1264890279
9105	Diptera	Culicidae	Aedes	Aedes aegypti	SPECIES	15.28000	-14.28000	1264888432
9106	Diptera	Culicidae	Aedes	Aedes aegypti	SPECIES	15.28000	-14.87000	1264890517
9107	Diptera	Culicidae	Aedes	Aedes aegypti	SPECIES	15.28139	-92.69028	4168709019
9108	Diptera	Culicidae	Aedes	Aedes aegypti	SPECIES	15.28231	-61.38828	177059776
9109	Diptera	Culicidae	Aedes	Aedes aegypti	SPECIES	15.29142	-61.39752	177060539
9110	Diptera	Culicidae	Aedes	Aedes aegypti	SPECIES	15.30000	-61.39000	1264889745
9111	Diptera	Culicidae	Aedes	Aedes aegypti	SPECIES	15.30000	-61.37000	4066961839
9112	Diptera	Culicidae	Aedes	Aedes aegypti	SPECIES	15.30895	99.72006	4145854622
9113	Diptera	Culicidae	Aedes	Aedes aegypti	SPECIES	15.30942	99.72014	4145851360
9114	Diptera	Culicidae	Aedes	Aedes aegypti	SPECIES	15.31944	-92.65833	2629263803
9115	Diptera	Culicidae	Aedes	Aedes aegypti	SPECIES	15.32000	-92.66000	1264886792
9116	Diptera	Culicidae	Aedes	Aedes aegypti	SPECIES	15.33000	108.74000	1264886109
9117	Diptera	Culicidae	Aedes	Aedes aegypti	SPECIES	15.33000	108.67000	1264890217
9118	Diptera	Culicidae	Aedes	Aedes aegypti	SPECIES	15.33944	-92.67417	4168708922
9119	Diptera	Culicidae	Aedes	Aedes aegypti	SPECIES	15.34000	-61.39000	1264889781
9120	Diptera	Culicidae	Aedes	Aedes aegypti	SPECIES	15.35359	104.72243	4145850619
9121	Diptera	Culicidae	Aedes	Aedes aegypti	SPECIES	15.36239	99.93022	4145859914
9122	Diptera	Culicidae	Aedes	Aedes aegypti	SPECIES	15.36400	99.93158	4145852128
9123	Diptera	Culicidae	Aedes	Aedes aegypti	SPECIES	15.36417	-92.24806	2629263953
9124	Diptera	Culicidae	Aedes	Aedes aegypti	SPECIES	15.37000	-92.25000	1264886829
9125	Diptera	Culicidae	Aedes	Aedes aegypti	SPECIES	15.37178	-61.26650	177059777
9126	Diptera	Culicidae	Aedes	Aedes aegypti	SPECIES	15.37639	100.01861	4145852003
9127	Diptera	Culicidae	Aedes	Aedes aegypti	SPECIES	15.37639	100.01667	4145855998
9128	Diptera	Culicidae	Aedes	Aedes aegypti	SPECIES	15.37678	100.10507	4145854772
9129	Diptera	Culicidae	Aedes	Aedes aegypti	SPECIES	15.38000	100.02000	1264886431
9130	Diptera	Culicidae	Aedes	Aedes aegypti	SPECIES	15.38306	100.01111	4145859755
9131	Diptera	Culicidae	Aedes	Aedes aegypti	SPECIES	15.38442	100.02896	4145850317
9132	Diptera	Culicidae	Aedes	Aedes aegypti	SPECIES	15.38836	100.09551	4145857809
9133	Diptera	Culicidae	Aedes	Aedes aegypti	SPECIES	15.40000	74.02000	1264886060
9134	Diptera	Culicidae	Aedes	Aedes aegypti	SPECIES	15.40000	-87.82000	1264889685
9135	Diptera	Culicidae	Aedes	Aedes aegypti	SPECIES	15.40156	104.76239	4145852210
9136	Diptera	Culicidae	Aedes	Aedes aegypti	SPECIES	15.41000	108.68000	1264886134
9137	Diptera	Culicidae	Aedes	Aedes aegypti	SPECIES	15.41459	100.09019	4145853141
9138	Diptera	Culicidae	Aedes	Aedes aegypti	SPECIES	15.43000	-92.90000	1264886796
9139	Diptera	Culicidae	Aedes	Aedes aegypti	SPECIES	15.44167	-92.89167	4168709027
9140	Diptera	Culicidae	Aedes	Aedes aegypti	SPECIES	15.45000	120.79000	1264891252
9141	Diptera	Culicidae	Aedes	Aedes aegypti	SPECIES	15.48000	120.96000	1264891239
9142	Diptera	Culicidae	Aedes	Aedes aegypti	SPECIES	15.49000	99.83000	1264886499
9143	Diptera	Culicidae	Aedes	Aedes aegypti	SPECIES	15.49000	73.82000	1264889270

№	Order	Family	Genus	Species	Taxon Rank	Latitude	Longitude	GBIF_ID
9144	Diptera	Culicidae	Aedes	Aedes aegypti	SPECIES	15.50000	120.85000	1264891196
9145	Diptera	Culicidae	Aedes	Aedes aegypti	SPECIES	15.50000	120.84000	1264891231
9146	Diptera	Culicidae	Aedes	Aedes aegypti	SPECIES	15.55683	100.07208	4145853204
9147	Diptera	Culicidae	Aedes	Aedes aegypti	SPECIES	15.56100	-88.00065	2988306077
9148	Diptera	Culicidae	Aedes	Aedes aegypti	SPECIES	15.57000	-61.32000	1264889763
9149	Diptera	Culicidae	Aedes	Aedes aegypti	SPECIES	15.57587	101.85793	4145851572
9150	Diptera	Culicidae	Aedes	Aedes aegypti	SPECIES	15.58000	100.01000	1264887507
9151	Diptera	Culicidae	Aedes	Aedes aegypti	SPECIES	15.58000	102.69000	1264887512
9152	Diptera	Culicidae	Aedes	Aedes aegypti	SPECIES	15.59586	101.90228	4145856045
9153	Diptera	Culicidae	Aedes	Aedes aegypti	SPECIES	15.60000	121.05000	1264891246
9154	Diptera	Culicidae	Aedes	Aedes aegypti	SPECIES	15.61000	105.05000	1264887663
9155	Diptera	Culicidae	Aedes	Aedes aegypti	SPECIES	15.62000	-16.22000	1264887529
9156	Diptera	Culicidae	Aedes	Aedes aegypti	SPECIES	15.62000	-16.25000	1264890402
9157	Diptera	Culicidae	Aedes	Aedes aegypti	SPECIES	15.63977	99.93910	226099234
9158	Diptera	Culicidae	Aedes	Aedes aegypti	SPECIES	15.65688	105.36191	4145854196
9159	Diptera	Culicidae	Aedes	Aedes aegypti	SPECIES	15.66000	101.11000	1264886489
9160	Diptera	Culicidae	Aedes	Aedes aegypti	SPECIES	15.66583	-96.49028	1894399046
9161	Diptera	Culicidae	Aedes	Aedes aegypti	SPECIES	15.67000	100.08000	1264886492
9162	Diptera	Culicidae	Aedes	Aedes aegypti	SPECIES	15.67000	103.82000	1264886504
9163	Diptera	Culicidae	Aedes	Aedes aegypti	SPECIES	15.67000	102.21000	1264887502
9164	Diptera	Culicidae	Aedes	Aedes aegypti	SPECIES	15.68000	-93.22000	1264888694
9165	Diptera	Culicidae	Aedes	Aedes aegypti	SPECIES	15.68000	-88.58000	1264889537
9166	Diptera	Culicidae	Aedes	Aedes aegypti	SPECIES	15.68000	-88.61000	1264889540
9167	Diptera	Culicidae	Aedes	Aedes aegypti	SPECIES	15.68000	100.16000	1264891052
9168	Diptera	Culicidae	Aedes	Aedes aegypti	SPECIES	15.68553	-96.23858	2629262517
9169	Diptera	Culicidae	Aedes	Aedes aegypti	SPECIES	15.68667	-93.20917	4168708382
9170	Diptera	Culicidae	Aedes	Aedes aegypti	SPECIES	15.69000	-93.21000	1264886807
9171	Diptera	Culicidae	Aedes	Aedes aegypti	SPECIES	15.69000	100.12000	1264887534
9172	Diptera	Culicidae	Aedes	Aedes aegypti	SPECIES	15.69000	-88.61000	1264889553
9173	Diptera	Culicidae	Aedes	Aedes aegypti	SPECIES	15.69517	105.50666	4145851579
9174	Diptera	Culicidae	Aedes	Aedes aegypti	SPECIES	15.70000	99.39000	1264887513
9175	Diptera	Culicidae	Aedes	Aedes aegypti	SPECIES	15.70000	100.12000	1264887657
9176	Diptera	Culicidae	Aedes	Aedes aegypti	SPECIES	15.70000	-88.58000	1264889524
9177	Diptera	Culicidae	Aedes	Aedes aegypti	SPECIES	15.71000	100.14000	1264889174
9178	Diptera	Culicidae	Aedes	Aedes aegypti	SPECIES	15.71000	-88.57000	1264889556
9179	Diptera	Culicidae	Aedes	Aedes aegypti	SPECIES	15.72000	105.17000	1264887621
9180	Diptera	Culicidae	Aedes	Aedes aegypti	SPECIES	15.72000	-88.64000	1264889517
9181	Diptera	Culicidae	Aedes	Aedes aegypti	SPECIES	15.72000	-88.55000	1264889544
9182	Diptera	Culicidae	Aedes	Aedes aegypti	SPECIES	15.72000	-88.57000	1264889551
9183	Diptera	Culicidae	Aedes	Aedes aegypti	SPECIES	15.72000	-15.33000	1264890586
9184	Diptera	Culicidae	Aedes	Aedes aegypti	SPECIES	15.72583	-96.54250	1894396891

№	Order	Family	Genus	Species	Taxon Rank	Latitude	Longitude	GBIF_ID
9185	Diptera	Culicidae	Aedes	Aedes aegypti	SPECIES	15.73000	-88.59000	1264889534
9186	Diptera	Culicidae	Aedes	Aedes aegypti	SPECIES	15.74444	-96.46528	1894398041
9187	Diptera	Culicidae	Aedes	Aedes aegypti	SPECIES	15.74774	101.25110	4145860448
9188	Diptera	Culicidae	Aedes	Aedes aegypti	SPECIES	15.75000	-96.47000	1264886599
9189	Diptera	Culicidae	Aedes	Aedes aegypti	SPECIES	15.75000	-96.46000	1264888671
9190	Diptera	Culicidae	Aedes	Aedes aegypti	SPECIES	15.75000	-88.58000	1264889548
9191	Diptera	Culicidae	Aedes	Aedes aegypti	SPECIES	15.76889	-96.13500	1894396384
9192	Diptera	Culicidae	Aedes	Aedes aegypti	SPECIES	15.77000	44.04000	1264891060
9193	Diptera	Culicidae	Aedes	Aedes aegypti	SPECIES	15.77889	104.30611	4145849306
9194	Diptera	Culicidae	Aedes	Aedes aegypti	SPECIES	15.77917	104.30611	4145857065
9195	Diptera	Culicidae	Aedes	Aedes aegypti	SPECIES	15.77944	104.30583	4145853785
9196	Diptera	Culicidae	Aedes	Aedes aegypti	SPECIES	15.78722	-96.46028	1894396088
9197	Diptera	Culicidae	Aedes	Aedes aegypti	SPECIES	15.78889	104.14333	4145849023
9198	Diptera	Culicidae	Aedes	Aedes aegypti	SPECIES	15.78889	104.14389	4145858313
9199	Diptera	Culicidae	Aedes	Aedes aegypti	SPECIES	15.79000	108.32000	1264886234
9200	Diptera	Culicidae	Aedes	Aedes aegypti	SPECIES	15.80522	101.25232	4145853869
9201	Diptera	Culicidae	Aedes	Aedes aegypti	SPECIES	15.80554	102.04277	4145855843
9202	Diptera	Culicidae	Aedes	Aedes aegypti	SPECIES	15.81000	102.02000	1264886477
9203	Diptera	Culicidae	Aedes	Aedes aegypti	SPECIES	15.81336	100.89312	4145856997
9204	Diptera	Culicidae	Aedes	Aedes aegypti	SPECIES	15.82000	99.32000	1264887521
9205	Diptera	Culicidae	Aedes	Aedes aegypti	SPECIES	15.82000	78.04000	1264887928
9206	Diptera	Culicidae	Aedes	Aedes aegypti	SPECIES	15.82778	-96.46389	1894398872
9207	Diptera	Culicidae	Aedes	Aedes aegypti	SPECIES	15.83000	78.05000	1264890913
9208	Diptera	Culicidae	Aedes	Aedes aegypti	SPECIES	15.83417	-96.32222	1894399661
9209	Diptera	Culicidae	Aedes	Aedes aegypti	SPECIES	15.84419	100.07561	4145858683
9210	Diptera	Culicidae	Aedes	Aedes aegypti	SPECIES	15.85000	73.62000	1264886727
9211	Diptera	Culicidae	Aedes	Aedes aegypti	SPECIES	15.85384	102.06685	4145857158
9212	Diptera	Culicidae	Aedes	Aedes aegypti	SPECIES	15.86000	-97.06000	1264888681
9213	Diptera	Culicidae	Aedes	Aedes aegypti	SPECIES	15.86194	-97.07167	2629265251
9214	Diptera	Culicidae	Aedes	Aedes aegypti	SPECIES	15.86250	-97.06667	1894400038
9215	Diptera	Culicidae	Aedes	Aedes aegypti	SPECIES	15.86861	-96.47861	1894398506
9216	Diptera	Culicidae	Aedes	Aedes aegypti	SPECIES	15.87000	73.78000	1264886713
9217	Diptera	Culicidae	Aedes	Aedes aegypti	SPECIES	15.87000	-97.08000	1264886761
9218	Diptera	Culicidae	Aedes	Aedes aegypti	SPECIES	15.87000	-97.07000	1264888774
9219	Diptera	Culicidae	Aedes	Aedes aegypti	SPECIES	15.87111	104.66167	4145860322
9220	Diptera	Culicidae	Aedes	Aedes aegypti	SPECIES	15.87139	104.66694	4145852533
9221	Diptera	Culicidae	Aedes	Aedes aegypti	SPECIES	15.88000	105.55000	1264886600
9222	Diptera	Culicidae	Aedes	Aedes aegypti	SPECIES	15.89861	-96.69111	1894396597
9223	Diptera	Culicidae	Aedes	Aedes aegypti	SPECIES	15.90000	73.82000	1264890544
9224	Diptera	Culicidae	Aedes	Aedes aegypti	SPECIES	15.90722	-96.52861	1894399888
9225	Diptera	Culicidae	Aedes	Aedes aegypti	SPECIES	15.91000	73.82000	1264886690

№	Order	Family	Genus	Species	Taxon Rank	Latitude	Longitude	GBIF_ID
9226	Diptera	Culicidae	Aedes	Aedes aegypti	SPECIES	15.92000	105.52000	1264886529
9227	Diptera	Culicidae	Aedes	Aedes aegypti	SPECIES	15.92000	120.34000	1264891228
9228	Diptera	Culicidae	Aedes	Aedes aegypti	SPECIES	15.92083	-97.12083	1894396630
9229	Diptera	Culicidae	Aedes	Aedes aegypti	SPECIES	15.92083	-97.89306	1894399670
9230	Diptera	Culicidae	Aedes	Aedes aegypti	SPECIES	15.94417	-93.37444	1894400009
9231	Diptera	Culicidae	Aedes	Aedes aegypti	SPECIES	15.95707	-61.31676	2826192675
9232	Diptera	Culicidae	Aedes	Aedes aegypti	SPECIES	15.97000	120.78000	1264891267
9233	Diptera	Culicidae	Aedes	Aedes aegypti	SPECIES	15.99034	99.76231	4129517428
9234	Diptera	Culicidae	Aedes	Aedes aegypti	SPECIES	16.00000	99.80000	1264886533
9235	Diptera	Culicidae	Aedes	Aedes aegypti	SPECIES	16.01111	-97.43333	1894394060
9236	Diptera	Culicidae	Aedes	Aedes aegypti	SPECIES	16.01667	-95.88889	1894399482
9237	Diptera	Culicidae	Aedes	Aedes aegypti	SPECIES	16.01678	103.98296	4145855069
9238	Diptera	Culicidae	Aedes	Aedes aegypti	SPECIES	16.02000	-16.49000	1264890411
9239	Diptera	Culicidae	Aedes	Aedes aegypti	SPECIES	16.03000	108.19000	1264886158
9240	Diptera	Culicidae	Aedes	Aedes aegypti	SPECIES	16.03000	-61.70000	1264886830
9241	Diptera	Culicidae	Aedes	Aedes aegypti	SPECIES	16.03000	-16.50000	1264887516
9242	Diptera	Culicidae	Aedes	Aedes aegypti	SPECIES	16.03000	108.01000	3349414749
9243	Diptera	Culicidae	Aedes	Aedes aegypti	SPECIES	16.03919	100.37844	4145852805
9244	Diptera	Culicidae	Aedes	Aedes aegypti	SPECIES	16.04120	104.66834	4145855737
9245	Diptera	Culicidae	Aedes	Aedes aegypti	SPECIES	16.04659	-61.62316	3848555707
9246	Diptera	Culicidae	Aedes	Aedes aegypti	SPECIES	16.06000	73.47000	1264886708
9247	Diptera	Culicidae	Aedes	Aedes aegypti	SPECIES	16.06000	73.46000	1264890559
9248	Diptera	Culicidae	Aedes	Aedes aegypti	SPECIES	16.06144	103.64500	4145853405
9249	Diptera	Culicidae	Aedes	Aedes aegypti	SPECIES	16.06342	103.64644	4145849222
9250	Diptera	Culicidae	Aedes	Aedes aegypti	SPECIES	16.07000	103.52000	1264889858
9251	Diptera	Culicidae	Aedes	Aedes aegypti	SPECIES	16.08639	-93.75500	4168708589
9252	Diptera	Culicidae	Aedes	Aedes aegypti	SPECIES	16.09000	-93.75000	1264888650
9253	Diptera	Culicidae	Aedes	Aedes aegypti	SPECIES	16.09286	100.25686	4145859814
9254	Diptera	Culicidae	Aedes	Aedes aegypti	SPECIES	16.11752	-88.79998	226099171
9255	Diptera	Culicidae	Aedes	Aedes aegypti	SPECIES	16.12000	102.53000	1264886554
9256	Diptera	Culicidae	Aedes	Aedes aegypti	SPECIES	16.13000	108.12000	1264886187
9257	Diptera	Culicidae	Aedes	Aedes aegypti	SPECIES	16.13886	104.72489	4145860109
9258	Diptera	Culicidae	Aedes	Aedes aegypti	SPECIES	16.15000	119.98000	1264891236
9259	Diptera	Culicidae	Aedes	Aedes aegypti	SPECIES	16.16230	120.42700	3059294343
9260	Diptera	Culicidae	Aedes	Aedes aegypti	SPECIES	16.16331	103.59138	4145853895
9261	Diptera	Culicidae	Aedes	Aedes aegypti	SPECIES	16.17000	102.47000	1264886566
9262	Diptera	Culicidae	Aedes	Aedes aegypti	SPECIES	16.17528	-95.15750	1421474390
9263	Diptera	Culicidae	Aedes	Aedes aegypti	SPECIES	16.18000	-15.75000	1264887558
9264	Diptera	Culicidae	Aedes	Aedes aegypti	SPECIES	16.18250	-95.19583	1894399737
9265	Diptera	Culicidae	Aedes	Aedes aegypti	SPECIES	16.18290	103.31084	4145858574
9266	Diptera	Culicidae	Aedes	Aedes aegypti	SPECIES	16.19000	-61.59000	1264886822

№	Order	Family	Genus	Species	Taxon Rank	Latitude	Longitude	GBIF_ID
9267	Diptera	Culicidae	Aedes	Aedes aegypti	SPECIES	16.19759	-61.53977	4050436631
9268	Diptera	Culicidae	Aedes	Aedes aegypti	SPECIES	16.19940	-91.33310	2979620565
9269	Diptera	Culicidae	Aedes	Aedes aegypti	SPECIES	16.20000	-15.69000	1264890393
9270	Diptera	Culicidae	Aedes	Aedes aegypti	SPECIES	16.20278	-97.93056	1894399137
9271	Diptera	Culicidae	Aedes	Aedes aegypti	SPECIES	16.20556	-94.07639	1894399771
9272	Diptera	Culicidae	Aedes	Aedes aegypti	SPECIES	16.21028	-94.12222	1894398915
9273	Diptera	Culicidae	Aedes	Aedes aegypti	SPECIES	16.23389	-93.89861	4168708656
9274	Diptera	Culicidae	Aedes	Aedes aegypti	SPECIES	16.23833	-98.18944	1894397633
9275	Diptera	Culicidae	Aedes	Aedes aegypti	SPECIES	16.23982	-92.16659	2629263164
9276	Diptera	Culicidae	Aedes	Aedes aegypti	SPECIES	16.24000	-61.53000	1264886834
9277	Diptera	Culicidae	Aedes	Aedes aegypti	SPECIES	16.25000	121.12000	1264891240
9278	Diptera	Culicidae	Aedes	Aedes aegypti	SPECIES	16.25294	103.23025	4145851024
9279	Diptera	Culicidae	Aedes	Aedes aegypti	SPECIES	16.25972	-61.57702	177060499
9280	Diptera	Culicidae	Aedes	Aedes aegypti	SPECIES	16.26000	101.15000	1264891057
9281	Diptera	Culicidae	Aedes	Aedes aegypti	SPECIES	16.27000	108.02000	1264886121
9282	Diptera	Culicidae	Aedes	Aedes aegypti	SPECIES	16.27000	-61.58000	1264886855
9283	Diptera	Culicidae	Aedes	Aedes aegypti	SPECIES	16.27917	-94.35333	1894399972
9284	Diptera	Culicidae	Aedes	Aedes aegypti	SPECIES	16.28056	-97.82333	1894398481
9285	Diptera	Culicidae	Aedes	Aedes aegypti	SPECIES	16.29028	-94.50222	1894399044
9286	Diptera	Culicidae	Aedes	Aedes aegypti	SPECIES	16.30194	-97.91333	1894396682
9287	Diptera	Culicidae	Aedes	Aedes aegypti	SPECIES	16.32000	102.78000	1264886496
9288	Diptera	Culicidae	Aedes	Aedes aegypti	SPECIES	16.32000	-95.23000	1264887905
9289	Diptera	Culicidae	Aedes	Aedes aegypti	SPECIES	16.32333	103.64644	4145856761
9290	Diptera	Culicidae	Aedes	Aedes aegypti	SPECIES	16.32444	-95.23889	1894399158
9291	Diptera	Culicidae	Aedes	Aedes aegypti	SPECIES	16.32889	-97.91778	1894400298
9292	Diptera	Culicidae	Aedes	Aedes aegypti	SPECIES	16.33000	-98.03000	1264888685
9293	Diptera	Culicidae	Aedes	Aedes aegypti	SPECIES	16.33806	-92.56389	1894399623
9294	Diptera	Culicidae	Aedes	Aedes aegypti	SPECIES	16.34167	-98.05222	1894399585
9295	Diptera	Culicidae	Aedes	Aedes aegypti	SPECIES	16.34444	-98.21944	1894395082
9296	Diptera	Culicidae	Aedes	Aedes aegypti	SPECIES	16.35083	-94.48250	1894400049
9297	Diptera	Culicidae	Aedes	Aedes aegypti	SPECIES	16.35472	-94.19167	1894399545
9298	Diptera	Culicidae	Aedes	Aedes aegypti	SPECIES	16.36000	99.65000	1264886320
9299	Diptera	Culicidae	Aedes	Aedes aegypti	SPECIES	16.36194	-94.20389	1894396436
9300	Diptera	Culicidae	Aedes	Aedes aegypti	SPECIES	16.36694	-94.19444	1894400200
9301	Diptera	Culicidae	Aedes	Aedes aegypti	SPECIES	16.37000	-94.20000	1264886608
9302	Diptera	Culicidae	Aedes	Aedes aegypti	SPECIES	16.37000	73.38000	1264886718
9303	Diptera	Culicidae	Aedes	Aedes aegypti	SPECIES	16.37000	99.63000	1264890530
9304	Diptera	Culicidae	Aedes	Aedes aegypti	SPECIES	16.37639	-94.19833	1894398937
9305	Diptera	Culicidae	Aedes	Aedes aegypti	SPECIES	16.37659	103.58879	4145849474
9306	Diptera	Culicidae	Aedes	Aedes aegypti	SPECIES	16.38000	-94.21000	1264888700
9307	Diptera	Culicidae	Aedes	Aedes aegypti	SPECIES	16.38889	-98.10972	1894397249

№	Order	Family	Genus	Species	Taxon Rank	Latitude	Longitude	GBIF_ID
9308	Diptera	Culicidae	Aedes	Aedes aegypti	SPECIES	16.39639	-99.19250	1894398507
9309	Diptera	Culicidae	Aedes	Aedes aegypti	SPECIES	16.39722	-97.90139	1894399417
9310	Diptera	Culicidae	Aedes	Aedes aegypti	SPECIES	16.39944	-95.76444	1894399458
9311	Diptera	Culicidae	Aedes	Aedes aegypti	SPECIES	16.40000	102.79000	1264886390
9312	Diptera	Culicidae	Aedes	Aedes aegypti	SPECIES	16.40000	103.37000	1264886478
9313	Diptera	Culicidae	Aedes	Aedes aegypti	SPECIES	16.40000	-94.95000	1264886592
9314	Diptera	Culicidae	Aedes	Aedes aegypti	SPECIES	16.40000	99.37000	1264890523
9315	Diptera	Culicidae	Aedes	Aedes aegypti	SPECIES	16.40000	-94.45750	1894400123
9316	Diptera	Culicidae	Aedes	Aedes aegypti	SPECIES	16.41000	102.85000	1264886032
9317	Diptera	Culicidae	Aedes	Aedes aegypti	SPECIES	16.41028	-95.01861	1894397506
9318	Diptera	Culicidae	Aedes	Aedes aegypti	SPECIES	16.41444	102.85153	4145850914
9319	Diptera	Culicidae	Aedes	Aedes aegypti	SPECIES	16.42000	102.83000	1264887551
9320	Diptera	Culicidae	Aedes	Aedes aegypti	SPECIES	16.42000	101.16000	1264887641
9321	Diptera	Culicidae	Aedes	Aedes aegypti	SPECIES	16.42083	103.50861	4145852005
9322	Diptera	Culicidae	Aedes	Aedes aegypti	SPECIES	16.42389	102.86667	4145854273
9323	Diptera	Culicidae	Aedes	Aedes aegypti	SPECIES	16.42861	-95.82500	1894397763
9324	Diptera	Culicidae	Aedes	Aedes aegypti	SPECIES	16.43000	99.61000	1264886361
9325	Diptera	Culicidae	Aedes	Aedes aegypti	SPECIES	16.43000	102.82000	1264887483
9326	Diptera	Culicidae	Aedes	Aedes aegypti	SPECIES	16.43000	102.84000	1264889675
9327	Diptera	Culicidae	Aedes	Aedes aegypti	SPECIES	16.43000	-95.02000	1264890536
9328	Diptera	Culicidae	Aedes	Aedes aegypti	SPECIES	16.43194	-94.26333	1894397686
9329	Diptera	Culicidae	Aedes	Aedes aegypti	SPECIES	16.43333	-95.01944	1894399951
9330	Diptera	Culicidae	Aedes	Aedes aegypti	SPECIES	16.43667	103.49556	4145858954
9331	Diptera	Culicidae	Aedes	Aedes aegypti	SPECIES	16.44000	102.84000	1264886447
9332	Diptera	Culicidae	Aedes	Aedes aegypti	SPECIES	16.44000	100.35000	1264886455
9333	Diptera	Culicidae	Aedes	Aedes aegypti	SPECIES	16.44971	101.09710	4145850988
9334	Diptera	Culicidae	Aedes	Aedes aegypti	SPECIES	16.45000	99.40000	1264889077
9335	Diptera	Culicidae	Aedes	Aedes aegypti	SPECIES	16.45278	-98.01444	1894398718
9336	Diptera	Culicidae	Aedes	Aedes aegypti	SPECIES	16.45778	-96.19111	1894394954
9337	Diptera	Culicidae	Aedes	Aedes aegypti	SPECIES	16.45833	-98.07639	1894394951
9338	Diptera	Culicidae	Aedes	Aedes aegypti	SPECIES	16.46000	99.53000	1264886420
9339	Diptera	Culicidae	Aedes	Aedes aegypti	SPECIES	16.46892	99.53664	4145859763
9340	Diptera	Culicidae	Aedes	Aedes aegypti	SPECIES	16.47000	-94.35000	1264886613
9341	Diptera	Culicidae	Aedes	Aedes aegypti	SPECIES	16.47000	-15.68000	1264887823
9342	Diptera	Culicidae	Aedes	Aedes aegypti	SPECIES	16.47000	-94.36000	1264888684
9343	Diptera	Culicidae	Aedes	Aedes aegypti	SPECIES	16.47000	99.53000	1264890409
9344	Diptera	Culicidae	Aedes	Aedes aegypti	SPECIES	16.47194	-96.10333	1894399177
9345	Diptera	Culicidae	Aedes	Aedes aegypti	SPECIES	16.47500	-94.82972	1894398835
9346	Diptera	Culicidae	Aedes	Aedes aegypti	SPECIES	16.47839	-94.34464	1421474960
9347	Diptera	Culicidae	Aedes	Aedes aegypti	SPECIES	16.47917	-94.35167	1894400143
9348	Diptera	Culicidae	Aedes	Aedes aegypti	SPECIES	16.48000	99.45000	1264886962

№	Order	Family	Genus	Species	Taxon Rank	Latitude	Longitude	GBIF_ID
9349	Diptera	Culicidae	Aedes	Aedes aegypti	SPECIES	16.48000	99.52000	1264887705
9350	Diptera	Culicidae	Aedes	Aedes aegypti	SPECIES	16.48472	-95.04167	1894400122
9351	Diptera	Culicidae	Aedes	Aedes aegypti	SPECIES	16.49000	97.63000	1264890133
9352	Diptera	Culicidae	Aedes	Aedes aegypti	SPECIES	16.49500	-96.10611	1894399709
9353	Diptera	Culicidae	Aedes	Aedes aegypti	SPECIES	16.50000	99.50000	1264890579
9354	Diptera	Culicidae	Aedes	Aedes aegypti	SPECIES	16.50111	-95.90639	1894399127
9355	Diptera	Culicidae	Aedes	Aedes aegypti	SPECIES	16.50194	-95.05917	1894396290
9356	Diptera	Culicidae	Aedes	Aedes aegypti	SPECIES	16.50892	101.15700	4145855238
9357	Diptera	Culicidae	Aedes	Aedes aegypti	SPECIES	16.50972	-98.34722	1894396121
9358	Diptera	Culicidae	Aedes	Aedes aegypti	SPECIES	16.51250	-96.03889	1894396112
9359	Diptera	Culicidae	Aedes	Aedes aegypti	SPECIES	16.52000	99.50000	1264886153
9360	Diptera	Culicidae	Aedes	Aedes aegypti	SPECIES	16.52000	107.48000	1264886257
9361	Diptera	Culicidae	Aedes	Aedes aegypti	SPECIES	16.53142	103.05919	4145855420
9362	Diptera	Culicidae	Aedes	Aedes aegypti	SPECIES	16.53944	-92.47361	1894394959
9363	Diptera	Culicidae	Aedes	Aedes aegypti	SPECIES	16.54806	104.72306	4145849196
9364	Diptera	Culicidae	Aedes	Aedes aegypti	SPECIES	16.55000	73.34000	1264886714
9365	Diptera	Culicidae	Aedes	Aedes aegypti	SPECIES	16.55000	73.33000	1264890589
9366	Diptera	Culicidae	Aedes	Aedes aegypti	SPECIES	16.55278	-94.94722	1894398132
9367	Diptera	Culicidae	Aedes	Aedes aegypti	SPECIES	16.55278	-96.02639	1894399979
9368	Diptera	Culicidae	Aedes	Aedes aegypti	SPECIES	16.56167	-93.90639	1894400204
9369	Diptera	Culicidae	Aedes	Aedes aegypti	SPECIES	16.56278	-95.10000	1894399487
9370	Diptera	Culicidae	Aedes	Aedes aegypti	SPECIES	16.58000	104.41944	4145852462
9371	Diptera	Culicidae	Aedes	Aedes aegypti	SPECIES	16.58028	104.42000	4145856780
9372	Diptera	Culicidae	Aedes	Aedes aegypti	SPECIES	16.60778	-95.97833	1894400245
9373	Diptera	Culicidae	Aedes	Aedes aegypti	SPECIES	16.61278	-98.15556	1894399378
9374	Diptera	Culicidae	Aedes	Aedes aegypti	SPECIES	16.62000	-98.63000	1264888697
9375	Diptera	Culicidae	Aedes	Aedes aegypti	SPECIES	16.62083	-98.12389	1894397302
9376	Diptera	Culicidae	Aedes	Aedes aegypti	SPECIES	16.62222	-96.03167	1894398631
9377	Diptera	Culicidae	Aedes	Aedes aegypti	SPECIES	16.62500	-97.84722	1894399188
9378	Diptera	Culicidae	Aedes	Aedes aegypti	SPECIES	16.63000	103.90000	1264886521
9379	Diptera	Culicidae	Aedes	Aedes aegypti	SPECIES	16.63667	-98.03056	1894398627
9380	Diptera	Culicidae	Aedes	Aedes aegypti	SPECIES	16.64000	73.36000	1264886686
9381	Diptera	Culicidae	Aedes	Aedes aegypti	SPECIES	16.65278	-98.08944	1894399846
9382	Diptera	Culicidae	Aedes	Aedes aegypti	SPECIES	16.65417	-95.90972	1894399905
9383	Diptera	Culicidae	Aedes	Aedes aegypti	SPECIES	16.65778	-96.09722	1894395510
9384	Diptera	Culicidae	Aedes	Aedes aegypti	SPECIES	16.66000	73.52000	1264890571
9385	Diptera	Culicidae	Aedes	Aedes aegypti	SPECIES	16.66028	-95.99861	1894399149
9386	Diptera	Culicidae	Aedes	Aedes aegypti	SPECIES	16.66944	-96.26667	1894399099
9387	Diptera	Culicidae	Aedes	Aedes aegypti	SPECIES	16.68000	74.46000	1264889236
9388	Diptera	Culicidae	Aedes	Aedes aegypti	SPECIES	16.68167	99.28775	4145851988
9389	Diptera	Culicidae	Aedes	Aedes aegypti	SPECIES	16.68528	-93.10417	1894399962

№	Order	Family	Genus	Species	Taxon Rank	Latitude	Longitude	GBIF_ID
9390	Diptera	Culicidae	Aedes	Aedes aegypti	SPECIES	16.69500	-93.72167	1894398913
9391	Diptera	Culicidae	Aedes	Aedes aegypti	SPECIES	16.70000	107.23000	1264886208
9392	Diptera	Culicidae	Aedes	Aedes aegypti	SPECIES	16.70000	74.23000	1264889257
9393	Diptera	Culicidae	Aedes	Aedes aegypti	SPECIES	16.70000	-62.22000	1264890268
9394	Diptera	Culicidae	Aedes	Aedes aegypti	SPECIES	16.70186	99.21214	4145859300
9395	Diptera	Culicidae	Aedes	Aedes aegypti	SPECIES	16.70599	-62.21901	177059989
9396	Diptera	Culicidae	Aedes	Aedes aegypti	SPECIES	16.70869	-62.21712	177059987
9397	Diptera	Culicidae	Aedes	Aedes aegypti	SPECIES	16.71000	-62.21000	1264890096
9398	Diptera	Culicidae	Aedes	Aedes aegypti	SPECIES	16.71000	98.57000	1264890519
9399	Diptera	Culicidae	Aedes	Aedes aegypti	SPECIES	16.71000	98.56000	1264891154
9400	Diptera	Culicidae	Aedes	Aedes aegypti	SPECIES	16.72042	101.18381	4145859964
9401	Diptera	Culicidae	Aedes	Aedes aegypti	SPECIES	16.73607	99.94937	4145857772
9402	Diptera	Culicidae	Aedes	Aedes aegypti	SPECIES	16.73756	-93.07783	2629265525
9403	Diptera	Culicidae	Aedes	Aedes aegypti	SPECIES	16.74000	98.52000	1264889128
9404	Diptera	Culicidae	Aedes	Aedes aegypti	SPECIES	16.74185	100.62445	4145855926
9405	Diptera	Culicidae	Aedes	Aedes aegypti	SPECIES	16.74772	-93.10861	2629265236
9406	Diptera	Culicidae	Aedes	Aedes aegypti	SPECIES	16.75000	73.32000	1264886751
9407	Diptera	Culicidae	Aedes	Aedes aegypti	SPECIES	16.75000	98.58000	1264889689
9408	Diptera	Culicidae	Aedes	Aedes aegypti	SPECIES	16.75000	98.55000	1264890374
9409	Diptera	Culicidae	Aedes	Aedes aegypti	SPECIES	16.75000	98.57000	1264890581
9410	Diptera	Culicidae	Aedes	Aedes aegypti	SPECIES	16.75278	-93.11667	1894400254
9411	Diptera	Culicidae	Aedes	Aedes aegypti	SPECIES	16.76111	-97.98889	1894397527
9412	Diptera	Culicidae	Aedes	Aedes aegypti	SPECIES	16.76548	101.19361	4145855702
9413	Diptera	Culicidae	Aedes	Aedes aegypti	SPECIES	16.77000	94.73000	1264890125
9414	Diptera	Culicidae	Aedes	Aedes aegypti	SPECIES	16.78000	96.15000	1264887613
9415	Diptera	Culicidae	Aedes	Aedes aegypti	SPECIES	16.78000	96.18000	1264889073
9416	Diptera	Culicidae	Aedes	Aedes aegypti	SPECIES	16.78000	96.17000	1264889084
9417	Diptera	Culicidae	Aedes	Aedes aegypti	SPECIES	16.78000	96.16000	1264891169
9418	Diptera	Culicidae	Aedes	Aedes aegypti	SPECIES	16.78000	96.11000	1264891185
9419	Diptera	Culicidae	Aedes	Aedes aegypti	SPECIES	16.78472	-93.27222	1894397853
9420	Diptera	Culicidae	Aedes	Aedes aegypti	SPECIES	16.78472	-98.06056	1894400142
9421	Diptera	Culicidae	Aedes	Aedes aegypti	SPECIES	16.80000	96.16000	1264889091
9422	Diptera	Culicidae	Aedes	Aedes aegypti	SPECIES	16.80111	-95.07222	1894396753
9423	Diptera	Culicidae	Aedes	Aedes aegypti	SPECIES	16.80389	-95.11222	1894397463
9424	Diptera	Culicidae	Aedes	Aedes aegypti	SPECIES	16.81000	107.12000	1264886221
9425	Diptera	Culicidae	Aedes	Aedes aegypti	SPECIES	16.81000	96.17000	1264889901
9426	Diptera	Culicidae	Aedes	Aedes aegypti	SPECIES	16.82000	100.26000	1264886469
9427	Diptera	Culicidae	Aedes	Aedes aegypti	SPECIES	16.82000	100.27000	1264887651
9428	Diptera	Culicidae	Aedes	Aedes aegypti	SPECIES	16.82000	98.61000	1264889124
9429	Diptera	Culicidae	Aedes	Aedes aegypti	SPECIES	16.82000	98.62000	1264889146
9430	Diptera	Culicidae	Aedes	Aedes aegypti	SPECIES	16.82000	74.64000	1264889221

№	Order	Family	Genus	Species	Taxon Rank	Latitude	Longitude	GBIF_ID
9431	Diptera	Culicidae	Aedes	Aedes aegypti	SPECIES	16.82000	96.18000	1264889919
9432	Diptera	Culicidae	Aedes	Aedes aegypti	SPECIES	16.82000	99.75000	1264891352
9433	Diptera	Culicidae	Aedes	Aedes aegypti	SPECIES	16.82167	-95.11972	1894397151
9434	Diptera	Culicidae	Aedes	Aedes aegypti	SPECIES	16.83000	96.12000	1264889081
9435	Diptera	Culicidae	Aedes	Aedes aegypti	SPECIES	16.83000	96.20000	1264889094
9436	Diptera	Culicidae	Aedes	Aedes aegypti	SPECIES	16.84135	100.17594	4145856657
9437	Diptera	Culicidae	Aedes	Aedes aegypti	SPECIES	16.84754	100.51086	4145856916
9438	Diptera	Culicidae	Aedes	Aedes aegypti	SPECIES	16.85000	-99.91000	1264889022
9439	Diptera	Culicidae	Aedes	Aedes aegypti	SPECIES	16.85000	96.19000	1264889108
9440	Diptera	Culicidae	Aedes	Aedes aegypti	SPECIES	16.86000	98.62000	1264888693
9441	Diptera	Culicidae	Aedes	Aedes aegypti	SPECIES	16.86000	74.57000	1264889223
9442	Diptera	Culicidae	Aedes	Aedes aegypti	SPECIES	16.87000	96.14000	1264889068
9443	Diptera	Culicidae	Aedes	Aedes aegypti	SPECIES	16.87111	-99.89778	1894399404
9444	Diptera	Culicidae	Aedes	Aedes aegypti	SPECIES	16.87222	-95.04167	1894399334
9445	Diptera	Culicidae	Aedes	Aedes aegypti	SPECIES	16.87797	100.19953	4145855616
9446	Diptera	Culicidae	Aedes	Aedes aegypti	SPECIES	16.88000	-99.86000	1264887422
9447	Diptera	Culicidae	Aedes	Aedes aegypti	SPECIES	16.88000	99.13000	1264887670
9448	Diptera	Culicidae	Aedes	Aedes aegypti	SPECIES	16.88000	98.62000	1264888792
9449	Diptera	Culicidae	Aedes	Aedes aegypti	SPECIES	16.88640	100.84155	4145854124
9450	Diptera	Culicidae	Aedes	Aedes aegypti	SPECIES	16.88908	99.12617	4145851715
9451	Diptera	Culicidae	Aedes	Aedes aegypti	SPECIES	16.89000	-99.85000	1264886329
9452	Diptera	Culicidae	Aedes	Aedes aegypti	SPECIES	16.89000	98.64000	1264887695
9453	Diptera	Culicidae	Aedes	Aedes aegypti	SPECIES	16.89858	104.18205	4145858433
9454	Diptera	Culicidae	Aedes	Aedes aegypti	SPECIES	16.89936	100.80284	4145853761
9455	Diptera	Culicidae	Aedes	Aedes aegypti	SPECIES	16.90000	103.47000	1264886530
9456	Diptera	Culicidae	Aedes	Aedes aegypti	SPECIES	16.91090	104.47324	4145860151
9457	Diptera	Culicidae	Aedes	Aedes aegypti	SPECIES	16.91340	100.75170	4145855301
9458	Diptera	Culicidae	Aedes	Aedes aegypti	SPECIES	16.92000	73.93000	1264889250
9459	Diptera	Culicidae	Aedes	Aedes aegypti	SPECIES	16.92000	99.98000	1264891348
9460	Diptera	Culicidae	Aedes	Aedes aegypti	SPECIES	16.92222	-96.36250	1894398135
9461	Diptera	Culicidae	Aedes	Aedes aegypti	SPECIES	16.92609	103.72841	4145852373
9462	Diptera	Culicidae	Aedes	Aedes aegypti	SPECIES	16.93596	103.23355	4145859916
9463	Diptera	Culicidae	Aedes	Aedes aegypti	SPECIES	16.95361	-96.47583	1894399382
9464	Diptera	Culicidae	Aedes	Aedes aegypti	SPECIES	16.95703	102.19898	4145857602
9465	Diptera	Culicidae	Aedes	Aedes aegypti	SPECIES	16.96167	-95.09306	1894399325
9466	Diptera	Culicidae	Aedes	Aedes aegypti	SPECIES	16.96748	-88.21663	226099175
9467	Diptera	Culicidae	Aedes	Aedes aegypti	SPECIES	16.97130	100.96035	4145848786
9468	Diptera	Culicidae	Aedes	Aedes aegypti	SPECIES	16.97639	-96.61528	1894397999
9469	Diptera	Culicidae	Aedes	Aedes aegypti	SPECIES	16.98000	81.78000	1264889132
9470	Diptera	Culicidae	Aedes	Aedes aegypti	SPECIES	16.98722	-95.01333	1894395350
9471	Diptera	Culicidae	Aedes	Aedes aegypti	SPECIES	16.99000	73.31000	1264886741

№	Order	Family	Genus	Species	Taxon Rank	Latitude	Longitude	GBIF_ID
9472	Diptera	Culicidae	Aedes	Aedes aegypti	SPECIES	16.99000	73.29000	1264890552
9473	Diptera	Culicidae	Aedes	Aedes aegypti	SPECIES	16.99000	73.48000	1264890591
9474	Diptera	Culicidae	Aedes	Aedes aegypti	SPECIES	16.99306	-95.03722	1894396623
9475	Diptera	Culicidae	Aedes	Aedes aegypti	SPECIES	17.00000	-96.72000	1264887214
9476	Diptera	Culicidae	Aedes	Aedes aegypti	SPECIES	17.00000	-100.09000	1264888022
9477	Diptera	Culicidae	Aedes	Aedes aegypti	SPECIES	17.00611	-96.63278	1894398633
9478	Diptera	Culicidae	Aedes	Aedes aegypti	SPECIES	17.00694	-96.58333	1894395577
9479	Diptera	Culicidae	Aedes	Aedes aegypti	SPECIES	17.00694	-96.59583	1894399692
9480	Diptera	Culicidae	Aedes	Aedes aegypti	SPECIES	17.00722	-100.08750	2629263569
9481	Diptera	Culicidae	Aedes	Aedes aegypti	SPECIES	17.01000	99.82000	1264886425
9482	Diptera	Culicidae	Aedes	Aedes aegypti	SPECIES	17.01000	-100.09000	1264888730
9483	Diptera	Culicidae	Aedes	Aedes aegypti	SPECIES	17.01000	81.79000	1264889978
9484	Diptera	Culicidae	Aedes	Aedes aegypti	SPECIES	17.01000	99.79000	1264891364
9485	Diptera	Culicidae	Aedes	Aedes aegypti	SPECIES	17.01028	-95.10694	1894400159
9486	Diptera	Culicidae	Aedes	Aedes aegypti	SPECIES	17.01250	-95.03750	1894398689
9487	Diptera	Culicidae	Aedes	Aedes aegypti	SPECIES	17.01583	-96.54000	1894397338
9488	Diptera	Culicidae	Aedes	Aedes aegypti	SPECIES	17.01656	99.70992	4145857438
9489	Diptera	Culicidae	Aedes	Aedes aegypti	SPECIES	17.01740	-61.76680	177059593
9490	Diptera	Culicidae	Aedes	Aedes aegypti	SPECIES	17.02000	73.41000	1264886734
9491	Diptera	Culicidae	Aedes	Aedes aegypti	SPECIES	17.02169	99.59292	4145852387
9492	Diptera	Culicidae	Aedes	Aedes aegypti	SPECIES	17.02186	99.70761	4145857099
9493	Diptera	Culicidae	Aedes	Aedes aegypti	SPECIES	17.02225	103.83365	4145858122
9494	Diptera	Culicidae	Aedes	Aedes aegypti	SPECIES	17.02583	-97.92917	1894400207
9495	Diptera	Culicidae	Aedes	Aedes aegypti	SPECIES	17.02833	-96.70056	1894397370
9496	Diptera	Culicidae	Aedes	Aedes aegypti	SPECIES	17.02917	-96.52000	1894396065
9497	Diptera	Culicidae	Aedes	Aedes aegypti	SPECIES	17.03000	-96.73000	1264887225
9498	Diptera	Culicidae	Aedes	Aedes aegypti	SPECIES	17.03000	99.48000	1264891346
9499	Diptera	Culicidae	Aedes	Aedes aegypti	SPECIES	17.03491	-61.82399	177059853
9500	Diptera	Culicidae	Aedes	Aedes aegypti	SPECIES	17.03889	-97.94028	1894396850
9501	Diptera	Culicidae	Aedes	Aedes aegypti	SPECIES	17.03901	103.59543	4145854591
9502	Diptera	Culicidae	Aedes	Aedes aegypti	SPECIES	17.04000	73.27000	1264886739
9503	Diptera	Culicidae	Aedes	Aedes aegypti	SPECIES	17.04170	-61.74879	177059797
9504	Diptera	Culicidae	Aedes	Aedes aegypti	SPECIES	17.04957	-61.70740	177059841
9505	Diptera	Culicidae	Aedes	Aedes aegypti	SPECIES	17.05000	107.03000	1264886148
9506	Diptera	Culicidae	Aedes	Aedes aegypti	SPECIES	17.05000	-96.72000	1264886580
9507	Diptera	Culicidae	Aedes	Aedes aegypti	SPECIES	17.05517	-61.88964	177059779
9508	Diptera	Culicidae	Aedes	Aedes aegypti	SPECIES	17.05667	-94.64694	1894399292
9509	Diptera	Culicidae	Aedes	Aedes aegypti	SPECIES	17.05915	-61.79565	177059858
9510	Diptera	Culicidae	Aedes	Aedes aegypti	SPECIES	17.06000	-96.72000	1264886597
9511	Diptera	Culicidae	Aedes	Aedes aegypti	SPECIES	17.06000	-96.73000	1264886604
9512	Diptera	Culicidae	Aedes	Aedes aegypti	SPECIES	17.06000	96.27000	1264891309

№	Order	Family	Genus	Species	Taxon Rank	Latitude	Longitude	GBIF_ID
9513	Diptera	Culicidae	Aedes	Aedes aegypti	SPECIES	17.06111	-95.01611	1894397586
9514	Diptera	Culicidae	Aedes	Aedes aegypti	SPECIES	17.06661	-61.83413	177059850
9515	Diptera	Culicidae	Aedes	Aedes aegypti	SPECIES	17.06770	-61.86701	177059831
9516	Diptera	Culicidae	Aedes	Aedes aegypti	SPECIES	17.06778	-96.72000	1894399628
9517	Diptera	Culicidae	Aedes	Aedes aegypti	SPECIES	17.06889	-95.02611	1894398576
9518	Diptera	Culicidae	Aedes	Aedes aegypti	SPECIES	17.06907	-61.79183	177059820
9519	Diptera	Culicidae	Aedes	Aedes aegypti	SPECIES	17.07000	-96.73000	1264886621
9520	Diptera	Culicidae	Aedes	Aedes aegypti	SPECIES	17.07000	-96.70000	1264887200
9521	Diptera	Culicidae	Aedes	Aedes aegypti	SPECIES	17.07000	-61.79000	1264889760
9522	Diptera	Culicidae	Aedes	Aedes aegypti	SPECIES	17.07000	-96.72000	1264889876
9523	Diptera	Culicidae	Aedes	Aedes aegypti	SPECIES	17.07222	-95.04583	1894395272
9524	Diptera	Culicidae	Aedes	Aedes aegypti	SPECIES	17.07318	-96.72659	3343991581
9525	Diptera	Culicidae	Aedes	Aedes aegypti	SPECIES	17.07779	-61.78986	225864831
9526	Diptera	Culicidae	Aedes	Aedes aegypti	SPECIES	17.09289	103.81186	4145849374
9527	Diptera	Culicidae	Aedes	Aedes aegypti	SPECIES	17.09345	-61.78791	177059802
9528	Diptera	Culicidae	Aedes	Aedes aegypti	SPECIES	17.09361	-93.21056	1894400061
9529	Diptera	Culicidae	Aedes	Aedes aegypti	SPECIES	17.09528	-94.89500	1894398259
9530	Diptera	Culicidae	Aedes	Aedes aegypti	SPECIES	17.09737	-61.84052	177059801
9531	Diptera	Culicidae	Aedes	Aedes aegypti	SPECIES	17.10253	102.94072	4145848423
9532	Diptera	Culicidae	Aedes	Aedes aegypti	SPECIES	17.10350	103.00956	3457078566
9533	Diptera	Culicidae	Aedes	Aedes aegypti	SPECIES	17.11000	-61.85000	1264889773
9534	Diptera	Culicidae	Aedes	Aedes aegypti	SPECIES	17.11756	99.85219	4145852783
9535	Diptera	Culicidae	Aedes	Aedes aegypti	SPECIES	17.11803	-61.81783	177059829
9536	Diptera	Culicidae	Aedes	Aedes aegypti	SPECIES	17.12000	101.86000	1264886486
9537	Diptera	Culicidae	Aedes	Aedes aegypti	SPECIES	17.12000	-61.85000	1264890276
9538	Diptera	Culicidae	Aedes	Aedes aegypti	SPECIES	17.12385	101.86309	4145848405
9539	Diptera	Culicidae	Aedes	Aedes aegypti	SPECIES	17.12898	-61.83562	177059791
9540	Diptera	Culicidae	Aedes	Aedes aegypti	SPECIES	17.12917	-97.60694	1894397550
9541	Diptera	Culicidae	Aedes	Aedes aegypti	SPECIES	17.13010	-62.58577	177059944
9542	Diptera	Culicidae	Aedes	Aedes aegypti	SPECIES	17.13094	-61.86287	177059787
9543	Diptera	Culicidae	Aedes	Aedes aegypti	SPECIES	17.13096	103.63334	4145854567
9544	Diptera	Culicidae	Aedes	Aedes aegypti	SPECIES	17.13380	-62.63089	177059966
9545	Diptera	Culicidae	Aedes	Aedes aegypti	SPECIES	17.13750	-95.05972	1894398989
9546	Diptera	Culicidae	Aedes	Aedes aegypti	SPECIES	17.15000	-61.80000	1264889741
9547	Diptera	Culicidae	Aedes	Aedes aegypti	SPECIES	17.15289	-61.75369	177059842
9548	Diptera	Culicidae	Aedes	Aedes aegypti	SPECIES	17.15380	-61.75556	177059843
9549	Diptera	Culicidae	Aedes	Aedes aegypti	SPECIES	17.15654	-61.75930	177059847
9550	Diptera	Culicidae	Aedes	Aedes aegypti	SPECIES	17.16000	-61.85000	1264887599
9551	Diptera	Culicidae	Aedes	Aedes aegypti	SPECIES	17.16000	99.71000	1264891307
9552	Diptera	Culicidae	Aedes	Aedes aegypti	SPECIES	17.16444	-94.78750	1894396071
9553	Diptera	Culicidae	Aedes	Aedes aegypti	SPECIES	17.17932	102.43290	4145848680

№	Order	Family	Genus	Species	Taxon Rank	Latitude	Longitude	GBIF_ID
9554	Diptera	Culicidae	Aedes	Aedes aegypti	SPECIES	17.18000	-96.77000	1264887207
9555	Diptera	Culicidae	Aedes	Aedes aegypti	SPECIES	17.18000	73.55000	1264890548
9556	Diptera	Culicidae	Aedes	Aedes aegypti	SPECIES	17.19000	73.55000	1264886745
9557	Diptera	Culicidae	Aedes	Aedes aegypti	SPECIES	17.19667	-95.21111	1894399809
9558	Diptera	Culicidae	Aedes	Aedes aegypti	SPECIES	17.19889	-95.04917	1894399847
9559	Diptera	Culicidae	Aedes	Aedes aegypti	SPECIES	17.22000	-100.63000	1264886317
9560	Diptera	Culicidae	Aedes	Aedes aegypti	SPECIES	17.22333	102.44478	4145860064
9561	Diptera	Culicidae	Aedes	Aedes aegypti	SPECIES	17.23830	99.85968	4145858188
9562	Diptera	Culicidae	Aedes	Aedes aegypti	SPECIES	17.29000	-62.69000	1264887454
9563	Diptera	Culicidae	Aedes	Aedes aegypti	SPECIES	17.29000	74.18000	1264889252
9564	Diptera	Culicidae	Aedes	Aedes aegypti	SPECIES	17.29000	73.22000	1264890556
9565	Diptera	Culicidae	Aedes	Aedes aegypti	SPECIES	17.30000	-62.73000	1264889757
9566	Diptera	Culicidae	Aedes	Aedes aegypti	SPECIES	17.30000	98.20000	1264890060
9567	Diptera	Culicidae	Aedes	Aedes aegypti	SPECIES	17.30000	99.83000	1264891371
9568	Diptera	Culicidae	Aedes	Aedes aegypti	SPECIES	17.30083	-94.99750	1894394294
9569	Diptera	Culicidae	Aedes	Aedes aegypti	SPECIES	17.31019	102.63544	4145859903
9570	Diptera	Culicidae	Aedes	Aedes aegypti	SPECIES	17.32097	101.99389	4145857151
9571	Diptera	Culicidae	Aedes	Aedes aegypti	SPECIES	17.32917	-95.05083	1894397440
9572	Diptera	Culicidae	Aedes	Aedes aegypti	SPECIES	17.33000	96.50000	1264890167
9573	Diptera	Culicidae	Aedes	Aedes aegypti	SPECIES	17.33000	99.95000	1264891341
9574	Diptera	Culicidae	Aedes	Aedes aegypti	SPECIES	17.34028	-95.37222	1894395629
9575	Diptera	Culicidae	Aedes	Aedes aegypti	SPECIES	17.34250	-95.31861	1894398503
9576	Diptera	Culicidae	Aedes	Aedes aegypti	SPECIES	17.34360	100.38224	4145858757
9577	Diptera	Culicidae	Aedes	Aedes aegypti	SPECIES	17.34917	-95.39861	1894396593
9578	Diptera	Culicidae	Aedes	Aedes aegypti	SPECIES	17.35000	-95.04806	1894399791
9579	Diptera	Culicidae	Aedes	Aedes aegypti	SPECIES	17.35333	-95.41389	1894399402
9580	Diptera	Culicidae	Aedes	Aedes aegypti	SPECIES	17.36283	101.75008	4145858749
9581	Diptera	Culicidae	Aedes	Aedes aegypti	SPECIES	17.37000	99.57000	1264891353
9582	Diptera	Culicidae	Aedes	Aedes aegypti	SPECIES	17.39250	-95.16528	1894400353
9583	Diptera	Culicidae	Aedes	Aedes aegypti	SPECIES	17.39589	102.78131	4145859975
9584	Diptera	Culicidae	Aedes	Aedes aegypti	SPECIES	17.40108	102.78656	4145852487
9585	Diptera	Culicidae	Aedes	Aedes aegypti	SPECIES	17.42000	102.79000	1264886457
9586	Diptera	Culicidae	Aedes	Aedes aegypti	SPECIES	17.43528	-95.31306	1894400083
9587	Diptera	Culicidae	Aedes	Aedes aegypti	SPECIES	17.44306	-95.32917	1894399367
9588	Diptera	Culicidae	Aedes	Aedes aegypti	SPECIES	17.44528	-95.19722	1894397979
9589	Diptera	Culicidae	Aedes	Aedes aegypti	SPECIES	17.44583	-95.43750	1894399659
9590	Diptera	Culicidae	Aedes	Aedes aegypti	SPECIES	17.44861	-95.16389	1894394740
9591	Diptera	Culicidae	Aedes	Aedes aegypti	SPECIES	17.44861	-95.43194	1894398144
9592	Diptera	Culicidae	Aedes	Aedes aegypti	SPECIES	17.46000	106.63000	1264886171
9593	Diptera	Culicidae	Aedes	Aedes aegypti	SPECIES	17.47000	73.20000	1264890590
9594	Diptera	Culicidae	Aedes	Aedes aegypti	SPECIES	17.47083	-91.42639	1894400201

№	Order	Family	Genus	Species	Taxon Rank	Latitude	Longitude	GBIF_ID
9595	Diptera	Culicidae	Aedes	Aedes aegypti	SPECIES	17.47500	-95.25556	1894397268
9596	Diptera	Culicidae	Aedes	Aedes aegypti	SPECIES	17.47666	100.12826	4145860393
9597	Diptera	Culicidae	Aedes	Aedes aegypti	SPECIES	17.48111	-95.20833	1894398825
9598	Diptera	Culicidae	Aedes	Aedes aegypti	SPECIES	17.48737	-62.99371	3018904346
9599	Diptera	Culicidae	Aedes	Aedes aegypti	SPECIES	17.49000	-88.19000	1264891220
9600	Diptera	Culicidae	Aedes	Aedes aegypti	SPECIES	17.50481	-62.98299	3018904331
9601	Diptera	Culicidae	Aedes	Aedes aegypti	SPECIES	17.51000	-93.11000	1264887256
9602	Diptera	Culicidae	Aedes	Aedes aegypti	SPECIES	17.51528	-95.14306	1894399929
9603	Diptera	Culicidae	Aedes	Aedes aegypti	SPECIES	17.53000	73.52000	1264886736
9604	Diptera	Culicidae	Aedes	Aedes aegypti	SPECIES	17.53000	73.51000	1264890553
9605	Diptera	Culicidae	Aedes	Aedes aegypti	SPECIES	17.53806	-95.26083	1894398188
9606	Diptera	Culicidae	Aedes	Aedes aegypti	SPECIES	17.54000	-98.58000	1264886331
9607	Diptera	Culicidae	Aedes	Aedes aegypti	SPECIES	17.54115	100.11276	4145855652
9608	Diptera	Culicidae	Aedes	Aedes aegypti	SPECIES	17.54337	100.62484	4145849894
9609	Diptera	Culicidae	Aedes	Aedes aegypti	SPECIES	17.55000	-99.50000	1264888640
9610	Diptera	Culicidae	Aedes	Aedes aegypti	SPECIES	17.55000	73.98000	1264890542
9611	Diptera	Culicidae	Aedes	Aedes aegypti	SPECIES	17.56000	-99.50000	1264886360
9612	Diptera	Culicidae	Aedes	Aedes aegypti	SPECIES	17.58000	99.70000	1264891333
9613	Diptera	Culicidae	Aedes	Aedes aegypti	SPECIES	17.60000	-101.46000	1264888060
9614	Diptera	Culicidae	Aedes	Aedes aegypti	SPECIES	17.61000	99.22000	1264886484
9615	Diptera	Culicidae	Aedes	Aedes aegypti	SPECIES	17.61785	-63.24702	3018904342
9616	Diptera	Culicidae	Aedes	Aedes aegypti	SPECIES	17.62000	121.72000	1264889443
9617	Diptera	Culicidae	Aedes	Aedes aegypti	SPECIES	17.62278	-98.34444	1894397204
9618	Diptera	Culicidae	Aedes	Aedes aegypti	SPECIES	17.62757	-63.24957	3018904338
9619	Diptera	Culicidae	Aedes	Aedes aegypti	SPECIES	17.62883	-63.23185	3018904376
9620	Diptera	Culicidae	Aedes	Aedes aegypti	SPECIES	17.63000	100.10000	1264887643
9621	Diptera	Culicidae	Aedes	Aedes aegypti	SPECIES	17.64000	-101.55000	1264886343
9622	Diptera	Culicidae	Aedes	Aedes aegypti	SPECIES	17.64000	99.24000	1264887646
9623	Diptera	Culicidae	Aedes	Aedes aegypti	SPECIES	17.64306	-95.36667	1894400131
9624	Diptera	Culicidae	Aedes	Aedes aegypti	SPECIES	17.65000	-101.53000	1264888743
9625	Diptera	Culicidae	Aedes	Aedes aegypti	SPECIES	17.65583	-98.41667	1894399337
9626	Diptera	Culicidae	Aedes	Aedes aegypti	SPECIES	17.65694	-101.59306	2629264047
9627	Diptera	Culicidae	Aedes	Aedes aegypti	SPECIES	17.67000	-101.60000	1264886772
9628	Diptera	Culicidae	Aedes	Aedes aegypti	SPECIES	17.67000	75.90000	1264889260
9629	Diptera	Culicidae	Aedes	Aedes aegypti	SPECIES	17.67000	-99.88000	1264891004
9630	Diptera	Culicidae	Aedes	Aedes aegypti	SPECIES	17.69000	74.00000	1264889239
9631	Diptera	Culicidae	Aedes	Aedes aegypti	SPECIES	17.70000	106.44000	1264886188
9632	Diptera	Culicidae	Aedes	Aedes aegypti	SPECIES	17.70000	83.29000	1264889927
9633	Diptera	Culicidae	Aedes	Aedes aegypti	SPECIES	17.72000	73.40000	1264886752
9634	Diptera	Culicidae	Aedes	Aedes aegypti	SPECIES	17.72000	83.22000	1264889939
9635	Diptera	Culicidae	Aedes	Aedes aegypti	SPECIES	17.72000	73.39000	1264890567

№	Order	Family	Genus	Species	Taxon Rank	Latitude	Longitude	GBIF_ID
9636	Diptera	Culicidae	Aedes	Aedes aegypti	SPECIES	17.73020	-64.74412	177059889
9637	Diptera	Culicidae	Aedes	Aedes aegypti	SPECIES	17.73225	-98.55088	3912289732
9638	Diptera	Culicidae	Aedes	Aedes aegypti	SPECIES	17.73957	-64.70649	177059940
9639	Diptera	Culicidae	Aedes	Aedes aegypti	SPECIES	17.74587	83.32911	3889071564
9640	Diptera	Culicidae	Aedes	Aedes aegypti	SPECIES	17.76000	73.19000	1264886721
9641	Diptera	Culicidae	Aedes	Aedes aegypti	SPECIES	17.77000	73.18000	1264890541
9642	Diptera	Culicidae	Aedes	Aedes aegypti	SPECIES	17.77167	102.76667	4145851261
9643	Diptera	Culicidae	Aedes	Aedes aegypti	SPECIES	17.77750	102.76778	4145852713
9644	Diptera	Culicidae	Aedes	Aedes aegypti	SPECIES	17.80000	-77.24000	1264889115
9645	Diptera	Culicidae	Aedes	Aedes aegypti	SPECIES	17.80472	-97.77611	1894398116
9646	Diptera	Culicidae	Aedes	Aedes aegypti	SPECIES	17.85000	102.58000	1264886057
9647	Diptera	Culicidae	Aedes	Aedes aegypti	SPECIES	17.85078	103.75409	4145856798
9648	Diptera	Culicidae	Aedes	Aedes aegypti	SPECIES	17.85833	-98.14083	1894395739
9649	Diptera	Culicidae	Aedes	Aedes aegypti	SPECIES	17.86419	102.57514	4145857730
9650	Diptera	Culicidae	Aedes	Aedes aegypti	SPECIES	17.88000	102.74000	1264886468
9651	Diptera	Culicidae	Aedes	Aedes aegypti	SPECIES	17.88181	102.73617	4145852912
9652	Diptera	Culicidae	Aedes	Aedes aegypti	SPECIES	17.88353	-94.95661	1421474461
9653	Diptera	Culicidae	Aedes	Aedes aegypti	SPECIES	17.88972	-93.16389	1894396620
9654	Diptera	Culicidae	Aedes	Aedes aegypti	SPECIES	17.90227	-62.83484	3712685833
9655	Diptera	Culicidae	Aedes	Aedes aegypti	SPECIES	17.90436	-62.83190	4050454687
9656	Diptera	Culicidae	Aedes	Aedes aegypti	SPECIES	17.92000	106.47000	1264886125
9657	Diptera	Culicidae	Aedes	Aedes aegypti	SPECIES	17.93917	-94.91383	1421477099
9658	Diptera	Culicidae	Aedes	Aedes aegypti	SPECIES	17.94833	-94.91333	2629264296
9659	Diptera	Culicidae	Aedes	Aedes aegypti	SPECIES	17.94940	-67.19403	3457177273
9660	Diptera	Culicidae	Aedes	Aedes aegypti	SPECIES	17.95000	-94.91000	1264887058
9661	Diptera	Culicidae	Aedes	Aedes aegypti	SPECIES	17.95000	-66.26000	1264887059
9662	Diptera	Culicidae	Aedes	Aedes aegypti	SPECIES	17.95000	-102.20000	1264888047
9663	Diptera	Culicidae	Aedes	Aedes aegypti	SPECIES	17.95000	-102.21000	1264888745
9664	Diptera	Culicidae	Aedes	Aedes aegypti	SPECIES	17.95000	-76.72000	1264889762
9665	Diptera	Culicidae	Aedes	Aedes aegypti	SPECIES	17.95256	-102.19325	2629265040
9666	Diptera	Culicidae	Aedes	Aedes aegypti	SPECIES	17.95361	-66.83585	4129343171
9667	Diptera	Culicidae	Aedes	Aedes aegypti	SPECIES	17.95556	-97.02361	1894399387
9668	Diptera	Culicidae	Aedes	Aedes aegypti	SPECIES	17.95583	-92.98750	2629264540
9669	Diptera	Culicidae	Aedes	Aedes aegypti	SPECIES	17.95611	-102.19222	2629264919
9670	Diptera	Culicidae	Aedes	Aedes aegypti	SPECIES	17.95794	-66.83555	4129342542
9671	Diptera	Culicidae	Aedes	Aedes aegypti	SPECIES	17.96000	-102.19000	1264886788
9672	Diptera	Culicidae	Aedes	Aedes aegypti	SPECIES	17.96000	-66.29000	1264887082
9673	Diptera	Culicidae	Aedes	Aedes aegypti	SPECIES	17.96000	-92.95694	2629265446
9674	Diptera	Culicidae	Aedes	Aedes aegypti	SPECIES	17.96720	-66.93183	1993809094
9675	Diptera	Culicidae	Aedes	Aedes aegypti	SPECIES	17.96783	-66.28463	3079585454
9676	Diptera	Culicidae	Aedes	Aedes aegypti	SPECIES	17.96851	-66.88271	3926403218

№	Order	Family	Genus	Species	Taxon Rank	Latitude	Longitude	GBIF_ID
9677	Diptera	Culicidae	Aedes	Aedes aegypti	SPECIES	17.96972	-92.96306	2629264914
9678	Diptera	Culicidae	Aedes	Aedes aegypti	SPECIES	17.97000	-66.06000	1264888628
9679	Diptera	Culicidae	Aedes	Aedes aegypti	SPECIES	17.97000	-94.37000	1264888718
9680	Diptera	Culicidae	Aedes	Aedes aegypti	SPECIES	17.97000	102.61000	1264888989
9681	Diptera	Culicidae	Aedes	Aedes aegypti	SPECIES	17.97000	-66.91000	1264890887
9682	Diptera	Culicidae	Aedes	Aedes aegypti	SPECIES	17.97061	-66.82944	3926403213
9683	Diptera	Culicidae	Aedes	Aedes aegypti	SPECIES	17.97244	-66.90844	1993809107
9684	Diptera	Culicidae	Aedes	Aedes aegypti	SPECIES	17.97356	-66.87471	4076582158
9685	Diptera	Culicidae	Aedes	Aedes aegypti	SPECIES	17.97362	-66.85491	4067945613
9686	Diptera	Culicidae	Aedes	Aedes aegypti	SPECIES	17.97564	-66.86790	4129342770
9687	Diptera	Culicidae	Aedes	Aedes aegypti	SPECIES	17.97600	-66.86800	3441537243
9688	Diptera	Culicidae	Aedes	Aedes aegypti	SPECIES	17.97735	-66.87771	4129343170
9689	Diptera	Culicidae	Aedes	Aedes aegypti	SPECIES	17.97917	-92.91389	2629265598
9690	Diptera	Culicidae	Aedes	Aedes aegypti	SPECIES	17.98000	-65.99000	1264886967
9691	Diptera	Culicidae	Aedes	Aedes aegypti	SPECIES	17.98000	-65.94000	1264886984
9692	Diptera	Culicidae	Aedes	Aedes aegypti	SPECIES	17.98000	-94.54000	1264887049
9693	Diptera	Culicidae	Aedes	Aedes aegypti	SPECIES	17.98000	-92.93000	1264888019
9694	Diptera	Culicidae	Aedes	Aedes aegypti	SPECIES	17.98000	-76.78000	1264888149
9695	Diptera	Culicidae	Aedes	Aedes aegypti	SPECIES	17.98000	-66.11000	1264888614
9696	Diptera	Culicidae	Aedes	Aedes aegypti	SPECIES	17.98000	-94.55000	1264888749
9697	Diptera	Culicidae	Aedes	Aedes aegypti	SPECIES	17.98000	-66.12000	1264889442
9698	Diptera	Culicidae	Aedes	Aedes aegypti	SPECIES	17.98000	-66.30000	1264890321
9699	Diptera	Culicidae	Aedes	Aedes aegypti	SPECIES	17.98000	-66.23000	1264890638
9700	Diptera	Culicidae	Aedes	Aedes aegypti	SPECIES	17.98227	-66.87202	3879283088
9701	Diptera	Culicidae	Aedes	Aedes aegypti	SPECIES	17.98646	-76.88095	177059739
9702	Diptera	Culicidae	Aedes	Aedes aegypti	SPECIES	17.98667	-94.34528	2629263698
9703	Diptera	Culicidae	Aedes	Aedes aegypti	SPECIES	17.98808	97.91254	4145852845
9704	Diptera	Culicidae	Aedes	Aedes aegypti	SPECIES	17.98833	-92.92889	2629265368
9705	Diptera	Culicidae	Aedes	Aedes aegypti	SPECIES	17.98889	-94.55861	2629265249
9706	Diptera	Culicidae	Aedes	Aedes aegypti	SPECIES	17.98917	-92.92806	2629262962
9707	Diptera	Culicidae	Aedes	Aedes aegypti	SPECIES	17.98936	-94.34492	1421476122
9708	Diptera	Culicidae	Aedes	Aedes aegypti	SPECIES	17.99000	-92.95000	1264886748
9709	Diptera	Culicidae	Aedes	Aedes aegypti	SPECIES	17.99000	-66.00000	1264886970
9710	Diptera	Culicidae	Aedes	Aedes aegypti	SPECIES	17.99000	-94.35000	1264887034
9711	Diptera	Culicidae	Aedes	Aedes aegypti	SPECIES	17.99000	-92.93000	1264888711
9712	Diptera	Culicidae	Aedes	Aedes aegypti	SPECIES	17.99000	-76.80000	1264888864
9713	Diptera	Culicidae	Aedes	Aedes aegypti	SPECIES	17.99056	-92.92806	1894399918
9714	Diptera	Culicidae	Aedes	Aedes aegypti	SPECIES	17.99189	-94.58764	1421474362
9715	Diptera	Culicidae	Aedes	Aedes aegypti	SPECIES	17.99481	-102.34539	2629262772
9716	Diptera	Culicidae	Aedes	Aedes aegypti	SPECIES	17.99528	-94.63694	2629265192
9717	Diptera	Culicidae	Aedes	Aedes aegypti	SPECIES	17.99708	-94.63431	1421475600

№	Order	Family	Genus	Species	Taxon Rank	Latitude	Longitude	GBIF_ID
9718	Diptera	Culicidae	Aedes	Aedes aegypti	SPECIES	18.00000	-94.64000	1264886821
9719	Diptera	Culicidae	Aedes	Aedes aegypti	SPECIES	18.00000	-92.90000	1264887029
9720	Diptera	Culicidae	Aedes	Aedes aegypti	SPECIES	18.00000	-94.63000	1264887032
9721	Diptera	Culicidae	Aedes	Aedes aegypti	SPECIES	18.00000	-66.59000	1264887855
9722	Diptera	Culicidae	Aedes	Aedes aegypti	SPECIES	18.00000	-76.81000	1264888190
9723	Diptera	Culicidae	Aedes	Aedes aegypti	SPECIES	18.00000	-93.37556	1894400151
9724	Diptera	Culicidae	Aedes	Aedes aegypti	SPECIES	18.01000	-94.63000	1264887416
9725	Diptera	Culicidae	Aedes	Aedes aegypti	SPECIES	18.01000	-66.01000	1264887452
9726	Diptera	Culicidae	Aedes	Aedes aegypti	SPECIES	18.01000	-66.02000	1264888605
9727	Diptera	Culicidae	Aedes	Aedes aegypti	SPECIES	18.01528	-92.95833	1894400135
9728	Diptera	Culicidae	Aedes	Aedes aegypti	SPECIES	18.01637	-67.07324	3926405407
9729	Diptera	Culicidae	Aedes	Aedes aegypti	SPECIES	18.01750	103.31250	4145849440
9730	Diptera	Culicidae	Aedes	Aedes aegypti	SPECIES	18.01805	-66.78982	1993809132
9731	Diptera	Culicidae	Aedes	Aedes aegypti	SPECIES	18.02000	-66.00000	1264886982
9732	Diptera	Culicidae	Aedes	Aedes aegypti	SPECIES	18.02000	-66.25000	1264887096
9733	Diptera	Culicidae	Aedes	Aedes aegypti	SPECIES	18.02000	-66.62000	1264888627
9734	Diptera	Culicidae	Aedes	Aedes aegypti	SPECIES	18.02000	-66.61000	1264890874
9735	Diptera	Culicidae	Aedes	Aedes aegypti	SPECIES	18.02028	-92.95611	1894399185
9736	Diptera	Culicidae	Aedes	Aedes aegypti	SPECIES	18.02333	-92.97222	1894396791
9737	Diptera	Culicidae	Aedes	Aedes aegypti	SPECIES	18.02444	-92.96444	1894398838
9738	Diptera	Culicidae	Aedes	Aedes aegypti	SPECIES	18.02444	103.26167	4145860336
9739	Diptera	Culicidae	Aedes	Aedes aegypti	SPECIES	18.02567	-67.08249	4129342400
9740	Diptera	Culicidae	Aedes	Aedes aegypti	SPECIES	18.02671	-66.32922	3005080533
9741	Diptera	Culicidae	Aedes	Aedes aegypti	SPECIES	18.02748	-67.07454	3926402812
9742	Diptera	Culicidae	Aedes	Aedes aegypti	SPECIES	18.03000	-66.03000	1264887450
9743	Diptera	Culicidae	Aedes	Aedes aegypti	SPECIES	18.03000	-76.82000	1264888180
9744	Diptera	Culicidae	Aedes	Aedes aegypti	SPECIES	18.03181	-67.07449	4129342538
9745	Diptera	Culicidae	Aedes	Aedes aegypti	SPECIES	18.03505	-63.20893	4050554287
9746	Diptera	Culicidae	Aedes	Aedes aegypti	SPECIES	18.04000	-67.15000	1264890911
9747	Diptera	Culicidae	Aedes	Aedes aegypti	SPECIES	18.04119	-65.87770	3327925512
9748	Diptera	Culicidae	Aedes	Aedes aegypti	SPECIES	18.04139	-92.94333	1894399885
9749	Diptera	Culicidae	Aedes	Aedes aegypti	SPECIES	18.04194	-92.91083	1894396411
9750	Diptera	Culicidae	Aedes	Aedes aegypti	SPECIES	18.05000	-66.03000	1264886989
9751	Diptera	Culicidae	Aedes	Aedes aegypti	SPECIES	18.05000	-66.08000	1264887001
9752	Diptera	Culicidae	Aedes	Aedes aegypti	SPECIES	18.05000	-77.37000	1264889793
9753	Diptera	Culicidae	Aedes	Aedes aegypti	SPECIES	18.05000	-65.91000	1264890630
9754	Diptera	Culicidae	Aedes	Aedes aegypti	SPECIES	18.05000	-66.49000	1264890906
9755	Diptera	Culicidae	Aedes	Aedes aegypti	SPECIES	18.05000	-76.76000	1264891253
9756	Diptera	Culicidae	Aedes	Aedes aegypti	SPECIES	18.05492	100.13848	4145852004
9757	Diptera	Culicidae	Aedes	Aedes aegypti	SPECIES	18.06333	-92.96222	1894398456
9758	Diptera	Culicidae	Aedes	Aedes aegypti	SPECIES	18.06667	-93.17500	1894398837

№	Order	Family	Genus	Species	Taxon Rank	Latitude	Longitude	GBIF_ID
9759	Diptera	Culicidae	Aedes	Aedes aegypti	SPECIES	18.07000	73.42000	1264890532
9760	Diptera	Culicidae	Aedes	Aedes aegypti	SPECIES	18.07000	-65.89000	1264890897
9761	Diptera	Culicidae	Aedes	Aedes aegypti	SPECIES	18.07083	-96.53472	1894398674
9762	Diptera	Culicidae	Aedes	Aedes aegypti	SPECIES	18.08000	100.20000	1264886522
9763	Diptera	Culicidae	Aedes	Aedes aegypti	SPECIES	18.08000	73.42000	1264886737
9764	Diptera	Culicidae	Aedes	Aedes aegypti	SPECIES	18.08000	-66.36000	1264888639
9765	Diptera	Culicidae	Aedes	Aedes aegypti	SPECIES	18.08000	-67.01000	1264890924
9766	Diptera	Culicidae	Aedes	Aedes aegypti	SPECIES	18.08285	-76.88529	3391776328
9767	Diptera	Culicidae	Aedes	Aedes aegypti	SPECIES	18.08290	-76.88526	3391344276
9768	Diptera	Culicidae	Aedes	Aedes aegypti	SPECIES	18.08611	-96.12389	1894400094
9769	Diptera	Culicidae	Aedes	Aedes aegypti	SPECIES	18.08639	-92.94889	1894394204
9770	Diptera	Culicidae	Aedes	Aedes aegypti	SPECIES	18.09000	-96.12000	1264890583
9771	Diptera	Culicidae	Aedes	Aedes aegypti	SPECIES	18.09169	-63.10736	3005142465
9772	Diptera	Culicidae	Aedes	Aedes aegypti	SPECIES	18.09250	-96.10694	1894399681
9773	Diptera	Culicidae	Aedes	Aedes aegypti	SPECIES	18.09811	-94.38744	1421477282
9774	Diptera	Culicidae	Aedes	Aedes aegypti	SPECIES	18.09833	-102.78361	1894398081
9775	Diptera	Culicidae	Aedes	Aedes aegypti	SPECIES	18.10075	-88.54997	226099173
9776	Diptera	Culicidae	Aedes	Aedes aegypti	SPECIES	18.10139	-92.94194	1894399787
9777	Diptera	Culicidae	Aedes	Aedes aegypti	SPECIES	18.10694	-95.88056	1894399183
9778	Diptera	Culicidae	Aedes	Aedes aegypti	SPECIES	18.11000	-67.04000	1264890905
9779	Diptera	Culicidae	Aedes	Aedes aegypti	SPECIES	18.11306	-92.96556	1894395367
9780	Diptera	Culicidae	Aedes	Aedes aegypti	SPECIES	18.11444	-95.76528	1421477985
9781	Diptera	Culicidae	Aedes	Aedes aegypti	SPECIES	18.11489	100.12008	4145859753
9782	Diptera	Culicidae	Aedes	Aedes aegypti	SPECIES	18.11806	-96.00139	1894398746
9783	Diptera	Culicidae	Aedes	Aedes aegypti	SPECIES	18.12000	-66.53000	1264890915
9784	Diptera	Culicidae	Aedes	Aedes aegypti	SPECIES	18.12028	-96.05278	1894399033
9785	Diptera	Culicidae	Aedes	Aedes aegypti	SPECIES	18.12083	-95.97500	1894400108
9786	Diptera	Culicidae	Aedes	Aedes aegypti	SPECIES	18.12472	-92.94861	1894397257
9787	Diptera	Culicidae	Aedes	Aedes aegypti	SPECIES	18.13000	-66.49000	1264889857
9788	Diptera	Culicidae	Aedes	Aedes aegypti	SPECIES	18.13000	-66.47000	1264890951
9789	Diptera	Culicidae	Aedes	Aedes aegypti	SPECIES	18.13222	97.91549	4145853836
9790	Diptera	Culicidae	Aedes	Aedes aegypti	SPECIES	18.13500	-93.04639	1894397516
9791	Diptera	Culicidae	Aedes	Aedes aegypti	SPECIES	18.13778	-92.96361	1894397564
9792	Diptera	Culicidae	Aedes	Aedes aegypti	SPECIES	18.13778	-94.43528	2629265556
9793	Diptera	Culicidae	Aedes	Aedes aegypti	SPECIES	18.13794	100.15047	4145850316
9794	Diptera	Culicidae	Aedes	Aedes aegypti	SPECIES	18.14000	-94.44000	1264886688
9795	Diptera	Culicidae	Aedes	Aedes aegypti	SPECIES	18.14000	-94.43000	1264886816
9796	Diptera	Culicidae	Aedes	Aedes aegypti	SPECIES	18.14000	-94.42000	1264887434
9797	Diptera	Culicidae	Aedes	Aedes aegypti	SPECIES	18.14000	73.40000	1264890549
9798	Diptera	Culicidae	Aedes	Aedes aegypti	SPECIES	18.14528	-92.76556	1894400025
9799	Diptera	Culicidae	Aedes	Aedes aegypti	SPECIES	18.14583	-91.02778	1894395796

№	Order	Family	Genus	Species	Taxon Rank	Latitude	Longitude	GBIF_ID
9800	Diptera	Culicidae	Aedes	Aedes aegypti	SPECIES	18.14934	-94.43960	1894398786
9801	Diptera	Culicidae	Aedes	Aedes aegypti	SPECIES	18.15000	100.14000	1264887498
9802	Diptera	Culicidae	Aedes	Aedes aegypti	SPECIES	18.15000	-94.41000	1264888554
9803	Diptera	Culicidae	Aedes	Aedes aegypti	SPECIES	18.15000	-65.83000	1264889465
9804	Diptera	Culicidae	Aedes	Aedes aegypti	SPECIES	18.15000	-96.71806	1894397457
9805	Diptera	Culicidae	Aedes	Aedes aegypti	SPECIES	18.15917	-93.08194	1894395438
9806	Diptera	Culicidae	Aedes	Aedes aegypti	SPECIES	18.16000	97.97000	1264887655
9807	Diptera	Culicidae	Aedes	Aedes aegypti	SPECIES	18.16000	-65.83000	1264888618
9808	Diptera	Culicidae	Aedes	Aedes aegypti	SPECIES	18.16000	73.85000	1264891247
9809	Diptera	Culicidae	Aedes	Aedes aegypti	SPECIES	18.16556	-92.97694	1894397135
9810	Diptera	Culicidae	Aedes	Aedes aegypti	SPECIES	18.16583	-93.01806	1894400219
9811	Diptera	Culicidae	Aedes	Aedes aegypti	SPECIES	18.17000	-65.96000	1264888469
9812	Diptera	Culicidae	Aedes	Aedes aegypti	SPECIES	18.17000	73.77000	1264888677
9813	Diptera	Culicidae	Aedes	Aedes aegypti	SPECIES	18.17000	76.03000	1264889237
9814	Diptera	Culicidae	Aedes	Aedes aegypti	SPECIES	18.17000	-63.15000	1264890252
9815	Diptera	Culicidae	Aedes	Aedes aegypti	SPECIES	18.17000	-63.16000	1264890263
9816	Diptera	Culicidae	Aedes	Aedes aegypti	SPECIES	18.17167	-68.78041	3031882568
9817	Diptera	Culicidae	Aedes	Aedes aegypti	SPECIES	18.17556	-93.06222	1894400224
9818	Diptera	Culicidae	Aedes	Aedes aegypti	SPECIES	18.18000	-66.05000	1264887917
9819	Diptera	Culicidae	Aedes	Aedes aegypti	SPECIES	18.18000	-66.31000	1264888329
9820	Diptera	Culicidae	Aedes	Aedes aegypti	SPECIES	18.18000	-65.87000	1264888647
9821	Diptera	Culicidae	Aedes	Aedes aegypti	SPECIES	18.18000	-63.16000	1264889585
9822	Diptera	Culicidae	Aedes	Aedes aegypti	SPECIES	18.18000	-63.14000	1264889594
9823	Diptera	Culicidae	Aedes	Aedes aegypti	SPECIES	18.18000	-63.13000	1264889621
9824	Diptera	Culicidae	Aedes	Aedes aegypti	SPECIES	18.18444	-91.36278	1894399081
9825	Diptera	Culicidae	Aedes	Aedes aegypti	SPECIES	18.18892	-88.69319	1421478039
9826	Diptera	Culicidae	Aedes	Aedes aegypti	SPECIES	18.20000	-67.13000	1264887889
9827	Diptera	Culicidae	Aedes	Aedes aegypti	SPECIES	18.20000	-67.14000	1264887901
9828	Diptera	Culicidae	Aedes	Aedes aegypti	SPECIES	18.20112	-67.13844	1993809163
9829	Diptera	Culicidae	Aedes	Aedes aegypti	SPECIES	18.21000	-67.14000	1264890260
9830	Diptera	Culicidae	Aedes	Aedes aegypti	SPECIES	18.21000	-63.05000	1264890272
9831	Diptera	Culicidae	Aedes	Aedes aegypti	SPECIES	18.21389	-96.26250	1894396274
9832	Diptera	Culicidae	Aedes	Aedes aegypti	SPECIES	18.21472	-93.03167	1894399315
9833	Diptera	Culicidae	Aedes	Aedes aegypti	SPECIES	18.22000	-66.06000	1264887879
9834	Diptera	Culicidae	Aedes	Aedes aegypti	SPECIES	18.22000	74.87000	1264888954
9835	Diptera	Culicidae	Aedes	Aedes aegypti	SPECIES	18.22000	-63.05000	1264890235
9836	Diptera	Culicidae	Aedes	Aedes aegypti	SPECIES	18.22000	-66.59000	1264890912
9837	Diptera	Culicidae	Aedes	Aedes aegypti	SPECIES	18.22306	-93.11056	1894399323
9838	Diptera	Culicidae	Aedes	Aedes aegypti	SPECIES	18.22917	-96.27917	1894399836
9839	Diptera	Culicidae	Aedes	Aedes aegypti	SPECIES	18.22937	-66.87031	3872876067
9840	Diptera	Culicidae	Aedes	Aedes aegypti	SPECIES	18.22940	-66.87048	3337799236

№	Order	Family	Genus	Species	Taxon Rank	Latitude	Longitude	GBIF_ID
9841	Diptera	Culicidae	Aedes	Aedes aegypti	SPECIES	18.23000	99.43000	1264886546
9842	Diptera	Culicidae	Aedes	Aedes aegypti	SPECIES	18.23000	-65.92000	1264888620
9843	Diptera	Culicidae	Aedes	Aedes aegypti	SPECIES	18.23000	-63.00000	1264889573
9844	Diptera	Culicidae	Aedes	Aedes aegypti	SPECIES	18.23000	-63.04000	1264889774
9845	Diptera	Culicidae	Aedes	Aedes aegypti	SPECIES	18.23000	-63.07000	1264890274
9846	Diptera	Culicidae	Aedes	Aedes aegypti	SPECIES	18.23000	-65.75000	1264890879
9847	Diptera	Culicidae	Aedes	Aedes aegypti	SPECIES	18.23472	-93.00583	1894396191
9848	Diptera	Culicidae	Aedes	Aedes aegypti	SPECIES	18.23778	-66.04758	3070392621
9849	Diptera	Culicidae	Aedes	Aedes aegypti	SPECIES	18.23880	-66.03525	3079696344
9850	Diptera	Culicidae	Aedes	Aedes aegypti	SPECIES	18.24000	-67.14000	1264887874
9851	Diptera	Culicidae	Aedes	Aedes aegypti	SPECIES	18.24000	-66.04000	1264888612
9852	Diptera	Culicidae	Aedes	Aedes aegypti	SPECIES	18.24000	-63.00000	1264889608
9853	Diptera	Culicidae	Aedes	Aedes aegypti	SPECIES	18.24000	75.69000	1264889932
9854	Diptera	Culicidae	Aedes	Aedes aegypti	SPECIES	18.24000	-65.76000	1264890901
9855	Diptera	Culicidae	Aedes	Aedes aegypti	SPECIES	18.25000	-63.02000	1264889595
9856	Diptera	Culicidae	Aedes	Aedes aegypti	SPECIES	18.25000	-63.00000	1264889620
9857	Diptera	Culicidae	Aedes	Aedes aegypti	SPECIES	18.25000	42.51000	3351439885
9858	Diptera	Culicidae	Aedes	Aedes aegypti	SPECIES	18.25170	-66.05029	3391715567
9859	Diptera	Culicidae	Aedes	Aedes aegypti	SPECIES	18.25556	-92.09167	1894394394
9860	Diptera	Culicidae	Aedes	Aedes aegypti	SPECIES	18.26000	-65.67000	1264890876
9861	Diptera	Culicidae	Aedes	Aedes aegypti	SPECIES	18.26043	-66.04948	2603481007
9862	Diptera	Culicidae	Aedes	Aedes aegypti	SPECIES	18.27000	-66.70000	1264888601
9863	Diptera	Culicidae	Aedes	Aedes aegypti	SPECIES	18.27000	-65.65000	1264888634
9864	Diptera	Culicidae	Aedes	Aedes aegypti	SPECIES	18.27600	99.65850	4145854857
9865	Diptera	Culicidae	Aedes	Aedes aegypti	SPECIES	18.28306	-96.31750	1894399165
9866	Diptera	Culicidae	Aedes	Aedes aegypti	SPECIES	18.29000	99.51000	1264887675
9867	Diptera	Culicidae	Aedes	Aedes aegypti	SPECIES	18.29461	99.49653	4145853225
9868	Diptera	Culicidae	Aedes	Aedes aegypti	SPECIES	18.29722	-96.68167	1894398532
9869	Diptera	Culicidae	Aedes	Aedes aegypti	SPECIES	18.30000	-66.88000	1264888676
9870	Diptera	Culicidae	Aedes	Aedes aegypti	SPECIES	18.30000	-66.25000	1264889780
9871	Diptera	Culicidae	Aedes	Aedes aegypti	SPECIES	18.30000	-96.33556	1894397693
9872	Diptera	Culicidae	Aedes	Aedes aegypti	SPECIES	18.30208	-65.28690	3356892312
9873	Diptera	Culicidae	Aedes	Aedes aegypti	SPECIES	18.31500	-100.28389	1894397944
9874	Diptera	Culicidae	Aedes	Aedes aegypti	SPECIES	18.32000	-65.67000	1264890904
9875	Diptera	Culicidae	Aedes	Aedes aegypti	SPECIES	18.32105	-65.81972	1993809117
9876	Diptera	Culicidae	Aedes	Aedes aegypti	SPECIES	18.33000	-65.66000	1264888632
9877	Diptera	Culicidae	Aedes	Aedes aegypti	SPECIES	18.33000	-66.41000	1264889750
9878	Diptera	Culicidae	Aedes	Aedes aegypti	SPECIES	18.33000	72.96000	1264890576
9879	Diptera	Culicidae	Aedes	Aedes aegypti	SPECIES	18.33092	-66.40660	3496606930
9880	Diptera	Culicidae	Aedes	Aedes aegypti	SPECIES	18.34000	-99.53000	1264886344
9881	Diptera	Culicidae	Aedes	Aedes aegypti	SPECIES	18.34000	-99.54000	1264887405

№	Order	Family	Genus	Species	Taxon Rank	Latitude	Longitude	GBIF_ID
9882	Diptera	Culicidae	Aedes	Aedes aegypti	SPECIES	18.34361	-99.53917	1894400223
9883	Diptera	Culicidae	Aedes	Aedes aegypti	SPECIES	18.34459	-64.93183	177059860
9884	Diptera	Culicidae	Aedes	Aedes aegypti	SPECIES	18.34806	-96.34111	1894399756
9885	Diptera	Culicidae	Aedes	Aedes aegypti	SPECIES	18.35000	-66.32000	1264889790
9886	Diptera	Culicidae	Aedes	Aedes aegypti	SPECIES	18.35000	-66.12000	1264890894
9887	Diptera	Culicidae	Aedes	Aedes aegypti	SPECIES	18.35000	105.81000	1264891227
9888	Diptera	Culicidae	Aedes	Aedes aegypti	SPECIES	18.35278	-96.35000	1894398301
9889	Diptera	Culicidae	Aedes	Aedes aegypti	SPECIES	18.35592	-102.29972	2629264727
9890	Diptera	Culicidae	Aedes	Aedes aegypti	SPECIES	18.36000	-99.54000	1264888668
9891	Diptera	Culicidae	Aedes	Aedes aegypti	SPECIES	18.36000	-66.57000	1264889893
9892	Diptera	Culicidae	Aedes	Aedes aegypti	SPECIES	18.36000	-66.11000	1264890923
9893	Diptera	Culicidae	Aedes	Aedes aegypti	SPECIES	18.36013	-66.06963	3496611726
9894	Diptera	Culicidae	Aedes	Aedes aegypti	SPECIES	18.36500	-95.79528	1894398667
9895	Diptera	Culicidae	Aedes	Aedes aegypti	SPECIES	18.37000	-95.81000	1264888570
9896	Diptera	Culicidae	Aedes	Aedes aegypti	SPECIES	18.37000	-66.57000	1264888823
9897	Diptera	Culicidae	Aedes	Aedes aegypti	SPECIES	18.37000	-66.19000	1264889729
9898	Diptera	Culicidae	Aedes	Aedes aegypti	SPECIES	18.37264	-66.05670	2609212030
9899	Diptera	Culicidae	Aedes	Aedes aegypti	SPECIES	18.37288	-65.86329	2603330403
9900	Diptera	Culicidae	Aedes	Aedes aegypti	SPECIES	18.38000	-65.96000	1264888670
9901	Diptera	Culicidae	Aedes	Aedes aegypti	SPECIES	18.38000	-65.72000	1264888765
9902	Diptera	Culicidae	Aedes	Aedes aegypti	SPECIES	18.38000	-65.82000	1264888794
9903	Diptera	Culicidae	Aedes	Aedes aegypti	SPECIES	18.39000	-66.16000	1264890882
9904	Diptera	Culicidae	Aedes	Aedes aegypti	SPECIES	18.39556	-93.21222	1894400216
9905	Diptera	Culicidae	Aedes	Aedes aegypti	SPECIES	18.40000	-66.04000	1264888847
9906	Diptera	Culicidae	Aedes	Aedes aegypti	SPECIES	18.40000	-66.08000	1264889904
9907	Diptera	Culicidae	Aedes	Aedes aegypti	SPECIES	18.40000	-64.64000	1264890302
9908	Diptera	Culicidae	Aedes	Aedes aegypti	SPECIES	18.40306	103.55722	4145857096
9909	Diptera	Culicidae	Aedes	Aedes aegypti	SPECIES	18.41000	-64.64000	1264889737
9910	Diptera	Culicidae	Aedes	Aedes aegypti	SPECIES	18.41000	-66.08000	1264889942
9911	Diptera	Culicidae	Aedes	Aedes aegypti	SPECIES	18.41806	-70.11787	3456354725
9912	Diptera	Culicidae	Aedes	Aedes aegypti	SPECIES	18.41944	-95.11361	1894400280
9913	Diptera	Culicidae	Aedes	Aedes aegypti	SPECIES	18.42000	-66.74000	1264887898
9914	Diptera	Culicidae	Aedes	Aedes aegypti	SPECIES	18.42000	-66.05000	1264889581
9915	Diptera	Culicidae	Aedes	Aedes aegypti	SPECIES	18.42472	-93.15083	1894399901
9916	Diptera	Culicidae	Aedes	Aedes aegypti	SPECIES	18.43000	-65.88000	1264888631
9917	Diptera	Culicidae	Aedes	Aedes aegypti	SPECIES	18.43000	-67.15000	1264889864
9918	Diptera	Culicidae	Aedes	Aedes aegypti	SPECIES	18.44000	-66.05000	1264888651
9919	Diptera	Culicidae	Aedes	Aedes aegypti	SPECIES	18.44000	73.87000	1264888920
9920	Diptera	Culicidae	Aedes	Aedes aegypti	SPECIES	18.44000	-66.49000	1264890264
9921	Diptera	Culicidae	Aedes	Aedes aegypti	SPECIES	18.44085	-69.97596	3496614795
9922	Diptera	Culicidae	Aedes	Aedes aegypti	SPECIES	18.44556	-96.42639	1894397874

№	Order	Family	Genus	Species	Taxon Rank	Latitude	Longitude	GBIF_ID
9923	Diptera	Culicidae	Aedes	Aedes aegypti	SPECIES	18.44778	-96.36056	1421475606
9924	Diptera	Culicidae	Aedes	Aedes aegypti	SPECIES	18.44778	-96.36056	1894400166
9925	Diptera	Culicidae	Aedes	Aedes aegypti	SPECIES	18.45000	-66.26000	1264887881
9926	Diptera	Culicidae	Aedes	Aedes aegypti	SPECIES	18.45000	-95.22000	1264888546
9927	Diptera	Culicidae	Aedes	Aedes aegypti	SPECIES	18.45000	-66.06000	1264888673
9928	Diptera	Culicidae	Aedes	Aedes aegypti	SPECIES	18.45000	-66.07000	1264889715
9929	Diptera	Culicidae	Aedes	Aedes aegypti	SPECIES	18.45300	-66.06919	3705451846
9930	Diptera	Culicidae	Aedes	Aedes aegypti	SPECIES	18.45333	-69.93427	177060242
9931	Diptera	Culicidae	Aedes	Aedes aegypti	SPECIES	18.45922	-69.91730	3784983401
9932	Diptera	Culicidae	Aedes	Aedes aegypti	SPECIES	18.45928	-69.91732	3764151169
9933	Diptera	Culicidae	Aedes	Aedes aegypti	SPECIES	18.46000	105.77000	1264886215
9934	Diptera	Culicidae	Aedes	Aedes aegypti	SPECIES	18.46000	-66.05000	1264888629
9935	Diptera	Culicidae	Aedes	Aedes aegypti	SPECIES	18.46000	74.58000	1264889230
9936	Diptera	Culicidae	Aedes	Aedes aegypti	SPECIES	18.46000	-66.55000	1264890277
9937	Diptera	Culicidae	Aedes	Aedes aegypti	SPECIES	18.46204	-69.99777	177060161
9938	Diptera	Culicidae	Aedes	Aedes aegypti	SPECIES	18.46500	-88.51694	1894396343
9939	Diptera	Culicidae	Aedes	Aedes aegypti	SPECIES	18.46595	-66.11849	3456843474
9940	Diptera	Culicidae	Aedes	Aedes aegypti	SPECIES	18.46732	-66.11615	225864843
9941	Diptera	Culicidae	Aedes	Aedes aegypti	SPECIES	18.46777	-69.28307	3090891412
9942	Diptera	Culicidae	Aedes	Aedes aegypti	SPECIES	18.46974	-69.90406	177060332
9943	Diptera	Culicidae	Aedes	Aedes aegypti	SPECIES	18.47000	-66.11000	1264887466
9944	Diptera	Culicidae	Aedes	Aedes aegypti	SPECIES	18.47000	-66.73000	1264887882
9945	Diptera	Culicidae	Aedes	Aedes aegypti	SPECIES	18.47000	-66.75000	1264887909
9946	Diptera	Culicidae	Aedes	Aedes aegypti	SPECIES	18.47000	-66.94000	1264888675
9947	Diptera	Culicidae	Aedes	Aedes aegypti	SPECIES	18.47000	74.28000	1264889502
9948	Diptera	Culicidae	Aedes	Aedes aegypti	SPECIES	18.47000	-77.92000	1264890249
9949	Diptera	Culicidae	Aedes	Aedes aegypti	SPECIES	18.47116	-77.91883	1933070159
9950	Diptera	Culicidae	Aedes	Aedes aegypti	SPECIES	18.48000	-66.73000	1264890880
9951	Diptera	Culicidae	Aedes	Aedes aegypti	SPECIES	18.48411	73.79719	2620056345
9952	Diptera	Culicidae	Aedes	Aedes aegypti	SPECIES	18.48934	-70.12674	177060072
9953	Diptera	Culicidae	Aedes	Aedes aegypti	SPECIES	18.49000	-66.83000	1264888644
9954	Diptera	Culicidae	Aedes	Aedes aegypti	SPECIES	18.49000	-69.89000	1264890150
9955	Diptera	Culicidae	Aedes	Aedes aegypti	SPECIES	18.49361	-88.39306	1894396353
9956	Diptera	Culicidae	Aedes	Aedes aegypti	SPECIES	18.49678	-88.39728	2629265678
9957	Diptera	Culicidae	Aedes	Aedes aegypti	SPECIES	18.50000	73.87000	1264888690
9958	Diptera	Culicidae	Aedes	Aedes aegypti	SPECIES	18.50000	-88.30000	1264888747
9959	Diptera	Culicidae	Aedes	Aedes aegypti	SPECIES	18.50000	-69.88000	1264889680
9960	Diptera	Culicidae	Aedes	Aedes aegypti	SPECIES	18.50000	-88.29000	1264889797
9961	Diptera	Culicidae	Aedes	Aedes aegypti	SPECIES	18.50000	-99.56000	1264891376
9962	Diptera	Culicidae	Aedes	Aedes aegypti	SPECIES	18.50000	-88.31000	3818435885
9963	Diptera	Culicidae	Aedes	Aedes aegypti	SPECIES	18.50278	-88.51806	1894400145

№	Order	Family	Genus	Species	Taxon Rank	Latitude	Longitude	GBIF_ID
9964	Diptera	Culicidae	Aedes	Aedes aegypti	SPECIES	18.50361	-88.30528	1894400255
9965	Diptera	Culicidae	Aedes	Aedes aegypti	SPECIES	18.50694	-96.45833	1894399602
9966	Diptera	Culicidae	Aedes	Aedes aegypti	SPECIES	18.50889	-89.39167	2629265693
9967	Diptera	Culicidae	Aedes	Aedes aegypti	SPECIES	18.51000	73.86000	1264888706
9968	Diptera	Culicidae	Aedes	Aedes aegypti	SPECIES	18.51000	73.87000	1264888729
9969	Diptera	Culicidae	Aedes	Aedes aegypti	SPECIES	18.51000	-88.30000	1264888737
9970	Diptera	Culicidae	Aedes	Aedes aegypti	SPECIES	18.51000	-69.89000	1264890160
9971	Diptera	Culicidae	Aedes	Aedes aegypti	SPECIES	18.51000	-72.63000	1264890499
9972	Diptera	Culicidae	Aedes	Aedes aegypti	SPECIES	18.51333	-88.36694	2629264123
9973	Diptera	Culicidae	Aedes	Aedes aegypti	SPECIES	18.51389	-93.52500	1894394361
9974	Diptera	Culicidae	Aedes	Aedes aegypti	SPECIES	18.51528	-88.42778	1894398591
9975	Diptera	Culicidae	Aedes	Aedes aegypti	SPECIES	18.51750	-89.90250	1897511345
9976	Diptera	Culicidae	Aedes	Aedes aegypti	SPECIES	18.52000	-88.34000	1264886774
9977	Diptera	Culicidae	Aedes	Aedes aegypti	SPECIES	18.52000	-88.33000	1264887342
9978	Diptera	Culicidae	Aedes	Aedes aegypti	SPECIES	18.52000	73.88000	1264888669
9979	Diptera	Culicidae	Aedes	Aedes aegypti	SPECIES	18.52000	73.87000	1264888674
9980	Diptera	Culicidae	Aedes	Aedes aegypti	SPECIES	18.52000	73.86000	1264888698
9981	Diptera	Culicidae	Aedes	Aedes aegypti	SPECIES	18.52000	73.85000	1264889018
9982	Diptera	Culicidae	Aedes	Aedes aegypti	SPECIES	18.52000	-69.93000	1264889654
9983	Diptera	Culicidae	Aedes	Aedes aegypti	SPECIES	18.52079	73.83963	3496610675
9984	Diptera	Culicidae	Aedes	Aedes aegypti	SPECIES	18.52278	-98.79056	1894395311
9985	Diptera	Culicidae	Aedes	Aedes aegypti	SPECIES	18.52806	-72.33044	177060042
9986	Diptera	Culicidae	Aedes	Aedes aegypti	SPECIES	18.52828	-88.30367	2629264529
9987	Diptera	Culicidae	Aedes	Aedes aegypti	SPECIES	18.53000	-88.30000	1264887343
9988	Diptera	Culicidae	Aedes	Aedes aegypti	SPECIES	18.53000	73.90000	1264888667
9989	Diptera	Culicidae	Aedes	Aedes aegypti	SPECIES	18.53000	73.87000	1264888678
9990	Diptera	Culicidae	Aedes	Aedes aegypti	SPECIES	18.53000	73.91000	1264888719
9991	Diptera	Culicidae	Aedes	Aedes aegypti	SPECIES	18.53000	73.89000	1264888724
9992	Diptera	Culicidae	Aedes	Aedes aegypti	SPECIES	18.53000	73.85000	1264889960
9993	Diptera	Culicidae	Aedes	Aedes aegypti	SPECIES	18.53000	73.86000	1264891248
9994	Diptera	Culicidae	Aedes	Aedes aegypti	SPECIES	18.53472	-89.90083	1897511327
9995	Diptera	Culicidae	Aedes	Aedes aegypti	SPECIES	18.53722	-89.91750	2629264857
9996	Diptera	Culicidae	Aedes	Aedes aegypti	SPECIES	18.53889	-96.60556	1894399970
9997	Diptera	Culicidae	Aedes	Aedes aegypti	SPECIES	18.53944	-89.92139	2629265084
9998	Diptera	Culicidae	Aedes	Aedes aegypti	SPECIES	18.54000	73.22000	1264886731
9999	Diptera	Culicidae	Aedes	Aedes aegypti	SPECIES	18.54000	-88.30000	1264887332
10000	Diptera	Culicidae	Aedes	Aedes aegypti	SPECIES	18.54000	-72.34000	1264890223
10001	Diptera	Culicidae	Aedes	Aedes aegypti	SPECIES	18.54000	-88.26000	3818421863
10002	Diptera	Culicidae	Aedes	Aedes aegypti	SPECIES	18.55000	-88.26000	1264886789
10003	Diptera	Culicidae	Aedes	Aedes aegypti	SPECIES	18.55000	73.83000	1264888688
10004	Diptera	Culicidae	Aedes	Aedes aegypti	SPECIES	18.55000	-72.33000	1264891105

№	Order	Family	Genus	Species	Taxon Rank	Latitude	Longitude	GBIF_ID
10005	Diptera	Culicidae	Aedes	Aedes aegypti	SPECIES	18.55000	75.55000	1264891362
10006	Diptera	Culicidae	Aedes	Aedes aegypti	SPECIES	18.55139	-96.63139	1894399289
10007	Diptera	Culicidae	Aedes	Aedes aegypti	SPECIES	18.55236	99.85337	226099229
10008	Diptera	Culicidae	Aedes	Aedes aegypti	SPECIES	18.55444	-89.94278	1897511379
10009	Diptera	Culicidae	Aedes	Aedes aegypti	SPECIES	18.55444	-88.25833	2629264319
10010	Diptera	Culicidae	Aedes	Aedes aegypti	SPECIES	18.55500	-98.92583	1894396209
10011	Diptera	Culicidae	Aedes	Aedes aegypti	SPECIES	18.55683	-88.25631	2629265665
10012	Diptera	Culicidae	Aedes	Aedes aegypti	SPECIES	18.55722	-89.79833	1897511390
10013	Diptera	Culicidae	Aedes	Aedes aegypti	SPECIES	18.55861	-98.84667	1894399554
10014	Diptera	Culicidae	Aedes	Aedes aegypti	SPECIES	18.56000	-88.26000	1264887315
10015	Diptera	Culicidae	Aedes	Aedes aegypti	SPECIES	18.56000	73.76000	1264888696
10016	Diptera	Culicidae	Aedes	Aedes aegypti	SPECIES	18.56286	98.08520	4145852573
10017	Diptera	Culicidae	Aedes	Aedes aegypti	SPECIES	18.56500	-88.25778	2629264964
10018	Diptera	Culicidae	Aedes	Aedes aegypti	SPECIES	18.57000	99.01000	1264887472
10019	Diptera	Culicidae	Aedes	Aedes aegypti	SPECIES	18.57083	-89.89611	1897511371
10020	Diptera	Culicidae	Aedes	Aedes aegypti	SPECIES	18.57611	-89.89861	1897511391
10021	Diptera	Culicidae	Aedes	Aedes aegypti	SPECIES	18.57722	-89.89806	1897511338
10022	Diptera	Culicidae	Aedes	Aedes aegypti	SPECIES	18.57722	-89.89694	1897511403
10023	Diptera	Culicidae	Aedes	Aedes aegypti	SPECIES	18.57722	-89.89917	1897511406
10024	Diptera	Culicidae	Aedes	Aedes aegypti	SPECIES	18.57750	-99.18056	1894398754
10025	Diptera	Culicidae	Aedes	Aedes aegypti	SPECIES	18.57750	-99.29722	1894399168
10026	Diptera	Culicidae	Aedes	Aedes aegypti	SPECIES	18.57750	-89.89611	1897511384
10027	Diptera	Culicidae	Aedes	Aedes aegypti	SPECIES	18.57806	-89.89722	1897511258
10028	Diptera	Culicidae	Aedes	Aedes aegypti	SPECIES	18.57806	-89.89611	1897511347
10029	Diptera	Culicidae	Aedes	Aedes aegypti	SPECIES	18.57833	-89.89889	1897511372
10030	Diptera	Culicidae	Aedes	Aedes aegypti	SPECIES	18.58000	99.02000	1264886465
10031	Diptera	Culicidae	Aedes	Aedes aegypti	SPECIES	18.58000	73.83000	1264889289
10032	Diptera	Culicidae	Aedes	Aedes aegypti	SPECIES	18.58194	-100.30333	1894395798
10033	Diptera	Culicidae	Aedes	Aedes aegypti	SPECIES	18.58500	-95.07000	1421474950
10034	Diptera	Culicidae	Aedes	Aedes aegypti	SPECIES	18.59306	-89.41417	2629264640
10035	Diptera	Culicidae	Aedes	Aedes aegypti	SPECIES	18.59639	-99.17306	1894395473
10036	Diptera	Culicidae	Aedes	Aedes aegypti	SPECIES	18.59639	-99.18639	1894398756
10037	Diptera	Culicidae	Aedes	Aedes aegypti	SPECIES	18.59639	-98.84361	1894399650
10038	Diptera	Culicidae	Aedes	Aedes aegypti	SPECIES	18.59861	-96.68333	1894396327
10039	Diptera	Culicidae	Aedes	Aedes aegypti	SPECIES	18.60000	-96.66167	1894397659
10040	Diptera	Culicidae	Aedes	Aedes aegypti	SPECIES	18.60028	-90.42333	1894396404
10041	Diptera	Culicidae	Aedes	Aedes aegypti	SPECIES	18.60583	-89.80389	1897511292
10042	Diptera	Culicidae	Aedes	Aedes aegypti	SPECIES	18.60611	-99.20639	1894400197
10043	Diptera	Culicidae	Aedes	Aedes aegypti	SPECIES	18.60667	-90.73444	1894400260
10044	Diptera	Culicidae	Aedes	Aedes aegypti	SPECIES	18.61000	-99.18000	1264887606
10045	Diptera	Culicidae	Aedes	Aedes aegypti	SPECIES	18.61111	-99.25972	1894397637

№	Order	Family	Genus	Species	Taxon Rank	Latitude	Longitude	GBIF_ID
10046	Diptera	Culicidae	Aedes	Aedes aegypti	SPECIES	18.61472	-99.18028	1894400228
10047	Diptera	Culicidae	Aedes	Aedes aegypti	SPECIES	18.61931	-95.49117	1421478825
10048	Diptera	Culicidae	Aedes	Aedes aegypti	SPECIES	18.62000	99.00000	1264886537
10049	Diptera	Culicidae	Aedes	Aedes aegypti	SPECIES	18.62000	73.81000	1264888705
10050	Diptera	Culicidae	Aedes	Aedes aegypti	SPECIES	18.62000	73.84000	1264888708
10051	Diptera	Culicidae	Aedes	Aedes aegypti	SPECIES	18.62889	-99.16028	1894397661
10052	Diptera	Culicidae	Aedes	Aedes aegypti	SPECIES	18.62972	-96.41500	1421478836
10053	Diptera	Culicidae	Aedes	Aedes aegypti	SPECIES	18.63000	-99.16000	1264887624
10054	Diptera	Culicidae	Aedes	Aedes aegypti	SPECIES	18.63000	-95.52000	1264888064
10055	Diptera	Culicidae	Aedes	Aedes aegypti	SPECIES	18.63000	73.78000	1264888665
10056	Diptera	Culicidae	Aedes	Aedes aegypti	SPECIES	18.64111	-99.20972	1894397208
10057	Diptera	Culicidae	Aedes	Aedes aegypti	SPECIES	18.64333	-91.83083	2629262437
10058	Diptera	Culicidae	Aedes	Aedes aegypti	SPECIES	18.64528	-91.84056	2629264005
10059	Diptera	Culicidae	Aedes	Aedes aegypti	SPECIES	18.64896	-91.83176	3496610924
10060	Diptera	Culicidae	Aedes	Aedes aegypti	SPECIES	18.65000	-91.83000	1264887052
10061	Diptera	Culicidae	Aedes	Aedes aegypti	SPECIES	18.65000	-91.82000	1264888739
10062	Diptera	Culicidae	Aedes	Aedes aegypti	SPECIES	18.65278	-99.99028	1894397368
10063	Diptera	Culicidae	Aedes	Aedes aegypti	SPECIES	18.65361	-99.26556	1894399272
10064	Diptera	Culicidae	Aedes	Aedes aegypti	SPECIES	18.65639	-99.19111	1894398977
10065	Diptera	Culicidae	Aedes	Aedes aegypti	SPECIES	18.65811	-88.21575	1421475134
10066	Diptera	Culicidae	Aedes	Aedes aegypti	SPECIES	18.66000	72.87000	1264890563
10067	Diptera	Culicidae	Aedes	Aedes aegypti	SPECIES	18.67275	-88.19514	1421478105
10068	Diptera	Culicidae	Aedes	Aedes aegypti	SPECIES	18.67594	-88.39494	1421477257
10069	Diptera	Culicidae	Aedes	Aedes aegypti	SPECIES	18.67694	-88.39194	1894398710
10070	Diptera	Culicidae	Aedes	Aedes aegypti	SPECIES	18.68000	73.73000	1264889213
10071	Diptera	Culicidae	Aedes	Aedes aegypti	SPECIES	18.68500	-99.24389	1894398432
10072	Diptera	Culicidae	Aedes	Aedes aegypti	SPECIES	18.68944	-98.77500	1894396661
10073	Diptera	Culicidae	Aedes	Aedes aegypti	SPECIES	18.69000	73.74000	1264888686
10074	Diptera	Culicidae	Aedes	Aedes aegypti	SPECIES	18.69329	-68.42550	3301904797
10075	Diptera	Culicidae	Aedes	Aedes aegypti	SPECIES	18.69444	-100.36833	1894399667
10076	Diptera	Culicidae	Aedes	Aedes aegypti	SPECIES	18.71000	73.03000	1264886722
10077	Diptera	Culicidae	Aedes	Aedes aegypti	SPECIES	18.71611	-87.70722	1894398180
10078	Diptera	Culicidae	Aedes	Aedes aegypti	SPECIES	18.72000	-97.32000	1264887069
10079	Diptera	Culicidae	Aedes	Aedes aegypti	SPECIES	18.72000	-98.99306	1894399400
10080	Diptera	Culicidae	Aedes	Aedes aegypti	SPECIES	18.72833	-98.95583	1894395423
10081	Diptera	Culicidae	Aedes	Aedes aegypti	SPECIES	18.72833	-99.16139	1894396906
10082	Diptera	Culicidae	Aedes	Aedes aegypti	SPECIES	18.73000	73.10000	1264886720
10083	Diptera	Culicidae	Aedes	Aedes aegypti	SPECIES	18.73000	-99.01000	1264891009
10084	Diptera	Culicidae	Aedes	Aedes aegypti	SPECIES	18.73139	-88.49500	1894397141
10085	Diptera	Culicidae	Aedes	Aedes aegypti	SPECIES	18.73208	100.83542	4145848328
10086	Diptera	Culicidae	Aedes	Aedes aegypti	SPECIES	18.73694	-99.89250	1894396538

№	Order	Family	Genus	Species	Taxon Rank	Latitude	Longitude	GBIF_ID
10087	Diptera	Culicidae	Aedes	Aedes aegypti	SPECIES	18.74000	73.09000	1264890603
10088	Diptera	Culicidae	Aedes	Aedes aegypti	SPECIES	18.74456	-98.90900	3112635878
10089	Diptera	Culicidae	Aedes	Aedes aegypti	SPECIES	18.75917	-99.47500	1894398340
10090	Diptera	Culicidae	Aedes	Aedes aegypti	SPECIES	18.76694	-99.46500	1894395576
10091	Diptera	Culicidae	Aedes	Aedes aegypti	SPECIES	18.76781	-95.75086	1421474099
10092	Diptera	Culicidae	Aedes	Aedes aegypti	SPECIES	18.76861	-99.35944	1894397695
10093	Diptera	Culicidae	Aedes	Aedes aegypti	SPECIES	18.77000	98.93000	1264886495
10094	Diptera	Culicidae	Aedes	Aedes aegypti	SPECIES	18.77000	-95.76000	1264887066
10095	Diptera	Culicidae	Aedes	Aedes aegypti	SPECIES	18.77028	-95.76056	2629264716
10096	Diptera	Culicidae	Aedes	Aedes aegypti	SPECIES	18.77089	-87.73511	1421473435
10097	Diptera	Culicidae	Aedes	Aedes aegypti	SPECIES	18.77300	-88.42525	1421474589
10098	Diptera	Culicidae	Aedes	Aedes aegypti	SPECIES	18.77639	-100.42806	1894400330
10099	Diptera	Culicidae	Aedes	Aedes aegypti	SPECIES	18.78000	72.93000	1264890560
10100	Diptera	Culicidae	Aedes	Aedes aegypti	SPECIES	18.78903	100.78600	4145857676
10101	Diptera	Culicidae	Aedes	Aedes aegypti	SPECIES	18.79000	98.98000	1264886448
10102	Diptera	Culicidae	Aedes	Aedes aegypti	SPECIES	18.79000	72.93000	1264886733
10103	Diptera	Culicidae	Aedes	Aedes aegypti	SPECIES	18.79000	98.99000	1264887681
10104	Diptera	Culicidae	Aedes	Aedes aegypti	SPECIES	18.79000	99.00000	1264890599
10105	Diptera	Culicidae	Aedes	Aedes aegypti	SPECIES	18.79056	-99.46056	1894396546
10106	Diptera	Culicidae	Aedes	Aedes aegypti	SPECIES	18.79264	-98.95904	3327710543
10107	Diptera	Culicidae	Aedes	Aedes aegypti	SPECIES	18.79500	-99.46722	1894394201
10108	Diptera	Culicidae	Aedes	Aedes aegypti	SPECIES	18.80000	72.90000	1264886738
10109	Diptera	Culicidae	Aedes	Aedes aegypti	SPECIES	18.80000	98.99000	1264889506
10110	Diptera	Culicidae	Aedes	Aedes aegypti	SPECIES	18.80111	-99.66972	1894399634
10111	Diptera	Culicidae	Aedes	Aedes aegypti	SPECIES	18.80333	-97.18083	1894397394
10112	Diptera	Culicidae	Aedes	Aedes aegypti	SPECIES	18.80361	-98.96500	1894399572
10113	Diptera	Culicidae	Aedes	Aedes aegypti	SPECIES	18.80367	-89.37136	2629265661
10114	Diptera	Culicidae	Aedes	Aedes aegypti	SPECIES	18.80375	-89.38676	2629265184
10115	Diptera	Culicidae	Aedes	Aedes aegypti	SPECIES	18.81000	-97.27000	1264887078
10116	Diptera	Culicidae	Aedes	Aedes aegypti	SPECIES	18.81000	-97.18000	1264887103
10117	Diptera	Culicidae	Aedes	Aedes aegypti	SPECIES	18.81000	-98.96000	1264887592
10118	Diptera	Culicidae	Aedes	Aedes aegypti	SPECIES	18.81139	-98.92194	1894399431
10119	Diptera	Culicidae	Aedes	Aedes aegypti	SPECIES	18.81167	-90.74639	1894397902
10120	Diptera	Culicidae	Aedes	Aedes aegypti	SPECIES	18.81167	-98.95472	1894400232
10121	Diptera	Culicidae	Aedes	Aedes aegypti	SPECIES	18.81239	97.93582	4145857590
10122	Diptera	Culicidae	Aedes	Aedes aegypti	SPECIES	18.81697	-96.75564	1421474387
10123	Diptera	Culicidae	Aedes	Aedes aegypti	SPECIES	18.82000	72.95000	1264886704
10124	Diptera	Culicidae	Aedes	Aedes aegypti	SPECIES	18.83639	-99.22444	1894399594
10125	Diptera	Culicidae	Aedes	Aedes aegypti	SPECIES	18.83944	-99.18417	1894395329
10126	Diptera	Culicidae	Aedes	Aedes aegypti	SPECIES	18.84000	-97.14000	1264887061
10127	Diptera	Culicidae	Aedes	Aedes aegypti	SPECIES	18.84000	-99.67000	3349411425

№	Order	Family	Genus	Species	Taxon Rank	Latitude	Longitude	GBIF_ID
10128	Diptera	Culicidae	Aedes	Aedes aegypti	SPECIES	18.84556	-99.61389	1894399727
10129	Diptera	Culicidae	Aedes	Aedes aegypti	SPECIES	18.85000	-97.10000	1264887067
10130	Diptera	Culicidae	Aedes	Aedes aegypti	SPECIES	18.85000	-99.45000	3351667830
10131	Diptera	Culicidae	Aedes	Aedes aegypti	SPECIES	18.85280	-99.45280	2633675717
10132	Diptera	Culicidae	Aedes	Aedes aegypti	SPECIES	18.85750	-100.14111	1894396217
10133	Diptera	Culicidae	Aedes	Aedes aegypti	SPECIES	18.85975	-103.96489	2629265445
10134	Diptera	Culicidae	Aedes	Aedes aegypti	SPECIES	18.86322	-97.10717	3416096525
10135	Diptera	Culicidae	Aedes	Aedes aegypti	SPECIES	18.86556	-98.96083	1894396168
10136	Diptera	Culicidae	Aedes	Aedes aegypti	SPECIES	18.86556	-100.11917	1894396174
10137	Diptera	Culicidae	Aedes	Aedes aegypti	SPECIES	18.86917	-100.15194	1894396455
10138	Diptera	Culicidae	Aedes	Aedes aegypti	SPECIES	18.87000	99.14000	1264888878
10139	Diptera	Culicidae	Aedes	Aedes aegypti	SPECIES	18.88000	-96.92000	1264887055
10140	Diptera	Culicidae	Aedes	Aedes aegypti	SPECIES	18.88000	74.58000	1264888940
10141	Diptera	Culicidae	Aedes	Aedes aegypti	SPECIES	18.88361	-99.06306	1894399069
10142	Diptera	Culicidae	Aedes	Aedes aegypti	SPECIES	18.88504	-99.22128	3496608698
10143	Diptera	Culicidae	Aedes	Aedes aegypti	SPECIES	18.88917	-100.16472	1894397946
10144	Diptera	Culicidae	Aedes	Aedes aegypti	SPECIES	18.89000	-96.92000	1264888562
10145	Diptera	Culicidae	Aedes	Aedes aegypti	SPECIES	18.89411	-100.75078	2629265112
10146	Diptera	Culicidae	Aedes	Aedes aegypti	SPECIES	18.89889	-103.90917	1421474426
10147	Diptera	Culicidae	Aedes	Aedes aegypti	SPECIES	18.90000	-98.45000	1264887042
10148	Diptera	Culicidae	Aedes	Aedes aegypti	SPECIES	18.90000	72.81000	1264889238
10149	Diptera	Culicidae	Aedes	Aedes aegypti	SPECIES	18.90771	-98.96977	1949992362
10150	Diptera	Culicidae	Aedes	Aedes aegypti	SPECIES	18.90889	-99.48889	1894394972
10151	Diptera	Culicidae	Aedes	Aedes aegypti	SPECIES	18.91025	-103.87175	2629262327
10152	Diptera	Culicidae	Aedes	Aedes aegypti	SPECIES	18.91556	-96.77667	1894396094
10153	Diptera	Culicidae	Aedes	Aedes aegypti	SPECIES	18.92000	-100.29833	1894400239
10154	Diptera	Culicidae	Aedes	Aedes aegypti	SPECIES	18.92000	-100.15000	3349260056
10155	Diptera	Culicidae	Aedes	Aedes aegypti	SPECIES	18.92421	-99.22157	2826400679
10156	Diptera	Culicidae	Aedes	Aedes aegypti	SPECIES	18.93400	-99.23793	2814100637
10157	Diptera	Culicidae	Aedes	Aedes aegypti	SPECIES	18.93733	-103.95294	2629262588
10158	Diptera	Culicidae	Aedes	Aedes aegypti	SPECIES	18.93744	-102.29828	2629264663
10159	Diptera	Culicidae	Aedes	Aedes aegypti	SPECIES	18.94103	-99.49690	2856562326
10160	Diptera	Culicidae	Aedes	Aedes aegypti	SPECIES	18.94184	-99.23919	1913145824
10161	Diptera	Culicidae	Aedes	Aedes aegypti	SPECIES	18.94752	-99.49934	2826163648
10162	Diptera	Culicidae	Aedes	Aedes aegypti	SPECIES	18.95000	72.95000	1264886750
10163	Diptera	Culicidae	Aedes	Aedes aegypti	SPECIES	18.95000	72.80000	1264889228
10164	Diptera	Culicidae	Aedes	Aedes aegypti	SPECIES	18.95000	72.83000	1264890672
10165	Diptera	Culicidae	Aedes	Aedes aegypti	SPECIES	18.95556	-98.98111	1894397025
10166	Diptera	Culicidae	Aedes	Aedes aegypti	SPECIES	18.96119	-101.81417	2629263041
10167	Diptera	Culicidae	Aedes	Aedes aegypti	SPECIES	18.96250	-101.80611	1894397345
10168	Diptera	Culicidae	Aedes	Aedes aegypti	SPECIES	18.97000	72.82000	1264891234

№	Order	Family	Genus	Species	Taxon Rank	Latitude	Longitude	GBIF_ID
10169	Diptera	Culicidae	Aedes	Aedes aegypti	SPECIES	18.97528	-90.06083	1897511373
10170	Diptera	Culicidae	Aedes	Aedes aegypti	SPECIES	18.97583	-90.05083	1897511387
10171	Diptera	Culicidae	Aedes	Aedes aegypti	SPECIES	18.97639	-91.16944	1894399252
10172	Diptera	Culicidae	Aedes	Aedes aegypti	SPECIES	18.97834	-99.24655	2451588003
10173	Diptera	Culicidae	Aedes	Aedes aegypti	SPECIES	18.98000	72.84000	1264889229
10174	Diptera	Culicidae	Aedes	Aedes aegypti	SPECIES	18.98048	-99.24349	2802785743
10175	Diptera	Culicidae	Aedes	Aedes aegypti	SPECIES	18.98861	-99.44528	1894398716
10176	Diptera	Culicidae	Aedes	Aedes aegypti	SPECIES	18.99000	73.12000	1264886757
10177	Diptera	Culicidae	Aedes	Aedes aegypti	SPECIES	18.99000	73.11000	1264889244
10178	Diptera	Culicidae	Aedes	Aedes aegypti	SPECIES	19.00000	72.84000	1264889282
10179	Diptera	Culicidae	Aedes	Aedes aegypti	SPECIES	19.00000	-72.42000	1843580100
10180	Diptera	Culicidae	Aedes	Aedes aegypti	SPECIES	19.01000	72.86000	1264889231
10181	Diptera	Culicidae	Aedes	Aedes aegypti	SPECIES	19.01161	-101.81736	1421474369
10182	Diptera	Culicidae	Aedes	Aedes aegypti	SPECIES	19.01833	-103.40417	1894399955
10183	Diptera	Culicidae	Aedes	Aedes aegypti	SPECIES	19.02417	-88.10833	1894399571
10184	Diptera	Culicidae	Aedes	Aedes aegypti	SPECIES	19.02778	-88.11053	1421475417
10185	Diptera	Culicidae	Aedes	Aedes aegypti	SPECIES	19.02842	-88.11072	2629265567
10186	Diptera	Culicidae	Aedes	Aedes aegypti	SPECIES	19.03219	-102.09319	2629264784
10187	Diptera	Culicidae	Aedes	Aedes aegypti	SPECIES	19.04000	-98.21000	1264887104
10188	Diptera	Culicidae	Aedes	Aedes aegypti	SPECIES	19.04475	-104.32206	2629265418
10189	Diptera	Culicidae	Aedes	Aedes aegypti	SPECIES	19.04881	-104.30497	2629264011
10190	Diptera	Culicidae	Aedes	Aedes aegypti	SPECIES	19.05167	-104.31556	1894396101
10191	Diptera	Culicidae	Aedes	Aedes aegypti	SPECIES	19.07297	-102.36942	2629264958
10192	Diptera	Culicidae	Aedes	Aedes aegypti	SPECIES	19.07308	-102.37172	2629264803
10193	Diptera	Culicidae	Aedes	Aedes aegypti	SPECIES	19.08417	-103.10000	1894397724
10194	Diptera	Culicidae	Aedes	Aedes aegypti	SPECIES	19.09222	-103.50083	1894399259
10195	Diptera	Culicidae	Aedes	Aedes aegypti	SPECIES	19.09394	-103.61886	2629264809
10196	Diptera	Culicidae	Aedes	Aedes aegypti	SPECIES	19.10000	76.45000	1264888943
10197	Diptera	Culicidae	Aedes	Aedes aegypti	SPECIES	19.10094	-96.10931	1421478127
10198	Diptera	Culicidae	Aedes	Aedes aegypti	SPECIES	19.12158	-104.35822	2629264981
10199	Diptera	Culicidae	Aedes	Aedes aegypti	SPECIES	19.12250	-102.01722	1894397485
10200	Diptera	Culicidae	Aedes	Aedes aegypti	SPECIES	19.12944	-88.14750	1894396177
10201	Diptera	Culicidae	Aedes	Aedes aegypti	SPECIES	19.14000	98.87000	1264887829
10202	Diptera	Culicidae	Aedes	Aedes aegypti	SPECIES	19.14000	-103.93000	1264890833
10203	Diptera	Culicidae	Aedes	Aedes aegypti	SPECIES	19.14136	-102.22917	2629263510
10204	Diptera	Culicidae	Aedes	Aedes aegypti	SPECIES	19.14306	-88.16917	1894400047
10205	Diptera	Culicidae	Aedes	Aedes aegypti	SPECIES	19.14433	-155.85116	1572339130
10206	Diptera	Culicidae	Aedes	Aedes aegypti	SPECIES	19.15000	77.31000	1264889261
10207	Diptera	Culicidae	Aedes	Aedes aegypti	SPECIES	19.15750	-102.05083	1894399228
10208	Diptera	Culicidae	Aedes	Aedes aegypti	SPECIES	19.16278	-88.10278	2629264810
10209	Diptera	Culicidae	Aedes	Aedes aegypti	SPECIES	19.16583	-88.10194	2629262421

№	Order	Family	Genus	Species	Taxon Rank	Latitude	Longitude	GBIF_ID
10210	Diptera	Culicidae	Aedes	Aedes aegypti	SPECIES	19.17000	-96.13000	1264887065
10211	Diptera	Culicidae	Aedes	Aedes aegypti	SPECIES	19.17000	-96.22000	1264887404
10212	Diptera	Culicidae	Aedes	Aedes aegypti	SPECIES	19.18439	-102.84725	2629263301
10213	Diptera	Culicidae	Aedes	Aedes aegypti	SPECIES	19.18650	-155.90613	1305158786
10214	Diptera	Culicidae	Aedes	Aedes aegypti	SPECIES	19.18652	-155.90601	1305158729
10215	Diptera	Culicidae	Aedes	Aedes aegypti	SPECIES	19.18789	-155.90574	1291112614
10216	Diptera	Culicidae	Aedes	Aedes aegypti	SPECIES	19.18835	-155.90554	1291109634
10217	Diptera	Culicidae	Aedes	Aedes aegypti	SPECIES	19.19000	-96.14000	1264888555
10218	Diptera	Culicidae	Aedes	Aedes aegypti	SPECIES	19.19000	72.97000	1264889217
10219	Diptera	Culicidae	Aedes	Aedes aegypti	SPECIES	19.19556	-103.42722	1894399446
10220	Diptera	Culicidae	Aedes	Aedes aegypti	SPECIES	19.19917	-96.13778	2629264117
10221	Diptera	Culicidae	Aedes	Aedes aegypti	SPECIES	19.20000	-101.89000	1264890994
10222	Diptera	Culicidae	Aedes	Aedes aegypti	SPECIES	19.20250	-100.58475	2629263325
10223	Diptera	Culicidae	Aedes	Aedes aegypti	SPECIES	19.20417	-104.68278	1894399752
10224	Diptera	Culicidae	Aedes	Aedes aegypti	SPECIES	19.20574	97.98278	4145856034
10225	Diptera	Culicidae	Aedes	Aedes aegypti	SPECIES	19.20772	-102.59297	2629263643
10226	Diptera	Culicidae	Aedes	Aedes aegypti	SPECIES	19.20972	-102.97639	1894397669
10227	Diptera	Culicidae	Aedes	Aedes aegypti	SPECIES	19.21000	98.88000	1264887821
10228	Diptera	Culicidae	Aedes	Aedes aegypti	SPECIES	19.21000	73.16000	1264889246
10229	Diptera	Culicidae	Aedes	Aedes aegypti	SPECIES	19.22028	-103.45889	1894397142
10230	Diptera	Culicidae	Aedes	Aedes aegypti	SPECIES	19.22472	-104.64222	1894398763
10231	Diptera	Culicidae	Aedes	Aedes aegypti	SPECIES	19.22572	-103.64903	2629263412
10232	Diptera	Culicidae	Aedes	Aedes aegypti	SPECIES	19.22800	-69.61111	735187181
10233	Diptera	Culicidae	Aedes	Aedes aegypti	SPECIES	19.23000	-104.68528	1894399994
10234	Diptera	Culicidae	Aedes	Aedes aegypti	SPECIES	19.23103	-103.71594	2629264839
10235	Diptera	Culicidae	Aedes	Aedes aegypti	SPECIES	19.23306	-104.57169	2629265697
10236	Diptera	Culicidae	Aedes	Aedes aegypti	SPECIES	19.23633	-101.46431	2629263406
10237	Diptera	Culicidae	Aedes	Aedes aegypti	SPECIES	19.23750	-104.56194	1894400312
10238	Diptera	Culicidae	Aedes	Aedes aegypti	SPECIES	19.23833	-103.41917	1894398836
10239	Diptera	Culicidae	Aedes	Aedes aegypti	SPECIES	19.24000	-103.73000	1264888493
10240	Diptera	Culicidae	Aedes	Aedes aegypti	SPECIES	19.24000	-104.71500	1894400185
10241	Diptera	Culicidae	Aedes	Aedes aegypti	SPECIES	19.24306	-103.73056	1421477187
10242	Diptera	Culicidae	Aedes	Aedes aegypti	SPECIES	19.24306	-103.73056	1894398811
10243	Diptera	Culicidae	Aedes	Aedes aegypti	SPECIES	19.24678	-103.70725	2629264332
10244	Diptera	Culicidae	Aedes	Aedes aegypti	SPECIES	19.25000	-103.71000	1264888475
10245	Diptera	Culicidae	Aedes	Aedes aegypti	SPECIES	19.25194	-103.37861	1894399321
10246	Diptera	Culicidae	Aedes	Aedes aegypti	SPECIES	19.26000	76.77000	1264889271
10247	Diptera	Culicidae	Aedes	Aedes aegypti	SPECIES	19.26548	-154.98348	1315057363
10248	Diptera	Culicidae	Aedes	Aedes aegypti	SPECIES	19.26861	-102.70611	1894397538
10249	Diptera	Culicidae	Aedes	Aedes aegypti	SPECIES	19.27155	-154.88798	1233602102
10250	Diptera	Culicidae	Aedes	Aedes aegypti	SPECIES	19.27422	-103.75089	2629264924

№	Order	Family	Genus	Species	Taxon Rank	Latitude	Longitude	GBIF_ID
10251	Diptera	Culicidae	Aedes	Aedes aegypti	SPECIES	19.28000	-81.39000	1264890678
10252	Diptera	Culicidae	Aedes	Aedes aegypti	SPECIES	19.28531	99.37945	4145850584
10253	Diptera	Culicidae	Aedes	Aedes aegypti	SPECIES	19.28815	97.97254	4145850871
10254	Diptera	Culicidae	Aedes	Aedes aegypti	SPECIES	19.29000	-81.39000	1264891054
10255	Diptera	Culicidae	Aedes	Aedes aegypti	SPECIES	19.29972	-102.94889	1894400247
10256	Diptera	Culicidae	Aedes	Aedes aegypti	SPECIES	19.30000	73.06000	1264889243
10257	Diptera	Culicidae	Aedes	Aedes aegypti	SPECIES	19.30807	98.45425	4145856144
10258	Diptera	Culicidae	Aedes	Aedes aegypti	SPECIES	19.30917	-102.70500	1894399950
10259	Diptera	Culicidae	Aedes	Aedes aegypti	SPECIES	19.31000	84.80000	3349489166
10260	Diptera	Culicidae	Aedes	Aedes aegypti	SPECIES	19.31083	-104.30306	1894396694
10261	Diptera	Culicidae	Aedes	Aedes aegypti	SPECIES	19.31300	-154.80534	1315086664
10262	Diptera	Culicidae	Aedes	Aedes aegypti	SPECIES	19.31389	-102.96556	1894399841
10263	Diptera	Culicidae	Aedes	Aedes aegypti	SPECIES	19.32000	-81.24000	1264886373
10264	Diptera	Culicidae	Aedes	Aedes aegypti	SPECIES	19.32000	98.74000	1264887470
10265	Diptera	Culicidae	Aedes	Aedes aegypti	SPECIES	19.32000	-81.25000	1264891077
10266	Diptera	Culicidae	Aedes	Aedes aegypti	SPECIES	19.32000	84.79000	3351677605
10267	Diptera	Culicidae	Aedes	Aedes aegypti	SPECIES	19.32083	-103.15278	1894399969
10268	Diptera	Culicidae	Aedes	Aedes aegypti	SPECIES	19.32141	-69.53579	2988269882
10269	Diptera	Culicidae	Aedes	Aedes aegypti	SPECIES	19.32436	-103.76283	2629264081
10270	Diptera	Culicidae	Aedes	Aedes aegypti	SPECIES	19.32472	-104.71444	1894394817
10271	Diptera	Culicidae	Aedes	Aedes aegypti	SPECIES	19.32500	-102.87333	1894399807
10272	Diptera	Culicidae	Aedes	Aedes aegypti	SPECIES	19.32722	-103.37611	1894396657
10273	Diptera	Culicidae	Aedes	Aedes aegypti	SPECIES	19.33250	-104.94806	1894400157
10274	Diptera	Culicidae	Aedes	Aedes aegypti	SPECIES	19.34000	99.82000	1264887476
10275	Diptera	Culicidae	Aedes	Aedes aegypti	SPECIES	19.35000	-81.21000	1264891102
10276	Diptera	Culicidae	Aedes	Aedes aegypti	SPECIES	19.35016	-154.81103	1315057353
10277	Diptera	Culicidae	Aedes	Aedes aegypti	SPECIES	19.35250	-104.89750	1894398530
10278	Diptera	Culicidae	Aedes	Aedes aegypti	SPECIES	19.35447	-99.08892	4052768089
10279	Diptera	Culicidae	Aedes	Aedes aegypti	SPECIES	19.35472	-90.72056	1894399892
10280	Diptera	Culicidae	Aedes	Aedes aegypti	SPECIES	19.35556	-90.72333	2629264282
10281	Diptera	Culicidae	Aedes	Aedes aegypti	SPECIES	19.36028	-104.89778	1894395259
10282	Diptera	Culicidae	Aedes	Aedes aegypti	SPECIES	19.36073	-154.86225	1315086582
10283	Diptera	Culicidae	Aedes	Aedes aegypti	SPECIES	19.36278	-96.35408	1421475553
10284	Diptera	Culicidae	Aedes	Aedes aegypti	SPECIES	19.36617	-154.98518	1265543810
10285	Diptera	Culicidae	Aedes	Aedes aegypti	SPECIES	19.37056	-103.01944	1894399163
10286	Diptera	Culicidae	Aedes	Aedes aegypti	SPECIES	19.38000	105.74000	1264886237
10287	Diptera	Culicidae	Aedes	Aedes aegypti	SPECIES	19.38000	-96.39000	1264891010
10288	Diptera	Culicidae	Aedes	Aedes aegypti	SPECIES	19.38222	-104.96500	1894398909
10289	Diptera	Culicidae	Aedes	Aedes aegypti	SPECIES	19.38500	-104.96833	1894400072
10290	Diptera	Culicidae	Aedes	Aedes aegypti	SPECIES	19.38611	-104.51889	1894399750
10291	Diptera	Culicidae	Aedes	Aedes aegypti	SPECIES	19.39427	-154.93350	1453201257

№	Order	Family	Genus	Species	Taxon Rank	Latitude	Longitude	GBIF_ID
10292	Diptera	Culicidae	Aedes	Aedes aegypti	SPECIES	19.39509	-154.92902	1306583246
10293	Diptera	Culicidae	Aedes	Aedes aegypti	SPECIES	19.39518	-154.93280	2862487811
10294	Diptera	Culicidae	Aedes	Aedes aegypti	SPECIES	19.39539	-154.93279	2963918101
10295	Diptera	Culicidae	Aedes	Aedes aegypti	SPECIES	19.39571	-154.93256	1269542929
10296	Diptera	Culicidae	Aedes	Aedes aegypti	SPECIES	19.39571	-154.93253	1453099345
10297	Diptera	Culicidae	Aedes	Aedes aegypti	SPECIES	19.39572	-154.93259	1269562028
10298	Diptera	Culicidae	Aedes	Aedes aegypti	SPECIES	19.39572	-154.93259	2826144562
10299	Diptera	Culicidae	Aedes	Aedes aegypti	SPECIES	19.39573	-154.93264	2826208851
10300	Diptera	Culicidae	Aedes	Aedes aegypti	SPECIES	19.39576	-154.93254	1265545519
10301	Diptera	Culicidae	Aedes	Aedes aegypti	SPECIES	19.39577	-154.93261	2823433324
10302	Diptera	Culicidae	Aedes	Aedes aegypti	SPECIES	19.39577	-154.93261	2802630704
10303	Diptera	Culicidae	Aedes	Aedes aegypti	SPECIES	19.39579	-154.93265	1265589016
10304	Diptera	Culicidae	Aedes	Aedes aegypti	SPECIES	19.39581	-154.93257	1265895236
10305	Diptera	Culicidae	Aedes	Aedes aegypti	SPECIES	19.39581	-154.93250	2574135600
10306	Diptera	Culicidae	Aedes	Aedes aegypti	SPECIES	19.39581	-154.93262	1265575620
10307	Diptera	Culicidae	Aedes	Aedes aegypti	SPECIES	19.39584	-154.93257	1315059481
10308	Diptera	Culicidae	Aedes	Aedes aegypti	SPECIES	19.39584	-154.93257	2563568885
10309	Diptera	Culicidae	Aedes	Aedes aegypti	SPECIES	19.39585	-154.93253	1841206911
10310	Diptera	Culicidae	Aedes	Aedes aegypti	SPECIES	19.39590	-154.93250	2596330641
10311	Diptera	Culicidae	Aedes	Aedes aegypti	SPECIES	19.39591	-154.93254	1837083169
10312	Diptera	Culicidae	Aedes	Aedes aegypti	SPECIES	19.39931	-154.85896	1453481715
10313	Diptera	Culicidae	Aedes	Aedes aegypti	SPECIES	19.40000	-88.13306	1894394637
10314	Diptera	Culicidae	Aedes	Aedes aegypti	SPECIES	19.40010	-154.92622	1315057375
10315	Diptera	Culicidae	Aedes	Aedes aegypti	SPECIES	19.40026	-154.92642	1453131255
10316	Diptera	Culicidae	Aedes	Aedes aegypti	SPECIES	19.40528	-154.94025	1453184922
10317	Diptera	Culicidae	Aedes	Aedes aegypti	SPECIES	19.40667	-99.19111	1894398005
10318	Diptera	Culicidae	Aedes	Aedes aegypti	SPECIES	19.40806	-103.54889	1894398733
10319	Diptera	Culicidae	Aedes	Aedes aegypti	SPECIES	19.41167	-102.06686	2629265258
10320	Diptera	Culicidae	Aedes	Aedes aegypti	SPECIES	19.41517	-154.92489	1315057354
10321	Diptera	Culicidae	Aedes	Aedes aegypti	SPECIES	19.41667	-88.04861	1894398996
10322	Diptera	Culicidae	Aedes	Aedes aegypti	SPECIES	19.43170	98.39954	4145854178
10323	Diptera	Culicidae	Aedes	Aedes aegypti	SPECIES	19.43504	100.64544	4145851903
10324	Diptera	Culicidae	Aedes	Aedes aegypti	SPECIES	19.43688	98.32998	4145859373
10325	Diptera	Culicidae	Aedes	Aedes aegypti	SPECIES	19.44000	-96.41000	1264887046
10326	Diptera	Culicidae	Aedes	Aedes aegypti	SPECIES	19.44333	-96.40722	2629265574
10327	Diptera	Culicidae	Aedes	Aedes aegypti	SPECIES	19.44500	-103.50417	1894395808
10328	Diptera	Culicidae	Aedes	Aedes aegypti	SPECIES	19.44506	-96.40189	1421475532
10329	Diptera	Culicidae	Aedes	Aedes aegypti	SPECIES	19.45000	105.78000	1264886150
10330	Diptera	Culicidae	Aedes	Aedes aegypti	SPECIES	19.45000	-96.96000	1264887081
10331	Diptera	Culicidae	Aedes	Aedes aegypti	SPECIES	19.45056	-104.35917	1894398990
10332	Diptera	Culicidae	Aedes	Aedes aegypti	SPECIES	19.45972	-104.19444	1894399098

№	Order	Family	Genus	Species	Taxon Rank	Latitude	Longitude	GBIF_ID
10333	Diptera	Culicidae	Aedes	Aedes aegypti	SPECIES	19.46333	-103.76500	1894398596
10334	Diptera	Culicidae	Aedes	Aedes aegypti	SPECIES	19.46423	-99.04578	2540879065
10335	Diptera	Culicidae	Aedes	Aedes aegypti	SPECIES	19.46917	-103.30639	1894398289
10336	Diptera	Culicidae	Aedes	Aedes aegypti	SPECIES	19.47439	-88.05153	1421477880
10337	Diptera	Culicidae	Aedes	Aedes aegypti	SPECIES	19.47506	-88.05203	1421474819
10338	Diptera	Culicidae	Aedes	Aedes aegypti	SPECIES	19.48000	76.56000	1264888931
10339	Diptera	Culicidae	Aedes	Aedes aegypti	SPECIES	19.48111	-104.63917	1894399916
10340	Diptera	Culicidae	Aedes	Aedes aegypti	SPECIES	19.49000	105.81000	1264886175
10341	Diptera	Culicidae	Aedes	Aedes aegypti	SPECIES	19.49183	-87.99642	2629264443
10342	Diptera	Culicidae	Aedes	Aedes aegypti	SPECIES	19.49444	-104.67028	1894400100
10343	Diptera	Culicidae	Aedes	Aedes aegypti	SPECIES	19.49933	-96.38611	1421474076
10344	Diptera	Culicidae	Aedes	Aedes aegypti	SPECIES	19.49944	-104.56139	1894398579
10345	Diptera	Culicidae	Aedes	Aedes aegypti	SPECIES	19.50000	76.56000	1264890770
10346	Diptera	Culicidae	Aedes	Aedes aegypti	SPECIES	19.51000	110.17000	1264890735
10347	Diptera	Culicidae	Aedes	Aedes aegypti	SPECIES	19.51020	-96.92247	2963932460
10348	Diptera	Culicidae	Aedes	Aedes aegypti	SPECIES	19.53000	-96.91000	1264888559
10349	Diptera	Culicidae	Aedes	Aedes aegypti	SPECIES	19.54000	-96.93000	1264887056
10350	Diptera	Culicidae	Aedes	Aedes aegypti	SPECIES	19.54056	-103.25306	1894394422
10351	Diptera	Culicidae	Aedes	Aedes aegypti	SPECIES	19.55000	99.74000	1264889676
10352	Diptera	Culicidae	Aedes	Aedes aegypti	SPECIES	19.55000	-104.48556	1894399344
10353	Diptera	Culicidae	Aedes	Aedes aegypti	SPECIES	19.55389	-103.37556	1894399724
10354	Diptera	Culicidae	Aedes	Aedes aegypti	SPECIES	19.57000	105.81000	1264886197
10355	Diptera	Culicidae	Aedes	Aedes aegypti	SPECIES	19.57861	-88.04528	1894400154
10356	Diptera	Culicidae	Aedes	Aedes aegypti	SPECIES	19.58000	37.19000	1264887373
10357	Diptera	Culicidae	Aedes	Aedes aegypti	SPECIES	19.58194	-100.34514	2629262871
10358	Diptera	Culicidae	Aedes	Aedes aegypti	SPECIES	19.58333	-88.05000	2629263752
10359	Diptera	Culicidae	Aedes	Aedes aegypti	SPECIES	19.58472	-88.59167	1894397526
10360	Diptera	Culicidae	Aedes	Aedes aegypti	SPECIES	19.58528	-102.90889	1894397429
10361	Diptera	Culicidae	Aedes	Aedes aegypti	SPECIES	19.58861	-105.12583	1894399155
10362	Diptera	Culicidae	Aedes	Aedes aegypti	SPECIES	19.60000	-96.38000	1264890429
10363	Diptera	Culicidae	Aedes	Aedes aegypti	SPECIES	19.60000	76.21000	3347197775
10364	Diptera	Culicidae	Aedes	Aedes aegypti	SPECIES	19.60222	-104.43528	1894400115
10365	Diptera	Culicidae	Aedes	Aedes aegypti	SPECIES	19.60460	37.20310	1843580213
10366	Diptera	Culicidae	Aedes	Aedes aegypti	SPECIES	19.60944	-103.22611	1894394213
10367	Diptera	Culicidae	Aedes	Aedes aegypti	SPECIES	19.62000	37.22000	1264887392
10368	Diptera	Culicidae	Aedes	Aedes aegypti	SPECIES	19.62139	-104.53528	1894398624
10369	Diptera	Culicidae	Aedes	Aedes aegypti	SPECIES	19.62472	-103.41583	1894400008
10370	Diptera	Culicidae	Aedes	Aedes aegypti	SPECIES	19.62861	-103.27944	1894394305
10371	Diptera	Culicidae	Aedes	Aedes aegypti	SPECIES	19.63000	37.23000	1264887381
10372	Diptera	Culicidae	Aedes	Aedes aegypti	SPECIES	19.63667	-88.28611	1894398080
10373	Diptera	Culicidae	Aedes	Aedes aegypti	SPECIES	19.63833	-90.68778	1894396685

№	Order	Family	Genus	Species	Taxon Rank	Latitude	Longitude	GBIF_ID
10374	Diptera	Culicidae	Aedes	Aedes aegypti	SPECIES	19.64000	37.16000	1264887375
10375	Diptera	Culicidae	Aedes	Aedes aegypti	SPECIES	19.64000	96.20000	3818475083
10376	Diptera	Culicidae	Aedes	Aedes aegypti	SPECIES	19.64424	-70.85671	2456509538
10377	Diptera	Culicidae	Aedes	Aedes aegypti	SPECIES	19.64778	-103.25000	1894399202
10378	Diptera	Culicidae	Aedes	Aedes aegypti	SPECIES	19.66778	-103.26278	1894397510
10379	Diptera	Culicidae	Aedes	Aedes aegypti	SPECIES	19.67556	-103.25361	1894399645
10380	Diptera	Culicidae	Aedes	Aedes aegypti	SPECIES	19.67583	-105.18583	1894399907
10381	Diptera	Culicidae	Aedes	Aedes aegypti	SPECIES	19.67690	-101.21228	2898523335
10382	Diptera	Culicidae	Aedes	Aedes aegypti	SPECIES	19.67861	-103.31639	1894396359
10383	Diptera	Culicidae	Aedes	Aedes aegypti	SPECIES	19.67917	-104.72639	1894399818
10384	Diptera	Culicidae	Aedes	Aedes aegypti	SPECIES	19.69000	-103.48000	1264887165
10385	Diptera	Culicidae	Aedes	Aedes aegypti	SPECIES	19.69000	-100.56000	1264890202
10386	Diptera	Culicidae	Aedes	Aedes aegypti	SPECIES	19.69028	-103.34028	1894397285
10387	Diptera	Culicidae	Aedes	Aedes aegypti	SPECIES	19.69028	-103.28278	1894399210
10388	Diptera	Culicidae	Aedes	Aedes aegypti	SPECIES	19.70278	-103.46250	1894399379
10389	Diptera	Culicidae	Aedes	Aedes aegypti	SPECIES	19.70459	-101.18781	4052769556
10390	Diptera	Culicidae	Aedes	Aedes aegypti	SPECIES	19.70471	-101.18786	4052768915
10391	Diptera	Culicidae	Aedes	Aedes aegypti	SPECIES	19.70739	-103.47756	2629265109
10392	Diptera	Culicidae	Aedes	Aedes aegypti	SPECIES	19.70838	-101.21964	2619930750
10393	Diptera	Culicidae	Aedes	Aedes aegypti	SPECIES	19.70842	-101.21976	2626174848
10394	Diptera	Culicidae	Aedes	Aedes aegypti	SPECIES	19.70911	-71.05653	177060464
10395	Diptera	Culicidae	Aedes	Aedes aegypti	SPECIES	19.71572	-104.60561	2629262732
10396	Diptera	Culicidae	Aedes	Aedes aegypti	SPECIES	19.72000	-103.47000	1264887142
10397	Diptera	Culicidae	Aedes	Aedes aegypti	SPECIES	19.72000	-103.46000	1264887149
10398	Diptera	Culicidae	Aedes	Aedes aegypti	SPECIES	19.73000	-103.45000	1264887159
10399	Diptera	Culicidae	Aedes	Aedes aegypti	SPECIES	19.73000	109.18000	1264891145
10400	Diptera	Culicidae	Aedes	Aedes aegypti	SPECIES	19.74444	-89.84306	1894399946
10401	Diptera	Culicidae	Aedes	Aedes aegypti	SPECIES	19.74667	-103.76861	1894399708
10402	Diptera	Culicidae	Aedes	Aedes aegypti	SPECIES	19.74722	-103.28056	1894398881
10403	Diptera	Culicidae	Aedes	Aedes aegypti	SPECIES	19.75000	109.18000	1264889710
10404	Diptera	Culicidae	Aedes	Aedes aegypti	SPECIES	19.75250	-103.18083	1894394889
10405	Diptera	Culicidae	Aedes	Aedes aegypti	SPECIES	19.77000	-105.24778	1894397601
10406	Diptera	Culicidae	Aedes	Aedes aegypti	SPECIES	19.77078	-96.43053	1421475605
10407	Diptera	Culicidae	Aedes	Aedes aegypti	SPECIES	19.77083	-104.36944	1894399598
10408	Diptera	Culicidae	Aedes	Aedes aegypti	SPECIES	19.77092	-103.46997	2629263920
10409	Diptera	Culicidae	Aedes	Aedes aegypti	SPECIES	19.77528	-103.12111	1894399363
10410	Diptera	Culicidae	Aedes	Aedes aegypti	SPECIES	19.77556	-103.89722	1894399322
10411	Diptera	Culicidae	Aedes	Aedes aegypti	SPECIES	19.77807	-87.47608	2629263163
10412	Diptera	Culicidae	Aedes	Aedes aegypti	SPECIES	19.77889	-103.77611	1894399023
10413	Diptera	Culicidae	Aedes	Aedes aegypti	SPECIES	19.78000	109.21000	1264891119
10414	Diptera	Culicidae	Aedes	Aedes aegypti	SPECIES	19.78583	-103.97111	1894396668

№	Order	Family	Genus	Species	Taxon Rank	Latitude	Longitude	GBIF_ID
10415	Diptera	Culicidae	Aedes	Aedes aegypti	SPECIES	19.78889	-103.11611	1894400106
10416	Diptera	Culicidae	Aedes	Aedes aegypti	SPECIES	19.79306	-104.05500	1894396184
10417	Diptera	Culicidae	Aedes	Aedes aegypti	SPECIES	19.79703	-87.47664	2629265502
10418	Diptera	Culicidae	Aedes	Aedes aegypti	SPECIES	19.80419	-87.47817	2629264712
10419	Diptera	Culicidae	Aedes	Aedes aegypti	SPECIES	19.80528	-104.21694	1894396568
10420	Diptera	Culicidae	Aedes	Aedes aegypti	SPECIES	19.82167	-104.33444	1894399784
10421	Diptera	Culicidae	Aedes	Aedes aegypti	SPECIES	19.82389	-104.15611	1894396981
10422	Diptera	Culicidae	Aedes	Aedes aegypti	SPECIES	19.82668	-90.55201	2629265554
10423	Diptera	Culicidae	Aedes	Aedes aegypti	SPECIES	19.83000	-90.53000	1264887054
10424	Diptera	Culicidae	Aedes	Aedes aegypti	SPECIES	19.83000	75.89000	3347169042
10425	Diptera	Culicidae	Aedes	Aedes aegypti	SPECIES	19.83605	-90.55192	2629265508
10426	Diptera	Culicidae	Aedes	Aedes aegypti	SPECIES	19.84000	75.89000	1264889248
10427	Diptera	Culicidae	Aedes	Aedes aegypti	SPECIES	19.84222	-90.53167	2629263972
10428	Diptera	Culicidae	Aedes	Aedes aegypti	SPECIES	19.84583	-104.28444	1894399340
10429	Diptera	Culicidae	Aedes	Aedes aegypti	SPECIES	19.85000	99.11000	1264887479
10430	Diptera	Culicidae	Aedes	Aedes aegypti	SPECIES	19.85000	-90.52000	1264888038
10431	Diptera	Culicidae	Aedes	Aedes aegypti	SPECIES	19.85000	-90.53000	1264888699
10432	Diptera	Culicidae	Aedes	Aedes aegypti	SPECIES	19.85000	75.40000	1264891384
10433	Diptera	Culicidae	Aedes	Aedes aegypti	SPECIES	19.85000	75.91000	3347181092
10434	Diptera	Culicidae	Aedes	Aedes aegypti	SPECIES	19.86983	-101.14236	3031640042
10435	Diptera	Culicidae	Aedes	Aedes aegypti	SPECIES	19.87000	75.72000	3347250721
10436	Diptera	Culicidae	Aedes	Aedes aegypti	SPECIES	19.88000	75.32000	1264887965
10437	Diptera	Culicidae	Aedes	Aedes aegypti	SPECIES	19.88000	75.33000	1264891412
10438	Diptera	Culicidae	Aedes	Aedes aegypti	SPECIES	19.88278	-103.60056	1894398867
10439	Diptera	Culicidae	Aedes	Aedes aegypti	SPECIES	19.88669	-103.60953	2629265606
10440	Diptera	Culicidae	Aedes	Aedes aegypti	SPECIES	19.89000	75.32000	1264889267
10441	Diptera	Culicidae	Aedes	Aedes aegypti	SPECIES	19.89750	-88.80556	1894398121
10442	Diptera	Culicidae	Aedes	Aedes aegypti	SPECIES	19.90194	-70.84004	4009829418
10443	Diptera	Culicidae	Aedes	Aedes aegypti	SPECIES	19.91000	99.83000	1264887473
10444	Diptera	Culicidae	Aedes	Aedes aegypti	SPECIES	19.91000	109.68000	1264889109
10445	Diptera	Culicidae	Aedes	Aedes aegypti	SPECIES	19.93797	-105.25206	2629265116
10446	Diptera	Culicidae	Aedes	Aedes aegypti	SPECIES	19.94083	-105.24861	1894397160
10447	Diptera	Culicidae	Aedes	Aedes aegypti	SPECIES	19.96000	-75.70000	1264891128
10448	Diptera	Culicidae	Aedes	Aedes aegypti	SPECIES	19.96500	-100.92306	1894255159
10449	Diptera	Culicidae	Aedes	Aedes aegypti	SPECIES	19.96861	-100.91694	1894254639
10450	Diptera	Culicidae	Aedes	Aedes aegypti	SPECIES	19.97000	-97.21000	1264888796
10451	Diptera	Culicidae	Aedes	Aedes aegypti	SPECIES	19.97432	-155.83119	1272095432
10452	Diptera	Culicidae	Aedes	Aedes aegypti	SPECIES	19.97436	-155.83136	1272095303
10453	Diptera	Culicidae	Aedes	Aedes aegypti	SPECIES	19.97889	-100.82694	1894255934
10454	Diptera	Culicidae	Aedes	Aedes aegypti	SPECIES	19.98917	-103.64167	1894399497
10455	Diptera	Culicidae	Aedes	Aedes aegypti	SPECIES	19.99000	72.73000	1264889283

№	Order	Family	Genus	Species	Taxon Rank	Latitude	Longitude	GBIF_ID
10456	Diptera	Culicidae	Aedes	Aedes aegypti	SPECIES	20.00223	-155.81973	2242881793
10457	Diptera	Culicidae	Aedes	Aedes aegypti	SPECIES	20.00417	-89.74750	1894399720
10458	Diptera	Culicidae	Aedes	Aedes aegypti	SPECIES	20.00611	-105.45806	1894398868
10459	Diptera	Culicidae	Aedes	Aedes aegypti	SPECIES	20.01028	-103.60000	1894396721
10460	Diptera	Culicidae	Aedes	Aedes aegypti	SPECIES	20.01233	-155.76565	3044686773
10461	Diptera	Culicidae	Aedes	Aedes aegypti	SPECIES	20.01836	-155.74914	1703198381
10462	Diptera	Culicidae	Aedes	Aedes aegypti	SPECIES	20.01870	-155.75122	1323023044
10463	Diptera	Culicidae	Aedes	Aedes aegypti	SPECIES	20.01870	-155.75096	1323015249
10464	Diptera	Culicidae	Aedes	Aedes aegypti	SPECIES	20.01876	-155.75127	1453094457
10465	Diptera	Culicidae	Aedes	Aedes aegypti	SPECIES	20.01901	-155.74918	1453128437
10466	Diptera	Culicidae	Aedes	Aedes aegypti	SPECIES	20.01952	-98.79186	3923514394
10467	Diptera	Culicidae	Aedes	Aedes aegypti	SPECIES	20.02000	-75.83000	1264887355
10468	Diptera	Culicidae	Aedes	Aedes aegypti	SPECIES	20.02257	-155.80980	1272103025
10469	Diptera	Culicidae	Aedes	Aedes aegypti	SPECIES	20.02335	-155.80999	1291080496
10470	Diptera	Culicidae	Aedes	Aedes aegypti	SPECIES	20.02352	-155.81051	1272089634
10471	Diptera	Culicidae	Aedes	Aedes aegypti	SPECIES	20.02361	-100.74167	1894252121
10472	Diptera	Culicidae	Aedes	Aedes aegypti	SPECIES	20.02376	-155.81091	1273844809
10473	Diptera	Culicidae	Aedes	Aedes aegypti	SPECIES	20.02400	-155.81102	1272096617
10474	Diptera	Culicidae	Aedes	Aedes aegypti	SPECIES	20.02510	-100.71824	3005098541
10475	Diptera	Culicidae	Aedes	Aedes aegypti	SPECIES	20.02511	-100.71824	2823327969
10476	Diptera	Culicidae	Aedes	Aedes aegypti	SPECIES	20.02719	-155.71880	1703188299
10477	Diptera	Culicidae	Aedes	Aedes aegypti	SPECIES	20.02722	-105.24361	1894397522
10478	Diptera	Culicidae	Aedes	Aedes aegypti	SPECIES	20.02728	-155.71907	1802656994
10479	Diptera	Culicidae	Aedes	Aedes aegypti	SPECIES	20.02784	-155.72062	1699372445
10480	Diptera	Culicidae	Aedes	Aedes aegypti	SPECIES	20.02792	-155.72008	2294415995
10481	Diptera	Culicidae	Aedes	Aedes aegypti	SPECIES	20.02801	-155.72091	1699372406
10482	Diptera	Culicidae	Aedes	Aedes aegypti	SPECIES	20.02810	-155.71912	4009920610
10483	Diptera	Culicidae	Aedes	Aedes aegypti	SPECIES	20.02817	-155.72055	1920782707
10484	Diptera	Culicidae	Aedes	Aedes aegypti	SPECIES	20.02819	-155.72052	3338039425
10485	Diptera	Culicidae	Aedes	Aedes aegypti	SPECIES	20.02819	-155.72060	1802665381
10486	Diptera	Culicidae	Aedes	Aedes aegypti	SPECIES	20.02820	-155.72060	1802724702
10487	Diptera	Culicidae	Aedes	Aedes aegypti	SPECIES	20.02823	-155.72060	1838305621
10488	Diptera	Culicidae	Aedes	Aedes aegypti	SPECIES	20.02823	-155.72046	1677281176
10489	Diptera	Culicidae	Aedes	Aedes aegypti	SPECIES	20.02825	-155.72070	3416138593
10490	Diptera	Culicidae	Aedes	Aedes aegypti	SPECIES	20.02874	-155.72024	1703146738
10491	Diptera	Culicidae	Aedes	Aedes aegypti	SPECIES	20.02896	-100.72136	4111690270
10492	Diptera	Culicidae	Aedes	Aedes aegypti	SPECIES	20.03000	-75.82000	1264889872
10493	Diptera	Culicidae	Aedes	Aedes aegypti	SPECIES	20.03139	-100.72167	1894396066
10494	Diptera	Culicidae	Aedes	Aedes aegypti	SPECIES	20.03250	-100.72139	1894255936
10495	Diptera	Culicidae	Aedes	Aedes aegypti	SPECIES	20.03467	-100.72013	3902119867
10496	Diptera	Culicidae	Aedes	Aedes aegypti	SPECIES	20.03529	-155.82819	1453481237

№	Order	Family	Genus	Species	Taxon Rank	Latitude	Longitude	GBIF_ID
10497	Diptera	Culicidae	Aedes	Aedes aegypti	SPECIES	20.03628	-90.23214	2629265106
10498	Diptera	Culicidae	Aedes	Aedes aegypti	SPECIES	20.03851	-155.82881	1802695709
10499	Diptera	Culicidae	Aedes	Aedes aegypti	SPECIES	20.03904	-155.82931	2451686528
10500	Diptera	Culicidae	Aedes	Aedes aegypti	SPECIES	20.03925	-155.83000	1802647405
10501	Diptera	Culicidae	Aedes	Aedes aegypti	SPECIES	20.04000	-75.85000	1264891153
10502	Diptera	Culicidae	Aedes	Aedes aegypti	SPECIES	20.04000	-75.94000	1264891179
10503	Diptera	Culicidae	Aedes	Aedes aegypti	SPECIES	20.04009	-100.72370	3391387535
10504	Diptera	Culicidae	Aedes	Aedes aegypti	SPECIES	20.04018	-100.72387	3070490697
10505	Diptera	Culicidae	Aedes	Aedes aegypti	SPECIES	20.04028	-100.72392	3070526050
10506	Diptera	Culicidae	Aedes	Aedes aegypti	SPECIES	20.04426	-155.83529	1837755099
10507	Diptera	Culicidae	Aedes	Aedes aegypti	SPECIES	20.04437	-155.83393	1453208812
10508	Diptera	Culicidae	Aedes	Aedes aegypti	SPECIES	20.04448	-155.83406	1453397168
10509	Diptera	Culicidae	Aedes	Aedes aegypti	SPECIES	20.04549	-155.83484	1453188247
10510	Diptera	Culicidae	Aedes	Aedes aegypti	SPECIES	20.04808	-155.83567	1837088829
10511	Diptera	Culicidae	Aedes	Aedes aegypti	SPECIES	20.05000	-97.04000	1264887070
10512	Diptera	Culicidae	Aedes	Aedes aegypti	SPECIES	20.05000	78.96000	1264889266
10513	Diptera	Culicidae	Aedes	Aedes aegypti	SPECIES	20.05000	110.41000	1264890760
10514	Diptera	Culicidae	Aedes	Aedes aegypti	SPECIES	20.05000	-75.77000	1264891124
10515	Diptera	Culicidae	Aedes	Aedes aegypti	SPECIES	20.05275	-90.21787	2629264975
10516	Diptera	Culicidae	Aedes	Aedes aegypti	SPECIES	20.05692	-102.71331	2629264902
10517	Diptera	Culicidae	Aedes	Aedes aegypti	SPECIES	20.05812	-90.21579	2629265317
10518	Diptera	Culicidae	Aedes	Aedes aegypti	SPECIES	20.06000	-97.05000	1264886787
10519	Diptera	Culicidae	Aedes	Aedes aegypti	SPECIES	20.06000	-97.04000	1264888586
10520	Diptera	Culicidae	Aedes	Aedes aegypti	SPECIES	20.06056	-97.05417	2629265607
10521	Diptera	Culicidae	Aedes	Aedes aegypti	SPECIES	20.06299	-155.83740	2574144500
10522	Diptera	Culicidae	Aedes	Aedes aegypti	SPECIES	20.06889	-100.69222	1894252004
10523	Diptera	Culicidae	Aedes	Aedes aegypti	SPECIES	20.07000	-97.04000	1264887397
10524	Diptera	Culicidae	Aedes	Aedes aegypti	SPECIES	20.07000	-75.80000	1264891148
10525	Diptera	Culicidae	Aedes	Aedes aegypti	SPECIES	20.07067	-155.84478	1453408246
10526	Diptera	Culicidae	Aedes	Aedes aegypti	SPECIES	20.07087	-155.84471	1455571582
10527	Diptera	Culicidae	Aedes	Aedes aegypti	SPECIES	20.07099	-155.84442	1453449897
10528	Diptera	Culicidae	Aedes	Aedes aegypti	SPECIES	20.07117	-155.84424	1562904441
10529	Diptera	Culicidae	Aedes	Aedes aegypti	SPECIES	20.07123	-155.84446	1562960163
10530	Diptera	Culicidae	Aedes	Aedes aegypti	SPECIES	20.07139	-155.84555	1453451866
10531	Diptera	Culicidae	Aedes	Aedes aegypti	SPECIES	20.07165	-155.84385	1562928094
10532	Diptera	Culicidae	Aedes	Aedes aegypti	SPECIES	20.07293	-155.73623	1453122314
10533	Diptera	Culicidae	Aedes	Aedes aegypti	SPECIES	20.07296	-155.85787	1453078270
10534	Diptera	Culicidae	Aedes	Aedes aegypti	SPECIES	20.07312	-155.85714	2242879360
10535	Diptera	Culicidae	Aedes	Aedes aegypti	SPECIES	20.07316	-155.85756	1453452503
10536	Diptera	Culicidae	Aedes	Aedes aegypti	SPECIES	20.07317	-155.85753	1291093669
10537	Diptera	Culicidae	Aedes	Aedes aegypti	SPECIES	20.07317	-155.85755	1291095298

№	Order	Family	Genus	Species	Taxon Rank	Latitude	Longitude	GBIF_ID
10538	Diptera	Culicidae	Aedes	Aedes aegypti	SPECIES	20.07317	-155.85748	1453094326
10539	Diptera	Culicidae	Aedes	Aedes aegypti	SPECIES	20.07318	-155.85750	1453094170
10540	Diptera	Culicidae	Aedes	Aedes aegypti	SPECIES	20.07319	-155.85748	1453452414
10541	Diptera	Culicidae	Aedes	Aedes aegypti	SPECIES	20.07320	-155.85745	1453452455
10542	Diptera	Culicidae	Aedes	Aedes aegypti	SPECIES	20.07335	-155.85660	2242881044
10543	Diptera	Culicidae	Aedes	Aedes aegypti	SPECIES	20.07339	-155.85708	1562960234
10544	Diptera	Culicidae	Aedes	Aedes aegypti	SPECIES	20.07348	-155.85689	2242881607
10545	Diptera	Culicidae	Aedes	Aedes aegypti	SPECIES	20.07359	-155.85681	2465307158
10546	Diptera	Culicidae	Aedes	Aedes aegypti	SPECIES	20.07361	-155.85654	1453472237
10547	Diptera	Culicidae	Aedes	Aedes aegypti	SPECIES	20.07374	-155.85648	1562904411
10548	Diptera	Culicidae	Aedes	Aedes aegypti	SPECIES	20.07379	-155.84898	1262380482
10549	Diptera	Culicidae	Aedes	Aedes aegypti	SPECIES	20.07381	-97.03803	1421477661
10550	Diptera	Culicidae	Aedes	Aedes aegypti	SPECIES	20.07395	-155.85632	1453452477
10551	Diptera	Culicidae	Aedes	Aedes aegypti	SPECIES	20.07408	-155.85637	1453452388
10552	Diptera	Culicidae	Aedes	Aedes aegypti	SPECIES	20.07484	-155.83444	1270049445
10553	Diptera	Culicidae	Aedes	Aedes aegypti	SPECIES	20.07528	-87.60778	2629263033
10554	Diptera	Culicidae	Aedes	Aedes aegypti	SPECIES	20.07639	-87.61444	1894399903
10555	Diptera	Culicidae	Aedes	Aedes aegypti	SPECIES	20.07639	-87.61806	2629265270
10556	Diptera	Culicidae	Aedes	Aedes aegypti	SPECIES	20.07955	-155.75022	1323033369
10557	Diptera	Culicidae	Aedes	Aedes aegypti	SPECIES	20.08054	-155.83415	2445012105
10558	Diptera	Culicidae	Aedes	Aedes aegypti	SPECIES	20.09834	-155.87140	1453452355
10559	Diptera	Culicidae	Aedes	Aedes aegypti	SPECIES	20.10000	77.14000	1264889224
10560	Diptera	Culicidae	Aedes	Aedes aegypti	SPECIES	20.10167	-100.82750	1894253161
10561	Diptera	Culicidae	Aedes	Aedes aegypti	SPECIES	20.11000	-75.74000	1264891174
10562	Diptera	Culicidae	Aedes	Aedes aegypti	SPECIES	20.12000	-88.93000	1264886250
10563	Diptera	Culicidae	Aedes	Aedes aegypti	SPECIES	20.12000	105.85000	1264889052
10564	Diptera	Culicidae	Aedes	Aedes aegypti	SPECIES	20.12556	-88.92139	1894397656
10565	Diptera	Culicidae	Aedes	Aedes aegypti	SPECIES	20.12972	-101.19000	1894255922
10566	Diptera	Culicidae	Aedes	Aedes aegypti	SPECIES	20.13000	99.85000	1264886520
10567	Diptera	Culicidae	Aedes	Aedes aegypti	SPECIES	20.13889	-101.17972	1894255690
10568	Diptera	Culicidae	Aedes	Aedes aegypti	SPECIES	20.14000	94.90000	1264890123
10569	Diptera	Culicidae	Aedes	Aedes aegypti	SPECIES	20.14000	-75.21000	1264890385
10570	Diptera	Culicidae	Aedes	Aedes aegypti	SPECIES	20.14944	-100.50944	1894250545
10571	Diptera	Culicidae	Aedes	Aedes aegypti	SPECIES	20.15361	-103.18444	1894395058
10572	Diptera	Culicidae	Aedes	Aedes aegypti	SPECIES	20.15417	-88.15750	1894396136
10573	Diptera	Culicidae	Aedes	Aedes aegypti	SPECIES	20.16000	73.98000	1264890794
10574	Diptera	Culicidae	Aedes	Aedes aegypti	SPECIES	20.16667	-90.22889	2629265087
10575	Diptera	Culicidae	Aedes	Aedes aegypti	SPECIES	20.17000	85.02000	1264886629
10576	Diptera	Culicidae	Aedes	Aedes aegypti	SPECIES	20.17083	-87.99833	1894394689
10577	Diptera	Culicidae	Aedes	Aedes aegypti	SPECIES	20.17333	-100.99111	1894255844
10578	Diptera	Culicidae	Aedes	Aedes aegypti	SPECIES	20.17917	-101.18472	1894251945

№	Order	Family	Genus	Species	Taxon Rank	Latitude	Longitude	GBIF_ID
10579	Diptera	Culicidae	Aedes	Aedes aegypti	SPECIES	20.18417	-100.96000	1894249918
10580	Diptera	Culicidae	Aedes	Aedes aegypti	SPECIES	20.19000	-75.96000	1264890984
10581	Diptera	Culicidae	Aedes	Aedes aegypti	SPECIES	20.19667	-103.30500	1894397881
10582	Diptera	Culicidae	Aedes	Aedes aegypti	SPECIES	20.19861	-100.36444	1894252710
10583	Diptera	Culicidae	Aedes	Aedes aegypti	SPECIES	20.20000	-89.28000	1264886285
10584	Diptera	Culicidae	Aedes	Aedes aegypti	SPECIES	20.20000	-76.14000	1264890161
10585	Diptera	Culicidae	Aedes	Aedes aegypti	SPECIES	20.20000	76.01000	3347223017
10586	Diptera	Culicidae	Aedes	Aedes aegypti	SPECIES	20.20369	-104.04567	2629265428
10587	Diptera	Culicidae	Aedes	Aedes aegypti	SPECIES	20.20639	-101.02389	1894253866
10588	Diptera	Culicidae	Aedes	Aedes aegypti	SPECIES	20.20806	-87.46639	1894400248
10589	Diptera	Culicidae	Aedes	Aedes aegypti	SPECIES	20.21222	-101.12750	1894255888
10590	Diptera	Culicidae	Aedes	Aedes aegypti	SPECIES	20.21389	-100.87833	1894398688
10591	Diptera	Culicidae	Aedes	Aedes aegypti	SPECIES	20.21500	-100.87806	1894255894
10592	Diptera	Culicidae	Aedes	Aedes aegypti	SPECIES	20.21556	-100.84056	1894254161
10593	Diptera	Culicidae	Aedes	Aedes aegypti	SPECIES	20.22000	-74.91000	1264890851
10594	Diptera	Culicidae	Aedes	Aedes aegypti	SPECIES	20.22250	-100.73972	1894252288
10595	Diptera	Culicidae	Aedes	Aedes aegypti	SPECIES	20.22778	-103.56944	1894399410
10596	Diptera	Culicidae	Aedes	Aedes aegypti	SPECIES	20.23000	-76.21000	1264891140
10597	Diptera	Culicidae	Aedes	Aedes aegypti	SPECIES	20.24778	-100.96639	1894255673
10598	Diptera	Culicidae	Aedes	Aedes aegypti	SPECIES	20.25000	100.24000	1264887664
10599	Diptera	Culicidae	Aedes	Aedes aegypti	SPECIES	20.25000	105.83000	1264890220
10600	Diptera	Culicidae	Aedes	Aedes aegypti	SPECIES	20.25000	75.27000	3347255919
10601	Diptera	Culicidae	Aedes	Aedes aegypti	SPECIES	20.25028	-101.93167	1894251069
10602	Diptera	Culicidae	Aedes	Aedes aegypti	SPECIES	20.27722	-102.54972	1894397876
10603	Diptera	Culicidae	Aedes	Aedes aegypti	SPECIES	20.27833	-101.35806	1894254318
10604	Diptera	Culicidae	Aedes	Aedes aegypti	SPECIES	20.28015	-87.38213	2234817728
10605	Diptera	Culicidae	Aedes	Aedes aegypti	SPECIES	20.28167	-101.84500	1894253087
10606	Diptera	Culicidae	Aedes	Aedes aegypti	SPECIES	20.28389	-101.00472	1894255524
10607	Diptera	Culicidae	Aedes	Aedes aegypti	SPECIES	20.28525	-102.55575	2629263024
10608	Diptera	Culicidae	Aedes	Aedes aegypti	SPECIES	20.28550	-102.55406	2629262535
10609	Diptera	Culicidae	Aedes	Aedes aegypti	SPECIES	20.28800	-102.55744	2629264173
10610	Diptera	Culicidae	Aedes	Aedes aegypti	SPECIES	20.28861	-100.75806	1894250522
10611	Diptera	Culicidae	Aedes	Aedes aegypti	SPECIES	20.29000	-102.70944	1894397507
10612	Diptera	Culicidae	Aedes	Aedes aegypti	SPECIES	20.29000	-103.18917	1894399307
10613	Diptera	Culicidae	Aedes	Aedes aegypti	SPECIES	20.29056	-100.91694	1894249727
10614	Diptera	Culicidae	Aedes	Aedes aegypti	SPECIES	20.29611	-101.25861	1894253578
10615	Diptera	Culicidae	Aedes	Aedes aegypti	SPECIES	20.29611	-101.41861	1894254826
10616	Diptera	Culicidae	Aedes	Aedes aegypti	SPECIES	20.29986	-103.18892	2629264783
10617	Diptera	Culicidae	Aedes	Aedes aegypti	SPECIES	20.30000	106.27000	1264886900
10618	Diptera	Culicidae	Aedes	Aedes aegypti	SPECIES	20.30000	78.45000	1264888923
10619	Diptera	Culicidae	Aedes	Aedes aegypti	SPECIES	20.30278	-89.41833	1894394345

№	Order	Family	Genus	Species	Taxon Rank	Latitude	Longitude	GBIF_ID
10620	Diptera	Culicidae	Aedes	Aedes aegypti	SPECIES	20.30639	-101.01778	1894254571
10621	Diptera	Culicidae	Aedes	Aedes aegypti	SPECIES	20.31000	-89.43000	1264886314
10622	Diptera	Culicidae	Aedes	Aedes aegypti	SPECIES	20.31056	-101.18139	1894254504
10623	Diptera	Culicidae	Aedes	Aedes aegypti	SPECIES	20.31861	-90.05556	1894398375
10624	Diptera	Culicidae	Aedes	Aedes aegypti	SPECIES	20.31889	-101.44250	1894254634
10625	Diptera	Culicidae	Aedes	Aedes aegypti	SPECIES	20.33333	-88.48639	1894399373
10626	Diptera	Culicidae	Aedes	Aedes aegypti	SPECIES	20.34000	79.16000	1264889277
10627	Diptera	Culicidae	Aedes	Aedes aegypti	SPECIES	20.34056	-100.79278	1894250701
10628	Diptera	Culicidae	Aedes	Aedes aegypti	SPECIES	20.34167	-101.20889	1894255426
10629	Diptera	Culicidae	Aedes	Aedes aegypti	SPECIES	20.34583	-102.28083	1894396548
10630	Diptera	Culicidae	Aedes	Aedes aegypti	SPECIES	20.34722	-102.01583	1894255833
10631	Diptera	Culicidae	Aedes	Aedes aegypti	SPECIES	20.34833	-101.67417	1894254153
10632	Diptera	Culicidae	Aedes	Aedes aegypti	SPECIES	20.34944	-102.43000	1894395268
10633	Diptera	Culicidae	Aedes	Aedes aegypti	SPECIES	20.35083	-102.77167	1894399230
10634	Diptera	Culicidae	Aedes	Aedes aegypti	SPECIES	20.35222	-101.24528	1894253816
10635	Diptera	Culicidae	Aedes	Aedes aegypti	SPECIES	20.35472	-101.52750	1894252205
10636	Diptera	Culicidae	Aedes	Aedes aegypti	SPECIES	20.35750	-101.13722	1894253668
10637	Diptera	Culicidae	Aedes	Aedes aegypti	SPECIES	20.35750	-101.58861	1894255758
10638	Diptera	Culicidae	Aedes	Aedes aegypti	SPECIES	20.35944	-101.53861	1894255853
10639	Diptera	Culicidae	Aedes	Aedes aegypti	SPECIES	20.36056	-101.21000	1894254313
10640	Diptera	Culicidae	Aedes	Aedes aegypti	SPECIES	20.36311	-102.77106	2629263733
10641	Diptera	Culicidae	Aedes	Aedes aegypti	SPECIES	20.36378	-102.77139	2629262505
10642	Diptera	Culicidae	Aedes	Aedes aegypti	SPECIES	20.36444	-101.20833	1894253955
10643	Diptera	Culicidae	Aedes	Aedes aegypti	SPECIES	20.36528	-103.82222	1894399240
10644	Diptera	Culicidae	Aedes	Aedes aegypti	SPECIES	20.36722	-101.62806	1894255234
10645	Diptera	Culicidae	Aedes	Aedes aegypti	SPECIES	20.36750	-101.49694	1894255347
10646	Diptera	Culicidae	Aedes	Aedes aegypti	SPECIES	20.36806	-101.17333	1894253113
10647	Diptera	Culicidae	Aedes	Aedes aegypti	SPECIES	20.37000	-97.28000	1264889129
10648	Diptera	Culicidae	Aedes	Aedes aegypti	SPECIES	20.37167	-103.72694	1894397607
10649	Diptera	Culicidae	Aedes	Aedes aegypti	SPECIES	20.37333	-101.06639	1894255846
10650	Diptera	Culicidae	Aedes	Aedes aegypti	SPECIES	20.37389	-90.05222	1894399920
10651	Diptera	Culicidae	Aedes	Aedes aegypti	SPECIES	20.37444	-102.86333	1894395859
10652	Diptera	Culicidae	Aedes	Aedes aegypti	SPECIES	20.37556	-100.94583	1894252095
10653	Diptera	Culicidae	Aedes	Aedes aegypti	SPECIES	20.37722	-101.15861	1894255145
10654	Diptera	Culicidae	Aedes	Aedes aegypti	SPECIES	20.38639	-101.39667	1894251907
10655	Diptera	Culicidae	Aedes	Aedes aegypti	SPECIES	20.39139	-101.94000	1894253079
10656	Diptera	Culicidae	Aedes	Aedes aegypti	SPECIES	20.39139	-101.39528	1894255158
10657	Diptera	Culicidae	Aedes	Aedes aegypti	SPECIES	20.39175	-103.87006	2629264541
10658	Diptera	Culicidae	Aedes	Aedes aegypti	SPECIES	20.39278	-101.79333	1894251510
10659	Diptera	Culicidae	Aedes	Aedes aegypti	SPECIES	20.39278	-101.19139	1894255906
10660	Diptera	Culicidae	Aedes	Aedes aegypti	SPECIES	20.39389	-101.25472	1894252592

№	Order	Family	Genus	Species	Taxon Rank	Latitude	Longitude	GBIF_ID
10661	Diptera	Culicidae	Aedes	Aedes aegypti	SPECIES	20.39500	-100.88028	1894254939
10662	Diptera	Culicidae	Aedes	Aedes aegypti	SPECIES	20.39528	-89.53389	1894399372
10663	Diptera	Culicidae	Aedes	Aedes aegypti	SPECIES	20.39972	-101.48028	1894255950
10664	Diptera	Culicidae	Aedes	Aedes aegypti	SPECIES	20.40000	-89.55000	1264886244
10665	Diptera	Culicidae	Aedes	Aedes aegypti	SPECIES	20.40000	78.14000	1264889251
10666	Diptera	Culicidae	Aedes	Aedes aegypti	SPECIES	20.40000	78.13000	1264891396
10667	Diptera	Culicidae	Aedes	Aedes aegypti	SPECIES	20.40028	-101.70639	1894255164
10668	Diptera	Culicidae	Aedes	Aedes aegypti	SPECIES	20.40639	-101.67583	1894395671
10669	Diptera	Culicidae	Aedes	Aedes aegypti	SPECIES	20.40667	-101.67611	1894253338
10670	Diptera	Culicidae	Aedes	Aedes aegypti	SPECIES	20.40694	-103.76917	1894400124
10671	Diptera	Culicidae	Aedes	Aedes aegypti	SPECIES	20.40943	-102.92878	3407894827
10672	Diptera	Culicidae	Aedes	Aedes aegypti	SPECIES	20.41556	-101.99083	1894255502
10673	Diptera	Culicidae	Aedes	Aedes aegypti	SPECIES	20.41639	-101.96083	1894254270
10674	Diptera	Culicidae	Aedes	Aedes aegypti	SPECIES	20.42020	-98.08316	1894400045
10675	Diptera	Culicidae	Aedes	Aedes aegypti	SPECIES	20.42056	-103.59111	1894396522
10676	Diptera	Culicidae	Aedes	Aedes aegypti	SPECIES	20.42167	-101.25056	1894254043
10677	Diptera	Culicidae	Aedes	Aedes aegypti	SPECIES	20.42417	-101.26417	1894254638
10678	Diptera	Culicidae	Aedes	Aedes aegypti	SPECIES	20.42558	-98.08203	1894396927
10679	Diptera	Culicidae	Aedes	Aedes aegypti	SPECIES	20.42944	-101.49722	1894253877
10680	Diptera	Culicidae	Aedes	Aedes aegypti	SPECIES	20.43111	-101.72222	1894255883
10681	Diptera	Culicidae	Aedes	Aedes aegypti	SPECIES	20.43306	-100.57889	1894254162
10682	Diptera	Culicidae	Aedes	Aedes aegypti	SPECIES	20.43639	-101.38056	1894254722
10683	Diptera	Culicidae	Aedes	Aedes aegypti	SPECIES	20.43833	-101.31056	1894251633
10684	Diptera	Culicidae	Aedes	Aedes aegypti	SPECIES	20.44111	-101.20639	1894252082
10685	Diptera	Culicidae	Aedes	Aedes aegypti	SPECIES	20.44167	-102.39306	1894397931
10686	Diptera	Culicidae	Aedes	Aedes aegypti	SPECIES	20.44778	-101.16139	1894249660
10687	Diptera	Culicidae	Aedes	Aedes aegypti	SPECIES	20.44917	-97.08389	1894399419
10688	Diptera	Culicidae	Aedes	Aedes aegypti	SPECIES	20.45028	-100.66861	1894252302
10689	Diptera	Culicidae	Aedes	Aedes aegypti	SPECIES	20.45111	-101.52889	1894255818
10690	Diptera	Culicidae	Aedes	Aedes aegypti	SPECIES	20.45250	-101.77722	1894251661
10691	Diptera	Culicidae	Aedes	Aedes aegypti	SPECIES	20.45333	-101.55667	1894249725
10692	Diptera	Culicidae	Aedes	Aedes aegypti	SPECIES	20.45556	-100.62167	1894255457
10693	Diptera	Culicidae	Aedes	Aedes aegypti	SPECIES	20.45583	-103.16583	1894399932
10694	Diptera	Culicidae	Aedes	Aedes aegypti	SPECIES	20.46000	-89.22000	1264886245
10695	Diptera	Culicidae	Aedes	Aedes aegypti	SPECIES	20.46000	99.91000	1264890131
10696	Diptera	Culicidae	Aedes	Aedes aegypti	SPECIES	20.46000	-98.07611	1894399174
10697	Diptera	Culicidae	Aedes	Aedes aegypti	SPECIES	20.46028	-101.50306	1894255371
10698	Diptera	Culicidae	Aedes	Aedes aegypti	SPECIES	20.46222	-101.03889	1894255285
10699	Diptera	Culicidae	Aedes	Aedes aegypti	SPECIES	20.46639	-101.14361	1894254189
10700	Diptera	Culicidae	Aedes	Aedes aegypti	SPECIES	20.46944	-99.21417	2397606354
10701	Diptera	Culicidae	Aedes	Aedes aegypti	SPECIES	20.47000	-89.08000	1264886232

№	Order	Family	Genus	Species	Taxon Rank	Latitude	Longitude	GBIF_ID
10702	Diptera	Culicidae	Aedes	Aedes aegypti	SPECIES	20.47000	-101.61639	1894253317
10703	Diptera	Culicidae	Aedes	Aedes aegypti	SPECIES	20.47389	-101.40778	1894254607
10704	Diptera	Culicidae	Aedes	Aedes aegypti	SPECIES	20.47750	-101.33583	1894255712
10705	Diptera	Culicidae	Aedes	Aedes aegypti	SPECIES	20.47750	-101.27944	1894255726
10706	Diptera	Culicidae	Aedes	Aedes aegypti	SPECIES	20.48058	-99.21724	2397530399
10707	Diptera	Culicidae	Aedes	Aedes aegypti	SPECIES	20.48145	-99.20850	2283076633
10708	Diptera	Culicidae	Aedes	Aedes aegypti	SPECIES	20.48278	-100.96111	1894255767
10709	Diptera	Culicidae	Aedes	Aedes aegypti	SPECIES	20.48389	-101.16306	1894255442
10710	Diptera	Culicidae	Aedes	Aedes aegypti	SPECIES	20.48611	-102.34917	1894395846
10711	Diptera	Culicidae	Aedes	Aedes aegypti	SPECIES	20.49583	-97.52417	1894400241
10712	Diptera	Culicidae	Aedes	Aedes aegypti	SPECIES	20.49667	-103.95417	1894394834
10713	Diptera	Culicidae	Aedes	Aedes aegypti	SPECIES	20.49944	-98.67806	1894400177
10714	Diptera	Culicidae	Aedes	Aedes aegypti	SPECIES	20.50111	-101.54806	1894253959
10715	Diptera	Culicidae	Aedes	Aedes aegypti	SPECIES	20.50165	-100.89375	4162588817
10716	Diptera	Culicidae	Aedes	Aedes aegypti	SPECIES	20.50500	-101.39250	1894254643
10717	Diptera	Culicidae	Aedes	Aedes aegypti	SPECIES	20.50583	-101.17750	1894250549
10718	Diptera	Culicidae	Aedes	Aedes aegypti	SPECIES	20.50684	-100.86210	4161981822
10719	Diptera	Culicidae	Aedes	Aedes aegypti	SPECIES	20.50750	-101.20611	1894253312
10720	Diptera	Culicidae	Aedes	Aedes aegypti	SPECIES	20.50806	-100.63167	1894254519
10721	Diptera	Culicidae	Aedes	Aedes aegypti	SPECIES	20.50972	-90.24167	2629263976
10722	Diptera	Culicidae	Aedes	Aedes aegypti	SPECIES	20.51028	-103.85694	1894396371
10723	Diptera	Culicidae	Aedes	Aedes aegypti	SPECIES	20.51222	-100.54167	1894255663
10724	Diptera	Culicidae	Aedes	Aedes aegypti	SPECIES	20.51417	-97.49611	1894399065
10725	Diptera	Culicidae	Aedes	Aedes aegypti	SPECIES	20.51446	-87.23483	3070296625
10726	Diptera	Culicidae	Aedes	Aedes aegypti	SPECIES	20.51667	-86.94167	1421477194
10727	Diptera	Culicidae	Aedes	Aedes aegypti	SPECIES	20.51889	-103.17833	1894399921
10728	Diptera	Culicidae	Aedes	Aedes aegypti	SPECIES	20.52056	-100.43361	1894394595
10729	Diptera	Culicidae	Aedes	Aedes aegypti	SPECIES	20.52222	-100.91972	1894253000
10730	Diptera	Culicidae	Aedes	Aedes aegypti	SPECIES	20.52250	-101.30222	1894252994
10731	Diptera	Culicidae	Aedes	Aedes aegypti	SPECIES	20.52694	-101.37333	1894255323
10732	Diptera	Culicidae	Aedes	Aedes aegypti	SPECIES	20.52889	-100.81500	1894255845
10733	Diptera	Culicidae	Aedes	Aedes aegypti	SPECIES	20.53000	-97.45000	1264886696
10734	Diptera	Culicidae	Aedes	Aedes aegypti	SPECIES	20.53000	-97.46000	1264886795
10735	Diptera	Culicidae	Aedes	Aedes aegypti	SPECIES	20.53000	76.18000	1264889284
10736	Diptera	Culicidae	Aedes	Aedes aegypti	SPECIES	20.53028	-100.67139	1894254357
10737	Diptera	Culicidae	Aedes	Aedes aegypti	SPECIES	20.53111	-101.07083	1894254906
10738	Diptera	Culicidae	Aedes	Aedes aegypti	SPECIES	20.53500	-97.45389	2629264916
10739	Diptera	Culicidae	Aedes	Aedes aegypti	SPECIES	20.53661	-100.83172	4162572628
10740	Diptera	Culicidae	Aedes	Aedes aegypti	SPECIES	20.53722	-103.93750	1894399725
10741	Diptera	Culicidae	Aedes	Aedes aegypti	SPECIES	20.53917	-102.51147	2629263724
10742	Diptera	Culicidae	Aedes	Aedes aegypti	SPECIES	20.54000	-97.47000	1264887060

№	Order	Family	Genus	Species	Taxon Rank	Latitude	Longitude	GBIF_ID
10743	Diptera	Culicidae	Aedes	Aedes aegypti	SPECIES	20.54111	-101.43500	1894253536
10744	Diptera	Culicidae	Aedes	Aedes aegypti	SPECIES	20.54389	-101.45417	1894251916
10745	Diptera	Culicidae	Aedes	Aedes aegypti	SPECIES	20.54667	-101.62750	1894255496
10746	Diptera	Culicidae	Aedes	Aedes aegypti	SPECIES	20.54667	-103.33806	1894398083
10747	Diptera	Culicidae	Aedes	Aedes aegypti	SPECIES	20.54778	-104.04722	1894400014
10748	Diptera	Culicidae	Aedes	Aedes aegypti	SPECIES	20.55000	-97.47000	1264888553
10749	Diptera	Culicidae	Aedes	Aedes aegypti	SPECIES	20.55000	74.53000	1264890537
10750	Diptera	Culicidae	Aedes	Aedes aegypti	SPECIES	20.55083	-101.37333	1894254753
10751	Diptera	Culicidae	Aedes	Aedes aegypti	SPECIES	20.55333	-100.51722	1894254204
10752	Diptera	Culicidae	Aedes	Aedes aegypti	SPECIES	20.55333	-101.18111	1894255370
10753	Diptera	Culicidae	Aedes	Aedes aegypti	SPECIES	20.55444	-101.52556	1894255676
10754	Diptera	Culicidae	Aedes	Aedes aegypti	SPECIES	20.55831	-104.04922	2629263105
10755	Diptera	Culicidae	Aedes	Aedes aegypti	SPECIES	20.56000	78.84000	1264889275
10756	Diptera	Culicidae	Aedes	Aedes aegypti	SPECIES	20.56222	-103.95389	1894400120
10757	Diptera	Culicidae	Aedes	Aedes aegypti	SPECIES	20.56556	-101.41750	1894249823
10758	Diptera	Culicidae	Aedes	Aedes aegypti	SPECIES	20.56621	-100.92191	4162362156
10759	Diptera	Culicidae	Aedes	Aedes aegypti	SPECIES	20.56639	-101.59139	1894254588
10760	Diptera	Culicidae	Aedes	Aedes aegypti	SPECIES	20.56639	-101.38167	1894255037
10761	Diptera	Culicidae	Aedes	Aedes aegypti	SPECIES	20.56917	-103.15306	1894399473
10762	Diptera	Culicidae	Aedes	Aedes aegypti	SPECIES	20.56917	-101.19750	1894399549
10763	Diptera	Culicidae	Aedes	Aedes aegypti	SPECIES	20.56933	-105.14786	2629262827
10764	Diptera	Culicidae	Aedes	Aedes aegypti	SPECIES	20.57000	105.85000	1264886658
10765	Diptera	Culicidae	Aedes	Aedes aegypti	SPECIES	20.57000	-97.43000	1264887415
10766	Diptera	Culicidae	Aedes	Aedes aegypti	SPECIES	20.57000	-103.24000	1264890979
10767	Diptera	Culicidae	Aedes	Aedes aegypti	SPECIES	20.57028	-101.19722	1894255850
10768	Diptera	Culicidae	Aedes	Aedes aegypti	SPECIES	20.57086	-103.48481	4052761770
10769	Diptera	Culicidae	Aedes	Aedes aegypti	SPECIES	20.57361	-98.03056	1894397863
10770	Diptera	Culicidae	Aedes	Aedes aegypti	SPECIES	20.57394	-103.85339	2629263618
10771	Diptera	Culicidae	Aedes	Aedes aegypti	SPECIES	20.57614	-97.43064	1421474089
10772	Diptera	Culicidae	Aedes	Aedes aegypti	SPECIES	20.57722	-101.15806	1894255794
10773	Diptera	Culicidae	Aedes	Aedes aegypti	SPECIES	20.58000	86.66000	1264886662
10774	Diptera	Culicidae	Aedes	Aedes aegypti	SPECIES	20.58000	76.83000	1264888934
10775	Diptera	Culicidae	Aedes	Aedes aegypti	SPECIES	20.58167	-100.49500	1894250638
10776	Diptera	Culicidae	Aedes	Aedes aegypti	SPECIES	20.58192	-100.37607	2813896545
10777	Diptera	Culicidae	Aedes	Aedes aegypti	SPECIES	20.58306	-90.00083	1894399186
10778	Diptera	Culicidae	Aedes	Aedes aegypti	SPECIES	20.58500	-101.43583	1894254492
10779	Diptera	Culicidae	Aedes	Aedes aegypti	SPECIES	20.58750	-100.39278	1894399595
10780	Diptera	Culicidae	Aedes	Aedes aegypti	SPECIES	20.58839	-97.49411	1421477581
10781	Diptera	Culicidae	Aedes	Aedes aegypti	SPECIES	20.58926	-100.38989	3031669926
10782	Diptera	Culicidae	Aedes	Aedes aegypti	SPECIES	20.59000	-90.00000	1264886260
10783	Diptera	Culicidae	Aedes	Aedes aegypti	SPECIES	20.59306	-101.12667	1894255822

№	Order	Family	Genus	Species	Taxon Rank	Latitude	Longitude	GBIF_ID
10784	Diptera	Culicidae	Aedes	Aedes aegypti	SPECIES	20.59583	-89.00611	1894398174
10785	Diptera	Culicidae	Aedes	Aedes aegypti	SPECIES	20.59611	-98.76306	1894399656
10786	Diptera	Culicidae	Aedes	Aedes aegypti	SPECIES	20.59694	-98.06833	1894399713
10787	Diptera	Culicidae	Aedes	Aedes aegypti	SPECIES	20.60000	-105.24000	1264888065
10788	Diptera	Culicidae	Aedes	Aedes aegypti	SPECIES	20.60111	-101.08194	1894249378
10789	Diptera	Culicidae	Aedes	Aedes aegypti	SPECIES	20.60278	-98.05000	1894397374
10790	Diptera	Culicidae	Aedes	Aedes aegypti	SPECIES	20.60278	-98.78389	1894398966
10791	Diptera	Culicidae	Aedes	Aedes aegypti	SPECIES	20.60500	-101.62333	1894254776
10792	Diptera	Culicidae	Aedes	Aedes aegypti	SPECIES	20.60500	-103.72056	1894398743
10793	Diptera	Culicidae	Aedes	Aedes aegypti	SPECIES	20.60667	-100.33000	1894397412
10794	Diptera	Culicidae	Aedes	Aedes aegypti	SPECIES	20.60778	-100.94667	1894255447
10795	Diptera	Culicidae	Aedes	Aedes aegypti	SPECIES	20.60917	-100.44528	1894399052
10796	Diptera	Culicidae	Aedes	Aedes aegypti	SPECIES	20.61333	-105.23111	2629262492
10797	Diptera	Culicidae	Aedes	Aedes aegypti	SPECIES	20.61778	-101.06250	1894254361
10798	Diptera	Culicidae	Aedes	Aedes aegypti	SPECIES	20.61944	-100.89500	1894255495
10799	Diptera	Culicidae	Aedes	Aedes aegypti	SPECIES	20.62222	-103.06833	1894398207
10800	Diptera	Culicidae	Aedes	Aedes aegypti	SPECIES	20.62361	-103.24111	1894400076
10801	Diptera	Culicidae	Aedes	Aedes aegypti	SPECIES	20.62389	-87.07667	1421476061
10802	Diptera	Culicidae	Aedes	Aedes aegypti	SPECIES	20.62583	-101.67389	1894255755
10803	Diptera	Culicidae	Aedes	Aedes aegypti	SPECIES	20.62750	-87.08111	2629264106
10804	Diptera	Culicidae	Aedes	Aedes aegypti	SPECIES	20.63000	86.13000	1264886160
10805	Diptera	Culicidae	Aedes	Aedes aegypti	SPECIES	20.63083	-88.21722	1894397405
10806	Diptera	Culicidae	Aedes	Aedes aegypti	SPECIES	20.63139	-101.04944	1894251790
10807	Diptera	Culicidae	Aedes	Aedes aegypti	SPECIES	20.63764	-100.47569	2864754069
10808	Diptera	Culicidae	Aedes	Aedes aegypti	SPECIES	20.63768	-100.47571	2864769835
10809	Diptera	Culicidae	Aedes	Aedes aegypti	SPECIES	20.63769	-100.47551	2850758897
10810	Diptera	Culicidae	Aedes	Aedes aegypti	SPECIES	20.63833	-103.31056	1894400153
10811	Diptera	Culicidae	Aedes	Aedes aegypti	SPECIES	20.63972	-98.92111	1894396189
10812	Diptera	Culicidae	Aedes	Aedes aegypti	SPECIES	20.64111	-98.82639	1894397819
10813	Diptera	Culicidae	Aedes	Aedes aegypti	SPECIES	20.64333	-100.99500	1894255718
10814	Diptera	Culicidae	Aedes	Aedes aegypti	SPECIES	20.64361	-90.21250	2629265372
10815	Diptera	Culicidae	Aedes	Aedes aegypti	SPECIES	20.64389	-100.96194	1894254956
10816	Diptera	Culicidae	Aedes	Aedes aegypti	SPECIES	20.64583	-89.46417	1894399057
10817	Diptera	Culicidae	Aedes	Aedes aegypti	SPECIES	20.64639	-98.80667	1894399232
10818	Diptera	Culicidae	Aedes	Aedes aegypti	SPECIES	20.64833	-101.51917	1894253744
10819	Diptera	Culicidae	Aedes	Aedes aegypti	SPECIES	20.64833	-103.70111	1894397196
10820	Diptera	Culicidae	Aedes	Aedes aegypti	SPECIES	20.65000	-105.23000	1264886758
10821	Diptera	Culicidae	Aedes	Aedes aegypti	SPECIES	20.65083	-101.52778	1894254825
10822	Diptera	Culicidae	Aedes	Aedes aegypti	SPECIES	20.65250	-103.91028	1894394503
10823	Diptera	Culicidae	Aedes	Aedes aegypti	SPECIES	20.65312	-103.37594	3496614692
10824	Diptera	Culicidae	Aedes	Aedes aegypti	SPECIES	20.65361	-101.04389	1894254044

№	Order	Family	Genus	Species	Taxon Rank	Latitude	Longitude	GBIF_ID
10825	Diptera	Culicidae	Aedes	Aedes aegypti	SPECIES	20.65472	-87.93639	1894400227
10826	Diptera	Culicidae	Aedes	Aedes aegypti	SPECIES	20.65750	-101.47583	1894254111
10827	Diptera	Culicidae	Aedes	Aedes aegypti	SPECIES	20.65917	-101.39750	1894254719
10828	Diptera	Culicidae	Aedes	Aedes aegypti	SPECIES	20.65944	-101.55528	1894253247
10829	Diptera	Culicidae	Aedes	Aedes aegypti	SPECIES	20.66000	-87.94000	1264886222
10830	Diptera	Culicidae	Aedes	Aedes aegypti	SPECIES	20.66083	-100.88417	1894254962
10831	Diptera	Culicidae	Aedes	Aedes aegypti	SPECIES	20.66306	-101.68111	1894255776
10832	Diptera	Culicidae	Aedes	Aedes aegypti	SPECIES	20.66306	-102.84333	1894398585
10833	Diptera	Culicidae	Aedes	Aedes aegypti	SPECIES	20.66361	-101.29639	1894252933
10834	Diptera	Culicidae	Aedes	Aedes aegypti	SPECIES	20.66389	-100.41528	1894397121
10835	Diptera	Culicidae	Aedes	Aedes aegypti	SPECIES	20.66944	-101.84972	1894254529
10836	Diptera	Culicidae	Aedes	Aedes aegypti	SPECIES	20.67056	-100.60917	1894249763
10837	Diptera	Culicidae	Aedes	Aedes aegypti	SPECIES	20.67073	-87.04507	2963883435
10838	Diptera	Culicidae	Aedes	Aedes aegypti	SPECIES	20.67083	-103.73750	1894396725
10839	Diptera	Culicidae	Aedes	Aedes aegypti	SPECIES	20.67250	-100.74972	1894251703
10840	Diptera	Culicidae	Aedes	Aedes aegypti	SPECIES	20.67306	-101.34778	1894398103
10841	Diptera	Culicidae	Aedes	Aedes aegypti	SPECIES	20.67341	-87.04469	2626133699
10842	Diptera	Culicidae	Aedes	Aedes aegypti	SPECIES	20.67356	-87.04459	3005193607
10843	Diptera	Culicidae	Aedes	Aedes aegypti	SPECIES	20.67417	-101.34750	1894255988
10844	Diptera	Culicidae	Aedes	Aedes aegypti	SPECIES	20.67556	-101.68444	1894252816
10845	Diptera	Culicidae	Aedes	Aedes aegypti	SPECIES	20.67639	-103.34611	1894400217
10846	Diptera	Culicidae	Aedes	Aedes aegypti	SPECIES	20.67917	-98.77778	1894394928
10847	Diptera	Culicidae	Aedes	Aedes aegypti	SPECIES	20.68333	-103.84778	1894394854
10848	Diptera	Culicidae	Aedes	Aedes aegypti	SPECIES	20.68367	-103.39182	1132411710
10849	Diptera	Culicidae	Aedes	Aedes aegypti	SPECIES	20.69000	-88.22000	1264886266
10850	Diptera	Culicidae	Aedes	Aedes aegypti	SPECIES	20.69056	-101.11917	1894255498
10851	Diptera	Culicidae	Aedes	Aedes aegypti	SPECIES	20.69056	-88.20139	1894399874
10852	Diptera	Culicidae	Aedes	Aedes aegypti	SPECIES	20.69139	-101.25972	1894252972
10853	Diptera	Culicidae	Aedes	Aedes aegypti	SPECIES	20.69259	-105.22381	4080722343
10854	Diptera	Culicidae	Aedes	Aedes aegypti	SPECIES	20.69299	-103.41717	3327750523
10855	Diptera	Culicidae	Aedes	Aedes aegypti	SPECIES	20.69319	-100.48474	3031657967
10856	Diptera	Culicidae	Aedes	Aedes aegypti	SPECIES	20.69367	-90.45333	2629264445
10857	Diptera	Culicidae	Aedes	Aedes aegypti	SPECIES	20.69401	-101.36890	2963811978
10858	Diptera	Culicidae	Aedes	Aedes aegypti	SPECIES	20.69806	-88.58861	1894399258
10859	Diptera	Culicidae	Aedes	Aedes aegypti	SPECIES	20.69958	-103.36738	2963774854
10860	Diptera	Culicidae	Aedes	Aedes aegypti	SPECIES	20.69959	-100.44351	2826204730
10861	Diptera	Culicidae	Aedes	Aedes aegypti	SPECIES	20.69972	-101.58722	1894255805
10862	Diptera	Culicidae	Aedes	Aedes aegypti	SPECIES	20.70000	76.57000	1264889263
10863	Diptera	Culicidae	Aedes	Aedes aegypti	SPECIES	20.70000	77.00000	1264889264
10864	Diptera	Culicidae	Aedes	Aedes aegypti	SPECIES	20.70000	-105.24722	1894400193
10865	Diptera	Culicidae	Aedes	Aedes aegypti	SPECIES	20.70028	-103.97611	1894395692

№	Order	Family	Genus	Species	Taxon Rank	Latitude	Longitude	GBIF_ID
10866	Diptera	Culicidae	Aedes	Aedes aegypti	SPECIES	20.70358	-103.98353	2629265220
10867	Diptera	Culicidae	Aedes	Aedes aegypti	SPECIES	20.70500	-102.34389	1894397303
10868	Diptera	Culicidae	Aedes	Aedes aegypti	SPECIES	20.71000	106.79000	1264889030
10869	Diptera	Culicidae	Aedes	Aedes aegypti	SPECIES	20.71028	-103.14889	1894394342
10870	Diptera	Culicidae	Aedes	Aedes aegypti	SPECIES	20.71222	-101.43722	1894255867
10871	Diptera	Culicidae	Aedes	Aedes aegypti	SPECIES	20.71417	-105.20444	1894400086
10872	Diptera	Culicidae	Aedes	Aedes aegypti	SPECIES	20.71500	-100.75667	1894254495
10873	Diptera	Culicidae	Aedes	Aedes aegypti	SPECIES	20.71611	-103.63889	1894395417
10874	Diptera	Culicidae	Aedes	Aedes aegypti	SPECIES	20.71816	-103.52686	2963856478
10875	Diptera	Culicidae	Aedes	Aedes aegypti	SPECIES	20.71944	-101.62250	1894253038
10876	Diptera	Culicidae	Aedes	Aedes aegypti	SPECIES	20.71944	-101.26139	1894253611
10877	Diptera	Culicidae	Aedes	Aedes aegypti	SPECIES	20.72028	-103.39194	1894400214
10878	Diptera	Culicidae	Aedes	Aedes aegypti	SPECIES	20.72278	-103.28056	1894398779
10879	Diptera	Culicidae	Aedes	Aedes aegypti	SPECIES	20.72359	-103.39743	2898299209
10880	Diptera	Culicidae	Aedes	Aedes aegypti	SPECIES	20.72472	-101.37194	1894249702
10881	Diptera	Culicidae	Aedes	Aedes aegypti	SPECIES	20.72472	-101.24694	1894255907
10882	Diptera	Culicidae	Aedes	Aedes aegypti	SPECIES	20.72833	-101.34417	1894254343
10883	Diptera	Culicidae	Aedes	Aedes aegypti	SPECIES	20.72833	-101.94917	1894255741
10884	Diptera	Culicidae	Aedes	Aedes aegypti	SPECIES	20.72861	-101.64694	1894250543
10885	Diptera	Culicidae	Aedes	Aedes aegypti	SPECIES	20.72861	-105.15444	1894394751
10886	Diptera	Culicidae	Aedes	Aedes aegypti	SPECIES	20.73000	-89.17000	1264886253
10887	Diptera	Culicidae	Aedes	Aedes aegypti	SPECIES	20.73000	78.32000	1264889258
10888	Diptera	Culicidae	Aedes	Aedes aegypti	SPECIES	20.73000	95.45000	1264890159
10889	Diptera	Culicidae	Aedes	Aedes aegypti	SPECIES	20.73000	-77.50000	1264890902
10890	Diptera	Culicidae	Aedes	Aedes aegypti	SPECIES	20.73000	78.48000	1264891380
10891	Diptera	Culicidae	Aedes	Aedes aegypti	SPECIES	20.73000	-101.71222	1894252906
10892	Diptera	Culicidae	Aedes	Aedes aegypti	SPECIES	20.73222	-105.14972	1894398980
10893	Diptera	Culicidae	Aedes	Aedes aegypti	SPECIES	20.73611	-97.54889	1894399327
10894	Diptera	Culicidae	Aedes	Aedes aegypti	SPECIES	20.73694	-99.38194	1894394295
10895	Diptera	Culicidae	Aedes	Aedes aegypti	SPECIES	20.73778	-101.88250	1894255594
10896	Diptera	Culicidae	Aedes	Aedes aegypti	SPECIES	20.74194	-89.47444	1894395382
10897	Diptera	Culicidae	Aedes	Aedes aegypti	SPECIES	20.74222	-105.18000	1894395932
10898	Diptera	Culicidae	Aedes	Aedes aegypti	SPECIES	20.74972	-101.60750	1894255467
10899	Diptera	Culicidae	Aedes	Aedes aegypti	SPECIES	20.75000	77.12000	1264888929
10900	Diptera	Culicidae	Aedes	Aedes aegypti	SPECIES	20.75000	78.60000	1264889262
10901	Diptera	Culicidae	Aedes	Aedes aegypti	SPECIES	20.75000	77.11000	1264891416
10902	Diptera	Culicidae	Aedes	Aedes aegypti	SPECIES	20.75083	-100.50583	1894394851
10903	Diptera	Culicidae	Aedes	Aedes aegypti	SPECIES	20.75583	-101.40250	1894254880
10904	Diptera	Culicidae	Aedes	Aedes aegypti	SPECIES	20.75833	-105.09917	1894394810
10905	Diptera	Culicidae	Aedes	Aedes aegypti	SPECIES	20.75861	-101.51861	1894252111
10906	Diptera	Culicidae	Aedes	Aedes aegypti	SPECIES	20.76416	-103.43370	1950036567

№	Order	Family	Genus	Species	Taxon Rank	Latitude	Longitude	GBIF_ID
10907	Diptera	Culicidae	Aedes	Aedes aegypti	SPECIES	20.76944	-105.15500	1894398852
10908	Diptera	Culicidae	Aedes	Aedes aegypti	SPECIES	20.77000	-89.21000	1264886228
10909	Diptera	Culicidae	Aedes	Aedes aegypti	SPECIES	20.77000	-88.39000	1264886272
10910	Diptera	Culicidae	Aedes	Aedes aegypti	SPECIES	20.77556	-103.69333	1894395974
10911	Diptera	Culicidae	Aedes	Aedes aegypti	SPECIES	20.77611	-101.49472	1894251532
10912	Diptera	Culicidae	Aedes	Aedes aegypti	SPECIES	20.77833	-101.49639	1894254750
10913	Diptera	Culicidae	Aedes	Aedes aegypti	SPECIES	20.77861	-100.80889	1894250571
10914	Diptera	Culicidae	Aedes	Aedes aegypti	SPECIES	20.77861	-105.15417	1894395933
10915	Diptera	Culicidae	Aedes	Aedes aegypti	SPECIES	20.77889	-101.39500	1894253740
10916	Diptera	Culicidae	Aedes	Aedes aegypti	SPECIES	20.77917	-102.90944	1894399852
10917	Diptera	Culicidae	Aedes	Aedes aegypti	SPECIES	20.78000	107.03000	1264886702
10918	Diptera	Culicidae	Aedes	Aedes aegypti	SPECIES	20.78000	97.04000	1264890115
10919	Diptera	Culicidae	Aedes	Aedes aegypti	SPECIES	20.78556	-101.16694	1894252018
10920	Diptera	Culicidae	Aedes	Aedes aegypti	SPECIES	20.78917	-103.46361	1894395771
10921	Diptera	Culicidae	Aedes	Aedes aegypti	SPECIES	20.79111	-89.18139	1894399875
10922	Diptera	Culicidae	Aedes	Aedes aegypti	SPECIES	20.79694	-103.47944	1894396633
10923	Diptera	Culicidae	Aedes	Aedes aegypti	SPECIES	20.80000	-98.99833	1894395983
10924	Diptera	Culicidae	Aedes	Aedes aegypti	SPECIES	20.80253	-102.75897	2629262350
10925	Diptera	Culicidae	Aedes	Aedes aegypti	SPECIES	20.80778	-104.40667	1894399544
10926	Diptera	Culicidae	Aedes	Aedes aegypti	SPECIES	20.81250	-99.00500	1894394246
10927	Diptera	Culicidae	Aedes	Aedes aegypti	SPECIES	20.81667	-102.76111	1894400218
10928	Diptera	Culicidae	Aedes	Aedes aegypti	SPECIES	20.81722	-101.63972	1894254854
10929	Diptera	Culicidae	Aedes	Aedes aegypti	SPECIES	20.81750	-101.49111	1894255745
10930	Diptera	Culicidae	Aedes	Aedes aegypti	SPECIES	20.81917	-88.24028	1894400242
10931	Diptera	Culicidae	Aedes	Aedes aegypti	SPECIES	20.82333	-98.26167	1894399332
10932	Diptera	Culicidae	Aedes	Aedes aegypti	SPECIES	20.82361	-105.09944	1894400259
10933	Diptera	Culicidae	Aedes	Aedes aegypti	SPECIES	20.82417	-89.66111	2629264033
10934	Diptera	Culicidae	Aedes	Aedes aegypti	SPECIES	20.82917	-101.90556	1894249657
10935	Diptera	Culicidae	Aedes	Aedes aegypti	SPECIES	20.83000	-89.19000	1264886259
10936	Diptera	Culicidae	Aedes	Aedes aegypti	SPECIES	20.83333	-103.72750	1894399277
10937	Diptera	Culicidae	Aedes	Aedes aegypti	SPECIES	20.83389	-98.28528	1894399540
10938	Diptera	Culicidae	Aedes	Aedes aegypti	SPECIES	20.83500	-101.46944	1894253273
10939	Diptera	Culicidae	Aedes	Aedes aegypti	SPECIES	20.83750	-99.09528	1894398621
10940	Diptera	Culicidae	Aedes	Aedes aegypti	SPECIES	20.83972	-101.32694	1894254725
10941	Diptera	Culicidae	Aedes	Aedes aegypti	SPECIES	20.84083	-89.47639	1894399977
10942	Diptera	Culicidae	Aedes	Aedes aegypti	SPECIES	20.84333	-99.08444	1894399672
10943	Diptera	Culicidae	Aedes	Aedes aegypti	SPECIES	20.84444	-101.26944	1894251302
10944	Diptera	Culicidae	Aedes	Aedes aegypti	SPECIES	20.84444	-103.55556	1894399040
10945	Diptera	Culicidae	Aedes	Aedes aegypti	SPECIES	20.85000	106.66000	1264889007
10946	Diptera	Culicidae	Aedes	Aedes aegypti	SPECIES	20.85361	-101.59528	1894250371
10947	Diptera	Culicidae	Aedes	Aedes aegypti	SPECIES	20.85444	-99.04639	1894399221

№	Order	Family	Genus	Species	Taxon Rank	Latitude	Longitude	GBIF_ID
10948	Diptera	Culicidae	Aedes	Aedes aegypti	SPECIES	20.85450	-90.39983	2629263862
10949	Diptera	Culicidae	Aedes	Aedes aegypti	SPECIES	20.85639	-101.31083	1894255963
10950	Diptera	Culicidae	Aedes	Aedes aegypti	SPECIES	20.85686	-90.37656	2629264487
10951	Diptera	Culicidae	Aedes	Aedes aegypti	SPECIES	20.85703	-98.44493	2611031392
10952	Diptera	Culicidae	Aedes	Aedes aegypti	SPECIES	20.85806	-101.31750	1894255236
10953	Diptera	Culicidae	Aedes	Aedes aegypti	SPECIES	20.85833	-87.66806	1894399915
10954	Diptera	Culicidae	Aedes	Aedes aegypti	SPECIES	20.85917	-90.40000	1894397058
10955	Diptera	Culicidae	Aedes	Aedes aegypti	SPECIES	20.86000	-90.40000	1264887820
10956	Diptera	Culicidae	Aedes	Aedes aegypti	SPECIES	20.86000	106.69000	1264889028
10957	Diptera	Culicidae	Aedes	Aedes aegypti	SPECIES	20.86000	77.73000	1264889254
10958	Diptera	Culicidae	Aedes	Aedes aegypti	SPECIES	20.86000	-90.39000	1264890480
10959	Diptera	Culicidae	Aedes	Aedes aegypti	SPECIES	20.86056	-98.45472	1894396666
10960	Diptera	Culicidae	Aedes	Aedes aegypti	SPECIES	20.86417	-101.29500	1894253157
10961	Diptera	Culicidae	Aedes	Aedes aegypti	SPECIES	20.86472	-89.57833	1894399580
10962	Diptera	Culicidae	Aedes	Aedes aegypti	SPECIES	20.86500	-89.65306	1894396792
10963	Diptera	Culicidae	Aedes	Aedes aegypti	SPECIES	20.86583	-89.65333	2629263837
10964	Diptera	Culicidae	Aedes	Aedes aegypti	SPECIES	20.86861	-100.70417	1894254341
10965	Diptera	Culicidae	Aedes	Aedes aegypti	SPECIES	20.86972	-88.98833	1894399880
10966	Diptera	Culicidae	Aedes	Aedes aegypti	SPECIES	20.87000	-89.21000	1264886246
10967	Diptera	Culicidae	Aedes	Aedes aegypti	SPECIES	20.87000	-88.99000	1264886270
10968	Diptera	Culicidae	Aedes	Aedes aegypti	SPECIES	20.87000	-89.65000	1264886818
10969	Diptera	Culicidae	Aedes	Aedes aegypti	SPECIES	20.87000	106.70000	1264889072
10970	Diptera	Culicidae	Aedes	Aedes aegypti	SPECIES	20.87028	-101.51667	1894255807
10971	Diptera	Culicidae	Aedes	Aedes aegypti	SPECIES	20.87306	-99.44389	1894395260
10972	Diptera	Culicidae	Aedes	Aedes aegypti	SPECIES	20.87417	-89.25611	1894399103
10973	Diptera	Culicidae	Aedes	Aedes aegypti	SPECIES	20.87639	-99.06250	1894400190
10974	Diptera	Culicidae	Aedes	Aedes aegypti	SPECIES	20.88000	-89.75000	1264886248
10975	Diptera	Culicidae	Aedes	Aedes aegypti	SPECIES	20.88000	-101.98167	1894253527
10976	Diptera	Culicidae	Aedes	Aedes aegypti	SPECIES	20.88167	-101.31194	1894254868
10977	Diptera	Culicidae	Aedes	Aedes aegypti	SPECIES	20.88197	-89.75483	2629265069
10978	Diptera	Culicidae	Aedes	Aedes aegypti	SPECIES	20.88278	-101.92278	1894251156
10979	Diptera	Culicidae	Aedes	Aedes aegypti	SPECIES	20.88278	-103.83667	1894400075
10980	Diptera	Culicidae	Aedes	Aedes aegypti	SPECIES	20.88333	-101.43722	1894255796
10981	Diptera	Culicidae	Aedes	Aedes aegypti	SPECIES	20.88333	-89.75000	1894399871
10982	Diptera	Culicidae	Aedes	Aedes aegypti	SPECIES	20.88361	-89.59778	1894399717
10983	Diptera	Culicidae	Aedes	Aedes aegypti	SPECIES	20.88472	-98.49000	1894396075
10984	Diptera	Culicidae	Aedes	Aedes aegypti	SPECIES	20.88472	-100.38972	1894399354
10985	Diptera	Culicidae	Aedes	Aedes aegypti	SPECIES	20.88500	-101.97750	1894251104
10986	Diptera	Culicidae	Aedes	Aedes aegypti	SPECIES	20.89000	-101.35972	1894255536
10987	Diptera	Culicidae	Aedes	Aedes aegypti	SPECIES	20.89139	-101.93028	1894253986
10988	Diptera	Culicidae	Aedes	Aedes aegypti	SPECIES	20.89667	-98.58194	1894399814

№	Order	Family	Genus	Species	Taxon Rank	Latitude	Longitude	GBIF_ID
10989	Diptera	Culicidae	Aedes	Aedes aegypti	SPECIES	20.89693	-98.58008	3384413802
10990	Diptera	Culicidae	Aedes	Aedes aegypti	SPECIES	20.89728	-98.35370	3031834951
10991	Diptera	Culicidae	Aedes	Aedes aegypti	SPECIES	20.89734	-98.35372	3031644921
10992	Diptera	Culicidae	Aedes	Aedes aegypti	SPECIES	20.89807	-98.35245	3031860965
10993	Diptera	Culicidae	Aedes	Aedes aegypti	SPECIES	20.89807	-98.35238	3031589904
10994	Diptera	Culicidae	Aedes	Aedes aegypti	SPECIES	20.89833	-101.19139	1894254935
10995	Diptera	Culicidae	Aedes	Aedes aegypti	SPECIES	20.89833	-98.35556	1894396226
10996	Diptera	Culicidae	Aedes	Aedes aegypti	SPECIES	20.89835	-98.59042	3947132964
10997	Diptera	Culicidae	Aedes	Aedes aegypti	SPECIES	20.89932	-98.35449	3031616968
10998	Diptera	Culicidae	Aedes	Aedes aegypti	SPECIES	20.89988	-98.35513	3031649961
10999	Diptera	Culicidae	Aedes	Aedes aegypti	SPECIES	20.90222	-98.44889	1894399333
11000	Diptera	Culicidae	Aedes	Aedes aegypti	SPECIES	20.90639	-103.97972	1894398636
11001	Diptera	Culicidae	Aedes	Aedes aegypti	SPECIES	20.90806	-101.31361	1894255353
11002	Diptera	Culicidae	Aedes	Aedes aegypti	SPECIES	20.91000	-101.50972	1894253559
11003	Diptera	Culicidae	Aedes	Aedes aegypti	SPECIES	20.91083	-101.25444	1894253676
11004	Diptera	Culicidae	Aedes	Aedes aegypti	SPECIES	20.91167	-101.86056	1894255793
11005	Diptera	Culicidae	Aedes	Aedes aegypti	SPECIES	20.91333	-98.39583	1894398564
11006	Diptera	Culicidae	Aedes	Aedes aegypti	SPECIES	20.91428	-98.32871	3031668907
11007	Diptera	Culicidae	Aedes	Aedes aegypti	SPECIES	20.91450	-98.32722	3031836889
11008	Diptera	Culicidae	Aedes	Aedes aegypti	SPECIES	20.91458	-98.32843	3031951881
11009	Diptera	Culicidae	Aedes	Aedes aegypti	SPECIES	20.91488	-89.69618	3067868286
11010	Diptera	Culicidae	Aedes	Aedes aegypti	SPECIES	20.91526	-98.32750	3031837934
11011	Diptera	Culicidae	Aedes	Aedes aegypti	SPECIES	20.91528	-100.74389	1894255808
11012	Diptera	Culicidae	Aedes	Aedes aegypti	SPECIES	20.91556	-99.91944	1894395562
11013	Diptera	Culicidae	Aedes	Aedes aegypti	SPECIES	20.91556	-99.76694	1894396722
11014	Diptera	Culicidae	Aedes	Aedes aegypti	SPECIES	20.91567	-98.32881	3031973956
11015	Diptera	Culicidae	Aedes	Aedes aegypti	SPECIES	20.91694	-98.32309	3031865992
11016	Diptera	Culicidae	Aedes	Aedes aegypti	SPECIES	20.91722	-98.25389	1894394321
11017	Diptera	Culicidae	Aedes	Aedes aegypti	SPECIES	20.91728	-98.32322	3031970994
11018	Diptera	Culicidae	Aedes	Aedes aegypti	SPECIES	20.91762	-98.32424	3031590970
11019	Diptera	Culicidae	Aedes	Aedes aegypti	SPECIES	20.91772	-98.32554	3031612851
11020	Diptera	Culicidae	Aedes	Aedes aegypti	SPECIES	20.91778	-99.92083	1894396866
11021	Diptera	Culicidae	Aedes	Aedes aegypti	SPECIES	20.91795	-98.32396	3031878956
11022	Diptera	Culicidae	Aedes	Aedes aegypti	SPECIES	20.91961	-98.32562	3031622980
11023	Diptera	Culicidae	Aedes	Aedes aegypti	SPECIES	20.91977	-98.32454	3031900947
11024	Diptera	Culicidae	Aedes	Aedes aegypti	SPECIES	20.91979	-98.32479	3031590969
11025	Diptera	Culicidae	Aedes	Aedes aegypti	SPECIES	20.91992	-98.32487	3031652983
11026	Diptera	Culicidae	Aedes	Aedes aegypti	SPECIES	20.92000	-89.63000	1264886797
11027	Diptera	Culicidae	Aedes	Aedes aegypti	SPECIES	20.92081	-98.32823	3031595962
11028	Diptera	Culicidae	Aedes	Aedes aegypti	SPECIES	20.92083	-101.69722	1894396090
11029	Diptera	Culicidae	Aedes	Aedes aegypti	SPECIES	20.92092	-98.32751	3031893912

№	Order	Family	Genus	Species	Taxon Rank	Latitude	Longitude	GBIF_ID
11030	Diptera	Culicidae	Aedes	Aedes aegypti	SPECIES	20.92167	-101.69694	1894254094
11031	Diptera	Culicidae	Aedes	Aedes aegypti	SPECIES	20.92168	-98.32521	3031851981
11032	Diptera	Culicidae	Aedes	Aedes aegypti	SPECIES	20.92210	-98.32491	3031856963
11033	Diptera	Culicidae	Aedes	Aedes aegypti	SPECIES	20.92250	-98.95000	1894400272
11034	Diptera	Culicidae	Aedes	Aedes aegypti	SPECIES	20.92806	-101.91917	1894255708
11035	Diptera	Culicidae	Aedes	Aedes aegypti	SPECIES	20.93000	-89.60000	1264886836
11036	Diptera	Culicidae	Aedes	Aedes aegypti	SPECIES	20.93139	-89.01778	1894396967
11037	Diptera	Culicidae	Aedes	Aedes aegypti	SPECIES	20.93222	-101.27917	1894255340
11038	Diptera	Culicidae	Aedes	Aedes aegypti	SPECIES	20.93278	-89.12500	1894399967
11039	Diptera	Culicidae	Aedes	Aedes aegypti	SPECIES	20.93500	-101.73417	1894253236
11040	Diptera	Culicidae	Aedes	Aedes aegypti	SPECIES	20.93582	-98.43680	3337995985
11041	Diptera	Culicidae	Aedes	Aedes aegypti	SPECIES	20.93611	-101.28889	1894255854
11042	Diptera	Culicidae	Aedes	Aedes aegypti	SPECIES	20.94000	-89.56000	1264886269
11043	Diptera	Culicidae	Aedes	Aedes aegypti	SPECIES	20.94000	-89.62000	1264890314
11044	Diptera	Culicidae	Aedes	Aedes aegypti	SPECIES	20.94000	-101.47000	1264890939
11045	Diptera	Culicidae	Aedes	Aedes aegypti	SPECIES	20.94000	-99.18111	1894395944
11046	Diptera	Culicidae	Aedes	Aedes aegypti	SPECIES	20.94361	-101.42667	1894255899
11047	Diptera	Culicidae	Aedes	Aedes aegypti	SPECIES	20.94444	-99.16444	1894399617
11048	Diptera	Culicidae	Aedes	Aedes aegypti	SPECIES	20.94861	-89.30194	1894399608
11049	Diptera	Culicidae	Aedes	Aedes aegypti	SPECIES	20.94917	-98.38194	1894399968
11050	Diptera	Culicidae	Aedes	Aedes aegypti	SPECIES	20.95000	-97.46000	1264886267
11051	Diptera	Culicidae	Aedes	Aedes aegypti	SPECIES	20.95000	-97.39000	1264886283
11052	Diptera	Culicidae	Aedes	Aedes aegypti	SPECIES	20.95000	-89.64000	1264888734
11053	Diptera	Culicidae	Aedes	Aedes aegypti	SPECIES	20.95111	-101.25194	1894255748
11054	Diptera	Culicidae	Aedes	Aedes aegypti	SPECIES	20.95139	-98.52528	1894395356
11055	Diptera	Culicidae	Aedes	Aedes aegypti	SPECIES	20.95243	-98.27234	3873024363
11056	Diptera	Culicidae	Aedes	Aedes aegypti	SPECIES	20.95639	-101.57889	1894254964
11057	Diptera	Culicidae	Aedes	Aedes aegypti	SPECIES	20.95889	-97.40306	1894399824
11058	Diptera	Culicidae	Aedes	Aedes aegypti	SPECIES	20.96000	-89.59000	1264887402
11059	Diptera	Culicidae	Aedes	Aedes aegypti	SPECIES	20.96000	-97.41000	1264888757
11060	Diptera	Culicidae	Aedes	Aedes aegypti	SPECIES	20.96000	105.50000	1264889039
11061	Diptera	Culicidae	Aedes	Aedes aegypti	SPECIES	20.96000	-89.61000	1264891435
11062	Diptera	Culicidae	Aedes	Aedes aegypti	SPECIES	20.96222	-98.38306	1894394332
11063	Diptera	Culicidae	Aedes	Aedes aegypti	SPECIES	20.96306	-101.64500	1894255890
11064	Diptera	Culicidae	Aedes	Aedes aegypti	SPECIES	20.96444	-98.78083	1894394801
11065	Diptera	Culicidae	Aedes	Aedes aegypti	SPECIES	20.96500	-101.31639	1894255601
11066	Diptera	Culicidae	Aedes	Aedes aegypti	SPECIES	20.96556	-88.60250	1894399547
11067	Diptera	Culicidae	Aedes	Aedes aegypti	SPECIES	20.96583	-89.62318	2629265466
11068	Diptera	Culicidae	Aedes	Aedes aegypti	SPECIES	20.96778	-101.27333	1894255656
11069	Diptera	Culicidae	Aedes	Aedes aegypti	SPECIES	20.96778	-89.62167	2629264532
11070	Diptera	Culicidae	Aedes	Aedes aegypti	SPECIES	20.96815	-89.62987	3355031886

№	Order	Family	Genus	Species	Taxon Rank	Latitude	Longitude	GBIF_ID
11071	Diptera	Culicidae	Aedes	Aedes aegypti	SPECIES	20.97000	-89.62000	1264886230
11072	Diptera	Culicidae	Aedes	Aedes aegypti	SPECIES	20.97000	-97.40000	1264886255
11073	Diptera	Culicidae	Aedes	Aedes aegypti	SPECIES	20.97000	-97.33000	1264886274
11074	Diptera	Culicidae	Aedes	Aedes aegypti	SPECIES	20.97000	-89.58000	1264886768
11075	Diptera	Culicidae	Aedes	Aedes aegypti	SPECIES	20.97000	-89.68000	1264886802
11076	Diptera	Culicidae	Aedes	Aedes aegypti	SPECIES	20.97000	-89.57000	1264887126
11077	Diptera	Culicidae	Aedes	Aedes aegypti	SPECIES	20.97000	-89.63000	1264887576
11078	Diptera	Culicidae	Aedes	Aedes aegypti	SPECIES	20.97000	-89.67000	1264888732
11079	Diptera	Culicidae	Aedes	Aedes aegypti	SPECIES	20.97000	-98.39972	1894400147
11080	Diptera	Culicidae	Aedes	Aedes aegypti	SPECIES	20.97127	-89.62160	4052766552
11081	Diptera	Culicidae	Aedes	Aedes aegypti	SPECIES	20.98000	-89.66000	1264888752
11082	Diptera	Culicidae	Aedes	Aedes aegypti	SPECIES	20.98000	-89.62000	1264890848
11083	Diptera	Culicidae	Aedes	Aedes aegypti	SPECIES	20.98083	-98.50778	1894394589
11084	Diptera	Culicidae	Aedes	Aedes aegypti	SPECIES	20.98611	-98.45167	1894396738
11085	Diptera	Culicidae	Aedes	Aedes aegypti	SPECIES	20.98986	-89.61290	3747280141
11086	Diptera	Culicidae	Aedes	Aedes aegypti	SPECIES	20.99000	-89.58000	1264888714
11087	Diptera	Culicidae	Aedes	Aedes aegypti	SPECIES	20.99000	-89.62000	1264888751
11088	Diptera	Culicidae	Aedes	Aedes aegypti	SPECIES	20.99028	-98.97472	1894395822
11089	Diptera	Culicidae	Aedes	Aedes aegypti	SPECIES	20.99056	-98.99417	1894397008
11090	Diptera	Culicidae	Aedes	Aedes aegypti	SPECIES	20.99279	-98.39544	3031582963
11091	Diptera	Culicidae	Aedes	Aedes aegypti	SPECIES	20.99409	-98.39528	3872894381
11092	Diptera	Culicidae	Aedes	Aedes aegypti	SPECIES	20.99433	-98.39473	3031607940
11093	Diptera	Culicidae	Aedes	Aedes aegypti	SPECIES	20.99750	-101.28556	1894255651
11094	Diptera	Culicidae	Aedes	Aedes aegypti	SPECIES	20.99972	-98.36889	1894398059
11095	Diptera	Culicidae	Aedes	Aedes aegypti	SPECIES	21.00000	-89.70000	1264886261
11096	Diptera	Culicidae	Aedes	Aedes aegypti	SPECIES	21.00222	-89.39361	1894398992
11097	Diptera	Culicidae	Aedes	Aedes aegypti	SPECIES	21.00500	-99.19000	1894400003
11098	Diptera	Culicidae	Aedes	Aedes aegypti	SPECIES	21.00667	-101.62139	1894250106
11099	Diptera	Culicidae	Aedes	Aedes aegypti	SPECIES	21.00744	-89.63144	2629264729
11100	Diptera	Culicidae	Aedes	Aedes aegypti	SPECIES	21.00889	-98.29944	1894394228
11101	Diptera	Culicidae	Aedes	Aedes aegypti	SPECIES	21.01000	-89.63000	1264888741
11102	Diptera	Culicidae	Aedes	Aedes aegypti	SPECIES	21.01444	-98.39389	1894399194
11103	Diptera	Culicidae	Aedes	Aedes aegypti	SPECIES	21.01500	-101.53083	1894254272
11104	Diptera	Culicidae	Aedes	Aedes aegypti	SPECIES	21.01502	-98.39663	3873048399
11105	Diptera	Culicidae	Aedes	Aedes aegypti	SPECIES	21.01510	-98.39737	3873008360
11106	Diptera	Culicidae	Aedes	Aedes aegypti	SPECIES	21.01516	-98.39239	3873403366
11107	Diptera	Culicidae	Aedes	Aedes aegypti	SPECIES	21.01523	-98.39281	3872871385
11108	Diptera	Culicidae	Aedes	Aedes aegypti	SPECIES	21.01528	-89.87444	1894400093
11109	Diptera	Culicidae	Aedes	Aedes aegypti	SPECIES	21.01528	-98.39317	3873056406
11110	Diptera	Culicidae	Aedes	Aedes aegypti	SPECIES	21.01530	-98.39465	3872884390
11111	Diptera	Culicidae	Aedes	Aedes aegypti	SPECIES	21.01541	-98.39502	3873425399

№	Order	Family	Genus	Species	Taxon Rank	Latitude	Longitude	GBIF_ID
11112	Diptera	Culicidae	Aedes	Aedes aegypti	SPECIES	21.01545	-98.39349	3873362388
11113	Diptera	Culicidae	Aedes	Aedes aegypti	SPECIES	21.01559	-98.34789	3873727378
11114	Diptera	Culicidae	Aedes	Aedes aegypti	SPECIES	21.01570	-98.39739	3031660973
11115	Diptera	Culicidae	Aedes	Aedes aegypti	SPECIES	21.01581	-98.39487	3873679383
11116	Diptera	Culicidae	Aedes	Aedes aegypti	SPECIES	21.01595	-98.39313	3872995398
11117	Diptera	Culicidae	Aedes	Aedes aegypti	SPECIES	21.01595	-98.34597	3873549383
11118	Diptera	Culicidae	Aedes	Aedes aegypti	SPECIES	21.01597	-98.34503	3031632892
11119	Diptera	Culicidae	Aedes	Aedes aegypti	SPECIES	21.01598	-98.34505	3872956394
11120	Diptera	Culicidae	Aedes	Aedes aegypti	SPECIES	21.01604	-98.34492	3873048376
11121	Diptera	Culicidae	Aedes	Aedes aegypti	SPECIES	21.01608	-98.39630	3031850940
11122	Diptera	Culicidae	Aedes	Aedes aegypti	SPECIES	21.01609	-98.39598	3031869988
11123	Diptera	Culicidae	Aedes	Aedes aegypti	SPECIES	21.01614	-98.34428	3031575958
11124	Diptera	Culicidae	Aedes	Aedes aegypti	SPECIES	21.01617	-98.34643	3873509377
11125	Diptera	Culicidae	Aedes	Aedes aegypti	SPECIES	21.01619	-98.34646	3873013364
11126	Diptera	Culicidae	Aedes	Aedes aegypti	SPECIES	21.01625	-98.34488	3872960374
11127	Diptera	Culicidae	Aedes	Aedes aegypti	SPECIES	21.01628	-98.34690	3873328360
11128	Diptera	Culicidae	Aedes	Aedes aegypti	SPECIES	21.01630	-98.34583	3873711356
11129	Diptera	Culicidae	Aedes	Aedes aegypti	SPECIES	21.01635	-98.34645	3873649364
11130	Diptera	Culicidae	Aedes	Aedes aegypti	SPECIES	21.01638	-98.34595	3031851900
11131	Diptera	Culicidae	Aedes	Aedes aegypti	SPECIES	21.01642	-98.39502	3031944927
11132	Diptera	Culicidae	Aedes	Aedes aegypti	SPECIES	21.01642	-98.34579	3872943367
11133	Diptera	Culicidae	Aedes	Aedes aegypti	SPECIES	21.01653	-98.39482	3031627021
11134	Diptera	Culicidae	Aedes	Aedes aegypti	SPECIES	21.01654	-98.34648	3873015361
11135	Diptera	Culicidae	Aedes	Aedes aegypti	SPECIES	21.01661	-98.34749	3872894382
11136	Diptera	Culicidae	Aedes	Aedes aegypti	SPECIES	21.01667	-99.19111	1894398947
11137	Diptera	Culicidae	Aedes	Aedes aegypti	SPECIES	21.01667	-98.34652	3873040368
11138	Diptera	Culicidae	Aedes	Aedes aegypti	SPECIES	21.01693	-98.34687	3873233350
11139	Diptera	Culicidae	Aedes	Aedes aegypti	SPECIES	21.01694	-98.34806	1894400192
11140	Diptera	Culicidae	Aedes	Aedes aegypti	SPECIES	21.01722	-101.85806	1894399196
11141	Diptera	Culicidae	Aedes	Aedes aegypti	SPECIES	21.01744	-98.34744	3872998356
11142	Diptera	Culicidae	Aedes	Aedes aegypti	SPECIES	21.01746	-98.34390	3031816921
11143	Diptera	Culicidae	Aedes	Aedes aegypti	SPECIES	21.01758	-98.34745	2580115194
11144	Diptera	Culicidae	Aedes	Aedes aegypti	SPECIES	21.01778	-101.25667	1894255930
11145	Diptera	Culicidae	Aedes	Aedes aegypti	SPECIES	21.01785	-98.34871	3031584909
11146	Diptera	Culicidae	Aedes	Aedes aegypti	SPECIES	21.01806	-101.64306	1894398317
11147	Diptera	Culicidae	Aedes	Aedes aegypti	SPECIES	21.01807	-98.34753	3873275344
11148	Diptera	Culicidae	Aedes	Aedes aegypti	SPECIES	21.01815	-98.34193	3873468379
11149	Diptera	Culicidae	Aedes	Aedes aegypti	SPECIES	21.01833	-101.85778	1894255849
11150	Diptera	Culicidae	Aedes	Aedes aegypti	SPECIES	21.01833	-98.48556	1894397978
11151	Diptera	Culicidae	Aedes	Aedes aegypti	SPECIES	21.01849	-98.34906	3031837933
11152	Diptera	Culicidae	Aedes	Aedes aegypti	SPECIES	21.01851	-98.34605	3872951379

№	Order	Family	Genus	Species	Taxon Rank	Latitude	Longitude	GBIF_ID
11153	Diptera	Culicidae	Aedes	Aedes aegypti	SPECIES	21.01853	-98.34376	3873450376
11154	Diptera	Culicidae	Aedes	Aedes aegypti	SPECIES	21.01866	-98.34718	3873680377
11155	Diptera	Culicidae	Aedes	Aedes aegypti	SPECIES	21.01872	-101.65327	4145484252
11156	Diptera	Culicidae	Aedes	Aedes aegypti	SPECIES	21.01883	-98.34199	3873572382
11157	Diptera	Culicidae	Aedes	Aedes aegypti	SPECIES	21.01889	-101.64278	1894255876
11158	Diptera	Culicidae	Aedes	Aedes aegypti	SPECIES	21.01890	-98.34598	3873031352
11159	Diptera	Culicidae	Aedes	Aedes aegypti	SPECIES	21.01896	-98.34590	3872997379
11160	Diptera	Culicidae	Aedes	Aedes aegypti	SPECIES	21.01905	-98.34900	3872956395
11161	Diptera	Culicidae	Aedes	Aedes aegypti	SPECIES	21.01951	-98.34581	3873387380
11162	Diptera	Culicidae	Aedes	Aedes aegypti	SPECIES	21.01964	-98.34635	2580117221
11163	Diptera	Culicidae	Aedes	Aedes aegypti	SPECIES	21.01996	-98.34643	3031848916
11164	Diptera	Culicidae	Aedes	Aedes aegypti	SPECIES	21.01996	-98.34556	3873719383
11165	Diptera	Culicidae	Aedes	Aedes aegypti	SPECIES	21.02000	-89.88000	1264886240
11166	Diptera	Culicidae	Aedes	Aedes aegypti	SPECIES	21.02000	106.15000	1264889532
11167	Diptera	Culicidae	Aedes	Aedes aegypti	SPECIES	21.02000	-101.41083	1894249629
11168	Diptera	Culicidae	Aedes	Aedes aegypti	SPECIES	21.02000	-99.19583	1894397539
11169	Diptera	Culicidae	Aedes	Aedes aegypti	SPECIES	21.02015	-98.34555	3873502361
11170	Diptera	Culicidae	Aedes	Aedes aegypti	SPECIES	21.02025	-98.34681	3873638386
11171	Diptera	Culicidae	Aedes	Aedes aegypti	SPECIES	21.02081	-98.34754	3873378363
11172	Diptera	Culicidae	Aedes	Aedes aegypti	SPECIES	21.02095	-98.35063	2580227426
11173	Diptera	Culicidae	Aedes	Aedes aegypti	SPECIES	21.02125	-98.34744	3872911383
11174	Diptera	Culicidae	Aedes	Aedes aegypti	SPECIES	21.02134	-98.34572	3873634381
11175	Diptera	Culicidae	Aedes	Aedes aegypti	SPECIES	21.02134	-98.34597	2580093215
11176	Diptera	Culicidae	Aedes	Aedes aegypti	SPECIES	21.02152	-98.34602	3872969350
11177	Diptera	Culicidae	Aedes	Aedes aegypti	SPECIES	21.02167	-98.52694	1894395047
11178	Diptera	Culicidae	Aedes	Aedes aegypti	SPECIES	21.02212	-98.34570	3873022354
11179	Diptera	Culicidae	Aedes	Aedes aegypti	SPECIES	21.02216	-98.34825	3031943958
11180	Diptera	Culicidae	Aedes	Aedes aegypti	SPECIES	21.02220	-98.34724	2580264419
11181	Diptera	Culicidae	Aedes	Aedes aegypti	SPECIES	21.02222	-98.24667	1894399641
11182	Diptera	Culicidae	Aedes	Aedes aegypti	SPECIES	21.02230	-98.34786	3873464407
11183	Diptera	Culicidae	Aedes	Aedes aegypti	SPECIES	21.02260	-98.34720	3031950940
11184	Diptera	Culicidae	Aedes	Aedes aegypti	SPECIES	21.02278	-98.24575	3031859853
11185	Diptera	Culicidae	Aedes	Aedes aegypti	SPECIES	21.02306	-101.53917	1894253928
11186	Diptera	Culicidae	Aedes	Aedes aegypti	SPECIES	21.02353	-98.34933	3031581866
11187	Diptera	Culicidae	Aedes	Aedes aegypti	SPECIES	21.02404	-98.34296	3031882879
11188	Diptera	Culicidae	Aedes	Aedes aegypti	SPECIES	21.02456	-98.24888	3031617969
11189	Diptera	Culicidae	Aedes	Aedes aegypti	SPECIES	21.02471	-98.24648	1894397932
11190	Diptera	Culicidae	Aedes	Aedes aegypti	SPECIES	21.02472	-101.31417	1894254540
11191	Diptera	Culicidae	Aedes	Aedes aegypti	SPECIES	21.02475	-98.24625	3031663934
11192	Diptera	Culicidae	Aedes	Aedes aegypti	SPECIES	21.02480	-98.34530	3873727379
11193	Diptera	Culicidae	Aedes	Aedes aegypti	SPECIES	21.02494	-98.34100	3873048375

№	Order	Family	Genus	Species	Taxon Rank	Latitude	Longitude	GBIF_ID
11194	Diptera	Culicidae	Aedes	Aedes aegypti	SPECIES	21.02514	-98.24656	3873471378
11195	Diptera	Culicidae	Aedes	Aedes aegypti	SPECIES	21.02522	-98.34556	3872966342
11196	Diptera	Culicidae	Aedes	Aedes aegypti	SPECIES	21.02525	-98.24860	3872907395
11197	Diptera	Culicidae	Aedes	Aedes aegypti	SPECIES	21.02525	-98.34582	3872971387
11198	Diptera	Culicidae	Aedes	Aedes aegypti	SPECIES	21.02536	-98.34427	3872957397
11199	Diptera	Culicidae	Aedes	Aedes aegypti	SPECIES	21.02546	-98.34207	3872967377
11200	Diptera	Culicidae	Aedes	Aedes aegypti	SPECIES	21.02548	-98.24796	3873520365
11201	Diptera	Culicidae	Aedes	Aedes aegypti	SPECIES	21.02564	-98.34319	3872912356
11202	Diptera	Culicidae	Aedes	Aedes aegypti	SPECIES	21.02593	-98.34014	3873022355
11203	Diptera	Culicidae	Aedes	Aedes aegypti	SPECIES	21.02596	-98.34083	3031807914
11204	Diptera	Culicidae	Aedes	Aedes aegypti	SPECIES	21.02597	-98.34373	3873710395
11205	Diptera	Culicidae	Aedes	Aedes aegypti	SPECIES	21.02603	-98.34184	3873726404
11206	Diptera	Culicidae	Aedes	Aedes aegypti	SPECIES	21.02630	-98.24546	3031664851
11207	Diptera	Culicidae	Aedes	Aedes aegypti	SPECIES	21.02634	-98.34103	3873384348
11208	Diptera	Culicidae	Aedes	Aedes aegypti	SPECIES	21.02638	-98.34094	3031819896
11209	Diptera	Culicidae	Aedes	Aedes aegypti	SPECIES	21.02663	-98.24584	3031971877
11210	Diptera	Culicidae	Aedes	Aedes aegypti	SPECIES	21.02664	-98.24799	3872966363
11211	Diptera	Culicidae	Aedes	Aedes aegypti	SPECIES	21.02685	-98.28900	3873706361
11212	Diptera	Culicidae	Aedes	Aedes aegypti	SPECIES	21.02688	-98.28900	3031664897
11213	Diptera	Culicidae	Aedes	Aedes aegypti	SPECIES	21.02690	-98.24588	3872911385
11214	Diptera	Culicidae	Aedes	Aedes aegypti	SPECIES	21.02692	-98.24745	3872914369
11215	Diptera	Culicidae	Aedes	Aedes aegypti	SPECIES	21.02708	-98.24761	3031619993
11216	Diptera	Culicidae	Aedes	Aedes aegypti	SPECIES	21.02732	-98.24777	3873462367
11217	Diptera	Culicidae	Aedes	Aedes aegypti	SPECIES	21.02733	-98.28899	3031972854
11218	Diptera	Culicidae	Aedes	Aedes aegypti	SPECIES	21.02784	-98.29040	3031969821
11219	Diptera	Culicidae	Aedes	Aedes aegypti	SPECIES	21.02807	-98.28775	3031850880
11220	Diptera	Culicidae	Aedes	Aedes aegypti	SPECIES	21.02820	-98.28872	3031661932
11221	Diptera	Culicidae	Aedes	Aedes aegypti	SPECIES	21.02861	-98.29167	1894394409
11222	Diptera	Culicidae	Aedes	Aedes aegypti	SPECIES	21.02885	-98.28687	3031966941
11223	Diptera	Culicidae	Aedes	Aedes aegypti	SPECIES	21.02936	-98.28950	3873718382
11224	Diptera	Culicidae	Aedes	Aedes aegypti	SPECIES	21.02940	-98.28637	3031965861
11225	Diptera	Culicidae	Aedes	Aedes aegypti	SPECIES	21.02944	-101.87944	1894395070
11226	Diptera	Culicidae	Aedes	Aedes aegypti	SPECIES	21.02948	-98.29010	3031643945
11227	Diptera	Culicidae	Aedes	Aedes aegypti	SPECIES	21.02951	-98.28574	3873048382
11228	Diptera	Culicidae	Aedes	Aedes aegypti	SPECIES	21.02962	-98.29332	3031674860
11229	Diptera	Culicidae	Aedes	Aedes aegypti	SPECIES	21.02965	-98.29205	3031639843
11230	Diptera	Culicidae	Aedes	Aedes aegypti	SPECIES	21.02986	-98.28991	3873362371
11231	Diptera	Culicidae	Aedes	Aedes aegypti	SPECIES	21.02994	-98.28696	3872909383
11232	Diptera	Culicidae	Aedes	Aedes aegypti	SPECIES	21.03000	-89.56000	1264886812
11233	Diptera	Culicidae	Aedes	Aedes aegypti	SPECIES	21.03000	105.85000	1264889514
11234	Diptera	Culicidae	Aedes	Aedes aegypti	SPECIES	21.03000	106.15000	1264889522

№	Order	Family	Genus	Species	Taxon Rank	Latitude	Longitude	GBIF_ID
11235	Diptera	Culicidae	Aedes	Aedes aegypti	SPECIES	21.03007	-98.29176	3031806827
11236	Diptera	Culicidae	Aedes	Aedes aegypti	SPECIES	21.03016	-98.28937	3873608369
11237	Diptera	Culicidae	Aedes	Aedes aegypti	SPECIES	21.03019	-98.29010	3031963887
11238	Diptera	Culicidae	Aedes	Aedes aegypti	SPECIES	21.03056	-101.87917	1894255851
11239	Diptera	Culicidae	Aedes	Aedes aegypti	SPECIES	21.03083	-98.28639	1894400258
11240	Diptera	Culicidae	Aedes	Aedes aegypti	SPECIES	21.03104	-98.28700	3873307359
11241	Diptera	Culicidae	Aedes	Aedes aegypti	SPECIES	21.03193	-98.28989	3031669868
11242	Diptera	Culicidae	Aedes	Aedes aegypti	SPECIES	21.03200	-98.29020	3873016388
11243	Diptera	Culicidae	Aedes	Aedes aegypti	SPECIES	21.03214	-98.28395	3873349387
11244	Diptera	Culicidae	Aedes	Aedes aegypti	SPECIES	21.03214	-98.29014	3873003399
11245	Diptera	Culicidae	Aedes	Aedes aegypti	SPECIES	21.03220	-98.28580	3873296382
11246	Diptera	Culicidae	Aedes	Aedes aegypti	SPECIES	21.03235	-98.28993	3031617892
11247	Diptera	Culicidae	Aedes	Aedes aegypti	SPECIES	21.03242	-98.29003	3873506380
11248	Diptera	Culicidae	Aedes	Aedes aegypti	SPECIES	21.03247	-98.28780	3872923379
11249	Diptera	Culicidae	Aedes	Aedes aegypti	SPECIES	21.03251	-98.29141	3031613909
11250	Diptera	Culicidae	Aedes	Aedes aegypti	SPECIES	21.03266	-98.28913	3872976376
11251	Diptera	Culicidae	Aedes	Aedes aegypti	SPECIES	21.03275	-98.29070	2580094667
11252	Diptera	Culicidae	Aedes	Aedes aegypti	SPECIES	21.03283	-98.28756	3873584392
11253	Diptera	Culicidae	Aedes	Aedes aegypti	SPECIES	21.03287	-98.28761	3872867384
11254	Diptera	Culicidae	Aedes	Aedes aegypti	SPECIES	21.03292	-98.28962	3031651888
11255	Diptera	Culicidae	Aedes	Aedes aegypti	SPECIES	21.03292	-98.29056	3031673907
11256	Diptera	Culicidae	Aedes	Aedes aegypti	SPECIES	21.03311	-98.28671	3031641867
11257	Diptera	Culicidae	Aedes	Aedes aegypti	SPECIES	21.03332	-98.28657	3031867856
11258	Diptera	Culicidae	Aedes	Aedes aegypti	SPECIES	21.03344	-98.35112	3031953910
11259	Diptera	Culicidae	Aedes	Aedes aegypti	SPECIES	21.03348	-98.28393	3873048381
11260	Diptera	Culicidae	Aedes	Aedes aegypti	SPECIES	21.03361	-99.77694	1894399195
11261	Diptera	Culicidae	Aedes	Aedes aegypti	SPECIES	21.03369	-98.28710	3031605922
11262	Diptera	Culicidae	Aedes	Aedes aegypti	SPECIES	21.03372	-98.28843	3031865939
11263	Diptera	Culicidae	Aedes	Aedes aegypti	SPECIES	21.03376	-98.28879	3031648917
11264	Diptera	Culicidae	Aedes	Aedes aegypti	SPECIES	21.03397	-98.28792	3031849876
11265	Diptera	Culicidae	Aedes	Aedes aegypti	SPECIES	21.03411	-98.28762	3873660377
11266	Diptera	Culicidae	Aedes	Aedes aegypti	SPECIES	21.03414	-98.28537	3031878840
11267	Diptera	Culicidae	Aedes	Aedes aegypti	SPECIES	21.03429	-98.28598	3031849854
11268	Diptera	Culicidae	Aedes	Aedes aegypti	SPECIES	21.03477	-98.28717	3873549387
11269	Diptera	Culicidae	Aedes	Aedes aegypti	SPECIES	21.03481	-98.28767	3873265376
11270	Diptera	Culicidae	Aedes	Aedes aegypti	SPECIES	21.03490	-98.28743	3031582909
11271	Diptera	Culicidae	Aedes	Aedes aegypti	SPECIES	21.03501	-98.28642	3031659852
11272	Diptera	Culicidae	Aedes	Aedes aegypti	SPECIES	21.03556	-98.79722	1894399047
11273	Diptera	Culicidae	Aedes	Aedes aegypti	SPECIES	21.03561	-98.28707	3873492397
11274	Diptera	Culicidae	Aedes	Aedes aegypti	SPECIES	21.03589	-98.28698	3873287393
11275	Diptera	Culicidae	Aedes	Aedes aegypti	SPECIES	21.03608	-98.28840	3873050380

№	Order	Family	Genus	Species	Taxon Rank	Latitude	Longitude	GBIF_ID
11276	Diptera	Culicidae	Aedes	Aedes aegypti	SPECIES	21.03636	-98.28846	2580090665
11277	Diptera	Culicidae	Aedes	Aedes aegypti	SPECIES	21.03651	-98.28397	3872932393
11278	Diptera	Culicidae	Aedes	Aedes aegypti	SPECIES	21.03656	-98.28402	3031654932
11279	Diptera	Culicidae	Aedes	Aedes aegypti	SPECIES	21.03759	-98.28913	3031575911
11280	Diptera	Culicidae	Aedes	Aedes aegypti	SPECIES	21.03759	-98.28265	3031658847
11281	Diptera	Culicidae	Aedes	Aedes aegypti	SPECIES	21.03799	-98.28290	3873309383
11282	Diptera	Culicidae	Aedes	Aedes aegypti	SPECIES	21.03833	-99.79861	1894396532
11283	Diptera	Culicidae	Aedes	Aedes aegypti	SPECIES	21.03856	-98.28272	3873052380
11284	Diptera	Culicidae	Aedes	Aedes aegypti	SPECIES	21.03857	-98.28969	3031656928
11285	Diptera	Culicidae	Aedes	Aedes aegypti	SPECIES	21.03861	-101.44250	1894254315
11286	Diptera	Culicidae	Aedes	Aedes aegypti	SPECIES	21.03936	-98.37691	3873706397
11287	Diptera	Culicidae	Aedes	Aedes aegypti	SPECIES	21.03938	-98.31057	3031874971
11288	Diptera	Culicidae	Aedes	Aedes aegypti	SPECIES	21.03941	-98.37137	3872981400
11289	Diptera	Culicidae	Aedes	Aedes aegypti	SPECIES	21.03947	-98.31084	3031596964
11290	Diptera	Culicidae	Aedes	Aedes aegypti	SPECIES	21.03964	-98.37705	3873010354
11291	Diptera	Culicidae	Aedes	Aedes aegypti	SPECIES	21.03969	-98.28168	3031619924
11292	Diptera	Culicidae	Aedes	Aedes aegypti	SPECIES	21.03973	-98.28176	3873682387
11293	Diptera	Culicidae	Aedes	Aedes aegypti	SPECIES	21.03996	-98.31214	3031672967
11294	Diptera	Culicidae	Aedes	Aedes aegypti	SPECIES	21.04000	-89.64000	1264887087
11295	Diptera	Culicidae	Aedes	Aedes aegypti	SPECIES	21.04002	-98.37686	3031636872
11296	Diptera	Culicidae	Aedes	Aedes aegypti	SPECIES	21.04008	-98.37915	3872903361
11297	Diptera	Culicidae	Aedes	Aedes aegypti	SPECIES	21.04014	-98.30982	3031970995
11298	Diptera	Culicidae	Aedes	Aedes aegypti	SPECIES	21.04046	-98.37267	3873611387
11299	Diptera	Culicidae	Aedes	Aedes aegypti	SPECIES	21.04047	-98.37834	2814103498
11300	Diptera	Culicidae	Aedes	Aedes aegypti	SPECIES	21.04055	-98.31243	3031663062
11301	Diptera	Culicidae	Aedes	Aedes aegypti	SPECIES	21.04056	-98.31139	1894396170
11302	Diptera	Culicidae	Aedes	Aedes aegypti	SPECIES	21.04065	-98.37697	3873503369
11303	Diptera	Culicidae	Aedes	Aedes aegypti	SPECIES	21.04079	-98.37212	3873409374
11304	Diptera	Culicidae	Aedes	Aedes aegypti	SPECIES	21.04094	-98.31241	3031641933
11305	Diptera	Culicidae	Aedes	Aedes aegypti	SPECIES	21.04127	-98.31182	3031611999
11306	Diptera	Culicidae	Aedes	Aedes aegypti	SPECIES	21.04170	-98.37842	3873003374
11307	Diptera	Culicidae	Aedes	Aedes aegypti	SPECIES	21.04177	-98.37730	3872882374
11308	Diptera	Culicidae	Aedes	Aedes aegypti	SPECIES	21.04187	-98.37777	3872988391
11309	Diptera	Culicidae	Aedes	Aedes aegypti	SPECIES	21.04192	-98.37717	3872998342
11310	Diptera	Culicidae	Aedes	Aedes aegypti	SPECIES	21.04194	-98.39472	1894398044
11311	Diptera	Culicidae	Aedes	Aedes aegypti	SPECIES	21.04208	-98.37750	3872881376
11312	Diptera	Culicidae	Aedes	Aedes aegypti	SPECIES	21.04209	-98.28047	3872971389
11313	Diptera	Culicidae	Aedes	Aedes aegypti	SPECIES	21.04279	-98.35161	3031605982
11314	Diptera	Culicidae	Aedes	Aedes aegypti	SPECIES	21.04289	-98.28015	3873226375
11315	Diptera	Culicidae	Aedes	Aedes aegypti	SPECIES	21.04302	-98.35032	3872984363
11316	Diptera	Culicidae	Aedes	Aedes aegypti	SPECIES	21.04306	-89.55639	2629263758

№	Order	Family	Genus	Species	Taxon Rank	Latitude	Longitude	GBIF_ID
11317	Diptera	Culicidae	Aedes	Aedes aegypti	SPECIES	21.04308	-98.28002	2856575503
11318	Diptera	Culicidae	Aedes	Aedes aegypti	SPECIES	21.04314	-98.28359	3031853873
11319	Diptera	Culicidae	Aedes	Aedes aegypti	SPECIES	21.04329	-98.28349	3031864976
11320	Diptera	Culicidae	Aedes	Aedes aegypti	SPECIES	21.04333	-100.64278	1894255539
11321	Diptera	Culicidae	Aedes	Aedes aegypti	SPECIES	21.04343	-98.28111	3031972950
11322	Diptera	Culicidae	Aedes	Aedes aegypti	SPECIES	21.04347	-98.27735	3031619955
11323	Diptera	Culicidae	Aedes	Aedes aegypti	SPECIES	21.04352	-98.28328	2856864514
11324	Diptera	Culicidae	Aedes	Aedes aegypti	SPECIES	21.04356	-98.28099	2856648481
11325	Diptera	Culicidae	Aedes	Aedes aegypti	SPECIES	21.04386	-98.35056	3872998358
11326	Diptera	Culicidae	Aedes	Aedes aegypti	SPECIES	21.04410	-98.34691	2813969169
11327	Diptera	Culicidae	Aedes	Aedes aegypti	SPECIES	21.04421	-98.34722	3873023370
11328	Diptera	Culicidae	Aedes	Aedes aegypti	SPECIES	21.04438	-98.27966	3872984362
11329	Diptera	Culicidae	Aedes	Aedes aegypti	SPECIES	21.04442	-98.27978	3031655927
11330	Diptera	Culicidae	Aedes	Aedes aegypti	SPECIES	21.04471	-98.27949	3872949369
11331	Diptera	Culicidae	Aedes	Aedes aegypti	SPECIES	21.04498	-98.34955	3873607392
11332	Diptera	Culicidae	Aedes	Aedes aegypti	SPECIES	21.04558	-98.34832	3031581865
11333	Diptera	Culicidae	Aedes	Aedes aegypti	SPECIES	21.04570	-98.35025	3872989362
11334	Diptera	Culicidae	Aedes	Aedes aegypti	SPECIES	21.04861	-100.55083	1894255204
11335	Diptera	Culicidae	Aedes	Aedes aegypti	SPECIES	21.04889	-89.07333	1894398229
11336	Diptera	Culicidae	Aedes	Aedes aegypti	SPECIES	21.04889	-98.37509	3873230362
11337	Diptera	Culicidae	Aedes	Aedes aegypti	SPECIES	21.05000	-89.07000	1264886241
11338	Diptera	Culicidae	Aedes	Aedes aegypti	SPECIES	21.05000	-89.04000	1264886254
11339	Diptera	Culicidae	Aedes	Aedes aegypti	SPECIES	21.05056	-99.29583	1894397001
11340	Diptera	Culicidae	Aedes	Aedes aegypti	SPECIES	21.05056	-99.70444	1894398172
11341	Diptera	Culicidae	Aedes	Aedes aegypti	SPECIES	21.05089	-98.51132	3872967376
11342	Diptera	Culicidae	Aedes	Aedes aegypti	SPECIES	21.05111	-98.51146	3872877361
11343	Diptera	Culicidae	Aedes	Aedes aegypti	SPECIES	21.05135	-98.51128	3031819897
11344	Diptera	Culicidae	Aedes	Aedes aegypti	SPECIES	21.05147	-98.50886	3031598861
11345	Diptera	Culicidae	Aedes	Aedes aegypti	SPECIES	21.05155	-98.51118	3031587902
11346	Diptera	Culicidae	Aedes	Aedes aegypti	SPECIES	21.05157	-98.50951	3031659894
11347	Diptera	Culicidae	Aedes	Aedes aegypti	SPECIES	21.05169	-98.50867	3031830910
11348	Diptera	Culicidae	Aedes	Aedes aegypti	SPECIES	21.05194	-100.62722	1894255056
11349	Diptera	Culicidae	Aedes	Aedes aegypti	SPECIES	21.05194	-99.81500	1894398186
11350	Diptera	Culicidae	Aedes	Aedes aegypti	SPECIES	21.05194	-99.31083	1894399913
11351	Diptera	Culicidae	Aedes	Aedes aegypti	SPECIES	21.05199	-98.51119	3873702381
11352	Diptera	Culicidae	Aedes	Aedes aegypti	SPECIES	21.05210	-98.51080	2580253300
11353	Diptera	Culicidae	Aedes	Aedes aegypti	SPECIES	21.05232	-98.50875	2580122080
11354	Diptera	Culicidae	Aedes	Aedes aegypti	SPECIES	21.05233	-98.51023	3873037354
11355	Diptera	Culicidae	Aedes	Aedes aegypti	SPECIES	21.05250	-98.50778	1894399826
11356	Diptera	Culicidae	Aedes	Aedes aegypti	SPECIES	21.05255	-98.51275	3873028371
11357	Diptera	Culicidae	Aedes	Aedes aegypti	SPECIES	21.05258	-98.51270	3031609913

№	Order	Family	Genus	Species	Taxon Rank	Latitude	Longitude	GBIF_ID
11358	Diptera	Culicidae	Aedes	Aedes aegypti	SPECIES	21.05258	-98.51295	3031833857
11359	Diptera	Culicidae	Aedes	Aedes aegypti	SPECIES	21.05263	-98.51265	3873506359
11360	Diptera	Culicidae	Aedes	Aedes aegypti	SPECIES	21.05267	-98.50884	3872969349
11361	Diptera	Culicidae	Aedes	Aedes aegypti	SPECIES	21.05276	-98.50698	2580254292
11362	Diptera	Culicidae	Aedes	Aedes aegypti	SPECIES	21.05283	-98.50817	3031815967
11363	Diptera	Culicidae	Aedes	Aedes aegypti	SPECIES	21.05284	-98.50817	3031647921
11364	Diptera	Culicidae	Aedes	Aedes aegypti	SPECIES	21.05287	-98.51009	3873045395
11365	Diptera	Culicidae	Aedes	Aedes aegypti	SPECIES	21.05298	-98.50708	3873709377
11366	Diptera	Culicidae	Aedes	Aedes aegypti	SPECIES	21.05306	-98.50997	3031575942
11367	Diptera	Culicidae	Aedes	Aedes aegypti	SPECIES	21.05307	-98.50998	2580213281
11368	Diptera	Culicidae	Aedes	Aedes aegypti	SPECIES	21.05311	-98.50604	3031580962
11369	Diptera	Culicidae	Aedes	Aedes aegypti	SPECIES	21.05318	-98.50976	3873593376
11370	Diptera	Culicidae	Aedes	Aedes aegypti	SPECIES	21.05324	-98.51003	3873611359
11371	Diptera	Culicidae	Aedes	Aedes aegypti	SPECIES	21.05335	-98.51356	3872923387
11372	Diptera	Culicidae	Aedes	Aedes aegypti	SPECIES	21.05337	-98.50975	3031806889
11373	Diptera	Culicidae	Aedes	Aedes aegypti	SPECIES	21.05337	-98.51126	3031867855
11374	Diptera	Culicidae	Aedes	Aedes aegypti	SPECIES	21.05372	-98.50645	3031973924
11375	Diptera	Culicidae	Aedes	Aedes aegypti	SPECIES	21.05372	-98.50926	3873226384
11376	Diptera	Culicidae	Aedes	Aedes aegypti	SPECIES	21.05379	-98.51304	3031850879
11377	Diptera	Culicidae	Aedes	Aedes aegypti	SPECIES	21.05389	-89.67139	1894398291
11378	Diptera	Culicidae	Aedes	Aedes aegypti	SPECIES	21.05397	-98.50990	3031967910
11379	Diptera	Culicidae	Aedes	Aedes aegypti	SPECIES	21.05413	-98.50935	3031587936
11380	Diptera	Culicidae	Aedes	Aedes aegypti	SPECIES	21.05439	-98.50657	2580078992
11381	Diptera	Culicidae	Aedes	Aedes aegypti	SPECIES	21.05448	-98.50809	2580125076
11382	Diptera	Culicidae	Aedes	Aedes aegypti	SPECIES	21.05467	-98.50863	3031955896
11383	Diptera	Culicidae	Aedes	Aedes aegypti	SPECIES	21.05483	-98.50900	3031651929
11384	Diptera	Culicidae	Aedes	Aedes aegypti	SPECIES	21.05485	-98.50776	3031807915
11385	Diptera	Culicidae	Aedes	Aedes aegypti	SPECIES	21.05492	-98.50870	3031835935
11386	Diptera	Culicidae	Aedes	Aedes aegypti	SPECIES	21.05514	-98.50962	3031944873
11387	Diptera	Culicidae	Aedes	Aedes aegypti	SPECIES	21.05516	-98.50772	3873275374
11388	Diptera	Culicidae	Aedes	Aedes aegypti	SPECIES	21.05518	-98.50875	3873245382
11389	Diptera	Culicidae	Aedes	Aedes aegypti	SPECIES	21.05523	-98.50652	3872971399
11390	Diptera	Culicidae	Aedes	Aedes aegypti	SPECIES	21.05523	-98.50803	3031604873
11391	Diptera	Culicidae	Aedes	Aedes aegypti	SPECIES	21.05531	-98.50775	3031867853
11392	Diptera	Culicidae	Aedes	Aedes aegypti	SPECIES	21.05536	-98.50802	3031669902
11393	Diptera	Culicidae	Aedes	Aedes aegypti	SPECIES	21.05557	-98.50843	3031651931
11394	Diptera	Culicidae	Aedes	Aedes aegypti	SPECIES	21.05569	-98.50691	3031935883
11395	Diptera	Culicidae	Aedes	Aedes aegypti	SPECIES	21.05569	-98.50687	3873720407
11396	Diptera	Culicidae	Aedes	Aedes aegypti	SPECIES	21.05571	-98.50682	3031953876
11397	Diptera	Culicidae	Aedes	Aedes aegypti	SPECIES	21.05583	-98.50736	3031613889
11398	Diptera	Culicidae	Aedes	Aedes aegypti	SPECIES	21.05603	-98.50515	3872997393

№	Order	Family	Genus	Species	Taxon Rank	Latitude	Longitude	GBIF_ID
11399	Diptera	Culicidae	Aedes	Aedes aegypti	SPECIES	21.05604	-98.50759	3873007375
11400	Diptera	Culicidae	Aedes	Aedes aegypti	SPECIES	21.05606	-98.50731	3873506374
11401	Diptera	Culicidae	Aedes	Aedes aegypti	SPECIES	21.05612	-98.50720	3031803865
11402	Diptera	Culicidae	Aedes	Aedes aegypti	SPECIES	21.05619	-98.50538	3031869920
11403	Diptera	Culicidae	Aedes	Aedes aegypti	SPECIES	21.05620	-98.50569	3873270358
11404	Diptera	Culicidae	Aedes	Aedes aegypti	SPECIES	21.05623	-98.50559	3031596928
11405	Diptera	Culicidae	Aedes	Aedes aegypti	SPECIES	21.05630	-98.50694	3031576921
11406	Diptera	Culicidae	Aedes	Aedes aegypti	SPECIES	21.05633	-98.50531	3031838916
11407	Diptera	Culicidae	Aedes	Aedes aegypti	SPECIES	21.05641	-98.50543	3873520347
11408	Diptera	Culicidae	Aedes	Aedes aegypti	SPECIES	21.05642	-98.50629	3031577889
11409	Diptera	Culicidae	Aedes	Aedes aegypti	SPECIES	21.05644	-98.50538	3031652944
11410	Diptera	Culicidae	Aedes	Aedes aegypti	SPECIES	21.05670	-98.50659	3031618946
11411	Diptera	Culicidae	Aedes	Aedes aegypti	SPECIES	21.05679	-98.50604	3031655884
11412	Diptera	Culicidae	Aedes	Aedes aegypti	SPECIES	21.05682	-98.50626	3872969378
11413	Diptera	Culicidae	Aedes	Aedes aegypti	SPECIES	21.05691	-98.50576	3872915381
11414	Diptera	Culicidae	Aedes	Aedes aegypti	SPECIES	21.05750	-99.80250	1894400211
11415	Diptera	Culicidae	Aedes	Aedes aegypti	SPECIES	21.05778	-101.63611	1894253206
11416	Diptera	Culicidae	Aedes	Aedes aegypti	SPECIES	21.05807	-98.50385	2580261274
11417	Diptera	Culicidae	Aedes	Aedes aegypti	SPECIES	21.05817	-98.50400	3031589873
11418	Diptera	Culicidae	Aedes	Aedes aegypti	SPECIES	21.05836	-98.50391	3031600929
11419	Diptera	Culicidae	Aedes	Aedes aegypti	SPECIES	21.05845	-98.50443	3873722379
11420	Diptera	Culicidae	Aedes	Aedes aegypti	SPECIES	21.05915	-98.50142	3031850878
11421	Diptera	Culicidae	Aedes	Aedes aegypti	SPECIES	21.05917	-101.67583	1894255722
11422	Diptera	Culicidae	Aedes	Aedes aegypti	SPECIES	21.05944	-101.40444	1894254977
11423	Diptera	Culicidae	Aedes	Aedes aegypti	SPECIES	21.05964	-98.50251	3873311393
11424	Diptera	Culicidae	Aedes	Aedes aegypti	SPECIES	21.05972	-98.49472	1894398443
11425	Diptera	Culicidae	Aedes	Aedes aegypti	SPECIES	21.06021	-98.50152	3873232383
11426	Diptera	Culicidae	Aedes	Aedes aegypti	SPECIES	21.06030	-98.34502	3873048351
11427	Diptera	Culicidae	Aedes	Aedes aegypti	SPECIES	21.06472	-100.60611	1894254516
11428	Diptera	Culicidae	Aedes	Aedes aegypti	SPECIES	21.06806	-98.47500	1894399184
11429	Diptera	Culicidae	Aedes	Aedes aegypti	SPECIES	21.07111	-98.50000	1894400264
11430	Diptera	Culicidae	Aedes	Aedes aegypti	SPECIES	21.07167	-100.60528	1894253814
11431	Diptera	Culicidae	Aedes	Aedes aegypti	SPECIES	21.07167	-101.54722	1894254911
11432	Diptera	Culicidae	Aedes	Aedes aegypti	SPECIES	21.07333	-98.95444	1894395245
11433	Diptera	Culicidae	Aedes	Aedes aegypti	SPECIES	21.07361	-101.37528	1894252716
11434	Diptera	Culicidae	Aedes	Aedes aegypti	SPECIES	21.07361	-101.72083	1894252944
11435	Diptera	Culicidae	Aedes	Aedes aegypti	SPECIES	21.07389	-101.47278	1894255839
11436	Diptera	Culicidae	Aedes	Aedes aegypti	SPECIES	21.07463	-101.33573	4165358118
11437	Diptera	Culicidae	Aedes	Aedes aegypti	SPECIES	21.07750	-100.15944	1894253241
11438	Diptera	Culicidae	Aedes	Aedes aegypti	SPECIES	21.07944	-87.01417	1421473645
11439	Diptera	Culicidae	Aedes	Aedes aegypti	SPECIES	21.08000	105.45000	1264889048

№	Order	Family	Genus	Species	Taxon Rank	Latitude	Longitude	GBIF_ID
11440	Diptera	Culicidae	Aedes	Aedes aegypti	SPECIES	21.08167	-101.86944	1894252560
11441	Diptera	Culicidae	Aedes	Aedes aegypti	SPECIES	21.08194	-98.94778	1894397654
11442	Diptera	Culicidae	Aedes	Aedes aegypti	SPECIES	21.08472	-101.55361	1894254119
11443	Diptera	Culicidae	Aedes	Aedes aegypti	SPECIES	21.08611	-101.51972	1894254122
11444	Diptera	Culicidae	Aedes	Aedes aegypti	SPECIES	21.08821	-89.29054	2629263499
11445	Diptera	Culicidae	Aedes	Aedes aegypti	SPECIES	21.09000	-89.29000	1264886282
11446	Diptera	Culicidae	Aedes	Aedes aegypti	SPECIES	21.09179	-98.21109	2860176630
11447	Diptera	Culicidae	Aedes	Aedes aegypti	SPECIES	21.09306	-98.52750	1894398978
11448	Diptera	Culicidae	Aedes	Aedes aegypti	SPECIES	21.09443	-98.75843	1894397376
11449	Diptera	Culicidae	Aedes	Aedes aegypti	SPECIES	21.09500	-89.28306	1894398148
11450	Diptera	Culicidae	Aedes	Aedes aegypti	SPECIES	21.09528	-98.49389	1894397684
11451	Diptera	Culicidae	Aedes	Aedes aegypti	SPECIES	21.09639	-98.66222	1894396960
11452	Diptera	Culicidae	Aedes	Aedes aegypti	SPECIES	21.09758	-98.21984	3031855933
11453	Diptera	Culicidae	Aedes	Aedes aegypti	SPECIES	21.09772	-98.22018	3031856920
11454	Diptera	Culicidae	Aedes	Aedes aegypti	SPECIES	21.09787	-98.21999	3031638894
11455	Diptera	Culicidae	Aedes	Aedes aegypti	SPECIES	21.09805	-98.22129	3031953878
11456	Diptera	Culicidae	Aedes	Aedes aegypti	SPECIES	21.09808	-98.21963	3031963891
11457	Diptera	Culicidae	Aedes	Aedes aegypti	SPECIES	21.09809	-98.22388	3031651930
11458	Diptera	Culicidae	Aedes	Aedes aegypti	SPECIES	21.09815	-98.22375	3031873903
11459	Diptera	Culicidae	Aedes	Aedes aegypti	SPECIES	21.09833	-98.22110	3031587905
11460	Diptera	Culicidae	Aedes	Aedes aegypti	SPECIES	21.09840	-98.22382	3031815924
11461	Diptera	Culicidae	Aedes	Aedes aegypti	SPECIES	21.09850	-98.22144	3031811894
11462	Diptera	Culicidae	Aedes	Aedes aegypti	SPECIES	21.09889	-98.38306	1894397406
11463	Diptera	Culicidae	Aedes	Aedes aegypti	SPECIES	21.09915	-98.22282	3031904889
11464	Diptera	Culicidae	Aedes	Aedes aegypti	SPECIES	21.09927	-98.22281	3031939889
11465	Diptera	Culicidae	Aedes	Aedes aegypti	SPECIES	21.09929	-98.22111	3031671886
11466	Diptera	Culicidae	Aedes	Aedes aegypti	SPECIES	21.09942	-98.22348	3031893905
11467	Diptera	Culicidae	Aedes	Aedes aegypti	SPECIES	21.10000	-87.48667	1421477012
11468	Diptera	Culicidae	Aedes	Aedes aegypti	SPECIES	21.10006	-98.22120	3031879910
11469	Diptera	Culicidae	Aedes	Aedes aegypti	SPECIES	21.10012	-98.21850	3031796937
11470	Diptera	Culicidae	Aedes	Aedes aegypti	SPECIES	21.10021	-98.22246	3031950890
11471	Diptera	Culicidae	Aedes	Aedes aegypti	SPECIES	21.10028	-98.22293	3031585895
11472	Diptera	Culicidae	Aedes	Aedes aegypti	SPECIES	21.10111	-100.15778	1894249837
11473	Diptera	Culicidae	Aedes	Aedes aegypti	SPECIES	21.10116	-98.22122	3031576880
11474	Diptera	Culicidae	Aedes	Aedes aegypti	SPECIES	21.10120	-98.22291	3031956913
11475	Diptera	Culicidae	Aedes	Aedes aegypti	SPECIES	21.10177	-98.48820	1894399906
11476	Diptera	Culicidae	Aedes	Aedes aegypti	SPECIES	21.10278	-98.58389	1894397106
11477	Diptera	Culicidae	Aedes	Aedes aegypti	SPECIES	21.10308	-101.64244	4153624161
11478	Diptera	Culicidae	Aedes	Aedes aegypti	SPECIES	21.10333	-98.50000	1894396894
11479	Diptera	Culicidae	Aedes	Aedes aegypti	SPECIES	21.10360	-98.58593	1894394905
11480	Diptera	Culicidae	Aedes	Aedes aegypti	SPECIES	21.10500	-98.96917	1894398963

№	Order	Family	Genus	Species	Taxon Rank	Latitude	Longitude	GBIF_ID
11481	Diptera	Culicidae	Aedes	Aedes aegypti	SPECIES	21.10556	-101.55083	1894255860
11482	Diptera	Culicidae	Aedes	Aedes aegypti	SPECIES	21.10694	-101.78528	1894249942
11483	Diptera	Culicidae	Aedes	Aedes aegypti	SPECIES	21.10694	-98.89667	1894394989
11484	Diptera	Culicidae	Aedes	Aedes aegypti	SPECIES	21.11000	-98.46111	1894399235
11485	Diptera	Culicidae	Aedes	Aedes aegypti	SPECIES	21.11111	-98.42889	1894396887
11486	Diptera	Culicidae	Aedes	Aedes aegypti	SPECIES	21.11134	-98.49863	2580137097
11487	Diptera	Culicidae	Aedes	Aedes aegypti	SPECIES	21.11333	-98.67694	1894400282
11488	Diptera	Culicidae	Aedes	Aedes aegypti	SPECIES	21.11472	-98.62444	1894396490
11489	Diptera	Culicidae	Aedes	Aedes aegypti	SPECIES	21.11500	-98.92056	1894395603
11490	Diptera	Culicidae	Aedes	Aedes aegypti	SPECIES	21.11528	-98.93667	1894397820
11491	Diptera	Culicidae	Aedes	Aedes aegypti	SPECIES	21.11667	-98.42722	1894395772
11492	Diptera	Culicidae	Aedes	Aedes aegypti	SPECIES	21.11667	-98.51722	1894398163
11493	Diptera	Culicidae	Aedes	Aedes aegypti	SPECIES	21.11830	-101.67123	4153791278
11494	Diptera	Culicidae	Aedes	Aedes aegypti	SPECIES	21.11861	-101.68083	1894400013
11495	Diptera	Culicidae	Aedes	Aedes aegypti	SPECIES	21.11889	-98.59500	1894395410
11496	Diptera	Culicidae	Aedes	Aedes aegypti	SPECIES	21.11917	-98.47556	1894399694
11497	Diptera	Culicidae	Aedes	Aedes aegypti	SPECIES	21.11972	-101.68056	1894255981
11498	Diptera	Culicidae	Aedes	Aedes aegypti	SPECIES	21.12056	-98.48639	1894397438
11499	Diptera	Culicidae	Aedes	Aedes aegypti	SPECIES	21.12111	-100.05167	1894252254
11500	Diptera	Culicidae	Aedes	Aedes aegypti	SPECIES	21.12122	-101.68809	4165424749
11501	Diptera	Culicidae	Aedes	Aedes aegypti	SPECIES	21.12218	-98.54301	3031904833
11502	Diptera	Culicidae	Aedes	Aedes aegypti	SPECIES	21.12221	-98.54312	3872948392
11503	Diptera	Culicidae	Aedes	Aedes aegypti	SPECIES	21.12245	-98.54245	3031626956
11504	Diptera	Culicidae	Aedes	Aedes aegypti	SPECIES	21.12288	-98.54269	3873349410
11505	Diptera	Culicidae	Aedes	Aedes aegypti	SPECIES	21.12288	-98.54256	3031961896
11506	Diptera	Culicidae	Aedes	Aedes aegypti	SPECIES	21.12306	-98.47972	1894396809
11507	Diptera	Culicidae	Aedes	Aedes aegypti	SPECIES	21.12361	-100.01278	1894395645
11508	Diptera	Culicidae	Aedes	Aedes aegypti	SPECIES	21.12401	-98.54425	3031647821
11509	Diptera	Culicidae	Aedes	Aedes aegypti	SPECIES	21.12403	-98.54247	3031935894
11510	Diptera	Culicidae	Aedes	Aedes aegypti	SPECIES	21.12417	-98.98583	1894399203
11511	Diptera	Culicidae	Aedes	Aedes aegypti	SPECIES	21.12429	-98.54326	3873352347
11512	Diptera	Culicidae	Aedes	Aedes aegypti	SPECIES	21.12441	-98.54387	3031644829
11513	Diptera	Culicidae	Aedes	Aedes aegypti	SPECIES	21.12447	-98.54303	3031862939
11514	Diptera	Culicidae	Aedes	Aedes aegypti	SPECIES	21.12472	-98.45972	1894399362
11515	Diptera	Culicidae	Aedes	Aedes aegypti	SPECIES	21.12477	-98.41116	3031607919
11516	Diptera	Culicidae	Aedes	Aedes aegypti	SPECIES	21.12501	-98.54165	3031965862
11517	Diptera	Culicidae	Aedes	Aedes aegypti	SPECIES	21.12526	-98.54156	3031817903
11518	Diptera	Culicidae	Aedes	Aedes aegypti	SPECIES	21.12531	-98.54281	3031664939
11519	Diptera	Culicidae	Aedes	Aedes aegypti	SPECIES	21.12533	-98.54165	3031953846
11520	Diptera	Culicidae	Aedes	Aedes aegypti	SPECIES	21.12542	-98.54289	3031959911
11521	Diptera	Culicidae	Aedes	Aedes aegypti	SPECIES	21.12554	-98.41253	3031962952

№	Order	Family	Genus	Species	Taxon Rank	Latitude	Longitude	GBIF_ID
11522	Diptera	Culicidae	Aedes	Aedes aegypti	SPECIES	21.12568	-98.54252	3031878897
11523	Diptera	Culicidae	Aedes	Aedes aegypti	SPECIES	21.12574	-98.54250	3031597847
11524	Diptera	Culicidae	Aedes	Aedes aegypti	SPECIES	21.12579	-98.54115	3873056408
11525	Diptera	Culicidae	Aedes	Aedes aegypti	SPECIES	21.12594	-98.41270	3031660951
11526	Diptera	Culicidae	Aedes	Aedes aegypti	SPECIES	21.12601	-98.41169	3031899995
11527	Diptera	Culicidae	Aedes	Aedes aegypti	SPECIES	21.12631	-98.54082	3031621869
11528	Diptera	Culicidae	Aedes	Aedes aegypti	SPECIES	21.12657	-98.41227	3031597972
11529	Diptera	Culicidae	Aedes	Aedes aegypti	SPECIES	21.12658	-98.54143	3873680393
11530	Diptera	Culicidae	Aedes	Aedes aegypti	SPECIES	21.12667	-98.65083	1894394522
11531	Diptera	Culicidae	Aedes	Aedes aegypti	SPECIES	21.12667	-98.41972	1894399911
11532	Diptera	Culicidae	Aedes	Aedes aegypti	SPECIES	21.12689	-98.54233	3031838915
11533	Diptera	Culicidae	Aedes	Aedes aegypti	SPECIES	21.12705	-98.54206	3031670908
11534	Diptera	Culicidae	Aedes	Aedes aegypti	SPECIES	21.12714	-98.54252	3031935895
11535	Diptera	Culicidae	Aedes	Aedes aegypti	SPECIES	21.12717	-98.54246	3031869919
11536	Diptera	Culicidae	Aedes	Aedes aegypti	SPECIES	21.12725	-98.54393	3031603894
11537	Diptera	Culicidae	Aedes	Aedes aegypti	SPECIES	21.12762	-98.41722	3031633939
11538	Diptera	Culicidae	Aedes	Aedes aegypti	SPECIES	21.12778	-88.89056	1894399940
11539	Diptera	Culicidae	Aedes	Aedes aegypti	SPECIES	21.12779	-98.54251	3031866892
11540	Diptera	Culicidae	Aedes	Aedes aegypti	SPECIES	21.12781	-98.54172	3031848939
11541	Diptera	Culicidae	Aedes	Aedes aegypti	SPECIES	21.12800	-98.54360	3873592347
11542	Diptera	Culicidae	Aedes	Aedes aegypti	SPECIES	21.12806	-101.05806	1894255747
11543	Diptera	Culicidae	Aedes	Aedes aegypti	SPECIES	21.12806	-98.39599	3031671858
11544	Diptera	Culicidae	Aedes	Aedes aegypti	SPECIES	21.12807	-98.39571	3031962871
11545	Diptera	Culicidae	Aedes	Aedes aegypti	SPECIES	21.12812	-98.39588	3031597874
11546	Diptera	Culicidae	Aedes	Aedes aegypti	SPECIES	21.12813	-98.41823	3031823978
11547	Diptera	Culicidae	Aedes	Aedes aegypti	SPECIES	21.12814	-98.54260	3031674925
11548	Diptera	Culicidae	Aedes	Aedes aegypti	SPECIES	21.12819	-98.41920	3031830929
11549	Diptera	Culicidae	Aedes	Aedes aegypti	SPECIES	21.12822	-98.54325	3031834921
11550	Diptera	Culicidae	Aedes	Aedes aegypti	SPECIES	21.12834	-98.54146	3872877360
11551	Diptera	Culicidae	Aedes	Aedes aegypti	SPECIES	21.12843	-98.41867	3031601973
11552	Diptera	Culicidae	Aedes	Aedes aegypti	SPECIES	21.12847	-98.41871	3031868982
11553	Diptera	Culicidae	Aedes	Aedes aegypti	SPECIES	21.12850	-98.54146	3031803888
11554	Diptera	Culicidae	Aedes	Aedes aegypti	SPECIES	21.12852	-98.53900	3872967352
11555	Diptera	Culicidae	Aedes	Aedes aegypti	SPECIES	21.12855	-98.54157	2579361566
11556	Diptera	Culicidae	Aedes	Aedes aegypti	SPECIES	21.12870	-98.54133	3873592346
11557	Diptera	Culicidae	Aedes	Aedes aegypti	SPECIES	21.12870	-98.53997	3031848866
11558	Diptera	Culicidae	Aedes	Aedes aegypti	SPECIES	21.12888	-98.54108	3873502400
11559	Diptera	Culicidae	Aedes	Aedes aegypti	SPECIES	21.12899	-98.53946	2580203420
11560	Diptera	Culicidae	Aedes	Aedes aegypti	SPECIES	21.12903	-98.54122	3873450405
11561	Diptera	Culicidae	Aedes	Aedes aegypti	SPECIES	21.12945	-98.54365	3031594959
11562	Diptera	Culicidae	Aedes	Aedes aegypti	SPECIES	21.12950	-98.41057	3031863945

№	Order	Family	Genus	Species	Taxon Rank	Latitude	Longitude	GBIF_ID
11563	Diptera	Culicidae	Aedes	Aedes aegypti	SPECIES	21.12970	-98.53967	3031966909
11564	Diptera	Culicidae	Aedes	Aedes aegypti	SPECIES	21.12996	-98.41159	2580110205
11565	Diptera	Culicidae	Aedes	Aedes aegypti	SPECIES	21.12997	-98.41107	3031855983
11566	Diptera	Culicidae	Aedes	Aedes aegypti	SPECIES	21.12999	-98.53966	3031950898
11567	Diptera	Culicidae	Aedes	Aedes aegypti	SPECIES	21.13000	-86.82000	1264888042
11568	Diptera	Culicidae	Aedes	Aedes aegypti	SPECIES	21.13000	-86.81000	1264888716
11569	Diptera	Culicidae	Aedes	Aedes aegypti	SPECIES	21.13003	-98.54153	3031575906
11570	Diptera	Culicidae	Aedes	Aedes aegypti	SPECIES	21.13006	-98.54001	3872909349
11571	Diptera	Culicidae	Aedes	Aedes aegypti	SPECIES	21.13017	-98.41162	3031966954
11572	Diptera	Culicidae	Aedes	Aedes aegypti	SPECIES	21.13017	-98.54158	3873052398
11573	Diptera	Culicidae	Aedes	Aedes aegypti	SPECIES	21.13032	-98.54069	3031590954
11574	Diptera	Culicidae	Aedes	Aedes aegypti	SPECIES	21.13041	-98.54010	3031644881
11575	Diptera	Culicidae	Aedes	Aedes aegypti	SPECIES	21.13042	-98.40915	2579268823
11576	Diptera	Culicidae	Aedes	Aedes aegypti	SPECIES	21.13044	-98.40932	3031817886
11577	Diptera	Culicidae	Aedes	Aedes aegypti	SPECIES	21.13045	-98.40844	3031819943
11578	Diptera	Culicidae	Aedes	Aedes aegypti	SPECIES	21.13049	-98.41007	3031579932
11579	Diptera	Culicidae	Aedes	Aedes aegypti	SPECIES	21.13053	-98.54144	3873349408
11580	Diptera	Culicidae	Aedes	Aedes aegypti	SPECIES	21.13054	-98.53849	3031638888
11581	Diptera	Culicidae	Aedes	Aedes aegypti	SPECIES	21.13056	-98.53852	3031590907
11582	Diptera	Culicidae	Aedes	Aedes aegypti	SPECIES	21.13063	-98.54052	3873256364
11583	Diptera	Culicidae	Aedes	Aedes aegypti	SPECIES	21.13068	-98.53711	3873328395
11584	Diptera	Culicidae	Aedes	Aedes aegypti	SPECIES	21.13069	-98.54007	3872888390
11585	Diptera	Culicidae	Aedes	Aedes aegypti	SPECIES	21.13071	-98.53716	3873488355
11586	Diptera	Culicidae	Aedes	Aedes aegypti	SPECIES	21.13082	-98.53825	3031826975
11587	Diptera	Culicidae	Aedes	Aedes aegypti	SPECIES	21.13084	-98.53853	3031598868
11588	Diptera	Culicidae	Aedes	Aedes aegypti	SPECIES	21.13089	-98.39647	3031954901
11589	Diptera	Culicidae	Aedes	Aedes aegypti	SPECIES	21.13090	-98.41230	2580135192
11590	Diptera	Culicidae	Aedes	Aedes aegypti	SPECIES	21.13100	-98.53939	3872933378
11591	Diptera	Culicidae	Aedes	Aedes aegypti	SPECIES	21.13104	-98.53983	3873347381
11592	Diptera	Culicidae	Aedes	Aedes aegypti	SPECIES	21.13105	-98.53980	2579377509
11593	Diptera	Culicidae	Aedes	Aedes aegypti	SPECIES	21.13106	-98.54053	3873495375
11594	Diptera	Culicidae	Aedes	Aedes aegypti	SPECIES	21.13111	-98.53826	3031654947
11595	Diptera	Culicidae	Aedes	Aedes aegypti	SPECIES	21.13111	-98.25056	1894396582
11596	Diptera	Culicidae	Aedes	Aedes aegypti	SPECIES	21.13111	-98.69056	1894398158
11597	Diptera	Culicidae	Aedes	Aedes aegypti	SPECIES	21.13111	-98.44889	1894398616
11598	Diptera	Culicidae	Aedes	Aedes aegypti	SPECIES	21.13115	-86.85289	3391698222
11599	Diptera	Culicidae	Aedes	Aedes aegypti	SPECIES	21.13118	-98.40953	3031637977
11600	Diptera	Culicidae	Aedes	Aedes aegypti	SPECIES	21.13118	-98.39630	3031626986
11601	Diptera	Culicidae	Aedes	Aedes aegypti	SPECIES	21.13120	-98.54106	3873525370
11602	Diptera	Culicidae	Aedes	Aedes aegypti	SPECIES	21.13120	-98.41063	3031586952
11603	Diptera	Culicidae	Aedes	Aedes aegypti	SPECIES	21.13130	-98.54042	3031965860

№	Order	Family	Genus	Species	Taxon Rank	Latitude	Longitude	GBIF_ID
11604	Diptera	Culicidae	Aedes	Aedes aegypti	SPECIES	21.13145	-98.54066	3031656971
11605	Diptera	Culicidae	Aedes	Aedes aegypti	SPECIES	21.13148	-98.41642	3031669927
11606	Diptera	Culicidae	Aedes	Aedes aegypti	SPECIES	21.13161	-98.41654	3031960928
11607	Diptera	Culicidae	Aedes	Aedes aegypti	SPECIES	21.13175	-98.53758	3873697387
11608	Diptera	Culicidae	Aedes	Aedes aegypti	SPECIES	21.13184	-98.39602	3031658964
11609	Diptera	Culicidae	Aedes	Aedes aegypti	SPECIES	21.13188	-98.41259	3031893949
11610	Diptera	Culicidae	Aedes	Aedes aegypti	SPECIES	21.13191	-98.53937	2579240408
11611	Diptera	Culicidae	Aedes	Aedes aegypti	SPECIES	21.13191	-98.53724	3031625874
11612	Diptera	Culicidae	Aedes	Aedes aegypti	SPECIES	21.13193	-98.41685	3031953898
11613	Diptera	Culicidae	Aedes	Aedes aegypti	SPECIES	21.13194	-98.55222	1894399215
11614	Diptera	Culicidae	Aedes	Aedes aegypti	SPECIES	21.13195	-98.53933	3031612896
11615	Diptera	Culicidae	Aedes	Aedes aegypti	SPECIES	21.13197	-98.54095	3031829908
11616	Diptera	Culicidae	Aedes	Aedes aegypti	SPECIES	21.13202	-98.54066	3873002399
11617	Diptera	Culicidae	Aedes	Aedes aegypti	SPECIES	21.13210	-98.54076	3031888927
11618	Diptera	Culicidae	Aedes	Aedes aegypti	SPECIES	21.13212	-98.41897	3031671953
11619	Diptera	Culicidae	Aedes	Aedes aegypti	SPECIES	21.13222	-98.53907	3031965859
11620	Diptera	Culicidae	Aedes	Aedes aegypti	SPECIES	21.13228	-98.42065	3031882936
11621	Diptera	Culicidae	Aedes	Aedes aegypti	SPECIES	21.13232	-98.42039	3031620930
11622	Diptera	Culicidae	Aedes	Aedes aegypti	SPECIES	21.13234	-98.39702	3031837906
11623	Diptera	Culicidae	Aedes	Aedes aegypti	SPECIES	21.13238	-98.40636	3031604907
11624	Diptera	Culicidae	Aedes	Aedes aegypti	SPECIES	21.13241	-98.41959	3031597984
11625	Diptera	Culicidae	Aedes	Aedes aegypti	SPECIES	21.13252	-98.54031	3031587900
11626	Diptera	Culicidae	Aedes	Aedes aegypti	SPECIES	21.13255	-98.41616	3031643972
11627	Diptera	Culicidae	Aedes	Aedes aegypti	SPECIES	21.13266	-98.40989	3031648954
11628	Diptera	Culicidae	Aedes	Aedes aegypti	SPECIES	21.13271	-98.55251	2580104097
11629	Diptera	Culicidae	Aedes	Aedes aegypti	SPECIES	21.13271	-98.55214	2580197290
11630	Diptera	Culicidae	Aedes	Aedes aegypti	SPECIES	21.13275	-98.41848	3031604926
11631	Diptera	Culicidae	Aedes	Aedes aegypti	SPECIES	21.13285	-98.41929	3031951932
11632	Diptera	Culicidae	Aedes	Aedes aegypti	SPECIES	21.13287	-98.39068	3872864349
11633	Diptera	Culicidae	Aedes	Aedes aegypti	SPECIES	21.13297	-98.40447	3031650956
11634	Diptera	Culicidae	Aedes	Aedes aegypti	SPECIES	21.13306	-98.49222	1894399427
11635	Diptera	Culicidae	Aedes	Aedes aegypti	SPECIES	21.13306	-98.53806	1894400284
11636	Diptera	Culicidae	Aedes	Aedes aegypti	SPECIES	21.13306	-98.40411	3031581912
11637	Diptera	Culicidae	Aedes	Aedes aegypti	SPECIES	21.13307	-98.54151	3031638941
11638	Diptera	Culicidae	Aedes	Aedes aegypti	SPECIES	21.13309	-98.55270	2580116098
11639	Diptera	Culicidae	Aedes	Aedes aegypti	SPECIES	21.13313	-98.40403	3031809850
11640	Diptera	Culicidae	Aedes	Aedes aegypti	SPECIES	21.13315	-98.40385	3031839884
11641	Diptera	Culicidae	Aedes	Aedes aegypti	SPECIES	21.13319	-98.39071	3872947359
11642	Diptera	Culicidae	Aedes	Aedes aegypti	SPECIES	21.13319	-98.54200	3031824903
11643	Diptera	Culicidae	Aedes	Aedes aegypti	SPECIES	21.13320	-98.54075	3031603874
11644	Diptera	Culicidae	Aedes	Aedes aegypti	SPECIES	21.13321	-98.54037	3031832938

№	Order	Family	Genus	Species	Taxon Rank	Latitude	Longitude	GBIF_ID
11645	Diptera	Culicidae	Aedes	Aedes aegypti	SPECIES	21.13330	-98.39579	3031843910
11646	Diptera	Culicidae	Aedes	Aedes aegypti	SPECIES	21.13332	-98.40306	3031653832
11647	Diptera	Culicidae	Aedes	Aedes aegypti	SPECIES	21.13333	-100.06083	1894250456
11648	Diptera	Culicidae	Aedes	Aedes aegypti	SPECIES	21.13343	-98.39070	3873468352
11649	Diptera	Culicidae	Aedes	Aedes aegypti	SPECIES	21.13346	-98.40206	3031959883
11650	Diptera	Culicidae	Aedes	Aedes aegypti	SPECIES	21.13347	-98.54135	3031882949
11651	Diptera	Culicidae	Aedes	Aedes aegypti	SPECIES	21.13350	-98.39160	3872975370
11652	Diptera	Culicidae	Aedes	Aedes aegypti	SPECIES	21.13350	-98.40568	3031904861
11653	Diptera	Culicidae	Aedes	Aedes aegypti	SPECIES	21.13352	-98.39032	3873717368
11654	Diptera	Culicidae	Aedes	Aedes aegypti	SPECIES	21.13356	-98.39654	3031827904
11655	Diptera	Culicidae	Aedes	Aedes aegypti	SPECIES	21.13364	-98.54046	3031598916
11656	Diptera	Culicidae	Aedes	Aedes aegypti	SPECIES	21.13374	-98.40767	3031594892
11657	Diptera	Culicidae	Aedes	Aedes aegypti	SPECIES	21.13379	-98.39586	3031803835
11658	Diptera	Culicidae	Aedes	Aedes aegypti	SPECIES	21.13384	-98.54152	2579366528
11659	Diptera	Culicidae	Aedes	Aedes aegypti	SPECIES	21.13387	-98.41055	2856686490
11660	Diptera	Culicidae	Aedes	Aedes aegypti	SPECIES	21.13389	-98.39593	3031617863
11661	Diptera	Culicidae	Aedes	Aedes aegypti	SPECIES	21.13389	-98.39573	3031846841
11662	Diptera	Culicidae	Aedes	Aedes aegypti	SPECIES	21.13389	-98.43556	1894397602
11663	Diptera	Culicidae	Aedes	Aedes aegypti	SPECIES	21.13389	-98.41187	3031670940
11664	Diptera	Culicidae	Aedes	Aedes aegypti	SPECIES	21.13399	-98.53773	2579274461
11665	Diptera	Culicidae	Aedes	Aedes aegypti	SPECIES	21.13403	-98.39768	3031952880
11666	Diptera	Culicidae	Aedes	Aedes aegypti	SPECIES	21.13407	-98.53782	3031673899
11667	Diptera	Culicidae	Aedes	Aedes aegypti	SPECIES	21.13408	-98.53922	3031614922
11668	Diptera	Culicidae	Aedes	Aedes aegypti	SPECIES	21.13414	-98.53929	3031672956
11669	Diptera	Culicidae	Aedes	Aedes aegypti	SPECIES	21.13415	-98.39748	3031618884
11670	Diptera	Culicidae	Aedes	Aedes aegypti	SPECIES	21.13422	-98.41459	2579253402
11671	Diptera	Culicidae	Aedes	Aedes aegypti	SPECIES	21.13434	-98.41440	3031965911
11672	Diptera	Culicidae	Aedes	Aedes aegypti	SPECIES	21.13442	-98.39773	3031586944
11673	Diptera	Culicidae	Aedes	Aedes aegypti	SPECIES	21.13444	-98.42774	2580097195
11674	Diptera	Culicidae	Aedes	Aedes aegypti	SPECIES	21.13444	-98.41027	2856790469
11675	Diptera	Culicidae	Aedes	Aedes aegypti	SPECIES	21.13449	-98.42793	3031643947
11676	Diptera	Culicidae	Aedes	Aedes aegypti	SPECIES	21.13450	-98.54207	3031858867
11677	Diptera	Culicidae	Aedes	Aedes aegypti	SPECIES	21.13450	-98.42797	3031900924
11678	Diptera	Culicidae	Aedes	Aedes aegypti	SPECIES	21.13460	-98.53813	3031607011
11679	Diptera	Culicidae	Aedes	Aedes aegypti	SPECIES	21.13469	-98.41035	2856783508
11680	Diptera	Culicidae	Aedes	Aedes aegypti	SPECIES	21.13469	-98.41255	3031857998
11681	Diptera	Culicidae	Aedes	Aedes aegypti	SPECIES	21.13471	-98.41143	3872987401
11682	Diptera	Culicidae	Aedes	Aedes aegypti	SPECIES	21.13473	-98.39922	3031578892
11683	Diptera	Culicidae	Aedes	Aedes aegypti	SPECIES	21.13476	-98.53752	3873232357
11684	Diptera	Culicidae	Aedes	Aedes aegypti	SPECIES	21.13477	-98.53754	3031881905
11685	Diptera	Culicidae	Aedes	Aedes aegypti	SPECIES	21.13482	-98.53749	3873230360

№	Order	Family	Genus	Species	Taxon Rank	Latitude	Longitude	GBIF_ID
11686	Diptera	Culicidae	Aedes	Aedes aegypti	SPECIES	21.13484	-98.39885	3031848835
11687	Diptera	Culicidae	Aedes	Aedes aegypti	SPECIES	21.13484	-98.53879	3031634951
11688	Diptera	Culicidae	Aedes	Aedes aegypti	SPECIES	21.13486	-98.42633	3031881948
11689	Diptera	Culicidae	Aedes	Aedes aegypti	SPECIES	21.13491	-98.39758	3031829946
11690	Diptera	Culicidae	Aedes	Aedes aegypti	SPECIES	21.13494	-98.53863	3031849869
11691	Diptera	Culicidae	Aedes	Aedes aegypti	SPECIES	21.13495	-98.42613	3031855982
11692	Diptera	Culicidae	Aedes	Aedes aegypti	SPECIES	21.13496	-98.42882	3031838949
11693	Diptera	Culicidae	Aedes	Aedes aegypti	SPECIES	21.13499	-98.42072	3031590960
11694	Diptera	Culicidae	Aedes	Aedes aegypti	SPECIES	21.13499	-98.54232	3031663046
11695	Diptera	Culicidae	Aedes	Aedes aegypti	SPECIES	21.13499	-98.41353	3031608945
11696	Diptera	Culicidae	Aedes	Aedes aegypti	SPECIES	21.13504	-98.39624	3031817934
11697	Diptera	Culicidae	Aedes	Aedes aegypti	SPECIES	21.13509	-98.53909	3031650980
11698	Diptera	Culicidae	Aedes	Aedes aegypti	SPECIES	21.13509	-98.42903	3031952904
11699	Diptera	Culicidae	Aedes	Aedes aegypti	SPECIES	21.13509	-98.41558	2579310511
11700	Diptera	Culicidae	Aedes	Aedes aegypti	SPECIES	21.13511	-98.39571	3031615890
11701	Diptera	Culicidae	Aedes	Aedes aegypti	SPECIES	21.13512	-98.41220	2856668539
11702	Diptera	Culicidae	Aedes	Aedes aegypti	SPECIES	21.13514	-98.41254	3873471396
11703	Diptera	Culicidae	Aedes	Aedes aegypti	SPECIES	21.13514	-98.53723	3031943950
11704	Diptera	Culicidae	Aedes	Aedes aegypti	SPECIES	21.13515	-98.54311	3031812888
11705	Diptera	Culicidae	Aedes	Aedes aegypti	SPECIES	21.13517	-98.39800	3031833865
11706	Diptera	Culicidae	Aedes	Aedes aegypti	SPECIES	21.13519	-98.42744	3031833860
11707	Diptera	Culicidae	Aedes	Aedes aegypti	SPECIES	21.13528	-98.42735	3872909350
11708	Diptera	Culicidae	Aedes	Aedes aegypti	SPECIES	21.13528	-98.42556	3031858909
11709	Diptera	Culicidae	Aedes	Aedes aegypti	SPECIES	21.13528	-98.42556	2580239407
11710	Diptera	Culicidae	Aedes	Aedes aegypti	SPECIES	21.13529	-98.41416	3031837998
11711	Diptera	Culicidae	Aedes	Aedes aegypti	SPECIES	21.13537	-98.42544	3031796935
11712	Diptera	Culicidae	Aedes	Aedes aegypti	SPECIES	21.13539	-98.53744	3031901864
11713	Diptera	Culicidae	Aedes	Aedes aegypti	SPECIES	21.13547	-98.41046	2856831523
11714	Diptera	Culicidae	Aedes	Aedes aegypti	SPECIES	21.13550	-98.42727	3031846891
11715	Diptera	Culicidae	Aedes	Aedes aegypti	SPECIES	21.13550	-98.40183	3031670864
11716	Diptera	Culicidae	Aedes	Aedes aegypti	SPECIES	21.13553	-98.40522	3031615920
11717	Diptera	Culicidae	Aedes	Aedes aegypti	SPECIES	21.13555	-98.42500	3873425365
11718	Diptera	Culicidae	Aedes	Aedes aegypti	SPECIES	21.13557	-98.41889	3031576947
11719	Diptera	Culicidae	Aedes	Aedes aegypti	SPECIES	21.13557	-98.40131	3031582870
11720	Diptera	Culicidae	Aedes	Aedes aegypti	SPECIES	21.13560	-98.53806	3031633900
11721	Diptera	Culicidae	Aedes	Aedes aegypti	SPECIES	21.13562	-98.42500	2580266384
11722	Diptera	Culicidae	Aedes	Aedes aegypti	SPECIES	21.13563	-98.41339	2580239414
11723	Diptera	Culicidae	Aedes	Aedes aegypti	SPECIES	21.13569	-98.40527	3031847918
11724	Diptera	Culicidae	Aedes	Aedes aegypti	SPECIES	21.13570	-98.53685	3031664860
11725	Diptera	Culicidae	Aedes	Aedes aegypti	SPECIES	21.13571	-98.53893	3031867906
11726	Diptera	Culicidae	Aedes	Aedes aegypti	SPECIES	21.13574	-98.42506	3872905380

№	Order	Family	Genus	Species	Taxon Rank	Latitude	Longitude	GBIF_ID
11727	Diptera	Culicidae	Aedes	Aedes aegypti	SPECIES	21.13575	-98.53846	3031587901
11728	Diptera	Culicidae	Aedes	Aedes aegypti	SPECIES	21.13575	-98.53854	3031649949
11729	Diptera	Culicidae	Aedes	Aedes aegypti	SPECIES	21.13575	-98.41381	2856823471
11730	Diptera	Culicidae	Aedes	Aedes aegypti	SPECIES	21.13578	-98.42496	3872946356
11731	Diptera	Culicidae	Aedes	Aedes aegypti	SPECIES	21.13579	-98.42481	3873719373
11732	Diptera	Culicidae	Aedes	Aedes aegypti	SPECIES	21.13584	-98.41363	2856727552
11733	Diptera	Culicidae	Aedes	Aedes aegypti	SPECIES	21.13586	-98.53889	3031644882
11734	Diptera	Culicidae	Aedes	Aedes aegypti	SPECIES	21.13587	-98.40995	2856819491
11735	Diptera	Culicidae	Aedes	Aedes aegypti	SPECIES	21.13590	-98.53872	3031611987
11736	Diptera	Culicidae	Aedes	Aedes aegypti	SPECIES	21.13592	-98.40060	3031603914
11737	Diptera	Culicidae	Aedes	Aedes aegypti	SPECIES	21.13595	-98.39011	3873700349
11738	Diptera	Culicidae	Aedes	Aedes aegypti	SPECIES	21.13597	-98.41880	3031957952
11739	Diptera	Culicidae	Aedes	Aedes aegypti	SPECIES	21.13599	-98.41435	2579255432
11740	Diptera	Culicidae	Aedes	Aedes aegypti	SPECIES	21.13601	-98.41566	2579380489
11741	Diptera	Culicidae	Aedes	Aedes aegypti	SPECIES	21.13606	-98.42932	2580220426
11742	Diptera	Culicidae	Aedes	Aedes aegypti	SPECIES	21.13609	-98.42967	2580140226
11743	Diptera	Culicidae	Aedes	Aedes aegypti	SPECIES	21.13612	-98.42995	2580122186
11744	Diptera	Culicidae	Aedes	Aedes aegypti	SPECIES	21.13617	-98.42529	3873307347
11745	Diptera	Culicidae	Aedes	Aedes aegypti	SPECIES	21.13618	-98.39915	3031620879
11746	Diptera	Culicidae	Aedes	Aedes aegypti	SPECIES	21.13618	-98.39914	3031972925
11747	Diptera	Culicidae	Aedes	Aedes aegypti	SPECIES	21.13625	-98.41921	3031577922
11748	Diptera	Culicidae	Aedes	Aedes aegypti	SPECIES	21.13627	-98.42653	3031846863
11749	Diptera	Culicidae	Aedes	Aedes aegypti	SPECIES	21.13628	-98.43046	3031973886
11750	Diptera	Culicidae	Aedes	Aedes aegypti	SPECIES	21.13638	-98.42571	2580118199
11751	Diptera	Culicidae	Aedes	Aedes aegypti	SPECIES	21.13639	-89.51667	1894400032
11752	Diptera	Culicidae	Aedes	Aedes aegypti	SPECIES	21.13644	-98.41612	2579362529
11753	Diptera	Culicidae	Aedes	Aedes aegypti	SPECIES	21.13645	-98.42571	2580238408
11754	Diptera	Culicidae	Aedes	Aedes aegypti	SPECIES	21.13646	-98.42470	2580183415
11755	Diptera	Culicidae	Aedes	Aedes aegypti	SPECIES	21.13647	-98.39500	3031815975
11756	Diptera	Culicidae	Aedes	Aedes aegypti	SPECIES	21.13647	-98.41180	3031816956
11757	Diptera	Culicidae	Aedes	Aedes aegypti	SPECIES	21.13657	-98.39872	3873009362
11758	Diptera	Culicidae	Aedes	Aedes aegypti	SPECIES	21.13659	-98.39539	3031664903
11759	Diptera	Culicidae	Aedes	Aedes aegypti	SPECIES	21.13665	-98.40224	3031601893
11760	Diptera	Culicidae	Aedes	Aedes aegypti	SPECIES	21.13668	-98.41253	2856820473
11761	Diptera	Culicidae	Aedes	Aedes aegypti	SPECIES	21.13684	-98.41248	3031958889
11762	Diptera	Culicidae	Aedes	Aedes aegypti	SPECIES	21.13685	-98.42178	3031807913
11763	Diptera	Culicidae	Aedes	Aedes aegypti	SPECIES	21.13687	-98.53166	3873284373
11764	Diptera	Culicidae	Aedes	Aedes aegypti	SPECIES	21.13690	-98.41421	3873471395
11765	Diptera	Culicidae	Aedes	Aedes aegypti	SPECIES	21.13692	-98.41310	3873722390
11766	Diptera	Culicidae	Aedes	Aedes aegypti	SPECIES	21.13694	-98.39497	3031640913
11767	Diptera	Culicidae	Aedes	Aedes aegypti	SPECIES	21.13694	-98.39358	3031631871

№	Order	Family	Genus	Species	Taxon Rank	Latitude	Longitude	GBIF_ID
11768	Diptera	Culicidae	Aedes	Aedes aegypti	SPECIES	21.13696	-98.39521	3031578891
11769	Diptera	Culicidae	Aedes	Aedes aegypti	SPECIES	21.13699	-98.41403	2856843536
11770	Diptera	Culicidae	Aedes	Aedes aegypti	SPECIES	21.13699	-98.41130	2856814509
11771	Diptera	Culicidae	Aedes	Aedes aegypti	SPECIES	21.13703	-98.42902	3873556344
11772	Diptera	Culicidae	Aedes	Aedes aegypti	SPECIES	21.13707	-98.40249	3872986342
11773	Diptera	Culicidae	Aedes	Aedes aegypti	SPECIES	21.13707	-98.42685	3031810887
11774	Diptera	Culicidae	Aedes	Aedes aegypti	SPECIES	21.13712	-98.53451	3873023356
11775	Diptera	Culicidae	Aedes	Aedes aegypti	SPECIES	21.13717	-98.41102	1894398561
11776	Diptera	Culicidae	Aedes	Aedes aegypti	SPECIES	21.13722	-98.42731	3031643892
11777	Diptera	Culicidae	Aedes	Aedes aegypti	SPECIES	21.13723	-98.42938	3031857936
11778	Diptera	Culicidae	Aedes	Aedes aegypti	SPECIES	21.13732	-98.42911	3031971876
11779	Diptera	Culicidae	Aedes	Aedes aegypti	SPECIES	21.13735	-98.43023	3031596889
11780	Diptera	Culicidae	Aedes	Aedes aegypti	SPECIES	21.13739	-98.39534	3031663842
11781	Diptera	Culicidae	Aedes	Aedes aegypti	SPECIES	21.13741	-98.39655	3031944879
11782	Diptera	Culicidae	Aedes	Aedes aegypti	SPECIES	21.13743	-98.41042	2580253411
11783	Diptera	Culicidae	Aedes	Aedes aegypti	SPECIES	21.13753	-98.42201	3031598860
11784	Diptera	Culicidae	Aedes	Aedes aegypti	SPECIES	21.13753	-98.40120	3031833831
11785	Diptera	Culicidae	Aedes	Aedes aegypti	SPECIES	21.13754	-98.40777	3031650888
11786	Diptera	Culicidae	Aedes	Aedes aegypti	SPECIES	21.13754	-98.42417	3031657951
11787	Diptera	Culicidae	Aedes	Aedes aegypti	SPECIES	21.13756	-98.42196	3031869987
11788	Diptera	Culicidae	Aedes	Aedes aegypti	SPECIES	21.13763	-98.39641	3031669835
11789	Diptera	Culicidae	Aedes	Aedes aegypti	SPECIES	21.13767	-98.41496	2856723455
11790	Diptera	Culicidae	Aedes	Aedes aegypti	SPECIES	21.13770	-98.39432	3031646890
11791	Diptera	Culicidae	Aedes	Aedes aegypti	SPECIES	21.13772	-98.41470	2856612488
11792	Diptera	Culicidae	Aedes	Aedes aegypti	SPECIES	21.13773	-98.40034	3031645976
11793	Diptera	Culicidae	Aedes	Aedes aegypti	SPECIES	21.13773	-98.42767	2580135752
11794	Diptera	Culicidae	Aedes	Aedes aegypti	SPECIES	21.13774	-98.40270	3873660357
11795	Diptera	Culicidae	Aedes	Aedes aegypti	SPECIES	21.13774	-98.42773	3031893897
11796	Diptera	Culicidae	Aedes	Aedes aegypti	SPECIES	21.13778	-98.40308	3031592859
11797	Diptera	Culicidae	Aedes	Aedes aegypti	SPECIES	21.13787	-98.41481	2580107198
11798	Diptera	Culicidae	Aedes	Aedes aegypti	SPECIES	21.13791	-98.43008	3031618962
11799	Diptera	Culicidae	Aedes	Aedes aegypti	SPECIES	21.13792	-98.39673	2576414059
11800	Diptera	Culicidae	Aedes	Aedes aegypti	SPECIES	21.13792	-98.53523	2579291512
11801	Diptera	Culicidae	Aedes	Aedes aegypti	SPECIES	21.13793	-98.53360	3031964961
11802	Diptera	Culicidae	Aedes	Aedes aegypti	SPECIES	21.13796	-98.39647	3031661904
11803	Diptera	Culicidae	Aedes	Aedes aegypti	SPECIES	21.13800	-98.40941	3031647924
11804	Diptera	Culicidae	Aedes	Aedes aegypti	SPECIES	21.13802	-98.42933	3031611998
11805	Diptera	Culicidae	Aedes	Aedes aegypti	SPECIES	21.13803	-98.42329	3031869986
11806	Diptera	Culicidae	Aedes	Aedes aegypti	SPECIES	21.13815	-98.40303	2859893601
11807	Diptera	Culicidae	Aedes	Aedes aegypti	SPECIES	21.13816	-98.42299	3873310350
11808	Diptera	Culicidae	Aedes	Aedes aegypti	SPECIES	21.13821	-98.41503	2856638462

№	Order	Family	Genus	Species	Taxon Rank	Latitude	Longitude	GBIF_ID
11809	Diptera	Culicidae	Aedes	Aedes aegypti	SPECIES	21.13823	-98.53561	3031623945
11810	Diptera	Culicidae	Aedes	Aedes aegypti	SPECIES	21.13827	-98.42973	3031835968
11811	Diptera	Culicidae	Aedes	Aedes aegypti	SPECIES	21.13831	-98.53623	3031881906
11812	Diptera	Culicidae	Aedes	Aedes aegypti	SPECIES	21.13832	-98.42286	3031882948
11813	Diptera	Culicidae	Aedes	Aedes aegypti	SPECIES	21.13834	-98.41520	2856577493
11814	Diptera	Culicidae	Aedes	Aedes aegypti	SPECIES	21.13834	-98.41248	3031587937
11815	Diptera	Culicidae	Aedes	Aedes aegypti	SPECIES	21.13835	-98.41536	2856588486
11816	Diptera	Culicidae	Aedes	Aedes aegypti	SPECIES	21.13846	-98.41958	3031834985
11817	Diptera	Culicidae	Aedes	Aedes aegypti	SPECIES	21.13849	-98.53506	3872913401
11818	Diptera	Culicidae	Aedes	Aedes aegypti	SPECIES	21.13856	-98.42859	3031580928
11819	Diptera	Culicidae	Aedes	Aedes aegypti	SPECIES	21.13856	-98.41176	3031951914
11820	Diptera	Culicidae	Aedes	Aedes aegypti	SPECIES	21.13858	-98.41257	3031674952
11821	Diptera	Culicidae	Aedes	Aedes aegypti	SPECIES	21.13859	-98.39999	3872941354
11822	Diptera	Culicidae	Aedes	Aedes aegypti	SPECIES	21.13864	-98.39996	3031632855
11823	Diptera	Culicidae	Aedes	Aedes aegypti	SPECIES	21.13867	-98.53376	3031601982
11824	Diptera	Culicidae	Aedes	Aedes aegypti	SPECIES	21.13870	-98.41489	3031848856
11825	Diptera	Culicidae	Aedes	Aedes aegypti	SPECIES	21.13879	-98.53373	3031867862
11826	Diptera	Culicidae	Aedes	Aedes aegypti	SPECIES	21.13882	-98.42868	3031581864
11827	Diptera	Culicidae	Aedes	Aedes aegypti	SPECIES	21.13886	-98.42814	3031588923
11828	Diptera	Culicidae	Aedes	Aedes aegypti	SPECIES	21.13887	-98.53532	3031863004
11829	Diptera	Culicidae	Aedes	Aedes aegypti	SPECIES	21.13889	-98.85278	1894399316
11830	Diptera	Culicidae	Aedes	Aedes aegypti	SPECIES	21.13896	-98.53374	3031601928
11831	Diptera	Culicidae	Aedes	Aedes aegypti	SPECIES	21.13896	-98.40048	3873609349
11832	Diptera	Culicidae	Aedes	Aedes aegypti	SPECIES	21.13909	-98.53380	3031898953
11833	Diptera	Culicidae	Aedes	Aedes aegypti	SPECIES	21.13916	-98.40924	3873021352
11834	Diptera	Culicidae	Aedes	Aedes aegypti	SPECIES	21.13917	-100.93083	1894255869
11835	Diptera	Culicidae	Aedes	Aedes aegypti	SPECIES	21.13920	-98.41692	3031644959
11836	Diptera	Culicidae	Aedes	Aedes aegypti	SPECIES	21.13920	-98.53255	3031898996
11837	Diptera	Culicidae	Aedes	Aedes aegypti	SPECIES	21.13927	-98.42687	3031964899
11838	Diptera	Culicidae	Aedes	Aedes aegypti	SPECIES	21.13932	-98.41508	3031632927
11839	Diptera	Culicidae	Aedes	Aedes aegypti	SPECIES	21.13951	-98.41504	3031844904
11840	Diptera	Culicidae	Aedes	Aedes aegypti	SPECIES	21.13952	-98.41503	3031869885
11841	Diptera	Culicidae	Aedes	Aedes aegypti	SPECIES	21.13956	-98.53487	3031963885
11842	Diptera	Culicidae	Aedes	Aedes aegypti	SPECIES	21.13959	-98.53345	3873480403
11843	Diptera	Culicidae	Aedes	Aedes aegypti	SPECIES	21.13960	-98.42730	3031587979
11844	Diptera	Culicidae	Aedes	Aedes aegypti	SPECIES	21.13963	-98.53307	3031962903
11845	Diptera	Culicidae	Aedes	Aedes aegypti	SPECIES	21.13967	-98.40052	3031672943
11846	Diptera	Culicidae	Aedes	Aedes aegypti	SPECIES	21.13968	-98.42186	3031598873
11847	Diptera	Culicidae	Aedes	Aedes aegypti	SPECIES	21.13970	-98.42035	3031807851
11848	Diptera	Culicidae	Aedes	Aedes aegypti	SPECIES	21.13970	-98.53499	3031664859
11849	Diptera	Culicidae	Aedes	Aedes aegypti	SPECIES	21.14000	-86.88000	1264887076

№	Order	Family	Genus	Species	Taxon Rank	Latitude	Longitude	GBIF_ID
11850	Diptera	Culicidae	Aedes	Aedes aegypti	SPECIES	21.14004	-98.40953	3873232351
11851	Diptera	Culicidae	Aedes	Aedes aegypti	SPECIES	21.14013	-98.53482	3873520389
11852	Diptera	Culicidae	Aedes	Aedes aegypti	SPECIES	21.14028	-98.42000	1894400178
11853	Diptera	Culicidae	Aedes	Aedes aegypti	SPECIES	21.14031	-98.41402	3031617938
11854	Diptera	Culicidae	Aedes	Aedes aegypti	SPECIES	21.14035	-98.42721	2603290816
11855	Diptera	Culicidae	Aedes	Aedes aegypti	SPECIES	21.14035	-98.41088	3031657856
11856	Diptera	Culicidae	Aedes	Aedes aegypti	SPECIES	21.14039	-98.53259	3031878954
11857	Diptera	Culicidae	Aedes	Aedes aegypti	SPECIES	21.14047	-98.53129	3031823915
11858	Diptera	Culicidae	Aedes	Aedes aegypti	SPECIES	21.14049	-98.42903	3031673951
11859	Diptera	Culicidae	Aedes	Aedes aegypti	SPECIES	21.14049	-98.52958	3873722388
11860	Diptera	Culicidae	Aedes	Aedes aegypti	SPECIES	21.14050	-98.52926	3031848865
11861	Diptera	Culicidae	Aedes	Aedes aegypti	SPECIES	21.14053	-98.52919	3031856882
11862	Diptera	Culicidae	Aedes	Aedes aegypti	SPECIES	21.14068	-98.41238	3031609951
11863	Diptera	Culicidae	Aedes	Aedes aegypti	SPECIES	21.14082	-98.42003	3031810835
11864	Diptera	Culicidae	Aedes	Aedes aegypti	SPECIES	21.14092	-98.42686	2603339841
11865	Diptera	Culicidae	Aedes	Aedes aegypti	SPECIES	21.14100	-98.53608	3031968878
11866	Diptera	Culicidae	Aedes	Aedes aegypti	SPECIES	21.14103	-98.42707	3873409349
11867	Diptera	Culicidae	Aedes	Aedes aegypti	SPECIES	21.14103	-98.42864	3031578985
11868	Diptera	Culicidae	Aedes	Aedes aegypti	SPECIES	21.14109	-98.42333	3031673950
11869	Diptera	Culicidae	Aedes	Aedes aegypti	SPECIES	21.14122	-98.42107	3031635841
11870	Diptera	Culicidae	Aedes	Aedes aegypti	SPECIES	21.14130	-98.41879	3031815998
11871	Diptera	Culicidae	Aedes	Aedes aegypti	SPECIES	21.14140	-98.41877	3031580990
11872	Diptera	Culicidae	Aedes	Aedes aegypti	SPECIES	21.14145	-98.42167	2580136212
11873	Diptera	Culicidae	Aedes	Aedes aegypti	SPECIES	21.14174	-98.42559	3031639903
11874	Diptera	Culicidae	Aedes	Aedes aegypti	SPECIES	21.14181	-98.41876	3031617911
11875	Diptera	Culicidae	Aedes	Aedes aegypti	SPECIES	21.14195	-98.41635	3031957931
11876	Diptera	Culicidae	Aedes	Aedes aegypti	SPECIES	21.14199	-98.41883	3031816858
11877	Diptera	Culicidae	Aedes	Aedes aegypti	SPECIES	21.14214	-98.41079	3031950923
11878	Diptera	Culicidae	Aedes	Aedes aegypti	SPECIES	21.14225	-98.42508	2603458126
11879	Diptera	Culicidae	Aedes	Aedes aegypti	SPECIES	21.14227	-98.42492	3031656908
11880	Diptera	Culicidae	Aedes	Aedes aegypti	SPECIES	21.14228	-98.40696	3031808917
11881	Diptera	Culicidae	Aedes	Aedes aegypti	SPECIES	21.14236	-98.42422	3031630845
11882	Diptera	Culicidae	Aedes	Aedes aegypti	SPECIES	21.14257	-86.88026	2629264858
11883	Diptera	Culicidae	Aedes	Aedes aegypti	SPECIES	21.14264	-98.42379	3031668847
11884	Diptera	Culicidae	Aedes	Aedes aegypti	SPECIES	21.14266	-98.42672	2603325830
11885	Diptera	Culicidae	Aedes	Aedes aegypti	SPECIES	21.14266	-98.42210	3031595878
11886	Diptera	Culicidae	Aedes	Aedes aegypti	SPECIES	21.14272	-98.42740	3031834971
11887	Diptera	Culicidae	Aedes	Aedes aegypti	SPECIES	21.14273	-98.42172	3031671892
11888	Diptera	Culicidae	Aedes	Aedes aegypti	SPECIES	21.14282	-98.42746	3031853932
11889	Diptera	Culicidae	Aedes	Aedes aegypti	SPECIES	21.14282	-98.42561	2603307837
11890	Diptera	Culicidae	Aedes	Aedes aegypti	SPECIES	21.14288	-98.42065	3031829911

№	Order	Family	Genus	Species	Taxon Rank	Latitude	Longitude	GBIF_ID
11891	Diptera	Culicidae	Aedes	Aedes aegypti	SPECIES	21.14291	-98.42434	3031828988
11892	Diptera	Culicidae	Aedes	Aedes aegypti	SPECIES	21.14299	-98.40717	3031955939
11893	Diptera	Culicidae	Aedes	Aedes aegypti	SPECIES	21.14301	-98.40664	3031827982
11894	Diptera	Culicidae	Aedes	Aedes aegypti	SPECIES	21.14303	-98.41877	3031645947
11895	Diptera	Culicidae	Aedes	Aedes aegypti	SPECIES	21.14306	-98.42191	2580260411
11896	Diptera	Culicidae	Aedes	Aedes aegypti	SPECIES	21.14307	-98.42832	3031833822
11897	Diptera	Culicidae	Aedes	Aedes aegypti	SPECIES	21.14319	-98.40990	3031796936
11898	Diptera	Culicidae	Aedes	Aedes aegypti	SPECIES	21.14335	-98.41916	3031636880
11899	Diptera	Culicidae	Aedes	Aedes aegypti	SPECIES	21.14338	-98.41905	3031592954
11900	Diptera	Culicidae	Aedes	Aedes aegypti	SPECIES	21.14346	-98.41190	3873492373
11901	Diptera	Culicidae	Aedes	Aedes aegypti	SPECIES	21.14347	-98.42225	3031586862
11902	Diptera	Culicidae	Aedes	Aedes aegypti	SPECIES	21.14348	-98.40664	3031868969
11903	Diptera	Culicidae	Aedes	Aedes aegypti	SPECIES	21.14353	-98.41936	3031832861
11904	Diptera	Culicidae	Aedes	Aedes aegypti	SPECIES	21.14356	-98.41174	2596260258
11905	Diptera	Culicidae	Aedes	Aedes aegypti	SPECIES	21.14375	-98.41271	2596225255
11906	Diptera	Culicidae	Aedes	Aedes aegypti	SPECIES	21.14383	-98.40392	1894395506
11907	Diptera	Culicidae	Aedes	Aedes aegypti	SPECIES	21.14400	-98.42806	3031587899
11908	Diptera	Culicidae	Aedes	Aedes aegypti	SPECIES	21.14401	-98.41020	3031869955
11909	Diptera	Culicidae	Aedes	Aedes aegypti	SPECIES	21.14402	-98.41887	3031602807
11910	Diptera	Culicidae	Aedes	Aedes aegypti	SPECIES	21.14407	-98.41874	3031838862
11911	Diptera	Culicidae	Aedes	Aedes aegypti	SPECIES	21.14409	-98.42371	3031663879
11912	Diptera	Culicidae	Aedes	Aedes aegypti	SPECIES	21.14410	-98.41871	3031657894
11913	Diptera	Culicidae	Aedes	Aedes aegypti	SPECIES	21.14411	-98.42363	3031593843
11914	Diptera	Culicidae	Aedes	Aedes aegypti	SPECIES	21.14412	-98.41915	3031828946
11915	Diptera	Culicidae	Aedes	Aedes aegypti	SPECIES	21.14417	-98.41390	3031674958
11916	Diptera	Culicidae	Aedes	Aedes aegypti	SPECIES	21.14419	-98.42349	2580109235
11917	Diptera	Culicidae	Aedes	Aedes aegypti	SPECIES	21.14419	-98.41998	3031610839
11918	Diptera	Culicidae	Aedes	Aedes aegypti	SPECIES	21.14426	-98.40643	3031663911
11919	Diptera	Culicidae	Aedes	Aedes aegypti	SPECIES	21.14434	-98.42256	3031878870
11920	Diptera	Culicidae	Aedes	Aedes aegypti	SPECIES	21.14438	-98.42260	3031605916
11921	Diptera	Culicidae	Aedes	Aedes aegypti	SPECIES	21.14455	-98.41529	3031974963
11922	Diptera	Culicidae	Aedes	Aedes aegypti	SPECIES	21.14455	-98.41991	3031605949
11923	Diptera	Culicidae	Aedes	Aedes aegypti	SPECIES	21.14456	-98.42150	3031672869
11924	Diptera	Culicidae	Aedes	Aedes aegypti	SPECIES	21.14458	-98.42105	3031641878
11925	Diptera	Culicidae	Aedes	Aedes aegypti	SPECIES	21.14461	-98.42423	3031835886
11926	Diptera	Culicidae	Aedes	Aedes aegypti	SPECIES	21.14470	-98.42317	3031965838
11927	Diptera	Culicidae	Aedes	Aedes aegypti	SPECIES	21.14474	-98.42225	3031646892
11928	Diptera	Culicidae	Aedes	Aedes aegypti	SPECIES	21.14479	-98.42800	3031639904
11929	Diptera	Culicidae	Aedes	Aedes aegypti	SPECIES	21.14487	-98.42300	3031641879
11930	Diptera	Culicidae	Aedes	Aedes aegypti	SPECIES	21.14491	-98.42712	3031858933
11931	Diptera	Culicidae	Aedes	Aedes aegypti	SPECIES	21.14495	-98.42492	3031810903

№	Order	Family	Genus	Species	Taxon Rank	Latitude	Longitude	GBIF_ID
11932	Diptera	Culicidae	Aedes	Aedes aegypti	SPECIES	21.14495	-98.42225	3031962916
11933	Diptera	Culicidae	Aedes	Aedes aegypti	SPECIES	21.14513	-98.41598	3031967913
11934	Diptera	Culicidae	Aedes	Aedes aegypti	SPECIES	21.14516	-98.39969	3873448396
11935	Diptera	Culicidae	Aedes	Aedes aegypti	SPECIES	21.14518	-98.42272	3031877872
11936	Diptera	Culicidae	Aedes	Aedes aegypti	SPECIES	21.14520	-98.42018	3031660935
11937	Diptera	Culicidae	Aedes	Aedes aegypti	SPECIES	21.14520	-98.42479	3031939861
11938	Diptera	Culicidae	Aedes	Aedes aegypti	SPECIES	21.14532	-98.42784	3031593924
11939	Diptera	Culicidae	Aedes	Aedes aegypti	SPECIES	21.14540	-98.42252	3031816953
11940	Diptera	Culicidae	Aedes	Aedes aegypti	SPECIES	21.14547	-98.42192	2580238566
11941	Diptera	Culicidae	Aedes	Aedes aegypti	SPECIES	21.14547	-98.42301	2580238565
11942	Diptera	Culicidae	Aedes	Aedes aegypti	SPECIES	21.14548	-98.42534	3031620940
11943	Diptera	Culicidae	Aedes	Aedes aegypti	SPECIES	21.14566	-98.42227	3031951912
11944	Diptera	Culicidae	Aedes	Aedes aegypti	SPECIES	21.14567	-98.42176	2580093366
11945	Diptera	Culicidae	Aedes	Aedes aegypti	SPECIES	21.14576	-98.41479	3031961918
11946	Diptera	Culicidae	Aedes	Aedes aegypti	SPECIES	21.14579	-98.42246	3031823955
11947	Diptera	Culicidae	Aedes	Aedes aegypti	SPECIES	21.14588	-98.42258	3031877834
11948	Diptera	Culicidae	Aedes	Aedes aegypti	SPECIES	21.14591	-98.41500	3031605946
11949	Diptera	Culicidae	Aedes	Aedes aegypti	SPECIES	21.14593	-98.41741	3031635943
11950	Diptera	Culicidae	Aedes	Aedes aegypti	SPECIES	21.14598	-98.41716	3873479356
11951	Diptera	Culicidae	Aedes	Aedes aegypti	SPECIES	21.14603	-98.42787	3031862936
11952	Diptera	Culicidae	Aedes	Aedes aegypti	SPECIES	21.14606	-98.42206	3031863927
11953	Diptera	Culicidae	Aedes	Aedes aegypti	SPECIES	21.14612	-98.41408	3031967917
11954	Diptera	Culicidae	Aedes	Aedes aegypti	SPECIES	21.14613	-98.42199	3031617968
11955	Diptera	Culicidae	Aedes	Aedes aegypti	SPECIES	21.14616	-98.42363	3031857939
11956	Diptera	Culicidae	Aedes	Aedes aegypti	SPECIES	21.14619	-98.41951	3031901919
11957	Diptera	Culicidae	Aedes	Aedes aegypti	SPECIES	21.14623	-98.39911	3031961881
11958	Diptera	Culicidae	Aedes	Aedes aegypti	SPECIES	21.14626	-98.42157	3031873818
11959	Diptera	Culicidae	Aedes	Aedes aegypti	SPECIES	21.14630	-98.42274	3031964844
11960	Diptera	Culicidae	Aedes	Aedes aegypti	SPECIES	21.14633	-98.39974	3031956909
11961	Diptera	Culicidae	Aedes	Aedes aegypti	SPECIES	21.14640	-98.42815	3031819982
11962	Diptera	Culicidae	Aedes	Aedes aegypti	SPECIES	21.14643	-98.42199	3031812890
11963	Diptera	Culicidae	Aedes	Aedes aegypti	SPECIES	21.14646	-98.40434	3031597960
11964	Diptera	Culicidae	Aedes	Aedes aegypti	SPECIES	21.14648	-98.43062	3031603843
11965	Diptera	Culicidae	Aedes	Aedes aegypti	SPECIES	21.14649	-98.42281	3031619855
11966	Diptera	Culicidae	Aedes	Aedes aegypti	SPECIES	21.14650	-98.42195	3031585842
11967	Diptera	Culicidae	Aedes	Aedes aegypti	SPECIES	21.14650	-98.42212	3031882881
11968	Diptera	Culicidae	Aedes	Aedes aegypti	SPECIES	21.14654	-98.42788	2580140231
11969	Diptera	Culicidae	Aedes	Aedes aegypti	SPECIES	21.14656	-98.42184	3031972951
11970	Diptera	Culicidae	Aedes	Aedes aegypti	SPECIES	21.14657	-98.42727	3031644888
11971	Diptera	Culicidae	Aedes	Aedes aegypti	SPECIES	21.14660	-98.42215	2580200569
11972	Diptera	Culicidae	Aedes	Aedes aegypti	SPECIES	21.14661	-98.42791	2580096226

№	Order	Family	Genus	Species	Taxon Rank	Latitude	Longitude	GBIF_ID
11973	Diptera	Culicidae	Aedes	Aedes aegypti	SPECIES	21.14661	-98.42733	3031944880
11974	Diptera	Culicidae	Aedes	Aedes aegypti	SPECIES	21.14661	-98.42667	3031669836
11975	Diptera	Culicidae	Aedes	Aedes aegypti	SPECIES	21.14662	-98.42120	3031935951
11976	Diptera	Culicidae	Aedes	Aedes aegypti	SPECIES	21.14663	-98.42760	3031615859
11977	Diptera	Culicidae	Aedes	Aedes aegypti	SPECIES	21.14672	-98.42253	2580081262
11978	Diptera	Culicidae	Aedes	Aedes aegypti	SPECIES	21.14676	-98.42712	3031821889
11979	Diptera	Culicidae	Aedes	Aedes aegypti	SPECIES	21.14679	-98.41803	3873287370
11980	Diptera	Culicidae	Aedes	Aedes aegypti	SPECIES	21.14685	-98.39934	3031877898
11981	Diptera	Culicidae	Aedes	Aedes aegypti	SPECIES	21.14689	-98.41964	3031863944
11982	Diptera	Culicidae	Aedes	Aedes aegypti	SPECIES	21.14693	-98.42850	3031594893
11983	Diptera	Culicidae	Aedes	Aedes aegypti	SPECIES	21.14697	-98.42870	3031625832
11984	Diptera	Culicidae	Aedes	Aedes aegypti	SPECIES	21.14702	-98.40384	3031591957
11985	Diptera	Culicidae	Aedes	Aedes aegypti	SPECIES	21.14705	-98.40528	3031808906
11986	Diptera	Culicidae	Aedes	Aedes aegypti	SPECIES	21.14707	-98.42917	2580256404
11987	Diptera	Culicidae	Aedes	Aedes aegypti	SPECIES	21.14711	-98.41896	3872907378
11988	Diptera	Culicidae	Aedes	Aedes aegypti	SPECIES	21.14713	-98.41641	3031577893
11989	Diptera	Culicidae	Aedes	Aedes aegypti	SPECIES	21.14719	-98.42705	3031577856
11990	Diptera	Culicidae	Aedes	Aedes aegypti	SPECIES	21.14724	-98.42161	3031577891
11991	Diptera	Culicidae	Aedes	Aedes aegypti	SPECIES	21.14726	-98.40085	3872950394
11992	Diptera	Culicidae	Aedes	Aedes aegypti	SPECIES	21.14729	-98.42210	3031837935
11993	Diptera	Culicidae	Aedes	Aedes aegypti	SPECIES	21.14735	-98.42675	3031935922
11994	Diptera	Culicidae	Aedes	Aedes aegypti	SPECIES	21.14736	-98.42703	3031868950
11995	Diptera	Culicidae	Aedes	Aedes aegypti	SPECIES	21.14738	-98.41678	3031580966
11996	Diptera	Culicidae	Aedes	Aedes aegypti	SPECIES	21.14738	-98.41775	3031665915
11997	Diptera	Culicidae	Aedes	Aedes aegypti	SPECIES	21.14740	-98.42728	3031954898
11998	Diptera	Culicidae	Aedes	Aedes aegypti	SPECIES	21.14741	-98.40200	3031637927
11999	Diptera	Culicidae	Aedes	Aedes aegypti	SPECIES	21.14742	-98.40119	3873500380
12000	Diptera	Culicidae	Aedes	Aedes aegypti	SPECIES	21.14743	-98.39986	3031834906
12001	Diptera	Culicidae	Aedes	Aedes aegypti	SPECIES	21.14744	-98.40334	3873714373
12002	Diptera	Culicidae	Aedes	Aedes aegypti	SPECIES	21.14745	-98.40338	3031901865
12003	Diptera	Culicidae	Aedes	Aedes aegypti	SPECIES	21.14745	-98.40168	3873309392
12004	Diptera	Culicidae	Aedes	Aedes aegypti	SPECIES	21.14754	-98.42734	3031578964
12005	Diptera	Culicidae	Aedes	Aedes aegypti	SPECIES	21.14760	-98.40367	3031881907
12006	Diptera	Culicidae	Aedes	Aedes aegypti	SPECIES	21.14764	-98.40518	3873478355
12007	Diptera	Culicidae	Aedes	Aedes aegypti	SPECIES	21.14764	-98.40614	3031815970
12008	Diptera	Culicidae	Aedes	Aedes aegypti	SPECIES	21.14767	-98.41929	3031829944
12009	Diptera	Culicidae	Aedes	Aedes aegypti	SPECIES	21.14767	-98.41780	3031612880
12010	Diptera	Culicidae	Aedes	Aedes aegypti	SPECIES	21.14768	-98.40411	3031622953
12011	Diptera	Culicidae	Aedes	Aedes aegypti	SPECIES	21.14779	-98.42222	1894395261
12012	Diptera	Culicidae	Aedes	Aedes aegypti	SPECIES	21.14785	-98.41631	3031606977
12013	Diptera	Culicidae	Aedes	Aedes aegypti	SPECIES	21.14785	-98.41623	3873505364

№	Order	Family	Genus	Species	Taxon Rank	Latitude	Longitude	GBIF_ID
12014	Diptera	Culicidae	Aedes	Aedes aegypti	SPECIES	21.14792	-98.40717	2596229285
12015	Diptera	Culicidae	Aedes	Aedes aegypti	SPECIES	21.14795	-98.42978	3031807916
12016	Diptera	Culicidae	Aedes	Aedes aegypti	SPECIES	21.14805	-98.40721	3031963921
12017	Diptera	Culicidae	Aedes	Aedes aegypti	SPECIES	21.14806	-98.41656	3031581906
12018	Diptera	Culicidae	Aedes	Aedes aegypti	SPECIES	21.14813	-98.41653	3031839947
12019	Diptera	Culicidae	Aedes	Aedes aegypti	SPECIES	21.14816	-98.42990	3031877833
12020	Diptera	Culicidae	Aedes	Aedes aegypti	SPECIES	21.14820	-98.40731	3031960911
12021	Diptera	Culicidae	Aedes	Aedes aegypti	SPECIES	21.14820	-98.41757	3873012358
12022	Diptera	Culicidae	Aedes	Aedes aegypti	SPECIES	21.14824	-98.43144	3031616859
12023	Diptera	Culicidae	Aedes	Aedes aegypti	SPECIES	21.14825	-98.43056	3031808862
12024	Diptera	Culicidae	Aedes	Aedes aegypti	SPECIES	21.14825	-98.43149	2580100295
12025	Diptera	Culicidae	Aedes	Aedes aegypti	SPECIES	21.14833	-102.68889	1894397553
12026	Diptera	Culicidae	Aedes	Aedes aegypti	SPECIES	21.14834	-98.40728	2596238252
12027	Diptera	Culicidae	Aedes	Aedes aegypti	SPECIES	21.14838	-98.40680	3031848897
12028	Diptera	Culicidae	Aedes	Aedes aegypti	SPECIES	21.14841	-98.41659	2580104222
12029	Diptera	Culicidae	Aedes	Aedes aegypti	SPECIES	21.14847	-98.43103	3031859942
12030	Diptera	Culicidae	Aedes	Aedes aegypti	SPECIES	21.14850	-98.41714	3031576927
12031	Diptera	Culicidae	Aedes	Aedes aegypti	SPECIES	21.14861	-101.76194	1894255254
12032	Diptera	Culicidae	Aedes	Aedes aegypti	SPECIES	21.14864	-98.43122	3031959980
12033	Diptera	Culicidae	Aedes	Aedes aegypti	SPECIES	21.14867	-98.42785	3031614934
12034	Diptera	Culicidae	Aedes	Aedes aegypti	SPECIES	21.14879	-98.43082	2580081229
12035	Diptera	Culicidae	Aedes	Aedes aegypti	SPECIES	21.14889	-98.40256	3031900885
12036	Diptera	Culicidae	Aedes	Aedes aegypti	SPECIES	21.14909	-98.42682	3031874970
12037	Diptera	Culicidae	Aedes	Aedes aegypti	SPECIES	21.14918	-98.42984	3031659926
12038	Diptera	Culicidae	Aedes	Aedes aegypti	SPECIES	21.14929	-98.41680	3031874938
12039	Diptera	Culicidae	Aedes	Aedes aegypti	SPECIES	21.14939	-98.42203	3031853904
12040	Diptera	Culicidae	Aedes	Aedes aegypti	SPECIES	21.14942	-98.40102	3031612844
12041	Diptera	Culicidae	Aedes	Aedes aegypti	SPECIES	21.14953	-98.41645	3031966943
12042	Diptera	Culicidae	Aedes	Aedes aegypti	SPECIES	21.14960	-98.43031	3031667898
12043	Diptera	Culicidae	Aedes	Aedes aegypti	SPECIES	21.14965	-98.43034	3031654982
12044	Diptera	Culicidae	Aedes	Aedes aegypti	SPECIES	21.14965	-98.41671	3031659896
12045	Diptera	Culicidae	Aedes	Aedes aegypti	SPECIES	21.14972	-98.43064	3031628952
12046	Diptera	Culicidae	Aedes	Aedes aegypti	SPECIES	21.14972	-101.60750	1894251736
12047	Diptera	Culicidae	Aedes	Aedes aegypti	SPECIES	21.14975	-98.42167	2580078287
12048	Diptera	Culicidae	Aedes	Aedes aegypti	SPECIES	21.14989	-98.43026	2580088317
12049	Diptera	Culicidae	Aedes	Aedes aegypti	SPECIES	21.14992	-98.43056	3031607013
12050	Diptera	Culicidae	Aedes	Aedes aegypti	SPECIES	21.14995	-98.43053	2580263564
12051	Diptera	Culicidae	Aedes	Aedes aegypti	SPECIES	21.15000	-86.89000	1264886030
12052	Diptera	Culicidae	Aedes	Aedes aegypti	SPECIES	21.15000	-88.15000	1264886229
12053	Diptera	Culicidae	Aedes	Aedes aegypti	SPECIES	21.15000	-86.88000	1264887077
12054	Diptera	Culicidae	Aedes	Aedes aegypti	SPECIES	21.15000	-98.36500	1894400309

№	Order	Family	Genus	Species	Taxon Rank	Latitude	Longitude	GBIF_ID
12055	Diptera	Culicidae	Aedes	Aedes aegypti	SPECIES	21.15003	-98.43060	2580092331
12056	Diptera	Culicidae	Aedes	Aedes aegypti	SPECIES	21.15003	-98.41671	3031810926
12057	Diptera	Culicidae	Aedes	Aedes aegypti	SPECIES	21.15007	-98.43076	3031575981
12058	Diptera	Culicidae	Aedes	Aedes aegypti	SPECIES	21.15007	-98.40780	3031962940
12059	Diptera	Culicidae	Aedes	Aedes aegypti	SPECIES	21.15019	-98.43081	3031972913
12060	Diptera	Culicidae	Aedes	Aedes aegypti	SPECIES	21.15019	-98.39924	3031651889
12061	Diptera	Culicidae	Aedes	Aedes aegypti	SPECIES	21.15021	-98.40135	3031881949
12062	Diptera	Culicidae	Aedes	Aedes aegypti	SPECIES	21.15024	-98.42235	3031838948
12063	Diptera	Culicidae	Aedes	Aedes aegypti	SPECIES	21.15029	-98.42480	3031650999
12064	Diptera	Culicidae	Aedes	Aedes aegypti	SPECIES	21.15044	-98.38469	3873638387
12065	Diptera	Culicidae	Aedes	Aedes aegypti	SPECIES	21.15045	-98.39554	3031846865
12066	Diptera	Culicidae	Aedes	Aedes aegypti	SPECIES	21.15056	-98.34528	1894399455
12067	Diptera	Culicidae	Aedes	Aedes aegypti	SPECIES	21.15061	-98.38647	3031671881
12068	Diptera	Culicidae	Aedes	Aedes aegypti	SPECIES	21.15063	-98.38382	3872945379
12069	Diptera	Culicidae	Aedes	Aedes aegypti	SPECIES	21.15063	-98.38643	3873722380
12070	Diptera	Culicidae	Aedes	Aedes aegypti	SPECIES	21.15069	-98.42576	2580267561
12071	Diptera	Culicidae	Aedes	Aedes aegypti	SPECIES	21.15080	-98.42428	1894395843
12072	Diptera	Culicidae	Aedes	Aedes aegypti	SPECIES	21.15098	-98.42854	3031617893
12073	Diptera	Culicidae	Aedes	Aedes aegypti	SPECIES	21.15102	-98.43007	3031869990
12074	Diptera	Culicidae	Aedes	Aedes aegypti	SPECIES	21.15107	-98.42546	3031644960
12075	Diptera	Culicidae	Aedes	Aedes aegypti	SPECIES	21.15107	-98.38329	3872942373
12076	Diptera	Culicidae	Aedes	Aedes aegypti	SPECIES	21.15109	-98.43013	3031843943
12077	Diptera	Culicidae	Aedes	Aedes aegypti	SPECIES	21.15109	-98.42854	2580105400
12078	Diptera	Culicidae	Aedes	Aedes aegypti	SPECIES	21.15114	-98.38426	3873230382
12079	Diptera	Culicidae	Aedes	Aedes aegypti	SPECIES	21.15117	-98.42539	3031851943
12080	Diptera	Culicidae	Aedes	Aedes aegypti	SPECIES	21.15117	-98.38195	3873447391
12081	Diptera	Culicidae	Aedes	Aedes aegypti	SPECIES	21.15121	-98.42821	3031935950
12082	Diptera	Culicidae	Aedes	Aedes aegypti	SPECIES	21.15134	-98.42414	3031674910
12083	Diptera	Culicidae	Aedes	Aedes aegypti	SPECIES	21.15136	-98.39579	3031803870
12084	Diptera	Culicidae	Aedes	Aedes aegypti	SPECIES	21.15137	-98.39614	3031607869
12085	Diptera	Culicidae	Aedes	Aedes aegypti	SPECIES	21.15137	-98.38251	3872883380
12086	Diptera	Culicidae	Aedes	Aedes aegypti	SPECIES	21.15138	-98.42824	3031838022
12087	Diptera	Culicidae	Aedes	Aedes aegypti	SPECIES	21.15140	-98.38648	3031633894
12088	Diptera	Culicidae	Aedes	Aedes aegypti	SPECIES	21.15144	-98.42488	3031657880
12089	Diptera	Culicidae	Aedes	Aedes aegypti	SPECIES	21.15172	-98.38330	3873721395
12090	Diptera	Culicidae	Aedes	Aedes aegypti	SPECIES	21.15175	-98.39750	3031575894
12091	Diptera	Culicidae	Aedes	Aedes aegypti	SPECIES	21.15176	-98.39557	3031899948
12092	Diptera	Culicidae	Aedes	Aedes aegypti	SPECIES	21.15183	-98.39615	3873260381
12093	Diptera	Culicidae	Aedes	Aedes aegypti	SPECIES	21.15183	-98.39507	3031646878
12094	Diptera	Culicidae	Aedes	Aedes aegypti	SPECIES	21.15184	-98.38271	3031648902
12095	Diptera	Culicidae	Aedes	Aedes aegypti	SPECIES	21.15187	-98.38218	3031853908

№	Order	Family	Genus	Species	Taxon Rank	Latitude	Longitude	GBIF_ID
12096	Diptera	Culicidae	Aedes	Aedes aegypti	SPECIES	21.15191	-98.38416	3031943899
12097	Diptera	Culicidae	Aedes	Aedes aegypti	SPECIES	21.15192	-98.39682	3031655898
12098	Diptera	Culicidae	Aedes	Aedes aegypti	SPECIES	21.15192	-98.42473	3031595940
12099	Diptera	Culicidae	Aedes	Aedes aegypti	SPECIES	21.15193	-98.39472	3031637926
12100	Diptera	Culicidae	Aedes	Aedes aegypti	SPECIES	21.15194	-100.07472	1894249675
12101	Diptera	Culicidae	Aedes	Aedes aegypti	SPECIES	21.15194	-98.38611	1894394797
12102	Diptera	Culicidae	Aedes	Aedes aegypti	SPECIES	21.15194	-98.52444	1894400243
12103	Diptera	Culicidae	Aedes	Aedes aegypti	SPECIES	21.15198	-98.39429	3031584910
12104	Diptera	Culicidae	Aedes	Aedes aegypti	SPECIES	21.15198	-98.39451	3031585890
12105	Diptera	Culicidae	Aedes	Aedes aegypti	SPECIES	21.15200	-98.39466	3872894383
12106	Diptera	Culicidae	Aedes	Aedes aegypti	SPECIES	21.15201	-98.38267	3031836894
12107	Diptera	Culicidae	Aedes	Aedes aegypti	SPECIES	21.15211	-98.42383	3031615881
12108	Diptera	Culicidae	Aedes	Aedes aegypti	SPECIES	21.15212	-98.39716	3031957898
12109	Diptera	Culicidae	Aedes	Aedes aegypti	SPECIES	21.15218	-98.38412	3873226385
12110	Diptera	Culicidae	Aedes	Aedes aegypti	SPECIES	21.15220	-98.41042	3031809933
12111	Diptera	Culicidae	Aedes	Aedes aegypti	SPECIES	21.15222	-98.38500	3031627877
12112	Diptera	Culicidae	Aedes	Aedes aegypti	SPECIES	21.15229	-98.38247	3031638889
12113	Diptera	Culicidae	Aedes	Aedes aegypti	SPECIES	21.15229	-98.38393	3031674922
12114	Diptera	Culicidae	Aedes	Aedes aegypti	SPECIES	21.15229	-98.38227	3031970976
12115	Diptera	Culicidae	Aedes	Aedes aegypti	SPECIES	21.15249	-98.38236	3031581875
12116	Diptera	Culicidae	Aedes	Aedes aegypti	SPECIES	21.15250	-98.42526	3031644883
12117	Diptera	Culicidae	Aedes	Aedes aegypti	SPECIES	21.15250	-101.75889	1894253175
12118	Diptera	Culicidae	Aedes	Aedes aegypti	SPECIES	21.15250	-98.42451	3031819939
12119	Diptera	Culicidae	Aedes	Aedes aegypti	SPECIES	21.15252	-98.38433	3031581924
12120	Diptera	Culicidae	Aedes	Aedes aegypti	SPECIES	21.15254	-98.41190	2580197397
12121	Diptera	Culicidae	Aedes	Aedes aegypti	SPECIES	21.15256	-98.41210	3031972956
12122	Diptera	Culicidae	Aedes	Aedes aegypti	SPECIES	21.15261	-98.38275	3031576926
12123	Diptera	Culicidae	Aedes	Aedes aegypti	SPECIES	21.15261	-98.38321	3031957932
12124	Diptera	Culicidae	Aedes	Aedes aegypti	SPECIES	21.15263	-98.38320	3031634900
12125	Diptera	Culicidae	Aedes	Aedes aegypti	SPECIES	21.15264	-98.41288	3031824945
12126	Diptera	Culicidae	Aedes	Aedes aegypti	SPECIES	21.15278	-101.77833	1894252096
12127	Diptera	Culicidae	Aedes	Aedes aegypti	SPECIES	21.15281	-98.39654	3031968891
12128	Diptera	Culicidae	Aedes	Aedes aegypti	SPECIES	21.15282	-98.41080	2580113198
12129	Diptera	Culicidae	Aedes	Aedes aegypti	SPECIES	21.15283	-98.38246	3031819946
12130	Diptera	Culicidae	Aedes	Aedes aegypti	SPECIES	21.15286	-98.40166	3031657916
12131	Diptera	Culicidae	Aedes	Aedes aegypti	SPECIES	21.15294	-98.42416	2580104360
12132	Diptera	Culicidae	Aedes	Aedes aegypti	SPECIES	21.15297	-98.51907	3031599883
12133	Diptera	Culicidae	Aedes	Aedes aegypti	SPECIES	21.15299	-98.41304	3031859923
12134	Diptera	Culicidae	Aedes	Aedes aegypti	SPECIES	21.15316	-98.42446	3031654933
12135	Diptera	Culicidae	Aedes	Aedes aegypti	SPECIES	21.15319	-98.42454	3031624899
12136	Diptera	Culicidae	Aedes	Aedes aegypti	SPECIES	21.15333	-98.38889	1894398010

№	Order	Family	Genus	Species	Taxon Rank	Latitude	Longitude	GBIF_ID
12137	Diptera	Culicidae	Aedes	Aedes aegypti	SPECIES	21.15333	-98.79944	1894399084
12138	Diptera	Culicidae	Aedes	Aedes aegypti	SPECIES	21.15371	-98.42478	3031877897
12139	Diptera	Culicidae	Aedes	Aedes aegypti	SPECIES	21.15382	-98.42470	2580089348
12140	Diptera	Culicidae	Aedes	Aedes aegypti	SPECIES	21.15388	-98.40164	3031863929
12141	Diptera	Culicidae	Aedes	Aedes aegypti	SPECIES	21.15409	-98.79701	3915505805
12142	Diptera	Culicidae	Aedes	Aedes aegypti	SPECIES	21.15491	-98.40223	3031660932
12143	Diptera	Culicidae	Aedes	Aedes aegypti	SPECIES	21.15611	-98.59444	1894399820
12144	Diptera	Culicidae	Aedes	Aedes aegypti	SPECIES	21.15662	-98.50457	3031619959
12145	Diptera	Culicidae	Aedes	Aedes aegypti	SPECIES	21.15662	-98.50586	3031608934
12146	Diptera	Culicidae	Aedes	Aedes aegypti	SPECIES	21.15672	-98.44508	3031626944
12147	Diptera	Culicidae	Aedes	Aedes aegypti	SPECIES	21.15703	-98.44499	3873352366
12148	Diptera	Culicidae	Aedes	Aedes aegypti	SPECIES	21.15721	-98.44447	3873513364
12149	Diptera	Culicidae	Aedes	Aedes aegypti	SPECIES	21.15731	-98.44392	3873577404
12150	Diptera	Culicidae	Aedes	Aedes aegypti	SPECIES	21.15750	-98.44449	3872896382
12151	Diptera	Culicidae	Aedes	Aedes aegypti	SPECIES	21.15750	-98.93389	1894399993
12152	Diptera	Culicidae	Aedes	Aedes aegypti	SPECIES	21.15769	-98.50541	3031854974
12153	Diptera	Culicidae	Aedes	Aedes aegypti	SPECIES	21.15771	-98.44465	3031611958
12154	Diptera	Culicidae	Aedes	Aedes aegypti	SPECIES	21.15778	-98.52167	1894399437
12155	Diptera	Culicidae	Aedes	Aedes aegypti	SPECIES	21.15782	-98.44482	3031583882
12156	Diptera	Culicidae	Aedes	Aedes aegypti	SPECIES	21.15783	-98.44599	3873678391
12157	Diptera	Culicidae	Aedes	Aedes aegypti	SPECIES	21.15783	-98.61920	3031640874
12158	Diptera	Culicidae	Aedes	Aedes aegypti	SPECIES	21.15785	-98.44464	3872913381
12159	Diptera	Culicidae	Aedes	Aedes aegypti	SPECIES	21.15798	-98.44311	3873053368
12160	Diptera	Culicidae	Aedes	Aedes aegypti	SPECIES	21.15802	-98.44549	3031627950
12161	Diptera	Culicidae	Aedes	Aedes aegypti	SPECIES	21.15804	-98.44722	3873362368
12162	Diptera	Culicidae	Aedes	Aedes aegypti	SPECIES	21.15805	-98.44474	3873017369
12163	Diptera	Culicidae	Aedes	Aedes aegypti	SPECIES	21.15815	-98.44579	3031880915
12164	Diptera	Culicidae	Aedes	Aedes aegypti	SPECIES	21.15817	-98.44521	3873245373
12165	Diptera	Culicidae	Aedes	Aedes aegypti	SPECIES	21.15832	-98.44695	3873721377
12166	Diptera	Culicidae	Aedes	Aedes aegypti	SPECIES	21.15861	-98.44667	1894399470
12167	Diptera	Culicidae	Aedes	Aedes aegypti	SPECIES	21.15886	-98.44548	3031657953
12168	Diptera	Culicidae	Aedes	Aedes aegypti	SPECIES	21.15889	-98.44585	3872986362
12169	Diptera	Culicidae	Aedes	Aedes aegypti	SPECIES	21.15889	-98.90250	1894399306
12170	Diptera	Culicidae	Aedes	Aedes aegypti	SPECIES	21.15893	-98.44494	3031628991
12171	Diptera	Culicidae	Aedes	Aedes aegypti	SPECIES	21.15895	-98.44718	3873658377
12172	Diptera	Culicidae	Aedes	Aedes aegypti	SPECIES	21.15898	-98.44691	3031900948
12173	Diptera	Culicidae	Aedes	Aedes aegypti	SPECIES	21.15922	-98.41654	2580258403
12174	Diptera	Culicidae	Aedes	Aedes aegypti	SPECIES	21.15978	-98.41840	3873577369
12175	Diptera	Culicidae	Aedes	Aedes aegypti	SPECIES	21.16000	-86.85000	1264886767
12176	Diptera	Culicidae	Aedes	Aedes aegypti	SPECIES	21.16000	79.09000	1264889253
12177	Diptera	Culicidae	Aedes	Aedes aegypti	SPECIES	21.16010	-98.44666	3031618910

№	Order	Family	Genus	Species	Taxon Rank	Latitude	Longitude	GBIF_ID
12178	Diptera	Culicidae	Aedes	Aedes aegypti	SPECIES	21.16022	-98.41859	2580180411
12179	Diptera	Culicidae	Aedes	Aedes aegypti	SPECIES	21.16044	-98.42183	2580084192
12180	Diptera	Culicidae	Aedes	Aedes aegypti	SPECIES	21.16056	-86.84750	2629263126
12181	Diptera	Culicidae	Aedes	Aedes aegypti	SPECIES	21.16056	-98.44584	3873432395
12182	Diptera	Culicidae	Aedes	Aedes aegypti	SPECIES	21.16062	-98.44671	3031660974
12183	Diptera	Culicidae	Aedes	Aedes aegypti	SPECIES	21.16068	-98.44624	3872997380
12184	Diptera	Culicidae	Aedes	Aedes aegypti	SPECIES	21.16069	-98.42069	3031626977
12185	Diptera	Culicidae	Aedes	Aedes aegypti	SPECIES	21.16069	-98.42123	3031939916
12186	Diptera	Culicidae	Aedes	Aedes aegypti	SPECIES	21.16083	-98.55944	1894397556
12187	Diptera	Culicidae	Aedes	Aedes aegypti	SPECIES	21.16094	-98.41910	3873700347
12188	Diptera	Culicidae	Aedes	Aedes aegypti	SPECIES	21.16111	-86.83361	1894399894
12189	Diptera	Culicidae	Aedes	Aedes aegypti	SPECIES	21.16123	-86.85546	3415626411
12190	Diptera	Culicidae	Aedes	Aedes aegypti	SPECIES	21.16144	-98.42152	3872939378
12191	Diptera	Culicidae	Aedes	Aedes aegypti	SPECIES	21.16194	-98.31917	3872890373
12192	Diptera	Culicidae	Aedes	Aedes aegypti	SPECIES	21.16196	-98.31920	3873649389
12193	Diptera	Culicidae	Aedes	Aedes aegypti	SPECIES	21.16201	-98.42229	3872978350
12194	Diptera	Culicidae	Aedes	Aedes aegypti	SPECIES	21.16233	-98.61583	2579258816
12195	Diptera	Culicidae	Aedes	Aedes aegypti	SPECIES	21.16245	-98.31874	3873610384
12196	Diptera	Culicidae	Aedes	Aedes aegypti	SPECIES	21.16252	-98.66141	3872975369
12197	Diptera	Culicidae	Aedes	Aedes aegypti	SPECIES	21.16253	-98.31657	3031615954
12198	Diptera	Culicidae	Aedes	Aedes aegypti	SPECIES	21.16294	-98.66600	3873034352
12199	Diptera	Culicidae	Aedes	Aedes aegypti	SPECIES	21.16321	-98.31694	3031858907
12200	Diptera	Culicidae	Aedes	Aedes aegypti	SPECIES	21.16335	-98.61451	2596180899
12201	Diptera	Culicidae	Aedes	Aedes aegypti	SPECIES	21.16354	-98.31760	3873035384
12202	Diptera	Culicidae	Aedes	Aedes aegypti	SPECIES	21.16355	-98.31739	3031651956
12203	Diptera	Culicidae	Aedes	Aedes aegypti	SPECIES	21.16361	-98.61394	2579229816
12204	Diptera	Culicidae	Aedes	Aedes aegypti	SPECIES	21.16367	-98.31561	3031642906
12205	Diptera	Culicidae	Aedes	Aedes aegypti	SPECIES	21.16370	-98.31745	3031590939
12206	Diptera	Culicidae	Aedes	Aedes aegypti	SPECIES	21.16375	-98.61383	3872891371
12207	Diptera	Culicidae	Aedes	Aedes aegypti	SPECIES	21.16417	-98.99972	1894397742
12208	Diptera	Culicidae	Aedes	Aedes aegypti	SPECIES	21.16479	-98.61241	2596202862
12209	Diptera	Culicidae	Aedes	Aedes aegypti	SPECIES	21.16504	-98.61251	3873651356
12210	Diptera	Culicidae	Aedes	Aedes aegypti	SPECIES	21.16511	-98.66483	2580203315
12211	Diptera	Culicidae	Aedes	Aedes aegypti	SPECIES	21.16528	-98.48444	1894398308
12212	Diptera	Culicidae	Aedes	Aedes aegypti	SPECIES	21.16528	-98.66556	1894399947
12213	Diptera	Culicidae	Aedes	Aedes aegypti	SPECIES	21.16552	-98.66449	3873705361
12214	Diptera	Culicidae	Aedes	Aedes aegypti	SPECIES	21.16598	-98.66606	2580243306
12215	Diptera	Culicidae	Aedes	Aedes aegypti	SPECIES	21.16624	-98.48514	3031826958
12216	Diptera	Culicidae	Aedes	Aedes aegypti	SPECIES	21.16640	-98.66628	3873450358
12217	Diptera	Culicidae	Aedes	Aedes aegypti	SPECIES	21.16695	-98.60784	3031626980
12218	Diptera	Culicidae	Aedes	Aedes aegypti	SPECIES	21.16699	-98.60805	3031859879

№	Order	Family	Genus	Species	Taxon Rank	Latitude	Longitude	GBIF_ID
12219	Diptera	Culicidae	Aedes	Aedes aegypti	SPECIES	21.16700	-86.85539	3325800695
12220	Diptera	Culicidae	Aedes	Aedes aegypti	SPECIES	21.16728	-98.60868	3873236400
12221	Diptera	Culicidae	Aedes	Aedes aegypti	SPECIES	21.16748	-98.59726	2596086156
12222	Diptera	Culicidae	Aedes	Aedes aegypti	SPECIES	21.16756	-98.59700	3031854924
12223	Diptera	Culicidae	Aedes	Aedes aegypti	SPECIES	21.16761	-98.60408	3031635916
12224	Diptera	Culicidae	Aedes	Aedes aegypti	SPECIES	21.16813	-98.60539	3031617890
12225	Diptera	Culicidae	Aedes	Aedes aegypti	SPECIES	21.16817	-98.60559	3031668949
12226	Diptera	Culicidae	Aedes	Aedes aegypti	SPECIES	21.16823	-98.59760	3031594913
12227	Diptera	Culicidae	Aedes	Aedes aegypti	SPECIES	21.16829	-98.60425	3031856897
12228	Diptera	Culicidae	Aedes	Aedes aegypti	SPECIES	21.16833	-98.60455	3031826973
12229	Diptera	Culicidae	Aedes	Aedes aegypti	SPECIES	21.16833	-100.19861	1894254575
12230	Diptera	Culicidae	Aedes	Aedes aegypti	SPECIES	21.16839	-98.60424	3873309395
12231	Diptera	Culicidae	Aedes	Aedes aegypti	SPECIES	21.16842	-98.68607	3031587935
12232	Diptera	Culicidae	Aedes	Aedes aegypti	SPECIES	21.16847	-98.60471	3873302366
12233	Diptera	Culicidae	Aedes	Aedes aegypti	SPECIES	21.16861	-98.74889	1894400071
12234	Diptera	Culicidae	Aedes	Aedes aegypti	SPECIES	21.16870	-98.60272	2596251254
12235	Diptera	Culicidae	Aedes	Aedes aegypti	SPECIES	21.16873	-98.60416	3872989376
12236	Diptera	Culicidae	Aedes	Aedes aegypti	SPECIES	21.16881	-98.60625	3031630888
12237	Diptera	Culicidae	Aedes	Aedes aegypti	SPECIES	21.16885	-98.48609	3873725373
12238	Diptera	Culicidae	Aedes	Aedes aegypti	SPECIES	21.16896	-98.60373	3873608382
12239	Diptera	Culicidae	Aedes	Aedes aegypti	SPECIES	21.16902	-98.60670	3031625895
12240	Diptera	Culicidae	Aedes	Aedes aegypti	SPECIES	21.16906	-98.61050	3031674908
12241	Diptera	Culicidae	Aedes	Aedes aegypti	SPECIES	21.16913	-98.60726	3031587934
12242	Diptera	Culicidae	Aedes	Aedes aegypti	SPECIES	21.16914	-98.48574	3873275367
12243	Diptera	Culicidae	Aedes	Aedes aegypti	SPECIES	21.16925	-98.60328	3872968390
12244	Diptera	Culicidae	Aedes	Aedes aegypti	SPECIES	21.16929	-98.60373	3873695399
12245	Diptera	Culicidae	Aedes	Aedes aegypti	SPECIES	21.16941	-98.60766	3031858908
12246	Diptera	Culicidae	Aedes	Aedes aegypti	SPECIES	21.16942	-98.59752	3031618911
12247	Diptera	Culicidae	Aedes	Aedes aegypti	SPECIES	21.16947	-98.60623	3031935882
12248	Diptera	Culicidae	Aedes	Aedes aegypti	SPECIES	21.16968	-98.60432	3031959963
12249	Diptera	Culicidae	Aedes	Aedes aegypti	SPECIES	21.17000	79.66000	1264889240
12250	Diptera	Culicidae	Aedes	Aedes aegypti	SPECIES	21.17018	-98.48670	3873722389
12251	Diptera	Culicidae	Aedes	Aedes aegypti	SPECIES	21.17028	-98.78000	1894396238
12252	Diptera	Culicidae	Aedes	Aedes aegypti	SPECIES	21.17034	-98.48658	3031865923
12253	Diptera	Culicidae	Aedes	Aedes aegypti	SPECIES	21.17048	-98.60626	3031970922
12254	Diptera	Culicidae	Aedes	Aedes aegypti	SPECIES	21.17079	-98.60370	3031651887
12255	Diptera	Culicidae	Aedes	Aedes aegypti	SPECIES	21.17086	-98.60470	3031959910
12256	Diptera	Culicidae	Aedes	Aedes aegypti	SPECIES	21.17096	-98.48644	3031660931
12257	Diptera	Culicidae	Aedes	Aedes aegypti	SPECIES	21.17098	-98.48670	3031900923
12258	Diptera	Culicidae	Aedes	Aedes aegypti	SPECIES	21.17099	-98.60630	3031625912
12259	Diptera	Culicidae	Aedes	Aedes aegypti	SPECIES	21.17103	-98.48811	3031604874

№	Order	Family	Genus	Species	Taxon Rank	Latitude	Longitude	GBIF_ID
12260	Diptera	Culicidae	Aedes	Aedes aegypti	SPECIES	21.17107	-98.48638	3031618973
12261	Diptera	Culicidae	Aedes	Aedes aegypti	SPECIES	21.17111	-98.60861	1894399917
12262	Diptera	Culicidae	Aedes	Aedes aegypti	SPECIES	21.17116	-98.48605	3031858943
12263	Diptera	Culicidae	Aedes	Aedes aegypti	SPECIES	21.17139	-98.60575	3873031386
12264	Diptera	Culicidae	Aedes	Aedes aegypti	SPECIES	21.17140	-98.60582	3031901926
12265	Diptera	Culicidae	Aedes	Aedes aegypti	SPECIES	21.17144	-98.48765	3872978379
12266	Diptera	Culicidae	Aedes	Aedes aegypti	SPECIES	21.17145	-98.48841	3031672941
12267	Diptera	Culicidae	Aedes	Aedes aegypti	SPECIES	21.17149	-98.48736	3031864932
12268	Diptera	Culicidae	Aedes	Aedes aegypti	SPECIES	21.17154	-98.60379	3031588966
12269	Diptera	Culicidae	Aedes	Aedes aegypti	SPECIES	21.17158	-98.48730	3031810957
12270	Diptera	Culicidae	Aedes	Aedes aegypti	SPECIES	21.17158	-98.59471	3031648960
12271	Diptera	Culicidae	Aedes	Aedes aegypti	SPECIES	21.17162	-98.59350	3031827930
12272	Diptera	Culicidae	Aedes	Aedes aegypti	SPECIES	21.17169	-98.60122	2596317284
12273	Diptera	Culicidae	Aedes	Aedes aegypti	SPECIES	21.17170	-98.48626	3031640870
12274	Diptera	Culicidae	Aedes	Aedes aegypti	SPECIES	21.17174	-98.59367	3031834904
12275	Diptera	Culicidae	Aedes	Aedes aegypti	SPECIES	21.17178	-98.59400	3031803866
12276	Diptera	Culicidae	Aedes	Aedes aegypti	SPECIES	21.17189	-98.61734	3873053376
12277	Diptera	Culicidae	Aedes	Aedes aegypti	SPECIES	21.17200	-98.59498	3031846864
12278	Diptera	Culicidae	Aedes	Aedes aegypti	SPECIES	21.17204	-98.60463	3031666925
12279	Diptera	Culicidae	Aedes	Aedes aegypti	SPECIES	21.17213	-98.59557	3031592940
12280	Diptera	Culicidae	Aedes	Aedes aegypti	SPECIES	21.17218	-98.61625	3031863891
12281	Diptera	Culicidae	Aedes	Aedes aegypti	SPECIES	21.17243	-98.61247	3031623890
12282	Diptera	Culicidae	Aedes	Aedes aegypti	SPECIES	21.17261	-98.60417	3031627888
12283	Diptera	Culicidae	Aedes	Aedes aegypti	SPECIES	21.17263	-98.60699	2596265164
12284	Diptera	Culicidae	Aedes	Aedes aegypti	SPECIES	21.17265	-98.59841	3031868968
12285	Diptera	Culicidae	Aedes	Aedes aegypti	SPECIES	21.17266	-98.60790	3872872391
12286	Diptera	Culicidae	Aedes	Aedes aegypti	SPECIES	21.17269	-98.61659	3873311394
12287	Diptera	Culicidae	Aedes	Aedes aegypti	SPECIES	21.17269	-98.60824	3873448397
12288	Diptera	Culicidae	Aedes	Aedes aegypti	SPECIES	21.17269	-98.60411	3873722381
12289	Diptera	Culicidae	Aedes	Aedes aegypti	SPECIES	21.17273	-98.60552	3873725387
12290	Diptera	Culicidae	Aedes	Aedes aegypti	SPECIES	21.17274	-98.60540	2596052081
12291	Diptera	Culicidae	Aedes	Aedes aegypti	SPECIES	21.17275	-102.46561	2629265411
12292	Diptera	Culicidae	Aedes	Aedes aegypti	SPECIES	21.17278	-98.65611	1894394498
12293	Diptera	Culicidae	Aedes	Aedes aegypti	SPECIES	21.17278	-98.59583	1894396619
12294	Diptera	Culicidae	Aedes	Aedes aegypti	SPECIES	21.17283	-98.60816	3031632935
12295	Diptera	Culicidae	Aedes	Aedes aegypti	SPECIES	21.17293	-98.60672	3873495410
12296	Diptera	Culicidae	Aedes	Aedes aegypti	SPECIES	21.17298	-98.60329	3031586950
12297	Diptera	Culicidae	Aedes	Aedes aegypti	SPECIES	21.17314	-98.60507	3872928380
12298	Diptera	Culicidae	Aedes	Aedes aegypti	SPECIES	21.17315	-98.60656	3031610901
12299	Diptera	Culicidae	Aedes	Aedes aegypti	SPECIES	21.17385	-98.60038	3031965865
12300	Diptera	Culicidae	Aedes	Aedes aegypti	SPECIES	21.17386	-98.60643	3872985380

№	Order	Family	Genus	Species	Taxon Rank	Latitude	Longitude	GBIF_ID
12301	Diptera	Culicidae	Aedes	Aedes aegypti	SPECIES	21.17464	-98.60826	3031956914
12302	Diptera	Culicidae	Aedes	Aedes aegypti	SPECIES	21.17467	-98.61211	3872951352
12303	Diptera	Culicidae	Aedes	Aedes aegypti	SPECIES	21.17469	-98.60981	3031672903
12304	Diptera	Culicidae	Aedes	Aedes aegypti	SPECIES	21.17556	-98.60889	1894396484
12305	Diptera	Culicidae	Aedes	Aedes aegypti	SPECIES	21.17566	-98.59159	3031674915
12306	Diptera	Culicidae	Aedes	Aedes aegypti	SPECIES	21.17569	-98.60984	3873708382
12307	Diptera	Culicidae	Aedes	Aedes aegypti	SPECIES	21.17585	-98.60957	3872935388
12308	Diptera	Culicidae	Aedes	Aedes aegypti	SPECIES	21.17850	-101.38922	4165627096
12309	Diptera	Culicidae	Aedes	Aedes aegypti	SPECIES	21.18103	-101.64840	4145673756
12310	Diptera	Culicidae	Aedes	Aedes aegypti	SPECIES	21.18167	-98.54806	1894398205
12311	Diptera	Culicidae	Aedes	Aedes aegypti	SPECIES	21.18167	-102.89056	1894399533
12312	Diptera	Culicidae	Aedes	Aedes aegypti	SPECIES	21.18278	-98.76278	1894399816
12313	Diptera	Culicidae	Aedes	Aedes aegypti	SPECIES	21.18417	-99.04000	1894396241
12314	Diptera	Culicidae	Aedes	Aedes aegypti	SPECIES	21.18639	-101.64472	1894255599
12315	Diptera	Culicidae	Aedes	Aedes aegypti	SPECIES	21.18833	-101.68917	1894252719
12316	Diptera	Culicidae	Aedes	Aedes aegypti	SPECIES	21.19028	-86.80583	1894398809
12317	Diptera	Culicidae	Aedes	Aedes aegypti	SPECIES	21.19417	-101.49778	1894254522
12318	Diptera	Culicidae	Aedes	Aedes aegypti	SPECIES	21.19417	-99.00639	1894399743
12319	Diptera	Culicidae	Aedes	Aedes aegypti	SPECIES	21.19901	-101.77125	4162374484
12320	Diptera	Culicidae	Aedes	Aedes aegypti	SPECIES	21.19972	-98.49250	1894397922
12321	Diptera	Culicidae	Aedes	Aedes aegypti	SPECIES	21.20000	72.82000	1264889278
12322	Diptera	Culicidae	Aedes	Aedes aegypti	SPECIES	21.20278	-98.60944	1894398336
12323	Diptera	Culicidae	Aedes	Aedes aegypti	SPECIES	21.20472	-98.91556	1894397131
12324	Diptera	Culicidae	Aedes	Aedes aegypti	SPECIES	21.20500	-98.67361	1894399802
12325	Diptera	Culicidae	Aedes	Aedes aegypti	SPECIES	21.20722	-98.67194	1894400221
12326	Diptera	Culicidae	Aedes	Aedes aegypti	SPECIES	21.21000	-89.65778	1894399300
12327	Diptera	Culicidae	Aedes	Aedes aegypti	SPECIES	21.21028	-89.30889	1894396417
12328	Diptera	Culicidae	Aedes	Aedes aegypti	SPECIES	21.21222	-100.21444	1894253415
12329	Diptera	Culicidae	Aedes	Aedes aegypti	SPECIES	21.21667	-99.47250	1894395106
12330	Diptera	Culicidae	Aedes	Aedes aegypti	SPECIES	21.22000	79.20000	1264889247
12331	Diptera	Culicidae	Aedes	Aedes aegypti	SPECIES	21.22222	-99.92444	1894255386
12332	Diptera	Culicidae	Aedes	Aedes aegypti	SPECIES	21.23000	79.20000	1264889027
12333	Diptera	Culicidae	Aedes	Aedes aegypti	SPECIES	21.23056	-98.87778	1894395034
12334	Diptera	Culicidae	Aedes	Aedes aegypti	SPECIES	21.23250	-99.80250	1894252812
12335	Diptera	Culicidae	Aedes	Aedes aegypti	SPECIES	21.23250	-89.11667	1894397216
12336	Diptera	Culicidae	Aedes	Aedes aegypti	SPECIES	21.23694	-104.90056	1421477862
12337	Diptera	Culicidae	Aedes	Aedes aegypti	SPECIES	21.23750	-98.86944	1894398489
12338	Diptera	Culicidae	Aedes	Aedes aegypti	SPECIES	21.24000	-98.56250	1894398410
12339	Diptera	Culicidae	Aedes	Aedes aegypti	SPECIES	21.24056	-98.94417	1894397073
12340	Diptera	Culicidae	Aedes	Aedes aegypti	SPECIES	21.24225	-102.32197	2629262826
12341	Diptera	Culicidae	Aedes	Aedes aegypti	SPECIES	21.24333	-99.95167	1894254133

№	Order	Family	Genus	Species	Taxon Rank	Latitude	Longitude	GBIF_ID
12342	Diptera	Culicidae	Aedes	Aedes aegypti	SPECIES	21.24583	-102.33083	1894400118
12343	Diptera	Culicidae	Aedes	Aedes aegypti	SPECIES	21.24694	-98.95333	1894397617
12344	Diptera	Culicidae	Aedes	Aedes aegypti	SPECIES	21.24833	-100.27389	1894254499
12345	Diptera	Culicidae	Aedes	Aedes aegypti	SPECIES	21.24833	-98.74944	1894398373
12346	Diptera	Culicidae	Aedes	Aedes aegypti	SPECIES	21.24944	-98.77833	1894397774
12347	Diptera	Culicidae	Aedes	Aedes aegypti	SPECIES	21.25000	77.51000	1264889305
12348	Diptera	Culicidae	Aedes	Aedes aegypti	SPECIES	21.25333	-98.79472	1894399978
12349	Diptera	Culicidae	Aedes	Aedes aegypti	SPECIES	21.25472	-98.81111	1894397177
12350	Diptera	Culicidae	Aedes	Aedes aegypti	SPECIES	21.25806	-86.75167	1894400322
12351	Diptera	Culicidae	Aedes	Aedes aegypti	SPECIES	21.26056	-98.95750	1894396077
12352	Diptera	Culicidae	Aedes	Aedes aegypti	SPECIES	21.26111	-98.79167	1894399849
12353	Diptera	Culicidae	Aedes	Aedes aegypti	SPECIES	21.26472	-99.93250	1894249563
12354	Diptera	Culicidae	Aedes	Aedes aegypti	SPECIES	21.26528	-102.77694	1894398123
12355	Diptera	Culicidae	Aedes	Aedes aegypti	SPECIES	21.26778	-99.71833	1894255939
12356	Diptera	Culicidae	Aedes	Aedes aegypti	SPECIES	21.27194	-98.94972	1894400059
12357	Diptera	Culicidae	Aedes	Aedes aegypti	SPECIES	21.27278	-98.77139	1894399741
12358	Diptera	Culicidae	Aedes	Aedes aegypti	SPECIES	21.28000	-89.68000	1264886214
12359	Diptera	Culicidae	Aedes	Aedes aegypti	SPECIES	21.28064	-89.66789	2629264655
12360	Diptera	Culicidae	Aedes	Aedes aegypti	SPECIES	21.28444	-89.66333	1894399716
12361	Diptera	Culicidae	Aedes	Aedes aegypti	SPECIES	21.28833	-100.05833	1894249917
12362	Diptera	Culicidae	Aedes	Aedes aegypti	SPECIES	21.29861	-100.51639	1894254097
12363	Diptera	Culicidae	Aedes	Aedes aegypti	SPECIES	21.29972	-100.05528	1894255902
12364	Diptera	Culicidae	Aedes	Aedes aegypti	SPECIES	21.30528	-100.79472	1894254720
12365	Diptera	Culicidae	Aedes	Aedes aegypti	SPECIES	21.30944	-99.51083	1894395778
12366	Diptera	Culicidae	Aedes	Aedes aegypti	SPECIES	21.31056	-98.80750	1894398955
12367	Diptera	Culicidae	Aedes	Aedes aegypti	SPECIES	21.32333	-99.94611	1894255421
12368	Diptera	Culicidae	Aedes	Aedes aegypti	SPECIES	21.32778	-98.65944	1894398462
12369	Diptera	Culicidae	Aedes	Aedes aegypti	SPECIES	21.33250	-98.51278	1894398079
12370	Diptera	Culicidae	Aedes	Aedes aegypti	SPECIES	21.33611	-98.82694	1894399671
12371	Diptera	Culicidae	Aedes	Aedes aegypti	SPECIES	21.34000	-89.26000	1264886268
12372	Diptera	Culicidae	Aedes	Aedes aegypti	SPECIES	21.34000	39.98000	1264886911
12373	Diptera	Culicidae	Aedes	Aedes aegypti	SPECIES	21.34000	-98.23000	1264887062
12374	Diptera	Culicidae	Aedes	Aedes aegypti	SPECIES	21.34000	-98.22000	1264887391
12375	Diptera	Culicidae	Aedes	Aedes aegypti	SPECIES	21.34178	-98.21972	1421476990
12376	Diptera	Culicidae	Aedes	Aedes aegypti	SPECIES	21.34500	-98.22611	2629265299
12377	Diptera	Culicidae	Aedes	Aedes aegypti	SPECIES	21.34583	-98.63972	1894397446
12378	Diptera	Culicidae	Aedes	Aedes aegypti	SPECIES	21.34806	-100.02722	1894255351
12379	Diptera	Culicidae	Aedes	Aedes aegypti	SPECIES	21.34972	-97.68667	1894398840
12380	Diptera	Culicidae	Aedes	Aedes aegypti	SPECIES	21.35000	-98.23000	1264886799
12381	Diptera	Culicidae	Aedes	Aedes aegypti	SPECIES	21.35000	39.98000	1264886882
12382	Diptera	Culicidae	Aedes	Aedes aegypti	SPECIES	21.35000	105.28000	1264890258

№	Order	Family	Genus	Species	Taxon Rank	Latitude	Longitude	GBIF_ID
12383	Diptera	Culicidae	Aedes	Aedes aegypti	SPECIES	21.35000	85.24000	3349902212
12384	Diptera	Culicidae	Aedes	Aedes aegypti	SPECIES	21.35500	-99.94278	1894254876
12385	Diptera	Culicidae	Aedes	Aedes aegypti	SPECIES	21.35583	-101.93333	1894399765
12386	Diptera	Culicidae	Aedes	Aedes aegypti	SPECIES	21.36000	-77.71000	1264890888
12387	Diptera	Culicidae	Aedes	Aedes aegypti	SPECIES	21.36056	-98.50389	1894397225
12388	Diptera	Culicidae	Aedes	Aedes aegypti	SPECIES	21.36750	-100.06667	1894254730
12389	Diptera	Culicidae	Aedes	Aedes aegypti	SPECIES	21.37028	-98.65972	1894399481
12390	Diptera	Culicidae	Aedes	Aedes aegypti	SPECIES	21.37583	-98.55694	1894400102
12391	Diptera	Culicidae	Aedes	Aedes aegypti	SPECIES	21.37861	-98.54417	1894396635
12392	Diptera	Culicidae	Aedes	Aedes aegypti	SPECIES	21.38000	-77.91000	1264886625
12393	Diptera	Culicidae	Aedes	Aedes aegypti	SPECIES	21.38000	-77.92000	1264887156
12394	Diptera	Culicidae	Aedes	Aedes aegypti	SPECIES	21.38611	-98.99028	1894399983
12395	Diptera	Culicidae	Aedes	Aedes aegypti	SPECIES	21.39000	78.92000	1264889273
12396	Diptera	Culicidae	Aedes	Aedes aegypti	SPECIES	21.39417	-99.93556	1894254284
12397	Diptera	Culicidae	Aedes	Aedes aegypti	SPECIES	21.39500	-98.87278	1894395580
12398	Diptera	Culicidae	Aedes	Aedes aegypti	SPECIES	21.40000	75.50000	1264890728
12399	Diptera	Culicidae	Aedes	Aedes aegypti	SPECIES	21.40139	-98.72778	1894400048
12400	Diptera	Culicidae	Aedes	Aedes aegypti	SPECIES	21.40667	-100.03028	1894251589
12401	Diptera	Culicidae	Aedes	Aedes aegypti	SPECIES	21.41306	-87.70361	2629264502
12402	Diptera	Culicidae	Aedes	Aedes aegypti	SPECIES	21.42898	-71.14451	3466440321
12403	Diptera	Culicidae	Aedes	Aedes aegypti	SPECIES	21.43361	-102.57389	1894395848
12404	Diptera	Culicidae	Aedes	Aedes aegypti	SPECIES	21.43442	-105.19461	2629264289
12405	Diptera	Culicidae	Aedes	Aedes aegypti	SPECIES	21.43750	-98.87500	1894400037
12406	Diptera	Culicidae	Aedes	Aedes aegypti	SPECIES	21.43861	-100.79917	1894250423
12407	Diptera	Culicidae	Aedes	Aedes aegypti	SPECIES	21.44500	-99.63556	1894395845
12408	Diptera	Culicidae	Aedes	Aedes aegypti	SPECIES	21.44556	-99.99639	1894255913
12409	Diptera	Culicidae	Aedes	Aedes aegypti	SPECIES	21.44722	-98.90972	1894394742
12410	Diptera	Culicidae	Aedes	Aedes aegypti	SPECIES	21.46000	80.20000	1264889242
12411	Diptera	Culicidae	Aedes	Aedes aegypti	SPECIES	21.46250	-98.89444	1894399945
12412	Diptera	Culicidae	Aedes	Aedes aegypti	SPECIES	21.46556	-100.87194	1894255831
12413	Diptera	Culicidae	Aedes	Aedes aegypti	SPECIES	21.48056	-100.24000	1894254813
12414	Diptera	Culicidae	Aedes	Aedes aegypti	SPECIES	21.48333	-98.96806	1894396262
12415	Diptera	Culicidae	Aedes	Aedes aegypti	SPECIES	21.51194	-100.17806	1894252155
12416	Diptera	Culicidae	Aedes	Aedes aegypti	SPECIES	21.51197	-87.67717	2629262664
12417	Diptera	Culicidae	Aedes	Aedes aegypti	SPECIES	21.51441	-87.68544	2629265232
12418	Diptera	Culicidae	Aedes	Aedes aegypti	SPECIES	21.51456	-87.66735	3859659917
12419	Diptera	Culicidae	Aedes	Aedes aegypti	SPECIES	21.51917	-87.69278	1894400269
12420	Diptera	Culicidae	Aedes	Aedes aegypti	SPECIES	21.52417	-102.23694	1894399199
12421	Diptera	Culicidae	Aedes	Aedes aegypti	SPECIES	21.52861	-99.05694	1894396197
12422	Diptera	Culicidae	Aedes	Aedes aegypti	SPECIES	21.52972	-100.14472	1894251781
12423	Diptera	Culicidae	Aedes	Aedes aegypti	SPECIES	21.53000	79.34000	1264889297

№	Order	Family	Genus	Species	Taxon Rank	Latitude	Longitude	GBIF_ID
12424	Diptera	Culicidae	Aedes	Aedes aegypti	SPECIES	21.54000	39.17000	1264886896
12425	Diptera	Culicidae	Aedes	Aedes aegypti	SPECIES	21.54556	-100.15944	1894254213
12426	Diptera	Culicidae	Aedes	Aedes aegypti	SPECIES	21.55833	-98.81806	1894397655
12427	Diptera	Culicidae	Aedes	Aedes aegypti	SPECIES	21.56972	-100.09667	1894254661
12428	Diptera	Culicidae	Aedes	Aedes aegypti	SPECIES	21.58222	-104.82417	1421475737
12429	Diptera	Culicidae	Aedes	Aedes aegypti	SPECIES	21.60139	-103.82750	1894396859
12430	Diptera	Culicidae	Aedes	Aedes aegypti	SPECIES	21.60139	-99.01028	1894398323
12431	Diptera	Culicidae	Aedes	Aedes aegypti	SPECIES	21.60222	-98.66083	1894400238
12432	Diptera	Culicidae	Aedes	Aedes aegypti	SPECIES	21.60250	-100.17278	1894255564
12433	Diptera	Culicidae	Aedes	Aedes aegypti	SPECIES	21.61944	-98.90278	1894400233
12434	Diptera	Culicidae	Aedes	Aedes aegypti	SPECIES	21.62111	-99.02111	1894400251
12435	Diptera	Culicidae	Aedes	Aedes aegypti	SPECIES	21.62806	-98.66528	1894398461
12436	Diptera	Culicidae	Aedes	Aedes aegypti	SPECIES	21.63000	41.21000	1264891025
12437	Diptera	Culicidae	Aedes	Aedes aegypti	SPECIES	21.63333	-106.54139	1894399873
12438	Diptera	Culicidae	Aedes	Aedes aegypti	SPECIES	21.63444	-98.69667	1894399501
12439	Diptera	Culicidae	Aedes	Aedes aegypti	SPECIES	21.63472	-99.02583	1894399548
12440	Diptera	Culicidae	Aedes	Aedes aegypti	SPECIES	21.65000	78.18000	1264889281
12441	Diptera	Culicidae	Aedes	Aedes aegypti	SPECIES	21.65167	-99.07194	1894395439
12442	Diptera	Culicidae	Aedes	Aedes aegypti	SPECIES	21.65250	-99.81972	1894400173
12443	Diptera	Culicidae	Aedes	Aedes aegypti	SPECIES	21.66111	-97.85056	1421477413
12444	Diptera	Culicidae	Aedes	Aedes aegypti	SPECIES	21.66389	-100.71806	1894398021
12445	Diptera	Culicidae	Aedes	Aedes aegypti	SPECIES	21.66500	-100.33306	1894249625
12446	Diptera	Culicidae	Aedes	Aedes aegypti	SPECIES	21.66583	-98.88722	1894399817
12447	Diptera	Culicidae	Aedes	Aedes aegypti	SPECIES	21.66861	-98.71750	1894398085
12448	Diptera	Culicidae	Aedes	Aedes aegypti	SPECIES	21.67333	-98.92694	1894397868
12449	Diptera	Culicidae	Aedes	Aedes aegypti	SPECIES	21.67361	-102.58889	1894395264
12450	Diptera	Culicidae	Aedes	Aedes aegypti	SPECIES	21.67417	-98.85694	1894397087
12451	Diptera	Culicidae	Aedes	Aedes aegypti	SPECIES	21.68056	-98.89167	1894394186
12452	Diptera	Culicidae	Aedes	Aedes aegypti	SPECIES	21.68278	-103.81194	1894399615
12453	Diptera	Culicidae	Aedes	Aedes aegypti	SPECIES	21.68806	-98.63833	1894398603
12454	Diptera	Culicidae	Aedes	Aedes aegypti	SPECIES	21.70000	70.58000	1264889255
12455	Diptera	Culicidae	Aedes	Aedes aegypti	SPECIES	21.70194	-98.83750	1894395813
12456	Diptera	Culicidae	Aedes	Aedes aegypti	SPECIES	21.70889	-98.96167	1894399398
12457	Diptera	Culicidae	Aedes	Aedes aegypti	SPECIES	21.71194	-100.66278	1894398067
12458	Diptera	Culicidae	Aedes	Aedes aegypti	SPECIES	21.71417	-100.97056	1894254234
12459	Diptera	Culicidae	Aedes	Aedes aegypti	SPECIES	21.71750	-98.58722	1894400292
12460	Diptera	Culicidae	Aedes	Aedes aegypti	SPECIES	21.73528	-101.04917	1894254501
12461	Diptera	Culicidae	Aedes	Aedes aegypti	SPECIES	21.74444	-98.49167	1894394461
12462	Diptera	Culicidae	Aedes	Aedes aegypti	SPECIES	21.75333	-99.05639	1894399897
12463	Diptera	Culicidae	Aedes	Aedes aegypti	SPECIES	21.76028	-100.68833	1894398549
12464	Diptera	Culicidae	Aedes	Aedes aegypti	SPECIES	21.77000	-79.27000	1264886543

№	Order	Family	Genus	Species	Taxon Rank	Latitude	Longitude	GBIF_ID
12465	Diptera	Culicidae	Aedes	Aedes aegypti	SPECIES	21.77000	72.14000	1264889268
12466	Diptera	Culicidae	Aedes	Aedes aegypti	SPECIES	21.77194	-98.63056	1894396784
12467	Diptera	Culicidae	Aedes	Aedes aegypti	SPECIES	21.77639	-98.63139	1894400141
12468	Diptera	Culicidae	Aedes	Aedes aegypti	SPECIES	21.78000	-104.87000	1264891007
12469	Diptera	Culicidae	Aedes	Aedes aegypti	SPECIES	21.78333	-98.78028	1894400011
12470	Diptera	Culicidae	Aedes	Aedes aegypti	SPECIES	21.78417	-98.62306	1894398618
12471	Diptera	Culicidae	Aedes	Aedes aegypti	SPECIES	21.78500	-101.00056	1894394871
12472	Diptera	Culicidae	Aedes	Aedes aegypti	SPECIES	21.78861	-102.80639	1894394451
12473	Diptera	Culicidae	Aedes	Aedes aegypti	SPECIES	21.79583	-98.97167	1894399499
12474	Diptera	Culicidae	Aedes	Aedes aegypti	SPECIES	21.79750	-100.73611	1894400035
12475	Diptera	Culicidae	Aedes	Aedes aegypti	SPECIES	21.80000	-79.98000	1264886602
12476	Diptera	Culicidae	Aedes	Aedes aegypti	SPECIES	21.80333	-102.72056	1894399027
12477	Diptera	Culicidae	Aedes	Aedes aegypti	SPECIES	21.80389	-100.93333	1894400163
12478	Diptera	Culicidae	Aedes	Aedes aegypti	SPECIES	21.81000	-105.21000	1264891296
12479	Diptera	Culicidae	Aedes	Aedes aegypti	SPECIES	21.81442	-105.21325	2629265187
12480	Diptera	Culicidae	Aedes	Aedes aegypti	SPECIES	21.82028	-102.77000	1894396544
12481	Diptera	Culicidae	Aedes	Aedes aegypti	SPECIES	21.82417	-102.18806	1894398384
12482	Diptera	Culicidae	Aedes	Aedes aegypti	SPECIES	21.82639	-102.75861	1894397195
12483	Diptera	Culicidae	Aedes	Aedes aegypti	SPECIES	21.83222	-102.74972	1894396579
12484	Diptera	Culicidae	Aedes	Aedes aegypti	SPECIES	21.84361	-99.64583	1894399844
12485	Diptera	Culicidae	Aedes	Aedes aegypti	SPECIES	21.84611	-102.71833	1894399923
12486	Diptera	Culicidae	Aedes	Aedes aegypti	SPECIES	21.84667	-100.75417	1894395902
12487	Diptera	Culicidae	Aedes	Aedes aegypti	SPECIES	21.84944	-98.93917	1894398431
12488	Diptera	Culicidae	Aedes	Aedes aegypti	SPECIES	21.85000	70.70000	1264889249
12489	Diptera	Culicidae	Aedes	Aedes aegypti	SPECIES	21.85833	-102.62639	1894399640
12490	Diptera	Culicidae	Aedes	Aedes aegypti	SPECIES	21.85944	-102.66444	1894398271
12491	Diptera	Culicidae	Aedes	Aedes aegypti	SPECIES	21.86056	-102.20639	1894396040
12492	Diptera	Culicidae	Aedes	Aedes aegypti	SPECIES	21.86361	-102.69667	1894397305
12493	Diptera	Culicidae	Aedes	Aedes aegypti	SPECIES	21.86444	-99.08056	1894396020
12494	Diptera	Culicidae	Aedes	Aedes aegypti	SPECIES	21.86528	-102.67750	1894396612
12495	Diptera	Culicidae	Aedes	Aedes aegypti	SPECIES	21.86528	-103.77278	1894398206
12496	Diptera	Culicidae	Aedes	Aedes aegypti	SPECIES	21.87556	-102.67528	1894398098
12497	Diptera	Culicidae	Aedes	Aedes aegypti	SPECIES	21.87889	-102.30000	1894400171
12498	Diptera	Culicidae	Aedes	Aedes aegypti	SPECIES	21.88000	106.35000	1264889041
12499	Diptera	Culicidae	Aedes	Aedes aegypti	SPECIES	21.88611	-99.97556	1894399396
12500	Diptera	Culicidae	Aedes	Aedes aegypti	SPECIES	21.91306	-98.85694	1894395520
12501	Diptera	Culicidae	Aedes	Aedes aegypti	SPECIES	21.91806	-102.69833	1894395089
12502	Diptera	Culicidae	Aedes	Aedes aegypti	SPECIES	21.92028	-99.39167	1894398888
12503	Diptera	Culicidae	Aedes	Aedes aegypti	SPECIES	21.92167	-100.10111	1894394860
12504	Diptera	Culicidae	Aedes	Aedes aegypti	SPECIES	21.93000	-79.44000	1264886601
12505	Diptera	Culicidae	Aedes	Aedes aegypti	SPECIES	21.93194	-99.99667	1894400253

№	Order	Family	Genus	Species	Taxon Rank	Latitude	Longitude	GBIF_ID
12506	Diptera	Culicidae	Aedes	Aedes aegypti	SPECIES	21.93906	-105.29586	2629264721
12507	Diptera	Culicidae	Aedes	Aedes aegypti	SPECIES	21.94222	-100.01083	1894400150
12508	Diptera	Culicidae	Aedes	Aedes aegypti	SPECIES	21.95000	-79.17000	1264886588
12509	Diptera	Culicidae	Aedes	Aedes aegypti	SPECIES	21.95000	70.24000	1264889307
12510	Diptera	Culicidae	Aedes	Aedes aegypti	SPECIES	21.95778	-99.39222	1894397210
12511	Diptera	Culicidae	Aedes	Aedes aegypti	SPECIES	21.95972	-99.30667	1894399586
12512	Diptera	Culicidae	Aedes	Aedes aegypti	SPECIES	21.96028	-99.99694	1894398790
12513	Diptera	Culicidae	Aedes	Aedes aegypti	SPECIES	21.96222	-102.34472	1894398479
12514	Diptera	Culicidae	Aedes	Aedes aegypti	SPECIES	21.97000	120.74000	1264877599
12515	Diptera	Culicidae	Aedes	Aedes aegypti	SPECIES	21.97278	-98.91944	1894399467
12516	Diptera	Culicidae	Aedes	Aedes aegypti	SPECIES	21.97500	-99.25694	1894399607
12517	Diptera	Culicidae	Aedes	Aedes aegypti	SPECIES	21.98000	120.75000	1264876916
12518	Diptera	Culicidae	Aedes	Aedes aegypti	SPECIES	21.98000	120.72000	1264883642
12519	Diptera	Culicidae	Aedes	Aedes aegypti	SPECIES	21.98028	-98.93250	1894397910
12520	Diptera	Culicidae	Aedes	Aedes aegypti	SPECIES	21.98194	-99.00972	1894400205
12521	Diptera	Culicidae	Aedes	Aedes aegypti	SPECIES	21.99000	120.73000	1264876835
12522	Diptera	Culicidae	Aedes	Aedes aegypti	SPECIES	21.99000	120.83000	1264876885
12523	Diptera	Culicidae	Aedes	Aedes aegypti	SPECIES	21.99111	-98.76583	1894400196
12524	Diptera	Culicidae	Aedes	Aedes aegypti	SPECIES	21.99667	-99.64389	1894400307
12525	Diptera	Culicidae	Aedes	Aedes aegypti	SPECIES	21.99917	-99.12583	1894396631
12526	Diptera	Culicidae	Aedes	Aedes aegypti	SPECIES	22.00000	120.80000	1264876836
12527	Diptera	Culicidae	Aedes	Aedes aegypti	SPECIES	22.00000	120.76000	1264877718
12528	Diptera	Culicidae	Aedes	Aedes aegypti	SPECIES	22.00000	120.75000	1264884215
12529	Diptera	Culicidae	Aedes	Aedes aegypti	SPECIES	22.00111	-99.04444	1894396588
12530	Diptera	Culicidae	Aedes	Aedes aegypti	SPECIES	22.00333	-98.77889	1894400055
12531	Diptera	Culicidae	Aedes	Aedes aegypti	SPECIES	22.00806	-99.22333	1894396696
12532	Diptera	Culicidae	Aedes	Aedes aegypti	SPECIES	22.01000	120.71000	1264877572
12533	Diptera	Culicidae	Aedes	Aedes aegypti	SPECIES	22.01000	120.75000	1264884194
12534	Diptera	Culicidae	Aedes	Aedes aegypti	SPECIES	22.01000	120.74000	1264884196
12535	Diptera	Culicidae	Aedes	Aedes aegypti	SPECIES	22.01000	-79.27000	1264886590
12536	Diptera	Culicidae	Aedes	Aedes aegypti	SPECIES	22.01361	-99.01917	1894399631
12537	Diptera	Culicidae	Aedes	Aedes aegypti	SPECIES	22.01583	-98.97111	1894400225
12538	Diptera	Culicidae	Aedes	Aedes aegypti	SPECIES	22.02000	120.75000	1264884220
12539	Diptera	Culicidae	Aedes	Aedes aegypti	SPECIES	22.02611	-99.26500	1894395227
12540	Diptera	Culicidae	Aedes	Aedes aegypti	SPECIES	22.02778	-98.97361	1894395446
12541	Diptera	Culicidae	Aedes	Aedes aegypti	SPECIES	22.03000	120.82000	1264876775
12542	Diptera	Culicidae	Aedes	Aedes aegypti	SPECIES	22.03000	-99.03167	1894398794
12543	Diptera	Culicidae	Aedes	Aedes aegypti	SPECIES	22.03472	-98.87361	1894399542
12544	Diptera	Culicidae	Aedes	Aedes aegypti	SPECIES	22.04000	120.71000	1264876869
12545	Diptera	Culicidae	Aedes	Aedes aegypti	SPECIES	22.04000	-98.20000	1264887426
12546	Diptera	Culicidae	Aedes	Aedes aegypti	SPECIES	22.04028	-98.46056	1894399154

№	Order	Family	Genus	Species	Taxon Rank	Latitude	Longitude	GBIF_ID
12547	Diptera	Culicidae	Aedes	Aedes aegypti	SPECIES	22.04167	-103.73472	1894397013
12548	Diptera	Culicidae	Aedes	Aedes aegypti	SPECIES	22.05000	120.71000	1264877315
12549	Diptera	Culicidae	Aedes	Aedes aegypti	SPECIES	22.05000	-98.19000	1264887074
12550	Diptera	Culicidae	Aedes	Aedes aegypti	SPECIES	22.05000	-98.18000	1264888557
12551	Diptera	Culicidae	Aedes	Aedes aegypti	SPECIES	22.05000	70.85000	1264889306
12552	Diptera	Culicidae	Aedes	Aedes aegypti	SPECIES	22.05000	72.56000	1264890709
12553	Diptera	Culicidae	Aedes	Aedes aegypti	SPECIES	22.05528	-98.17750	2629263548
12554	Diptera	Culicidae	Aedes	Aedes aegypti	SPECIES	22.06639	-99.08611	1894400070
12555	Diptera	Culicidae	Aedes	Aedes aegypti	SPECIES	22.07000	120.80000	1264876758
12556	Diptera	Culicidae	Aedes	Aedes aegypti	SPECIES	22.07000	120.78000	1264877486
12557	Diptera	Culicidae	Aedes	Aedes aegypti	SPECIES	22.07000	120.71000	1264881231
12558	Diptera	Culicidae	Aedes	Aedes aegypti	SPECIES	22.07000	120.72000	1264883669
12559	Diptera	Culicidae	Aedes	Aedes aegypti	SPECIES	22.08000	120.75000	1264877350
12560	Diptera	Culicidae	Aedes	Aedes aegypti	SPECIES	22.08000	120.72000	1264877372
12561	Diptera	Culicidae	Aedes	Aedes aegypti	SPECIES	22.08000	-79.50000	1264886594
12562	Diptera	Culicidae	Aedes	Aedes aegypti	SPECIES	22.09000	120.75000	1264876891
12563	Diptera	Culicidae	Aedes	Aedes aegypti	SPECIES	22.09500	-98.48139	1894396412
12564	Diptera	Culicidae	Aedes	Aedes aegypti	SPECIES	22.10000	120.87000	1264876863
12565	Diptera	Culicidae	Aedes	Aedes aegypti	SPECIES	22.10444	-99.20333	1894400084
12566	Diptera	Culicidae	Aedes	Aedes aegypti	SPECIES	22.11000	120.72000	1264877279
12567	Diptera	Culicidae	Aedes	Aedes aegypti	SPECIES	22.11000	-79.72000	1264886584
12568	Diptera	Culicidae	Aedes	Aedes aegypti	SPECIES	22.11000	95.14000	1264890122
12569	Diptera	Culicidae	Aedes	Aedes aegypti	SPECIES	22.12000	120.85000	1264876730
12570	Diptera	Culicidae	Aedes	Aedes aegypti	SPECIES	22.12139	-98.98361	1894394343
12571	Diptera	Culicidae	Aedes	Aedes aegypti	SPECIES	22.12861	-99.29639	1894397788
12572	Diptera	Culicidae	Aedes	Aedes aegypti	SPECIES	22.13000	120.87000	1264877742
12573	Diptera	Culicidae	Aedes	Aedes aegypti	SPECIES	22.13000	120.77000	1264881224
12574	Diptera	Culicidae	Aedes	Aedes aegypti	SPECIES	22.13000	70.63000	1264889290
12575	Diptera	Culicidae	Aedes	Aedes aegypti	SPECIES	22.14000	-80.43000	1264891191
12576	Diptera	Culicidae	Aedes	Aedes aegypti	SPECIES	22.15000	-80.44000	1264886411
12577	Diptera	Culicidae	Aedes	Aedes aegypti	SPECIES	22.15111	-100.97611	1894400198
12578	Diptera	Culicidae	Aedes	Aedes aegypti	SPECIES	22.15417	-99.31167	1894399622
12579	Diptera	Culicidae	Aedes	Aedes aegypti	SPECIES	22.15701	-100.98690	3391297584
12580	Diptera	Culicidae	Aedes	Aedes aegypti	SPECIES	22.17222	-98.46250	1894394107
12581	Diptera	Culicidae	Aedes	Aedes aegypti	SPECIES	22.17778	-98.50583	1894398782
12582	Diptera	Culicidae	Aedes	Aedes aegypti	SPECIES	22.17917	-99.13556	1894398464
12583	Diptera	Culicidae	Aedes	Aedes aegypti	SPECIES	22.18000	120.83000	1264876731
12584	Diptera	Culicidae	Aedes	Aedes aegypti	SPECIES	22.18694	-100.93694	1894399909
12585	Diptera	Culicidae	Aedes	Aedes aegypti	SPECIES	22.19000	120.88000	1264876739
12586	Diptera	Culicidae	Aedes	Aedes aegypti	SPECIES	22.19000	120.69000	1264879327
12587	Diptera	Culicidae	Aedes	Aedes aegypti	SPECIES	22.19194	-102.07333	1894394962

№	Order	Family	Genus	Species	Taxon Rank	Latitude	Longitude	GBIF_ID
12588	Diptera	Culicidae	Aedes	Aedes aegypti	SPECIES	22.19722	-101.02056	1894398228
12589	Diptera	Culicidae	Aedes	Aedes aegypti	SPECIES	22.20000	120.85000	1264876846
12590	Diptera	Culicidae	Aedes	Aedes aegypti	SPECIES	22.20000	120.70000	1264876972
12591	Diptera	Culicidae	Aedes	Aedes aegypti	SPECIES	22.20000	120.71000	1264877051
12592	Diptera	Culicidae	Aedes	Aedes aegypti	SPECIES	22.20000	120.75000	1264879091
12593	Diptera	Culicidae	Aedes	Aedes aegypti	SPECIES	22.21000	-97.85000	1264891155
12594	Diptera	Culicidae	Aedes	Aedes aegypti	SPECIES	22.21361	-98.99139	1894398702
12595	Diptera	Culicidae	Aedes	Aedes aegypti	SPECIES	22.21389	-98.37528	1894399992
12596	Diptera	Culicidae	Aedes	Aedes aegypti	SPECIES	22.21667	-100.87500	1894396214
12597	Diptera	Culicidae	Aedes	Aedes aegypti	SPECIES	22.21667	-99.24000	1894398967
12598	Diptera	Culicidae	Aedes	Aedes aegypti	SPECIES	22.21944	-101.02889	1894395678
12599	Diptera	Culicidae	Aedes	Aedes aegypti	SPECIES	22.22000	-97.86000	1264888710
12600	Diptera	Culicidae	Aedes	Aedes aegypti	SPECIES	22.22000	-100.96528	1894394359
12601	Diptera	Culicidae	Aedes	Aedes aegypti	SPECIES	22.22306	-98.35278	1894398958
12602	Diptera	Culicidae	Aedes	Aedes aegypti	SPECIES	22.22639	-102.27917	1894394494
12603	Diptera	Culicidae	Aedes	Aedes aegypti	SPECIES	22.23000	120.80000	1264877010
12604	Diptera	Culicidae	Aedes	Aedes aegypti	SPECIES	22.23000	84.81000	1264889884
12605	Diptera	Culicidae	Aedes	Aedes aegypti	SPECIES	22.23000	-102.31917	1894397290
12606	Diptera	Culicidae	Aedes	Aedes aegypti	SPECIES	22.23389	-98.79056	1894395217
12607	Diptera	Culicidae	Aedes	Aedes aegypti	SPECIES	22.24000	120.72000	1264877060
12608	Diptera	Culicidae	Aedes	Aedes aegypti	SPECIES	22.24000	-80.39000	1264891190
12609	Diptera	Culicidae	Aedes	Aedes aegypti	SPECIES	22.24167	-101.00361	1894397346
12610	Diptera	Culicidae	Aedes	Aedes aegypti	SPECIES	22.24500	-100.65528	1894398826
12611	Diptera	Culicidae	Aedes	Aedes aegypti	SPECIES	22.25000	-79.78000	1264890922
12612	Diptera	Culicidae	Aedes	Aedes aegypti	SPECIES	22.25222	-99.27722	1894397439
12613	Diptera	Culicidae	Aedes	Aedes aegypti	SPECIES	22.25528	-97.86861	1894400278
12614	Diptera	Culicidae	Aedes	Aedes aegypti	SPECIES	22.26000	120.66000	1264876955
12615	Diptera	Culicidae	Aedes	Aedes aegypti	SPECIES	22.26111	-98.59167	1894400041
12616	Diptera	Culicidae	Aedes	Aedes aegypti	SPECIES	22.27389	-100.57639	1894400208
12617	Diptera	Culicidae	Aedes	Aedes aegypti	SPECIES	22.28222	-100.66861	1894397697
12618	Diptera	Culicidae	Aedes	Aedes aegypti	SPECIES	22.28444	-102.25111	1894395743
12619	Diptera	Culicidae	Aedes	Aedes aegypti	SPECIES	22.28611	-98.65139	1894396770
12620	Diptera	Culicidae	Aedes	Aedes aegypti	SPECIES	22.28750	-100.69639	1894394931
12621	Diptera	Culicidae	Aedes	Aedes aegypti	SPECIES	22.29000	-97.88000	1264887012
12622	Diptera	Culicidae	Aedes	Aedes aegypti	SPECIES	22.30000	120.72000	1264878944
12623	Diptera	Culicidae	Aedes	Aedes aegypti	SPECIES	22.30000	70.80000	1264890715
12624	Diptera	Culicidae	Aedes	Aedes aegypti	SPECIES	22.30000	73.20000	1264890738
12625	Diptera	Culicidae	Aedes	Aedes aegypti	SPECIES	22.30000	-97.85000	1264891410
12626	Diptera	Culicidae	Aedes	Aedes aegypti	SPECIES	22.30000	113.23000	3348956960
12627	Diptera	Culicidae	Aedes	Aedes aegypti	SPECIES	22.30444	-100.60750	1894400294
12628	Diptera	Culicidae	Aedes	Aedes aegypti	SPECIES	22.30750	-98.86083	1894397872

№	Order	Family	Genus	Species	Taxon Rank	Latitude	Longitude	GBIF_ID
12629	Diptera	Culicidae	Aedes	Aedes aegypti	SPECIES	22.31000	73.19000	1264890677
12630	Diptera	Culicidae	Aedes	Aedes aegypti	SPECIES	22.31000	74.01000	1264890698
12631	Diptera	Culicidae	Aedes	Aedes aegypti	SPECIES	22.32389	-100.26611	1894400236
12632	Diptera	Culicidae	Aedes	Aedes aegypti	SPECIES	22.32556	-100.94667	1894399680
12633	Diptera	Culicidae	Aedes	Aedes aegypti	SPECIES	22.33000	120.62000	1264880736
12634	Diptera	Culicidae	Aedes	Aedes aegypti	SPECIES	22.33000	-79.24000	1264886539
12635	Diptera	Culicidae	Aedes	Aedes aegypti	SPECIES	22.33000	120.37000	1264890285
12636	Diptera	Culicidae	Aedes	Aedes aegypti	SPECIES	22.33000	120.36000	1264890316
12637	Diptera	Culicidae	Aedes	Aedes aegypti	SPECIES	22.33972	-98.70833	1894398575
12638	Diptera	Culicidae	Aedes	Aedes aegypti	SPECIES	22.34000	120.61000	1264877842
12639	Diptera	Culicidae	Aedes	Aedes aegypti	SPECIES	22.34000	120.37000	1264890275
12640	Diptera	Culicidae	Aedes	Aedes aegypti	SPECIES	22.35000	120.39000	1264876861
12641	Diptera	Culicidae	Aedes	Aedes aegypti	SPECIES	22.35000	120.37000	1264890311
12642	Diptera	Culicidae	Aedes	Aedes aegypti	SPECIES	22.35000	120.38000	1264890338
12643	Diptera	Culicidae	Aedes	Aedes aegypti	SPECIES	22.36000	120.61000	1264877662
12644	Diptera	Culicidae	Aedes	Aedes aegypti	SPECIES	22.36000	120.60000	1264877734
12645	Diptera	Culicidae	Aedes	Aedes aegypti	SPECIES	22.37000	120.60000	1264877019
12646	Diptera	Culicidae	Aedes	Aedes aegypti	SPECIES	22.37000	-80.12000	1264890849
12647	Diptera	Culicidae	Aedes	Aedes aegypti	SPECIES	22.37361	-99.29278	1894397337
12648	Diptera	Culicidae	Aedes	Aedes aegypti	SPECIES	22.38000	120.60000	1264877612
12649	Diptera	Culicidae	Aedes	Aedes aegypti	SPECIES	22.38000	120.58000	1264877709
12650	Diptera	Culicidae	Aedes	Aedes aegypti	SPECIES	22.38000	120.64000	1264884257
12651	Diptera	Culicidae	Aedes	Aedes aegypti	SPECIES	22.38833	-103.72889	1894396496
12652	Diptera	Culicidae	Aedes	Aedes aegypti	SPECIES	22.39000	120.59000	1264876628
12653	Diptera	Culicidae	Aedes	Aedes aegypti	SPECIES	22.39000	120.61000	1264877641
12654	Diptera	Culicidae	Aedes	Aedes aegypti	SPECIES	22.39000	120.56000	1264877678
12655	Diptera	Culicidae	Aedes	Aedes aegypti	SPECIES	22.39722	-100.57472	1894395308
12656	Diptera	Culicidae	Aedes	Aedes aegypti	SPECIES	22.39833	-99.60417	1894399678
12657	Diptera	Culicidae	Aedes	Aedes aegypti	SPECIES	22.40000	120.58000	1264877645
12658	Diptera	Culicidae	Aedes	Aedes aegypti	SPECIES	22.40000	120.56000	1264877736
12659	Diptera	Culicidae	Aedes	Aedes aegypti	SPECIES	22.40361	-99.33444	1894397542
12660	Diptera	Culicidae	Aedes	Aedes aegypti	SPECIES	22.40408	-105.45911	2629263912
12661	Diptera	Culicidae	Aedes	Aedes aegypti	SPECIES	22.41000	120.59000	1264877670
12662	Diptera	Culicidae	Aedes	Aedes aegypti	SPECIES	22.41000	120.62000	1264877715
12663	Diptera	Culicidae	Aedes	Aedes aegypti	SPECIES	22.41000	120.65000	1264884823
12664	Diptera	Culicidae	Aedes	Aedes aegypti	SPECIES	22.41472	-100.63444	1894399102
12665	Diptera	Culicidae	Aedes	Aedes aegypti	SPECIES	22.42000	120.56000	1264877169
12666	Diptera	Culicidae	Aedes	Aedes aegypti	SPECIES	22.42000	120.54000	1264877314
12667	Diptera	Culicidae	Aedes	Aedes aegypti	SPECIES	22.42000	120.52000	1264877370
12668	Diptera	Culicidae	Aedes	Aedes aegypti	SPECIES	22.42000	120.51000	1264877769
12669	Diptera	Culicidae	Aedes	Aedes aegypti	SPECIES	22.42000	120.71000	1264878915

№	Order	Family	Genus	Species	Taxon Rank	Latitude	Longitude	GBIF_ID
12670	Diptera	Culicidae	Aedes	Aedes aegypti	SPECIES	22.43000	120.56000	1264876819
12671	Diptera	Culicidae	Aedes	Aedes aegypti	SPECIES	22.43000	120.53000	1264877182
12672	Diptera	Culicidae	Aedes	Aedes aegypti	SPECIES	22.43000	120.52000	1264877199
12673	Diptera	Culicidae	Aedes	Aedes aegypti	SPECIES	22.43000	120.50000	1264877231
12674	Diptera	Culicidae	Aedes	Aedes aegypti	SPECIES	22.43000	120.51000	1264877332
12675	Diptera	Culicidae	Aedes	Aedes aegypti	SPECIES	22.43000	70.60000	1264889302
12676	Diptera	Culicidae	Aedes	Aedes aegypti	SPECIES	22.43000	91.79000	1264891200
12677	Diptera	Culicidae	Aedes	Aedes aegypti	SPECIES	22.43056	-100.70000	1894397916
12678	Diptera	Culicidae	Aedes	Aedes aegypti	SPECIES	22.43278	-100.28167	1894400054
12679	Diptera	Culicidae	Aedes	Aedes aegypti	SPECIES	22.43353	-102.24805	2283098618
12680	Diptera	Culicidae	Aedes	Aedes aegypti	SPECIES	22.44000	120.59000	1264877208
12681	Diptera	Culicidae	Aedes	Aedes aegypti	SPECIES	22.44000	120.47000	1264877230
12682	Diptera	Culicidae	Aedes	Aedes aegypti	SPECIES	22.44000	120.51000	1264877382
12683	Diptera	Culicidae	Aedes	Aedes aegypti	SPECIES	22.44000	120.50000	1264877400
12684	Diptera	Culicidae	Aedes	Aedes aegypti	SPECIES	22.44000	120.56000	1264877594
12685	Diptera	Culicidae	Aedes	Aedes aegypti	SPECIES	22.44306	-100.62389	1894395818
12686	Diptera	Culicidae	Aedes	Aedes aegypti	SPECIES	22.44944	-100.67750	1894398970
12687	Diptera	Culicidae	Aedes	Aedes aegypti	SPECIES	22.45000	120.51000	1264877005
12688	Diptera	Culicidae	Aedes	Aedes aegypti	SPECIES	22.45000	120.49000	1264877100
12689	Diptera	Culicidae	Aedes	Aedes aegypti	SPECIES	22.45000	120.55000	1264877441
12690	Diptera	Culicidae	Aedes	Aedes aegypti	SPECIES	22.45000	120.53000	1264877705
12691	Diptera	Culicidae	Aedes	Aedes aegypti	SPECIES	22.45000	120.57000	1264877706
12692	Diptera	Culicidae	Aedes	Aedes aegypti	SPECIES	22.45000	120.45000	1264884392
12693	Diptera	Culicidae	Aedes	Aedes aegypti	SPECIES	22.45000	120.72000	1264884890
12694	Diptera	Culicidae	Aedes	Aedes aegypti	SPECIES	22.46000	120.59000	1264877592
12695	Diptera	Culicidae	Aedes	Aedes aegypti	SPECIES	22.46000	120.47000	1264884374
12696	Diptera	Culicidae	Aedes	Aedes aegypti	SPECIES	22.46000	120.68000	1264884825
12697	Diptera	Culicidae	Aedes	Aedes aegypti	SPECIES	22.46000	120.45000	1264885096
12698	Diptera	Culicidae	Aedes	Aedes aegypti	SPECIES	22.46000	120.44000	1264885105
12699	Diptera	Culicidae	Aedes	Aedes aegypti	SPECIES	22.47000	120.47000	1264876749
12700	Diptera	Culicidae	Aedes	Aedes aegypti	SPECIES	22.47000	120.54000	1264876882
12701	Diptera	Culicidae	Aedes	Aedes aegypti	SPECIES	22.47000	120.49000	1264876893
12702	Diptera	Culicidae	Aedes	Aedes aegypti	SPECIES	22.47000	120.51000	1264876895
12703	Diptera	Culicidae	Aedes	Aedes aegypti	SPECIES	22.47000	120.52000	1264877130
12704	Diptera	Culicidae	Aedes	Aedes aegypti	SPECIES	22.47000	120.56000	1264877580
12705	Diptera	Culicidae	Aedes	Aedes aegypti	SPECIES	22.47000	120.44000	1264878998
12706	Diptera	Culicidae	Aedes	Aedes aegypti	SPECIES	22.47000	120.63000	1264881518
12707	Diptera	Culicidae	Aedes	Aedes aegypti	SPECIES	22.47000	120.45000	1264885081
12708	Diptera	Culicidae	Aedes	Aedes aegypti	SPECIES	22.48000	120.50000	1264876862
12709	Diptera	Culicidae	Aedes	Aedes aegypti	SPECIES	22.48000	120.52000	1264876883
12710	Diptera	Culicidae	Aedes	Aedes aegypti	SPECIES	22.48000	120.57000	1264877546

№	Order	Family	Genus	Species	Taxon Rank	Latitude	Longitude	GBIF_ID
12711	Diptera	Culicidae	Aedes	Aedes aegypti	SPECIES	22.48000	120.41000	1264880810
12712	Diptera	Culicidae	Aedes	Aedes aegypti	SPECIES	22.48000	120.40000	1264881522
12713	Diptera	Culicidae	Aedes	Aedes aegypti	SPECIES	22.48000	120.46000	1264882642
12714	Diptera	Culicidae	Aedes	Aedes aegypti	SPECIES	22.48000	120.44000	1264885045
12715	Diptera	Culicidae	Aedes	Aedes aegypti	SPECIES	22.48000	120.43000	1264885140
12716	Diptera	Culicidae	Aedes	Aedes aegypti	SPECIES	22.48000	120.47000	1264885862
12717	Diptera	Culicidae	Aedes	Aedes aegypti	SPECIES	22.48389	-100.55111	1894395821
12718	Diptera	Culicidae	Aedes	Aedes aegypti	SPECIES	22.49000	120.41000	1264876601
12719	Diptera	Culicidae	Aedes	Aedes aegypti	SPECIES	22.49000	120.50000	1264876777
12720	Diptera	Culicidae	Aedes	Aedes aegypti	SPECIES	22.49000	120.48000	1264876974
12721	Diptera	Culicidae	Aedes	Aedes aegypti	SPECIES	22.49000	120.61000	1264877123
12722	Diptera	Culicidae	Aedes	Aedes aegypti	SPECIES	22.49000	120.64000	1264877347
12723	Diptera	Culicidae	Aedes	Aedes aegypti	SPECIES	22.49000	120.51000	1264877458
12724	Diptera	Culicidae	Aedes	Aedes aegypti	SPECIES	22.49000	120.39000	1264883076
12725	Diptera	Culicidae	Aedes	Aedes aegypti	SPECIES	22.49000	120.55000	1264890021
12726	Diptera	Culicidae	Aedes	Aedes aegypti	SPECIES	22.49222	-105.36056	1894398263
12727	Diptera	Culicidae	Aedes	Aedes aegypti	SPECIES	22.49611	-100.61028	1894399061
12728	Diptera	Culicidae	Aedes	Aedes aegypti	SPECIES	22.50000	120.50000	1264877271
12729	Diptera	Culicidae	Aedes	Aedes aegypti	SPECIES	22.50000	120.54000	1264877570
12730	Diptera	Culicidae	Aedes	Aedes aegypti	SPECIES	22.50000	120.56000	1264877632
12731	Diptera	Culicidae	Aedes	Aedes aegypti	SPECIES	22.50000	120.46000	1264877638
12732	Diptera	Culicidae	Aedes	Aedes aegypti	SPECIES	22.50000	120.48000	1264877652
12733	Diptera	Culicidae	Aedes	Aedes aegypti	SPECIES	22.50000	120.52000	1264880707
12734	Diptera	Culicidae	Aedes	Aedes aegypti	SPECIES	22.50000	120.40000	1264885283
12735	Diptera	Culicidae	Aedes	Aedes aegypti	SPECIES	22.50000	120.39000	1264885284
12736	Diptera	Culicidae	Aedes	Aedes aegypti	SPECIES	22.50000	120.37000	1264890016
12737	Diptera	Culicidae	Aedes	Aedes aegypti	SPECIES	22.50000	120.55000	1264890033
12738	Diptera	Culicidae	Aedes	Aedes aegypti	SPECIES	22.50611	-100.66333	1894400139
12739	Diptera	Culicidae	Aedes	Aedes aegypti	SPECIES	22.51000	120.53000	1264876605
12740	Diptera	Culicidae	Aedes	Aedes aegypti	SPECIES	22.51000	120.47000	1264877033
12741	Diptera	Culicidae	Aedes	Aedes aegypti	SPECIES	22.51000	120.43000	1264877456
12742	Diptera	Culicidae	Aedes	Aedes aegypti	SPECIES	22.51000	120.59000	1264877679
12743	Diptera	Culicidae	Aedes	Aedes aegypti	SPECIES	22.51000	120.69000	1264878992
12744	Diptera	Culicidae	Aedes	Aedes aegypti	SPECIES	22.51000	120.37000	1264878995
12745	Diptera	Culicidae	Aedes	Aedes aegypti	SPECIES	22.51000	120.36000	1264879261
12746	Diptera	Culicidae	Aedes	Aedes aegypti	SPECIES	22.51000	120.40000	1264885068
12747	Diptera	Culicidae	Aedes	Aedes aegypti	SPECIES	22.51000	120.39000	1264890035
12748	Diptera	Culicidae	Aedes	Aedes aegypti	SPECIES	22.51667	-99.33778	1894396250
12749	Diptera	Culicidae	Aedes	Aedes aegypti	SPECIES	22.52000	120.56000	1264876839
12750	Diptera	Culicidae	Aedes	Aedes aegypti	SPECIES	22.52000	120.51000	1264877075
12751	Diptera	Culicidae	Aedes	Aedes aegypti	SPECIES	22.52000	120.35000	1264877283

№	Order	Family	Genus	Species	Taxon Rank	Latitude	Longitude	GBIF_ID
12752	Diptera	Culicidae	Aedes	Aedes aegypti	SPECIES	22.52000	120.65000	1264877443
12753	Diptera	Culicidae	Aedes	Aedes aegypti	SPECIES	22.52000	120.72000	1264877461
12754	Diptera	Culicidae	Aedes	Aedes aegypti	SPECIES	22.52000	120.45000	1264877619
12755	Diptera	Culicidae	Aedes	Aedes aegypti	SPECIES	22.52000	120.47000	1264877674
12756	Diptera	Culicidae	Aedes	Aedes aegypti	SPECIES	22.52000	120.40000	1264878694
12757	Diptera	Culicidae	Aedes	Aedes aegypti	SPECIES	22.52000	120.36000	1264879171
12758	Diptera	Culicidae	Aedes	Aedes aegypti	SPECIES	22.52000	120.34000	1264881197
12759	Diptera	Culicidae	Aedes	Aedes aegypti	SPECIES	22.52000	120.39000	1264885054
12760	Diptera	Culicidae	Aedes	Aedes aegypti	SPECIES	22.52000	88.39000	1264889720
12761	Diptera	Culicidae	Aedes	Aedes aegypti	SPECIES	22.52000	-83.51000	1264890026
12762	Diptera	Culicidae	Aedes	Aedes aegypti	SPECIES	22.52000	-99.36139	1894396613
12763	Diptera	Culicidae	Aedes	Aedes aegypti	SPECIES	22.52083	-99.32250	1894400203
12764	Diptera	Culicidae	Aedes	Aedes aegypti	SPECIES	22.53000	120.50000	1264876954
12765	Diptera	Culicidae	Aedes	Aedes aegypti	SPECIES	22.53000	120.54000	1264877336
12766	Diptera	Culicidae	Aedes	Aedes aegypti	SPECIES	22.53000	120.51000	1264877426
12767	Diptera	Culicidae	Aedes	Aedes aegypti	SPECIES	22.53000	120.56000	1264878434
12768	Diptera	Culicidae	Aedes	Aedes aegypti	SPECIES	22.53000	120.35000	1264880763
12769	Diptera	Culicidae	Aedes	Aedes aegypti	SPECIES	22.53000	120.40000	1264884271
12770	Diptera	Culicidae	Aedes	Aedes aegypti	SPECIES	22.53000	88.36000	1264889671
12771	Diptera	Culicidae	Aedes	Aedes aegypti	SPECIES	22.54000	120.67000	1264876810
12772	Diptera	Culicidae	Aedes	Aedes aegypti	SPECIES	22.54000	120.49000	1264876833
12773	Diptera	Culicidae	Aedes	Aedes aegypti	SPECIES	22.54000	120.56000	1264876859
12774	Diptera	Culicidae	Aedes	Aedes aegypti	SPECIES	22.54000	120.41000	1264876953
12775	Diptera	Culicidae	Aedes	Aedes aegypti	SPECIES	22.54000	120.48000	1264876969
12776	Diptera	Culicidae	Aedes	Aedes aegypti	SPECIES	22.54000	120.65000	1264877244
12777	Diptera	Culicidae	Aedes	Aedes aegypti	SPECIES	22.54000	120.53000	1264877383
12778	Diptera	Culicidae	Aedes	Aedes aegypti	SPECIES	22.54000	120.46000	1264877465
12779	Diptera	Culicidae	Aedes	Aedes aegypti	SPECIES	22.54000	120.54000	1264877489
12780	Diptera	Culicidae	Aedes	Aedes aegypti	SPECIES	22.54000	120.44000	1264877567
12781	Diptera	Culicidae	Aedes	Aedes aegypti	SPECIES	22.54000	120.55000	1264877588
12782	Diptera	Culicidae	Aedes	Aedes aegypti	SPECIES	22.54000	120.34000	1264880369
12783	Diptera	Culicidae	Aedes	Aedes aegypti	SPECIES	22.54000	120.40000	1264883127
12784	Diptera	Culicidae	Aedes	Aedes aegypti	SPECIES	22.54250	-98.76694	1894397164
12785	Diptera	Culicidae	Aedes	Aedes aegypti	SPECIES	22.54722	-101.04278	1894396223
12786	Diptera	Culicidae	Aedes	Aedes aegypti	SPECIES	22.54861	-99.32056	1894396146
12787	Diptera	Culicidae	Aedes	Aedes aegypti	SPECIES	22.54889	-100.42111	1894396743
12788	Diptera	Culicidae	Aedes	Aedes aegypti	SPECIES	22.54972	-99.13000	1894399399
12789	Diptera	Culicidae	Aedes	Aedes aegypti	SPECIES	22.55000	120.58000	1264876797
12790	Diptera	Culicidae	Aedes	Aedes aegypti	SPECIES	22.55000	120.42000	1264876981
12791	Diptera	Culicidae	Aedes	Aedes aegypti	SPECIES	22.55000	120.40000	1264877044
12792	Diptera	Culicidae	Aedes	Aedes aegypti	SPECIES	22.55000	120.53000	1264877387

№	Order	Family	Genus	Species	Taxon Rank	Latitude	Longitude	GBIF_ID
12793	Diptera	Culicidae	Aedes	Aedes aegypti	SPECIES	22.55000	120.50000	1264877602
12794	Diptera	Culicidae	Aedes	Aedes aegypti	SPECIES	22.55000	120.45000	1264877623
12795	Diptera	Culicidae	Aedes	Aedes aegypti	SPECIES	22.55000	120.61000	1264877661
12796	Diptera	Culicidae	Aedes	Aedes aegypti	SPECIES	22.55000	120.47000	1264878205
12797	Diptera	Culicidae	Aedes	Aedes aegypti	SPECIES	22.55000	120.39000	1264878655
12798	Diptera	Culicidae	Aedes	Aedes aegypti	SPECIES	22.55000	120.35000	1264885231
12799	Diptera	Culicidae	Aedes	Aedes aegypti	SPECIES	22.55000	120.38000	1264885290
12800	Diptera	Culicidae	Aedes	Aedes aegypti	SPECIES	22.55000	120.54000	1264885475
12801	Diptera	Culicidae	Aedes	Aedes aegypti	SPECIES	22.55000	120.55000	1264890061
12802	Diptera	Culicidae	Aedes	Aedes aegypti	SPECIES	22.55833	-100.64722	1894396193
12803	Diptera	Culicidae	Aedes	Aedes aegypti	SPECIES	22.56000	120.53000	1264876802
12804	Diptera	Culicidae	Aedes	Aedes aegypti	SPECIES	22.56000	120.49000	1264876866
12805	Diptera	Culicidae	Aedes	Aedes aegypti	SPECIES	22.56000	120.69000	1264876975
12806	Diptera	Culicidae	Aedes	Aedes aegypti	SPECIES	22.56000	120.64000	1264877061
12807	Diptera	Culicidae	Aedes	Aedes aegypti	SPECIES	22.56000	120.60000	1264877656
12808	Diptera	Culicidae	Aedes	Aedes aegypti	SPECIES	22.56000	120.33000	1264885204
12809	Diptera	Culicidae	Aedes	Aedes aegypti	SPECIES	22.56000	120.35000	1264885328
12810	Diptera	Culicidae	Aedes	Aedes aegypti	SPECIES	22.56000	120.34000	1264885441
12811	Diptera	Culicidae	Aedes	Aedes aegypti	SPECIES	22.56000	120.31000	1264885538
12812	Diptera	Culicidae	Aedes	Aedes aegypti	SPECIES	22.56000	120.54000	1264885567
12813	Diptera	Culicidae	Aedes	Aedes aegypti	SPECIES	22.56000	120.55000	1264885591
12814	Diptera	Culicidae	Aedes	Aedes aegypti	SPECIES	22.56000	120.36000	1264885948
12815	Diptera	Culicidae	Aedes	Aedes aegypti	SPECIES	22.56000	120.39000	1264886026
12816	Diptera	Culicidae	Aedes	Aedes aegypti	SPECIES	22.57000	120.62000	1264876793
12817	Diptera	Culicidae	Aedes	Aedes aegypti	SPECIES	22.57000	120.47000	1264876822
12818	Diptera	Culicidae	Aedes	Aedes aegypti	SPECIES	22.57000	120.54000	1264876837
12819	Diptera	Culicidae	Aedes	Aedes aegypti	SPECIES	22.57000	120.40000	1264876997
12820	Diptera	Culicidae	Aedes	Aedes aegypti	SPECIES	22.57000	120.43000	1264877042
12821	Diptera	Culicidae	Aedes	Aedes aegypti	SPECIES	22.57000	120.52000	1264877510
12822	Diptera	Culicidae	Aedes	Aedes aegypti	SPECIES	22.57000	120.57000	1264877610
12823	Diptera	Culicidae	Aedes	Aedes aegypti	SPECIES	22.57000	120.58000	1264877628
12824	Diptera	Culicidae	Aedes	Aedes aegypti	SPECIES	22.57000	120.59000	1264877758
12825	Diptera	Culicidae	Aedes	Aedes aegypti	SPECIES	22.57000	120.46000	1264877795
12826	Diptera	Culicidae	Aedes	Aedes aegypti	SPECIES	22.57000	120.49000	1264880550
12827	Diptera	Culicidae	Aedes	Aedes aegypti	SPECIES	22.57000	120.37000	1264883372
12828	Diptera	Culicidae	Aedes	Aedes aegypti	SPECIES	22.57000	120.38000	1264884712
12829	Diptera	Culicidae	Aedes	Aedes aegypti	SPECIES	22.57000	120.30000	1264884880
12830	Diptera	Culicidae	Aedes	Aedes aegypti	SPECIES	22.57000	120.34000	1264884995
12831	Diptera	Culicidae	Aedes	Aedes aegypti	SPECIES	22.57000	120.33000	1264885184
12832	Diptera	Culicidae	Aedes	Aedes aegypti	SPECIES	22.57000	120.36000	1264885366
12833	Diptera	Culicidae	Aedes	Aedes aegypti	SPECIES	22.57000	120.35000	1264885986

№	Order	Family	Genus	Species	Taxon Rank	Latitude	Longitude	GBIF_ID
12834	Diptera	Culicidae	Aedes	Aedes aegypti	SPECIES	22.57000	120.32000	1264885990
12835	Diptera	Culicidae	Aedes	Aedes aegypti	SPECIES	22.57000	88.36000	1264888076
12836	Diptera	Culicidae	Aedes	Aedes aegypti	SPECIES	22.57000	88.35000	1264889764
12837	Diptera	Culicidae	Aedes	Aedes aegypti	SPECIES	22.58000	120.53000	1264876824
12838	Diptera	Culicidae	Aedes	Aedes aegypti	SPECIES	22.58000	120.39000	1264876962
12839	Diptera	Culicidae	Aedes	Aedes aegypti	SPECIES	22.58000	120.42000	1264876991
12840	Diptera	Culicidae	Aedes	Aedes aegypti	SPECIES	22.58000	120.40000	1264877040
12841	Diptera	Culicidae	Aedes	Aedes aegypti	SPECIES	22.58000	120.65000	1264877058
12842	Diptera	Culicidae	Aedes	Aedes aegypti	SPECIES	22.58000	120.30000	1264877323
12843	Diptera	Culicidae	Aedes	Aedes aegypti	SPECIES	22.58000	120.55000	1264877451
12844	Diptera	Culicidae	Aedes	Aedes aegypti	SPECIES	22.58000	120.57000	1264877597
12845	Diptera	Culicidae	Aedes	Aedes aegypti	SPECIES	22.58000	120.48000	1264877625
12846	Diptera	Culicidae	Aedes	Aedes aegypti	SPECIES	22.58000	120.52000	1264877631
12847	Diptera	Culicidae	Aedes	Aedes aegypti	SPECIES	22.58000	120.58000	1264877644
12848	Diptera	Culicidae	Aedes	Aedes aegypti	SPECIES	22.58000	120.56000	1264877650
12849	Diptera	Culicidae	Aedes	Aedes aegypti	SPECIES	22.58000	120.62000	1264877655
12850	Diptera	Culicidae	Aedes	Aedes aegypti	SPECIES	22.58000	120.61000	1264877712
12851	Diptera	Culicidae	Aedes	Aedes aegypti	SPECIES	22.58000	120.50000	1264883777
12852	Diptera	Culicidae	Aedes	Aedes aegypti	SPECIES	22.58000	120.37000	1264884722
12853	Diptera	Culicidae	Aedes	Aedes aegypti	SPECIES	22.58000	120.34000	1264884796
12854	Diptera	Culicidae	Aedes	Aedes aegypti	SPECIES	22.58000	120.31000	1264884993
12855	Diptera	Culicidae	Aedes	Aedes aegypti	SPECIES	22.58000	120.35000	1264885243
12856	Diptera	Culicidae	Aedes	Aedes aegypti	SPECIES	22.58000	120.29000	1264885273
12857	Diptera	Culicidae	Aedes	Aedes aegypti	SPECIES	22.58000	120.32000	1264885644
12858	Diptera	Culicidae	Aedes	Aedes aegypti	SPECIES	22.58000	120.33000	1264885669
12859	Diptera	Culicidae	Aedes	Aedes aegypti	SPECIES	22.58000	120.36000	1264885677
12860	Diptera	Culicidae	Aedes	Aedes aegypti	SPECIES	22.58000	88.36000	1264890681
12861	Diptera	Culicidae	Aedes	Aedes aegypti	SPECIES	22.59000	120.47000	1264876867
12862	Diptera	Culicidae	Aedes	Aedes aegypti	SPECIES	22.59000	120.56000	1264876976
12863	Diptera	Culicidae	Aedes	Aedes aegypti	SPECIES	22.59000	120.43000	1264876992
12864	Diptera	Culicidae	Aedes	Aedes aegypti	SPECIES	22.59000	120.39000	1264877046
12865	Diptera	Culicidae	Aedes	Aedes aegypti	SPECIES	22.59000	120.46000	1264877611
12866	Diptera	Culicidae	Aedes	Aedes aegypti	SPECIES	22.59000	120.55000	1264877617
12867	Diptera	Culicidae	Aedes	Aedes aegypti	SPECIES	22.59000	120.50000	1264880597
12868	Diptera	Culicidae	Aedes	Aedes aegypti	SPECIES	22.59000	120.28000	1264881182
12869	Diptera	Culicidae	Aedes	Aedes aegypti	SPECIES	22.59000	120.35000	1264884963
12870	Diptera	Culicidae	Aedes	Aedes aegypti	SPECIES	22.59000	120.48000	1264885415
12871	Diptera	Culicidae	Aedes	Aedes aegypti	SPECIES	22.59000	120.31000	1264885634
12872	Diptera	Culicidae	Aedes	Aedes aegypti	SPECIES	22.59000	120.32000	1264885653
12873	Diptera	Culicidae	Aedes	Aedes aegypti	SPECIES	22.59000	120.34000	1264885726
12874	Diptera	Culicidae	Aedes	Aedes aegypti	SPECIES	22.59000	120.49000	1264885942

№	Order	Family	Genus	Species	Taxon Rank	Latitude	Longitude	GBIF_ID
12875	Diptera	Culicidae	Aedes	Aedes aegypti	SPECIES	22.59000	120.33000	1264885950
12876	Diptera	Culicidae	Aedes	Aedes aegypti	SPECIES	22.59000	88.31000	1264887830
12877	Diptera	Culicidae	Aedes	Aedes aegypti	SPECIES	22.59460	120.32230	1948738608
12878	Diptera	Culicidae	Aedes	Aedes aegypti	SPECIES	22.59597	120.33754	2236868778
12879	Diptera	Culicidae	Aedes	Aedes aegypti	SPECIES	22.59597	120.33749	2429416066
12880	Diptera	Culicidae	Aedes	Aedes aegypti	SPECIES	22.59600	120.33749	3994203319
12881	Diptera	Culicidae	Aedes	Aedes aegypti	SPECIES	22.59602	120.33755	2235473223
12882	Diptera	Culicidae	Aedes	Aedes aegypti	SPECIES	22.59602	120.33750	2456496101
12883	Diptera	Culicidae	Aedes	Aedes aegypti	SPECIES	22.59604	120.33741	2242919610
12884	Diptera	Culicidae	Aedes	Aedes aegypti	SPECIES	22.59604	120.33749	2273210537
12885	Diptera	Culicidae	Aedes	Aedes aegypti	SPECIES	22.59604	120.33740	2235534277
12886	Diptera	Culicidae	Aedes	Aedes aegypti	SPECIES	22.59604	120.33743	2456496195
12887	Diptera	Culicidae	Aedes	Aedes aegypti	SPECIES	22.59605	120.33741	2265873020
12888	Diptera	Culicidae	Aedes	Aedes aegypti	SPECIES	22.59605	120.33747	2006078984
12889	Diptera	Culicidae	Aedes	Aedes aegypti	SPECIES	22.59607	120.33745	2460109387
12890	Diptera	Culicidae	Aedes	Aedes aegypti	SPECIES	22.59608	120.33747	2236895563
12891	Diptera	Culicidae	Aedes	Aedes aegypti	SPECIES	22.59612	120.33740	1948782495
12892	Diptera	Culicidae	Aedes	Aedes aegypti	SPECIES	22.59828	120.33730	4034650865
12893	Diptera	Culicidae	Aedes	Aedes aegypti	SPECIES	22.59832	120.33772	4034624651
12894	Diptera	Culicidae	Aedes	Aedes aegypti	SPECIES	22.59845	120.33458	1986512432
12895	Diptera	Culicidae	Aedes	Aedes aegypti	SPECIES	22.59891	120.33937	1990469047
12896	Diptera	Culicidae	Aedes	Aedes aegypti	SPECIES	22.59894	120.33942	2619970507
12897	Diptera	Culicidae	Aedes	Aedes aegypti	SPECIES	22.60000	120.51000	1264876611
12898	Diptera	Culicidae	Aedes	Aedes aegypti	SPECIES	22.60000	120.46000	1264876840
12899	Diptera	Culicidae	Aedes	Aedes aegypti	SPECIES	22.60000	120.57000	1264877003
12900	Diptera	Culicidae	Aedes	Aedes aegypti	SPECIES	22.60000	120.53000	1264877269
12901	Diptera	Culicidae	Aedes	Aedes aegypti	SPECIES	22.60000	120.55000	1264877475
12902	Diptera	Culicidae	Aedes	Aedes aegypti	SPECIES	22.60000	120.60000	1264877479
12903	Diptera	Culicidae	Aedes	Aedes aegypti	SPECIES	22.60000	120.58000	1264877591
12904	Diptera	Culicidae	Aedes	Aedes aegypti	SPECIES	22.60000	120.41000	1264880056
12905	Diptera	Culicidae	Aedes	Aedes aegypti	SPECIES	22.60000	120.27000	1264884237
12906	Diptera	Culicidae	Aedes	Aedes aegypti	SPECIES	22.60000	120.28000	1264884636
12907	Diptera	Culicidae	Aedes	Aedes aegypti	SPECIES	22.60000	120.30000	1264885014
12908	Diptera	Culicidae	Aedes	Aedes aegypti	SPECIES	22.60000	120.32000	1264885216
12909	Diptera	Culicidae	Aedes	Aedes aegypti	SPECIES	22.60000	120.49000	1264885429
12910	Diptera	Culicidae	Aedes	Aedes aegypti	SPECIES	22.60000	120.35000	1264885515
12911	Diptera	Culicidae	Aedes	Aedes aegypti	SPECIES	22.60000	120.31000	1264885603
12912	Diptera	Culicidae	Aedes	Aedes aegypti	SPECIES	22.60000	120.29000	1264885687
12913	Diptera	Culicidae	Aedes	Aedes aegypti	SPECIES	22.60000	120.36000	1264885753
12914	Diptera	Culicidae	Aedes	Aedes aegypti	SPECIES	22.60000	120.34000	1264885834
12915	Diptera	Culicidae	Aedes	Aedes aegypti	SPECIES	22.60000	120.33000	1264885972

№	Order	Family	Genus	Species	Taxon Rank	Latitude	Longitude	GBIF_ID
12916	Diptera	Culicidae	Aedes	Aedes aegypti	SPECIES	22.61000	120.46000	1264876826
12917	Diptera	Culicidae	Aedes	Aedes aegypti	SPECIES	22.61000	120.57000	1264876849
12918	Diptera	Culicidae	Aedes	Aedes aegypti	SPECIES	22.61000	120.38000	1264876901
12919	Diptera	Culicidae	Aedes	Aedes aegypti	SPECIES	22.61000	120.65000	1264877084
12920	Diptera	Culicidae	Aedes	Aedes aegypti	SPECIES	22.61000	120.29000	1264877266
12921	Diptera	Culicidae	Aedes	Aedes aegypti	SPECIES	22.61000	120.47000	1264877539
12922	Diptera	Culicidae	Aedes	Aedes aegypti	SPECIES	22.61000	120.62000	1264877576
12923	Diptera	Culicidae	Aedes	Aedes aegypti	SPECIES	22.61000	120.52000	1264877585
12924	Diptera	Culicidae	Aedes	Aedes aegypti	SPECIES	22.61000	120.39000	1264878993
12925	Diptera	Culicidae	Aedes	Aedes aegypti	SPECIES	22.61000	120.40000	1264881455
12926	Diptera	Culicidae	Aedes	Aedes aegypti	SPECIES	22.61000	120.26000	1264883277
12927	Diptera	Culicidae	Aedes	Aedes aegypti	SPECIES	22.61000	120.28000	1264884657
12928	Diptera	Culicidae	Aedes	Aedes aegypti	SPECIES	22.61000	120.36000	1264885375
12929	Diptera	Culicidae	Aedes	Aedes aegypti	SPECIES	22.61000	120.30000	1264885662
12930	Diptera	Culicidae	Aedes	Aedes aegypti	SPECIES	22.61000	120.31000	1264885903
12931	Diptera	Culicidae	Aedes	Aedes aegypti	SPECIES	22.61000	120.27000	1264885921
12932	Diptera	Culicidae	Aedes	Aedes aegypti	SPECIES	22.61000	120.32000	1264885934
12933	Diptera	Culicidae	Aedes	Aedes aegypti	SPECIES	22.61000	120.34000	1264885937
12934	Diptera	Culicidae	Aedes	Aedes aegypti	SPECIES	22.61000	120.35000	1264885951
12935	Diptera	Culicidae	Aedes	Aedes aegypti	SPECIES	22.61000	120.33000	1264885983
12936	Diptera	Culicidae	Aedes	Aedes aegypti	SPECIES	22.61806	-100.39861	1894397567
12937	Diptera	Culicidae	Aedes	Aedes aegypti	SPECIES	22.62000	120.53000	1264876860
12938	Diptera	Culicidae	Aedes	Aedes aegypti	SPECIES	22.62000	120.42000	1264877030
12939	Diptera	Culicidae	Aedes	Aedes aegypti	SPECIES	22.62000	120.54000	1264877034
12940	Diptera	Culicidae	Aedes	Aedes aegypti	SPECIES	22.62000	120.41000	1264877038
12941	Diptera	Culicidae	Aedes	Aedes aegypti	SPECIES	22.62000	120.47000	1264877462
12942	Diptera	Culicidae	Aedes	Aedes aegypti	SPECIES	22.62000	120.61000	1264877600
12943	Diptera	Culicidae	Aedes	Aedes aegypti	SPECIES	22.62000	120.39000	1264884701
12944	Diptera	Culicidae	Aedes	Aedes aegypti	SPECIES	22.62000	120.36000	1264885537
12945	Diptera	Culicidae	Aedes	Aedes aegypti	SPECIES	22.62000	120.29000	1264885638
12946	Diptera	Culicidae	Aedes	Aedes aegypti	SPECIES	22.62000	120.37000	1264885667
12947	Diptera	Culicidae	Aedes	Aedes aegypti	SPECIES	22.62000	120.38000	1264885668
12948	Diptera	Culicidae	Aedes	Aedes aegypti	SPECIES	22.62000	120.34000	1264885840
12949	Diptera	Culicidae	Aedes	Aedes aegypti	SPECIES	22.62000	120.33000	1264885891
12950	Diptera	Culicidae	Aedes	Aedes aegypti	SPECIES	22.62000	120.32000	1264885961
12951	Diptera	Culicidae	Aedes	Aedes aegypti	SPECIES	22.62000	120.35000	1264886013
12952	Diptera	Culicidae	Aedes	Aedes aegypti	SPECIES	22.62000	120.30000	1264886290
12953	Diptera	Culicidae	Aedes	Aedes aegypti	SPECIES	22.62000	120.27000	1264889725
12954	Diptera	Culicidae	Aedes	Aedes aegypti	SPECIES	22.62000	120.31000	1264890022
12955	Diptera	Culicidae	Aedes	Aedes aegypti	SPECIES	22.62000	120.28000	1264890290
12956	Diptera	Culicidae	Aedes	Aedes aegypti	SPECIES	22.62600	120.31000	1264878692

№	Order	Family	Genus	Species	Taxon Rank	Latitude	Longitude	GBIF_ID
12957	Diptera	Culicidae	Aedes	Aedes aegypti	SPECIES	22.63000	120.50000	1264876842
12958	Diptera	Culicidae	Aedes	Aedes aegypti	SPECIES	22.63000	120.51000	1264876999
12959	Diptera	Culicidae	Aedes	Aedes aegypti	SPECIES	22.63000	120.67000	1264877047
12960	Diptera	Culicidae	Aedes	Aedes aegypti	SPECIES	22.63000	120.55000	1264877203
12961	Diptera	Culicidae	Aedes	Aedes aegypti	SPECIES	22.63000	120.53000	1264877303
12962	Diptera	Culicidae	Aedes	Aedes aegypti	SPECIES	22.63000	120.47000	1264877408
12963	Diptera	Culicidae	Aedes	Aedes aegypti	SPECIES	22.63000	120.48000	1264878928
12964	Diptera	Culicidae	Aedes	Aedes aegypti	SPECIES	22.63000	120.27000	1264884835
12965	Diptera	Culicidae	Aedes	Aedes aegypti	SPECIES	22.63000	120.28000	1264885535
12966	Diptera	Culicidae	Aedes	Aedes aegypti	SPECIES	22.63000	120.40000	1264885605
12967	Diptera	Culicidae	Aedes	Aedes aegypti	SPECIES	22.63000	120.38000	1264885852
12968	Diptera	Culicidae	Aedes	Aedes aegypti	SPECIES	22.63000	120.36000	1264885877
12969	Diptera	Culicidae	Aedes	Aedes aegypti	SPECIES	22.63000	120.34000	1264885955
12970	Diptera	Culicidae	Aedes	Aedes aegypti	SPECIES	22.63000	120.37000	1264885964
12971	Diptera	Culicidae	Aedes	Aedes aegypti	SPECIES	22.63000	120.29000	1264885966
12972	Diptera	Culicidae	Aedes	Aedes aegypti	SPECIES	22.63000	120.31000	1264885974
12973	Diptera	Culicidae	Aedes	Aedes aegypti	SPECIES	22.63000	120.32000	1264885978
12974	Diptera	Culicidae	Aedes	Aedes aegypti	SPECIES	22.63000	120.33000	1264886942
12975	Diptera	Culicidae	Aedes	Aedes aegypti	SPECIES	22.63000	120.30000	1264890028
12976	Diptera	Culicidae	Aedes	Aedes aegypti	SPECIES	22.63000	120.35000	1264891279
12977	Diptera	Culicidae	Aedes	Aedes aegypti	SPECIES	22.64000	120.61000	1264876870
12978	Diptera	Culicidae	Aedes	Aedes aegypti	SPECIES	22.64000	120.43000	1264876909
12979	Diptera	Culicidae	Aedes	Aedes aegypti	SPECIES	22.64000	120.58000	1264877018
12980	Diptera	Culicidae	Aedes	Aedes aegypti	SPECIES	22.64000	120.41000	1264877068
12981	Diptera	Culicidae	Aedes	Aedes aegypti	SPECIES	22.64000	120.52000	1264877342
12982	Diptera	Culicidae	Aedes	Aedes aegypti	SPECIES	22.64000	120.50000	1264877365
12983	Diptera	Culicidae	Aedes	Aedes aegypti	SPECIES	22.64000	120.45000	1264877412
12984	Diptera	Culicidae	Aedes	Aedes aegypti	SPECIES	22.64000	120.53000	1264877499
12985	Diptera	Culicidae	Aedes	Aedes aegypti	SPECIES	22.64000	120.48000	1264878502
12986	Diptera	Culicidae	Aedes	Aedes aegypti	SPECIES	22.64000	120.26000	1264882250
12987	Diptera	Culicidae	Aedes	Aedes aegypti	SPECIES	22.64000	120.27000	1264884984
12988	Diptera	Culicidae	Aedes	Aedes aegypti	SPECIES	22.64000	120.36000	1264885134
12989	Diptera	Culicidae	Aedes	Aedes aegypti	SPECIES	22.64000	120.28000	1264885301
12990	Diptera	Culicidae	Aedes	Aedes aegypti	SPECIES	22.64000	120.37000	1264885439
12991	Diptera	Culicidae	Aedes	Aedes aegypti	SPECIES	22.64000	120.39000	1264885823
12992	Diptera	Culicidae	Aedes	Aedes aegypti	SPECIES	22.64000	120.29000	1264885839
12993	Diptera	Culicidae	Aedes	Aedes aegypti	SPECIES	22.64000	120.31000	1264885890
12994	Diptera	Culicidae	Aedes	Aedes aegypti	SPECIES	22.64000	120.34000	1264885936
12995	Diptera	Culicidae	Aedes	Aedes aegypti	SPECIES	22.64000	120.33000	1264885980
12996	Diptera	Culicidae	Aedes	Aedes aegypti	SPECIES	22.64000	120.35000	1264886002
12997	Diptera	Culicidae	Aedes	Aedes aegypti	SPECIES	22.64000	120.30000	1264886090

№	Order	Family	Genus	Species	Taxon Rank	Latitude	Longitude	GBIF_ID
12998	Diptera	Culicidae	Aedes	Aedes aegypti	SPECIES	22.64000	120.32000	1264890017
12999	Diptera	Culicidae	Aedes	Aedes aegypti	SPECIES	22.65000	120.50000	1264876534
13000	Diptera	Culicidae	Aedes	Aedes aegypti	SPECIES	22.65000	120.54000	1264876798
13001	Diptera	Culicidae	Aedes	Aedes aegypti	SPECIES	22.65000	120.53000	1264876800
13002	Diptera	Culicidae	Aedes	Aedes aegypti	SPECIES	22.65000	120.59000	1264876950
13003	Diptera	Culicidae	Aedes	Aedes aegypti	SPECIES	22.65000	120.56000	1264876994
13004	Diptera	Culicidae	Aedes	Aedes aegypti	SPECIES	22.65000	120.66000	1264877133
13005	Diptera	Culicidae	Aedes	Aedes aegypti	SPECIES	22.65000	120.41000	1264877691
13006	Diptera	Culicidae	Aedes	Aedes aegypti	SPECIES	22.65000	120.49000	1264877761
13007	Diptera	Culicidae	Aedes	Aedes aegypti	SPECIES	22.65000	120.42000	1264879019
13008	Diptera	Culicidae	Aedes	Aedes aegypti	SPECIES	22.65000	120.45000	1264879250
13009	Diptera	Culicidae	Aedes	Aedes aegypti	SPECIES	22.65000	120.27000	1264880339
13010	Diptera	Culicidae	Aedes	Aedes aegypti	SPECIES	22.65000	120.39000	1264880826
13011	Diptera	Culicidae	Aedes	Aedes aegypti	SPECIES	22.65000	120.48000	1264884044
13012	Diptera	Culicidae	Aedes	Aedes aegypti	SPECIES	22.65000	120.28000	1264885339
13013	Diptera	Culicidae	Aedes	Aedes aegypti	SPECIES	22.65000	120.30000	1264885340
13014	Diptera	Culicidae	Aedes	Aedes aegypti	SPECIES	22.65000	120.34000	1264885447
13015	Diptera	Culicidae	Aedes	Aedes aegypti	SPECIES	22.65000	120.29000	1264885686
13016	Diptera	Culicidae	Aedes	Aedes aegypti	SPECIES	22.65000	120.31000	1264885901
13017	Diptera	Culicidae	Aedes	Aedes aegypti	SPECIES	22.65000	120.32000	1264885954
13018	Diptera	Culicidae	Aedes	Aedes aegypti	SPECIES	22.65000	120.33000	1264885993
13019	Diptera	Culicidae	Aedes	Aedes aegypti	SPECIES	22.65000	88.28000	1264889711
13020	Diptera	Culicidae	Aedes	Aedes aegypti	SPECIES	22.66000	120.36000	1264876684
13021	Diptera	Culicidae	Aedes	Aedes aegypti	SPECIES	22.66000	120.54000	1264876783
13022	Diptera	Culicidae	Aedes	Aedes aegypti	SPECIES	22.66000	120.61000	1264876827
13023	Diptera	Culicidae	Aedes	Aedes aegypti	SPECIES	22.66000	120.53000	1264876843
13024	Diptera	Culicidae	Aedes	Aedes aegypti	SPECIES	22.66000	120.42000	1264876903
13025	Diptera	Culicidae	Aedes	Aedes aegypti	SPECIES	22.66000	120.43000	1264877587
13026	Diptera	Culicidae	Aedes	Aedes aegypti	SPECIES	22.66000	120.51000	1264878896
13027	Diptera	Culicidae	Aedes	Aedes aegypti	SPECIES	22.66000	120.39000	1264878979
13028	Diptera	Culicidae	Aedes	Aedes aegypti	SPECIES	22.66000	120.45000	1264880225
13029	Diptera	Culicidae	Aedes	Aedes aegypti	SPECIES	22.66000	120.49000	1264880367
13030	Diptera	Culicidae	Aedes	Aedes aegypti	SPECIES	22.66000	120.27000	1264880588
13031	Diptera	Culicidae	Aedes	Aedes aegypti	SPECIES	22.66000	120.40000	1264881334
13032	Diptera	Culicidae	Aedes	Aedes aegypti	SPECIES	22.66000	120.34000	1264883487
13033	Diptera	Culicidae	Aedes	Aedes aegypti	SPECIES	22.66000	120.31000	1264884888
13034	Diptera	Culicidae	Aedes	Aedes aegypti	SPECIES	22.66000	120.30000	1264885034
13035	Diptera	Culicidae	Aedes	Aedes aegypti	SPECIES	22.66000	120.50000	1264885765
13036	Diptera	Culicidae	Aedes	Aedes aegypti	SPECIES	22.66000	120.33000	1264885801
13037	Diptera	Culicidae	Aedes	Aedes aegypti	SPECIES	22.66000	120.32000	1264885816
13038	Diptera	Culicidae	Aedes	Aedes aegypti	SPECIES	22.66000	120.29000	1264885922

№	Order	Family	Genus	Species	Taxon Rank	Latitude	Longitude	GBIF_ID
13039	Diptera	Culicidae	Aedes	Aedes aegypti	SPECIES	22.66000	120.28000	1264885968
13040	Diptera	Culicidae	Aedes	Aedes aegypti	SPECIES	22.66000	120.48000	1264885979
13041	Diptera	Culicidae	Aedes	Aedes aegypti	SPECIES	22.66000	70.76000	1264889274
13042	Diptera	Culicidae	Aedes	Aedes aegypti	SPECIES	22.66000	120.47000	1264890095
13043	Diptera	Culicidae	Aedes	Aedes aegypti	SPECIES	22.67000	120.73000	1264877224
13044	Diptera	Culicidae	Aedes	Aedes aegypti	SPECIES	22.67000	120.52000	1264877261
13045	Diptera	Culicidae	Aedes	Aedes aegypti	SPECIES	22.67000	120.45000	1264878311
13046	Diptera	Culicidae	Aedes	Aedes aegypti	SPECIES	22.67000	120.46000	1264878903
13047	Diptera	Culicidae	Aedes	Aedes aegypti	SPECIES	22.67000	120.51000	1264878960
13048	Diptera	Culicidae	Aedes	Aedes aegypti	SPECIES	22.67000	120.41000	1264880776
13049	Diptera	Culicidae	Aedes	Aedes aegypti	SPECIES	22.67000	120.36000	1264883424
13050	Diptera	Culicidae	Aedes	Aedes aegypti	SPECIES	22.67000	120.47000	1264883999
13051	Diptera	Culicidae	Aedes	Aedes aegypti	SPECIES	22.67000	120.27000	1264884834
13052	Diptera	Culicidae	Aedes	Aedes aegypti	SPECIES	22.67000	120.28000	1264885187
13053	Diptera	Culicidae	Aedes	Aedes aegypti	SPECIES	22.67000	120.32000	1264885194
13054	Diptera	Culicidae	Aedes	Aedes aegypti	SPECIES	22.67000	120.33000	1264885389
13055	Diptera	Culicidae	Aedes	Aedes aegypti	SPECIES	22.67000	120.30000	1264885440
13056	Diptera	Culicidae	Aedes	Aedes aegypti	SPECIES	22.67000	120.50000	1264885629
13057	Diptera	Culicidae	Aedes	Aedes aegypti	SPECIES	22.67000	120.31000	1264885659
13058	Diptera	Culicidae	Aedes	Aedes aegypti	SPECIES	22.67000	120.49000	1264885822
13059	Diptera	Culicidae	Aedes	Aedes aegypti	SPECIES	22.67000	120.29000	1264885831
13060	Diptera	Culicidae	Aedes	Aedes aegypti	SPECIES	22.67000	120.48000	1264886018
13061	Diptera	Culicidae	Aedes	Aedes aegypti	SPECIES	22.68000	120.77000	1264876560
13062	Diptera	Culicidae	Aedes	Aedes aegypti	SPECIES	22.68000	120.54000	1264876813
13063	Diptera	Culicidae	Aedes	Aedes aegypti	SPECIES	22.68000	120.52000	1264876844
13064	Diptera	Culicidae	Aedes	Aedes aegypti	SPECIES	22.68000	120.62000	1264877013
13065	Diptera	Culicidae	Aedes	Aedes aegypti	SPECIES	22.68000	120.65000	1264877017
13066	Diptera	Culicidae	Aedes	Aedes aegypti	SPECIES	22.68000	120.60000	1264877422
13067	Diptera	Culicidae	Aedes	Aedes aegypti	SPECIES	22.68000	120.59000	1264877642
13068	Diptera	Culicidae	Aedes	Aedes aegypti	SPECIES	22.68000	120.41000	1264877648
13069	Diptera	Culicidae	Aedes	Aedes aegypti	SPECIES	22.68000	120.36000	1264878452
13070	Diptera	Culicidae	Aedes	Aedes aegypti	SPECIES	22.68000	120.34000	1264880694
13071	Diptera	Culicidae	Aedes	Aedes aegypti	SPECIES	22.68000	120.45000	1264884555
13072	Diptera	Culicidae	Aedes	Aedes aegypti	SPECIES	22.68000	120.37000	1264884625
13073	Diptera	Culicidae	Aedes	Aedes aegypti	SPECIES	22.68000	120.30000	1264884827
13074	Diptera	Culicidae	Aedes	Aedes aegypti	SPECIES	22.68000	120.31000	1264885210
13075	Diptera	Culicidae	Aedes	Aedes aegypti	SPECIES	22.68000	120.35000	1264885518
13076	Diptera	Culicidae	Aedes	Aedes aegypti	SPECIES	22.68000	120.32000	1264885561
13077	Diptera	Culicidae	Aedes	Aedes aegypti	SPECIES	22.68000	120.29000	1264885685
13078	Diptera	Culicidae	Aedes	Aedes aegypti	SPECIES	22.68000	120.28000	1264885698
13079	Diptera	Culicidae	Aedes	Aedes aegypti	SPECIES	22.68000	120.50000	1264885919

№	Order	Family	Genus	Species	Taxon Rank	Latitude	Longitude	GBIF_ID
13080	Diptera	Culicidae	Aedes	Aedes aegypti	SPECIES	22.68000	120.48000	1264885952
13081	Diptera	Culicidae	Aedes	Aedes aegypti	SPECIES	22.68000	120.51000	1264885971
13082	Diptera	Culicidae	Aedes	Aedes aegypti	SPECIES	22.68000	120.49000	1264886070
13083	Diptera	Culicidae	Aedes	Aedes aegypti	SPECIES	22.69000	120.44000	1264876785
13084	Diptera	Culicidae	Aedes	Aedes aegypti	SPECIES	22.69000	120.61000	1264876834
13085	Diptera	Culicidae	Aedes	Aedes aegypti	SPECIES	22.69000	120.38000	1264877021
13086	Diptera	Culicidae	Aedes	Aedes aegypti	SPECIES	22.69000	120.63000	1264877037
13087	Diptera	Culicidae	Aedes	Aedes aegypti	SPECIES	22.69000	120.43000	1264877263
13088	Diptera	Culicidae	Aedes	Aedes aegypti	SPECIES	22.69000	120.54000	1264877464
13089	Diptera	Culicidae	Aedes	Aedes aegypti	SPECIES	22.69000	120.41000	1264877629
13090	Diptera	Culicidae	Aedes	Aedes aegypti	SPECIES	22.69000	120.33000	1264881220
13091	Diptera	Culicidae	Aedes	Aedes aegypti	SPECIES	22.69000	120.50000	1264882032
13092	Diptera	Culicidae	Aedes	Aedes aegypti	SPECIES	22.69000	120.30000	1264884907
13093	Diptera	Culicidae	Aedes	Aedes aegypti	SPECIES	22.69000	120.28000	1264884971
13094	Diptera	Culicidae	Aedes	Aedes aegypti	SPECIES	22.69000	120.29000	1264885042
13095	Diptera	Culicidae	Aedes	Aedes aegypti	SPECIES	22.69000	120.49000	1264885373
13096	Diptera	Culicidae	Aedes	Aedes aegypti	SPECIES	22.69000	120.32000	1264885413
13097	Diptera	Culicidae	Aedes	Aedes aegypti	SPECIES	22.69000	120.51000	1264885775
13098	Diptera	Culicidae	Aedes	Aedes aegypti	SPECIES	22.69000	120.36000	1264885906
13099	Diptera	Culicidae	Aedes	Aedes aegypti	SPECIES	22.69000	72.86000	1264888736
13100	Diptera	Culicidae	Aedes	Aedes aegypti	SPECIES	22.69639	-100.21833	1894395853
13101	Diptera	Culicidae	Aedes	Aedes aegypti	SPECIES	22.70000	120.57000	1264876816
13102	Diptera	Culicidae	Aedes	Aedes aegypti	SPECIES	22.70000	120.47000	1264876865
13103	Diptera	Culicidae	Aedes	Aedes aegypti	SPECIES	22.70000	120.50000	1264876988
13104	Diptera	Culicidae	Aedes	Aedes aegypti	SPECIES	22.70000	120.60000	1264877256
13105	Diptera	Culicidae	Aedes	Aedes aegypti	SPECIES	22.70000	120.55000	1264877259
13106	Diptera	Culicidae	Aedes	Aedes aegypti	SPECIES	22.70000	120.53000	1264877362
13107	Diptera	Culicidae	Aedes	Aedes aegypti	SPECIES	22.70000	120.29000	1264877563
13108	Diptera	Culicidae	Aedes	Aedes aegypti	SPECIES	22.70000	120.51000	1264877666
13109	Diptera	Culicidae	Aedes	Aedes aegypti	SPECIES	22.70000	120.43000	1264877673
13110	Diptera	Culicidae	Aedes	Aedes aegypti	SPECIES	22.70000	120.35000	1264878969
13111	Diptera	Culicidae	Aedes	Aedes aegypti	SPECIES	22.70000	120.66000	1264880600
13112	Diptera	Culicidae	Aedes	Aedes aegypti	SPECIES	22.70000	120.36000	1264883560
13113	Diptera	Culicidae	Aedes	Aedes aegypti	SPECIES	22.70000	120.30000	1264885625
13114	Diptera	Culicidae	Aedes	Aedes aegypti	SPECIES	22.70000	120.34000	1264885879
13115	Diptera	Culicidae	Aedes	Aedes aegypti	SPECIES	22.70000	71.68000	1264890733
13116	Diptera	Culicidae	Aedes	Aedes aegypti	SPECIES	22.71000	120.54000	1264876821
13117	Diptera	Culicidae	Aedes	Aedes aegypti	SPECIES	22.71000	120.52000	1264877200
13118	Diptera	Culicidae	Aedes	Aedes aegypti	SPECIES	22.71000	120.34000	1264877234
13119	Diptera	Culicidae	Aedes	Aedes aegypti	SPECIES	22.71000	120.59000	1264877367
13120	Diptera	Culicidae	Aedes	Aedes aegypti	SPECIES	22.71000	120.57000	1264877403

№	Order	Family	Genus	Species	Taxon Rank	Latitude	Longitude	GBIF_ID
13121	Diptera	Culicidae	Aedes	Aedes aegypti	SPECIES	22.71000	120.56000	1264877436
13122	Diptera	Culicidae	Aedes	Aedes aegypti	SPECIES	22.71000	120.38000	1264877634
13123	Diptera	Culicidae	Aedes	Aedes aegypti	SPECIES	22.71000	120.33000	1264881199
13124	Diptera	Culicidae	Aedes	Aedes aegypti	SPECIES	22.71000	120.31000	1264882793
13125	Diptera	Culicidae	Aedes	Aedes aegypti	SPECIES	22.71000	120.64000	1264883782
13126	Diptera	Culicidae	Aedes	Aedes aegypti	SPECIES	22.71000	120.32000	1264883797
13127	Diptera	Culicidae	Aedes	Aedes aegypti	SPECIES	22.71000	120.30000	1264885637
13128	Diptera	Culicidae	Aedes	Aedes aegypti	SPECIES	22.71000	120.28000	1264885708
13129	Diptera	Culicidae	Aedes	Aedes aegypti	SPECIES	22.71000	120.29000	1264885925
13130	Diptera	Culicidae	Aedes	Aedes aegypti	SPECIES	22.71000	71.67000	1264889304
13131	Diptera	Culicidae	Aedes	Aedes aegypti	SPECIES	22.71917	-98.88111	1894398368
13132	Diptera	Culicidae	Aedes	Aedes aegypti	SPECIES	22.72000	120.45000	1264876939
13133	Diptera	Culicidae	Aedes	Aedes aegypti	SPECIES	22.72000	120.69000	1264877253
13134	Diptera	Culicidae	Aedes	Aedes aegypti	SPECIES	22.72000	120.51000	1264877659
13135	Diptera	Culicidae	Aedes	Aedes aegypti	SPECIES	22.72000	120.26000	1264880296
13136	Diptera	Culicidae	Aedes	Aedes aegypti	SPECIES	22.72000	120.42000	1264880761
13137	Diptera	Culicidae	Aedes	Aedes aegypti	SPECIES	22.72000	120.36000	1264880766
13138	Diptera	Culicidae	Aedes	Aedes aegypti	SPECIES	22.72000	120.27000	1264881362
13139	Diptera	Culicidae	Aedes	Aedes aegypti	SPECIES	22.72000	120.31000	1264883889
13140	Diptera	Culicidae	Aedes	Aedes aegypti	SPECIES	22.72000	120.34000	1264884777
13141	Diptera	Culicidae	Aedes	Aedes aegypti	SPECIES	22.72000	120.28000	1264884832
13142	Diptera	Culicidae	Aedes	Aedes aegypti	SPECIES	22.72000	120.33000	1264885244
13143	Diptera	Culicidae	Aedes	Aedes aegypti	SPECIES	22.72000	120.32000	1264885255
13144	Diptera	Culicidae	Aedes	Aedes aegypti	SPECIES	22.72000	120.29000	1264885458
13145	Diptera	Culicidae	Aedes	Aedes aegypti	SPECIES	22.72000	120.30000	1264885832
13146	Diptera	Culicidae	Aedes	Aedes aegypti	SPECIES	22.73000	120.56000	1264877457
13147	Diptera	Culicidae	Aedes	Aedes aegypti	SPECIES	22.73000	120.45000	1264877627
13148	Diptera	Culicidae	Aedes	Aedes aegypti	SPECIES	22.73000	120.49000	1264877676
13149	Diptera	Culicidae	Aedes	Aedes aegypti	SPECIES	22.73000	120.55000	1264877688
13150	Diptera	Culicidae	Aedes	Aedes aegypti	SPECIES	22.73000	120.50000	1264878950
13151	Diptera	Culicidae	Aedes	Aedes aegypti	SPECIES	22.73000	120.29000	1264879874
13152	Diptera	Culicidae	Aedes	Aedes aegypti	SPECIES	22.73000	120.36000	1264881174
13153	Diptera	Culicidae	Aedes	Aedes aegypti	SPECIES	22.73000	120.26000	1264881212
13154	Diptera	Culicidae	Aedes	Aedes aegypti	SPECIES	22.73000	120.37000	1264882858
13155	Diptera	Culicidae	Aedes	Aedes aegypti	SPECIES	22.73000	120.66000	1264884296
13156	Diptera	Culicidae	Aedes	Aedes aegypti	SPECIES	22.73000	120.25000	1264884820
13157	Diptera	Culicidae	Aedes	Aedes aegypti	SPECIES	22.73000	120.34000	1264884889
13158	Diptera	Culicidae	Aedes	Aedes aegypti	SPECIES	22.73000	120.30000	1264885147
13159	Diptera	Culicidae	Aedes	Aedes aegypti	SPECIES	22.73000	120.32000	1264885570
13160	Diptera	Culicidae	Aedes	Aedes aegypti	SPECIES	22.73000	120.33000	1264886004
13161	Diptera	Culicidae	Aedes	Aedes aegypti	SPECIES	22.74000	120.63000	1264877007

№	Order	Family	Genus	Species	Taxon Rank	Latitude	Longitude	GBIF_ID
13162	Diptera	Culicidae	Aedes	Aedes aegypti	SPECIES	22.74000	120.72000	1264877069
13163	Diptera	Culicidae	Aedes	Aedes aegypti	SPECIES	22.74000	120.33000	1264877185
13164	Diptera	Culicidae	Aedes	Aedes aegypti	SPECIES	22.74000	120.34000	1264877345
13165	Diptera	Culicidae	Aedes	Aedes aegypti	SPECIES	22.74000	120.49000	1264877616
13166	Diptera	Culicidae	Aedes	Aedes aegypti	SPECIES	22.74000	120.43000	1264877668
13167	Diptera	Culicidae	Aedes	Aedes aegypti	SPECIES	22.74000	120.47000	1264878443
13168	Diptera	Culicidae	Aedes	Aedes aegypti	SPECIES	22.74000	120.29000	1264879506
13169	Diptera	Culicidae	Aedes	Aedes aegypti	SPECIES	22.74000	120.37000	1264880744
13170	Diptera	Culicidae	Aedes	Aedes aegypti	SPECIES	22.74000	120.26000	1264881167
13171	Diptera	Culicidae	Aedes	Aedes aegypti	SPECIES	22.74000	120.36000	1264881597
13172	Diptera	Culicidae	Aedes	Aedes aegypti	SPECIES	22.74778	-105.08611	1894395982
13173	Diptera	Culicidae	Aedes	Aedes aegypti	SPECIES	22.75000	120.50000	1264876850
13174	Diptera	Culicidae	Aedes	Aedes aegypti	SPECIES	22.75000	120.65000	1264877637
13175	Diptera	Culicidae	Aedes	Aedes aegypti	SPECIES	22.75000	120.30000	1264880310
13176	Diptera	Culicidae	Aedes	Aedes aegypti	SPECIES	22.75000	120.25000	1264880954
13177	Diptera	Culicidae	Aedes	Aedes aegypti	SPECIES	22.75000	120.26000	1264881650
13178	Diptera	Culicidae	Aedes	Aedes aegypti	SPECIES	22.75000	120.35000	1264884629
13179	Diptera	Culicidae	Aedes	Aedes aegypti	SPECIES	22.75000	121.16000	1264890038
13180	Diptera	Culicidae	Aedes	Aedes aegypti	SPECIES	22.76000	120.46000	1264876801
13181	Diptera	Culicidae	Aedes	Aedes aegypti	SPECIES	22.76000	120.49000	1264876896
13182	Diptera	Culicidae	Aedes	Aedes aegypti	SPECIES	22.76000	120.48000	1264877286
13183	Diptera	Culicidae	Aedes	Aedes aegypti	SPECIES	22.76000	120.52000	1264877414
13184	Diptera	Culicidae	Aedes	Aedes aegypti	SPECIES	22.76000	120.51000	1264877620
13185	Diptera	Culicidae	Aedes	Aedes aegypti	SPECIES	22.76000	120.58000	1264877757
13186	Diptera	Culicidae	Aedes	Aedes aegypti	SPECIES	22.76000	120.30000	1264878863
13187	Diptera	Culicidae	Aedes	Aedes aegypti	SPECIES	22.76000	120.31000	1264883302
13188	Diptera	Culicidae	Aedes	Aedes aegypti	SPECIES	22.76000	120.44000	1264884912
13189	Diptera	Culicidae	Aedes	Aedes aegypti	SPECIES	22.76000	121.16000	1264889995
13190	Diptera	Culicidae	Aedes	Aedes aegypti	SPECIES	22.76000	121.14000	1264890064
13191	Diptera	Culicidae	Aedes	Aedes aegypti	SPECIES	22.76333	-101.50611	1894394902
13192	Diptera	Culicidae	Aedes	Aedes aegypti	SPECIES	22.77000	120.66000	1264876773
13193	Diptera	Culicidae	Aedes	Aedes aegypti	SPECIES	22.77000	120.69000	1264876805
13194	Diptera	Culicidae	Aedes	Aedes aegypti	SPECIES	22.77000	120.62000	1264877614
13195	Diptera	Culicidae	Aedes	Aedes aegypti	SPECIES	22.77000	120.39000	1264880909
13196	Diptera	Culicidae	Aedes	Aedes aegypti	SPECIES	22.77000	120.50000	1264883703
13197	Diptera	Culicidae	Aedes	Aedes aegypti	SPECIES	22.78000	120.57000	1264877213
13198	Diptera	Culicidae	Aedes	Aedes aegypti	SPECIES	22.78000	120.59000	1264877221
13199	Diptera	Culicidae	Aedes	Aedes aegypti	SPECIES	22.78000	120.51000	1264877246
13200	Diptera	Culicidae	Aedes	Aedes aegypti	SPECIES	22.78000	120.23000	1264877574
13201	Diptera	Culicidae	Aedes	Aedes aegypti	SPECIES	22.78000	120.26000	1264878209
13202	Diptera	Culicidae	Aedes	Aedes aegypti	SPECIES	22.78000	120.49000	1264880655

№	Order	Family	Genus	Species	Taxon Rank	Latitude	Longitude	GBIF_ID
13203	Diptera	Culicidae	Aedes	Aedes aegypti	SPECIES	22.78000	120.50000	1264880662
13204	Diptera	Culicidae	Aedes	Aedes aegypti	SPECIES	22.78000	120.25000	1264881471
13205	Diptera	Culicidae	Aedes	Aedes aegypti	SPECIES	22.78000	120.75000	1264888270
13206	Diptera	Culicidae	Aedes	Aedes aegypti	SPECIES	22.79000	120.74000	1264876841
13207	Diptera	Culicidae	Aedes	Aedes aegypti	SPECIES	22.79000	120.53000	1264877653
13208	Diptera	Culicidae	Aedes	Aedes aegypti	SPECIES	22.79000	120.50000	1264880645
13209	Diptera	Culicidae	Aedes	Aedes aegypti	SPECIES	22.79000	120.24000	1264881162
13210	Diptera	Culicidae	Aedes	Aedes aegypti	SPECIES	22.79000	120.25000	1264881264
13211	Diptera	Culicidae	Aedes	Aedes aegypti	SPECIES	22.79000	120.30000	1264883291
13212	Diptera	Culicidae	Aedes	Aedes aegypti	SPECIES	22.79000	120.29000	1264883322
13213	Diptera	Culicidae	Aedes	Aedes aegypti	SPECIES	22.79000	120.37000	1264884933
13214	Diptera	Culicidae	Aedes	Aedes aegypti	SPECIES	22.79000	120.31000	1264885251
13215	Diptera	Culicidae	Aedes	Aedes aegypti	SPECIES	22.79583	-100.16222	1894397280
13216	Diptera	Culicidae	Aedes	Aedes aegypti	SPECIES	22.80000	120.25000	1264876933
13217	Diptera	Culicidae	Aedes	Aedes aegypti	SPECIES	22.80000	120.48000	1264877008
13218	Diptera	Culicidae	Aedes	Aedes aegypti	SPECIES	22.80000	120.31000	1264881191
13219	Diptera	Culicidae	Aedes	Aedes aegypti	SPECIES	22.80000	120.27000	1264881448
13220	Diptera	Culicidae	Aedes	Aedes aegypti	SPECIES	22.80000	120.33000	1264881718
13221	Diptera	Culicidae	Aedes	Aedes aegypti	SPECIES	22.80000	120.36000	1264883407
13222	Diptera	Culicidae	Aedes	Aedes aegypti	SPECIES	22.80000	120.29000	1264885291
13223	Diptera	Culicidae	Aedes	Aedes aegypti	SPECIES	22.80000	120.30000	1264890009
13224	Diptera	Culicidae	Aedes	Aedes aegypti	SPECIES	22.80000	120.28000	1264890065
13225	Diptera	Culicidae	Aedes	Aedes aegypti	SPECIES	22.81000	120.51000	1264876780
13226	Diptera	Culicidae	Aedes	Aedes aegypti	SPECIES	22.81000	120.57000	1264877237
13227	Diptera	Culicidae	Aedes	Aedes aegypti	SPECIES	22.81000	120.61000	1264877308
13228	Diptera	Culicidae	Aedes	Aedes aegypti	SPECIES	22.81000	120.21000	1264877654
13229	Diptera	Culicidae	Aedes	Aedes aegypti	SPECIES	22.81000	120.27000	1264880663
13230	Diptera	Culicidae	Aedes	Aedes aegypti	SPECIES	22.81000	120.23000	1264884885
13231	Diptera	Culicidae	Aedes	Aedes aegypti	SPECIES	22.82000	120.22000	1264876629
13232	Diptera	Culicidae	Aedes	Aedes aegypti	SPECIES	22.82000	120.59000	1264876823
13233	Diptera	Culicidae	Aedes	Aedes aegypti	SPECIES	22.82000	120.66000	1264876907
13234	Diptera	Culicidae	Aedes	Aedes aegypti	SPECIES	22.82000	120.51000	1264877431
13235	Diptera	Culicidae	Aedes	Aedes aegypti	SPECIES	22.82000	120.61000	1264878541
13236	Diptera	Culicidae	Aedes	Aedes aegypti	SPECIES	22.83000	120.24000	1264876924
13237	Diptera	Culicidae	Aedes	Aedes aegypti	SPECIES	22.83000	120.58000	1264877270
13238	Diptera	Culicidae	Aedes	Aedes aegypti	SPECIES	22.83000	108.31000	1264889140
13239	Diptera	Culicidae	Aedes	Aedes aegypti	SPECIES	22.84000	120.59000	1264876818
13240	Diptera	Culicidae	Aedes	Aedes aegypti	SPECIES	22.84000	120.53000	1264876987
13241	Diptera	Culicidae	Aedes	Aedes aegypti	SPECIES	22.84000	120.72000	1264877354
13242	Diptera	Culicidae	Aedes	Aedes aegypti	SPECIES	22.84000	120.43000	1264880687
13243	Diptera	Culicidae	Aedes	Aedes aegypti	SPECIES	22.84000	120.26000	1264885223

№	Order	Family	Genus	Species	Taxon Rank	Latitude	Longitude	GBIF_ID
13244	Diptera	Culicidae	Aedes	Aedes aegypti	SPECIES	22.84167	-99.67083	1894398406
13245	Diptera	Culicidae	Aedes	Aedes aegypti	SPECIES	22.85000	120.46000	1264876884
13246	Diptera	Culicidae	Aedes	Aedes aegypti	SPECIES	22.85000	120.55000	1264876904
13247	Diptera	Culicidae	Aedes	Aedes aegypti	SPECIES	22.85000	120.65000	1264876970
13248	Diptera	Culicidae	Aedes	Aedes aegypti	SPECIES	22.85000	120.31000	1264880566
13249	Diptera	Culicidae	Aedes	Aedes aegypti	SPECIES	22.85000	120.27000	1264881253
13250	Diptera	Culicidae	Aedes	Aedes aegypti	SPECIES	22.86000	120.57000	1264876977
13251	Diptera	Culicidae	Aedes	Aedes aegypti	SPECIES	22.86000	120.25000	1264880584
13252	Diptera	Culicidae	Aedes	Aedes aegypti	SPECIES	22.86000	120.23000	1264885970
13253	Diptera	Culicidae	Aedes	Aedes aegypti	SPECIES	22.87000	120.35000	1264876965
13254	Diptera	Culicidae	Aedes	Aedes aegypti	SPECIES	22.87000	120.64000	1264877254
13255	Diptera	Culicidae	Aedes	Aedes aegypti	SPECIES	22.87000	120.27000	1264879948
13256	Diptera	Culicidae	Aedes	Aedes aegypti	SPECIES	22.87000	120.26000	1264881180
13257	Diptera	Culicidae	Aedes	Aedes aegypti	SPECIES	22.87000	120.25000	1264885171
13258	Diptera	Culicidae	Aedes	Aedes aegypti	SPECIES	22.87000	-82.21000	1264890944
13259	Diptera	Culicidae	Aedes	Aedes aegypti	SPECIES	22.88000	120.53000	1264876877
13260	Diptera	Culicidae	Aedes	Aedes aegypti	SPECIES	22.88000	120.40000	1264876946
13261	Diptera	Culicidae	Aedes	Aedes aegypti	SPECIES	22.88000	120.21000	1264878121
13262	Diptera	Culicidae	Aedes	Aedes aegypti	SPECIES	22.88000	120.33000	1264880812
13263	Diptera	Culicidae	Aedes	Aedes aegypti	SPECIES	22.88000	120.49000	1264881329
13264	Diptera	Culicidae	Aedes	Aedes aegypti	SPECIES	22.88000	120.47000	1264885982
13265	Diptera	Culicidae	Aedes	Aedes aegypti	SPECIES	22.89000	120.32000	1264876546
13266	Diptera	Culicidae	Aedes	Aedes aegypti	SPECIES	22.89000	120.25000	1264879142
13267	Diptera	Culicidae	Aedes	Aedes aegypti	SPECIES	22.89000	120.55000	1264880651
13268	Diptera	Culicidae	Aedes	Aedes aegypti	SPECIES	22.89000	120.49000	1264880753
13269	Diptera	Culicidae	Aedes	Aedes aegypti	SPECIES	22.89000	120.33000	1264881277
13270	Diptera	Culicidae	Aedes	Aedes aegypti	SPECIES	22.89000	120.19000	1264881855
13271	Diptera	Culicidae	Aedes	Aedes aegypti	SPECIES	22.89000	120.48000	1264884906
13272	Diptera	Culicidae	Aedes	Aedes aegypti	SPECIES	22.89000	120.47000	1264884932
13273	Diptera	Culicidae	Aedes	Aedes aegypti	SPECIES	22.90000	120.40000	1264876571
13274	Diptera	Culicidae	Aedes	Aedes aegypti	SPECIES	22.90000	120.55000	1264877272
13275	Diptera	Culicidae	Aedes	Aedes aegypti	SPECIES	22.90000	120.54000	1264877276
13276	Diptera	Culicidae	Aedes	Aedes aegypti	SPECIES	22.90000	120.20000	1264880701
13277	Diptera	Culicidae	Aedes	Aedes aegypti	SPECIES	22.90000	120.18000	1264881656
13278	Diptera	Culicidae	Aedes	Aedes aegypti	SPECIES	22.90000	120.19000	1264884821
13279	Diptera	Culicidae	Aedes	Aedes aegypti	SPECIES	22.91000	120.61000	1264876612
13280	Diptera	Culicidae	Aedes	Aedes aegypti	SPECIES	22.91000	120.29000	1264876621
13281	Diptera	Culicidae	Aedes	Aedes aegypti	SPECIES	22.91000	120.48000	1264878911
13282	Diptera	Culicidae	Aedes	Aedes aegypti	SPECIES	22.91000	120.19000	1264881661
13283	Diptera	Culicidae	Aedes	Aedes aegypti	SPECIES	22.91000	120.18000	1264884809
13284	Diptera	Culicidae	Aedes	Aedes aegypti	SPECIES	22.91417	-98.97500	2629263507

№	Order	Family	Genus	Species	Taxon Rank	Latitude	Longitude	GBIF_ID
13285	Diptera	Culicidae	Aedes	Aedes aegypti	SPECIES	22.92000	120.21000	1264876632
13286	Diptera	Culicidae	Aedes	Aedes aegypti	SPECIES	22.92000	120.18000	1264882789
13287	Diptera	Culicidae	Aedes	Aedes aegypti	SPECIES	22.93000	120.19000	1264878612
13288	Diptera	Culicidae	Aedes	Aedes aegypti	SPECIES	22.93000	120.18000	1264883649
13289	Diptera	Culicidae	Aedes	Aedes aegypti	SPECIES	22.93333	-101.09278	1894397312
13290	Diptera	Culicidae	Aedes	Aedes aegypti	SPECIES	22.94000	120.23000	1264876759
13291	Diptera	Culicidae	Aedes	Aedes aegypti	SPECIES	22.94000	120.47000	1264876868
13292	Diptera	Culicidae	Aedes	Aedes aegypti	SPECIES	22.94000	120.19000	1264877132
13293	Diptera	Culicidae	Aedes	Aedes aegypti	SPECIES	22.94000	120.18000	1264877140
13294	Diptera	Culicidae	Aedes	Aedes aegypti	SPECIES	22.94000	97.75000	1264890094
13295	Diptera	Culicidae	Aedes	Aedes aegypti	SPECIES	22.95000	120.24000	1264876576
13296	Diptera	Culicidae	Aedes	Aedes aegypti	SPECIES	22.95000	120.28000	1264876636
13297	Diptera	Culicidae	Aedes	Aedes aegypti	SPECIES	22.95000	120.61000	1264877264
13298	Diptera	Culicidae	Aedes	Aedes aegypti	SPECIES	22.95000	120.34000	1264878861
13299	Diptera	Culicidae	Aedes	Aedes aegypti	SPECIES	22.95000	120.22000	1264879070
13300	Diptera	Culicidae	Aedes	Aedes aegypti	SPECIES	22.95000	120.26000	1264879268
13301	Diptera	Culicidae	Aedes	Aedes aegypti	SPECIES	22.95000	120.33000	1264880209
13302	Diptera	Culicidae	Aedes	Aedes aegypti	SPECIES	22.95000	120.18000	1264880513
13303	Diptera	Culicidae	Aedes	Aedes aegypti	SPECIES	22.95000	120.42000	1264883401
13304	Diptera	Culicidae	Aedes	Aedes aegypti	SPECIES	22.96000	120.47000	1264876898
13305	Diptera	Culicidae	Aedes	Aedes aegypti	SPECIES	22.96000	120.28000	1264876985
13306	Diptera	Culicidae	Aedes	Aedes aegypti	SPECIES	22.96000	120.17000	1264878848
13307	Diptera	Culicidae	Aedes	Aedes aegypti	SPECIES	22.96000	120.30000	1264878945
13308	Diptera	Culicidae	Aedes	Aedes aegypti	SPECIES	22.96000	120.32000	1264879264
13309	Diptera	Culicidae	Aedes	Aedes aegypti	SPECIES	22.96000	120.22000	1264880275
13310	Diptera	Culicidae	Aedes	Aedes aegypti	SPECIES	22.96000	120.34000	1264880802
13311	Diptera	Culicidae	Aedes	Aedes aegypti	SPECIES	22.96000	120.18000	1264883799
13312	Diptera	Culicidae	Aedes	Aedes aegypti	SPECIES	22.96000	120.20000	1264884289
13313	Diptera	Culicidae	Aedes	Aedes aegypti	SPECIES	22.96000	120.19000	1264884574
13314	Diptera	Culicidae	Aedes	Aedes aegypti	SPECIES	22.96000	120.23000	1264885794
13315	Diptera	Culicidae	Aedes	Aedes aegypti	SPECIES	22.96000	120.33000	1264886974
13316	Diptera	Culicidae	Aedes	Aedes aegypti	SPECIES	22.97000	120.35000	1264877696
13317	Diptera	Culicidae	Aedes	Aedes aegypti	SPECIES	22.97000	120.33000	1264878869
13318	Diptera	Culicidae	Aedes	Aedes aegypti	SPECIES	22.97000	120.20000	1264879328
13319	Diptera	Culicidae	Aedes	Aedes aegypti	SPECIES	22.97000	120.29000	1264879688
13320	Diptera	Culicidae	Aedes	Aedes aegypti	SPECIES	22.97000	120.32000	1264880527
13321	Diptera	Culicidae	Aedes	Aedes aegypti	SPECIES	22.97000	120.64000	1264881297
13322	Diptera	Culicidae	Aedes	Aedes aegypti	SPECIES	22.97000	120.34000	1264881306
13323	Diptera	Culicidae	Aedes	Aedes aegypti	SPECIES	22.97000	120.30000	1264883982
13324	Diptera	Culicidae	Aedes	Aedes aegypti	SPECIES	22.97000	120.31000	1264884001
13325	Diptera	Culicidae	Aedes	Aedes aegypti	SPECIES	22.97000	120.23000	1264885205

№	Order	Family	Genus	Species	Taxon Rank	Latitude	Longitude	GBIF_ID
13326	Diptera	Culicidae	Aedes	Aedes aegypti	SPECIES	22.97000	120.21000	1264885545
13327	Diptera	Culicidae	Aedes	Aedes aegypti	SPECIES	22.97000	120.24000	1264885607
13328	Diptera	Culicidae	Aedes	Aedes aegypti	SPECIES	22.97000	120.22000	1264885935
13329	Diptera	Culicidae	Aedes	Aedes aegypti	SPECIES	22.97000	120.19000	1264885956
13330	Diptera	Culicidae	Aedes	Aedes aegypti	SPECIES	22.97000	120.18000	1264890053
13331	Diptera	Culicidae	Aedes	Aedes aegypti	SPECIES	22.98000	120.16000	1264877643
13332	Diptera	Culicidae	Aedes	Aedes aegypti	SPECIES	22.98000	120.35000	1264878622
13333	Diptera	Culicidae	Aedes	Aedes aegypti	SPECIES	22.98000	120.17000	1264880546
13334	Diptera	Culicidae	Aedes	Aedes aegypti	SPECIES	22.98000	120.19000	1264883554
13335	Diptera	Culicidae	Aedes	Aedes aegypti	SPECIES	22.98000	120.24000	1264884176
13336	Diptera	Culicidae	Aedes	Aedes aegypti	SPECIES	22.98000	120.25000	1264885507
13337	Diptera	Culicidae	Aedes	Aedes aegypti	SPECIES	22.98000	120.21000	1264885575
13338	Diptera	Culicidae	Aedes	Aedes aegypti	SPECIES	22.98000	120.23000	1264885624
13339	Diptera	Culicidae	Aedes	Aedes aegypti	SPECIES	22.98000	120.22000	1264885892
13340	Diptera	Culicidae	Aedes	Aedes aegypti	SPECIES	22.98000	71.97000	1264889285
13341	Diptera	Culicidae	Aedes	Aedes aegypti	SPECIES	22.98000	120.20000	1264890018
13342	Diptera	Culicidae	Aedes	Aedes aegypti	SPECIES	22.98000	72.38000	1264891398
13343	Diptera	Culicidae	Aedes	Aedes aegypti	SPECIES	22.99000	120.16000	1264878967
13344	Diptera	Culicidae	Aedes	Aedes aegypti	SPECIES	22.99000	120.66000	1264880793
13345	Diptera	Culicidae	Aedes	Aedes aegypti	SPECIES	22.99000	120.18000	1264883228
13346	Diptera	Culicidae	Aedes	Aedes aegypti	SPECIES	22.99000	120.17000	1264883573
13347	Diptera	Culicidae	Aedes	Aedes aegypti	SPECIES	22.99000	120.24000	1264884545
13348	Diptera	Culicidae	Aedes	Aedes aegypti	SPECIES	22.99000	120.21000	1264885263
13349	Diptera	Culicidae	Aedes	Aedes aegypti	SPECIES	22.99000	120.25000	1264885300
13350	Diptera	Culicidae	Aedes	Aedes aegypti	SPECIES	22.99000	120.23000	1264885697
13351	Diptera	Culicidae	Aedes	Aedes aegypti	SPECIES	22.99000	120.22000	1264885889
13352	Diptera	Culicidae	Aedes	Aedes aegypti	SPECIES	22.99000	-82.41000	1264887140
13353	Diptera	Culicidae	Aedes	Aedes aegypti	SPECIES	22.99000	120.20000	1264889865
13354	Diptera	Culicidae	Aedes	Aedes aegypti	SPECIES	22.99000	120.19000	1264890034
13355	Diptera	Culicidae	Aedes	Aedes aegypti	SPECIES	22.99097	-99.71892	1895893000
13356	Diptera	Culicidae	Aedes	Aedes aegypti	SPECIES	22.99583	-98.94472	1894399007
13357	Diptera	Culicidae	Aedes	Aedes aegypti	SPECIES	23.00000	120.31000	1264876566
13358	Diptera	Culicidae	Aedes	Aedes aegypti	SPECIES	23.00000	120.26000	1264876791
13359	Diptera	Culicidae	Aedes	Aedes aegypti	SPECIES	23.00000	120.28000	1264876956
13360	Diptera	Culicidae	Aedes	Aedes aegypti	SPECIES	23.00000	120.15000	1264877754
13361	Diptera	Culicidae	Aedes	Aedes aegypti	SPECIES	23.00000	120.18000	1264878491
13362	Diptera	Culicidae	Aedes	Aedes aegypti	SPECIES	23.00000	120.64000	1264878823
13363	Diptera	Culicidae	Aedes	Aedes aegypti	SPECIES	23.00000	120.65000	1264878868
13364	Diptera	Culicidae	Aedes	Aedes aegypti	SPECIES	23.00000	120.17000	1264878965
13365	Diptera	Culicidae	Aedes	Aedes aegypti	SPECIES	23.00000	120.25000	1264879709
13366	Diptera	Culicidae	Aedes	Aedes aegypti	SPECIES	23.00000	120.24000	1264879894

№	Order	Family	Genus	Species	Taxon Rank	Latitude	Longitude	GBIF_ID
13367	Diptera	Culicidae	Aedes	Aedes aegypti	SPECIES	23.00000	120.16000	1264880258
13368	Diptera	Culicidae	Aedes	Aedes aegypti	SPECIES	23.00000	120.21000	1264883596
13369	Diptera	Culicidae	Aedes	Aedes aegypti	SPECIES	23.00000	120.19000	1264885489
13370	Diptera	Culicidae	Aedes	Aedes aegypti	SPECIES	23.00000	120.22000	1264885551
13371	Diptera	Culicidae	Aedes	Aedes aegypti	SPECIES	23.00000	120.23000	1264885909
13372	Diptera	Culicidae	Aedes	Aedes aegypti	SPECIES	23.00000	120.20000	1264889998
13373	Diptera	Culicidae	Aedes	Aedes aegypti	SPECIES	23.00278	-99.70833	1894399931
13374	Diptera	Culicidae	Aedes	Aedes aegypti	SPECIES	23.01000	120.13000	1264877056
13375	Diptera	Culicidae	Aedes	Aedes aegypti	SPECIES	23.01000	120.17000	1264879064
13376	Diptera	Culicidae	Aedes	Aedes aegypti	SPECIES	23.01000	120.26000	1264879538
13377	Diptera	Culicidae	Aedes	Aedes aegypti	SPECIES	23.01000	120.27000	1264879920
13378	Diptera	Culicidae	Aedes	Aedes aegypti	SPECIES	23.01000	120.25000	1264879978
13379	Diptera	Culicidae	Aedes	Aedes aegypti	SPECIES	23.01000	120.24000	1264880030
13380	Diptera	Culicidae	Aedes	Aedes aegypti	SPECIES	23.01000	120.19000	1264883415
13381	Diptera	Culicidae	Aedes	Aedes aegypti	SPECIES	23.01000	120.18000	1264883447
13382	Diptera	Culicidae	Aedes	Aedes aegypti	SPECIES	23.01000	120.20000	1264884054
13383	Diptera	Culicidae	Aedes	Aedes aegypti	SPECIES	23.01000	120.23000	1264884134
13384	Diptera	Culicidae	Aedes	Aedes aegypti	SPECIES	23.01000	120.21000	1264884768
13385	Diptera	Culicidae	Aedes	Aedes aegypti	SPECIES	23.01000	-82.40000	1264887845
13386	Diptera	Culicidae	Aedes	Aedes aegypti	SPECIES	23.01000	120.22000	1264890039
13387	Diptera	Culicidae	Aedes	Aedes aegypti	SPECIES	23.02000	120.33000	1264877152
13388	Diptera	Culicidae	Aedes	Aedes aegypti	SPECIES	23.02000	120.24000	1264879486
13389	Diptera	Culicidae	Aedes	Aedes aegypti	SPECIES	23.02000	120.26000	1264879509
13390	Diptera	Culicidae	Aedes	Aedes aegypti	SPECIES	23.02000	120.23000	1264879985
13391	Diptera	Culicidae	Aedes	Aedes aegypti	SPECIES	23.02000	120.25000	1264879995
13392	Diptera	Culicidae	Aedes	Aedes aegypti	SPECIES	23.02000	120.21000	1264880420
13393	Diptera	Culicidae	Aedes	Aedes aegypti	SPECIES	23.02000	120.28000	1264881245
13394	Diptera	Culicidae	Aedes	Aedes aegypti	SPECIES	23.02000	120.18000	1264883919
13395	Diptera	Culicidae	Aedes	Aedes aegypti	SPECIES	23.02000	120.19000	1264884217
13396	Diptera	Culicidae	Aedes	Aedes aegypti	SPECIES	23.02000	120.22000	1264884459
13397	Diptera	Culicidae	Aedes	Aedes aegypti	SPECIES	23.02000	120.63000	1264885028
13398	Diptera	Culicidae	Aedes	Aedes aegypti	SPECIES	23.02000	-82.33000	1264887148
13399	Diptera	Culicidae	Aedes	Aedes aegypti	SPECIES	23.03000	120.13000	1264876610
13400	Diptera	Culicidae	Aedes	Aedes aegypti	SPECIES	23.03000	120.45000	1264876622
13401	Diptera	Culicidae	Aedes	Aedes aegypti	SPECIES	23.03000	120.42000	1264876645
13402	Diptera	Culicidae	Aedes	Aedes aegypti	SPECIES	23.03000	120.25000	1264876722
13403	Diptera	Culicidae	Aedes	Aedes aegypti	SPECIES	23.03000	120.28000	1264876919
13404	Diptera	Culicidae	Aedes	Aedes aegypti	SPECIES	23.03000	120.29000	1264877326
13405	Diptera	Culicidae	Aedes	Aedes aegypti	SPECIES	23.03000	120.31000	1264878258
13406	Diptera	Culicidae	Aedes	Aedes aegypti	SPECIES	23.03000	120.18000	1264879023
13407	Diptera	Culicidae	Aedes	Aedes aegypti	SPECIES	23.03000	120.32000	1264879024

№	Order	Family	Genus	Species	Taxon Rank	Latitude	Longitude	GBIF_ID
13408	Diptera	Culicidae	Aedes	Aedes aegypti	SPECIES	23.03000	120.30000	1264879029
13409	Diptera	Culicidae	Aedes	Aedes aegypti	SPECIES	23.03000	120.26000	1264879066
13410	Diptera	Culicidae	Aedes	Aedes aegypti	SPECIES	23.03000	120.22000	1264879078
13411	Diptera	Culicidae	Aedes	Aedes aegypti	SPECIES	23.03000	120.21000	1264884018
13412	Diptera	Culicidae	Aedes	Aedes aegypti	SPECIES	23.03000	120.20000	1264884095
13413	Diptera	Culicidae	Aedes	Aedes aegypti	SPECIES	23.03000	120.23000	1264884212
13414	Diptera	Culicidae	Aedes	Aedes aegypti	SPECIES	23.03000	-82.25000	1264887134
13415	Diptera	Culicidae	Aedes	Aedes aegypti	SPECIES	23.03889	-99.05361	1894395667
13416	Diptera	Culicidae	Aedes	Aedes aegypti	SPECIES	23.04000	120.14000	1264876538
13417	Diptera	Culicidae	Aedes	Aedes aegypti	SPECIES	23.04000	120.26000	1264876700
13418	Diptera	Culicidae	Aedes	Aedes aegypti	SPECIES	23.04000	120.28000	1264877136
13419	Diptera	Culicidae	Aedes	Aedes aegypti	SPECIES	23.04000	120.32000	1264877240
13420	Diptera	Culicidae	Aedes	Aedes aegypti	SPECIES	23.04000	120.22000	1264878849
13421	Diptera	Culicidae	Aedes	Aedes aegypti	SPECIES	23.04000	120.21000	1264879014
13422	Diptera	Culicidae	Aedes	Aedes aegypti	SPECIES	23.04000	120.30000	1264879028
13423	Diptera	Culicidae	Aedes	Aedes aegypti	SPECIES	23.04000	120.24000	1264879587
13424	Diptera	Culicidae	Aedes	Aedes aegypti	SPECIES	23.04000	120.20000	1264879717
13425	Diptera	Culicidae	Aedes	Aedes aegypti	SPECIES	23.04000	120.31000	1264880232
13426	Diptera	Culicidae	Aedes	Aedes aegypti	SPECIES	23.04000	120.19000	1264882575
13427	Diptera	Culicidae	Aedes	Aedes aegypti	SPECIES	23.04000	72.56000	1264889016
13428	Diptera	Culicidae	Aedes	Aedes aegypti	SPECIES	23.04000	-82.27000	1264890568
13429	Diptera	Culicidae	Aedes	Aedes aegypti	SPECIES	23.04000	120.17000	1264891100
13430	Diptera	Culicidae	Aedes	Aedes aegypti	SPECIES	23.04611	-99.15417	1421477463
13431	Diptera	Culicidae	Aedes	Aedes aegypti	SPECIES	23.05000	120.18000	1264876543
13432	Diptera	Culicidae	Aedes	Aedes aegypti	SPECIES	23.05000	120.26000	1264876747
13433	Diptera	Culicidae	Aedes	Aedes aegypti	SPECIES	23.05000	120.10000	1264877025
13434	Diptera	Culicidae	Aedes	Aedes aegypti	SPECIES	23.05000	120.21000	1264877102
13435	Diptera	Culicidae	Aedes	Aedes aegypti	SPECIES	23.05000	120.29000	1264877178
13436	Diptera	Culicidae	Aedes	Aedes aegypti	SPECIES	23.05000	120.20000	1264877189
13437	Diptera	Culicidae	Aedes	Aedes aegypti	SPECIES	23.05000	120.31000	1264878997
13438	Diptera	Culicidae	Aedes	Aedes aegypti	SPECIES	23.05000	120.34000	1264879044
13439	Diptera	Culicidae	Aedes	Aedes aegypti	SPECIES	23.05000	120.25000	1264881276
13440	Diptera	Culicidae	Aedes	Aedes aegypti	SPECIES	23.05000	120.22000	1264883216
13441	Diptera	Culicidae	Aedes	Aedes aegypti	SPECIES	23.05000	-82.46000	1264887154
13442	Diptera	Culicidae	Aedes	Aedes aegypti	SPECIES	23.05000	72.10000	1264889046
13443	Diptera	Culicidae	Aedes	Aedes aegypti	SPECIES	23.05000	113.13000	1264889127
13444	Diptera	Culicidae	Aedes	Aedes aegypti	SPECIES	23.05000	-82.21000	1264890873
13445	Diptera	Culicidae	Aedes	Aedes aegypti	SPECIES	23.06000	120.20000	1264876658
13446	Diptera	Culicidae	Aedes	Aedes aegypti	SPECIES	23.06000	120.14000	1264877070
13447	Diptera	Culicidae	Aedes	Aedes aegypti	SPECIES	23.06000	120.21000	1264877138
13448	Diptera	Culicidae	Aedes	Aedes aegypti	SPECIES	23.06000	120.32000	1264878784

№	Order	Family	Genus	Species	Taxon Rank	Latitude	Longitude	GBIF_ID
13449	Diptera	Culicidae	Aedes	Aedes aegypti	SPECIES	23.06000	120.24000	1264878940
13450	Diptera	Culicidae	Aedes	Aedes aegypti	SPECIES	23.06000	120.18000	1264879347
13451	Diptera	Culicidae	Aedes	Aedes aegypti	SPECIES	23.06000	120.13000	1264879618
13452	Diptera	Culicidae	Aedes	Aedes aegypti	SPECIES	23.06000	120.17000	1264879635
13453	Diptera	Culicidae	Aedes	Aedes aegypti	SPECIES	23.06000	120.61000	1264881148
13454	Diptera	Culicidae	Aedes	Aedes aegypti	SPECIES	23.06139	-109.70806	1894400290
13455	Diptera	Culicidae	Aedes	Aedes aegypti	SPECIES	23.06500	-109.69601	3356894312
13456	Diptera	Culicidae	Aedes	Aedes aegypti	SPECIES	23.07000	120.16000	1264876620
13457	Diptera	Culicidae	Aedes	Aedes aegypti	SPECIES	23.07000	120.18000	1264877097
13458	Diptera	Culicidae	Aedes	Aedes aegypti	SPECIES	23.07000	120.15000	1264877128
13459	Diptera	Culicidae	Aedes	Aedes aegypti	SPECIES	23.07000	120.17000	1264877135
13460	Diptera	Culicidae	Aedes	Aedes aegypti	SPECIES	23.07000	120.26000	1264879018
13461	Diptera	Culicidae	Aedes	Aedes aegypti	SPECIES	23.07000	120.22000	1264879534
13462	Diptera	Culicidae	Aedes	Aedes aegypti	SPECIES	23.07000	120.13000	1264879567
13463	Diptera	Culicidae	Aedes	Aedes aegypti	SPECIES	23.07000	120.21000	1264884785
13464	Diptera	Culicidae	Aedes	Aedes aegypti	SPECIES	23.07000	-82.42000	1264887168
13465	Diptera	Culicidae	Aedes	Aedes aegypti	SPECIES	23.07000	-82.44000	1264889259
13466	Diptera	Culicidae	Aedes	Aedes aegypti	SPECIES	23.07000	-82.43000	1264890862
13467	Diptera	Culicidae	Aedes	Aedes aegypti	SPECIES	23.08000	120.12000	1264876625
13468	Diptera	Culicidae	Aedes	Aedes aegypti	SPECIES	23.08000	120.30000	1264876709
13469	Diptera	Culicidae	Aedes	Aedes aegypti	SPECIES	23.08000	120.22000	1264876742
13470	Diptera	Culicidae	Aedes	Aedes aegypti	SPECIES	23.08000	120.29000	1264876756
13471	Diptera	Culicidae	Aedes	Aedes aegypti	SPECIES	23.08000	120.20000	1264877181
13472	Diptera	Culicidae	Aedes	Aedes aegypti	SPECIES	23.08000	120.14000	1264877729
13473	Diptera	Culicidae	Aedes	Aedes aegypti	SPECIES	23.08000	-82.31000	1264887132
13474	Diptera	Culicidae	Aedes	Aedes aegypti	SPECIES	23.08000	-82.42000	1264889265
13475	Diptera	Culicidae	Aedes	Aedes aegypti	SPECIES	23.08000	-82.37000	1264890961
13476	Diptera	Culicidae	Aedes	Aedes aegypti	SPECIES	23.09000	120.23000	1264876606
13477	Diptera	Culicidae	Aedes	Aedes aegypti	SPECIES	23.09000	120.05000	1264876788
13478	Diptera	Culicidae	Aedes	Aedes aegypti	SPECIES	23.09000	120.54000	1264879034
13479	Diptera	Culicidae	Aedes	Aedes aegypti	SPECIES	23.09000	120.47000	1264879813
13480	Diptera	Culicidae	Aedes	Aedes aegypti	SPECIES	23.09000	120.63000	1264880837
13481	Diptera	Culicidae	Aedes	Aedes aegypti	SPECIES	23.09000	-82.45000	1264887164
13482	Diptera	Culicidae	Aedes	Aedes aegypti	SPECIES	23.09000	-82.36000	1264889313
13483	Diptera	Culicidae	Aedes	Aedes aegypti	SPECIES	23.09000	-82.42000	1264889325
13484	Diptera	Culicidae	Aedes	Aedes aegypti	SPECIES	23.10000	120.35000	1264876706
13485	Diptera	Culicidae	Aedes	Aedes aegypti	SPECIES	23.10000	120.36000	1264876711
13486	Diptera	Culicidae	Aedes	Aedes aegypti	SPECIES	23.10000	120.24000	1264876712
13487	Diptera	Culicidae	Aedes	Aedes aegypti	SPECIES	23.10000	120.22000	1264878800
13488	Diptera	Culicidae	Aedes	Aedes aegypti	SPECIES	23.10000	120.49000	1264879880
13489	Diptera	Culicidae	Aedes	Aedes aegypti	SPECIES	23.10000	120.59000	1264881176

№	Order	Family	Genus	Species	Taxon Rank	Latitude	Longitude	GBIF_ID
13490	Diptera	Culicidae	Aedes	Aedes aegypti	SPECIES	23.10000	-82.36000	1264887171
13491	Diptera	Culicidae	Aedes	Aedes aegypti	SPECIES	23.10000	-82.39000	1264887173
13492	Diptera	Culicidae	Aedes	Aedes aegypti	SPECIES	23.10000	-82.16000	1264887176
13493	Diptera	Culicidae	Aedes	Aedes aegypti	SPECIES	23.10000	-82.42000	1264887817
13494	Diptera	Culicidae	Aedes	Aedes aegypti	SPECIES	23.10000	-82.38000	1264890877
13495	Diptera	Culicidae	Aedes	Aedes aegypti	SPECIES	23.10000	-82.45000	1264890883
13496	Diptera	Culicidae	Aedes	Aedes aegypti	SPECIES	23.10000	-82.43000	1264890972
13497	Diptera	Culicidae	Aedes	Aedes aegypti	SPECIES	23.11000	120.22000	1264876776
13498	Diptera	Culicidae	Aedes	Aedes aegypti	SPECIES	23.11000	-82.25000	1264887170
13499	Diptera	Culicidae	Aedes	Aedes aegypti	SPECIES	23.11000	-82.43000	1264887382
13500	Diptera	Culicidae	Aedes	Aedes aegypti	SPECIES	23.11000	-82.42000	1264887816
13501	Diptera	Culicidae	Aedes	Aedes aegypti	SPECIES	23.11000	-82.41000	1264887849
13502	Diptera	Culicidae	Aedes	Aedes aegypti	SPECIES	23.11000	-82.37000	1264890685
13503	Diptera	Culicidae	Aedes	Aedes aegypti	SPECIES	23.11000	-82.35000	1264891160
13504	Diptera	Culicidae	Aedes	Aedes aegypti	SPECIES	23.11000	72.03000	1264891375
13505	Diptera	Culicidae	Aedes	Aedes aegypti	SPECIES	23.11280	-82.38957	3068156302
13506	Diptera	Culicidae	Aedes	Aedes aegypti	SPECIES	23.12000	120.31000	1264876552
13507	Diptera	Culicidae	Aedes	Aedes aegypti	SPECIES	23.12000	120.23000	1264876692
13508	Diptera	Culicidae	Aedes	Aedes aegypti	SPECIES	23.12000	120.25000	1264876744
13509	Diptera	Culicidae	Aedes	Aedes aegypti	SPECIES	23.12000	120.63000	1264876922
13510	Diptera	Culicidae	Aedes	Aedes aegypti	SPECIES	23.12000	120.28000	1264877697
13511	Diptera	Culicidae	Aedes	Aedes aegypti	SPECIES	23.12000	120.46000	1264880016
13512	Diptera	Culicidae	Aedes	Aedes aegypti	SPECIES	23.12000	-82.42000	1264887387
13513	Diptera	Culicidae	Aedes	Aedes aegypti	SPECIES	23.12000	-82.41000	1264887390
13514	Diptera	Culicidae	Aedes	Aedes aegypti	SPECIES	23.12000	72.54000	1264888992
13515	Diptera	Culicidae	Aedes	Aedes aegypti	SPECIES	23.12000	-82.39000	1264889329
13516	Diptera	Culicidae	Aedes	Aedes aegypti	SPECIES	23.13000	120.48000	1264876587
13517	Diptera	Culicidae	Aedes	Aedes aegypti	SPECIES	23.13000	120.17000	1264876704
13518	Diptera	Culicidae	Aedes	Aedes aegypti	SPECIES	23.13000	120.33000	1264876765
13519	Diptera	Culicidae	Aedes	Aedes aegypti	SPECIES	23.13000	120.23000	1264876874
13520	Diptera	Culicidae	Aedes	Aedes aegypti	SPECIES	23.13000	120.21000	1264877681
13521	Diptera	Culicidae	Aedes	Aedes aegypti	SPECIES	23.13000	120.20000	1264877732
13522	Diptera	Culicidae	Aedes	Aedes aegypti	SPECIES	23.13000	120.46000	1264879073
13523	Diptera	Culicidae	Aedes	Aedes aegypti	SPECIES	23.13000	120.52000	1264881587
13524	Diptera	Culicidae	Aedes	Aedes aegypti	SPECIES	23.13000	-82.34000	1264887169
13525	Diptera	Culicidae	Aedes	Aedes aegypti	SPECIES	23.13000	-82.41000	1264889656
13526	Diptera	Culicidae	Aedes	Aedes aegypti	SPECIES	23.13000	-82.37000	1264890713
13527	Diptera	Culicidae	Aedes	Aedes aegypti	SPECIES	23.13000	-82.36000	1264891243
13528	Diptera	Culicidae	Aedes	Aedes aegypti	SPECIES	23.13194	-82.36417	1933069983
13529	Diptera	Culicidae	Aedes	Aedes aegypti	SPECIES	23.14000	120.28000	1264876781
13530	Diptera	Culicidae	Aedes	Aedes aegypti	SPECIES	23.14000	120.27000	1264876784

№	Order	Family	Genus	Species	Taxon Rank	Latitude	Longitude	GBIF_ID
13531	Diptera	Culicidae	Aedes	Aedes aegypti	SPECIES	23.14000	120.46000	1264876998
13532	Diptera	Culicidae	Aedes	Aedes aegypti	SPECIES	23.14000	120.25000	1264886086
13533	Diptera	Culicidae	Aedes	Aedes aegypti	SPECIES	23.14000	-82.37000	1264887155
13534	Diptera	Culicidae	Aedes	Aedes aegypti	SPECIES	23.14000	-82.36000	1264890942
13535	Diptera	Culicidae	Aedes	Aedes aegypti	SPECIES	23.15000	120.23000	1264876593
13536	Diptera	Culicidae	Aedes	Aedes aegypti	SPECIES	23.15000	120.14000	1264876710
13537	Diptera	Culicidae	Aedes	Aedes aegypti	SPECIES	23.15000	120.77000	1264877108
13538	Diptera	Culicidae	Aedes	Aedes aegypti	SPECIES	23.15000	120.27000	1264877711
13539	Diptera	Culicidae	Aedes	Aedes aegypti	SPECIES	23.15000	120.33000	1264877723
13540	Diptera	Culicidae	Aedes	Aedes aegypti	SPECIES	23.15000	120.17000	1264879403
13541	Diptera	Culicidae	Aedes	Aedes aegypti	SPECIES	23.15000	120.48000	1264883329
13542	Diptera	Culicidae	Aedes	Aedes aegypti	SPECIES	23.15028	-99.68944	1894395861
13543	Diptera	Culicidae	Aedes	Aedes aegypti	SPECIES	23.16000	120.19000	1264876656
13544	Diptera	Culicidae	Aedes	Aedes aegypti	SPECIES	23.16000	120.23000	1264876669
13545	Diptera	Culicidae	Aedes	Aedes aegypti	SPECIES	23.16000	120.28000	1264876951
13546	Diptera	Culicidae	Aedes	Aedes aegypti	SPECIES	23.16000	120.29000	1264877028
13547	Diptera	Culicidae	Aedes	Aedes aegypti	SPECIES	23.16000	120.16000	1264883590
13548	Diptera	Culicidae	Aedes	Aedes aegypti	SPECIES	23.17000	120.18000	1264876687
13549	Diptera	Culicidae	Aedes	Aedes aegypti	SPECIES	23.17000	120.35000	1264876714
13550	Diptera	Culicidae	Aedes	Aedes aegypti	SPECIES	23.17000	120.48000	1264876715
13551	Diptera	Culicidae	Aedes	Aedes aegypti	SPECIES	23.17000	120.30000	1264877716
13552	Diptera	Culicidae	Aedes	Aedes aegypti	SPECIES	23.18000	120.25000	1264876596
13553	Diptera	Culicidae	Aedes	Aedes aegypti	SPECIES	23.18000	120.15000	1264876634
13554	Diptera	Culicidae	Aedes	Aedes aegypti	SPECIES	23.18000	120.24000	1264876660
13555	Diptera	Culicidae	Aedes	Aedes aegypti	SPECIES	23.18000	120.12000	1264876724
13556	Diptera	Culicidae	Aedes	Aedes aegypti	SPECIES	23.19000	120.24000	1264876657
13557	Diptera	Culicidae	Aedes	Aedes aegypti	SPECIES	23.19000	120.13000	1264876792
13558	Diptera	Culicidae	Aedes	Aedes aegypti	SPECIES	23.19000	120.32000	1264876913
13559	Diptera	Culicidae	Aedes	Aedes aegypti	SPECIES	23.19000	120.23000	1264877071
13560	Diptera	Culicidae	Aedes	Aedes aegypti	SPECIES	23.19000	82.35000	1264889195
13561	Diptera	Culicidae	Aedes	Aedes aegypti	SPECIES	23.19000	82.36000	1264889235
13562	Diptera	Culicidae	Aedes	Aedes aegypti	SPECIES	23.19401	-106.42586	2862508313
13563	Diptera	Culicidae	Aedes	Aedes aegypti	SPECIES	23.19402	-106.42606	3330761512
13564	Diptera	Culicidae	Aedes	Aedes aegypti	SPECIES	23.19403	-106.42596	2862341252
13565	Diptera	Culicidae	Aedes	Aedes aegypti	SPECIES	23.20000	120.31000	1264876938
13566	Diptera	Culicidae	Aedes	Aedes aegypti	SPECIES	23.21000	120.26000	1264876591
13567	Diptera	Culicidae	Aedes	Aedes aegypti	SPECIES	23.21000	-106.42000	1264888024
13568	Diptera	Culicidae	Aedes	Aedes aegypti	SPECIES	23.21944	-106.38069	2629265652
13569	Diptera	Culicidae	Aedes	Aedes aegypti	SPECIES	23.22000	120.20000	1264876567
13570	Diptera	Culicidae	Aedes	Aedes aegypti	SPECIES	23.22000	120.25000	1264876573
13571	Diptera	Culicidae	Aedes	Aedes aegypti	SPECIES	23.22000	120.14000	1264884135

№	Order	Family	Genus	Species	Taxon Rank	Latitude	Longitude	GBIF_ID
13572	Diptera	Culicidae	Aedes	Aedes aegypti	SPECIES	23.22069	-106.38153	2629263403
13573	Diptera	Culicidae	Aedes	Aedes aegypti	SPECIES	23.23000	120.26000	1264876598
13574	Diptera	Culicidae	Aedes	Aedes aegypti	SPECIES	23.23000	120.24000	1264876616
13575	Diptera	Culicidae	Aedes	Aedes aegypti	SPECIES	23.23000	120.18000	1264876665
13576	Diptera	Culicidae	Aedes	Aedes aegypti	SPECIES	23.23000	120.35000	1264878561
13577	Diptera	Culicidae	Aedes	Aedes aegypti	SPECIES	23.23611	-106.41528	1421477113
13578	Diptera	Culicidae	Aedes	Aedes aegypti	SPECIES	23.24000	120.13000	1264876525
13579	Diptera	Culicidae	Aedes	Aedes aegypti	SPECIES	23.24000	120.26000	1264876637
13580	Diptera	Culicidae	Aedes	Aedes aegypti	SPECIES	23.24000	120.18000	1264876672
13581	Diptera	Culicidae	Aedes	Aedes aegypti	SPECIES	23.24000	120.11000	1264878569
13582	Diptera	Culicidae	Aedes	Aedes aegypti	SPECIES	23.24000	120.33000	1264878721
13583	Diptera	Culicidae	Aedes	Aedes aegypti	SPECIES	23.24139	-106.40972	2629263712
13584	Diptera	Culicidae	Aedes	Aedes aegypti	SPECIES	23.24194	-106.45303	3355024469
13585	Diptera	Culicidae	Aedes	Aedes aegypti	SPECIES	23.25000	120.44000	1264876639
13586	Diptera	Culicidae	Aedes	Aedes aegypti	SPECIES	23.25000	120.17000	1264876690
13587	Diptera	Culicidae	Aedes	Aedes aegypti	SPECIES	23.25000	-106.41000	1264886773
13588	Diptera	Culicidae	Aedes	Aedes aegypti	SPECIES	23.25806	-100.17444	1894396450
13589	Diptera	Culicidae	Aedes	Aedes aegypti	SPECIES	23.25891	-106.44727	3468867991
13590	Diptera	Culicidae	Aedes	Aedes aegypti	SPECIES	23.25913	-106.44727	3457219491
13591	Diptera	Culicidae	Aedes	Aedes aegypti	SPECIES	23.26000	120.17000	1264876563
13592	Diptera	Culicidae	Aedes	Aedes aegypti	SPECIES	23.26000	120.27000	1264876630
13593	Diptera	Culicidae	Aedes	Aedes aegypti	SPECIES	23.26000	120.36000	1264879396
13594	Diptera	Culicidae	Aedes	Aedes aegypti	SPECIES	23.26251	-106.46274	4103223277
13595	Diptera	Culicidae	Aedes	Aedes aegypti	SPECIES	23.27000	120.40000	1264876686
13596	Diptera	Culicidae	Aedes	Aedes aegypti	SPECIES	23.27000	120.71000	1264876879
13597	Diptera	Culicidae	Aedes	Aedes aegypti	SPECIES	23.27000	120.29000	1264879319
13598	Diptera	Culicidae	Aedes	Aedes aegypti	SPECIES	23.27000	120.74000	1264888305
13599	Diptera	Culicidae	Aedes	Aedes aegypti	SPECIES	23.27972	-98.92194	1894396680
13600	Diptera	Culicidae	Aedes	Aedes aegypti	SPECIES	23.28000	120.36000	1264876602
13601	Diptera	Culicidae	Aedes	Aedes aegypti	SPECIES	23.28000	120.27000	1264877194
13602	Diptera	Culicidae	Aedes	Aedes aegypti	SPECIES	23.28000	120.31000	1264879398
13603	Diptera	Culicidae	Aedes	Aedes aegypti	SPECIES	23.29000	120.33000	1264876607
13604	Diptera	Culicidae	Aedes	Aedes aegypti	SPECIES	23.29000	120.37000	1264876608
13605	Diptera	Culicidae	Aedes	Aedes aegypti	SPECIES	23.29000	120.30000	1264876655
13606	Diptera	Culicidae	Aedes	Aedes aegypti	SPECIES	23.29000	120.26000	1264876659
13607	Diptera	Culicidae	Aedes	Aedes aegypti	SPECIES	23.29000	71.92000	1264889280
13608	Diptera	Culicidae	Aedes	Aedes aegypti	SPECIES	23.30000	120.31000	1264877171
13609	Diptera	Culicidae	Aedes	Aedes aegypti	SPECIES	23.30000	120.29000	1264877179
13610	Diptera	Culicidae	Aedes	Aedes aegypti	SPECIES	23.30000	120.32000	1264877188
13611	Diptera	Culicidae	Aedes	Aedes aegypti	SPECIES	23.30914	-99.73442	1895892965
13612	Diptera	Culicidae	Aedes	Aedes aegypti	SPECIES	23.31000	120.36000	1264876530

№	Order	Family	Genus	Species	Taxon Rank	Latitude	Longitude	GBIF_ID
13613	Diptera	Culicidae	Aedes	Aedes aegypti	SPECIES	23.31000	120.42000	1264876547
13614	Diptera	Culicidae	Aedes	Aedes aegypti	SPECIES	23.31000	120.30000	1264876562
13615	Diptera	Culicidae	Aedes	Aedes aegypti	SPECIES	23.31000	120.27000	1264876572
13616	Diptera	Culicidae	Aedes	Aedes aegypti	SPECIES	23.31000	120.32000	1264876599
13617	Diptera	Culicidae	Aedes	Aedes aegypti	SPECIES	23.31000	120.31000	1264876708
13618	Diptera	Culicidae	Aedes	Aedes aegypti	SPECIES	23.31000	120.33000	1264877145
13619	Diptera	Culicidae	Aedes	Aedes aegypti	SPECIES	23.31809	-101.18810	3067853485
13620	Diptera	Culicidae	Aedes	Aedes aegypti	SPECIES	23.32000	120.28000	1264876631
13621	Diptera	Culicidae	Aedes	Aedes aegypti	SPECIES	23.32000	120.34000	1264876666
13622	Diptera	Culicidae	Aedes	Aedes aegypti	SPECIES	23.32000	120.27000	1264876689
13623	Diptera	Culicidae	Aedes	Aedes aegypti	SPECIES	23.32000	120.32000	1264877146
13624	Diptera	Culicidae	Aedes	Aedes aegypti	SPECIES	23.33000	120.32000	1264877154
13625	Diptera	Culicidae	Aedes	Aedes aegypti	SPECIES	23.33003	-99.76189	1895893049
13626	Diptera	Culicidae	Aedes	Aedes aegypti	SPECIES	23.34000	120.42000	1264876764
13627	Diptera	Culicidae	Aedes	Aedes aegypti	SPECIES	23.35000	120.46000	1264876886
13628	Diptera	Culicidae	Aedes	Aedes aegypti	SPECIES	23.35000	120.39000	1264876905
13629	Diptera	Culicidae	Aedes	Aedes aegypti	SPECIES	23.36000	120.36000	1264877029
13630	Diptera	Culicidae	Aedes	Aedes aegypti	SPECIES	23.36000	120.40000	1264882999
13631	Diptera	Culicidae	Aedes	Aedes aegypti	SPECIES	23.36633	-106.27948	3044733693
13632	Diptera	Culicidae	Aedes	Aedes aegypti	SPECIES	23.37222	-100.75556	1894399167
13633	Diptera	Culicidae	Aedes	Aedes aegypti	SPECIES	23.37417	-98.70250	1895892998
13634	Diptera	Culicidae	Aedes	Aedes aegypti	SPECIES	23.39000	120.39000	1264876667
13635	Diptera	Culicidae	Aedes	Aedes aegypti	SPECIES	23.39439	-99.75211	1895892973
13636	Diptera	Culicidae	Aedes	Aedes aegypti	SPECIES	23.40000	120.44000	1264876577
13637	Diptera	Culicidae	Aedes	Aedes aegypti	SPECIES	23.40444	-99.43528	1894398682
13638	Diptera	Culicidae	Aedes	Aedes aegypti	SPECIES	23.40528	-99.38028	1894399887
13639	Diptera	Culicidae	Aedes	Aedes aegypti	SPECIES	23.42000	-98.97944	1895893063
13640	Diptera	Culicidae	Aedes	Aedes aegypti	SPECIES	23.42194	-100.11667	1894395133
13641	Diptera	Culicidae	Aedes	Aedes aegypti	SPECIES	23.44972	-99.33861	1894394796
13642	Diptera	Culicidae	Aedes	Aedes aegypti	SPECIES	23.46194	-99.29972	1894396468
13643	Diptera	Culicidae	Aedes	Aedes aegypti	SPECIES	23.47611	-100.66361	1894395858
13644	Diptera	Culicidae	Aedes	Aedes aegypti	SPECIES	23.47861	-99.30722	1894397887
13645	Diptera	Culicidae	Aedes	Aedes aegypti	SPECIES	23.50000	72.95000	1264889287
13646	Diptera	Culicidae	Aedes	Aedes aegypti	SPECIES	23.51528	-99.33194	1894399422
13647	Diptera	Culicidae	Aedes	Aedes aegypti	SPECIES	23.51972	-100.62111	1894398887
13648	Diptera	Culicidae	Aedes	Aedes aegypti	SPECIES	23.56000	119.57000	1264882855
13649	Diptera	Culicidae	Aedes	Aedes aegypti	SPECIES	23.57000	119.58000	1264882458
13650	Diptera	Culicidae	Aedes	Aedes aegypti	SPECIES	23.57000	119.56000	1264882559
13651	Diptera	Culicidae	Aedes	Aedes aegypti	SPECIES	23.58000	119.57000	1264882459
13652	Diptera	Culicidae	Aedes	Aedes aegypti	SPECIES	23.58528	-99.35583	1894394502
13653	Diptera	Culicidae	Aedes	Aedes aegypti	SPECIES	23.59556	-100.65694	1894397650

№	Order	Family	Genus	Species	Taxon Rank	Latitude	Longitude	GBIF_ID
13654	Diptera	Culicidae	Aedes	Aedes aegypti	SPECIES	23.60000	72.95000	1264890720
13655	Diptera	Culicidae	Aedes	Aedes aegypti	SPECIES	23.60000	72.40000	1264890734
13656	Diptera	Culicidae	Aedes	Aedes aegypti	SPECIES	23.64556	-100.64361	1894400183
13657	Diptera	Culicidae	Aedes	Aedes aegypti	SPECIES	23.67000	-99.25000	1264887025
13658	Diptera	Culicidae	Aedes	Aedes aegypti	SPECIES	23.67222	-100.18028	1894400105
13659	Diptera	Culicidae	Aedes	Aedes aegypti	SPECIES	23.67444	-100.47167	1894399264
13660	Diptera	Culicidae	Aedes	Aedes aegypti	SPECIES	23.67583	-100.70778	1894399900
13661	Diptera	Culicidae	Aedes	Aedes aegypti	SPECIES	23.70000	90.38000	1264889078
13662	Diptera	Culicidae	Aedes	Aedes aegypti	SPECIES	23.70000	90.42000	1264890973
13663	Diptera	Culicidae	Aedes	Aedes aegypti	SPECIES	23.71000	90.41000	1264890641
13664	Diptera	Culicidae	Aedes	Aedes aegypti	SPECIES	23.72000	90.40000	1264891122
13665	Diptera	Culicidae	Aedes	Aedes aegypti	SPECIES	23.72158	-106.78081	2629262354
13666	Diptera	Culicidae	Aedes	Aedes aegypti	SPECIES	23.73000	90.40000	1264890975
13667	Diptera	Culicidae	Aedes	Aedes aegypti	SPECIES	23.73611	-99.14611	1894400019
13668	Diptera	Culicidae	Aedes	Aedes aegypti	SPECIES	23.74000	-99.14000	1264890278
13669	Diptera	Culicidae	Aedes	Aedes aegypti	SPECIES	23.74000	90.38000	1264890980
13670	Diptera	Culicidae	Aedes	Aedes aegypti	SPECIES	23.74000	90.40000	1264890996
13671	Diptera	Culicidae	Aedes	Aedes aegypti	SPECIES	23.74000	90.41000	1264891021
13672	Diptera	Culicidae	Aedes	Aedes aegypti	SPECIES	23.75817	-97.75600	1418703918
13673	Diptera	Culicidae	Aedes	Aedes aegypti	SPECIES	23.76000	90.37000	1264891006
13674	Diptera	Culicidae	Aedes	Aedes aegypti	SPECIES	23.76750	-98.20778	1894400046
13675	Diptera	Culicidae	Aedes	Aedes aegypti	SPECIES	23.77111	-99.07583	1894397342
13676	Diptera	Culicidae	Aedes	Aedes aegypti	SPECIES	23.79286	-106.59775	2629264409
13677	Diptera	Culicidae	Aedes	Aedes aegypti	SPECIES	23.80000	90.26000	1264890976
13678	Diptera	Culicidae	Aedes	Aedes aegypti	SPECIES	23.80000	90.35000	3351428103
13679	Diptera	Culicidae	Aedes	Aedes aegypti	SPECIES	23.81972	-99.98917	1894398404
13680	Diptera	Culicidae	Aedes	Aedes aegypti	SPECIES	23.82083	-100.72472	1894400222
13681	Diptera	Culicidae	Aedes	Aedes aegypti	SPECIES	23.88194	-100.95083	1894396255
13682	Diptera	Culicidae	Aedes	Aedes aegypti	SPECIES	23.90361	-99.11556	1894399465
13683	Diptera	Culicidae	Aedes	Aedes aegypti	SPECIES	23.91861	-99.00667	1894399876
13684	Diptera	Culicidae	Aedes	Aedes aegypti	SPECIES	23.94011	-106.42544	2629263645
13685	Diptera	Culicidae	Aedes	Aedes aegypti	SPECIES	23.97222	-99.77083	1894399251
13686	Diptera	Culicidae	Aedes	Aedes aegypti	SPECIES	23.97489	-106.70697	2629262425
13687	Diptera	Culicidae	Aedes	Aedes aegypti	SPECIES	24.01419	-104.64585	3925307626
13688	Diptera	Culicidae	Aedes	Aedes aegypti	SPECIES	24.01528	-99.20194	1894398459
13689	Diptera	Culicidae	Aedes	Aedes aegypti	SPECIES	24.02216	-104.69392	3966568554
13690	Diptera	Culicidae	Aedes	Aedes aegypti	SPECIES	24.02361	-104.66972	1894400327
13691	Diptera	Culicidae	Aedes	Aedes aegypti	SPECIES	24.04833	-98.90083	1894399349
13692	Diptera	Culicidae	Aedes	Aedes aegypti	SPECIES	24.05639	-98.37556	1894398848
13693	Diptera	Culicidae	Aedes	Aedes aegypti	SPECIES	24.09944	-99.81722	1894398490
13694	Diptera	Culicidae	Aedes	Aedes aegypti	SPECIES	24.13000	90.29000	1264891226

№	Order	Family	Genus	Species	Taxon Rank	Latitude	Longitude	GBIF_ID
13695	Diptera	Culicidae	Aedes	Aedes aegypti	SPECIES	24.14222	-110.31083	1894400089
13696	Diptera	Culicidae	Aedes	Aedes aegypti	SPECIES	24.15250	-99.40861	1894394432
13697	Diptera	Culicidae	Aedes	Aedes aegypti	SPECIES	24.18000	90.29000	1264890991
13698	Diptera	Culicidae	Aedes	Aedes aegypti	SPECIES	24.21444	-98.48333	1894399829
13699	Diptera	Culicidae	Aedes	Aedes aegypti	SPECIES	24.23139	-99.61583	1894396983
13700	Diptera	Culicidae	Aedes	Aedes aegypti	SPECIES	24.25000	-107.18000	1264891282
13701	Diptera	Culicidae	Aedes	Aedes aegypti	SPECIES	24.34189	-107.36156	2629263531
13702	Diptera	Culicidae	Aedes	Aedes aegypti	SPECIES	24.47000	85.60000	1264886053
13703	Diptera	Culicidae	Aedes	Aedes aegypti	SPECIES	24.48000	73.20000	1264890743
13704	Diptera	Culicidae	Aedes	Aedes aegypti	SPECIES	24.48972	-97.74500	1418704063
13705	Diptera	Culicidae	Aedes	Aedes aegypti	SPECIES	24.49578	-97.74128	1418704098
13706	Diptera	Culicidae	Aedes	Aedes aegypti	SPECIES	24.49617	-100.50403	1895892882
13707	Diptera	Culicidae	Aedes	Aedes aegypti	SPECIES	24.53389	-97.73503	1418703956
13708	Diptera	Culicidae	Aedes	Aedes aegypti	SPECIES	24.55000	-81.78000	1264888984
13709	Diptera	Culicidae	Aedes	Aedes aegypti	SPECIES	24.55675	-97.67486	1418703921
13710	Diptera	Culicidae	Aedes	Aedes aegypti	SPECIES	24.56000	-81.78000	1264888854
13711	Diptera	Culicidae	Aedes	Aedes aegypti	SPECIES	24.57000	-81.74000	1264888876
13712	Diptera	Culicidae	Aedes	Aedes aegypti	SPECIES	24.57000	77.72000	1264889045
13713	Diptera	Culicidae	Aedes	Aedes aegypti	SPECIES	24.57000	112.08000	1264889104
13714	Diptera	Culicidae	Aedes	Aedes aegypti	SPECIES	24.57000	-81.75000	1264889784
13715	Diptera	Culicidae	Aedes	Aedes aegypti	SPECIES	24.57889	-99.91028	1894395928
13716	Diptera	Culicidae	Aedes	Aedes aegypti	SPECIES	24.59000	-81.68000	1264888863
13717	Diptera	Culicidae	Aedes	Aedes aegypti	SPECIES	24.59000	-81.63000	1264888882
13718	Diptera	Culicidae	Aedes	Aedes aegypti	SPECIES	24.60000	-81.66000	1264888866
13719	Diptera	Culicidae	Aedes	Aedes aegypti	SPECIES	24.62283	-98.29478	1895893054
13720	Diptera	Culicidae	Aedes	Aedes aegypti	SPECIES	24.62639	-97.71667	1421475472
13721	Diptera	Culicidae	Aedes	Aedes aegypti	SPECIES	24.63000	-81.55000	1264888865
13722	Diptera	Culicidae	Aedes	Aedes aegypti	SPECIES	24.64681	-107.43858	3784668447
13723	Diptera	Culicidae	Aedes	Aedes aegypti	SPECIES	24.66000	93.91000	1264887334
13724	Diptera	Culicidae	Aedes	Aedes aegypti	SPECIES	24.66000	-81.41000	1264888897
13725	Diptera	Culicidae	Aedes	Aedes aegypti	SPECIES	24.66931	-99.59044	1895892933
13726	Diptera	Culicidae	Aedes	Aedes aegypti	SPECIES	24.66931	-81.39091	3068155310
13727	Diptera	Culicidae	Aedes	Aedes aegypti	SPECIES	24.67000	-81.50000	1264888873
13728	Diptera	Culicidae	Aedes	Aedes aegypti	SPECIES	24.67000	-81.39000	1264888886
13729	Diptera	Culicidae	Aedes	Aedes aegypti	SPECIES	24.67000	-81.44000	1264888894
13730	Diptera	Culicidae	Aedes	Aedes aegypti	SPECIES	24.67125	-99.59314	1895892962
13731	Diptera	Culicidae	Aedes	Aedes aegypti	SPECIES	24.69000	-81.36000	1264888870
13732	Diptera	Culicidae	Aedes	Aedes aegypti	SPECIES	24.69000	-81.33000	1264888877
13733	Diptera	Culicidae	Aedes	Aedes aegypti	SPECIES	24.69372	-81.37310	3068161304
13734	Diptera	Culicidae	Aedes	Aedes aegypti	SPECIES	24.69697	-99.55725	1421476048
13735	Diptera	Culicidae	Aedes	Aedes aegypti	SPECIES	24.71105	-81.08521	3314090328

№	Order	Family	Genus	Species	Taxon Rank	Latitude	Longitude	GBIF_ID
13736	Diptera	Culicidae	Aedes	Aedes aegypti	SPECIES	24.71720	81.38730	3030814889
13737	Diptera	Culicidae	Aedes	Aedes aegypti	SPECIES	24.71903	-81.06896	3068157318
13738	Diptera	Culicidae	Aedes	Aedes aegypti	SPECIES	24.73272	-98.49694	1895893002
13739	Diptera	Culicidae	Aedes	Aedes aegypti	SPECIES	24.76000	-80.96000	3030927004
13740	Diptera	Culicidae	Aedes	Aedes aegypti	SPECIES	24.76300	-107.68306	2629263637
13741	Diptera	Culicidae	Aedes	Aedes aegypti	SPECIES	24.76606	-98.52292	1895892866
13742	Diptera	Culicidae	Aedes	Aedes aegypti	SPECIES	24.79194	-107.39806	2629263490
13743	Diptera	Culicidae	Aedes	Aedes aegypti	SPECIES	24.79199	-104.04173	3408212210
13744	Diptera	Culicidae	Aedes	Aedes aegypti	SPECIES	24.79335	-104.03828	2609334569
13745	Diptera	Culicidae	Aedes	Aedes aegypti	SPECIES	24.79345	-104.03879	3384261033
13746	Diptera	Culicidae	Aedes	Aedes aegypti	SPECIES	24.79917	-107.38528	1421476240
13747	Diptera	Culicidae	Aedes	Aedes aegypti	SPECIES	24.79917	-107.38444	1894399502
13748	Diptera	Culicidae	Aedes	Aedes aegypti	SPECIES	24.80000	-107.40000	1264888727
13749	Diptera	Culicidae	Aedes	Aedes aegypti	SPECIES	24.80194	-107.38428	2629263121
13750	Diptera	Culicidae	Aedes	Aedes aegypti	SPECIES	24.81000	-107.38000	1264886775
13751	Diptera	Culicidae	Aedes	Aedes aegypti	SPECIES	24.82618	-107.38008	3784934803
13752	Diptera	Culicidae	Aedes	Aedes aegypti	SPECIES	24.84750	-98.15833	1894398409
13753	Diptera	Culicidae	Aedes	Aedes aegypti	SPECIES	24.85000	67.01000	1264888941
13754	Diptera	Culicidae	Aedes	Aedes aegypti	SPECIES	24.85472	-98.11417	1895892980
13755	Diptera	Culicidae	Aedes	Aedes aegypti	SPECIES	24.85475	-98.11417	1895892817
13756	Diptera	Culicidae	Aedes	Aedes aegypti	SPECIES	24.85722	-99.58528	1895893038
13757	Diptera	Culicidae	Aedes	Aedes aegypti	SPECIES	24.85972	-99.56667	1894395750
13758	Diptera	Culicidae	Aedes	Aedes aegypti	SPECIES	24.86000	67.01000	1264887325
13759	Diptera	Culicidae	Aedes	Aedes aegypti	SPECIES	24.87389	-99.34222	1895892916
13760	Diptera	Culicidae	Aedes	Aedes aegypti	SPECIES	24.87528	-100.09806	1894400179
13761	Diptera	Culicidae	Aedes	Aedes aegypti	SPECIES	24.93000	-98.64000	1264890656
13762	Diptera	Culicidae	Aedes	Aedes aegypti	SPECIES	24.97111	-98.09861	1894396946
13763	Diptera	Culicidae	Aedes	Aedes aegypti	SPECIES	24.97158	-98.78203	1895892842
13764	Diptera	Culicidae	Aedes	Aedes aegypti	SPECIES	24.98000	-107.49000	1264891001
13765	Diptera	Culicidae	Aedes	Aedes aegypti	SPECIES	25.01419	-98.07908	1895892805
13766	Diptera	Culicidae	Aedes	Aedes aegypti	SPECIES	25.03039	-100.55667	1895892709
13767	Diptera	Culicidae	Aedes	Aedes aegypti	SPECIES	25.04000	-111.66000	1264888568
13768	Diptera	Culicidae	Aedes	Aedes aegypti	SPECIES	25.07000	-77.34000	1264889786
13769	Diptera	Culicidae	Aedes	Aedes aegypti	SPECIES	25.07730	55.13365	4162248491
13770	Diptera	Culicidae	Aedes	Aedes aegypti	SPECIES	25.08911	-107.69014	2629263956
13771	Diptera	Culicidae	Aedes	Aedes aegypti	SPECIES	25.13000	76.13000	1264890778
13772	Diptera	Culicidae	Aedes	Aedes aegypti	SPECIES	25.14242	-80.39522	3858160894
13773	Diptera	Culicidae	Aedes	Aedes aegypti	SPECIES	25.18000	75.84000	1264890611
13774	Diptera	Culicidae	Aedes	Aedes aegypti	SPECIES	25.18694	-99.82639	1421473891
13775	Diptera	Culicidae	Aedes	Aedes aegypti	SPECIES	25.23167	-97.89361	1894397802
13776	Diptera	Culicidae	Aedes	Aedes aegypti	SPECIES	25.24103	-97.43958	1418704082

№	Order	Family	Genus	Species	Taxon Rank	Latitude	Longitude	GBIF_ID
13777	Diptera	Culicidae	Aedes	Aedes aegypti	SPECIES	25.25222	-100.03528	1421478113
13778	Diptera	Culicidae	Aedes	Aedes aegypti	SPECIES	25.26147	-98.68125	1895892922
13779	Diptera	Culicidae	Aedes	Aedes aegypti	SPECIES	25.28000	110.28000	1264889125
13780	Diptera	Culicidae	Aedes	Aedes aegypti	SPECIES	25.28333	-100.02000	1421477566
13781	Diptera	Culicidae	Aedes	Aedes aegypti	SPECIES	25.28655	-100.00675	1895893016
13782	Diptera	Culicidae	Aedes	Aedes aegypti	SPECIES	25.29000	-100.01000	1264886801
13783	Diptera	Culicidae	Aedes	Aedes aegypti	SPECIES	25.29167	-108.51469	2629265590
13784	Diptera	Culicidae	Aedes	Aedes aegypti	SPECIES	25.29903	-100.00577	1895892956
13785	Diptera	Culicidae	Aedes	Aedes aegypti	SPECIES	25.30000	-100.04000	1264889704
13786	Diptera	Culicidae	Aedes	Aedes aegypti	SPECIES	25.30785	-100.04968	1895892823
13787	Diptera	Culicidae	Aedes	Aedes aegypti	SPECIES	25.31000	83.01000	1264889205
13788	Diptera	Culicidae	Aedes	Aedes aegypti	SPECIES	25.35000	72.61000	1264891374
13789	Diptera	Culicidae	Aedes	Aedes aegypti	SPECIES	25.36750	-100.43528	1421475868
13790	Diptera	Culicidae	Aedes	Aedes aegypti	SPECIES	25.36867	-100.20077	1421475217
13791	Diptera	Culicidae	Aedes	Aedes aegypti	SPECIES	25.38000	68.37000	1264887312
13792	Diptera	Culicidae	Aedes	Aedes aegypti	SPECIES	25.38589	-99.92675	1895892950
13793	Diptera	Culicidae	Aedes	Aedes aegypti	SPECIES	25.38969	-99.92750	1895892906
13794	Diptera	Culicidae	Aedes	Aedes aegypti	SPECIES	25.40453	-100.13353	1421477063
13795	Diptera	Culicidae	Aedes	Aedes aegypti	SPECIES	25.42459	-100.15131	1621789911
13796	Diptera	Culicidae	Aedes	Aedes aegypti	SPECIES	25.42567	-100.14537	1421475196
13797	Diptera	Culicidae	Aedes	Aedes aegypti	SPECIES	25.42667	-100.15222	1421477563
13798	Diptera	Culicidae	Aedes	Aedes aegypti	SPECIES	25.43000	92.20000	1264888455
13799	Diptera	Culicidae	Aedes	Aedes aegypti	SPECIES	25.45161	-108.07900	2629262338
13800	Diptera	Culicidae	Aedes	Aedes aegypti	SPECIES	25.46528	-100.21444	1421475867
13801	Diptera	Culicidae	Aedes	Aedes aegypti	SPECIES	25.46566	-99.95622	3892470748
13802	Diptera	Culicidae	Aedes	Aedes aegypti	SPECIES	25.47000	-100.17000	1264888722
13803	Diptera	Culicidae	Aedes	Aedes aegypti	SPECIES	25.47540	-103.55413	3337531685
13804	Diptera	Culicidae	Aedes	Aedes aegypti	SPECIES	25.48000	-80.49000	1264889987
13805	Diptera	Culicidae	Aedes	Aedes aegypti	SPECIES	25.49000	-80.99000	1264871578
13806	Diptera	Culicidae	Aedes	Aedes aegypti	SPECIES	25.49528	-103.59500	1421478649
13807	Diptera	Culicidae	Aedes	Aedes aegypti	SPECIES	25.49889	-103.61194	1421475364
13808	Diptera	Culicidae	Aedes	Aedes aegypti	SPECIES	25.50000	-100.08000	1264888763
13809	Diptera	Culicidae	Aedes	Aedes aegypti	SPECIES	25.51000	90.65000	1264888427
13810	Diptera	Culicidae	Aedes	Aedes aegypti	SPECIES	25.52000	90.23000	1264888479
13811	Diptera	Culicidae	Aedes	Aedes aegypti	SPECIES	25.53000	-108.60000	1264891293
13812	Diptera	Culicidae	Aedes	Aedes aegypti	SPECIES	25.53222	-103.43517	2459989659
13813	Diptera	Culicidae	Aedes	Aedes aegypti	SPECIES	25.53944	-100.94750	1894400172
13814	Diptera	Culicidae	Aedes	Aedes aegypti	SPECIES	25.53988	-103.38121	2963760505
13815	Diptera	Culicidae	Aedes	Aedes aegypti	SPECIES	25.54152	-103.54656	1421478752
13816	Diptera	Culicidae	Aedes	Aedes aegypti	SPECIES	25.55000	-103.37000	1264886679
13817	Diptera	Culicidae	Aedes	Aedes aegypti	SPECIES	25.55000	-108.46000	1264886763

№	Order	Family	Genus	Species	Taxon Rank	Latitude	Longitude	GBIF_ID
13818	Diptera	Culicidae	Aedes	Aedes aegypti	SPECIES	25.55034	-103.43336	3773014549
13819	Diptera	Culicidae	Aedes	Aedes aegypti	SPECIES	25.55930	-103.32008	1421475655
13820	Diptera	Culicidae	Aedes	Aedes aegypti	SPECIES	25.56000	-108.47000	1264886766
13821	Diptera	Culicidae	Aedes	Aedes aegypti	SPECIES	25.56147	-103.32475	1421476378
13822	Diptera	Culicidae	Aedes	Aedes aegypti	SPECIES	25.56845	-80.40423	3113403402
13823	Diptera	Culicidae	Aedes	Aedes aegypti	SPECIES	25.56867	-108.44008	2629265468
13824	Diptera	Culicidae	Aedes	Aedes aegypti	SPECIES	25.56897	-108.46914	2629263324
13825	Diptera	Culicidae	Aedes	Aedes aegypti	SPECIES	25.57000	-108.47000	1264886732
13826	Diptera	Culicidae	Aedes	Aedes aegypti	SPECIES	25.57000	91.88000	1264888464
13827	Diptera	Culicidae	Aedes	Aedes aegypti	SPECIES	25.58000	-108.47000	1264886753
13828	Diptera	Culicidae	Aedes	Aedes aegypti	SPECIES	25.58000	-108.46000	1264886776
13829	Diptera	Culicidae	Aedes	Aedes aegypti	SPECIES	25.58167	-100.12361	1894396188
13830	Diptera	Culicidae	Aedes	Aedes aegypti	SPECIES	25.59000	-108.46000	1264886743
13831	Diptera	Culicidae	Aedes	Aedes aegypti	SPECIES	25.59028	-100.00111	1894397150
13832	Diptera	Culicidae	Aedes	Aedes aegypti	SPECIES	25.60057	-99.98683	1421477973
13833	Diptera	Culicidae	Aedes	Aedes aegypti	SPECIES	25.60239	-100.25803	1421475042
13834	Diptera	Culicidae	Aedes	Aedes aegypti	SPECIES	25.60347	-80.56056	3112612680
13835	Diptera	Culicidae	Aedes	Aedes aegypti	SPECIES	25.61000	-100.28000	1264887463
13836	Diptera	Culicidae	Aedes	Aedes aegypti	SPECIES	25.61028	-100.15417	1894399711
13837	Diptera	Culicidae	Aedes	Aedes aegypti	SPECIES	25.62000	-80.35000	1264890230
13838	Diptera	Culicidae	Aedes	Aedes aegypti	SPECIES	25.62193	-80.33954	3330480063
13839	Diptera	Culicidae	Aedes	Aedes aegypti	SPECIES	25.62216	-80.33973	2860094226
13840	Diptera	Culicidae	Aedes	Aedes aegypti	SPECIES	25.62237	-80.34119	3330538317
13841	Diptera	Culicidae	Aedes	Aedes aegypti	SPECIES	25.62239	-80.34116	3499615039
13842	Diptera	Culicidae	Aedes	Aedes aegypti	SPECIES	25.62241	-80.33943	3398748672
13843	Diptera	Culicidae	Aedes	Aedes aegypti	SPECIES	25.62244	-80.34017	3330499627
13844	Diptera	Culicidae	Aedes	Aedes aegypti	SPECIES	25.62253	-80.33919	3017976576
13845	Diptera	Culicidae	Aedes	Aedes aegypti	SPECIES	25.62261	-80.34063	2859936698
13846	Diptera	Culicidae	Aedes	Aedes aegypti	SPECIES	25.62299	-80.33968	3328069604
13847	Diptera	Culicidae	Aedes	Aedes aegypti	SPECIES	25.62583	-100.15667	1894399192
13848	Diptera	Culicidae	Aedes	Aedes aegypti	SPECIES	25.62867	-80.43124	3070314006
13849	Diptera	Culicidae	Aedes	Aedes aegypti	SPECIES	25.62995	-80.35831	3912143551
13850	Diptera	Culicidae	Aedes	Aedes aegypti	SPECIES	25.63019	-97.20153	1418703940
13851	Diptera	Culicidae	Aedes	Aedes aegypti	SPECIES	25.63133	-80.38326	3772909785
13852	Diptera	Culicidae	Aedes	Aedes aegypti	SPECIES	25.63232	-80.43568	3070294022
13853	Diptera	Culicidae	Aedes	Aedes aegypti	SPECIES	25.63261	-80.23699	3773347950
13854	Diptera	Culicidae	Aedes	Aedes aegypti	SPECIES	25.63444	-100.12806	1894399486
13855	Diptera	Culicidae	Aedes	Aedes aegypti	SPECIES	25.63779	-100.26294	3773485725
13856	Diptera	Culicidae	Aedes	Aedes aegypti	SPECIES	25.63786	-100.26292	3858572682
13857	Diptera	Culicidae	Aedes	Aedes aegypti	SPECIES	25.63794	-100.26302	3058735197
13858	Diptera	Culicidae	Aedes	Aedes aegypti	SPECIES	25.64067	-100.08920	1895892874

№	Order	Family	Genus	Species	Taxon Rank	Latitude	Longitude	GBIF_ID
13859	Diptera	Culicidae	Aedes	Aedes aegypti	SPECIES	25.64105	-100.27803	1895893022
13860	Diptera	Culicidae	Aedes	Aedes aegypti	SPECIES	25.64250	-97.63611	1894396558
13861	Diptera	Culicidae	Aedes	Aedes aegypti	SPECIES	25.64252	-100.24416	3925426998
13862	Diptera	Culicidae	Aedes	Aedes aegypti	SPECIES	25.64288	-100.31822	3031622967
13863	Diptera	Culicidae	Aedes	Aedes aegypti	SPECIES	25.64722	-100.11528	1894400028
13864	Diptera	Culicidae	Aedes	Aedes aegypti	SPECIES	25.64725	-100.26591	3090525045
13865	Diptera	Culicidae	Aedes	Aedes aegypti	SPECIES	25.64730	-100.26587	3399417334
13866	Diptera	Culicidae	Aedes	Aedes aegypti	SPECIES	25.64732	-100.26590	3985640163
13867	Diptera	Culicidae	Aedes	Aedes aegypti	SPECIES	25.64733	-100.26590	3330747518
13868	Diptera	Culicidae	Aedes	Aedes aegypti	SPECIES	25.64733	-100.26588	3327970543
13869	Diptera	Culicidae	Aedes	Aedes aegypti	SPECIES	25.64733	-100.26593	3985880704
13870	Diptera	Culicidae	Aedes	Aedes aegypti	SPECIES	25.64735	-100.26584	3307333402
13871	Diptera	Culicidae	Aedes	Aedes aegypti	SPECIES	25.65743	-100.41010	1895892904
13872	Diptera	Culicidae	Aedes	Aedes aegypti	SPECIES	25.65792	-100.27492	1421477559
13873	Diptera	Culicidae	Aedes	Aedes aegypti	SPECIES	25.65864	-100.17594	1895893014
13874	Diptera	Culicidae	Aedes	Aedes aegypti	SPECIES	25.65867	-100.17750	1421477094
13875	Diptera	Culicidae	Aedes	Aedes aegypti	SPECIES	25.65867	-100.17750	1895892968
13876	Diptera	Culicidae	Aedes	Aedes aegypti	SPECIES	25.65925	-100.17656	1421475745
13877	Diptera	Culicidae	Aedes	Aedes aegypti	SPECIES	25.66389	-100.40139	1894399675
13878	Diptera	Culicidae	Aedes	Aedes aegypti	SPECIES	25.66444	-100.07694	1894397787
13879	Diptera	Culicidae	Aedes	Aedes aegypti	SPECIES	25.66794	-100.36525	1895893012
13880	Diptera	Culicidae	Aedes	Aedes aegypti	SPECIES	25.67000	94.11000	1264886665
13881	Diptera	Culicidae	Aedes	Aedes aegypti	SPECIES	25.67000	-100.30000	1264888738
13882	Diptera	Culicidae	Aedes	Aedes aegypti	SPECIES	25.67111	-100.30833	1894400226
13883	Diptera	Culicidae	Aedes	Aedes aegypti	SPECIES	25.67139	-100.30861	2629264547
13884	Diptera	Culicidae	Aedes	Aedes aegypti	SPECIES	25.67528	-100.46194	1894400175
13885	Diptera	Culicidae	Aedes	Aedes aegypti	SPECIES	25.67573	-80.26581	4103171268
13886	Diptera	Culicidae	Aedes	Aedes aegypti	SPECIES	25.67889	-100.23472	1894400265
13887	Diptera	Culicidae	Aedes	Aedes aegypti	SPECIES	25.68000	-100.37000	1264888726
13888	Diptera	Culicidae	Aedes	Aedes aegypti	SPECIES	25.68000	-80.27000	1264889536
13889	Diptera	Culicidae	Aedes	Aedes aegypti	SPECIES	25.68000	-100.31000	1264889816
13890	Diptera	Culicidae	Aedes	Aedes aegypti	SPECIES	25.68000	-100.29000	1264889885
13891	Diptera	Culicidae	Aedes	Aedes aegypti	SPECIES	25.68400	-80.33884	4052761538
13892	Diptera	Culicidae	Aedes	Aedes aegypti	SPECIES	25.68550	-100.24572	1421476888
13893	Diptera	Culicidae	Aedes	Aedes aegypti	SPECIES	25.68691	-80.15989	4165547516
13894	Diptera	Culicidae	Aedes	Aedes aegypti	SPECIES	25.69000	-100.32000	1264886838
13895	Diptera	Culicidae	Aedes	Aedes aegypti	SPECIES	25.69248	-100.30065	1421474882
13896	Diptera	Culicidae	Aedes	Aedes aegypti	SPECIES	25.69353	-97.78953	1895893030
13897	Diptera	Culicidae	Aedes	Aedes aegypti	SPECIES	25.69675	-100.29181	1421478823
13898	Diptera	Culicidae	Aedes	Aedes aegypti	SPECIES	25.69788	-100.25978	1421475656
13899	Diptera	Culicidae	Aedes	Aedes aegypti	SPECIES	25.69903	-99.62425	1895892942

№	Order	Family	Genus	Species	Taxon Rank	Latitude	Longitude	GBIF_ID
13900	Diptera	Culicidae	Aedes	Aedes aegypti	SPECIES	25.70000	94.11000	1264888438
13901	Diptera	Culicidae	Aedes	Aedes aegypti	SPECIES	25.70067	-100.29728	1421476810
13902	Diptera	Culicidae	Aedes	Aedes aegypti	SPECIES	25.70166	-100.35022	3966557678
13903	Diptera	Culicidae	Aedes	Aedes aegypti	SPECIES	25.70169	-100.35024	3966558704
13904	Diptera	Culicidae	Aedes	Aedes aegypti	SPECIES	25.70254	-100.33867	3947495329
13905	Diptera	Culicidae	Aedes	Aedes aegypti	SPECIES	25.70442	-99.24525	1895892943
13906	Diptera	Culicidae	Aedes	Aedes aegypti	SPECIES	25.70472	-99.24414	1895892771
13907	Diptera	Culicidae	Aedes	Aedes aegypti	SPECIES	25.70556	-99.23972	1895893026
13908	Diptera	Culicidae	Aedes	Aedes aegypti	SPECIES	25.70683	-100.35244	1421477799
13909	Diptera	Culicidae	Aedes	Aedes aegypti	SPECIES	25.70757	-80.40433	4067558554
13910	Diptera	Culicidae	Aedes	Aedes aegypti	SPECIES	25.70930	-99.25083	1421478083
13911	Diptera	Culicidae	Aedes	Aedes aegypti	SPECIES	25.71461	-99.22650	1895892966
13912	Diptera	Culicidae	Aedes	Aedes aegypti	SPECIES	25.71672	-100.33900	1421478840
13913	Diptera	Culicidae	Aedes	Aedes aegypti	SPECIES	25.72011	-80.21693	2634131919
13914	Diptera	Culicidae	Aedes	Aedes aegypti	SPECIES	25.72136	-97.16914	1418703972
13915	Diptera	Culicidae	Aedes	Aedes aegypti	SPECIES	25.72200	-80.33873	4145299536
13916	Diptera	Culicidae	Aedes	Aedes aegypti	SPECIES	25.72201	-80.33878	3044724507
13917	Diptera	Culicidae	Aedes	Aedes aegypti	SPECIES	25.72658	-99.29742	1895892944
13918	Diptera	Culicidae	Aedes	Aedes aegypti	SPECIES	25.72872	-100.31431	1421478164
13919	Diptera	Culicidae	Aedes	Aedes aegypti	SPECIES	25.72917	-99.34139	1894396118
13920	Diptera	Culicidae	Aedes	Aedes aegypti	SPECIES	25.72922	-100.30731	1421477800
13921	Diptera	Culicidae	Aedes	Aedes aegypti	SPECIES	25.72944	-100.26883	1421478031
13922	Diptera	Culicidae	Aedes	Aedes aegypti	SPECIES	25.73000	-100.31000	1264887418
13923	Diptera	Culicidae	Aedes	Aedes aegypti	SPECIES	25.73363	-80.35997	3966583845
13924	Diptera	Culicidae	Aedes	Aedes aegypti	SPECIES	25.73398	-80.33230	3112520480
13925	Diptera	Culicidae	Aedes	Aedes aegypti	SPECIES	25.73708	-100.25678	1895893028
13926	Diptera	Culicidae	Aedes	Aedes aegypti	SPECIES	25.74083	-80.43000	3333268336
13927	Diptera	Culicidae	Aedes	Aedes aegypti	SPECIES	25.74139	-100.45056	1421474703
13928	Diptera	Culicidae	Aedes	Aedes aegypti	SPECIES	25.74243	-80.33482	4096662575
13929	Diptera	Culicidae	Aedes	Aedes aegypti	SPECIES	25.75650	-100.29917	1421475786
13930	Diptera	Culicidae	Aedes	Aedes aegypti	SPECIES	25.75698	-80.32850	2826336943
13931	Diptera	Culicidae	Aedes	Aedes aegypti	SPECIES	25.75826	-80.33461	4075727919
13932	Diptera	Culicidae	Aedes	Aedes aegypti	SPECIES	25.75833	-102.97889	1421474114
13933	Diptera	Culicidae	Aedes	Aedes aegypti	SPECIES	25.75855	-80.34272	4091559463
13934	Diptera	Culicidae	Aedes	Aedes aegypti	SPECIES	25.75988	-80.32203	3320916122
13935	Diptera	Culicidae	Aedes	Aedes aegypti	SPECIES	25.76000	93.87000	1264886667
13936	Diptera	Culicidae	Aedes	Aedes aegypti	SPECIES	25.76056	-100.32075	1421477437
13937	Diptera	Culicidae	Aedes	Aedes aegypti	SPECIES	25.76347	-80.33739	2984399648
13938	Diptera	Culicidae	Aedes	Aedes aegypti	SPECIES	25.77000	93.90000	1264888415
13939	Diptera	Culicidae	Aedes	Aedes aegypti	SPECIES	25.77297	-100.31825	1421473755
13940	Diptera	Culicidae	Aedes	Aedes aegypti	SPECIES	25.77584	-80.34986	4127185303

№	Order	Family	Genus	Species	Taxon Rank	Latitude	Longitude	GBIF_ID
13941	Diptera	Culicidae	Aedes	Aedes aegypti	SPECIES	25.77994	-100.04531	1895892991
13942	Diptera	Culicidae	Aedes	Aedes aegypti	SPECIES	25.78000	-103.27000	1264890370
13943	Diptera	Culicidae	Aedes	Aedes aegypti	SPECIES	25.78108	-100.05028	1895892995
13944	Diptera	Culicidae	Aedes	Aedes aegypti	SPECIES	25.78139	-100.18861	1894400002
13945	Diptera	Culicidae	Aedes	Aedes aegypti	SPECIES	25.78217	-108.98986	2629264680
13946	Diptera	Culicidae	Aedes	Aedes aegypti	SPECIES	25.78294	-99.18561	1895892869
13947	Diptera	Culicidae	Aedes	Aedes aegypti	SPECIES	25.78472	-100.05083	1421477554
13948	Diptera	Culicidae	Aedes	Aedes aegypti	SPECIES	25.78650	-99.18469	1895893053
13949	Diptera	Culicidae	Aedes	Aedes aegypti	SPECIES	25.78733	-100.06418	1895892953
13950	Diptera	Culicidae	Aedes	Aedes aegypti	SPECIES	25.79194	-100.19972	1894397186
13951	Diptera	Culicidae	Aedes	Aedes aegypti	SPECIES	25.79333	-100.38861	1894399610
13952	Diptera	Culicidae	Aedes	Aedes aegypti	SPECIES	25.79394	-80.39264	3330450533
13953	Diptera	Culicidae	Aedes	Aedes aegypti	SPECIES	25.79471	-80.34401	3912205769
13954	Diptera	Culicidae	Aedes	Aedes aegypti	SPECIES	25.79908	-100.46878	1421475523
13955	Diptera	Culicidae	Aedes	Aedes aegypti	SPECIES	25.80000	93.78000	1264886674
13956	Diptera	Culicidae	Aedes	Aedes aegypti	SPECIES	25.80106	-100.27544	1421473462
13957	Diptera	Culicidae	Aedes	Aedes aegypti	SPECIES	25.80167	-100.27642	1421474353
13958	Diptera	Culicidae	Aedes	Aedes aegypti	SPECIES	25.80628	-99.17463	1895892737
13959	Diptera	Culicidae	Aedes	Aedes aegypti	SPECIES	25.80778	-107.55789	2629265244
13960	Diptera	Culicidae	Aedes	Aedes aegypti	SPECIES	25.81000	-98.37000	1264890785
13961	Diptera	Culicidae	Aedes	Aedes aegypti	SPECIES	25.81028	-100.24444	1894399386
13962	Diptera	Culicidae	Aedes	Aedes aegypti	SPECIES	25.81194	-100.59889	1894394298
13963	Diptera	Culicidae	Aedes	Aedes aegypti	SPECIES	25.82000	-100.02000	1264889328
13964	Diptera	Culicidae	Aedes	Aedes aegypti	SPECIES	25.83000	-80.22000	1264889986
13965	Diptera	Culicidae	Aedes	Aedes aegypti	SPECIES	25.83658	-100.14263	1895893052
13966	Diptera	Culicidae	Aedes	Aedes aegypti	SPECIES	25.84519	-97.50736	1895892928
13967	Diptera	Culicidae	Aedes	Aedes aegypti	SPECIES	25.85363	-80.19411	3384516141
13968	Diptera	Culicidae	Aedes	Aedes aegypti	SPECIES	25.85373	-80.19418	4062583280
13969	Diptera	Culicidae	Aedes	Aedes aegypti	SPECIES	25.85383	-80.19415	4034915890
13970	Diptera	Culicidae	Aedes	Aedes aegypti	SPECIES	25.85623	-99.94933	1421477867
13971	Diptera	Culicidae	Aedes	Aedes aegypti	SPECIES	25.85623	-99.94943	1895892993
13972	Diptera	Culicidae	Aedes	Aedes aegypti	SPECIES	25.85935	-80.18522	3963226102
13973	Diptera	Culicidae	Aedes	Aedes aegypti	SPECIES	25.86303	-97.49722	1895892775
13974	Diptera	Culicidae	Aedes	Aedes aegypti	SPECIES	25.86585	-80.18415	4039311772
13975	Diptera	Culicidae	Aedes	Aedes aegypti	SPECIES	25.86981	-100.28914	1421475021
13976	Diptera	Culicidae	Aedes	Aedes aegypti	SPECIES	25.87000	-97.50000	1264887040
13977	Diptera	Culicidae	Aedes	Aedes aegypti	SPECIES	25.87000	-97.51000	1264890864
13978	Diptera	Culicidae	Aedes	Aedes aegypti	SPECIES	25.87306	-97.62056	1894399457
13979	Diptera	Culicidae	Aedes	Aedes aegypti	SPECIES	25.87778	-103.61694	1421476841
13980	Diptera	Culicidae	Aedes	Aedes aegypti	SPECIES	25.87917	-100.03167	1894399132
13981	Diptera	Culicidae	Aedes	Aedes aegypti	SPECIES	25.87972	-97.50417	1894400098

№	Order	Family	Genus	Species	Taxon Rank	Latitude	Longitude	GBIF_ID
13982	Diptera	Culicidae	Aedes	Aedes aegypti	SPECIES	25.88000	-97.50000	1264891184
13983	Diptera	Culicidae	Aedes	Aedes aegypti	SPECIES	25.88219	-100.28717	1421476917
13984	Diptera	Culicidae	Aedes	Aedes aegypti	SPECIES	25.88247	-100.34581	1421475650
13985	Diptera	Culicidae	Aedes	Aedes aegypti	SPECIES	25.89102	-100.11578	1895892831
13986	Diptera	Culicidae	Aedes	Aedes aegypti	SPECIES	25.89528	-100.10750	1421476192
13987	Diptera	Culicidae	Aedes	Aedes aegypti	SPECIES	25.90000	-97.50000	1264890898
13988	Diptera	Culicidae	Aedes	Aedes aegypti	SPECIES	25.90175	-97.49748	3802038336
13989	Diptera	Culicidae	Aedes	Aedes aegypti	SPECIES	25.90472	-99.40222	1894400162
13990	Diptera	Culicidae	Aedes	Aedes aegypti	SPECIES	25.91000	-97.50000	1264890891
13991	Diptera	Culicidae	Aedes	Aedes aegypti	SPECIES	25.91958	-108.98581	2629264398
13992	Diptera	Culicidae	Aedes	Aedes aegypti	SPECIES	25.92000	93.74000	1264888433
13993	Diptera	Culicidae	Aedes	Aedes aegypti	SPECIES	25.92552	-100.16027	1421473506
13994	Diptera	Culicidae	Aedes	Aedes aegypti	SPECIES	25.94261	-109.05019	2629264980
13995	Diptera	Culicidae	Aedes	Aedes aegypti	SPECIES	25.94493	-80.27587	2251947466
13996	Diptera	Culicidae	Aedes	Aedes aegypti	SPECIES	25.94550	-100.41311	1895892828
13997	Diptera	Culicidae	Aedes	Aedes aegypti	SPECIES	25.94553	-100.41308	1895892917
13998	Diptera	Culicidae	Aedes	Aedes aegypti	SPECIES	25.95000	-100.17000	1264887421
13999	Diptera	Culicidae	Aedes	Aedes aegypti	SPECIES	25.95111	-97.73917	1894397635
14000	Diptera	Culicidae	Aedes	Aedes aegypti	SPECIES	25.95270	-100.17248	1895892992
14001	Diptera	Culicidae	Aedes	Aedes aegypti	SPECIES	25.96250	-100.29167	1895892887
14002	Diptera	Culicidae	Aedes	Aedes aegypti	SPECIES	25.97068	-100.43948	1421475651
14003	Diptera	Culicidae	Aedes	Aedes aegypti	SPECIES	25.97372	-100.28390	1895893023
14004	Diptera	Culicidae	Aedes	Aedes aegypti	SPECIES	25.98000	-97.60000	1264890319
14005	Diptera	Culicidae	Aedes	Aedes aegypti	SPECIES	25.98120	-80.14838	3314079324
14006	Diptera	Culicidae	Aedes	Aedes aegypti	SPECIES	25.98220	-80.18081	3963373081
14007	Diptera	Culicidae	Aedes	Aedes aegypti	SPECIES	25.99611	-100.52944	1895892957
14008	Diptera	Culicidae	Aedes	Aedes aegypti	SPECIES	25.99833	-105.52806	1894398232
14009	Diptera	Culicidae	Aedes	Aedes aegypti	SPECIES	26.00111	-100.51111	1895892900
14010	Diptera	Culicidae	Aedes	Aedes aegypti	SPECIES	26.00394	-98.09050	1895892815
14011	Diptera	Culicidae	Aedes	Aedes aegypti	SPECIES	26.00944	-98.57417	1421475965
14012	Diptera	Culicidae	Aedes	Aedes aegypti	SPECIES	26.01000	-98.29000	1264888959
14013	Diptera	Culicidae	Aedes	Aedes aegypti	SPECIES	26.01371	-80.36271	3337717313
14014	Diptera	Culicidae	Aedes	Aedes aegypti	SPECIES	26.02000	89.98000	1264888416
14015	Diptera	Culicidae	Aedes	Aedes aegypti	SPECIES	26.02000	-111.36000	1264888595
14016	Diptera	Culicidae	Aedes	Aedes aegypti	SPECIES	26.02000	-97.54000	1264890303
14017	Diptera	Culicidae	Aedes	Aedes aegypti	SPECIES	26.02714	-99.29114	1895892798
14018	Diptera	Culicidae	Aedes	Aedes aegypti	SPECIES	26.03000	91.87000	1264888431
14019	Diptera	Culicidae	Aedes	Aedes aegypti	SPECIES	26.03128	-80.16937	3391547012
14020	Diptera	Culicidae	Aedes	Aedes aegypti	SPECIES	26.05000	-97.76000	1264890331
14021	Diptera	Culicidae	Aedes	Aedes aegypti	SPECIES	26.05038	-80.30774	3384109744
14022	Diptera	Culicidae	Aedes	Aedes aegypti	SPECIES	26.05854	-80.22445	3456920302

№	Order	Family	Genus	Species	Taxon Rank	Latitude	Longitude	GBIF_ID
14023	Diptera	Culicidae	Aedes	Aedes aegypti	SPECIES	26.06481	-98.26158	1895893021
14024	Diptera	Culicidae	Aedes	Aedes aegypti	SPECIES	26.07438	-97.22502	2466129940
14025	Diptera	Culicidae	Aedes	Aedes aegypti	SPECIES	26.08000	-98.29000	1264890658
14026	Diptera	Culicidae	Aedes	Aedes aegypti	SPECIES	26.08000	-98.28000	1264891151
14027	Diptera	Culicidae	Aedes	Aedes aegypti	SPECIES	26.08028	-99.18111	1894397996
14028	Diptera	Culicidae	Aedes	Aedes aegypti	SPECIES	26.09000	-98.28000	1264887019
14029	Diptera	Culicidae	Aedes	Aedes aegypti	SPECIES	26.09000	-98.29000	1264888748
14030	Diptera	Culicidae	Aedes	Aedes aegypti	SPECIES	26.09152	-80.23152	2634102304
14031	Diptera	Culicidae	Aedes	Aedes aegypti	SPECIES	26.10000	-98.27000	1264890292
14032	Diptera	Culicidae	Aedes	Aedes aegypti	SPECIES	26.11000	91.47000	3350951862
14033	Diptera	Culicidae	Aedes	Aedes aegypti	SPECIES	26.11023	-80.23640	2626592731
14034	Diptera	Culicidae	Aedes	Aedes aegypti	SPECIES	26.12000	-81.33000	1264871575
14035	Diptera	Culicidae	Aedes	Aedes aegypti	SPECIES	26.12000	-80.15000	1264888120
14036	Diptera	Culicidae	Aedes	Aedes aegypti	SPECIES	26.12000	-97.53000	1264890852
14037	Diptera	Culicidae	Aedes	Aedes aegypti	SPECIES	26.13000	-97.55000	1264871567
14038	Diptera	Culicidae	Aedes	Aedes aegypti	SPECIES	26.13000	-97.63000	1264890294
14039	Diptera	Culicidae	Aedes	Aedes aegypti	SPECIES	26.13911	-80.25192	4121140593
14040	Diptera	Culicidae	Aedes	Aedes aegypti	SPECIES	26.14000	-97.91000	1264890299
14041	Diptera	Culicidae	Aedes	Aedes aegypti	SPECIES	26.14204	-81.79481	3039176774
14042	Diptera	Culicidae	Aedes	Aedes aegypti	SPECIES	26.14241	-80.24470	4091423866
14043	Diptera	Culicidae	Aedes	Aedes aegypti	SPECIES	26.15000	-80.49000	1264871576
14044	Diptera	Culicidae	Aedes	Aedes aegypti	SPECIES	26.15000	-97.98000	1264890265
14045	Diptera	Culicidae	Aedes	Aedes aegypti	SPECIES	26.15128	91.66271	2598665275
14046	Diptera	Culicidae	Aedes	Aedes aegypti	SPECIES	26.15667	-80.14999	2237422089
14047	Diptera	Culicidae	Aedes	Aedes aegypti	SPECIES	26.16000	-97.83000	1264890266
14048	Diptera	Culicidae	Aedes	Aedes aegypti	SPECIES	26.17000	-98.05000	1264890307
14049	Diptera	Culicidae	Aedes	Aedes aegypti	SPECIES	26.18000	91.75000	1264888429
14050	Diptera	Culicidae	Aedes	Aedes aegypti	SPECIES	26.18000	90.62000	1264888471
14051	Diptera	Culicidae	Aedes	Aedes aegypti	SPECIES	26.18193	-80.12595	3456626413
14052	Diptera	Culicidae	Aedes	Aedes aegypti	SPECIES	26.18195	-80.12607	3759438154
14053	Diptera	Culicidae	Aedes	Aedes aegypti	SPECIES	26.18195	-80.12599	3407970971
14054	Diptera	Culicidae	Aedes	Aedes aegypti	SPECIES	26.18197	-80.12595	3415804916
14055	Diptera	Culicidae	Aedes	Aedes aegypti	SPECIES	26.18197	-80.12604	3337625363
14056	Diptera	Culicidae	Aedes	Aedes aegypti	SPECIES	26.18197	-80.12601	3344116493
14057	Diptera	Culicidae	Aedes	Aedes aegypti	SPECIES	26.18200	-80.12602	3384438885
14058	Diptera	Culicidae	Aedes	Aedes aegypti	SPECIES	26.18269	-98.33095	2265806311
14059	Diptera	Culicidae	Aedes	Aedes aegypti	SPECIES	26.18462	-80.12978	3455436879
14060	Diptera	Culicidae	Aedes	Aedes aegypti	SPECIES	26.19000	-97.69000	1264890305
14061	Diptera	Culicidae	Aedes	Aedes aegypti	SPECIES	26.20000	-98.19000	1264890308
14062	Diptera	Culicidae	Aedes	Aedes aegypti	SPECIES	26.20000	-98.23000	1264890333
14063	Diptera	Culicidae	Aedes	Aedes aegypti	SPECIES	26.20264	-80.15524	2350395128

№	Order	Family	Genus	Species	Taxon Rank	Latitude	Longitude	GBIF_ID
14064	Diptera	Culicidae	Aedes	Aedes aegypti	SPECIES	26.20371	-98.26693	1841192162
14065	Diptera	Culicidae	Aedes	Aedes aegypti	SPECIES	26.21000	-98.23000	1264889907
14066	Diptera	Culicidae	Aedes	Aedes aegypti	SPECIES	26.23159	-98.21122	3859591102
14067	Diptera	Culicidae	Aedes	Aedes aegypti	SPECIES	26.23689	-98.56897	1895892971
14068	Diptera	Culicidae	Aedes	Aedes aegypti	SPECIES	26.23979	-98.23310	4046768307
14069	Diptera	Culicidae	Aedes	Aedes aegypti	SPECIES	26.25000	-98.48000	1264890365
14070	Diptera	Culicidae	Aedes	Aedes aegypti	SPECIES	26.26697	-80.18823	3109155366
14071	Diptera	Culicidae	Aedes	Aedes aegypti	SPECIES	26.28000	73.02000	1264890645
14072	Diptera	Culicidae	Aedes	Aedes aegypti	SPECIES	26.30580	-80.21270	3961249863
14073	Diptera	Culicidae	Aedes	Aedes aegypti	SPECIES	26.31056	-99.53889	1894397209
14074	Diptera	Culicidae	Aedes	Aedes aegypti	SPECIES	26.32000	94.51000	1264886644
14075	Diptera	Culicidae	Aedes	Aedes aegypti	SPECIES	26.33480	-81.77500	3030961684
14076	Diptera	Culicidae	Aedes	Aedes aegypti	SPECIES	26.34500	-100.05000	1894398903
14077	Diptera	Culicidae	Aedes	Aedes aegypti	SPECIES	26.34556	-99.94000	1894397358
14078	Diptera	Culicidae	Aedes	Aedes aegypti	SPECIES	26.34561	-109.02208	2629264376
14079	Diptera	Culicidae	Aedes	Aedes aegypti	SPECIES	26.35000	-80.09000	1264890003
14080	Diptera	Culicidae	Aedes	Aedes aegypti	SPECIES	26.37009	-80.10153	3947346151
14081	Diptera	Culicidae	Aedes	Aedes aegypti	SPECIES	26.38000	-99.00000	1264888731
14082	Diptera	Culicidae	Aedes	Aedes aegypti	SPECIES	26.38058	-80.14440	4029112981
14083	Diptera	Culicidae	Aedes	Aedes aegypti	SPECIES	26.38059	-80.14443	3330677538
14084	Diptera	Culicidae	Aedes	Aedes aegypti	SPECIES	26.38061	-80.14450	2013686258
14085	Diptera	Culicidae	Aedes	Aedes aegypti	SPECIES	26.39000	-99.06000	1264887035
14086	Diptera	Culicidae	Aedes	Aedes aegypti	SPECIES	26.39000	-98.82000	1264890348
14087	Diptera	Culicidae	Aedes	Aedes aegypti	SPECIES	26.39000	-98.18000	1264890853
14088	Diptera	Culicidae	Aedes	Aedes aegypti	SPECIES	26.39022	-100.44353	1895893017
14089	Diptera	Culicidae	Aedes	Aedes aegypti	SPECIES	26.39150	-98.97894	1895892876
14090	Diptera	Culicidae	Aedes	Aedes aegypti	SPECIES	26.39556	-100.39139	1894399764
14091	Diptera	Culicidae	Aedes	Aedes aegypti	SPECIES	26.40000	-98.19000	1264871568
14092	Diptera	Culicidae	Aedes	Aedes aegypti	SPECIES	26.40000	-99.03000	1264886744
14093	Diptera	Culicidae	Aedes	Aedes aegypti	SPECIES	26.40992	-108.61292	2629264661
14094	Diptera	Culicidae	Aedes	Aedes aegypti	SPECIES	26.43000	92.04000	1264888450
14095	Diptera	Culicidae	Aedes	Aedes aegypti	SPECIES	26.43664	-82.12590	3456999738
14096	Diptera	Culicidae	Aedes	Aedes aegypti	SPECIES	26.43665	-82.12590	3333332426
14097	Diptera	Culicidae	Aedes	Aedes aegypti	SPECIES	26.43672	-82.12597	3008384717
14098	Diptera	Culicidae	Aedes	Aedes aegypti	SPECIES	26.46130	-81.78120	3030649536
14099	Diptera	Culicidae	Aedes	Aedes aegypti	SPECIES	26.47000	-97.68000	1264890839
14100	Diptera	Culicidae	Aedes	Aedes aegypti	SPECIES	26.47070	-81.82060	3030865646
14101	Diptera	Culicidae	Aedes	Aedes aegypti	SPECIES	26.47070	-82.15630	3030877768
14102	Diptera	Culicidae	Aedes	Aedes aegypti	SPECIES	26.47550	-81.82060	3030942919
14103	Diptera	Culicidae	Aedes	Aedes aegypti	SPECIES	26.47570	-81.82850	3030936721
14104	Diptera	Culicidae	Aedes	Aedes aegypti	SPECIES	26.48000	90.55000	1264888461

№	Order	Family	Genus	Species	Taxon Rank	Latitude	Longitude	GBIF_ID
14105	Diptera	Culicidae	Aedes	Aedes aegypti	SPECIES	26.48050	-81.89630	3030641260
14106	Diptera	Culicidae	Aedes	Aedes aegypti	SPECIES	26.48080	-81.86220	3030776124
14107	Diptera	Culicidae	Aedes	Aedes aegypti	SPECIES	26.48460	-82.16720	3030873546
14108	Diptera	Culicidae	Aedes	Aedes aegypti	SPECIES	26.48500	-81.82610	3030944001
14109	Diptera	Culicidae	Aedes	Aedes aegypti	SPECIES	26.48510	-81.80630	3030943712
14110	Diptera	Culicidae	Aedes	Aedes aegypti	SPECIES	26.48760	-81.93490	3030934635
14111	Diptera	Culicidae	Aedes	Aedes aegypti	SPECIES	26.49028	-100.05833	1894396871
14112	Diptera	Culicidae	Aedes	Aedes aegypti	SPECIES	26.49167	-100.07611	1894399687
14113	Diptera	Culicidae	Aedes	Aedes aegypti	SPECIES	26.49292	-100.18444	1895892808
14114	Diptera	Culicidae	Aedes	Aedes aegypti	SPECIES	26.49303	-100.23028	1421473989
14115	Diptera	Culicidae	Aedes	Aedes aegypti	SPECIES	26.49861	-99.52222	1894395769
14116	Diptera	Culicidae	Aedes	Aedes aegypti	SPECIES	26.49865	-100.19473	1895892901
14117	Diptera	Culicidae	Aedes	Aedes aegypti	SPECIES	26.50000	-100.18000	1264889782
14118	Diptera	Culicidae	Aedes	Aedes aegypti	SPECIES	26.50111	-100.42722	1894398392
14119	Diptera	Culicidae	Aedes	Aedes aegypti	SPECIES	26.50278	-100.18417	1894400266
14120	Diptera	Culicidae	Aedes	Aedes aegypti	SPECIES	26.51970	-81.89100	3030918952
14121	Diptera	Culicidae	Aedes	Aedes aegypti	SPECIES	26.53000	-78.78000	1264889950
14122	Diptera	Culicidae	Aedes	Aedes aegypti	SPECIES	26.53770	-81.97830	3030873836
14123	Diptera	Culicidae	Aedes	Aedes aegypti	SPECIES	26.54100	-81.89740	3030943580
14124	Diptera	Culicidae	Aedes	Aedes aegypti	SPECIES	26.54860	-81.85400	3030940897
14125	Diptera	Culicidae	Aedes	Aedes aegypti	SPECIES	26.55000	-81.17000	1264871579
14126	Diptera	Culicidae	Aedes	Aedes aegypti	SPECIES	26.55000	-78.82000	1264889981
14127	Diptera	Culicidae	Aedes	Aedes aegypti	SPECIES	26.56190	-81.92000	3030956047
14128	Diptera	Culicidae	Aedes	Aedes aegypti	SPECIES	26.56866	-99.22339	1895892997
14129	Diptera	Culicidae	Aedes	Aedes aegypti	SPECIES	26.57000	-98.75000	1264871571
14130	Diptera	Culicidae	Aedes	Aedes aegypti	SPECIES	26.58000	-81.80000	1264871566
14131	Diptera	Culicidae	Aedes	Aedes aegypti	SPECIES	26.58000	-78.85000	1264889997
14132	Diptera	Culicidae	Aedes	Aedes aegypti	SPECIES	26.58000	73.85000	1264890926
14133	Diptera	Culicidae	Aedes	Aedes aegypti	SPECIES	26.60000	93.02000	1264888446
14134	Diptera	Culicidae	Aedes	Aedes aegypti	SPECIES	26.61110	-81.60290	3030959720
14135	Diptera	Culicidae	Aedes	Aedes aegypti	SPECIES	26.61343	-80.16843	3902897014
14136	Diptera	Culicidae	Aedes	Aedes aegypti	SPECIES	26.61500	-81.81230	3030923370
14137	Diptera	Culicidae	Aedes	Aedes aegypti	SPECIES	26.62000	92.80000	1264888451
14138	Diptera	Culicidae	Aedes	Aedes aegypti	SPECIES	26.62000	-81.66850	3030915461
14139	Diptera	Culicidae	Aedes	Aedes aegypti	SPECIES	26.65000	-80.48000	1264871563
14140	Diptera	Culicidae	Aedes	Aedes aegypti	SPECIES	26.65000	-81.65000	1264889005
14141	Diptera	Culicidae	Aedes	Aedes aegypti	SPECIES	26.65120	-81.80790	3030737162
14142	Diptera	Culicidae	Aedes	Aedes aegypti	SPECIES	26.65330	-81.84410	3030964085
14143	Diptera	Culicidae	Aedes	Aedes aegypti	SPECIES	26.65350	-81.80740	3030943919
14144	Diptera	Culicidae	Aedes	Aedes aegypti	SPECIES	26.65590	-81.80830	3030942586
14145	Diptera	Culicidae	Aedes	Aedes aegypti	SPECIES	26.65972	-99.98694	1894395515

№	Order	Family	Genus	Species	Taxon Rank	Latitude	Longitude	GBIF_ID
14146	Diptera	Culicidae	Aedes	Aedes aegypti	SPECIES	26.66730	-81.81300	3030942952
14147	Diptera	Culicidae	Aedes	Aedes aegypti	SPECIES	26.67500	-81.86620	3030956139
14148	Diptera	Culicidae	Aedes	Aedes aegypti	SPECIES	26.67600	-81.81680	3030943982
14149	Diptera	Culicidae	Aedes	Aedes aegypti	SPECIES	26.67840	-81.89140	3030942493
14150	Diptera	Culicidae	Aedes	Aedes aegypti	SPECIES	26.68000	-78.97000	1264890005
14151	Diptera	Culicidae	Aedes	Aedes aegypti	SPECIES	26.68110	-81.81600	3030940407
14152	Diptera	Culicidae	Aedes	Aedes aegypti	SPECIES	26.71000	-80.06000	1264889163
14153	Diptera	Culicidae	Aedes	Aedes aegypti	SPECIES	26.71000	-78.16000	1264889971
14154	Diptera	Culicidae	Aedes	Aedes aegypti	SPECIES	26.71722	-108.32386	2629263774
14155	Diptera	Culicidae	Aedes	Aedes aegypti	SPECIES	26.71981	-100.53119	1895893036
14156	Diptera	Culicidae	Aedes	Aedes aegypti	SPECIES	26.72080	-81.76920	3030957057
14157	Diptera	Culicidae	Aedes	Aedes aegypti	SPECIES	26.75000	72.79000	1264890764
14158	Diptera	Culicidae	Aedes	Aedes aegypti	SPECIES	26.76000	94.21000	1264888467
14159	Diptera	Culicidae	Aedes	Aedes aegypti	SPECIES	26.77861	-99.34167	1894399088
14160	Diptera	Culicidae	Aedes	Aedes aegypti	SPECIES	26.78810	92.92860	2249985236
14161	Diptera	Culicidae	Aedes	Aedes aegypti	SPECIES	26.79000	92.93000	3350970987
14162	Diptera	Culicidae	Aedes	Aedes aegypti	SPECIES	26.83960	-80.10191	3384703405
14163	Diptera	Culicidae	Aedes	Aedes aegypti	SPECIES	26.89476	-80.06568	4028756273
14164	Diptera	Culicidae	Aedes	Aedes aegypti	SPECIES	26.90083	-101.41806	1421473513
14165	Diptera	Culicidae	Aedes	Aedes aegypti	SPECIES	26.91000	-81.89000	1264871570
14166	Diptera	Culicidae	Aedes	Aedes aegypti	SPECIES	26.91000	-99.28000	1264890309
14167	Diptera	Culicidae	Aedes	Aedes aegypti	SPECIES	26.91000	75.81000	1264890643
14168	Diptera	Culicidae	Aedes	Aedes aegypti	SPECIES	26.91000	-109.63000	1264891314
14169	Diptera	Culicidae	Aedes	Aedes aegypti	SPECIES	26.92000	-109.63000	1264890659
14170	Diptera	Culicidae	Aedes	Aedes aegypti	SPECIES	26.93000	-97.73000	1264871573
14171	Diptera	Culicidae	Aedes	Aedes aegypti	SPECIES	26.95082	-82.35089	4015343400
14172	Diptera	Culicidae	Aedes	Aedes aegypti	SPECIES	26.96000	-81.18000	1264871574
14173	Diptera	Culicidae	Aedes	Aedes aegypti	SPECIES	26.96000	75.79000	1264886351
14174	Diptera	Culicidae	Aedes	Aedes aegypti	SPECIES	26.97121	-82.14874	1249277253
14175	Diptera	Culicidae	Aedes	Aedes aegypti	SPECIES	26.98000	94.63000	1264888463
14176	Diptera	Culicidae	Aedes	Aedes aegypti	SPECIES	26.98000	75.72000	1264890774
14177	Diptera	Culicidae	Aedes	Aedes aegypti	SPECIES	26.98417	-102.06917	1421477119
14178	Diptera	Culicidae	Aedes	Aedes aegypti	SPECIES	26.99970	94.64870	2249986208
14179	Diptera	Culicidae	Aedes	Aedes aegypti	SPECIES	27.00000	94.65000	3348343151
14180	Diptera	Culicidae	Aedes	Aedes aegypti	SPECIES	27.02000	95.57000	1264888410
14181	Diptera	Culicidae	Aedes	Aedes aegypti	SPECIES	27.02000	94.16000	1264888442
14182	Diptera	Culicidae	Aedes	Aedes aegypti	SPECIES	27.02119	-100.51342	1895892924
14183	Diptera	Culicidae	Aedes	Aedes aegypti	SPECIES	27.02333	-100.50667	1894398454
14184	Diptera	Culicidae	Aedes	Aedes aegypti	SPECIES	27.04000	-98.23000	1264871572
14185	Diptera	Culicidae	Aedes	Aedes aegypti	SPECIES	27.05000	-98.71000	1264871560
14186	Diptera	Culicidae	Aedes	Aedes aegypti	SPECIES	27.05000	88.26000	1264890623

№	Order	Family	Genus	Species	Taxon Rank	Latitude	Longitude	GBIF_ID
14187	Diptera	Culicidae	Aedes	Aedes aegypti	SPECIES	27.07000	-80.45000	1264871580
14188	Diptera	Culicidae	Aedes	Aedes aegypti	SPECIES	27.14000	-80.20000	1264888993
14189	Diptera	Culicidae	Aedes	Aedes aegypti	SPECIES	27.14494	-80.21042	4145324638
14190	Diptera	Culicidae	Aedes	Aedes aegypti	SPECIES	27.14494	-80.21042	4137886060
14191	Diptera	Culicidae	Aedes	Aedes aegypti	SPECIES	27.16000	95.48000	1264888462
14192	Diptera	Culicidae	Aedes	Aedes aegypti	SPECIES	27.18000	-81.81000	1264871564
14193	Diptera	Culicidae	Aedes	Aedes aegypti	SPECIES	27.18000	-82.32000	1264871583
14194	Diptera	Culicidae	Aedes	Aedes aegypti	SPECIES	27.18000	95.33000	1264888419
14195	Diptera	Culicidae	Aedes	Aedes aegypti	SPECIES	27.19000	-80.25000	1264889990
14196	Diptera	Culicidae	Aedes	Aedes aegypti	SPECIES	27.20617	77.88740	3496614858
14197	Diptera	Culicidae	Aedes	Aedes aegypti	SPECIES	27.20933	-100.19136	1895892976
14198	Diptera	Culicidae	Aedes	Aedes aegypti	SPECIES	27.22000	-98.15000	1264890336
14199	Diptera	Culicidae	Aedes	Aedes aegypti	SPECIES	27.22783	-100.15800	1895893001
14200	Diptera	Culicidae	Aedes	Aedes aegypti	SPECIES	27.23000	78.00000	1264887329
14201	Diptera	Culicidae	Aedes	Aedes aegypti	SPECIES	27.24000	94.11000	1264888470
14202	Diptera	Culicidae	Aedes	Aedes aegypti	SPECIES	27.24028	-100.13194	1894400096
14203	Diptera	Culicidae	Aedes	Aedes aegypti	SPECIES	27.25000	77.27000	1264890763
14204	Diptera	Culicidae	Aedes	Aedes aegypti	SPECIES	27.27300	-82.11650	3893365436
14205	Diptera	Culicidae	Aedes	Aedes aegypti	SPECIES	27.27697	-82.51848	3902602771
14206	Diptera	Culicidae	Aedes	Aedes aegypti	SPECIES	27.28000	95.33000	1264888441
14207	Diptera	Culicidae	Aedes	Aedes aegypti	SPECIES	27.28000	95.68000	1264888474
14208	Diptera	Culicidae	Aedes	Aedes aegypti	SPECIES	27.29953	-82.50708	2826202554
14209	Diptera	Culicidae	Aedes	Aedes aegypti	SPECIES	27.29957	-82.50723	3112852922
14210	Diptera	Culicidae	Aedes	Aedes aegypti	SPECIES	27.29966	-82.50712	3112953341
14211	Diptera	Culicidae	Aedes	Aedes aegypti	SPECIES	27.31000	84.72000	1264891315
14212	Diptera	Culicidae	Aedes	Aedes aegypti	SPECIES	27.31926	-82.50369	3112462911
14213	Diptera	Culicidae	Aedes	Aedes aegypti	SPECIES	27.34000	-81.34000	1264871569
14214	Diptera	Culicidae	Aedes	Aedes aegypti	SPECIES	27.34710	-82.24350	3030955609
14215	Diptera	Culicidae	Aedes	Aedes aegypti	SPECIES	27.34970	-82.15760	3893360277
14216	Diptera	Culicidae	Aedes	Aedes aegypti	SPECIES	27.37000	94.89000	1264886367
14217	Diptera	Culicidae	Aedes	Aedes aegypti	SPECIES	27.37000	95.32000	1264888456
14218	Diptera	Culicidae	Aedes	Aedes aegypti	SPECIES	27.38000	-80.49000	1264871559
14219	Diptera	Culicidae	Aedes	Aedes aegypti	SPECIES	27.39000	-80.89000	1264871581
14220	Diptera	Culicidae	Aedes	Aedes aegypti	SPECIES	27.39000	95.62000	1264888472
14221	Diptera	Culicidae	Aedes	Aedes aegypti	SPECIES	27.40000	-80.30000	1264889001
14222	Diptera	Culicidae	Aedes	Aedes aegypti	SPECIES	27.40194	-99.65417	1894399212
14223	Diptera	Culicidae	Aedes	Aedes aegypti	SPECIES	27.40650	-82.50450	3893366381
14224	Diptera	Culicidae	Aedes	Aedes aegypti	SPECIES	27.40670	-82.50410	3030958935
14225	Diptera	Culicidae	Aedes	Aedes aegypti	SPECIES	27.40780	-82.30400	3030793498
14226	Diptera	Culicidae	Aedes	Aedes aegypti	SPECIES	27.40810	-82.26030	3030851814
14227	Diptera	Culicidae	Aedes	Aedes aegypti	SPECIES	27.41000	95.10000	1264886366

№	Order	Family	Genus	Species	Taxon Rank	Latitude	Longitude	GBIF_ID
14228	Diptera	Culicidae	Aedes	Aedes aegypti	SPECIES	27.41220	-82.28760	3030896872
14229	Diptera	Culicidae	Aedes	Aedes aegypti	SPECIES	27.41950	-82.42620	3030765720
14230	Diptera	Culicidae	Aedes	Aedes aegypti	SPECIES	27.41970	-82.47980	3030782991
14231	Diptera	Culicidae	Aedes	Aedes aegypti	SPECIES	27.42660	-82.52470	3030926369
14232	Diptera	Culicidae	Aedes	Aedes aegypti	SPECIES	27.42700	-82.13330	3893356947
14233	Diptera	Culicidae	Aedes	Aedes aegypti	SPECIES	27.43000	-97.73000	1264890824
14234	Diptera	Culicidae	Aedes	Aedes aegypti	SPECIES	27.43580	-82.39110	3030487683
14235	Diptera	Culicidae	Aedes	Aedes aegypti	SPECIES	27.43890	-82.43860	3030951959
14236	Diptera	Culicidae	Aedes	Aedes aegypti	SPECIES	27.43890	-82.43870	3893360055
14237	Diptera	Culicidae	Aedes	Aedes aegypti	SPECIES	27.43940	-82.54890	3030958634
14238	Diptera	Culicidae	Aedes	Aedes aegypti	SPECIES	27.44720	-82.62050	3030842126
14239	Diptera	Culicidae	Aedes	Aedes aegypti	SPECIES	27.45000	-110.10000	1264891320
14240	Diptera	Culicidae	Aedes	Aedes aegypti	SPECIES	27.46360	-82.30380	3030951856
14241	Diptera	Culicidae	Aedes	Aedes aegypti	SPECIES	27.46670	-82.68510	3893362142
14242	Diptera	Culicidae	Aedes	Aedes aegypti	SPECIES	27.46850	-82.68430	3893361110
14243	Diptera	Culicidae	Aedes	Aedes aegypti	SPECIES	27.46950	-82.52400	3893356300
14244	Diptera	Culicidae	Aedes	Aedes aegypti	SPECIES	27.47000	-82.30000	1264871561
14245	Diptera	Culicidae	Aedes	Aedes aegypti	SPECIES	27.47080	-82.16990	3030493443
14246	Diptera	Culicidae	Aedes	Aedes aegypti	SPECIES	27.47460	-82.32400	3030862474
14247	Diptera	Culicidae	Aedes	Aedes aegypti	SPECIES	27.47510	-82.55130	3893366327
14248	Diptera	Culicidae	Aedes	Aedes aegypti	SPECIES	27.47570	-82.61340	3893361283
14249	Diptera	Culicidae	Aedes	Aedes aegypti	SPECIES	27.47981	-99.54842	1895892930
14250	Diptera	Culicidae	Aedes	Aedes aegypti	SPECIES	27.47983	-99.54842	1895892938
14251	Diptera	Culicidae	Aedes	Aedes aegypti	SPECIES	27.48000	-99.55000	1264886749
14252	Diptera	Culicidae	Aedes	Aedes aegypti	SPECIES	27.48000	94.91000	1264888458
14253	Diptera	Culicidae	Aedes	Aedes aegypti	SPECIES	27.48000	95.18000	1264888494
14254	Diptera	Culicidae	Aedes	Aedes aegypti	SPECIES	27.48000	-99.51000	1264888770
14255	Diptera	Culicidae	Aedes	Aedes aegypti	SPECIES	27.48000	94.90000	3350977143
14256	Diptera	Culicidae	Aedes	Aedes aegypti	SPECIES	27.48130	-82.48040	3030956913
14257	Diptera	Culicidae	Aedes	Aedes aegypti	SPECIES	27.48460	-82.40160	3893365311
14258	Diptera	Culicidae	Aedes	Aedes aegypti	SPECIES	27.48639	-99.50806	2629263739
14259	Diptera	Culicidae	Aedes	Aedes aegypti	SPECIES	27.48890	-82.59210	3893367319
14260	Diptera	Culicidae	Aedes	Aedes aegypti	SPECIES	27.48919	-99.46089	3355811440
14261	Diptera	Culicidae	Aedes	Aedes aegypti	SPECIES	27.48940	-82.36170	3030917156
14262	Diptera	Culicidae	Aedes	Aedes aegypti	SPECIES	27.48990	-82.53380	3893355149
14263	Diptera	Culicidae	Aedes	Aedes aegypti	SPECIES	27.49000	-81.81000	1264871565
14264	Diptera	Culicidae	Aedes	Aedes aegypti	SPECIES	27.49000	95.36000	3348194763
14265	Diptera	Culicidae	Aedes	Aedes aegypti	SPECIES	27.49110	95.35770	2249595092
14266	Diptera	Culicidae	Aedes	Aedes aegypti	SPECIES	27.49528	-99.57194	1894398258
14267	Diptera	Culicidae	Aedes	Aedes aegypti	SPECIES	27.49750	-99.59278	1894394806
14268	Diptera	Culicidae	Aedes	Aedes aegypti	SPECIES	27.50000	-99.47000	1264887018

№	Order	Family	Genus	Species	Taxon Rank	Latitude	Longitude	GBIF_ID
14269	Diptera	Culicidae	Aedes	Aedes aegypti	SPECIES	27.50310	-82.65740	3030959283
14270	Diptera	Culicidae	Aedes	Aedes aegypti	SPECIES	27.51000	-99.49000	1264888975
14271	Diptera	Culicidae	Aedes	Aedes aegypti	SPECIES	27.51000	-99.51000	1264890843
14272	Diptera	Culicidae	Aedes	Aedes aegypti	SPECIES	27.51583	-99.57667	1894394128
14273	Diptera	Culicidae	Aedes	Aedes aegypti	SPECIES	27.51630	-82.53770	3030956776
14274	Diptera	Culicidae	Aedes	Aedes aegypti	SPECIES	27.52000	-82.58000	1264889008
14275	Diptera	Culicidae	Aedes	Aedes aegypti	SPECIES	27.52860	-82.58780	3893366344
14276	Diptera	Culicidae	Aedes	Aedes aegypti	SPECIES	27.52880	-82.59050	3030862116
14277	Diptera	Culicidae	Aedes	Aedes aegypti	SPECIES	27.52920	-82.58810	3030633519
14278	Diptera	Culicidae	Aedes	Aedes aegypti	SPECIES	27.53100	-82.58240	3893367423
14279	Diptera	Culicidae	Aedes	Aedes aegypti	SPECIES	27.53200	-82.62820	3030955959
14280	Diptera	Culicidae	Aedes	Aedes aegypti	SPECIES	27.53258	-82.63076	3860059514
14281	Diptera	Culicidae	Aedes	Aedes aegypti	SPECIES	27.53330	-82.56570	3893365416
14282	Diptera	Culicidae	Aedes	Aedes aegypti	SPECIES	27.53881	-80.38978	2898472295
14283	Diptera	Culicidae	Aedes	Aedes aegypti	SPECIES	27.55400	-82.54550	3893360245
14284	Diptera	Culicidae	Aedes	Aedes aegypti	SPECIES	27.56500	-82.48030	3030949703
14285	Diptera	Culicidae	Aedes	Aedes aegypti	SPECIES	27.56500	-82.42490	3030956818
14286	Diptera	Culicidae	Aedes	Aedes aegypti	SPECIES	27.56950	-82.59320	3030958890
14287	Diptera	Culicidae	Aedes	Aedes aegypti	SPECIES	27.57090	-82.13780	3030812938
14288	Diptera	Culicidae	Aedes	Aedes aegypti	SPECIES	27.57209	-99.43385	3985619085
14289	Diptera	Culicidae	Aedes	Aedes aegypti	SPECIES	27.57410	-82.50410	3030885355
14290	Diptera	Culicidae	Aedes	Aedes aegypti	SPECIES	27.57510	-82.41970	3893362360
14291	Diptera	Culicidae	Aedes	Aedes aegypti	SPECIES	27.57840	-82.48370	3030394381
14292	Diptera	Culicidae	Aedes	Aedes aegypti	SPECIES	27.59000	-80.37000	1264888767
14293	Diptera	Culicidae	Aedes	Aedes aegypti	SPECIES	27.59090	-82.54800	3893369325
14294	Diptera	Culicidae	Aedes	Aedes aegypti	SPECIES	27.59150	-82.19980	3030437225
14295	Diptera	Culicidae	Aedes	Aedes aegypti	SPECIES	27.60230	-82.53150	3030953493
14296	Diptera	Culicidae	Aedes	Aedes aegypti	SPECIES	27.60310	-82.09810	3030387718
14297	Diptera	Culicidae	Aedes	Aedes aegypti	SPECIES	27.60310	-82.53570	3030884345
14298	Diptera	Culicidae	Aedes	Aedes aegypti	SPECIES	27.60310	-82.53550	3893366453
14299	Diptera	Culicidae	Aedes	Aedes aegypti	SPECIES	27.61640	-82.53530	3030902157
14300	Diptera	Culicidae	Aedes	Aedes aegypti	SPECIES	27.62800	-82.49680	3030387842
14301	Diptera	Culicidae	Aedes	Aedes aegypti	SPECIES	27.63920	-82.41710	3030464968
14302	Diptera	Culicidae	Aedes	Aedes aegypti	SPECIES	27.64000	-80.40000	1264889770
14303	Diptera	Culicidae	Aedes	Aedes aegypti	SPECIES	27.64000	-80.38000	1264890097
14304	Diptera	Culicidae	Aedes	Aedes aegypti	SPECIES	27.64000	-80.39000	1264890195
14305	Diptera	Culicidae	Aedes	Aedes aegypti	SPECIES	27.64050	-82.55270	3030884648
14306	Diptera	Culicidae	Aedes	Aedes aegypti	SPECIES	27.65306	-99.70722	1894396628
14307	Diptera	Culicidae	Aedes	Aedes aegypti	SPECIES	27.69000	-80.62000	1264871582
14308	Diptera	Culicidae	Aedes	Aedes aegypti	SPECIES	27.71297	-97.32511	1913145713
14309	Diptera	Culicidae	Aedes	Aedes aegypti	SPECIES	27.73000	-97.64000	1264871694

№	Order	Family	Genus	Species	Taxon Rank	Latitude	Longitude	GBIF_ID
14310	Diptera	Culicidae	Aedes	Aedes aegypti	SPECIES	27.76000	-99.33000	1264890869
14311	Diptera	Culicidae	Aedes	Aedes aegypti	SPECIES	27.76609	-82.67631	1933070517
14312	Diptera	Culicidae	Aedes	Aedes aegypti	SPECIES	27.77000	-97.41000	1264888016
14313	Diptera	Culicidae	Aedes	Aedes aegypti	SPECIES	27.80000	-97.39000	1264890863
14314	Diptera	Culicidae	Aedes	Aedes aegypti	SPECIES	27.83899	-82.65324	3947617517
14315	Diptera	Culicidae	Aedes	Aedes aegypti	SPECIES	27.84833	-101.12000	1894399412
14316	Diptera	Culicidae	Aedes	Aedes aegypti	SPECIES	27.85000	-82.50000	1264889354
14317	Diptera	Culicidae	Aedes	Aedes aegypti	SPECIES	27.86106	-82.55678	3988624901
14318	Diptera	Culicidae	Aedes	Aedes aegypti	SPECIES	27.87750	-101.51444	1894400000
14319	Diptera	Culicidae	Aedes	Aedes aegypti	SPECIES	27.88000	-101.52000	1264889862
14320	Diptera	Culicidae	Aedes	Aedes aegypti	SPECIES	27.88000	-98.62000	1264890288
14321	Diptera	Culicidae	Aedes	Aedes aegypti	SPECIES	27.88692	-82.62695	3773709954
14322	Diptera	Culicidae	Aedes	Aedes aegypti	SPECIES	27.88972	-101.42333	1894400288
14323	Diptera	Culicidae	Aedes	Aedes aegypti	SPECIES	27.89000	79.91000	1264889515
14324	Diptera	Culicidae	Aedes	Aedes aegypti	SPECIES	27.91833	-110.89889	1894400033
14325	Diptera	Culicidae	Aedes	Aedes aegypti	SPECIES	27.92000	-110.90000	1264889736
14326	Diptera	Culicidae	Aedes	Aedes aegypti	SPECIES	27.93000	-82.31000	1264871562
14327	Diptera	Culicidae	Aedes	Aedes aegypti	SPECIES	27.93000	-110.91000	1264888725
14328	Diptera	Culicidae	Aedes	Aedes aegypti	SPECIES	27.93266	-82.54326	3858619589
14329	Diptera	Culicidae	Aedes	Aedes aegypti	SPECIES	27.95000	-81.70000	1264871594
14330	Diptera	Culicidae	Aedes	Aedes aegypti	SPECIES	27.95000	-82.46000	1264889014
14331	Diptera	Culicidae	Aedes	Aedes aegypti	SPECIES	27.95647	-82.59731	3947303754
14332	Diptera	Culicidae	Aedes	Aedes aegypti	SPECIES	27.96000	-82.46000	1264889002
14333	Diptera	Culicidae	Aedes	Aedes aegypti	SPECIES	27.98000	-82.46000	1264890505
14334	Diptera	Culicidae	Aedes	Aedes aegypti	SPECIES	27.99000	-82.41000	1264890497
14335	Diptera	Culicidae	Aedes	Aedes aegypti	SPECIES	28.00000	-97.51000	1264871706
14336	Diptera	Culicidae	Aedes	Aedes aegypti	SPECIES	28.00556	-82.78742	1948790083
14337	Diptera	Culicidae	Aedes	Aedes aegypti	SPECIES	28.01504	-82.46983	4133646417
14338	Diptera	Culicidae	Aedes	Aedes aegypti	SPECIES	28.01873	-82.56642	3911975468
14339	Diptera	Culicidae	Aedes	Aedes aegypti	SPECIES	28.02472	-82.49768	3860222120
14340	Diptera	Culicidae	Aedes	Aedes aegypti	SPECIES	28.03000	-97.50000	1264887962
14341	Diptera	Culicidae	Aedes	Aedes aegypti	SPECIES	28.03409	-82.50609	3743207453
14342	Diptera	Culicidae	Aedes	Aedes aegypti	SPECIES	28.03697	-81.76508	3328136496
14343	Diptera	Culicidae	Aedes	Aedes aegypti	SPECIES	28.05792	-82.42374	1946193227
14344	Diptera	Culicidae	Aedes	Aedes aegypti	SPECIES	28.06000	-81.15000	1264871591
14345	Diptera	Culicidae	Aedes	Aedes aegypti	SPECIES	28.06000	95.33000	3348303196
14346	Diptera	Culicidae	Aedes	Aedes aegypti	SPECIES	28.12973	-81.42635	4067946110
14347	Diptera	Culicidae	Aedes	Aedes aegypti	SPECIES	28.15151	-82.78336	2235473181
14348	Diptera	Culicidae	Aedes	Aedes aegypti	SPECIES	28.20944	-105.48181	4116307491
14349	Diptera	Culicidae	Aedes	Aedes aegypti	SPECIES	28.22000	-82.72000	1264890487
14350	Diptera	Culicidae	Aedes	Aedes aegypti	SPECIES	28.26320	-105.49398	4046498075

№	Order	Family	Genus	Species	Taxon Rank	Latitude	Longitude	GBIF_ID
14351	Diptera	Culicidae	Aedes	Aedes aegypti	SPECIES	28.26320	-105.49397	4054865630
14352	Diptera	Culicidae	Aedes	Aedes aegypti	SPECIES	28.26321	-105.49398	4121073275
14353	Diptera	Culicidae	Aedes	Aedes aegypti	SPECIES	28.26331	-105.49387	4054934255
14354	Diptera	Culicidae	Aedes	Aedes aegypti	SPECIES	28.27303	-105.47154	3337415138
14355	Diptera	Culicidae	Aedes	Aedes aegypti	SPECIES	28.27303	-105.47154	3337618353
14356	Diptera	Culicidae	Aedes	Aedes aegypti	SPECIES	28.27307	-105.47156	3391625407
14357	Diptera	Culicidae	Aedes	Aedes aegypti	SPECIES	28.27307	-105.47167	3327776744
14358	Diptera	Culicidae	Aedes	Aedes aegypti	SPECIES	28.27311	-105.47173	2988696503
14359	Diptera	Culicidae	Aedes	Aedes aegypti	SPECIES	28.27312	-105.47158	2883026058
14360	Diptera	Culicidae	Aedes	Aedes aegypti	SPECIES	28.27313	-105.47158	3031830966
14361	Diptera	Culicidae	Aedes	Aedes aegypti	SPECIES	28.31000	-82.39000	1264871586
14362	Diptera	Culicidae	Aedes	Aedes aegypti	SPECIES	28.32099	-80.72527	2596442424
14363	Diptera	Culicidae	Aedes	Aedes aegypti	SPECIES	28.32615	-81.31201	3330526564
14364	Diptera	Culicidae	Aedes	Aedes aegypti	SPECIES	28.36003	-82.70314	2634034294
14365	Diptera	Culicidae	Aedes	Aedes aegypti	SPECIES	28.38000	76.96000	1264891024
14366	Diptera	Culicidae	Aedes	Aedes aegypti	SPECIES	28.42000	-97.74000	1264890886
14367	Diptera	Culicidae	Aedes	Aedes aegypti	SPECIES	28.44000	-99.23000	1264890322
14368	Diptera	Culicidae	Aedes	Aedes aegypti	SPECIES	28.45000	-98.55000	1264890335
14369	Diptera	Culicidae	Aedes	Aedes aegypti	SPECIES	28.46000	77.02000	1264889934
14370	Diptera	Culicidae	Aedes	Aedes aegypti	SPECIES	28.46100	-82.63050	3030861748
14371	Diptera	Culicidae	Aedes	Aedes aegypti	SPECIES	28.49000	77.61000	1264887884
14372	Diptera	Culicidae	Aedes	Aedes aegypti	SPECIES	28.49270	-82.55500	3030858213
14373	Diptera	Culicidae	Aedes	Aedes aegypti	SPECIES	28.51000	-81.32000	1264871620
14374	Diptera	Culicidae	Aedes	Aedes aegypti	SPECIES	28.51000	77.22000	1264886072
14375	Diptera	Culicidae	Aedes	Aedes aegypti	SPECIES	28.52000	-99.86000	1264890346
14376	Diptera	Culicidae	Aedes	Aedes aegypti	SPECIES	28.52840	-82.28920	3030852979
14377	Diptera	Culicidae	Aedes	Aedes aegypti	SPECIES	28.53430	-82.61780	3030779923
14378	Diptera	Culicidae	Aedes	Aedes aegypti	SPECIES	28.55000	77.28000	1264886389
14379	Diptera	Culicidae	Aedes	Aedes aegypti	SPECIES	28.55000	77.18000	1264888005
14380	Diptera	Culicidae	Aedes	Aedes aegypti	SPECIES	28.55000	-81.33000	1264888502
14381	Diptera	Culicidae	Aedes	Aedes aegypti	SPECIES	28.55000	77.25000	1264889412
14382	Diptera	Culicidae	Aedes	Aedes aegypti	SPECIES	28.55000	77.17000	1264889417
14383	Diptera	Culicidae	Aedes	Aedes aegypti	SPECIES	28.55000	-81.77000	1264889994
14384	Diptera	Culicidae	Aedes	Aedes aegypti	SPECIES	28.56000	-81.37000	1264888965
14385	Diptera	Culicidae	Aedes	Aedes aegypti	SPECIES	28.57000	-81.37000	1264887935
14386	Diptera	Culicidae	Aedes	Aedes aegypti	SPECIES	28.57000	77.10000	1264890647
14387	Diptera	Culicidae	Aedes	Aedes aegypti	SPECIES	28.58000	77.17000	1264887890
14388	Diptera	Culicidae	Aedes	Aedes aegypti	SPECIES	28.58000	77.22000	1264888003
14389	Diptera	Culicidae	Aedes	Aedes aegypti	SPECIES	28.58387	-81.35660	3383973844
14390	Diptera	Culicidae	Aedes	Aedes aegypti	SPECIES	28.59000	-81.35000	1264889968
14391	Diptera	Culicidae	Aedes	Aedes aegypti	SPECIES	28.59000	77.22000	1264890644

№	Order	Family	Genus	Species	Taxon Rank	Latitude	Longitude	GBIF_ID
14392	Diptera	Culicidae	Aedes	Aedes aegypti	SPECIES	28.59355	-81.34799	3384009038
14393	Diptera	Culicidae	Aedes	Aedes aegypti	SPECIES	28.60000	77.16000	1264886091
14394	Diptera	Culicidae	Aedes	Aedes aegypti	SPECIES	28.61000	76.98000	1264891026
14395	Diptera	Culicidae	Aedes	Aedes aegypti	SPECIES	28.61399	-106.12106	1948728464
14396	Diptera	Culicidae	Aedes	Aedes aegypti	SPECIES	28.61438	-106.07298	3383927288
14397	Diptera	Culicidae	Aedes	Aedes aegypti	SPECIES	28.62000	77.30000	1264886110
14398	Diptera	Culicidae	Aedes	Aedes aegypti	SPECIES	28.63300	-106.06910	3355130932
14399	Diptera	Culicidae	Aedes	Aedes aegypti	SPECIES	28.64000	77.21000	1264886103
14400	Diptera	Culicidae	Aedes	Aedes aegypti	SPECIES	28.64654	-106.09632	2576248005
14401	Diptera	Culicidae	Aedes	Aedes aegypti	SPECIES	28.65000	77.19000	1264891005
14402	Diptera	Culicidae	Aedes	Aedes aegypti	SPECIES	28.65000	77.11000	1264891055
14403	Diptera	Culicidae	Aedes	Aedes aegypti	SPECIES	28.66000	77.20000	1264886088
14404	Diptera	Culicidae	Aedes	Aedes aegypti	SPECIES	28.66000	77.21000	1264888013
14405	Diptera	Culicidae	Aedes	Aedes aegypti	SPECIES	28.66000	77.23000	1264888017
14406	Diptera	Culicidae	Aedes	Aedes aegypti	SPECIES	28.67000	77.07000	1264886112
14407	Diptera	Culicidae	Aedes	Aedes aegypti	SPECIES	28.67000	77.18000	1264886372
14408	Diptera	Culicidae	Aedes	Aedes aegypti	SPECIES	28.67000	-80.66000	1264889309
14409	Diptera	Culicidae	Aedes	Aedes aegypti	SPECIES	28.67000	77.28000	1264891035
14410	Diptera	Culicidae	Aedes	Aedes aegypti	SPECIES	28.68000	77.21000	1264886059
14411	Diptera	Culicidae	Aedes	Aedes aegypti	SPECIES	28.68000	77.22000	1264886081
14412	Diptera	Culicidae	Aedes	Aedes aegypti	SPECIES	28.70000	77.10000	1264886055
14413	Diptera	Culicidae	Aedes	Aedes aegypti	SPECIES	28.70000	77.22000	1264886098
14414	Diptera	Culicidae	Aedes	Aedes aegypti	SPECIES	28.70000	-100.52306	1894399776
14415	Diptera	Culicidae	Aedes	Aedes aegypti	SPECIES	28.71000	-100.52000	1264890342
14416	Diptera	Culicidae	Aedes	Aedes aegypti	SPECIES	28.72000	77.17000	1264886082
14417	Diptera	Culicidae	Aedes	Aedes aegypti	SPECIES	28.74000	-100.31000	1264890858
14418	Diptera	Culicidae	Aedes	Aedes aegypti	SPECIES	28.75000	77.21000	1264886106
14419	Diptera	Culicidae	Aedes	Aedes aegypti	SPECIES	28.79000	-81.28000	1264890013
14420	Diptera	Culicidae	Aedes	Aedes aegypti	SPECIES	28.81000	-81.88000	1264889959
14421	Diptera	Culicidae	Aedes	Aedes aegypti	SPECIES	28.84984	-105.93639	1914241085
14422	Diptera	Culicidae	Aedes	Aedes aegypti	SPECIES	28.88000	-99.10000	1264890330
14423	Diptera	Culicidae	Aedes	Aedes aegypti	SPECIES	28.99000	77.01000	1264889806
14424	Diptera	Culicidae	Aedes	Aedes aegypti	SPECIES	28.99000	77.02000	1264890024
14425	Diptera	Culicidae	Aedes	Aedes aegypti	SPECIES	29.03000	-81.31000	1264890007
14426	Diptera	Culicidae	Aedes	Aedes aegypti	SPECIES	29.03535	-80.89767	3328118489
14427	Diptera	Culicidae	Aedes	Aedes aegypti	SPECIES	29.06000	-81.19000	1264871611
14428	Diptera	Culicidae	Aedes	Aedes aegypti	SPECIES	29.06000	-82.46000	1264890000
14429	Diptera	Culicidae	Aedes	Aedes aegypti	SPECIES	29.07000	-110.96000	1264886760
14430	Diptera	Culicidae	Aedes	Aedes aegypti	SPECIES	29.08000	79.52000	1264886289
14431	Diptera	Culicidae	Aedes	Aedes aegypti	SPECIES	29.09000	-110.95000	1264888054
14432	Diptera	Culicidae	Aedes	Aedes aegypti	SPECIES	29.09889	-110.95417	2629262723

№	Order	Family	Genus	Species	Taxon Rank	Latitude	Longitude	GBIF_ID
14433	Diptera	Culicidae	Aedes	Aedes aegypti	SPECIES	29.10000	-110.95000	1264888772
14434	Diptera	Culicidae	Aedes	Aedes aegypti	SPECIES	29.14000	79.60000	1264887395
14435	Diptera	Culicidae	Aedes	Aedes aegypti	SPECIES	29.15000	76.33000	1264890739
14436	Diptera	Culicidae	Aedes	Aedes aegypti	SPECIES	29.17391	-110.85784	2237435852
14437	Diptera	Culicidae	Aedes	Aedes aegypti	SPECIES	29.19000	-95.46000	1264871685
14438	Diptera	Culicidae	Aedes	Aedes aegypti	SPECIES	29.19000	-82.13000	1264889822
14439	Diptera	Culicidae	Aedes	Aedes aegypti	SPECIES	29.19000	-82.14000	1264889976
14440	Diptera	Culicidae	Aedes	Aedes aegypti	SPECIES	29.20000	-100.95000	1264889732
14441	Diptera	Culicidae	Aedes	Aedes aegypti	SPECIES	29.21000	-82.15000	1264889923
14442	Diptera	Culicidae	Aedes	Aedes aegypti	SPECIES	29.21000	-81.03000	1264890008
14443	Diptera	Culicidae	Aedes	Aedes aegypti	SPECIES	29.21000	-99.79000	1264890324
14444	Diptera	Culicidae	Aedes	Aedes aegypti	SPECIES	29.23000	79.53000	1264887411
14445	Diptera	Culicidae	Aedes	Aedes aegypti	SPECIES	29.26000	-81.04000	1264889546
14446	Diptera	Culicidae	Aedes	Aedes aegypti	SPECIES	29.26000	-94.87000	1264890111
14447	Diptera	Culicidae	Aedes	Aedes aegypti	SPECIES	29.28000	-94.82000	2805283396
14448	Diptera	Culicidae	Aedes	Aedes aegypti	SPECIES	29.29000	-94.80000	1264889383
14449	Diptera	Culicidae	Aedes	Aedes aegypti	SPECIES	29.30000	-94.81000	1264890508
14450	Diptera	Culicidae	Aedes	Aedes aegypti	SPECIES	29.33000	-82.74000	1264871623
14451	Diptera	Culicidae	Aedes	Aedes aegypti	SPECIES	29.34684	-98.43265	3049138811
14452	Diptera	Culicidae	Aedes	Aedes aegypti	SPECIES	29.35000	-98.44000	1264889362
14453	Diptera	Culicidae	Aedes	Aedes aegypti	SPECIES	29.38000	-98.58000	1264889343
14454	Diptera	Culicidae	Aedes	Aedes aegypti	SPECIES	29.38044	-98.58459	3048748853
14455	Diptera	Culicidae	Aedes	Aedes aegypti	SPECIES	29.38646	-98.62783	3049105809
14456	Diptera	Culicidae	Aedes	Aedes aegypti	SPECIES	29.38656	-98.68401	3407911709
14457	Diptera	Culicidae	Aedes	Aedes aegypti	SPECIES	29.39000	-98.62000	1264889353
14458	Diptera	Culicidae	Aedes	Aedes aegypti	SPECIES	29.40878	-98.49069	3963512017
14459	Diptera	Culicidae	Aedes	Aedes aegypti	SPECIES	29.40884	-98.49102	3456597569
14460	Diptera	Culicidae	Aedes	Aedes aegypti	SPECIES	29.40895	-98.49065	3466453416
14461	Diptera	Culicidae	Aedes	Aedes aegypti	SPECIES	29.41000	-94.96000	1264871723
14462	Diptera	Culicidae	Aedes	Aedes aegypti	SPECIES	29.41000	-82.12000	1264888978
14463	Diptera	Culicidae	Aedes	Aedes aegypti	SPECIES	29.42000	-98.50000	1264888466
14464	Diptera	Culicidae	Aedes	Aedes aegypti	SPECIES	29.42412	-98.49363	3031898885
14465	Diptera	Culicidae	Aedes	Aedes aegypti	SPECIES	29.43000	-98.50000	1264889360
14466	Diptera	Culicidae	Aedes	Aedes aegypti	SPECIES	29.45000	-82.64000	1264888969
14467	Diptera	Culicidae	Aedes	Aedes aegypti	SPECIES	29.45000	-98.52000	1264890857
14468	Diptera	Culicidae	Aedes	Aedes aegypti	SPECIES	29.45027	-98.51161	3005159555
14469	Diptera	Culicidae	Aedes	Aedes aegypti	SPECIES	29.45895	-98.45789	3391356164
14470	Diptera	Culicidae	Aedes	Aedes aegypti	SPECIES	29.46000	-81.31000	1264871593
14471	Diptera	Culicidae	Aedes	Aedes aegypti	SPECIES	29.46069	-98.50481	3112849502
14472	Diptera	Culicidae	Aedes	Aedes aegypti	SPECIES	29.46435	-98.48375	4091581798
14473	Diptera	Culicidae	Aedes	Aedes aegypti	SPECIES	29.46505	-98.47646	3356899310

№	Order	Family	Genus	Species	Taxon Rank	Latitude	Longitude	GBIF_ID
14474	Diptera	Culicidae	Aedes	Aedes aegypti	SPECIES	29.46536	-98.43269	2466128293
14475	Diptera	Culicidae	Aedes	Aedes aegypti	SPECIES	29.47000	-98.48000	1264889312
14476	Diptera	Culicidae	Aedes	Aedes aegypti	SPECIES	29.47554	-98.51766	2466133963
14477	Diptera	Culicidae	Aedes	Aedes aegypti	SPECIES	29.50427	-98.51124	1914353586
14478	Diptera	Culicidae	Aedes	Aedes aegypti	SPECIES	29.50473	-98.34368	3399241083
14479	Diptera	Culicidae	Aedes	Aedes aegypti	SPECIES	29.52945	-98.54139	2265865903
14480	Diptera	Culicidae	Aedes	Aedes aegypti	SPECIES	29.53000	-95.78000	1264871717
14481	Diptera	Culicidae	Aedes	Aedes aegypti	SPECIES	29.53000	-98.28000	1264889351
14482	Diptera	Culicidae	Aedes	Aedes aegypti	SPECIES	29.53451	-98.54646	1914227935
14483	Diptera	Culicidae	Aedes	Aedes aegypti	SPECIES	29.57000	-97.97000	1264887976
14484	Diptera	Culicidae	Aedes	Aedes aegypti	SPECIES	29.57414	-98.58049	2859951208
14485	Diptera	Culicidae	Aedes	Aedes aegypti	SPECIES	29.58138	-98.37206	2626122817
14486	Diptera	Culicidae	Aedes	Aedes aegypti	SPECIES	29.58273	-98.61891	3385166639
14487	Diptera	Culicidae	Aedes	Aedes aegypti	SPECIES	29.58324	-98.61858	2397631670
14488	Diptera	Culicidae	Aedes	Aedes aegypti	SPECIES	29.58700	-98.20900	3970354755
14489	Diptera	Culicidae	Aedes	Aedes aegypti	SPECIES	29.59471	-98.59051	2460089550
14490	Diptera	Culicidae	Aedes	Aedes aegypti	SPECIES	29.60833	-95.53468	2988680479
14491	Diptera	Culicidae	Aedes	Aedes aegypti	SPECIES	29.61430	-98.49119	2850990066
14492	Diptera	Culicidae	Aedes	Aedes aegypti	SPECIES	29.62616	-98.22216	4103104975
14493	Diptera	Culicidae	Aedes	Aedes aegypti	SPECIES	29.62619	-98.22225	3391640192
14494	Diptera	Culicidae	Aedes	Aedes aegypti	SPECIES	29.62620	-98.22217	4145785207
14495	Diptera	Culicidae	Aedes	Aedes aegypti	SPECIES	29.62633	-98.22201	3384901244
14496	Diptera	Culicidae	Aedes	Aedes aegypti	SPECIES	29.64000	-81.66000	1264889567
14497	Diptera	Culicidae	Aedes	Aedes aegypti	SPECIES	29.65000	-82.33000	1264889913
14498	Diptera	Culicidae	Aedes	Aedes aegypti	SPECIES	29.65000	-81.65000	1264890011
14499	Diptera	Culicidae	Aedes	Aedes aegypti	SPECIES	29.65163	-82.32483	3356898310
14500	Diptera	Culicidae	Aedes	Aedes aegypti	SPECIES	29.66000	-82.34000	1264889554
14501	Diptera	Culicidae	Aedes	Aedes aegypti	SPECIES	29.67000	-82.36000	1264889933
14502	Diptera	Culicidae	Aedes	Aedes aegypti	SPECIES	29.69000	-95.34000	1264889408
14503	Diptera	Culicidae	Aedes	Aedes aegypti	SPECIES	29.69971	-95.45197	3398831571
14504	Diptera	Culicidae	Aedes	Aedes aegypti	SPECIES	29.70000	-95.34000	1264889344
14505	Diptera	Culicidae	Aedes	Aedes aegypti	SPECIES	29.70000	-95.37000	1264889350
14506	Diptera	Culicidae	Aedes	Aedes aegypti	SPECIES	29.70000	-98.11000	1264890329
14507	Diptera	Culicidae	Aedes	Aedes aegypti	SPECIES	29.70008	-98.48731	2429190105
14508	Diptera	Culicidae	Aedes	Aedes aegypti	SPECIES	29.70300	-98.12445	3356883311
14509	Diptera	Culicidae	Aedes	Aedes aegypti	SPECIES	29.71000	-95.32000	1264889369
14510	Diptera	Culicidae	Aedes	Aedes aegypti	SPECIES	29.71000	-95.37000	1264889386
14511	Diptera	Culicidae	Aedes	Aedes aegypti	SPECIES	29.72955	-95.40966	3902581371
14512	Diptera	Culicidae	Aedes	Aedes aegypti	SPECIES	29.73000	-95.37000	1264889385
14513	Diptera	Culicidae	Aedes	Aedes aegypti	SPECIES	29.73000	-94.97000	1264890315
14514	Diptera	Culicidae	Aedes	Aedes aegypti	SPECIES	29.73045	-95.29447	2859913020

№	Order	Family	Genus	Species	Taxon Rank	Latitude	Longitude	GBIF_ID
14515	Diptera	Culicidae	Aedes	Aedes aegypti	SPECIES	29.74000	-95.36000	1264889357
14516	Diptera	Culicidae	Aedes	Aedes aegypti	SPECIES	29.75000	-95.35000	1264890667
14517	Diptera	Culicidae	Aedes	Aedes aegypti	SPECIES	29.76000	-95.37000	1264886764
14518	Diptera	Culicidae	Aedes	Aedes aegypti	SPECIES	29.76000	-95.36000	1264890147
14519	Diptera	Culicidae	Aedes	Aedes aegypti	SPECIES	29.76043	-95.36980	4129908544
14520	Diptera	Culicidae	Aedes	Aedes aegypti	SPECIES	29.76046	-95.45360	2634302749
14521	Diptera	Culicidae	Aedes	Aedes aegypti	SPECIES	29.77000	-95.33000	1264889382
14522	Diptera	Culicidae	Aedes	Aedes aegypti	SPECIES	29.77984	-95.36121	2860163217
14523	Diptera	Culicidae	Aedes	Aedes aegypti	SPECIES	29.78000	-90.12000	1264871614
14524	Diptera	Culicidae	Aedes	Aedes aegypti	SPECIES	29.78000	-95.30000	1264889388
14525	Diptera	Culicidae	Aedes	Aedes aegypti	SPECIES	29.79447	-98.42792	3455423574
14526	Diptera	Culicidae	Aedes	Aedes aegypti	SPECIES	29.79532	-95.38533	3399120697
14527	Diptera	Culicidae	Aedes	Aedes aegypti	SPECIES	29.80000	-95.52000	1264889392
14528	Diptera	Culicidae	Aedes	Aedes aegypti	SPECIES	29.82000	-95.54000	1264889368
14529	Diptera	Culicidae	Aedes	Aedes aegypti	SPECIES	29.83000	-95.37000	1264889398
14530	Diptera	Culicidae	Aedes	Aedes aegypti	SPECIES	29.83000	-95.40000	1264890334
14531	Diptera	Culicidae	Aedes	Aedes aegypti	SPECIES	29.86000	-92.32000	1264871584
14532	Diptera	Culicidae	Aedes	Aedes aegypti	SPECIES	29.86000	-95.40000	1264871684
14533	Diptera	Culicidae	Aedes	Aedes aegypti	SPECIES	29.88000	-94.18000	1264871693
14534	Diptera	Culicidae	Aedes	Aedes aegypti	SPECIES	29.88000	-97.95000	1264888960
14535	Diptera	Culicidae	Aedes	Aedes aegypti	SPECIES	29.89000	-96.28000	1264890890
14536	Diptera	Culicidae	Aedes	Aedes aegypti	SPECIES	29.90000	-81.34000	1264889572
14537	Diptera	Culicidae	Aedes	Aedes aegypti	SPECIES	29.90000	-81.32000	1264890041
14538	Diptera	Culicidae	Aedes	Aedes aegypti	SPECIES	29.91738	-90.10944	3860198606
14539	Diptera	Culicidae	Aedes	Aedes aegypti	SPECIES	29.91774	-90.10949	3773590981
14540	Diptera	Culicidae	Aedes	Aedes aegypti	SPECIES	29.91781	-90.10953	2242969721
14541	Diptera	Culicidae	Aedes	Aedes aegypti	SPECIES	29.91781	-90.10977	3337984934
14542	Diptera	Culicidae	Aedes	Aedes aegypti	SPECIES	29.91782	-90.10973	3384771906
14543	Diptera	Culicidae	Aedes	Aedes aegypti	SPECIES	29.91785	-90.10953	3802374021
14544	Diptera	Culicidae	Aedes	Aedes aegypti	SPECIES	29.91786	-90.10946	2826153774
14545	Diptera	Culicidae	Aedes	Aedes aegypti	SPECIES	29.91789	-90.10963	3335078104
14546	Diptera	Culicidae	Aedes	Aedes aegypti	SPECIES	29.91789	-90.10955	3858614082
14547	Diptera	Culicidae	Aedes	Aedes aegypti	SPECIES	29.91789	-90.10942	3330611511
14548	Diptera	Culicidae	Aedes	Aedes aegypti	SPECIES	29.91790	-90.10973	3384118021
14549	Diptera	Culicidae	Aedes	Aedes aegypti	SPECIES	29.91791	-90.10972	3327885457
14550	Diptera	Culicidae	Aedes	Aedes aegypti	SPECIES	29.91791	-90.10938	1913148567
14551	Diptera	Culicidae	Aedes	Aedes aegypti	SPECIES	29.91791	-90.10923	3355413850
14552	Diptera	Culicidae	Aedes	Aedes aegypti	SPECIES	29.91793	-90.10966	3874106145
14553	Diptera	Culicidae	Aedes	Aedes aegypti	SPECIES	29.91794	-90.10957	2883161976
14554	Diptera	Culicidae	Aedes	Aedes aegypti	SPECIES	29.91795	-90.10934	2273269792
14555	Diptera	Culicidae	Aedes	Aedes aegypti	SPECIES	29.91796	-90.10966	4133809024

№	Order	Family	Genus	Species	Taxon Rank	Latitude	Longitude	GBIF_ID
14556	Diptera	Culicidae	Aedes	Aedes aegypti	SPECIES	29.91803	-90.10941	2803106567
14557	Diptera	Culicidae	Aedes	Aedes aegypti	SPECIES	29.91811	-90.10958	3383862049
14558	Diptera	Culicidae	Aedes	Aedes aegypti	SPECIES	29.92133	-90.12217	4080855336
14559	Diptera	Culicidae	Aedes	Aedes aegypti	SPECIES	29.93000	-90.08000	1264890108
14560	Diptera	Culicidae	Aedes	Aedes aegypti	SPECIES	29.94000	-90.09000	1264887853
14561	Diptera	Culicidae	Aedes	Aedes aegypti	SPECIES	29.94000	-90.05000	1264890081
14562	Diptera	Culicidae	Aedes	Aedes aegypti	SPECIES	29.94287	-89.96352	3802824014
14563	Diptera	Culicidae	Aedes	Aedes aegypti	SPECIES	29.95000	-90.05000	1264887987
14564	Diptera	Culicidae	Aedes	Aedes aegypti	SPECIES	29.95000	-90.14000	1264888014
14565	Diptera	Culicidae	Aedes	Aedes aegypti	SPECIES	29.95000	-90.12000	1264888599
14566	Diptera	Culicidae	Aedes	Aedes aegypti	SPECIES	29.95000	-82.11000	1264890014
14567	Diptera	Culicidae	Aedes	Aedes aegypti	SPECIES	29.95814	-90.06485	2466125745
14568	Diptera	Culicidae	Aedes	Aedes aegypti	SPECIES	29.96000	-90.08000	1264888321
14569	Diptera	Culicidae	Aedes	Aedes aegypti	SPECIES	29.96000	-90.01000	1264888622
14570	Diptera	Culicidae	Aedes	Aedes aegypti	SPECIES	29.96000	-90.06000	1264890093
14571	Diptera	Culicidae	Aedes	Aedes aegypti	SPECIES	29.97000	-90.10000	1264887835
14572	Diptera	Culicidae	Aedes	Aedes aegypti	SPECIES	29.97000	-90.00000	1264887851
14573	Diptera	Culicidae	Aedes	Aedes aegypti	SPECIES	29.97000	78.17000	1264888374
14574	Diptera	Culicidae	Aedes	Aedes aegypti	SPECIES	29.97000	-90.09000	1264888577
14575	Diptera	Culicidae	Aedes	Aedes aegypti	SPECIES	29.97000	-90.02000	1264890056
14576	Diptera	Culicidae	Aedes	Aedes aegypti	SPECIES	29.97000	-92.13000	1264890103
14577	Diptera	Culicidae	Aedes	Aedes aegypti	SPECIES	29.97000	-90.07000	1264890688
14578	Diptera	Culicidae	Aedes	Aedes aegypti	SPECIES	29.97090	-90.07104	1986557125
14579	Diptera	Culicidae	Aedes	Aedes aegypti	SPECIES	29.98000	-90.07000	1264888604
14580	Diptera	Culicidae	Aedes	Aedes aegypti	SPECIES	29.98000	-90.05000	1264890112
14581	Diptera	Culicidae	Aedes	Aedes aegypti	SPECIES	29.98702	-95.33462	3994257696
14582	Diptera	Culicidae	Aedes	Aedes aegypti	SPECIES	29.99000	-85.47000	1264889322
14583	Diptera	Culicidae	Aedes	Aedes aegypti	SPECIES	30.00000	-90.09000	1264888012
14584	Diptera	Culicidae	Aedes	Aedes aegypti	SPECIES	30.00000	-90.10000	1264888593
14585	Diptera	Culicidae	Aedes	Aedes aegypti	SPECIES	30.00000	-81.69000	1264889970
14586	Diptera	Culicidae	Aedes	Aedes aegypti	SPECIES	30.00000	-90.05000	1264890211
14587	Diptera	Culicidae	Aedes	Aedes aegypti	SPECIES	30.01000	-90.02000	1264888039
14588	Diptera	Culicidae	Aedes	Aedes aegypti	SPECIES	30.05139	-111.02417	1894398982
14589	Diptera	Culicidae	Aedes	Aedes aegypti	SPECIES	30.08000	-94.10000	1264890102
14590	Diptera	Culicidae	Aedes	Aedes aegypti	SPECIES	30.10364	-98.42592	1668884949
14591	Diptera	Culicidae	Aedes	Aedes aegypti	SPECIES	30.10389	-98.42556	1948738963
14592	Diptera	Culicidae	Aedes	Aedes aegypti	SPECIES	30.13000	-93.90000	1264871697
14593	Diptera	Culicidae	Aedes	Aedes aegypti	SPECIES	30.15973	-97.84598	2980827287
14594	Diptera	Culicidae	Aedes	Aedes aegypti	SPECIES	30.20000	71.47000	1264887317
14595	Diptera	Culicidae	Aedes	Aedes aegypti	SPECIES	30.20399	-98.08012	3124571626
14596	Diptera	Culicidae	Aedes	Aedes aegypti	SPECIES	30.20509	-97.77710	3330532756

№	Order	Family	Genus	Species	Taxon Rank	Latitude	Longitude	GBIF_ID
14597	Diptera	Culicidae	Aedes	Aedes aegypti	SPECIES	30.21000	-92.02000	1264890339
14598	Diptera	Culicidae	Aedes	Aedes aegypti	SPECIES	30.21000	71.51000	3347280141
14599	Diptera	Culicidae	Aedes	Aedes aegypti	SPECIES	30.21710	-97.86157	2429308350
14600	Diptera	Culicidae	Aedes	Aedes aegypti	SPECIES	30.22100	-81.45546	4091384396
14601	Diptera	Culicidae	Aedes	Aedes aegypti	SPECIES	30.24504	-97.82702	1932355337
14602	Diptera	Culicidae	Aedes	Aedes aegypti	SPECIES	30.26000	-97.74000	1264890126
14603	Diptera	Culicidae	Aedes	Aedes aegypti	SPECIES	30.26923	-97.52132	2609286310
14604	Diptera	Culicidae	Aedes	Aedes aegypti	SPECIES	30.27631	-97.81504	3966637783
14605	Diptera	Culicidae	Aedes	Aedes aegypti	SPECIES	30.27790	-97.70406	2626644589
14606	Diptera	Culicidae	Aedes	Aedes aegypti	SPECIES	30.27799	-97.70408	2818697859
14607	Diptera	Culicidae	Aedes	Aedes aegypti	SPECIES	30.27799	-97.70409	1945441627
14608	Diptera	Culicidae	Aedes	Aedes aegypti	SPECIES	30.27801	-97.70409	3355178832
14609	Diptera	Culicidae	Aedes	Aedes aegypti	SPECIES	30.28370	-97.75410	2995029143
14610	Diptera	Culicidae	Aedes	Aedes aegypti	SPECIES	30.29000	-81.40000	1264890029
14611	Diptera	Culicidae	Aedes	Aedes aegypti	SPECIES	30.30000	-95.50000	1264871710
14612	Diptera	Culicidae	Aedes	Aedes aegypti	SPECIES	30.30000	-97.75000	2805285364
14613	Diptera	Culicidae	Aedes	Aedes aegypti	SPECIES	30.31864	-97.71934	2634243394
14614	Diptera	Culicidae	Aedes	Aedes aegypti	SPECIES	30.32075	-97.68823	1913200524
14615	Diptera	Culicidae	Aedes	Aedes aegypti	SPECIES	30.32800	-97.75400	2805286544
14616	Diptera	Culicidae	Aedes	Aedes aegypti	SPECIES	30.32813	-97.75510	1913143383
14617	Diptera	Culicidae	Aedes	Aedes aegypti	SPECIES	30.32968	-97.75408	1913142687
14618	Diptera	Culicidae	Aedes	Aedes aegypti	SPECIES	30.33000	-82.28000	1264871615
14619	Diptera	Culicidae	Aedes	Aedes aegypti	SPECIES	30.33000	-81.65000	1264890350
14620	Diptera	Culicidae	Aedes	Aedes aegypti	SPECIES	30.34000	-97.79000	1264871682
14621	Diptera	Culicidae	Aedes	Aedes aegypti	SPECIES	30.34000	-81.71000	1264887943
14622	Diptera	Culicidae	Aedes	Aedes aegypti	SPECIES	30.34000	-95.09000	1264888045
14623	Diptera	Culicidae	Aedes	Aedes aegypti	SPECIES	30.35000	-81.69000	1264887947
14624	Diptera	Culicidae	Aedes	Aedes aegypti	SPECIES	30.35000	-81.76000	1264887955
14625	Diptera	Culicidae	Aedes	Aedes aegypti	SPECIES	30.35223	-97.69411	2366160094
14626	Diptera	Culicidae	Aedes	Aedes aegypti	SPECIES	30.35273	-97.69306	1703213885
14627	Diptera	Culicidae	Aedes	Aedes aegypti	SPECIES	30.35750	-97.72340	3925164236
14628	Diptera	Culicidae	Aedes	Aedes aegypti	SPECIES	30.37000	-97.72000	2805288584
14629	Diptera	Culicidae	Aedes	Aedes aegypti	SPECIES	30.37933	-97.71502	2818547580
14630	Diptera	Culicidae	Aedes	Aedes aegypti	SPECIES	30.40300	-97.92850	3910014308
14631	Diptera	Culicidae	Aedes	Aedes aegypti	SPECIES	30.40658	-97.75225	2984525601
14632	Diptera	Culicidae	Aedes	Aedes aegypti	SPECIES	30.41220	-97.74949	2451744611
14633	Diptera	Culicidae	Aedes	Aedes aegypti	SPECIES	30.42000	-87.23000	1264888805
14634	Diptera	Culicidae	Aedes	Aedes aegypti	SPECIES	30.42162	-97.63257	2862337681
14635	Diptera	Culicidae	Aedes	Aedes aegypti	SPECIES	30.42501	-97.63755	2826302732
14636	Diptera	Culicidae	Aedes	Aedes aegypti	SPECIES	30.43826	-84.28073	3356901313
14637	Diptera	Culicidae	Aedes	Aedes aegypti	SPECIES	30.44000	-93.43000	1264889521

№	Order	Family	Genus	Species	Taxon Rank	Latitude	Longitude	GBIF_ID
14638	Diptera	Culicidae	Aedes	Aedes aegypti	SPECIES	30.45454	-97.79899	2803120493
14639	Diptera	Culicidae	Aedes	Aedes aegypti	SPECIES	30.46000	-91.15000	1264890373
14640	Diptera	Culicidae	Aedes	Aedes aegypti	SPECIES	30.47695	-97.91331	4127113171
14641	Diptera	Culicidae	Aedes	Aedes aegypti	SPECIES	30.48000	-90.11000	1264888814
14642	Diptera	Culicidae	Aedes	Aedes aegypti	SPECIES	30.50826	-97.67890	3456164403
14643	Diptera	Culicidae	Aedes	Aedes aegypti	SPECIES	30.59495	-96.29319	3048748843
14644	Diptera	Culicidae	Aedes	Aedes aegypti	SPECIES	30.62947	-96.33769	3049136795
14645	Diptera	Culicidae	Aedes	Aedes aegypti	SPECIES	30.63000	-87.05000	1264888803
14646	Diptera	Culicidae	Aedes	Aedes aegypti	SPECIES	30.67000	-96.30000	1264871686
14647	Diptera	Culicidae	Aedes	Aedes aegypti	SPECIES	30.68000	-81.45000	1264890036
14648	Diptera	Culicidae	Aedes	Aedes aegypti	SPECIES	30.69000	-88.05000	1264888970
14649	Diptera	Culicidae	Aedes	Aedes aegypti	SPECIES	30.73000	76.77000	1264890899
14650	Diptera	Culicidae	Aedes	Aedes aegypti	SPECIES	30.77806	-110.85639	1894396674
14651	Diptera	Culicidae	Aedes	Aedes aegypti	SPECIES	30.81000	75.82000	1264891066
14652	Diptera	Culicidae	Aedes	Aedes aegypti	SPECIES	30.81000	73.45000	3350231535
14653	Diptera	Culicidae	Aedes	Aedes aegypti	SPECIES	30.81300	73.44600	2250855285
14654	Diptera	Culicidae	Aedes	Aedes aegypti	SPECIES	30.85000	-96.98000	1264888822
14655	Diptera	Culicidae	Aedes	Aedes aegypti	SPECIES	31.00000	73.97000	3350214297
14656	Diptera	Culicidae	Aedes	Aedes aegypti	SPECIES	31.00300	73.96700	2250857874
14657	Diptera	Culicidae	Aedes	Aedes aegypti	SPECIES	31.05994	-93.20772	3356894313
14658	Diptera	Culicidae	Aedes	Aedes aegypti	SPECIES	31.09000	-97.36000	1264888830
14659	Diptera	Culicidae	Aedes	Aedes aegypti	SPECIES	31.09370	-91.10060	3801942359
14660	Diptera	Culicidae	Aedes	Aedes aegypti	SPECIES	31.14352	-93.26100	3356888312
14661	Diptera	Culicidae	Aedes	Aedes aegypti	SPECIES	31.16000	72.19000	3347209181
14662	Diptera	Culicidae	Aedes	Aedes aegypti	SPECIES	31.21000	-82.37000	1264888947
14663	Diptera	Culicidae	Aedes	Aedes aegypti	SPECIES	31.26000	-110.96000	1264889347
14664	Diptera	Culicidae	Aedes	Aedes aegypti	SPECIES	31.26000	-110.94000	1264889361
14665	Diptera	Culicidae	Aedes	Aedes aegypti	SPECIES	31.27000	-110.93000	1264889336
14666	Diptera	Culicidae	Aedes	Aedes aegypti	SPECIES	31.27000	-110.97000	1264889359
14667	Diptera	Culicidae	Aedes	Aedes aegypti	SPECIES	31.27000	72.31000	3350261459
14668	Diptera	Culicidae	Aedes	Aedes aegypti	SPECIES	31.28000	-110.96000	1264889358
14669	Diptera	Culicidae	Aedes	Aedes aegypti	SPECIES	31.29000	-110.93000	1264889355
14670	Diptera	Culicidae	Aedes	Aedes aegypti	SPECIES	31.32000	-92.53000	1264889381
14671	Diptera	Culicidae	Aedes	Aedes aegypti	SPECIES	31.33000	-110.94000	1264888473
14672	Diptera	Culicidae	Aedes	Aedes aegypti	SPECIES	31.33000	-109.95000	1264888485
14673	Diptera	Culicidae	Aedes	Aedes aegypti	SPECIES	31.33000	-85.70000	1264888613
14674	Diptera	Culicidae	Aedes	Aedes aegypti	SPECIES	31.33000	-94.73000	1264888801
14675	Diptera	Culicidae	Aedes	Aedes aegypti	SPECIES	31.33000	-110.93000	1264889364
14676	Diptera	Culicidae	Aedes	Aedes aegypti	SPECIES	31.33000	-110.95000	1264889380
14677	Diptera	Culicidae	Aedes	Aedes aegypti	SPECIES	31.34000	-110.94000	1264889371
14678	Diptera	Culicidae	Aedes	Aedes aegypti	SPECIES	31.34000	-109.95000	1264889379

№	Order	Family	Genus	Species	Taxon Rank	Latitude	Longitude	GBIF_ID
14679	Diptera	Culicidae	Aedes	Aedes aegypti	SPECIES	31.34000	-110.93000	1264890636
14680	Diptera	Culicidae	Aedes	Aedes aegypti	SPECIES	31.35000	-110.94000	1264888548
14681	Diptera	Culicidae	Aedes	Aedes aegypti	SPECIES	31.35000	-109.54000	1264889391
14682	Diptera	Culicidae	Aedes	Aedes aegypti	SPECIES	31.42000	73.08000	1264887296
14683	Diptera	Culicidae	Aedes	Aedes aegypti	SPECIES	31.42000	73.05000	3350381479
14684	Diptera	Culicidae	Aedes	Aedes aegypti	SPECIES	31.43000	-100.42000	1264889324
14685	Diptera	Culicidae	Aedes	Aedes aegypti	SPECIES	31.45122	-97.12582	3985765218
14686	Diptera	Culicidae	Aedes	Aedes aegypti	SPECIES	31.46000	-97.20000	1264888428
14687	Diptera	Culicidae	Aedes	Aedes aegypti	SPECIES	31.49000	-97.25000	1264888411
14688	Diptera	Culicidae	Aedes	Aedes aegypti	SPECIES	31.49000	-99.36000	1264890759
14689	Diptera	Culicidae	Aedes	Aedes aegypti	SPECIES	31.50763	74.32375	3496616097
14690	Diptera	Culicidae	Aedes	Aedes aegypti	SPECIES	31.50770	74.32355	3496607434
14691	Diptera	Culicidae	Aedes	Aedes aegypti	SPECIES	31.53000	-110.85000	1264871664
14692	Diptera	Culicidae	Aedes	Aedes aegypti	SPECIES	31.53000	-110.29000	1264889426
14693	Diptera	Culicidae	Aedes	Aedes aegypti	SPECIES	31.54000	74.30000	1264886278
14694	Diptera	Culicidae	Aedes	Aedes aegypti	SPECIES	31.54000	-110.30000	1264889415
14695	Diptera	Culicidae	Aedes	Aedes aegypti	SPECIES	31.54000	-110.29000	1264889433
14696	Diptera	Culicidae	Aedes	Aedes aegypti	SPECIES	31.55000	74.36000	1264887465
14697	Diptera	Culicidae	Aedes	Aedes aegypti	SPECIES	31.55000	-97.14000	1264890004
14698	Diptera	Culicidae	Aedes	Aedes aegypti	SPECIES	31.56000	-97.20000	1264871699
14699	Diptera	Culicidae	Aedes	Aedes aegypti	SPECIES	31.56207	-110.24168	3314426214
14700	Diptera	Culicidae	Aedes	Aedes aegypti	SPECIES	31.56539	-110.27128	3760207751
14701	Diptera	Culicidae	Aedes	Aedes aegypti	SPECIES	31.56701	-97.14939	4102682950
14702	Diptera	Culicidae	Aedes	Aedes aegypti	SPECIES	31.56763	-111.05096	3330495568
14703	Diptera	Culicidae	Aedes	Aedes aegypti	SPECIES	31.56973	-106.26509	1453122919
14704	Diptera	Culicidae	Aedes	Aedes aegypti	SPECIES	31.57000	74.37000	1264886313
14705	Diptera	Culicidae	Aedes	Aedes aegypti	SPECIES	31.57000	74.33000	3347315335
14706	Diptera	Culicidae	Aedes	Aedes aegypti	SPECIES	31.57000	74.34000	3350365584
14707	Diptera	Culicidae	Aedes	Aedes aegypti	SPECIES	31.57000	74.28000	3350438370
14708	Diptera	Culicidae	Aedes	Aedes aegypti	SPECIES	31.58745	-97.16974	3912077897
14709	Diptera	Culicidae	Aedes	Aedes aegypti	SPECIES	31.59000	74.33000	1264886854
14710	Diptera	Culicidae	Aedes	Aedes aegypti	SPECIES	31.60000	-94.66000	1264888821
14711	Diptera	Culicidae	Aedes	Aedes aegypti	SPECIES	31.62000	73.50000	3350349545
14712	Diptera	Culicidae	Aedes	Aedes aegypti	SPECIES	31.68000	-106.48000	1264889403
14713	Diptera	Culicidae	Aedes	Aedes aegypti	SPECIES	31.69000	73.92000	3347348310
14714	Diptera	Culicidae	Aedes	Aedes aegypti	SPECIES	31.72000	73.99000	1264887302
14715	Diptera	Culicidae	Aedes	Aedes aegypti	SPECIES	31.72483	-110.88018	3911982663
14716	Diptera	Culicidae	Aedes	Aedes aegypti	SPECIES	31.74000	-106.44000	1264886650
14717	Diptera	Culicidae	Aedes	Aedes aegypti	SPECIES	31.74430	-106.44395	3947400473
14718	Diptera	Culicidae	Aedes	Aedes aegypti	SPECIES	31.74683	-106.44263	4111743357
14719	Diptera	Culicidae	Aedes	Aedes aegypti	SPECIES	31.75000	-106.47000	1264889396

№	Order	Family	Genus	Species	Taxon Rank	Latitude	Longitude	GBIF_ID
14720	Diptera	Culicidae	Aedes	Aedes aegypti	SPECIES	31.75067	-111.06517	3384416294
14721	Diptera	Culicidae	Aedes	Aedes aegypti	SPECIES	31.76000	-95.63000	1264888948
14722	Diptera	Culicidae	Aedes	Aedes aegypti	SPECIES	31.76823	-106.34427	3773050068
14723	Diptera	Culicidae	Aedes	Aedes aegypti	SPECIES	31.77000	-106.24000	1264871654
14724	Diptera	Culicidae	Aedes	Aedes aegypti	SPECIES	31.77000	-106.49000	1264889397
14725	Diptera	Culicidae	Aedes	Aedes aegypti	SPECIES	31.77290	-110.85736	4067942524
14726	Diptera	Culicidae	Aedes	Aedes aegypti	SPECIES	31.79000	-95.15000	1264888824
14727	Diptera	Culicidae	Aedes	Aedes aegypti	SPECIES	31.79268	-106.45338	3384955845
14728	Diptera	Culicidae	Aedes	Aedes aegypti	SPECIES	31.81523	-116.60139	3455794564
14729	Diptera	Culicidae	Aedes	Aedes aegypti	SPECIES	31.82647	-102.36923	2901721931
14730	Diptera	Culicidae	Aedes	Aedes aegypti	SPECIES	31.84671	-106.54939	2242763284
14731	Diptera	Culicidae	Aedes	Aedes aegypti	SPECIES	31.86000	-112.85000	1264886725
14732	Diptera	Culicidae	Aedes	Aedes aegypti	SPECIES	31.86000	-81.63000	1264888652
14733	Diptera	Culicidae	Aedes	Aedes aegypti	SPECIES	31.86340	-97.38265	2814414167
14734	Diptera	Culicidae	Aedes	Aedes aegypti	SPECIES	31.86361	-116.61083	1894399059
14735	Diptera	Culicidae	Aedes	Aedes aegypti	SPECIES	31.87000	-102.03000	1264871700
14736	Diptera	Culicidae	Aedes	Aedes aegypti	SPECIES	31.88265	-116.62430	2883009869
14737	Diptera	Culicidae	Aedes	Aedes aegypti	SPECIES	31.88841	-102.32916	3355560683
14738	Diptera	Culicidae	Aedes	Aedes aegypti	SPECIES	31.89000	-109.75000	1264871629
14739	Diptera	Culicidae	Aedes	Aedes aegypti	SPECIES	31.91000	-110.21000	1264889427
14740	Diptera	Culicidae	Aedes	Aedes aegypti	SPECIES	31.93000	34.80000	1264887843
14741	Diptera	Culicidae	Aedes	Aedes aegypti	SPECIES	31.95000	-110.93000	1264888528
14742	Diptera	Culicidae	Aedes	Aedes aegypti	SPECIES	31.96000	-110.30000	1264889434
14743	Diptera	Culicidae	Aedes	Aedes aegypti	SPECIES	32.01000	-81.15000	1264888664
14744	Diptera	Culicidae	Aedes	Aedes aegypti	SPECIES	32.04133	-111.08847	3902413351
14745	Diptera	Culicidae	Aedes	Aedes aegypti	SPECIES	32.04845	-102.10611	2864892377
14746	Diptera	Culicidae	Aedes	Aedes aegypti	SPECIES	32.06000	-93.73000	1264871618
14747	Diptera	Culicidae	Aedes	Aedes aegypti	SPECIES	32.06000	-81.09000	1264889058
14748	Diptera	Culicidae	Aedes	Aedes aegypti	SPECIES	32.10000	-111.79000	1264871640
14749	Diptera	Culicidae	Aedes	Aedes aegypti	SPECIES	32.10000	74.17000	3350377367
14750	Diptera	Culicidae	Aedes	Aedes aegypti	SPECIES	32.15000	74.18000	1264887279
14751	Diptera	Culicidae	Aedes	Aedes aegypti	SPECIES	32.16083	-111.03118	2851072701
14752	Diptera	Culicidae	Aedes	Aedes aegypti	SPECIES	32.16675	-110.83498	3343798843
14753	Diptera	Culicidae	Aedes	Aedes aegypti	SPECIES	32.18000	-110.82000	1264889389
14754	Diptera	Culicidae	Aedes	Aedes aegypti	SPECIES	32.19000	-110.83000	1264889366
14755	Diptera	Culicidae	Aedes	Aedes aegypti	SPECIES	32.21000	-110.93000	3818537209
14756	Diptera	Culicidae	Aedes	Aedes aegypti	SPECIES	32.21104	-110.83764	2251935721
14757	Diptera	Culicidae	Aedes	Aedes aegypti	SPECIES	32.21370	-110.92600	3443540631
14758	Diptera	Culicidae	Aedes	Aedes aegypti	SPECIES	32.22000	-110.99000	1264888746
14759	Diptera	Culicidae	Aedes	Aedes aegypti	SPECIES	32.22000	-110.98000	1264888869
14760	Diptera	Culicidae	Aedes	Aedes aegypti	SPECIES	32.22000	-110.93000	1264889342

№	Order	Family	Genus	Species	Taxon Rank	Latitude	Longitude	GBIF_ID
14761	Diptera	Culicidae	Aedes	Aedes aegypti	SPECIES	32.22000	-110.90000	1264889365
14762	Diptera	Culicidae	Aedes	Aedes aegypti	SPECIES	32.22000	-110.81000	1264889402
14763	Diptera	Culicidae	Aedes	Aedes aegypti	SPECIES	32.22282	-110.97027	2813941437
14764	Diptera	Culicidae	Aedes	Aedes aegypti	SPECIES	32.23000	-110.83000	1264889374
14765	Diptera	Culicidae	Aedes	Aedes aegypti	SPECIES	32.23000	-110.98000	1264890633
14766	Diptera	Culicidae	Aedes	Aedes aegypti	SPECIES	32.23167	-110.89806	2294427126
14767	Diptera	Culicidae	Aedes	Aedes aegypti	SPECIES	32.23278	-110.94972	2456506239
14768	Diptera	Culicidae	Aedes	Aedes aegypti	SPECIES	32.23417	-110.95262	4111722291
14769	Diptera	Culicidae	Aedes	Aedes aegypti	SPECIES	32.23901	-111.03335	1946201705
14770	Diptera	Culicidae	Aedes	Aedes aegypti	SPECIES	32.24000	-110.81000	1264889378
14771	Diptera	Culicidae	Aedes	Aedes aegypti	SPECIES	32.24036	-110.79606	2518092755
14772	Diptera	Culicidae	Aedes	Aedes aegypti	SPECIES	32.24188	-111.16557	3902969993
14773	Diptera	Culicidae	Aedes	Aedes aegypti	SPECIES	32.24383	-111.16528	2422881733
14774	Diptera	Culicidae	Aedes	Aedes aegypti	SPECIES	32.24645	-110.88481	3385016647
14775	Diptera	Culicidae	Aedes	Aedes aegypti	SPECIES	32.24645	-110.88484	3902412633
14776	Diptera	Culicidae	Aedes	Aedes aegypti	SPECIES	32.25000	-109.84000	1264889370
14777	Diptera	Culicidae	Aedes	Aedes aegypti	SPECIES	32.25000	-110.94000	1264889406
14778	Diptera	Culicidae	Aedes	Aedes aegypti	SPECIES	32.26221	-111.03107	3090542529
14779	Diptera	Culicidae	Aedes	Aedes aegypti	SPECIES	32.27000	-110.80000	1264889373
14780	Diptera	Culicidae	Aedes	Aedes aegypti	SPECIES	32.27000	-110.91000	1264889390
14781	Diptera	Culicidae	Aedes	Aedes aegypti	SPECIES	32.27128	-106.78178	1933461592
14782	Diptera	Culicidae	Aedes	Aedes aegypti	SPECIES	32.27301	-106.76435	1913143547
14783	Diptera	Culicidae	Aedes	Aedes aegypti	SPECIES	32.27351	-106.78396	1933461648
14784	Diptera	Culicidae	Aedes	Aedes aegypti	SPECIES	32.29061	-106.78735	3355071856
14785	Diptera	Culicidae	Aedes	Aedes aegypti	SPECIES	32.29450	-106.75950	2620127492
14786	Diptera	Culicidae	Aedes	Aedes aegypti	SPECIES	32.30000	-99.89000	1264871681
14787	Diptera	Culicidae	Aedes	Aedes aegypti	SPECIES	32.30273	-106.73653	3384261377
14788	Diptera	Culicidae	Aedes	Aedes aegypti	SPECIES	32.31140	-97.01421	1913204994
14789	Diptera	Culicidae	Aedes	Aedes aegypti	SPECIES	32.31141	-97.01493	1453090024
14790	Diptera	Culicidae	Aedes	Aedes aegypti	SPECIES	32.31168	-97.01586	1453105037
14791	Diptera	Culicidae	Aedes	Aedes aegypti	SPECIES	32.31169	-97.01589	1315060438
14792	Diptera	Culicidae	Aedes	Aedes aegypti	SPECIES	32.31172	-97.01078	1572334990
14793	Diptera	Culicidae	Aedes	Aedes aegypti	SPECIES	32.31175	-97.01588	1453210679
14794	Diptera	Culicidae	Aedes	Aedes aegypti	SPECIES	32.31177	-97.01594	4138002987
14795	Diptera	Culicidae	Aedes	Aedes aegypti	SPECIES	32.31178	-97.01585	1305163436
14796	Diptera	Culicidae	Aedes	Aedes aegypti	SPECIES	32.31178	-97.01590	1453187920
14797	Diptera	Culicidae	Aedes	Aedes aegypti	SPECIES	32.31180	-97.01592	2265879362
14798	Diptera	Culicidae	Aedes	Aedes aegypti	SPECIES	32.31181	-97.01592	2456496318
14799	Diptera	Culicidae	Aedes	Aedes aegypti	SPECIES	32.32000	-89.03000	1264888804
14800	Diptera	Culicidae	Aedes	Aedes aegypti	SPECIES	32.32000	-90.89000	1264888826
14801	Diptera	Culicidae	Aedes	Aedes aegypti	SPECIES	32.32000	-89.02000	1264888979

№	Order	Family	Genus	Species	Taxon Rank	Latitude	Longitude	GBIF_ID
14802	Diptera	Culicidae	Aedes	Aedes aegypti	SPECIES	32.33000	-88.93000	1264888966
14803	Diptera	Culicidae	Aedes	Aedes aegypti	SPECIES	32.33947	-110.98173	3399163916
14804	Diptera	Culicidae	Aedes	Aedes aegypti	SPECIES	32.34600	-108.70990	3902903538
14805	Diptera	Culicidae	Aedes	Aedes aegypti	SPECIES	32.34676	-106.81374	3355245310
14806	Diptera	Culicidae	Aedes	Aedes aegypti	SPECIES	32.36000	-96.80000	1264871676
14807	Diptera	Culicidae	Aedes	Aedes aegypti	SPECIES	32.36000	-106.82000	1264871677
14808	Diptera	Culicidae	Aedes	Aedes aegypti	SPECIES	32.36000	-111.00000	1264889401
14809	Diptera	Culicidae	Aedes	Aedes aegypti	SPECIES	32.37000	-84.96000	1264888659
14810	Diptera	Culicidae	Aedes	Aedes aegypti	SPECIES	32.37000	-88.71000	1264888815
14811	Diptera	Culicidae	Aedes	Aedes aegypti	SPECIES	32.38000	-97.37000	1264871748
14812	Diptera	Culicidae	Aedes	Aedes aegypti	SPECIES	32.38000	-88.71000	1264888552
14813	Diptera	Culicidae	Aedes	Aedes aegypti	SPECIES	32.38000	-86.36000	1264889363
14814	Diptera	Culicidae	Aedes	Aedes aegypti	SPECIES	32.38000	-111.05000	1264889372
14815	Diptera	Culicidae	Aedes	Aedes aegypti	SPECIES	32.38000	34.93000	1264889672
14816	Diptera	Culicidae	Aedes	Aedes aegypti	SPECIES	32.39639	-110.97694	2423038471
14817	Diptera	Culicidae	Aedes	Aedes aegypti	SPECIES	32.40669	-99.69080	1946367959
14818	Diptera	Culicidae	Aedes	Aedes aegypti	SPECIES	32.41248	-96.82021	3915404709
14819	Diptera	Culicidae	Aedes	Aedes aegypti	SPECIES	32.41264	-96.82085	3903453726
14820	Diptera	Culicidae	Aedes	Aedes aegypti	SPECIES	32.41265	-96.82086	3903494334
14821	Diptera	Culicidae	Aedes	Aedes aegypti	SPECIES	32.42000	-104.23000	1264890375
14822	Diptera	Culicidae	Aedes	Aedes aegypti	SPECIES	32.42682	-99.77450	3399388366
14823	Diptera	Culicidae	Aedes	Aedes aegypti	SPECIES	32.43177	-99.70112	2423260022
14824	Diptera	Culicidae	Aedes	Aedes aegypti	SPECIES	32.43360	-111.07160	3031640061
14825	Diptera	Culicidae	Aedes	Aedes aegypti	SPECIES	32.44989	-110.77432	3118483395
14826	Diptera	Culicidae	Aedes	Aedes aegypti	SPECIES	32.46720	-97.23640	3030687770
14827	Diptera	Culicidae	Aedes	Aedes aegypti	SPECIES	32.48000	-92.15000	1264871588
14828	Diptera	Culicidae	Aedes	Aedes aegypti	SPECIES	32.48000	-104.30000	1264871745
14829	Diptera	Culicidae	Aedes	Aedes aegypti	SPECIES	32.48000	-93.77000	1264890388
14830	Diptera	Culicidae	Aedes	Aedes aegypti	SPECIES	32.49000	-94.07000	1264888816
14831	Diptera	Culicidae	Aedes	Aedes aegypti	SPECIES	32.49260	-97.33570	3030747298
14832	Diptera	Culicidae	Aedes	Aedes aegypti	SPECIES	32.49530	-97.36410	3030756515
14833	Diptera	Culicidae	Aedes	Aedes aegypti	SPECIES	32.50000	-93.56000	1264889377
14834	Diptera	Culicidae	Aedes	Aedes aegypti	SPECIES	32.50046	-116.70552	2864882904
14835	Diptera	Culicidae	Aedes	Aedes aegypti	SPECIES	32.50508	-93.65210	3048749868
14836	Diptera	Culicidae	Aedes	Aedes aegypti	SPECIES	32.51000	-84.87000	1264871742
14837	Diptera	Culicidae	Aedes	Aedes aegypti	SPECIES	32.51530	-97.34390	3030747396
14838	Diptera	Culicidae	Aedes	Aedes aegypti	SPECIES	32.52630	-97.33390	3030749284
14839	Diptera	Culicidae	Aedes	Aedes aegypti	SPECIES	32.52650	-97.29460	3030742866
14840	Diptera	Culicidae	Aedes	Aedes aegypti	SPECIES	32.52980	-97.29790	3030702773
14841	Diptera	Culicidae	Aedes	Aedes aegypti	SPECIES	32.53000	-117.02000	1264888574
14842	Diptera	Culicidae	Aedes	Aedes aegypti	SPECIES	32.53060	-97.27080	3030746988

№	Order	Family	Genus	Species	Taxon Rank	Latitude	Longitude	GBIF_ID
14843	Diptera	Culicidae	Aedes	Aedes aegypti	SPECIES	32.53100	-97.36140	3030758566
14844	Diptera	Culicidae	Aedes	Aedes aegypti	SPECIES	32.54020	-97.32520	3030749297
14845	Diptera	Culicidae	Aedes	Aedes aegypti	SPECIES	32.54210	-97.32090	3030746986
14846	Diptera	Culicidae	Aedes	Aedes aegypti	SPECIES	32.54550	-97.33660	3030758507
14847	Diptera	Culicidae	Aedes	Aedes aegypti	SPECIES	32.54640	-97.34120	3030752480
14848	Diptera	Culicidae	Aedes	Aedes aegypti	SPECIES	32.54688	-116.93515	2451644902
14849	Diptera	Culicidae	Aedes	Aedes aegypti	SPECIES	32.54790	-97.32480	3030747937
14850	Diptera	Culicidae	Aedes	Aedes aegypti	SPECIES	32.55000	-94.37000	1264890850
14851	Diptera	Culicidae	Aedes	Aedes aegypti	SPECIES	32.55650	-97.41710	3030747337
14852	Diptera	Culicidae	Aedes	Aedes aegypti	SPECIES	32.55660	-97.41760	3030749143
14853	Diptera	Culicidae	Aedes	Aedes aegypti	SPECIES	32.55690	-97.41790	3030221346
14854	Diptera	Culicidae	Aedes	Aedes aegypti	SPECIES	32.55780	-97.35660	3030742796
14855	Diptera	Culicidae	Aedes	Aedes aegypti	SPECIES	32.55790	-97.35640	3030749069
14856	Diptera	Culicidae	Aedes	Aedes aegypti	SPECIES	32.55800	-97.26650	3030727524
14857	Diptera	Culicidae	Aedes	Aedes aegypti	SPECIES	32.55860	-97.26650	3030605570
14858	Diptera	Culicidae	Aedes	Aedes aegypti	SPECIES	32.55860	-97.26660	3030756533
14859	Diptera	Culicidae	Aedes	Aedes aegypti	SPECIES	32.55870	-97.26690	3030585468
14860	Diptera	Culicidae	Aedes	Aedes aegypti	SPECIES	32.56000	35.33000	1264889692
14861	Diptera	Culicidae	Aedes	Aedes aegypti	SPECIES	32.56120	-97.33740	3030758520
14862	Diptera	Culicidae	Aedes	Aedes aegypti	SPECIES	32.56190	-97.14030	3030639373
14863	Diptera	Culicidae	Aedes	Aedes aegypti	SPECIES	32.56310	-97.33920	3030747998
14864	Diptera	Culicidae	Aedes	Aedes aegypti	SPECIES	32.56310	-97.14120	3030752378
14865	Diptera	Culicidae	Aedes	Aedes aegypti	SPECIES	32.56970	-97.20750	3030747343
14866	Diptera	Culicidae	Aedes	Aedes aegypti	SPECIES	32.57000	-116.63000	1264888538
14867	Diptera	Culicidae	Aedes	Aedes aegypti	SPECIES	32.57010	-97.20700	3030656875
14868	Diptera	Culicidae	Aedes	Aedes aegypti	SPECIES	32.57030	-97.20660	3030746103
14869	Diptera	Culicidae	Aedes	Aedes aegypti	SPECIES	32.57180	-97.07810	3030757379
14870	Diptera	Culicidae	Aedes	Aedes aegypti	SPECIES	32.57210	-97.35890	3030757324
14871	Diptera	Culicidae	Aedes	Aedes aegypti	SPECIES	32.57370	-97.37680	3030755550
14872	Diptera	Culicidae	Aedes	Aedes aegypti	SPECIES	32.57890	-97.34650	3030748289
14873	Diptera	Culicidae	Aedes	Aedes aegypti	SPECIES	32.57900	-97.37250	3030711626
14874	Diptera	Culicidae	Aedes	Aedes aegypti	SPECIES	32.57907	-110.85089	3408192649
14875	Diptera	Culicidae	Aedes	Aedes aegypti	SPECIES	32.57910	-97.34660	3030756029
14876	Diptera	Culicidae	Aedes	Aedes aegypti	SPECIES	32.58010	-97.15970	3030747353
14877	Diptera	Culicidae	Aedes	Aedes aegypti	SPECIES	32.58230	-97.21130	3030695469
14878	Diptera	Culicidae	Aedes	Aedes aegypti	SPECIES	32.58560	-97.12600	3030752346
14879	Diptera	Culicidae	Aedes	Aedes aegypti	SPECIES	32.58780	-97.09110	3030746786
14880	Diptera	Culicidae	Aedes	Aedes aegypti	SPECIES	32.58790	-97.34310	3030629761
14881	Diptera	Culicidae	Aedes	Aedes aegypti	SPECIES	32.58920	-97.22320	3030606736
14882	Diptera	Culicidae	Aedes	Aedes aegypti	SPECIES	32.58950	-97.48780	3030639405
14883	Diptera	Culicidae	Aedes	Aedes aegypti	SPECIES	32.59010	-97.18880	3030670441

№	Order	Family	Genus	Species	Taxon Rank	Latitude	Longitude	GBIF_ID
14884	Diptera	Culicidae	Aedes	Aedes aegypti	SPECIES	32.59010	-97.48750	3030757457
14885	Diptera	Culicidae	Aedes	Aedes aegypti	SPECIES	32.59080	-97.18690	3030758548
14886	Diptera	Culicidae	Aedes	Aedes aegypti	SPECIES	32.59870	-97.25590	3030673434
14887	Diptera	Culicidae	Aedes	Aedes aegypti	SPECIES	32.59880	-97.25610	3030748043
14888	Diptera	Culicidae	Aedes	Aedes aegypti	SPECIES	32.59950	-97.21550	3030746095
14889	Diptera	Culicidae	Aedes	Aedes aegypti	SPECIES	32.60400	-97.13960	3030748831
14890	Diptera	Culicidae	Aedes	Aedes aegypti	SPECIES	32.60698	-96.89624	2238930511
14891	Diptera	Culicidae	Aedes	Aedes aegypti	SPECIES	32.61310	-117.06436	2423258218
14892	Diptera	Culicidae	Aedes	Aedes aegypti	SPECIES	32.61650	-97.09840	3030756543
14893	Diptera	Culicidae	Aedes	Aedes aegypti	SPECIES	32.61930	-97.22100	3030747344
14894	Diptera	Culicidae	Aedes	Aedes aegypti	SPECIES	32.61950	-97.22110	3030249174
14895	Diptera	Culicidae	Aedes	Aedes aegypti	SPECIES	32.61990	-97.09850	3030631790
14896	Diptera	Culicidae	Aedes	Aedes aegypti	SPECIES	32.62060	-97.11190	3030630884
14897	Diptera	Culicidae	Aedes	Aedes aegypti	SPECIES	32.62140	-97.27050	3030645845
14898	Diptera	Culicidae	Aedes	Aedes aegypti	SPECIES	32.62180	-97.22090	3030728572
14899	Diptera	Culicidae	Aedes	Aedes aegypti	SPECIES	32.62250	-97.21960	3030694464
14900	Diptera	Culicidae	Aedes	Aedes aegypti	SPECIES	32.62380	-97.27010	3030631787
14901	Diptera	Culicidae	Aedes	Aedes aegypti	SPECIES	32.62480	-97.28500	3030633189
14902	Diptera	Culicidae	Aedes	Aedes aegypti	SPECIES	32.62560	-97.53590	3030669646
14903	Diptera	Culicidae	Aedes	Aedes aegypti	SPECIES	32.62623	-117.08305	3337725659
14904	Diptera	Culicidae	Aedes	Aedes aegypti	SPECIES	32.62658	-116.97756	3415463960
14905	Diptera	Culicidae	Aedes	Aedes aegypti	SPECIES	32.62690	-97.19600	3030182906
14906	Diptera	Culicidae	Aedes	Aedes aegypti	SPECIES	32.62740	-97.31020	3030582462
14907	Diptera	Culicidae	Aedes	Aedes aegypti	SPECIES	32.62780	-97.40090	3030629934
14908	Diptera	Culicidae	Aedes	Aedes aegypti	SPECIES	32.62970	-97.36950	3030634100
14909	Diptera	Culicidae	Aedes	Aedes aegypti	SPECIES	32.63119	-117.03661	3005179534
14910	Diptera	Culicidae	Aedes	Aedes aegypti	SPECIES	32.63200	-97.28710	3030630853
14911	Diptera	Culicidae	Aedes	Aedes aegypti	SPECIES	32.63270	-97.28710	3030223404
14912	Diptera	Culicidae	Aedes	Aedes aegypti	SPECIES	32.63300	-97.28720	3030756967
14913	Diptera	Culicidae	Aedes	Aedes aegypti	SPECIES	32.63330	-97.10800	3030651110
14914	Diptera	Culicidae	Aedes	Aedes aegypti	SPECIES	32.63360	-97.12460	3030748214
14915	Diptera	Culicidae	Aedes	Aedes aegypti	SPECIES	32.63370	-97.18200	3030671417
14916	Diptera	Culicidae	Aedes	Aedes aegypti	SPECIES	32.63410	-97.12440	3030747239
14917	Diptera	Culicidae	Aedes	Aedes aegypti	SPECIES	32.63460	-97.26620	3030584329
14918	Diptera	Culicidae	Aedes	Aedes aegypti	SPECIES	32.63470	-97.26240	3030665551
14919	Diptera	Culicidae	Aedes	Aedes aegypti	SPECIES	32.63480	-97.13950	3030204402
14920	Diptera	Culicidae	Aedes	Aedes aegypti	SPECIES	32.63620	-97.22990	3030705061
14921	Diptera	Culicidae	Aedes	Aedes aegypti	SPECIES	32.63730	-97.20920	3030755395
14922	Diptera	Culicidae	Aedes	Aedes aegypti	SPECIES	32.64050	-97.28060	3030685366
14923	Diptera	Culicidae	Aedes	Aedes aegypti	SPECIES	32.64070	-97.28080	3030657708
14924	Diptera	Culicidae	Aedes	Aedes aegypti	SPECIES	32.64120	-97.28060	3030189075

№	Order	Family	Genus	Species	Taxon Rank	Latitude	Longitude	GBIF_ID
14925	Diptera	Culicidae	Aedes	Aedes aegypti	SPECIES	32.64641	-117.07496	3031592925
14926	Diptera	Culicidae	Aedes	Aedes aegypti	SPECIES	32.64690	-97.23030	3030719492
14927	Diptera	Culicidae	Aedes	Aedes aegypti	SPECIES	32.64700	-97.23030	3030671652
14928	Diptera	Culicidae	Aedes	Aedes aegypti	SPECIES	32.64890	-97.15020	3030288812
14929	Diptera	Culicidae	Aedes	Aedes aegypti	SPECIES	32.64900	-97.35080	3030724559
14930	Diptera	Culicidae	Aedes	Aedes aegypti	SPECIES	32.64980	-97.34030	3030265605
14931	Diptera	Culicidae	Aedes	Aedes aegypti	SPECIES	32.64980	-97.34760	3030580802
14932	Diptera	Culicidae	Aedes	Aedes aegypti	SPECIES	32.65000	74.21000	3350269623
14933	Diptera	Culicidae	Aedes	Aedes aegypti	SPECIES	32.65020	-97.34060	3030631516
14934	Diptera	Culicidae	Aedes	Aedes aegypti	SPECIES	32.65060	-97.36140	3030685348
14935	Diptera	Culicidae	Aedes	Aedes aegypti	SPECIES	32.65090	-97.34950	3030630505
14936	Diptera	Culicidae	Aedes	Aedes aegypti	SPECIES	32.65120	-97.20850	3030180576
14937	Diptera	Culicidae	Aedes	Aedes aegypti	SPECIES	32.65120	-97.20860	3030702190
14938	Diptera	Culicidae	Aedes	Aedes aegypti	SPECIES	32.65230	-97.15130	3030671474
14939	Diptera	Culicidae	Aedes	Aedes aegypti	SPECIES	32.65240	-97.21870	3030643538
14940	Diptera	Culicidae	Aedes	Aedes aegypti	SPECIES	32.65270	-97.34430	3030685048
14941	Diptera	Culicidae	Aedes	Aedes aegypti	SPECIES	32.65280	-97.21830	3030688705
14942	Diptera	Culicidae	Aedes	Aedes aegypti	SPECIES	32.65300	-97.12700	3030631073
14943	Diptera	Culicidae	Aedes	Aedes aegypti	SPECIES	32.65330	-97.33990	3030662639
14944	Diptera	Culicidae	Aedes	Aedes aegypti	SPECIES	32.65520	-97.07040	3030758530
14945	Diptera	Culicidae	Aedes	Aedes aegypti	SPECIES	32.65530	-97.12040	3030746362
14946	Diptera	Culicidae	Aedes	Aedes aegypti	SPECIES	32.65550	-97.34550	3030246583
14947	Diptera	Culicidae	Aedes	Aedes aegypti	SPECIES	32.65550	-97.34380	3030686306
14948	Diptera	Culicidae	Aedes	Aedes aegypti	SPECIES	32.65740	-97.49340	3030580852
14949	Diptera	Culicidae	Aedes	Aedes aegypti	SPECIES	32.65780	-97.33640	3030182036
14950	Diptera	Culicidae	Aedes	Aedes aegypti	SPECIES	32.65780	-97.35010	3030663412
14951	Diptera	Culicidae	Aedes	Aedes aegypti	SPECIES	32.65850	-97.39760	3030579163
14952	Diptera	Culicidae	Aedes	Aedes aegypti	SPECIES	32.65860	-97.34920	3030604839
14953	Diptera	Culicidae	Aedes	Aedes aegypti	SPECIES	32.65940	-97.19300	3030758020
14954	Diptera	Culicidae	Aedes	Aedes aegypti	SPECIES	32.65950	-97.34740	3030646001
14955	Diptera	Culicidae	Aedes	Aedes aegypti	SPECIES	32.65970	-97.48940	3030757308
14956	Diptera	Culicidae	Aedes	Aedes aegypti	SPECIES	32.66000	-97.47900	3030620076
14957	Diptera	Culicidae	Aedes	Aedes aegypti	SPECIES	32.66080	-97.34350	3030246877
14958	Diptera	Culicidae	Aedes	Aedes aegypti	SPECIES	32.66087	-97.20212	3902796485
14959	Diptera	Culicidae	Aedes	Aedes aegypti	SPECIES	32.66100	-97.34030	3030653872
14960	Diptera	Culicidae	Aedes	Aedes aegypti	SPECIES	32.66140	-97.34440	3030685076
14961	Diptera	Culicidae	Aedes	Aedes aegypti	SPECIES	32.66230	-97.42410	3030218446
14962	Diptera	Culicidae	Aedes	Aedes aegypti	SPECIES	32.66260	-97.35210	3030261710
14963	Diptera	Culicidae	Aedes	Aedes aegypti	SPECIES	32.66280	-97.16070	3030631975
14964	Diptera	Culicidae	Aedes	Aedes aegypti	SPECIES	32.66300	-97.16050	3030694507
14965	Diptera	Culicidae	Aedes	Aedes aegypti	SPECIES	32.66333	-115.46778	1894399777

№	Order	Family	Genus	Species	Taxon Rank	Latitude	Longitude	GBIF_ID
14966	Diptera	Culicidae	Aedes	Aedes aegypti	SPECIES	32.66376	-117.01965	2634051430
14967	Diptera	Culicidae	Aedes	Aedes aegypti	SPECIES	32.66400	-97.10460	3030752371
14968	Diptera	Culicidae	Aedes	Aedes aegypti	SPECIES	32.66470	-97.15170	3030296303
14969	Diptera	Culicidae	Aedes	Aedes aegypti	SPECIES	32.66551	-115.50034	4018277782
14970	Diptera	Culicidae	Aedes	Aedes aegypti	SPECIES	32.66630	-97.14410	3030582550
14971	Diptera	Culicidae	Aedes	Aedes aegypti	SPECIES	32.66840	-97.50050	3030688217
14972	Diptera	Culicidae	Aedes	Aedes aegypti	SPECIES	32.66860	-97.44600	3030758358
14973	Diptera	Culicidae	Aedes	Aedes aegypti	SPECIES	32.66880	-97.50140	3030700622
14974	Diptera	Culicidae	Aedes	Aedes aegypti	SPECIES	32.67000	74.47000	3350258300
14975	Diptera	Culicidae	Aedes	Aedes aegypti	SUBSPECIES	32.67000	-16.92000	3987240919
14976	Diptera	Culicidae	Aedes	Aedes aegypti	SPECIES	32.67030	-97.46920	3030747397
14977	Diptera	Culicidae	Aedes	Aedes aegypti	SPECIES	32.67090	-97.46800	3030192319
14978	Diptera	Culicidae	Aedes	Aedes aegypti	SPECIES	32.67140	-97.45810	3030686260
14979	Diptera	Culicidae	Aedes	Aedes aegypti	SPECIES	32.67380	-97.44570	3030186149
14980	Diptera	Culicidae	Aedes	Aedes aegypti	SPECIES	32.67570	-97.43500	3030193517
14981	Diptera	Culicidae	Aedes	Aedes aegypti	SPECIES	32.67740	-97.47660	3030755537
14982	Diptera	Culicidae	Aedes	Aedes aegypti	SPECIES	32.67750	-97.32440	3030632258
14983	Diptera	Culicidae	Aedes	Aedes aegypti	SPECIES	32.67760	-97.45750	3030695974
14984	Diptera	Culicidae	Aedes	Aedes aegypti	SPECIES	32.67780	-97.44700	3030221637
14985	Diptera	Culicidae	Aedes	Aedes aegypti	SPECIES	32.67782	-117.08242	2862366413
14986	Diptera	Culicidae	Aedes	Aedes aegypti	SPECIES	32.67880	-97.44520	3030671704
14987	Diptera	Culicidae	Aedes	Aedes aegypti	SPECIES	32.67890	-97.46110	3030698375
14988	Diptera	Culicidae	Aedes	Aedes aegypti	SPECIES	32.67910	-97.45760	3030655622
14989	Diptera	Culicidae	Aedes	Aedes aegypti	SPECIES	32.68260	-97.50860	3030632197
14990	Diptera	Culicidae	Aedes	Aedes aegypti	SPECIES	32.68290	-97.50910	3030756335
14991	Diptera	Culicidae	Aedes	Aedes aegypti	SPECIES	32.68320	-97.09490	3030758026
14992	Diptera	Culicidae	Aedes	Aedes aegypti	SPECIES	32.68570	-97.41770	3030180062
14993	Diptera	Culicidae	Aedes	Aedes aegypti	SPECIES	32.68580	-97.12650	3030215683
14994	Diptera	Culicidae	Aedes	Aedes aegypti	SPECIES	32.68650	-97.21160	3030755400
14995	Diptera	Culicidae	Aedes	Aedes aegypti	SPECIES	32.68670	-97.21130	3030747965
14996	Diptera	Culicidae	Aedes	Aedes aegypti	SPECIES	32.68790	-97.41860	3030197358
14997	Diptera	Culicidae	Aedes	Aedes aegypti	SPECIES	32.68860	-97.17370	3030689102
14998	Diptera	Culicidae	Aedes	Aedes aegypti	SPECIES	32.69020	-97.44950	3030312415
14999	Diptera	Culicidae	Aedes	Aedes aegypti	SPECIES	32.69030	-97.46220	3030300421
15000	Diptera	Culicidae	Aedes	Aedes aegypti	SPECIES	32.69080	-97.16120	3030306179
15001	Diptera	Culicidae	Aedes	Aedes aegypti	SPECIES	32.69210	-97.19940	3030685315
15002	Diptera	Culicidae	Aedes	Aedes aegypti	SPECIES	32.69260	-97.08090	3030748987
15003	Diptera	Culicidae	Aedes	Aedes aegypti	SPECIES	32.69500	-97.41700	3030306238
15004	Diptera	Culicidae	Aedes	Aedes aegypti	SPECIES	32.69550	-97.16140	3030757381
15005	Diptera	Culicidae	Aedes	Aedes aegypti	SPECIES	32.69570	-97.43670	3030213811
15006	Diptera	Culicidae	Aedes	Aedes aegypti	SPECIES	32.69570	-97.15240	3030588388

№	Order	Family	Genus	Species	Taxon Rank	Latitude	Longitude	GBIF_ID
15007	Diptera	Culicidae	Aedes	Aedes aegypti	SPECIES	32.69610	-97.36740	3030672456
15008	Diptera	Culicidae	Aedes	Aedes aegypti	SPECIES	32.69930	-97.25130	3030639548
15009	Diptera	Culicidae	Aedes	Aedes aegypti	SPECIES	32.69940	-97.12790	3030312547
15010	Diptera	Culicidae	Aedes	Aedes aegypti	SPECIES	32.70210	-97.15530	3030696362
15011	Diptera	Culicidae	Aedes	Aedes aegypti	SPECIES	32.70350	-97.33130	3030575003
15012	Diptera	Culicidae	Aedes	Aedes aegypti	SPECIES	32.70560	-97.14310	3030715802
15013	Diptera	Culicidae	Aedes	Aedes aegypti	SPECIES	32.70626	-16.76879	4162140366
15014	Diptera	Culicidae	Aedes	Aedes aegypti	SPECIES	32.70626	-16.76882	4162183413
15015	Diptera	Culicidae	Aedes	Aedes aegypti	SPECIES	32.70710	-97.13810	3030245033
15016	Diptera	Culicidae	Aedes	Aedes aegypti	SPECIES	32.70740	-97.18520	3030752459
15017	Diptera	Culicidae	Aedes	Aedes aegypti	SPECIES	32.70770	-97.43770	3030632504
15018	Diptera	Culicidae	Aedes	Aedes aegypti	SPECIES	32.70810	-97.29920	3030746335
15019	Diptera	Culicidae	Aedes	Aedes aegypti	SPECIES	32.70810	-97.18560	3030748862
15020	Diptera	Culicidae	Aedes	Aedes aegypti	SPECIES	32.70860	-97.46740	3030748375
15021	Diptera	Culicidae	Aedes	Aedes aegypti	SPECIES	32.71000	-97.18580	3030574462
15022	Diptera	Culicidae	Aedes	Aedes aegypti	SPECIES	32.71020	-97.14790	3030631964
15023	Diptera	Culicidae	Aedes	Aedes aegypti	SPECIES	32.71060	-97.06720	3030758423
15024	Diptera	Culicidae	Aedes	Aedes aegypti	SPECIES	32.71120	-97.15240	3030695337
15025	Diptera	Culicidae	Aedes	Aedes aegypti	SPECIES	32.71120	-97.53110	3030755511
15026	Diptera	Culicidae	Aedes	Aedes aegypti	SPECIES	32.71130	-97.53170	3030632481
15027	Diptera	Culicidae	Aedes	Aedes aegypti	SPECIES	32.71170	-97.15300	3030302424
15028	Diptera	Culicidae	Aedes	Aedes aegypti	SPECIES	32.71180	-97.16360	3030748922
15029	Diptera	Culicidae	Aedes	Aedes aegypti	SPECIES	32.71210	-97.46650	3030287944
15030	Diptera	Culicidae	Aedes	Aedes aegypti	SPECIES	32.71220	-97.06690	3030758357
15031	Diptera	Culicidae	Aedes	Aedes aegypti	SPECIES	32.71310	-97.16280	3030632457
15032	Diptera	Culicidae	Aedes	Aedes aegypti	SPECIES	32.71310	-97.11420	3030747963
15033	Diptera	Culicidae	Aedes	Aedes aegypti	SPECIES	32.71350	-97.16210	3030746093
15034	Diptera	Culicidae	Aedes	Aedes aegypti	SPECIES	32.71360	-97.15050	3030300077
15035	Diptera	Culicidae	Aedes	Aedes aegypti	SPECIES	32.71380	-97.15260	3030644971
15036	Diptera	Culicidae	Aedes	Aedes aegypti	SPECIES	32.71380	-97.11460	3030748380
15037	Diptera	Culicidae	Aedes	Aedes aegypti	SPECIES	32.71390	-97.15960	3030695308
15038	Diptera	Culicidae	Aedes	Aedes aegypti	SPECIES	32.71410	-97.15840	3030634246
15039	Diptera	Culicidae	Aedes	Aedes aegypti	SPECIES	32.71420	-97.16030	3030233491
15040	Diptera	Culicidae	Aedes	Aedes aegypti	SPECIES	32.71420	-97.15420	3030757528
15041	Diptera	Culicidae	Aedes	Aedes aegypti	SPECIES	32.71430	-97.45800	3030756449
15042	Diptera	Culicidae	Aedes	Aedes aegypti	SPECIES	32.71440	-97.15600	3030687046
15043	Diptera	Culicidae	Aedes	Aedes aegypti	SPECIES	32.71460	-97.16140	3030594564
15044	Diptera	Culicidae	Aedes	Aedes aegypti	SPECIES	32.71470	-97.15960	3030752344
15045	Diptera	Culicidae	Aedes	Aedes aegypti	SPECIES	32.71480	-97.16060	3030200540
15046	Diptera	Culicidae	Aedes	Aedes aegypti	SPECIES	32.71490	-97.15650	3030673510
15047	Diptera	Culicidae	Aedes	Aedes aegypti	SPECIES	32.71520	-97.14950	3030685217

№	Order	Family	Genus	Species	Taxon Rank	Latitude	Longitude	GBIF_ID
15048	Diptera	Culicidae	Aedes	Aedes aegypti	SPECIES	32.71530	-97.15370	3030758506
15049	Diptera	Culicidae	Aedes	Aedes aegypti	SPECIES	32.71550	-97.15740	3030195647
15050	Diptera	Culicidae	Aedes	Aedes aegypti	SPECIES	32.71560	-97.15060	3030598141
15051	Diptera	Culicidae	Aedes	Aedes aegypti	SPECIES	32.71574	-117.16108	2980847160
15052	Diptera	Culicidae	Aedes	Aedes aegypti	SPECIES	32.71580	-97.15220	3030748809
15053	Diptera	Culicidae	Aedes	Aedes aegypti	SPECIES	32.71580	-97.16600	3030749160
15054	Diptera	Culicidae	Aedes	Aedes aegypti	SPECIES	32.71580	-97.15630	3030758440
15055	Diptera	Culicidae	Aedes	Aedes aegypti	SPECIES	32.71600	-97.16350	3030686111
15056	Diptera	Culicidae	Aedes	Aedes aegypti	SPECIES	32.71600	-97.14990	3030748827
15057	Diptera	Culicidae	Aedes	Aedes aegypti	SPECIES	32.71620	-97.16650	3030214669
15058	Diptera	Culicidae	Aedes	Aedes aegypti	SPECIES	32.71700	-97.15650	3030232535
15059	Diptera	Culicidae	Aedes	Aedes aegypti	SPECIES	32.71720	-97.15510	3030579132
15060	Diptera	Culicidae	Aedes	Aedes aegypti	SPECIES	32.71730	-97.15850	3030659887
15061	Diptera	Culicidae	Aedes	Aedes aegypti	SPECIES	32.71740	-97.15510	3030668519
15062	Diptera	Culicidae	Aedes	Aedes aegypti	SPECIES	32.71740	-97.16110	3030746226
15063	Diptera	Culicidae	Aedes	Aedes aegypti	SPECIES	32.71750	-117.14451	3924995797
15064	Diptera	Culicidae	Aedes	Aedes aegypti	SPECIES	32.71760	-97.15460	3030306288
15065	Diptera	Culicidae	Aedes	Aedes aegypti	SPECIES	32.71760	-97.15260	3030651461
15066	Diptera	Culicidae	Aedes	Aedes aegypti	SPECIES	32.71790	-97.15160	3030646603
15067	Diptera	Culicidae	Aedes	Aedes aegypti	SPECIES	32.71800	-97.16260	3030652196
15068	Diptera	Culicidae	Aedes	Aedes aegypti	SPECIES	32.71810	-97.16180	3030294391
15069	Diptera	Culicidae	Aedes	Aedes aegypti	SPECIES	32.71820	-97.14990	3030313505
15070	Diptera	Culicidae	Aedes	Aedes aegypti	SPECIES	32.71820	-97.15610	3030631847
15071	Diptera	Culicidae	Aedes	Aedes aegypti	SPECIES	32.71850	-97.15300	3030608973
15072	Diptera	Culicidae	Aedes	Aedes aegypti	SPECIES	32.71867	-97.33740	2901691671
15073	Diptera	Culicidae	Aedes	Aedes aegypti	SPECIES	32.71910	-97.16320	3030244905
15074	Diptera	Culicidae	Aedes	Aedes aegypti	SPECIES	32.71910	-97.35700	3030631043
15075	Diptera	Culicidae	Aedes	Aedes aegypti	SPECIES	32.71910	-97.12420	3030661541
15076	Diptera	Culicidae	Aedes	Aedes aegypti	SPECIES	32.71920	-97.15780	3030697436
15077	Diptera	Culicidae	Aedes	Aedes aegypti	SPECIES	32.71940	-97.15980	3030662543
15078	Diptera	Culicidae	Aedes	Aedes aegypti	SPECIES	32.71950	-97.16050	3030634067
15079	Diptera	Culicidae	Aedes	Aedes aegypti	SPECIES	32.71950	-97.12360	3030749270
15080	Diptera	Culicidae	Aedes	Aedes aegypti	SPECIES	32.71970	-97.16320	3030248022
15081	Diptera	Culicidae	Aedes	Aedes aegypti	SPECIES	32.71980	-97.15210	3030756374
15082	Diptera	Culicidae	Aedes	Aedes aegypti	SPECIES	32.71990	-97.16180	3030639468
15083	Diptera	Culicidae	Aedes	Aedes aegypti	SPECIES	32.72010	-97.16610	3030742362
15084	Diptera	Culicidae	Aedes	Aedes aegypti	SPECIES	32.72040	-97.45720	3030232031
15085	Diptera	Culicidae	Aedes	Aedes aegypti	SPECIES	32.72040	-97.15960	3030633244
15086	Diptera	Culicidae	Aedes	Aedes aegypti	SPECIES	32.72040	-97.16050	3030670631
15087	Diptera	Culicidae	Aedes	Aedes aegypti	SPECIES	32.72040	-97.15540	3030758400
15088	Diptera	Culicidae	Aedes	Aedes aegypti	SPECIES	32.72100	-97.16630	3030300182

№	Order	Family	Genus	Species	Taxon Rank	Latitude	Longitude	GBIF_ID
15089	Diptera	Culicidae	Aedes	Aedes aegypti	SPECIES	32.72110	-97.25240	3030673445
15090	Diptera	Culicidae	Aedes	Aedes aegypti	SPECIES	32.72140	-97.16260	3030301077
15091	Diptera	Culicidae	Aedes	Aedes aegypti	SPECIES	32.72200	-97.16640	3030757416
15092	Diptera	Culicidae	Aedes	Aedes aegypti	SPECIES	32.72410	-97.16620	3030286040
15093	Diptera	Culicidae	Aedes	Aedes aegypti	SPECIES	32.72520	-97.35420	3030634236
15094	Diptera	Culicidae	Aedes	Aedes aegypti	SPECIES	32.72528	-97.32056	3802034305
15095	Diptera	Culicidae	Aedes	Aedes aegypti	SPECIES	32.72550	-97.14930	3030614917
15096	Diptera	Culicidae	Aedes	Aedes aegypti	SPECIES	32.72635	-117.13274	2862307319
15097	Diptera	Culicidae	Aedes	Aedes aegypti	SPECIES	32.72720	-97.18140	3030184844
15098	Diptera	Culicidae	Aedes	Aedes aegypti	SPECIES	32.72730	-97.46890	3030639473
15099	Diptera	Culicidae	Aedes	Aedes aegypti	SPECIES	32.72830	-97.41310	3030643406
15100	Diptera	Culicidae	Aedes	Aedes aegypti	SPECIES	32.73040	-97.11820	3030746818
15101	Diptera	Culicidae	Aedes	Aedes aegypti	SPECIES	32.73080	-97.31890	3030728542
15102	Diptera	Culicidae	Aedes	Aedes aegypti	SPECIES	32.73101	-97.11762	1323010708
15103	Diptera	Culicidae	Aedes	Aedes aegypti	SPECIES	32.73287	-117.24903	2856535259
15104	Diptera	Culicidae	Aedes	Aedes aegypti	SPECIES	32.73290	-97.34710	3030198435
15105	Diptera	Culicidae	Aedes	Aedes aegypti	SPECIES	32.73320	-97.14380	3030273395
15106	Diptera	Culicidae	Aedes	Aedes aegypti	SPECIES	32.73390	-97.09300	3030758327
15107	Diptera	Culicidae	Aedes	Aedes aegypti	SPECIES	32.73420	-97.21870	3030631878
15108	Diptera	Culicidae	Aedes	Aedes aegypti	SPECIES	32.73456	-97.13148	2826237448
15109	Diptera	Culicidae	Aedes	Aedes aegypti	SPECIES	32.73587	-117.10613	3455821796
15110	Diptera	Culicidae	Aedes	Aedes aegypti	SPECIES	32.73587	-117.10612	3961350076
15111	Diptera	Culicidae	Aedes	Aedes aegypti	SPECIES	32.73588	-117.10611	3859621065
15112	Diptera	Culicidae	Aedes	Aedes aegypti	SPECIES	32.73589	-117.10611	3391426088
15113	Diptera	Culicidae	Aedes	Aedes aegypti	SPECIES	32.73589	-117.10609	3408024097
15114	Diptera	Culicidae	Aedes	Aedes aegypti	SPECIES	32.73666	-117.25321	2445061678
15115	Diptera	Culicidae	Aedes	Aedes aegypti	SPECIES	32.73669	-117.25318	3399256320
15116	Diptera	Culicidae	Aedes	Aedes aegypti	SPECIES	32.73716	-16.79544	4162124353
15117	Diptera	Culicidae	Aedes	Aedes aegypti	SPECIES	32.73825	-16.73751	4162070568
15118	Diptera	Culicidae	Aedes	Aedes aegypti	SPECIES	32.73900	-97.40310	3030711395
15119	Diptera	Culicidae	Aedes	Aedes aegypti	SPECIES	32.73960	-97.40360	3030757499
15120	Diptera	Culicidae	Aedes	Aedes aegypti	SPECIES	32.73970	-97.41000	3030749029
15121	Diptera	Culicidae	Aedes	Aedes aegypti	SPECIES	32.74000	129.88000	1264888383
15122	Diptera	Culicidae	Aedes	Aedes aegypti	SPECIES	32.74040	-97.36270	3030725554
15123	Diptera	Culicidae	Aedes	Aedes aegypti	SPECIES	32.74080	-97.40670	3030578998
15124	Diptera	Culicidae	Aedes	Aedes aegypti	SPECIES	32.74100	-97.38540	3030631588
15125	Diptera	Culicidae	Aedes	Aedes aegypti	SPECIES	32.74210	-97.40730	3030187602
15126	Diptera	Culicidae	Aedes	Aedes aegypti	SPECIES	32.74340	-97.14930	3030212401
15127	Diptera	Culicidae	Aedes	Aedes aegypti	SPECIES	32.74370	-97.28600	3030686312
15128	Diptera	Culicidae	Aedes	Aedes aegypti	SPECIES	32.74396	-117.23793	3966805324
15129	Diptera	Culicidae	Aedes	Aedes aegypti	SPECIES	32.74430	-97.41320	3030186519

№	Order	Family	Genus	Species	Taxon Rank	Latitude	Longitude	GBIF_ID
15130	Diptera	Culicidae	Aedes	Aedes aegypti	SPECIES	32.74440	-97.41310	3030748883
15131	Diptera	Culicidae	Aedes	Aedes aegypti	SPECIES	32.74440	-97.41370	3030756504
15132	Diptera	Culicidae	Aedes	Aedes aegypti	SPECIES	32.74500	-97.41060	3030684964
15133	Diptera	Culicidae	Aedes	Aedes aegypti	SPECIES	32.74550	-97.40760	3030743113
15134	Diptera	Culicidae	Aedes	Aedes aegypti	SPECIES	32.74644	-117.14826	3415676333
15135	Diptera	Culicidae	Aedes	Aedes aegypti	SPECIES	32.74699	-117.11921	1914250435
15136	Diptera	Culicidae	Aedes	Aedes aegypti	SPECIES	32.74830	-97.33530	3030671442
15137	Diptera	Culicidae	Aedes	Aedes aegypti	SPECIES	32.74845	-97.27052	3947424409
15138	Diptera	Culicidae	Aedes	Aedes aegypti	SPECIES	32.75000	-97.33000	1264888498
15139	Diptera	Culicidae	Aedes	Aedes aegypti	SPECIES	32.75167	-117.05966	1913206121
15140	Diptera	Culicidae	Aedes	Aedes aegypti	SPECIES	32.75250	-97.15980	3030188066
15141	Diptera	Culicidae	Aedes	Aedes aegypti	SPECIES	32.75260	-97.07950	3030702978
15142	Diptera	Culicidae	Aedes	Aedes aegypti	SPECIES	32.75304	-97.32667	3079658376
15143	Diptera	Culicidae	Aedes	Aedes aegypti	SPECIES	32.75320	-117.15642	2823424528
15144	Diptera	Culicidae	Aedes	Aedes aegypti	SPECIES	32.75470	-97.10620	3030688895
15145	Diptera	Culicidae	Aedes	Aedes aegypti	SPECIES	32.75550	-97.40700	3030209178
15146	Diptera	Culicidae	Aedes	Aedes aegypti	SPECIES	32.75628	-97.45727	3784964852
15147	Diptera	Culicidae	Aedes	Aedes aegypti	SPECIES	32.75692	-117.02652	1946343089
15148	Diptera	Culicidae	Aedes	Aedes aegypti	SPECIES	32.75770	-97.36110	3030725577
15149	Diptera	Culicidae	Aedes	Aedes aegypti	SPECIES	32.75780	-97.46020	3030758562
15150	Diptera	Culicidae	Aedes	Aedes aegypti	SPECIES	32.75781	-117.05318	3399193139
15151	Diptera	Culicidae	Aedes	Aedes aegypti	SPECIES	32.75782	-117.05318	3385211547
15152	Diptera	Culicidae	Aedes	Aedes aegypti	SPECIES	32.75782	-117.05318	2823094844
15153	Diptera	Culicidae	Aedes	Aedes aegypti	SPECIES	32.75800	-97.46020	3030749159
15154	Diptera	Culicidae	Aedes	Aedes aegypti	SPECIES	32.75926	-97.46688	3947169704
15155	Diptera	Culicidae	Aedes	Aedes aegypti	SPECIES	32.75950	-97.50090	3030643448
15156	Diptera	Culicidae	Aedes	Aedes aegypti	SPECIES	32.75960	-97.32960	3030633299
15157	Diptera	Culicidae	Aedes	Aedes aegypti	SPECIES	32.76020	-97.43270	3030641577
15158	Diptera	Culicidae	Aedes	Aedes aegypti	SPECIES	32.76235	-97.11821	1562879946
15159	Diptera	Culicidae	Aedes	Aedes aegypti	SPECIES	32.76280	-97.41080	3030300861
15160	Diptera	Culicidae	Aedes	Aedes aegypti	SPECIES	32.76315	-117.09201	3398991623
15161	Diptera	Culicidae	Aedes	Aedes aegypti	SPECIES	32.76420	-97.14170	3030697518
15162	Diptera	Culicidae	Aedes	Aedes aegypti	SPECIES	32.76440	-97.39910	3030749216
15163	Diptera	Culicidae	Aedes	Aedes aegypti	SPECIES	32.76460	-97.39830	3030181177
15164	Diptera	Culicidae	Aedes	Aedes aegypti	SPECIES	32.76460	-97.40400	3030574761
15165	Diptera	Culicidae	Aedes	Aedes aegypti	SPECIES	32.76460	-97.14140	3030688844
15166	Diptera	Culicidae	Aedes	Aedes aegypti	SPECIES	32.76470	-97.39870	3030755349
15167	Diptera	Culicidae	Aedes	Aedes aegypti	SPECIES	32.76480	-97.24120	3030684323
15168	Diptera	Culicidae	Aedes	Aedes aegypti	SPECIES	32.76550	-97.39100	3030595188
15169	Diptera	Culicidae	Aedes	Aedes aegypti	SPECIES	32.76570	-97.40210	3030747880
15170	Diptera	Culicidae	Aedes	Aedes aegypti	SPECIES	32.76590	-97.39210	3030605954

№	Order	Family	Genus	Species	Taxon Rank	Latitude	Longitude	GBIF_ID
15171	Diptera	Culicidae	Aedes	Aedes aegypti	SPECIES	32.76590	-97.40220	3030670537
15172	Diptera	Culicidae	Aedes	Aedes aegypti	SPECIES	32.76610	-97.39430	3030684912
15173	Diptera	Culicidae	Aedes	Aedes aegypti	SPECIES	32.76610	-97.19040	3030748388
15174	Diptera	Culicidae	Aedes	Aedes aegypti	SPECIES	32.76650	-97.39160	3030186249
15175	Diptera	Culicidae	Aedes	Aedes aegypti	SPECIES	32.76650	-97.39900	3030643433
15176	Diptera	Culicidae	Aedes	Aedes aegypti	SPECIES	32.76670	-97.40070	3030702781
15177	Diptera	Culicidae	Aedes	Aedes aegypti	SPECIES	32.76680	-97.41990	3030633521
15178	Diptera	Culicidae	Aedes	Aedes aegypti	SPECIES	32.76740	-97.40110	3030583213
15179	Diptera	Culicidae	Aedes	Aedes aegypti	SPECIES	32.76760	-97.40110	3030189241
15180	Diptera	Culicidae	Aedes	Aedes aegypti	SPECIES	32.76770	-97.39110	3030749293
15181	Diptera	Culicidae	Aedes	Aedes aegypti	SPECIES	32.76790	-97.40470	3030748275
15182	Diptera	Culicidae	Aedes	Aedes aegypti	SPECIES	32.76800	-97.40310	3030749299
15183	Diptera	Culicidae	Aedes	Aedes aegypti	SPECIES	32.76821	-117.11392	3391529210
15184	Diptera	Culicidae	Aedes	Aedes aegypti	SPECIES	32.76824	-117.11393	3005184537
15185	Diptera	Culicidae	Aedes	Aedes aegypti	SPECIES	32.76845	-117.08372	3455160693
15186	Diptera	Culicidae	Aedes	Aedes aegypti	SPECIES	32.76930	-97.40350	3030688234
15187	Diptera	Culicidae	Aedes	Aedes aegypti	SPECIES	32.76950	-97.39300	3030583237
15188	Diptera	Culicidae	Aedes	Aedes aegypti	SPECIES	32.76960	-97.40750	3030260286
15189	Diptera	Culicidae	Aedes	Aedes aegypti	SPECIES	32.76970	-97.40520	3030747372
15190	Diptera	Culicidae	Aedes	Aedes aegypti	SPECIES	32.76980	-97.39500	3030233667
15191	Diptera	Culicidae	Aedes	Aedes aegypti	SPECIES	32.77000	-96.77000	1264871712
15192	Diptera	Culicidae	Aedes	Aedes aegypti	SPECIES	32.77000	-97.43000	1264889332
15193	Diptera	Culicidae	Aedes	Aedes aegypti	SPECIES	32.77000	-97.40620	3030673535
15194	Diptera	Culicidae	Aedes	Aedes aegypti	SPECIES	32.77000	-79.98000	4066964835
15195	Diptera	Culicidae	Aedes	Aedes aegypti	SPECIES	32.77040	-97.39870	3030646519
15196	Diptera	Culicidae	Aedes	Aedes aegypti	SPECIES	32.77070	-97.39690	3030660641
15197	Diptera	Culicidae	Aedes	Aedes aegypti	SPECIES	32.77080	-97.39420	3030285109
15198	Diptera	Culicidae	Aedes	Aedes aegypti	SPECIES	32.77090	-97.40530	3030748992
15199	Diptera	Culicidae	Aedes	Aedes aegypti	SPECIES	32.77100	-97.08890	3030661790
15200	Diptera	Culicidae	Aedes	Aedes aegypti	SPECIES	32.77120	-97.40730	3030299356
15201	Diptera	Culicidae	Aedes	Aedes aegypti	SPECIES	32.77120	-97.40410	3030686234
15202	Diptera	Culicidae	Aedes	Aedes aegypti	SPECIES	32.77130	-97.47192	3963702350
15203	Diptera	Culicidae	Aedes	Aedes aegypti	SPECIES	32.77130	-97.39830	3030687315
15204	Diptera	Culicidae	Aedes	Aedes aegypti	SPECIES	32.77140	-97.08830	3030716880
15205	Diptera	Culicidae	Aedes	Aedes aegypti	SPECIES	32.77150	-97.46420	3030709736
15206	Diptera	Culicidae	Aedes	Aedes aegypti	SPECIES	32.77150	-97.46440	3030749205
15207	Diptera	Culicidae	Aedes	Aedes aegypti	SPECIES	32.77200	-97.39490	3030629870
15208	Diptera	Culicidae	Aedes	Aedes aegypti	SPECIES	32.77230	-97.40090	3030618436
15209	Diptera	Culicidae	Aedes	Aedes aegypti	SPECIES	32.77233	-116.91944	2980808996
15210	Diptera	Culicidae	Aedes	Aedes aegypti	SPECIES	32.77250	-97.11520	3030756470
15211	Diptera	Culicidae	Aedes	Aedes aegypti	SPECIES	32.77260	-97.11530	3030702720

№	Order	Family	Genus	Species	Taxon Rank	Latitude	Longitude	GBIF_ID
15212	Diptera	Culicidae	Aedes	Aedes aegypti	SPECIES	32.77265	-117.06943	2901635319
15213	Diptera	Culicidae	Aedes	Aedes aegypti	SPECIES	32.77290	-97.39090	3030742365
15214	Diptera	Culicidae	Aedes	Aedes aegypti	SPECIES	32.77350	-97.39660	3030670407
15215	Diptera	Culicidae	Aedes	Aedes aegypti	SPECIES	32.77400	-97.40170	3030673508
15216	Diptera	Culicidae	Aedes	Aedes aegypti	SPECIES	32.77410	-97.40090	3030742831
15217	Diptera	Culicidae	Aedes	Aedes aegypti	SPECIES	32.77450	-97.39430	3030672659
15218	Diptera	Culicidae	Aedes	Aedes aegypti	SPECIES	32.77480	-97.39220	3030745818
15219	Diptera	Culicidae	Aedes	Aedes aegypti	SPECIES	32.77500	-97.39220	3030183271
15220	Diptera	Culicidae	Aedes	Aedes aegypti	SPECIES	32.77530	-97.39530	3030634135
15221	Diptera	Culicidae	Aedes	Aedes aegypti	SPECIES	32.77540	-97.09450	3030286175
15222	Diptera	Culicidae	Aedes	Aedes aegypti	SPECIES	32.77590	-97.40290	3030584310
15223	Diptera	Culicidae	Aedes	Aedes aegypti	SPECIES	32.77590	-97.40840	3030631173
15224	Diptera	Culicidae	Aedes	Aedes aegypti	SPECIES	32.77600	-97.40870	3030273333
15225	Diptera	Culicidae	Aedes	Aedes aegypti	SPECIES	32.77630	-97.46610	3030632463
15226	Diptera	Culicidae	Aedes	Aedes aegypti	SPECIES	32.77680	-97.39820	3030579094
15227	Diptera	Culicidae	Aedes	Aedes aegypti	SPECIES	32.77680	-97.40560	3030686120
15228	Diptera	Culicidae	Aedes	Aedes aegypti	SPECIES	32.77700	-97.39810	3030576147
15229	Diptera	Culicidae	Aedes	Aedes aegypti	SPECIES	32.77760	-97.39200	3030743234
15230	Diptera	Culicidae	Aedes	Aedes aegypti	SPECIES	32.77770	-97.39990	3030301150
15231	Diptera	Culicidae	Aedes	Aedes aegypti	SPECIES	32.77830	-97.39410	3030631821
15232	Diptera	Culicidae	Aedes	Aedes aegypti	SPECIES	32.77860	-97.39260	3030746975
15233	Diptera	Culicidae	Aedes	Aedes aegypti	SPECIES	32.77880	-97.39420	3030746239
15234	Diptera	Culicidae	Aedes	Aedes aegypti	SPECIES	32.77900	-97.40530	3030579046
15235	Diptera	Culicidae	Aedes	Aedes aegypti	SPECIES	32.77900	-97.40170	3030671553
15236	Diptera	Culicidae	Aedes	Aedes aegypti	SPECIES	32.77910	-97.39920	3030755310
15237	Diptera	Culicidae	Aedes	Aedes aegypti	SPECIES	32.77960	-97.39690	3030214577
15238	Diptera	Culicidae	Aedes	Aedes aegypti	SPECIES	32.77970	-97.40050	3030261779
15239	Diptera	Culicidae	Aedes	Aedes aegypti	SPECIES	32.78000	-97.29000	1264871680
15240	Diptera	Culicidae	Aedes	Aedes aegypti	SPECIES	32.78080	-97.40480	3030698310
15241	Diptera	Culicidae	Aedes	Aedes aegypti	SPECIES	32.78080	-97.40180	3030748981
15242	Diptera	Culicidae	Aedes	Aedes aegypti	SPECIES	32.78090	-97.39810	3030641586
15243	Diptera	Culicidae	Aedes	Aedes aegypti	SPECIES	32.78170	-97.39930	3030217411
15244	Diptera	Culicidae	Aedes	Aedes aegypti	SPECIES	32.78190	-97.40120	3030575953
15245	Diptera	Culicidae	Aedes	Aedes aegypti	SPECIES	32.78260	-97.39420	3030686077
15246	Diptera	Culicidae	Aedes	Aedes aegypti	SPECIES	32.78280	-97.39790	3030189094
15247	Diptera	Culicidae	Aedes	Aedes aegypti	SPECIES	32.78280	-97.39720	3030748976
15248	Diptera	Culicidae	Aedes	Aedes aegypti	SPECIES	32.78336	-79.93658	1914250733
15249	Diptera	Culicidae	Aedes	Aedes aegypti	SPECIES	32.78420	-97.40380	3030300408
15250	Diptera	Culicidae	Aedes	Aedes aegypti	SPECIES	32.78440	-97.35000	3030748423
15251	Diptera	Culicidae	Aedes	Aedes aegypti	SPECIES	32.78450	-97.39050	3030715310
15252	Diptera	Culicidae	Aedes	Aedes aegypti	SPECIES	32.78452	-96.70898	4145752296

№	Order	Family	Genus	Species	Taxon Rank	Latitude	Longitude	GBIF_ID
15253	Diptera	Culicidae	Aedes	Aedes aegypti	SPECIES	32.78470	-97.40230	3030181502
15254	Diptera	Culicidae	Aedes	Aedes aegypti	SPECIES	32.78470	-97.30840	3030666509
15255	Diptera	Culicidae	Aedes	Aedes aegypti	SPECIES	32.78480	-97.38580	3030700204
15256	Diptera	Culicidae	Aedes	Aedes aegypti	SPECIES	32.78480	-97.38760	3030742916
15257	Diptera	Culicidae	Aedes	Aedes aegypti	SPECIES	32.78511	-97.34073	3773385380
15258	Diptera	Culicidae	Aedes	Aedes aegypti	SPECIES	32.78530	-97.38690	3030584336
15259	Diptera	Culicidae	Aedes	Aedes aegypti	SPECIES	32.78530	-97.39320	3030752328
15260	Diptera	Culicidae	Aedes	Aedes aegypti	SPECIES	32.78570	-97.38680	3030607313
15261	Diptera	Culicidae	Aedes	Aedes aegypti	SPECIES	32.78590	-97.38930	3030698306
15262	Diptera	Culicidae	Aedes	Aedes aegypti	SPECIES	32.78670	-97.40020	3030632818
15263	Diptera	Culicidae	Aedes	Aedes aegypti	SPECIES	32.78700	-97.06540	3030719544
15264	Diptera	Culicidae	Aedes	Aedes aegypti	SPECIES	32.78740	-97.06520	3030196327
15265	Diptera	Culicidae	Aedes	Aedes aegypti	SPECIES	32.78760	-97.39090	3030696476
15266	Diptera	Culicidae	Aedes	Aedes aegypti	SPECIES	32.78810	-97.39430	3030305994
15267	Diptera	Culicidae	Aedes	Aedes aegypti	SPECIES	32.78850	-97.39090	3030634161
15268	Diptera	Culicidae	Aedes	Aedes aegypti	SPECIES	32.78850	-97.39080	3030642535
15269	Diptera	Culicidae	Aedes	Aedes aegypti	SPECIES	32.79150	-97.40500	3030686237
15270	Diptera	Culicidae	Aedes	Aedes aegypti	SPECIES	32.79160	-97.40350	3030698442
15271	Diptera	Culicidae	Aedes	Aedes aegypti	SPECIES	32.79170	-97.27170	3030287924
15272	Diptera	Culicidae	Aedes	Aedes aegypti	SPECIES	32.79170	-97.40560	3030756969
15273	Diptera	Culicidae	Aedes	Aedes aegypti	SPECIES	32.79490	-97.25790	3030752988
15274	Diptera	Culicidae	Aedes	Aedes aegypti	SPECIES	32.79590	-97.39970	3030596112
15275	Diptera	Culicidae	Aedes	Aedes aegypti	SPECIES	32.79780	-97.39540	3030559431
15276	Diptera	Culicidae	Aedes	Aedes aegypti	SPECIES	32.79800	-97.39490	3030749225
15277	Diptera	Culicidae	Aedes	Aedes aegypti	SPECIES	32.79950	-97.22720	3030629892
15278	Diptera	Culicidae	Aedes	Aedes aegypti	SPECIES	32.80030	-97.18060	3030575170
15279	Diptera	Culicidae	Aedes	Aedes aegypti	SPECIES	32.80170	-97.28800	3030684375
15280	Diptera	Culicidae	Aedes	Aedes aegypti	SPECIES	32.80173	-117.23650	3398757152
15281	Diptera	Culicidae	Aedes	Aedes aegypti	SPECIES	32.80220	-97.40860	3030758407
15282	Diptera	Culicidae	Aedes	Aedes aegypti	SPECIES	32.80270	-97.38390	3030633172
15283	Diptera	Culicidae	Aedes	Aedes aegypti	SPECIES	32.80290	-97.38370	3030643569
15284	Diptera	Culicidae	Aedes	Aedes aegypti	SPECIES	32.80350	-97.40220	3030687994
15285	Diptera	Culicidae	Aedes	Aedes aegypti	SPECIES	32.80380	-97.27450	3030742314
15286	Diptera	Culicidae	Aedes	Aedes aegypti	SPECIES	32.80430	-97.23020	3030748786
15287	Diptera	Culicidae	Aedes	Aedes aegypti	SPECIES	32.80550	-97.39240	3030757440
15288	Diptera	Culicidae	Aedes	Aedes aegypti	SPECIES	32.80610	-97.21600	3030634137
15289	Diptera	Culicidae	Aedes	Aedes aegypti	SPECIES	32.80660	-97.44620	3030643019
15290	Diptera	Culicidae	Aedes	Aedes aegypti	SPECIES	32.80730	-97.23820	3030757460
15291	Diptera	Culicidae	Aedes	Aedes aegypti	SPECIES	32.80740	-97.44360	3030605871
15292	Diptera	Culicidae	Aedes	Aedes aegypti	SPECIES	32.80800	-97.22700	3030695382
15293	Diptera	Culicidae	Aedes	Aedes aegypti	SPECIES	32.80830	-97.23100	3030745832

№	Order	Family	Genus	Species	Taxon Rank	Latitude	Longitude	GBIF_ID
15294	Diptera	Culicidae	Aedes	Aedes aegypti	SPECIES	32.80910	-97.17530	3030757425
15295	Diptera	Culicidae	Aedes	Aedes aegypti	SPECIES	32.80920	-97.22950	3030190586
15296	Diptera	Culicidae	Aedes	Aedes aegypti	SPECIES	32.80980	-97.17530	3030758309
15297	Diptera	Culicidae	Aedes	Aedes aegypti	SPECIES	32.81015	-117.19677	2984386829
15298	Diptera	Culicidae	Aedes	Aedes aegypti	SPECIES	32.81030	-97.23550	3030285381
15299	Diptera	Culicidae	Aedes	Aedes aegypti	SPECIES	32.81030	-97.24020	3030702631
15300	Diptera	Culicidae	Aedes	Aedes aegypti	SPECIES	32.81030	-97.40640	3030757322
15301	Diptera	Culicidae	Aedes	Aedes aegypti	SPECIES	32.81040	-97.43530	3030669473
15302	Diptera	Culicidae	Aedes	Aedes aegypti	SPECIES	32.81230	-97.35150	3030671462
15303	Diptera	Culicidae	Aedes	Aedes aegypti	SPECIES	32.81240	-97.44890	3030701292
15304	Diptera	Culicidae	Aedes	Aedes aegypti	SPECIES	32.81270	-97.25450	3030187175
15305	Diptera	Culicidae	Aedes	Aedes aegypti	SPECIES	32.81270	-97.35150	3030728540
15306	Diptera	Culicidae	Aedes	Aedes aegypti	SPECIES	32.81310	-97.25910	3030757997
15307	Diptera	Culicidae	Aedes	Aedes aegypti	SPECIES	32.81360	-97.16120	3030685905
15308	Diptera	Culicidae	Aedes	Aedes aegypti	SPECIES	32.81380	-97.49290	3030575028
15309	Diptera	Culicidae	Aedes	Aedes aegypti	SPECIES	32.81530	-97.15140	3030758565
15310	Diptera	Culicidae	Aedes	Aedes aegypti	SPECIES	32.81540	-97.15170	3030757502
15311	Diptera	Culicidae	Aedes	Aedes aegypti	SPECIES	32.81610	-97.19900	3030631600
15312	Diptera	Culicidae	Aedes	Aedes aegypti	SPECIES	32.81648	-117.17242	3966635762
15313	Diptera	Culicidae	Aedes	Aedes aegypti	SPECIES	32.81656	-117.05673	2864605828
15314	Diptera	Culicidae	Aedes	Aedes aegypti	SPECIES	32.81680	-97.43510	3030749166
15315	Diptera	Culicidae	Aedes	Aedes aegypti	SPECIES	32.81690	-97.43500	3030756396
15316	Diptera	Culicidae	Aedes	Aedes aegypti	SPECIES	32.81710	-97.19810	3030748996
15317	Diptera	Culicidae	Aedes	Aedes aegypti	SPECIES	32.81710	-97.19780	3030758558
15318	Diptera	Culicidae	Aedes	Aedes aegypti	SPECIES	32.81760	-97.20290	3030187480
15319	Diptera	Culicidae	Aedes	Aedes aegypti	SPECIES	32.81770	-97.18090	3030285880
15320	Diptera	Culicidae	Aedes	Aedes aegypti	SPECIES	32.81810	-97.26230	3030634210
15321	Diptera	Culicidae	Aedes	Aedes aegypti	SPECIES	32.81810	-97.23400	3030696963
15322	Diptera	Culicidae	Aedes	Aedes aegypti	SPECIES	32.81840	-97.17950	3030667518
15323	Diptera	Culicidae	Aedes	Aedes aegypti	SPECIES	32.81840	-97.49920	3030669640
15324	Diptera	Culicidae	Aedes	Aedes aegypti	SPECIES	32.81850	-97.16750	3030599085
15325	Diptera	Culicidae	Aedes	Aedes aegypti	SPECIES	32.81860	-97.24400	3030755986
15326	Diptera	Culicidae	Aedes	Aedes aegypti	SPECIES	32.81870	-97.04960	3030688361
15327	Diptera	Culicidae	Aedes	Aedes aegypti	SPECIES	32.81870	-97.22230	3030755432
15328	Diptera	Culicidae	Aedes	Aedes aegypti	SPECIES	32.81880	-97.24400	3030716740
15329	Diptera	Culicidae	Aedes	Aedes aegypti	SPECIES	32.81890	-97.22220	3030757556
15330	Diptera	Culicidae	Aedes	Aedes aegypti	SPECIES	32.81920	-97.16600	3030223066
15331	Diptera	Culicidae	Aedes	Aedes aegypti	SPECIES	32.81980	-97.52620	3030703200
15332	Diptera	Culicidae	Aedes	Aedes aegypti	SPECIES	32.82000	-97.52580	3030746382
15333	Diptera	Culicidae	Aedes	Aedes aegypti	SPECIES	32.82010	-97.21290	3030194483
15334	Diptera	Culicidae	Aedes	Aedes aegypti	SPECIES	32.82090	-97.35530	3030189101

№	Order	Family	Genus	Species	Taxon Rank	Latitude	Longitude	GBIF_ID
15335	Diptera	Culicidae	Aedes	Aedes aegypti	SPECIES	32.82130	-97.26340	3030755545
15336	Diptera	Culicidae	Aedes	Aedes aegypti	SPECIES	32.82150	-97.22690	3030684346
15337	Diptera	Culicidae	Aedes	Aedes aegypti	SPECIES	32.82250	-97.15070	3030311337
15338	Diptera	Culicidae	Aedes	Aedes aegypti	SPECIES	32.82300	-97.15130	3030618379
15339	Diptera	Culicidae	Aedes	Aedes aegypti	SPECIES	32.82410	-97.14880	3030575101
15340	Diptera	Culicidae	Aedes	Aedes aegypti	SPECIES	32.82410	-97.23500	3030755355
15341	Diptera	Culicidae	Aedes	Aedes aegypti	SPECIES	32.82420	-97.27400	3030641396
15342	Diptera	Culicidae	Aedes	Aedes aegypti	SPECIES	32.82530	-97.15110	3030757555
15343	Diptera	Culicidae	Aedes	Aedes aegypti	SPECIES	32.82590	-97.31100	3030728534
15344	Diptera	Culicidae	Aedes	Aedes aegypti	SPECIES	32.82618	-116.99624	3963367678
15345	Diptera	Culicidae	Aedes	Aedes aegypti	SPECIES	32.82680	-97.18370	3030645025
15346	Diptera	Culicidae	Aedes	Aedes aegypti	SPECIES	32.82720	-97.19000	3030757408
15347	Diptera	Culicidae	Aedes	Aedes aegypti	SPECIES	32.82740	-97.08430	3030702691
15348	Diptera	Culicidae	Aedes	Aedes aegypti	SPECIES	32.82740	-97.18970	3030757321
15349	Diptera	Culicidae	Aedes	Aedes aegypti	SPECIES	32.82740	-97.08450	3030758587
15350	Diptera	Culicidae	Aedes	Aedes aegypti	SPECIES	32.82820	-97.19480	3030647769
15351	Diptera	Culicidae	Aedes	Aedes aegypti	SPECIES	32.82880	-97.23820	3030215564
15352	Diptera	Culicidae	Aedes	Aedes aegypti	SPECIES	32.82930	-97.17870	3030189281
15353	Diptera	Culicidae	Aedes	Aedes aegypti	SPECIES	32.83000	-97.22970	3030300400
15354	Diptera	Culicidae	Aedes	Aedes aegypti	SPECIES	32.83020	-97.11320	3030748180
15355	Diptera	Culicidae	Aedes	Aedes aegypti	SPECIES	32.83030	-97.14970	3030752512
15356	Diptera	Culicidae	Aedes	Aedes aegypti	SPECIES	32.83090	-97.15870	3030581208
15357	Diptera	Culicidae	Aedes	Aedes aegypti	SPECIES	32.83100	-97.13140	3030623087
15358	Diptera	Culicidae	Aedes	Aedes aegypti	SPECIES	32.83110	-97.18580	3030287857
15359	Diptera	Culicidae	Aedes	Aedes aegypti	SPECIES	32.83120	-97.42120	3030631941
15360	Diptera	Culicidae	Aedes	Aedes aegypti	SPECIES	32.83130	-97.13650	3030303183
15361	Diptera	Culicidae	Aedes	Aedes aegypti	SPECIES	32.83240	-97.16730	3030311566
15362	Diptera	Culicidae	Aedes	Aedes aegypti	SPECIES	32.83260	-97.13360	3030281535
15363	Diptera	Culicidae	Aedes	Aedes aegypti	SPECIES	32.83260	-97.13330	3030630967
15364	Diptera	Culicidae	Aedes	Aedes aegypti	SPECIES	32.83330	-97.11760	3030197522
15365	Diptera	Culicidae	Aedes	Aedes aegypti	SPECIES	32.83380	-97.27510	3030583034
15366	Diptera	Culicidae	Aedes	Aedes aegypti	SPECIES	32.83590	-97.25440	3030698461
15367	Diptera	Culicidae	Aedes	Aedes aegypti	SPECIES	32.83600	-97.16930	3030660192
15368	Diptera	Culicidae	Aedes	Aedes aegypti	SPECIES	32.83670	-97.18800	3030631093
15369	Diptera	Culicidae	Aedes	Aedes aegypti	SPECIES	32.83770	-97.22880	3030656314
15370	Diptera	Culicidae	Aedes	Aedes aegypti	SPECIES	32.83790	-97.26860	3030748181
15371	Diptera	Culicidae	Aedes	Aedes aegypti	SPECIES	32.83790	-97.21330	3030749013
15372	Diptera	Culicidae	Aedes	Aedes aegypti	SPECIES	32.83870	-97.15260	3030672685
15373	Diptera	Culicidae	Aedes	Aedes aegypti	SPECIES	32.83950	-97.13130	3030222949
15374	Diptera	Culicidae	Aedes	Aedes aegypti	SPECIES	32.84030	-97.10640	3030630811
15375	Diptera	Culicidae	Aedes	Aedes aegypti	SPECIES	32.84130	-97.18060	3030303108

№	Order	Family	Genus	Species	Taxon Rank	Latitude	Longitude	GBIF_ID
15376	Diptera	Culicidae	Aedes	Aedes aegypti	SPECIES	32.84130	-97.17180	3030670479
15377	Diptera	Culicidae	Aedes	Aedes aegypti	SPECIES	32.84170	-97.07910	3030757318
15378	Diptera	Culicidae	Aedes	Aedes aegypti	SPECIES	32.84180	-97.14010	3030650218
15379	Diptera	Culicidae	Aedes	Aedes aegypti	SPECIES	32.84200	-97.22790	3030592772
15380	Diptera	Culicidae	Aedes	Aedes aegypti	SPECIES	32.84210	-97.22760	3030746866
15381	Diptera	Culicidae	Aedes	Aedes aegypti	SPECIES	32.84350	-97.15210	3030694448
15382	Diptera	Culicidae	Aedes	Aedes aegypti	SPECIES	32.84410	-97.14240	3030247996
15383	Diptera	Culicidae	Aedes	Aedes aegypti	SPECIES	32.84430	-97.18370	3030695018
15384	Diptera	Culicidae	Aedes	Aedes aegypti	SPECIES	32.84440	-97.13800	3030203035
15385	Diptera	Culicidae	Aedes	Aedes aegypti	SPECIES	32.84480	-97.12800	3030746096
15386	Diptera	Culicidae	Aedes	Aedes aegypti	SPECIES	32.84500	-97.24750	3030586313
15387	Diptera	Culicidae	Aedes	Aedes aegypti	SPECIES	32.84590	-97.47580	3030583296
15388	Diptera	Culicidae	Aedes	Aedes aegypti	SPECIES	32.84620	-97.14780	3030747743
15389	Diptera	Culicidae	Aedes	Aedes aegypti	SPECIES	32.84640	-97.14050	3030699831
15390	Diptera	Culicidae	Aedes	Aedes aegypti	SPECIES	32.84661	-109.76237	3985644066
15391	Diptera	Culicidae	Aedes	Aedes aegypti	SPECIES	32.84670	-97.12760	3030670545
15392	Diptera	Culicidae	Aedes	Aedes aegypti	SPECIES	32.84670	-97.09740	3030757429
15393	Diptera	Culicidae	Aedes	Aedes aegypti	SPECIES	32.84760	-97.11530	3030618417
15394	Diptera	Culicidae	Aedes	Aedes aegypti	SPECIES	32.84790	-97.34900	3030696458
15395	Diptera	Culicidae	Aedes	Aedes aegypti	SPECIES	32.84800	-97.37650	3030288298
15396	Diptera	Culicidae	Aedes	Aedes aegypti	SPECIES	32.84800	-97.10250	3030747984
15397	Diptera	Culicidae	Aedes	Aedes aegypti	SPECIES	32.84810	-97.23560	3030742303
15398	Diptera	Culicidae	Aedes	Aedes aegypti	SPECIES	32.84840	-97.36530	3030231543
15399	Diptera	Culicidae	Aedes	Aedes aegypti	SPECIES	32.85020	-97.10240	3030746742
15400	Diptera	Culicidae	Aedes	Aedes aegypti	SPECIES	32.85030	-97.20090	3030700178
15401	Diptera	Culicidae	Aedes	Aedes aegypti	SPECIES	32.85130	-97.33450	3030578810
15402	Diptera	Culicidae	Aedes	Aedes aegypti	SPECIES	32.85140	-97.52720	3030756973
15403	Diptera	Culicidae	Aedes	Aedes aegypti	SPECIES	32.85150	-97.38540	3030630503
15404	Diptera	Culicidae	Aedes	Aedes aegypti	SPECIES	32.85150	-97.52600	3030672428
15405	Diptera	Culicidae	Aedes	Aedes aegypti	SPECIES	32.85170	-97.17190	3030583108
15406	Diptera	Culicidae	Aedes	Aedes aegypti	SPECIES	32.85170	-97.12760	3030650231
15407	Diptera	Culicidae	Aedes	Aedes aegypti	SPECIES	32.85180	-97.17210	3030673985
15408	Diptera	Culicidae	Aedes	Aedes aegypti	SPECIES	32.85210	-97.27540	3030758528
15409	Diptera	Culicidae	Aedes	Aedes aegypti	SPECIES	32.85270	-97.12250	3030652901
15410	Diptera	Culicidae	Aedes	Aedes aegypti	SPECIES	32.85330	-97.27440	3030189019
15411	Diptera	Culicidae	Aedes	Aedes aegypti	SPECIES	32.85370	-97.14130	3030190487
15412	Diptera	Culicidae	Aedes	Aedes aegypti	SPECIES	32.85510	-97.16000	3030193558
15413	Diptera	Culicidae	Aedes	Aedes aegypti	SPECIES	32.85530	-97.12100	3030258804
15414	Diptera	Culicidae	Aedes	Aedes aegypti	SPECIES	32.85560	-97.21500	3030606219
15415	Diptera	Culicidae	Aedes	Aedes aegypti	SPECIES	32.85600	-97.17520	3030724546
15416	Diptera	Culicidae	Aedes	Aedes aegypti	SPECIES	32.85610	-97.13170	3030229461

№	Order	Family	Genus	Species	Taxon Rank	Latitude	Longitude	GBIF_ID
15417	Diptera	Culicidae	Aedes	Aedes aegypti	SPECIES	32.85690	-97.36430	3030585428
15418	Diptera	Culicidae	Aedes	Aedes aegypti	SPECIES	32.85698	-96.68576	4009887629
15419	Diptera	Culicidae	Aedes	Aedes aegypti	SPECIES	32.85702	-96.68577	1802731564
15420	Diptera	Culicidae	Aedes	Aedes aegypti	SPECIES	32.85760	-97.22990	3030748399
15421	Diptera	Culicidae	Aedes	Aedes aegypti	SPECIES	32.85770	-97.27160	3030716329
15422	Diptera	Culicidae	Aedes	Aedes aegypti	SPECIES	32.85780	-97.27140	3030746953
15423	Diptera	Culicidae	Aedes	Aedes aegypti	SPECIES	32.85790	-97.23170	3030745898
15424	Diptera	Culicidae	Aedes	Aedes aegypti	SPECIES	32.85810	-97.28980	3030218450
15425	Diptera	Culicidae	Aedes	Aedes aegypti	SPECIES	32.85810	-97.12460	3030631943
15426	Diptera	Culicidae	Aedes	Aedes aegypti	SPECIES	32.85840	-97.17670	3030645558
15427	Diptera	Culicidae	Aedes	Aedes aegypti	SPECIES	32.85860	-97.17600	3030185153
15428	Diptera	Culicidae	Aedes	Aedes aegypti	SPECIES	32.85870	-97.38020	3030672503
15429	Diptera	Culicidae	Aedes	Aedes aegypti	SPECIES	32.85900	-97.17590	3030757480
15430	Diptera	Culicidae	Aedes	Aedes aegypti	SPECIES	32.85930	-97.12960	3030190373
15431	Diptera	Culicidae	Aedes	Aedes aegypti	SPECIES	32.85940	-97.11910	3030281563
15432	Diptera	Culicidae	Aedes	Aedes aegypti	SPECIES	32.85990	-97.06600	3030186050
15433	Diptera	Culicidae	Aedes	Aedes aegypti	SPECIES	32.85990	-97.11090	3030210874
15434	Diptera	Culicidae	Aedes	Aedes aegypti	SPECIES	32.86050	-97.14260	3030191465
15435	Diptera	Culicidae	Aedes	Aedes aegypti	SPECIES	32.86150	-97.13470	3030752999
15436	Diptera	Culicidae	Aedes	Aedes aegypti	SPECIES	32.86210	-97.15070	3030757339
15437	Diptera	Culicidae	Aedes	Aedes aegypti	SPECIES	32.86330	-97.11500	3030300947
15438	Diptera	Culicidae	Aedes	Aedes aegypti	SPECIES	32.86370	-97.16460	3030260868
15439	Diptera	Culicidae	Aedes	Aedes aegypti	SPECIES	32.86380	-97.08780	3030755443
15440	Diptera	Culicidae	Aedes	Aedes aegypti	SPECIES	32.86400	-97.16390	3030688123
15441	Diptera	Culicidae	Aedes	Aedes aegypti	SPECIES	32.86440	-97.26550	3030758342
15442	Diptera	Culicidae	Aedes	Aedes aegypti	SPECIES	32.86560	-97.16380	3030703060
15443	Diptera	Culicidae	Aedes	Aedes aegypti	SPECIES	32.86590	-97.18040	3030632047
15444	Diptera	Culicidae	Aedes	Aedes aegypti	SPECIES	32.86703	-97.27596	3079523450
15445	Diptera	Culicidae	Aedes	Aedes aegypti	SPECIES	32.86720	-97.29250	3030631087
15446	Diptera	Culicidae	Aedes	Aedes aegypti	SPECIES	32.86740	-97.13670	3030755329
15447	Diptera	Culicidae	Aedes	Aedes aegypti	SPECIES	32.86770	-97.35700	3030576096
15448	Diptera	Culicidae	Aedes	Aedes aegypti	SPECIES	32.86770	-97.21840	3030665486
15449	Diptera	Culicidae	Aedes	Aedes aegypti	SPECIES	32.86770	-97.26720	3030749214
15450	Diptera	Culicidae	Aedes	Aedes aegypti	SPECIES	32.86870	-97.51890	3030688138
15451	Diptera	Culicidae	Aedes	Aedes aegypti	SPECIES	32.86890	-97.13820	3030640407
15452	Diptera	Culicidae	Aedes	Aedes aegypti	SPECIES	32.86890	-97.52020	3030685266
15453	Diptera	Culicidae	Aedes	Aedes aegypti	SPECIES	32.86890	-97.20410	3030749056
15454	Diptera	Culicidae	Aedes	Aedes aegypti	SPECIES	32.86910	-97.38540	3030607387
15455	Diptera	Culicidae	Aedes	Aedes aegypti	SPECIES	32.86930	-97.13880	3030746334
15456	Diptera	Culicidae	Aedes	Aedes aegypti	SPECIES	32.86980	-97.13890	3030684931
15457	Diptera	Culicidae	Aedes	Aedes aegypti	SPECIES	32.86980	-97.28014	1913681945

№	Order	Family	Genus	Species	Taxon Rank	Latitude	Longitude	GBIF_ID
15458	Diptera	Culicidae	Aedes	Aedes aegypti	SPECIES	32.87040	-97.16920	3030583273
15459	Diptera	Culicidae	Aedes	Aedes aegypti	SPECIES	32.87040	-97.11500	3030694385
15460	Diptera	Culicidae	Aedes	Aedes aegypti	SPECIES	32.87070	-97.17700	3030747355
15461	Diptera	Culicidae	Aedes	Aedes aegypti	SPECIES	32.87120	-97.17360	3030576212
15462	Diptera	Culicidae	Aedes	Aedes aegypti	SPECIES	32.87150	-97.15190	3030640307
15463	Diptera	Culicidae	Aedes	Aedes aegypti	SPECIES	32.87162	-96.64594	1949983383
15464	Diptera	Culicidae	Aedes	Aedes aegypti	SPECIES	32.87174	-96.64588	2641518308
15465	Diptera	Culicidae	Aedes	Aedes aegypti	SPECIES	32.87360	-97.37970	3030297350
15466	Diptera	Culicidae	Aedes	Aedes aegypti	SPECIES	32.87460	-97.18040	3030616446
15467	Diptera	Culicidae	Aedes	Aedes aegypti	SPECIES	32.87470	-97.37850	3030617379
15468	Diptera	Culicidae	Aedes	Aedes aegypti	SPECIES	32.87480	-97.05920	3030728600
15469	Diptera	Culicidae	Aedes	Aedes aegypti	SPECIES	32.87520	-97.08290	3030758501
15470	Diptera	Culicidae	Aedes	Aedes aegypti	SPECIES	32.87530	-97.13730	3030686719
15471	Diptera	Culicidae	Aedes	Aedes aegypti	SPECIES	32.87540	-97.17410	3030684333
15472	Diptera	Culicidae	Aedes	Aedes aegypti	SPECIES	32.87637	-96.70337	3912113853
15473	Diptera	Culicidae	Aedes	Aedes aegypti	SPECIES	32.87740	-97.19110	3030639301
15474	Diptera	Culicidae	Aedes	Aedes aegypti	SPECIES	32.87760	-97.18450	3030289273
15475	Diptera	Culicidae	Aedes	Aedes aegypti	SPECIES	32.87780	-97.20650	3030218766
15476	Diptera	Culicidae	Aedes	Aedes aegypti	SPECIES	32.87820	-97.27620	3030618475
15477	Diptera	Culicidae	Aedes	Aedes aegypti	SPECIES	32.87835	-97.53551	3947464986
15478	Diptera	Culicidae	Aedes	Aedes aegypti	SPECIES	32.87850	-97.20320	3030695374
15479	Diptera	Culicidae	Aedes	Aedes aegypti	SPECIES	32.87860	-97.27640	3030727525
15480	Diptera	Culicidae	Aedes	Aedes aegypti	SPECIES	32.87890	-97.42680	3030632954
15481	Diptera	Culicidae	Aedes	Aedes aegypti	SPECIES	32.87930	-97.52790	3030688750
15482	Diptera	Culicidae	Aedes	Aedes aegypti	SPECIES	32.87930	-97.17960	3030698533
15483	Diptera	Culicidae	Aedes	Aedes aegypti	SPECIES	32.87970	-97.11580	3030696972
15484	Diptera	Culicidae	Aedes	Aedes aegypti	SPECIES	32.87970	-97.17940	3030758316
15485	Diptera	Culicidae	Aedes	Aedes aegypti	SPECIES	32.88070	-97.06010	3030632221
15486	Diptera	Culicidae	Aedes	Aedes aegypti	SPECIES	32.88090	-97.22880	3030585988
15487	Diptera	Culicidae	Aedes	Aedes aegypti	SPECIES	32.88110	-97.35180	3030745863
15488	Diptera	Culicidae	Aedes	Aedes aegypti	SPECIES	32.88130	-97.23110	3030697370
15489	Diptera	Culicidae	Aedes	Aedes aegypti	SPECIES	32.88160	-97.34990	3030746332
15490	Diptera	Culicidae	Aedes	Aedes aegypti	SPECIES	32.88180	-97.12720	3030695453
15491	Diptera	Culicidae	Aedes	Aedes aegypti	SPECIES	32.88230	-97.18400	3030648171
15492	Diptera	Culicidae	Aedes	Aedes aegypti	SPECIES	32.88230	-97.20310	3030686110
15493	Diptera	Culicidae	Aedes	Aedes aegypti	SPECIES	32.88340	-97.53700	3030632573
15494	Diptera	Culicidae	Aedes	Aedes aegypti	SPECIES	32.88360	-97.53710	3030745787
15495	Diptera	Culicidae	Aedes	Aedes aegypti	SPECIES	32.88370	-97.11010	3030299400
15496	Diptera	Culicidae	Aedes	Aedes aegypti	SPECIES	32.88420	-97.17570	3030644924
15497	Diptera	Culicidae	Aedes	Aedes aegypti	SPECIES	32.88500	-97.15370	3030756305
15498	Diptera	Culicidae	Aedes	Aedes aegypti	SPECIES	32.88580	-97.01520	3030289078

№	Order	Family	Genus	Species	Taxon Rank	Latitude	Longitude	GBIF_ID
15499	Diptera	Culicidae	Aedes	Aedes aegypti	SPECIES	32.88580	-97.34170	3030643321
15500	Diptera	Culicidae	Aedes	Aedes aegypti	SPECIES	32.88590	-97.16140	3030724512
15501	Diptera	Culicidae	Aedes	Aedes aegypti	SPECIES	32.88636	-97.28145	3923359629
15502	Diptera	Culicidae	Aedes	Aedes aegypti	SPECIES	32.88740	-97.35270	3030581163
15503	Diptera	Culicidae	Aedes	Aedes aegypti	SPECIES	32.88760	-97.21810	3030755478
15504	Diptera	Culicidae	Aedes	Aedes aegypti	SPECIES	32.88790	-97.22890	3030746984
15505	Diptera	Culicidae	Aedes	Aedes aegypti	SPECIES	32.88850	-97.09690	3030223710
15506	Diptera	Culicidae	Aedes	Aedes aegypti	SPECIES	32.88880	-97.45940	3030205549
15507	Diptera	Culicidae	Aedes	Aedes aegypti	SPECIES	32.88930	-97.23790	3030757538
15508	Diptera	Culicidae	Aedes	Aedes aegypti	SPECIES	32.89010	-97.20340	3030216438
15509	Diptera	Culicidae	Aedes	Aedes aegypti	SPECIES	32.89020	-97.20350	3030696491
15510	Diptera	Culicidae	Aedes	Aedes aegypti	SPECIES	32.89020	-97.20340	3030757506
15511	Diptera	Culicidae	Aedes	Aedes aegypti	SPECIES	32.89030	-97.26560	3030642407
15512	Diptera	Culicidae	Aedes	Aedes aegypti	SPECIES	32.89040	-97.26560	3030642427
15513	Diptera	Culicidae	Aedes	Aedes aegypti	SPECIES	32.89054	-96.80564	2429493289
15514	Diptera	Culicidae	Aedes	Aedes aegypti	SPECIES	32.89090	-97.23180	3030630023
15515	Diptera	Culicidae	Aedes	Aedes aegypti	SPECIES	32.89120	-97.09290	3030716822
15516	Diptera	Culicidae	Aedes	Aedes aegypti	SPECIES	32.89130	-97.17230	3030746892
15517	Diptera	Culicidae	Aedes	Aedes aegypti	SPECIES	32.89130	-97.09160	3030756965
15518	Diptera	Culicidae	Aedes	Aedes aegypti	SPECIES	32.89170	-97.14270	3030758512
15519	Diptera	Culicidae	Aedes	Aedes aegypti	SPECIES	32.89240	-97.14290	3030758339
15520	Diptera	Culicidae	Aedes	Aedes aegypti	SPECIES	32.89360	-97.21580	3030666566
15521	Diptera	Culicidae	Aedes	Aedes aegypti	SPECIES	32.89410	-97.10790	3030747773
15522	Diptera	Culicidae	Aedes	Aedes aegypti	SPECIES	32.89430	-97.54600	3030662547
15523	Diptera	Culicidae	Aedes	Aedes aegypti	SPECIES	32.89480	-97.53780	3030652074
15524	Diptera	Culicidae	Aedes	Aedes aegypti	SPECIES	32.89650	-97.20050	3030180202
15525	Diptera	Culicidae	Aedes	Aedes aegypti	SPECIES	32.89930	-97.10500	3030618412
15526	Diptera	Culicidae	Aedes	Aedes aegypti	SPECIES	32.89960	-97.10170	3030671543
15527	Diptera	Culicidae	Aedes	Aedes aegypti	SPECIES	32.89980	-97.27670	3030667521
15528	Diptera	Culicidae	Aedes	Aedes aegypti	SPECIES	32.90000	-80.05000	1264889311
15529	Diptera	Culicidae	Aedes	Aedes aegypti	SPECIES	32.90000	-97.17000	3030746176
15530	Diptera	Culicidae	Aedes	Aedes aegypti	SPECIES	32.90050	-97.13000	3030695405
15531	Diptera	Culicidae	Aedes	Aedes aegypti	SPECIES	32.90080	-97.44420	3030747350
15532	Diptera	Culicidae	Aedes	Aedes aegypti	SPECIES	32.90090	-97.44510	3030755436
15533	Diptera	Culicidae	Aedes	Aedes aegypti	SPECIES	32.90130	-97.20640	3030747047
15534	Diptera	Culicidae	Aedes	Aedes aegypti	SPECIES	32.90159	-96.74344	3355376403
15535	Diptera	Culicidae	Aedes	Aedes aegypti	SPECIES	32.90200	-97.15690	3030685756
15536	Diptera	Culicidae	Aedes	Aedes aegypti	SPECIES	32.90230	-97.29250	3030639490
15537	Diptera	Culicidae	Aedes	Aedes aegypti	SPECIES	32.90330	-97.21100	3030219691
15538	Diptera	Culicidae	Aedes	Aedes aegypti	SPECIES	32.90340	-97.16690	3030647608
15539	Diptera	Culicidae	Aedes	Aedes aegypti	SPECIES	32.90400	-97.15750	3030582485

№	Order	Family	Genus	Species	Taxon Rank	Latitude	Longitude	GBIF_ID
15540	Diptera	Culicidae	Aedes	Aedes aegypti	SPECIES	32.90450	-97.17050	3030670412
15541	Diptera	Culicidae	Aedes	Aedes aegypti	SPECIES	32.90530	-97.17290	3030671769
15542	Diptera	Culicidae	Aedes	Aedes aegypti	SPECIES	32.90648	-117.11253	3889033811
15543	Diptera	Culicidae	Aedes	Aedes aegypti	SPECIES	32.90710	-97.14430	3030663359
15544	Diptera	Culicidae	Aedes	Aedes aegypti	SPECIES	32.90760	-97.22430	3030716912
15545	Diptera	Culicidae	Aedes	Aedes aegypti	SPECIES	32.90790	-97.19070	3030300008
15546	Diptera	Culicidae	Aedes	Aedes aegypti	SPECIES	32.90830	-97.14310	3030630983
15547	Diptera	Culicidae	Aedes	Aedes aegypti	SPECIES	32.90930	-97.12860	3030656791
15548	Diptera	Culicidae	Aedes	Aedes aegypti	SPECIES	32.91000	-111.35000	1264871637
15549	Diptera	Culicidae	Aedes	Aedes aegypti	SPECIES	32.91030	-97.11320	3030752370
15550	Diptera	Culicidae	Aedes	Aedes aegypti	SPECIES	32.91100	-97.53850	3030673702
15551	Diptera	Culicidae	Aedes	Aedes aegypti	SPECIES	32.91430	-97.54980	3030630075
15552	Diptera	Culicidae	Aedes	Aedes aegypti	SPECIES	32.91690	-97.14010	3030273405
15553	Diptera	Culicidae	Aedes	Aedes aegypti	SPECIES	32.91730	-97.18810	3030747958
15554	Diptera	Culicidae	Aedes	Aedes aegypti	SPECIES	32.91800	-97.18830	3030752971
15555	Diptera	Culicidae	Aedes	Aedes aegypti	SPECIES	32.91820	-97.16230	3030716798
15556	Diptera	Culicidae	Aedes	Aedes aegypti	SPECIES	32.91840	-97.16100	3030671679
15557	Diptera	Culicidae	Aedes	Aedes aegypti	SPECIES	32.91870	-97.03520	3030641545
15558	Diptera	Culicidae	Aedes	Aedes aegypti	SPECIES	32.91880	-97.05820	3030584328
15559	Diptera	Culicidae	Aedes	Aedes aegypti	SPECIES	32.91980	-97.26640	3030758347
15560	Diptera	Culicidae	Aedes	Aedes aegypti	SPECIES	32.91990	-97.26610	3030686319
15561	Diptera	Culicidae	Aedes	Aedes aegypti	SPECIES	32.92010	-97.14090	3030596587
15562	Diptera	Culicidae	Aedes	Aedes aegypti	SPECIES	32.92150	-97.13980	3030652508
15563	Diptera	Culicidae	Aedes	Aedes aegypti	SPECIES	32.92160	-97.54940	3030695400
15564	Diptera	Culicidae	Aedes	Aedes aegypti	SPECIES	32.92230	-97.52730	3030748171
15565	Diptera	Culicidae	Aedes	Aedes aegypti	SPECIES	32.92280	-97.13560	3030633052
15566	Diptera	Culicidae	Aedes	Aedes aegypti	SPECIES	32.92300	-97.14060	3030608760
15567	Diptera	Culicidae	Aedes	Aedes aegypti	SPECIES	32.92320	-97.15690	3030295512
15568	Diptera	Culicidae	Aedes	Aedes aegypti	SPECIES	32.92370	-97.13610	3030216986
15569	Diptera	Culicidae	Aedes	Aedes aegypti	SPECIES	32.92390	-97.22070	3030688323
15570	Diptera	Culicidae	Aedes	Aedes aegypti	SPECIES	32.92410	-97.28330	3030668483
15571	Diptera	Culicidae	Aedes	Aedes aegypti	SPECIES	32.92440	-97.14130	3030756348
15572	Diptera	Culicidae	Aedes	Aedes aegypti	SPECIES	32.92460	-97.12240	3030300989
15573	Diptera	Culicidae	Aedes	Aedes aegypti	SPECIES	32.92460	-97.10680	3030701009
15574	Diptera	Culicidae	Aedes	Aedes aegypti	SPECIES	32.92471	-96.70831	3384256544
15575	Diptera	Culicidae	Aedes	Aedes aegypti	SPECIES	32.92620	-97.25700	3030756441
15576	Diptera	Culicidae	Aedes	Aedes aegypti	SPECIES	32.92630	-97.14000	3030188024
15577	Diptera	Culicidae	Aedes	Aedes aegypti	SPECIES	32.92650	-97.16210	3030285794
15578	Diptera	Culicidae	Aedes	Aedes aegypti	SPECIES	32.92730	-97.12050	3030671457
15579	Diptera	Culicidae	Aedes	Aedes aegypti	SPECIES	32.92820	-97.19770	3030631079
15580	Diptera	Culicidae	Aedes	Aedes aegypti	SPECIES	32.92850	-97.16240	3030184943

№	Order	Family	Genus	Species	Taxon Rank	Latitude	Longitude	GBIF_ID
15581	Diptera	Culicidae	Aedes	Aedes aegypti	SPECIES	32.92850	-97.16060	3030756987
15582	Diptera	Culicidae	Aedes	Aedes aegypti	SPECIES	32.92860	-97.16470	3030301028
15583	Diptera	Culicidae	Aedes	Aedes aegypti	SPECIES	32.92940	-97.15110	3030755306
15584	Diptera	Culicidae	Aedes	Aedes aegypti	SPECIES	32.93020	-97.16110	3030263785
15585	Diptera	Culicidae	Aedes	Aedes aegypti	SPECIES	32.93040	-97.14900	3030618526
15586	Diptera	Culicidae	Aedes	Aedes aegypti	SPECIES	32.93080	-97.15050	3030686050
15587	Diptera	Culicidae	Aedes	Aedes aegypti	SPECIES	32.93090	-97.17040	3030597844
15588	Diptera	Culicidae	Aedes	Aedes aegypti	SPECIES	32.93090	-97.16610	3030669652
15589	Diptera	Culicidae	Aedes	Aedes aegypti	SPECIES	32.93150	-97.15040	3030688964
15590	Diptera	Culicidae	Aedes	Aedes aegypti	SPECIES	32.93180	-97.40210	3030688324
15591	Diptera	Culicidae	Aedes	Aedes aegypti	SPECIES	32.93200	-97.40230	3030748791
15592	Diptera	Culicidae	Aedes	Aedes aegypti	SPECIES	32.93220	-97.08040	3030758469
15593	Diptera	Culicidae	Aedes	Aedes aegypti	SPECIES	32.93250	-97.08050	3030748802
15594	Diptera	Culicidae	Aedes	Aedes aegypti	SPECIES	32.93260	-97.19570	3030641319
15595	Diptera	Culicidae	Aedes	Aedes aegypti	SPECIES	32.93310	-97.15980	3030648648
15596	Diptera	Culicidae	Aedes	Aedes aegypti	SPECIES	32.93330	-97.14140	3030222355
15597	Diptera	Culicidae	Aedes	Aedes aegypti	SPECIES	32.93340	-97.14200	3030756992
15598	Diptera	Culicidae	Aedes	Aedes aegypti	SPECIES	32.93390	-97.14460	3030576214
15599	Diptera	Culicidae	Aedes	Aedes aegypti	SPECIES	32.93440	-97.12890	3030247198
15600	Diptera	Culicidae	Aedes	Aedes aegypti	SPECIES	32.93443	-117.06128	4009715601
15601	Diptera	Culicidae	Aedes	Aedes aegypti	SPECIES	32.93450	-97.13570	3030197573
15602	Diptera	Culicidae	Aedes	Aedes aegypti	SPECIES	32.93520	-97.46430	3030601661
15603	Diptera	Culicidae	Aedes	Aedes aegypti	SPECIES	32.93520	-97.46620	3030669383
15604	Diptera	Culicidae	Aedes	Aedes aegypti	SPECIES	32.93530	-97.46580	3030644992
15605	Diptera	Culicidae	Aedes	Aedes aegypti	SPECIES	32.93570	-97.18640	3030712877
15606	Diptera	Culicidae	Aedes	Aedes aegypti	SPECIES	32.93580	-97.21860	3030697412
15607	Diptera	Culicidae	Aedes	Aedes aegypti	SPECIES	32.93630	-97.11000	3030617670
15608	Diptera	Culicidae	Aedes	Aedes aegypti	SPECIES	32.93640	-97.17210	3030261011
15609	Diptera	Culicidae	Aedes	Aedes aegypti	SPECIES	32.93640	-97.18770	3030685924
15610	Diptera	Culicidae	Aedes	Aedes aegypti	SPECIES	32.93650	-97.39190	3030685321
15611	Diptera	Culicidae	Aedes	Aedes aegypti	SPECIES	32.93660	-97.13320	3030295049
15612	Diptera	Culicidae	Aedes	Aedes aegypti	SPECIES	32.93840	-97.14590	3030654307
15613	Diptera	Culicidae	Aedes	Aedes aegypti	SPECIES	32.93850	-97.18810	3030294453
15614	Diptera	Culicidae	Aedes	Aedes aegypti	SPECIES	32.94040	-97.52610	3030632036
15615	Diptera	Culicidae	Aedes	Aedes aegypti	SPECIES	32.94060	-97.52400	3030758581
15616	Diptera	Culicidae	Aedes	Aedes aegypti	SPECIES	32.94110	-97.09530	3030701583
15617	Diptera	Culicidae	Aedes	Aedes aegypti	SPECIES	32.94110	-97.09500	3030748820
15618	Diptera	Culicidae	Aedes	Aedes aegypti	SPECIES	32.94122	-96.71718	3398713281
15619	Diptera	Culicidae	Aedes	Aedes aegypti	SPECIES	32.94150	-97.19760	3030653768
15620	Diptera	Culicidae	Aedes	Aedes aegypti	SPECIES	32.94160	-97.18510	3030186008
15621	Diptera	Culicidae	Aedes	Aedes aegypti	SPECIES	32.94190	-97.18240	3030752449

№	Order	Family	Genus	Species	Taxon Rank	Latitude	Longitude	GBIF_ID
15622	Diptera	Culicidae	Aedes	Aedes aegypti	SPECIES	32.94260	-97.13120	3030752386
15623	Diptera	Culicidae	Aedes	Aedes aegypti	SPECIES	32.94410	-97.15320	3030694431
15624	Diptera	Culicidae	Aedes	Aedes aegypti	SPECIES	32.94420	-97.07010	3030551785
15625	Diptera	Culicidae	Aedes	Aedes aegypti	SPECIES	32.94540	-97.42090	3030631593
15626	Diptera	Culicidae	Aedes	Aedes aegypti	SPECIES	32.94540	-97.49450	3030633240
15627	Diptera	Culicidae	Aedes	Aedes aegypti	SPECIES	32.94550	-97.42100	3030758356
15628	Diptera	Culicidae	Aedes	Aedes aegypti	SPECIES	32.94560	-97.49430	3030285972
15629	Diptera	Culicidae	Aedes	Aedes aegypti	SPECIES	32.94560	-97.15010	3030712331
15630	Diptera	Culicidae	Aedes	Aedes aegypti	SPECIES	32.94640	-97.08080	3030660309
15631	Diptera	Culicidae	Aedes	Aedes aegypti	SPECIES	32.94650	-97.19620	3030170126
15632	Diptera	Culicidae	Aedes	Aedes aegypti	SPECIES	32.94660	-97.34710	3030287171
15633	Diptera	Culicidae	Aedes	Aedes aegypti	SPECIES	32.94670	-97.29740	3030220132
15634	Diptera	Culicidae	Aedes	Aedes aegypti	SPECIES	32.94700	-97.08100	3030669542
15635	Diptera	Culicidae	Aedes	Aedes aegypti	SPECIES	32.94700	-97.09940	3030748157
15636	Diptera	Culicidae	Aedes	Aedes aegypti	SPECIES	32.94740	-97.13220	3030185125
15637	Diptera	Culicidae	Aedes	Aedes aegypti	SPECIES	32.94740	-97.13530	3030301048
15638	Diptera	Culicidae	Aedes	Aedes aegypti	SPECIES	32.94750	-97.09070	3030686003
15639	Diptera	Culicidae	Aedes	Aedes aegypti	SPECIES	32.94780	-97.18130	3030221422
15640	Diptera	Culicidae	Aedes	Aedes aegypti	SPECIES	32.94880	-97.19540	3030314036
15641	Diptera	Culicidae	Aedes	Aedes aegypti	SPECIES	32.94970	-97.16880	3030263045
15642	Diptera	Culicidae	Aedes	Aedes aegypti	SPECIES	32.95140	-97.13670	3030654189
15643	Diptera	Culicidae	Aedes	Aedes aegypti	SPECIES	32.95200	-97.11370	3030700880
15644	Diptera	Culicidae	Aedes	Aedes aegypti	SPECIES	32.95250	-97.16220	3030686008
15645	Diptera	Culicidae	Aedes	Aedes aegypti	SPECIES	32.95260	-97.16780	3030264006
15646	Diptera	Culicidae	Aedes	Aedes aegypti	SPECIES	32.95278	-97.16789	3079485943
15647	Diptera	Culicidae	Aedes	Aedes aegypti	SPECIES	32.95330	-97.14900	3030711572
15648	Diptera	Culicidae	Aedes	Aedes aegypti	SPECIES	32.95390	-97.16830	3030303103
15649	Diptera	Culicidae	Aedes	Aedes aegypti	SPECIES	32.95520	-97.17360	3030303069
15650	Diptera	Culicidae	Aedes	Aedes aegypti	SPECIES	32.95550	-97.07380	3030743119
15651	Diptera	Culicidae	Aedes	Aedes aegypti	SPECIES	32.95570	-97.09830	3030656376
15652	Diptera	Culicidae	Aedes	Aedes aegypti	SPECIES	32.95580	-97.09750	3030231637
15653	Diptera	Culicidae	Aedes	Aedes aegypti	SPECIES	32.95600	-97.10890	3030660645
15654	Diptera	Culicidae	Aedes	Aedes aegypti	SPECIES	32.95610	-97.15970	3030188240
15655	Diptera	Culicidae	Aedes	Aedes aegypti	SPECIES	32.95610	-97.15660	3030685003
15656	Diptera	Culicidae	Aedes	Aedes aegypti	SPECIES	32.95790	-97.09080	3030208333
15657	Diptera	Culicidae	Aedes	Aedes aegypti	SPECIES	32.95870	-97.17600	3030184779
15658	Diptera	Culicidae	Aedes	Aedes aegypti	SPECIES	32.95870	-97.15970	3030757986
15659	Diptera	Culicidae	Aedes	Aedes aegypti	SPECIES	32.95980	-97.09270	3030616613
15660	Diptera	Culicidae	Aedes	Aedes aegypti	SPECIES	32.96050	-97.13400	3030632908
15661	Diptera	Culicidae	Aedes	Aedes aegypti	SPECIES	32.96120	-97.16870	3030211466
15662	Diptera	Culicidae	Aedes	Aedes aegypti	SPECIES	32.96180	-97.11860	3030287064

№	Order	Family	Genus	Species	Taxon Rank	Latitude	Longitude	GBIF_ID
15663	Diptera	Culicidae	Aedes	Aedes aegypti	SPECIES	32.96280	-97.52530	3030698521
15664	Diptera	Culicidae	Aedes	Aedes aegypti	SPECIES	32.96290	-97.14160	3030687877
15665	Diptera	Culicidae	Aedes	Aedes aegypti	SPECIES	32.96300	-97.52530	3030574979
15666	Diptera	Culicidae	Aedes	Aedes aegypti	SPECIES	32.96310	-97.12040	3030617530
15667	Diptera	Culicidae	Aedes	Aedes aegypti	SPECIES	32.96370	-97.17590	3030757385
15668	Diptera	Culicidae	Aedes	Aedes aegypti	SPECIES	32.96410	-97.17080	3030587386
15669	Diptera	Culicidae	Aedes	Aedes aegypti	SPECIES	32.96470	-97.27910	3030580440
15670	Diptera	Culicidae	Aedes	Aedes aegypti	SPECIES	32.96520	-97.04570	3030672415
15671	Diptera	Culicidae	Aedes	Aedes aegypti	SPECIES	32.96540	-97.12490	3030229656
15672	Diptera	Culicidae	Aedes	Aedes aegypti	SPECIES	32.96680	-97.17780	3030581072
15673	Diptera	Culicidae	Aedes	Aedes aegypti	SPECIES	32.96720	-97.21630	3030618670
15674	Diptera	Culicidae	Aedes	Aedes aegypti	SPECIES	32.96720	-97.21250	3030698012
15675	Diptera	Culicidae	Aedes	Aedes aegypti	SPECIES	32.96760	-97.03660	3030747763
15676	Diptera	Culicidae	Aedes	Aedes aegypti	SPECIES	32.96790	-97.03660	3030219588
15677	Diptera	Culicidae	Aedes	Aedes aegypti	SPECIES	32.96870	-97.12740	3030287233
15678	Diptera	Culicidae	Aedes	Aedes aegypti	SPECIES	32.97020	-97.16000	3030634150
15679	Diptera	Culicidae	Aedes	Aedes aegypti	SPECIES	32.97100	-97.10360	3030742329
15680	Diptera	Culicidae	Aedes	Aedes aegypti	SPECIES	32.97140	-97.26230	3030643443
15681	Diptera	Culicidae	Aedes	Aedes aegypti	SPECIES	32.97200	-97.34820	3030629859
15682	Diptera	Culicidae	Aedes	Aedes aegypti	SPECIES	32.97250	-97.14060	3030711608
15683	Diptera	Culicidae	Aedes	Aedes aegypti	SPECIES	32.97300	-97.14340	3030576043
15684	Diptera	Culicidae	Aedes	Aedes aegypti	SPECIES	32.97410	-97.35050	3030629520
15685	Diptera	Culicidae	Aedes	Aedes aegypti	SPECIES	32.97450	-97.34830	3030217413
15686	Diptera	Culicidae	Aedes	Aedes aegypti	SPECIES	32.97512	-115.54208	1703143219
15687	Diptera	Culicidae	Aedes	Aedes aegypti	SPECIES	32.97540	-97.13960	3030673643
15688	Diptera	Culicidae	Aedes	Aedes aegypti	SPECIES	32.97620	-97.13990	3030210858
15689	Diptera	Culicidae	Aedes	Aedes aegypti	SPECIES	32.97640	-97.41370	3030755483
15690	Diptera	Culicidae	Aedes	Aedes aegypti	SPECIES	32.97800	-97.41400	3030640399
15691	Diptera	Culicidae	Aedes	Aedes aegypti	SPECIES	32.98120	-97.02760	3030667507
15692	Diptera	Culicidae	Aedes	Aedes aegypti	SPECIES	32.98160	-97.13860	3030606463
15693	Diptera	Culicidae	Aedes	Aedes aegypti	SPECIES	32.98210	-97.15490	3030632120
15694	Diptera	Culicidae	Aedes	Aedes aegypti	SPECIES	32.98250	-97.11610	3030698301
15695	Diptera	Culicidae	Aedes	Aedes aegypti	SPECIES	32.98250	-97.11620	3030727515
15696	Diptera	Culicidae	Aedes	Aedes aegypti	SPECIES	32.98270	-97.31040	3030581071
15697	Diptera	Culicidae	Aedes	Aedes aegypti	SPECIES	32.98300	-97.12210	3030200257
15698	Diptera	Culicidae	Aedes	Aedes aegypti	SPECIES	32.98330	-97.14710	3030759057
15699	Diptera	Culicidae	Aedes	Aedes aegypti	SPECIES	32.98430	-97.38560	3030643301
15700	Diptera	Culicidae	Aedes	Aedes aegypti	SPECIES	32.99760	-97.14760	3030684321
15701	Diptera	Culicidae	Aedes	Aedes aegypti	SPECIES	32.99790	-97.14860	3030188245
15702	Diptera	Culicidae	Aedes	Aedes aegypti	SPECIES	32.99820	-97.15690	3030584538
15703	Diptera	Culicidae	Aedes	Aedes aegypti	SPECIES	33.01554	-117.05368	3407902517

№	Order	Family	Genus	Species	Taxon Rank	Latitude	Longitude	GBIF_ID
15704	Diptera	Culicidae	Aedes	Aedes aegypti	SPECIES	33.01556	-116.80315	3961070603
15705	Diptera	Culicidae	Aedes	Aedes aegypti	SPECIES	33.03000	73.60000	3350315661
15706	Diptera	Culicidae	Aedes	Aedes aegypti	SPECIES	33.03000	73.59000	3350315662
15707	Diptera	Culicidae	Aedes	Aedes aegypti	SPECIES	33.03000	73.61000	3350364546
15708	Diptera	Culicidae	Aedes	Aedes aegypti	SPECIES	33.03190	-97.32320	3030748367
15709	Diptera	Culicidae	Aedes	Aedes aegypti	SPECIES	33.03532	-96.72803	4153747628
15710	Diptera	Culicidae	Aedes	Aedes aegypti	SPECIES	33.03536	-96.72633	3330482501
15711	Diptera	Culicidae	Aedes	Aedes aegypti	SPECIES	33.04336	-97.17875	3314513354
15712	Diptera	Culicidae	Aedes	Aedes aegypti	SPECIES	33.05110	-96.79826	2626599075
15713	Diptera	Culicidae	Aedes	Aedes aegypti	SPECIES	33.09302	-96.99362	2626563357
15714	Diptera	Culicidae	Aedes	Aedes aegypti	SPECIES	33.12508	-96.87820	4153424695
15715	Diptera	Culicidae	Aedes	Aedes aegypti	SPECIES	33.14681	-97.09587	2898398747
15716	Diptera	Culicidae	Aedes	Aedes aegypti	SPECIES	33.14689	-97.09592	2984534927
15717	Diptera	Culicidae	Aedes	Aedes aegypti	SPECIES	33.15055	-96.99894	2633968670
15718	Diptera	Culicidae	Aedes	Aedes aegypti	SPECIES	33.15983	-96.61491	1978870386
15719	Diptera	Culicidae	Aedes	Aedes aegypti	SPECIES	33.17179	-96.70316	4165478700
15720	Diptera	Culicidae	Aedes	Aedes aegypti	SPECIES	33.19000	-96.56000	1264871691
15721	Diptera	Culicidae	Aedes	Aedes aegypti	SPECIES	33.20488	-111.86434	2803087155
15722	Diptera	Culicidae	Aedes	Aedes aegypti	SPECIES	33.21000	-97.11000	1264871688
15723	Diptera	Culicidae	Aedes	Aedes aegypti	SPECIES	33.21441	-97.15134	3912115701
15724	Diptera	Culicidae	Aedes	Aedes aegypti	SPECIES	33.22000	-87.58000	1264888953
15725	Diptera	Culicidae	Aedes	Aedes aegypti	SPECIES	33.22443	-96.94097	2864576655
15726	Diptera	Culicidae	Aedes	Aedes aegypti	SPECIES	33.30136	-111.77813	1914202485
15727	Diptera	Culicidae	Aedes	Aedes aegypti	SPECIES	33.31999	-79.32001	4076241201
15728	Diptera	Culicidae	Aedes	Aedes aegypti	SPECIES	33.33083	-97.58652	3059752296
15729	Diptera	Culicidae	Aedes	Aedes aegypti	SPECIES	33.36000	-112.49000	1264871674
15730	Diptera	Culicidae	Aedes	Aedes aegypti	SPECIES	33.38973	-117.21881	3335386158
15731	Diptera	Culicidae	Aedes	Aedes aegypti	SPECIES	33.39433	-97.65418	4129347080
15732	Diptera	Culicidae	Aedes	Aedes aegypti	SPECIES	33.40548	-111.93263	3924989167
15733	Diptera	Culicidae	Aedes	Aedes aegypti	SPECIES	33.40786	-111.64343	2251872907
15734	Diptera	Culicidae	Aedes	Aedes aegypti	SPECIES	33.42000	-82.16000	1264888645
15735	Diptera	Culicidae	Aedes	Aedes aegypti	SPECIES	33.42000	-111.94000	1264890660
15736	Diptera	Culicidae	Aedes	Aedes aegypti	SPECIES	33.42000	-111.95000	1264890673
15737	Diptera	Culicidae	Aedes	Aedes aegypti	SPECIES	33.42728	-111.84510	4145235861
15738	Diptera	Culicidae	Aedes	Aedes aegypti	SPECIES	33.45363	-111.78527	3355281037
15739	Diptera	Culicidae	Aedes	Aedes aegypti	SPECIES	33.46025	-111.94549	3760087053
15740	Diptera	Culicidae	Aedes	Aedes aegypti	SPECIES	33.47000	-81.99000	1264888955
15741	Diptera	Culicidae	Aedes	Aedes aegypti	SPECIES	33.48139	-112.30323	3355180466
15742	Diptera	Culicidae	Aedes	Aedes aegypti	SPECIES	33.48191	-112.30344	3925425144
15743	Diptera	Culicidae	Aedes	Aedes aegypti	SPECIES	33.48195	-112.30350	2620209718
15744	Diptera	Culicidae	Aedes	Aedes aegypti	SPECIES	33.48195	-112.30347	2641456625

№	Order	Family	Genus	Species	Taxon Rank	Latitude	Longitude	GBIF_ID
15745	Diptera	Culicidae	Aedes	Aedes aegypti	SPECIES	33.48205	-112.30345	3858372833
15746	Diptera	Culicidae	Aedes	Aedes aegypti	SPECIES	33.48596	-117.72701	3923640345
15747	Diptera	Culicidae	Aedes	Aedes aegypti	SPECIES	33.50827	-112.29941	3925003298
15748	Diptera	Culicidae	Aedes	Aedes aegypti	SPECIES	33.53122	-117.77487	4039200570
15749	Diptera	Culicidae	Aedes	Aedes aegypti	SPECIES	33.53996	-101.89526	3399349144
15750	Diptera	Culicidae	Aedes	Aedes aegypti	SPECIES	33.54659	-101.93841	3391709688
15751	Diptera	Culicidae	Aedes	Aedes aegypti	SPECIES	33.58342	-112.03829	1931485931
15752	Diptera	Culicidae	Aedes	Aedes aegypti	SPECIES	33.59268	-117.71254	4052769336
15753	Diptera	Culicidae	Aedes	Aedes aegypti	SPECIES	33.60000	73.04000	1264887304
15754	Diptera	Culicidae	Aedes	Aedes aegypti	SPECIES	33.60659	-117.67563	1931537913
15755	Diptera	Culicidae	Aedes	Aedes aegypti	SPECIES	33.60685	-117.67421	1913205470
15756	Diptera	Culicidae	Aedes	Aedes aegypti	SPECIES	33.62969	-117.81697	3039458804
15757	Diptera	Culicidae	Aedes	Aedes aegypti	SPECIES	33.63000	-88.45000	1264889335
15758	Diptera	Culicidae	Aedes	Aedes aegypti	SPECIES	33.63000	73.07000	3347324348
15759	Diptera	Culicidae	Aedes	Aedes aegypti	SPECIES	33.65104	-112.01102	2456508638
15760	Diptera	Culicidae	Aedes	Aedes aegypti	SPECIES	33.65218	-117.65510	3031858884
15761	Diptera	Culicidae	Aedes	Aedes aegypti	SPECIES	33.65346	-117.75404	3961147728
15762	Diptera	Culicidae	Aedes	Aedes aegypti	SPECIES	33.66716	-112.20293	3923355737
15763	Diptera	Culicidae	Aedes	Aedes aegypti	SPECIES	33.67000	73.12000	1264891033
15764	Diptera	Culicidae	Aedes	Aedes aegypti	SPECIES	33.67090	-117.29100	2245559304
15765	Diptera	Culicidae	Aedes	Aedes aegypti	SPECIES	33.68000	73.15000	3350438369
15766	Diptera	Culicidae	Aedes	Aedes aegypti	SPECIES	33.68400	73.14700	2250857624
15767	Diptera	Culicidae	Aedes	Aedes aegypti	SPECIES	33.69559	-116.26460	3872942765
15768	Diptera	Culicidae	Aedes	Aedes aegypti	SPECIES	33.70000	73.03000	3350253361
15769	Diptera	Culicidae	Aedes	Aedes aegypti	SPECIES	33.70000	-84.30000	4066950598
15770	Diptera	Culicidae	Aedes	Aedes aegypti	SPECIES	33.70413	-116.20741	4116339549
15771	Diptera	Culicidae	Aedes	Aedes aegypti	SPECIES	33.71000	-84.43000	1264888626
15772	Diptera	Culicidae	Aedes	Aedes aegypti	SPECIES	33.72000	-85.78000	1264888648
15773	Diptera	Culicidae	Aedes	Aedes aegypti	SPECIES	33.72243	-118.30600	2963898805
15774	Diptera	Culicidae	Aedes	Aedes aegypti	SPECIES	33.72773	-117.98925	3330517985
15775	Diptera	Culicidae	Aedes	Aedes aegypti	SPECIES	33.74000	72.80000	1264887299
15776	Diptera	Culicidae	Aedes	Aedes aegypti	SPECIES	33.74900	-84.38798	3059295305
15777	Diptera	Culicidae	Aedes	Aedes aegypti	SPECIES	33.75900	-116.43689	3384888933
15778	Diptera	Culicidae	Aedes	Aedes aegypti	SPECIES	33.76129	-118.19738	3960996054
15779	Diptera	Culicidae	Aedes	Aedes aegypti	SPECIES	33.77000	72.36000	1264887323
15780	Diptera	Culicidae	Aedes	Aedes aegypti	SPECIES	33.77491	-112.10983	4127100451
15781	Diptera	Culicidae	Aedes	Aedes aegypti	SPECIES	33.77826	-118.14996	3391406128
15782	Diptera	Culicidae	Aedes	Aedes aegypti	SPECIES	33.80501	-118.11769	3328008145
15783	Diptera	Culicidae	Aedes	Aedes aegypti	SPECIES	33.81818	-86.69628	1933070206
15784	Diptera	Culicidae	Aedes	Aedes aegypti	SPECIES	33.81873	-117.84312	4102887509
15785	Diptera	Culicidae	Aedes	Aedes aegypti	SPECIES	33.82000	72.69000	1264887360

№	Order	Family	Genus	Species	Taxon Rank	Latitude	Longitude	GBIF_ID
15786	Diptera	Culicidae	Aedes	Aedes aegypti	SPECIES	33.82000	73.23000	3350340365
15787	Diptera	Culicidae	Aedes	Aedes aegypti	SPECIES	33.82310	-117.56500	2245454175
15788	Diptera	Culicidae	Aedes	Aedes aegypti	SPECIES	33.82991	-117.31733	3355119857
15789	Diptera	Culicidae	Aedes	Aedes aegypti	SPECIES	33.82996	-118.09933	3355127758
15790	Diptera	Culicidae	Aedes	Aedes aegypti	SPECIES	33.83399	-118.03188	3032078775
15791	Diptera	Culicidae	Aedes	Aedes aegypti	SPECIES	33.83478	-117.83394	3415672926
15792	Diptera	Culicidae	Aedes	Aedes aegypti	SPECIES	33.83784	-117.92730	3067754275
15793	Diptera	Culicidae	Aedes	Aedes aegypti	SPECIES	33.83943	-117.88313	3067857765
15794	Diptera	Culicidae	Aedes	Aedes aegypti	SPECIES	33.84361	-117.91845	4028907116
15795	Diptera	Culicidae	Aedes	Aedes aegypti	SPECIES	33.84479	-117.86984	3321260430
15796	Diptera	Culicidae	Aedes	Aedes aegypti	SPECIES	33.85120	-117.53700	2245454920
15797	Diptera	Culicidae	Aedes	Aedes aegypti	SPECIES	33.86504	-117.86548	1586123938
15798	Diptera	Culicidae	Aedes	Aedes aegypti	SPECIES	33.86682	-118.06864	4009892652
15799	Diptera	Culicidae	Aedes	Aedes aegypti	SPECIES	33.86800	-117.59900	2245412136
15800	Diptera	Culicidae	Aedes	Aedes aegypti	SPECIES	33.87670	-117.57600	2245456999
15801	Diptera	Culicidae	Aedes	Aedes aegypti	SPECIES	33.87748	-118.28747	2963885067
15802	Diptera	Culicidae	Aedes	Aedes aegypti	SPECIES	33.87930	-117.88356	3947443876
15803	Diptera	Culicidae	Aedes	Aedes aegypti	SPECIES	33.88041	-117.75144	3044870646
15804	Diptera	Culicidae	Aedes	Aedes aegypti	SPECIES	33.88585	-118.09554	3455531107
15805	Diptera	Culicidae	Aedes	Aedes aegypti	SPECIES	33.88850	-117.32900	2245495804
15806	Diptera	Culicidae	Aedes	Aedes aegypti	SPECIES	33.89810	-117.50737	3384736128
15807	Diptera	Culicidae	Aedes	Aedes aegypti	SPECIES	33.89811	-117.50768	3966458343
15808	Diptera	Culicidae	Aedes	Aedes aegypti	SPECIES	33.89834	-117.50727	3903031356
15809	Diptera	Culicidae	Aedes	Aedes aegypti	SPECIES	33.89840	-117.50688	3408008743
15810	Diptera	Culicidae	Aedes	Aedes aegypti	SPECIES	33.89840	-117.50728	4111573064
15811	Diptera	Culicidae	Aedes	Aedes aegypti	SPECIES	33.89840	-117.50734	3398960701
15812	Diptera	Culicidae	Aedes	Aedes aegypti	SPECIES	33.89841	-117.50724	3415987683
15813	Diptera	Culicidae	Aedes	Aedes aegypti	SPECIES	33.89843	-117.50716	3399419357
15814	Diptera	Culicidae	Aedes	Aedes aegypti	SPECIES	33.89846	-117.50733	3031829088
15815	Diptera	Culicidae	Aedes	Aedes aegypti	SPECIES	33.89846	-117.50715	4127196498
15816	Diptera	Culicidae	Aedes	Aedes aegypti	SPECIES	33.89846	-117.50725	2901713664
15817	Diptera	Culicidae	Aedes	Aedes aegypti	SPECIES	33.89846	-117.50713	3963462386
15818	Diptera	Culicidae	Aedes	Aedes aegypti	SPECIES	33.89848	-117.50732	2873887655
15819	Diptera	Culicidae	Aedes	Aedes aegypti	SPECIES	33.89848	-117.50705	3333486472
15820	Diptera	Culicidae	Aedes	Aedes aegypti	SPECIES	33.89848	-117.50707	3859975718
15821	Diptera	Culicidae	Aedes	Aedes aegypti	SPECIES	33.89849	-117.50719	2883183170
15822	Diptera	Culicidae	Aedes	Aedes aegypti	SPECIES	33.89849	-117.50732	3058759955
15823	Diptera	Culicidae	Aedes	Aedes aegypti	SPECIES	33.89850	-117.50719	3391374709
15824	Diptera	Culicidae	Aedes	Aedes aegypti	SPECIES	33.89850	-117.50731	3947090751
15825	Diptera	Culicidae	Aedes	Aedes aegypti	SPECIES	33.89850	-117.50732	2864620912
15826	Diptera	Culicidae	Aedes	Aedes aegypti	SPECIES	33.89851	-117.50733	2823149128

№	Order	Family	Genus	Species	Taxon Rank	Latitude	Longitude	GBIF_ID
15827	Diptera	Culicidae	Aedes	Aedes aegypti	SPECIES	33.89851	-117.50719	3923422606
15828	Diptera	Culicidae	Aedes	Aedes aegypti	SPECIES	33.89851	-117.50717	3415649706
15829	Diptera	Culicidae	Aedes	Aedes aegypti	SPECIES	33.89854	-117.50713	3384386716
15830	Diptera	Culicidae	Aedes	Aedes aegypti	SPECIES	33.89854	-117.50717	3963743340
15831	Diptera	Culicidae	Aedes	Aedes aegypti	SPECIES	33.89855	-117.50713	3408184502
15832	Diptera	Culicidae	Aedes	Aedes aegypti	SPECIES	33.89855	-117.50721	3892327689
15833	Diptera	Culicidae	Aedes	Aedes aegypti	SPECIES	33.89855	-117.50712	3994184606
15834	Diptera	Culicidae	Aedes	Aedes aegypti	SPECIES	33.89856	-117.50715	3355236572
15835	Diptera	Culicidae	Aedes	Aedes aegypti	SPECIES	33.89857	-117.50733	3327981144
15836	Diptera	Culicidae	Aedes	Aedes aegypti	SPECIES	33.89858	-117.50723	4165625567
15837	Diptera	Culicidae	Aedes	Aedes aegypti	SPECIES	33.89861	-117.50727	3320925229
15838	Diptera	Culicidae	Aedes	Aedes aegypti	SPECIES	33.90052	-117.46035	3415934265
15839	Diptera	Culicidae	Aedes	Aedes aegypti	SPECIES	33.90820	-117.42600	2245455789
15840	Diptera	Culicidae	Aedes	Aedes aegypti	SPECIES	33.90838	-117.49190	3759563808
15841	Diptera	Culicidae	Aedes	Aedes aegypti	SPECIES	33.90877	-118.01815	2611245519
15842	Diptera	Culicidae	Aedes	Aedes aegypti	SPECIES	33.91190	-117.45200	2245559367
15843	Diptera	Culicidae	Aedes	Aedes aegypti	SPECIES	33.91270	-117.45000	2245561238
15844	Diptera	Culicidae	Aedes	Aedes aegypti	SPECIES	33.91310	-117.45100	2245457170
15845	Diptera	Culicidae	Aedes	Aedes aegypti	SPECIES	33.91350	-117.44700	2245457301
15846	Diptera	Culicidae	Aedes	Aedes aegypti	SPECIES	33.91490	-117.44600	2245560194
15847	Diptera	Culicidae	Aedes	Aedes aegypti	SPECIES	33.91500	-117.45400	2245561065
15848	Diptera	Culicidae	Aedes	Aedes aegypti	SPECIES	33.91510	-117.44300	2245496991
15849	Diptera	Culicidae	Aedes	Aedes aegypti	SPECIES	33.91530	-117.44500	2245457894
15850	Diptera	Culicidae	Aedes	Aedes aegypti	SPECIES	33.91540	-117.44400	2245457453
15851	Diptera	Culicidae	Aedes	Aedes aegypti	SPECIES	33.91570	-117.44400	2245497056
15852	Diptera	Culicidae	Aedes	Aedes aegypti	SPECIES	33.91630	-117.45000	2245457848
15853	Diptera	Culicidae	Aedes	Aedes aegypti	SPECIES	33.91660	-117.45000	2245561429
15854	Diptera	Culicidae	Aedes	Aedes aegypti	SPECIES	33.91710	-117.45100	2245457764
15855	Diptera	Culicidae	Aedes	Aedes aegypti	SPECIES	33.92432	-117.33550	3915497804
15856	Diptera	Culicidae	Aedes	Aedes aegypti	SPECIES	33.92446	-117.33561	2826486647
15857	Diptera	Culicidae	Aedes	Aedes aegypti	SPECIES	33.93362	-117.95060	1931505616
15858	Diptera	Culicidae	Aedes	Aedes aegypti	SPECIES	33.93362	-117.95060	2813933010
15859	Diptera	Culicidae	Aedes	Aedes aegypti	SPECIES	33.93370	-117.95056	1933547931
15860	Diptera	Culicidae	Aedes	Aedes aegypti	SPECIES	33.93401	-118.14848	2826493849
15861	Diptera	Culicidae	Aedes	Aedes aegypti	SPECIES	33.93420	-117.54000	2245456677
15862	Diptera	Culicidae	Aedes	Aedes aegypti	SPECIES	33.94038	-117.23989	3407893518
15863	Diptera	Culicidae	Aedes	Aedes aegypti	SPECIES	33.94143	-118.15029	3407957544
15864	Diptera	Culicidae	Aedes	Aedes aegypti	SPECIES	33.95180	-117.58600	2245455551
15865	Diptera	Culicidae	Aedes	Aedes aegypti	SPECIES	33.95224	-117.26402	3923647778
15866	Diptera	Culicidae	Aedes	Aedes aegypti	SPECIES	33.95226	-117.26395	3327762291
15867	Diptera	Culicidae	Aedes	Aedes aegypti	SPECIES	33.95780	-117.40000	2245456733

№	Order	Family	Genus	Species	Taxon Rank	Latitude	Longitude	GBIF_ID
15868	Diptera	Culicidae	Aedes	Aedes aegypti	SPECIES	33.95880	-117.39700	2245457468
15869	Diptera	Culicidae	Aedes	Aedes aegypti	SPECIES	33.95910	-117.40000	2245456447
15870	Diptera	Culicidae	Aedes	Aedes aegypti	SPECIES	33.96030	-117.39800	2245457385
15871	Diptera	Culicidae	Aedes	Aedes aegypti	SPECIES	33.96080	-117.35000	2245457949
15872	Diptera	Culicidae	Aedes	Aedes aegypti	SPECIES	33.96340	-117.42300	2245458090
15873	Diptera	Culicidae	Aedes	Aedes aegypti	SPECIES	33.96350	-117.38600	2245457100
15874	Diptera	Culicidae	Aedes	Aedes aegypti	SPECIES	33.96360	-117.42700	2245458038
15875	Diptera	Culicidae	Aedes	Aedes aegypti	SPECIES	33.96660	-117.38700	2245457372
15876	Diptera	Culicidae	Aedes	Aedes aegypti	SPECIES	33.96800	-117.38900	2245457596
15877	Diptera	Culicidae	Aedes	Aedes aegypti	SPECIES	33.96840	-117.36500	2245457215
15878	Diptera	Culicidae	Aedes	Aedes aegypti	SPECIES	33.96890	-117.38800	2245457941
15879	Diptera	Culicidae	Aedes	Aedes aegypti	SPECIES	33.97000	-80.48000	1264889352
15880	Diptera	Culicidae	Aedes	Aedes aegypti	SPECIES	33.97119	-117.32894	3881814361
15881	Diptera	Culicidae	Aedes	Aedes aegypti	SPECIES	33.97251	-117.32758	4103164933
15882	Diptera	Culicidae	Aedes	Aedes aegypti	SPECIES	33.97330	-117.39500	2245561903
15883	Diptera	Culicidae	Aedes	Aedes aegypti	SPECIES	33.97350	-117.39100	2245454405
15884	Diptera	Culicidae	Aedes	Aedes aegypti	SPECIES	33.97490	-117.38700	2245456339
15885	Diptera	Culicidae	Aedes	Aedes aegypti	SPECIES	33.97505	-118.02734	1640057475
15886	Diptera	Culicidae	Aedes	Aedes aegypti	SPECIES	33.97620	-117.39000	2245457181
15887	Diptera	Culicidae	Aedes	Aedes aegypti	SPECIES	33.97681	-117.32229	2850714257
15888	Diptera	Culicidae	Aedes	Aedes aegypti	SPECIES	33.97840	-117.35500	2245456287
15889	Diptera	Culicidae	Aedes	Aedes aegypti	SPECIES	33.98570	-117.97148	3343777963
15890	Diptera	Culicidae	Aedes	Aedes aegypti	SPECIES	33.98818	-117.71700	3858486942
15891	Diptera	Culicidae	Aedes	Aedes aegypti	SPECIES	33.99069	-116.35843	4129725322
15892	Diptera	Culicidae	Aedes	Aedes aegypti	SPECIES	33.99070	-117.37800	2245456726
15893	Diptera	Culicidae	Aedes	Aedes aegypti	SPECIES	33.99200	-117.40600	2245454077
15894	Diptera	Culicidae	Aedes	Aedes aegypti	SPECIES	33.99230	-117.39700	2245458318
15895	Diptera	Culicidae	Aedes	Aedes aegypti	SPECIES	33.99380	-117.39900	2245457347
15896	Diptera	Culicidae	Aedes	Aedes aegypti	SPECIES	34.00000	72.94000	1264887318
15897	Diptera	Culicidae	Aedes	Aedes aegypti	SPECIES	34.00000	-81.04000	1264889004
15898	Diptera	Culicidae	Aedes	Aedes aegypti	SPECIES	34.00958	-117.27287	2999067005
15899	Diptera	Culicidae	Aedes	Aedes aegypti	SPECIES	34.01000	71.58000	1264887327
15900	Diptera	Culicidae	Aedes	Aedes aegypti	SPECIES	34.01050	-117.35300	2245453732
15901	Diptera	Culicidae	Aedes	Aedes aegypti	SPECIES	34.01477	-118.11595	3408000497
15902	Diptera	Culicidae	Aedes	Aedes aegypti	SPECIES	34.01511	-117.09529	2634410839
15903	Diptera	Culicidae	Aedes	Aedes aegypti	SPECIES	34.01754	-118.28852	3873835230
15904	Diptera	Culicidae	Aedes	Aedes aegypti	SPECIES	34.01850	-118.29628	3860014003
15905	Diptera	Culicidae	Aedes	Aedes aegypti	SPECIES	34.01850	-118.29628	3873303091
15906	Diptera	Culicidae	Aedes	Aedes aegypti	SPECIES	34.01851	-118.29630	2826318386
15907	Diptera	Culicidae	Aedes	Aedes aegypti	SPECIES	34.01854	-118.29627	3468674868
15908	Diptera	Culicidae	Aedes	Aedes aegypti	SPECIES	34.02044	-118.23157	2901604132

№	Order	Family	Genus	Species	Taxon Rank	Latitude	Longitude	GBIF_ID
15909	Diptera	Culicidae	Aedes	Aedes aegypti	SPECIES	34.02452	-118.42607	3415399229
15910	Diptera	Culicidae	Aedes	Aedes aegypti	SPECIES	34.02464	-118.15375	3391309155
15911	Diptera	Culicidae	Aedes	Aedes aegypti	SPECIES	34.02465	-118.15336	3337869566
15912	Diptera	Culicidae	Aedes	Aedes aegypti	SPECIES	34.02465	-118.15337	3355698002
15913	Diptera	Culicidae	Aedes	Aedes aegypti	SPECIES	34.02467	-118.15333	3355280984
15914	Diptera	Culicidae	Aedes	Aedes aegypti	SPECIES	34.02473	-118.15364	2818774258
15915	Diptera	Culicidae	Aedes	Aedes aegypti	SPECIES	34.02479	-118.15361	3013991756
15916	Diptera	Culicidae	Aedes	Aedes aegypti	SPECIES	34.02988	-118.17061	2988291955
15917	Diptera	Culicidae	Aedes	Aedes aegypti	SPECIES	34.03108	-118.16763	2331903887
15918	Diptera	Culicidae	Aedes	Aedes aegypti	SPECIES	34.03196	-118.38139	3302365309
15919	Diptera	Culicidae	Aedes	Aedes aegypti	SPECIES	34.03629	-117.76743	3416246283
15920	Diptera	Culicidae	Aedes	Aedes aegypti	SPECIES	34.03978	-118.46085	3343780058
15921	Diptera	Culicidae	Aedes	Aedes aegypti	SPECIES	34.04160	-118.45068	3802628438
15922	Diptera	Culicidae	Aedes	Aedes aegypti	SPECIES	34.04256	-118.44503	3415867126
15923	Diptera	Culicidae	Aedes	Aedes aegypti	SPECIES	34.04387	-118.29868	2814267376
15924	Diptera	Culicidae	Aedes	Aedes aegypti	SPECIES	34.04429	-118.37026	2460096636
15925	Diptera	Culicidae	Aedes	Aedes aegypti	SPECIES	34.04459	-117.92169	3764636332
15926	Diptera	Culicidae	Aedes	Aedes aegypti	SPECIES	34.04693	-117.04711	3343918628
15927	Diptera	Culicidae	Aedes	Aedes aegypti	SPECIES	34.04953	-118.21100	3496614107
15928	Diptera	Culicidae	Aedes	Aedes aegypti	SPECIES	34.04956	-118.21095	4052769258
15929	Diptera	Culicidae	Aedes	Aedes aegypti	SPECIES	34.04968	-118.21118	3496616053
15930	Diptera	Culicidae	Aedes	Aedes aegypti	SPECIES	34.05000	-80.78000	1264888653
15931	Diptera	Culicidae	Aedes	Aedes aegypti	SPECIES	34.05410	-117.14001	3947369218
15932	Diptera	Culicidae	Aedes	Aedes aegypti	SPECIES	34.05513	-118.27225	3915616563
15933	Diptera	Culicidae	Aedes	Aedes aegypti	SPECIES	34.05518	-117.68931	2397594035
15934	Diptera	Culicidae	Aedes	Aedes aegypti	SPECIES	34.05783	-117.82525	4009931600
15935	Diptera	Culicidae	Aedes	Aedes aegypti	SPECIES	34.06182	-117.16453	3902209855
15936	Diptera	Culicidae	Aedes	Aedes aegypti	SPECIES	34.06247	-117.16286	3398922216
15937	Diptera	Culicidae	Aedes	Aedes aegypti	SPECIES	34.06444	-118.12694	1831120514
15938	Diptera	Culicidae	Aedes	Aedes aegypti	SPECIES	34.06934	-118.44161	3923453920
15939	Diptera	Culicidae	Aedes	Aedes aegypti	SPECIES	34.07104	-118.40416	3760409460
15940	Diptera	Culicidae	Aedes	Aedes aegypti	SPECIES	34.07796	-118.13739	2860170342
15941	Diptera	Culicidae	Aedes	Aedes aegypti	SPECIES	34.07805	-118.23434	3018023561
15942	Diptera	Culicidae	Aedes	Aedes aegypti	SPECIES	34.07832	-117.70963	3785139728
15943	Diptera	Culicidae	Aedes	Aedes aegypti	SPECIES	34.08463	-118.39258	2992786940
15944	Diptera	Culicidae	Aedes	Aedes aegypti	SPECIES	34.08660	-118.05474	3112690876
15945	Diptera	Culicidae	Aedes	Aedes aegypti	SPECIES	34.09534	-118.13274	2814003717
15946	Diptera	Culicidae	Aedes	Aedes aegypti	SPECIES	34.09609	-118.41750	3415741428
15947	Diptera	Culicidae	Aedes	Aedes aegypti	SPECIES	34.10000	35.85000	1264889089
15948	Diptera	Culicidae	Aedes	Aedes aegypti	SPECIES	34.10000	71.15000	1264890042
15949	Diptera	Culicidae	Aedes	Aedes aegypti	SPECIES	34.10526	-118.37488	3961462246

№	Order	Family	Genus	Species	Taxon Rank	Latitude	Longitude	GBIF_ID
15950	Diptera	Culicidae	Aedes	Aedes aegypti	SPECIES	34.10640	-117.59311	3988553616
15951	Diptera	Culicidae	Aedes	Aedes aegypti	SPECIES	34.10711	-118.05109	3416237569
15952	Diptera	Culicidae	Aedes	Aedes aegypti	SPECIES	34.10788	-118.43600	3391691130
15953	Diptera	Culicidae	Aedes	Aedes aegypti	SPECIES	34.10791	-118.43593	3960997969
15954	Diptera	Culicidae	Aedes	Aedes aegypti	SPECIES	34.11021	-118.07462	4165489216
15955	Diptera	Culicidae	Aedes	Aedes aegypti	SPECIES	34.11028	-118.07455	3415361421
15956	Diptera	Culicidae	Aedes	Aedes aegypti	SPECIES	34.11167	-118.15610	2851282284
15957	Diptera	Culicidae	Aedes	Aedes aegypti	SPECIES	34.11172	-118.15583	2862381351
15958	Diptera	Culicidae	Aedes	Aedes aegypti	SPECIES	34.11966	-118.25211	3327700084
15959	Diptera	Culicidae	Aedes	Aedes aegypti	SPECIES	34.12363	-118.18073	2873831858
15960	Diptera	Culicidae	Aedes	Aedes aegypti	SPECIES	34.12555	-118.25998	2898419130
15961	Diptera	Culicidae	Aedes	Aedes aegypti	SPECIES	34.12610	-118.21910	3344075507
15962	Diptera	Culicidae	Aedes	Aedes aegypti	SPECIES	34.12617	-118.21262	2634225331
15963	Diptera	Culicidae	Aedes	Aedes aegypti	SPECIES	34.13364	-118.51935	2850836572
15964	Diptera	Culicidae	Aedes	Aedes aegypti	SPECIES	34.13918	-118.20029	3384812315
15965	Diptera	Culicidae	Aedes	Aedes aegypti	SPECIES	34.14110	-118.42474	2851002109
15966	Diptera	Culicidae	Aedes	Aedes aegypti	SPECIES	34.14273	-118.48651	3915317184
15967	Diptera	Culicidae	Aedes	Aedes aegypti	SPECIES	34.14434	-118.11611	2451688033
15968	Diptera	Culicidae	Aedes	Aedes aegypti	SPECIES	34.14453	-118.38336	2883154629
15969	Diptera	Culicidae	Aedes	Aedes aegypti	SPECIES	34.15000	71.72000	3350377368
15970	Diptera	Culicidae	Aedes	Aedes aegypti	SPECIES	34.15120	-118.55356	1946311185
15971	Diptera	Culicidae	Aedes	Aedes aegypti	SPECIES	34.15208	-117.31215	2984468607
15972	Diptera	Culicidae	Aedes	Aedes aegypti	SPECIES	34.15768	-118.29933	3355755572
15973	Diptera	Culicidae	Aedes	Aedes aegypti	SPECIES	34.15860	-118.28323	3892532768
15974	Diptera	Culicidae	Aedes	Aedes aegypti	SPECIES	34.16000	-82.13000	4066959835
15975	Diptera	Culicidae	Aedes	Aedes aegypti	SPECIES	34.16058	-117.18449	3109361496
15976	Diptera	Culicidae	Aedes	Aedes aegypti	SPECIES	34.16388	-118.29200	2898320109
15977	Diptera	Culicidae	Aedes	Aedes aegypti	SPECIES	34.16969	-118.34822	2423149218
15978	Diptera	Culicidae	Aedes	Aedes aegypti	SPECIES	34.17837	-103.35002	3923485854
15979	Diptera	Culicidae	Aedes	Aedes aegypti	SPECIES	34.17905	-118.29744	3031645012
15980	Diptera	Culicidae	Aedes	Aedes aegypti	SPECIES	34.19000	-79.78000	1264888997
15981	Diptera	Culicidae	Aedes	Aedes aegypti	SPECIES	34.19061	-118.20041	3399360701
15982	Diptera	Culicidae	Aedes	Aedes aegypti	SPECIES	34.19720	-118.20723	2898319201
15983	Diptera	Culicidae	Aedes	Aedes aegypti	SPECIES	34.20529	-117.36237	2641402224
15984	Diptera	Culicidae	Aedes	Aedes aegypti	SPECIES	34.22147	-118.60881	3915567412
15985	Diptera	Culicidae	Aedes	Aedes aegypti	SPECIES	34.23081	-118.23548	2862375399
15986	Diptera	Culicidae	Aedes	Aedes aegypti	SPECIES	34.23334	-118.23247	2859907070
15987	Diptera	Culicidae	Aedes	Aedes aegypti	SPECIES	34.24865	-118.55058	3314299527
15988	Diptera	Culicidae	Aedes	Aedes aegypti	SPECIES	34.24891	-118.37612	3031828838
15989	Diptera	Culicidae	Aedes	Aedes aegypti	SPECIES	34.25200	-118.26218	3031594845
15990	Diptera	Culicidae	Aedes	Aedes aegypti	SPECIES	34.26526	-118.35712	2851120798

№	Order	Family	Genus	Species	Taxon Rank	Latitude	Longitude	GBIF_ID
15991	Diptera	Culicidae	Aedes	Aedes aegypti	SPECIES	34.27765	-118.74323	3384296838
15992	Diptera	Culicidae	Aedes	Aedes aegypti	SPECIES	34.29157	-118.56304	3398821017
15993	Diptera	Culicidae	Aedes	Aedes aegypti	SPECIES	34.37815	-118.46710	3384129204
15994	Diptera	Culicidae	Aedes	Aedes aegypti	SPECIES	34.39308	-118.56199	3385197308
15995	Diptera	Culicidae	Aedes	Aedes aegypti	SPECIES	34.40717	-118.54219	3499444846
15996	Diptera	Culicidae	Aedes	Aedes aegypti	SPECIES	34.41487	-118.56947	3925211587
15997	Diptera	Culicidae	Aedes	Aedes aegypti	SPECIES	34.45000	-81.78000	1264889840
15998	Diptera	Culicidae	Aedes	Aedes aegypti	SPECIES	34.53000	69.17000	1264890855
15999	Diptera	Culicidae	Aedes	Aedes aegypti	SPECIES	34.61000	-112.55000	1264871695
16000	Diptera	Culicidae	Aedes	Aedes aegypti	SPECIES	34.65300	-82.83300	4066958833
16001	Diptera	Culicidae	Aedes	Aedes aegypti	SPECIES	34.74639	-92.28944	1141855442
16002	Diptera	Culicidae	Aedes	Aedes aegypti	SPECIES	34.85000	-82.41000	1264888990
16003	Diptera	Culicidae	Aedes	Aedes aegypti	SPECIES	34.86000	33.59000	3969164392
16004	Diptera	Culicidae	Aedes	Aedes aegypti	SPECIES	34.86610	-111.82190	3355371109
16005	Diptera	Culicidae	Aedes	Aedes aegypti	SPECIES	34.89000	-82.72000	4066964833
16006	Diptera	Culicidae	Aedes	Aedes aegypti	SPECIES	34.91000	-92.13000	1264889327
16007	Diptera	Culicidae	Aedes	Aedes aegypti	SPECIES	34.95000	-81.93000	1264888968
16008	Diptera	Culicidae	Aedes	Aedes aegypti	SPECIES	35.03584	-106.72936	3912135071
16009	Diptera	Culicidae	Aedes	Aedes aegypti	SPECIES	35.06012	-106.59446	3915660552
16010	Diptera	Culicidae	Aedes	Aedes aegypti	SPECIES	35.06920	-106.59141	3923377127
16011	Diptera	Culicidae	Aedes	Aedes aegypti	SPECIES	35.07979	-106.62571	3915388822
16012	Diptera	Culicidae	Aedes	Aedes aegypti	SPECIES	35.08176	-106.62222	3947081195
16013	Diptera	Culicidae	Aedes	Aedes aegypti	SPECIES	35.09866	-106.60897	3903224257
16014	Diptera	Culicidae	Aedes	Aedes aegypti	SPECIES	35.10273	-106.62900	1948774818
16015	Diptera	Culicidae	Aedes	Aedes aegypti	SPECIES	35.10956	-106.55515	3923383804
16016	Diptera	Culicidae	Aedes	Aedes aegypti	SPECIES	35.11348	-106.48100	3966687922
16017	Diptera	Culicidae	Aedes	Aedes aegypti	SPECIES	35.11941	-106.50786	3888914514
16018	Diptera	Culicidae	Aedes	Aedes aegypti	SPECIES	35.11954	-106.50778	3902270866
16019	Diptera	Culicidae	Aedes	Aedes aegypti	SPECIES	35.13300	-106.54220	3888750908
16020	Diptera	Culicidae	Aedes	Aedes aegypti	SPECIES	35.13898	-106.52785	3888828579
16021	Diptera	Culicidae	Aedes	Aedes aegypti	SPECIES	35.14000	-90.04000	1264889778
16022	Diptera	Culicidae	Aedes	Aedes aegypti	SPECIES	35.14667	-106.64913	3902391536
16023	Diptera	Culicidae	Aedes	Aedes aegypti	SPECIES	35.14937	-106.57035	3915409732
16024	Diptera	Culicidae	Aedes	Aedes aegypti	SPECIES	35.18495	-106.42194	3902595038
16025	Diptera	Culicidae	Aedes	Aedes aegypti	SPECIES	35.35000	-77.96000	1264889340
16026	Diptera	Culicidae	Aedes	Aedes aegypti	SPECIES	35.37315	-119.01871	2898319892
16027	Diptera	Culicidae	Aedes	Aedes aegypti	SPECIES	35.42000	-97.39000	1264889356
16028	Diptera	Culicidae	Aedes	Aedes aegypti	SPECIES	35.47000	139.62000	1264889032
16029	Diptera	Culicidae	Aedes	Aedes aegypti	SPECIES	35.49000	-83.32000	1264889799
16030	Diptera	Culicidae	Aedes	Aedes aegypti	SPECIES	35.51000	-83.30000	1264889817
16031	Diptera	Culicidae	Aedes	Aedes aegypti	SPECIES	35.59220	0.06487	1843543115

№	Order	Family	Genus	Species	Taxon Rank	Latitude	Longitude	GBIF_ID
16032	Diptera	Culicidae	Aedes	Aedes aegypti	SPECIES	35.63000	139.70000	1264886881
16033	Diptera	Culicidae	Aedes	Aedes aegypti	SPECIES	35.68130	-0.61416	1843528900
16034	Diptera	Culicidae	Aedes	Aedes aegypti	SPECIES	35.75000	140.38000	1264887510
16035	Diptera	Culicidae	Aedes	Aedes aegypti	SPECIES	35.79000	-78.65000	4066964834
16036	Diptera	Culicidae	Aedes	Aedes aegypti	SPECIES	35.96000	0.27000	1843538459
16037	Diptera	Culicidae	Aedes	Aedes aegypti	SPECIES	36.09410	-119.05630	2634123633
16038	Diptera	Culicidae	Aedes	Aedes aegypti	SPECIES	36.13841	-82.85547	4165442528
16039	Diptera	Culicidae	Aedes	Aedes aegypti	SPECIES	36.34000	-94.25000	1264871898
16040	Diptera	Culicidae	Aedes	Aedes aegypti	SPECIES	36.58334	-119.58333	1264885976
16041	Diptera	Culicidae	Aedes	Aedes aegypti	SPECIES	36.59238	-119.75000	1264885996
16042	Diptera	Culicidae	Aedes	Aedes aegypti	SPECIES	36.60002	-119.94751	4130290469
16043	Diptera	Culicidae	Aedes	Aedes aegypti	SPECIES	36.61146	-119.84308	3328162641
16044	Diptera	Culicidae	Aedes	Aedes aegypti	SPECIES	36.62710	-119.84498	3759967959
16045	Diptera	Culicidae	Aedes	Aedes aegypti	SPECIES	36.64397	-119.85836	4046394804
16046	Diptera	Culicidae	Aedes	Aedes aegypti	SPECIES	36.64791	-119.95998	2851276035
16047	Diptera	Culicidae	Aedes	Aedes aegypti	SPECIES	36.64876	-119.75000	3328113504
16048	Diptera	Culicidae	Aedes	Aedes aegypti	SPECIES	36.69444	-119.95283	2851315957
16049	Diptera	Culicidae	Aedes	Aedes aegypti	SPECIES	36.70000	-76.64000	1264871806
16050	Diptera	Culicidae	Aedes	Aedes aegypti	SPECIES	36.72776	-119.83547	3923574482
16051	Diptera	Culicidae	Aedes	Aedes aegypti	SPECIES	36.73000	-76.58700	3030358519
16052	Diptera	Culicidae	Aedes	Aedes aegypti	SPECIES	36.75000	-119.75000	1264885995
16053	Diptera	Culicidae	Aedes	Aedes aegypti	SPECIES	36.75000	-119.64000	1264876391
16054	Diptera	Culicidae	Aedes	Aedes aegypti	SPECIES	36.75000	-119.58333	1264886038
16055	Diptera	Culicidae	Aedes	Aedes aegypti	SPECIES	36.75227	-119.99708	2851291091
16056	Diptera	Culicidae	Aedes	Aedes aegypti	SPECIES	36.84667	-76.28556	3801474371
16057	Diptera	Culicidae	Aedes	Aedes aegypti	SPECIES	36.84682	-76.28522	3356901312
16058	Diptera	Culicidae	Aedes	Aedes aegypti	SPECIES	36.91663	-119.75000	1264886001
16059	Diptera	Culicidae	Aedes	Aedes aegypti	SPECIES	36.91667	-120.08333	1264885989
16060	Diptera	Culicidae	Aedes	Aedes aegypti	SPECIES	36.91667	-119.91667	1264886028
16061	Diptera	Culicidae	Aedes	Aedes aegypti	SPECIES	37.08333	-120.08333	1264886006
16062	Diptera	Culicidae	Aedes	Aedes aegypti	SPECIES	37.14000	-78.06000	1264871656
16063	Diptera	Culicidae	Aedes	Aedes aegypti	SPECIES	37.38000	-77.59000	1264871651
16064	Diptera	Culicidae	Aedes	Aedes aegypti	SPECIES	37.41667	-122.25000	1264886005
16065	Diptera	Culicidae	Aedes	Aedes aegypti	SPECIES	37.46000	126.95000	1264889080
16066	Diptera	Culicidae	Aedes	Aedes aegypti	SPECIES	37.77642	-122.27602	3356895309
16067	Diptera	Culicidae	Aedes	Aedes aegypti	SPECIES	37.77769	-122.27734	3356896312
16068	Diptera	Culicidae	Aedes	Aedes aegypti	SPECIES	37.98000	-87.56000	1264890355
16069	Diptera	Culicidae	Aedes	Aedes aegypti	SPECIES	38.03000	-77.35000	1264871692
16070	Diptera	Culicidae	Aedes	Aedes aegypti	SPECIES	38.24000	27.97000	1264888313
16071	Diptera	Culicidae	Aedes	Aedes aegypti	SPECIES	38.33600	-90.15000	3801945333
16072	Diptera	Culicidae	Aedes	Aedes aegypti	SPECIES	38.52005	-89.98399	3802032332

№	Order	Family	Genus	Species	Taxon Rank	Latitude	Longitude	GBIF_ID
16073	Diptera	Culicidae	Aedes	Aedes aegypti	SPECIES	38.77305	-77.06297	1913148110
16074	Diptera	Culicidae	Aedes	Aedes aegypti	SPECIES	38.80970	-76.99510	3030838479
16075	Diptera	Culicidae	Aedes	Aedes aegypti	SPECIES	38.81139	-89.95306	3802025286
16076	Diptera	Culicidae	Aedes	Aedes aegypti	SPECIES	38.82000	-77.09000	1264871784
16077	Diptera	Culicidae	Aedes	Aedes aegypti	SPECIES	38.83220	-77.10183	3985662119
16078	Diptera	Culicidae	Aedes	Aedes aegypti	SPECIES	38.84000	-77.28000	1264871655
16079	Diptera	Culicidae	Aedes	Aedes aegypti	SPECIES	38.86565	-77.17915	1306581260
16080	Diptera	Culicidae	Aedes	Aedes aegypti	SPECIES	38.87882	-76.97617	2447958852
16081	Diptera	Culicidae	Aedes	Aedes aegypti	SPECIES	38.88000	-77.10000	1264871665
16082	Diptera	Culicidae	Aedes	Aedes aegypti	SPECIES	38.88713	-77.00611	3902203579
16083	Diptera	Culicidae	Aedes	Aedes aegypti	SPECIES	38.89200	-76.90620	3030843906
16084	Diptera	Culicidae	Aedes	Aedes aegypti	SPECIES	38.89210	-76.90620	3030932229
16085	Diptera	Culicidae	Aedes	Aedes aegypti	SPECIES	38.89220	-76.90620	3030332292
16086	Diptera	Culicidae	Aedes	Aedes aegypti	SPECIES	38.89230	-76.90580	3030370070
16087	Diptera	Culicidae	Aedes	Aedes aegypti	SPECIES	38.89320	-76.90810	3030772063
16088	Diptera	Culicidae	Aedes	Aedes aegypti	SPECIES	38.90000	-77.02000	1264871690
16089	Diptera	Culicidae	Aedes	Aedes aegypti	SPECIES	38.90400	-76.92000	3030834867
16090	Diptera	Culicidae	Aedes	Aedes aegypti	SPECIES	38.90752	-76.99513	1913152863
16091	Diptera	Culicidae	Aedes	Aedes aegypti	SPECIES	38.91089	-77.04360	3912471466
16092	Diptera	Culicidae	Aedes	Aedes aegypti	SPECIES	38.92160	-76.92100	3030938309
16093	Diptera	Culicidae	Aedes	Aedes aegypti	SPECIES	38.92300	-76.92190	3030496112
16094	Diptera	Culicidae	Aedes	Aedes aegypti	SPECIES	38.92310	-76.92100	3030931130
16095	Diptera	Culicidae	Aedes	Aedes aegypti	SPECIES	38.92320	-76.92220	3030860188
16096	Diptera	Culicidae	Aedes	Aedes aegypti	SPECIES	38.92669	-76.96534	4127030837
16097	Diptera	Culicidae	Aedes	Aedes aegypti	SPECIES	38.92845	-77.06043	3915687425
16098	Diptera	Culicidae	Aedes	Aedes aegypti	SPECIES	38.93498	-77.05384	3385106024
16099	Diptera	Culicidae	Aedes	Aedes aegypti	SPECIES	38.95963	-77.08287	4009797600
16100	Diptera	Culicidae	Aedes	Aedes aegypti	SPECIES	38.96570	-76.89630	3893360858
16101	Diptera	Culicidae	Aedes	Aedes aegypti	SPECIES	38.97170	-95.23500	1141856020
16102	Diptera	Culicidae	Aedes	Aedes aegypti	SPECIES	38.98070	-76.93690	3059277369
16103	Diptera	Culicidae	Aedes	Aedes aegypti	SPECIES	38.98969	-76.91621	3961014968
16104	Diptera	Culicidae	Aedes	Aedes aegypti	SPECIES	38.99628	-77.03207	2429284254
16105	Diptera	Culicidae	Aedes	Aedes aegypti	SPECIES	39.00000	-76.61000	1264871705
16106	Diptera	Culicidae	Aedes	Aedes aegypti	SPECIES	39.00164	-76.94201	2429524805
16107	Diptera	Culicidae	Aedes	Aedes aegypti	SPECIES	39.07730	-76.88240	3030843237
16108	Diptera	Culicidae	Aedes	Aedes aegypti	SPECIES	39.09000	-77.64000	1264871831
16109	Diptera	Culicidae	Aedes	Aedes aegypti	SPECIES	39.14000	-77.20000	1264871724
16110	Diptera	Culicidae	Aedes	Aedes aegypti	SPECIES	39.22000	-85.91000	1264888811
16111	Diptera	Culicidae	Aedes	Aedes aegypti	SPECIES	39.25000	-76.93000	1264871669
16112	Diptera	Culicidae	Aedes	Aedes aegypti	SPECIES	39.99610	-83.01890	1019031532
16113	Diptera	Culicidae	Aedes	Aedes aegypti	SPECIES	39.99868	-83.01698	2466128143

№	Order	Family	Genus	Species	Taxon Rank	Latitude	Longitude	GBIF_ID
16114	Diptera	Culicidae	Aedes	Aedes aegypti	SPECIES	40.00000	-83.01250	3801537142
16115	Diptera	Culicidae	Aedes	Aedes aegypti	SPECIES	40.10972	-88.20417	3802038335
16116	Diptera	Culicidae	Aedes	Aedes aegypti	SPECIES	40.67000	-74.50000	1264888407
16117	Diptera	Culicidae	Aedes	Aedes aegypti	SPECIES	41.06390	-80.05670	1455681919
16118	Diptera	Culicidae	Aedes	Aedes aegypti	SPECIES	41.18000	-73.89000	1264888037
16119	Diptera	Culicidae	Aedes	Aedes aegypti	SPECIES	41.30815	-72.92816	3068147319
16120	Diptera	Culicidae	Aedes	Aedes aegypti	SPECIES	41.31614	-72.93084	3068159344
16121	Diptera	Culicidae	Aedes	Aedes aegypti	SPECIES	41.36767	-72.91032	3068158321
16122	Diptera	Culicidae	Aedes	Aedes aegypti	SPECIES	41.61000	-71.25000	1264888269
16123	Diptera	Culicidae	Aedes	Aedes aegypti	SPECIES	41.88000	-87.62000	1264890343
16124	Diptera	Culicidae	Aedes	Aedes aegypti	SPECIES	42.31000	-83.03000	3348612622
16125	Diptera	Culicidae	Aedes	Aedes aegypti	SPECIES	42.73016	-84.47622	3385771758
16126	Diptera	Culicidae	Aedes	Aedes aegypti	SPECIES	43.00154	41.02342	1264891479
16127	Diptera	Culicidae	Aedes	Aedes aegypti	SPECIES	43.05000	40.48000	3348846128
16128	Diptera	Culicidae	Aedes	Aedes aegypti	SPECIES	43.09000	40.82000	3351572524
16129	Diptera	Culicidae	Aedes	Aedes aegypti	SPECIES	43.10186	40.61859	1264891484
16130	Diptera	Culicidae	Aedes	Aedes aegypti	SPECIES	43.26000	39.55000	3351548163
16131	Diptera	Culicidae	Aedes	Aedes aegypti	SPECIES	43.45000	39.90000	3351485766
16132	Diptera	Culicidae	Aedes	Aedes aegypti	SPECIES	43.60000	39.75000	3351437027
16133	Diptera	Culicidae	Aedes	Aedes aegypti	SPECIES	43.63000	39.69000	3351517521
16134	Diptera	Culicidae	Aedes	Aedes aegypti	SPECIES	44.06000	39.05000	3348758945
16135	Diptera	Culicidae	Aedes	Aedes aegypti	SPECIES	44.11000	39.07000	3351547732
16136	Diptera	Culicidae	Aedes	Aedes aegypti	SPECIES	45.08000	33.35000	3351260539
16137	Diptera	Culicidae	Aedes	Aedes aegypti	SPECIES	45.45000	-76.47000	4066960835
16138	Diptera	Culicidae	Aedes	Aedes aegypti	SPECIES	45.58110	-84.69690	1141858167
16139	Diptera	Culicidae	Aedes	Aedes aegypti	SPECIES	46.35000	-83.58000	1264888817
16140	Diptera	Culicidae	Aedes	Aedes aegypti	SPECIES	48.90361	2.47472	732501139
16141	Diptera	Culicidae	Aedes	Aedes aegypti	SPECIES	49.25100	-123.13700	866398371
16142	Diptera	Culicidae	Aedes	Aedes aegypti	SPECIES	51.63513	0.09380	2924309244
16143	Diptera	Culicidae	Aedes	Aedes aegypti	SPECIES	53.49318	9.31902	2251764573
16144	Diptera	Culicidae	Aedes	Aedes aegypti	SPECIES	53.51000	-2.98000	3351352296

№	Order	Family	Genus	Species	Taxon Rank	Latitude	Longitude	GBIF_ID
1	Diptera	Culicidae	Anopheles	Anopheles stephensi	SPECIES	6.73000	44.27000	3817947835
2	Diptera	Culicidae	Anopheles	Anopheles stephensi	SPECIES	9.03000	79.88000	3351196667
3	Diptera	Culicidae	Anopheles	Anopheles stephensi	SPECIES	9.07000	79.84000	3348520470
4	Diptera	Culicidae	Anopheles	Anopheles stephensi	SPECIES	9.08000	79.82000	3348451429
5	Diptera	Culicidae	Anopheles	Anopheles stephensi	SPECIES	9.08000	79.84000	3351094782
6	Diptera	Culicidae	Anopheles	Anopheles stephensi	SPECIES	9.42000	77.17000	3349084219
7	Diptera	Culicidae	Anopheles	Anopheles stephensi	SPECIES	9.91000	78.24000	3348987105
8	Diptera	Culicidae	Anopheles	Anopheles stephensi	SPECIES	10.22000	77.57000	3349071248
9	Diptera	Culicidae	Anopheles	Anopheles stephensi	SPECIES	10.48000	78.47000	3349170184
10	Diptera	Culicidae	Anopheles	Anopheles stephensi	SPECIES	11.02000	76.95000	3351599238
11	Diptera	Culicidae	Anopheles	Anopheles stephensi	SPECIES	11.10000	77.34000	3351594156
12	Diptera	Culicidae	Anopheles	Anopheles stephensi	SPECIES	11.14000	78.09000	3351790061
13	Diptera	Culicidae	Anopheles	Anopheles stephensi	SPECIES	11.18000	76.35000	3349174003
14	Diptera	Culicidae	Anopheles	Anopheles stephensi	SPECIES	11.34000	77.71000	3351640357
15	Diptera	Culicidae	Anopheles	Anopheles stephensi	SPECIES	11.40000	76.69000	3347906282
16	Diptera	Culicidae	Anopheles	Anopheles stephensi	SPECIES	11.48000	43.23000	3347255269
17	Diptera	Culicidae	Anopheles	Anopheles stephensi	SPECIES	11.66000	78.14000	3351589359
18	Diptera	Culicidae	Anopheles	Anopheles stephensi	SPECIES	11.80000	76.00000	3351888239
19	Diptera	Culicidae	Anopheles	Anopheles stephensi	SPECIES	12.20000	78.31000	3349165818
20	Diptera	Culicidae	Anopheles	Anopheles stephensi	SPECIES	12.30000	78.13000	3349157006
21	Diptera	Culicidae	Anopheles	Anopheles stephensi	SPECIES	13.30975	101.04814	4145854583
22	Diptera	Culicidae	Anopheles	Anopheles stephensi	SPECIES	13.49568	101.79434	4145854258
23	Diptera	Culicidae	Anopheles	Anopheles stephensi	SPECIES	14.36274	101.75755	4145854935
24	Diptera	Culicidae	Anopheles	Anopheles stephensi	SPECIES	14.44955	103.44497	4145857885
25	Diptera	Culicidae	Anopheles	Anopheles stephensi	SPECIES	16.04819	98.84433	4145858915
26	Diptera	Culicidae	Anopheles	Anopheles stephensi	SPECIES	17.04043	98.49219	4145858400
27	Diptera	Culicidae	Anopheles	Anopheles stephensi	SPECIES	17.37520	78.43780	2249598383
28	Diptera	Culicidae	Anopheles	Anopheles stephensi	SPECIES	17.38000	78.44000	3348989799
29	Diptera	Culicidae	Anopheles	Anopheles stephensi	SPECIES	17.91431	98.40958	4145848770
30	Diptera	Culicidae	Anopheles	Anopheles stephensi	SPECIES	19.30000	84.88000	3349243838
31	Diptera	Culicidae	Anopheles	Anopheles stephensi	SPECIES	19.32000	84.79000	3351799113
32	Diptera	Culicidae	Anopheles	Anopheles stephensi	SPECIES	24.18000	55.74000	3349121409
33	Diptera	Culicidae	Anopheles	Anopheles stephensi	SPECIES	25.47000	51.37000	3818082454
34	Diptera	Culicidae	Anopheles	Anopheles stephensi	SPECIES	30.20000	71.43000	3347208276
35	Diptera	Culicidae	Anopheles	Anopheles stephensi	SPECIES	30.66000	73.02000	3347216445
36	Diptera	Culicidae	Anopheles	Anopheles stephensi	SPECIES	30.97000	74.03000	3347208278
37	Diptera	Culicidae	Anopheles	Anopheles stephensi	SPECIES	30.98000	72.88000	3347348316
38	Diptera	Culicidae	Anopheles	Anopheles stephensi	SPECIES	30.99000	74.28000	3347328386
39	Diptera	Culicidae	Anopheles	Anopheles stephensi	SPECIES	31.08000	72.97000	3347282420
40	Diptera	Culicidae	Anopheles	Anopheles stephensi	SPECIES	31.14000	72.67000	3347263277
41	Diptera	Culicidae	Anopheles	Anopheles stephensi	SPECIES	31.14100	72.67400	2250858024

№	Order	Family	Genus	Species	Taxon Rank	Latitude	Longitude	GBIF_ID
42	Diptera	Culicidae	Anopheles	Anopheles stephensi	SPECIES	31.48000	74.27000	3350438380
43	Diptera	Culicidae	Anopheles	Anopheles stephensi	SPECIES	31.49000	74.25000	3347216446
44	Diptera	Culicidae	Anopheles	Anopheles stephensi	SPECIES	31.49000	74.27000	3347315346
45	Diptera	Culicidae	Anopheles	Anopheles stephensi	SPECIES	31.50000	74.25000	3347166264
46	Diptera	Culicidae	Anopheles	Anopheles stephensi	SPECIES	31.57000	74.33000	3347324355
47	Diptera	Culicidae	Anopheles	Anopheles stephensi	SPECIES	31.58000	74.33000	3347315345
48	Diptera	Culicidae	Anopheles	Anopheles stephensi	SPECIES	31.68000	73.54000	3347209192
49	Diptera	Culicidae	Anopheles	Anopheles stephensi	SPECIES	31.69000	73.92000	3347191114
50	Diptera	Culicidae	Anopheles	Anopheles stephensi	SPECIES	31.70000	73.98000	3347282419
51	Diptera	Culicidae	Anopheles	Anopheles stephensi	SPECIES	31.75000	73.83000	3347248313
52	Diptera	Culicidae	Anopheles	Anopheles stephensi	SPECIES	32.48000	74.35000	3347314464
53	Diptera	Culicidae	Anopheles	Anopheles stephensi	SPECIES	32.77000	-79.98000	4066964790
54	Diptera	Culicidae	Anopheles	Anopheles stephensi	SPECIES	34.01000	71.49000	3347195278
55	Diptera	Culicidae	Anopheles	Anopheles stephensi	SPECIES	34.14000	71.70000	3350369471
56	Diptera	Culicidae	Anopheles	Anopheles stephensi	SPECIES	34.15000	71.72000	3350438379
57	Diptera	Culicidae	Anopheles	Anopheles stephensi	SPECIES	38.98067	-77.10026	3052569310

№	Order	Family	Genus	Species	Taxon Rank	Latitude	Longitude	GBIF_ID
1	Diptera	Culicidae	Culex	Culex pipiens	SPECIES	-38.53450	143.54440	3894410577
2	Diptera	Culicidae	Culex	Culex pipiens	SUBSPECIES	-38.07000	147.24000	3350865161
3	Diptera	Culicidae	Culex	Culex pipiens	SPECIES	-37.81667	144.96666	177216447
4	Diptera	Culicidae	Culex	Culex pipiens	SPECIES	-36.96150	144.49100	3895355665
5	Diptera	Culicidae	Culex	Culex pipiens	SPECIES	-36.08260	150.13090	1916659486
6	Diptera	Culicidae	Culex	Culex pipiens	SUBSPECIES	-35.73000	143.94000	3818302077
7	Diptera	Culicidae	Culex	Culex pipiens	SPECIES	-35.64120	150.12690	3901679418
8	Diptera	Culicidae	Culex	Culex pipiens	SUBSPECIES	-35.39000	149.38000	3351725852
9	Diptera	Culicidae	Culex	Culex pipiens	SPECIES	-35.33333	143.55000	177216446
10	Diptera	Culicidae	Culex	Culex pipiens	SUBSPECIES	-35.06459	138.86240	3039458036
11	Diptera	Culicidae	Culex	Culex pipiens	SPECIES	-35.04262	138.76193	4103094398
12	Diptera	Culicidae	Culex	Culex pipiens	SPECIES	-34.79146	138.78026	3743278637
13	Diptera	Culicidae	Culex	Culex pipiens	SPECIES	-33.98000	19.06000	1299998646
14	Diptera	Culicidae	Culex	Culex pipiens	SPECIES	-33.97000	18.48000	1299985984
15	Diptera	Culicidae	Culex	Culex pipiens	SPECIES	-33.83310	148.67650	3901660782
16	Diptera	Culicidae	Culex	Culex pipiens	SPECIES	-33.81330	151.12400	3901660516
17	Diptera	Culicidae	Culex	Culex pipiens	SPECIES	-33.74510	151.03790	3901720639
18	Diptera	Culicidae	Culex	Culex pipiens	SPECIES	-33.73333	151.26666	177216448
19	Diptera	Culicidae	Culex	Culex pipiens	SPECIES	-33.63000	18.98000	1300042884
20	Diptera	Culicidae	Culex	Culex pipiens	SPECIES	-32.76667	151.75000	177216444
21	Diptera	Culicidae	Culex	Culex pipiens	SPECIES	-31.98306	115.85361	177216443
22	Diptera	Culicidae	Culex	Culex pipiens	SPECIES	-29.98333	30.96667	1224613144
23	Diptera	Culicidae	Culex	Culex pipiens	SPECIES	-29.89056	30.97778	1275275847
24	Diptera	Culicidae	Culex	Culex pipiens	SPECIES	-29.10500	26.33000	3441596484
25	Diptera	Culicidae	Culex	Culex pipiens	SPECIES	-29.05300	26.21400	3442637572
26	Diptera	Culicidae	Culex	Culex pipiens	SPECIES	-27.71250	32.25139	1275275845
27	Diptera	Culicidae	Culex	Culex pipiens	SPECIES	-27.51667	144.31667	177216445
28	Diptera	Culicidae	Culex	Culex pipiens	SPECIES	-26.20000	32.13000	3987180731
29	Diptera	Culicidae	Culex	Culex pipiens	SPECIES	-25.99000	28.03000	3987194723
30	Diptera	Culicidae	Culex	Culex pipiens	SPECIES	-25.98333	32.70000	1275275841
31	Diptera	Culicidae	Culex	Culex pipiens	SPECIES	-25.84000	28.43000	3987357036
32	Diptera	Culicidae	Culex	Culex pipiens	SPECIES	-25.16000	32.00000	1300007026
33	Diptera	Culicidae	Culex	Culex pipiens	SPECIES	-24.42000	27.60000	3987183861
34	Diptera	Culicidae	Culex	Culex pipiens	SPECIES	-23.94000	28.86000	3987194722
35	Diptera	Culicidae	Culex	Culex pipiens	SPECIES	-21.30364	47.63869	732520473
36	Diptera	Culicidae	Culex	Culex pipiens	SPECIES	-21.13317	55.53241	3847000626
37	Diptera	Culicidae	Culex	Culex pipiens	SPECIES	-19.28280	146.80100	2632922490
38	Diptera	Culicidae	Culex	Culex pipiens	SPECIES	-18.83333	47.43333	732520532
39	Diptera	Culicidae	Culex	Culex pipiens	SPECIES	-17.61000	-149.31000	3348198156
40	Diptera	Culicidae	Culex	Culex pipiens	SPECIES	-17.51000	-149.82000	3348292284
41	Diptera	Culicidae	Culex	Culex pipiens	SPECIES	-17.49000	-149.83000	3348314249

№	Order	Family	Genus	Species	Taxon Rank	Latitude	Longitude	GBIF_ID
42	Diptera	Culicidae	Culex	Culex pipiens	SPECIES	-16.84000	-151.36000	3348180991
43	Diptera	Culicidae	Culex	Culex pipiens	SPECIES	-12.82060	45.14743	3846950104
44	Diptera	Culicidae	Culex	Culex pipiens	SPECIES	-11.63330	169.83330	3059297324
45	Diptera	Culicidae	Culex	Culex pipiens	SPECIES	-4.17000	39.45000	3349200215
46	Diptera	Culicidae	Culex	Culex pipiens	SPECIES	-4.14000	39.32000	3349016124
47	Diptera	Culicidae	Culex	Culex pipiens	SPECIES	-4.00433	35.77960	4065055380
48	Diptera	Culicidae	Culex	Culex pipiens	SPECIES	-3.99549	35.78000	4065055883
49	Diptera	Culicidae	Culex	Culex pipiens	SPECIES	-3.98352	36.07590	4065055915
50	Diptera	Culicidae	Culex	Culex pipiens	SPECIES	-3.97070	35.75010	4065055389
51	Diptera	Culicidae	Culex	Culex pipiens	SPECIES	-3.78895	36.04540	4065055605
52	Diptera	Culicidae	Culex	Culex pipiens	SPECIES	-3.77435	35.97300	4065055750
53	Diptera	Culicidae	Culex	Culex pipiens	SPECIES	-3.77035	35.96890	4065056053
54	Diptera	Culicidae	Culex	Culex pipiens	SPECIES	-3.70148	35.91830	4065055985
55	Diptera	Culicidae	Culex	Culex pipiens	SPECIES	-3.39729	35.86990	4065055504
56	Diptera	Culicidae	Culex	Culex pipiens	SPECIES	-3.39652	35.87010	4065055431
57	Diptera	Culicidae	Culex	Culex pipiens	SPECIES	-3.38218	35.85700	4065056159
58	Diptera	Culicidae	Culex	Culex pipiens	SPECIES	-3.37778	35.86590	4065055502
59	Diptera	Culicidae	Culex	Culex pipiens	SPECIES	-3.37497	35.85580	4065055331
60	Diptera	Culicidae	Culex	Culex pipiens	SPECIES	-3.37298	35.59460	4065055407
61	Diptera	Culicidae	Culex	Culex pipiens	SPECIES	-3.37076	35.86800	4065056069
62	Diptera	Culicidae	Culex	Culex pipiens	SPECIES	-3.37027	35.86760	4065056005
63	Diptera	Culicidae	Culex	Culex pipiens	SPECIES	-3.36825	35.85440	4065056071
64	Diptera	Culicidae	Culex	Culex pipiens	SPECIES	-3.35425	35.85590	4065055507
65	Diptera	Culicidae	Culex	Culex pipiens	SPECIES	-3.35257	35.62510	4065055554
66	Diptera	Culicidae	Culex	Culex pipiens	SPECIES	-3.34447	35.65040	4065055542
67	Diptera	Culicidae	Culex	Culex pipiens	SPECIES	-3.33573	35.85780	4065055305
68	Diptera	Culicidae	Culex	Culex pipiens	SPECIES	-1.68000	35.31000	3349113257
69	Diptera	Culicidae	Culex	Culex pipiens	SPECIES	-1.28330	36.81670	177216450
70	Diptera	Culicidae	Culex	Culex pipiens	SPECIES	-0.61000	34.10000	3351002084
71	Diptera	Culicidae	Culex	Culex pipiens	SPECIES	-0.45000	34.06000	3350953888
72	Diptera	Culicidae	Culex	Culex pipiens	SPECIES	-0.42000	34.20000	3348136088
73	Diptera	Culicidae	Culex	Culex pipiens	SPECIES	3.89847	11.51985	732520599
74	Diptera	Culicidae	Culex	Culex pipiens	SPECIES	4.68400	6.36593	226110897
75	Diptera	Culicidae	Culex	Culex pipiens	SPECIES	5.96721	10.14932	226110882
76	Diptera	Culicidae	Culex	Culex pipiens	SPECIES	6.45049	3.39921	226110893
77	Diptera	Culicidae	Culex	Culex pipiens	SPECIES	6.90045	3.01587	226110887
78	Diptera	Culicidae	Culex	Culex pipiens	SPECIES	6.98378	7.44927	226110886
79	Diptera	Culicidae	Culex	Culex pipiens	SPECIES	7.78370	4.48256	226110898
80	Diptera	Culicidae	Culex	Culex pipiens	SPECIES	7.80037	6.73259	226110894
81	Diptera	Culicidae	Culex	Culex pipiens	SPECIES	9.10025	6.01591	226110884
82	Diptera	Culicidae	Culex	Culex pipiens	SPECIES	9.30023	5.06590	226110896

№	Order	Family	Genus	Species	Taxon Rank	Latitude	Longitude	GBIF_ID
83	Diptera	Culicidae	Culex	Culex pipiens	SPECIES	9.86685	8.89929	226110888
84	Diptera	Culicidae	Culex	Culex pipiens	SPECIES	10.00000	-61.00000	3328467524
85	Diptera	Culicidae	Culex	Culex pipiens	SPECIES	10.28348	9.81597	226110883
86	Diptera	Culicidae	Culex	Culex pipiens	SPECIES	10.46680	7.39927	226110890
87	Diptera	Culicidae	Culex	Culex pipiens	SPECIES	10.50013	7.44927	226110889
88	Diptera	Culicidae	Culex	Culex pipiens	SPECIES	10.71677	7.53260	226110892
89	Diptera	Culicidae	Culex	Culex pipiens	SPECIES	11.13290	75.88920	3442407459
90	Diptera	Culicidae	Culex	Culex pipiens	SPECIES	11.51672	13.69937	226110881
91	Diptera	Culicidae	Culex	Culex pipiens	SPECIES	11.66669	10.16598	226110880
92	Diptera	Culicidae	Culex	Culex pipiens	SPECIES	11.80000	76.00000	3969179895
93	Diptera	Culicidae	Culex	Culex pipiens	SPECIES	11.80000	76.00400	4039073868
94	Diptera	Culicidae	Culex	Culex pipiens	SPECIES	11.85002	13.08269	226110895
95	Diptera	Culicidae	Culex	Culex pipiens	SPECIES	12.04999	8.53261	226110891
96	Diptera	Culicidae	Culex	Culex pipiens	SPECIES	12.46661	4.18254	226110885
97	Diptera	Culicidae	Culex	Culex pipiens	SPECIES	17.35000	78.53000	3348908596
98	Diptera	Culicidae	Culex	Culex pipiens	SPECIES	18.16145	-66.99711	1993809177
99	Diptera	Culicidae	Culex	Culex pipiens	SPECIES	18.32105	-65.81972	1993808525
100	Diptera	Culicidae	Culex	Culex pipiens	SPECIES	19.54800	-96.90351	4096440305
101	Diptera	Culicidae	Culex	Culex pipiens	SPECIES	19.64000	96.20000	3818537299
102	Diptera	Culicidae	Culex	Culex pipiens	SPECIES	22.33208	31.59553	226112378
103	Diptera	Culicidae	Culex	Culex pipiens	SPECIES	22.33208	31.58858	226112396
104	Diptera	Culicidae	Culex	Culex pipiens	SPECIES	22.59488	120.33527	1945426298
105	Diptera	Culicidae	Culex	Culex pipiens	SPECIES	22.60677	120.30918	1946312097
106	Diptera	Culicidae	Culex	Culex pipiens	SPECIES	23.04611	-99.15417	1421477380
107	Diptera	Culicidae	Culex	Culex pipiens	SUBSPECIES	23.45000	90.24000	4152808321
108	Diptera	Culicidae	Culex	Culex pipiens	SUBSPECIES	23.47000	90.24000	4152770888
109	Diptera	Culicidae	Culex	Culex pipiens	SPECIES	23.62061	120.26128	4111755076
110	Diptera	Culicidae	Culex	Culex pipiens	SPECIES	23.71895	-97.76843	1418704006
111	Diptera	Culicidae	Culex	Culex pipiens	SPECIES	23.73611	-99.14611	1421477666
112	Diptera	Culicidae	Culex	Culex pipiens	SPECIES	23.74303	-97.75920	1418703919
113	Diptera	Culicidae	Culex	Culex pipiens	SPECIES	23.74737	32.49340	226111662
114	Diptera	Culicidae	Culex	Culex pipiens	SPECIES	23.78000	90.40000	3351500549
115	Diptera	Culicidae	Culex	Culex pipiens	SPECIES	23.88000	90.27000	4038898902
116	Diptera	Culicidae	Culex	Culex pipiens	SPECIES	23.92917	-98.93750	1421475328
117	Diptera	Culicidae	Culex	Culex pipiens	SPECIES	23.94878	35.63314	226112349
118	Diptera	Culicidae	Culex	Culex pipiens	SPECIES	23.97378	32.96640	226112044
119	Diptera	Culicidae	Culex	Culex pipiens	SPECIES	23.97378	32.89140	226112297
120	Diptera	Culicidae	Culex	Culex pipiens	SPECIES	23.97893	121.59462	4126677706
121	Diptera	Culicidae	Culex	Culex pipiens	SPECIES	23.98211	32.96640	226112198
122	Diptera	Culicidae	Culex	Culex pipiens	SPECIES	23.98628	32.84139	226112224
123	Diptera	Culicidae	Culex	Culex pipiens	SPECIES	24.03881	32.87680	226111234

№	Order	Family	Genus	Species	Taxon Rank	Latitude	Longitude	GBIF_ID
124	Diptera	Culicidae	Culex	Culex pipiens	SPECIES	24.06594	32.91809	226111270
125	Diptera	Culicidae	Culex	Culex pipiens	SPECIES	24.06684	32.91809	226111351
126	Diptera	Culicidae	Culex	Culex pipiens	SPECIES	24.08490	32.91020	226111531
127	Diptera	Culicidae	Culex	Culex pipiens	SPECIES	24.08761	32.91414	226111469
128	Diptera	Culicidae	Culex	Culex pipiens	SPECIES	24.11380	32.90526	226111568
129	Diptera	Culicidae	Culex	Culex pipiens	SPECIES	24.12102	32.89837	226111446
130	Diptera	Culicidae	Culex	Culex pipiens	SPECIES	24.13457	32.89048	226111452
131	Diptera	Culicidae	Culex	Culex pipiens	SPECIES	24.21585	32.87958	226111414
132	Diptera	Culicidae	Culex	Culex pipiens	SPECIES	24.21585	32.88648	226111667
133	Diptera	Culicidae	Culex	Culex pipiens	SPECIES	24.21765	32.87958	226111388
134	Diptera	Culicidae	Culex	Culex pipiens	SPECIES	24.21766	32.88352	226110938
135	Diptera	Culicidae	Culex	Culex pipiens	SPECIES	24.39561	32.92971	226111660
136	Diptera	Culicidae	Culex	Culex pipiens	SPECIES	24.47058	32.94546	226111551
137	Diptera	Culicidae	Culex	Culex pipiens	SPECIES	24.48972	-97.74500	1418704059
138	Diptera	Culicidae	Culex	Culex pipiens	SPECIES	24.49578	-97.74128	1418704130
139	Diptera	Culicidae	Culex	Culex pipiens	SPECIES	24.53389	-97.73503	1418704076
140	Diptera	Culicidae	Culex	Culex pipiens	SPECIES	24.57535	32.94936	226111450
141	Diptera	Culicidae	Culex	Culex pipiens	SPECIES	24.88238	32.88785	226111434
142	Diptera	Culicidae	Culex	Culex pipiens	SPECIES	24.96458	32.90660	226111432
143	Diptera	Culicidae	Culex	Culex pipiens	SPECIES	25.06546	34.88312	226112341
144	Diptera	Culicidae	Culex	Culex pipiens	SPECIES	25.17272	-97.67072	1418703939
145	Diptera	Culicidae	Culex	Culex pipiens	SPECIES	25.24103	-97.43958	1418704004
146	Diptera	Culicidae	Culex	Culex pipiens	SPECIES	25.56111	-103.49833	1421478376
147	Diptera	Culicidae	Culex	Culex pipiens	SPECIES	25.65864	-100.17594	1421476295
148	Diptera	Culicidae	Culex	Culex pipiens	SPECIES	25.70067	-100.29728	1421476717
149	Diptera	Culicidae	Culex	Culex pipiens	SPECIES	25.72136	-97.16914	1418703974
150	Diptera	Culicidae	Culex	Culex pipiens	SPECIES	25.72922	-100.30731	1421477714
151	Diptera	Culicidae	Culex	Culex pipiens	SPECIES	25.73258	-100.30794	1421477020
152	Diptera	Culicidae	Culex	Culex pipiens	SPECIES	25.74139	-100.45056	1421474570
153	Diptera	Culicidae	Culex	Culex pipiens	SPECIES	25.78472	-100.05083	1421477531
154	Diptera	Culicidae	Culex	Culex pipiens	SPECIES	25.78513	-100.29210	1421474170
155	Diptera	Culicidae	Culex	Culex pipiens	SPECIES	25.78561	-97.15739	1418704144
156	Diptera	Culicidae	Culex	Culex pipiens	SPECIES	25.80814	-100.37347	1421474848
157	Diptera	Culicidae	Culex	Culex pipiens	SPECIES	25.96250	-100.29167	1421475264
158	Diptera	Culicidae	Culex	Culex pipiens	SPECIES	26.11548	34.26643	226112345
159	Diptera	Culicidae	Culex	Culex pipiens	SPECIES	26.11548	34.23310	226112347
160	Diptera	Culicidae	Culex	Culex pipiens	SPECIES	26.43056	-99.14833	1421475646
161	Diptera	Culicidae	Culex	Culex pipiens	SPECIES	27.27300	-82.11650	3030840097
162	Diptera	Culicidae	Culex	Culex pipiens	SPECIES	27.34710	-82.24350	3030792703
163	Diptera	Culicidae	Culex	Culex pipiens	SPECIES	27.34970	-82.15760	3030336741
164	Diptera	Culicidae	Culex	Culex pipiens	SPECIES	27.40650	-82.50450	3030792229

№	Order	Family	Genus	Species	Taxon Rank	Latitude	Longitude	GBIF_ID
165	Diptera	Culicidae	Culex	Culex pipiens	SPECIES	27.40810	-82.26030	3030336478
166	Diptera	Culicidae	Culex	Culex pipiens	SPECIES	27.41220	-82.28760	3030791659
167	Diptera	Culicidae	Culex	Culex pipiens	SPECIES	27.41950	-82.42620	3030858203
168	Diptera	Culicidae	Culex	Culex pipiens	SPECIES	27.42660	-82.52470	3030717703
169	Diptera	Culicidae	Culex	Culex pipiens	SPECIES	27.43890	-82.43860	3030436171
170	Diptera	Culicidae	Culex	Culex pipiens	SPECIES	27.43940	-82.54890	3030359461
171	Diptera	Culicidae	Culex	Culex pipiens	SPECIES	27.44000	-80.32000	3328467518
172	Diptera	Culicidae	Culex	Culex pipiens	SPECIES	27.47510	-82.55130	3030416070
173	Diptera	Culicidae	Culex	Culex pipiens	SPECIES	27.48460	-82.40160	3030850605
174	Diptera	Culicidae	Culex	Culex pipiens	SPECIES	27.48890	-82.59210	3030411869
175	Diptera	Culicidae	Culex	Culex pipiens	SPECIES	27.48940	-82.36170	3030422676
176	Diptera	Culicidae	Culex	Culex pipiens	SPECIES	27.50310	-82.65740	3030375973
177	Diptera	Culicidae	Culex	Culex pipiens	SPECIES	27.52880	-82.59050	3030411979
178	Diptera	Culicidae	Culex	Culex pipiens	SPECIES	27.53330	-82.56570	3030782874
179	Diptera	Culicidae	Culex	Culex pipiens	SPECIES	27.56500	-82.42490	3030915050
180	Diptera	Culicidae	Culex	Culex pipiens	SPECIES	27.57090	-82.13780	3030898827
181	Diptera	Culicidae	Culex	Culex pipiens	SPECIES	27.57840	-82.48370	3030495073
182	Diptera	Culicidae	Culex	Culex pipiens	SPECIES	27.58320	-82.14350	3801539200
183	Diptera	Culicidae	Culex	Culex pipiens	SPECIES	27.59090	-82.54800	3030526245
184	Diptera	Culicidae	Culex	Culex pipiens	SPECIES	27.60310	-82.53570	3030390824
185	Diptera	Culicidae	Culex	Culex pipiens	SPECIES	27.61640	-82.53530	3030898189
186	Diptera	Culicidae	Culex	Culex pipiens	SPECIES	27.62800	-82.49680	3030410784
187	Diptera	Culicidae	Culex	Culex pipiens	SPECIES	27.63950	-82.41710	3030473263
188	Diptera	Culicidae	Culex	Culex pipiens	SPECIES	27.64050	-82.55270	3030422593
189	Diptera	Culicidae	Culex	Culex pipiens	SPECIES	28.92472	32.33286	226111092
190	Diptera	Culicidae	Culex	Culex pipiens	SPECIES	29.17801	25.50366	226112422
191	Diptera	Culicidae	Culex	Culex pipiens	SPECIES	29.20718	25.54116	226112038
192	Diptera	Culicidae	Culex	Culex pipiens	SPECIES	29.26135	25.31337	226112026
193	Diptera	Culicidae	Culex	Culex pipiens	SPECIES	29.31081	30.69435	226111155
194	Diptera	Culicidae	Culex	Culex pipiens	SPECIES	29.31466	30.65617	226111153
195	Diptera	Culicidae	Culex	Culex pipiens	SPECIES	29.42152	30.84024	226111117
196	Diptera	Culicidae	Culex	Culex pipiens	SPECIES	29.63217	34.98305	226112738
197	Diptera	Culicidae	Culex	Culex pipiens	SPECIES	29.65000	-82.33300	43173069
198	Diptera	Culicidae	Culex	Culex pipiens	SPECIES	29.86480	31.22410	226110956
199	Diptera	Culicidae	Culex	Culex pipiens	SPECIES	29.86497	31.23652	226111031
200	Diptera	Culicidae	Culex	Culex pipiens	SPECIES	29.86663	31.22614	226111018
201	Diptera	Culicidae	Culex	Culex pipiens	SPECIES	30.01555	32.56637	226112143
202	Diptera	Culicidae	Culex	Culex pipiens	SPECIES	30.05154	31.09321	226112015
203	Diptera	Culicidae	Culex	Culex pipiens	SPECIES	30.07761	31.28255	226111685
204	Diptera	Culicidae	Culex	Culex pipiens	SPECIES	30.07849	31.28150	226111748
205	Diptera	Culicidae	Culex	Culex pipiens	SPECIES	30.23565	31.29436	226111964

№	Order	Family	Genus	Species	Taxon Rank	Latitude	Longitude	GBIF_ID
206	Diptera	Culicidae	Culex	Culex pipiens	SPECIES	30.26700	-97.75000	43172938
207	Diptera	Culicidae	Culex	Culex pipiens	SPECIES	30.35914	30.36434	226111038
208	Diptera	Culicidae	Culex	Culex pipiens	SPECIES	30.37241	30.35150	226111036
209	Diptera	Culicidae	Culex	Culex pipiens	SPECIES	30.56700	-84.95000	43173071
210	Diptera	Culicidae	Culex	Culex pipiens	SPECIES	30.56735	32.19885	226111090
211	Diptera	Culicidae	Culex	Culex pipiens	SPECIES	30.58359	32.19871	226111078
212	Diptera	Culicidae	Culex	Culex pipiens	SPECIES	30.74185	32.25800	226111065
213	Diptera	Culicidae	Culex	Culex pipiens	SPECIES	30.74186	32.25904	226111072
214	Diptera	Culicidae	Culex	Culex pipiens	SPECIES	30.74816	32.25690	226111054
215	Diptera	Culicidae	Culex	Culex pipiens	SPECIES	30.81552	34.79970	226112491
216	Diptera	Culicidae	Culex	Culex pipiens	SPECIES	30.81552	34.76637	226112761
217	Diptera	Culicidae	Culex	Culex pipiens	SPECIES	30.83300	-81.55000	43173621
218	Diptera	Culicidae	Culex	Culex pipiens	SPECIES	30.95997	35.36639	226112699
219	Diptera	Culicidae	Culex	Culex pipiens	SPECIES	31.01553	35.36639	226112701
220	Diptera	Culicidae	Culex	Culex pipiens	SPECIES	31.12711	30.13262	226111171
221	Diptera	Culicidae	Culex	Culex pipiens	SPECIES	31.22030	32.29943	226111928
222	Diptera	Culicidae	Culex	Culex pipiens	SPECIES	31.23219	34.49970	226112581
223	Diptera	Culicidae	Culex	Culex pipiens	SPECIES	31.24166	29.98447	226111216
224	Diptera	Culicidae	Culex	Culex pipiens	SPECIES	31.24191	32.29087	226111829
225	Diptera	Culicidae	Culex	Culex pipiens	SPECIES	31.25023	32.32861	226111883
226	Diptera	Culicidae	Culex	Culex pipiens	SPECIES	31.30000	121.57000	3350966900
227	Diptera	Culicidae	Culex	Culex pipiens	SPECIES	31.30720	34.49136	226112558
228	Diptera	Culicidae	Culex	Culex pipiens	SPECIES	31.40744	30.42378	226111191
229	Diptera	Culicidae	Culex	Culex pipiens	SPECIES	31.43220	34.59970	226112645
230	Diptera	Culicidae	Culex	Culex pipiens	SPECIES	31.43220	34.99971	226112693
231	Diptera	Culicidae	Culex	Culex pipiens	SPECIES	31.49887	34.76637	226112495
232	Diptera	Culicidae	Culex	Culex pipiens	SPECIES	31.49887	34.59970	226112639
233	Diptera	Culicidae	Culex	Culex pipiens	SPECIES	31.54887	34.73859	226112675
234	Diptera	Culicidae	Culex	Culex pipiens	SPECIES	31.63220	34.58859	226112620
235	Diptera	Culicidae	Culex	Culex pipiens	SPECIES	31.70443	34.75803	226112657
236	Diptera	Culicidae	Culex	Culex pipiens	SPECIES	31.76917	35.21278	4017641262
237	Diptera	Culicidae	Culex	Culex pipiens	SPECIES	31.78221	35.03304	226112602
238	Diptera	Culicidae	Culex	Culex pipiens	SPECIES	31.81713	-81.61037	2466111920
239	Diptera	Culicidae	Culex	Culex pipiens	SPECIES	32.10000	74.17000	3347166268
240	Diptera	Culicidae	Culex	Culex pipiens	SPECIES	32.60793	-106.85916	2441826983
241	Diptera	Culicidae	Culex	Culex pipiens	SPECIES	32.67600	-16.90400	4069455370
242	Diptera	Culicidae	Culex	Culex pipiens	SPECIES	32.77000	-79.98000	4066968687
243	Diptera	Culicidae	Culex	Culex pipiens	SPECIES	32.85230	35.75254	1562933413
244	Diptera	Culicidae	Culex	Culex pipiens	SPECIES	32.89334	35.36638	226112899
245	Diptera	Culicidae	Culex	Culex pipiens	SPECIES	32.91001	35.37055	226112891
246	Diptera	Culicidae	Culex	Culex pipiens	SPECIES	32.95664	35.73739	2237435364

№	Order	Family	Genus	Species	Taxon Rank	Latitude	Longitude	GBIF_ID
247	Diptera	Culicidae	Culex	Culex pipiens	SPECIES	33.06695	35.22332	226112482
248	Diptera	Culicidae	Culex	Culex pipiens	SPECIES	33.13918	35.74417	226112477
249	Diptera	Culicidae	Culex	Culex pipiens	SPECIES	33.16140	35.72889	226112454
250	Diptera	Culicidae	Culex	Culex pipiens	SPECIES	33.16140	35.72750	226112459
251	Diptera	Culicidae	Culex	Culex pipiens	SPECIES	33.23223	35.69972	226112774
252	Diptera	Culicidae	Culex	Culex pipiens	SPECIES	33.23779	35.71639	226112878
253	Diptera	Culicidae	Culex	Culex pipiens	SPECIES	33.42337	-111.93849	3443938308
254	Diptera	Culicidae	Culex	Culex pipiens	SUBSPECIES	33.49627	126.80492	3001423270
255	Diptera	Culicidae	Culex	Culex pipiens	SPECIES	33.58300	-101.98300	43172790
256	Diptera	Culicidae	Culex	Culex pipiens	SUBSPECIES	33.80000	-118.30000	3417796332
257	Diptera	Culicidae	Culex	Culex pipiens	SPECIES	34.00919	-81.07484	4066960931
258	Diptera	Culicidae	Culex	Culex pipiens	SPECIES	34.00930	-81.06963	4066962933
259	Diptera	Culicidae	Culex	Culex pipiens	SPECIES	34.00965	-81.07558	4066961937
260	Diptera	Culicidae	Culex	Culex pipiens	SPECIES	34.00993	-81.07204	4066959928
261	Diptera	Culicidae	Culex	Culex pipiens	SPECIES	34.00999	-81.07036	4066962932
262	Diptera	Culicidae	Culex	Culex pipiens	SUBSPECIES	34.57714	126.75908	3444226762
263	Diptera	Culicidae	Culex	Culex pipiens	SPECIES	34.59000	32.97492	3714036498
264	Diptera	Culicidae	Culex	Culex pipiens	SPECIES	34.59000	32.97569	3714036962
265	Diptera	Culicidae	Culex	Culex pipiens	SPECIES	34.59000	32.97563	3714042143
266	Diptera	Culicidae	Culex	Culex pipiens	SUBSPECIES	34.59032	126.68985	3444546716
267	Diptera	Culicidae	Culex	Culex pipiens	SUBSPECIES	34.60361	126.67967	3444581521
268	Diptera	Culicidae	Culex	Culex pipiens	SUBSPECIES	34.61323	126.73848	3444540575
269	Diptera	Culicidae	Culex	Culex pipiens	SPECIES	34.64590	33.00370	3714049115
270	Diptera	Culicidae	Culex	Culex pipiens	SPECIES	34.64830	33.00010	3714036013
271	Diptera	Culicidae	Culex	Culex pipiens	SPECIES	34.64890	33.00490	3714034013
272	Diptera	Culicidae	Culex	Culex pipiens	SPECIES	34.64920	33.00280	3714033622
273	Diptera	Culicidae	Culex	Culex pipiens	SPECIES	34.65090	33.00690	3714032668
274	Diptera	Culicidae	Culex	Culex pipiens	SPECIES	34.65130	33.00730	3714051676
275	Diptera	Culicidae	Culex	Culex pipiens	SPECIES	34.65170	33.00460	3714037653
276	Diptera	Culicidae	Culex	Culex pipiens	SPECIES	34.65180	32.99920	3714033186
277	Diptera	Culicidae	Culex	Culex pipiens	SPECIES	34.65300	-82.83300	4066946820
278	Diptera	Culicidae	Culex	Culex pipiens	SPECIES	34.65306	-82.83409	4066958927
279	Diptera	Culicidae	Culex	Culex pipiens	SPECIES	34.65420	33.00710	3714032602
280	Diptera	Culicidae	Culex	Culex pipiens	SPECIES	34.65500	33.00100	3714037912
281	Diptera	Culicidae	Culex	Culex pipiens	SPECIES	34.65530	33.00910	3714034652
282	Diptera	Culicidae	Culex	Culex pipiens	SPECIES	34.65640	33.00450	3714034218
283	Diptera	Culicidae	Culex	Culex pipiens	SPECIES	34.67000	-82.84000	4066947823
284	Diptera	Culicidae	Culex	Culex pipiens	SPECIES	34.70440	33.00758	3714032814
285	Diptera	Culicidae	Culex	Culex pipiens	SPECIES	34.72940	32.51490	3714040987
286	Diptera	Culicidae	Culex	Culex pipiens	SUBSPECIES	34.76342	127.30655	3001932239
287	Diptera	Culicidae	Culex	Culex pipiens	SUBSPECIES	34.76802	127.05706	3001413092

№	Order	Family	Genus	Species	Taxon Rank	Latitude	Longitude	GBIF_ID
288	Diptera	Culicidae	Culex	Culex pipiens	SUBSPECIES	34.78680	127.35086	3001411137
289	Diptera	Culicidae	Culex	Culex pipiens	SUBSPECIES	34.79326	126.73131	3444552318
290	Diptera	Culicidae	Culex	Culex pipiens	SUBSPECIES	34.80409	127.06756	3001909244
291	Diptera	Culicidae	Culex	Culex pipiens	SUBSPECIES	34.81252	126.74116	3444236458
292	Diptera	Culicidae	Culex	Culex pipiens	SUBSPECIES	34.81519	127.34741	3001940211
293	Diptera	Culicidae	Culex	Culex pipiens	SUBSPECIES	34.87175	127.21308	3001421318
294	Diptera	Culicidae	Culex	Culex pipiens	SPECIES	34.87330	33.60880	3714050982
295	Diptera	Culicidae	Culex	Culex pipiens	SPECIES	34.89000	-82.72000	4066966680
296	Diptera	Culicidae	Culex	Culex pipiens	SPECIES	34.93333	-95.76944	1141858884
297	Diptera	Culicidae	Culex	Culex pipiens	SUBSPECIES	34.94831	127.06878	3001414037
298	Diptera	Culicidae	Culex	Culex pipiens	SPECIES	35.14142	33.38118	3714037374
299	Diptera	Culicidae	Culex	Culex pipiens	SPECIES	35.30017	38.83751	3443928308
300	Diptera	Culicidae	Culex	Culex pipiens	SPECIES	35.48670	24.06210	3893360007
301	Diptera	Culicidae	Culex	Culex pipiens	SUBSPECIES	35.71990	140.00924	1829762499
302	Diptera	Culicidae	Culex	Culex pipiens	SPECIES	35.92379	-84.43161	3926387532
303	Diptera	Culicidae	Culex	Culex pipiens	SPECIES	36.20000	35.86000	3350388707
304	Diptera	Culicidae	Culex	Culex pipiens	SPECIES	36.23573	-88.38874	2466117159
305	Diptera	Culicidae	Culex	Culex pipiens	SUBSPECIES	36.32373	136.44738	1828796105
306	Diptera	Culicidae	Culex	Culex pipiens	SPECIES	36.48000	27.06000	3348232335
307	Diptera	Culicidae	Culex	Culex pipiens	SPECIES	36.59300	-76.67900	3030320096
308	Diptera	Culicidae	Culex	Culex pipiens	SPECIES	36.59600	-76.87000	3030348284
309	Diptera	Culicidae	Culex	Culex pipiens	SPECIES	36.61000	-76.66200	3030424392
310	Diptera	Culicidae	Culex	Culex pipiens	SPECIES	36.61700	-76.57900	3030377452
311	Diptera	Culicidae	Culex	Culex pipiens	SPECIES	36.61800	-76.56000	3030461939
312	Diptera	Culicidae	Culex	Culex pipiens	SPECIES	36.64400	-76.54700	3030740216
313	Diptera	Culicidae	Culex	Culex pipiens	SPECIES	36.64500	-76.71300	3030344790
314	Diptera	Culicidae	Culex	Culex pipiens	SPECIES	36.68200	-76.78700	3030954137
315	Diptera	Culicidae	Culex	Culex pipiens	SPECIES	36.68700	-76.60600	3030387780
316	Diptera	Culicidae	Culex	Culex pipiens	SPECIES	36.69400	-76.58500	3030495011
317	Diptera	Culicidae	Culex	Culex pipiens	SPECIES	36.69500	-76.60000	3030847195
318	Diptera	Culicidae	Culex	Culex pipiens	SPECIES	36.70000	34.35000	3347266640
319	Diptera	Culicidae	Culex	Culex pipiens	SPECIES	36.71500	-76.54900	3030485291
320	Diptera	Culicidae	Culex	Culex pipiens	SPECIES	36.71500	-76.58400	3030958462
321	Diptera	Culicidae	Culex	Culex pipiens	SPECIES	36.71600	-76.65500	3030414461
322	Diptera	Culicidae	Culex	Culex pipiens	SPECIES	36.71600	-76.57700	3030424609
323	Diptera	Culicidae	Culex	Culex pipiens	SPECIES	36.71700	-76.64700	3030367852
324	Diptera	Culicidae	Culex	Culex pipiens	SPECIES	36.71800	-76.62200	3030954254
325	Diptera	Culicidae	Culex	Culex pipiens	SPECIES	36.72000	-76.58200	3030826584
326	Diptera	Culicidae	Culex	Culex pipiens	SPECIES	36.72300	-76.59500	3030452588
327	Diptera	Culicidae	Culex	Culex pipiens	SPECIES	36.72500	-76.53700	3030329559
328	Diptera	Culicidae	Culex	Culex pipiens	SPECIES	36.72500	-76.60300	3030405998

№	Order	Family	Genus	Species	Taxon Rank	Latitude	Longitude	GBIF_ID
329	Diptera	Culicidae	Culex	Culex pipiens	SPECIES	36.72600	-76.58500	3030366918
330	Diptera	Culicidae	Culex	Culex pipiens	SPECIES	36.72600	-76.72700	3030885193
331	Diptera	Culicidae	Culex	Culex pipiens	SPECIES	36.72600	-76.56300	3030891854
332	Diptera	Culicidae	Culex	Culex pipiens	SPECIES	36.72800	-76.54600	3030371016
333	Diptera	Culicidae	Culex	Culex pipiens	SPECIES	36.72800	-76.55500	3030425110
334	Diptera	Culicidae	Culex	Culex pipiens	SPECIES	36.73000	-76.57000	3030400097
335	Diptera	Culicidae	Culex	Culex pipiens	SPECIES	36.73000	-76.58700	3030400103
336	Diptera	Culicidae	Culex	Culex pipiens	SPECIES	36.73000	-76.58500	3030502439
337	Diptera	Culicidae	Culex	Culex pipiens	SPECIES	36.73200	-76.58800	3030358071
338	Diptera	Culicidae	Culex	Culex pipiens	SPECIES	36.73200	-76.58300	3030425216
339	Diptera	Culicidae	Culex	Culex pipiens	SPECIES	36.73200	-76.51800	3030863897
340	Diptera	Culicidae	Culex	Culex pipiens	SPECIES	36.73272	3.08571	3443931309
341	Diptera	Culicidae	Culex	Culex pipiens	SPECIES	36.73300	-76.60200	3030140991
342	Diptera	Culicidae	Culex	Culex pipiens	SPECIES	36.73300	-76.58600	3030154887
343	Diptera	Culicidae	Culex	Culex pipiens	SPECIES	36.73300	-76.57000	3030406022
344	Diptera	Culicidae	Culex	Culex pipiens	SPECIES	36.73400	-76.56200	3030896776
345	Diptera	Culicidae	Culex	Culex pipiens	SPECIES	36.73430	-76.55000	3030762270
346	Diptera	Culicidae	Culex	Culex pipiens	SPECIES	36.73500	-76.57900	3030149564
347	Diptera	Culicidae	Culex	Culex pipiens	SPECIES	36.73600	-76.58400	3030954155
348	Diptera	Culicidae	Culex	Culex pipiens	SPECIES	36.73800	-76.59000	3030415659
349	Diptera	Culicidae	Culex	Culex pipiens	SPECIES	36.74100	-76.57200	3030400316
350	Diptera	Culicidae	Culex	Culex pipiens	SPECIES	36.74200	-76.54700	3030534422
351	Diptera	Culicidae	Culex	Culex pipiens	SPECIES	36.74600	-76.58400	3030734546
352	Diptera	Culicidae	Culex	Culex pipiens	SPECIES	36.74700	-76.53400	3030901036
353	Diptera	Culicidae	Culex	Culex pipiens	SPECIES	36.74700	-76.53800	3030958391
354	Diptera	Culicidae	Culex	Culex pipiens	SPECIES	36.75000	-76.55400	3030406172
355	Diptera	Culicidae	Culex	Culex pipiens	SPECIES	36.75100	-76.61800	3030335250
356	Diptera	Culicidae	Culex	Culex pipiens	SPECIES	36.75200	-76.58500	3030891654
357	Diptera	Culicidae	Culex	Culex pipiens	SPECIES	36.75800	-76.57400	3030828247
358	Diptera	Culicidae	Culex	Culex pipiens	SPECIES	36.76400	-76.54200	3030388296
359	Diptera	Culicidae	Culex	Culex pipiens	SPECIES	36.76600	-76.58000	3030424985
360	Diptera	Culicidae	Culex	Culex pipiens	SUBSPECIES	36.76739	126.29242	3444340006
361	Diptera	Culicidae	Culex	Culex pipiens	SPECIES	36.76900	-76.57100	3030154529
362	Diptera	Culicidae	Culex	Culex pipiens	SPECIES	36.77600	-76.60600	3030367826
363	Diptera	Culicidae	Culex	Culex pipiens	SPECIES	36.77900	-76.52300	3030425165
364	Diptera	Culicidae	Culex	Culex pipiens	SUBSPECIES	36.78089	126.14492	3444340051
365	Diptera	Culicidae	Culex	Culex pipiens	SUBSPECIES	36.78739	126.45103	3444340068
366	Diptera	Culicidae	Culex	Culex pipiens	SUBSPECIES	36.79373	127.09622	4028809236
367	Diptera	Culicidae	Culex	Culex pipiens	SPECIES	36.79500	-76.49000	3030199801
368	Diptera	Culicidae	Culex	Culex pipiens	SPECIES	36.79500	-76.62200	3030388237
369	Diptera	Culicidae	Culex	Culex pipiens	SPECIES	36.79900	-76.57100	3030357340

№	Order	Family	Genus	Species	Taxon Rank	Latitude	Longitude	GBIF_ID
370	Diptera	Culicidae	Culex	Culex pipiens	SPECIES	36.80700	-76.51900	3030377369
371	Diptera	Culicidae	Culex	Culex pipiens	SPECIES	36.82000	-76.46200	3030316933
372	Diptera	Culicidae	Culex	Culex pipiens	SPECIES	36.82400	-76.47500	3030729405
373	Diptera	Culicidae	Culex	Culex pipiens	SPECIES	36.82500	-76.50900	3030357430
374	Diptera	Culicidae	Culex	Culex pipiens	SPECIES	36.82500	-76.64200	3030425362
375	Diptera	Culicidae	Culex	Culex pipiens	SPECIES	36.83000	-76.53900	3030443299
376	Diptera	Culicidae	Culex	Culex pipiens	SPECIES	36.83800	-76.52300	3030461000
377	Diptera	Culicidae	Culex	Culex pipiens	SPECIES	36.84000	54.44000	3351181060
378	Diptera	Culicidae	Culex	Culex pipiens	SPECIES	36.84500	-76.58600	3030199538
379	Diptera	Culicidae	Culex	Culex pipiens	SPECIES	36.84500	-76.49500	3030304610
380	Diptera	Culicidae	Culex	Culex pipiens	SPECIES	36.84700	-76.47500	3030425116
381	Diptera	Culicidae	Culex	Culex pipiens	SPECIES	36.85200	-76.58400	3030424413
382	Diptera	Culicidae	Culex	Culex pipiens	SPECIES	36.85300	-76.44500	3030958319
383	Diptera	Culicidae	Culex	Culex pipiens	SPECIES	36.86100	-76.43400	3030357685
384	Diptera	Culicidae	Culex	Culex pipiens	SPECIES	36.86100	-76.45400	3030415764
385	Diptera	Culicidae	Culex	Culex pipiens	SPECIES	36.86200	-76.50200	3030954174
386	Diptera	Culicidae	Culex	Culex pipiens	SPECIES	36.86500	-76.47700	3030425013
387	Diptera	Culicidae	Culex	Culex pipiens	SPECIES	36.86889	-94.36778	1141858621
388	Diptera	Culicidae	Culex	Culex pipiens	SPECIES	36.87200	-76.55000	3030425215
389	Diptera	Culicidae	Culex	Culex pipiens	SPECIES	36.87300	-76.43300	3030424944
390	Diptera	Culicidae	Culex	Culex pipiens	SPECIES	36.87444	-94.87722	1141858945
391	Diptera	Culicidae	Culex	Culex pipiens	SPECIES	36.87500	-76.45900	3030221856
392	Diptera	Culicidae	Culex	Culex pipiens	SPECIES	36.87667	-89.58778	1141858413
393	Diptera	Culicidae	Culex	Culex pipiens	SPECIES	36.88000	35.86000	3350403752
394	Diptera	Culicidae	Culex	Culex pipiens	SPECIES	36.88300	-76.41400	3030405781
395	Diptera	Culicidae	Culex	Culex pipiens	SPECIES	36.88500	-76.41900	3030399885
396	Diptera	Culicidae	Culex	Culex pipiens	SPECIES	36.88800	-76.44500	3030435009
397	Diptera	Culicidae	Culex	Culex pipiens	SPECIES	36.89900	-76.51000	3030495047
398	Diptera	Culicidae	Culex	Culex pipiens	SPECIES	36.90100	-76.53600	3030331415
399	Diptera	Culicidae	Culex	Culex pipiens	SPECIES	36.91000	-76.48800	3030399890
400	Diptera	Culicidae	Culex	Culex pipiens	SPECIES	36.91500	-76.48300	3030415330
401	Diptera	Culicidae	Culex	Culex pipiens	SPECIES	37.01046	-96.10444	872534679
402	Diptera	Culicidae	Culex	Culex pipiens	SPECIES	37.02361	-94.73528	1141856693
403	Diptera	Culicidae	Culex	Culex pipiens	SUBSPECIES	37.02986	127.85557	3001404717
404	Diptera	Culicidae	Culex	Culex pipiens	SUBSPECIES	37.03677	127.94156	3001931759
405	Diptera	Culicidae	Culex	Culex pipiens	SUBSPECIES	37.03867	127.80280	3001887558
406	Diptera	Culicidae	Culex	Culex pipiens	SUBSPECIES	37.04110	127.91310	3001415698
407	Diptera	Culicidae	Culex	Culex pipiens	SPECIES	37.08417	-94.50167	1141858688
408	Diptera	Culicidae	Culex	Culex pipiens	SPECIES	37.08679	-119.73253	3059749637
409	Diptera	Culicidae	Culex	Culex pipiens	SPECIES	37.08996	-119.72358	2441810829
410	Diptera	Culicidae	Culex	Culex pipiens	SPECIES	37.10901	-119.74951	3059749351

№	Order	Family	Genus	Species	Taxon Rank	Latitude	Longitude	GBIF_ID
411	Diptera	Culicidae	Culex	Culex pipiens	SPECIES	37.11400	-89.19800	3802138171
412	Diptera	Culicidae	Culex	Culex pipiens	SPECIES	37.16598	-94.08426	1141853703
413	Diptera	Culicidae	Culex	Culex pipiens	SUBSPECIES	37.22794	127.78540	3001413601
414	Diptera	Culicidae	Culex	Culex pipiens	SPECIES	37.41528	-88.89778	3048748861
415	Diptera	Culicidae	Culex	Culex pipiens	SPECIES	37.45424	-94.69802	1141856531
416	Diptera	Culicidae	Culex	Culex pipiens	SPECIES	37.48522	-122.23636	3443934310
417	Diptera	Culicidae	Culex	Culex pipiens	SPECIES	37.50716	-122.26052	3443920310
418	Diptera	Culicidae	Culex	Culex pipiens	SPECIES	37.56000	126.98000	3328469068
419	Diptera	Culicidae	Culex	Culex pipiens	SPECIES	37.56076	-95.74214	872534626
420	Diptera	Culicidae	Culex	Culex pipiens	SPECIES	37.56299	-122.32553	3443923308
421	Diptera	Culicidae	Culex	Culex pipiens	SPECIES	37.57757	-122.34857	3443922309
422	Diptera	Culicidae	Culex	Culex pipiens	SPECIES	37.60905	-122.40305	3112401705
423	Diptera	Culicidae	Culex	Culex pipiens	SPECIES	37.62127	-122.49211	899956413
424	Diptera	Culicidae	Culex	Culex pipiens	SUBSPECIES	37.68856	126.79011	3444015153
425	Diptera	Culicidae	Culex	Culex pipiens	SUBSPECIES	37.70119	127.06206	3444333148
426	Diptera	Culicidae	Culex	Culex pipiens	SPECIES	37.72100	-89.08000	3802061245
427	Diptera	Culicidae	Culex	Culex pipiens	SPECIES	37.73000	-122.45000	3347752571
428	Diptera	Culicidae	Culex	Culex pipiens	SPECIES	37.73071	-25.68644	1917547780
429	Diptera	Culicidae	Culex	Culex pipiens	SPECIES	37.73490	-25.65800	1917548801
430	Diptera	Culicidae	Culex	Culex pipiens	SPECIES	37.73497	-25.66367	1917548235
431	Diptera	Culicidae	Culex	Culex pipiens	SPECIES	37.73503	-25.66934	1917548635
432	Diptera	Culicidae	Culex	Culex pipiens	SPECIES	37.73509	-25.67501	1917547647
433	Diptera	Culicidae	Culex	Culex pipiens	SPECIES	37.73516	-25.68069	1917547703
434	Diptera	Culicidae	Culex	Culex pipiens	SPECIES	37.73522	-25.68636	1917555122
435	Diptera	Culicidae	Culex	Culex pipiens	SPECIES	37.73528	-25.69203	1917555124
436	Diptera	Culicidae	Culex	Culex pipiens	SPECIES	37.73934	-25.65224	1917547169
437	Diptera	Culicidae	Culex	Culex pipiens	SPECIES	37.73941	-25.65791	1917547759
438	Diptera	Culicidae	Culex	Culex pipiens	SPECIES	37.73947	-25.66359	1917548256
439	Diptera	Culicidae	Culex	Culex pipiens	SPECIES	37.73953	-25.66926	1917554241
440	Diptera	Culicidae	Culex	Culex pipiens	SPECIES	37.73960	-25.67493	1917554187
441	Diptera	Culicidae	Culex	Culex pipiens	SPECIES	37.73966	-25.68061	1917547746
442	Diptera	Culicidae	Culex	Culex pipiens	SPECIES	37.73973	-25.68628	1917548167
443	Diptera	Culicidae	Culex	Culex pipiens	SPECIES	37.73979	-25.69195	1917554552
444	Diptera	Culicidae	Culex	Culex pipiens	SPECIES	37.74365	-25.63514	1917548316
445	Diptera	Culicidae	Culex	Culex pipiens	SPECIES	37.74372	-25.64081	1917548861
446	Diptera	Culicidae	Culex	Culex pipiens	SPECIES	37.74378	-25.64649	1917547786
447	Diptera	Culicidae	Culex	Culex pipiens	SPECIES	37.74385	-25.65216	1917548675
448	Diptera	Culicidae	Culex	Culex pipiens	SPECIES	37.74391	-25.65783	1917547584
449	Diptera	Culicidae	Culex	Culex pipiens	SPECIES	37.74398	-25.66351	1917554125
450	Diptera	Culicidae	Culex	Culex pipiens	SPECIES	37.74404	-25.66918	1917554729
451	Diptera	Culicidae	Culex	Culex pipiens	SPECIES	37.74410	-25.67485	1917547676

№	Order	Family	Genus	Species	Taxon Rank	Latitude	Longitude	GBIF_ID
452	Diptera	Culicidae	Culex	Culex pipiens	SPECIES	37.74417	-25.68053	1917548652
453	Diptera	Culicidae	Culex	Culex pipiens	SPECIES	37.74423	-25.68620	1917547603
454	Diptera	Culicidae	Culex	Culex pipiens	SPECIES	37.74429	-25.69188	1917547543
455	Diptera	Culicidae	Culex	Culex pipiens	SPECIES	37.74436	-25.69755	1917548641
456	Diptera	Culicidae	Culex	Culex pipiens	SUBSPECIES	37.74475	126.44569	3444340206
457	Diptera	Culicidae	Culex	Culex pipiens	SPECIES	37.74816	-25.63506	1917548648
458	Diptera	Culicidae	Culex	Culex pipiens	SPECIES	37.74822	-25.64073	1917548697
459	Diptera	Culicidae	Culex	Culex pipiens	SPECIES	37.74829	-25.64640	1917547670
460	Diptera	Culicidae	Culex	Culex pipiens	SPECIES	37.74835	-25.65208	1917548180
461	Diptera	Culicidae	Culex	Culex pipiens	SPECIES	37.74842	-25.65775	1917548729
462	Diptera	Culicidae	Culex	Culex pipiens	SPECIES	37.74848	-25.66343	1917548205
463	Diptera	Culicidae	Culex	Culex pipiens	SPECIES	37.74855	-25.66910	1917554184
464	Diptera	Culicidae	Culex	Culex pipiens	SPECIES	37.74861	-25.67477	1917555118
465	Diptera	Culicidae	Culex	Culex pipiens	SPECIES	37.74867	-25.68045	1917555053
466	Diptera	Culicidae	Culex	Culex pipiens	SPECIES	37.74874	-25.68612	1917554607
467	Diptera	Culicidae	Culex	Culex pipiens	SPECIES	37.74880	-25.69180	1917554174
468	Diptera	Culicidae	Culex	Culex pipiens	SPECIES	37.74886	-25.69747	1917554674
469	Diptera	Culicidae	Culex	Culex pipiens	SPECIES	37.74893	-25.70314	1917548591
470	Diptera	Culicidae	Culex	Culex pipiens	SPECIES	37.75260	-25.62930	1917547646
471	Diptera	Culicidae	Culex	Culex pipiens	SPECIES	37.75266	-25.63497	1917548647
472	Diptera	Culicidae	Culex	Culex pipiens	SPECIES	37.75273	-25.64065	1917548739
473	Diptera	Culicidae	Culex	Culex pipiens	SPECIES	37.75279	-25.64632	1917547552
474	Diptera	Culicidae	Culex	Culex pipiens	SPECIES	37.75286	-25.65200	1917547743
475	Diptera	Culicidae	Culex	Culex pipiens	SPECIES	37.75292	-25.65767	1917547053
476	Diptera	Culicidae	Culex	Culex pipiens	SPECIES	37.75299	-25.66334	1917554177
477	Diptera	Culicidae	Culex	Culex pipiens	SPECIES	37.75305	-25.66902	1917650878
478	Diptera	Culicidae	Culex	Culex pipiens	SPECIES	37.75312	-25.67469	1917554155
479	Diptera	Culicidae	Culex	Culex pipiens	SPECIES	37.75318	-25.68037	1917554268
480	Diptera	Culicidae	Culex	Culex pipiens	SPECIES	37.75324	-25.68604	1917554750
481	Diptera	Culicidae	Culex	Culex pipiens	SPECIES	37.75331	-25.69172	1917554994
482	Diptera	Culicidae	Culex	Culex pipiens	SPECIES	37.75710	-25.62922	1917548772
483	Diptera	Culicidae	Culex	Culex pipiens	SPECIES	37.75717	-25.63489	1917547710
484	Diptera	Culicidae	Culex	Culex pipiens	SPECIES	37.75723	-25.64056	1917548662
485	Diptera	Culicidae	Culex	Culex pipiens	SPECIES	37.75730	-25.64624	1917547671
486	Diptera	Culicidae	Culex	Culex pipiens	SPECIES	37.75736	-25.65191	1917547684
487	Diptera	Culicidae	Culex	Culex pipiens	SPECIES	37.75743	-25.65759	1917548758
488	Diptera	Culicidae	Culex	Culex pipiens	SPECIES	37.75749	-25.66326	1917547687
489	Diptera	Culicidae	Culex	Culex pipiens	SPECIES	37.75756	-25.66894	1917547688
490	Diptera	Culicidae	Culex	Culex pipiens	SPECIES	37.75762	-25.67461	1917554708
491	Diptera	Culicidae	Culex	Culex pipiens	SPECIES	37.75768	-25.68029	1917548146
492	Diptera	Culicidae	Culex	Culex pipiens	SPECIES	37.75775	-25.68596	1917554587

№	Order	Family	Genus	Species	Taxon Rank	Latitude	Longitude	GBIF_ID
493	Diptera	Culicidae	Culex	Culex pipiens	SPECIES	37.75781	-25.69164	1917548147
494	Diptera	Culicidae	Culex	Culex pipiens	SPECIES	37.76161	-25.62913	1917547056
495	Diptera	Culicidae	Culex	Culex pipiens	SPECIES	37.76167	-25.63481	1917547580
496	Diptera	Culicidae	Culex	Culex pipiens	SPECIES	37.76174	-25.64048	1917546991
497	Diptera	Culicidae	Culex	Culex pipiens	SPECIES	37.76180	-25.64616	1917547702
498	Diptera	Culicidae	Culex	Culex pipiens	SPECIES	37.76187	-25.65183	1917548643
499	Diptera	Culicidae	Culex	Culex pipiens	SPECIES	37.76193	-25.65751	1917548201
500	Diptera	Culicidae	Culex	Culex pipiens	SPECIES	37.76200	-25.66318	1917554682
501	Diptera	Culicidae	Culex	Culex pipiens	SPECIES	37.76206	-25.66886	1917548776
502	Diptera	Culicidae	Culex	Culex pipiens	SPECIES	37.76213	-25.67453	1917548685
503	Diptera	Culicidae	Culex	Culex pipiens	SPECIES	37.76219	-25.68021	1917547014
504	Diptera	Culicidae	Culex	Culex pipiens	SPECIES	37.76225	-25.68588	1917554691
505	Diptera	Culicidae	Culex	Culex pipiens	SPECIES	37.76641	-25.30549	1917547730
506	Diptera	Culicidae	Culex	Culex pipiens	SPECIES	37.76649	-25.31116	1917547598
507	Diptera	Culicidae	Culex	Culex pipiens	SPECIES	37.76650	-25.66310	1917554678
508	Diptera	Culicidae	Culex	Culex pipiens	SPECIES	37.76657	-25.31683	1917547641
509	Diptera	Culicidae	Culex	Culex pipiens	SPECIES	37.76657	-25.66878	1917555113
510	Diptera	Culicidae	Culex	Culex pipiens	SPECIES	37.76663	-25.67445	1917554770
511	Diptera	Culicidae	Culex	Culex pipiens	SPECIES	37.76665	-25.32251	1917554233
512	Diptera	Culicidae	Culex	Culex pipiens	SPECIES	37.76670	-25.68013	1917555069
513	Diptera	Culicidae	Culex	Culex pipiens	SPECIES	37.77091	-25.30538	1917548171
514	Diptera	Culicidae	Culex	Culex pipiens	SPECIES	37.77099	-25.31106	1917546967
515	Diptera	Culicidae	Culex	Culex pipiens	SPECIES	37.77108	-25.31673	1917554573
516	Diptera	Culicidae	Culex	Culex pipiens	SPECIES	37.77116	-25.32241	1917554179
517	Diptera	Culicidae	Culex	Culex pipiens	SUBSPECIES	37.77188	124.76219	3444593360
518	Diptera	Culicidae	Culex	Culex pipiens	SPECIES	37.77542	-25.30528	1917554747
519	Diptera	Culicidae	Culex	Culex pipiens	SPECIES	37.77550	-25.31096	1917553739
520	Diptera	Culicidae	Culex	Culex pipiens	SPECIES	37.77552	-122.27597	3443937308
521	Diptera	Culicidae	Culex	Culex pipiens	SPECIES	37.77558	-25.31663	1917548152
522	Diptera	Culicidae	Culex	Culex pipiens	SPECIES	37.77566	-25.32231	1917548683
523	Diptera	Culicidae	Culex	Culex pipiens	SPECIES	37.77992	-25.30518	1917554662
524	Diptera	Culicidae	Culex	Culex pipiens	SPECIES	37.78000	-25.31085	1917548191
525	Diptera	Culicidae	Culex	Culex pipiens	SPECIES	37.78009	-25.31653	1917554218
526	Diptera	Culicidae	Culex	Culex pipiens	SPECIES	37.78017	-25.32220	1917554209
527	Diptera	Culicidae	Culex	Culex pipiens	SPECIES	37.80383	-122.26524	1453064406
528	Diptera	Culicidae	Culex	Culex pipiens	SUBSPECIES	37.82374	124.71524	3444214176
529	Diptera	Culicidae	Culex	Culex pipiens	SPECIES	37.83970	-94.70800	1141856573
530	Diptera	Culicidae	Culex	Culex pipiens	SPECIES	37.85952	-4.77440	3456874773
531	Diptera	Culicidae	Culex	Culex pipiens	SPECIES	37.87159	-122.27275	3113791302
532	Diptera	Culicidae	Culex	Culex pipiens	SPECIES	37.90000	-122.65000	3347608452
533	Diptera	Culicidae	Culex	Culex pipiens	SPECIES	37.90000	-122.70000	3350510674

№	Order	Family	Genus	Species	Taxon Rank	Latitude	Longitude	GBIF_ID
534	Diptera	Culicidae	Culex	Culex pipiens	SPECIES	37.91613	-95.17025	1141856311
535	Diptera	Culicidae	Culex	Culex pipiens	SPECIES	37.93576	-122.34775	3113793302
536	Diptera	Culicidae	Culex	Culex pipiens	SUBSPECIES	37.96618	124.70130	3444577092
537	Diptera	Culicidae	Culex	Culex pipiens	SPECIES	37.97000	-122.50000	3347668407
538	Diptera	Culicidae	Culex	Culex pipiens	SPECIES	37.98542	23.70807	2580213194
539	Diptera	Culicidae	Culex	Culex pipiens	SPECIES	38.00000	128.00000	3328469069
540	Diptera	Culicidae	Culex	Culex pipiens	SPECIES	38.02540	-76.95370	3030912545
541	Diptera	Culicidae	Culex	Culex pipiens	SUBSPECIES	38.13825	127.36046	3865961522
542	Diptera	Culicidae	Culex	Culex pipiens	SPECIES	38.29000	-8.76000	3987263034
543	Diptera	Culicidae	Culex	Culex pipiens	SPECIES	38.30158	-95.24338	872534640
544	Diptera	Culicidae	Culex	Culex pipiens	SPECIES	38.31889	-76.40528	3059289352
545	Diptera	Culicidae	Culex	Culex pipiens	SPECIES	38.33300	-123.05000	43173547
546	Diptera	Culicidae	Culex	Culex pipiens	SPECIES	38.36800	-88.06180	3030290282
547	Diptera	Culicidae	Culex	Culex pipiens	SPECIES	38.36800	-88.06250	3030293202
548	Diptera	Culicidae	Culex	Culex pipiens	SPECIES	38.36860	-88.06300	3030294271
549	Diptera	Culicidae	Culex	Culex pipiens	SPECIES	38.36900	-88.33830	3030275130
550	Diptera	Culicidae	Culex	Culex pipiens	SPECIES	38.37000	-88.34030	3030275243
551	Diptera	Culicidae	Culex	Culex pipiens	SPECIES	38.37010	-88.34060	3030675826
552	Diptera	Culicidae	Culex	Culex pipiens	SPECIES	38.39960	-87.77190	3030677260
553	Diptera	Culicidae	Culex	Culex pipiens	SPECIES	38.40070	-87.77180	3030292295
554	Diptera	Culicidae	Culex	Culex pipiens	SPECIES	38.40400	-87.75160	3801579440
555	Diptera	Culicidae	Culex	Culex pipiens	SPECIES	38.40960	-87.74950	3030274956
556	Diptera	Culicidae	Culex	Culex pipiens	SPECIES	38.41428	27.15194	3048723884
557	Diptera	Culicidae	Culex	Culex pipiens	SPECIES	38.51528	-28.63164	1917555022
558	Diptera	Culicidae	Culex	Culex pipiens	SPECIES	38.51536	-28.62591	1917554850
559	Diptera	Culicidae	Culex	Culex pipiens	SPECIES	38.51544	-28.62018	1917555038
560	Diptera	Culicidae	Culex	Culex pipiens	SPECIES	38.51978	-28.63174	1917553862
561	Diptera	Culicidae	Culex	Culex pipiens	SPECIES	38.51986	-28.62601	1917553772
562	Diptera	Culicidae	Culex	Culex pipiens	SPECIES	38.51994	-28.62028	1917554141
563	Diptera	Culicidae	Culex	Culex pipiens	SPECIES	38.52396	-28.65478	1917553802
564	Diptera	Culicidae	Culex	Culex pipiens	SPECIES	38.52404	-28.64905	1917553618
565	Diptera	Culicidae	Culex	Culex pipiens	SPECIES	38.52412	-28.64331	1917554681
566	Diptera	Culicidae	Culex	Culex pipiens	SPECIES	38.52420	-28.63758	1917554431
567	Diptera	Culicidae	Culex	Culex pipiens	SPECIES	38.52428	-28.63185	1917553876
568	Diptera	Culicidae	Culex	Culex pipiens	SPECIES	38.52436	-28.62611	1917553933
569	Diptera	Culicidae	Culex	Culex pipiens	SPECIES	38.52444	-28.62038	1917554409
570	Diptera	Culicidae	Culex	Culex pipiens	SPECIES	38.52847	-28.65488	1917553719
571	Diptera	Culicidae	Culex	Culex pipiens	SPECIES	38.52855	-28.64915	1917554162
572	Diptera	Culicidae	Culex	Culex pipiens	SPECIES	38.52863	-28.64341	1917554641
573	Diptera	Culicidae	Culex	Culex pipiens	SPECIES	38.52871	-28.63768	1917554167
574	Diptera	Culicidae	Culex	Culex pipiens	SPECIES	38.52879	-28.63195	1917554539

№	Order	Family	Genus	Species	Taxon Rank	Latitude	Longitude	GBIF_ID
575	Diptera	Culicidae	Culex	Culex pipiens	SPECIES	38.52887	-28.62621	1917553835
576	Diptera	Culicidae	Culex	Culex pipiens	SPECIES	38.52895	-28.62048	1917554010
577	Diptera	Culicidae	Culex	Culex pipiens	SPECIES	38.53297	-28.65499	1917554199
578	Diptera	Culicidae	Culex	Culex pipiens	SPECIES	38.53305	-28.64925	1917554186
579	Diptera	Culicidae	Culex	Culex pipiens	SPECIES	38.53313	-28.64352	1917554706
580	Diptera	Culicidae	Culex	Culex pipiens	SPECIES	38.53321	-28.63778	1917554644
581	Diptera	Culicidae	Culex	Culex pipiens	SPECIES	38.53329	-28.63205	1917554713
582	Diptera	Culicidae	Culex	Culex pipiens	SPECIES	38.53337	-28.62631	1917554737
583	Diptera	Culicidae	Culex	Culex pipiens	SPECIES	38.53345	-28.62058	1917554439
584	Diptera	Culicidae	Culex	Culex pipiens	SPECIES	38.53748	-28.65509	1917553660
585	Diptera	Culicidae	Culex	Culex pipiens	SPECIES	38.53756	-28.64935	1917554129
586	Diptera	Culicidae	Culex	Culex pipiens	SPECIES	38.53764	-28.64362	1917554666
587	Diptera	Culicidae	Culex	Culex pipiens	SPECIES	38.53772	-28.63789	1917554563
588	Diptera	Culicidae	Culex	Culex pipiens	SPECIES	38.53780	-28.63215	1917554107
589	Diptera	Culicidae	Culex	Culex pipiens	SPECIES	38.53788	-28.62642	1917554206
590	Diptera	Culicidae	Culex	Culex pipiens	SPECIES	38.53944	-89.86278	3801593239
591	Diptera	Culicidae	Culex	Culex pipiens	SPECIES	38.54206	-28.64946	1917553832
592	Diptera	Culicidae	Culex	Culex pipiens	SPECIES	38.54214	-28.64372	1917553830
593	Diptera	Culicidae	Culex	Culex pipiens	SPECIES	38.54222	-28.63799	1917555061
594	Diptera	Culicidae	Culex	Culex pipiens	SPECIES	38.54230	-28.63225	1917553673
595	Diptera	Culicidae	Culex	Culex pipiens	SPECIES	38.54238	-28.62652	1917554658
596	Diptera	Culicidae	Culex	Culex pipiens	SPECIES	38.54246	-28.62078	1917554133
597	Diptera	Culicidae	Culex	Culex pipiens	SPECIES	38.54254	-28.61505	1917554395
598	Diptera	Culicidae	Culex	Culex pipiens	SPECIES	38.54262	-28.60931	1917555007
599	Diptera	Culicidae	Culex	Culex pipiens	SPECIES	38.54270	-28.60358	1917554397
600	Diptera	Culicidae	Culex	Culex pipiens	SPECIES	38.54490	-121.74051	1993809152
601	Diptera	Culicidae	Culex	Culex pipiens	SPECIES	38.54665	-28.64383	1917553708
602	Diptera	Culicidae	Culex	Culex pipiens	SPECIES	38.54673	-28.63809	1917554675
603	Diptera	Culicidae	Culex	Culex pipiens	SPECIES	38.54681	-28.63235	1917554253
604	Diptera	Culicidae	Culex	Culex pipiens	SPECIES	38.54689	-28.62662	1917554280
605	Diptera	Culicidae	Culex	Culex pipiens	SPECIES	38.54697	-28.62088	1917553677
606	Diptera	Culicidae	Culex	Culex pipiens	SPECIES	38.54704	-28.61515	1917554247
607	Diptera	Culicidae	Culex	Culex pipiens	SPECIES	38.54712	-28.60941	1917554204
608	Diptera	Culicidae	Culex	Culex pipiens	SPECIES	38.54720	-28.60368	1917554689
609	Diptera	Culicidae	Culex	Culex pipiens	SPECIES	38.54942	-121.49075	2609067497
610	Diptera	Culicidae	Culex	Culex pipiens	SPECIES	38.55107	-28.64966	1917554267
611	Diptera	Culicidae	Culex	Culex pipiens	SPECIES	38.55115	-28.64393	1917555056
612	Diptera	Culicidae	Culex	Culex pipiens	SPECIES	38.55123	-28.63819	1917553680
613	Diptera	Culicidae	Culex	Culex pipiens	SPECIES	38.55131	-28.63246	1917554193
614	Diptera	Culicidae	Culex	Culex pipiens	SPECIES	38.55139	-28.62672	1917554180
615	Diptera	Culicidae	Culex	Culex pipiens	SPECIES	38.55147	-28.62098	1917553816

№	Order	Family	Genus	Species	Taxon Rank	Latitude	Longitude	GBIF_ID
616	Diptera	Culicidae	Culex	Culex pipiens	SPECIES	38.55557	-28.64977	1917553822
617	Diptera	Culicidae	Culex	Culex pipiens	SPECIES	38.55565	-28.64403	1917554201
618	Diptera	Culicidae	Culex	Culex pipiens	SPECIES	38.55573	-28.63829	1917554759
619	Diptera	Culicidae	Culex	Culex pipiens	SPECIES	38.55581	-28.63256	1917554277
620	Diptera	Culicidae	Culex	Culex pipiens	SPECIES	38.56480	-76.68490	3030963713
621	Diptera	Culicidae	Culex	Culex pipiens	SPECIES	38.57030	-76.68400	3030513595
622	Diptera	Culicidae	Culex	Culex pipiens	SPECIES	38.57030	-76.68380	3030816619
623	Diptera	Culicidae	Culex	Culex pipiens	SPECIES	38.61250	-90.32444	1141858589
624	Diptera	Culicidae	Culex	Culex pipiens	SPECIES	38.61667	-90.13333	3802055256
625	Diptera	Culicidae	Culex	Culex pipiens	SPECIES	38.63906	-27.23268	1917554422
626	Diptera	Culicidae	Culex	Culex pipiens	SPECIES	38.63907	-27.22693	1917554948
627	Diptera	Culicidae	Culex	Culex pipiens	SPECIES	38.63909	-27.22119	1917555449
628	Diptera	Culicidae	Culex	Culex pipiens	SPECIES	38.64357	-27.23269	1917555136
629	Diptera	Culicidae	Culex	Culex pipiens	SPECIES	38.64358	-27.22695	1917554260
630	Diptera	Culicidae	Culex	Culex pipiens	SPECIES	38.64359	-27.22120	1917554687
631	Diptera	Culicidae	Culex	Culex pipiens	SPECIES	38.64360	-27.21546	1917554472
632	Diptera	Culicidae	Culex	Culex pipiens	SPECIES	38.64808	-27.23271	1917555474
633	Diptera	Culicidae	Culex	Culex pipiens	SPECIES	38.64809	-27.22696	1917554630
634	Diptera	Culicidae	Culex	Culex pipiens	SPECIES	38.64810	-27.22122	1917554748
635	Diptera	Culicidae	Culex	Culex pipiens	SPECIES	38.65258	-27.23272	1917555461
636	Diptera	Culicidae	Culex	Culex pipiens	SPECIES	38.65259	-27.22698	1917554614
637	Diptera	Culicidae	Culex	Culex pipiens	SPECIES	38.65260	-27.22123	1917554316
638	Diptera	Culicidae	Culex	Culex pipiens	SPECIES	38.65261	-27.21548	1917555083
639	Diptera	Culicidae	Culex	Culex pipiens	SPECIES	38.65262	-27.20974	1917554451
640	Diptera	Culicidae	Culex	Culex pipiens	SPECIES	38.65706	-27.24423	1917554207
641	Diptera	Culicidae	Culex	Culex pipiens	SPECIES	38.65708	-27.23848	1917553661
642	Diptera	Culicidae	Culex	Culex pipiens	SPECIES	38.65709	-27.23274	1917554777
643	Diptera	Culicidae	Culex	Culex pipiens	SPECIES	38.65710	-27.22699	1917553738
644	Diptera	Culicidae	Culex	Culex pipiens	SPECIES	38.65711	-27.22124	1917554284
645	Diptera	Culicidae	Culex	Culex pipiens	SPECIES	38.65712	-27.21550	1917553703
646	Diptera	Culicidae	Culex	Culex pipiens	SPECIES	38.65713	-27.20975	1917554336
647	Diptera	Culicidae	Culex	Culex pipiens	SPECIES	38.65714	-27.20400	1917553715
648	Diptera	Culicidae	Culex	Culex pipiens	SPECIES	38.66157	-27.24425	1917554208
649	Diptera	Culicidae	Culex	Culex pipiens	SPECIES	38.66158	-27.23850	1917554604
650	Diptera	Culicidae	Culex	Culex pipiens	SPECIES	38.66159	-27.23275	1917554648
651	Diptera	Culicidae	Culex	Culex pipiens	SPECIES	38.66160	-27.22700	1917554744
652	Diptera	Culicidae	Culex	Culex pipiens	SPECIES	38.66162	-27.22126	1917554228
653	Diptera	Culicidae	Culex	Culex pipiens	SPECIES	38.66163	-27.21551	1917555046
654	Diptera	Culicidae	Culex	Culex pipiens	SPECIES	38.66164	-27.20976	1917554628
655	Diptera	Culicidae	Culex	Culex pipiens	SPECIES	38.66165	-27.20402	1917554673
656	Diptera	Culicidae	Culex	Culex pipiens	SPECIES	38.66166	-27.19827	1917555045

№	Order	Family	Genus	Species	Taxon Rank	Latitude	Longitude	GBIF_ID
657	Diptera	Culicidae	Culex	Culex pipiens	SPECIES	38.66609	-27.23851	1917555033
658	Diptera	Culicidae	Culex	Culex pipiens	SPECIES	38.66610	-27.23277	1917554242
659	Diptera	Culicidae	Culex	Culex pipiens	SPECIES	38.66611	-27.22702	1917554191
660	Diptera	Culicidae	Culex	Culex pipiens	SPECIES	38.66612	-27.22127	1917553653
661	Diptera	Culicidae	Culex	Culex pipiens	SPECIES	38.66613	-27.21552	1917553756
662	Diptera	Culicidae	Culex	Culex pipiens	SPECIES	38.66614	-27.20978	1917554214
663	Diptera	Culicidae	Culex	Culex pipiens	SPECIES	38.66615	-27.20403	1917554270
664	Diptera	Culicidae	Culex	Culex pipiens	SPECIES	38.66616	-27.19828	1917553597
665	Diptera	Culicidae	Culex	Culex pipiens	SPECIES	38.67063	-27.22129	1917555065
666	Diptera	Culicidae	Culex	Culex pipiens	SPECIES	38.67064	-27.21554	1917553776
667	Diptera	Culicidae	Culex	Culex pipiens	SPECIES	38.67065	-27.20979	1917554702
668	Diptera	Culicidae	Culex	Culex pipiens	SPECIES	38.67066	-27.20404	1917554258
669	Diptera	Culicidae	Culex	Culex pipiens	SPECIES	38.67514	-27.21555	1917554205
670	Diptera	Culicidae	Culex	Culex pipiens	SPECIES	38.67515	-27.20980	1917554239
671	Diptera	Culicidae	Culex	Culex pipiens	SPECIES	38.67516	-27.20406	1917555063
672	Diptera	Culicidae	Culex	Culex pipiens	SPECIES	38.71137	-27.06038	1917554623
673	Diptera	Culicidae	Culex	Culex pipiens	SPECIES	38.71138	-27.05463	1917554632
674	Diptera	Culicidae	Culex	Culex pipiens	SPECIES	38.71330	-77.01430	3030514162
675	Diptera	Culicidae	Culex	Culex pipiens	SPECIES	38.71330	-77.01450	3030857280
676	Diptera	Culicidae	Culex	Culex pipiens	SPECIES	38.71340	-76.01440	3030797802
677	Diptera	Culicidae	Culex	Culex pipiens	SPECIES	38.71520	-77.01200	3030332284
678	Diptera	Culicidae	Culex	Culex pipiens	SPECIES	38.71540	-77.01220	3030514015
679	Diptera	Culicidae	Culex	Culex pipiens	SPECIES	38.71587	-27.07189	1917554250
680	Diptera	Culicidae	Culex	Culex pipiens	SPECIES	38.71587	-27.08339	1917554786
681	Diptera	Culicidae	Culex	Culex pipiens	SPECIES	38.71587	-27.07764	1917555106
682	Diptera	Culicidae	Culex	Culex pipiens	SPECIES	38.71588	-27.06039	1917554388
683	Diptera	Culicidae	Culex	Culex pipiens	SPECIES	38.71588	-27.06614	1917554780
684	Diptera	Culicidae	Culex	Culex pipiens	SPECIES	38.71840	-77.01900	3030544521
685	Diptera	Culicidae	Culex	Culex pipiens	SPECIES	38.72000	-76.81030	3030413357
686	Diptera	Culicidae	Culex	Culex pipiens	SPECIES	38.72037	-27.08340	1917554796
687	Diptera	Culicidae	Culex	Culex pipiens	SPECIES	38.72037	-27.08915	1919386901
688	Diptera	Culicidae	Culex	Culex pipiens	SPECIES	38.72038	-27.07765	1917554232
689	Diptera	Culicidae	Culex	Culex pipiens	SPECIES	38.72038	-27.06614	1917554651
690	Diptera	Culicidae	Culex	Culex pipiens	SPECIES	38.72038	-27.07190	1917554703
691	Diptera	Culicidae	Culex	Culex pipiens	SPECIES	38.72039	-27.06039	1917555272
692	Diptera	Culicidae	Culex	Culex pipiens	SPECIES	38.72080	-76.99940	3030962632
693	Diptera	Culicidae	Culex	Culex pipiens	SPECIES	38.72260	-77.02270	3030518147
694	Diptera	Culicidae	Culex	Culex pipiens	SPECIES	38.72487	-27.08916	1917554326
695	Diptera	Culicidae	Culex	Culex pipiens	SPECIES	38.72488	-27.07765	1917532510
696	Diptera	Culicidae	Culex	Culex pipiens	SPECIES	38.72488	-27.08340	1917554671
697	Diptera	Culicidae	Culex	Culex pipiens	SPECIES	38.72489	-27.07190	1917554200

№	Order	Family	Genus	Species	Taxon Rank	Latitude	Longitude	GBIF_ID
698	Diptera	Culicidae	Culex	Culex pipiens	SPECIES	38.72489	-27.06615	1917554220
699	Diptera	Culicidae	Culex	Culex pipiens	SPECIES	38.72489	-27.06040	1917554915
700	Diptera	Culicidae	Culex	Culex pipiens	SPECIES	38.72938	-27.08916	1917531407
701	Diptera	Culicidae	Culex	Culex pipiens	SPECIES	38.72938	-27.08341	1917554212
702	Diptera	Culicidae	Culex	Culex pipiens	SPECIES	38.72939	-27.06615	1917554800
703	Diptera	Culicidae	Culex	Culex pipiens	SPECIES	38.72939	-27.07190	1917555075
704	Diptera	Culicidae	Culex	Culex pipiens	SPECIES	38.72939	-27.07766	1917555112
705	Diptera	Culicidae	Culex	Culex pipiens	SPECIES	38.72940	-27.05465	1917533348
706	Diptera	Culicidae	Culex	Culex pipiens	SPECIES	38.72940	-27.06040	1917555088
707	Diptera	Culicidae	Culex	Culex pipiens	SPECIES	38.73210	-87.68910	3030678862
708	Diptera	Culicidae	Culex	Culex pipiens	SPECIES	38.73220	-87.68980	3030275290
709	Diptera	Culicidae	Culex	Culex pipiens	SPECIES	38.73240	-87.69020	3030676079
710	Diptera	Culicidae	Culex	Culex pipiens	SPECIES	38.73389	-27.08917	1917531879
711	Diptera	Culicidae	Culex	Culex pipiens	SPECIES	38.73389	-27.07766	1917554694
712	Diptera	Culicidae	Culex	Culex pipiens	SPECIES	38.73389	-27.08341	1917554757
713	Diptera	Culicidae	Culex	Culex pipiens	SPECIES	38.73390	-27.06616	1917554712
714	Diptera	Culicidae	Culex	Culex pipiens	SPECIES	38.73390	-27.07191	1917555125
715	Diptera	Culicidae	Culex	Culex pipiens	SPECIES	38.73390	-27.06040	1917555133
716	Diptera	Culicidae	Culex	Culex pipiens	SPECIES	38.73391	-27.05465	1917516964
717	Diptera	Culicidae	Culex	Culex pipiens	SPECIES	38.73391	-27.04890	1917531831
718	Diptera	Culicidae	Culex	Culex pipiens	SPECIES	38.73391	-27.04315	1917532309
719	Diptera	Culicidae	Culex	Culex pipiens	SPECIES	38.73839	-27.08917	1917554802
720	Diptera	Culicidae	Culex	Culex pipiens	SPECIES	38.73840	-27.08342	1917554717
721	Diptera	Culicidae	Culex	Culex pipiens	SPECIES	38.73840	-27.07767	1917554733
722	Diptera	Culicidae	Culex	Culex pipiens	SPECIES	38.73840	-27.07191	1917555058
723	Diptera	Culicidae	Culex	Culex pipiens	SPECIES	38.73841	-27.04890	1917531822
724	Diptera	Culicidae	Culex	Culex pipiens	SPECIES	38.73841	-27.06041	1917554178
725	Diptera	Culicidae	Culex	Culex pipiens	SPECIES	38.73841	-27.05465	1917554655
726	Diptera	Culicidae	Culex	Culex pipiens	SPECIES	38.73841	-27.06616	1917554818
727	Diptera	Culicidae	Culex	Culex pipiens	SPECIES	38.74291	-27.07192	1917554211
728	Diptera	Culicidae	Culex	Culex pipiens	SPECIES	38.74291	-27.06616	1917554626
729	Diptera	Culicidae	Culex	Culex pipiens	SPECIES	38.74291	-27.07767	1917554695
730	Diptera	Culicidae	Culex	Culex pipiens	SPECIES	38.74292	-27.05466	1917553888
731	Diptera	Culicidae	Culex	Culex pipiens	SPECIES	38.74292	-27.06041	1917555103
732	Diptera	Culicidae	Culex	Culex pipiens	SPECIES	38.74741	-27.07192	1917555141
733	Diptera	Culicidae	Culex	Culex pipiens	SPECIES	38.74742	-27.06617	1917554266
734	Diptera	Culicidae	Culex	Culex pipiens	SPECIES	38.74742	-27.06042	1917554711
735	Diptera	Culicidae	Culex	Culex pipiens	SPECIES	38.74820	-76.88240	3030379366
736	Diptera	Culicidae	Culex	Culex pipiens	SPECIES	38.74830	-76.82080	3030513022
737	Diptera	Culicidae	Culex	Culex pipiens	SPECIES	38.75010	-76.82290	3030549723
738	Diptera	Culicidae	Culex	Culex pipiens	SPECIES	38.75020	-76.82320	3030371166

№	Order	Family	Genus	Species	Taxon Rank	Latitude	Longitude	GBIF_ID
739	Diptera	Culicidae	Culex	Culex pipiens	SPECIES	38.75570	-76.89010	3030430466
740	Diptera	Culicidae	Culex	Culex pipiens	SPECIES	38.75690	-76.88410	3030351500
741	Diptera	Culicidae	Culex	Culex pipiens	SPECIES	38.75690	-76.88440	3030771561
742	Diptera	Culicidae	Culex	Culex pipiens	SPECIES	38.76070	-76.93390	3030399071
743	Diptera	Culicidae	Culex	Culex pipiens	SPECIES	38.76120	-76.93000	3030851410
744	Diptera	Culicidae	Culex	Culex pipiens	SPECIES	38.76130	-76.92990	3030957129
745	Diptera	Culicidae	Culex	Culex pipiens	SPECIES	38.77000	-95.18000	3328467513
746	Diptera	Culicidae	Culex	Culex pipiens	SPECIES	38.78450	-76.82060	3030840762
747	Diptera	Culicidae	Culex	Culex pipiens	SPECIES	38.78790	-76.83490	3030933216
748	Diptera	Culicidae	Culex	Culex pipiens	SPECIES	38.78810	-76.83490	3030369179
749	Diptera	Culicidae	Culex	Culex pipiens	SPECIES	38.78810	-76.83460	3030915795
750	Diptera	Culicidae	Culex	Culex pipiens	SPECIES	38.79340	-76.94900	3030771014
751	Diptera	Culicidae	Culex	Culex pipiens	SPECIES	38.79650	-76.95460	3893354891
752	Diptera	Culicidae	Culex	Culex pipiens	SPECIES	38.79750	-76.94900	3030774212
753	Diptera	Culicidae	Culex	Culex pipiens	SPECIES	38.80353	-77.55282	4111648301
754	Diptera	Culicidae	Culex	Culex pipiens	SPECIES	38.80389	-89.95833	3801538208
755	Diptera	Culicidae	Culex	Culex pipiens	SPECIES	38.80840	-76.82810	3030519100
756	Diptera	Culicidae	Culex	Culex pipiens	SPECIES	38.80870	-76.82780	3030773259
757	Diptera	Culicidae	Culex	Culex pipiens	SPECIES	38.80960	-76.99320	3030411354
758	Diptera	Culicidae	Culex	Culex pipiens	SPECIES	38.80970	-76.99790	3030406389
759	Diptera	Culicidae	Culex	Culex pipiens	SPECIES	38.80970	-76.99510	3030841105
760	Diptera	Culicidae	Culex	Culex pipiens	SPECIES	38.81010	-76.99540	3030370745
761	Diptera	Culicidae	Culex	Culex pipiens	SPECIES	38.81030	-76.83750	3030372033
762	Diptera	Culicidae	Culex	Culex pipiens	SPECIES	38.81050	-77.00700	3030456105
763	Diptera	Culicidae	Culex	Culex pipiens	SPECIES	38.81050	-77.00720	3030512885
764	Diptera	Culicidae	Culex	Culex pipiens	SPECIES	38.81050	-76.83230	3030781387
765	Diptera	Culicidae	Culex	Culex pipiens	SPECIES	38.81070	-76.83240	3030513454
766	Diptera	Culicidae	Culex	Culex pipiens	SPECIES	38.81090	-76.99590	3030549687
767	Diptera	Culicidae	Culex	Culex pipiens	SPECIES	38.81110	-76.99570	3030406326
768	Diptera	Culicidae	Culex	Culex pipiens	SPECIES	38.81120	-76.99510	3030395844
769	Diptera	Culicidae	Culex	Culex pipiens	SPECIES	38.81220	-76.83820	3030458097
770	Diptera	Culicidae	Culex	Culex pipiens	SPECIES	38.81240	-76.83830	3030549961
771	Diptera	Culicidae	Culex	Culex pipiens	SPECIES	38.81290	-77.00020	3030907064
772	Diptera	Culicidae	Culex	Culex pipiens	SPECIES	38.81370	-76.75440	3030871061
773	Diptera	Culicidae	Culex	Culex pipiens	SPECIES	38.81400	-76.75390	3030411322
774	Diptera	Culicidae	Culex	Culex pipiens	SPECIES	38.81770	-76.76380	3030545507
775	Diptera	Culicidae	Culex	Culex pipiens	SPECIES	38.81980	-76.81470	3030394829
776	Diptera	Culicidae	Culex	Culex pipiens	SPECIES	38.82090	-76.75510	3030807871
777	Diptera	Culicidae	Culex	Culex pipiens	SPECIES	38.82300	-76.89840	3893360876
778	Diptera	Culicidae	Culex	Culex pipiens	SPECIES	38.82330	-76.75240	3030513437
779	Diptera	Culicidae	Culex	Culex pipiens	SPECIES	38.82430	-76.81000	3030396704

№	Order	Family	Genus	Species	Taxon Rank	Latitude	Longitude	GBIF_ID
780	Diptera	Culicidae	Culex	Culex pipiens	SPECIES	38.82880	-76.78600	3030513696
781	Diptera	Culicidae	Culex	Culex pipiens	SPECIES	38.83200	-76.76510	3030964042
782	Diptera	Culicidae	Culex	Culex pipiens	SPECIES	38.83342	-77.51697	4112117301
783	Diptera	Culicidae	Culex	Culex pipiens	SPECIES	38.84720	-76.91120	3893358907
784	Diptera	Culicidae	Culex	Culex pipiens	SPECIES	38.84930	-76.88970	3030772880
785	Diptera	Culicidae	Culex	Culex pipiens	SPECIES	38.85070	-76.90040	3030771079
786	Diptera	Culicidae	Culex	Culex pipiens	SPECIES	38.85080	-76.90040	3030547112
787	Diptera	Culicidae	Culex	Culex pipiens	SPECIES	38.85150	-76.82080	3030964143
788	Diptera	Culicidae	Culex	Culex pipiens	SPECIES	38.85240	-76.86550	3030433697
789	Diptera	Culicidae	Culex	Culex pipiens	SPECIES	38.85330	-76.86370	3030732675
790	Diptera	Culicidae	Culex	Culex pipiens	SPECIES	38.85740	-76.87680	3030513202
791	Diptera	Culicidae	Culex	Culex pipiens	SPECIES	38.85900	-76.78920	3030402826
792	Diptera	Culicidae	Culex	Culex pipiens	SPECIES	38.85930	-76.89300	3030445166
793	Diptera	Culicidae	Culex	Culex pipiens	SPECIES	38.85940	-76.89280	3030411499
794	Diptera	Culicidae	Culex	Culex pipiens	SPECIES	38.85940	-76.89300	3030545171
795	Diptera	Culicidae	Culex	Culex pipiens	SPECIES	38.85990	-76.79030	3030733178
796	Diptera	Culicidae	Culex	Culex pipiens	SPECIES	38.86300	-76.88260	3893361984
797	Diptera	Culicidae	Culex	Culex pipiens	SPECIES	38.86310	-76.88270	3030376364
798	Diptera	Culicidae	Culex	Culex pipiens	SPECIES	38.86470	-76.87820	3030919056
799	Diptera	Culicidae	Culex	Culex pipiens	SPECIES	38.87085	-76.54140	2571297012
800	Diptera	Culicidae	Culex	Culex pipiens	SPECIES	38.87220	-76.90390	3030505186
801	Diptera	Culicidae	Culex	Culex pipiens	SPECIES	38.87310	-76.90520	3030732747
802	Diptera	Culicidae	Culex	Culex pipiens	SPECIES	38.87340	-76.90550	3030370624
803	Diptera	Culicidae	Culex	Culex pipiens	SPECIES	38.87523	-76.54249	2571297063
804	Diptera	Culicidae	Culex	Culex pipiens	SPECIES	38.87690	-76.77440	3030911052
805	Diptera	Culicidae	Culex	Culex pipiens	SPECIES	38.88140	-76.91750	3030741609
806	Diptera	Culicidae	Culex	Culex pipiens	SPECIES	38.88173	-78.13381	2571297059
807	Diptera	Culicidae	Culex	Culex pipiens	SPECIES	38.88430	-76.91740	3030835275
808	Diptera	Culicidae	Culex	Culex pipiens	SPECIES	38.88678	-76.55526	2571295999
809	Diptera	Culicidae	Culex	Culex pipiens	SPECIES	38.88750	-76.86760	3030370646
810	Diptera	Culicidae	Culex	Culex pipiens	SPECIES	38.89098	-78.16604	2571295988
811	Diptera	Culicidae	Culex	Culex pipiens	SPECIES	38.89160	-76.74720	3030372792
812	Diptera	Culicidae	Culex	Culex pipiens	SPECIES	38.89200	-76.90620	3030771271
813	Diptera	Culicidae	Culex	Culex pipiens	SPECIES	38.89220	-76.90620	3030341500
814	Diptera	Culicidae	Culex	Culex pipiens	SPECIES	38.89230	-76.90760	3030770653
815	Diptera	Culicidae	Culex	Culex pipiens	SPECIES	38.89230	-76.90580	3030840903
816	Diptera	Culicidae	Culex	Culex pipiens	SPECIES	38.89240	-76.82840	3030379500
817	Diptera	Culicidae	Culex	Culex pipiens	SPECIES	38.89260	-76.90720	3030780356
818	Diptera	Culicidae	Culex	Culex pipiens	SPECIES	38.89280	-76.83310	3030329731
819	Diptera	Culicidae	Culex	Culex pipiens	SPECIES	38.89280	-76.83330	3030371114
820	Diptera	Culicidae	Culex	Culex pipiens	SPECIES	38.89290	-76.90540	3030771684

№	Order	Family	Genus	Species	Taxon Rank	Latitude	Longitude	GBIF_ID
821	Diptera	Culicidae	Culex	Culex pipiens	SPECIES	38.89300	-76.90460	3030370878
822	Diptera	Culicidae	Culex	Culex pipiens	SPECIES	38.89320	-76.90810	3030771562
823	Diptera	Culicidae	Culex	Culex pipiens	SPECIES	38.89340	-76.73870	3030395794
824	Diptera	Culicidae	Culex	Culex pipiens	SPECIES	38.89380	-76.76120	3030513134
825	Diptera	Culicidae	Culex	Culex pipiens	SPECIES	38.89390	-76.76120	3030493299
826	Diptera	Culicidae	Culex	Culex pipiens	SPECIES	38.89430	-76.85310	3030398799
827	Diptera	Culicidae	Culex	Culex pipiens	SPECIES	38.89530	-76.89920	3030496092
828	Diptera	Culicidae	Culex	Culex pipiens	SPECIES	38.89542	-78.15101	3926392855
829	Diptera	Culicidae	Culex	Culex pipiens	SPECIES	38.89550	-76.89940	3030371649
830	Diptera	Culicidae	Culex	Culex pipiens	SPECIES	38.89606	-78.16585	2571296920
831	Diptera	Culicidae	Culex	Culex pipiens	SPECIES	38.89640	-76.55296	2441813328
832	Diptera	Culicidae	Culex	Culex pipiens	SPECIES	38.89790	-76.88540	3030772714
833	Diptera	Culicidae	Culex	Culex pipiens	SPECIES	38.90092	-78.15529	2571297062
834	Diptera	Culicidae	Culex	Culex pipiens	SPECIES	38.90258	-76.53665	2571297016
835	Diptera	Culicidae	Culex	Culex pipiens	SPECIES	38.90390	-76.92000	3893355976
836	Diptera	Culicidae	Culex	Culex pipiens	SPECIES	38.90400	-76.92000	3030411359
837	Diptera	Culicidae	Culex	Culex pipiens	SPECIES	38.90520	-76.75020	3030396038
838	Diptera	Culicidae	Culex	Culex pipiens	SPECIES	38.90690	-76.72830	3030545043
839	Diptera	Culicidae	Culex	Culex pipiens	SPECIES	38.90740	-76.75050	3030342124
840	Diptera	Culicidae	Culex	Culex pipiens	SPECIES	38.90740	-76.75020	3030493276
841	Diptera	Culicidae	Culex	Culex pipiens	SPECIES	38.90830	-76.73850	3030839289
842	Diptera	Culicidae	Culex	Culex pipiens	SPECIES	38.90850	-76.73800	3030367495
843	Diptera	Culicidae	Culex	Culex pipiens	SPECIES	38.90950	-76.74240	3030365503
844	Diptera	Culicidae	Culex	Culex pipiens	SPECIES	38.90960	-76.80870	3030960707
845	Diptera	Culicidae	Culex	Culex pipiens	SPECIES	38.91250	-76.80600	3030342688
846	Diptera	Culicidae	Culex	Culex pipiens	SPECIES	38.91260	-76.73460	3030806106
847	Diptera	Culicidae	Culex	Culex pipiens	SPECIES	38.91300	-76.91050	3030959529
848	Diptera	Culicidae	Culex	Culex pipiens	SPECIES	38.91350	-76.82980	3030371214
849	Diptera	Culicidae	Culex	Culex pipiens	SPECIES	38.91510	-76.91340	3030369238
850	Diptera	Culicidae	Culex	Culex pipiens	SPECIES	38.91510	-76.73680	3030513114
851	Diptera	Culicidae	Culex	Culex pipiens	SPECIES	38.91540	-76.91240	3030514109
852	Diptera	Culicidae	Culex	Culex pipiens	SPECIES	38.91550	-76.91210	3030396158
853	Diptera	Culicidae	Culex	Culex pipiens	SPECIES	38.91550	-76.91250	3030464304
854	Diptera	Culicidae	Culex	Culex pipiens	SPECIES	38.91770	-76.71980	3030413431
855	Diptera	Culicidae	Culex	Culex pipiens	SPECIES	38.91920	-76.75410	3030374291
856	Diptera	Culicidae	Culex	Culex pipiens	SPECIES	38.91920	-76.73650	3893358985
857	Diptera	Culicidae	Culex	Culex pipiens	SPECIES	38.91980	-76.79360	3030772064
858	Diptera	Culicidae	Culex	Culex pipiens	SPECIES	38.92030	-76.91180	3030770645
859	Diptera	Culicidae	Culex	Culex pipiens	SPECIES	38.92220	-76.72830	3030372839
860	Diptera	Culicidae	Culex	Culex pipiens	SPECIES	38.92220	-76.72820	3030399780
861	Diptera	Culicidae	Culex	Culex pipiens	SPECIES	38.92300	-76.92200	3030411493

№	Order	Family	Genus	Species	Taxon Rank	Latitude	Longitude	GBIF_ID
862	Diptera	Culicidae	Culex	Culex pipiens	SPECIES	38.92300	-76.92190	3030512707
863	Diptera	Culicidae	Culex	Culex pipiens	SPECIES	38.92310	-76.92100	3030491116
864	Diptera	Culicidae	Culex	Culex pipiens	SPECIES	38.92350	-76.92120	3030370642
865	Diptera	Culicidae	Culex	Culex pipiens	SPECIES	38.92360	-76.90470	3030753531
866	Diptera	Culicidae	Culex	Culex pipiens	SPECIES	38.92370	-76.90440	3030399181
867	Diptera	Culicidae	Culex	Culex pipiens	SPECIES	38.92390	-76.74990	3030781366
868	Diptera	Culicidae	Culex	Culex pipiens	SPECIES	38.92420	-76.90840	3030917440
869	Diptera	Culicidae	Culex	Culex pipiens	SPECIES	38.92430	-76.90840	3030769886
870	Diptera	Culicidae	Culex	Culex pipiens	SPECIES	38.92440	-76.73860	3030370744
871	Diptera	Culicidae	Culex	Culex pipiens	SPECIES	38.92450	-76.90840	3030367428
872	Diptera	Culicidae	Culex	Culex pipiens	SPECIES	38.92450	-76.74990	3030964113
873	Diptera	Culicidae	Culex	Culex pipiens	SPECIES	38.92470	-76.73880	3030933157
874	Diptera	Culicidae	Culex	Culex pipiens	SPECIES	38.92690	-76.92040	3030773765
875	Diptera	Culicidae	Culex	Culex pipiens	SPECIES	38.92850	-76.83290	3030769888
876	Diptera	Culicidae	Culex	Culex pipiens	SPECIES	38.92870	-76.92140	3030773718
877	Diptera	Culicidae	Culex	Culex pipiens	SPECIES	38.92940	-76.90650	3030545406
878	Diptera	Culicidae	Culex	Culex pipiens	SPECIES	38.92960	-76.90500	3030374396
879	Diptera	Culicidae	Culex	Culex pipiens	SPECIES	38.93010	-76.74790	3030838640
880	Diptera	Culicidae	Culex	Culex pipiens	SPECIES	38.93100	-76.72700	3030963127
881	Diptera	Culicidae	Culex	Culex pipiens	SPECIES	38.93110	-76.83190	3030544704
882	Diptera	Culicidae	Culex	Culex pipiens	SPECIES	38.93170	-76.91770	3030411433
883	Diptera	Culicidae	Culex	Culex pipiens	SPECIES	38.93200	-76.94540	3030371103
884	Diptera	Culicidae	Culex	Culex pipiens	SPECIES	38.93240	-76.79170	3030734730
885	Diptera	Culicidae	Culex	Culex pipiens	SPECIES	38.93300	-76.94520	3030733129
886	Diptera	Culicidae	Culex	Culex pipiens	SPECIES	38.93300	-76.71920	3030740146
887	Diptera	Culicidae	Culex	Culex pipiens	SPECIES	38.93310	-76.71920	3030379302
888	Diptera	Culicidae	Culex	Culex pipiens	SPECIES	38.93310	-76.93690	3030834546
889	Diptera	Culicidae	Culex	Culex pipiens	SPECIES	38.93320	-76.94510	3030516903
890	Diptera	Culicidae	Culex	Culex pipiens	SPECIES	38.93530	-76.75120	3030741017
891	Diptera	Culicidae	Culex	Culex pipiens	SPECIES	38.93540	-76.75100	3030501320
892	Diptera	Culicidae	Culex	Culex pipiens	SPECIES	38.93560	-76.92020	3030734187
893	Diptera	Culicidae	Culex	Culex pipiens	SPECIES	38.93640	-76.72260	3030366371
894	Diptera	Culicidae	Culex	Culex pipiens	SPECIES	38.93640	-76.47230	3030915871
895	Diptera	Culicidae	Culex	Culex pipiens	SPECIES	38.93670	-76.72270	3030370673
896	Diptera	Culicidae	Culex	Culex pipiens	SPECIES	38.93760	-76.74440	3030407447
897	Diptera	Culicidae	Culex	Culex pipiens	SPECIES	38.93780	-76.74440	3030366739
898	Diptera	Culicidae	Culex	Culex pipiens	SPECIES	38.93850	-76.94540	3893356980
899	Diptera	Culicidae	Culex	Culex pipiens	SPECIES	38.93860	-76.94510	3893359861
900	Diptera	Culicidae	Culex	Culex pipiens	SPECIES	38.93870	-76.94510	3030372061
901	Diptera	Culicidae	Culex	Culex pipiens	SPECIES	38.93870	-76.94520	3030857007
902	Diptera	Culicidae	Culex	Culex pipiens	SPECIES	38.93920	-76.95340	3030517831

№	Order	Family	Genus	Species	Taxon Rank	Latitude	Longitude	GBIF_ID
903	Diptera	Culicidae	Culex	Culex pipiens	SPECIES	38.93930	-76.78630	3030769823
904	Diptera	Culicidae	Culex	Culex pipiens	SPECIES	38.94000	-1.11000	4040515240
905	Diptera	Culicidae	Culex	Culex pipiens	SPECIES	38.94040	-76.89090	3030378374
906	Diptera	Culicidae	Culex	Culex pipiens	SPECIES	38.94170	-76.89480	3030370788
907	Diptera	Culicidae	Culex	Culex pipiens	SPECIES	38.94170	-76.89420	3030512950
908	Diptera	Culicidae	Culex	Culex pipiens	SPECIES	38.94200	-76.94850	3030552422
909	Diptera	Culicidae	Culex	Culex pipiens	SPECIES	38.94210	-76.94850	3030930054
910	Diptera	Culicidae	Culex	Culex pipiens	SPECIES	38.94220	-76.94850	3030398877
911	Diptera	Culicidae	Culex	Culex pipiens	SPECIES	38.94400	-76.92670	3030835213
912	Diptera	Culicidae	Culex	Culex pipiens	SPECIES	38.94420	-76.91730	3030407470
913	Diptera	Culicidae	Culex	Culex pipiens	SPECIES	38.94430	-76.74590	3030355147
914	Diptera	Culicidae	Culex	Culex pipiens	SPECIES	38.94440	-76.87730	3030860296
915	Diptera	Culicidae	Culex	Culex pipiens	SPECIES	38.94450	-76.87690	3030395813
916	Diptera	Culicidae	Culex	Culex pipiens	SPECIES	38.94460	-76.87690	3030544986
917	Diptera	Culicidae	Culex	Culex pipiens	SPECIES	38.94530	-76.72520	3030448147
918	Diptera	Culicidae	Culex	Culex pipiens	SPECIES	38.94530	-76.94870	3030455192
919	Diptera	Culicidae	Culex	Culex pipiens	SPECIES	38.94540	-76.94880	3030370695
920	Diptera	Culicidae	Culex	Culex pipiens	SPECIES	38.94540	-76.72520	3893358018
921	Diptera	Culicidae	Culex	Culex pipiens	SPECIES	38.94550	-76.72520	3030840982
922	Diptera	Culicidae	Culex	Culex pipiens	SPECIES	38.94560	-76.93550	3030400831
923	Diptera	Culicidae	Culex	Culex pipiens	SPECIES	38.94620	-76.91780	3030401047
924	Diptera	Culicidae	Culex	Culex pipiens	SPECIES	38.94630	-76.72440	3030398791
925	Diptera	Culicidae	Culex	Culex pipiens	SPECIES	38.94640	-76.72430	3030513731
926	Diptera	Culicidae	Culex	Culex pipiens	SPECIES	38.94660	-76.74970	3030396927
927	Diptera	Culicidae	Culex	Culex pipiens	SPECIES	38.94960	-76.94890	3030513162
928	Diptera	Culicidae	Culex	Culex pipiens	SPECIES	38.94960	-76.94680	3893360960
929	Diptera	Culicidae	Culex	Culex pipiens	SPECIES	38.94970	-76.94910	3030370950
930	Diptera	Culicidae	Culex	Culex pipiens	SPECIES	38.94990	-76.93000	3030808516
931	Diptera	Culicidae	Culex	Culex pipiens	SPECIES	38.95050	-76.72430	3030381414
932	Diptera	Culicidae	Culex	Culex pipiens	SPECIES	38.95050	-76.97450	3030545801
933	Diptera	Culicidae	Culex	Culex pipiens	SPECIES	38.95070	-76.88080	3030514111
934	Diptera	Culicidae	Culex	Culex pipiens	SPECIES	38.95100	-76.97900	3030917769
935	Diptera	Culicidae	Culex	Culex pipiens	SPECIES	38.95130	-76.95320	3030844134
936	Diptera	Culicidae	Culex	Culex pipiens	SPECIES	38.95210	-76.93250	3030771551
937	Diptera	Culicidae	Culex	Culex pipiens	SPECIES	38.95220	76.97360	3030549927
938	Diptera	Culicidae	Culex	Culex pipiens	SPECIES	38.95220	-76.97360	3893357977
939	Diptera	Culicidae	Culex	Culex pipiens	SPECIES	38.95230	-76.97360	3893355883
940	Diptera	Culicidae	Culex	Culex pipiens	SPECIES	38.95240	-76.70040	3030910458
941	Diptera	Culicidae	Culex	Culex pipiens	SPECIES	38.95250	-76.82510	3030964137
942	Diptera	Culicidae	Culex	Culex pipiens	SPECIES	38.95260	-76.70020	3030932198
943	Diptera	Culicidae	Culex	Culex pipiens	SPECIES	38.95270	-76.70670	3893355012

№	Order	Family	Genus	Species	Taxon Rank	Latitude	Longitude	GBIF_ID
944	Diptera	Culicidae	Culex	Culex pipiens	SPECIES	38.95480	-76.93320	3030483129
945	Diptera	Culicidae	Culex	Culex pipiens	SPECIES	38.95490	-76.93330	3030429979
946	Diptera	Culicidae	Culex	Culex pipiens	SPECIES	38.95500	-76.93370	3030964151
947	Diptera	Culicidae	Culex	Culex pipiens	SPECIES	38.95520	-76.92770	3030848085
948	Diptera	Culicidae	Culex	Culex pipiens	SPECIES	38.95530	-76.92780	3030513184
949	Diptera	Culicidae	Culex	Culex pipiens	SPECIES	38.95620	-76.73770	3030370746
950	Diptera	Culicidae	Culex	Culex pipiens	SPECIES	38.95620	-76.73750	3030964031
951	Diptera	Culicidae	Culex	Culex pipiens	SPECIES	38.95790	-76.75750	3030395181
952	Diptera	Culicidae	Culex	Culex pipiens	SPECIES	38.95810	-76.75140	3030371734
953	Diptera	Culicidae	Culex	Culex pipiens	SPECIES	38.95860	-76.81950	3893358892
954	Diptera	Culicidae	Culex	Culex pipiens	SPECIES	38.95870	-76.82200	3030397000
955	Diptera	Culicidae	Culex	Culex pipiens	SPECIES	38.95950	-76.93180	3030366713
956	Diptera	Culicidae	Culex	Culex pipiens	SPECIES	38.95950	-76.93200	3030369070
957	Diptera	Culicidae	Culex	Culex pipiens	SPECIES	38.95950	-76.93190	3030549542
958	Diptera	Culicidae	Culex	Culex pipiens	SPECIES	38.95960	-76.75060	3030838785
959	Diptera	Culicidae	Culex	Culex pipiens	SPECIES	38.95980	-76.96370	3030357055
960	Diptera	Culicidae	Culex	Culex pipiens	SPECIES	38.95980	-76.75110	3030370759
961	Diptera	Culicidae	Culex	Culex pipiens	SPECIES	38.96020	-76.75130	3030964098
962	Diptera	Culicidae	Culex	Culex pipiens	SPECIES	38.96130	-76.86920	3030781395
963	Diptera	Culicidae	Culex	Culex pipiens	SPECIES	38.96250	-76.74680	3030411367
964	Diptera	Culicidae	Culex	Culex pipiens	SPECIES	38.96280	-76.76010	3030371810
965	Diptera	Culicidae	Culex	Culex pipiens	SPECIES	38.96290	-76.75300	3030771072
966	Diptera	Culicidae	Culex	Culex pipiens	SPECIES	38.96290	-76.75990	3030962133
967	Diptera	Culicidae	Culex	Culex pipiens	SPECIES	38.96350	-76.77410	3893361992
968	Diptera	Culicidae	Culex	Culex pipiens	SPECIES	38.96380	-76.96090	3030517538
969	Diptera	Culicidae	Culex	Culex pipiens	SPECIES	38.96390	-76.82020	3893355898
970	Diptera	Culicidae	Culex	Culex pipiens	SPECIES	38.96420	-76.75540	3030396788
971	Diptera	Culicidae	Culex	Culex pipiens	SPECIES	38.96420	-76.95890	3030917495
972	Diptera	Culicidae	Culex	Culex pipiens	SPECIES	38.96420	-76.95900	3030918034
973	Diptera	Culicidae	Culex	Culex pipiens	SPECIES	38.96580	-76.93270	3030396701
974	Diptera	Culicidae	Culex	Culex pipiens	SPECIES	38.96600	-76.75620	3030381459
975	Diptera	Culicidae	Culex	Culex pipiens	SPECIES	38.96610	-76.77460	3030857209
976	Diptera	Culicidae	Culex	Culex pipiens	SPECIES	38.96620	-76.77420	3030512745
977	Diptera	Culicidae	Culex	Culex pipiens	SPECIES	38.96630	-76.77430	3030805834
978	Diptera	Culicidae	Culex	Culex pipiens	SPECIES	38.96660	-76.81720	3030339963
979	Diptera	Culicidae	Culex	Culex pipiens	SPECIES	38.96660	-76.88400	3030401065
980	Diptera	Culicidae	Culex	Culex pipiens	SPECIES	38.96670	-76.86900	3030379469
981	Diptera	Culicidae	Culex	Culex pipiens	SPECIES	38.96670	-76.81690	3030931106
982	Diptera	Culicidae	Culex	Culex pipiens	SPECIES	38.96680	-76.81690	3030370633
983	Diptera	Culicidae	Culex	Culex pipiens	SPECIES	38.96690	-76.93320	3030379439
984	Diptera	Culicidae	Culex	Culex pipiens	SPECIES	38.96690	-76.74310	3030516400

№	Order	Family	Genus	Species	Taxon Rank	Latitude	Longitude	GBIF_ID
985	Diptera	Culicidae	Culex	Culex pipiens	SPECIES	38.96690	-76.86580	3030545729
986	Diptera	Culicidae	Culex	Culex pipiens	SPECIES	38.96810	-76.89180	3030782391
987	Diptera	Culicidae	Culex	Culex pipiens	SPECIES	38.96850	-76.79110	3030771891
988	Diptera	Culicidae	Culex	Culex pipiens	SPECIES	38.96860	-76.74110	3030513619
989	Diptera	Culicidae	Culex	Culex pipiens	SPECIES	38.96860	-76.79110	3030514123
990	Diptera	Culicidae	Culex	Culex pipiens	SPECIES	38.96870	-76.74120	3030552572
991	Diptera	Culicidae	Culex	Culex pipiens	SPECIES	38.96870	-76.79140	3030859074
992	Diptera	Culicidae	Culex	Culex pipiens	SPECIES	38.96880	-76.77310	3030915792
993	Diptera	Culicidae	Culex	Culex pipiens	SPECIES	38.96920	-76.88110	3030402810
994	Diptera	Culicidae	Culex	Culex pipiens	SPECIES	38.96920	-76.88120	3030773081
995	Diptera	Culicidae	Culex	Culex pipiens	SPECIES	38.96920	-76.88140	3030859197
996	Diptera	Culicidae	Culex	Culex pipiens	SPECIES	38.96930	-76.76380	3030351747
997	Diptera	Culicidae	Culex	Culex pipiens	SPECIES	38.96970	-76.76230	3030841148
998	Diptera	Culicidae	Culex	Culex pipiens	SPECIES	38.97000	-95.24000	3328467511
999	Diptera	Culicidae	Culex	Culex pipiens	SPECIES	38.97050	-76.79590	3030492055
1000	Diptera	Culicidae	Culex	Culex pipiens	SPECIES	38.97100	-76.81450	3030516901
1001	Diptera	Culicidae	Culex	Culex pipiens	SPECIES	38.97170	-95.23500	1141856482
1002	Diptera	Culicidae	Culex	Culex pipiens	SPECIES	38.97180	-76.88750	3030370835
1003	Diptera	Culicidae	Culex	Culex pipiens	SPECIES	38.97180	-77.00900	3030742232
1004	Diptera	Culicidae	Culex	Culex pipiens	SPECIES	38.97420	-76.71730	3030382468
1005	Diptera	Culicidae	Culex	Culex pipiens	SPECIES	38.97440	-76.93180	3030781399
1006	Diptera	Culicidae	Culex	Culex pipiens	SPECIES	38.97600	-76.87330	3030329747
1007	Diptera	Culicidae	Culex	Culex pipiens	SPECIES	38.97650	-76.91700	3030549553
1008	Diptera	Culicidae	Culex	Culex pipiens	SPECIES	38.97650	-76.91690	3030932140
1009	Diptera	Culicidae	Culex	Culex pipiens	SPECIES	38.97660	-76.77550	3030804824
1010	Diptera	Culicidae	Culex	Culex pipiens	SPECIES	38.97670	-76.77510	3030549976
1011	Diptera	Culicidae	Culex	Culex pipiens	SPECIES	38.97700	-76.91730	3030395864
1012	Diptera	Culicidae	Culex	Culex pipiens	SPECIES	38.97720	-76.77440	3030395272
1013	Diptera	Culicidae	Culex	Culex pipiens	SPECIES	38.97730	-76.77420	3030544700
1014	Diptera	Culicidae	Culex	Culex pipiens	SPECIES	38.97767	-77.04159	1453105914
1015	Diptera	Culicidae	Culex	Culex pipiens	SPECIES	38.97850	-76.95860	3030549503
1016	Diptera	Culicidae	Culex	Culex pipiens	SPECIES	38.97860	-76.95860	3030395862
1017	Diptera	Culicidae	Culex	Culex pipiens	SPECIES	38.98000	-76.92850	3030773233
1018	Diptera	Culicidae	Culex	Culex pipiens	SPECIES	38.98090	-77.06970	3030801351
1019	Diptera	Culicidae	Culex	Culex pipiens	SPECIES	38.98170	-76.75610	3893358962
1020	Diptera	Culicidae	Culex	Culex pipiens	SPECIES	38.98300	-76.74830	3030371058
1021	Diptera	Culicidae	Culex	Culex pipiens	SPECIES	38.98320	-76.74370	3030377414
1022	Diptera	Culicidae	Culex	Culex pipiens	SPECIES	38.98320	-76.74380	3030773278
1023	Diptera	Culicidae	Culex	Culex pipiens	SPECIES	38.98350	-77.05760	3030803860
1024	Diptera	Culicidae	Culex	Culex pipiens	SPECIES	38.98380	-76.78850	3030370831
1025	Diptera	Culicidae	Culex	Culex pipiens	SPECIES	38.98430	-76.88290	3893361981

№	Order	Family	Genus	Species	Taxon Rank	Latitude	Longitude	GBIF_ID
1026	Diptera	Culicidae	Culex	Culex pipiens	SPECIES	38.98550	-76.91710	3030395082
1027	Diptera	Culicidae	Culex	Culex pipiens	SPECIES	38.98630	-76.78720	3030771728
1028	Diptera	Culicidae	Culex	Culex pipiens	SPECIES	38.98640	-76.78740	3030512574
1029	Diptera	Culicidae	Culex	Culex pipiens	SPECIES	38.98650	-76.75580	3030552340
1030	Diptera	Culicidae	Culex	Culex pipiens	SPECIES	38.98650	-76.78730	3030855177
1031	Diptera	Culicidae	Culex	Culex pipiens	SPECIES	38.98690	-76.91640	3030870778
1032	Diptera	Culicidae	Culex	Culex pipiens	SPECIES	38.98720	-76.98320	3030357027
1033	Diptera	Culicidae	Culex	Culex pipiens	SPECIES	38.98720	-76.91670	3030513036
1034	Diptera	Culicidae	Culex	Culex pipiens	SPECIES	38.98720	-76.78320	3030917728
1035	Diptera	Culicidae	Culex	Culex pipiens	SPECIES	38.98980	-76.76360	3030371753
1036	Diptera	Culicidae	Culex	Culex pipiens	SPECIES	38.98990	-76.76350	3030860220
1037	Diptera	Culicidae	Culex	Culex pipiens	SPECIES	38.99000	-76.72110	3030398959
1038	Diptera	Culicidae	Culex	Culex pipiens	SPECIES	38.99000	-76.76360	3030553169
1039	Diptera	Culicidae	Culex	Culex pipiens	SPECIES	38.99040	-76.92810	3030512596
1040	Diptera	Culicidae	Culex	Culex pipiens	SPECIES	38.99050	-76.92840	3030370692
1041	Diptera	Culicidae	Culex	Culex pipiens	SPECIES	38.99060	-76.92800	3893361969
1042	Diptera	Culicidae	Culex	Culex pipiens	SPECIES	38.99110	-76.74930	3030844078
1043	Diptera	Culicidae	Culex	Culex pipiens	SPECIES	38.99140	-76.91890	3030754334
1044	Diptera	Culicidae	Culex	Culex pipiens	SPECIES	38.99190	-76.92630	3030374800
1045	Diptera	Culicidae	Culex	Culex pipiens	SPECIES	38.99220	-76.78600	3030378732
1046	Diptera	Culicidae	Culex	Culex pipiens	SPECIES	38.99270	-76.92400	3030807717
1047	Diptera	Culicidae	Culex	Culex pipiens	SPECIES	38.99380	-76.72540	3030401020
1048	Diptera	Culicidae	Culex	Culex pipiens	SPECIES	38.99390	-76.72540	3030754385
1049	Diptera	Culicidae	Culex	Culex pipiens	SPECIES	38.99430	-76.92210	3030372284
1050	Diptera	Culicidae	Culex	Culex pipiens	SPECIES	38.99660	-77.00410	3030402808
1051	Diptera	Culicidae	Culex	Culex pipiens	SPECIES	38.99730	-76.73070	3030401284
1052	Diptera	Culicidae	Culex	Culex pipiens	SPECIES	38.99780	-76.93670	3030963586
1053	Diptera	Culicidae	Culex	Culex pipiens	SPECIES	38.99910	-76.74760	3030770952
1054	Diptera	Culicidae	Culex	Culex pipiens	SPECIES	38.99930	-76.74810	3030934122
1055	Diptera	Culicidae	Culex	Culex pipiens	SPECIES	39.00050	-76.88710	3030398862
1056	Diptera	Culicidae	Culex	Culex pipiens	SPECIES	39.00070	-76.88710	3893359901
1057	Diptera	Culicidae	Culex	Culex pipiens	SPECIES	39.00171	-76.94202	2573803192
1058	Diptera	Culicidae	Culex	Culex pipiens	SPECIES	39.00182	-76.94195	2448000010
1059	Diptera	Culicidae	Culex	Culex pipiens	SPECIES	39.00200	-76.92180	3030513160
1060	Diptera	Culicidae	Culex	Culex pipiens	SPECIES	39.00210	-76.92150	3030771753
1061	Diptera	Culicidae	Culex	Culex pipiens	SPECIES	39.00210	-76.73610	3893359870
1062	Diptera	Culicidae	Culex	Culex pipiens	SPECIES	39.00220	-76.92810	3030513730
1063	Diptera	Culicidae	Culex	Culex pipiens	SPECIES	39.00220	-76.92830	3030772293
1064	Diptera	Culicidae	Culex	Culex pipiens	SPECIES	39.00220	-76.73610	3030841136
1065	Diptera	Culicidae	Culex	Culex pipiens	SPECIES	39.00260	-76.78160	3030373733
1066	Diptera	Culicidae	Culex	Culex pipiens	SPECIES	39.00320	-76.73450	3030771584

№	Order	Family	Genus	Species	Taxon Rank	Latitude	Longitude	GBIF_ID
1067	Diptera	Culicidae	Culex	Culex pipiens	SPECIES	39.00380	-76.87830	3030399163
1068	Diptera	Culicidae	Culex	Culex pipiens	SPECIES	39.00410	-76.74150	3030912675
1069	Diptera	Culicidae	Culex	Culex pipiens	SPECIES	39.00420	-76.74150	3030772756
1070	Diptera	Culicidae	Culex	Culex pipiens	SPECIES	39.00550	-76.91740	3030329883
1071	Diptera	Culicidae	Culex	Culex pipiens	SPECIES	39.00550	-76.77550	3030843024
1072	Diptera	Culicidae	Culex	Culex pipiens	SPECIES	39.00570	-76.94160	3030395784
1073	Diptera	Culicidae	Culex	Culex pipiens	SPECIES	39.00570	-76.91690	3030963095
1074	Diptera	Culicidae	Culex	Culex pipiens	SPECIES	39.00579	-76.94173	2573978227
1075	Diptera	Culicidae	Culex	Culex pipiens	SPECIES	39.00580	-76.86730	3030406374
1076	Diptera	Culicidae	Culex	Culex pipiens	SPECIES	39.00580	-76.94150	3030918753
1077	Diptera	Culicidae	Culex	Culex pipiens	SPECIES	39.00620	-76.94490	3030352600
1078	Diptera	Culicidae	Culex	Culex pipiens	SPECIES	39.00620	-76.92120	3030553128
1079	Diptera	Culicidae	Culex	Culex pipiens	SPECIES	39.00630	-76.94470	3030845137
1080	Diptera	Culicidae	Culex	Culex pipiens	SPECIES	39.00640	-76.94450	3030783359
1081	Diptera	Culicidae	Culex	Culex pipiens	SPECIES	39.00640	-76.86660	3893355913
1082	Diptera	Culicidae	Culex	Culex pipiens	SPECIES	39.00660	-76.95310	3030373288
1083	Diptera	Culicidae	Culex	Culex pipiens	SPECIES	39.00660	-76.95300	3030963789
1084	Diptera	Culicidae	Culex	Culex pipiens	SPECIES	39.00700	-76.73760	3030370826
1085	Diptera	Culicidae	Culex	Culex pipiens	SPECIES	39.00720	-76.91520	3030857210
1086	Diptera	Culicidae	Culex	Culex pipiens	SPECIES	39.00750	-76.74770	3030912958
1087	Diptera	Culicidae	Culex	Culex pipiens	SPECIES	39.00760	-76.74750	3030395847
1088	Diptera	Culicidae	Culex	Culex pipiens	SPECIES	39.00810	-76.91720	3030963835
1089	Diptera	Culicidae	Culex	Culex pipiens	SPECIES	39.00830	-76.74540	3893358910
1090	Diptera	Culicidae	Culex	Culex pipiens	SPECIES	39.00950	-76.77320	3030835015
1091	Diptera	Culicidae	Culex	Culex pipiens	SPECIES	39.00950	-76.77330	3030959972
1092	Diptera	Culicidae	Culex	Culex pipiens	SPECIES	39.01000	-76.87900	3030395298
1093	Diptera	Culicidae	Culex	Culex pipiens	SPECIES	39.01020	-76.88790	3030372621
1094	Diptera	Culicidae	Culex	Culex pipiens	SPECIES	39.01020	-76.91420	3030401149
1095	Diptera	Culicidae	Culex	Culex pipiens	SPECIES	39.01140	-76.91420	3030516379
1096	Diptera	Culicidae	Culex	Culex pipiens	SPECIES	39.01360	-76.92170	3030407309
1097	Diptera	Culicidae	Culex	Culex pipiens	SPECIES	39.01420	-87.72470	3030293074
1098	Diptera	Culicidae	Culex	Culex pipiens	SPECIES	39.01430	-87.72630	3030677178
1099	Diptera	Culicidae	Culex	Culex pipiens	SPECIES	39.01470	-87.72470	3030277290
1100	Diptera	Culicidae	Culex	Culex pipiens	SPECIES	39.01510	-76.93370	3030371167
1101	Diptera	Culicidae	Culex	Culex pipiens	SPECIES	39.01580	-76.93430	3030398772
1102	Diptera	Culicidae	Culex	Culex pipiens	SPECIES	39.01580	-76.93440	3893360919
1103	Diptera	Culicidae	Culex	Culex pipiens	SPECIES	39.01720	-76.92400	3030961078
1104	Diptera	Culicidae	Culex	Culex pipiens	SPECIES	39.02200	-76.96070	3030545173
1105	Diptera	Culicidae	Culex	Culex pipiens	SPECIES	39.02570	-77.07640	3059277343
1106	Diptera	Culicidae	Culex	Culex pipiens	SPECIES	39.03040	-76.98670	3030366968
1107	Diptera	Culicidae	Culex	Culex pipiens	SPECIES	39.03750	-76.77900	3893359984

№	Order	Family	Genus	Species	Taxon Rank	Latitude	Longitude	GBIF_ID
1108	Diptera	Culicidae	Culex	Culex pipiens	SPECIES	39.03911	-95.20670	3059752436
1109	Diptera	Culicidae	Culex	Culex pipiens	SPECIES	39.03940	-76.75590	3030771082
1110	Diptera	Culicidae	Culex	Culex pipiens	SPECIES	39.03940	-76.78400	3893358873
1111	Diptera	Culicidae	Culex	Culex pipiens	SPECIES	39.04010	-76.75800	3030963595
1112	Diptera	Culicidae	Culex	Culex pipiens	SPECIES	39.04190	-76.79100	3030963759
1113	Diptera	Culicidae	Culex	Culex pipiens	SPECIES	39.04220	-76.79090	3030771597
1114	Diptera	Culicidae	Culex	Culex pipiens	SPECIES	39.04220	-76.78350	3030964159
1115	Diptera	Culicidae	Culex	Culex pipiens	SPECIES	39.04230	-76.78360	3030780384
1116	Diptera	Culicidae	Culex	Culex pipiens	SPECIES	39.04260	-76.78680	3030514137
1117	Diptera	Culicidae	Culex	Culex pipiens	SPECIES	39.04320	-76.76150	3030553153
1118	Diptera	Culicidae	Culex	Culex pipiens	SPECIES	39.04406	-95.19887	3404206983
1119	Diptera	Culicidae	Culex	Culex pipiens	SPECIES	39.04450	-76.78950	3030459109
1120	Diptera	Culicidae	Culex	Culex pipiens	SPECIES	39.04460	-76.78940	3030553182
1121	Diptera	Culicidae	Culex	Culex pipiens	SPECIES	39.04480	-76.78900	3030771662
1122	Diptera	Culicidae	Culex	Culex pipiens	SPECIES	39.04770	-76.78350	3893361952
1123	Diptera	Culicidae	Culex	Culex pipiens	SPECIES	39.04840	-76.78700	3030963344
1124	Diptera	Culicidae	Culex	Culex pipiens	SPECIES	39.04850	-76.78680	3030371608
1125	Diptera	Culicidae	Culex	Culex pipiens	SPECIES	39.05600	-76.85310	3030963349
1126	Diptera	Culicidae	Culex	Culex pipiens	SPECIES	39.05920	-76.80540	3893359854
1127	Diptera	Culicidae	Culex	Culex pipiens	SPECIES	39.06070	-76.97240	3030804958
1128	Diptera	Culicidae	Culex	Culex pipiens	SPECIES	39.06100	-76.86390	3893356842
1129	Diptera	Culicidae	Culex	Culex pipiens	SPECIES	39.06610	-76.93620	3893359852
1130	Diptera	Culicidae	Culex	Culex pipiens	SPECIES	39.06980	-76.87800	3030406472
1131	Diptera	Culicidae	Culex	Culex pipiens	SPECIES	39.07000	-0.79000	1500654470
1132	Diptera	Culicidae	Culex	Culex pipiens	SPECIES	39.07020	-76.77260	3030370786
1133	Diptera	Culicidae	Culex	Culex pipiens	SPECIES	39.07020	-76.77270	3030513056
1134	Diptera	Culicidae	Culex	Culex pipiens	SPECIES	39.07080	-76.88340	3030492210
1135	Diptera	Culicidae	Culex	Culex pipiens	SPECIES	39.07400	-76.87880	3030496011
1136	Diptera	Culicidae	Culex	Culex pipiens	SPECIES	39.07410	-76.75510	3030401799
1137	Diptera	Culicidae	Culex	Culex pipiens	SPECIES	39.07410	-76.87920	3030963773
1138	Diptera	Culicidae	Culex	Culex pipiens	SPECIES	39.07580	-76.87840	3030841284
1139	Diptera	Culicidae	Culex	Culex pipiens	SPECIES	39.07600	-76.87820	3030396100
1140	Diptera	Culicidae	Culex	Culex pipiens	SPECIES	39.07730	-76.88240	3030399190
1141	Diptera	Culicidae	Culex	Culex pipiens	SPECIES	39.08220	-76.88010	3030399263
1142	Diptera	Culicidae	Culex	Culex pipiens	SPECIES	39.08230	-76.88020	3030491190
1143	Diptera	Culicidae	Culex	Culex pipiens	SPECIES	39.08230	-76.88010	3030795283
1144	Diptera	Culicidae	Culex	Culex pipiens	SPECIES	39.08350	-76.84290	3030816561
1145	Diptera	Culicidae	Culex	Culex pipiens	SPECIES	39.08789	-77.95700	2571297056
1146	Diptera	Culicidae	Culex	Culex pipiens	SPECIES	39.09070	-76.86970	3030369655
1147	Diptera	Culicidae	Culex	Culex pipiens	SPECIES	39.09190	-76.88170	3030456189
1148	Diptera	Culicidae	Culex	Culex pipiens	SPECIES	39.09190	-76.88160	3030963346

№	Order	Family	Genus	Species	Taxon Rank	Latitude	Longitude	GBIF_ID
1149	Diptera	Culicidae	Culex	Culex pipiens	SPECIES	39.09406	-96.61177	3926386862
1150	Diptera	Culicidae	Culex	Culex pipiens	SPECIES	39.09495	-96.59023	3312316354
1151	Diptera	Culicidae	Culex	Culex pipiens	SPECIES	39.09563	-77.98264	3126776130
1152	Diptera	Culicidae	Culex	Culex pipiens	SPECIES	39.09630	-76.86560	3030503328
1153	Diptera	Culicidae	Culex	Culex pipiens	SPECIES	39.09630	-76.86550	3030916061
1154	Diptera	Culicidae	Culex	Culex pipiens	SPECIES	39.09750	-76.85770	3030366448
1155	Diptera	Culicidae	Culex	Culex pipiens	SPECIES	39.09770	-76.86530	3030499331
1156	Diptera	Culicidae	Culex	Culex pipiens	SPECIES	39.09780	-76.86520	3030514152
1157	Diptera	Culicidae	Culex	Culex pipiens	SPECIES	39.09970	-94.57830	1141858471
1158	Diptera	Culicidae	Culex	Culex pipiens	SPECIES	39.10080	-76.84070	3030456273
1159	Diptera	Culicidae	Culex	Culex pipiens	SPECIES	39.10090	-76.84080	3030398648
1160	Diptera	Culicidae	Culex	Culex pipiens	SPECIES	39.10090	-76.86580	3030545357
1161	Diptera	Culicidae	Culex	Culex pipiens	SPECIES	39.10210	-76.88240	3030512878
1162	Diptera	Culicidae	Culex	Culex pipiens	SPECIES	39.10230	-76.87890	3030960030
1163	Diptera	Culicidae	Culex	Culex pipiens	SPECIES	39.10340	-76.85020	3030365628
1164	Diptera	Culicidae	Culex	Culex pipiens	SPECIES	39.10350	-76.85050	3030771642
1165	Diptera	Culicidae	Culex	Culex pipiens	SPECIES	39.10700	-76.84520	3030398735
1166	Diptera	Culicidae	Culex	Culex pipiens	SPECIES	39.12712	-96.63906	4112273232
1167	Diptera	Culicidae	Culex	Culex pipiens	SPECIES	39.13160	-77.22620	3030343574
1168	Diptera	Culicidae	Culex	Culex pipiens	SPECIES	39.13600	-77.25650	3030368191
1169	Diptera	Culicidae	Culex	Culex pipiens	SPECIES	39.14531	-96.62796	2441831588
1170	Diptera	Culicidae	Culex	Culex pipiens	SPECIES	39.18361	-96.57139	1141856727
1171	Diptera	Culicidae	Culex	Culex pipiens	SPECIES	39.20000	-0.33000	1502254167
1172	Diptera	Culicidae	Culex	Culex pipiens	SPECIES	39.20779	-96.59494	3312316358
1173	Diptera	Culicidae	Culex	Culex pipiens	SPECIES	39.21297	-96.59740	3312316349
1174	Diptera	Culicidae	Culex	Culex pipiens	SPECIES	39.21405	-96.60165	2441828026
1175	Diptera	Culicidae	Culex	Culex pipiens	SPECIES	39.21500	-96.59400	2979449346
1176	Diptera	Culicidae	Culex	Culex pipiens	SPECIES	39.21545	-96.59370	3312316353
1177	Diptera	Culicidae	Culex	Culex pipiens	SPECIES	39.21718	-74.92428	3090996304
1178	Diptera	Culicidae	Culex	Culex pipiens	SPECIES	39.21884	-96.59687	3312316347
1179	Diptera	Culicidae	Culex	Culex pipiens	SPECIES	39.22980	-76.85510	3030342253
1180	Diptera	Culicidae	Culex	Culex pipiens	SPECIES	39.23290	-76.83330	3030797895
1181	Diptera	Culicidae	Culex	Culex pipiens	SPECIES	39.24000	-0.34000	1502254043
1182	Diptera	Culicidae	Culex	Culex pipiens	SPECIES	39.25000	-0.32000	1501648946
1183	Diptera	Culicidae	Culex	Culex pipiens	SPECIES	39.25000	-8.50000	3349254811
1184	Diptera	Culicidae	Culex	Culex pipiens	SPECIES	39.31944	-89.57194	3802154556
1185	Diptera	Culicidae	Culex	Culex pipiens	SPECIES	39.34000	-0.31000	1501664354
1186	Diptera	Culicidae	Culex	Culex pipiens	SPECIES	39.35000	-0.41000	1502254093
1187	Diptera	Culicidae	Culex	Culex pipiens	SPECIES	39.35680	3.00639	921337095
1188	Diptera	Culicidae	Culex	Culex pipiens	SPECIES	39.38000	-0.36000	1502254150
1189	Diptera	Culicidae	Culex	Culex pipiens	SPECIES	39.38510	-87.69490	3030302321

№	Order	Family	Genus	Species	Taxon Rank	Latitude	Longitude	GBIF_ID
1190	Diptera	Culicidae	Culex	Culex pipiens	SPECIES	39.38930	-87.68100	3030277097
1191	Diptera	Culicidae	Culex	Culex pipiens	SPECIES	39.39129	-76.57151	2576390719
1192	Diptera	Culicidae	Culex	Culex pipiens	SPECIES	39.39390	-87.69730	3030677022
1193	Diptera	Culicidae	Culex	Culex pipiens	SPECIES	39.41000	-0.37000	1502254305
1194	Diptera	Culicidae	Culex	Culex pipiens	SPECIES	39.42780	-77.46670	3030399673
1195	Diptera	Culicidae	Culex	Culex pipiens	SPECIES	39.43189	-76.12959	3961409652
1196	Diptera	Culicidae	Culex	Culex pipiens	SPECIES	39.44415	-31.13944	1917554292
1197	Diptera	Culicidae	Culex	Culex pipiens	SPECIES	39.44436	-31.13365	1917535465
1198	Diptera	Culicidae	Culex	Culex pipiens	SPECIES	39.44456	-31.12785	1917555463
1199	Diptera	Culicidae	Culex	Culex pipiens	SPECIES	39.44864	-31.13971	1917554619
1200	Diptera	Culicidae	Culex	Culex pipiens	SPECIES	39.44885	-31.13391	1917554172
1201	Diptera	Culicidae	Culex	Culex pipiens	SPECIES	39.44906	-31.12812	1917555077
1202	Diptera	Culicidae	Culex	Culex pipiens	SPECIES	39.44926	-31.12232	1917554959
1203	Diptera	Culicidae	Culex	Culex pipiens	SPECIES	39.45108	-76.08092	3961298638
1204	Diptera	Culicidae	Culex	Culex pipiens	SPECIES	39.45334	-31.13418	1917554190
1205	Diptera	Culicidae	Culex	Culex pipiens	SPECIES	39.45355	-31.12838	1917554693
1206	Diptera	Culicidae	Culex	Culex pipiens	SPECIES	39.45375	-31.12259	1917554810
1207	Diptera	Culicidae	Culex	Culex pipiens	SPECIES	39.45784	-31.13444	1917555009
1208	Diptera	Culicidae	Culex	Culex pipiens	SPECIES	39.45804	-31.12865	1917555104
1209	Diptera	Culicidae	Culex	Culex pipiens	SPECIES	39.45825	-31.12285	1917554908
1210	Diptera	Culicidae	Culex	Culex pipiens	SPECIES	39.46233	-31.13471	1917554645
1211	Diptera	Culicidae	Culex	Culex pipiens	SPECIES	39.46254	-31.12891	1917555121
1212	Diptera	Culicidae	Culex	Culex pipiens	SPECIES	39.46274	-31.12312	1917555761
1213	Diptera	Culicidae	Culex	Culex pipiens	SPECIES	39.48346	-76.10065	3961278712
1214	Diptera	Culicidae	Culex	Culex pipiens	SPECIES	39.48839	-79.29185	3858492192
1215	Diptera	Culicidae	Culex	Culex pipiens	SPECIES	39.50000	30.98000	3347370666
1216	Diptera	Culicidae	Culex	Culex pipiens	SPECIES	39.50636	-76.09075	3961431675
1217	Diptera	Culicidae	Culex	Culex pipiens	SPECIES	39.53693	-105.00391	3985700738
1218	Diptera	Culicidae	Culex	Culex pipiens	SPECIES	39.55278	-95.52165	1141856523
1219	Diptera	Culicidae	Culex	Culex pipiens	SPECIES	39.60320	-87.69290	3030275165
1220	Diptera	Culicidae	Culex	Culex pipiens	SPECIES	39.61920	-87.66600	3030277238
1221	Diptera	Culicidae	Culex	Culex pipiens	SPECIES	39.62000	-0.26000	1501648932
1222	Diptera	Culicidae	Culex	Culex pipiens	SPECIES	39.63270	-87.67690	3030674893
1223	Diptera	Culicidae	Culex	Culex pipiens	SPECIES	39.63940	-86.19910	3030405504
1224	Diptera	Culicidae	Culex	Culex pipiens	SPECIES	39.66640	-86.10280	3030232986
1225	Diptera	Culicidae	Culex	Culex pipiens	SPECIES	39.67520	-86.11800	3030405449
1226	Diptera	Culicidae	Culex	Culex pipiens	SPECIES	39.67880	-86.12330	3030400225
1227	Diptera	Culicidae	Culex	Culex pipiens	SPECIES	39.68670	-85.95960	3030405687
1228	Diptera	Culicidae	Culex	Culex pipiens	SPECIES	39.69960	-86.14400	3030405391
1229	Diptera	Culicidae	Culex	Culex pipiens	SPECIES	39.73090	-86.19600	3030400188
1230	Diptera	Culicidae	Culex	Culex pipiens	SPECIES	39.73600	-85.97620	3030405322

№	Order	Family	Genus	Species	Taxon Rank	Latitude	Longitude	GBIF_ID
1231	Diptera	Culicidae	Culex	Culex pipiens	SPECIES	39.73900	-86.31720	3030400240
1232	Diptera	Culicidae	Culex	Culex pipiens	SPECIES	39.75520	-86.05610	3030154567
1233	Diptera	Culicidae	Culex	Culex pipiens	SPECIES	39.77470	-86.24820	3030405584
1234	Diptera	Culicidae	Culex	Culex pipiens	SPECIES	39.79730	-86.29090	3030400230
1235	Diptera	Culicidae	Culex	Culex pipiens	SPECIES	39.81000	-86.06970	3030405498
1236	Diptera	Culicidae	Culex	Culex pipiens	SPECIES	39.83417	-96.06389	1141856538
1237	Diptera	Culicidae	Culex	Culex pipiens	SPECIES	39.84110	-85.99290	3030199330
1238	Diptera	Culicidae	Culex	Culex pipiens	SPECIES	39.84210	-82.81440	3030482956
1239	Diptera	Culicidae	Culex	Culex pipiens	SPECIES	39.84280	-86.11380	3030400212
1240	Diptera	Culicidae	Culex	Culex pipiens	SPECIES	39.84530	-83.06060	3030310921
1241	Diptera	Culicidae	Culex	Culex pipiens	SPECIES	39.85030	-86.17970	3030144810
1242	Diptera	Culicidae	Culex	Culex pipiens	SPECIES	39.85210	-82.89230	3030445102
1243	Diptera	Culicidae	Culex	Culex pipiens	SPECIES	39.87167	-96.25861	1141856526
1244	Diptera	Culicidae	Culex	Culex pipiens	SPECIES	39.87271	32.73589	4076064540
1245	Diptera	Culicidae	Culex	Culex pipiens	SPECIES	39.87340	20.00024	3714034582
1246	Diptera	Culicidae	Culex	Culex pipiens	SPECIES	39.87350	-86.32260	3030405414
1247	Diptera	Culicidae	Culex	Culex pipiens	SPECIES	39.88850	-86.05390	3030400284
1248	Diptera	Culicidae	Culex	Culex pipiens	SPECIES	39.89130	-85.98780	3030154571
1249	Diptera	Culicidae	Culex	Culex pipiens	SPECIES	39.89350	-86.14560	3030199916
1250	Diptera	Culicidae	Culex	Culex pipiens	SPECIES	39.89490	-86.09080	3030400211
1251	Diptera	Culicidae	Culex	Culex pipiens	SPECIES	39.89490	-83.05770	3030479830
1252	Diptera	Culicidae	Culex	Culex pipiens	SPECIES	39.96370	-82.77350	3030445862
1253	Diptera	Culicidae	Culex	Culex pipiens	SPECIES	40.00248	-86.15750	2251927975
1254	Diptera	Culicidae	Culex	Culex pipiens	SPECIES	40.02180	-83.23960	3030484045
1255	Diptera	Culicidae	Culex	Culex pipiens	SPECIES	40.03110	-82.94970	3030923791
1256	Diptera	Culicidae	Culex	Culex pipiens	SPECIES	40.04000	-1.14000	1501153058
1257	Diptera	Culicidae	Culex	Culex pipiens	SPECIES	40.07110	-83.01210	3030848723
1258	Diptera	Culicidae	Culex	Culex pipiens	SPECIES	40.07194	-88.29115	1986615250
1259	Diptera	Culicidae	Culex	Culex pipiens	SPECIES	40.09223	-88.29071	1986615733
1260	Diptera	Culicidae	Culex	Culex pipiens	SPECIES	40.09960	-83.06140	3030444888
1261	Diptera	Culicidae	Culex	Culex pipiens	SPECIES	40.10800	-83.16260	3030482787
1262	Diptera	Culicidae	Culex	Culex pipiens	SPECIES	40.11056	-88.20722	3801578421
1263	Diptera	Culicidae	Culex	Culex pipiens	SPECIES	40.12580	-82.89600	3030923061
1264	Diptera	Culicidae	Culex	Culex pipiens	SPECIES	40.12700	-83.04110	3030483950
1265	Diptera	Culicidae	Culex	Culex pipiens	SPECIES	40.13701	-97.18248	2883168004
1266	Diptera	Culicidae	Culex	Culex pipiens	SPECIES	40.14840	-83.14220	3030919775
1267	Diptera	Culicidae	Culex	Culex pipiens	SPECIES	40.17423	-105.53091	3059751723
1268	Diptera	Culicidae	Culex	Culex pipiens	SPECIES	40.19000	0.22000	1501648901
1269	Diptera	Culicidae	Culex	Culex pipiens	SPECIES	40.19340	-87.72920	3030290220
1270	Diptera	Culicidae	Culex	Culex pipiens	SPECIES	40.19360	-87.72920	3030679197
1271	Diptera	Culicidae	Culex	Culex pipiens	SPECIES	40.19380	-87.72940	3030276246

№	Order	Family	Genus	Species	Taxon Rank	Latitude	Longitude	GBIF_ID
1272	Diptera	Culicidae	Culex	Culex pipiens	SPECIES	40.19677	-112.42831	3404206692
1273	Diptera	Culicidae	Culex	Culex pipiens	SPECIES	40.30001	-90.06083	3801627576
1274	Diptera	Culicidae	Culex	Culex pipiens	SPECIES	40.32440	-79.70110	1434744363
1275	Diptera	Culicidae	Culex	Culex pipiens	SPECIES	40.34000	33.87000	3347283423
1276	Diptera	Culicidae	Culex	Culex pipiens	SPECIES	40.41051	-90.34095	2466131479
1277	Diptera	Culicidae	Culex	Culex pipiens	SPECIES	40.46570	19.47248	3714034765
1278	Diptera	Culicidae	Culex	Culex pipiens	SPECIES	40.54022	-111.80826	2864833083
1279	Diptera	Culicidae	Culex	Culex pipiens	SPECIES	40.61000	-74.00000	3349471838
1280	Diptera	Culicidae	Culex	Culex pipiens	SPECIES	40.64000	-73.97000	3351695275
1281	Diptera	Culicidae	Culex	Culex pipiens	SPECIES	40.64892	-74.00255	3337571733
1282	Diptera	Culicidae	Culex	Culex pipiens	SPECIES	40.68800	22.85820	3893357993
1283	Diptera	Culicidae	Culex	Culex pipiens	SPECIES	40.70590	-111.80700	3030434623
1284	Diptera	Culicidae	Culex	Culex pipiens	SPECIES	40.71040	-111.87300	3030472907
1285	Diptera	Culicidae	Culex	Culex pipiens	SPECIES	40.71080	-111.85900	3030424737
1286	Diptera	Culicidae	Culex	Culex pipiens	SPECIES	40.71530	-111.84700	3030327918
1287	Diptera	Culicidae	Culex	Culex pipiens	SPECIES	40.72240	-111.86000	3030434784
1288	Diptera	Culicidae	Culex	Culex pipiens	SPECIES	40.72360	-111.82300	3030425751
1289	Diptera	Culicidae	Culex	Culex pipiens	SPECIES	40.72452	19.56071	3714032698
1290	Diptera	Culicidae	Culex	Culex pipiens	SPECIES	40.72760	-111.93200	3030304889
1291	Diptera	Culicidae	Culex	Culex pipiens	SPECIES	40.72780	-112.04600	3030328040
1292	Diptera	Culicidae	Culex	Culex pipiens	SPECIES	40.72950	-111.84200	3030304809
1293	Diptera	Culicidae	Culex	Culex pipiens	SPECIES	40.72960	-111.88300	3030328028
1294	Diptera	Culicidae	Culex	Culex pipiens	SPECIES	40.73090	-111.85700	3030434619
1295	Diptera	Culicidae	Culex	Culex pipiens	SPECIES	40.73870	-111.83700	3030472805
1296	Diptera	Culicidae	Culex	Culex pipiens	SPECIES	40.74040	-111.81700	3030424752
1297	Diptera	Culicidae	Culex	Culex pipiens	SPECIES	40.74040	-112.04700	3030472574
1298	Diptera	Culicidae	Culex	Culex pipiens	SPECIES	40.74060	-112.03000	3030472607
1299	Diptera	Culicidae	Culex	Culex pipiens	SPECIES	40.74090	-111.89100	3030434686
1300	Diptera	Culicidae	Culex	Culex pipiens	SPECIES	40.74220	-111.87500	3030305007
1301	Diptera	Culicidae	Culex	Culex pipiens	SPECIES	40.74750	-111.96800	3030304858
1302	Diptera	Culicidae	Culex	Culex pipiens	SPECIES	40.75000	-111.86100	3030434717
1303	Diptera	Culicidae	Culex	Culex pipiens	SPECIES	40.75000	-73.85000	4152804468
1304	Diptera	Culicidae	Culex	Culex pipiens	SPECIES	40.75190	-111.82600	3030434776
1305	Diptera	Culicidae	Culex	Culex pipiens	SPECIES	40.75230	-111.91800	3030435635
1306	Diptera	Culicidae	Culex	Culex pipiens	SPECIES	40.75280	-111.89800	3030434609
1307	Diptera	Culicidae	Culex	Culex pipiens	SPECIES	40.75420	-112.00400	3030304810
1308	Diptera	Culicidae	Culex	Culex pipiens	SPECIES	40.75480	-112.03900	3030327958
1309	Diptera	Culicidae	Culex	Culex pipiens	SPECIES	40.75520	-111.89300	3030324832
1310	Diptera	Culicidae	Culex	Culex pipiens	SPECIES	40.76403	-73.99378	3456560083
1311	Diptera	Culicidae	Culex	Culex pipiens	SPECIES	40.76430	-111.87600	3030434531
1312	Diptera	Culicidae	Culex	Culex pipiens	SPECIES	40.76430	-111.86200	3030434807

№	Order	Family	Genus	Species	Taxon Rank	Latitude	Longitude	GBIF_ID
1313	Diptera	Culicidae	Culex	Culex pipiens	SPECIES	40.76500	-112.00600	3030836630
1314	Diptera	Culicidae	Culex	Culex pipiens	SPECIES	40.76890	-112.08300	3030472635
1315	Diptera	Culicidae	Culex	Culex pipiens	SPECIES	40.77150	-111.97700	3030434829
1316	Diptera	Culicidae	Culex	Culex pipiens	SPECIES	40.77230	-111.92300	3030434823
1317	Diptera	Culicidae	Culex	Culex pipiens	SPECIES	40.77550	-111.92000	3030307000
1318	Diptera	Culicidae	Culex	Culex pipiens	SPECIES	40.77630	-111.89900	3030435683
1319	Diptera	Culicidae	Culex	Culex pipiens	SPECIES	40.77870	-112.02900	3030472496
1320	Diptera	Culicidae	Culex	Culex pipiens	SPECIES	40.78160	-111.86500	3030434789
1321	Diptera	Culicidae	Culex	Culex pipiens	SPECIES	40.78230	-111.95800	3030837444
1322	Diptera	Culicidae	Culex	Culex pipiens	SPECIES	40.78440	-112.10900	3030424760
1323	Diptera	Culicidae	Culex	Culex pipiens	SPECIES	40.78530	-111.92500	3030434539
1324	Diptera	Culicidae	Culex	Culex pipiens	SPECIES	40.78770	-112.06200	3030327908
1325	Diptera	Culicidae	Culex	Culex pipiens	SPECIES	40.79330	-112.01200	3030434642
1326	Diptera	Culicidae	Culex	Culex pipiens	SPECIES	40.79480	-112.12200	3030434910
1327	Diptera	Culicidae	Culex	Culex pipiens	SPECIES	40.80010	-111.93700	3030434632
1328	Diptera	Culicidae	Culex	Culex pipiens	SPECIES	40.80210	-112.05000	3030327892
1329	Diptera	Culicidae	Culex	Culex pipiens	SPECIES	40.80670	-112.01700	3030327849
1330	Diptera	Culicidae	Culex	Culex pipiens	SPECIES	40.80770	-112.00700	3030916812
1331	Diptera	Culicidae	Culex	Culex pipiens	SPECIES	40.81240	-111.93700	3030708945
1332	Diptera	Culicidae	Culex	Culex pipiens	SPECIES	40.81570	-111.94200	3030472646
1333	Diptera	Culicidae	Culex	Culex pipiens	SPECIES	40.81600	-112.10100	3030434530
1334	Diptera	Culicidae	Culex	Culex pipiens	SPECIES	40.82200	-112.01500	3030327039
1335	Diptera	Culicidae	Culex	Culex pipiens	SPECIES	40.82410	-112.06900	3030304780
1336	Diptera	Culicidae	Culex	Culex pipiens	SPECIES	40.82430	-112.05900	3030304976
1337	Diptera	Culicidae	Culex	Culex pipiens	SPECIES	40.82630	-112.11400	3030327868
1338	Diptera	Culicidae	Culex	Culex pipiens	SPECIES	40.82650	-111.94400	3030435901
1339	Diptera	Culicidae	Culex	Culex pipiens	SPECIES	40.82990	-111.98600	3030424744
1340	Diptera	Culicidae	Culex	Culex pipiens	SPECIES	40.83100	-112.03200	3030304844
1341	Diptera	Culicidae	Culex	Culex pipiens	SPECIES	40.84180	-111.97000	3030434610
1342	Diptera	Culicidae	Culex	Culex pipiens	SPECIES	40.84210	-111.95300	3030328058
1343	Diptera	Culicidae	Culex	Culex pipiens	SPECIES	40.84450	-112.00300	3030304960
1344	Diptera	Culicidae	Culex	Culex pipiens	SPECIES	40.84590	-111.98200	3030434800
1345	Diptera	Culicidae	Culex	Culex pipiens	SPECIES	40.84600	-112.02900	3030829649
1346	Diptera	Culicidae	Culex	Culex pipiens	SPECIES	40.84690	-111.96200	3030469832
1347	Diptera	Culicidae	Culex	Culex pipiens	SPECIES	40.84778	-104.72561	3802059072
1348	Diptera	Culicidae	Culex	Culex pipiens	SPECIES	40.85646	19.64251	3714035266
1349	Diptera	Culicidae	Culex	Culex pipiens	SPECIES	40.86990	-112.02300	3030327927
1350	Diptera	Culicidae	Culex	Culex pipiens	SPECIES	40.87000	-73.45000	3351757812
1351	Diptera	Culicidae	Culex	Culex pipiens	SPECIES	41.00010	19.52954	3714034521
1352	Diptera	Culicidae	Culex	Culex pipiens	SPECIES	41.10387	22.92132	3384358241
1353	Diptera	Culicidae	Culex	Culex pipiens	SPECIES	41.17000	27.74000	3347283422

№	Order	Family	Genus	Species	Taxon Rank	Latitude	Longitude	GBIF_ID
1354	Diptera	Culicidae	Culex	Culex pipiens	SPECIES	41.19000	27.80000	3350291534
1355	Diptera	Culicidae	Culex	Culex pipiens	SPECIES	41.21000	27.75000	3347322316
1356	Diptera	Culicidae	Culex	Culex pipiens	SPECIES	41.22000	26.82000	3350283728
1357	Diptera	Culicidae	Culex	Culex pipiens	SPECIES	41.23000	-95.91000	2245458372
1358	Diptera	Culicidae	Culex	Culex pipiens	SPECIES	41.25000	-95.91000	2245429891
1359	Diptera	Culicidae	Culex	Culex pipiens	SPECIES	41.26000	-95.92000	2245416608
1360	Diptera	Culicidae	Culex	Culex pipiens	SPECIES	41.27117	-72.95048	3090988350
1361	Diptera	Culicidae	Culex	Culex pipiens	SPECIES	41.28000	-95.88000	2245433718
1362	Diptera	Culicidae	Culex	Culex pipiens	SPECIES	41.28000	-95.86000	2245459804
1363	Diptera	Culicidae	Culex	Culex pipiens	SPECIES	41.29638	19.50392	3714032354
1364	Diptera	Culicidae	Culex	Culex pipiens	SPECIES	41.31583	19.49222	2980867326
1365	Diptera	Culicidae	Culex	Culex pipiens	SPECIES	41.34607	2.12528	3859874238
1366	Diptera	Culicidae	Culex	Culex pipiens	SPECIES	41.34730	19.85098	3714033692
1367	Diptera	Culicidae	Culex	Culex pipiens	SPECIES	41.35260	-73.00844	3090987340
1368	Diptera	Culicidae	Culex	Culex pipiens	SPECIES	41.36533	19.73007	3714034458
1369	Diptera	Culicidae	Culex	Culex pipiens	SPECIES	41.38395	-72.90278	3090997312
1370	Diptera	Culicidae	Culex	Culex pipiens	SPECIES	41.44560	-83.79930	3030216257
1371	Diptera	Culicidae	Culex	Culex pipiens	SPECIES	41.45500	2.25400	3714033443
1372	Diptera	Culicidae	Culex	Culex pipiens	SPECIES	41.48470	-83.85740	3030150289
1373	Diptera	Culicidae	Culex	Culex pipiens	SPECIES	41.49000	-90.63000	2245419456
1374	Diptera	Culicidae	Culex	Culex pipiens	SPECIES	41.50740	-83.72800	3030685496
1375	Diptera	Culicidae	Culex	Culex pipiens	SPECIES	41.52500	2.04000	3714041004
1376	Diptera	Culicidae	Culex	Culex pipiens	SPECIES	41.53000	-90.68000	2245416268
1377	Diptera	Culicidae	Culex	Culex pipiens	SPECIES	41.53000	-90.61000	2245425727
1378	Diptera	Culicidae	Culex	Culex pipiens	SPECIES	41.53110	-83.77240	3030273182
1379	Diptera	Culicidae	Culex	Culex pipiens	SPECIES	41.53536	19.51141	3714037475
1380	Diptera	Culicidae	Culex	Culex pipiens	SPECIES	41.53659	-73.93431	3090981312
1381	Diptera	Culicidae	Culex	Culex pipiens	SPECIES	41.54000	-93.58000	2245417451
1382	Diptera	Culicidae	Culex	Culex pipiens	SPECIES	41.54000	-90.53000	2245419054
1383	Diptera	Culicidae	Culex	Culex pipiens	SPECIES	41.54340	-83.84050	3030144925
1384	Diptera	Culicidae	Culex	Culex pipiens	SPECIES	41.54454	-73.20900	3355440228
1385	Diptera	Culicidae	Culex	Culex pipiens	SPECIES	41.55000	-90.49000	2245423766
1386	Diptera	Culicidae	Culex	Culex pipiens	SPECIES	41.55010	-83.69870	3030282312
1387	Diptera	Culicidae	Culex	Culex pipiens	SPECIES	41.56920	-83.79230	3030155213
1388	Diptera	Culicidae	Culex	Culex pipiens	SPECIES	41.58000	-93.68000	2245420808
1389	Diptera	Culicidae	Culex	Culex pipiens	SPECIES	41.58000	2.22000	3714032474
1390	Diptera	Culicidae	Culex	Culex pipiens	SPECIES	41.58290	-83.62970	3030399802
1391	Diptera	Culicidae	Culex	Culex pipiens	SPECIES	41.61000	-93.61000	2245416136
1392	Diptera	Culicidae	Culex	Culex pipiens	SPECIES	41.61700	-83.65320	3030329794
1393	Diptera	Culicidae	Culex	Culex pipiens	SPECIES	41.61840	-83.72120	3030399811
1394	Diptera	Culicidae	Culex	Culex pipiens	SPECIES	41.62000	-91.55000	2245431278

№	Order	Family	Genus	Species	Taxon Rank	Latitude	Longitude	GBIF_ID
1395	Diptera	Culicidae	Culex	Culex pipiens	SPECIES	41.62000	-93.56000	2245436315
1396	Diptera	Culicidae	Culex	Culex pipiens	SPECIES	41.62000	-93.67000	2245460777
1397	Diptera	Culicidae	Culex	Culex pipiens	SPECIES	41.62000	26.97000	3347277727
1398	Diptera	Culicidae	Culex	Culex pipiens	SPECIES	41.62290	-83.83550	3030233072
1399	Diptera	Culicidae	Culex	Culex pipiens	SPECIES	41.62910	-83.49930	3030329854
1400	Diptera	Culicidae	Culex	Culex pipiens	SPECIES	41.63000	-93.67000	2245436562
1401	Diptera	Culicidae	Culex	Culex pipiens	SPECIES	41.63900	-93.62860	2245416168
1402	Diptera	Culicidae	Culex	Culex pipiens	SPECIES	41.64000	-93.66000	2245421541
1403	Diptera	Culicidae	Culex	Culex pipiens	SPECIES	41.64030	-83.59840	3030358139
1404	Diptera	Culicidae	Culex	Culex pipiens	SPECIES	41.64183	-87.11457	2466114981
1405	Diptera	Culicidae	Culex	Culex pipiens	SPECIES	41.64430	-83.43720	3030330247
1406	Diptera	Culicidae	Culex	Culex pipiens	SPECIES	41.65429	-87.04364	2466121925
1407	Diptera	Culicidae	Culex	Culex pipiens	SPECIES	41.65700	-83.40790	3030155295
1408	Diptera	Culicidae	Culex	Culex pipiens	SPECIES	41.66300	-83.47080	3030155088
1409	Diptera	Culicidae	Culex	Culex pipiens	SPECIES	41.66700	-83.75930	3030144887
1410	Diptera	Culicidae	Culex	Culex pipiens	SPECIES	41.66722	-87.83028	3802099484
1411	Diptera	Culicidae	Culex	Culex pipiens	SPECIES	41.66820	-83.66030	3030144923
1412	Diptera	Culicidae	Culex	Culex pipiens	SPECIES	41.67380	-83.33700	3030233111
1413	Diptera	Culicidae	Culex	Culex pipiens	SPECIES	41.67480	-83.52190	3030302583
1414	Diptera	Culicidae	Culex	Culex pipiens	SPECIES	41.67576	-87.63633	2466128204
1415	Diptera	Culicidae	Culex	Culex pipiens	SPECIES	41.68400	41.85980	1038612303
1416	Diptera	Culicidae	Culex	Culex pipiens	SPECIES	41.68580	-83.82030	3030368261
1417	Diptera	Culicidae	Culex	Culex pipiens	SPECIES	41.68740	-83.57600	3030155054
1418	Diptera	Culicidae	Culex	Culex pipiens	SPECIES	41.69000	-91.60000	2245429481
1419	Diptera	Culicidae	Culex	Culex pipiens	SPECIES	41.69340	-83.66870	3030233213
1420	Diptera	Culicidae	Culex	Culex pipiens	SPECIES	41.70480	-83.48080	3030285487
1421	Diptera	Culicidae	Culex	Culex pipiens	SPECIES	41.71960	-83.72070	3030233263
1422	Diptera	Culicidae	Culex	Culex pipiens	SPECIES	41.72290	-83.60090	3030155018
1423	Diptera	Culicidae	Culex	Culex pipiens	SPECIES	41.72460	-87.90620	3030954090
1424	Diptera	Culicidae	Culex	Culex pipiens	SPECIES	41.72500	-87.90620	3030954053
1425	Diptera	Culicidae	Culex	Culex pipiens	SPECIES	41.72908	-87.88162	2466130107
1426	Diptera	Culicidae	Culex	Culex pipiens	SPECIES	41.73243	-111.84400	2517952599
1427	Diptera	Culicidae	Culex	Culex pipiens	SPECIES	41.73281	-87.55699	2466122214
1428	Diptera	Culicidae	Culex	Culex pipiens	SPECIES	41.73580	-81.29920	1434744341
1429	Diptera	Culicidae	Culex	Culex pipiens	SPECIES	41.73740	-87.81940	3030399769
1430	Diptera	Culicidae	Culex	Culex pipiens	SPECIES	41.73766	-87.69755	2466113555
1431	Diptera	Culicidae	Culex	Culex pipiens	SPECIES	41.73766	-87.69755	2466131927
1432	Diptera	Culicidae	Culex	Culex pipiens	SPECIES	41.73766	-87.69755	2466130169
1433	Diptera	Culicidae	Culex	Culex pipiens	SPECIES	41.73970	-87.88510	3030399784
1434	Diptera	Culicidae	Culex	Culex pipiens	SPECIES	41.74140	-87.86590	3030954037
1435	Diptera	Culicidae	Culex	Culex pipiens	SPECIES	41.74598	-87.58619	2466130524

№	Order	Family	Genus	Species	Taxon Rank	Latitude	Longitude	GBIF_ID
1436	Diptera	Culicidae	Culex	Culex pipiens	SPECIES	41.76820	-87.85480	3030399746
1437	Diptera	Culicidae	Culex	Culex pipiens	SPECIES	41.78030	-87.61896	2466128691
1438	Diptera	Culicidae	Culex	Culex pipiens	SPECIES	41.78180	-87.90590	3030446212
1439	Diptera	Culicidae	Culex	Culex pipiens	SPECIES	41.78198	-87.61088	2466124854
1440	Diptera	Culicidae	Culex	Culex pipiens	SPECIES	41.78390	-87.80340	3030399939
1441	Diptera	Culicidae	Culex	Culex pipiens	SPECIES	41.78482	-73.93319	3090987312
1442	Diptera	Culicidae	Culex	Culex pipiens	SPECIES	41.78550	-87.89700	3030434732
1443	Diptera	Culicidae	Culex	Culex pipiens	SPECIES	41.78740	-87.89660	3030399768
1444	Diptera	Culicidae	Culex	Culex pipiens	SPECIES	41.78850	-87.80540	3030154502
1445	Diptera	Culicidae	Culex	Culex pipiens	SPECIES	41.79800	-74.74483	2466127843
1446	Diptera	Culicidae	Culex	Culex pipiens	SPECIES	41.81020	-87.86580	3030399790
1447	Diptera	Culicidae	Culex	Culex pipiens	SPECIES	41.82000	-81.24000	1434744374
1448	Diptera	Culicidae	Culex	Culex pipiens	SPECIES	41.82080	-87.64076	2466116641
1449	Diptera	Culicidae	Culex	Culex pipiens	SPECIES	41.82170	-87.88860	3030405808
1450	Diptera	Culicidae	Culex	Culex pipiens	SPECIES	41.82380	-87.89390	3030399852
1451	Diptera	Culicidae	Culex	Culex pipiens	SPECIES	41.82460	-87.88460	3030446260
1452	Diptera	Culicidae	Culex	Culex pipiens	SPECIES	41.83050	-87.81040	3030400015
1453	Diptera	Culicidae	Culex	Culex pipiens	SPECIES	41.83080	-87.83990	3030953989
1454	Diptera	Culicidae	Culex	Culex pipiens	SPECIES	41.83094	-87.81863	2466131872
1455	Diptera	Culicidae	Culex	Culex pipiens	SPECIES	41.83260	-87.81440	3030953963
1456	Diptera	Culicidae	Culex	Culex pipiens	SPECIES	41.83730	-87.80520	3030400169
1457	Diptera	Culicidae	Culex	Culex pipiens	SPECIES	41.84190	-87.80470	3030954055
1458	Diptera	Culicidae	Culex	Culex pipiens	SPECIES	41.84340	-87.80660	3030415639
1459	Diptera	Culicidae	Culex	Culex pipiens	SPECIES	41.84440	-87.81510	3030434497
1460	Diptera	Culicidae	Culex	Culex pipiens	SPECIES	41.85050	-87.81690	3030399973
1461	Diptera	Culicidae	Culex	Culex pipiens	SPECIES	41.85361	-87.68576	2466115295
1462	Diptera	Culicidae	Culex	Culex pipiens	SPECIES	41.85590	-87.88830	3030399792
1463	Diptera	Culicidae	Culex	Culex pipiens	SPECIES	41.86070	-87.83070	3030406028
1464	Diptera	Culicidae	Culex	Culex pipiens	SPECIES	41.86190	-87.81700	3030405806
1465	Diptera	Culicidae	Culex	Culex pipiens	SPECIES	41.86190	-87.88090	3030954130
1466	Diptera	Culicidae	Culex	Culex pipiens	SPECIES	41.86600	-87.87460	3030405833
1467	Diptera	Culicidae	Culex	Culex pipiens	SPECIES	41.86920	-87.86600	3030399785
1468	Diptera	Culicidae	Culex	Culex pipiens	SPECIES	41.87090	-87.78460	3030400126
1469	Diptera	Culicidae	Culex	Culex pipiens	SPECIES	41.87130	-87.78850	3030399873
1470	Diptera	Culicidae	Culex	Culex pipiens	SPECIES	41.87550	-87.82580	3030953928
1471	Diptera	Culicidae	Culex	Culex pipiens	SPECIES	41.88230	-87.79200	3030954078
1472	Diptera	Culicidae	Culex	Culex pipiens	SPECIES	41.88710	-87.90620	3030953924
1473	Diptera	Culicidae	Culex	Culex pipiens	SPECIES	41.89330	-87.82640	3030406035
1474	Diptera	Culicidae	Culex	Culex pipiens	SPECIES	41.89340	-87.82640	3030406266
1475	Diptera	Culicidae	Culex	Culex pipiens	SPECIES	41.89410	-87.79410	3030959050
1476	Diptera	Culicidae	Culex	Culex pipiens	SPECIES	41.89630	-87.86560	3030399763

№	Order	Family	Genus	Species	Taxon Rank	Latitude	Longitude	GBIF_ID
1477	Diptera	Culicidae	Culex	Culex pipiens	SPECIES	41.90010	-71.08977	3090998341
1478	Diptera	Culicidae	Culex	Culex pipiens	SPECIES	41.90270	-87.81910	3030405682
1479	Diptera	Culicidae	Culex	Culex pipiens	SPECIES	41.90430	-87.78610	3030405892
1480	Diptera	Culicidae	Culex	Culex pipiens	SPECIES	41.90530	-87.79170	3030399834
1481	Diptera	Culicidae	Culex	Culex pipiens	SPECIES	41.90580	-87.82270	3030953941
1482	Diptera	Culicidae	Culex	Culex pipiens	SPECIES	41.91944	12.51917	3714032349
1483	Diptera	Culicidae	Culex	Culex pipiens	SPECIES	41.92254	-87.70456	2856703547
1484	Diptera	Culicidae	Culex	Culex pipiens	SPECIES	41.98000	-91.54000	2245429667
1485	Diptera	Culicidae	Culex	Culex pipiens	SPECIES	42.00000	-93.60000	2245417214
1486	Diptera	Culicidae	Culex	Culex pipiens	SPECIES	42.00000	-71.33700	3030398918
1487	Diptera	Culicidae	Culex	Culex pipiens	SPECIES	42.03099	8.92206	4138336989
1488	Diptera	Culicidae	Culex	Culex pipiens	SPECIES	42.03472	-93.62000	3802093573
1489	Diptera	Culicidae	Culex	Culex pipiens	SPECIES	42.05000	-93.46000	2245436600
1490	Diptera	Culicidae	Culex	Culex pipiens	SPECIES	42.05000	-71.32800	3030317153
1491	Diptera	Culicidae	Culex	Culex pipiens	SPECIES	42.06500	12.66694	3714033074
1492	Diptera	Culicidae	Culex	Culex pipiens	SPECIES	42.07000	-91.74000	2245416378
1493	Diptera	Culicidae	Culex	Culex pipiens	SPECIES	42.08000	-71.41100	3030328108
1494	Diptera	Culicidae	Culex	Culex pipiens	SPECIES	42.08100	-71.25600	3030370715
1495	Diptera	Culicidae	Culex	Culex pipiens	SPECIES	42.09000	-91.58000	2245420500
1496	Diptera	Culicidae	Culex	Culex pipiens	SPECIES	42.09500	-71.19900	3030317152
1497	Diptera	Culicidae	Culex	Culex pipiens	SPECIES	42.09800	-71.19100	3030772598
1498	Diptera	Culicidae	Culex	Culex pipiens	SPECIES	42.10600	-71.19200	3030395603
1499	Diptera	Culicidae	Culex	Culex pipiens	SPECIES	42.11500	-71.07600	3030370600
1500	Diptera	Culicidae	Culex	Culex pipiens	SPECIES	42.11800	-71.27000	3030398594
1501	Diptera	Culicidae	Culex	Culex pipiens	SPECIES	42.12200	-71.04200	3030398583
[truncated: 577,769 more chars]
